# Effector granules in human T lymphocytes: the luminal proteome of secretory lysosomes from human T cells

**DOI:** 10.1186/1478-811X-9-4

**Published:** 2011-01-21

**Authors:** Hendrik Schmidt, Christoph Gelhaus, Melanie Nebendahl, Marcus Lettau, Ralph Lucius, Matthias Leippe, Dietrich Kabelitz, Ottmar Janssen

**Affiliations:** 1 Institute of Immunology, Christian-Albrechts-University, UK S-H Campus Kiel, Kiel, Germany; 2 Department of Zoophysiology, Zoological Institute, Christian-Albrechts-University, Kiel, Germany; 3 Institute of Anatomy, Christian-Albrechts-University, Kiel, Germany

## Abstract

**Background:**

Cytotoxic cells of the immune system have evolved a lysosomal compartment to store and mobilize effector molecules. In T lymphocytes and NK cells, the death factor FasL is one of the characteristic marker proteins of these so-called secretory lysosomes, which combine properties of conventional lysosomes and exocytotic vesicles. Although these vesicles are crucial for immune effector function, their protein content in T cells has so far not been investigated in detail.

**Results:**

In the present study, intact membranous vesicles were enriched from homogenates of polyclonally activated T cells and initially characterized by Western blotting and electron microscopic inspection. The vesicular fraction that contained the marker proteins of secretory lysosomes was subsequently analyzed by 2D electrophoresis and mass spectrometry. The proteome analysis and data evaluation revealed that 70% of the 397 annotated proteins had been associated with different lysosome-related organelles in previous proteome studies.

**Conclusion:**

We provide the first comprehensive proteome map of T cell-derived secretory lysosomes with only minor contaminations by cytosolic, nuclear or other proteins. This information will be useful to more precisely address the activation-dependent maturation and the specific distribution of effector organelles and proteins in individual T or NK cell populations in future studies.

## Background

Cytotoxic T lymphocytes (CTL) and Natural Killer (NK) cells are the main cytotoxic effector cells of the immune system. In order to effectively eliminate virus-infected and tumorigenic cells, they rapidly mobilize effector molecules including granzymes, perforin, granulysin and the death factor FasL (CD178) that are presumably stored in preformed organelles termed secretory lysosomes (SL) [1]. Secretory lysosomes combine degradative properties of conventional lysosomes with characteristics of exocytotic vesicles. At the level of morphology, conventional and secretory lysosomes are hardly distinguishable and both appear to represent endpoints of an endocytotic pathway and are formed by fusion and fission of endosomes and lysosomes [2]. Similar to conventional lysosomes, large membrane areas are covered by lysosome-associated membrane-proteins (LAMPs) including LAMP-1 (CD107a), LAMP-2 (CD107b) and LAMP-3 (CD63) [3-5]. However, secretory effector lysosomes are characterized by a specific set of membrane and luminal marker proteins [6,7]. The current consensus is that SL of CTLs and NK cells carry the aforementioned effector proteins either in the lysosomal lumen (granzymes, perforin and granulysin) or as characteristic transmembrane compounds (FasL) [8-10].

Recently, we provided a protocol that allows a substantial enrichment of intact SL from *in vitro *expanded lymphocyte populations [11]. Employing this procedure for subcellular fractionation of a crude organelle preparation, we obtained a fraction of intact vesicles that is significantly enriched in SL marker proteins. We were thus able to report the first comprehensive analysis of the luminal proteome of secretory lysosomes from NK cells [12]. At that time, 234 different proteins were identified by mass spectrometry, 77% of which had been associated with SL or other lysosomal compartments before. Applying 2D difference gel electrophoresis, we also described a cell line-specific distribution of functionally relevant proteins in SL from human NK cell lines and primary NK cells [12].

Based on this study, it appears likely that different T cell populations utilize the SL organelles to store and mobilize lineage-specific cargo proteins. However, the proteome of secretory lysosomes in T cells has not been deciphered. To provide the first proteome map for T cell-derived SL, we enriched organelles from activated T lymphoblasts. Organelle extracts were subjected to SDS-PAGE and Western blotting to identify the FasL-containing SL fraction. This fraction was analyzed by electron microscopy to demonstrate the enrichment of a homogeneous population of intact vesicles. In order to define the luminal proteome of the respective SL compartment, the organelles were lysed and proteins were separated by 2D gel electrophoresis. Mass spectrometry was applied to identify individual spots. We annotated 397 proteins, 70% of which had been associated with lysosome-related organelles before. With the present report, we thus provide the first comprehensive description of the content of FasL-carrying effector vesicles isolated from activated human T lymphocytes.

## Results and Discussion

In our preceding analysis of the SL compartment of NK cell lines and primary NK cells, we annotated 234 individual proteins and demonstrated a cell line-specific distribution of several functionally relevant molecules including cytotoxic effector proteins, lysosomal proteases and MHC molecules [12]. As a basis to address unsolved issues regarding the maturation, function and cell type-specific composition of the cytotoxic effector compartment in T cell populations, we now analyzed the proteome of enriched secretory lysosomes from *in vitro *activated human T cell blasts.

### FasL-associated secretory lysosomes in activated lymphocytes

**Figure 1:**
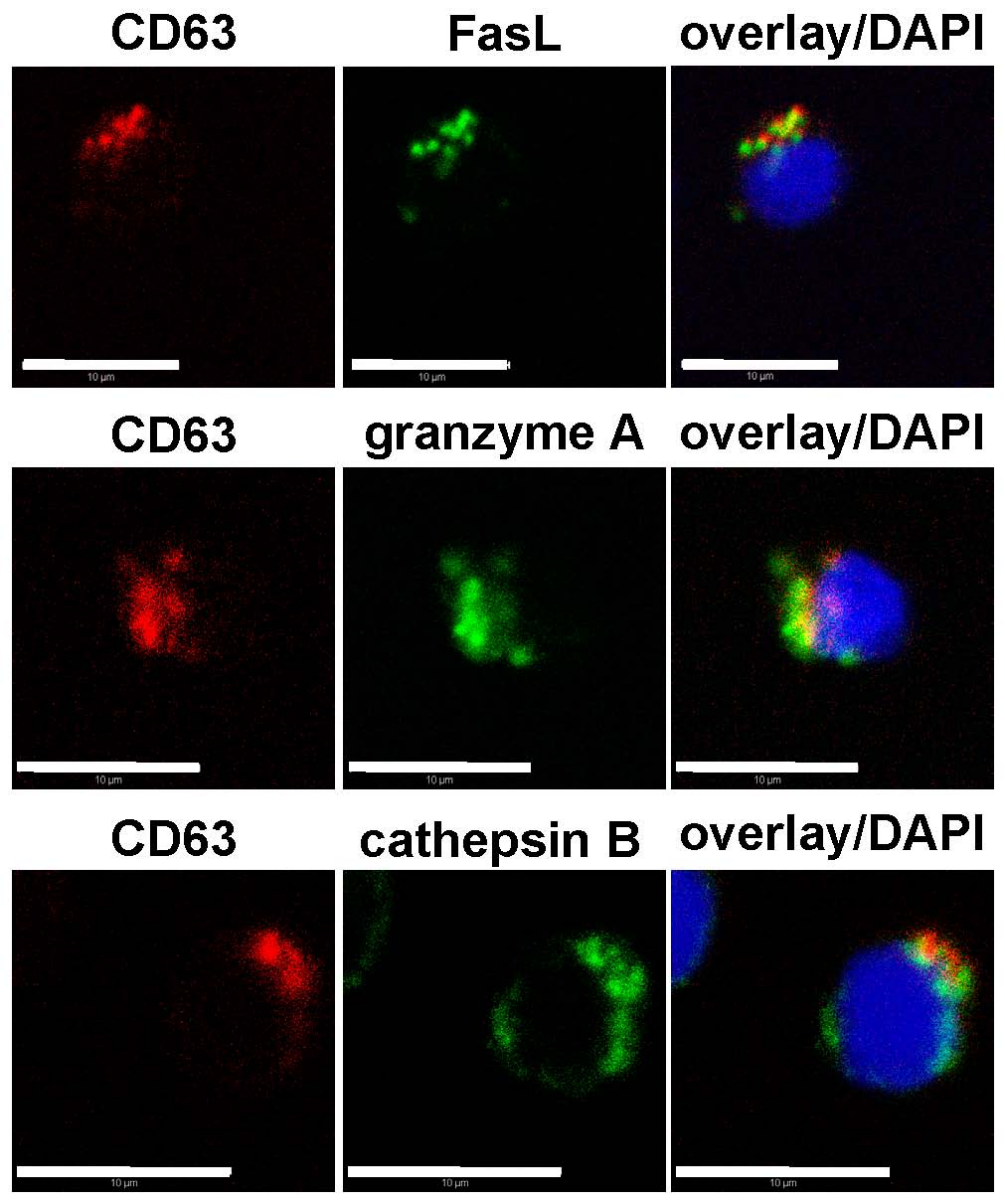
**In T cell blasts, FasL associates with lysosomal vesicles**. PHA blasts (d14) were fixed and stained for FasL with NOK1 and Alexa Fluor488-conjugated donkey anti-mouse IgG or for granzyme A with GrA-11 FITC-conjugated mAbs or for cathepsin B with polyclonal goat anti-cathepsin B (N-19) antibodies and Alexa Fluor488-conjugated donkey anti-goat IgG. After extensive washing, all samples were stained for CD63 with Alexa Fluor555-conjugated mAb MEM-259. Nuclei were visualized by DAPI (bar: 10 μm).

We and others have shown that in CTLs, preformed FasL accumulates in the limiting membrane of secretory lysosomes with late endosome or multi-vesicular-body structure and there co-localizes with characteristic lysosomal marker proteins including CD63 or lysosomal hydrolases and cytoskeletal adapter proteins [7-9,13-15]. Confocal laser-scanning microscopy (CLSM) was applied to confirm that FasL also might serve as a marker for secretory lysosomes in *in vitro *expanded PHA-stimulated T lymphocytes used in the present study. As depicted in Figure 1, we detected an apparent co-localization of CD63 with FasL, granzyme A and the lysosomal protease cathepsin B. It should be mentioned that a common or distinct localization of LAMP-3 (CD63) and FasL is still controversially discussed. Several reports suggest a co-localization of FasL with granule proteins, such as cathepsin D, CD63, granzyme B, perforin and LAMP-1 in a single granular entity [8,9] whereas other studies indicate that CD63 and FasL are located in distinct subcellular compartments [16].

**Figure 2:**
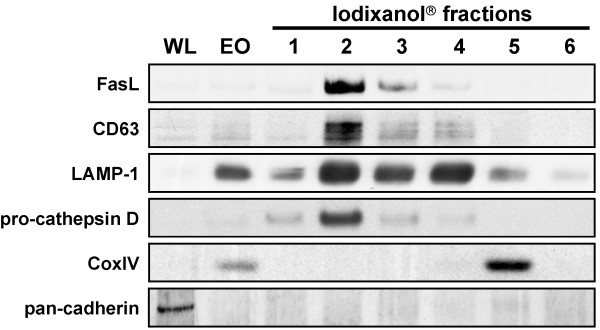
**Western blot analysis of subcellular fractions**. Individual lanes represent whole cell lysate (WL), enriched organelles (EO) and the six fractions collected after density gradient centrifugation of IL-2 expanded T lymphocytes. The blots were probed with antibodies against respective markers for lysosomal (CD63, LAMP-1, cathepsin D) and secretory organelles (FasL). Pan-cadherin served as a marker for plasma membranes, CoxIV for mitochondria.

Our protocol for the enrichment of secretory lysosomes yielded six separate fractions that were subjected to further analysis by Western blotting or 2D gel electrophoresis. To demonstrate an effective enrichment of the SL fraction, we first separated the proteins of individual fractions by SDS-PAGE and stained for characteristic organelle marker proteins after Western blotting. As shown in Figure 2, indicated by the high abundance of FasL, CD63 and cathepsin D, SL were enriched in fraction 2. Although LAMP-1 was also enriched in this fraction, the presence of this lysosomal membrane protein in other fractions might indicate the complex composition of the lysosomal compartment in general and that other lysosome-related vesicles might exist with distinct biophysical properties that separate at different media densities. As further indicators for the effective organelle enrichment and separation, we used cytochrome oxidase subunit IV (CoxIV) as a marker for mitochondria (see enriched organelles and fraction 5 in Figure 2) and pan-cadherin as a marker for the plasma membrane (only present in whole cell lysates). Of note, all proteins that were enriched in separate fractions were of course also present in the enriched organelle (EO) fraction placed on the gradient. However, due to the the relatively low abundance of individual proteins in the EO fraction, Western blot detection at the displayed exposure time did only reveal very faint bands. This is in agreement with our previous report [11] in which we showed a massive enrichment of FasL in fraction 2 while in the starting EO material from different T cell populations, FasL was almost not detectable at the same exposure time.

Regarding the "purity" of the obtained fraction, it should be stressed that most if not all enrichment protocols published so far do not allow a "purification" rather than an "enrichment" of a given organelle population. This is presumably based on the fact that lysosome formation and protein loading is a highly dynamic process that implies fusion and fission of several membraneous compartments and a complex protein sorting and transport machinery. For the initial characterization of enriched SL [11], we already pointed to potential "contaminations" in fraction 2, using antibodies against EEA1, a putative marker for endosomes, or Bip/Grp78, a marker for ER, respectively. Interestingly, during these analyses, golgin, a marker for the golgi apparatus/cisternae was only detected in fractions 3-6, but not in fractions 1 and 2 [11]. For the present study, we thus restricted ourselves to routinely check for the marker proteins depicted in Figure 2.

### The enriched SL fraction consists of homogeneous intact vesicles

**Figure 3:**
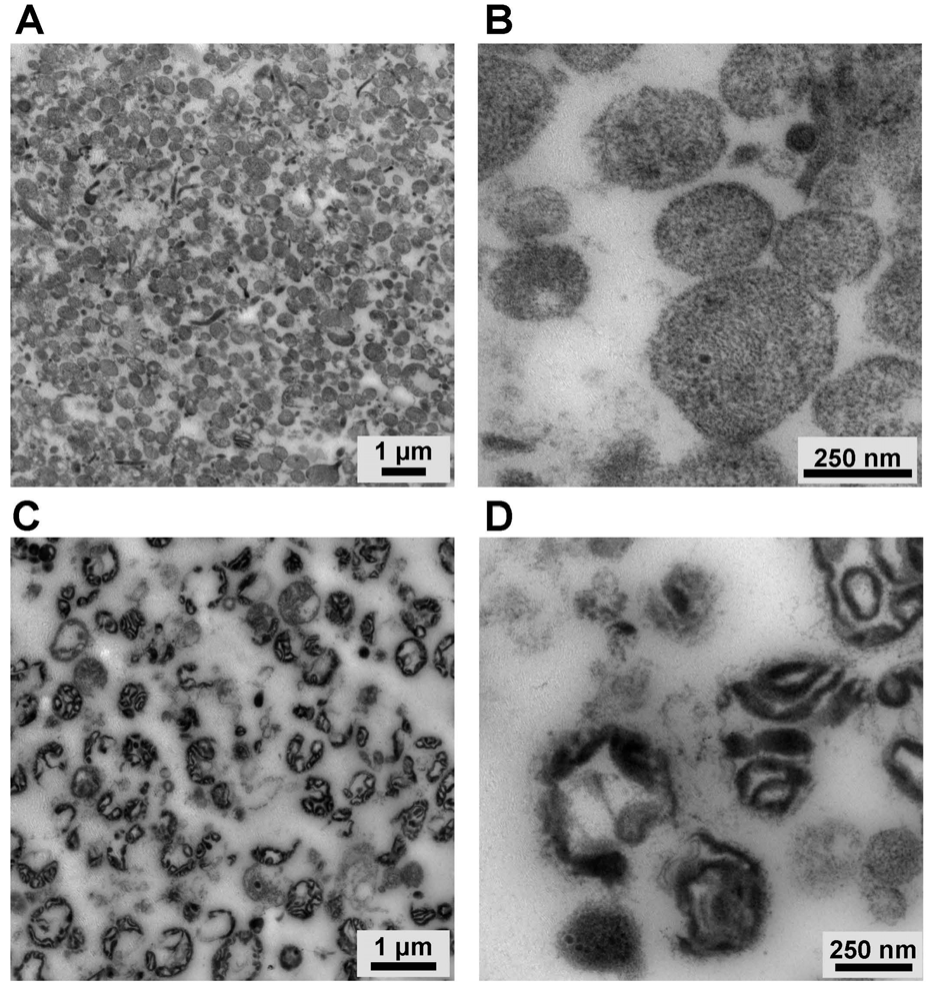
**Electron micrographs of fractions 2 and 5**. Enriched organelles from PHA blasts corresponding to fractions 2 (A, B) and 5 (C, D) were examined under an electron microscope. Overview pictures are given in A and C, magnified areas are shown in B and D. (scale as indicated)

In addition to the biochemical analysis of the individual fractions, we visualized the obtained lysosomal fraction 2 by electron microscopy in comparison to the putative mitochondrial fraction 5. Figure 3 provides characteristic overview pictures of the two fractions. In both cases, the organelles within one fraction display a high degree of homogeneity with respect to their morphology (Figure 3A,C). At higher magnification, the characteristics of the organelles in fraction 2 become apparent. These membranous vesicles are round-shaped with a maximum size of about 700 nm and display a characteristic electron density. In contrast, organelles of fraction 5 are characterized by irregular internal membranous structures (Figure 3B,D) as expected for mitochondria.

### The luminal proteome of enriched SL as analyzed by 2D-PAGE and mass spectrometry

**Figure 4:**
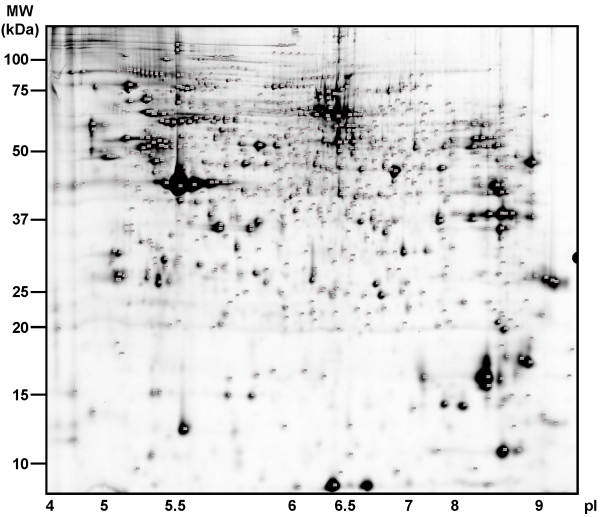
**2D proteome map of enriched secretory lysosomes from activated T cell blasts (overview)**. A total of 250 μg of fraction 2 protein were separated on pH 3-11NL IPG strips in the first and on 12.5% Tris-glycin gels in the second dimension. Proteins stained by Flamingo Pink were detected using fluorescence imaging. From a total of six gels, 742 spots were identified and annotated as 397 individual proteins. Enlarged sections of all four quadrants are available as additional file to allow the positioning of individual annotations given in table 1.

In order to obtain a comprehensive list of putative luminal proteins of secretory lysosomes, enriched fraction 2 vesicles of PHA-stimulated T lymphoblasts were subjected to 2D-PAGE. More than 1600 spots from 6 replicate gels were subsequently subjected to proteolytic cleavage and lead to the mass spectrometric identification of 1335 spots. Due to repetitive identifications at respective spot locations in different gels, the actual number of identified individual spots decreased to 742. The resulting proteome map is shown as an overview in Figure 4. Additional information on identified proteins and images of individual quadrants to match proteins to respective spots are given as additional files 1, 2 and 3 (Table S1, FigureS1, Dataset S1). Multiple (up to six) identifications in separate gels from individual secretory lysosomes preparations from T cells of different donors also underscore the reproducibility of the isolation protocol [11]. Overall, the identified spots represent a total of 397 separate protein entries in the NCBI database that are listed according to their protein names, the predicted subcellular distribution and function in Table 1.

**Table 1:** Proteins identified in enriched secretory lysosomes from activated T cells.

Protein name	Spot #	Predicted/annotated subcellular localisation	Predicted function
100 kDa coactivator	122	ER,ME,PL	biosynthesis
14-3-3 protein beta	714	CY,ME	adapter
14-3-3 protein epsilon	685	CY,ME	adapter
14-3-3 protein zeta/delta	708	CY,ME	adapter
2',3'-cyclic-nucleotide 3'-phosphodiesterase	542	ME,NG	hydrolase
26S protease regulatory subunit 6A	1037	CY,NU	protein degradation
26S proteasome non-ATPase regulatory subunit 2	165	ME	proteasome
3-phosphoglycerate dehydrogenase	408	ME	biosythesis
acetyl-CoA acetyltransferase, cytosolic	594	CY	biosynthesis
ACTB protein	525	ER,ME,EX,PL,SY	cell motility
actin related protein 2 isoform b	564	EN,ER,ME	trafficking
actin related protein 2/3 complex subunit 1B	590	ME,PL	trafficking
actin related protein 2/3 complex subunit 2	674	EN,ME,PL,ER	trafficking
actin related protein 2/3 complex subunit 3	799	EN,ME,PL,ER	trafficking
actin related protein 2/3 complex subunit 4	798	ER,EX,ME,SY	trafficking
actin related protein 2/3 complex subunit 5-like	804	ME	trafficking
actin, alpha, cardiac muscle	570	ME	cell motility
actin, gamma 1 propeptide	701	ME,EX,PL,SY	cell motility
actinin, alpha 4	144	ME,NU,CY	cell motility
acylamino acid-releasing enzyme	203	PL, CY	hydrolase
acyl-CoA synthetase long-chain family member 4 isoform 2	199	MT,PE,ME	metabolism
adenine phosphoribosyltransferase isoform b	784	ME,EX,PL	biosynthesis
adenosine deaminase	550	CY,LY	hydrolase
adenylosuccinate lyase	439	CY,PL	biosynthesis
adenylosuccinate synthetase	1023	CY	biosynthesis
adenylyl cyclase-associated protein variant	395	PL,ME	trafficking
aflatoxin aldehyde reductase AFAR	1056	PL,GO	redox protein
aging-associated gene 12	418	unknown	unclassified
alanyl-tRNA synthetase	111	ME,PL	biosynthesis
alcohol dehydrogenase class-3	587	PL,CY	redox protein
aldo-keto reductase family 1, member A1	1078	PL,SY	metabolism
aldolase A	560	EN,ME	metabolism
aldose 1-epimerase (BLOCK25)	596	CY	metabolism
aldose reductase	981	ME,EX,MT	metabolism
alkyldihydroxyacetonephosphate synthase, peroxisomal	273	PE	biosynthesis
alpha-tubulin	362	PL	cell motility
annexin A1	615	ME,MT	trafficking
annexin A11	415	MT,EX,ME	trafficking
annexin A2	631	MT,EX,ME,secreted	exocytosis
annexin A4	668	MT,EX,ME,SY	signal trans.
annexin A5	660	MT,EX,ME,ER	trafficking
annexin A6	263	MT,EX,ME,ER	trafficking
annexin A7 isoform 2	504	EX,ME,PL	exocytosis
ARP3 actin-related protein 3 homolog	465	EN,ER,ME	trafficking
ARTS-1	136	ER,ME	immunity
aryl hydrocarbon receptor interacting protein	955	unknown	unclassified
asparaginyl-tRNA synthetase	306	ME	biosynthesis
aspartate aminotransferase	575	CY	biosynthesis
ATP citrate lyase	96	ME,EX,PL	biosynthesis
ATP synthase, alpha subunit precursor	426	ER,LY,NG,SY,MT	channel
ATPase, H+ transporting, lysosomal 56/58kDa, V1 subunit B2	413	LY,ME,NG,SY	channel
axin interactor, dorsalization associated protein	632	unknown	signal trans.
beta adrenergic receptor kinase 1	220	CY	GTPase
bifunctional purine biosynthesis protein PURH	326	ME,PL	mutifunctional
bleomycin hydrolase	463	CY,PL	hydrolase
BolA-like protein 2	867	ME	unclassified
calcium binding protein 39	622	EX	unclassified
CALM3 protein	816	unknown	unclassified
calreticulin precursor variant	287	ER,ME,NG,EX,PL,MT	chaperone
carboxyl terminal LIM domain protein	612	ER,ME,PL,EN	cell motility
catalase	361	PE,ER,LY,EN,ME	metabolism
cathepsin B	691	LY,ME,NG	hydrolase
cathepsin D preproprotein	696	LY,ME,NG,EX,MT	hydrolase
cathepsin H	738	LY	hydrolase
cathepsin S	749	LY	immunity
Cbr1 In Complex With Hydroxy-Pp	666	ME	redox protein
Cdc42ACK GTPASE	790	ME	cell motility
centaurin beta1	214	unknown	GTPase
chaperonin (HSP60)	338	ME,NG,EX,SY,MT	chaperone
chaperonin containing TCP1, subunit 2β	424	ER,EN,ME,PL,MT,CY	chaperone
chaperonin containing TCP1, subunit 7η	376	EN,ME,PL	chaperone
chaperonin containing TCP1, subunti 5ε	339	EX,PL	chaperone
chaperonin containing TCP1, subunti 8τ	357	EN,ME,EX	chaperone
chaperonin containing TCP1, subunti 8τ	358	EN,ME,EX	chaperone
chromatin modifying protein 4B	639	ME,EX	trafficking
chromosome 20 open reading frame 3 (BSCv)	489	ME	unclassified
chromosome 9 open reading frame 19	831	EX,GO	unclassified
N2 protein	651	unknown	unclassified
coactosin-like protein	841	PL,SY	unclassified
cofilin 1	810	ER,ME,EX,MT	cell motility
copine I	310	ME	trafficking
copine III	340	ME,EX,PL	trafficking
coronin 7	113	CY,GO	trafficking
coronin, actin binding protein, 1A	371	LY,PL	cell motility
coronin, actin binding protein, 1C	353	ME	multifunctional
c-src tyrosine kinase	475	CY,PL	signal trans.
cyclophilin A	826	ME,EX,MT	chaperone
cyclophilin B	800	ER,ME	chaperone
cystatin B	857	ME	protein inhibitor
cysteine and glycine-rich protein 1	769	NU	unclassified
cytoskeleton associated protein	952	CY	cell motility
cytosolic malate dehydrogenase	642	ME,EX,PL,SY,MT	metabolism
DCHT2 Serine/threonine-protein kinase OSR1	332	ME	signal trans.
destrin isoform a	814	ME,EX,MT	cell motility
differentially expressed in FDCP 6 homolog (mouse), isoform CRA_b	228	unknown	unclassified
dihydropyrimidinase-like 2	319	SY	signal trans.
dimethylarginine dimethylaminohydrolase 2	683	unknown	hydrolase
dipeptidyl peptidase 4	81	ER,LY,EN,ME,EX	hydrolase
DJ-1 protein	764	ME,PL,SY,MT	redox protein
DnaJ (Hsp40) homolog, subfamily A, member 1, isoform CRA_d	499	ER,ME	chaperone
DnaJ (Hsp40) homolog, subfamily B, member 11 precursor	551	ER	chaperone
docking protein 2	455	unknown	unclassified
dynamin 2 isoform 1	148	EN	trafficking
echinoderm microtubule associated protein like 2 variant	929	CY	cell motility
EF-hand domain family, member D2	1012	unknown	unclassified
EH-domain containing 1	342	ER,LY,EN,EX,PL	trafficking
Ena-VASP-like protein	447	CY	cell motility
ENC-1AS aka Beta-hexosaminidase subunit beta	431	LY	multifunctional
endoplasmic reticulum protein 29 isoform 1 precursor	721	ER,ME,PL	chaperone
enolase 1 variant	496	ME,EX,SY,MT	metabolism
ERAP2 protein	99	ER	immunity
ERBB2IP protein	197	NU,CY	multifunctional
ERO1L	311	ME,ER	redox protein
esterase D/formylglutathione hydrolase	656	ME	hydrolase
eukaryotic translation elongation factor 1 alpha 1	462	ER,LY,EN,ME,EX,PL	biosynthesis
eukaryotic translation elongation factor 1 gamma, isoform CRA_d	947	ME	biosynthesis
eukaryotic translation elongation factor 2	158	ER,EN,ME,EX	biosynthesis
eukaryotic translation initiation factor 4A	505	ME	biosynthesis
eukaryotic translation initiation factor 5A	817	ME	biosynthesis
extended-synaptotagmin-1 KIAA0747 protein	155	ME	unclassified
ezrin	208	CY	cell motility
F-actin capping protein alpha-1 subunit	611	ER,EN,ME	actin binding
F-actin capping protein alpha-1 subunit variant	623	ER,EN,ME	actin binding
F-actin capping protein alpha-2 subunit	616	ER,EN,ME,PL	cell motility
F-actin capping protein beta subunit	663	ER,EN,ME	actin binding
farnesyl pyrophosphate synthetase	579	CY	biosynthesis
FK506 binding protein 1A	856	ME,SY,MT	signal trans.
flotillin 1	486	LY,ME,EX	membrane
formin-binding protein 1	1059	SL, LY, CY	adapter
fructose-bisphosphate aldolase C	565	ME,SY,MT	metabolism
fumarate hydratase, mitochondrial	507	EN,SY,MT	cell cycle
FYN-binding protein	71	CY,NU	adapter
G protein beta subunit	638	ME,MT	signal trans.
galectin-1	851	ME,PL	immunity
galectin-3	718	ME,NU	immunity
gamma-enolase	476	ME,PL,SY	glycolysis
gamma-glutamyl hydrolase	629	LY,ME,NG,PL	hydrolase
GDP-mannose pyrophosphorylase A	541	unknown	biosythesis
gelsolin-like capping protein isoform 9	572	ME,CY,NU	cell motility
GIPC1 protein	598	SY,CY	protein binding
glia maturation factor gamma	818	unknown	unclassified
glucosamine-6-phosphate deaminase 1	677	CY	hydrolase
glucose-6-phosphate dehydrogenase isoform b	409	ME	metabolism
glucosidase II subunit beta	126	ER,ME,PL	hydrolase
glucosidase, alpha; neutral AB, isoform CRA_a	936	ER,ME,PL	hydrolase
glutamate carboxypeptidase	430	unknown	hydrolase
glutamate Dehydrogenase-Apo Form	437	ER,ME,PL,MT	unclassified
glutaredoxin 3	589	CY	redox protein
glutathione S-transferase P1	766	ER,ME,EX,PL	metabolism
glutathione synthetase	461	PL	redox protein
glutathione-S-transferase kappa 1	765	PL,ME,MT,PE	unclassified
glutathione-S-transferase omega 1	698	LY,ME,NG,EX,PL,SY,MT	metabolism
glyceraldehyde-3-phosphate dehydrogenase	610	LY,ME,NG,EX,PL,SY,MT	metabolism
glycyl-tRNA synthetase	244	ME	biosynthesis
glyoxalase domain containing 4	653	MT	unclassified
GNAS complex locus isoform f	531	EX	multifunctional
GNB1 protein	634	EN,ME,EX,PL,SY	signal trans.
granzyme A	724	SL	immunity
GRAP2 protein	957	unknown	unclassified
GRB2 protein	756	SY	adapter
GTP-binding nuclear protein Ran	755	ME,EX	trafficking
guanine nucleotide binding protein (G protein), alpha inhibiting activity polypeptide 2, isoform CRA_c	582	EX	GTPase
guanine nucleotide binding protein (G protein), beta polypeptide 2-like 1, isoform CRA_d	664	ER	signal trans.
guanine nucleotide-binding protein G(k) subunit alpha	585	ME,EX	trafficking
guanine nucleotide-binding protein subunit alpha-13	989	ME	signal trans.
haloacid dehalogenase-like hydrolase domain containing 2	690	unknown	hydrolase
heat shock 70kDa protein 1A	278	ER,EN,ME,EX,MT	chaperone
heat shock 70kDa protein 5	226	ER,ME,EX,PL,MT	chaperone
heat shock 70kDa protein 8 isoform 1	259	LY,ME,NG,EX,PL,SY,MT	chaperone
heat shock 70kDa protein 8 isoform 1	260	LY,ME,NG,EX,PL,SY,MT	chaperone
heat shock protein 70	112	EX	chaperone
heat shock protein HSP 90-alpha	969	ME,NG,MT	chaperone
heat shock protein HSP 90-beta	177	ME,EX,MT	chaperone
hematopoietic cell-specific Lyn substrate 1	181	CY,MT	signal trans.
HEXA protein	422	LY	multifunctional
hexose-6-phosphate dehydrogenase	194	ER	metabolism
HIP-55	377	CY	signal trans.
histidine triad nucleotide binding protein 1	852	ME,PL,SY	hydrolase
histocompatibility (minor) HA-1	1072	unknown	GTPase
hypothetical protein	216	unknown	unclassified
hypothetical protein LOC79624	472	unknown	unclassified
hypoxia up-regulated protein 1	47	ER,PL,ME	chaperone
importin subunit beta-1	164	ME	trafficking
integrin beta-2	80	PL	membrane
interleukin-16	210	secreted	immunity
isocitrate dehydrogenase 1 (NADP+), soluble, isoform CRA_b	540	ME,EX,PL	redox protein
isocitrate dehydrogenase 2 (NADP+), mitochondrial, isoform CRA_b	510	PL,MT	redox protein
kinase/transmembrane domain fusion protein	1061	unknown	unclassified
laminin-binding protein	543	ME,ER	cell adhesion
leucine aminopeptidase 3	432	CY	protein degradation
leucine rich repeat containing 57	747	unknown	unclassified
leucine-rich repeat and calponin homology domain-containing protein 5	908	MT	protein binding
leucocyte antigen CD97	872	ME,secreted	cell adhesion
leukocyte-derived arginine aminopeptidase long form variant	102	unknown	hydrolase
leukotriene A4 hydrolase	309	CY	hydrolase
LIM and SH3 domain protein 1	606	ER,EN,ME,PL	adapter
LIM domain-containing protein 2	834	unknown	unclassified
lin 7 homolog c	1070	SY	exocytosis
L-lactate dehydrogenase	645	ME,EX,SY	metabolism
L-lactate dehydrogenase B chain	626	ME,EX,PL,SY,MT	redox protein
L-plastin	266	CY	actin binding
L-plastin variant	267	unknown	cell motility
LPXN protein	474	unknown	unclassified
lymphocyte cytosolic protein 2	229	CY	immunity
lymphocyte-specific protein 1	959	PL	immunity
lysosomal acid alpha-mannosidase	265	LY,ME	hydrolase
M2-type pyruvate kinase	356	ME,EX,SY	metabolism
Macrophage Migration Inhibitory Factor (Mif) With Hydroxphenylpyruvate	862	ME,EX,PL,SY	immunity
MAGUK p55 subfamily member 7	292	PL	protein binding
methylenetetrahydrofolate dehydrogenase 1	139	EN,ME,PL,MT	multifunctional
methylthioadenosine phosphorylase	697	CY	metabolism
MHC class I antigen	533	ME	immunity
MHC class I antigen	865	ME	immunity
MHC class II antigen	953	ME	immunity
MHC class II antigen DR alpha chain	1050	LY	immunity
MHC class II antigen DR52	1083	ME	immunity
microtubule-associated protein, RP/EB family, member 1	665	ME,PL	cell motility
mitochondrial ATP synthase, H+ transporting F1 complex beta subunit	443	MT	trafficking
mitochondrial trifunctional protein, alpha subunit precursor	253	PL, MT	metabolism
mitogen-activated protein kinase 1	569	ME,PL	signal trans.
mitogen-activated protein kinase kinase 1 interacting protein 1	943	LY	adapter
mitogen-activated protein kinase kinase 2	509	unknown	signal trans.
moesin, isoform CRA_b	246	EN,ME,EX,PL,MT	cell motility
mps one binder kinase activator-like 1B	758	unknown	unclassified
myosin IG	75	unknown	trafficking
myosin light polypeptide 6	830	ME	cell motility
NADH dehydrogenase (ubiquinone) Fe-S protein 1, 75 kDa (NADH-coenzyme Q reductase)	896	ER,ME,MT	trafficking
NCK adaptor protein 1	506	CY,ER	adapter
NECAP endocytosis associated 2	1010	EN	trafficking
NESH protein	434	unknown	unclassified
N-ethylmaleimide-sensitive factor attachment protein, alpha	652	ME,NG,PL	trafficking
neuroblastoma RAS viral (v-ras) oncogene homolog	779	GO,CY	trafficking
neuropolypeptide h3	781	ME,EX,SY	protein inhibitor
neutrophil adherence receptor alpha-M subunit	36	membrane	cell adhesion
niban protein isoform 2	38	CY	signal trans.
NME1-NME2 protein	823	CY,NU	multifunctional
nuclear chloride channel	684	ME,EX,PL,MT	channel
nucleobindin 1 variant	335	unknown	unclassified
nucleoside phosphorylase	670	CY,PL	cell cycle
nucleosome assembly protein 1-like 1, isoform CRA_d	315	ME,PL,NU	cell cycle
Obg-like ATPase 1	511	EN,ME,PL	hydrolase
otubain 1	637	ME	hydrolase
PA2G4 protein	490	unknown	unclassified
PDCD6IP protein	171	unknown	unclassified
perforin-1	280	SL	immunity
peroxiredoxin 1	774	ER,LY,EN,ME,NG,PL,MT	redox protein
peroxiredoxin 2	778	ER,EN,ME,SY,MT	redox protein
peroxiredoxin 3	768	ME,PL,MT	redox protein
peroxiredoxin 4	737	ER,EN,ME	redox protein
peroxiredoxin 6	945	LY,ME,EX,PL,SY	redox protein
PGAM1	730	ME,EX,SY	metabolism
PHB	948	unknown	unclassified
phosphatase 2a	316	MT	multifunctional
phosphatidylinositol-5-phosphate 4-kinase, type II, alpha	457	NG,PL	metabolism
phosphofructokinase, liver	939	unknown	glycolysis
phosphofructokinase, platelet	196	ME,PL	glycolysis
phosphoglucose isomerase	390	ME,EX,PL,MT	multifunctional
phosphoglycerate kinase 1	537	ME,EX,SY,MT	metabolism
phospholipase C, delta 1 variant	178	unknown	signal trans.
phosphoribosyl pyrophosphate synthetase 1 variant	1080	unknown	biosynthesis
phosphoribosylaminoimidazole carboxylase, - succinocarboxamide synthetase, isoform CRA_b	523	EN,SY	multifunctional
phosphoribosylformylglycinamidine synthase	64	CY	biosynthesis
phosphoserine aminotransferase 1	988	ME	biosynthesis
phostensin	91	CY	unclassified
poly(A) binding protein, cytoplasmic 1, isoform CRA_c	256	ER,EN,ME,PL	metabolism
poly(rC) binding protein 1	1082	ME,CY,NU	unclassified
potassium voltage-gated channel, shaker-related subfamily, beta member 2 isoform 2	635	CY	channel
PPP5C protein	364	CY,NU	hydrolase
profilin-1	848	ME,EX,PL,MT	actin binding
programmed cell death protein 10	741	unknown	apoptosis
proline synthetase co-transcribed homolog	699	CY	unclassified
prolyl 4-hydroxylase, alpha subunit	337	ER,ME	redox protein
prolyl 4-hydroxylase, beta subunit precursor	348	ER,ME,EX,PL,MT	redox protein
prolyl endopeptidase	234	CY	protein degradation
proteasome (prosome, macropain) subunit, alpha type, 7(PSMA7)	729	CY,Proteasom	hydrolase
proteasome 26S non-ATPase subunit 13 isoform 1	577	ME	proteasome
proteasome 26S subunit, ATPase, 2	498	CY,NU	unclassified
proteasome 26S subunit, ATPase, 5	514	CY,NU	unclassified
proteasome activator complex subunit 1 isoform 1	703	PL,MT	immunity
proteasome activator complex subunit 2	689	ME	immunity
proteasome alpha 2 subunit variant	754	CY	hydrolase
proteasome subunit, alpha type, 1	687	ME	hydrolase
proteasome subunit, alpha type, 5	1009	ME	hydrolase
proteasome subunit, alpha type, 6	734	CY,NU	hydrolase
proteasome subunit, beta type, 1	750	ME,CY	hydrolase
proteasome subunit, beta type, 2	780	CY,NU	hydrolase
proteasome subunit, beta type, 4	944	CY,NU	hydrolase
proteasome subunit, beta type, 8	773	PL,CY,NU	immunity
protein ARMET	805	ME, secreted	unclassified
protein diaphanous homolog 1	45	ME	cell motility
protein disulfide isomerase-associated 4	1060	ER,ME,PL	chaperone
protein disulfide isomerase-related protein 5	458	ER,ME	chaperone
protein disulfide-isomerase A3	379	ER,LY,ME,NG,EX,PL	chaperone
protein phosphatase 1, catalytic subunit, alpha isoform 1	603	EX	hydrolase
protein phosphatase 1, catalytic subunit, beta isoform	617	ME,PL	hydrolase
protein tyrosine phosphatase 1b	536	ME,ER	hydrolase
protein tyrosine phosphatase, non-receptor type 6 isoform 1 variant	317	unknown	hydrolase
protein-tyrosine kinase fyn isoform c	373	EN,CY	signal trans.
PYD and CARD domain containing	771	CY	apoptosis
pyrophosphatase 1	654	ME,MT	hydrolase
pyruvate kinase 3 isoform 2	346	ME,EX,SY	metabolism
R33729_1 (Interleukin-25)	837	ME,secreted	signal trans.
Rab GDP dissociation inhibitor beta	469	ME,EX,PL,MT	GTPase
raftlin cell migration-inducing gene 2	193	PL	unclassified
Rap1a	785	EN,ME,MT	GTPase
Rap1-GTP-interacting adapter molecule	141	CY	signal trans.
Ras GTPase-activating-like protein IQGAP2	1069a	EN	signal trans.
related RAS viral (r-ras) oncogene homolog 2 isoform a	1049	LY,ME,EX	GTPase
Rho GDP dissociation inhibitor (GDI) alpha	716	ME,PL,MT	GTPase
Rho GDP dissociation inhibitor (GDI) beta	728	CY	GTPase
Rho GTPase activating protein 1	441	PL	GTPase
Rho GTPase-activating protein 9	998	unknown	GTPase
ribosomal protein L11	797	ribosome	biosynthesis
ribosomal protein L12	809	EN,ribosom	biosynthesis
S-adenosylhomocysteine hydrolase	513	ME	hydrolase
Sec23 homolog A	221	ER,ME,PL	trafficking
Sec23B protein	1001	EN	trafficking
septin 2	554	ME,EX,SY	unclassified
septin 7	484	ME,PL,SY	unclassified
septin-9 delta	558	ME	unclassified
septin-9 gamma	973	ME	unclassified
serine/threonine phosphatase 1 gamma	985	MT,SY	hydrolase
serine/threonine-protein kinase PAK 2	352	PL	signal trans.
serine/threonine-protein phosphatase 2A catalytic subunit alpha isoform	621	MT	signal trans.
serine/threonine-protein phosphatase 2A regulatory subunit B	592	NU	signal trans.
serpin peptidase inhibitor,clade B,member 1	548	CY	protein inhibitor
seryl-tRNA synthetase	365	ME,PL	tRNA processing
SH2 domain protein 1A	840	CY	signal trans.
SH3-containing protein, Endophilin-B1	1081	CY,GO,MT	apoptosis
SHUJUN-1	795	CY	cell motility
signal transducer and activator of transcription 1, 91kDa, isoform CRA_d	188	CY,NU	signal trans.
similar to metallo-beta-lactamase superfamily protein	686	unknown	hydrolase
small GTP binding protein Rac2, isoform CRA_c	1006	unknown	signal trans.
soc-2 suppressor of clear homolog	318	CY	unclassified
solute carrier family 9 (sodium/hydrogen exchanger), isoform 3 regulator 1	460	ME,EX,PL	scaffolding
sorting nexin 17	421	EN,ME,PL	trafficking
sorting nexin 6	466	CY	trafficking
src kinase associated phosphoprotein 1 isoform 1	417	CY,NU	signal trans.
stathmin 1/oncoprotein 18	820	SY	cell motility
stress-induced-phosphoprotein 1 (Hsp70/Hsp90-organizing protein)	323a	ME,PL,SY	chaperone
stromal cell-derived factor 2-like 1 precursor	767	ER	unclassified
superoxide dismutase 1, soluble	806	ME,EX,MT	redox protein
syntaxin binding protein 1	305	ME,NG,EX,PL,SY	trafficking
syntaxin binding protein 2	302	EX,PL	trafficking
syntaxin binding protein 3 variant	294	ME,PL	trafficking
talin-1	920	EN,ME,PL	cell motility
tapasin isoform 3 precursor	495	ER,ME	immunity
TC4 protein	736	NU	GTPase
T-complex polypeptide 1	1030	ER,EN,ME,EX	chaperone
T-complex protein 1 subunit gamma	307	CY	chaperone
testin isoform 1	456	unknown	unclassified
thioredoxin domain-containing protein 4 precursor	502	ER,ME,PL	scaffolding
transfer RNA-Trp synthetase	411	ME,PL	biosynthesis
transgelin-2	787	ME,MT	unclassified
transketolase	276	ME,EN	unclassified
translocon-associated protein subunit delta	811	ME,ER	trafficking
triosephosphate isomerase 1	742	ME,EX,SY,MT	unclassified
tripeptidyl-peptidase 1	992	LY,ME,NG,PL,MT	protein degradation
tropomodulin 3	561	ER,ME	cell motility
tropomyosin 3 isoform 2	676	unknown	unclassified
tropomyosin 4	672	ME	unclassified
Tu translation elongation factor, mitochondrial	517	LY,ME,PL,MT	biosynthesis
tubulin alpha 6 variant	363	ME,PL	cell motility
tubulin tyrosine ligase-like family, member 12	897	ME	trafficking
tubulin, beta	407	ME,PL,SY	cell motility
tubulin, beta polypeptide	433	ME,PL,SY	cell motility
tumor rejection antigen (gp96) 1	118	ER,ME,PL,GO	chaperone
tumor susceptibility gene 101	470	EX	trafficking
twinfilin-like protein	578	CY	cell motility
tyrosine kinase LCK	399	CY	signal trans.
tyrosine-protein phosphatase non-receptor type 6	325	CY,NU	signal trans.
tyrosyl-tRNA synthetase	366	ME,PL	signal trans.
ubiquitin associated and SH3 domain containing protein A	913	CY,NU	protein degradation
ubiquitin specific peptidase 5 isoform 2	154	LY,ME,NG	protein degradation
ubiquitin specific protease 14 isoform a	344	PL	protein degradation
ubiquitin-conjugating enzyme E2 L3	882	MT	protein degradation
ubiquitin-conjugating enzyme E2 N	839	ME,EX,MT	differentiation
ubiquitin-like modifier-activating enzyme 1	120	MT,ME	protein degradation
UDP-glucose ceramide glucosyltransferase-like 1 isoform 1	39	ER,ME	chaperone
UDP-glucose pyrophosphorylase 2 isoform b	442	EN,ME	metabolism
UNC-112 related protein 2 long form	971	PL	cell adhesion
unnamed protein product	706	unknown	unclassified
UPF0550 protein C7orf28	450	ME	unclassified
vacuolar H+-ATPase 56,000 subunit	414	LY,ME,NG,SY	channel
vacuolar protein sorting 45A	322	LY,EN	trafficking
vacuolar sorting protein 33A	1067	EN,LY	trafficking
valosin-containing protein	159	unknown	unclassified
vasodilator-stimulated phosphoprotein	503	PL	cell motility
vinculin	108	ME	cell motility
voltage-dependent anion channel 1	658	ER,LY,ME,NG,EX,PL,SY,MT	channel
voltage-dependent anion channel 2	657	SY,MT	channel
voltage-dependent anion channel 3	688	EN,ME,MT	channel
V-type proton ATPase subunit d 1	599	LY,EN,ME,SY	channel
WD repeat domain 1	304	EN,ME,EX	cell motility
Wiskott-Aldrich syndrome protein	323b	CY	cell motility
XRP2 protein	546	ME	signal trans.
zeta-chain associated protein kinase 70kDa	277	CY	signal trans.

397 individual proteins were identified to be associated with enriched secretory lysosomes from human T cell blasts. The proteins are listed by name, followed by individual spot numbers and the predicted/annotated subcellular localisation and function. Abbreviations: LY: lysosomes, ME: melanosomes, PL: platelet granules, SY: synaptosomes, EX: exosomes, CG: cytotoxic granules, NG: neuromelanin granules, EN: endosomes, MT: mitochondria, GO: Golgi, PE: peroxisomes, CY: cytoplasm, ER: endoplasmic reticulum and NU: nucleus. For detailed information on individual spots/proteins, please refer to the additional files.

**Figure 5:**
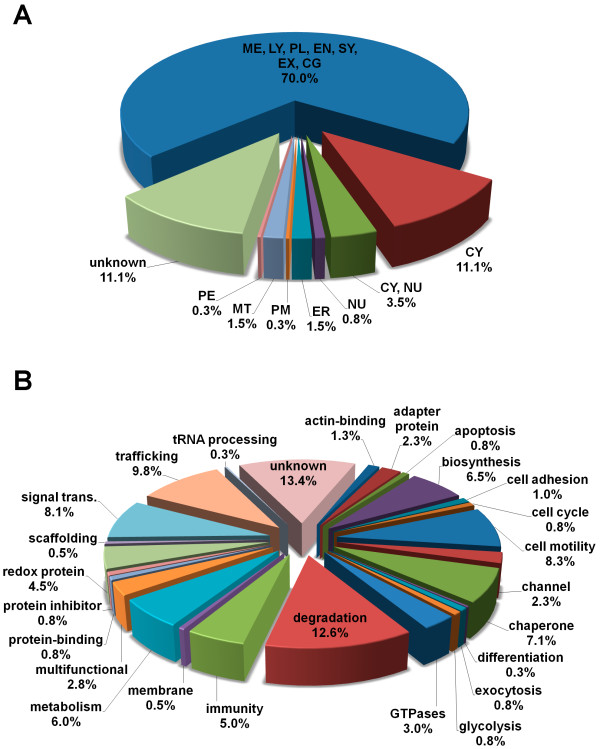
**Classification of the identified proteins according to their localization (A) and function (B)**. The cellular localization and function of 397 identified proteins were classified as detailed in material and methods. Lysosomal and secretory vesicles are represented by melanosomes (ME), lysosomes (LY), platelet granules (PL), endosomes (EN), synaptosomes (SY), exosomes (EX) or cytolytic granules (CG). Other cellular compartments are: cytosol (CY), nuclei (NU), peroxisomes (PE), plasma membrane (PM), mitochondria (MT), golgi (GO) or endoplasmic reticulum (ER).

Importantly, based on database annotations combining proteome analyses of different organelles [17], 70% of the 397 proteins were assigned to lysosomal or secretory vesicles (including cytolytic granules (CG), lysosomes (LY), exosomes (EX), endosomes (EN), melanosomes (ME), platelet granules (PL) and synaptosomes (SY)) (Table 1, Figure 5). The majority of the remaining 30% was classified as proteins of unknown (11%) or cytosolic (11%) localization, and as cytosolic or nuclear proteins (CY/NU, 3.5%). The low percentage of mitochondrial (MT, 1.5%), nuclear (NU, 0.8%), plasma membrane (PM, 0.3%) or endoplasmic strictly reticulum-associated (ER, 1.5%) and peroxisomal proteins (PE, 0.3%) again underscores the selective enrichment of lysosomal organelles in the present study. In terms of function, the classification revealed a large heterogeneity and a broad spectrum of potential activities. However, as expected, proteins associated with degradation, signal transduction, trafficking and immunity formed about 35% of the total proteome of enriched SL (Figure 5B). The important role of these organelles in cytotoxicity is also supported by the identified effector molecules perforin (#280) and granzyme A (#707, 717, 720, 724).

Interestingly, and in contrast to the published SL proteome of NK cells [12], we did not detect significant amounts of granzyme B at the respective position in 2D gels from T cell blasts. However, this is in line with our previous observation that granzyme B might be stored in a separate compartment formed by electron dense granules that do not contain transmembrane FasL and that sediment as fraction 6 in our enrichment gradient [11]. To prove this result and address this issue in more detail, we started to analyze fraction 2 and fraction 6 vesicles (granzyme B granules). The direct comparison of the two granule populations by 2D DIGE and Western blotting clearly verified the result of the present analysis and provided first biochemical and proteomic evidence for two distinct species of cytotoxic effector vesicles in T cell blasts [18].

Surprisingly, it is still unknown to date whether functionally distinct TCRαβ and TCRγδ T cells, CD4^+ ^and CD8^+ ^T cells, vδ1^+ ^and vδ2^+ ^T cells, or normal and leukemic T cells also differ in terms of protein content and function of their lysosomal compartment(s). Based on the present description of the luminal proteome of FasL-containing secretory lysosomes in fully differentiated T cells, it will be possible to directly compare the content of cytotoxic effector organelles in different T cell subpopulations, e.g. by 2D difference gel electrophoresis. In addition, based on a larger set of marker proteins, the maturation of effector vesicles in the course of T cell activation can now be addressed in detail. Of note, using the applied protocol, we identified the luminal rather than the membrane proteome of this vesicular population. In addition, one has to consider that due to methodological limitations, the applied 2D technique might cover only about 20-30% of the total proteome and thus might be complemented in future studies employing LC-coupled mass spectrometric approaches.

## Conclusion

We provide the first comprehensive proteome map of T cell-derived secretory lysosomes with only minor contaminations by cytosolic, nuclear or other proteins. This information will be useful to more precisely address the activation-dependent maturation and the specific distribution of effector organelles and proteins in individual T or NK cell populations in future studies.

## Methods

### Cells

Human peripheral blood mononuclear cells (PBMC) were isolated from buffy coat preparations by Ficoll density gradient centrifugation. For the generation of PHA-stimulated lymphoblasts, T cells were purified by magnetic cell sorting (MACS) using cell isolation kits from Miltenyi Biotech (Bergisch Gladbach, Germany). The cells were stimulated with phytohemagglutinin A (PHA, 0.5 μg/ml, Remel, Lenexa, KS, USA) and expanded in the presence of irradiated EBV-transformed B cells and allogenic PBMC and subsequently with recombinant interleukin 2 (rIL-2, 100 U/ml, Chiron GmbH, Marburg, Germany). Before the cells were analyzed on day 12-14, dead cells were removed by Ficoll-gradient centrifugation resulting in a > 98% pure T cell population as judged by CD3 FACS analysis.

### Confocal microscopy

Cells were fixed with 3% paraformaldehyde and permeabilized with 1% Triton X-100 as described [13]. The following antibodies were used: mouse IgG1 isotype-control MOPC-21 (Abcam, Cambridge, UK), anti-FasL mAb NOK1 (BD Biosciences, Heidelberg, Germany) with AlexaFluor488-conjugated goat anti-mouse IgG (Invitrogen, Karlsruhe, Germany), anti-CD63 mAb clone MEM-259 (Immunotools, Friesoythe, Germany) conjugated to AlexaFluor555 (Invitrogen), anti-Granzyme A-FITC (Immunotools) and anti-Cathepsin B (Santa Cruz Biotechnology, Santa Cruz, CA, USA) with AlexaFluor488-conjugated donkey anti-goat IgG (Invitrogen). Stained samples were mounted with ProLong Gold antifade reagent with DAPI (Invitrogen) and analyzed on a laser scanning microscope (LSM 510 Meta, Carl Zeiss, Jena, Germany) with appropriate filter settings. Images were acquired via scanning through the x-y-plane with 63 × objective lense. Laser intensity and detectors were adjusted to a uniformly negative signal of the control samples stained with control IgG and second step antibodies.

### Subcellular fractionation

For subcellular fractionation and enrichment of secretory lysosomes, at least 4x10^8 ^T cells were used. The fractionation procedure has been recently described in detail [11]. Briefly, the cells were mechanically disrupted and organelles were enriched by differential centrifugation steps. The enriched organelles were then loaded on a discontinuous density gradient (4.4 ml volume) with 27%, 22.5%, 19%, 16%, 12%, 8% Optiprep^® ^which is a 60% Iodixanol solution (Sigma, Deisenhofen, Germany) and subjected to ultracentrifugation. Interphases were collected from the top of the gradient resulting in six 400 μl fractions named and numbered 1 to 6. The protein content in each fraction was determined using a Coomassie Protein Assay Reagent (Thermo, Rockford, IL, USA).

### Western blot analysis

For Western blotting, 5 μg of protein were separated by SDS-PAGE on pre-casted 4-12% gradient Bis-Tris gels (Invitrogen). After transfer to nitrocellulose (NC) membranes (Biometra, Goettingen, Germany) and blocking with 5% BSA or dry milk, the fractions were analyzed for subcellular marker proteins with the following antibodies: anti-FasL clone G-247.4 (BD Biosciences), anti-CD63 clone MEM-259 (Acris Antibodies, Herford, Germany), anti-LAMP-1 clone 25 (BD Biosciences), anti-cathepsin D clone CTD-19 (Sigma), anti-cytochrome oxidase IV (CoxIV) mAb clone 10G8D12C12 (1/1000, MitoScience, Eugene, OR, USA), anti pan-cadherin clone ab22744 (Abcam, Cambridge, UK) and horseradish peroxidase (HRP)-conjugated goat anti-mouse secondary antibody (GE Healthcare, Munich, Germany). Membranes were prepared for reprobing by incubation in stripping solution (100 mM 2-mercaptoethanol, 2% SDS, 60 mM Tris) for 25 min at 56°C. ECL reagents in combination with Hyper Film (GE Healthcare) were used for chemiluminescence detection.

### Transmission electron microscopy

Enriched organelles of fractions 2 and 5 were fixed with a mixture of 3% paraformaldehyde and 0.05% glutaraldehyde in PBS at 4°C overnight, washed in PBS, postfixed in 2% OsO_4_, dehydrated in ethanol, and embedded in araldite (Sigma, Deisenhofen, Germany). Ultrathin sections were mounted on formvar-coated grids and double-stained with a saturated solution of uranyl acetate in 70% methanol and lead citrate. The grids were examined with a Zeiss EM 900 transmission electron microscope equipped with a digital camera system.

### 2D electrophoresis, image analysis and spot picking

The 2D electrophoresis was performed as described before [11]. Briefly, SL pellets of fraction 2 were lysed on ice for 30 min with 30 μl lysis buffer (pH 8.5) containing 7 M urea, 2 M thiourea, 30 mM Tris, 4% CHAPS. The supernatant was recovered after centrifugation for 20 min at 20.000 × g at 4°C. A total amount of 250 μg of protein was mixed with rehydration buffer (7 M urea, 2 M thiourea, 4% CHAPS, 2% (v/v), IPG buffer pH 3-11 and 2% (w/v) DTT) and applied by cup-loading onto 24 cm non-linear pH 3-11 IPG gel strips for isoelectric focusing (IEF). The second dimension was performed on 26 × 20 cm large 12.5% polyacrylamide gels after reduction and alkylation using the Ettan DALTsix large vertical electrophoresis system from GE Healthcare. The gels were removed from the glass plates, stained with Flamingo Pink (Bio Rad), mounted on a non-backed gel frame, scanned on a Typhoon Trio imager (GE Healthcare) and analyzed using Image Master 6.0 (GE Healthcare). Selected spots were picked with a 2 mm picking head. The picked gels were again scanned to verify the correct location of the punched spots.

### In-gel tryptic digestion and mass spectrometry

Gel plugs were washed with water and 12.5 mM ammonium bicarbonate (ABC) in 50% acetonitrile (ACN) and dehydrated in pure ACN. The dry gel pieces were rehydrated with 100 ng sequencing-grade trypsin (Serva, Heidelberg, Germany) in 5 mM ABC and tryptic in-gel digestion was performed at 37°C overnight. For peptide extraction, 0.3% trifluoroacetic acid (TFA) in ACN was added and the samples were sonicated for 15 min. The liquid phases were collected, lyophilized, redissolved in 0.5 to 1 μl MALDI matrix solution (3.2 mg/ml α-cyanohydroxycinnamic acid (Sigma) in 65% ACN/0.1% TFA), spotted onto 192-well stainless steel MALDI plates and air-dried. The samples were analyzed by peptide mass finger printing in positive reflectron mode followed by MSMS analyses of the most apparent five peptides using the 4700 Proteomics Analyzer mass spectrometer (Applied Biosystems, Framingham, MA, USA) as described elsewhere [12]. Peptide mass spectra were processed by internal calibration with autolytic fragments of porcine trypsin with 25 ppm mass tolerance. MSMS spectra were acquired using default calibration updated prior to the run. Spectral data were searched against human proteins in the NCBI database (*Homo sapiens*, 192,176 entries) using MASCOT V2.0 (Matrix Sciences, London, UK).

### Database analysis

Database searches with MASCOT were performed using the following parameters: the modification on cysteine residues by carbamidomethylation was set as obligate, methionine oxidation was considered as a potential modification; the maximum number of missed tryptic cleavages was one; the monoisotopic masses were considered and the mass tolerance was set to ± 50 ppm, and the fragment-ion mass tolerance was set to 0.2 Da (MS/MS). A protein was accepted to be identified when the total protein score reached or exceeded the MASCOT score threshold (≥ 65 with a probability of identification greater 95%). A repeated search against a randomized decoy database (http://www.matrixscience.com/help/decoy_help.html) using the decoy.pl script and identical search parameters let to a false-positive rate of 1.2%.

The classification according the localization and function of individual proteins was based on the Uni-Prot knowledge base, the iHOP database [19] and the iProXpress database [17] available through the Protein Information Resource (PIR) (GUMC, Washington DC, USA). Identified proteins were searched in this organelle-proteome reference dataset according to their Uni-Prot numbers.

## Competing interests

All authors declare that they have no competing interests.

## Authors' contributions

HS and MN performed all experiments regarding cell culture, lysosome enrichment and 2D gel electrophoresis. MLet performed the confocal imaging experiments and was involved in the establishment of the lysosomal purification protocol. RL performed the electron microscopy. CG carried out all mass spectrometrical analyses. HS, MN and CG performed data analyses and assignments. HS, MLei, DK and OJ conceived of the study, and participated in its design and coordination. HS, CG and OJ drafted the manuscript. All authors read and approved the manuscript.

## Supplementary Material

Additional file 1 **Table S1. List of identified spots in enriched SL preparations from activated T cells**. 742 spots representing 397 proteins were identified and annotated according to Figure S1 A-D. Proteins (3) are listed with spot numbers (1), the number of iterant identifications (2), respective NCBI accession (4) and Uni-Prot (5) numbers, theoretical molecular weights (MW) (6) and isoelectric points (pI) (7). In addition, the total MASCOT score (8), matched (9) and unmatched (10) peptides and the sequence coverage (11) are given. The protein function (12), and the subcellular localization (13) of the respective protein are assigned according to PIR, Uni-Prot and iHOP databases. Abbreviations: LY: lysosomes, ME: melanosomes, PL: platelet granules, SL: secretory lysosomes, NG: neuromelanin granules, SY: synaptosomes, EX: exosomes, EN: endosomes, MT: mitochondria, GO: Golgi, PE: peroxisomes, CY: cytoplasm, ER: endoplasmic reticulum and NU: nucleus. Click here for file

Additional file 2 **Figure S1. Proteome map of enriched secretory lysosomes from T cells**. The 742 annotated spots are displayed in four separately enlarged quadrants (A-D) of one representative of the six performed 2D gels. Identifications are combined based on six repetitive experiments. Click here for file

Additional file 3 **Dataset S1. Protein identification data**. Protein identification data are displayed as MASCOT's "Protein View" including matched peptides, sequence coverage and ion scores (for MS/MS identifications). Please use bookmarks for navigation. Click here for file
