# Supplementary material for: Effector granules in human T lymphocytes: the luminal proteome of secretory lysosomes from human T cells
Source: Cell Commun Signal. 2011 Jan 21;9:4. doi: 10.1186/1478-811X-9-4 (PMC3034720; doi:10.1186/1478-811X-9-4)
Supplement: Additional file 1 — Table S1. List of identified spots in enriched SL preparations from activated T cells. 742 spots representing 397 proteins were identified and annotated according to Figure S1 A-D. Proteins (3) are listed with spot numbers (1), the number of iterant identifications (2), respective NCBI accession (4) and Uni-Prot (5) numbers, theoretical molecular weights (MW) (6) and isoelectric points (pI) (7). In addition, the total MASCOT score (8), matched (9) and unmatched (10) peptides and the sequence coverage (11) are given. The protein function (12), and the subcellular localization (13) of the respective protein are assigned according to PIR, Uni-Prot and iHOP databases. Abbreviations: LY: lysosomes, ME: melanosomes, PL: platelet granules, SL: secretory lysosomes, NG: neuromelanin granules, SY: synaptosomes, EX: exosomes, EN: endosomes, MT: mitochondria, GO: Golgi, PE: peroxisomes, CY: cytoplasm, ER: endoplasmic reticulum and NU: nucleus. [file 1478-811X-9-4-S1.PDF]

Additional file 1

Effector granules in T human lymphocytes: the luminal proteome of secretory lysosomes from human T cells.

Hendrik Schmidt[1], Christoph Gelhaus[2], Melanie Nebendahl[1], Marcus Lettau[1], Ralph Lucius[3], Dieter Kabelitz[1] and Ottmar Janssen[1]

[1]Institute of Immunology, Christian-Albrechts-University, UK S-H Campus Kiel, Kiel, Germany
[2]Zophysiology, Zoological Institute, Christian-Albrechts-University, Kiel, Germany
[3]Institute of Anatomy, Christian-Albrechts-University, Kiel, Germany

Table S1. List of identified spots in enriched SL preparations from activated T cells. 742 spots representing 397 proteins were identified and annotated according to Figure S1 A-D. Proteins (3) are listed with spot numbers (1), the number of iterant identifications (2), respective NCBI accession (4) and Uni-Prot (5) numbers, theoretical molecular weights (MW) (6) and isoelectric points (pI) (7). In addition, the total MASCOT score (8), matched (9) and unmatched (10) peptides and the sequence coverage (11) are given. The protein function (12), and the subcellular localization (13) of the respective protein are assigned according to PIR, Uni-Prot and iHOP databases. Abbreviations: LY: lysosomes, ME: melanosomes, PL: platelet granules, SL: secretory lysosomes, NG: neuromelanin granules, SY: synaptosomes, EX: exosomes, EN: endosomes, MT: mitochondria, GO: Golgi, PE: peroxisomes, CY: cytoplasm, ER: endoplasmic reticulum and NU: nucleus.

Table S1. List of identified spots of enriched secretory lysosomes of T cell blasts.

spot no.(1	replicats(2	protein name (3	gene	acc.no.(4	Uni Prot no.(5	MW, Da(6	pI(7	MASCOT (8	matched peptides(9	unmatched peptides (10	% seq. coverage (11	function (12	subcellular localisation (13
8	2	coronin, actin binding protein, 1A	CORO1A	gi|1002923	P31146	51722	6.1	208	12	22	24	cytoskeleton	LY,PL
9	1	moesin, isoform CRA_b	MSN	gi|119625804	P26038	66678	5.9	203	15	25	24	cytoskeleton	EN,ME,EX,PL,MT
15	3	coronin, actin binding protein, 1A	CORO1A	gi|5902134	P31146	51678	6.3	412	25	32	47	cytoskeleton	LY,PL
18	1	ACTB protein	ACTB	gi|15277503	P60709	40536	5.6	265	8	24	31	cytoskeleton	ER,ME,EX,PL,SY
27	3	ACTB protein	ACTB	gi|15277503	P60709	40536	5.6	351	11	19	38	cytoskeleton	ER,ME,EX,PL,SY
32	2	glucosidase, alpha; neutral AB, isoform CRA_a	GANAB	gi|119594451	Q14697	104930	5.9	116	8	15	9	hydrolases	ER,ME,PL
34	2	glucosidase, alpha; neutral AB, isoform CRA_a	GANAB	gi|119594451	Q14697	104930	5.9	95	9	17	12	hydrolases	ER,ME,PL
35	1	neutrophil adherence receptor alpha-M subunit	ITGAM	gi|386975	P11215	127628	6.7	88	7	5	8	adhesion	membrane
36	1	neutrophil adherence receptor alpha-M subunit	ITGAM	gi|386975	P11215	127628	6.7	89	7	5	8	adhesion	membrane
38	2	niban protein isoform 2	FAM129A	gi|16757970	Q9BZQ8	104039	4.7	265	10	14	12	signal trans.	CY
39	2	UDP-glucose ceramide glucosyltransferase-like 1 isoform 1	UGCGL1	gi|9910280	Q9NYU2	177819	5.4	124	10	13	7	chaperone	ER,ME
41	1	UDP-glucose ceramide glucosyltransferase-like 1 isoform 1	UGCGL1	gi|9910280	Q9NYU2	177819	5.4	113	8	9	5	chaperone	ER,ME
43	1	hypoxia up-regulated protein 1	HYOU1	gi|5453832	Q9Y4L1	111494	5.2	488	17	9	19	chaperone	ER,PL,ME
44	1	hypoxia up-regulated protein 1	HYOU1	gi|5453832	Q9Y4L1	111494	5.2	257	13	15	12	chaperone	ER,PL,ME
45	1	protein diaphanous homolog 1	DIAPH1	gi|119582323	O60610	139473	5.3	85	6	8	6	cytoskeleton	ME
47	2	hypoxia up-regulated protein 1	HYOU1	gi|5453832	Q9Y4L1	111494	5.2	537	34	23	35	chaperone	ER,PL,ME
48	1	histocompatibility (minor) HA-1	HMHA1	gi|47834348	Q92619	125848	5.8	105	3	22	3	immunity	unknown
49	3	coronin, actin binding protein, 1A	CORO1A	gi|1002923	P31146	51722	6.1	274	9	12	22	cytoskeleton	LY,PL
52	2	moesin, isoform CRA_b	MSN	gi|119625804	P26038	66678	5.9	642	32	33	50	cytoskeleton	EN,ME,EX,PL,MT
58	1	moesin, isoform CRA_b	MSN	gi|119625804	P26038	66678	5.9	211	17	28	29	cytoskeleton	EN,ME,EX,PL,MT
64	1	phosphoribosylformylglycinamidine synthase	PFAS	gi|119610473	O15067	103226	5.6	79	6	7	6	biosynthesis	CY
67	1	dipeptidyl peptidase 4	DPP4	gi|35336	P27487	88947	5.7	90	7	28	11	hydrolase	ER,LY,EN,ME,EX
69	1	myosin IG	MYO1G	gi|54873627	B0I1T2	117393	9.0	124	7	10	10	trafficking	unknown
70	3	myosin IG	MYO1G	gi|54873627	B0I1T2	117393	9.0	220	11	9	15	trafficking	unknown
71	1	FYN-binding protein	FYB	gi|2078273	O15117	85627	6.1	128	6	6	8	adapter	CY,NU
75	1	myosin IG	MYO1G	gi|54873627	B0I1T2	117393	9.0	617	37	28	42	trafficking	unknown
77	2	dipeptidyl peptidase 4	DPP4	gi|27574040	P27487	85008	5.7	107	7	21	11	hydrolase	ER,LY,EN,ME,EX
79	1	myosin IG	MYO1G	gi|54873627	B0I1T2	117393	9.0	304	15	9	18	trafficking	unknown
80	2	integrin beta-2	ITGB2	gi|119629788	P05107	81553	6.5	215	11	6	17	membrane	PL
81	3	dipeptidyl peptidase 4	DPP4	gi|27574040	P27487	85008	5.7	449	25	33	39	hydrolase	ER,LY,EN,ME,EX
83	3	dipeptidyl peptidase 4	DPP4	gi|50513374	P27487	85693	5.9	296	16	32	22	hydrolase	ER,LY,EN,ME,EX
84	3	dipeptidyl peptidase 4	DPP4	gi|27574040	P27487	85008	5.7	431	19	21	31	hydrolase	ER,LY,EN,ME,EX
85	2	dipeptidyl peptidase 4	DPP4	gi|27574040	P27487	85008	5.7	244	6	8	10	hydrolase	ER,LY,EN,ME,EX
86	3	dipeptidyl peptidase 4	DPP4	gi|110590190	P27487	84797	5.7	185	11	25	16	hydrolase	ER,LY,EN,ME,EX
88	1	dipeptidyl peptidase 4	DPP4	gi|27574040	P27487	85008	5.7	144	3	8	5	hydrolase	ER,LY,EN,ME,EX
89	1	phostensin	KIAA1949	gi|51555766	Q6NYC8	68157	5.4	146	6	13	12	unclassified	CY
90	2	phostensin	KIAA1949	gi|51555766	Q6NYC8	68157	5.4	185	9	17	15	unclassified	CY
91	3	phostensin	KIAA1949	gi|123209015	Q6NYC8	68286	5.4	318	13	15	26	unclassified	CY
92	1	phostensin	KIAA1949	gi|123209015	Q6NYC8	68286	5.4	264	9	16	21	unclassified	CY
93	2	coronin, actin binding protein, 1A	CORO1A	gi|1002923	P31146	51722	6.1	183	12	20	30	cytoskeleton	LY,PL
94	1	ATP citrate lyase isoform 1	ACLY	gi|38569421	P53396	121674	7.0	99	7	8	8	biosynthesis	ME,EX,PL
95	1	phostensin	KIAA1949	gi|123209015	Q6NYC8	68286	5.4	115	7	17	18	unclassified	CY
96	1	ATP citrate lyase	ACLY	gi|603074	P53396	121660	7.0	144	11	15	10	biosynthesis	ME,EX,PL
99	2	ERAP2 protein	ERAP2	gi|40807029	Q6P179	106086	6.4	87	5	22	6	immunity	ER
102	2	leukocyte-derived arginine aminopeptidase long form variant	LRAP	gi|32400649	Q6P179	111061	6.2	151	9	25	9	hydrolases	unknown
104	1	leukocyte-derived arginine aminopeptidase	LRAP	gi|40807029	Q6P179	106086	6.4	67	7	19	7	hydrolases	unknown
106	1	ERAP2 protein	ERAP2	gi|40807029	Q6P179	106086	6.4	79	6	21	7	immunity	ER
108	1	vinculin	VCL	gi|24657579	P18206	117234	5.8	188	4	11	7	cytoskeleton	ME
109	1	alanyl-tRNA synthetase	AARS	gi|109148542	P49588	107485	5.3	93	3	6	3	biosynthesis	ME,PL
111	4	alanyl-tRNA synthetase	AARS	gi|109148542	P49588	107485	5.3	160	4	6	5	biosynthesis	ME,PL
112	2	heat shock protein 70	HSP74	gi|292160	P34932	79858	5.1	119	5	10	9	chaperone	EX
113	3	coronin 7	CORO7	gi|109658548	Q17RK4	101626	5.5	447	24	34	28	trafficking	CY,GO
114	1	coronin 7	CORO7	gi|119605714	B3KSY4	77412	5.0	145	7	19	12	trafficking	CY,GO
118	3	tumor rejection antigen (gp96) 1	ENPL	gi|4507677	P14625	92696	4.8	836	41	24	51	chaperone	ER,ME,PL,GO
119	2	ubiquitin-like modifier-activating enzyme 1	UBA1	gi|24485	P22314	91182	5.8	220	6	14	14	degradation	MT,ME
120	2	ubiquitin-like modifier-activating enzyme 1	UBA1	gi|24485	P22314	91182	5.8	344	7	11	14	degradation	MT,ME
122	3	100 kDa coactivator	SND1	gi|799177	Q7KZF4	100313	6.6	277	24	22	29	biosynthesis	ER,ME,PL
124	1	tumor rejection antigen (gp96) 1	ENPL	gi|15010550	P14625	90309	4.7	282	10	12	14	chaperone	ER,ME,PL,GO
126	2	glucosidase II subunit beta	PRKCSH	gi|48255891	P14314	60110	4.3	168	10	28	28	hydrolases	ER,ME,PL
127	1	ARTS-1	ERAP1	gi|20521069	Q9NZ08	108595	5.9	108	4	7	4	immunity	ER,ME
129	1	glucosidase II subunit alpha	GANAB	gi|2274968	Q14697	107289	5.7	111	14	30	17	hydrolases	ER,ME,PL
130	2	methylenetetrahydrofolate dehydrogenase 1	MTHFD1	gi|13699868	P11586	102152	6.8	100	5	10	6	multifunctional	EN,ME,PL,MT
131	1	glucosidase II subunit alpha	GANAB	gi|577295	Q14697	107158	5.7	78	6	14	8	hydrolases	ER,ME,PL
132	3	glucosidase II subunit alpha	GANAB	gi|2274968	Q14697	107289	5.7	473	32	27	38	hydrolases	ER,ME,PL
133	1	endoplasmic reticulum aminopeptidase 1	ERAP1	gi|119616483	Q9NZ08	107706	5.9	181	9	12	12	immunity	ER,ME
134	2	adipocyte-derived leucine aminopeptidase isoform 2	ERAP1	gi|19879274	Q9NZ08	108264	5.8	93	4	11	5	hydrolase	ER,ME
135	1	moesin, isoform CRA_b	MSN	gi|119625804	P26038	66678	5.9	166	17	26	29	cytoskeleton	EN,ME,EX,PL,MT
136	2	ARTS-1	ERAP1	gi|37182302	Q9NZ08	107645	5.9	222	8	11	11	immunity	ER,ME
137	3	methylenetetrahydrofolate dehydrogenase 1	MTHFD1	gi|13699868	P11586	102152	6.8	288	25	22	33	multifunctional	EN,ME,PL,MT
138	3	glucosidase, alpha; neutral AB, isoform CRA_a	GANAB	gi|119594451	Q14697	104930	5.9	459	31	28	38	hydrolases	ER,ME,PL
139	3	methylenetetrahydrofolate dehydrogenase 1	MTHFD1	gi|13699868	P11586	102152	6.8	297	22	21	28	multifunctional	EN,ME,PL,MT
140	1	actinin, alpha 4	ACTN4	gi|12025678	O43707	105245	5.3	432	25	27	32	cytoskeleton	ME,NU,CY
141	2	Rap1-GTP-interacting adapter molecule	APBB1IP	gi|26000235	Q7Z5R6	73496	5.4	165	8	23	12	signal trans.	CY
143	2	actinin, alpha 4	ACTN4	gi|12025678	O43707	105245	5.3	585	36	28	45	cytoskeleton	ME,NU,CY
144	1	actinin, alpha 4	ACTN4	gi|119577215	O43707	104555	5.2	705	42	23	55	cytoskeleton	ME,NU,CY
146	1	moesin	MSN	gi|50513540	P26038	35010	9.0	251	6	14	17	cytoskeleton	EN,ME,EX,PL,MT
147	5	eukaryotic translation elongation factor 2	EEF2	gi|4503483	P13639	96246	6.4	267	13	17	15	biosynthesis	ER,EN,ME,EX
148	3	dynamin 2 isoform 1	DYN2	gi|56549121	P50570	98345	7.0	157	14	26	18	trafficking	EN
149	1	moesin, isoform CRA_b	MSN	gi|119625804	P26038	66678	5.9	198	19	24	30	cytoskeleton	EN,ME,EX,PL,MT
151	2	eukaryotic translation elongation factor 2	EEF2	gi|4503483	P13639	96246	6.4	98	7	20	10	biosynthesis	ER,EN,ME,EX
152	1	eukaryotic translation elongation factor 2	EEF2	gi|4503483	P13639	96246	6.4	175	13	26	17	biosynthesis	ER,EN,ME,EX
153	1	tumor rejection antigen (gp96) 1	ENPL	gi|4507677	P14625	92696	4.8	480	22	13	26	chaperone	ER,ME,PL,GO
154	2	ubiquitin specific peptidase 5 isoform 2	UBP5	gi|148727247	P45974	94104	5.0	114	6	14	11	degradation	LY,ME,NG
155	1	extended-synaptotagmin-1 KIAA0747 protein	ESYT1	gi|3882215	Q9BSJ8	120296	5.7	84	5	18	6	unknown	ME
156	1	extended-synaptotagmin-1 KIAA0747 protein	ESYT1	gi|3882215	Q9BSJ8	120296	5.7	66	3	7	3	unknown	ME
157	1	moesin, isoform CRA_b	MSN	gi|119625804	P26038	66678	5.9	315	29	33	51	cytoskeleton	EN,ME,EX,PL,MT
158	3	eukaryotic translation elongation factor 2	EEF2	gi|4503483	P13639	96246	6.4	349	22	22	29	biosynthesis	ER,EN,ME,EX
159	1	valosin-containing protein	VCP	gi|48257098	Q96IF9	71534	4.9	207	7	9	17	unknown	unknown
162	1	moesin, isoform CRA_b	MSN	gi|119625804	P26038	66678	5.9	646	30	30	48	cytoskeleton	EN,ME,EX,PL,MT
163	1	moesin, isoform CRA_b	MSN	gi|119625804	P26038	66678	5.9	546	25	24	42	cytoskeleton	EN,ME,EX,PL,MT
164	1	importin subunit beta-1	KPNB1	gi|119615215	Q14974	95094	4.7	148	11	21	20	transport	ME
165	1	26S proteasome non-ATPase regulatory subunit 2	PSMD2	gi|2134674	Q13200	99994	5.1	121	4	10	6	proteasome	ME
167	2	MHC class I antigen	HLA-A	gi|51100948	Q6AW25	31659	5.7	282	13	25	60	Immunity	ME
171	1	PDCD6IP protein	PDCD6IP	gi|46249756	Q6NUS1	97385	6.1	290	21	25	32	unclassified	unknown
174	1	moesin, isoform CRA_b	MSN	gi|119625804	P26038	66678	5.9	92	10	29	18	cytoskeleton	EN,ME,EX,PL,MT
177	1	heat shock protein HSP 90-beta	HS90B	gi|20149594	P08238	83554	5.0	299	20	33	29	chaperone	ME,EX,MT
178	1	phospholipase C, delta 1 variant	nd	gi|62089310	Q59EE9	88289	6.4	118	7	28	11	signal trans.	unknown
179	1	ezrin	EZR	gi|31283	P15311	69470	5.9	148	12	22	17	cytoskeleton	ME,EX,MT
180	1	signal transducer and activator of transcription 1, 91kDa, isoform CRA_c	STAT1	gi|119631258	P42224	82379	6.1	181	5	4	7	signal trans.	CY,NU
181	3	hematopoietic cell-specific Lyn substrate 1	HCLS1	gi|48145705	P14317	54065	4.7	397	19	36	44	signal trans.	CY,MT

spot no.[1]	replicats[2]	protein name [3]	gene	acc.no.[4]	Uni Prot no.[5]	MW, Da[6]	pI[7]	MASCOT [8]	matched peptides[9]	unmatched peptides [10]	% seq. coverage [11]	function [12]	subcellular localisation [13]
182	1	signal transducer and activator of transcription 1, 91kDa, isoform CRA_d	STAT1	gi|62738788	P42224	80465	6.3	114	9	21	14	signal trans.	CY,NU
184	1	hematopoietic cell-specific Lyn substrate 1	HCLS1	gi|119599912	P14317	57793	4.8	163	8	23	19	signal trans.	CY,MT
185	2	ACTB protein	ACTB	gi|15277503	P60709	40536	5.6	228	5	11	18	cytoskeleton	ER,ME,EX,PL,SY
187	1	hematopoietic cell-specific Lyn substrate 1	HCLS1	gi|48145705	P14317	54065	4.7	91	4	30	9	signal trans.	CY,MT
188	4	signal transducer and activator of transcription 1, 91kDa, isoform CRA_d	STAT1	gi|119631259	P42224	87076	5.8	191	10	23	13	signal trans.	CY,NU
189	1	signal transducer and activator of transcription 1, 91kDa, isoform CRA_d	STAT1	gi|62738788	P42224	80465	6.3	78	8	22	13	signal trans.	CY,NU
191	1	beta adrenergic receptor kinase 1	ADRBK1	gi|148539876	P25098	80321	6.9	70	9	25	14	GTPases	CY
193	1	raftlin cell migration-inducing gene 2	RFTN1	gi|37930520	Q14699	63691	5.5	120	9	21	22	unclassified	PL
194	1	hexose-6-phosphate dehydrogenase	H6PD	gi|52145310	O95479	89407	6.8	106	13	26	19	metabolism	ER
196	3	phosphofructokinase, platelet	PFKP	gi|119606901	Q01813	93725	8.6	262	20	26	26	glycolysis	ME,PL
197	2	ERBB2IP protein	LAP2	gi|29791785	Q96RT1	72831	4.8	149	5	7	9	multifunctional	NU,CY
199	3	acyl-CoA synthetase long-chain family member 4 isoform 2	ACSL4	gi|12669909	O60488	80220	8.7	232	18	25	31	metabolism	MT,PE,ME
202	1	Sec23 homolog A	SC23A	gi|22477159	Q15436	87022	6.6	91	8	22	14	trafficking	ER,ME,PL
203	5	acylamino acid-releasing enzyme	ACPH	gi|556514	P13798	82210	5.3	178	10	25	18	hydrolase	PL, CY
206	1	ezrin	EZR	gi|46249758	Q6NUR7	69313	5.9	387	28	34	41	cytoskeleton	CY
207	4	ezrin	EZR	gi|46249758	Q6NUR7	69313	5.9	430	28	22	40	cytoskeleton	CY
208	3	ezrin	EZR	gi|46249758	Q6NUR7	69313	5.9	569	29	21	42	cytoskeleton	CY
209	1	interleukin-16	IL16	gi|27262655	Q14005	66948	5.7	299	7	13	20	Immunity	secreted
210	5	interleukin-16	IL16	gi|27262655	Q14005	66948	5.7	406	18	19	40	Immunity	secreted
211	3	leucine-rich repeat and calponin homology domain-containing protein 4	LRCH4	gi|119596926	O75427	70861	6.9	259	14	20	28	protein binding	MT
212	1	heat shock 70kDa protein 5	GRP78	gi|16507237	P11021	72402	5.1	157	11	23	24	chaperone	ER,ME,EX,PL,MT
213	2	leucine-rich repeat and calponin homology domain-containing protein 4	LRCH4	gi|119596926	O75427	70861	6.9	152	6	13	14	protein binding	MT
214	1	centaurin beta1	ACAP1	gi|7661880	Q15027	82397	7.6	142	10	25	16	GTPase	unknown
215	1	Sec23B protein	SC23B	gi|13529299	Q15437	87377	6.4	65	9	28	17	trafficking	EN
216	1	hypothetical protein	nd	gi|62896583	Q53HR8	71794	6.1	330	16	19	30	unclassified	unknown
217	2	interleukin-16	IL16	gi|2114410	Q9UME6	67047	5.7	285	5	8	13	Immunity	secreted
218	2	interleukin-16	IL16	gi|2114410	Q9UME7	67047	5.7	283	5	10	13	Immunity	secreted
220	2	beta adrenergic receptor kinase 1	ADRBK1	gi|148539876	P25098	80321	6.9	247	18	16	24	GTPases	CY
221	2	Sec23 homolog A	SC23A	gi|22477159	Q15436	87022	6.6	228	8	9	15	trafficking	ER,ME,PL
223	1	heat shock 70kDa protein 5	GRP78	gi|386758	P11021	72185	5.0	599	25	14	41	chaperone	ER,ME,EX,PL,MT
225	1	heat shock 70kDa protein 5	GRP78	gi|386758	P11021	72185	5.0	551	27	17	46	chaperone	ER,ME,EX,PL,MT
226	1	heat shock 70kDa protein 5	GRP78	gi|16507237	P11021	72402	5.1	720	32	24	53	chaperone	ER,ME,EX,PL,MT
228	1	differentially expressed in FDCP 6 homolog (mouse), isoform CRA_b	DEF6	gi|119624227	B3KSI1	44508	6.6	103	5	8	15	unknown	unknown
229	2	lymphocyte cytosolic protein 2	LCP2	gi|119581882	Q13094	59922	6.3	142	7	23	15	immunity	CY
234	2	prolyl endopeptidase	PPCE	gi|41349456	P48147	81560	5.5	125	6	8	11	proteolysis	CY
237	2	moesin, isoform CRA_b	MSN	gi|119625804	P26038	66678	5.9	650	30	30	48	cytoskeleton	EN,ME,EX,PL,MT
238	1	L-plastin variant	nd	gi|62898171	Q53FI1	70785	5.2	348	20	37	37	cytoskeleton	unknown
239	2	septin-9 gamma	SEPT9	gi|14530105	Q9UHD8	64894	7.2	201	11	13	18	unclassified	ME
240	1	moesin	MSN	gi|4505257	P26038	67892	6.1	354	23	25	37	cytoskeleton	EN,ME,EX,PL,MT
241	2	moesin, isoform CRA_b	MSN	gi|119625804	P26038	66678	5.9	573	33	32	46	cytoskeleton	EN,ME,EX,PL,MT
242	2	moesin, isoform CRA_b	MSN	gi|119625804	P26038	66678	5.9	459	29	27	42	cytoskeleton	EN,ME,EX,PL,MT
244	1	glycyl-tRNA synthetase	SYG	gi|116805340	P41250	83854	6.6	246	15	25	23	biosynthesis	ME
246	2	moesin, isoform CRA_b	MSN	gi|119625804	P26038	66678	5.9	718	35	30	49	cytoskeleton	EN,ME,EX,PL,MT
247	4	moesin	MSN	gi|4505257	P26038	67892	6.1	566	35	30	57	cytoskeleton	EN,ME,EX,PL,MT
248	3	moesin	MSN	gi|14625824	P26038	62004	7.6	329	8	7	16	cytoskeleton	EN,ME,EX,PL,MT
249	1	moesin	MSN	gi|14625824	P26038	62004	7.6	268	7	10	14	cytoskeleton	EN,ME,EX,PL,MT
250	1	moesin	MSN	gi|14625824	P26038	62004	7.6	361	15	12	29	cytoskeleton	EN,ME,EX,PL,MT
251	1	moesin	MSN	gi|8569616	P26038	34554	8.9	130	11	54	34	cytoskeleton	EN,ME,EX,PL,MT
253	1	mitochondrial trifunctional protein, alpha subunit precursor	ECHA	gi|20127408	P40939	83688	9.2	126	5	8	7	metabolism	PL, MT
256	1	poly(A) binding protein, cytoplasmic 1, isoform CRA_c	PABPC1	gi|119612222	P11940	47647	9.0	270	5	6	15	metabolism	ER,EN,ME,PL
259	2	heat shock 70kDa protein 8 isoform 1	nd	gi|5729877	Q53GZ6	71082	5.4	517	23	29	46	chaperone	LY,ME,NG,EX,PL,SY,MT
260	1	heat shock 70kDa protein 8 isoform 1	nd	gi|5729877	Q53GZ6	71082	5.4	382	19	28	37	chaperone	LY,ME,NG,EX,PL,SY,MT
261	1	annexin A6	ANXA6	gi|35218	P08133	76154	5.4	353	28	23	48	trafficking	MT,EX,ME,ER
262	2	annexin A6	ANXA6	gi|35218	P08133	76154	5.4	524	39	26	63	trafficking	MT,EX,ME,ER
263	2	annexin A6	ANXA6	gi|35218	P08133	76154	5.4	552	37	17	59	trafficking	MT,EX,ME,ER
264	1	syntaxin binding protein 3	STXBP3	gi|2810993	O00186	68386	8.3	170	6	32	13	trafficking	ME,PL
265	1	lysosomal acid alpha-mannosidase	MAN2B1	gi|1658374	O00754	111986	6.7	77	3	9	3	hydrolases	LY,ME
266	1	L-plastin	LCP1	gi|4504965	P13796	70815	5.2	838	33	32	65	actin binding	CY
267	1	L-plastin variant	nd	gi|62898171	Q53FI1	70785	5.2	427	24	28	41	cytoskeleton	unknown
268	2	L-plastin	LCP1	gi|4504965	P13796	70815	5.2	585	27	25	53	actin binding	CY
269	1	L-plastin	LCP1	gi|4504965	P13796	70815	5.2	440	28	33	48	actin binding	CY
273	2	alkyldihydroxyacetonephosphate synthase, peroxisomal	AGPS	gi|119631462	O00116	60804	6.0	172	6	8	15	biosynthesis	PE
274	2	zeta-chain associated protein kinase 70kDa isoform 1	ZAP70	gi|31455611	P43403	70796	7.8	161	5	9	10	signal trans.	CY
275	1	L-plastin	LCP1	gi|4504965	P13796	70815	5.2	544	27	30	50	actin binding	CY
276	5	transketolase	TKT	gi|31417921	P29401	50335	8.0	376	12	26	38	unclassified	ME,EN
277	2	zeta-chain associated protein kinase 70kDa	ZAP70	gi|149242795	P43403	70122	7.8	363	22	17	46	signal trans.	CY
278	2	heat shock 70kDa protein 1A	HSPA1A	gi|5123454	P08107	70280	5.5	422	24	33	39	chaperone	ER,EN,ME,EX,MT
280	1	perforin-1	PERF	gi|35386	P14222	62495	8.1	163	6	8	13	Immunity	SL
281	2	syntaxin binding protein 3	STXBP3	gi|118600975	O00186	68633	8.0	198	8	15	17	trafficking	ME,PL
284	1	Wiskott-Aldrich syndrome protein	WAS	gi|4507909	P42768	53108	6.2	148	3	12	7	cytoskeleton	CY
286	3	calreticulin precursor variant	CALR	gi|62897681	P27797	47061	4.3	351	14	22	36	chaperone	ER,ME,NG,EX,PL,MT
287	5	calreticulin precursor variant	CALR	gi|62897681	P27797	47061	4.3	458	15	19	37	chaperone	ER,ME,NG,EX,PL,MT
291	1	syntaxin binding protein 3, isoform CRA_b	STXBP3	gi|119576735	O00186	38947	8.9	121	2	7	10	trafficking	ME,PL
292	3	MAGUK p55 subfamily member 7	MPP7	gi|55958705	Q5T2T1	36102	9.1	246	6	9	21	protein binding	PL
294	1	syntaxin binding protein 3 variant	STXBP3	gi|62897901	Q53FW1	68599	8.0	321	17	18	28	trafficking	ME,PL
297	1	WD repeat-containing protein 1 isoform 1 variant	WDR1	gi|62897087	Q53H17	66870	6.2	257	18	30	35	cytoskeleton	EN,ME,EX
298	2	WD repeat-containing protein 1 isoform 1 variant	WDR1	gi|62897087	Q53H17	66870	6.2	495	19	28	39	cytoskeleton	EN,ME,EX
299	2	WD repeat-containing protein 1 isoform 1 variant	WDR1	gi|62897087	Q53H17	66870	6.2	296	18	21	35	cytoskeleton	EN,ME,EX
300	2	WD repeat domain 1, isoform CRA_d	WDR1	gi|119613095	O75083	58579	6.4	176	9	17	21	cytoskeleton	EN,ME,EX
302	4	syntaxin binding protein 2	STXBP2	gi|12804033	Q9BU65	66553	6.1	616	28	28	55	trafficking	EX,PL
303	1	WD repeat-containing protein 1 isoform 1	WDR1	gi|9257257	O75083	66836	6.2	150	15	24	31	cytoskeleton	EN,ME,EX
304	1	WD repeat domain 1	WDR1	gi|12652891	O75083	66822	6.2	657	25	21	59	cytoskeleton	EN,ME,EX
305	1	syntaxin binding protein 1	STXBP1	gi|62087940	P61764	67568	6.5	166	8	11	21	trafficking	ME,NG,EX,PL,SY
306	2	asparaginyl-tRNA synthetase	NARS	gi|119583453	O43776	63687	5.9	167	3	6	6	biosynthesis	ME
307	1	T-complex protein 1 subunit gamma	CCT3	gi|671527	P49368	60862	6.2	223	10	23	20	chaperone	CY
308	1	ERO1L	ERO1L	gi|6272557	Q96HE7	45319	5.4	169	10	24	33	redox proteins	ME,ER
309	3	leukotriene A4 hydrolase	LTA4H	gi|119617964	P09960	63481	5.7	193	5	7	14	hydrolases	CY
310	1	copine I	CPNE1	gi|48145697	Q99829	59677	5.5	232	10	27	19	trafficking	ME
311	2	ERO1L	ERO1L	gi|119586051	Q96HE7	54839	5.6	348	15	23	36	redox proteins	ME,ER
312	2	ERO1L	ERO1L	gi|119586051	Q96HE7	54839	5.6	304	14	25	36	redox proteins	ME,ER
313	2	dihydropyrimidinase-like 2	DPYL2	gi|4503377	Q16555	62711	6.0	206	5	11	13	signal trans.	SY
315	1	nucleosome assembly protein 1-like 1, isoform CRA_d	NAP1L1	gi|119617726	P55209	40154	4.6	112	3	16	11	cell proliferation	ME,PL,NU
316	2	phosphatase 2a	PPP2R1A	gi|149243188	P30153	65232	5.1	227	12	15	26	multifunctional	MT
317	1	protein tyrosine phosphatase, non-receptor type 6 isoform 1 variant	nd	gi|62898990	Q53EL0	67966	7.7	202	15	28	32	hydrolases	unknown
318	1	soc-2 suppressor of clear homolog	SHOC2	gi|41281398	Q9UQ13	65304	8.7	105	3	5	5	unclassified	CY
319	2	dihydropyrimidinase-like 2	DPYL2	gi|4503377	Q16555	62711	6.0	479	17	19	40	signal trans.	SY

spot no.[1]	replicats[2]	protein name [3]	gene	acc.no.[4]	Uni Prot no.[5]	MW, Da[6]	pI[7]	MASCOT [8]	matched peptides[9]	unmatched peptides [10]	% seq. coverage [11]	function [12]	subcellular localisation [13]
320	1	copine I variant	CPNE1	gi|62089240	Q59EI4	57352	5.5	144	6	14	11	trafficking	ME
321	1	Wiskott-Aldrich syndrome protein	WAS	gi|4507909	P42768	53108	6.2	141	5	13	13	cytoskeleton	CY
322	1	vacuolar protein sorting 45A	VPS45	gi|18105063	Q9NRW7	65435	8.4	284	6	5	11	trafficking	LY,EN
323a	1	stress-induced-phosphoprotein 1 (Hsp70/Hsp90-organizing protein)	STIP1	gi|5803181	P31948	63227	6.4	156	17	25	28	response to stress	ME,PL,SY
323b	1	Wiskott-Aldrich syndrome protein	WAS	gi|12804207	P42768	54422	6.4	191	6	14	18	cytoskeleton	CY
324	1	tyrosine-protein phosphatase non-receptor type 6	PTN6	gi|557900	P29350	67924	7.3	95	5	9	9	signal trans.	CY,NU
325	2	tyrosine-protein phosphatase non-receptor type 6	PTN6	gi|82407989	P29350	60638	6.1	115	10	25	23	signal trans.	CY,NU
326	2	bifunctional purine biosynthesis protein PURH	ATIC	gi|119590931	P31939	65075	6.3	401	23	18	44	mutifunctional	ME,PL
327	3	Wiskott-Aldrich syndrome protein	WAS	gi|12804207	P42768	54422	6.4	174	4	11	13	cytoskeleton	CY
328	1	adenylyl cyclase-associated protein variant	CAP1	gi|62896585	Q01518	51899	8.1	103	5	27	18	trafficking	PL,ME
329	1	ezrin	EZR	gi|340217	P15311	68233	5.8	94	8	25	11	cytoskeleton	ME,EX,MT
331	1	T-complex polypeptide 1	TCPA	gi|36796	P17987	60869	6.0	162	6	11	10	chaperone	ER,EN,ME,EX
332	1	DCHT2 Serine/threonine-protein kinase OSR1	OXSR1	gi|33150628	O95747	32518	6.9	172	5	6	21	kinase	ME
335	2	nucleobindin 1 variant	Q53GX6	gi|62897169	Q53GX6	53874	5.2	482	22	24	46	unclassified	unknown
337	1	prolyl 4-hydroxylase, alpha subunit	P4HA1	gi|190786	P13674	61075	5.7	85	2	11	6	redox	ER,ME
338	2	chaperonin (HSP60)	CH60	gi|306890	P10809	61157	5.7	413	21	21	43	chaperone	ME,NG,EX,SY,MT
339	3	chaperonin containing TCP1, subunti 5ε	CCT5	gi|24307939	P48643	60089	5.5	262	15	17	32	chaperone	EX,PL
340	1	copine III	CPNE3	gi|4503015	O75131	60947	5.6	93	4	19	8	trafficking	ME,EX,PL
341	1	EH-domain containing 1	EHD1	gi|30240932	Q9H4M9	60646	6.4	236	13	23	26	trafficking	ER,LY,EN,EX,PL
342	4	EH-domain containing 1	EHD1	gi|119594723	Q9H4M9	61945	6.3	420	17	13	30	trafficking	ER,LY,EN,EX,PL
343	2	pyruvate kinase 3 isoform 2	PKM2	gi|67464392	P14618	60277	8.2	480	20	22	48	metabolism	ME,EX,SY
344	2	ubiquitin specific protease 14 isoform a	UBP14	gi|4827050	P54578	56489	5.2	115	4	6	12	degradation	PL
345	2	prolyl 4-hydroxylase, beta subunit precursor	P4HB	gi|20070125	P07237	57480	4.8	519	26	20	45	redox	ER,ME,EX,PL,MT
346	1	pyruvate kinase 3 isoform 2	PKM2	gi|67464392	P14618	60277	8.2	677	26	29	62	metabolism	ME,EX,SY
347	2	pyruvate kinase 3 isoform 2	PKM2	gi|119598292	P14618	60773	8.0	335	15	13	32	metabolism	ME,EX,SY
348	2	prolyl 4-hydroxylase, beta subunit precursor	P4HB	gi|20070125	P07237	57480	4.8	653	31	25	57	redox	ER,ME,EX,PL,MT
349	2	prolyl 4-hydroxylase, beta subunit precursor	P4HB	gi|20070125	P07237	57480	4.8	448	17	19	36	redox	ER,ME,EX,PL,MT
350	1	pyruvate kinase 3 isoform 2	PKM2	gi|67464392	P14618	60277	8.2	433	16	13	37	metabolism	ME,EX,SY
351	2	WD repeat domain 1	WDR1	gi|119613095	O75083	58579	6.4	354	7	10	17	cytoskeleton	EN,ME,EX
352	2	serine/threonine-protein kinase PAK 2	PAK2	gi|984305	Q13177	55166	6.0	196	3	10	11	signal trans.	PL
353	4	coronin, actin binding protein, 1C	CORO1C	gi|62897707	Q9ULV4	53931	6.7	265	7	11	17	multifunctional	ME
354	2	pyruvate kinase 3 isoform 2	PKM2	gi|33286420	P14618	58538	7.6	368	21	22	43	metabolism	ME,EX,SY
355	1	catalase	CATA	gi|179950	P04040	51550	7.8	105	5	9	13	metabolism	PE,ER,LY,EN,ME
356	2	M2-type pyruvate kinase	KPYM	gi|189998	unknown	58447	8.0	212	4	9	12	metabolism	ME,EX,SY
357	1	chaperonin containing TCP1, subunti 8τ	CCT8	gi|48762932	P50990	60153	5.4	314	7	6	15	chaperone	EN,ME,EX
358	1	chaperonin containing TCP1, subunti 8τ	CCT8	gi|48762932	P50990	60153	5.4	183	5	5	13	chaperone	EN,ME,EX
360	2	coronin, actin binding protein, 1A	CORO1A	gi|5902134	P31146	51678	6.3	360	15	29	37	cytoskeleton	LY,PL
361	2	catalase	CATA	gi|4557014	P04040	59947	6.9	274	11	27	29	metabolism	PE,ER,LY,EN,ME
362	2	alpha-tubulin	TBA1B	gi|340021	P68363	50804	4.9	479	15	30	45	cytoskeleton	PL
363	1	tubulin alpha 6 variant	nd	gi|62897609	Q53GA7	50476	5.0	460	12	11	34	cytoskeleton	ME,PL
364	1	PPP5C protein	Q9BPW0	gi|37589898	Q9BPW0	55632	5.8	223	4	6	11	hydrolase	CY,NU
365	2	seryl-tRNA synthetase	SARS	gi|119576769	P49591	53844	6.0	103	6	17	15	tRNA processing	ME,PL
366	3	tyrosyl-tRNA synthetase	SYYC	gi|62898948	P54577	59467	6.8	127	8	12	18	signal trans.	ME,PL
368	3	pyruvate kinase 3 isoform 2	PKM2	gi|33286420	P14618	58538	7.6	327	16	24	37	metabolism	ME,EX,SY
370	2	alpha-tubulin	TBA1B	gi|340021	P68363	50804	4.9	447	16	19	49	cytoskeleton	PL
371	2	coronin, actin binding protein, 1A	CORO1A	gi|5902134	P31146	51678	6.3	485	21	29	42	cytoskeleton	LY,PL
372	2	coronin, actin binding protein, 1A	CORO1A	gi|5902134	P31146	51678	6.3	476	17	23	40	cytoskeleton	LY,PL
373	1	protein-tyrosine kinase fyn isoform c	FYN	gi|23510364	P06241	54821	5.9	331	14	12	27	signal trans.	EN,CY
374	2	coronin, actin binding protein, 1A	CORO1A	gi|1002923	P31146	51722	6.1	145	10	31	22	cytoskeleton	LY,PL
375	1	coronin, actin binding protein, 1A	CORO1A	gi|1002923	P31146	51722	6.1	257	15	17	35	cytoskeleton	LY,PL
376	3	chaperonin containing TCP1, subunit 7η	CCT7	gi|62896515	Q53HV2	59816	7.6	237	7	14	19	chaperone	EN,ME,PL
377	1	HIP-55	DBNL	gi|48146451	Q9UJU6	48449	5.0	191	13	42	28	signal trans.	CY
378	1	protein disulfide-isomerase A3	PDIA3	gi|119597640	P30101	54454	6.8	138	4	15	9	chaperone	ER,LY,ME,NG,EX,PL
379	4	protein disulfide-isomerase A3	PDIA3	gi|119597640	P30101	54454	6.8	443	12	10	28	chaperone	ER,LY,ME,NG,EX,PL
381	3	protein disulfide-isomerase A3	PDIA3	gi|119597640	P30101	54454	6.8	190	9	17	20	chaperone	ER,LY,ME,NG,EX,PL
385	2	protein disulfide-isomerase A3	PDIA3	gi|114656687	P30101	55328	6.4	443	25	31	54	chaperone	ER,LY,ME,NG,EX,PL
386	3	phosphoglucose isomerase	G6PI	gi|14488680	P06744	63204	8.4	201	16	28	35	multifunctional	ME,EX,PL,MT
388	1	tubulin, beta	TBB5	gi|18088719	P07437	50096	4.8	466	21	27	52	cytoskeleton	ME,PL,SY
389	2	protein disulfide-isomerase A3	PDIA3	gi|114656687	P30101	55328	6.4	298	14	20	30	chaperone	ER,LY,ME,NG,EX,PL
390	3	phosphoglucose isomerase	G6PI	gi|14488680	P06744	63204	8.4	378	21	23	47	multifunctional	ME,EX,PL,MT
392	1	ACTB protein	ACTB	gi|15277503	P60709	40536	5.6	271	9	31	34	cytoskeleton	ER,ME,EX,PL,SY
393	1	phosphoglucose isomerase	G6PI	gi|14488680	P06744	63204	8.4	136	11	21	23	multifunctional	ME,EX,PL,MT
394	3	adenylyl cyclase-associated protein variant	CAP1	gi|62896585	Q01518	51899	8.1	289	8	24	36	trafficking	PL,ME
395	2	adenylyl cyclase-associated protein variant	CAP1	gi|62896585	Q01518	51899	8.1	755	18	25	58	trafficking	PL,ME
396	2	tyrosine kinase LCK	LCK	gi|6984209	P06239	56959	5.3	272	6	14	15	signal trans.	CY
397	4	adenylyl cyclase-associated protein variant	CAP1	gi|62896585	Q01518	51899	8.1	644	21	23	64	trafficking	PL,ME
398	2	moesin	MSN	gi|14625824	P26038	62004	7.6	368	11	11	19	cytoskeleton	EN,ME,EX,PL,MT
399	2	tyrosine kinase LCK	LCK	gi|187034	P06239	58574	5.3	533	19	22	53	signal trans.	CY
400	1	annexin A11	ANX11	gi|119600805	P50995	26961	6.9	130	5	11	27	trafficking	MT,EX,ME
401	2	tyrosine kinase LCK	LCK	gi|6984209	P06239	56959	5.3	533	17	23	43	signal trans.	CY
402	1	ACTB protein	ACTB	gi|15277503	P60709	40536	5.6	294	8	15	31	cytoskeleton	ER,ME,EX,PL,SY
403	2	adenylyl cyclase-associated protein	CAP1	gi|5453595	Q01518	51926	7.6	193	3	15	10	trafficking	PL,ME
404	5	adenylyl cyclase-associated protein variant	CAP1	gi|62896585	Q01518	51899	8.1	501	17	22	53	trafficking	PL,ME
406	2	adenylyl cyclase-associated protein	CAP1	gi|119627645	Q01518	51357	7.6	183	3	14	10	trafficking	PL,ME
407	2	tubulin, beta	TBB5	gi|18088719	P07437	50096	4.8	659	26	25	66	cytoskeleton	ME,PL,SY
408	2	3-phosphoglycerate dehydrogenase	PHGDH	gi|5771523	O43175	57370	6.3	411	15	21	38	biosynthesis	ME
409	3	glucose-6-phosphate dehydrogenase isoform b	G6PD	gi|108773793	P11413	59675	6.4	490	26	37	55	metabolism	ME
411	4	transfer RNA-Trp synthetase	SYWC	gi|340368	P23381	53396	5.7	480	16	13	46	biosynthesis	ME,PL
412	1	glucose-6-phosphate dehydrogenase	G6PD	gi|66361514	P11413	56688	6.7	418	15	15	38	metabolism	ME
413	2	ATPase, H+ transporting, lysosomal 56/58kDa, V1 subunit B2	ATP6V1B2	gi|21040528	P21281	56735	5.7	237	7	9	17	channel	LY,ME,NG,SY
414	2	vacuolar H+-ATPase 56,000 subunit	VATB2	gi|522193	P21281	56792	5.6	341	14	16	38	channel	LY,ME,NG,SY
415	4	annexin A11	ANX11	gi|4557317	P50995	54697	7.5	558	20	10	38	trafficking	MT,EX,ME
417	3	src kinase associated phosphoprotein 1 isoform 1	SKAP1	gi|115527074	Q86WV1	41692	4.5	211	6	9	18	signal trans.	CY,NU
418	1	aging-associated gene 12	Q2TSB6	gi|54632179	Q2TSB6	50022	6.9	165	6	12	15	unclassified	unknown
420	2	coronin, actin binding protein, 1A	CORO1A	gi|5902134	P31146	51678	6.3	420	15	24	34	cytoskeleton	LY,PL
421	2	sorting nexin 17	SNX17	gi|62896645	Q15036	53121	7.1	331	15	22	25	tranport	EN,ME,PL
422	1	HEXA protein	HEXA	gi|33876034	Q9BVJ8	47407	4.9	69	4	9	10	multifunctional	LY
424	2	chaperonin containing TCP1, subunit 2β	CCT2	gi|5453603	P78371	57794	6.0	421	17	24	42	chaperone	ER,EN,ME,PL,MT,CY
426	2	ATP synthase, alpha subunit precursor	ATP5A1	gi|4757810	P25705	59828	9.2	412	21	30	46	channel	ER,LY,NG,SY,MT
428	1	Rho GTPase activating protein 1	RHG01	gi|4757766	Q07960	50461	5.9	108	4	10	11	GTPases	PL
429	2	UDP-glucose pyrophosphorylase 2	UGP2	gi|62702281	Q53QE9	49362	8.8	173	6	9	16	metabolism	EN,ME
430	2	glutamate carboxypeptidase	CNDP2	gi|8922699	Q96KP4	53161	5.7	408	13	21	34	hydrolases	unknown
431	1	ENC-1AS aka Beta-hexosaminidase subunit beta	HEXB	gi|55274010	Q5URX0	38519	6.0	119	3	8	9	multifunctional	LY
432	3	leucine aminopeptidase 3	LAP3	gi|37588925	P28838	54754	6.8	330	17	25	39	proteolysis	CY
433	1	tubulin, beta polypeptide	TUBB	gi|57209813	Q5JP53	48135	4.7	161	13	22	33	cytoskeleton	ME,PL,SY
434	1	NESH protein	ABI3	gi|7705981	Q9P2A4	39293	5.0	221	5	8	19	unclassified	unknown
435	1	cytosolic non-specific dipeptidase	CNDP2	gi|119586960	Q96KP4	19432	6.1	109	2	11	10	proteolysis	CY
437	3	glutamate Dehydrogenase-Apo Form	GLUD1	gi|20151189	P00367	56315	6.7	276	10	12	29	unclassified	ER,ME,PL,MT
439	3	adenylosuccinate lyase	PUR8	gi|3211984	P30566	48981	7.9	155	7	12	21	biosynthesis	CY,PL
441	4	Rho GTPase activating protein 1	RHG01	gi|4757766	Q07960	50461	5.9	416	14	13	39	GTPases	PL

spot no.[1]	replicats[2]	protein name [3]	gene	acc.no.[4]	Uni Prot no.[5]	MW, Da[6]	pI[7]	MASCOT [8]	matched peptides[9]	unmatched peptides [10]	% seq. coverage [11]	function [12]	subcellular localisation [13]
442	3	UDP-glucose pyrophosphorylase 2 isoform b	UGP2	gi|48255968	Q16851	55813	7.7	321	12	11	28	metabolism	EN,ME
443	2	mitochondrial ATP synthase, H+ transporting F1 complex beta subunit	ATP5B	gi|89574029	Q0QEN7	48083	5.0	517	17	25	55	transport	MT
444	2	Ena-VASP-like B6	EVL	gi|33150556	Q9UI08	44905	8.9	158	4	11	13	cytoskeleton	CY
445	3	Ena-VASP-like protein	EVL	gi|6563206	Q9UI08	44696	8.9	323	19	28	51	cytoskeleton	CY
446	2	Ena-VASP-like, isoform CRA_b	EVL	gi|119602091	Q9UI08	40952	9.6	171	4	12	12	cytoskeleton	CY
447	4	Ena-VASP-like protein	EVL	gi|25090276	Q9UI08	44706	8.9	393	16	16	53	cytoskeleton	CY
448	2	lymphocyte-specific protein 1	LSP1	gi|12804709	P33241	37427	4.7	550	15	43	59	Immunity	PL
449	3	UPF0550 protein C7orf28	C7orf28B	gi|37674289	O95766	56286	6.1	124	5	10	12	unknown	ME
450	1	UPF0550 protein C7orf28	C7orf28B	gi|37674289	O95766	56286	6.1	209	7	10	15	unknown	ME
451	1	solute carrier family 9 (sodium/hydrogen exchanger), isoform 3 regulator 1	SLC9A3R1	gi|4759140	O14745	39130	5.6	76	2	16	12	scaffolding	ME,EX,PL
453	1	phosphatidylinositol-5-phosphate 4-kinase, type II, alpha	PI42A	gi|6857820	P48426	46424	6.5	221	9	21	23	metabolism	NG,PL
454	3	phosphatidylinositol-5-phosphate 4-kinase, type II, alpha	PI42A	gi|6857820	P48426	46424	6.5	184	2	8	7	metabolism	NG,PL
455	3	docking protein 2	DOK2	gi|41406050	O60496	45750	5.8	262	6	8	18	unknown	unknown
456	5	testin isoform 1	TES	gi|7661666	Q9UGI8	49789	8.0	578	18	18	42	unclassified	unknown
457	5	phosphatidylinositol-5-phosphate 4-kinase, type II, alpha	PI42A	gi|6857820	P48426	46424	6.5	554	16	10	46	metabolism	NG,PL
458	4	protein disulfide isomerase-related protein 5	PDIA6	gi|1710248	Q15084	46512	5.0	733	12	7	38	chaperone	ER,ME
459	2	protein disulfide isomerase-related protein 5	PDIA6	gi|1710248	Q15084	46512	5.0	515	11	12	36	chaperone	ER,ME
460	2	solute carrier family 9 (sodium/hydrogen exchanger), isoform 3 regulator 1	SLC9A3R1	gi|4759140	O14745	39130	5.6	525	13	14	51	scaffolding	ME,EX,PL
461	2	glutathione synthetase	GSHB	gi|4504169	P48637	52523	5.7	274	5	10	16	redox	PL
462	2	eukaryotic translation elongation factor 1 alpha 1	EEF1A1	gi|48734966	Q6IPN6	50433	9.1	182	8	13	24	biosynthesis	ER,LY,EN,ME,EX,PL
463	3	bleomycin hydrolase	BLMH	gi|7245509	Q13867	52869	6.0	264	12	14	31	hydrolase	CY,PL
464	3	ARP3 actin-related protein 3 homolog	ACTR3	gi|5031573	P61158	47797	5.6	715	19	10	54	trafficking	EN,ER,ME
465	4	ARP3 actin-related protein 3 homolog	ACTR3	gi|5031573	P61158	47797	5.6	729	22	23	64	trafficking	EN,ER,ME
466	1	sorting nexin 6	SNX6	gi|119586321	Q9UNH7	50493	6.7	106	11	41	35	tranport	CY
467	2	Rab GDP dissociation inhibitor beta	GDI2	gi|119606836	P50395	48680	7.5	384	13	17	43	GTPases	ME,EX,PL,MT
468	1	UDP-glucose pyrophosphorylase 2 isoform b	UGP2	gi|48255968	Q16851	55813	7.7	290	15	33	38	metabolism	EN,ME
469	2	Rab GDP dissociation inhibitor beta	GDI2	gi|119606836	P50395	48680	7.5	676	16	7	53	GTPases	ME,EX,PL,MT
470	1	tumor susceptibility gene 101	TS101	gi|5454140	Q99816	44088	6.1	109	3	16	9	trafficking	EX
471	4	c-src tyrosine kinase	CSK	gi|4758078	P41240	51242	6.6	482	15	25	43	signal trans.	CY,PL
472	1	hypothetical protein LOC79624	C6orf211	gi|13375746	Q9H993	51539	5.5	66	3	14	12	unclassified	unknown
473	5	solute carrier family 9 (sodium/hydrogen exchanger), isoform 3 regulator 1	SLC9A3R1	gi|4759140	O14745	39130	5.6	391	11	20	40	scaffolding	ME,EX,PL
474	1	LPXN protein	LPXN	gi|49168546	Q6FI07	44619	5.6	208	4	10	16	unclassified	unknown
475	3	c-src tyrosine kinase	CSK	gi|4758078	P41240	51242	6.6	647	19	18	45	signal trans.	CY,PL
476	2	gamma-enolase	ENO2	gi|182118	P09104	44568	4.9	294	6	14	21	glycolysis	ME,PL,SY
477	2	flotillin 1	FLOT1	gi|5031699	O75955	47554	7.1	296	7	8	22	membrane	LY,ME,EX
478	2	enolase 1 variant	ENOA	gi|62896593	P06733	47453	7.0	469	13	18	40	metabolism	ME,EX,SY,MT
479	1	enolase 1 variant	ENOA	gi|62896593	P06733	47453	7.0	226	15	35	34	metabolism	ME,EX,SY,MT
480	1	glyceraldehyde-3-phosphate dehydrogenase	GAPDH	gi|67464043	P04406	36483	8.6	507	8	13	27	metabolism	LY,ME,NG,EX,PL,SY,MT
482	1	septin 7	SEPT7	gi|62088934	Q59EY4	44084	7.7	139	3	15	13	unclassified	ME,PL,SY
483	3	enolase 1 variant	ENOA	gi|31873302	P06733	47405	7.6	386	8	10	25	metabolism	ME,EX,SY,MT
484	1	septin 7	SEPT7	gi|62088934	Q59EY4	44084	7.7	288	9	18	16	unclassified	ME,PL,SY
485	1	ACTB protein	ACTB	gi|15277503	P60709	40536	5.6	495	13	35	34	cytoskeleton	ER,ME,EX,PL,SY
486	4	flotillin 1	FLOT1	gi|5031699	O75955	47554	7.1	704	25	25	63	membrane	LY,ME,EX
487	3	enolase 1 variant	ENOA	gi|62896593	P06733	47453	7.0	597	17	23	55	metabolism	ME,EX,SY,MT
489	1	chromosome 20 open reading frame 3 (BSCv)	C20orf3	gi|9836652	Q9HDC9	47887	5.8	145	4	10	12	unclassified	ME
490	2	PA2G4 protein	PA2G4	gi|33879698	Q6PIN5	41996	7.1	132	8	19	18	unclassified	unknown
492	3	enolase 1 variant	ENOA	gi|62896593	P06733	47453	7.0	458	15	8	45	metabolism	ME,EX,SY,MT
495	2	tapasin isoform 3 precursor	TPSN	gi|27436897	O15533	44145	6.4	238	6	15	19	immunity	ER,ME
496	6	enolase 1 variant	ENOA	gi|62896593	P06733	47453	7.0	755	19	28	59	metabolism	ME,EX,SY,MT
497	3	eukaryotic translation elongation factor 1 gamma, isoform CRA_b	EEF1G	gi|119594430	P26641	44869	6.1	154	5	12	16	biosynthesis	ME
498	2	proteasome 26S subunit, ATPase, 2	Nbla10058	gi|76879893	Q3LIA5	48986	5.9	149	10	25	11	unclassified	CY,NU
499	1	DnaJ (Hsp40) homolog, subfamily A, member 1, isoform CRA_d	DNAJA1	gi|119578931	P31689	42864	7.5	68	2	9	9	chaperone	ER,ME
500	2	ACTB protein	ACTB	gi|15277503	P60709	40536	5.6	498	11	21	42	cytoskeleton	ER,ME,EX,PL,SY
501	1	thioredoxin domain-containing protein 4 precursor	TXND4	gi|37183214	Q9BS26	47297	5.1	256	8	20	26	scaffolding	ER,ME,PL
502	2	thioredoxin domain-containing protein 4 precursor	TXND4	gi|37183214	Q9BS26	47297	5.1	535	9	8	28	scaffolding	ER,ME,PL
503	2	vasodilator-stimulated phosphoprotein	VASP	gi|119577769	P50552	39848	9.1	333	9	13	22	cytoskeleton	PL
504	3	annexin A7 isoform 2	ANXA7	gi|4809279	P20073	52991	5.5	212	7	9	18	exocytosis	EX,ME,PL
505	1	eukaryotic translation initiation factor 4A	IF4A1	gi|4503529	P60842	46353	5.3	97	5	14	16	biosynthesis	ME
506	1	NCK adaptor protein 1	NCK1	gi|5453754	P16333	43066	6.1	192	7	8	21	adapter	CY,ER
507	1	fumarate hydratase, mitochondrial	FH	gi|19743875	P07954	54773	8.9	235	8	12	21	cell cycle	EN,SY,MT
508	1	ACTB protein	ACTB	gi|15277503	P60709	40536	5.6	554	13	24	51	cytoskeleton	ER,ME,EX,PL,SY
509	1	mitogen-activated protein kinase kinase 2	MP2K2	gi|13489054	P36507	44681	6.1	126	7	11	19	signal trans.	unknown
510	2	isocitrate dehydrogenase 2 (NADP+), mitochondrial, isoform CRA_b	IDH2	gi|119622488	P48735	48071	8.3	166	12	24	31	redox	PL,MT
511	1	Obg-like ATPase 1	OLA1	gi|58761500	Q9NTK5	44943	7.6	301	9	22	9	hydrolases	EN,ME,PL
512	1	tripeptidyl-peptidase 1	TPP1	gi|34452679	O14773	34670	5.7	206	4	18	13	degradation	LY,ME,NG,PL,MT
513	1	S-adenosylhomocysteine hydrolase	SAHH	gi|178277	P23526	48254	6.0	189	14	32	31	hydrolase	ME
514	1	proteasome 26S subunit, ATPase, 5	nd	gi|62087652	Q59GS3	38907	6.1	209	7	11	27	unclassified	CY,NU
516	1	ACTB protein	ACTB	gi|15277503	P60709	40536	5.6	485	10	21	32	cytoskeleton	ER,ME,EX,PL,SY
517	4	Tu translation elongation factor, mitochondrial	TUFM	gi|119572383	P49411	37775	6.1	253	5	11	17	biosynthesis	LY,ME,PL,MT
518	1	ACTB protein	ACTB	gi|15277503	P60709	40536	5.6	393	8	22	30	cytoskeleton	ER,ME,EX,PL,SY
519	2	actin related protein 2 isoform b	ACTR2	gi|5031571	P61160	45017	6.3	200	6	14	25	trafficking	EN,ER,ME
522	3	ACTB protein	ACTB	gi|15277503	P60709	40536	5.6	511	12	35	31	cytoskeleton	ER,ME,EX,PL,SY
523	3	phosphoribosylaminoimidazole carboxylase, phosphoribosylaminoimidazole succinocarboxamide synthetase, isoform CRA_b	PUR6	gi|126030593	P22234	47650	6.7	514	18	25	35	multifunctional	EN,SY
524	2	ACTB protein	ACTB	gi|15277503	P60709	40536	5.6	526	14	23	49	cytoskeleton	ER,ME,EX,PL,SY
525	1	ACTB protein	ACTB	gi|15277503	P60709	40536	5.6	680	21	31	65	cytoskeleton	ER,ME,EX,PL,SY
526	2	ACTB protein	ACTB	gi|15277503	P60709	40536	5.6	552	17	44	45	cytoskeleton	ER,ME,EX,PL,SY
527	2	ACTB protein	ACTB	gi|15277503	P60709	40536	5.6	615	19	33	51	cytoskeleton	ER,ME,EX,PL,SY
530	2	ACTB protein	ACTB	gi|15277503	P60709	40536	5.6	357	12	41	34	cytoskeleton	ER,ME,EX,PL,SY
531	3	GNAS complex locus isoform f	GNAS2	gi|117938762	P63092	46179	5.6	439	12	15	33	multifunctional	EX
533	1	MHC class I antigen	HLA-A	gi|64976582	Q4W6C4	31862	5.9	414	8	10	42	Immunity	ME
534	1	MHC class I antigen	HLA-A	gi|106879085	Q1EPW2	31527	5.7	230	9	26	31	Immunity	ME
535	1	MHC class I antigen	HLA-A	gi|124517293	A2V815	31959	6.1	126	5	11	29	Immunity	ME
536	1	protein tyrosine phosphatase 1b	PTPN1	gi|114793621	P18031	38453	6.3	224	11	35	38	hydrolases	ME,ER
537	2	phosphoglycerate kinase 1	PGK1	gi|48145549	P00558	44973	8.3	623	15	25	23	metabolism	ME,EX,SY,MT
539	3	phosphoglycerate kinase 1	PGK1	gi|48145549	P00558	44973	8.3	398	7	7	24	metabolism	ME,EX,SY,MT
540	2	isocitrate dehydrogenase 1 (NADP+), soluble, isoform CRA_b	IDH1	gi|119590846	O75874	32765	8.4	77	2	14	10	redox	ME,EX,PL
541	1	GDP-mannose pyrophosphorylase A	GMPPA	gi|31881779	Q96IJ6	46604	6.7	132	9	17	26	biosythesis	unknown
542	1	2',3'-cyclic-nucleotide 3'-phosphodiesterase	CNP	gi|180687	P09543	45469	8.7	244	15	21	22	hydrolase	ME,NG
543	1	laminin-binding protein	RPSA	gi|34234	P08865	31888	4.8	256	4	5	16	cell adhesion	ME,ER
544	2	MHC class I antigen	HLA-A	gi|73354293	Q3YBM1	31662	5.7	384	11	13	54	Immunity	ME
545	2	MHC class I antigen	HLA-A	gi|12598440	P01892	31963	6.0	348	12	23	55	immunity	GO,EN
546	1	XRP2 protein	XRP2	gi|5902060	O75695	40472	4.9	217	8	18	27	signal trans.	ME
547	2	MHC class I antigen	HLA-A	gi|113196585	Q0GC70	31863	5.4	210	5	10	25	Immunity	ME

spot no.[1]	replicats[2]	protein name [3]	gene	acc.no.[4]	Uni Prot no.[5]	MW, Da[6]	pI[7]	MASCOT [8]	matched peptides[9]	unmatched peptides [10]	% seq. coverage [11]	function [12]	subcellular localisation [13]
548	2	serpin peptidase inhibitor,clade B,member 1	SERPINB1	gi|62898301	P30740	42857	5.9	485	8	10	25	inhibitor	CY
550	4	adenosine deaminase	ADA	gi|1197210	P00813	35335	5.6	493	12	17	46	hydrolase	CY,LY
551	4	DnaJ (Hsp40) homolog, subfamily B, member 11 precursor	DNAJB11	gi|7706495	Q9UBS4	40774	5.8	311	9	13	31	chaperone	ER
552	2	ACTB protein	ACTB	gi|15277503	P60709	40536	5.6	209	3	16	12	cytoskeleton	ER,ME,EX,PL,SY
553	3	adenosine deaminase	ADA	gi|1197210	P00813	35335	5.6	211	10	15	38	hydrolase	CY,LY
554	1	septin 2	SEPT2	gi|119591666	Q15019	18259	6.2	210	6	27	59	unclassified	ME,EX,SY
555	1	aldolase A	ALDOA	gi|4557305	P04075	39851	8.3	291	11	18	35	metabolism	EN,ME
557	2	aldolase A	ALDOA	gi|4557305	P04075	39851	8.3	386	14	19	53	metabolism	EN,ME
558	2	septin-9 delta	SEPT9	gi|14530107	Q9UHD8	38690	7.1	204	6	13	24	unclassified	ME
559	2	aldolase A	ALDOA	gi|4557305	P04075	39851	8.3	150	9	20	29	metabolism	EN,ME
560	1	aldolase A	ALDOA	gi|4557305	P04075	39851	8.3	401	16	22	54	metabolism	EN,ME
561	2	tropomodulin 3	TMOD3	gi|6934244	Q9NYL9	39727	5.1	188	5	12	21	cytoskeleton	ER,ME
562	3	aldolase A	ALDOA	gi|4557305	P04075	39851	8.3	363	11	18	36	metabolism	EN,ME
563	3	fructose-bisphosphate aldolase C	ALDOC	gi|4885063	P09972	39830	6.4	156	5	10	22	metabolism	ME,SY,MT
564	3	actin related protein 2 isoform b	ACTR2	gi|5031571	P61160	45017	6.3	611	18	14	52	trafficking	EN,ER,ME
565	3	fructose-bisphosphate aldolase C	ALDOC	gi|4885063	P09972	39830	6.4	458	12	16	46	metabolism	ME,SY,MT
566	1	septin-9 delta	SEPT9	gi|14530107	Q9UHD8	38690	7.1	96	4	8	12	unclassified	ME
567	2	actin related protein 2/3 complex subunit 1B	ARPC1B	gi|5031601	O15143	41722	8.7	186	4	16	15	trafficking	ME,PL
568	3	gelsolin-like capping protein isoform 9	CAPG	gi|55597035	P40121	38779	5.9	229	5	13	23	cytoskeleton	ME,CY,NU
569	4	mitogen-activated protein kinase 1	MK01	gi|20986531	P28482	41762	6.5	351	8	9	28	signal trans.	ME,PL
570	1	actin, alpha, cardiac muscle	ACTC1	gi|119612724	P68032	30498	4.9	121	2	15	9	cytoskeleton	ME
571	2	actin related protein 2 isoform b	ACTR2	gi|5031571	P61160	45017	6.3	609	21	24	55	trafficking	EN,ER,ME
572	3	gelsolin-like capping protein isoform 9	CAPG	gi|55597035	P40121	38779	5.9	368	9	21	40	cytoskeleton	ME,CY,NU
573	1	ACTB protein	ACTB	gi|15277503	P60709	40536	5.6	403	7	15	27	cytoskeleton	ER,ME,EX,PL,SY
574	1	twinfilin-like protein	TWF2	gi|6005846	Q6IBS0	39751	6.4	614	15	30	64	cytoskeleton	CY
575	2	aspartate aminotransferase	GIG18	gi|46981967	Q2TU84	46519	6.3	129	9	14	20	biosynthesis	CY
576	3	twinfilin-like protein	TWF2	gi|6005846	Q6IBS0	39751	6.4	324	6	5	23	cytoskeleton	CY
577	1	proteasome 26S non-ATPase subunit 13 isoform 1	PSMD13	gi|157502193	Q9UNM6	43203	5.5	172	5	14	18	proteasome	ME
578	5	twinfilin-like protein	TWF2	gi|6005846	Q6IBS0	39751	6.4	628	11	12	50	cytoskeleton	CY
579	3	farnesyl pyrophosphate synthetase	FPPS	gi|182405	P14324	40102	5.1	168	4	18	14	biosynthesis	CY
581	1	guanine nucleotide-binding protein G(k) subunit alpha	GNAI3	gi|119390147	P08754	37644	5.6	311	10	11	30	signal trans.	ME,EX
582	4	guanine nucleotide binding protein (G protein), alpha inhibiting activity polypeptide 2, isoform CRA_c	GNAI2	gi|119585457	B4E2X5	35425	5.2	673	12	10	52	GTPases	EX
584	2	guanine nucleotide binding protein (G protein), alpha inhibiting activity polypeptide 2, isoform CRA_d	GNAI2	gi|119585458	B4E2X5	20295	5.5	314	6	10	45	GTPases	EX
585	3	guanine nucleotide-binding protein G(k) subunit alpha	GNAI3	gi|119390147	P08754	37644	5.6	445	11	16	39	transport	ME,EX
586	3	gelsolin-like capping protein	CAPG	gi|63252913	P40121	38760	5.8	334	9	26	41	cytoskeleton	ME,CY,NU
587	1	alcohol dehydrogenase class-3	ADHX	gi|110591508	P11766	40407	7.9	73	2	10	8	redox protein	PL,CY
588	1	guanine nucleotide-binding protein G(k) subunit alpha	GNAI3	gi|119390147	P08754	37644	5.6	388	9	8	31	transport	ME,EX
589	1	glutaredoxin 3	GLRX3	gi|95113651	O76003	37693	5.3	177	8	25	32	redox	CY
590	3	actin related protein 2/3 complex subunit 1B	ARPC1B	gi|5031601	O15143	41722	8.7	513	9	12	39	trafficking	ME,PL
591	3	guanine nucleotide binding protein (G protein), alpha inhibiting activity polypeptide 2, isoform CRA_c	GNAI2	gi|119585457	B4E2X5	35425	5	525	8	12	36	GTPases	EX
592	1	serine/threonine-protein phosphatase 2A regulatory subunit B	PPP2R4	gi|93279884	Q15257	37539	6.6	165	8	13	19	signal trans.	NU
594	2	acetyl-CoA acetyltransferase, cytosolic	THIC	gi|61680217	Q9BWD1	41789	6.5	449	6	12	23	biosynthesis	CY
596	2	aldose 1-epimerase (BLOCK25)	GALM	gi|20530221	Q96C23	24185	5.6	245	7	17	42	metabolism	CY
598	1	GIPC1 protein	GIPC1	gi|33872740	O14908	32792	5.9	99	5	11	26	protein binding	SY,CY
599	2	V-type proton ATPase subunit d 1	ATP6V0D1	gi|542837	P61421	32083	5.2	138	4	16	17	channel	LY,EN,ME,SY
600	1	protein phosphatase 1, catalytic subunit, beta isoform	PPP1CB	gi|54696354	P62140	37945	5.8	120	6	23	27	hydrolases	ME,PL
601	2	protein phosphatase 1, catalytic subunit, alpha isoform 1	PPP1CA	gi|4506003	P62136	38229	5.9	343	14	24	43	hydrolases	EX
602	1	protein phosphatase 1, catalytic subunit, alpha isoform 1	PPP1CA	gi|4506003	P62136	38229	5.9	127	7	18	27	hydrolases	EX
603	1	protein phosphatase 1, catalytic subunit, alpha isoform 1	PPP1CA	gi|4506003	P62136	38229	5.9	543	18	17	61	hydrolases	EX
604	1	annexin A1	ANXA1	gi|4502101	P04083	38918	6.6	453	9	10	34	trafficking	ME,MT
605	1	calcium binding protein 39	CAB39	gi|42543739	Q9Y376	39834	6.3	117	4	12	14	unclassified	EX
606	1	LIM and SH3 domain protein 1	LASP1	gi|1584035	Q14847	30185	6.1	303	11	24	37	adapter protein	ER,EN,ME,PL
607	1	glyceraldehyde-3-phosphate dehydrogenase	GAPDH	gi|31645	P04406	36202	8.3	552	16	20	48	metabolism	LY,ME,NG,EX,PL,SY,MT
608	1	glyceraldehyde-3-phosphate dehydrogenase	GAPDH	gi|67464043	P04406	36483	8.6	508	12	21	46	metabolism	LY,ME,NG,EX,PL,SY,MT
609	1	glyceraldehyde-3-phosphate dehydrogenase	GAPDH	gi|67464043	P04406	36483	8.6	567	15	31	47	metabolism	LY,ME,NG,EX,PL,SY,MT
610	4	glyceraldehyde-3-phosphate dehydrogenase	GAPDH	gi|31645	P04406	36202	8.3	667	16	23	50	metabolism	LY,ME,NG,EX,PL,SY,MT
611	3	F-actin capping protein alpha-1 subunit	CAPZA1	gi|5453597	P52907	33073	5.5	709	10	9	54	actin binding	ER,EN,ME
612	1	carboxyl terminal LIM domain protein	LDB1	gi|1905874	Q14847	36604	6.8	185	6	13	23	cytoskeleton	ER,ME,PL,EN
615	2	annexin A1	ANXA1	gi|4502101	P04083	38918	6.6	668	16	16	51	trafficking	ME,MT
616	3	F-actin capping protein alpha-2 subunit	CAPZA2	gi|119603918	P47755	31898	6.5	398	7	11	41	cytoskeleton	ER,EN,ME,PL
617	4	protein phosphatase 1, catalytic subunit, beta isoform	PPP1CB	gi|54696354	P62140	37945	5.8	460	8	9	30	hydrolases	ME,PL
618	1	annexin A2	ANXA2	gi|119597993	P07355	32600	5.9	445	12	10	47	exocytosis	MT,EX,ME,secreted
620	1	actin related protein 2/3 complex subunit 1B	ARPC1B	gi|5031601	O15143	41722	8.7	343	10	15	34	trafficking	ME,PL
621	2	serine/threonine-protein phosphatase 2A catalytic subunit alpha isoform	PP2AA	gi|122921196	P67775	34186	5.1	209	5	7	26	signal trans.	MT
622	2	calcium binding protein 39	CAB39	gi|7706481	Q9Y376	40015	6.4	378	16	31	37	unclassified	EX
623	1	F-actin capping protein alpha-1 subunit variant	CAPZA1	gi|62898013	P52907	33057	5.5	128	3	15	16	actin binding	ER,EN,ME
625	4	annexin A2	ANXA2	gi|56966699	P07355	38866	6.9	597	21	26	65	exocytosis	MT,EX,ME,secreted
626	3	L-lactate dehydrogenase B chain	LDHB	gi|49259209	P07195	36801	5.9	448	11	11	43	redox	ME,EX,PL,SY,MT
627	1	protein phosphatase 1, catalytic subunit, alpha isoform 1	PPP1CA	gi|4506003	P62136	38229	5.9	120	7	14	30	hydrolases	EX
628	3	annexin A2	ANXA2	gi|56966699	P07355	38866	6.9	232	11	15	34	exocytosis	MT,EX,ME,secreted
629	1	gamma-glutamyl hydrolase	GGH	gi|4503987	Q92820	36340	6.7	99	2	18	9	hydrolase	LY,ME,NG,PL
630	3	LIM and SH3 domain protein 1	LASP1	gi|1584035	Q14847	30185	6.1	266	10	13	33	adapter protein	ER,EN,ME,PL
631	3	annexin A2	ANXA2	gi|119597993	P07355	32600	5.9	759	21	32	74	exocytosis	MT,EX,ME,secreted
632	1	axin interactor, dorsalization associated protein	AIDA	gi|52545806	Q96BJ3	34954	6.2	531	11	9	40	signal trans.	unknown
633	1	LIM and SH3 domain protein 1	LASP1	gi|5453710	Q14847	30097	6.6	121	5	12	24	adapter protein	ER,EN,ME,PL
634	1	GNB1 protein	GNB1	gi|91992949	Q1RMY8	37073	5.6	67	4	19	15	signal trans.	EN,ME,EX,PL,SY
635	2	potassium voltage-gated channel, shaker-related subfamily, beta member 2 isoform 2	KCAB2	gi|27436969	Q13303	39547	8.8	360	15	28	50	channel	CY
636	2	L-lactate dehydrogenase B chain	LDHB	gi|49259209	P07195	36801	5.9	129	8	25	27	redox	ME,EX,PL,SY,MT
637	1	otubain 1	OTUB1	gi|109148508	Q96FW1	31493	4.9	175	5	19	28	hydrolases	ME
638	1	G protein beta subunit	GNB2	gi|306785	P62879	38061	5.8	510	12	17	44	signal trans.	ME,MT
639	1	chromatin modifying protein 4B	CHM4B	gi|28827795	Q9H444	24935	4.8	90	2	16	12	transport	ME,EX
642	2	cytosolic malate dehydrogenase	MDHC	gi|5174539	P40925	36631	6.9	223	9	21	32	metabolism	ME,EX,PL,SY,MT
643	1	L-lactate dehydrogenase	LDHA	gi|62897717	P00338	36951	7.6	253	8	14	26	metabolism	ME,EX,SY
644	2	L-lactate dehydrogenase	LDHA	gi|13786849	P00338	36819	8.5	133	8	29	21	metabolism	ME,EX,SY
645	2	L-lactate dehydrogenase	LDHA	gi|13786849	P00338	36819	8.5	554	19	27	50	metabolism	ME,EX,SY
646	2	G protein beta subunit	GNB2	gi|306785	P62879	38061	5.8	298	10	15	29	signal trans.	ME,MT
647	1	calponin 2	CNN2	gi|119589975	Q99439	31829	7.6	169	9	17	40	cytoskeleton	CY
648	1	esterase D/formylglutathione hydrolase	ESTD	gi|55957280	P10768	25695	6.4	131	2	10	12	Hydrolase	ME
649	1	G protein beta subunit	GNB2	gi|306785	P62879	38061	5.8	300	7	11	18	signal trans.	ME,MT
650	1	MHC class II antigen DR alpha chain	HLA-DRA	gi|3212400	P01903	20548	4.9	113	3	18	27	Immunity	ME
651	2	CNN2 protein	CNN2	gi|49456577	Q6FHE4	34086	7.0	480	18	30	63	unknown	unknown
652	1	N-ethylmaleimide-sensitive factor attachment protein, alpha	SNAA	gi|47933379	P54920	33667	5.2	368	13	21	58	trafficking	ME,NG,PL
653	1	glyoxalase domain containing 4	GLOD4	gi|16198390	Q9HC38	33536	5.4	150	6	20	22	unclassified	MT
654	1	pyrophosphatase 1	IPYR	gi|11056044	Q15181	33095	5.5	136	3	13	13	hydrolase	ME,MT
656	3	esterase D/formylglutathione hydrolase	ESTD	gi|55957281	P10768	28607	6.3	233	7	17	38	Hydrolase	ME
657	1	voltage-dependent anion channel 2	VDAC2	gi|55664661	P45880	30842	8.0	72	2	4	9	channel	SY,MT
658	2	voltage-dependent anion channel 1	VDAC1	gi|4507879	P21796	30868	8.6	357	8	8	44	channel	ER,LY,ME,NG,EX,PL,SY,MT

spot no.[1]	replicats[2]	protein name [3]	gene	acc.no.[4]	Uni Prot no.[5]	MW, Da[6]	pI[7]	MASCOT [8]	matched peptides[9]	unmatched peptides [10]	% seq. coverage [11]	function [12]	subcellular localisation [13]
659	1	annexin A5	ANXA5	gi|809185	P08758	35840	4.9	145	2	4	8	trafficking	MT,EX,ME,SY
660	2	annexin A5	ANXA5	gi|809185	P08758	35840	4.9	624	18	19	60	trafficking	MT,EX,ME,ER
662	1	F-actin capping protein beta subunit	CAPZB	gi|4826659	P47756	30952	5.7	100	4	21	12	actin binding	ER,EN,ME
663	1	F-actin capping protein beta subunit	CAPZB	gi|4826659	P47756	30952	5.7	333	7	11	26	actin binding	ER,EN,ME
664	1	guanine nucleotide binding protein (G protein), beta polypeptide 2-like 1, isoform CRA_d	GNB2L1	gi|119574080	P63244	30942	7.0	275	6	13	23	signal trans.	ER
665	1	microtubule-associated protein, RP/EB family, member 1	MAPRE1	gi|6912494	Q15691	30151	5.0	199	3	8	17	cytoskeleton	ME,PL
666	1	Cbr1 In Complex With Hydroxy-Pp	CBR1	gi|66360348	P16152	30510	8.6	247	8	15	37	redox protein	ME
668	2	annexin A4	ANXA4	gi|1703319	P09525	36088	5.8	398	8	7	29	signal trans.	MT,EX,ME,SY
669	2	MHC class II antigen	HLA-DRB1	gi|77019293	Q3LAB6	21042	6.1	270	5	12	38	Immunity	ME
670	3	nucleoside phosphorylase	PNPH	gi|157168362	P00491	32325	6.5	481	13	23	59	cell proliferation	CY,PL
671	2	actin related protein 2/3 complex subunit 2	ARPC2	gi|5031599	O15144	34426	6.8	528	11	6	39	trafficking	EN,ME,PL,ER
672	1	tropomyosin 4	TPM4	gi|4507651	P67936	28619	4.7	428	18	30	53	unclassified	ME
673	1	actin related protein 2/3 complex subunit 2	ARPC2	gi|7959903	O15144	27290	7.8	148	5	7	24	cytoskeleton	EN,ME,PL
674	2	actin related protein 2/3 complex subunit 2	ARPC2	gi|5031599	O15144	34426	6.8	775	24	24	70	trafficking	EN,ME,PL,ER
676	5	tropomyosin 3 isoform 2	TPM3	gi|24119203	P06753	29243	4.8	648	21	20	58	unclassified	unknown
677	1	glucosamine-6-phosphate deaminase 1	GNPI1	gi|13027378	P46926	32819	6.4	257	8	17	43	hydrolases	CY
678	2	actin related protein 2/3 complex subunit 2	ARPC2	gi|5031599	O15144	34426	6.8	370	12	17	40	trafficking	EN,ME,PL,ER
682	1	cathepsin D preproprotein	CATD	gi|4503143	P07339	45037	6.1	212	6	11	14	hydrolases	LY,ME,NG,EX,MT
683	1	dimethylarginine dimethylaminohydrolase 2	DDAH2	gi|7524354	O95865	29911	5.7	80	4	10	22	hydrolase	unknown
684	5	nuclear chloride channel	CLIC1	gi|4588526	O00299	27249	5.0	575	15	22	64	channel	ME,EX,PL,MT
685	2	14-3-3 protein epsilon	YWHAE	gi|67464424	P62258	26912	4.9	295	7	20	33	adapter protein	CY,ME
686	2	similar to metallo-beta-lactamase superfamily protein	nd	gi|42822880	Q68D91	31493	6.4	344	6	8	32	hydrolase	unknown
687	3	proteasome subunit, alpha type, 1	PSMA1	gi|13543551	P25786	29864	6.2	283	9	19	39	hydrolases	ME
688	2	voltage-dependent anion channel 3	VDAC3	gi|25188179	Q9Y277	30981	8.9	152	5	17	26	channel	EN,ME,MT
689	1	proteasome activator complex subunit 2	PSME2	gi|1008915	Q9UL46	27502	5.4	571	13	19	55	Immunity	ME
690	1	haloacid dehalogenase-like hydrolase domain containing 2	HDHD2	gi|14149777	Q9H0R4	28746	5.8	113	5	24	28	hydrolases	unknown
691	4	cathepsin B	CATB	gi|24158605	P07858	29571	5.3	173	3	14	16	hydrolases	LY,ME,NG
692	1	proteasome activator subunit 2 (PA28 beta)	PSME2	gi|119586505	Q9UL46	22023	5.4	85	2	14	14	Immunity	ME
694	2	nuclear chloride channel	CLIC1	gi|4588526	O00299	27249	5.0	290	6	13	30	channel	ME,EX,PL,MT
696	4	cathepsin D preproprotein	CATD	gi|4503143	P07339	45037	6.1	343	10	13	26	hydrolases	LY,ME,NG,EX,MT
697	2	methylthioadenosine phosphorylase	MTAP	gi|847724	Q13126	31743	6.8	129	6	6	28	metabolism	CY
698	1	glutathione-S-transferase omega 1	GSTO1	gi|4758484	P78417	27833	6.2	137	5	8	21	metabolism	LY,ME,NG,EX,PL,SY,MT
699	1	proline synthetase co-transcribed homolog	PROSC	gi|6005842	O94903	30610	7.1	127	2	5	12	unclassified	CY
701	2	actin, gamma 1 propeptide	ACTG1	gi|4501887	P63261	42108	5.3	304	7	14	23	cytoskeleton	ME,EX,PL,SY
703	3	proteasome activator complex subunit 1 isoform 1	PSME1	gi|5453990	Q06323	28876	5.8	297	12	12	51	Immunity	PL,MT
704	2	cathepsin D preproprotein	CATD	gi|4503143	P07339	45037	6.1	181	8	17	24	hydrolases	LY,ME,NG,EX,MT
706	1	unnamed protein product	unnamed	gi|28071074	Q86TY5	13945	9.2	350	7	14	64	unknown	unknown
707	4	granzyme A	GZMA	gi|33357774	P12544	26328	9.1	324	6	9	30	Immunity	SL
708	3	14-3-3 protein zeta/delta	YWHAZ	gi|49119653	P63104	30100	4.7	546	17	23	62	adapter protein	CY,ME
709	4	cathepsin D preproprotein	CATD	gi|4503143	P07339	45037	6.1	250	8	14	24	hydrolases	LY,ME,NG,EX,MT
712	1	profilin-1	PROF1	gi|5822002	P07737	15085	8.5	403	11	29	69	actin binding	ME,EX,PL,MT
714	1	14-3-3 protein beta	YWHAB	gi|4507949	P31946	28179	4.8	313	11	20	39	adapter protein	CY,ME
716	2	Rho GDP dissociation inhibitor (GDI) alpha	GDIR1	gi|4757768	P52565	23250	5.0	279	9	20	38	GTPases	ME,PL,MT
717	4	granzyme A	GZMA	gi|33357774	P12544	26328	9.1	372	7	13	35	Immunity	SL
718	5	galectin-3	LGALS3	gi|45786143	Q6NVH9	26238	8.6	352	7	9	31	Immunity	ME,NU
720	2	granzyme A	GZMA	gi|33357774	P12544	26328	9.1	448	9	14	51	Immunity	SL
721	2	endoplasmic reticulum protein 29 isoform 1 precursor	ERP29	gi|5803013	P30040	29032	6.8	360	11	26	42	chaperone	ER,ME,PL
724	3	granzyme A	GZMA	gi|33357774	P12544	26328	9.1	587	15	28	69	Immunity	SL
725	2	endoplasmic reticulum protein 29 isoform 1 precursor	ERP29	gi|5803013	P30040	29032	6.8	279	8	10	37	chaperone	ER,ME,PL
728	2	Rho GDP dissociation inhibitor (GDI) beta	GDIR2	gi|56676393	P52566	23031	5.1	346	7	14	46	GTPases	CY
729	2	proteasome (prosome, macropain) subunit, alpha type, 7(PSMA7)	PSMA7	gi|119595803	B2R515	18896	8.9	71	2	13	19	hydrolase	CY,Proteasom
730	2	PGAM1	PGAM1	gi|49456447	Q6FHK8	28931	6.7	393	11	13	48	metabolism	ME,EX,SY
734	2	proteasome subunit, alpha type, 6	PSMA6	gi|8394076	P60901	27838	6.3	206	5	10	24	hydrolase	CY,NU
736	1	TC4 protein	TC4	gi|114306762	Q0EFC9	12221	9.3	113	2	19	17	GTPases	NU
737	3	peroxiredoxin 4	PRDX4	gi|5453549	Q13162	30749	5.9	187	9	13	37	redox proteins	ER,EN,ME
738	1	cathepsin H	CATH	gi|29708	P09668	27844	7.0	124	2	2	12	hydrolases	LY
739	2	peroxiredoxin 6	PRDX6	gi|4758638	P30041	25133	6.0	379	13	24	51	redox proteins	LY,ME,EX,PL,SY
740	1	triosephosphate isomerase (TIM) (Triose-phosphate isomerase)	TPIS	gi|136066	P00939	26894	7.1	118	2	4	13	unclassified	ME,EX,SY,MT
741	2	programmed cell death protein 10	PDC10	gi|2465729	Q9BUL8	24642	8.6	122	7	18	28	apoptosis	unknown
742	2	triosephosphate isomerase 1	TPIS	gi|4507645	P60174	26938	6.5	542	13	11	68	unclassified	ME,EX,SY,MT
743	2	triosephosphate isomerase 1	TPIS	gi|999892	P60174	26807	6.5	98	2	8	13	unclassified	ME,EX,SY,MT
747	1	leucine rich repeat containing 57	LRRC57	gi|74760039	Q8N9N7	27065	8.5	153	4	9	19	unclassified	unknown
749	2	cathepsin S	CATS	gi|30749675	P25774	24491	7.6	272	6	11	31	immunity	LY
750	1	proteasome subunit, beta type, 1	PSMB1	gi|119567805	P20618	24634	8.7	169	5	14	34	hydrolases	ME,CY
754	3	proteasome alpha 2 subunit variant	nd	gi|62897513	Q53GF5	25938	7.7	254	4	11	23	hydrolase	CY
755	1	GTP-binding nuclear protein Ran	RAN	gi|5107682	P62826	23307	9.0	220	6	12	33	transport	ME,EX
756	3	GRB2 protein	GRB2	gi|47496673	Q6ICN0	25246	6.1	299	10	9	45	adapter protein	SY
758	1	mps one binder kinase activator-like 1B	MOL1B	gi|7023036	Q9H8S9	25163	6.7	161	3	8	18	unclassified	unknown
760	2	proteasome subunit, beta type, 1	PSMB1	gi|119567805	P20618	24634	8.7	135	10	21	60	hydrolases	ME,CY
762	1	peroxiredoxin 1	PRDX1	gi|55959887	Q06830	19135	6.4	216	5	9	29	redox proteins	ER,LY,EN,ME,NG,PL,MT
764	1	DJ-1 protein	PARK7	gi|42543006	Q99497	20063	6.3	125	5	9	32	redox proteins	ME,PL,SY,MT
765	1	glutathione-S-transferase kappa 1	GSTK1	gi|7705704	Q9Y2Q3	25594	8.5	147	3	4	15	unclassified	PL,ME,MT,PE
766	3	glutathione S-transferase P1	GSTP1	gi|20664358	P09211	23430	5.1	303	5	11	40	metabolism	ER,ME,EX,PL
767	1	stromal cell-derived factor 2-like 1 precursor	SDF2L	gi|56243533	Q9HCN8	23812	6.5	105	3	19	22	unclassified	ER
768	1	peroxiredoxin 3	PRDX3	gi|119569783	P30048	11158	6.1	113	3	7	36	redox	ME,PL,MT
769	2	cysteine and glycine-rich protein 1	CSRP1	gi|4758086	P21291	21409	8.9	592	6	8	54	unknown	NU
770	1	glutathione S-transferase P1	GSTP1	gi|2554831	P09211	23555	5.4	128	4	9	28	metabolism	ER,ME,EX,PL
771	1	PYD and CARD domain containing	ASC	gi|13325316	Q9ULZ3	15078	6.8	79	3	11	17	apoptosis	CY
772	2	peroxiredoxin 2	PRDX2	gi|1617118	P32119	18486	5.2	97	3	14	17	redox protein	ER,EN,ME,SY,MT
773	3	proteasome subunit, beta type, 8	PSMB8	gi|49456283	P28062	30019	5.5	279	6	10	22	immunity	PL,CY,NU
774	2	peroxiredoxin 1	PRDX1	gi|55959887	Q06830	19135	6.4	227	10	29	50	redox proteins	ER,LY,EN,ME,NG,PL,MT
775	1	proteasome subunit, beta type, 2	PSMB2	gi|119627813	P49721	20365	7.0	100	3	6	25	hydrolases	CY,NU
778	1	peroxiredoxin 2	PRDX2	gi|9955007	P32119	21909	5.4	319	9	15	35	redox proteins	ER,EN,ME,SY,MT
779	1	neuroblastoma RAS viral (v-ras) oncogene homolog	RASN	gi|4505451	P01111	21501	5.0	198	4	14	25	trafficking	GO,CY
780	3	proteasome subunit, beta type, 2	PSMB2	gi|119627813	P49721	20365	7.0	225	5	11	39	hydrolases	CY,NU
781	2	neuropolypeptide h3	PEBP1	gi|4261934	P30086	16068	8.8	149	2	30	24	inhibitor	ME,EX,SY
783	1	transgelin-2	TAGLN2	gi|9956026	P37802	24609	8.4	79	4	12	23	unclassified	ME,MT
784	2	adenine phosphoribosyltransferase isoform b	APRT	gi|71773201	P07741	14605	6.7	91	2	12	19	biosynthesis	ME,EX,PL
785	1	Rap1a	RAP1A	gi|1942609	P62834	19297	4.6	83	2	9	8	GTPases	EN,ME,MT
787	1	transgelin-2	TAGLN2	gi|9956026	P37802	24609	8.4	184	9	36	36	unclassified	ME,MT
790	3	Cdc42ACK GTPASE	CDC42	gi|20151145	P60953	21189	6.2	307	4	6	26	cytoskeleton	ME
794	1	galectin-3	LGALS3	gi|45786143	Q6NVH9	26238	8.6	346	7	8	31	Immunity	ME,NU
795	1	SHUJUN-1	MRLC2	gi|32187319	O14950	17047	4.2	83	2	8	14	cytoskeleton	CY
797	1	ribosomal protein L11	Q5VVC9	gi|55665352	Q5VVC9	15085	9.0	143	5	10	32	biosynthesis	ribosom
798	2	actin related protein 2/3 complex subunit 4	ARPC4	gi|5031595	P59998	19768	8.5	355	7	8	52	trafficking	ER,EX,ME,SY
799	1	actin related protein 2/3 complex subunit 3	ARPC3	gi|119618319	O15145	19108	8.7	120	4	27	24	trafficking	EN,ME,PL,ER
800	2	cyclophilin B	PPIB	gi|1310882	P23284	19705	9.2	447	15	11	69	chaperone	ER,ME
803	3	NME1-NME2 protein	NDKB	gi|66392203	P22392	30346	9.1	328	6	7	41	multifunctional	CY,NU

spot no.[1]	replicats[2]	protein name [3]	gene	acc.no.[4]	Uni Prot no.[5]	MW, Da[6]	pI[7]	MASCOT [8]	matched peptides[9]	unmatched peptides [10]	% seq. coverage [11]	function [12]	subcellular localisation [13]
804	2	actin related protein 2/3 complex subunit 5-like	ARPC5L	gi|33150554	Q9BPX5	16891	8.0	136	4	11	34	trafficking	ME
805	2	protein ARMET	ARMET	gi|23503040	P55145	20700	8.5	116	3	9	26	unclassified	ME, secreted
806	2	superoxide dismutase 1, soluble	SODC	gi|4507149	P00441	16154	5.7	181	2	7	12	redox proteins	ME,EX,MT
807	2	cofilin 1, isoform CRA_c	CFL1	gi|119594857	P23528	15877	8.5	205	5	9	32	cytoskeleton	ER,ME,EX,MT
808	2	cofilin 1, isoform CRA_c	CFL1	gi|119594857	P23528	15877	8.5	157	4	11	32	cytoskeleton	ER,ME,EX,MT
809	1	ribosomal protein L12	RPL12	gi|119608074	P30050	10457	9.9	113	2	18	32	biosynthesis	EN,ribosom
810	1	cofilin 1	CFL1	gi|5031635	P23528	18719	8.2	293	9	39	62	cytoskeleton	ER,ME,EX,MT
811	1	translocon-associated protein subunit delta	SSR4	gi|119593214	P51571	12311	6.4	91	3	24	28	trafficking	ME,ER
812	5	cofilin 1	CFL1	gi|5031635	P23528	18719	8.2	251	8	19	52	cytoskeleton	ER,ME,EX,MT
814	3	destrin isoform a	DEST	gi|5802966	P60981	18950	8.1	235	6	16	35	cytoskeleton	ME,EX,MT
816	2	CALM3 protein	CALM3	gi|13544110	Q9BRL5	16610	4.3	216	6	33	32	unknown	unknown
817	3	eukaryotic translation initiation factor 5A	IF5A1	gi|4503545	P63241	17049	5.1	213	3	8	23	biosynthesis	ME
818	2	glia maturation factor gamma	nd	gi|19697925	Q8TDZ6	17020	6.0	164	6	5	45	unclassified	unknown
820	2	stathmin 1/oncoprotein 18	STMN1	gi|122890670	P16949	9858	6.8	168	5	27	41	cytoskeleton	SY
821	2	galectin-3	LGALS3	gi|28071074	Q86TY5	13945	9.2	183	5	14	46	Immunity	ME,NU
822	1	destrin isoform a	DEST	gi|5802966	P60981	18950	8.1	86	3	22	18	cytoskeleton	ME,EX,MT
823	3	NME1-NME2 protein	NDKB	gi|66392203	P22392	30346	9.1	336	6	10	42	multifunctional	CY,NU
824	1	cyclophilin A	PPIA	gi|1633054	P62937	18098	7.8	205	7	18	43	chaperone	ME,EX,MT
826	1	cyclophilin A	PPIA	gi|1633054	P62937	18098	7.8	218	7	19	43	chaperone	ME,EX,MT
828	1	cyclophilin A	PPIA	gi|1633054	P62937	18098	7.8	78	2	10	19	chaperone	ME,EX,MT
829	1	cyclophilin A	PPIA	gi|1633054	P62937	18098	7.8	76	3	20	28	chaperone	ME,EX,MT
830	1	myosin light polypeptide 6	MYL6	gi|113812151	P60660	15923	4.6	273	6	8	44	cytoskeleton	ME
831	2	chromosome 9 open reading frame 19	GAPR1	gi|11641247	Q9H4G4	17322	9.4	217	4	15	45	unknown	EX,GO
832	4	galectin-3	LGALS3	gi|28071074	Q86TY5	13945	9.2	258	7	16	64	Immunity	ME,NU
834	2	LIM domain-containing protein 2	LIMD2	gi|13386490	Q9BT23	14460	9.2	183	4	8	18	unclassified	unknown
837	2	R33729_1 (Interleukin-25)	CS010	gi|3355455	Q969H8	11383	7.0	176	4	9	39	signal trans.	ME,secreted
839	1	ubiquitin-conjugating enzyme E2 N	UBE2N	gi|83754516	P61088	17268	6.2	111	3	8	25	differentiation	ME,EX,MT
840	1	SH2 domain protein 1A	SH2D1A	gi|4506923	O60880	14349	8.7	196	4	14	60	signal trans.	CY
841	2	coactosin-like protein	COTL1	gi|56966036	Q14019	15898	5.6	255	6	15	51	unclassified	PL,SY
847	1	profilin-1	PROF1	gi|5822002	P07737	15085	8.5	373	8	29	50	actin binding	ME,EX,PL,MT
848	1	profilin-1	PROF1	gi|5822002	P07737	15085	8.5	505	10	30	59	actin binding	ME,EX,PL,MT
849	3	profilin-1	PROF1	gi|5822002	P07737	15085	8.5	287	7	12	58	actin binding	ME,EX,PL,MT
851	4	galectin-1	LGALS1	gi|42542977	P09382	14868	5.3	440	8	16	61	Immunity	ME,PL
852	1	histidine triad nucleotide binding protein 1	HINT1	gi|4885413	P49773	13907	6.4	193	3	6	46	Hydrolase	ME,PL,SY
856	1	FK506 binding protein 1A	FKBP1A	gi|5107718	P62942	11869	8.1	113	3	12	41	signal trans.	ME,SY,MT
857	3	cystatin B	CYTB	gi|4503117	P04080	11190	7.0	122	2	9	33	inhibitor	ME
862	1	Macrophage Migration Inhibitory Factor (Mif) With Hydroxphenylpyruvate	MIF	gi|5542151	P14174	12508	8.2	117	3	9	17	immunity	ME,EX,PL,SY
865	1	MHC class I antigen	HLA-A2	gi|229995	P61769	11592	6.5	232	7	18	72	Immunity	ME
867	1	BolA-like protein 2	BOLA2B	gi|46577124	Q9H3K6	10281	6.1	102	3	11	29	unknown	ME
872	1	leucocyte antigen CD97	CD97	gi|840771	P48960	83872	6.7	236	5	7	9	cell adhesion	ME,secreted
873	1	leucocyte antigen CD97	CD97	gi|840771	P48960	83872	6.7	75	4	10	7	cell adhesion	ME,secreted
882	1	ubiquitin-conjugating enzyme E2 L3	UBE2L3	gi|4507789	P68036	18021	8.7	168	4	12	34	degradation	MT
896	1	NADH dehydrogenase (ubiquinone) Fe-S protein 1, 75kDa (NADH-coenzyme Q reductase)	NDUS1	gi|21411235	P28331	80415	5.8	112	4	10	7	transport	ER,ME,MT
897	2	tubulin tyrosine ligase-like family, member 12	TTL12	gi|11056036	Q14166	75154	5.3	178	6	11	11	trafficking	ME
908	1	leucine-rich repeat and calponin homology domain-containing protein 5	LRCH4	gi|119596926	O75427	70861	6.9	307	20	26	38	protein binding	MT
913	1	ubiquitin associated and SH3 domain containing protein A	UBASH3A	gi|119629967	P57075	51314	7.6	81	4	9	9	degradation	CY,NU
917	2	moesin	MSN	gi|4505257	P26038	67892	6.1	572	36	24	58	cytoskeleton	EN,ME,EX,PL,MT
919	2	talin-1	TLN1	gi|81175200	Q9Y490	271766	5.8	618	37	10	19	cytoskeleton	EN,ME,PL
920	2	talin-1	TLN1	gi|81175200	Q9Y490	271766	5.8	803	45	10	24	cytoskeleton	EN,ME,PL
922	1	lymphocyte cytosolic protein 2	LCP2	gi|5031855	Q13094	60265	5.9	104	5	8	12	immunity	CY
929	1	echinoderm microtubule associated protein like 2 variant	EMAL2	gi|62089132	O95834	62949	5.7	98	4	14	10	cytoskeleton	CY
930	1	asparaginyl-tRNA synthetase	NARS	gi|119583453	O43776	63687	5.9	78	6	22	11	biosynthesis	ME
932	3	septin 2	SEPT2	gi|119591666	Q15019	18259	6.2	176	4	13	34	unclassified	ME,EX,SY
936	1	glucosidase, alpha; neutral AB, isoform CRA_a	GANAB	gi|119594451	Q14697	104930	5.9	127	14	25	16	hydrolases	ER,ME,PL
939	1	phosphofructokinase, liver	PFKL	gi|14286326	P17858	85720	7.0	84	6	14	11	glycolysis	unknown
940	1	HCLS1 protein	HCLS1	gi|48145705	Q6IBK9	54065	4.7	89	4	23	9	multifunctional	CY,MT
941	1	heat shock protein HSP 90-beta	HS90B	gi|6807647	P08238	85189	5.0	288	19	26	29	chaperone	ME,EX,MT
943	1	mitogen-activated protein kinase kinase 1 interacting protein 1	MPKS1	gi|11496277	Q9UHA4	13671	6.7	190	3	16	43	adapter protein	LY
944	2	proteasome subunit, beta type, 4	PSMB4	gi|551547	P28070	25950	5.7	72	2	5	12	hydrolase	CY,NU
945	2	peroxiredoxin 6	PRDX6	gi|4758638	P30041	25133	6.0	415	8	9	42	redox proteins	LY,ME,EX,PL,SY
946	1	phosphoglycerate mutase 1	PGAM1	gi|119570325	P18669	14942	9.3	69	2	14	24	metabolism	ME,EX,SY
947	1	eukaryotic translation elongation factor 1 gamma, isoform CRA_d	EEF1G	gi|119594432	P26641	48749	6.2	166	8	24	19	biosynthesis	ME
948	1	PHB	PHB	gi|49456373	Q6FHP5	29871	5.6	110	6	15	33	unclassified	unknown
951	3	EF-hand domain family, member D2	EFHD2	gi|20149675	Q96C19	26795	5.2	307	12	24	36	unknown	unknown
952	2	cytoskeleton associated protein	TBCB	gi|736704	Q99426	21959	4.8	90	2	3	10	cytoskeleton	CY
953	2	MHC class II antigen	HLA-DRB1	gi|77019293	Q3LAB6	21042	6.1	287	5	9	41	Immunity	ME
954	1	ACTB protein	ACTB	gi|15277503	P60709	40536	5.6	94	2	5	9	cytoskeleton	ER,ME,EX,PL,SY
955	4	aryl hydrocarbon receptor interacting protein	AIP	gi|118084558	A0SZW6	38049	5.8	309	6	15	22	unknown	unknown
957	3	GRAP2 protein	GRAP2	gi|49456653	Q6FHA6	37971	6.6	133	6	15	23	unclassified	unknown
958	1	Rab GDP dissociation inhibitor beta	GDI2	gi|56410847	Q5SX88	46046	5.9	213	4	9	14	GTPases	ME,EX,PL,MT
959	1	lymphocyte-specific protein 1	LSP1	gi|10880979	P33241	37397	4.7	608	14	33	54	Immunity	PL
960	2	lymphocyte-specific protein 1	LSP1	gi|10880979	P33241	37397	4.7	602	12	27	51	Immunity	PL
961	2	lymphocyte-specific protein 1	LSP1	gi|12804709	P33241	37427	4.7	442	9	28	38	Immunity	PL
964	2	phosphatidylinositol-5-phosphate 4-kinase, type II, alpha	PI42A	gi|6857820	P48426	46424	6.5	361	7	10	17	metabolism	NG,PL
966	1	adenylyl cyclase-associated protein	CAP1	gi|5453595	Q01518	51926	8.1	116	4	25	13	trafficking	PL,ME
969	1	heat shock protein HSP 90-alpha	HS90A	gi|154146191	P07900	85006	4.9	401	25	37	37	chaperone	ME,NG,MT
971	1	UNC-112 related protein 2 long form	URP2	gi|41281905	Q86UX7	76475	6.5	206	12	21	28	cell adhesion	PL
973	1	septin-9 gamma	SEPT9	gi|14530105	Q9UHD8	64894	7.2	350	13	12	21	unclassified	ME
974	1	moesin	MSN	gi|4505257	P26038	67892	6.1	662	36	29	57	cytoskeleton	EN,ME,EX,PL,MT
980	1	actin, alpha, cardiac muscle	ACTC1	gi|119612724	P68032	30498	4.9	109	2	6	9	cytoskeleton	ME
981	2	aldose reductase	ALDR	gi|13096112	P15121	36099	6.6	99	3	10	12	metabolism	ME,EX,MT
984	2	glyceraldehyde-3-phosphate dehydrogenase	GAPDH	gi|67464043	P04406	36483	8.6	621	16	32	53	metabolism	LY,ME,NG,EX,PL,SY,MT
985	1	serine/threonine phosphatase 1 gamma	PPP1CC	gi|5668560	Q9UPN1	34493	5.1	303	8	10	28	hydrolase	MT,SY
986	1	protein phosphatase 1, catalytic subunit, alpha isoform 3	PPP1CA	gi|56790945	P62136	39405	6.2	258	7	10	25	hydrolases	EX
987	2	ACTB protein	ACTB	gi|15277503	P60709	40536	5.6	259	10	17	28	cytoskeleton	ER,ME,EX,PL,SY
988	1	phosphoserine aminotransferase 1	SERC	gi|16741698	Q9Y617	40782	7.6	101	2	6	9	biosynthesis	ME
989	2	guanine nucleotide-binding protein subunit alpha-13	GNA13	gi|24111250	Q14344	44364	8.1	320	15	26	46	signal trans.	ME
992	1	tripeptidyl-peptidase 1	TPP1	gi|34452679	O14773	34670	5.7	226	3	9	13	protease	LY,ME,NG,PL,MT
993a	1	N-myc downstream regulated gene 1, isoform CRA_b	NAP1L1	gi|119612571	P55209	39908	6.1	88	5	11	26	cell proliferation	ME,PL,NU
993b	1	ACTB protein	ACTB	gi|15277503	P60709	40536	5.6	169	6	20	23	cytoskeleton	ER,ME,EX,PL,SY
994	1	enolase 1 variant	ENOA	gi|62896593	P06733	47453	7.0	476	19	18	58	metabolism	ME,EX,SY,MT
995	1	enolase 1 variant	ENOA	gi|62896593	P06733	47453	7.0	419	12	8	37	metabolism	ME,EX,SY,MT
998	1	Rho GTPase-activating protein 9	RHG09	gi|19584567	Q9BRR9	39593	6.7	206	11	14	36	GTPases	unknown
1000	1	differentially expressed in FDCP 6 homolog (mouse), isoform CRA_b	DEF6	gi|119624227	B3KSI1	44508	6.6	76	4	13	14	unknown	unknown
1001	1	Sec23B protein	SC23B	gi|13529299	Q15437	87377	6.4	135	4	13	7	trafficking	EN

spot no.[1]	replicats[2]	protein name [3]	gene	acc.no.[4]	Uni Prot no.[5]	MW, Da[6]	pI[7]	MASCOT [8]	matched peptides[9]	unmatched peptides [10]	% seq. coverage [11]	function [12]	subcellular localisation [13]
1002	2	ezrin	EZR	gi|46249758	Q6NUR7	69313	5.9	305	26	26	42	cytoskeleton	CY
1003	1	talin-1	TLN1	gi|81175200	Q9Y490	271766	5.8	285	7	6	4	cytoskeleton	EN,ME,PL
1004	1	talin-1	TLN1	gi|81175200	Q9Y490	271766	5.8	369	9	6	6	cytoskeleton	EN,ME,PL
1005	1	SHUJUN-1	MRLC2	gi|32187319	O14950	17047	4.2	81	2	6	14	cytoskeleton	CY
1006	1	small GTP binding protein Rac2, isoform CRA_c	RAC2	gi|119580555	B1AH80	21128	8.2	159	4	6	24	signal trans.	unknown
1007	1	peroxiredoxin 1	PRDX1	gi|55959888	Q06830	10727	8.8	80	2	3	21	redox proteins	ER,LY,EN,ME,NG,PL,MT
1008	1	triosephosphate isomerase (TIM) (Triose-phosphate isomerase)	TPIS	gi|136066	P00939	26894	7.1	165	3	4	13	unclassified	ME,EX,SY,MT
1009	2	proteasome subunit, alpha type, 5	PSMA5	gi|54696300	P28066	26579	4.7	193	3	8	17	hydrolases	ME
1010	1	NECAP endocytosis associated 2	NECAP2	gi|119572169	Q9NVZ3	24856	7.7	183	2	6	20	transport	EN
1011	3	nuclear chloride channel	CLIC1	gi|4588526	O00299	27249	5.0	268	7	16	37	channel	ME,EX,PL,MT
1012	3	EF-hand domain family, member D2	EFHD2	gi|20149675	Q96C19	26795	5.2	376	12	23	36	unknown	unknown
1014	2	farnesyl pyrophosphate synthetase	FPPS	gi|90109287	P14324	40458	5.1	127	3	16	11	biosynthesis	CY
1015	1	XRP2 protein	XRP2	gi|60416394	O75695	40471	5.0	149	3	11	12	biosynthesis	ME
1016	1	MHC class I antigen	HLA-A	gi|64976582	Q4W6C4	31862	5.9	351	9	11	45	Immunity	ME
1017	1	MHC class I antigen	HLA-A	gi|64976582	Q4W6C4	31862	5.9	264	7	11	34	Immunity	ME
1018	1	actin related protein 2	ACTR2	gi|109103172	P61160	38988	5.9	70	2	8	9	trafficking	EN,ER,ME
1019	1	actin related protein 2 isoform b	ACTR2	gi|5031571	P61160	45017	6.3	332	9	14	34	trafficking	EN,ER,ME
1020	1	MHC class I antigen	HLA-A	gi|12598440	P01892	31963	6.0	273	5	8	28	immunity	ME,EX
1021	1	phosphoglycerate kinase 1	PGK1	gi|119619008	P00558	28662	9.2	132	3	14	16	metabolism	ME,EX,SY,MT
1022	1	phosphoglycerate kinase 1	PGK1	gi|119619008	P00558	28662	9.2	128	4	10	22	metabolism	ME,EX,SY,MT
1023	1	adenylosuccinate synthetase	PURA2	gi|415849	P30520	50283	6.0	123	4	9	14	biosynthesis	CY
1024	1	zeta-chain associated protein kinase 70kDa isoform 1	ZAP70	gi|31455611	P43403	70796	7.8	154	4	8	8	signal trans.	CY
1027	1	ubiquitin specific peptidase 5 isoform 2	UBP5	gi|148727247	P45974	94104	5.0	78	3	6	4	degradation	LY,ME,NG
1029	4	Rab GDP dissociation inhibitor beta	GDI2	gi|6598323	P50395	51087	6.1	445	13	11	38	GTPases	ME,EX,PL,MT
1030	4	T-complex polypeptide 1	TCPA	gi|57863257	P17987	60819	5.8	305	19	22	36	chaperone	ER,EN,ME,EX
1032	1	MHC class I antigen	HLA-A	gi|118721276	A0ZXY8	30727	5.2	84	3	11	20	Immunity	ME
1037	1	26S protease regulatory subunit 6A	PSMC3	gi|338700	P17980	45422	5.3	68	2	3	7	degradation	CY,NU
1040	2	ACTB protein	ACTB	gi|15277503	P60709	40536	5.6	407	8	11	33	cytoskeleton	ER,ME,EX,PL,SY
1044	1	annexin A6	ANXA6	gi|71773329	P08133	76168	5.4	185	12	19	22	trafficking	MT,EX,ME,ER
1049	1	related RAS viral (r-ras) oncogene homolog 2 isoform a	RRAS2	gi|21361416	P62070	23613	5.7	137	4	7	25	GTPases	LY,ME,EX
1050	1	MHC class II antigen DR alpha chain	HLA-DRA	gi|3212400	P01903	20548	4.9	115	3	8	19	immunity	LY
1051	1	tubulin alpha 6 variant	nd	gi|62897609	Q53GA7	50476	5.0	134	6	19	20	cytoskeleton	ME,PL
1052	2	actinin, alpha 4	ACTN4	gi|119577215	O43707	104555	5.2	277	16	18	22	cytoskeleton	ME,NU,CY
1053	1	tyrosine-protein phosphatase non-receptor type 6	PTN6	gi|82407989	P29350	60638	6.1	69	6	19	12	signal trans.	CY,NU
1054	1	phosphoglycerate kinase 1	PGK1	gi|48145549	P00558	44973	8.3	390	14	27	37	metabolism	ME,EX,SY,MT
1055	1	guanine nucleotide binding protein (G protein), alpha inhibiting activity polypeptide 2, isoform CRA_d	GNAI2	gi|119585458	B4E2X5	20295	5.5	168	5	15	40	GTPases	EX
1056	1	aflatoxin aldehyde reductase AFAR	ARK72	gi|2736256	O43488	37024	6.2	157	3	10	13	redox	PL,GO
1057	1	annexin A6	ANXA6	gi|71773329	P08133	76168	5.4	179	10	10	19	trafficking	MT,EX,ME,ER
1058	1	protein disulfide isomerase-associated 4	PDIA4	gi|4758304	P13667	73229	5.0	78	3	7	4	chaperone	ER,ME,PL
1059	2	formin-binding protein 1	FBP17	gi|119608323	Q96RU3	87380	5.9	92	2	3	3	adapter	SL, LY, CY
1060	1	protein disulfide isomerase-associated 4	PDIA4	gi|4758304	P13667	73229	5.0	79	3	5	4	chaperone	ER,ME,PL
1061	1	kinase/transmembrane domain fusion protein	STK4/SLC36AL	gi|28372404	Q86YK3	28640	4.4	68	2	13	10	unclassified	unknown
1062	1	opa-interacting protein OIP3	KPYM	gi|2815606	unknown	18244	9.6	66	2	8	20	metabolism	ME,EX,SY
1063	1	tubulin alpha 6 variant	nd	gi|62897609	Q53GA7	50476	5.0	251	8	16	24	cytoskeleton	ME,PL
1064	2	tubulin alpha 6 variant	nd	gi|62897609	Q53GA7	50476	5.0	165	7	28	19	cytoskeleton	ME,PL
1065	1	tubulin fragment	TUBB	gi|16198437	Q96B85	30625	4.8	99	6	9	20	cytoskeleton	CY
1066	1	moesin	MSN	gi|14625824	P26038	62004	7.6	220	14	33	25	cytoskeleton	EN,ME,EX,PL,MT
1067	1	vacuolar sorting protein 33A	VPS33A	gi|119618724	Q96AX1	56346	6.7	114	4	8	9	trafficking	EN,LY
1069a	1	Ras GTPase-activating-like protein IQGAP2	IQGAP2	gi|13959394	Q13576	181071	5.4	150	5	11	4	signal trans.	EN
1069b	1	GTP-binding nuclear protein Ran	RAN	gi|5107682	P62826	23307	9.0	68	5	32	26	transport	ME,EX
1070	1	lin 7 homolog c	LIN7C	gi|62897873	Q9NUP9	21920	7.8	320	7	7	43	exocytosis	SY
1071	1	talin-1	TLN1	gi|81175200	Q9Y490	271766	5.8	398	20	9	12	cytoskeleton	EN,ME,PL
1072	1	histocompatibility (minor) HA-1	HMHA1	gi|40807045	Q92619	125785	5.8	188	4	6	4	GTPases	unknown
1073	1	histocompatibility (minor) HA-1, isoform CRA_c	HMHA1	gi|119589959	Q92619	108894	5.5	143	4	8	4	GTPases	unknown
1074	2	histocompatibility (minor) HA-1	HMHA1	gi|40807045	Q92619	125785	5.8	135	5	9	5	GTPases	unknown
1076	1	pyruvate kinase 3 isoform 2	PKM2	gi|67464392	P14618	60277	8.2	332	15	13	35	metabolism	ME,EX,SY
1077	1	talin-1	TLN1	gi|81175200	Q9Y490	271766	5.8	556	27	10	16	cytoskeleton	EN,ME,PL
1078	1	aldo-keto reductase family 1, member A1	AK1A1	gi|5174391	P14550	36892	6.3	213	10	18	36	metabolism	PL,SY
1079	1	actin, gamma 1 propeptide	ACTG1	gi|4501887	P63261	42108	5.3	257	10	35	35	cytoskeleton	ME,EX,PL,SY
1080	1	phosphoribosyl pyrophosphate synthetase 1 variant	nd	gi|62897899	Q53FW2	35355	6.3	249	9	14	35	biosynthesis	unknown
1081	1	SH3-containing protein, Endophilin-B1	SH3GLB1	gi|7705773	Q9Y371	41056	5.8	130	4	19	13	apoptosis	CY,GO,MT
1082	1	poly(rC) binding protein 1	PCBP1	gi|5453854	Q15365	38015	6.7	359	11	29	45	unclassified	ME,CY,NU
1083	2	MHC class II antigen DR52	HLA-DRB3	gi|6715261	P01913	27220	6.8	178	6	29	34	Immunity	ME
