# Supplementary material for: Effector granules in human T lymphocytes: the luminal proteome of secretory lysosomes from human T cells
Source: Cell Commun Signal. 2011 Jan 21;9:4. doi: 10.1186/1478-811X-9-4 (PMC3034720; doi:10.1186/1478-811X-9-4)
Supplement: Additional file 2 — Figure S1. Proteome map of enriched secretory lysosomes from T cells. The 742 annotated spots are displayed in four separately enlarged quadrants (A-D) of one representative of the six performed 2D gels. Identifications are combined based on six repetitive experiments. [file 1478-811X-9-4-S2.PDF]

Additional file 2

Effector granules in T human lymphocytes: the luminal proteome of secretory lysosomes from human T cells.

Hendrik Schmidt[1], Christoph Gelhaus[2], Melanie Nebendahl[1], Marcus Lettau[1], Ralph Lucius[3], Dieter Kabelitz[1] and Ottmar Janssen[1]

[1]Institute of Immunology, Christian-Albrechts-University, UK S-H Campus Kiel, Kiel, Germany
[2]Zophysiology, Zoological Institute, Christian-Albrechts-University, Kiel, Germany
[3]Institute of Anatomy, Christian-Albrechts-University, Kiel, Germany

Figure S1. Proteome map of enriched secretory lysosomes from T cells. The 742 annotated spots are displayed in four separately enlarged quadrants (A-D) of one representative of the six performed 2D gels. Identifications are combined based on six repetitive experiments.

A

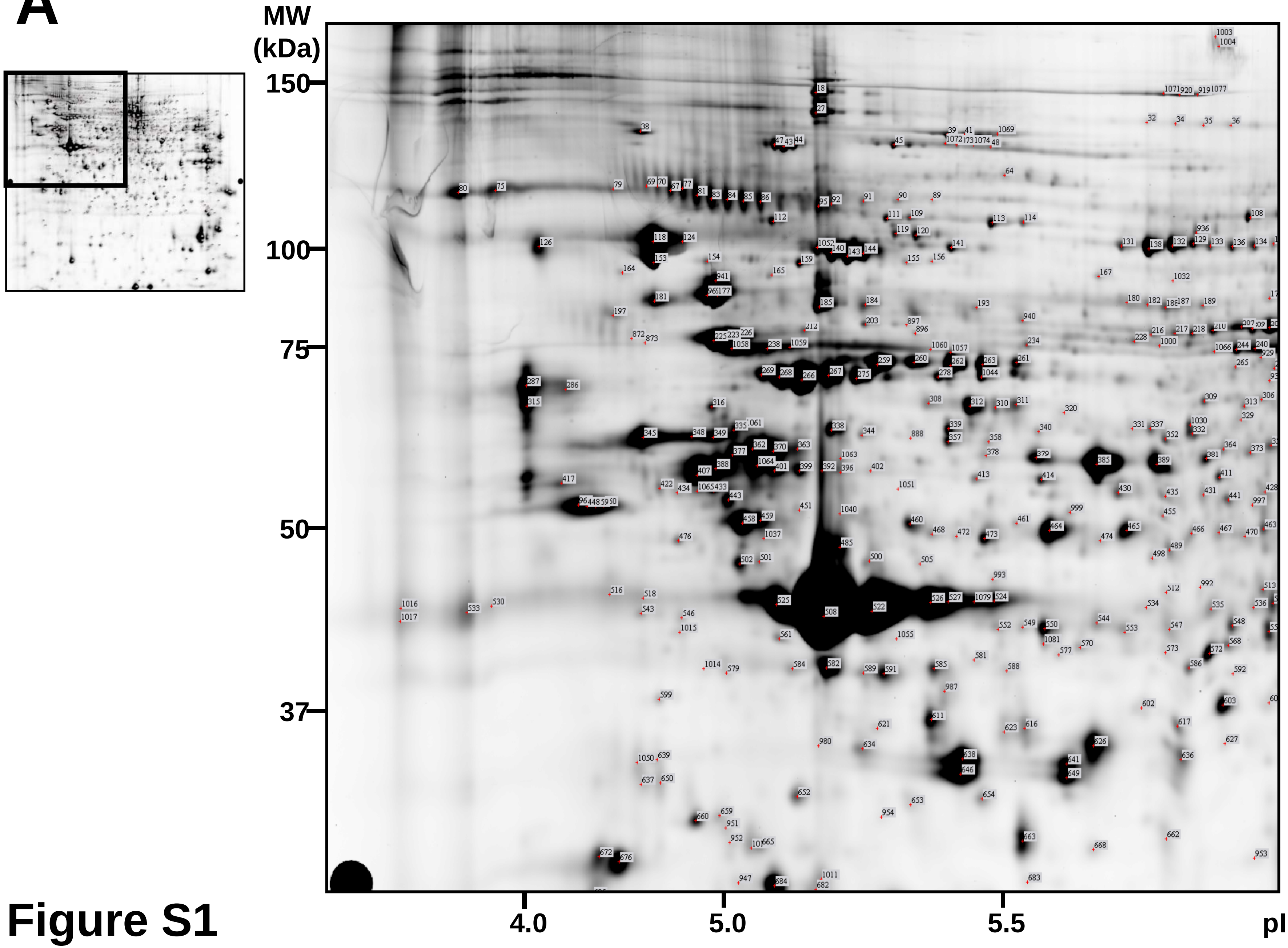

Figure S1

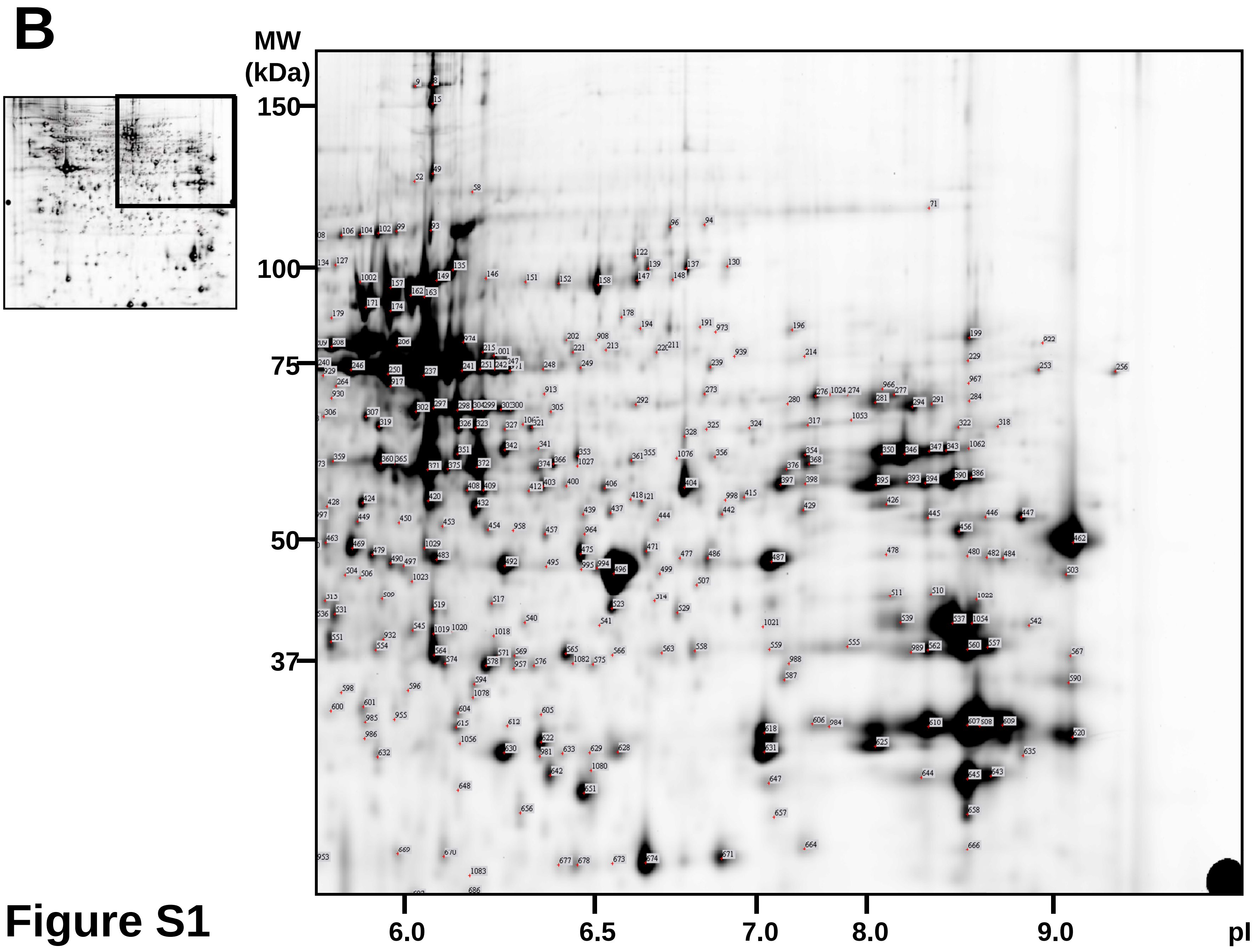

Figure S1

C

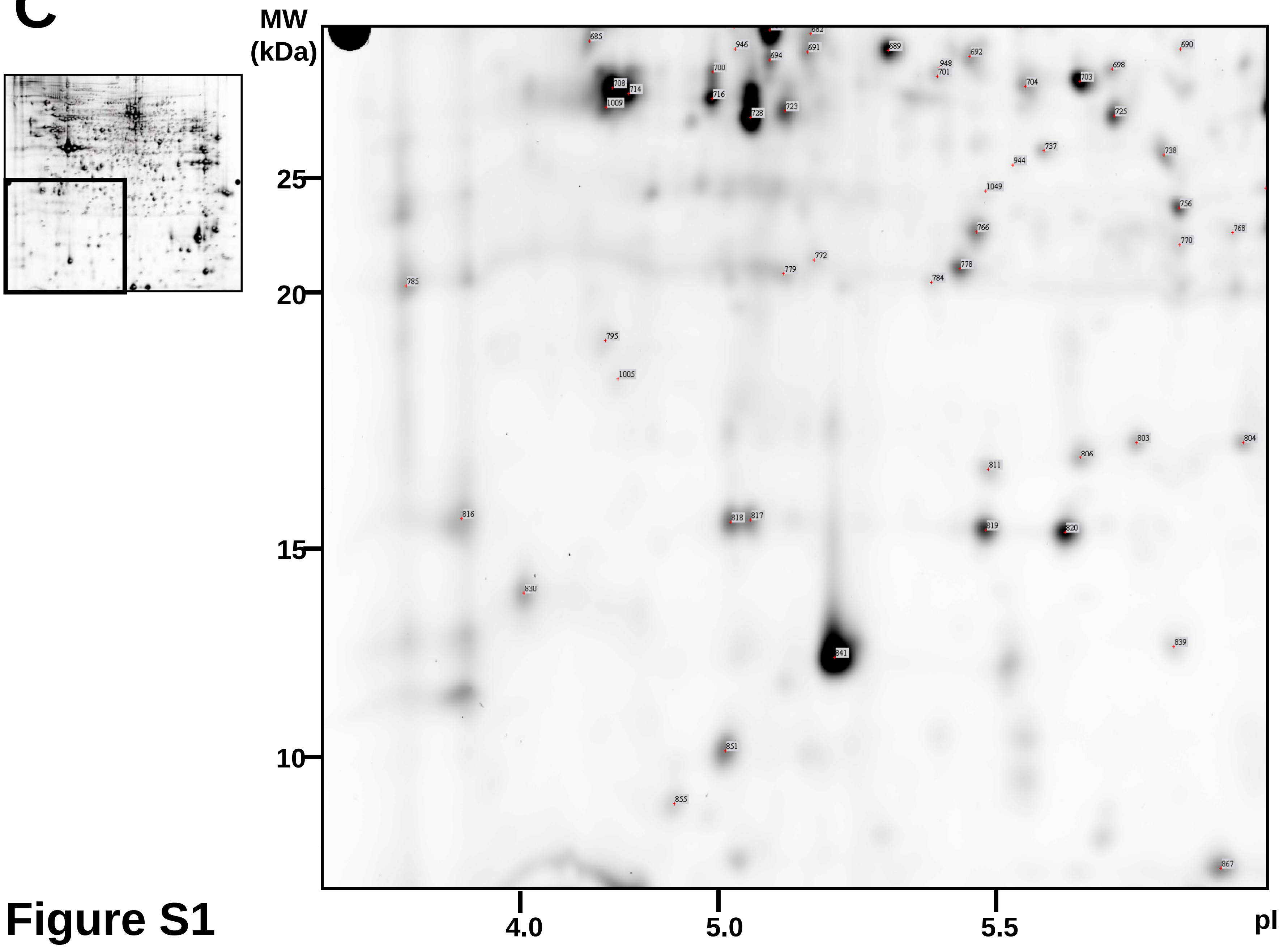

Figure S1

D

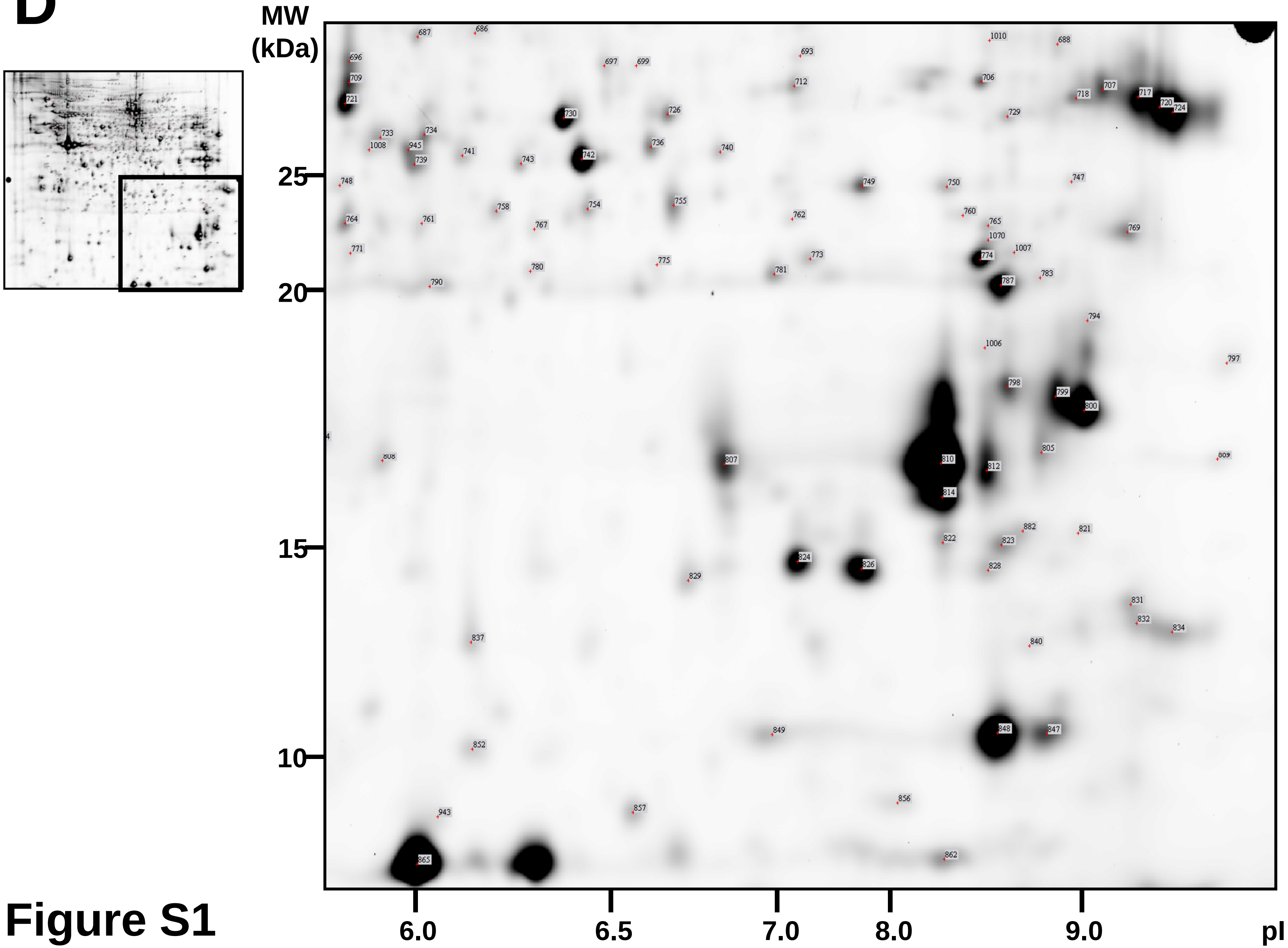

Figure S1
