# Supplementary material for: Effector granules in human T lymphocytes: the luminal proteome of secretory lysosomes from human T cells
Source: Cell Commun Signal. 2011 Jan 21;9:4. doi: 10.1186/1478-811X-9-4 (PMC3034720; doi:10.1186/1478-811X-9-4)
Supplement: Additional file 3 — Dataset S1. Protein identification data. Protein identification data are displayed as MASCOT's "Protein View" including matched peptides, sequence coverage and ion scores (for MS/MS identifications). Please use bookmarks for navigation. [file 1478-811X-9-4-S3.PDF]

Additional file 3

Effector granules in T human lymphocytes: the luminal proteome of secretory lysosomes from human T cells.

Hendrik Schmidt[1], Christoph Gelhaus[2], Melanie Nebendahl[1], Marcus Lettau[1], Ralph Lucius[3], Dieter Kabelitz[1] and Ottmar Janssen[1]

[1]Institute of Immunology, Christian-Albrechts-University, UK S-H Campus Kiel, Kiel, Germany
[2]Zophysiology, Zoological Institute, Christian-Albrechts-University, Kiel, Germany
[3]Institute of Anatomy, Christian-Albrechts-University, Kiel, Germany

Dataset S1. Protein identification data. Protein identification data are displayed as MASCOT's "Protein View" including matched peptides, sequence coverage and ion scores (for MS/MS identifications). Please use bookmarks for navigation.

Spot 8

Protein View

Match to: **gi|1002923** Score: **208** Expect: **3e-016**
coronin-like protein

Nominal mass (M_r): **51722**; Calculated pI value: **6.12**
NCBI BLAST search of gi|1002923 against nr
Unformatted sequence string for pasting into other applications

Taxonomy: Homo sapiens

Fixed modifications: Carbamidomethyl (C)
Variable modifications: Oxidation (M)
Cleavage by Trypsin: cuts C-term side of KR unless next residue is P
Sequence Coverage: **24%**

Matched peptides shown in **Bold Red**

```
  1 MSRQVVRTSK FRHVFGQPAK ADQCYEDVRV SQTTWDSGFC AVNPKFVALI
 51 CEASGGGAFL VLPLGKTGRV DKNAPTVCGH TAPVLDIAWC PHNDNVIASG
101 SEDCTVMVWE IPDGGLMLPL REPVVTLEGH TKRVGIVAWH TTAQNVLLSA
151 GCDNVIMVWD VGTGAAMLTL GPEVHPDTIY SVDWSRDGGL ICTSCRDKRV
201 RIIEPRKGTV VAEKDRPHEG TRPVRAVFVS EGKILTTGFS RMSEWQVALW
251 DTKHLEEPLS LQELDTSSGV LLPFFDPDTN IVYLCGKGDS SIRYFEITSE
301 APFLHYLSMF SSKESQRGMG YMPKRGLEVN KCEIARFYKL HERRCEPIAM
351 TVPRKSDLFQ EDLYPPTAGP DPALTAEEWL GGRDAGPLLI SLKDGYVPPK
401 SRELRVNRGL DTGRRRAAPE ASGTPSSDAV SRLEEEMRKL QATVQELQKR
451 LDRLEETVQA K
```

Residue Number Increasing Mass Decreasing Mass

Start - End	Observed	Mr(expt)	Mr(calc)	Delta	Miss	Sequence
11 - 20	1186.6351	1185.6278	1185.6406	-0.0128	1	FRHVFGQPAK (No match)
11 - 20	1186.6351	1185.6278	1185.6406	-0.0128	1	FRHVFGQPAK (Ions score 29)
13 - 20	883.5206	882.5133	882.4711	0.0422	0	HVFGQPAK (No match)
13 - 29	2019.9012	2018.8939	2018.9268	-0.0329	1	HVFGQPAKADQCYEDVR (No match)
13 - 29	2019.9012	2018.8939	2018.9268	-0.0329	1	HVFGQPAKADQCYEDVR (Ions score 21)
21 - 29	1155.4622	1154.4549	1154.4662	-0.0113	0	ADQCYEDVR (No match)
21 - 29	1155.4622	1154.4549	1154.4662	-0.0113	0	ADQCYEDVR (No match)

187 - 196	1138.4885	1137.4812	1137.4906	-0.0094	0	DGGLICTSCR	(Ions score 6)
187 - 196	1138.4885	1137.4812	1137.4906	-0.0094	0	DGGLICTSCR	(No match)
215 - 225	1319.6721	1318.6648	1318.6854	-0.0206	0	DRPHEGTRPVR	(Ions score 26)
215 - 225	1319.6721	1318.6648	1318.6854	-0.0206	0	DRPHEGTRPVR	(No match)
226 - 241	1711.9125	1710.9052	1710.9304	-0.0251	1	AVFVSEGKILTTGFSR	(No match)
234 - 241	894.4986	893.4913	893.4970	-0.0057	0	ILTTGFSR	(No match)
294 - 313	2413.1150	2412.1077	2412.1347	-0.0270	0	YFEITSEAPFLHYLSMFSSK	Oxidation (M) (No match)
326 - 336	1288.6566	1287.6493	1287.6604	-0.0111	1	GLEVNKCEIAR	(No match)
344 - 354	1345.6548	1344.6475	1344.6642	-0.0167	1	RCEPIAMTVPR	Oxidation (M) (No match)
384 - 400	1782.9717	1781.9644	1781.9927	-0.0282	1	DAGPLLISLKDGYVPPK	(No match)

Mascot: http://www.matrixscience.com/

Spot 9

Protein View

Match to: **gi|119625804** Score: **203** Expect: **9.6e-016**
moesin, isoform CRA_b [Homo sapiens]

Nominal mass (M_r): **66678;** Calculated pI value: **5.90**
NCBI BLAST search of gi|119625804 against nr
Unformatted sequence string for pasting into other applications

Taxonomy: Homo sapiens

Fixed modifications: Carbamidomethyl (C)
Variable modifications: Oxidation (M)
Cleavage by Trypsin: cuts C-term side of KR unless next residue is P
Sequence Coverage: **24%**

Matched peptides shown in **Bold Red**

```
  1 MDAELEFAIQ PNTTGKQLFD QVVKTIGLRE VWFFGLQYQD TKGFSTWLKL
 51 NKKVTAQDVR KESPLLFKFR AKFYPEDVSE ELIQDITQRL FFLQVKEGIL
101 NDDIYCPPET AVLLASYAVQ SKYGDFNKEV HKSGYLAGDK LLPQRVLEQH
151 KLNKDQWEER IQVWHEEHRG MLREDAVLEY LKIAQDLEMY GVNYFSIKNK
201 KGSELWLGVD ALGLNIYEQN DRLTPKIGFP WSEIRNISFN DKKFVIKPID
251 KKAPDFVFYA PRLRINKRIL ALCMGNHELY MRRRKPDTIE VQQMKAQARE
301 EKHQKQMERA MLENEKKKRE MAEKEKEKIE REKEELMERL KQIEEQTKKA
351 QQELEEQTRR ALELEQERKR AQSEAEKLAK ERQEAEEAKE ALLQASRDQK
401 KTQEQLALEM AELTARISQL EMARQKKESE AVEWQQKAQM VQEDLEKTRA
451 ELKTAMSTPH VAEPAENEQD EQDENGAEAS ADLRADAMAK DRSEEERTTE
501 AEKNERVQKH LKALTSELAN ARDESKKTAN DMIHAENMRL GRDKYKTLRQ
551 IRQGNTKQRI DEFESM
```

Residue Number Increasing Mass Decreasing Mass

Start - End	Observed	Mr(expt)	Mr(calc)	Delta	Miss	Sequence
17 - 24	976.5220	975.5147	975.5389	-0.0242	0	QLFDQVVK (No match)
71 - 89	2281.0923	2280.0850	2280.1273	-0.0423	1	AKFYPEDVSEELIQDITQR (No match)
73 - 89	2081.9663	2080.9590	2080.9953	-0.0362	0	FYPEDVSEELIQDITQR (No match)
90 - 96	894.5305	893.5232	893.5374	-0.0142	0	LFFLQVK (No match)
133 - 145	1417.7603	1416.7530	1416.7724	-0.0194	1	SGYLAGDKLLPQR (No match)

133 - 145	1417.7603	1416.7530	1416.7724	-0.0194	1	SGYLAGDKLLPQR (Ions score 18)
146 - 154	1108.5702	1107.5629	1107.6400	-0.0771	1	VLEQHKLNK (No match)
146 - 154	1108.5702	1107.5629	1107.6400	-0.0771	1	VLEQHKLNK (No match)
170 - 182	1552.7825	1551.7752	1551.7966	-0.0214	1	GMLREDAVLEYLK Oxidation (M) (No match)
227 - 235	1104.5780	1103.5707	1103.5763	-0.0056	0	IGFPWSEIR (No match)
252 - 262	1310.6732	1309.6659	1309.6818	-0.0159	1	KAPDFVFYAPR (No match)
252 - 262	1310.6732	1309.6659	1309.6818	-0.0159	1	KAPDFVFYAPR (Ions score 10)
253 - 262	1182.5789	1181.5716	1181.5869	-0.0153	0	APDFVFYAPR (Ions score 65)
253 - 262	1182.5789	1181.5716	1181.5869	-0.0153	0	APDFVFYAPR (No match)
269 - 282	1752.7969	1751.7896	1751.8157	-0.0260	0	ILALCMGNHELYMR 2 Oxidation (M) (No match)
350 - 360	1387.6729	1386.6656	1386.6851	-0.0195	1	AQQELEEQTRR (No match)
361 - 368	987.4987	986.4914	986.5032	-0.0118	0	ALELEQER (No match)
498 - 506	1077.5125	1076.5052	1076.5098	-0.0045	1	TTEAEKNER (No match)
558 - 566	1170.4918	1169.4845	1169.5022	-0.0177	1	QRIDEFESM Oxidation (M) (No match)

Mascot: http://www.matrixscience.com/

Spot 15

Protein View

Match to: **gi|5902134** Score: **412** Expect: **1.2e-036**
coronin, actin binding protein, 1A [Homo sapiens]

Nominal mass (M_r): **51678**; Calculated pI value: **6.25**
NCBI BLAST search of gi|5902134 against nr
Unformatted sequence string for pasting into other applications

Taxonomy: Homo sapiens
Links to retrieve other entries containing this sequence from NCBI Entrez:
gi|1706004 from Homo sapiens
gi|20271119 from Homo sapiens
gi|927649 from Homo sapiens
gi|1136140 from Homo sapiens
gi|82571468 from Homo sapiens
gi|116497053 from Homo sapiens
gi|116497211 from Homo sapiens
gi|119600312 from Homo sapiens
gi|119600313 from Homo sapiens
gi|119600314 from Homo sapiens
gi|119600316 from Homo sapiens

Fixed modifications: Carbamidomethyl (C)
Variable modifications: Oxidation (M)
Cleavage by Trypsin: cuts C-term side of KR unless next residue is P
Sequence Coverage: **47%**

Matched peptides shown in **Bold Red**

1 MSRQVVRSSK **FRHVFGQPAK ADQCYEDVRV SQTTWDSGFC AVNPKFVALI**
51 **CEASGGGAFL VLPLGK**TGRV DKNAPTVCGH TAPVLDIAWC PHNDNVIASG
101 SEDCTVMVWE IPDGGLMLPL REPVVTLEGH TKRVGIVAWH TTAQNVLLSA
151 GCDNVIMVWD VGTGAAMLTL GPEVHPDTIY SVDWSR**DGGL ICTSCR**DKRV
201 RIIEPR**KGTV VAEKDRPHEG TRPVRAVFVS EGKILTTGFS R**MSER**QVALW**
251 **DTK**HLEEPLS LQELDTSSGV LLPFFDPDTN IVYLCGKGDS SIR**YFEITSE**
301 **APFLHYLSMF SSK**ESQRGMG YMPK**RGLEVN KCEIARFYKL HERRCEPIAM**
351 **TVPRKSDLFQ EDLYPPTAGP DPALTAEEWL GGRDAGPLLI SLKDGYVPPK**
401 SR**ELRVNRGL DTGRR**RAAPE ASGTPSSDAV SRLEEEMRKL QATVQELQKR
451 LDRLEETVQA K

Residue Number Increasing Mass Decreasing Mass

Start - End	Observed	Mr(expt)	Mr(calc)	Delta	Miss	Sequence
11 - 20	1186.6328	1185.6255	1185.6406	-0.0151	1	**FRHVFGQPAK** (Ions score 36)
11 - 20	1186.6328	1185.6255	1185.6406	-0.0151	1	**FRHVFGQPAK** (No match)
13 - 20	883.5243	882.5170	882.4711	0.0459	0	**HVFGQPAK** (No match)
13 - 29	2019.9071	2018.8998	2018.9268	-0.0270	1	**HVFGQPAKADQCYEDVR** (No match)
21 - 29	1155.4618	1154.4545	1154.4662	-0.0117	0	**ADQCYEDVR** (No match)
21 - 29	1155.4618	1154.4545	1154.4662	-0.0117	0	**ADQCYEDVR** (Ions score 14)
30 - 45	1796.8093	1795.8020	1795.8199	-0.0179	0	**VSQTTWDSGFCAVNPK** (No match)
46 - 66	2119.1516	2118.1443	2118.1546	-0.0103	0	**FVALICEASGGGAFLVLPLGK** (No match)
187 - 196	1138.4874	1137.4801	1137.4906	-0.0105	0	**DGGLICTSCR** (Ions score 39)
187 - 196	1138.4874	1137.4801	1137.4906	-0.0105	0	**DGGLICTSCR** (No match)
207 - 214	831.4816	830.4743	830.4861	-0.0118	1	**KGTVVAEK** (No match)
208 - 225	2004.0524	2003.0451	2003.0660	-0.0209	1	**GTVVAEKDRPHEGTRPVR** (No match)
215 - 225	1319.6766	1318.6693	1318.6854	-0.0161	0	**DRPHEGTRPVR** (No match)
215 - 225	1319.6766	1318.6693	1318.6854	-0.0161	0	**DRPHEGTRPVR** (Ions score 46)
226 - 233	836.4387	835.4314	835.4439	-0.0125	0	**AVFVSEGK** (No match)
226 - 241	1711.9171	1710.9098	1710.9304	-0.0205	1	**AVFVSEGKILTTGFSR** (No match)
234 - 241	894.4968	893.4895	893.4970	-0.0075	0	**ILTTGFSR** (No match)
246 - 253	960.5007	959.4934	959.5076	-0.0142	0	**QVALWDTK** (No match)
294 - 313	2397.1074	2396.1001	2396.1398	-0.0397	0	**YFEITSEAPFLHYLSMFSSK** (No match)
294 - 313	2413.1279	2412.1206	2412.1347	-0.0141	0	**YFEITSEAPFLHYLSMFSSK** Oxidation (M) (No match)
325 - 331	815.4659	814.4586	814.4660	-0.0074	1	**RGLEVNK** (No match)
326 - 336	1288.6561	1287.6488	1287.6604	-0.0116	1	**GLEVNKCEIAR** (No match)
337 - 343	992.5209	991.5136	991.5239	-0.0103	1	**FYKLHER** (No match)
344 - 354	1329.6630	1328.6557	1328.6693	-0.0136	1	**RCEPIAMTVPR** (No match)
344 - 354	1345.6556	1344.6483	1344.6642	-0.0159	1	**RCEPIAMTVPR** Oxidation (M) (No match)
345 - 354	1173.5533	1172.5460	1172.5682	-0.0222	0	**CEPIAMTVPR** (No match)
345 - 354	1189.5760	1188.5687	1188.5631	0.0056	0	**CEPIAMTVPR** Oxidation (M) (No match)
355 - 383	3173.5107	3172.5034	3172.5352	-0.0318	1	**KSDLFQEDLYPPTAGPDPALTAEEWLGGR** (No match)
384 - 393	1026.6012	1025.5939	1025.6120	-0.0181	0	**DAGPLLISLK** (No match)
384 - 400	1782.9836	1781.9763	1781.9927	-0.0163	1	**DAGPLLISLKDGYVPPK** (No match)
403 - 408	786.4485	785.4412	785.4507	-0.0095	1	**ELRVNR** (No match)
409 - 415	774.4084	773.4011	773.4144	-0.0132	1	**GLDTGRR** (No match)

Spot 18

Protein View

Match to: **gi|15277503** Score: **265** Expect: **6.1e-022**
ACTB protein [Homo sapiens]

Nominal mass (M_r): **40536**; Calculated pI value: **5.55**
NCBI BLAST search of gi|15277503 against nr
Unformatted sequence string for pasting into other applications

Taxonomy: Homo sapiens

Fixed modifications: Carbamidomethyl (C)
Variable modifications: Oxidation (M)
Cleavage by Trypsin: cuts C-term side of KR unless next residue is P
Sequence Coverage: **31%**

Matched peptides shown in **Bold Red**

```
  1 MCKAGFAGDD APRAVFPSIV GRPRHQGVMV GMGQKDSYVG DEAQSKRGIL
 51 TLKYPIEHGI VTNWDDMEKI WHHTFYNELR VAPEEHPVLL TEAPLNPKAN
101 LEKMTQIMFE TFNTPAMYVA IQAVLSLYAS GRTTGIVMDS GDGVTHTVPI
151 YEGYALPHAI LRLDLAGRDL TDYLMKILTE RGYSFTTTAE REIVRDIKEK
201 LCYVALDFEQ EMATAASSSS LEKSYELPDG QVITIGNERF RCPEALFQPS
251 FLGMESCGIH ETTFNSIMKC DVDIRKDLYA NTVLSGGTTM YPGIADRMQK
301 EITALAPSTM KIKIIAPPER KYSVWIGGSI LASLSTFQQM WISKQEYDES
351 GPSIVHRKCF
```

Residue Number Increasing Mass Decreasing Mass

Start - End	Observed	Mr(expt)	Mr(calc)	Delta	Miss	Sequence
4 - 13	976.4481	975.4408	975.4409	-0.0001	0	AGFAGDDAPR (No match)
14 - 24	1198.6945	1197.6872	1197.6982	-0.0109	0	AVFPSIVGRPR (Ions score 23)
14 - 24	1198.6945	1197.6872	1197.6982	-0.0109	0	AVFPSIVGRPR (No match)
81 - 98	1954.0472	1953.0399	1953.0571	-0.0171	0	VAPEEHPVLLTEAPLNPK (No match)
81 - 98	1954.0472	1953.0399	1953.0571	-0.0171	0	VAPEEHPVLLTEAPLNPK (Ions score 61)
163 - 176	1639.8239	1638.8166	1638.8287	-0.0120	1	LDLAGRDLTDYLMK Oxidation (M) (No match)
182 - 191	1132.5208	1131.5135	1131.5196	-0.0061	0	GYSFTTTAER (No match)
182 - 191	1132.5208	1131.5135	1131.5196	-0.0061	0	GYSFTTTAER (No match)
224 - 239	1790.8745	1789.8672	1789.8846	-0.0173	0	SYELPDGQVITIGNER (Ions score 73)
224 - 239	1790.8745	1789.8672	1789.8846	-0.0173	0	SYELPDGQVITIGNER (No match)
277 - 297	2231.0674	2230.0601	2230.0575	0.0026	0	DLYANTVLSGGTTMYPGIADR Oxidation (M) (No match)

345 - 357	1516.6986	1515.6913	1515.6953	-0.0040	0	QEYDESGPSIVHR	(Ions score 27)
345 - 357	1516.6986	1515.6913	1515.6953	-0.0040	0	QEYDESGPSIVHR	(No match)

Mascot: http://www.matrixscience.com/

Spot 27

Protein View

Match to: **gi|15277503** Score: **351** Expect: **1.5e-030**
ACTB protein [Homo sapiens]

Nominal mass (M_r): **40536;** Calculated pI value: **5.55**
NCBI BLAST search of gi|15277503 against nr
Unformatted sequence string for pasting into other applications

Taxonomy: Homo sapiens

Fixed modifications: Carbamidomethyl (C)
Variable modifications: Oxidation (M)
Cleavage by Trypsin: cuts C-term side of KR unless next residue is P
Sequence Coverage: **38%**

Matched peptides shown in **Bold Red**

```
  1 MCKAGFAGDD APRAVFPSIV GRPRHQGVMV GMGQKDSYVG DEAQSKRGIL
 51 TLKYPIEHGI VTNWDDMEKI WHHTFYNELR VAPEEHPVLL TEAPLNPKAN
101 LEKMTQIMFE TFNTPAMYVA IQAVLSLYAS GRTTGIVMDS GDGVTHTVPI
151 YEGYALPHAI LRLDLAGRDL TDYLMKILTE RGYSFTTTAE REIVRDIKEK
201 LCYVALDFEQ EMATAASSSS LEKSYELPDG QVITIGNERF RCPEALFQPS
251 FLGMESCGIH ETTFNSIMKC DVDIRKDLYA NTVLSGGTTM YPGIADRMQK
301 EITALAPSTM KIKIIAPPER KYSVWIGGSI LASLSTFQQM WISKQEYDES
351 GPSIVHRKCF
```

Residue Number Increasing Mass Decreasing Mass

Start - End	Observed	Mr(expt)	Mr(calc)	Delta	Miss	Sequence
4 - 13	976.4473	975.4400	975.4409	-0.0009	0	AGFAGDDAPR (No match)
14 - 24	1198.6991	1197.6918	1197.6982	-0.0063	0	AVFPSIVGRPR (Ions score 44)
14 - 24	1198.6991	1197.6918	1197.6982	-0.0063	0	AVFPSIVGRPR (No match)
36 - 47	1354.6160	1353.6087	1353.6160	-0.0073	1	DSYVGDEAQSKR (No match)
81 - 98	1954.0554	1953.0481	1953.0571	-0.0089	0	VAPEEHPVLLTEAPLNPK (No match)
81 - 98	1954.0554	1953.0481	1953.0571	-0.0089	0	VAPEEHPVLLTEAPLNPK (Ions score 91)
163 - 176	1639.8292	1638.8219	1638.8287	-0.0067	1	LDLAGRDLTDYLMK Oxidation (M) (No match)
169 - 181	1626.8190	1625.8117	1625.8334	-0.0217	1	DLTDYLMKILTER Oxidation (M) (No match)
182 - 191	1132.5242	1131.5169	1131.5196	-0.0027	0	GYSFTTTAER (No match)

182 - 191	1132.5242	1131.5169	1131.5196	-0.0027	0	GYSFTTTAER (No match)
224 - 239	1790.8785	1789.8712	1789.8846	-0.0133	0	SYELPDGQVITIGNER (No match)
224 - 239	1790.8785	1789.8712	1789.8846	-0.0133	0	SYELPDGQVITIGNER (Ions score 89)
277 - 297	2231.0479	2230.0406	2230.0575	-0.0169	0	DLYANTVLSGGTTMYPGIADR Oxidation (M) (No match)
312 - 320	1036.6510	1035.6437	1035.6440	-0.0003	1	IKIIAPPER (No match)
345 - 357	1516.6985	1515.6912	1515.6953	-0.0041	0	QEYDESGPSIVHR (No match)

Mascot: http://www.matrixscience.com/

Spot 32

Protein View

Match to: **gi|119594451** Score: **116** Expect: **4.8e-007**
glucosidase, alpha; neutral AB, isoform CRA_a [Homo sapiens]

Nominal mass (M_r): **104930**; Calculated pI value: **5.85**
NCBI BLAST search of gi|119594451 against nr
Unformatted sequence string for pasting into other applications

Taxonomy: Homo sapiens

Fixed modifications: Carbamidomethyl (C)
Variable modifications: Oxidation (M)
Cleavage by Trypsin: cuts C-term side of KR unless next residue is P
Sequence Coverage: **9%**

Matched peptides shown in **Bold Red**

```
  1 MAAVAAVAAR RRRSWASLVL AFLGVCLGIT LAVDRSNFKT CEESSFCKRQ
 51 RSIRPGLSPY RALLDSLQLG PDSLTVHLIH EVTKVLLVLE LQGLQKNMTR
101 FRIDELEPRR PRYRVPDVLV ADPPIARLSV SGRDENSVEL TMAEGPYKII
151 LTARPFRLDL LEDRSLLLSV NARGLLEFEH QRAPRVSEEE TQGKAEKDEP
201 GAWEETFKTH SDSKPYGPMS VGLDFSLPGM EHVYGIPEHA DNLRLKVTEG
251 GEPYRLYNLD VFQYELYNPM ALYGSVPVLL AHNPHRDLGI FWLNAAETWV
301 DISSNTAGKT LFGKMMDYLQ GSGETPQTDV RWMSETGIID VFLLLGPSIS
351 DVFRQYASLT GTQALPPLFS LGYHQSRWNY RDEADVLEVD QGFDDHNLPC
401 DVIWLDIEHA DGKRYFTWDP SRFPQPRTML ERLASKRRKL VAIVDPHIKV
451 DSGYRVHEEL RNLGLYVKTR DGSDYEGWCW PGSAGYPDFT NPTMRAWWAN
501 MFSYDNYEGS APNLFVWNDM NEPSVFNGPE VTMLKDAQHY GGWEHRDVHN
551 IYGLYVHMAT ADGLRQRSGG MERPFVLARA FFAGSQRFGA VWTGDNTAEW
601 DHLKISIPMC LSLGLVGLSF CGADVGGFFK NPEPELLVRW YQMGAYQPFF
651 RAHAHLDTGR REPWLLPSQH NDIIRDALGQ RYSLLPFWYT LLYQAHREGI
701 PVMRPLWVQY PQDVTTFNID DQYLLGDALL VHPVSDSGAH GVQVYLPGQG
751 EVWYDIQSYQ KHHGPQTLYL PVTLSSIPVF QRGGTIVPRW MRVRRSSECM
801 KDDPITLFVA LSPQGTAQGE LFLDDGHTFN YQTRQEFLLR RFSFSGNTLV
851 SSSADPEGHF ETPIWIERVV IIGAGKPAAV VLQTKGSPES RLSFQHDPET
901 SVLVLRKPGI NVASDWSIHL R
```

Matched peptides (bold red): SIRPGLSPYR, FRIDELEPR, YRVPDVLVADPPIAR, GLLEFEHQR, DAQHYGGWEHR, AFFAGSQR, QEFLLR, LSFQHDPETSVLVLR

Residue Number Increasing Mass Decreasing Mass

Start - End	Observed	Mr(expt)	Mr(calc)	Delta	Miss	Sequence
52 - 61	1145.6152	1144.6079	1144.6352	-0.0273	0	SIRPGLSPYR (No match)
52 - 61	1145.6152	1144.6079	1144.6352	-0.0273	0	SIRPGLSPYR (No match)
101 - 109	1174.5950	1173.5877	1173.6142	-0.0264	1	FRIDELEPR (No match)
101 - 109	1174.5950	1173.5877	1173.6142	-0.0264	1	FRIDELEPR (No match)
113 - 127	1680.8951	1679.8878	1679.9358	-0.0480	1	YRVPDVLVADPPIAR (No match)
113 - 127	1680.8951	1679.8878	1679.9358	-0.0480	1	YRVPDVLVADPPIAR (Ions score 19)
174 - 182	1128.5533	1127.5460	1127.5723	-0.0263	0	GLLEFEHQR (Ions score 20)
174 - 182	1128.5533	1127.5460	1127.5723	-0.0263	0	GLLEFEHQR (No match)
536 - 546	1355.5625	1354.5552	1354.5802	-0.0250	0	DAQHYGGWEHR (No match)
580 - 587	883.4672	882.4599	882.4347	0.0252	0	AFFAGSQR (No match)
835 - 840	805.4469	804.4396	804.4493	-0.0097	0	QEFLLR (No match)
892 - 906	1740.8892	1739.8819	1739.9206	-0.0387	0	LSFQHDPETSVLVLR (No match)
892 - 906	1740.8892	1739.8819	1739.9206	-0.0387	0	LSFQHDPETSVLVLR (Ions score 13)

Mascot: http://www.matrixscience.com/

Spot 34

Protein View

Match to: **gi|119594451** Score: **95** Expect: **6.7e-005**
glucosidase, alpha; neutral AB, isoform CRA_a [Homo sapiens]

Nominal mass (M_r): **104930**; Calculated pI value: **5.85**
NCBI BLAST search of gi|119594451 against nr
Unformatted sequence string for pasting into other applications

Taxonomy: Homo sapiens

Fixed modifications: Carbamidomethyl (C)
Variable modifications: Oxidation (M)
Cleavage by Trypsin: cuts C-term side of KR unless next residue is P
Sequence Coverage: **12%**

Matched peptides shown in **Bold Red**

```
  1 MAAVAAVAAR RRRSWASLVL AFLGVCLGIT LAVDRSNFKT CEESSFCKRQ
 51 RSIRPGLSPY RALLDSLQLG PDSLTVHLIH EVTKVLLVLE LQGLQKNMTR
101 FRIDELEPRR PRYRVPDVLV ADPPIARLSV SGRDENSVEL TMAEGPYKII
151 LTARPFRLDL LEDRSLLLSV NARGLLEFEH QRAPRVSEEE TQGKAEKDEP
201 GAWEETFKTH SDSKPYGPMS VGLDFSLPGM EHVYGIPEHA DNLRLKVTEG
251 GEPYRLYNLD VFQYELYNPM ALYGSVPVLL AHNPHRDLGI FWLNAAETWV
301 DISSNTAGKT LFGKMMDYLQ GSGETPQTDV RWMSETGIID VFLLLGPSIS
351 DVFRQYASLT GTQALPPLFS LGYHQSRWNY RDEADVLEVD QGFDDHNLPC
401 DVIWLDIEHA DGKRYFTWDP SRFPQPRTML ERLASKRRKL VAIVDPHIKV
451 DSGYRVHEEL RNLGLYVKTR DGSDYEGWCW PGSAGYPDFT NPTMRAWWAN
501 MFSYDNYEGS APNLFVWNDM NEPSVFNGPE VTMLKDAQHY GGWEHRDVHN
551 IYGLYVHMAT ADGLRQRSGG MERPFVLARA FFAGSQRFGA VWTGDNTAEW
601 DHLKISIPMC LSLGLVGLSF CGADVGGFFK NPEPELLVRW YQMGAYQPFF
651 RAHAHLDTGR REPWLLPSQH NDIIRDALGQ RYSLLPFWYT LLYQAHREGI
701 PVMRPLWVQY PQDVTTFNID DQYLLGDALL VHPVSDSGAH GVQVYLPGQG
751 EVWYDIQSYQ KHHGPQTLYL PVTLSSIPVF QRGGTIVPRW MRVRRSSECM
801 KDDPITLFVA LSPQGTAQGE LFLDDGHTFN YQTRQEFLLR RFSFSGNTLV
851 SSSADPEGHF ETPIWIERVV IIGAGKPAAV VLQTKGSPES RLSFQHDPET
901 SVLVLRKPGI NVASDWSIHL R
```

Residue Number Increasing Mass Decreasing Mass

Start - End	Observed	Mr(expt)	Mr(calc)	Delta	Miss	Sequence
52 - 61	1145.6086	1144.6013	1144.6352	-0.0339	0	SIRPGLSPYR (No match)
52 - 61	1145.6086	1144.6013	1144.6352	-0.0339	0	SIRPGLSPYR (No match)
101 - 109	1174.5876	1173.5803	1173.6142	-0.0338	1	FRIDELEPR (No match)
113 - 127	1680.8868	1679.8795	1679.9358	-0.0563	1	YRVPDVLVADPPIAR (No match)
174 - 182	1128.5490	1127.5417	1127.5723	-0.0306	0	GLLEFEHQR (Ions score 13)
174 - 182	1128.5490	1127.5417	1127.5723	-0.0306	0	GLLEFEHQR (No match)
547 - 565	2144.9883	2143.9810	2144.0472	-0.0662	0	DVHNIYGLYVHMATADGLR (No match)
568 - 579	1335.6385	1334.6312	1334.6764	-0.0452	0	SGGMERPFVLAR Oxidation (M) (No match)
580 - 587	883.4818	882.4745	882.4347	0.0398	0	AFFAGSQR (No match)
762 - 782	2390.2170	2389.2097	2389.2906	-0.0809	0	HHGPQTLYLPVTLSSIPVFQR (No match)
892 - 906	1740.8774	1739.8701	1739.9206	-0.0505	0	LSFQHDPETSVLVLR (Ions score 12)
892 - 906	1740.8774	1739.8701	1739.9206	-0.0505	0	LSFQHDPETSVLVLR (No match)

Mascot: http://www.matrixscience.com/

Spot 35

Protein View

Match to: **gi|386975** Score: **88** Expect: **0.00032**
neutrophil adherence receptor alpha-M subunit

Nominal mass (M_r): **127628**; Calculated pI value: **6.73**
NCBI BLAST search of gi|386975 against nr
Unformatted sequence string for pasting into other applications

Taxonomy: Homo sapiens

Fixed modifications: Carbamidomethyl (C)
Variable modifications: Oxidation (M)
Cleavage by Trypsin: cuts C-term side of KR unless next residue is P
Sequence Coverage: **8%**

Matched peptides shown in **Bold Red**

```
   1 TALTLCHGFN LDTENAMTFQ ENARGFGQSV VQLQGSRVVV GAPQEIVAAN
  51 QRGSLYQCDY STGSCEPIRL QVPVEAVNMS LGLSLAATTS PPQLLACGPT
 101 VHQTCSENTY VKGLCFLFGS NLRQQPQKFP EALRGCPQED SDIAFLIDGS
 151 GSIIPHDFRR MKEFVSTVME QLKKSKTLFS LMQYSEEFRI HFTFKEFQNN
 201 PNPRSLVKPI TQLLGRTHTA TGIRKVVREL FNITNGARKN AFKILVVITD
 251 GEKFGDPLGY EDVIPEADRE GVIRYVIGVG DAFRSEKSRQ ELNTIASKPP
 301 RDHVFQVNNF EALKTIQNQL REKIFAIEGT QTGSSSSFEH EMSQEGFSAA
 351 ITSNGPLLST VGSYDWAGGV FLYTSKEKST FINMTRVDSD MNDAYLGYAA
 401 AIILRNRVQS LVLGAPRYQH IGLVAMFRQN TGMWESNANV KGTQIGAYFG
 451 ASLCSVDVDS NGSTDLVLIG APHYYEQTRG GQVSVCPLPR GQRARWQCDA
 501 VLYGEQGQPW GRFGAALTVL GDVNGDKLTD VAIGAPGEED NRGAVYLFHG
 551 TSGSGISPSH SQRIAGSKLS PRLQYFGQSL SGGQDLTMDG LVDLTVGAQG
 601 HVLLLRSQPV LRVKAIMEFN PREVARNVFE CNDQVVKGKE AGEVRVCLHV
 651 QKSTRDRLRE GQIQSVVTYD LALDSGRPHS RAVFNETKNS TRRQTQVLGL
 701 TQTCETLKLQ LPNCIEDPVS PIVLRLNFSL VGTPLSAFGN LRPVLAEDAQ
 751 RLFTALFPFE KNCGNDNICQ DDLSITFSFM SLDCLVVGGP REFNVTVTVR
 801 NDGEDSYRTQ VTFFFPLDLS YRKVSTLQNQ RSQRSWRLAC ESASSTEVSG
 851 ALKSTSCSIN HPIFPENSEV TFNITFDVDS KASLGNKLLL KANVTSENNM
 901 PRTNKTEFQL ELPVKYAVYM VVTSHGVSTK YLNFTASENT SRVMQHQYQV
 951 SNLGQRSLPI SLVFLVPVRL NQTVIWDRPQ VTFSENLSST CHTKERLPSH
1001 SDFLAELRKA PVVNCSIAVC QRIQCDIPFF GIQEEFNATL KGNLSFDWYI
1051 KTSHNHLLIV STAEILFNDS VFTLLPGQGA FVRSQTETKV EPFEVPNPLP
1101 LIVGSSVGGL LLLALITAAL YKLGFFKRQY KDMMSEGGPP GAEPQ
```

Residue Number Increasing Mass Decreasing Mass

Start - End	Observed	Mr(expt)	Mr(calc)	Delta	Miss	Sequence
25 - 37	1362.6971	1361.6898	1361.7051	-0.0153	0	GFGQSVVQLQGSR (No match)
38 - 52	1550.8485	1549.8412	1549.8575	-0.0163	0	VVVGAPQEIVAANQR (No match)
38 - 52	1550.8485	1549.8412	1549.8575	-0.0163	0	VVVGAPQEIVAANQR (Ions score 9)
205 - 216	1324.8065	1323.7992	1323.8238	-0.0245	0	SLVKPITQLLGR (No match)
275 - 284	1096.5714	1095.5641	1095.5712	-0.0071	0	YVIGVGDAFR (No match)
275 - 284	1096.5714	1095.5641	1095.5712	-0.0071	0	YVIGVGDAFR (Ions score 6)
543 - 563	2144.9988	2143.9915	2144.0398	-0.0483	0	GAVYLFHGTSGSGISPSHSQR (No match)
709 - 725	1963.0315	1962.0242	1962.0608	-0.0366	0	LQLPNCIEDPVSPIVLR (No match)
997 - 1008	1384.7024	1383.6951	1383.7146	-0.0195	0	LPSHSDFLAELR (No match)

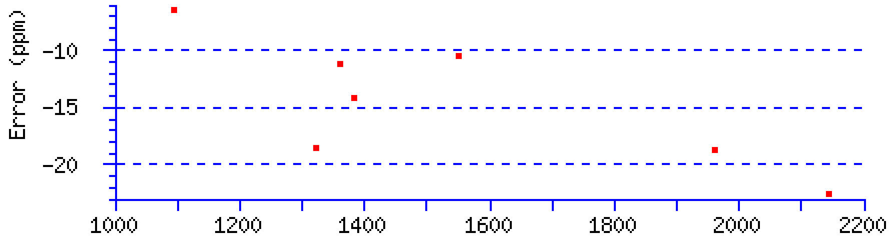

RMS error 14 ppm

Mascot: http://www.matrixscience.com/

Spot 36

Protein View

Match to: **gi|386975** Score: **89** Expect: **0.00023**
neutrophil adherence receptor alpha-M subunit

Nominal mass (M_r): **127628**; Calculated pI value: **6.73**
NCBI BLAST search of gi|386975 against nr
Unformatted sequence string for pasting into other applications

Taxonomy: Homo sapiens

Fixed modifications: Carbamidomethyl (C)
Variable modifications: Oxidation (M)
Cleavage by Trypsin: cuts C-term side of KR unless next residue is P
Sequence Coverage: **8%**

Matched peptides shown in **Bold Red**

```
   1 TALTLCHGFN LDTENAMTFQ ENARGFGQSV VQLQGSRVVV GAPQEIVAAN
  51 QRGSLYQCDY STGSCEPIRL QVPVEAVNMS LGLSLAATTS PPQLLACGPT
 101 VHQTCSENTY VKGLCFLFGS NLRQQPQKFP EALRGCPQED SDIAFLIDGS
 151 GSIIPHDFRR MKEFVSTVME QLKKSKTLFS LMQYSEEFRI HFTFKEFQNN
 201 PNPRSLVKPI TQLLGRTHTA TGIRKVVREL FNITNGARKN AFKILVVITD
 251 GEKFGDPLGY EDVIPEADRE GVIRYVIGVG DAFRSEKSRQ ELNTIASKPP
 301 RDHVFQVNNF EALKTIQNQL REKIFAIEGT QTGSSSSFEH EMSQEGFSAA
 351 ITSNGPLLST VGSYDWAGGV FLYTSKEKST FINMTRVDSD MNDAYLGYAA
 401 AIILRNRVQS LVLGAPRYQH IGLVAMFRQN TGMWESNANV KGTQIGAYFG
 451 ASLCSVDVDS NGSTDLVLIG APHYYEQTRG GQVSVCPLPR GQRARWQCDA
 501 VLYGEQGQPW GRFGAALTVL GDVNGDKLTD VAIGAPGEED NRGAVYLFHG
 551 TSGSGISPSH SQRIAGSKLS PRLQYFGQSL SGGQDLTMDG LVDLTVGAQG
 601 HVLLLRSQPV LRVKAIMEFN PREVARNVFE CNDQVVKGKE AGEVRVCLHV
 651 QKSTRDRLRE GQIQSVVTYD LALDSGRPHS RAVFNETKNS TRRQTQVLGL
 701 TQTCETLKLQ LPNCIEDPVS PIVLRLNFSL VGTPLSAFGN LRPVLAEDAQ
 751 RLFTALFPFE KNCGNDNICQ DDLSITFSFM SLDCLVVGGP REFNVTVTVR
 801 NDGEDSYRTQ VTFFFPLDLS YRKVSTLQNQ RSQRSWRLAC ESASSTEVSG
 851 ALKSTSCSIN HPIFPENSEV TFNITFDVDS KASLGNKLLL KANVTSENNM
 901 PRTNKTEFQL ELPVKYAVYM VVTSHGVSTK YLNFTASENT SRVMQHQYQV
 951 SNLGQRSLPI SLVFLVPVRL NQTVIWDRPQ VTFSENLSST CHTKERLPSH
1001 SDFLAELRKA PVVNCSIAVC QRIQCDIPFF GIQEEFNATL KGNLSFDWYI
1051 KTSHNHLLIV STAEILFNDS VFTLLPGQGA FVRSQTETKV EPFEVPNPLP
1101 LIVGSSVGGL LLLALITAAL YKLGFFKRQY KDMMSEGGPP GAEPQ
```

Residue Number Increasing Mass Decreasing Mass

Start - End	Observed	Mr(expt)	Mr(calc)	Delta	Miss	Sequence
25 - 37	1362.7065	1361.6992	1361.7051	-0.0059	0	GFGQSVVQLQGSR (No match)
38 - 52	1550.8535	1549.8462	1549.8575	-0.0113	0	VVVGAPQEIVAANQR (No match)
38 - 52	1550.8535	1549.8462	1549.8575	-0.0113	0	VVVGAPQEIVAANQR (Ions score 22)
113 - 123	1283.6445	1282.6372	1282.6492	-0.0119	0	GLCFLFGSNLR (No match)
205 - 216	1324.8221	1323.8148	1323.8238	-0.0089	0	SLVKPITQLLGR (No match)
275 - 284	1096.5696	1095.5623	1095.5712	-0.0089	0	YVIGVGDAFR (No match)
543 - 563	2145.0276	2144.0203	2144.0398	-0.0195	0	GAVYLFHGTSGSGISPSHSQR (No match)
997 - 1008	1384.7018	1383.6945	1383.7146	-0.0201	0	LPSHSDFLAELR (No match)

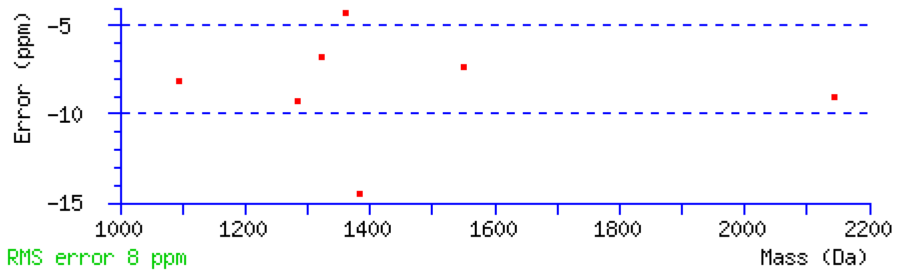

Mascot: http://www.matrixscience.com/

Spot 38

Protein View

Match to: **gi|16757970** Score: **265** Expect: **6.1e-022**
niban protein isoform 2 [Homo sapiens]

Nominal mass (M_r): **104039**; Calculated pI value: **4.74**
NCBI BLAST search of gi|16757970 against nr
Unformatted sequence string for pasting into other applications

Taxonomy: Homo sapiens
Links to retrieve other entries containing this sequence from NCBI Entrez:
gi|22256936 from Homo sapiens
gi|12620192 from Homo sapiens
gi|20988569 from Homo sapiens
gi|55957189 from Homo sapiens
gi|55957231 from Homo sapiens
gi|55957260 from Homo sapiens
gi|56203712 from Homo sapiens
gi|119611590 from Homo sapiens
gi|119611591 from Homo sapiens

Fixed modifications: Carbamidomethyl (C)
Variable modifications: Oxidation (M)
Cleavage by Trypsin: cuts C-term side of KR unless next residue is P
Sequence Coverage: **12%**

Matched peptides shown in **Bold Red**

1 MGGSASSQLD EGKCAYIRGK TEAAIK**NFSP YYSRQYSVAF CNHVR**TEVEQ
51 QRDLTSQFLK TKPPLAPGTI LYEAELSQFS EDIKKWKERY VVVK**NDYAVE**
101 **SYENKEAYQR** GAAPKCRILP AGGK**VLTSED EYNLLSDR**HF PDPLASSEKE
151 NTQPFVVLPK EFPVYLWQPF FR**HGYFCFHE AADQKRFSAL LSDCVR**HLNH
201 DYMKQMTFEA QAFLEAVQFF RQEKGHYGSW EMITGDEIQI LSNLVMEELL
251 PTLQTDLLPK MKGKKNDRKR TWLGLLEEAY TLVQHQVSEG LSALKEECRA
301 LTKGLEGTIR SDMDQIVNSK NYLIGKIKAM VAQPAEKSCL ESVQPFLASI
351 LEELMGPVSS GFSEVRVLFE KEVNEVSQNF QTTKDSVQLK EHLDRLMNLP
401 LHSVKMEPCY TK**VNLLHER**L QDLKSR**FRFP HIDLVVQR**TQ NYMQELMENA
451 VFTFEQLLSP HLQGEASKTA VAIEKVKLRV LKQYDYDSST IRKKIFQEAL
501 VQITLPTVQK ALASTCKPEL QKYEQFIFAD HTNMIHVENV YEEILHQILL
551 DETLKVIKEA AILKKHNLFE DNMALPSESV SSLTDLKPPT GSNQASPARR
601 ASAILPGVLG SETLSNEVFQ ESEEEKQPEV PSSLAK**GESL SLPGPSPPPD**
651 **GTEQVIISR**V DDPVVNPVAT EDTAGLPGTC SSELEFGGTL EDEEPAQEEP
701 EPITASGSLK ALRKLLTASV EVPVDSAPVM EEDTNGESHV PQENEEEEEK

```
751 EPSQAAAIHP DNCEESEVSE REAQPPCPEA HGEELGGFPE VGSPASPPAS
801 GGLTEEPLGP MEGELPGEAC TLTAHEGRGG KCTEEGDASQ QEGCTLGSDP
851 ICLSESQVSE EQEEMGGQSS AAQATASVNA EEIKVARIHE CQWVVEDAPN
901 PDVLLSHKDD VKEGEGGQES FPELPSEE
```

Residue Number Increasing Mass Decreasing Mass

Start - End	Observed	Mr(expt)	Mr(calc)	Delta	Miss	Sequence
27 - 34	1033.4569	1032.4496	1032.4664	-0.0168	0	**NFSPYYSR** (No match)
27 - 34	1033.4569	1032.4496	1032.4664	-0.0168	0	**NFSPYYSR** (No match)
35 - 45	1380.6117	1379.6044	1379.6404	-0.0360	0	**QYSVAFCNHVR** (Ions score 33)
35 - 45	1380.6117	1379.6044	1379.6404	-0.0360	0	**QYSVAFCNHVR** (No match)
95 - 110	1978.8060	1977.7987	1977.8704	-0.0716	1	**NDYAVESYENKEAYQR** (No match)
125 - 138	1653.7480	1652.7407	1652.7893	-0.0486	0	**VLTSEDEYNLLSDR** (No match)
173 - 186	1765.7271	1764.7198	1764.7790	-0.0592	1	**HGYFCFHEAADQKR** (No match)
187 - 196	1167.5676	1166.5603	1166.5753	-0.0150	0	**FSALLSDCVR** (No match)
413 - 419	880.4886	879.4813	879.4926	-0.0113	0	**VNLLHER** (No match)
427 - 438	1526.8136	1525.8063	1525.8517	-0.0454	1	**FRFPHIDLVVQR** (No match)
427 - 438	1526.8136	1525.8063	1525.8517	-0.0454	1	**FRFPHIDLVVQR** (Ions score 28)
429 - 438	1223.6624	1222.6551	1222.6822	-0.0271	0	**FPHIDLVVQR** (No match)
637 - 659	2333.1072	2332.0999	2332.1910	-0.0911	0	**GESLSLPGPSPPPDGTEQVIISR** (No match)
637 - 659	2333.1072	2332.0999	2332.1910	-0.0911	0	**GESLSLPGPSPPPDGTEQVIISR** (Ions score 118)

Spot 39

Protein View

Match to: **gi|9910280** Score: **124** Expect: **7.7e-008**
UDP-glucose ceramide glucosyltransferase-like 1 isoform 1 [Homo sapiens]

Nominal mass (M_r): **177819**; Calculated pI value: **5.42**
NCBI BLAST search of gi|9910280 against nr
Unformatted sequence string for pasting into other applications

Taxonomy: Homo sapiens
Links to retrieve other entries containing this sequence from NCBI Entrez:
gi|7670746 from Homo sapiens

Fixed modifications: Carbamidomethyl (C)
Variable modifications: Oxidation (M)
Cleavage by Trypsin: cuts C-term side of KR unless next residue is P
Sequence Coverage: **7%**

Matched peptides shown in **Bold Red**

1 MGCKGDASGA CAAGALPVTG VCYKMGVLVV LTVLWLFSSV KADSKAITTS
51 LTTKWFSTPL LLEASEFLAE DSQEKFWNFV EASQNIGSSD HDGTDYSYYH
101 AILEAAFQFL SPLQQNLFKF CLSLRSYSAT IQAFQQIAAD EPPPEGCNSF
151 FSVHGKKTCE SDTLEALLLT ASERPKPLLF KGDHRYPSSN PESPVVIFYS
201 EIGSEEFSNF HRQLISKSNA GK**INYVFRHY IFNPR**KEPVY LSGYGVELAI
251 KSTEYKAKDD TQVKGTEVNT TVIGENDPID EVQGFLFGKL RDLHPDLEGQ
301 LKELRKHLVE STNEMAPLK**V WQLQDLSFQT AAR**ILASPVE LALVVMKDLS
351 QNFPTKARAI TKTAVSSELR TEVEENQKYF KGTLGLQPGD SALFINGLHM
401 DLDTQDIFSL FDVLRNEARV MEGLHRLGIE GLSLHNVLK**L NIQPSEADYA**
451 **VDIR**SPAISW VNNLEVDSRY NSWPSSLQEL LRPTFPGVIR QIRKNLHNMV
501 FIVDPAHETT AELMNTAEMF LSNHIPLRIG FIFVVNDSED VDGMQDAGVA
551 VLRAYNYVAQ EVDDYHAFQT LTHIYNKVRT GEKVKVEHVV SVLEKKYPYV
601 EVNSILGIDS AYDRNRKEAR GYYEQTGVGP LPVVLFNGMP FEREQLDPDE
651 LETITMHK**IL ETTTFFQR**AV YLGELPHDQD VVEYIMNQPN VVPRINSRIL
701 TAERDYLDLT ASNNFFVDDY ARFTILDSQG KTAAVANSMN YLTKKGMSSK
751 EIYDDSFIRP VTFWIVGDFD SPSGRQLLYD AIKHQKSSNN VRISMINNPA
801 KEISYENTQI SRAIWAALQT QTSNAAKNFI TKMAKEGAAE ALAAGADIAE
851 FSVGGMDFSL FKEVFESSKM DFILSHAVYC RDVLKLKKGQ RAVISNGRII
901 GPLEDSELFN QDDFHLLENI ILKTSGQKIK SHIQQLRVEE DVASDLVMKV
951 DALLSAQPKG DPR**IEYQFFE DR**HSAIKLRP KEGETYFDVV AVVDPVTREA
1001 QRLAPLLLVL AQLINMNLRV FMNCQSKLSD MPLKSFYRYV LEPEISFTSD
1051 NSFAKGPIAK FLDMPQSPLF TLNLNTPESW MVESVRTPYD LDNIYLEEVD
1101 SVVAAEYELE YLLLEGHCYD ITTGQPPRGL QFTLGTSANP VIVDTIVMAN

```
1151 LGYFQLKANP GAWILRLRKG RSEDIYRIYS HDGTDSPPDA DEVVIVLNNF
1201 KSKIIKVKVQ KKADMVNEDL LSDGTSENES GFWDSFKWGF TGQKTEEVKQ
1251 DKDDIINIFS VASGHLYERF LRIMMLSVLK NTKTPVKFWF LKNYLSPTFK
1301 EFIPYMANEY NFQYELVQYK WPRWLHQQTE KQRIIWGYKI LFLDVLFPLV
1351 VDKFLFVDAD QIVRTDLKEL RDFNLDGAPY GYTPFCDSRR EMDGYRFWKS
1401 GYWASHLAGR KYHISALYVV DLKKFRKIAA GDRLRGQYQG LSQDPNSLSN
1451 LDQDLPNNMI HQVPIKSLPQ EWLWCETWCD DASKKRAKTI DLCNNPMTKE
1501 PKLEAAVRIV PEWQDYDQEI KQLQIRFQKE KETGALYKEK TKEPSREGPQ
1551 KREEL
```

Residue Number Increasing Mass Decreasing Mass

Start - End	Observed	Mr(expt)	Mr(calc)	Delta	Miss	Sequence
223 - 228	811.4439	810.4366	810.4388	-0.0021	0	INYVFR (No match)
229 - 235	946.4893	945.4820	945.4820	-0.0000	0	HYIFNPR (No match)
320 - 333	1662.8350	1661.8277	1661.8525	-0.0248	0	VWQLQDLSFQTAAR (No match)
440 - 454	1703.8497	1702.8424	1702.8526	-0.0101	0	LNIQPSEADYAVDIR (No match)
440 - 454	1703.8497	1702.8424	1702.8526	-0.0101	0	LNIQPSEADYAVDIR (Ions score 21)
659 - 668	1255.6663	1254.6590	1254.6608	-0.0018	0	ILETTTFFQR (Ions score 0)
659 - 668	1255.6663	1254.6590	1254.6608	-0.0018	0	ILETTTFFQR (No match)
964 - 972	1246.5728	1245.5655	1245.5665	-0.0010	0	IEYQFFEDR (Ions score 31)
964 - 972	1246.5728	1245.5655	1245.5665	-0.0010	0	IEYQFFEDR (No match)
1158 - 1166	997.5602	996.5529	996.5504	0.0025	0	ANPGAWILR (No match)
1354 - 1364	1322.7017	1321.6944	1321.7030	-0.0085	0	FLFVDADQIVR (No match)
1354 - 1364	1322.7017	1321.6944	1321.7030	-0.0085	0	FLFVDADQIVR (Ions score 5)
1372 - 1389	2094.8987	2093.8914	2093.8788	0.0126	0	DFNLDGAPYGYTPFCDSR (No match)
1400 - 1410	1204.5870	1203.5797	1203.5784	0.0013	0	SGYWASHLAGR (No match)

Mascot: http://www.matrixscience.com/

Spot 41

Protein View

Match to: **gi|9910280** Score: **113** Expect: **9.6e-007**
UDP-glucose ceramide glucosyltransferase-like 1 isoform 1 [Homo sapiens]

Nominal mass (M_r): **177819**; Calculated pI value: **5.42**
NCBI BLAST search of gi|9910280 against nr
Unformatted sequence string for pasting into other applications

Taxonomy: Homo sapiens
Links to retrieve other entries containing this sequence from NCBI Entrez:
gi|7670746 from Homo sapiens

Fixed modifications: Carbamidomethyl (C)
Variable modifications: Oxidation (M)
Cleavage by Trypsin: cuts C-term side of KR unless next residue is P
Sequence Coverage: **5%**

Matched peptides shown in **Bold Red**

```
   1 MGCKGDASGA CAAGALPVTG VCYKMGVLVV LTVLWLFSSV KADSKAITTS
  51 LTTKWFSTPL LLEASEFLAE DSQEKFWNFV EASQNIGSSD HDGTDYSYYH
 101 AILEAAFQFL SPLQQNLFKF CLSLRSYSAT IQAFQQIAAD EPPPEGCNSF
 151 FSVHGKKTCE SDTLEALLLT ASERPKPLLF KGDHRYPSSN PESPVVIFYS
 201 EIGSEEFSNF HRQLISKSNA GKINYVFRHY IFNPRKEPVY LSGYGVELAI
 251 KSTEYKAKDD TQVKGTEVNT TVIGENDPID EVQGFLFGKL RDLHPDLEGQ
 301 LKELRKHLVE STNEMAPLKV WQLQDLSFQT AARILASPVE LALVVMKDLS
 351 QNFPTKARAI TKTAVSSELR TEVEENQKYF KGTLGLQPGD SALFINGLHM
 401 DLDTQDIFSL FDVLRNEARV MEGLHRLGIE GLSLHNVLKL NIQPSEADYA
 451 VDIRSPAISW VNNLEVDSRY NSWPSSLQEL LRPTFPGVIR QIRKNLHNMV
 501 FIVDPAHETT AELMNTAEMF LSNHIPLRIG FIFVVNDSED VDGMQDAGVA
 551 VLRAYNYVAQ EVDDYHAFQT LTHIYNKVRT GEKVKVEHVV SVLEKKYPYV
 601 EVNSILGIDS AYDRNRKEAR GYYEQTGVGP LPVVLFNGMP FEREQLDPDE
 651 LETITMHKIL ETTTFFQRAV YLGELPHDQD VVEYIMNQPN VVPRINSRIL
 701 TAERDYLDLT ASNNFFVDDY ARFTILDSQG KTAAVANSMN YLTKKGMSSK
 751 EIYDDSFIRP VTFWIVGDFD SPSGRQLLYD AIKHQKSSNN VRISMINNPA
 801 KEISYENTQI SRAIWAALQT QTSNAAKNFI TKMAKEGAAE ALAAGADIAE
 851 FSVGGMDFSL FKEVFESSKM DFILSHAVYC RDVLKLKKGQ RAVISNGRII
 901 GPLEDSELFN QDDFHLLENI ILKTSGQKIK SHIQQLRVEE DVASDLVMKV
 951 DALLSAQPKG DPRIEYQFFE DRHSAIKLRP KEGETYFDVV AVVDPVTREA
1001 QRLAPLLLVL AQLINMNLRV FMNCQSKLSD MPLKSFYRYV LEPEISFTSD
1051 NSFAKGPIAK FLDMPQSPLF TLNLNTPESW MVESVRTPYD LDNIYLEEVD
1101 SVVAAEYELE YLLLEGHCYD ITTGQPPRGL QFTLGTSANP VIVDTIVMAN
```

Matched peptides (bold red): INYVFRHYIFNPR (223–235), VWQLQDLSFQTAAR (320–333), LNIQPSEADYAVDIR (440–454), ILETTTFFQR (659–668), IEYQFFEDR (964–972)

1151 LGYFQLKANP GAWILRLRKG RSEDIYRIYS HDGTDSPPDA DEVVIVLNNF
1201 KSKIIKVKVQ KKADMVNEDL LSDGTSENES GFWDSFKWGF TGQKTEEVKQ
1251 DKDDIINIFS VASGHLYERF LRIMMLSVLK NTKTPVKFWF LKNYLSPTFK
1301 EFIPYMANEY NFQYELVQYK WPRWLHQQTE KQRIIWGYKI LFLDVLFPLV
1351 VDK**FLFVDAD QIVR**TDLKEL R**DFNLDGAPY GYTPFCDSR**R EMDGYRFWKS
1401 GYWASHLAGR KYHISALYVV DLKKFRKIAA GDRLRGQYQG LSQDPNSLSN
1451 LDQDLPNNMI HQVPIKSLPQ EWLWCETWCD DASKKRAKTI DLCNNPMTKE
1501 PKLEAAVRIV PEWQDYDQEI KQLQIRFQKE KETGALYKEK TKEPSREGPQ
1551 KREEL

Residue Number Increasing Mass Decreasing Mass

Start - End	Observed	Mr(expt)	Mr(calc)	Delta	Miss	Sequence
223 - 228	811.4520	810.4447	810.4388	0.0060	0	**INYVFR** (No match)
229 - 235	946.4884	945.4811	945.4820	-0.0009	0	**HYIFNPR** (No match)
320 - 333	1662.8428	1661.8355	1661.8525	-0.0170	0	**VWQLQDLSFQTAAR** (No match)
440 - 454	1703.8535	1702.8462	1702.8526	-0.0063	0	**LNIQPSEADYAVDIR** (No match)
440 - 454	1703.8535	1702.8462	1702.8526	-0.0063	0	**LNIQPSEADYAVDIR** (Ions score 33)
659 - 668	1255.6647	1254.6574	1254.6608	-0.0034	0	**ILETTTFFQR** (Ions score 0)
659 - 668	1255.6647	1254.6574	1254.6608	-0.0034	0	**ILETTTFFQR** (No match)
964 - 972	1246.5699	1245.5626	1245.5665	-0.0039	0	**IEYQFFEDR** (Ions score 17)
964 - 972	1246.5699	1245.5626	1245.5665	-0.0039	0	**IEYQFFEDR** (No match)
1354 - 1364	1322.6996	1321.6923	1321.7030	-0.0106	0	**FLFVDADQIVR** (No match)
1354 - 1364	1322.6996	1321.6923	1321.7030	-0.0106	0	**FLFVDADQIVR** (No match)
1372 - 1389	2094.8828	2093.8755	2093.8788	-0.0033	0	**DFNLDGAPYGYTPFCDSR** (No match)

Mascot: http://www.matrixscience.com/

Spot 43

Protein View

Match to: **gi|5453832** Score: **488** Expect: **3e-044**
oxygen regulated protein precursor [Homo sapiens]

Nominal mass (M_r): **111494**; Calculated pI value: **5.16**
NCBI BLAST search of gi|5453832 against nr
Unformatted sequence string for pasting into other applications

Taxonomy: Homo sapiens
Links to retrieve other entries containing this sequence from NCBI Entrez:
gi|10720185 from Homo sapiens
gi|1794219 from Homo sapiens
gi|85718184 from Homo sapiens
gi|86611373 from Homo sapiens
gi|157362213 (no taxonomy information for this entry)

Fixed modifications: Carbamidomethyl (C)
Variable modifications: Oxidation (M)
Cleavage by Trypsin: cuts C-term side of KR unless next residue is P
Sequence Coverage: **19%**

Matched peptides shown in **Bold Red**

```
  1 MADKVRRQRP RRRVCWALVA VLLADLLALS DTLAVMSVDL GSESMKVAIV
 51 KPGVPMEIVL NKESRRKTPV IVTLKENERF FGDSAASMAI KNPKATLRYF
101 QHLLGKQADN PHVALYQARF PEHELTFDPQ RQTVHFQISS QLQFSPEEVL
151 GMVLNYSRSL AEDFAEQPIK DAVITVPVFF NQAERRAVLQ AARMAGLKVL
201 QLINDNTATA LSYGVFRRKD INTTAQNIMF YDMGSGSTVC TIVTYQMVKT
251 KEAGMQPQLQ IRGVGFDRTL GGLEMELRLR ERLAGLFNEQ RKGQRAKDVR
301 ENPRAMAKLL REANRLKTVL SANADHMAQI EGLMDDVDFK AKVTRVEFEE
351 LCADLFERVP GPVQQALQSA EMSLDEIEQV ILVGGATRVP RVQEVLLKAV
401 GKEELGKNIN ADEAAAMGAV YQAAALSKAF KVKPFVVRDA VVYPILVEFT
451 REVEEEPGIH SLKHNKRVLF SRMGPYPQRK VITFNRYSHD FNFHINYGDL
501 GFLGPEDLRV FGSQNLTTVK LKGVGDSFKK YPDYESKGIK AHFNLDESGV
551 LSLDRVESVF ETLVEDSAEE ESTLTKLGNT ISSLFGGGTT PDAKENGTDT
601 VQEEEESPAE GSKDEPGEQV ELKEEAEAPV EDGSQPPPPE PKGDATPEGE
651 KATEKENGDK SEAQKPSEKA EAGPEGVAPA PEGEKKQKPA RKRRMVEEIG
701 VELVVLDLPD LPEDKLAQSV QKLQDLTLRD LEKQEREKAA NSLEAFIFET
751 QDKLYQPEYQ EVSTEEQREE ISGKLSAAST WLEDEGVGAT TVMLKEKLAE
801 LRKLCQGLFF RVEERKKWPE RLSALDNLLN HSSMFLKGAR LIPEMDQIFT
851 EVEMTTLEKV INETWAWKNA TLAEQAKLPA TEKPVLLSKD IEAKMMALDR
901 EVQYLLNKAK FTKPRPRPKD KNGTRAEPPL NASASDQGEK VIPPAGQTED
```

951 AEPISEPEKV ETGSEPGDTE PLELGGPGAE PEQKEQSTGQ KRPLKNDEL

Residue Number Increasing Mass Decreasing Mass

Start - End	Observed	Mr(expt)	Mr(calc)	Delta	Miss	Sequence
107 - 119	1482.7484	1481.7411	1481.7375	0.0037	0	**QADNPHVALYQAR** (No match)
120 - 131	1515.7284	1514.7211	1514.7153	0.0058	0	**FPEHELTFDPQR** (No match)
159 - 185	3034.5408	3033.5335	3033.5446	-0.0111	1	**SLAEDFAEQPIKDAVITVPVFFNQAER** (Ions score 126)
159 - 185	3034.5408	3033.5335	3033.5446	-0.0111	1	**SLAEDFAEQPIKDAVITVPVFFNQAER** (No match)
199 - 217	2095.1201	2094.1128	2094.1109	0.0020	0	**VLQLINDNTATALSYGVFR** (No match)
199 - 217	2095.1201	2094.1128	2094.1109	0.0020	0	**VLQLINDNTATALSYGVFR** (Ions score 121)
252 - 262	1286.6556	1285.6483	1285.6448	0.0035	0	**EAGMQPQLQIR** Oxidation (M) (No match)
281 - 291	1332.7061	1331.6988	1331.6945	0.0043	1	**ERLAGLFNEQR** (No match)
283 - 291	1047.5618	1046.5545	1046.5508	0.0037	0	**LAGLFNEQR** (Ions score 6)
283 - 291	1047.5618	1046.5545	1046.5508	0.0037	0	**LAGLFNEQR** (No match)
343 - 358	2012.9756	2011.9683	2011.9672	0.0011	1	**VTRVEFEELCADLFER** (No match)
346 - 358	1656.7623	1655.7550	1655.7500	0.0050	0	**VEFEELCADLFER** (No match)
432 - 438	844.5499	843.5426	843.5330	0.0096	0	**VKPFVVR** (No match)
439 - 451	1521.8344	1520.8271	1520.8238	0.0033	0	**DAVVYPILVEFTR** (Ions score 59)
439 - 451	1521.8344	1520.8271	1520.8238	0.0033	0	**DAVVYPILVEFTR** (No match)
487 - 509	2726.2617	2725.2544	2725.2560	-0.0016	0	**YSHDFNFHINYGDLGFLGPEDLR** (No match)
541 - 555	1672.8325	1671.8252	1671.8216	0.0036	0	**AHFNLDESGVLSLDR** (No match)
754 - 768	1898.8812	1897.8739	1897.8693	0.0046	0	**LYQPEYQEVSTEEQR** (No match)
754 - 774	2542.1946	2541.1873	2541.1870	0.0003	1	**LYQPEYQEVSTEEQREEISGK** (No match)
803 - 811	1168.6364	1167.6291	1167.6222	0.0069	1	**KLCQGLFFR** (No match)
804 - 811	1040.5422	1039.5349	1039.5273	0.0077	0	**LCQGLFFR** (No match)

Mascot: http://www.matrixscience.com/

Spot 44

Protein View

Match to: **gi|5453832** Score: **257** Expect: **3.8e-021**
oxygen regulated protein precursor [Homo sapiens]

Nominal mass (M_r): **111494**; Calculated pI value: **5.16**
NCBI BLAST search of gi|5453832 against nr
Unformatted sequence string for pasting into other applications

Taxonomy: Homo sapiens
Links to retrieve other entries containing this sequence from NCBI Entrez:
gi|10720185 from Homo sapiens
gi|1794219 from Homo sapiens
gi|85718184 from Homo sapiens
gi|86611373 from Homo sapiens
gi|157362213 (no taxonomy information for this entry)

Fixed modifications: Carbamidomethyl (C)
Variable modifications: Oxidation (M)
Cleavage by Trypsin: cuts C-term side of KR unless next residue is P
Sequence Coverage: **12%**

Matched peptides shown in **Bold Red**

```
  1 MADKVRRQRP RRRVCWALVA VLLADLLALS DTLAVMSVDL GSESMKVAIV
 51 KPGVPMEIVL NKESRRKTPV IVTLKENERF FGDSAASMAI KNPKATLRYF
101 QHLLGKQADN PHVALYQARF PEHELTFDPQ RQTVHFQISS QLQFSPEEVL
151 GMVLNYSRSL AEDFAEQPIK DAVITVPVFF NQAERRAVLQ AARMAGLKVL
201 QLINDNTATA LSYGVFRRKD INTTAQNIMF YDMGSGSTVC TIVTYQMVKT
251 KEAGMQPQLQ IRGVGFDRTL GGLEMELRLR ERLAGLFNEQ RKGQRAKDVR
301 ENPRAMAKLL REANRLKTVL SANADHMAQI EGLMDDVDFK AKVTRVEFEE
351 LCADLFERVP GPVQQALQSA EMSLDEIEQV ILVGGATRVP RVQEVLLKAV
401 GKEELGKNIN ADEAAAMGAV YQAAALSKAF KVKPFVVRDA VVYPILVEFT
451 REVEEEPGIH SLKHNKRVLF SRMGPYPQRK VITFNRYSHD FNFHINYGDL
501 GFLGPEDLRV FGSQNLTTVK LKGVGDSFKK YPDYESKGIK AHFNLDESGV
551 LSLDRVESVF ETLVEDSAEE ESTLTKLGNT ISSLFGGGTT PDAKENGTDT
601 VQEEEESPAE GSKDEPGEQV ELKEEAEAPV EDGSQPPPPE PKGDATPEGE
651 KATEKENGDK SEAQKPSEKA EAGPEGVAPA PEGEKKQKPA RKRRMVEEIG
701 VELVVLDLPD LPEDKLAQSV QKLQDLTLRD LEKQEREKAA NSLEAFIFET
751 QDKLYQPEYQ EVSTEEQREE ISGKLSAAST WLEDEGVGAT TVMLKEKLAE
801 LRKLCQGLFF RVEERKKWPE RLSALDNLLN HSSMFLKGAR LIPEMDQIFT
851 EVEMTTLEKV INETWAWKNA TLAEQAKLPA TEKPVLLSKD IEAKMMALDR
901 EVQYLLNKAK FTKPRPRPKD KNGTRAEPPL NASASDQGEK VIPPAGQTED
```

951 AEPISEPEKV ETGSEPGDTE PLELGGPGAE PEQKEQSTGQ KRPLKNDEL

Residue Number Increasing Mass Decreasing Mass

Start - End	Observed	Mr(expt)	Mr(calc)	Delta	Miss	Sequence
95 - 106	1446.8250	1445.8177	1445.8143	0.0035	1	**ATLRYFQHLLGK** (No match)
99 - 106	1005.5578	1004.5505	1004.5443	0.0062	0	**YFQHLLGK** (No match)
107 - 119	1482.7455	1481.7382	1481.7375	0.0008	0	**QADNPHVALYQAR** (No match)
107 - 119	1482.7455	1481.7382	1481.7375	0.0008	0	**QADNPHVALYQAR** (Ions score 58)
120 - 131	1515.7311	1514.7238	1514.7153	0.0085	0	**FPEHELTFDPQR** (No match)
120 - 131	1515.7311	1514.7238	1514.7153	0.0085	0	**FPEHELTFDPQR** (Ions score 15)
199 - 217	2095.0986	2094.0913	2094.1109	-0.0195	0	**VLQLINDNTATALSYGVFR** (Ions score 86)
199 - 217	2095.0986	2094.0913	2094.1109	-0.0195	0	**VLQLINDNTATALSYGVFR** (No match)
252 - 262	1286.6556	1285.6483	1285.6448	0.0035	0	**EAGMQPQLQIR** Oxidation (M) (No match)
252 - 268	1901.9633	1900.9560	1900.9577	-0.0017	1	**EAGMQPQLQIRGVGFDR** (No match)
281 - 291	1332.7069	1331.6996	1331.6945	0.0051	1	**ERLAGLFNEQR** (No match)
283 - 291	1047.5721	1046.5648	1046.5508	0.0140	0	**LAGLFNEQR** (No match)
283 - 291	1047.5721	1046.5648	1046.5508	0.0140	0	**LAGLFNEQR** (Ions score 2)
439 - 451	1521.8325	1520.8252	1520.8238	0.0014	0	**DAVVYPILVEFTR** (No match)
473 - 480	992.5054	991.4981	991.4909	0.0072	1	**MGPYPQRK** Oxidation (M) (No match)
803 - 811	1168.6464	1167.6391	1167.6222	0.0169	1	**KLCQGLFFR** (No match)
911 - 919	1126.7017	1125.6944	1125.6770	0.0174	0	**FTKPRPRPK** (No match)

Spot 45

Protein View

Match to: **gi|119582323** Score: **85** Expect: **0.00061**
diaphanous homolog 1 (Drosophila), isoform CRA_a [Homo sapiens]

Nominal mass (M_r): **139473**; Calculated pI value: **5.28**
NCBI BLAST search of gi|119582323 against nr
Unformatted sequence string for pasting into other applications

Taxonomy: Homo sapiens

Fixed modifications: Carbamidomethyl (C)
Variable modifications: Oxidation (M)
Cleavage by Trypsin: cuts C-term side of KR unless next residue is P
Sequence Coverage: **6%**

Matched peptides shown in **Bold Red**

1 MEPPGGSLGP GRGTRDKKKG RSPDELPSAG GDGGKSKKFL ERFTSMRIKK
51 EKEKPNSAHR NSSASYGDDP TAQSLQDVSD EQVLVLFEQM LLDMNLNEEK
101 QQPLREKDII IKREMVSQYL YTSKAGMSQK ESSKSAMMYI QELRSGLRDM
151 PLLSCLESLR VSLNNNPVSW VQTFGAEGLA SLLDILKR**LH DEKEETAGSY**
201 **DSR**NKHEIIR CLKAFMNNKF GIKTMLETEE GILLLVRAMD PAVPNMMIDA
251 AKLLSALCIL PQPEDMNERV LEAMTERAEM DEVERFQPLL DGLKSGTTIA
301 LKVGCLQLIN ALITPAEELD FRVHIRSELM R**LGLHQVLQD LR**EIENEDMR
351 VQLNVFDEQG EEDSYDLKGR LDDIRMEMDD FNEVFQILLN TVKDSKAEPH
401 FLSILQHLLL VR**NDYEARPQ YYK**LIEECIS QIVLHKNGAD PDFK**CRHLQI**
451 **EIEGLIDQMI DK**TKVEKSEA KAAELEKKLD SELTARHELQ VEMKKMESDF
501 EQKLQDLQGE KDALHSEKQQ IATEKQDLEA EVSQLTGEVA KLTKELEDAK
551 KEMASLSAAA ITVPPSVPSR APVPPAPPLP GDSGTIIPPP PAPGDSTTPP
601 PPPPPPPPPP PLPGGTAISP PPPLSGDATI PPPPPLPEGV GIPSPSSLPG
651 GTAIPPPPPL PGSARIPPPP PPLPGSAGIP PPPPPLPGEA GMPPPPPPLP
701 GGPGIPPPPP FPGGPGIPPP PPGMGMPPPP PFGFGVPAAP VLPFGLTPK**K**
751 **LYKPEVQLR**R PNWSKLVAED LSQDCFWTKV KEDRFENNEL FAKLTLTFSA
801 QTKTKKDQEG GEEKKSVQKK KVKELKVLDS K**TAQNLSIFL GSFR**MPYQEI
851 KNVILEVNEA VLTESMIQNL IKQMPEPEQL KMLSELKDEY DDLAESEQFG
901 VVMGTVPRLR PRLNAILFKL QFSEQVENIK PEIVSVTAAC EELRKSESFS
951 NLLEITLLVG NYMNAGSRNA GAFGFNISFL CKLRDTKSTD QKMTLLHFLA
1001 ELCENDYPDV LKFPDELAHV EKASRVSAEN LQKNLDQMKK QISDVERDVQ
1051 NFPAATDEKD KFVEKMTIFV KDAQEQYNKL RMMHSNMETL YKELGEYFLF
1101 DPKKLSVEEF FMDLHNFRNM FLQAVKENQK RRETEEKMRR AKLAKEKAEK
1151 ERLEKQQKRE QLIDMNAEGD ETGVMDSLLE ALQSGAAFRR KRGPRQANRK
1201 AGCAVTSLLA SELTKDDAMA AVPAKVSKNS ETFPTILEEA KELVGRAS

Residue Number Increasing Mass Decreasing Mass

Start - End	Observed	Mr(expt)	Mr(calc)	Delta	Miss	Sequence
189 - 203	1736.7361	1735.7288	1735.7649	-0.0360	1	LHDEKEETAGSYDSR (No match)
189 - 203	1736.7361	1735.7288	1735.7649	-0.0360	1	LHDEKEETAGSYDSR (Ions score 9)
332 - 342	1291.7185	1290.7112	1290.7408	-0.0295	0	LGLHQVLQDLR (No match)
332 - 342	1291.7185	1290.7112	1290.7408	-0.0295	0	LGLHQVLQDLR (Ions score 19)
413 - 423	1446.6361	1445.6288	1445.6575	-0.0287	0	NDYEARPQYYK (No match)
445 - 462	2211.0850	2210.0777	2210.1187	-0.0410	1	CRHLQIEIEGLIDQMIDK (No match)
750 - 759	1273.7345	1272.7272	1272.7553	-0.0281	1	KLYKPEVQLR (No match)
832 - 844	1453.7484	1452.7411	1452.7724	-0.0313	0	TAQNLSIFLGSFR (Ions score 7)
832 - 844	1453.7484	1452.7411	1452.7724	-0.0313	0	TAQNLSIFLGSFR (No match)

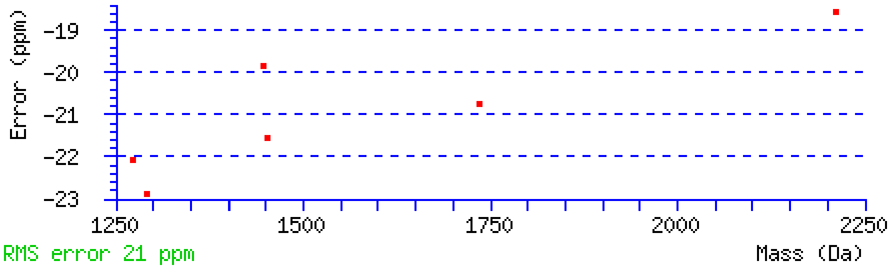

Mascot: http://www.matrixscience.com/

Spot 47

Protein View

Match to: **gi|5453832** Score: **537** Expect: **3.8e-049**
oxygen regulated protein precursor [Homo sapiens]

Nominal mass (M_r): **111494**; Calculated pI value: **5.16**
NCBI BLAST search of gi|5453832 against nr
Unformatted sequence string for pasting into other applications

Taxonomy: Homo sapiens
Links to retrieve other entries containing this sequence from NCBI Entrez:
gi|10720185 from Homo sapiens
gi|1794219 from Homo sapiens
gi|85718184 from Homo sapiens
gi|86611373 from Homo sapiens
gi|157362213 (no taxonomy information for this entry)

Fixed modifications: Carbamidomethyl (C)
Variable modifications: Oxidation (M)
Cleavage by Trypsin: cuts C-term side of KR unless next residue is P
Sequence Coverage: **35%**

Matched peptides shown in **Bold Red**

1 MADKVRRQRP RRRVCWALVA VLLADLLALS DTLAVMSVDL GSESMK**VAIV**
51 **KPGVPMEIVL NK**ESRRKTPV IVTLKENER**F FGDSAASMAI KNPK**ATLR**YF**
101 **QHLLGKQADN PHVALYQARF PEHELTFDPQ R**QTVHFQISS QLQFSPEEVL
151 GMVLNYSR**SL AEDFAEQPIK DAVITVPVFF NQAER**RAVLQ AARMAGLK**VL**
201 **QLINDNTATA LSYGVFR**RKD INTTAQNIMF YDMGSGSTVC TIVTYQMVKT
251 K**EAGMQPQLQ IRGVGFDRTL GGLEMELR**LR **ERLAGLFNEQ RK**GQRAKDVR
301 ENPRAMAKLL REANRLKTVL SANADHMAQI EGLMDDVDFK AK**VTRVEFEE**
351 **LCADLFER**VP GPVQQALQSA EMSLDEIEQV ILVGGATRVP RVQEVLLKAV
401 GKEELGKNIN ADEAAAMGAV YQAAALSKAF K**VKPFVVRDA VVYPILVEFT**
451 **REVEEEPGIH SLK**HNKRVLF SR**MGPYPQRK** VITFNR**YSHD FNFHINYGDL**
501 **GFLGPEDLR**V FGSQNLTTVK LKGVGDSFKK YPDYESKGIK **AHFNLDESGV**
551 **LSLDR**VESVF ETLVEDSAEE ESTLTK**LGNT ISSLFGGGTT PDAK**ENGTDT
601 VQEEEESPAE GSKDEPGEQV ELKEEAEAPV EDGSQPPPPE PKGDATPEGE
651 KATEKENGDK SEAQKPSEKA EAGPEGVAPA PEGEKKQKPA RKRRMVEEIG
701 VELVVLDLPD LPEDKLAQSV QK**LQDLTLRD LEKQER**EK**AA NSLEAFIFET**
751 **QDKLYQPEYQ EVSTEEQREE ISGK**LSAAST WLEDEGVGAT TVMLK**EKLAE**
801 **LRKLCQGLFF RVEER**KKWPE RLSALDNLLN HSSMFLKGAR LIPEMDQIFT
851 EVEMTTLEKV INETWAWKNA TLAEQAKLPA TEKPVLLSKD IEAK**MMALDR**
901 **EVQYLLNK**AK FTKPRPRPKD KNGTRAEPPL NASASDQGEK VIPPAGQTED

951 AEPISEPEKV ETGSEPGDTE PLELGGPGAE PEQKEQSTGQ K**RPLKNDEL**

Residue Number Increasing Mass Decreasing Mass

Start - End	Observed	Mr(expt)	Mr(calc)	Delta	Miss	Sequence
47 - 62	1723.0077	1722.0004	1722.0113	-0.0108	0	**VAIVKPGVPMEIVLNK** Oxidation (M) (No match)
80 - 94	1599.7662	1598.7589	1598.7762	-0.0173	1	**FFGDSAASMAIKNPK** Oxidation (M) (No match)
99 - 106	1005.5391	1004.5318	1004.5443	-0.0125	0	**YFQHLLGK** (No match)
107 - 119	1482.7300	1481.7227	1481.7375	-0.0147	0	**QADNPHVALYQAR** (No match)
107 - 119	1482.7300	1481.7227	1481.7375	-0.0147	0	**QADNPHVALYQAR** (Ions score 88)
120 - 131	1515.7124	1514.7051	1514.7153	-0.0102	0	**FPEHELTFDPQR** (No match)
159 - 185	3034.5227	3033.5154	3033.5446	-0.0292	1	**SLAEDFAEQPIKDAVITVPVFFNQAER** (No match)
199 - 217	2095.0911	2094.0838	2094.1109	-0.0270	0	**VLQLINDNTATALSYGVFR** (No match)
199 - 217	2095.0911	2094.0838	2094.1109	-0.0270	0	**VLQLINDNTATALSYGVFR** (Ions score 103)
252 - 262	1286.6422	1285.6349	1285.6448	-0.0099	0	**EAGMQPQLQIR** Oxidation (M) (No match)
252 - 268	1901.9548	1900.9475	1900.9577	-0.0102	1	**EAGMQPQLQIRGVGFDR** (No match)
269 - 278	1134.5712	1133.5639	1133.5750	-0.0111	0	**TLGGLEMELR** Oxidation (M) (No match)
281 - 291	1332.6918	1331.6845	1331.6945	-0.0100	1	**ERLAGLFNEQR** (No match)
283 - 291	1047.5520	1046.5447	1046.5508	-0.0061	0	**LAGLFNEQR** (Ions score 13)
283 - 291	1047.5520	1046.5447	1046.5508	-0.0061	0	**LAGLFNEQR** (No match)
283 - 292	1175.6472	1174.6399	1174.6458	-0.0059	1	**LAGLFNEQRK** (No match)
343 - 358	2012.9565	2011.9492	2011.9672	-0.0180	1	**VTRVEFEELCADLFER** (No match)
346 - 358	1656.7433	1655.7360	1655.7500	-0.0140	0	**VEFEELCADLFER** (No match)
432 - 438	844.5358	843.5285	843.5330	-0.0045	0	**VKPFVVR** (No match)
439 - 451	1521.8163	1520.8090	1520.8238	-0.0148	0	**DAVVYPILVEFTR** (Ions score 39)
439 - 451	1521.8163	1520.8090	1520.8238	-0.0148	0	**DAVVYPILVEFTR** (No match)
452 - 463	1366.6760	1365.6687	1365.6775	-0.0088	0	**EVEEEPGIHSLK** (No match)
473 - 480	992.4882	991.4809	991.4909	-0.0100	1	**MGPYPQRK** Oxidation (M) (No match)
487 - 509	2726.2261	2725.2188	2725.2560	-0.0372	0	**YSHDFNFHINYGDLGFLGPEDLR** (No match)
541 - 555	1672.8116	1671.8043	1671.8216	-0.0173	0	**AHFNLDESGVLSLDR** (No match)
577 - 594	1735.8717	1734.8644	1734.8788	-0.0143	0	**LGNTISSLFGGGTTPDAK** (No match)
723 - 733	1343.7416	1342.7343	1342.7456	-0.0113	1	**LQDLTLRDLEK** (No match)
730 - 736	917.4630	916.4557	916.4614	-0.0056	1	**DLEKQER** (No match)
739 - 753	1683.8221	1682.8148	1682.8151	-0.0003	0	**AANSLEAFIFETQDK** (No match)
754 - 768	1898.8549	1897.8476	1897.8693	-0.0217	0	**LYQPEYQEVSTEEQR** (No match)
754 - 774	2542.1553	2541.1480	2541.1870	-0.0390	1	**LYQPEYQEVSTEEQREEISGK** (No match)
796 - 802	858.5043	857.4970	857.4970	0.0000	1	**EKLAELR** (No match)
803 - 811	1168.6228	1167.6155	1167.6222	-0.0067	1	**KLCQGLFFR** (No match)
804 - 811	1040.5275	1039.5202	1039.5273	-0.0070	0	**LCQGLFFR** (No match)
804 - 815	1553.7776	1552.7703	1552.7820	-0.0116	1	**LCQGLFFRVEER** (No match)
895 - 908	1724.0029	1722.9956	1722.8797	0.1160	1	**MMALDREVQYLLNK** (No match)
895 - 908	1755.8723	1754.8650	1754.8695	-0.0044	1	**MMALDREVQYLLNK** 2 Oxidation (M) (No match)
901 - 908	1006.5408	1005.5335	1005.5494	-0.0159	0	**EVQYLLNK** (No match)
992 - 999	984.5438	983.5365	983.5399	-0.0034	1	**RPLKNDEL** (No match)

Spot 48

Protein View

Match to: **gi|47834348** Score: **105** Expect: **6.1e-006**
minor histocompatibility antigen HA-1 [Homo sapiens]

Nominal mass (M_r): **125848**; Calculated pI value: **5.76**
NCBI BLAST search of gi|47834348 against nr
Unformatted sequence string for pasting into other applications

Taxonomy: Homo sapiens
Links to retrieve other entries containing this sequence from NCBI Entrez:
gi|22725157 from Homo sapiens
gi|119589957 from Homo sapiens

Fixed modifications: Carbamidomethyl (C)
Variable modifications: Oxidation (M)
Cleavage by Trypsin: cuts C-term side of KR unless next residue is P
Sequence Coverage: **3%**

Matched peptides shown in **Bold Red**

```
   1 MFSRKKRELM KTPSISKKNR AGSPSPQPSG ELPRKDGADA VFPGPSLEPP
  51 AGSSGVKATG TLKRPTSLSR HASAAGFPLS GAASWTLGRS HRSPLTAASP
 101 GELPTEGAGP DVVEDISHLL ADVARFAEGL EKLKECVLRD DLLEARRPRA
 151 HECLGEALRV MHQIISKYPL LNTVETLTAA GTLIAKVKAF HYESNNDLEK
 201 QEFEKALETI AVAFSSTVSE FLMGEVDSST LLAVPPGDSS QSMESLYGPG
 251 SEGTPPSLED CDAGCLPAEE VDVLLQRCEG GVDAALLYAK NMAKYMKDLI
 301 SYLEKRTTLE MEFAKGLQKI AHNCRQSVMQ EPHMPLLSIY SLALEQDLEF
 351 GHSMVQAVGT LQTQTFMQPL TLRRLEHEKR RKEIKEAWHR AQRKLQEAES
 401 NLRKAKQGYV QRCEDHDKAR FLVAKAEEEQ AGSAPGAGST ATKTLDKRRR
 451 LEEEAKNKAE EAMATYRTCV ADAKTQKQEL EDTKVTALRQ IQEVIRQSDQ
 501 TIKSATISYY QMMHMQTAPL PVHFQMLCES SKLYDPGQQY ASHVRQLQRD
 551 QEPDVHYDFE PHVSANAWSP VMRARKSSFN VSDVARPEAA GSPPEEGGCT
 601 EGTPAKDHRA GRGHQVHKSW PLSISDSDSG LDPGPGAGDF KKFERTSSSG
 651 TMSSTEELVD PDGGAGASAF EQADLNGMTP ELPVAVPSGP FRHEGLSKAA
 701 RTHRLRKLRT PAKCRECNSY VYFQGAECEE CCLACHKKCL ETLAIQCGHK
 751 KLQGRLQLFG QDFSHAARSA PDGVPFIVKK CVCEIERRAL RTKGIYRVNG
 801 VKTRVEKLCQ AFENGKELVE LSQASPHDIS NVLKLYLRQL PEPLISFRLY
 851 HELVGLAKDS LKAEAEAKAA SRGRQDGSES EAVAVALAGR LRELLRDLPP
 901 ENRASLQYLL RHLRRIVEVE QDNKMTPGNL GIVFGPTLLR PRPTEATVSL
 951 SSLVDYPHQA RVIETLIVHY GLVFEEEPEE TPGGQDESSN QRAEVVVQVP
1001 YLEAGEAVVY PLQEAAADGC RESRVVSNDS DSDLEEASEL LSSSEASALG
1051 HLSFLEQQQS EASLEVASGS HSGSEEQLEA TAREDGDGDE DGPAQQLSGF
```

1101 NTNQSNNVLQ APLPPMRLRG GRMTLGSCRE RQPEFV

Residue Number Increasing Mass Decreasing Mass

Start - End	Observed	Mr(expt)	Mr(calc)	Delta	Miss	Sequence
533 - 545	1533.7351	1532.7278	1532.7371	-0.0093	0	LYDPGQQYASHVR (No match)
533 - 545	1533.7351	1532.7278	1532.7371	-0.0093	0	LYDPGQQYASHVR (Ions score 14)
756 - 768	1489.7471	1488.7398	1488.7473	-0.0075	0	LQLFGQDFSHAAR (No match)
756 - 768	1489.7471	1488.7398	1488.7473	-0.0075	0	LQLFGQDFSHAAR (Ions score 34)
839 - 848	1199.6760	1198.6687	1198.6710	-0.0022	0	QLPEPLISFR (No match)
839 - 848	1199.6760	1198.6687	1198.6710	-0.0022	0	QLPEPLISFR (Ions score 47)

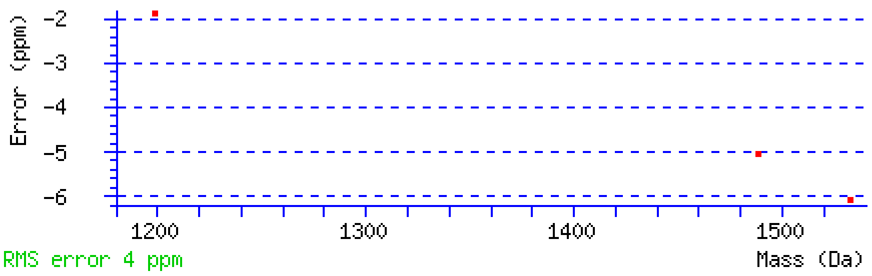

Mascot: http://www.matrixscience.com/

Spot 49

Protein View

Match to: **gi|1002923** Score: **274** Expect: **7.7e-023**
coronin-like protein

Nominal mass (M_r): **51722;** Calculated pI value: **6.12**
NCBI BLAST search of gi|1002923 against nr
Unformatted sequence string for pasting into other applications

Taxonomy: Homo sapiens

Fixed modifications: Carbamidomethyl (C)
Variable modifications: Oxidation (M)
Cleavage by Trypsin: cuts C-term side of KR unless next residue is P
Sequence Coverage: **22%**

Matched peptides shown in **Bold Red**

```
  1 MSRQVVRTSK FRHVFGQPAK ADQCYEDVRV SQTTWDSGFC AVNPKFVALI
 51 CEASGGGAFL VLPLGKTGRV DKNAPTVCGH TAPVLDIAWC PHNDNVIASG
101 SEDCTVMVWE IPDGGLMLPL REPVVTLEGH TKRVGIVAWH TTAQNVLLSA
151 GCDNVIMVWD VGTGAAMLTL GPEVHPDTIY SVDWSRDGGL ICTSCRDKRV
201 RIIEPRKGTV VAEKDRPHEG TRPVRAVFVS EGKILTTGFS RMSEWQVALW
251 DTKHLEEPLS LQELDTSSGV LLPFFDPDTN IVYLCGKGDS SIRYFEITSE
301 APFLHYLSMF SSKESQRGMG YMPKRGLEVN KCEIARFYKL HERRCEPIAM
351 TVPRKSDLFQ EDLYPPTAGP DPALTAEEWL GGRDAGPLLI SLKDGYVPPK
401 SRELRVNRGL DTGRRRAAPE ASGTPSSDAV SRLEEEMRKL QATVQELQKR
451 LDRLEETVQA K
```

Residue Number Increasing Mass Decreasing Mass

Start - End	Observed	Mr(expt)	Mr(calc)	Delta	Miss	Sequence
11 - 20	1186.6324	1185.6251	1185.6406	-0.0155	1	FRHVFGQPAK (No match)
21 - 29	1155.4594	1154.4521	1154.4662	-0.0141	0	ADQCYEDVR (Ions score 4)
21 - 29	1155.4594	1154.4521	1154.4662	-0.0141	0	ADQCYEDVR (No match)
30 - 45	1796.8058	1795.7985	1795.8199	-0.0214	0	VSQTTWDSGFCAVNPK (No match)
30 - 45	1796.8058	1795.7985	1795.8199	-0.0214	0	VSQTTWDSGFCAVNPK (Ions score 113)
187 - 196	1138.4857	1137.4784	1137.4906	-0.0122	0	DGGLICTSCR (No match)
187 - 196	1138.4857	1137.4784	1137.4906	-0.0122	0	DGGLICTSCR (Ions score 7)

215 - 225	1319.6725	1318.6652	1318.6854	-0.0202	0	**DRPHEGTRPVR** (Ions score 35)
215 - 225	1319.6725	1318.6652	1318.6854	-0.0202	0	**DRPHEGTRPVR** (No match)
234 - 241	894.4963	893.4890	893.4970	-0.0080	0	**ILTTGFSR** (No match)
344 - 354	1329.6503	1328.6430	1328.6693	-0.0263	1	**RCEPIAMTVPR** (No match)
344 - 354	1345.6523	1344.6450	1344.6642	-0.0192	1	**RCEPIAMTVPR** Oxidation (M) (No match)
344 - 354	1345.6523	1344.6450	1344.6642	-0.0192	1	**RCEPIAMTVPR** Oxidation (M) (No match)
345 - 354	1189.5577	1188.5504	1188.5631	-0.0127	0	**CEPIAMTVPR** Oxidation (M) (No match)
355 - 383	3173.5298	3172.5225	3172.5352	-0.0127	1	**KSDLFQEDLYPPTAGPDPALTAEEWLGGR** (No match)

Mascot: http://www.matrixscience.com/

Spot 52

Protein View

Match to: **gi|119625804** Score: **642** Expect: **1.2e-059**
moesin, isoform CRA_b [Homo sapiens]

Nominal mass (M_r): **66678;** Calculated pI value: **5.90**
NCBI BLAST search of gi|119625804 against nr
Unformatted sequence string for pasting into other applications

Taxonomy: Homo sapiens

Fixed modifications: Carbamidomethyl (C)
Variable modifications: Oxidation (M)
Cleavage by Trypsin: cuts C-term side of KR unless next residue is P
Sequence Coverage: **50%**

Matched peptides shown in **Bold Red**

```
  1 MDAELEFAIQ PNTTGKQLFD QVVKTIGLRE VWFFGLQYQD TKGFSTWLKL
 51 NKKVTAQDVR KESPLLFKFR AKFYPEDVSE ELIQDITQRL FFLQVKEGIL
101 NDDIYCPPET AVLLASYAVQ SKYGDFNKEV HKSGYLAGDK LLPQRVLEQH
151 KLNKDQWEER IQVWHEEHRG MLREDAVLEY LKIAQDLEMY GVNYFSIKNK
201 KGSELWLGVD ALGLNIYEQN DRLTPKIGFP WSEIRNISFN DKKFVIKPID
251 KKAPDFVFYA PRLRINKRIL ALCMGNHELY MRRRKPDTIE VQQMKAQARE
301 EKHQKQMERA MLENEKKKRE MAEKEKEKIE REKEELMERL KQIEEQTKKA
351 QQELEEQTRR ALELEQERKR AQSEAEKLAK ERQEAEEAKE ALLQASRDQK
401 KTQEQLALEM AELTARISQL EMARQKKESE AVEWQQKAQM VQEDLEKTRA
451 ELKTAMSTPH VAEPAENEQD EQDENGAEAS ADLRADAMAK DRSEEERTTE
501 AEKNERVQKH LKALTSELAN ARDESKKTAN DMIHAENMRL GRDKYKTLRQ
551 IRQGNTKQRI DEFESM
```

Residue Number Increasing Mass Decreasing Mass

Start - End	Observed	Mr(expt)	Mr(calc)	Delta	Miss	Sequence	
17 - 24	976.5308	975.5235	975.5389	-0.0154	0	QLFDQVVK	(No match)
54 - 60	788.4257	787.4184	787.4188	-0.0004	0	VTAQDVR	(No match)
54 - 61	916.5217	915.5144	915.5137	0.0007	1	VTAQDVRK	(No match)
71 - 89	2281.1101	2280.1028	2280.1273	-0.0245	1	AKFYPEDVSEELIQDITQR	(No match)
71 - 89	2281.1101	2280.1028	2280.1273	-0.0245	1	AKFYPEDVSEELIQDITQR	(Ions score 128)

73 - 89	2081.9839	2080.9766	2080.9953	-0.0186	0	**FYPEDVSEELIQDITQR** (No match)
90 - 96	894.5326	893.5253	893.5374	-0.0121	0	**LFFLQVK** (No match)
123 - 132	1236.5576	1235.5503	1235.5934	-0.0431	1	**YGDFNKEVHK** (No match)
133 - 145	1417.7678	1416.7605	1416.7724	-0.0119	1	**SGYLAGDKLLPQR** (Ions score 47)
133 - 145	1417.7678	1416.7605	1416.7724	-0.0119	1	**SGYLAGDKLLPQR** (No match)
146 - 154	1108.5725	1107.5652	1107.6400	-0.0748	1	**VLEQHKLNK** (No match)
161 - 169	1233.5962	1232.5889	1232.6050	-0.0161	0	**IQVWHEEHR** (No match)
170 - 182	1552.7897	1551.7824	1551.7966	-0.0142	1	**GMLREDAVLEYLK** Oxidation (M) (No match)
183 - 198	1906.9393	1905.9320	1905.9182	0.0139	0	**IAQDLEMYGVNYFSIK** Oxidation (M) (No match)
227 - 235	1104.5750	1103.5677	1103.5763	-0.0086	0	**IGFPWSEIR** (Ions score 39)
227 - 235	1104.5750	1103.5677	1103.5763	-0.0086	0	**IGFPWSEIR** (No match)
236 - 243	965.4927	964.4854	964.4977	-0.0123	1	**NISFNDKK** (No match)
252 - 262	1310.6801	1309.6728	1309.6818	-0.0090	1	**KAPDFVFYAPR** (Ions score 71)
252 - 262	1310.6801	1309.6728	1309.6818	-0.0090	1	**KAPDFVFYAPR** (No match)
253 - 262	1182.5859	1181.5786	1181.5869	-0.0083	0	**APDFVFYAPR** (Ions score 73)
253 - 262	1182.5859	1181.5786	1181.5869	-0.0083	0	**APDFVFYAPR** (No match)
269 - 282	1736.8115	1735.8042	1735.8208	-0.0165	0	**ILALCMGNHELYMR** Oxidation (M) (No match)
269 - 282	1752.8142	1751.8069	1751.8157	-0.0087	0	**ILALCMGNHELYMR** 2 Oxidation (M) (No match)
284 - 295	1488.7711	1487.7638	1487.7766	-0.0127	1	**RKPDTIEVQQMK** Oxidation (M) (No match)
332 - 339	1063.5072	1062.4999	1062.5015	-0.0016	1	**EKEELMER** (No match)
332 - 339	1079.4945	1078.4872	1078.4964	-0.0092	1	**EKEELMER** Oxidation (M) (No match)
350 - 359	1231.5829	1230.5756	1230.5840	-0.0084	0	**AQQELEEQTR** (No match)
350 - 360	1387.6813	1386.6740	1386.6851	-0.0111	1	**AQQELEEQTRR** (No match)
361 - 368	987.5059	986.4986	986.5032	-0.0046	0	**ALELEQER** (No match)
383 - 397	1672.8345	1671.8272	1671.8427	-0.0155	1	**QEAEEAKEALLQASR** (No match)
401 - 416	1831.9413	1830.9340	1830.9509	-0.0169	1	**KTQEQLALEMAELTAR** (No match)
401 - 416	1847.9420	1846.9347	1846.9458	-0.0111	1	**KTQEQLALEMAELTAR** Oxidation (M) (No match)
402 - 416	1703.8525	1702.8452	1702.8559	-0.0107	0	**TQEQLALEMAELTAR** (No match)
402 - 416	1719.8470	1718.8397	1718.8508	-0.0111	0	**TQEQLALEMAELTAR** Oxidation (M) (No match)
417 - 424	947.4966	946.4893	946.4906	-0.0012	0	**ISQLEMAR** (No match)
417 - 424	963.4842	962.4769	962.4855	-0.0086	0	**ISQLEMAR** Oxidation (M) (No match)
438 - 449	1463.7047	1462.6974	1462.7085	-0.0111	1	**AQMVQEDLEKTR** Oxidation (M) (No match)
491 - 497	920.4006	919.3933	919.3995	-0.0062	1	**DRSEEER** (No match)
498 - 506	1077.5123	1076.5050	1076.5098	-0.0047	1	**TTEAEKNER** (No match)
513 - 522	1045.5557	1044.5484	1044.5563	-0.0079	0	**ALTSELANAR** (No match)
513 - 526	1504.7566	1503.7493	1503.7528	-0.0035	1	**ALTSELANARDESK** (No match)
558 - 566	1170.4969	1169.4896	1169.5022	-0.0126	1	**QRIDEFESM** Oxidation (M) (No match)

Spot 58

Protein View

Match to: **gi|119625804** Score: **211** Expect: **1.5e-016**
moesin, isoform CRA_b [Homo sapiens]

Nominal mass (M_r): **66678**; Calculated pI value: **5.90**
NCBI BLAST search of gi|119625804 against nr
Unformatted sequence string for pasting into other applications

Taxonomy: Homo sapiens

Fixed modifications: Carbamidomethyl (C)
Variable modifications: Oxidation (M)
Cleavage by Trypsin: cuts C-term side of KR unless next residue is P
Sequence Coverage: **29%**

Matched peptides shown in **Bold Red**

```
  1 MDAELEFAIQ PNTTGKQLFD QVVKTIGLRE VWFFGLQYQD TKGFSTWLKL
 51 NKKVTAQDVR KESPLLFKFR AKFYPEDVSE ELIQDITQRL FFLQVKEGIL
101 NDDIYCPPET AVLLASYAVQ SKYGDFNKEV HKSGYLAGDK LLPQRVLEQH
151 KLNKDQWEER IQVWHEEHRG MLREDAVLEY LKIAQDLEMY GVNYFSIKNK
201 KGSELWLGVD ALGLNIYEQN DRLTPKIGFP WSEIRNISFN DKKFVIKPID
251 KKAPDFVFYA PRLRINKRIL ALCMGNHELY MRRRKPDTIE VQQMKAQARE
301 EKHQKQMERA MLENEKKKRE MAEKEKEKIE REKEELMERL KQIEEQTKKA
351 QQELEEQTRR ALELEQERKR AQSEAEKLAK ERQEAEEAKE ALLQASRDQK
401 KTQEQLALEM AELTARISQL EMARQKKESE AVEWQQKAQM VQEDLEKTRA
451 ELKTAMSTPH VAEPAENEQD EQDENGAEAS ADLRADAMAK DRSEEERTTE
501 AEKNERVQKH LKALTSELAN ARDESKKTAN DMIHAENMRL GRDKYKTLRQ
551 IRQGNTKQRI DEFESM
```

Residue Number Increasing Mass Decreasing Mass

Start - End	Observed	Mr(expt)	Mr(calc)	Delta	Miss	Sequence
17 - 24	976.5217	975.5144	975.5389	-0.0245	0	QLFDQVVK (No match)
71 - 89	2281.0745	2280.0672	2280.1273	-0.0601	1	AKFYPEDVSEELIQDITQR (No match)
73 - 89	2081.9587	2080.9514	2080.9953	-0.0438	0	FYPEDVSEELIQDITQR (No match)
90 - 96	894.5222	893.5149	893.5374	-0.0225	0	LFFLQVK (No match)
123 - 132	1236.5667	1235.5594	1235.5934	-0.0340	1	YGDFNKEVHK (No match)

133 - 145	1417.7543	1416.7470	1416.7724	-0.0254	1	**SGYLAGDKLLPQR**	(No match)
170 - 182	1552.7780	1551.7707	1551.7966	-0.0259	1	**GMLREDAVLEYLK**	Oxidation (M) (No match)
227 - 235	1104.5593	1103.5520	1103.5763	-0.0243	0	**IGFPWSEIR**	(No match)
252 - 262	1310.6636	1309.6563	1309.6818	-0.0255	1	**KAPDFVFYAPR**	(Ions score 33)
252 - 262	1310.6636	1309.6563	1309.6818	-0.0255	1	**KAPDFVFYAPR**	(No match)
253 - 262	1182.5712	1181.5639	1181.5869	-0.0230	0	**APDFVFYAPR**	(Ions score 56)
253 - 262	1182.5712	1181.5639	1181.5869	-0.0230	0	**APDFVFYAPR**	(No match)
269 - 282	1752.7915	1751.7842	1751.8157	-0.0314	0	**ILALCMGNHELYMR**	2 Oxidation (M) (No match)
284 - 295	1488.7512	1487.7439	1487.7766	-0.0326	1	**RKPDTIEVQQMK**	Oxidation (M) (No match)
350 - 360	1387.6676	1386.6603	1386.6851	-0.0248	1	**AQQELEEQTRR**	(No match)
361 - 368	987.4944	986.4871	986.5032	-0.0161	0	**ALELEQER**	(No match)
401 - 416	1847.9103	1846.9030	1846.9458	-0.0428	1	**KTQEQLALEMAELTAR**	Oxidation (M) (No match)
498 - 506	1077.4958	1076.4885	1076.5098	-0.0212	1	**TTEAEKNER**	(No match)
558 - 566	1170.4874	1169.4801	1169.5022	-0.0221	1	**QRIDEFESM**	Oxidation (M) (No match)

Mascot: http://www.matrixscience.com/

Spot 64

Protein View

Match to: **gi|119610473** Score: **79** Expect: **0.0027**
phosphoribosylformylglycinamidine synthase (FGAR amidotransferase), isoform CRA_a [Homo sapiens]

Nominal mass (M_r): **103226**; Calculated pI value: **5.60**
NCBI BLAST search of gi|119610473 against nr
Unformatted sequence string for pasting into other applications

Taxonomy: Homo sapiens

Fixed modifications: Carbamidomethyl (C)
Variable modifications: Oxidation (M)
Cleavage by Trypsin: cuts C-term side of KR unless next residue is P
Sequence Coverage: **6%**

Matched peptides shown in **Bold Red**

1 MSPVLHFYVR PSGHEGAASG HTRRKLQGKL PELQGVETEL CYNVNWTAEA
51 LPSAEETKKL MWLFGCPLLL DDVARESWLL PGSNDLLLEV GPRLNFSTPT
101 STNIVSVCRA TGLGPVDRVE TTRRYRLSFA HPPSAEVEAI ALATLHDRMT
151 EQHFPHPIQS FSPESMPEPL NGPINILGEG RLALEKANQE LGLALDSWDL
201 DFYTKRFQEL QRNPSTVEAF DLAQSNSEHS RHWFFKGQLH VDGQKLVHSL
251 FESIMSTQES SNPNNVLKFC DNSSAIQGKE VRFLRPEDPT RPSR**FQQQQG**
301 **LR**HVVFTAET HNFPTGVCPF SGATTGTGGR IRDVQCTGRG AHVVAGTAGY
351 CFGNLHIPGY NLPWEDLSFQ YPGNFARPLE VAIEASNGAS DYGNK**FGEPV**
401 **LAGFAR**SLGL QLPDGQRREW IKPIMFSGGI GSMEADHISK EAPEPGMEVV
451 KVGGPVYRIG VGGGAASSVQ VQGDNTSDLD FGAVQRGDPE MEQKMNRVIR
501 ACVEAPKGNP ICSLHDQGAG GNGNVLK**ELS DPAGAIIYTS R**FQLGDPTLN
551 ALEIWGAEYQ ESNALLLRSP NRDFLTHVSA RERCPACFVG TITGDRR**IVL**
601 **VDDRECPVR**R NGQGDAPPTP PPTPVDLELE WVLGKMPR**KE FFLQR**KPPML
651 QPLALPPGLS VHQALERVLR LPAVASKRYL TNKVDRSVGG LVAQQQCVGP
701 LQTPLADVAV VALSHEELIG AATALGEQPV KSLLDPKVAA RLAVAEALTN
751 LVFALVTDLR DVKCSGNWMW AAKLPGEGAA LADACEAMVA VMAALGVAVD
801 GGKDSLSMAA RVGTETVRAP GSLVISAYAV CPDITATVTP DLKHPEGR**GH**
851 **LLYVALSPGQ HR**LGGTALAQ CFSQLGEHPP DLDLPENLVR AFSITQGLLK
901 DRLLCSGHDV SDGGLVTCLL EMAFAGNCGL QVDVPVPRVD AELDGTG

Residue Number Increasing Mass Decreasing Mass

Start - End	Observed	Mr(expt)	Mr(calc)	Delta	Miss	Sequence
295 - 302	1004.5314	1003.5241	1003.5199	0.0042	0	FQQQQGLR (No match)
396 - 406	1163.6106	1162.6033	1162.6134	-0.0101	0	FGEPVLAGFAR (No match)
396 - 406	1163.6106	1162.6033	1162.6134	-0.0101	0	FGEPVLAGFAR (No match)
528 - 541	1492.7384	1491.7311	1491.7569	-0.0257	0	ELSDPAGAIIYTSR (No match)
598 - 609	1470.7594	1469.7521	1469.7660	-0.0139	1	IVLVDDRECPVR (No match)
639 - 645	967.5364	966.5291	966.5286	0.0005	1	KEFFLQR (No match)
849 - 862	1547.8214	1546.8141	1546.8368	-0.0227	0	GHLLYVALSPGQHR (No match)
849 - 862	1547.8214	1546.8141	1546.8368	-0.0227	0	GHLLYVALSPGQHR (Ions score 23)

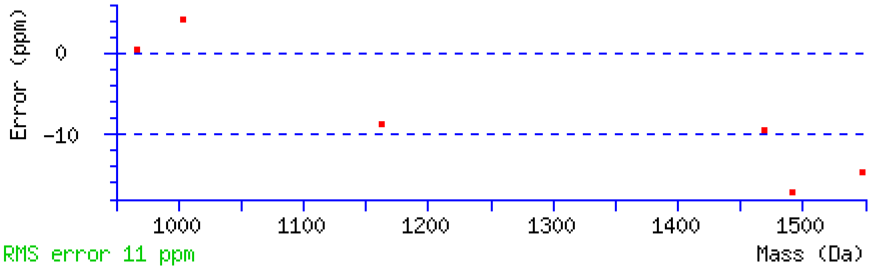

Spot 67

Protein View

Match to: **gi|35336** Score: **90** Expect: **0.00021**
dipeptidyl peptidase iv [Homo sapiens]

Nominal mass (M_r): **88947;** Calculated pI value: **5.67**
NCBI BLAST search of gi|35336 against nr
Unformatted sequence string for pasting into other applications

Taxonomy: Homo sapiens

Fixed modifications: Carbamidomethyl (C)
Variable modifications: Oxidation (M)
Cleavage by Trypsin: cuts C-term side of KR unless next residue is P
Sequence Coverage: **11%**

Matched peptides shown in **Bold Red**

```
  1 MKTPWKILLG LLGAAALVTI ITVPVVLLNK GTDDATADSR KTYTLTDYLK
 51 NTYRLKLYSL RWISDHEYLY KQENNILVFN AEYGNSSVFL ENSTFDEFGH
101 SINDYSISPD GQFILLEYNY VKQWRHSYTA SYDIYDLNKR QLITEERIPN
151 NTQWVTWSPV GHKLAYVWNN DIYVKIEPNL PSYRITWTGK EDIIYNGITD
201 WVYEEEVFSA YSALWWSPNG TFLAYAQFND TEVPLIEYSF YSDESLQYPK
251 TVRVPYPKAG AVNPTVKFFV VNTDSLSSVT NATSIQITAP ASMLIGDHYL
301 CDVTWATQER ISLQWLRRIQ NYSVMDICDY DESSGRWNCL VARQHIEMST
351 TGWVGRFRPS EPHFTLDGNS FYKIISNEEG YRHICYFQID KKDCTFITKG
401 TWEVIGIEAL TSDYLYYISN EYKGMPGGRN LYKIQLIDYT KVTCLSCELN
451 PERCQYYSVS FSKEAKYYQL RCSGPGLPLY TLHSSVNDKG LRVLEDNSAL
501 DKMLQNVQMP SKKLDFIILN ETKFWYQMIL PPHFDKSKKY PLLLDVYAGP
551 CSQKADTVFR LNWATYLAST ENIIVASFDG RGSGYQGDKI MHAINRRLGT
601 FEVEDQIEAA RQFSKMGFVD NKRIAIWGWS YGGYVTSMVL GSGSGVFKCG
651 IAVAPVSRWE YYDSVYTERY MGLPTPEDNL DHYRNSTVMS RAENFKQVEY
701 LLIHGTADDN VHFQQSAQIS KALVDVGVDF QAMWYTDEDH GIASSTAHQH
751 IYTHMSHFIK QCFSLP
```

Residue Number Increasing Mass Decreasing Mass

Start - End	Observed	Mr(expt)	Mr(calc)	Delta	Miss	Sequence
126 - 140	1845.8641	1844.8568	1844.8693	-0.0125	1	HSYTASYDIYDLNKR (Ions score 17)

126 - 140	1845.8641	1844.8568	1844.8693	-0.0125	1	HSYTASYDIYDLNKR	(No match)
176 - 184	1088.5791	1087.5718	1087.5662	0.0057	0	IEPNLPSYR	(No match)
176 - 184	1088.5791	1087.5718	1087.5662	0.0057	0	IEPNLPSYR	(No match)
357 - 373	2041.9663	2040.9590	2040.9693	-0.0103	0	FRPSEPHFTLDGNSFYK	(Ions score 9)
357 - 373	2041.9663	2040.9590	2040.9693	-0.0103	0	FRPSEPHFTLDGNSFYK	(No match)
374 - 382	1080.5363	1079.5290	1079.5247	0.0044	0	IISNEEGYR	(No match)
430 - 441	1511.8276	1510.8203	1510.8395	-0.0192	1	NLYKIQLIDYTK	(No match)
598 - 611	1577.7750	1576.7677	1576.7732	-0.0055	0	LGTFEVEDQIEAAR	(Ions score 21)
598 - 611	1577.7750	1576.7677	1576.7732	-0.0055	0	LGTFEVEDQIEAAR	(No match)
659 - 669	1510.6512	1509.6439	1509.6412	0.0027	0	WEYYDSVYTER	(No match)

Mascot: http://www.matrixscience.com/

Spot 69

Protein View

Match to: **gi|54873627** Score: **124** Expect: **7.7e-008**
myosin IG [Homo sapiens]

Nominal mass (M_r): **117393**; Calculated pI value: **9.00**
NCBI BLAST search of gi|54873627 against nr
Unformatted sequence string for pasting into other applications

Taxonomy: Homo sapiens
Links to retrieve other entries containing this sequence from NCBI Entrez:
gi|51094492 from Homo sapiens
gi|109658840 from Homo sapiens
gi|109731069 from Homo sapiens
gi|119581472 from Homo sapiens

Fixed modifications: Carbamidomethyl (C)
Variable modifications: Oxidation (M)
Cleavage by Trypsin: cuts C-term side of KR unless next residue is P
Sequence Coverage: **10%**

Matched peptides shown in **Bold Red**

```
  1 MEDEEGPEYG KPDFVLLDQV TMEDFMRNLQ LRFEKGRIYT YIGEVLVSVN
 51 PYQELPLYGP EAIARYQGRE LYERPPHLYA VANAAYKAMK HRSRDTCIVI
101 SGESGAGKTE ASKHIMQYIA AVTNPSQRAE VERVKDVLLK STCVLEAFGN
151 ARTNRNHNSS RFGKYMDINF DFKGDPIGGH IHSYLLEKSR VLKQHVGERN
201 FHAFYQLLRG SEDKQLHELH LERNPAVYNF THQGAGLNMT VHSALDSDEQ
251 SHQAVTEAMR VIGFSPEEVE SVHRILAAIL HLGNIEFVET EEGGLQKEGL
301 AVAEEALVDH VAELTATPRD LVLRSLLART VASGGRELIE KGHTAAEASY
351 ARDACAKAVY QRLFEWVVNR INSVMEPRGR DPRRDGKDTV IGVLDIYGFE
401 VFPVNSFEQF CINYCNEKLQ QLFIQLILKQ EQEEYEREGI TWQSVEYFNN
451 ATIVDLVERP HRGILAVLDE ACSSAGTITD RIFLQTLDTH HRHHLHYTSR
501 QLCPTDKTME FGRDFRIKHY AGDVTYSVEG FIDKNRDFLF QDFKRLLYNS
551 TDPTLRAMWP DGQQDITEVT KRPLTAGTLF KNSMVALVEN LASKEPFYVR
601 CIKPNEDKVA GKLDENHCRH QVAYLGLLEN VRVRRAGFAS RQPYSRFLLR
651 YKMTCEYTWP NHLLGSDKAA VSALLEQHGL QGDVAFGHSK LFIRSPRTLV
701 TLEQSRARLI PIIVLLLQKA WRGTLARWRC RRLRAIYTIM RWFRRHKVRA
751 HLAELQRRFQ AARQPPLYGR DLVWPLPPAV LQPFQDTCHA LFCRWRARQL
801 VKNIPPSDMP QIKAKVAAMG ALQGLRQDWG CRRAWARDYL SSATDNPTAS
851 SLFAQRLKTL RDKDGFGAVL FSSHVRKVNR FHKIRNRALL LTDQHLYKLD
901 PDRQYRVMRA VPLEAVTGLS VTSGGDQLVV LHARGQDDLV VCLHRSRPPL
951 DNRVGELVGV LAAHCQGEGR TLEVRVSDCI PLSHRGVRRL ISVEPRPEQP
```

1001 **EPDFR**CARGS FTLLWPSR

Residue Number Increasing Mass Decreasing Mass

Start - End	Observed	Mr(expt)	Mr(calc)	Delta	Miss	Sequence
70 - 87	2105.0588	2104.0515	2104.0741	-0.0226	0	ELYERPPHLYAVANAAYK (No match)
70 - 87	2105.0588	2104.0515	2104.0741	-0.0226	0	ELYERPPHLYAVANAAYK (Ions score 1)
141 - 152	1324.6190	1323.6117	1323.6241	-0.0123	0	STCVLEAFGNAR (No match)
200 - 209	1308.6716	1307.6643	1307.6774	-0.0131	0	NFHAFYQLLR (No match)
200 - 209	1308.6716	1307.6643	1307.6774	-0.0131	0	NFHAFYQLLR (No match)
261 - 274	1584.7902	1583.7829	1583.7943	-0.0114	0	VIGFSPEEVESVHR (No match)
261 - 274	1584.7902	1583.7829	1583.7943	-0.0114	0	VIGFSPEEVESVHR (Ions score 29)
862 - 876	1634.8130	1633.8057	1633.8212	-0.0154	1	DKDGFGAVLFSSHVR (No match)
862 - 876	1634.8130	1633.8057	1633.8212	-0.0154	1	DKDGFGAVLFSSHVR (Ions score 31)
954 - 970	1751.8676	1750.8603	1750.8783	-0.0180	0	VGELVGVLAAHCQGEGR (No match)
990 - 1005	1908.9635	1907.9562	1907.9741	-0.0178	0	LISVEPRPEQPEPDFR (No match)

Mascot: http://www.matrixscience.com/

Spot 70

Protein View

Match to: **gi|54873627** Score: **220** Expect: **1.9e-017**
myosin IG [Homo sapiens]

Nominal mass (M_r): **117393**; Calculated pI value: **9.00**
NCBI BLAST search of gi|54873627 against nr
Unformatted sequence string for pasting into other applications

Taxonomy: Homo sapiens
Links to retrieve other entries containing this sequence from NCBI Entrez:
gi|51094492 from Homo sapiens
gi|109658840 from Homo sapiens
gi|109731069 from Homo sapiens
gi|119581472 from Homo sapiens

Fixed modifications: Carbamidomethyl (C)
Variable modifications: Oxidation (M)
Cleavage by Trypsin: cuts C-term side of KR unless next residue is P
Sequence Coverage: **15%**

Matched peptides shown in **Bold Red**

1 MEDEEGPEYG KPDFVLLDQV TMEDFMRNLQ LRFEKGRIYT YIGEVLVSVN
51 PYQELPLYGP EAIARYQGR**E LYERPPHLYA VANAAYK**AMK HRSRDTCIVI
101 SGESGAGKTE ASKHIMQYIA AVTNPSQRAE VERVKDVLLK **STCVLEAFGN**
151 **AR**TNRNHNSS RFGKYMDINF DFKGDPIGGH IHSYLLEKSR VLKQHVGER**N**
201 **FHAFYQLLR**G SEDKQLHELH LERNPAVYNF THQGAGLNMT VHSALDSDEQ
251 SHQAVTEAMR **VIGFSPEEVE SVHR**ILAAIL HLGNIEFVET EEGGLQKEGL
301 AVAEEALVDH VAELTATPRD LVLRSLLART VASGGRELIE KGHTAAEASY
351 ARDACAKAVY QRLFEWVVNR INSVMEPRGR DPRRDGKDTV IGVLDIYGFE
401 VFPVNSFEQF CINYCNEKLQ QLFIQLILKQ EQEEYEREGI TWQSVEYFNN
451 ATIVDLVERP HRGILAVLDE ACSSAGTITD R**IFLQTLDTH HRHHLHYTSR**
501 QLCPTDKTME FGRDFR**IKHY AGDVTYSVEG FIDK**NRDFLF QDFKRLLYNS
551 TDPTLRAMWP DGQQDITEVT KRPLTAGTLF KNSMVALVEN LASKEPFYVR
601 CIKPNEDKVA GKLDENHCRH QVAYLGLLEN VRVRRAGFAS RQPYSRFLLR
651 YKMTCEYTWP NHLLGSDKAA VSALLEQHGL QGDVAFGHSK LFIRSPRTLV
701 TLEQSRARLI PIIVLLLQKA WRGTLARWRC RRLRAIYTIM RWFRRHKVRA
751 HLAELQRRFQ AARQPPLYGR DLVWPLPPAV LQPFQDTCHA LFCRWRARQL
801 VKNIPPSDMP QIKAKVAAMG ALQGLRQDWG CRRAWAR**DYL SSATDNPTAS**
851 **SLFAQR**LKTL R**DKDGFGAVL FSSHVR**KVNR FHKIRNRALL LTDQHLYKLD
901 PDRQYRVMRA VPLEAVTGLS VTSGGDQLVV LHARGQDDLV VCLHRSRPPL
951 DNR**VGELVGV LAAHCQGEGR** TLEVRVSDCI PLSHRGVRR**L ISVEPRPEQP**

1001 **EPDFR**CARGS FTLLWPSR

Residue Number Increasing Mass Decreasing Mass

Start - End	Observed	Mr(expt)	Mr(calc)	Delta	Miss	Sequence
70 - 87	2105.0747	2104.0674	2104.0741	-0.0067	0	ELYERPPHLYAVANAAYK (No match)
70 - 87	2105.0747	2104.0674	2104.0741	-0.0067	0	ELYERPPHLYAVANAAYK (Ions score 15)
141 - 152	1324.6265	1323.6192	1323.6241	-0.0048	0	STCVLEAFGNAR (No match)
200 - 209	1308.6786	1307.6713	1307.6774	-0.0061	0	NFHAFYQLLR (No match)
261 - 274	1584.7950	1583.7877	1583.7943	-0.0066	0	VIGFSPEEVESVHR (No match)
261 - 274	1584.7950	1583.7877	1583.7943	-0.0066	0	VIGFSPEEVESVHR (Ions score 52)
482 - 492	1380.7362	1379.7289	1379.7310	-0.0020	0	IFLQTLDTHHR (No match)
493 - 500	1050.5205	1049.5132	1049.5155	-0.0023	0	HHLHYTSR (No match)
517 - 534	2041.9717	2040.9644	2041.0156	-0.0511	1	IKHYAGDVTYSVEGFIDK (No match)
517 - 534	2041.9717	2040.9644	2041.0156	-0.0511	1	IKHYAGDVTYSVEGFIDK (No match)
838 - 856	2043.9648	2042.9575	2042.9545	0.0031	0	DYLSSATDNPTASSLFAQR (No match)
862 - 876	1634.8225	1633.8152	1633.8212	-0.0059	1	DKDGFGAVLFSSHVR (Ions score 38)
862 - 876	1634.8225	1633.8152	1633.8212	-0.0059	1	DKDGFGAVLFSSHVR (No match)
954 - 970	1751.8779	1750.8706	1750.8783	-0.0077	0	VGELVGVLAAHCQGEGR (No match)
990 - 1005	1908.9722	1907.9649	1907.9741	-0.0091	0	LISVEPRPEQPEPDFR (No match)

Mascot: http://www.matrixscience.com/

Spot 71

Protein View

Match to: **gi|2078273** Score: **128** Expect: **3e-008**
SLP-76 associated protein [Homo sapiens]

Nominal mass (M_r): **85627;** Calculated pI value: **6.11**
NCBI BLAST search of gi|2078273 against nr
Unformatted sequence string for pasting into other applications

Taxonomy: Homo sapiens

Fixed modifications: Carbamidomethyl (C)
Variable modifications: Oxidation (M)
Cleavage by Trypsin: cuts C-term side of KR unless next residue is P
Sequence Coverage: **8%**

Matched peptides shown in **Bold Red**

```
  1 MAKYNTGGNP TEDVSVNSRP FRVTGPNSSS GIQARKNLFN NQGNASPPAG
 51 PSNVPKFGSP KPPVAVKPSS EEKPDKEPKP PFLKPTGAGQ RFGTPASLTT
101 RDPEAKVGFL KPVGPKPINL PKEDSKPTFP WPPGNKPSLH SVNQDHDLKP
151 LGPKSGPTPP TSENEQKQAF PKLTGVKGKF MSASQDLEPK PLFPKPAFGQ
201 KPPLSTENSH EDESPMKNVS SSKGSPAPLG VRSKSGPLKP AREDSENKDH
251 AGEISSLPFP GVVLKPAASR GGLGLSKNGE EKKEDRKIDA AKNTFQSKIN
301 QEELASGTPP ARFPKAPSKL TVGGPWGQSQ EKEKGDKNSA TPKQKPLPPL
351 FTLGPPPPKP NRPPNVDLTK FHKTSSGNST SKGQTSYSTT SLPPPPPSHP
401 ASQPPLPASH PSQPPVPSLP PRNIKPPFDL KSPVNEDNQD GVTHSDGAGN
451 LDEEQDSEGE TYEDIEASKE REKKREKEEK KRLELEKKEQ KEKEKKEQEI
501 KKKFKLTGPI QVIHLAKACC DVKGGKNELS FKQGEQIEII RITDNPEGKW
551 LGRTARGSYG YIKTTAVEID YDSLKLKKDS LGAPSRPIED DQEVYDDVAE
601 QDDISSHSQS GSGGIFPPPP DDDIYDGIEE EDADDGFPAP PKQLDMGDEV
651 YDDVDTSDFP VSSAEMSQGT NFGKAKTEEK DLKKLKKQEK EEKDFRKKFK
701 YDGEIRVLYS TKVTTSITSK KWGTRDLQVK PGESLEVIQT TDDTKVLCRN
751 EEGKYGYVLR SYLADNDGEI YDDIADGCIY DND
```

Residue Number Increasing Mass Decreasing Mass

Start - End	Observed	Mr(expt)	Mr(calc)	Delta	Miss	Sequence
107 - 122	1704.0326	1703.0253	1703.0497	-0.0244	0	VGFLKPVGPKPINLPK (No match)
299 - 312	1482.7274	1481.7201	1481.7474	-0.0272	0	INQEELASGTPPAR (No match)
299 - 312	1482.7274	1481.7201	1481.7474	-0.0272	0	INQEELASGTPPAR (Ions score 46)
506 - 517	1289.7616	1288.7543	1288.7867	-0.0323	0	LTGPIQVIHLAK (No match)
506 - 517	1289.7616	1288.7543	1288.7867	-0.0323	0	LTGPIQVIHLAK (Ions score 21)
533 - 541	1085.5791	1084.5718	1084.5876	-0.0158	0	QGEQIEIIR (No match)
699 - 706	1027.5038	1026.4965	1026.5134	-0.0169	1	FKYDGEIR (No match)
755 - 760	770.4147	769.4074	769.4122	-0.0048	0	YGYVLR (No match)

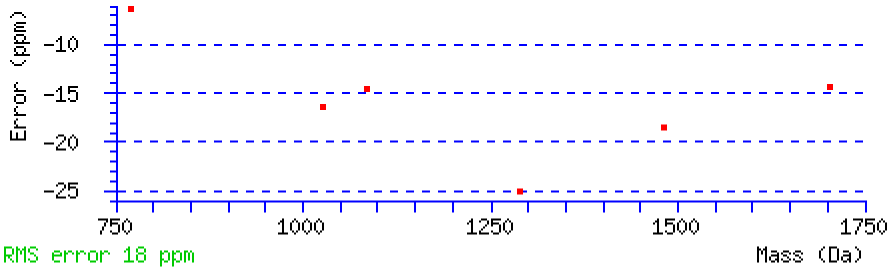

Mascot: http://www.matrixscience.com/

Spot 75

Protein View

Match to: **gi|54873627** Score: **617** Expect: **3.8e-057**
myosin IG [Homo sapiens]

Nominal mass (M_r): **117393**; Calculated pI value: **9.00**
NCBI BLAST search of gi|54873627 against nr
Unformatted sequence string for pasting into other applications

Taxonomy: Homo sapiens
Links to retrieve other entries containing this sequence from NCBI Entrez:
gi|51094492 from Homo sapiens
gi|109658840 from Homo sapiens
gi|109731069 from Homo sapiens
gi|119581472 from Homo sapiens

Fixed modifications: Carbamidomethyl (C)
Variable modifications: Oxidation (M)
Cleavage by Trypsin: cuts C-term side of KR unless next residue is P
Sequence Coverage: **42%**

Matched peptides shown in **Bold Red**

1 MEDEEGPEYG KPDFVLLDQV TMEDFMRNLQ LRFEKGRIYT YIGEVLVSVN
51 PYQELPLYGP EAIARYQGR**E LYERPPHLYA VANAAYK**AMK HRSRDTCIVI
101 SGESGAGKTE ASK**HIMQYIA AVTNPSQR**AE VERVKDVLLK **STCVLEAFGN**
151 **AR**TNRNHNSS RFGKYMDINF DFKGDPIGGH IHSYLLEKSR VLKQHVGER**N**
201 **FHAFYQLLRG SEDKQLHELH LER**NPAVYNF THQGAGLNMT VHSALDSDEQ
251 SHQAVTEAMR **VIGFSPEEVE SVHR**ILAAIL HLGNIEFVET EEGGLQK**EGL**
301 **AVAEEALVDH VAELTATPR**D LVLRSLLART VASGGR**ELIE KGHTAAEASY**
351 **AR**DACAKAVY QR**LFEWVVNR** INSVMEPRGR DPRRDGKDTV IGVLDIYGFE
401 VFPVNSFEQF CINYCNEKLQ QLFIQLILKQ EQEEYEREGI TWQSVEYFNN
451 ATIVDLVERP HR**GILAVLDE ACSSAGTITD RIFLQTLDTH HRHHLHYTSR**
501 QLCPTDKTME FGRDFRIKHY AGDVTYSVEG FIDKNR**DFLF QDFKRLLYNS**
551 **TDPTLR**AMWP DGQQDITEVT K**RPLTAGTLF KNSMVALVEN LASKEPFYVR**
601 CIKPNEDKVA GKLDENHCR**H QVAYLGLLEN VRVR**RAGFAS R**QPYSRFLLR**
651 YKMTCEYTWP NHLLGSDK**AA VSALLEQHGL QGDVAFGHSK** LFIRSPR**TLV**
701 **TLEQSRARLI PIIVLLLQK**A WRGTLARWRC RRLRAIYTIM RWFRRHKVR**A**
751 **HLAELQR**RFQ AAR**QPPLYGR** DLVWPLPPAV LQPFQDTCHA LFCRWRARQL
801 VKNIPPSDMP QIKAK**VAAMG ALQGLR**QDWG CRRAWAR**DYL SSATDNPTAS**
851 **SLFAQR**LKTL R**DKDGFGAVL FSSHVR**KVNR FHKIRNRALL LTDQHLYKLD
901 PDRQYRVMR**A VPLEAVTGLS VTSGGDQLVV LHARGQDDLV VCLHRSRPPL**
951 **DNRVGELVGV LAAHCQGEGR** TLEVR**VSDCI PLSHR**GVR**RL ISVEPRPEQP**

1001 **EPDFR**CARGS FTLLWPSR

Residue Number Increasing Mass Decreasing Mass

Start - End	Observed	Mr(expt)	Mr(calc)	Delta	Miss	Sequence
70 - 87	2105.0605	2104.0532	2104.0741	-0.0209	0	ELYERPPHLYAVANAAYK (No match)
114 - 128	1744.8690	1743.8617	1743.8726	-0.0109	0	HIMQYIAAVTNPSQR Oxidation (M) (No match)
141 - 152	1324.6283	1323.6210	1323.6241	-0.0030	0	STCVLEAFGNAR (No match)
200 - 209	1308.6813	1307.6740	1307.6774	-0.0034	0	NFHAFYQLLR (Ions score 33)
200 - 209	1308.6813	1307.6740	1307.6774	-0.0034	0	NFHAFYQLLR (No match)
210 - 223	1690.8394	1689.8321	1689.8434	-0.0113	1	GSEDKQLHELHLER (No match)
261 - 274	1584.7917	1583.7844	1583.7943	-0.0099	0	VIGFSPEEVESVHR (No match)
261 - 274	1584.7917	1583.7844	1583.7943	-0.0099	0	VIGFSPEEVESVHR (Ions score 62)
298 - 319	2291.1658	2290.1585	2290.1804	-0.0219	0	EGLAVAEEALVDHVAELTATPR (No match)
337 - 352	1745.8689	1744.8616	1744.8743	-0.0127	1	ELIEKGHTAAEASYAR (No match)
342 - 352	1133.5300	1132.5227	1132.5261	-0.0034	0	GHTAAEASYAR (No match)
363 - 370	1062.5543	1061.5470	1061.5658	-0.0187	0	LFEWVVNR (No match)
463 - 481	1948.9507	1947.9434	1947.9571	-0.0137	0	GILAVLDEACSSAGTITDR (No match)
482 - 492	1380.7328	1379.7255	1379.7310	-0.0054	0	IFLQTLDTHHR (No match)
482 - 500	2412.2185	2411.2112	2411.2359	-0.0247	1	IFLQTLDTHHRHHLHYTSR (No match)
493 - 500	1050.5220	1049.5147	1049.5155	-0.0008	0	HHLHYTSR (No match)
537 - 545	1215.6146	1214.6073	1214.6084	-0.0010	1	DFLFQDFKR (No match)
546 - 556	1292.6783	1291.6710	1291.6772	-0.0062	0	LLYNSTDPTLR (No match)
572 - 581	1103.6329	1102.6256	1102.6498	-0.0242	0	RPLTAGTLFK (No match)
582 - 600	2183.1033	2182.0960	2182.1091	-0.0131	1	NSMVALVENLASKEPFYVR Oxidation (M) (No match)
620 - 632	1511.8236	1510.8163	1510.8255	-0.0092	0	HQVAYLGLLENVR (Ions score 47)
620 - 632	1511.8236	1510.8163	1510.8255	-0.0092	0	HQVAYLGLLENVR (No match)
620 - 634	1766.8912	1765.8839	1765.9950	-0.1111	1	HQVAYLGLLENVRVR (No match)
642 - 650	1179.6141	1178.6068	1178.6560	-0.0492	1	QPYSRFLLR (No match)
669 - 690	2235.1411	2234.1338	2234.1443	-0.0104	0	AAVSALLEQHGLQGDVAFGHSK (No match)
698 - 706	1046.5814	1045.5741	1045.5767	-0.0026	0	TLVTLEQSR (No match)
707 - 719	1490.0146	1489.0073	1489.0119	-0.0046	1	ARLIPIIVLLLQK (No match)
750 - 757	937.5231	936.5158	936.5141	0.0018	0	AHLAELQR (No match)
764 - 770	830.4515	829.4442	829.4446	-0.0004	0	QPPLYGR (No match)
816 - 826	1102.5951	1101.5878	1101.5964	-0.0086	0	VAAMGALQGLR Oxidation (M) (No match)
838 - 856	2043.9462	2042.9389	2042.9545	-0.0155	0	DYLSSATDNPTASSLFAQR (No match)
838 - 856	2043.9462	2042.9389	2042.9545	-0.0155	0	DYLSSATDNPTASSLFAQR (Ions score 89)
862 - 876	1634.8175	1633.8102	1633.8212	-0.0109	1	DKDGFGAVLFSSHVR (Ions score 44)
862 - 876	1634.8175	1633.8102	1633.8212	-0.0109	1	DKDGFGAVLFSSHVR (No match)
864 - 876	1391.6959	1390.6886	1390.6993	-0.0106	0	DGFGAVLFSSHVR (No match)
910 - 934	2489.3459	2488.3386	2488.3648	-0.0262	0	AVPLEAVTGLSVTSGGDQLVVLHAR (No match)
935 - 945	1311.6616	1310.6543	1310.6401	0.0142	0	GQDDLVVCLHR (No match)
946 - 953	954.5061	953.4988	953.5042	-0.0054	0	SRPPLDNR (No match)
954 - 970	1751.8722	1750.8649	1750.8783	-0.0134	0	VGELVGVLAAHCQGEGR (No match)
976 - 985	1183.5902	1182.5829	1182.5815	0.0014	0	VSDCIPLSHR (No match)

989 - 1005	2065.0735	2064.0662	2064.0752	-0.0089	1	RLISVEPRPEQPEPDFR	(No match)
990 - 1005	1908.9680	1907.9607	1907.9741	-0.0133	0	LISVEPRPEQPEPDFR	(No match)

Mascot: http://www.matrixscience.com/

Spot 77

Protein View

Match to: **gi|27574040** Score: **107** Expect: **3.8e-006**
Chain A, Human Dipeptidyl Peptidase IvCD26 IN COMPLEX WITH AN Inhibitor

Nominal mass (M_r): **85008**; Calculated pI value: **5.67**
NCBI BLAST search of gi|27574040 against nr
Unformatted sequence string for pasting into other applications

Taxonomy: Homo sapiens
Links to retrieve other entries containing this sequence from NCBI Entrez:
gi|27574041 from Homo sapiens
gi|34810234 from Homo sapiens
gi|34810235 from Homo sapiens
gi|34810236 from Homo sapiens
gi|34810237 from Homo sapiens
gi|50513762 from Homo sapiens
gi|50513763 from Homo sapiens
gi|50513770 from Homo sapiens
gi|50513771 from Homo sapiens
gi|55670507 from Homo sapiens
gi|55670508 from Homo sapiens
gi|55670509 from Homo sapiens
gi|55670510 from Homo sapiens
gi|58176672 from Homo sapiens
gi|58176673 from Homo sapiens
gi|60594437 from Homo sapiens
gi|60594438 from Homo sapiens
gi|60594439 from Homo sapiens
gi|60594440 from Homo sapiens
gi|83754000 from Homo sapiens
gi|83754001 from Homo sapiens
gi|88192461 from Homo sapiens
gi|88192462 from Homo sapiens
gi|134105168 from Homo sapiens
gi|134105169 from Homo sapiens
gi|145580125 from Homo sapiens
gi|145580126 from Homo sapiens
gi|145580127 from Homo sapiens
gi|145580128 from Homo sapiens

Fixed modifications: Carbamidomethyl (C)
Variable modifications: Oxidation (M)

Cleavage by Trypsin: cuts C-term side of KR unless next residue is P

Sequence Coverage: **11%**

Matched peptides shown in **Bold Red**

1 SRKTYTLTDY LKNTYRLKLY SLRWISDHEY LYKQENNILV FNAEYGNSSV

51 FLENSTFDEF GHSINDYSIS PDGQFILLEY NYVKQWR**HSY** **TASYDIYDLN**

101 **KR**QLITEERI PNNTQWVTWS PVGHKLAYVW NNDIYVK**IEP** **NLPSYR**ITWT

151 GKEDIIYNGI TDWVYEEEVF SAYSALWWSP NGTFLAYAQF NDTEVPLIEY

201 SFYSDESLQY PKTVRVPYPK AGAVNPTVKF FVVNTDSLSS VTNATSIQIT

251 APASMLIGDH YLCDVTWATQ ER**ISLQWLR**R IQNYSVMDIC DYDESSGRWN

301 CLVARQHIEM STTGWVGR**FR** **PSEPHFTLDG** **NSFYKIISNE** **EGYR**HICYFQ

351 IDKKDCTFIT KGTWEVIGIE ALTSDYLYYI SNEYKGMPGG RNLYKIQLSD

401 YTKVTCLSCE LNPERCQYYS VSFSKEAKYY QLRCSGPGLP LYTLHSSVND

451 KGLRVLEDNS ALDKMLQNVQ MPSKKLDFII LNETKFWYQM ILPPHFDKSK

501 KYPLLLDVYA GPCSQKADTV FRLNWATYLA STENIIVASF DGRGSGYQGD

551 KIMHAINRR**L** **GTFEVEDQIE** **AAR**QFSKMGF VDNKRIAIWG WSYGGYVTSM

601 VLGSGSGVFK CGIAVAPVSR **WEYYDSVYTE** **R**YMGLPTPED NLDHYRNSTV

651 MSRAENFKQV EYLLIHGTAD DNVHFQQSAQ ISKALVDVGV DFQAMWYTDE

701 DHGIASSTAH QHIYTHMSHF IKQCFSLP

Residue Number Increasing Mass Decreasing Mass

Start - End	Observed	Mr(expt)	Mr(calc)	Delta	Miss	Sequence
88 - 102	1845.8623	1844.8550	1844.8693	-0.0143	1	HSYTASYDIYDLNKR (No match)
88 - 102	1845.8623	1844.8550	1844.8693	-0.0143	1	HSYTASYDIYDLNKR (Ions score 22)
138 - 146	1088.5652	1087.5579	1087.5662	-0.0082	0	IEPNLPSYR (No match)
138 - 146	1088.5652	1087.5579	1087.5662	-0.0082	0	IEPNLPSYR (Ions score 2)
273 - 279	915.5338	914.5265	914.5338	-0.0072	0	ISLQWLR (No match)
319 - 335	2041.9568	2040.9495	2040.9693	-0.0198	0	FRPSEPHFTLDGNSFYK (No match)
319 - 335	2041.9568	2040.9495	2040.9693	-0.0198	0	FRPSEPHFTLDGNSFYK (Ions score 18)
336 - 344	1080.5293	1079.5220	1079.5247	-0.0026	0	IISNEEGYR (No match)
560 - 573	1577.7671	1576.7598	1576.7732	-0.0134	0	LGTFEVEDQIEAAR (Ions score 15)
560 - 573	1577.7671	1576.7598	1576.7732	-0.0134	0	LGTFEVEDQIEAAR (No match)
621 - 631	1510.6403	1509.6330	1509.6412	-0.0081	0	WEYYDSVYTER (No match)

Spot 79

Protein View

Match to: **gi|54873627** Score: **304** Expect: **7.7e-026**
myosin IG [Homo sapiens]

Nominal mass (M_r): **117393**; Calculated pI value: **9.00**
NCBI BLAST search of gi|54873627 against nr
Unformatted sequence string for pasting into other applications

Taxonomy: Homo sapiens
Links to retrieve other entries containing this sequence from NCBI Entrez:
gi|51094492 from Homo sapiens
gi|109658840 from Homo sapiens
gi|109731069 from Homo sapiens
gi|119581472 from Homo sapiens

Fixed modifications: Carbamidomethyl (C)
Variable modifications: Oxidation (M)
Cleavage by Trypsin: cuts C-term side of KR unless next residue is P
Sequence Coverage: **18%**

Matched peptides shown in **Bold Red**

1 MEDEEGPEYG KPDFVLLDQV TMEDFMRNLQ LRFEKGRIYT YIGEVLVSVN
51 PYQELPLYGP EAIARYQGR**E** **LYERPPHLYA** **VANAAYK**AMK HRSRDTCIVI
101 SGESGAGKTE ASKHIMQYIA AVTNPSQRAE VERVKDVLLK **STCVLEAFGN**
151 **AR**TNRNHNSS RFGKYMDINF DFKGDPIGGH IHSYLLEKSR VLKQHVGER**N**
201 **FHAFYQLLR**G SEDKQLHELH LERNPAVYNF THQGAGLNMT VHSALDSDEQ
251 SHQAVTEAMR **VIGFSPEEVE** **SVHR**ILAAIL HLGNIEFVET EEGGLQKEGL
301 AVAEEALVDH VAELTATPRD LVLRSLLART VASGGRELIE KGHTAAEASY
351 ARDACAKAVY QR**LFEWVVNR** INSVMEPRGR DPRRDGKDTV IGVLDIYGFE
401 VFPVNSFEQF CINYCNEKLQ QLFIQLILKQ EQEEYEREGI TWQSVEYFNN
451 ATIVDLVERP HRGILAVLDE ACSSAGTITD R**IFLQTLDTH** **HRHHLHYTSR**
501 QLCPTDKTME FGRDFRIKHY AGDVTYSVEG FIDKNR**DFLF** **QDFKRLLYNS**
551 **TDPTLR**AMWP DGQQDITEVT K**RPLTAGTLF** **K**NSMVALVEN LASKEPFYVR
601 CIKPNEDKVA GKLDENHCR**H** **QVAYLGLLEN** **VR**VRRAGFAS RQPYSRFLLR
651 YKMTCEYTWP NHLLGSDKAA VSALLEQHGL QGDVAFGHSK LFIRSPRTLV
701 TLEQSRARLI PIIVLLLQKA WRGTLARWRC RRLRAIYTIM RWFRRHKVRA
751 HLAELQRRFQ AARQPPLYGR DLVWPLPPAV LQPFQDTCHA LFCRWRARQL
801 VKNIPPSDMP QIKAKVAAMG ALQGLRQDWG CRRAWAR**DYL** **SSATDNPTAS**
851 **SLFAQR**LKTL R**DKDGFGAVL** **FSSHVR**KVNR FHKIRNRALL LTDQHLYKLD
901 PDRQYRVMRA VPLEAVTGLS VTSGGDQLVV LHARGQDDLV VCLHRSRPPL
951 DNR**VGELVGV** **LAAHCQGEGR** TLEVRVSDCI PLSHRGVRR**L** **ISVEPRPEQP**

1001 **EPDFR**CARGS FTLLWPSR

Residue Number Increasing Mass Decreasing Mass

Start - End	Observed	Mr(expt)	Mr(calc)	Delta	Miss	Sequence
70 - 87	2105.0796	2104.0723	2104.0741	-0.0018	0	ELYERPPHLYAVANAAYK (Ions score 9)
70 - 87	2105.0796	2104.0723	2104.0741	-0.0018	0	ELYERPPHLYAVANAAYK (No match)
141 - 152	1324.6353	1323.6280	1323.6241	0.0040	0	STCVLEAFGNAR (No match)
200 - 209	1308.6868	1307.6795	1307.6774	0.0021	0	NFHAFYQLLR (No match)
200 - 209	1308.6868	1307.6795	1307.6774	0.0021	0	NFHAFYQLLR (No match)
261 - 274	1584.8019	1583.7946	1583.7943	0.0003	0	VIGFSPEEVESVHR (Ions score 24)
261 - 274	1584.8019	1583.7946	1583.7943	0.0003	0	VIGFSPEEVESVHR (No match)
363 - 370	1062.5759	1061.5686	1061.5658	0.0029	0	LFEWVVNR (No match)
482 - 492	1380.7434	1379.7361	1379.7310	0.0052	0	IFLQTLDTHHR (No match)
493 - 500	1050.5264	1049.5191	1049.5155	0.0036	0	HHLHYTSR (No match)
537 - 545	1215.6239	1214.6166	1214.6084	0.0083	1	DFLFQDFKR (No match)
546 - 556	1292.6907	1291.6834	1291.6772	0.0062	0	LLYNSTDPTLR (No match)
572 - 581	1103.6497	1102.6424	1102.6498	-0.0074	0	RPLTAGTLFK (No match)
620 - 632	1511.8296	1510.8223	1510.8255	-0.0032	0	HQVAYLGLLENVR (No match)
620 - 632	1511.8296	1510.8223	1510.8255	-0.0032	0	HQVAYLGLLENVR (Ions score 28)
838 - 856	2043.9617	2042.9544	2042.9545	-0.0000	0	DYLSSATDNPTASSLFAQR (No match)
838 - 856	2043.9617	2042.9544	2042.9545	-0.0000	0	DYLSSATDNPTASSLFAQR (Ions score 78)
862 - 876	1634.8271	1633.8198	1633.8212	-0.0013	1	DKDGFGAVLFSSHVR (No match)
954 - 970	1751.8837	1750.8764	1750.8783	-0.0019	0	VGELVGVLAAHCQGEGR (No match)
990 - 1005	1908.9851	1907.9778	1907.9741	0.0038	0	LISVEPRPEQPEPDFR (No match)

Mascot: http://www.matrixscience.com/

Spot 80

Protein View

Match to: **gi|119629788** Score: **215** Expect: **6.1e-017**
integrin, beta 2 (complement component 3 receptor 3 and 4 subunit), isoform CRA_b [Homo sapiens]

Nominal mass (M_r): **81553**; Calculated pI value: **6.54**
NCBI BLAST search of gi|119629788 against nr
Unformatted sequence string for pasting into other applications

Taxonomy: Homo sapiens
Links to retrieve other entries containing this sequence from NCBI Entrez:
gi|119629789 from Homo sapiens

Fixed modifications: Carbamidomethyl (C)
Variable modifications: Oxidation (M)
Cleavage by Trypsin: cuts C-term side of KR unless next residue is P
Sequence Coverage: **17%**

Matched peptides shown in **Bold Red**

```
  1 MLGLRPPLLA LVGLLSLGCV LSQECTKFKV SSCRECIESG PGCTWCQKLN
 51 FTGPGDPDSI RCDTRPQLLM RGCAADDIMD PTSLAETQED HNGGQKQLSP
101 QKVTLYLRPG FGSFVDKTVL PFVNTHPDKL RNPCPNKEKE CQPPFAFRHV
151 LKLTNNSNQF QTEVGKQLIS GNLDAPEGGL DAMMQVAACP EEIGWRNVTR
201 LLVFATDDGF HFAGDGKLGA ILTPNDGRCH LEDNLYKRSN EFDYPSVGQL
251 AHKLAENNIQ PIFAVTSRMV KTYEKLTEII PKSAVGELSE DSSNVVHLIK
301 NAYNKLSSRV FLDHNALPDT LKVTYDSFCS NGVTHRNQPR GDCDGVQINV
351 PITFQVKVTA TECIQEQSFV IRALGFTDIV TVQVLPQCEC RCRDQSRDRS
401 LCHGKGFLEC GICRCDTGYI GKNCECQTQG RSSQELEGSC RKDNNSIICS
451 GLGDCVCGQC LCHTSDVPGK LIYGQYCECD TINCERYNGQ VCGGPGRGLC
501 FCGKCRCHPG FEGSACQCER TTEGCLNPRR VECSGRGRCR CNVCECHSGY
551 QLPLCQECPG CPSPCGKYIS CAECLKFEKG PFGKNCSAAC PGLQLSNNPV
601 KGRTCKERDS EGCWVAYTLE QQDGMDRYLI YVDESRECVA GPNIAAIVGG
651 TVAGIVLIGI LLLVIWKALI HLSDLREYRR FEKEKLKSQW NNDNPLFKSA
701 TTTVMNPKFA ES
```

Residue Number Increasing Mass Decreasing Mass

Start - End	Observed	Mr(expt)	Mr(calc)	Delta	Miss	Sequence
138 - 148	1408.6656	1407.6583	1407.6605	-0.0021	1	EKECQPPFAFR (No match)
140 - 148	1151.5232	1150.5159	1150.5229	-0.0070	0	ECQPPFAFR (Ions score 7)

140 - 148	1151.5232	1150.5159	1150.5229	-0.0070	0	ECQPPFAFR	(No match)
239 - 253	1691.7924	1690.7851	1690.7950	-0.0099	0	SNEFDYPSVGQLAHK	(No match)
254 - 268	1672.8923	1671.8850	1671.8943	-0.0093	0	LAENNIQPIFAVTSR	(No match)
254 - 268	1672.8923	1671.8850	1671.8943	-0.0093	0	LAENNIQPIFAVTSR	(Ions score 54)
323 - 336	1642.7216	1641.7143	1641.7205	-0.0062	0	VTYDSFCSNGVTHR	(No match)
406 - 414	1111.4971	1110.4898	1110.4950	-0.0052	0	GFLECGICR	(No match)
406 - 414	1111.4971	1110.4898	1110.4950	-0.0052	0	GFLECGICR	(Ions score 8)
471 - 486	2093.8599	2092.8526	2092.8652	-0.0126	0	LIYGQYCECDTINCER	(No match)
487 - 497	1164.5157	1163.5084	1163.5141	-0.0057	0	YNGQVCGGPGR	(No match)
487 - 497	1164.5157	1163.5084	1163.5141	-0.0057	0	YNGQVCGGPGR	(No match)
498 - 506	1157.5769	1156.5696	1156.4939	0.0757	1	GLCFCGKCR	(No match)
507 - 520	1694.6338	1693.6265	1693.6395	-0.0130	0	CHPGFEGSACQCER	(No match)
628 - 636	1157.5769	1156.5696	1156.5764	-0.0068	0	YLIYVDESR	(No match)

Spot 81

Protein View

Match to: **gi|27574040** Score: **449** Expect: **2.4e-040**
Chain A, Human Dipeptidyl Peptidase IvCD26 IN COMPLEX WITH AN Inhibitor

Nominal mass (M_r): **85008**; Calculated pI value: **5.67**
NCBI BLAST search of gi|27574040 against nr
Unformatted sequence string for pasting into other applications

Taxonomy: Homo sapiens
Links to retrieve other entries containing this sequence from NCBI Entrez:
gi|27574041 from Homo sapiens
gi|34810234 from Homo sapiens
gi|34810235 from Homo sapiens
gi|34810236 from Homo sapiens
gi|34810237 from Homo sapiens
gi|50513762 from Homo sapiens
gi|50513763 from Homo sapiens
gi|50513770 from Homo sapiens
gi|50513771 from Homo sapiens
gi|55670507 from Homo sapiens
gi|55670508 from Homo sapiens
gi|55670509 from Homo sapiens
gi|55670510 from Homo sapiens
gi|58176672 from Homo sapiens
gi|58176673 from Homo sapiens
gi|60594437 from Homo sapiens
gi|60594438 from Homo sapiens
gi|60594439 from Homo sapiens
gi|60594440 from Homo sapiens
gi|83754000 from Homo sapiens
gi|83754001 from Homo sapiens
gi|88192461 from Homo sapiens
gi|88192462 from Homo sapiens
gi|134105168 from Homo sapiens
gi|134105169 from Homo sapiens
gi|145580125 from Homo sapiens
gi|145580126 from Homo sapiens
gi|145580127 from Homo sapiens
gi|145580128 from Homo sapiens

Fixed modifications: Carbamidomethyl (C)
Variable modifications: Oxidation (M)

Cleavage by Trypsin: cuts C-term side of KR unless next residue is P
Sequence Coverage: **39%**

Matched peptides shown in **Bold Red**

1 SRKTYTLTDY LKNTYRLKLY SLR**WISDHEY** **LYK**QENNILV FNAEYGNSSV
51 FLENSTFDEF GHSINDYSIS PDGQFILLEY NYVKQWR**HSY** **TASYDIYDLN**
101 **KRQLITEER**I PNNTQWVTWS PVGHK**LAYVW** **NNDIYVKIEP** **NLPSYR**ITWT
151 GKEDIIYNGI TDWVYEEEVF SAYSALWWSP NGTFLAYAQF NDTEVPLIEY
201 SFYSDESLQY PKTVRVPYPK AGAVNPTVKF FVVNTDSLSS VTNATSIQIT
251 APASMLIGDH YLCDVTWATQ ER**ISLQWLR**R IQNYSVMDIC DYDESSGR**WN**
301 **CLVARQHIEM** **STTGWVGRFR** **PSEPHFTLDG** **NSFYKIISNE** **EGYRHICYFQ**
351 **IDKK**DCTFIT KGTWEVIGIE ALTSDYLYYI SNEYKGMPGG RNLYKIQLSD
401 YTK**VTCLSCE** **LNPERCQYYS** **VSFSK**EAKYY QLR**CSGPGLP** **LYTLHSSVND**
451 **K**GLRVLEDNS ALDKMLQNVQ MPSKKLDFII LNETKFWYQM ILPPHFDKSK
501 **KYPLLLDVYA** **GPCSQK**ADTV FR**LNWATYLA** **STENIIVASF** **DGR**GSGYQGD
551 K**IMHAINRRL** **GTFEVEDQIE** **AAR**QFSK**MGF** **VDNKR**IAIWG WSYGGYVTSM
601 VLGSGSGVFK **CGIAVAPVSR** **WEYYDSVYTE** **RYMGLPTPED** **NLDHYR**NSTV
651 MSRAENFK**QV** **EYLLIHGTAD** **DNVHFQQSAQ** **ISK**ALVDVGV DFQAMWYTDE
701 DHGIASSTAH QHIYTHMSHF IKQCFSLP

Residue Number Increasing Mass Decreasing Mass

Start - End	Observed	Mr(expt)	Mr(calc)	Delta	Miss	Sequence
24 - 33	1353.6445	1352.6372	1352.6401	-0.0028	0	**WISDHEYLYK** (No match)
88 - 102	1845.8640	1844.8567	1844.8693	-0.0126	1	**HSYTASYDIYDLNKR** (No match)
88 - 102	1845.8640	1844.8567	1844.8693	-0.0126	1	**HSYTASYDIYDLNKR** (Ions score 31)
103 - 109	888.4846	887.4773	887.4712	0.0061	0	**QLITEER** (No match)
126 - 137	1497.7673	1496.7600	1496.7663	-0.0063	0	**LAYVWNNDIYVK** (No match)
138 - 146	1088.5718	1087.5645	1087.5662	-0.0016	0	**IEPNLPSYR** (Ions score 30)
138 - 146	1088.5718	1087.5645	1087.5662	-0.0016	0	**IEPNLPSYR** (No match)
273 - 279	915.5418	914.5345	914.5338	0.0008	0	**ISLQWLR** (No match)
299 - 305	918.4668	917.4595	917.4541	0.0054	0	**WNCLVAR** (No match)
306 - 318	1501.7118	1500.7045	1500.7143	-0.0098	0	**QHIEMSTTGWVGR** (No match)
306 - 318	1517.7091	1516.7018	1516.7092	-0.0074	0	**QHIEMSTTGWVGR** Oxidation (M) (No match)
319 - 335	2041.9608	2040.9535	2040.9693	-0.0158	0	**FRPSEPHFTLDGNSFYK** (Ions score 29)
319 - 335	2041.9608	2040.9535	2040.9693	-0.0158	0	**FRPSEPHFTLDGNSFYK** (No match)
336 - 344	1080.5328	1079.5255	1079.5247	0.0009	0	**IISNEEGYR** (No match)
345 - 354	1351.6743	1350.6670	1350.6754	-0.0084	1	**HICYFQIDKK** (No match)
404 - 415	1477.6696	1476.6623	1476.6701	-0.0077	0	**VTCLSCELNPER** (No match)
416 - 425	1268.5592	1267.5519	1267.5543	-0.0024	0	**CQYYSVSFSK** (No match)
434 - 451	1944.9379	1943.9306	1943.9411	-0.0104	0	**CSGPGLPLYTLHSSVNDK** (No match)
501 - 516	1851.9546	1850.9473	1850.9600	-0.0127	1	**KYPLLLDVYAGPCSQK** (No match)
523 - 543	2341.1545	2340.1472	2340.1749	-0.0277	0	**LNWATYLASTENIIVASFDGR** (No match)
552 - 558	870.4713	869.4640	869.4541	0.0099	0	**IMHAINR** Oxidation (M) (No match)

552 - 559	1026.5570	1025.5497	1025.5552	-0.0055	1	**IMHAINRR** Oxidation (M) (No match)
559 - 573	1733.8695	1732.8622	1732.8743	-0.0121	1	**RLGTFEVEDQIEAAR** (No match)
560 - 573	1577.7699	1576.7626	1576.7732	-0.0106	0	**LGTFEVEDQIEAAR** (No match)
560 - 573	1577.7699	1576.7626	1576.7732	-0.0106	0	**LGTFEVEDQIEAAR** (Ions score 60)
578 - 585	982.4999	981.4926	981.4701	0.0225	1	**MGFVDNKR** Oxidation (M) (No match)
611 - 620	1029.5515	1028.5442	1028.5436	0.0006	0	**CGIAVAPVSR** (No match)
621 - 631	1510.6409	1509.6336	1509.6412	-0.0076	0	**WEYYDSVYTER** (No match)
621 - 631	1510.6409	1509.6336	1509.6412	-0.0076	0	**WEYYDSVYTER** (Ions score 66)
632 - 646	1820.8167	1819.8094	1819.8199	-0.0105	0	**YMGLPTPEDNLDHYR** (No match)
632 - 646	1836.8141	1835.8068	1835.8148	-0.0080	0	**YMGLPTPEDNLDHYR** Oxidation (M) (No match)
659 - 683	2841.4067	2840.3994	2840.4092	-0.0098	0	**QVEYLLIHGTADDNVHFQQSAQISK** (No match)

Mascot: http://www.matrixscience.com/

Spot 83

Protein View

Match to: **gi|50513374** Score: **296** Expect: **4.8e-025**
Chain A, Crystal Structure Of Human Dipeptidyl Peptidase Iv At 2.1 Ang. Resolution.

Nominal mass (M_r): **85693**; Calculated pI value: **5.85**
NCBI BLAST search of gi|50513374 against nr
Unformatted sequence string for pasting into other applications

Taxonomy: Homo sapiens
Links to retrieve other entries containing this sequence from NCBI Entrez:
gi|50513375 from Homo sapiens
gi|50513376 from Homo sapiens
gi|50513377 from Homo sapiens

Fixed modifications: Carbamidomethyl (C)
Variable modifications: Oxidation (M)
Cleavage by Trypsin: cuts C-term side of KR unless next residue is P
Sequence Coverage: **22%**

Matched peptides shown in **Bold Red**

```
  1 HHHHHSRKTY TLTDYLKNTY RLKLYSLRWI SDHEYLYKQE NNILVFNAEY
 51 GNSSVFLENS TFDEFGHSIN DYSISPDGQF ILLEYNYVKQ WRHSYTASYD
101 IYDLNKRQLI TEERIPNNTQ WVTWSPVGHK LAYVWNNDIY VKIEPNLPSY
151 RITWTGKEDI IYNGITDWVY EEEVFSAYSA LWWSPNGTFL AYAQFNDTEV
201 PLIEYSFYSD ESLQYPKTVR VPYPKAGAVN PTVKFFVVNT DSLSSVTNAT
251 SIQITAPASM LIGDHYLCDV TWATQERISL QWLRRIQNYS VMDICDYDES
301 SGRWNCLVAR QHIEMSTTGW VGRFRPSEPH FTLDGNSFYK IISNEEGYRH
351 ICYFQIDKKD CTFITKGTWE VIGIEALTSD YLYYISNEYK GMPGGRNLYK
401 IQLSDYTKVT CLSCELNPER CQYYSVSFSK EAKYYQLRCS GPGLPLYTLH
451 SSVNDKGLRV LEDNSALDKM LQNVQMPSKK LDFIILNETK FWYQMILPPH
501 FDKSKKYPLL LDVYAGPCSQ KADTVFRLNW ATYLASTENI IVASFDGRGS
551 GYQGDKIMHA INRRLGTFEV EDQIEAARQF SKMGFVDNKR IAIWGWSYGG
601 YVTSMVLGSG SGVFKCGIAV APVSRWEYYD SVYTERYMGL PTPEDNLDHY
651 RNSTVMSRAE NFKQVEYLLI HGTADDNVHF QQSAQISKAL VDVGVDFQAM
701 WYTDEDHGIA SSTAHQHIYT HMSHFIKQCF SLP
```

Residue Number Increasing Mass Decreasing Mass

Start - End	Observed	Mr(expt)	Mr(calc)	Delta	Miss	Sequence
1 - 7	947.5103	946.5030	946.4382	0.0648	0	HHHHHSR (No match)
22 - 28	892.5464	891.5391	891.5541	-0.0150	1	LKLYSLR (No match)
93 - 107	1845.8284	1844.8211	1844.8693	-0.0482	1	HSYTASYDIYDLNKR (No match)
93 - 107	1845.8284	1844.8211	1844.8693	-0.0482	1	HSYTASYDIYDLNKR (Ions score 30)
108 - 114	888.4606	887.4533	887.4712	-0.0179	0	QLITEER (No match)
143 - 151	1088.5522	1087.5449	1087.5662	-0.0212	0	IEPNLPSYR (No match)
143 - 151	1088.5522	1087.5449	1087.5662	-0.0212	0	IEPNLPSYR (Ions score 14)
324 - 340	2041.9244	2040.9171	2040.9693	-0.0522	0	FRPSEPHFTLDGNSFYK (No match)
324 - 340	2041.9244	2040.9171	2040.9693	-0.0522	0	FRPSEPHFTLDGNSFYK (Ions score 14)
341 - 349	1080.5159	1079.5086	1079.5247	-0.0160	0	IISNEEGYR (No match)
350 - 359	1351.6517	1350.6444	1350.6754	-0.0310	1	HICYFQIDKK (No match)
409 - 420	1477.6454	1476.6381	1476.6701	-0.0319	0	VTCLSCELNPER (No match)
506 - 521	1851.9214	1850.9141	1850.9600	-0.0459	1	KYPLLLDVYAGPCSQK (No match)
564 - 578	1733.8414	1732.8341	1732.8743	-0.0402	1	RLGTFEVEDQIEAAR (No match)
565 - 578	1577.7434	1576.7361	1576.7732	-0.0371	0	LGTFEVEDQIEAAR (No match)
565 - 578	1577.7434	1576.7361	1576.7732	-0.0371	0	LGTFEVEDQIEAAR (Ions score 52)
565 - 582	2067.9949	2066.9876	2067.0272	-0.0396	1	LGTFEVEDQIEAARQFSK (No match)
583 - 590	982.5082	981.5009	981.4701	0.0308	1	MGFVDNKR Oxidation (M) (No match)
626 - 636	1510.6136	1509.6063	1509.6412	-0.0348	0	WEYYDSVYTER (No match)
626 - 636	1510.6136	1509.6063	1509.6412	-0.0348	0	WEYYDSVYTER (Ions score 60)
637 - 651	1836.7788	1835.7715	1835.8148	-0.0433	0	YMGLPTPEDNLDHYR Oxidation (M) (No match)

Mascot: http://www.matrixscience.com/

Spot 84

Protein View

Match to: **gi|27574040** Score: **431** Expect: **1.5e-038**
Chain A, Human Dipeptidyl Peptidase IvCD26 IN COMPLEX WITH AN Inhibitor

Nominal mass (M_r): **85008**; Calculated pI value: **5.67**
NCBI BLAST search of gi|27574040 against nr
Unformatted sequence string for pasting into other applications

Taxonomy: Homo sapiens
Links to retrieve other entries containing this sequence from NCBI Entrez:
gi|27574041 from Homo sapiens
gi|34810234 from Homo sapiens
gi|34810235 from Homo sapiens
gi|34810236 from Homo sapiens
gi|34810237 from Homo sapiens
gi|50513762 from Homo sapiens
gi|50513763 from Homo sapiens
gi|50513770 from Homo sapiens
gi|50513771 from Homo sapiens
gi|55670507 from Homo sapiens
gi|55670508 from Homo sapiens
gi|55670509 from Homo sapiens
gi|55670510 from Homo sapiens
gi|58176672 from Homo sapiens
gi|58176673 from Homo sapiens
gi|60594437 from Homo sapiens
gi|60594438 from Homo sapiens
gi|60594439 from Homo sapiens
gi|60594440 from Homo sapiens
gi|83754000 from Homo sapiens
gi|83754001 from Homo sapiens
gi|88192461 from Homo sapiens
gi|88192462 from Homo sapiens
gi|134105168 from Homo sapiens
gi|134105169 from Homo sapiens
gi|145580125 from Homo sapiens
gi|145580126 from Homo sapiens
gi|145580127 from Homo sapiens
gi|145580128 from Homo sapiens

Fixed modifications: Carbamidomethyl (C)
Variable modifications: Oxidation (M)

Cleavage by Trypsin: cuts C-term side of KR unless next residue is P
Sequence Coverage: **31%**

Matched peptides shown in **Bold Red**

1 SRKTYTLTDY LKNTYRLKLY SLR**WISDHEY** **LYK**QENNILV FNAEYGNSSV
51 FLENSTFDEF GHSINDYSIS PDGQFILLEY NYVKQWR**HSY** **TASYDIYDLN**
101 **KRQLITEER**I PNNTQWVTWS PVGHK**LAYVW** **NNDIYVKIEP** **NLPSYR**ITWT
151 GKEDIIYNGI TDWVYEEEVF SAYSALWWSP NGTFLAYAQF NDTEVPLIEY
201 SFYSDESLQY PKTVRVPYPK AGAVNPTVKF FVVNTDSLSS VTNATSIQIT
251 APASMLIGDH YLCDVTWATQ ER**ISLQWLR**R IQNYSVMDIC DYDESSGR**WN**
301 **CLVARQHIEM** **STTGWVGRFR** **PSEPHFTLDG** **NSFYKIISNE** **EGYRHICYFQ**
351 **IDKK**DCTFIT KGTWEVIGIE ALTSDYLYYI SNEYKGMPGG RNLYKIQLSD
401 YTK**VTCLSCE** **LNPERCQYYS** **VSFSK**EAKYY QLRCSGPGLP LYTLHSSVND
451 KGLRVLEDNS ALDKMLQNVQ MPSKKLDFII LNETKFWYQM ILPPHFDKSK
501 **KYPLLLDVYA** **GPCSQK**ADTV FRLNWATYLA STENIIVASF DGRGSGYQGD
551 KIMHAINRR**L** **GTFEVEDQIE** **AAR**QFSKMGF VDNKRIAIWG WSYGGYVTSM
601 VLGSGSGVFK **CGIAVAPVSR** **WEYYDSVYTE** **RYMGLPTPED** **NLDHYR**NSTV
651 MSRAENFK**QV** **EYLLIHGTAD** **DNVHFQQSAQ** **ISK**ALVDVGV DFQAMWYTDE
701 DHGIASSTAH QHIYTHMSHF IKQCFSLP

Residue Number Increasing Mass Decreasing Mass

Start - End	Observed	Mr(expt)	Mr(calc)	Delta	Miss	Sequence
24 - 33	1353.6396	1352.6323	1352.6401	-0.0077	0	WISDHEYLYK (No match)
88 - 102	1845.8540	1844.8467	1844.8693	-0.0226	1	HSYTASYDIYDLNKR (No match)
88 - 102	1845.8540	1844.8467	1844.8693	-0.0226	1	HSYTASYDIYDLNKR (Ions score 30)
103 - 109	888.4722	887.4649	887.4712	-0.0063	0	QLITEER (No match)
126 - 137	1497.7491	1496.7418	1496.7663	-0.0245	0	LAYVWNNDIYVK (No match)
138 - 146	1088.5646	1087.5573	1087.5662	-0.0088	0	IEPNLPSYR (Ions score 32)
138 - 146	1088.5646	1087.5573	1087.5662	-0.0088	0	IEPNLPSYR (No match)
273 - 279	915.5336	914.5263	914.5338	-0.0074	0	ISLQWLR (No match)
299 - 305	918.4601	917.4528	917.4541	-0.0013	0	WNCLVAR (No match)
306 - 318	1501.7043	1500.6970	1500.7143	-0.0173	0	QHIEMSTTGWVGR (No match)
306 - 318	1517.6910	1516.6837	1516.7092	-0.0255	0	QHIEMSTTGWVGR Oxidation (M) (No match)
319 - 335	2041.9489	2040.9416	2040.9693	-0.0277	0	FRPSEPHFTLDGNSFYK (Ions score 32)
319 - 335	2041.9489	2040.9416	2040.9693	-0.0277	0	FRPSEPHFTLDGNSFYK (No match)
336 - 344	1080.5242	1079.5169	1079.5247	-0.0077	0	IISNEEGYR (No match)
345 - 354	1351.6617	1350.6544	1350.6754	-0.0210	1	HICYFQIDKK (No match)
404 - 415	1477.6649	1476.6576	1476.6701	-0.0124	0	VTCLSCELNPER (No match)
416 - 425	1268.5435	1267.5362	1267.5543	-0.0181	0	CQYYSVSFSK (No match)
501 - 516	1851.9583	1850.9510	1850.9600	-0.0090	1	KYPLLLDVYAGPCSQK (No match)
560 - 573	1577.7615	1576.7542	1576.7732	-0.0190	0	LGTFEVEDQIEAAR (Ions score 69)
560 - 573	1577.7615	1576.7542	1576.7732	-0.0190	0	LGTFEVEDQIEAAR (No match)
611 - 620	1029.5369	1028.5296	1028.5436	-0.0140	0	CGIAVAPVSR (No match)

621 - 631	1510.6309	1509.6236	1509.6412	-0.0176	0	WEYYDSVYTER (Ions score 73)
621 - 631	1510.6309	1509.6236	1509.6412	-0.0176	0	WEYYDSVYTER (No match)
632 - 646	1820.8071	1819.7998	1819.8199	-0.0201	0	YMGLPTPEDNLDHYR (No match)
632 - 646	1836.8058	1835.7985	1835.8148	-0.0163	0	YMGLPTPEDNLDHYR Oxidation (M) (No match)
659 - 683	2841.3694	2840.3621	2840.4092	-0.0471	0	QVEYLLIHGTADDNVHFQQSAQISK (No match)

Mascot: http://www.matrixscience.com/

Spot 85

{MATRIX SCIENCE} Mascot Search Results

Protein View

Match to: **gi|27574040** Score: **244** Expect: **7.7e-020**
Chain A, Human Dipeptidyl Peptidase IvCD26 IN COMPLEX WITH AN Inhibitor

Nominal mass (M_r): **85008**; Calculated pI value: **5.67**
NCBI BLAST search of gi|27574040 against nr
Unformatted sequence string for pasting into other applications

Taxonomy: Homo sapiens
Links to retrieve other entries containing this sequence from NCBI Entrez:
gi|27574041 from Homo sapiens
gi|34810234 from Homo sapiens
gi|34810235 from Homo sapiens
gi|34810236 from Homo sapiens
gi|34810237 from Homo sapiens
gi|50513762 from Homo sapiens
gi|50513763 from Homo sapiens
gi|50513770 from Homo sapiens
gi|50513771 from Homo sapiens
gi|55670507 from Homo sapiens
gi|55670508 from Homo sapiens
gi|55670509 from Homo sapiens
gi|55670510 from Homo sapiens
gi|58176672 from Homo sapiens
gi|58176673 from Homo sapiens
gi|60594437 from Homo sapiens
gi|60594438 from Homo sapiens
gi|60594439 from Homo sapiens
gi|60594440 from Homo sapiens
gi|83754000 from Homo sapiens
gi|83754001 from Homo sapiens
gi|88192461 from Homo sapiens
gi|88192462 from Homo sapiens
gi|134105168 from Homo sapiens
gi|134105169 from Homo sapiens
gi|145580125 from Homo sapiens
gi|145580126 from Homo sapiens
gi|145580127 from Homo sapiens
gi|145580128 from Homo sapiens

Fixed modifications: Carbamidomethyl (C)
Variable modifications: Oxidation (M)
Cleavage by Trypsin: cuts C-term side of KR unless next residue is P
Sequence Coverage: **10%**

Matched peptides shown in **Bold Red**

1 SRKTYTLTDY LKNTYRLKLY SLRWISDHEY LYKQENNILV FNAEYGNSSV
51 FLENSTFDEF GHSINDYSIS PDGQFILLEY NYVKQWR**HSY TASYDIYDLN**
101 **KR**QLITEERI PNNTQWVTWS PVGHKLAYVW NNDIYVK**IEP NLPSYR**ITWT
151 GKEDIIYNGI TDWVYEEEVF SAYSALWWSP NGTFLAYAQF NDTEVPLIEY
201 SFYSDESLQY PKTVRVPYPK AGAVNPTVKF FVVNTDSLSS VTNATSIQIT
251 APASMLIGDH YLCDVTWATQ ERISLQWLRR IQNYSVMDIC DYDESSGRWN
301 CLVARQHIEM STTGWVGR**FR PSEPHFTLDG NSFYKIISNE EGYR**HICYFQ
351 IDKKDCTFIT KGTWEVIGIE ALTSDYLYYI SNEYKGMPGG RNLYKIQLSD
401 YTKVTCLSCE LNPERCQYYS VSFSKEAKYY QLRCSGPGLP LYTLHSSVND
451 KGLRVLEDNS ALDKMLQNVQ MPSKKLDFII LNETKFWYQM ILPPHFDKSK
501 KYPLLLDVYA GPCSQKADTV FRLNWATYLA STENIIVASF DGRGSGYQGD
551 KIMHAINRR**L GTFEVEDQIE AAR**QFSKMGF VDNKRIAIWG WSYGGYVTSM
601 VLGSGSGVFK CGIAVAPVSR **WEYYDSVYTE R**YMGLPTPED NLDHYRNSTV
651 MSRAENFKQV EYLLIHGTAD DNVHFQQSAQ ISKALVDVGV DFQAMWYTDE
701 DHGIASSTAH QHIYTHMSHF IKQCFSLP

Residue Number Increasing Mass Decreasing Mass

Start - End	Observed	Mr(expt)	Mr(calc)	Delta	Miss	Sequence
88 - 102	1845.8625	1844.8552	1844.8693	-0.0141	1	HSYTASYDIYDLNKR (No match)
88 - 102	1845.8625	1844.8552	1844.8693	-0.0141	1	HSYTASYDIYDLNKR (Ions score 62)
138 - 146	1088.5682	1087.5609	1087.5662	-0.0052	0	IEPNLPSYR (Ions score 9)
138 - 146	1088.5682	1087.5609	1087.5662	-0.0052	0	IEPNLPSYR (No match)
319 - 335	2041.9601	2040.9528	2040.9693	-0.0165	0	FRPSEPHFTLDGNSFYK (No match)
319 - 335	2041.9601	2040.9528	2040.9693	-0.0165	0	FRPSEPHFTLDGNSFYK (Ions score 11)
336 - 344	1080.5249	1079.5176	1079.5247	-0.0070	0	IISNEEGYR (No match)
560 - 573	1577.7720	1576.7647	1576.7732	-0.0085	0	LGTFEVEDQIEAAR (No match)
560 - 573	1577.7720	1576.7647	1576.7732	-0.0085	0	LGTFEVEDQIEAAR (Ions score 54)
621 - 631	1510.6422	1509.6349	1509.6412	-0.0063	0	WEYYDSVYTER (Ions score 38)
621 - 631	1510.6422	1509.6349	1509.6412	-0.0063	0	WEYYDSVYTER (No match)

Spot 86

Mascot Search Results

Protein View

Match to: **gi|110590190** Score: **185** Expect: **6.1e-014**
Chain A, Crystal Structure Of Human Dipeptidyl Peptidase Iv (Dppiv) Complexed With Cyanopyrrolidine

Nominal mass (M_r): **84797**; Calculated pI value: **5.67**
NCBI BLAST search of gi|110590190 against nr
Unformatted sequence string for pasting into other applications

Taxonomy: Homo sapiens
Links to retrieve other entries containing this sequence from NCBI Entrez:
gi|110590191 from Homo sapiens
gi|110590193 from Homo sapiens
gi|110590194 from Homo sapiens
gi|110590195 from Homo sapiens
gi|110590196 from Homo sapiens
gi|110590197 from Homo sapiens
gi|110590198 from Homo sapiens
gi|122920491 from Homo sapiens
gi|122920492 from Homo sapiens
gi|122920493 from Homo sapiens
gi|122920494 from Homo sapiens
gi|146387034 from Homo sapiens
gi|146387035 from Homo sapiens
gi|158428685 (no taxonomy information for this entry)
gi|158428686 (no taxonomy information for this entry)
gi|158428687 (no taxonomy information for this entry)
gi|158428688 (no taxonomy information for this entry)
gi|158429211 (no taxonomy information for this entry)
gi|158429212 (no taxonomy information for this entry)
gi|158429213 (no taxonomy information for this entry)
gi|158429214 (no taxonomy information for this entry)

Fixed modifications: Carbamidomethyl (C)
Variable modifications: Oxidation (M)
Cleavage by Trypsin: cuts C-term side of KR unless next residue is P
Sequence Coverage: **16%**

Matched peptides shown in **Bold Red**

```
  1 SRKTYTLTDY LKNTYRLKLY SLRWISDHEY LYKQENNILV FNAEYGNSSV
 51 FLENSTFDEF GHSINDYSIS PDGQFILLEY NYVKQWRHSY TASYDIYDLN
101 KRQLITEERI PNNTQWVTWS PVGHKLAYVW NNDIYVKIEP NLPSYRITWT
151 GKEDIIYNGI TDWVYEEEVF SAYSALWWSP NGTFLAYAQF NDTEVPLIEY
201 SFYSDESLQY PKTVRVPYPK AGAVNPTVKF FVVNTDSLSS VTNATSIQIT
251 APASMLIGDH YLCDVTWATQ ERISLQWLRR IQNYSVMDIC DYDESSGRWN
301 CLVARQHIEM STTGWVGRFR PSEPHFTLDG NSFYKIISNE EGYRHICYFQ
351 IDKKDCTFIT KGTWEVIGIE ALTSDYLYYI SNEYKGMPGG RNLYKIQLSD
401 YTKVTCLSCE LNPERCQYYS VSFSKEAKYY QLRCSGPGLP LYTLHSSVND
451 KGLRVLEDNS ALDKMLQNVQ MPSKKLDFII LNETKFWYQM ILPPHFDKSK
501 KYPLLLDVYA GPCSQKADTV FRLNWATYLA STENIIVASF DGRGSGYQGD
551 KIMHAINRRL GTFEVEDQIE AARQFSKMGF VDNKRIAIWG WSYGGYVTSM
601 VLGSGSGVFK CGIAVAPVSR WEYYDSVYTE RYMGLPTPED NLDHYRNSTV
651 MSRAENFKQV EYLLIHGTAD DNVHFQQSAQ ISKALVDVGV DFQAMWYTDE
701 DHGIASSTAH QHIYTHMSHF IKQCFS
```

Residue Number Increasing Mass Decreasing Mass

Start - End	Observed	Mr(expt)	Mr(calc)	Delta	Miss	Sequence
17 - 23	892.5600	891.5527	891.5541	-0.0014	1	LKLYSLR (No match)
88 - 102	1845.8683	1844.8610	1844.8693	-0.0083	1	HSYTASYDIYDLNKR (No match)
88 - 102	1845.8683	1844.8610	1844.8693	-0.0083	1	HSYTASYDIYDLNKR (Ions score 44)
138 - 146	1088.5771	1087.5698	1087.5662	0.0037	0	IEPNLPSYR (No match)
138 - 146	1088.5771	1087.5698	1087.5662	0.0037	0	IEPNLPSYR (Ions score 6)
273 - 279	915.5484	914.5411	914.5338	0.0074	0	ISLQWLR (No match)
319 - 335	2041.9648	2040.9575	2040.9693	-0.0118	0	FRPSEPHFTLDGNSFYK (Ions score 18)
319 - 335	2041.9648	2040.9575	2040.9693	-0.0118	0	FRPSEPHFTLDGNSFYK (No match)
336 - 344	1080.5365	1079.5292	1079.5247	0.0046	0	IISNEEGYR (No match)
336 - 344	1080.5365	1079.5292	1079.5247	0.0046	0	IISNEEGYR (No match)
404 - 415	1477.6783	1476.6710	1476.6701	0.0010	0	VTCLSCELNPER (No match)
434 - 451	1944.9606	1943.9533	1943.9411	0.0123	0	CSGPGLPLYTLHSSVNDK (No match)
559 - 573	1733.8749	1732.8676	1732.8743	-0.0067	1	RLGTFEVEDQIEAAR (No match)
560 - 573	1577.7737	1576.7664	1576.7732	-0.0068	0	LGTFEVEDQIEAAR (Ions score 30)
560 - 573	1577.7737	1576.7664	1576.7732	-0.0068	0	LGTFEVEDQIEAAR (No match)
621 - 631	1510.6471	1509.6398	1509.6412	-0.0014	0	WEYYDSVYTER (No match)

Mascot: http://www.matrixscience.com/

Spot 87

{MATRIX SCIENCE} Mascot Search Results

Protein View

Match to: **gi|27574040** Score: **70** Expect: **0.019**
Chain A, Human Dipeptidyl Peptidase IvCD26 IN COMPLEX WITH AN Inhibitor

Nominal mass (M_r): **85008**; Calculated pI value: **5.67**
NCBI BLAST search of gi|27574040 against nr
Unformatted sequence string for pasting into other applications

Taxonomy: Homo sapiens
Links to retrieve other entries containing this sequence from NCBI Entrez:
gi|27574041 from Homo sapiens
gi|34810234 from Homo sapiens
gi|34810235 from Homo sapiens
gi|34810236 from Homo sapiens
gi|34810237 from Homo sapiens
gi|50513762 from Homo sapiens
gi|50513763 from Homo sapiens
gi|50513770 from Homo sapiens
gi|50513771 from Homo sapiens
gi|55670507 from Homo sapiens
gi|55670508 from Homo sapiens
gi|55670509 from Homo sapiens
gi|55670510 from Homo sapiens
gi|58176672 from Homo sapiens
gi|58176673 from Homo sapiens
gi|60594437 from Homo sapiens
gi|60594438 from Homo sapiens
gi|60594439 from Homo sapiens
gi|60594440 from Homo sapiens
gi|83754000 from Homo sapiens
gi|83754001 from Homo sapiens
gi|88192461 from Homo sapiens
gi|88192462 from Homo sapiens
gi|134105168 from Homo sapiens
gi|134105169 from Homo sapiens
gi|145580125 from Homo sapiens
gi|145580126 from Homo sapiens
gi|145580127 from Homo sapiens
gi|145580128 from Homo sapiens

Fixed modifications: Carbamidomethyl (C)
Variable modifications: Oxidation (M)
Cleavage by Trypsin: cuts C-term side of KR unless next residue is P
Sequence Coverage: **4%**

Matched peptides shown in **Bold Red**

1 SRKTYTLTDY LKNTYRLKLY SLRWISDHEY LYKQENNILV FNAEYGNSSV

51 FLENSTFDEF GHSINDYSIS PDGQFILLEY NYVKQWR**HSY TASYDIYDLN**

101 **KR**QLITEERI PNNTQWVTWS PVGHKLAYVW NNDIYVKIEP NLPSYRITWT

151 GKEDIIYNGI TDWVYEEEVF SAYSALWWSP NGTFLAYAQF NDTEVPLIEY

201 SFYSDESLQY PKTVRVPYPK AGAVNPTVKF FVVNTDSLSS VTNATSIQIT

251 APASMLIGDH YLCDVTWATQ ERISLQWLRR IQNYSVMDIC DYDESSGRWN

301 CLVARQHIEM STTGWVGR**FR PSEPHFTLDG NSFYK**IISNE EGYRHICYFQ

351 IDKKDCTFIT KGTWEVIGIE ALTSDYLYYI SNEYKGMPGG RNLYKIQLSD

401 YTKVTCLSCE LNPERCQYYS VSFSKEAKYY QLRCSGPGLP LYTLHSSVND

451 KGLRVLEDNS ALDKMLQNVQ MPSKKLDFII LNETKFWYQM ILPPHFDKSK

501 KYPLLLDVYA GPCSQKADTV FRLNWATYLA STENIIVASF DGRGSGYQGD

551 KIMHAINRRL GTFEVEDQIE AARQFSKMGF VDNKRIAIWG WSYGGYVTSM

601 VLGSGSGVFK CGIAVAPVSR WEYYDSVYTE RYMGLPTPED NLDHYRNSTV

651 MSRAENFKQV EYLLIHGTAD DNVHFQQSAQ ISKALVDVGV DFQAMWYTDE

701 DHGIASSTAH QHIYTHMSHF IKQCFSLP

Residue Number Increasing Mass Decreasing Mass

Start - End	Observed	Mr(expt)	Mr(calc)	Delta	Miss	Sequence	
88 - 102	1845.8584	1844.8511	1844.8693	-0.0182	1	**HSYTASYDIYDLNKR**	(No match)
88 - 102	1845.8584	1844.8511	1844.8693	-0.0182	1	**HSYTASYDIYDLNKR**	(Ions score 32)
319 - 335	2041.9570	2040.9497	2040.9693	-0.0196	0	**FRPSEPHFTLDGNSFYK**	(No match)
319 - 335	2041.9570	2040.9497	2040.9693	-0.0196	0	**FRPSEPHFTLDGNSFYK**	(Ions score 14)

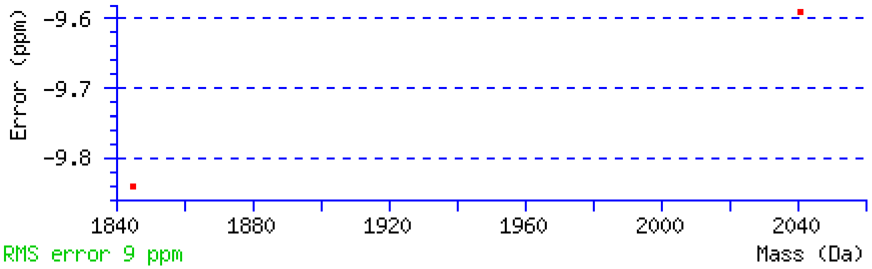

Spot 88

Mascot Search Results

Protein View

Match to: **gi|27574040** Score: **144** Expect: **7.7e-010**
Chain A, Human Dipeptidyl Peptidase IvCD26 IN COMPLEX WITH AN Inhibitor

Nominal mass (M_r): **85008**; Calculated pI value: **5.67**
NCBI BLAST search of gi|27574040 against nr
Unformatted sequence string for pasting into other applications

Taxonomy: Homo sapiens
Links to retrieve other entries containing this sequence from NCBI Entrez:
gi|27574041 from Homo sapiens
gi|34810234 from Homo sapiens
gi|34810235 from Homo sapiens
gi|34810236 from Homo sapiens
gi|34810237 from Homo sapiens
gi|50513762 from Homo sapiens
gi|50513763 from Homo sapiens
gi|50513770 from Homo sapiens
gi|50513771 from Homo sapiens
gi|55670507 from Homo sapiens
gi|55670508 from Homo sapiens
gi|55670509 from Homo sapiens
gi|55670510 from Homo sapiens
gi|58176672 from Homo sapiens
gi|58176673 from Homo sapiens
gi|60594437 from Homo sapiens
gi|60594438 from Homo sapiens
gi|60594439 from Homo sapiens
gi|60594440 from Homo sapiens
gi|83754000 from Homo sapiens
gi|83754001 from Homo sapiens
gi|88192461 from Homo sapiens
gi|88192462 from Homo sapiens
gi|134105168 from Homo sapiens
gi|134105169 from Homo sapiens
gi|145580125 from Homo sapiens
gi|145580126 from Homo sapiens
gi|145580127 from Homo sapiens
gi|145580128 from Homo sapiens

Fixed modifications: Carbamidomethyl (C)
Variable modifications: Oxidation (M)
Cleavage by Trypsin: cuts C-term side of KR unless next residue is P
Sequence Coverage: **5%**

Matched peptides shown in **Bold Red**

```
  1 SRKTYTLTDY LKNTYRLKLY SLRWISDHEY LYKQENNILV FNAEYGNSSV
 51 FLENSTFDEF GHSINDYSIS PDGQFILLEY NYVKQWRHSY TASYDIYDLN
101 KRQLITEERI PNNTQWVTWS PVGHKLAYVW NNDIYVKIEP NLPSYRITWT
151 GKEDIIYNGI TDWVYEEEVF SAYSALWWSP NGTFLAYAQF NDTEVPLIEY
201 SFYSDESLQY PKTVRVPYPK AGAVNPTVKF FVVNTDSLSS VTNATSIQIT
251 APASMLIGDH YLCDVTWATQ ERISLQWLRR IQNYSVMDIC DYDESSGRWN
301 CLVARQHIEM STTGWVGRFR PSEPHFTLDG NSFYKIISNE EGYRHICYFQ
351 IDKKDCTFIT KGTWEVIGIE ALTSDYLYYI SNEYKGMPGG RNLYKIQLSD
401 YTKVTCLSCE LNPERCQYYS VSFSKEAKYY QLRCSGPGLP LYTLHSSVND
451 KGLRVLEDNS ALDKMLQNVQ MPSKKLDFII LNETKFWYQM ILPPHFDKSK
501 KYPLLLDVYA GPCSQKADTV FRLNWATYLA STENIIVASF DGRGSGYQGD
551 KIMHAINRRL GTFEVEDQIE AARQFSKMGF VDNKRIAIWG WSYGGYVTSM
601 VLGSGSGVFK CGIAVAPVSR WEYYDSVYTE RYMGLPTPED NLDHYRNSTV
651 MSRAENFKQV EYLLIHGTAD DNVHFQQSAQ ISKALVDVGV DFQAMWYTDE
701 DHGIASSTAH QHIYTHMSHF IKQCFSLP
```

Residue Number Increasing Mass Decreasing Mass

Start - End	Observed	Mr(expt)	Mr(calc)	Delta	Miss	Sequence
88 - 102	1845.8712	1844.8639	1844.8693	-0.0054	1	**HSYTASYDIYDLNKR** (No match)
88 - 102	1845.8712	1844.8639	1844.8693	-0.0054	1	**HSYTASYDIYDLNKR** (Ions score 46)
319 - 335	2041.9670	2040.9597	2040.9693	-0.0096	0	**FRPSEPHFTLDGNSFYK** (No match)
319 - 335	2041.9670	2040.9597	2040.9693	-0.0096	0	**FRPSEPHFTLDGNSFYK** (Ions score 17)
621 - 631	1510.6454	1509.6381	1509.6412	-0.0031	0	**WEYYDSVYTER** (Ions score 53)
621 - 631	1510.6454	1509.6381	1509.6412	-0.0031	0	**WEYYDSVYTER** (No match)

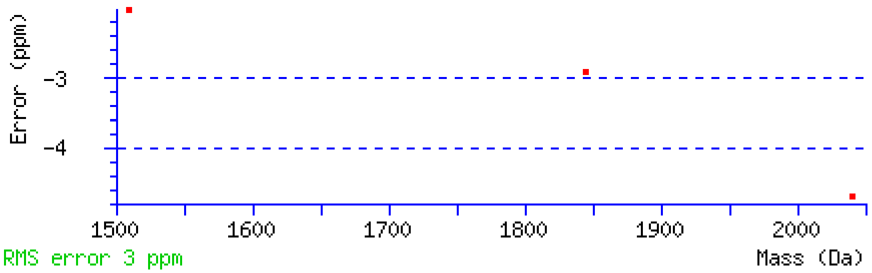

Spot 89

Protein View

Match to: **gi|51555766** Score: **146** Expect: **4.8e-010**
putative protein product of HKMT1098 [Homo sapiens]

Nominal mass (M_r): **68157;** Calculated pI value: **5.38**
NCBI BLAST search of gi|51555766 against nr
Unformatted sequence string for pasting into other applications

Taxonomy: Homo sapiens

Fixed modifications: Carbamidomethyl (C)
Variable modifications: Oxidation (M)
Cleavage by Trypsin: cuts C-term side of KR unless next residue is P
Sequence Coverage: **12%**

Matched peptides shown in **Bold Red**

```
  1 MATIPDWKLQ LLARRRQEEA SVRGREKAER ERLSQMPAWK RGLLERRRAK
 51 LGLSPGEPSP VLGTVEAGPP DPDESAVLLE AIGPVHQNRF IRQERQQQQQ
101 QQQRSEELLA ERKPGPLEAR ERRPSPGEMR DQSPKGRESR EERLSPRETR
151 ERRLGIGGAQ ELSLRPLEAR DWRQSPGEVG DRSSRLSEAW KWRLSPGETP
201 ERSLRLAESR EQSPRRKEVE SRLSPGESAY QKLGLTEAHK WRPDSRESQE
251 QSLVQLEATE WRLRSGEERQ DYSEECGRKE EWPVPGVAPK ETAELSETLT
301 REAQGNGSAG VEAAEQRPVE DGERGMKPTE GWKWTLNSGK AREWTPRDIE
351 AQTQKPEPPE SAEKLLESPG VEAGEGEAEK EEAGAQGRPL RALQNCCSVP
401 SPLPPEDAGT GGLRQQEEEA VELQPPPPAP LSPPPPAPTA PQPPGDPLMS
451 RLFYGVKAGP GVGAPRRSGH TFTVNPRRSV PPATPATPTS PATVDAAVPG
501 AGKKRYPTAE EILVLGGYLR LSRSCLAKGS PERHHKQLKI SFSETALETT
551 YQYPSESSVL EELGPEPEVP SAPNPPAAQP DDEEDEEELL LLQPELQGGL
601 RTKALIVDES CRR
```

Residue Number Increasing Mass Decreasing Mass

Start - End	Observed	Mr(expt)	Mr(calc)	Delta	Miss	Sequence
96 - 104	1199.6190	1198.6117	1198.5803	0.0314	0	QQQQQQQQR (No match)
96 - 104	1199.6190	1198.6117	1198.5803	0.0314	0	QQQQQQQQR (Ions score 12)
154 - 170	1780.0330	1779.0257	1779.0002	0.0255	0	LGIGGAQELSLRPLEAR (Ions score 3)
154 - 170	1780.0330	1779.0257	1779.0002	0.0255	0	LGIGGAQELSLRPLEAR (No match)
392 - 414	2396.1482	2395.1409	2395.1260	0.0150	0	ALQNCCSVPSPLPPEDAGTGGLR (No match)

392 - 414	2396.1482	2395.1409	2395.1260	0.0150	0	ALQNCCSVPSPLPPEDAGTGGLR	(Ions score 60)
468 - 478	1271.6898	1270.6825	1270.6530	0.0295	1	SGHTFTVNPRR	(No match)
505 - 520	1850.0529	1849.0456	1849.0097	0.0359	1	RYPTAEEILVLGGYLR	(No match)
506 - 520	1693.9382	1692.9309	1692.9086	0.0223	0	YPTAEEILVLGGYLR	(No match)
506 - 520	1693.9382	1692.9309	1692.9086	0.0223	0	YPTAEEILVLGGYLR	(Ions score 10)

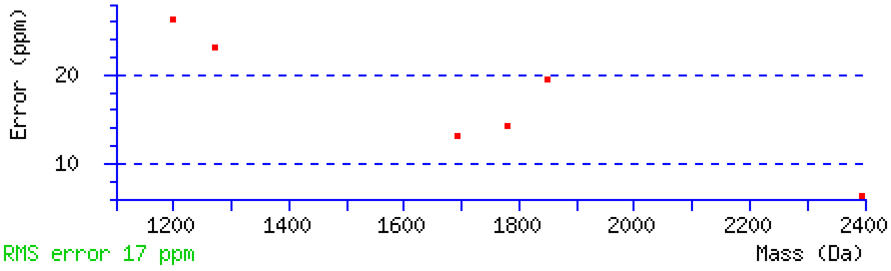

Spot 90

Mascot Search Results

Protein View

Match to: **gi|51555766** Score: **185** Expect: **6.1e-014**
putative protein product of HKMT1098 [Homo sapiens]

Nominal mass (M_r): **68157**; Calculated pI value: **5.38**
NCBI BLAST search of gi|51555766 against nr
Unformatted sequence string for pasting into other applications

Taxonomy: Homo sapiens

Fixed modifications: Carbamidomethyl (C)
Variable modifications: Oxidation (M)
Cleavage by Trypsin: cuts C-term side of KR unless next residue is P
Sequence Coverage: **15%**

Matched peptides shown in **Bold Red**

```
  1 MATIPDWKLQ LLARRRQEEA SVRGREKAER ERLSQMPAWK RGLLERRRAK
 51 LGLSPGEPSP VLGTVEAGPP DPDESAVLLE AIGPVHQNRF IRQERQQQQQ
101 QQQRSEELLA ERKPGPLEAR ERRPSPGEMR DQSPKGRESR EERLSPRETR
151 ERRLGIGGAQ ELSLRPLEAR DWRQSPGEVG DRSSRLSEAW KWRLSPGETP
201 ERSLRLAESR EQSPRRKEVE SRLSPGESAY QKLGLTEAHK WRPDSRESQE
251 QSLVQLEATE WRLRSGEERQ DYSEECGRKE EWPVPGVAPK ETAELSETLT
301 REAQGNGSAG VEAAEQRPVE DGERGMKPTE GWKWTLNSGK AREWTPRDIE
351 AQTQKPEPPE SAEKLLESPG VEAGEGEAEK EEAGAQGRPL RALQNCCSVP
401 SPLPPEDAGT GGLRQQEEEA VELQPPPPAP LSPPPPAPTA PQPPGDPLMS
451 RLFYGVKAGP GVGAPRRSGH TFTVNPRRSV PPATPATPTS PATVDAAVPG
501 AGKKRYPTAE EILVLGGYLR LSRSCLAKGS PERHHKQLKI SFSETALETT
551 YQYPSESSVL EELGPEPEVP SAPNPPAAQP DDEEDEEELL LLQPELQGGL
601 RTKALIVDES CRR
```

Residue Number Increasing Mass Decreasing Mass

Start - End	Observed	Mr(expt)	Mr(calc)	Delta	Miss	Sequence
90 - 95	848.5178	847.5105	847.4664	0.0441	1	FIRQER (No match)
96 - 104	1199.5839	1198.5766	1198.5803	-0.0037	0	QQQQQQQQR (No match)

96 - 104	1199.5839	1198.5766	1198.5803	-0.0037	0	QQQQQQQQR (Ions score 19)
154 - 170	1779.9993	1778.9920	1779.0002	-0.0082	0	LGIGGAQELSLRPLEAR (Ions score 19)
154 - 170	1779.9993	1778.9920	1779.0002	-0.0082	0	LGIGGAQELSLRPLEAR (No match)
247 - 262	1932.9183	1931.9110	1931.9224	-0.0114	0	ESQEQSLVQLEATEWR (No match)
392 - 414	2396.1160	2395.1087	2395.1260	-0.0172	0	ALQNCCSVPSPLPPEDAGTGGLR (No match)
392 - 414	2396.1160	2395.1087	2395.1260	-0.0172	0	ALQNCCSVPSPLPPEDAGTGGLR (Ions score 36)
468 - 477	1115.5559	1114.5486	1114.5519	-0.0033	0	SGHTFTVNPR (No match)
468 - 478	1271.6475	1270.6402	1270.6530	-0.0128	1	SGHTFTVNPRR (No match)
505 - 520	1849.9979	1848.9906	1849.0097	-0.0191	1	RYPTAEEILVLGGYLR (No match)
506 - 520	1693.9026	1692.8953	1692.9086	-0.0133	0	YPTAEEILVLGGYLR (Ions score 24)
506 - 520	1693.9026	1692.8953	1692.9086	-0.0133	0	YPTAEEILVLGGYLR (No match)

Mascot: http://www.matrixscience.com/

Spot 91

Mascot Search Results

Protein View

Match to: **gi|123209015** Score: **318** Expect: **3e-027**
KIAA1949 [Homo sapiens]

Nominal mass (M_r): **68286;** Calculated pI value: **5.42**
NCBI BLAST search of gi|123209015 against nr
Unformatted sequence string for pasting into other applications

Taxonomy: Homo sapiens

Fixed modifications: Carbamidomethyl (C)
Variable modifications: Oxidation (M)
Cleavage by Trypsin: cuts C-term side of KR unless next residue is P
Sequence Coverage: **26%**

Matched peptides shown in **Bold Red**

```
  1 MATIPDWKLQ LLARRRQEEA SVRGREKAER ERLSQMPAWK RGLLERRRAK
 51 LGLSPGEPSP VLGTVEAGPP DPDESAVLLE AIGPVHQNRF IRQERQQQQQ
101 QQQRSEELLA ERKPGPLEAR ERRPSPGEMR DQSPKGRESR EERLSPRETR
151 ERRLGIGGAQ ELSLRPLEAR DWRQSPGEVG DRSSRLSEAW KWRLSPGETP
201 ERSLRLAESR EQSPRRKEVE SRLSPGESAY QKLGLTEAHK WRPDSRESQE
251 QSLVQLEATE WRLRSGEERQ DYSEECGRKE EWPVPGVAPK ETAELSETLT
301 REAQGNSSAG VEAAEQRPVE DGERGMKPTE GWKWTLNSRK AREWTPRDIE
351 AQTQKPEPPE SAEKLLESPG VEAGEGEAEK EEAGAQGRPL RALQNCCSVP
401 SPLPPEDAGT GGLRQQEEEA VELQPPPPAP LSPPPPAPTA PQPPGDPLMS
451 RLFYGVKAGP GVGAPRRSGH TFTVNPRRSV PPATPATPTS PATVDAAVPG
501 AGKKRYPTAE EILVLGGYLR LSRSCLAKGS PERHHKQLKI SFSETALETT
551 YQYPSESSVL EELGPEPEVP SAPNPPAAQP DDEEDEEELL LLQPELQGGL
601 RTKALIVDES CRR
```

Residue Number Increasing Mass Decreasing Mass

Start - End	Observed	Mr(expt)	Mr(calc)	Delta	Miss	Sequence	
96 - 104	1199.5796	1198.5723	1198.5803	-0.0080	0	QQQQQQQQR	(No match)
96 - 104	1199.5796	1198.5723	1198.5803	-0.0080	0	QQQQQQQQR	(Ions score 38)

113 - 120	867.5047	866.4974	866.4973	0.0001	0	KPGPLEAR (No match)
154 - 170	1779.9810	1778.9737	1779.0002	-0.0265	0	LGIGGAQELSLRPLEAR (Ions score 28)
154 - 170	1779.9810	1778.9737	1779.0002	-0.0265	0	LGIGGAQELSLRPLEAR (No match)
241 - 246	816.4048	815.3975	815.4038	-0.0063	0	WRPDSR (No match)
247 - 262	1932.9087	1931.9014	1931.9224	-0.0210	0	ESQEQSLVQLEATEWR (No match)
302 - 324	2386.0635	2385.0562	2385.0791	-0.0229	0	EAQGNSSAGVEAAEQRPVEDGER (No match)
348 - 364	1896.9038	1895.8965	1895.9112	-0.0147	0	DIEAQTQKPEPPESAEK (No match)
392 - 414	2396.0979	2395.0906	2395.1260	-0.0353	0	ALQNCCSVPSPLPPEDAGTGGLR (No match)
458 - 466	781.4337	780.4264	780.4242	0.0023	0	AGPGVGAPR (No match)
468 - 477	1115.5481	1114.5408	1114.5519	-0.0111	0	SGHTFTVNPR (No match)
468 - 477	1115.5481	1114.5408	1114.5519	-0.0111	0	SGHTFTVNPR (Ions score 20)
468 - 478	1271.6381	1270.6308	1270.6530	-0.0222	1	SGHTFTVNPRR (No match)
506 - 520	1693.8906	1692.8833	1692.9086	-0.0253	0	YPTAEEILVLGGYLR (Ions score 89)
506 - 520	1693.8906	1692.8833	1692.9086	-0.0253	0	YPTAEEILVLGGYLR (No match)
604 - 613	1218.6100	1217.6027	1217.6186	-0.0159	1	ALIVDESCRR (No match)

Mascot: http://www.matrixscience.com/

Spot 92

{MATRIX SCIENCE} Mascot Search Results

Protein View

Match to: **gi|123209015** Score: **264** Expect: **7.7e-022**
KIAA1949 [Homo sapiens]

Nominal mass (M_r): **68286**; Calculated pI value: **5.42**
NCBI BLAST search of gi|123209015 against nr
Unformatted sequence string for pasting into other applications

Taxonomy: Homo sapiens

Fixed modifications: Carbamidomethyl (C)
Variable modifications: Oxidation (M)
Cleavage by Trypsin: cuts C-term side of KR unless next residue is P
Sequence Coverage: **21%**

Matched peptides shown in **Bold Red**

```
  1 MATIPDWKLQ LLARRRQEEA SVRGREKAER ERLSQMPAWK RGLLERRRAK
 51 LGLSPGEPSP VLGTVEAGPP DPDESAVLLE AIGPVHQNRF IRQERQQQQQ
101 QQQRSEELLA ERKPGPLEAR ERRPSPGEMR DQSPKGRESR EERLSPRETR
151 ERRLGIGGAQ ELSLRPLEAR DWRQSPGEVG DRSSRLSEAW KWRLSPGETP
201 ERSLRLAESR EQSPRRKEVE SRLSPGESAY QKLGLTEAHK WRPDSRESQE
251 QSLVQLEATE WRLRSGEERQ DYSEECGRKE EWPVPGVAPK ETAELSETLT
301 REAQGNSSAG VEAAEQRPVE DGERGMKPTE GWKWTLNSRK AREWTPRDIE
351 AQTQKPEPPE SAEKLLESPG VEAGEGEAEK EEAGAQGRPL RALQNCCSVP
401 SPLPPEDAGT GGLRQQEEEA VELQPPPPAP LSPPPPAPTA PQPPGDPLMS
451 RLFYGVKAGP GVGAPRRSGH TFTVNPRRSV PPATPATPTS PATVDAAVPG
501 AGKKRYPTAE EILVLGGYLR LSRSCLAKGS PERHHKQLKI SFSETALETT
551 YQYPSESSVL EELGPEPEVP SAPNPPAAQP DDEEDEEELL LLQPELQGGL
601 RTKALIVDES CRR
```

Residue Number Increasing Mass Decreasing Mass

Start - End	Observed	Mr(expt)	Mr(calc)	Delta	Miss	Sequence	
96 - 104	1199.5637	1198.5564	1198.5803	-0.0239	0	QQQQQQQQR	(No match)
96 - 104	1199.5637	1198.5564	1198.5803	-0.0239	0	QQQQQQQQR	(Ions score 33)

154 - 170	1779.9612	1778.9539	1779.0002	-0.0463	0	LGIGGAQELSLRPLEAR	(No match)
154 - 170	1779.9612	1778.9539	1779.0002	-0.0463	0	LGIGGAQELSLRPLEAR	(Ions score 36)
247 - 262	1932.8918	1931.8845	1931.9224	-0.0379	0	ESQEQSLVQLEATEWR	(No match)
302 - 324	2386.0237	2385.0164	2385.0791	-0.0627	0	EAQGNSSAGVEAAEQRPVEDGER	(No match)
348 - 364	1896.8730	1895.8657	1895.9112	-0.0455	0	DIEAQTQKPEPPESAEK	(No match)
392 - 414	2396.0759	2395.0686	2395.1260	-0.0573	0	ALQNCCSVPSPLPPEDAGTGGLR	(No match)
392 - 414	2396.0759	2395.0686	2395.1260	-0.0573	0	ALQNCCSVPSPLPPEDAGTGGLR	(Ions score 51)
468 - 477	1115.5320	1114.5247	1114.5519	-0.0272	0	SGHTFTVNPR	(No match)
505 - 520	1849.9640	1848.9567	1849.0097	-0.0530	1	RYPTAEEILVLGGYLR	(No match)
506 - 520	1693.8759	1692.8686	1692.9086	-0.0400	0	YPTAEEILVLGGYLR	(Ions score 51)
506 - 520	1693.8759	1692.8686	1692.9086	-0.0400	0	YPTAEEILVLGGYLR	(No match)

Mascot: http://www.matrixscience.com/

Spot 93

{MATRIX SCIENCE} **Mascot Search Results**

Protein View

Match to: **gi|1002923** Score: **183** Expect: **9.6e-014**
coronin-like protein

Nominal mass (M_r): **51722**; Calculated pI value: **6.12**
NCBI BLAST search of gi|1002923 against nr
Unformatted sequence string for pasting into other applications

Taxonomy: Homo sapiens

Fixed modifications: Carbamidomethyl (C)
Variable modifications: Oxidation (M)
Cleavage by Trypsin: cuts C-term side of KR unless next residue is P
Sequence Coverage: **30%**

Matched peptides shown in **Bold Red**

```
  1 MSRQVVRTSK FRHVFGQPAK ADQCYEDVRV SQTTWDSGFC AVNPKFVALI
 51 CEASGGGAFL VLPLGKTGRV DKNAPTVCGH TAPVLDIAWC PHNDNVIASG
101 SEDCTVMVWE IPDGGLMLPL REPVVTLEGH TKRVGIVAWH TTAQNVLLSA
151 GCDNVIMVWD VGTGAAMLTL GPEVHPDTIY SVDWSRDGGL ICTSCRDKRV
201 RIIEPRKGTV VAEKDRPHEG TRPVRAVFVS EGKILTTGFS RMSEWQVALW
251 DTKHLEEPLS LQELDTSSGV LLPFFDPDTN IVYLCGKGDS SIRYFEITSE
301 APFLHYLSMF SSKESQRGMG YMPKRGLEVN KCEIARFYKL HERRCEPIAM
351 TVPRKSDLFQ EDLYPPTAGP DPALTAEEWL GGRDAGPLLI SLKDGYVPPK
401 SRELRVNRGL DTGRRRAAPE ASGTPSSDAV SRLEEEMRKL QATVQELQKR
451 LDRLEETVQA K
```

Residue Number Increasing Mass Decreasing Mass

Start - End	Observed	Mr(expt)	Mr(calc)	Delta	Miss	Sequence
11 - 20	1186.6503	1185.6430	1185.6406	0.0024	1	FRHVFGQPAK (No match)
21 - 29	1155.4783	1154.4710	1154.4662	0.0048	0	ADQCYEDVR (No match)
21 - 29	1155.4783	1154.4710	1154.4662	0.0048	0	ADQCYEDVR (Ions score 14)
30 - 45	1796.8362	1795.8289	1795.8199	0.0090	0	VSQTTWDSGFCAVNPK (No match)
187 - 196	1138.5056	1137.4983	1137.4906	0.0077	0	DGGLICTSCR (Ions score 12)

187 - 196	1138.5056	1137.4983	1137.4906	0.0077	0	DGGLICTSCR (No match)
215 - 225	1319.6918	1318.6845	1318.6854	-0.0009	0	DRPHEGTRPVR (Ions score 26)
215 - 225	1319.6918	1318.6845	1318.6854	-0.0009	0	DRPHEGTRPVR (No match)
226 - 241	1711.9291	1710.9218	1710.9304	-0.0086	1	AVFVSEGKILTTGFSR (No match)
234 - 241	894.5117	893.5044	893.4970	0.0074	0	ILTTGFSR (No match)
294 - 313	2413.1997	2412.1924	2412.1347	0.0577	0	YFEITSEAPFLHYLSMFSSK Oxidation (M) (No match)
344 - 354	1329.6759	1328.6686	1328.6693	-0.0007	1	RCEPIAMTVPR (No match)
344 - 354	1345.6703	1344.6630	1344.6642	-0.0012	1	RCEPIAMTVPR Oxidation (M) (No match)
345 - 354	1189.5729	1188.5656	1188.5631	0.0025	0	CEPIAMTVPR Oxidation (M) (No match)
355 - 383	3173.5305	3172.5232	3172.5352	-0.0120	1	KSDLFQEDLYPPTAGPDPALTAEEWLGGR (No match)
384 - 393	1026.6210	1025.6137	1025.6120	0.0017	0	DAGPLLISLK (No match)

Mascot: http://www.matrixscience.com/

Spot 94

Mascot Search Results

Protein View

Match to: **gi|38569421** Score: **99** Expect: **2.7e-005**
ATP citrate lyase isoform 1 [Homo sapiens]

Nominal mass (M_r): **121674**; Calculated pI value: **6.95**
NCBI BLAST search of gi|38569421 against nr
Unformatted sequence string for pasting into other applications

Taxonomy: Homo sapiens
Links to retrieve other entries containing this sequence from NCBI Entrez:
gi|116241237 from Homo sapiens
gi|13623199 from Homo sapiens
gi|119581183 from Homo sapiens
gi|119581185 from Homo sapiens

Fixed modifications: Carbamidomethyl (C)
Variable modifications: Oxidation (M)
Cleavage by Trypsin: cuts C-term side of KR unless next residue is P
Sequence Coverage: **8%**

Matched peptides shown in **Bold Red**

```
  1 MSAKAISEQT GKELLYKFIC TTSAIQNRFK YARVTPDTDW ARLLQDHPWL
 51 LSQNLVVKPD QLIKRRGKLG LVGVNLTLDG VKSWLKPRLG QEATVGKATG
101 FLKNFLIEPF VPHSQAEEFY VCIYATREGD YVLFHHEGGV DVGDVDAKAQ
151 KLLVGVDEKL NPEDIKKHLL VHAPEDKKEI LASFISGLFN FYEDLYFTYL
201 EINPLVVTKD GVYVLDLAAK VDATADYICK VKWGDIEFPP PFGREAYPEE
251 AYIADLDAKS GASLKLTLLN PKGRIWTMVA GGGASVVYSD TICDLGGVNE
301 LANYGEYSGA PSEQQTYDYA KTILSLMTRE KHPDGKILII GGSIANFTNV
351 AATFKGIVRA IRDYQGPLKE HEVTIFVRRG GPNYQEGLRV MGEVGKTTGI
401 PIHVFGTETH MTAIVGMALG HRPIPNQPPT AAHTANFLLN ASGSTSTPAP
451 SRTASFSESR ADEVAPAKKA KPAMPQDSVP SPRSLQGKST TLFSRHTKAI
501 VWGMQTRAVQ GMLDFDYVCS RDEPSVAAMV YPFTGDHKQK FYWGHKEILI
551 PVFKNMADAM RKHPEVDVLI NFASLRSAYD STMETMNYAQ IRTIAIIAEG
601 IPEALTRKLI KKADQKGVTI IGPATVGGIK PGCFKIGNTG GMLDNILASK
651 LYRPGSVAYV SRSGGMSNEL NNIISRTTDG VYEGVAIGGD RYPGSTFMDH
701 VLRYQDTPGV KMIVVLGEIG GTEEYKICRG IKEGRLTKPI VCWCIGTCAT
751 MFSSEVQFGH AGACANQASE TAVAKNQALK EAGVFVPRSF DELGEIIQSV
801 YEDLVANGVI VPAQEVPPPT VPMDYSWARE LGLIRKPASF MTSICDERGQ
```

```
 851 ELIYAGMPIT EVFKEEMGIG GVLGLLWFQK RLPKYSCQFI EMCLMVTADH
 901 GPAVSGAHNT IICARAGKDL VSSLTSGLLT IGDRFGGALD AAAKMFSKAF
 951 DSGIIPMEFV NKMKKEGKLI MGIGHRVKSI NNPDMRVQIL KDYVRQHFPA
1001 TPLLDYALEV EKITTSKKPN LILNVDGLIG VAFVDMLRNC GSFTREEADE
1051 YIDIGALNGI FVLGRSMGFI GHYLDQKRLK QGLYRHPWDD ISYVLPEHMS
1101 M
```

Residue Number Increasing Mass Decreasing Mass

Start - End	Observed	Mr(expt)	Mr(calc)	Delta	Miss	Sequence
18 - 28	1310.6613	1309.6540	1309.6448	0.0092	0	FICTTSAIQNR (No match)
233 - 244	1417.6936	1416.6863	1416.6826	0.0038	0	WGDIEFPPPFGR (No match)
363 - 378	1931.0070	1929.9997	1929.9948	0.0049	1	DYQGPLKEHEVTIFVR (No match)
379 - 389	1246.6296	1245.6223	1245.6214	0.0010	1	RGGPNYQEGLR (No match)
562 - 576	1737.9622	1736.9549	1736.9573	-0.0024	1	KHPEVDVLINFASLR (No match)
562 - 576	1737.9622	1736.9549	1736.9573	-0.0024	1	KHPEVDVLINFASLR (No match)
593 - 607	1567.9033	1566.8960	1566.8980	-0.0020	0	TIAIIAEGIPEALTR (No match)
593 - 607	1567.9033	1566.8960	1566.8980	-0.0020	0	TIAIIAEGIPEALTR (Ions score 21)
651 - 662	1367.7422	1366.7349	1366.7357	-0.0007	0	LYRPGSVAYVSR (Ions score 14)
651 - 662	1367.7422	1366.7349	1366.7357	-0.0007	0	LYRPGSVAYVSR (No match)

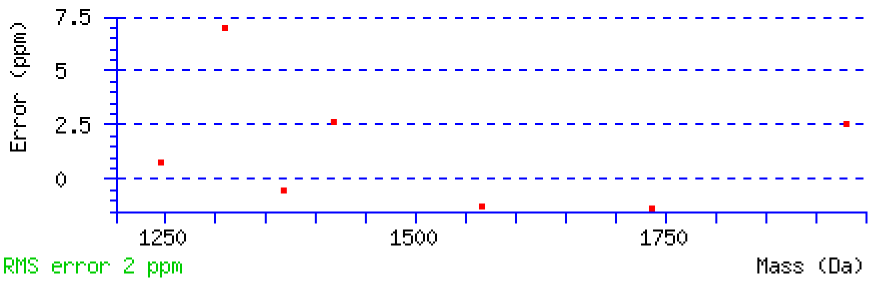

Mascot: http://www.matrixscience.com/

Spot 95

Mascot Search Results

Protein View

Match to: **gi|123209015** Score: **115** Expect: **6.1e-007**
KIAA1949 [Homo sapiens]

Nominal mass (M_r): **68286**; Calculated pI value: **5.42**
NCBI BLAST search of gi|123209015 against nr
Unformatted sequence string for pasting into other applications

Taxonomy: Homo sapiens

Fixed modifications: Carbamidomethyl (C)
Variable modifications: Oxidation (M)
Cleavage by Trypsin: cuts C-term side of KR unless next residue is P
Sequence Coverage: **18%**

Matched peptides shown in **Bold Red**

```
  1 MATIPDWKLQ LLARRRQEEA SVRGREKAER ERLSQMPAWK RGLLERRRAK
 51 LGLSPGEPSP VLGTVEAGPP DPDESAVLLE AIGPVHQNRF IRQERQQQQQ
101 QQQRSEELLA ERKPGPLEAR ERRPSPGEMR DQSPKGRESR EERLSPRETR
151 ERRLGIGGAQ ELSLRPLEAR DWRQSPGEVG DRSSRLSEAW KWRLSPGETP
201 ERSLRLAESR EQSPRRKEVE SRLSPGESAY QKLGLTEAHK WRPDSRESQE
251 QSLVQLEATE WRLRSGEERQ DYSEECGRKE EWPVPGVAPK ETAELSETLT
301 REAQGNSSAG VEAAEQRPVE DGERGMKPTE GWKWTLNSRK AREWTPRDIE
351 AQTQKPEPPE SAEKLLESPG VEAGEGEAEK EEAGAQGRPL RALQNCCSVP
401 SPLPPEDAGT GGLRQQEEEA VELQPPPPAP LSPPPPAPTA PQPPGDPLMS
451 RLFYGVKAGP GVGAPRRSGH TFTVNPRRSV PPATPATPTS PATVDAAVPG
501 AGKKRYPTAE EILVLGGYLR LSRSCLAKGS PERHHKQLKI SFSETALETT
551 YQYPSESSVL EELGPEPEVP SAPNPPAAQP DDEEDEEELL LLQPELQGGL
601 RTKALIVDES CRR
```

Residue Number Increasing Mass Decreasing Mass

Start - End	Observed	Mr(expt)	Mr(calc)	Delta	Miss	Sequence	
96 - 104	1199.6240	1198.6167	1198.5803	0.0364	0	QQQQQQQQR	(Ions score 3)
96 - 104	1199.6240	1198.6167	1198.5803	0.0364	0	QQQQQQQQR	(No match)

154 - 170	1779.9971	1778.9898	1779.0002	-0.0104	0	LGIGGAQELSLRPLEAR	(No match)
154 - 170	1779.9971	1778.9898	1779.0002	-0.0104	0	LGIGGAQELSLRPLEAR	(Ions score 22)
302 - 324	2386.0879	2385.0806	2385.0791	0.0015	0	EAQGNSSAGVEAAEQRPVEDGER	(No match)
325 - 339	1790.8760	1789.8687	1789.8933	-0.0246	1	GMKPTEGWKWTLNSR	(No match)
325 - 339	1790.8760	1789.8687	1789.8933	-0.0246	1	GMKPTEGWKWTLNSR	(No match)
392 - 414	2396.1216	2395.1143	2395.1260	-0.0116	0	ALQNCCSVPSPLPPEDAGTGGLR	(No match)
468 - 477	1115.5475	1114.5402	1114.5519	-0.0117	0	SGHTFTVNPR	(No match)
506 - 520	1693.8990	1692.8917	1692.9086	-0.0169	0	YPTAEEILVLGGYLR	(Ions score 24)
506 - 520	1693.8990	1692.8917	1692.9086	-0.0169	0	YPTAEEILVLGGYLR	(No match)

Mascot: http://www.matrixscience.com/

Spot 96

Mascot Search Results

Protein View

Match to: **gi|603074** Score: **144** Expect: **7.7e-010**
ATP:citrate lyase

Nominal mass (M_r): **121660**; Calculated pI value: **6.95**
NCBI BLAST search of gi|603074 against nr
Unformatted sequence string for pasting into other applications

Taxonomy: Homo sapiens

Fixed modifications: Carbamidomethyl (C)
Variable modifications: Oxidation (M)
Cleavage by Trypsin: cuts C-term side of KR unless next residue is P
Sequence Coverage: **10%**

Matched peptides shown in **Bold Red**

1 MSAKAISEQT GKELLYK**FIC TTSAIQNR**FK YARVTPDTDW ARLLQDHPWL
51 LSQNLVVKPD QLIKRRGKLG LVGVNLTLDG VKSWLKPRLG QEATVGKATG
101 FLKNFLIEPF VPHSQAEEFY VCIYATREGD YVLFHHEGGV DVGDVDAKAQ
151 KLLVGVDEKL NPEDIKKHLL VHAPDDKKEI LASFISGLFN FYEDLYFTYL
201 EINPLVVTKD GVYVLDLAAK VDATADYICK VK**WGDIEFPP PFGR**EAYPEE
251 AYIADLDAKS GASLKLTLLN PKGRIWTMVA GGGASVVYSD TICDLGGVNE
301 LANYGEYSGA PSEQQTYDYA KTILSLMTRE KHPDGKILII GGSIANFTNV
351 AATFKGIVRA IR**DYQGPLKE HEVTIFVRRG GPNYQEGLR**V MGEVGKTTGI
401 PIHVFGTETH MTAIVGMALG HRPIPNQPPT AAHTANFLLN ASGSTSTPAP
451 SRTASFSESR ADEVAPAKKA KPAMPQDSVP SPRSLQGKST TLFSRHTKAI
501 VWGMQTRAVQ GMLDFDYVCS RDEPSVAAMV YPFTGDHKQK FYWGHKEILI
551 PVFKNMADAM R**KHPEVDVLI NFASLR**SAYD STMETMNYAQ IR**TIAIIAEG**
601 **IPEALTR**KLI KKADQKGVTI IGPATVGGIK PGCFKIGNTG GMLDNILASK
651 **LYRPGSVAYV SR**SGGMSNEL NNIISRTTDG VYEGVAIGGD RYPGSTFMDH
701 VLRYQDTPGV KMIVVLGEIG GTEEYKICRG IKEGRLTKPI VCWCIGTCAT
751 MFSSEVQFGH AGACANQASE TAVAKNQALK **EAGVFVPR**SF DELGEIIQSV
801 YEDLVANGVI VPAQEVPPPT VPMDYSWARE LGLIRKPASF MTSICDERGQ
851 ELIYAGMPIT EVFKEEMGIG GVLGLLWFQK RLPKYSCQFI EMCLMVTADH
901 GPAVSGAHNT IICARAGKDL VSSLTSGLLT IGDRFGGALD AAAKMFSKAF
951 DSGIIPMEFV NKMKKEGKLI MGIGHRVKSI NNPDMRVQIL KDYVRQHFPA
1001 TPLLDYALEV EKITTSKKPN LILNVDGLIG VAFVDMLRNC GSFTR**EEADE**
1051 **YIDIGALNGI FVLGR**SMGFI GHYLDQKRLK QGLYRHPWDD ISYVLPEHMS

1101 M

Residue Number Increasing Mass Decreasing Mass

Start - End	Observed	Mr(expt)	Mr(calc)	Delta	Miss	Sequence
18 - 28	1310.6555	1309.6482	1309.6448	0.0034	0	FICTTSAIQNR (No match)
233 - 244	1417.6974	1416.6901	1416.6826	0.0076	0	WGDIEFPPPFGR (No match)
363 - 378	1931.0048	1929.9975	1929.9948	0.0027	1	DYQGPLKEHEVTIFVR (No match)
370 - 378	1129.5994	1128.5921	1128.5927	-0.0006	0	EHEVTIFVR (No match)
379 - 389	1246.6326	1245.6253	1245.6214	0.0040	1	RGGPNYQEGLR (Ions score 1)
379 - 389	1246.6326	1245.6253	1245.6214	0.0040	1	RGGPNYQEGLR (No match)
380 - 389	1090.5311	1089.5238	1089.5202	0.0036	0	GGPNYQEGLR (No match)
562 - 576	1737.9688	1736.9615	1736.9573	0.0042	1	KHPEVDVLINFASLR (Ions score 10)
562 - 576	1737.9688	1736.9615	1736.9573	0.0042	1	KHPEVDVLINFASLR (No match)
593 - 607	1567.9077	1566.9004	1566.8980	0.0024	0	TIAIIAEGIPEALTR (No match)
593 - 607	1567.9077	1566.9004	1566.8980	0.0024	0	TIAIIAEGIPEALTR (Ions score 28)
651 - 662	1367.7468	1366.7395	1366.7357	0.0039	0	LYRPGSVAYVSR (Ions score 15)
651 - 662	1367.7468	1366.7395	1366.7357	0.0039	0	LYRPGSVAYVSR (No match)
781 - 788	874.4866	873.4793	873.4708	0.0085	0	EAGVFVPR (No match)
1046 - 1065	2194.1021	2193.0948	2193.0952	-0.0004	0	EEADEYIDIGALNGIFVLGR (No match)

Mascot: http://www.matrixscience.com/

Spot 99

Mascot Search Results

Protein View

Match to: **gi|603074** Score: **144** Expect: **7.7e-010**
ATP:citrate lyase

Nominal mass (M_r): **121660**; Calculated pI value: **6.95**
NCBI BLAST search of gi|603074 against nr
Unformatted sequence string for pasting into other applications

Taxonomy: Homo sapiens

Fixed modifications: Carbamidomethyl (C)
Variable modifications: Oxidation (M)
Cleavage by Trypsin: cuts C-term side of KR unless next residue is P
Sequence Coverage: **10%**

Matched peptides shown in **Bold Red**

```
   1 MSAKAISEQT GKELLYKFIC TTSAIQNRFK YARVTPDTDW ARLLQDHPWL
  51 LSQNLVVKPD QLIKRRGKLG LVGVNLTLDG VKSWLKPRLG QEATVGKATG
 101 FLKNFLIEPF VPHSQAEEFY VCIYATREGD YVLFHHEGGV DVGDVDAKAQ
 151 KLLVGVDEKL NPEDIKKHLL VHAPDDKKEI LASFISGLFN FYEDLYFTYL
 201 EINPLVVTKD GVYVLDLAAK VDATADYICK VKWGDIEFPP PFGREAYPEE
 251 AYIADLDAKS GASLKLTLLN PKGRIWTMVA GGGASVVYSD TICDLGGVNE
 301 LANYGEYSGA PSEQQTYDYA KTILSLMTRE KHPDGKILII GGSIANFTNV
 351 AATFKGIVRA IRDYQGPLKE HEVTIFVRRG GPNYQEGLRV MGEVGKTTGI
 401 PIHVFGTETH MTAIVGMALG HRPIPNQPPT AAHTANFLLN ASGSTSTPAP
 451 SRTASFSESR ADEVAPAKKA KPAMPQDSVP SPRSLQGKST TLFSRHTKAI
 501 VWGMQTRAVQ GMLDFDYVCS RDEPSVAAMV YPFTGDHKQK FYWGHKEILI
 551 PVFKNMADAM RKHPEVDVLI NFASLRSAYD STMETMNYAQ IRTIAIIAEG
 601 IPEALTRKLI KKADQKGVTI IGPATVGGIK PGCFKIGNTG GMLDNILASK
 651 LYRPGSVAYV SRSGGMSNEL NNIISRTTDG VYEGVAIGGD RYPGSTFMDH
 701 VLRYQDTPGV KMIVVLGEIG GTEEYKICRG IKEGRLTKPI VCWCIGTCAT
 751 MFSSEVQFGH AGACANQASE TAVAKNQALK EAGVFVPRSF DELGEIIQSV
 801 YEDLVANGVI VPAQEVPPPT VPMDYSWARE LGLIRKPASF MTSICDERGQ
 851 ELIYAGMPIT EVFKEEMGIG GVLGLLWFQK RLPKYSCQFI EMCLMVTADH
 901 GPAVSGAHNT IICARAGKDL VSSLTSGLLT IGDRFGGALD AAAKMFSKAF
 951 DSGIIPMEFV NKMKKEGKLI MGIGHRVKSI NNPDMRVQIL KDYVRQHFPA
1001 TPLLDYALEV EKITTSKKPN LILNVDGLIG VAFVDMLRNC GSFTREEADE
1051 YIDIGALNGI FVLGRSMGFI GHYLDQKRLK QGLYRHPWDD ISYVLPEHMS
```

1101 M

Residue Number Increasing Mass Decreasing Mass

Start - End	Observed	Mr(expt)	Mr(calc)	Delta	Miss	Sequence
18 - 28	1310.6555	1309.6482	1309.6448	0.0034	0	FICTTSAIQNR (No match)
233 - 244	1417.6974	1416.6901	1416.6826	0.0076	0	WGDIEFPPPFGR (No match)
363 - 378	1931.0048	1929.9975	1929.9948	0.0027	1	DYQGPLKEHEVTIFVR (No match)
370 - 378	1129.5994	1128.5921	1128.5927	-0.0006	0	EHEVTIFVR (No match)
379 - 389	1246.6326	1245.6253	1245.6214	0.0040	1	RGGPNYQEGLR (Ions score 1)
379 - 389	1246.6326	1245.6253	1245.6214	0.0040	1	RGGPNYQEGLR (No match)
380 - 389	1090.5311	1089.5238	1089.5202	0.0036	0	GGPNYQEGLR (No match)
562 - 576	1737.9688	1736.9615	1736.9573	0.0042	1	KHPEVDVLINFASLR (Ions score 10)
562 - 576	1737.9688	1736.9615	1736.9573	0.0042	1	KHPEVDVLINFASLR (No match)
593 - 607	1567.9077	1566.9004	1566.8980	0.0024	0	TIAIIAEGIPEALTR (No match)
593 - 607	1567.9077	1566.9004	1566.8980	0.0024	0	TIAIIAEGIPEALTR (Ions score 28)
651 - 662	1367.7468	1366.7395	1366.7357	0.0039	0	LYRPGSVAYVSR (Ions score 15)
651 - 662	1367.7468	1366.7395	1366.7357	0.0039	0	LYRPGSVAYVSR (No match)
781 - 788	874.4866	873.4793	873.4708	0.0085	0	EAGVFVPR (No match)
1046 - 1065	2194.1021	2193.0948	2193.0952	-0.0004	0	EEADEYIDIGALNGIFVLGR (No match)

Mascot: http://www.matrixscience.com/

Spot 102

Mascot Search Results

Protein View

Match to: **gi|32400649** Score: **151** Expect: **1.5e-010**
leukocyte-derived arginine aminopeptidase long form variant [Homo sapiens]

Nominal mass (M_r): **111061**; Calculated pI value: **6.20**
NCBI BLAST search of gi|32400649 against nr
Unformatted sequence string for pasting into other applications

Taxonomy: Homo sapiens

Fixed modifications: Carbamidomethyl (C)
Variable modifications: Oxidation (M)
Cleavage by Trypsin: cuts C-term side of KR unless next residue is P
Sequence Coverage: **9%**

Matched peptides shown in **Bold Red**

```
  1 MFHSSAMVNS HRKPMFNIHR GFYCLTAILP QICICSQFSV PSSYHFTEDP
 51 GAFPVATNGE RFPWQELRLP SVVIPLHYDL FVHPNLTSLD FVASEKIEVL
101 VSNATQFIIL HSKDLEITNA TLQSEEDSRY MKPGKELKVL SYPAHEQIAL
151 LVPEKLTPHL KYYVAMDFQA KLGDGFEGFY KSTYRTLGGE TRILAVTDFE
201 PTQARMAFPC FDEPLFKANF SIKIRRESRH IALSNMPKVK TIELEGGLLE
251 DHFETTVKMS TYLVAYIVCD FHSLSGFTSS GVKVSIYASP DKRNQTHYAL
301 QASLKLLDFY EKYFDIYYPL SKLDLIAIPD FAPGAMENWG LITYRETSLL
351 FDPKTSSASD KLWVTRVIAH ELAHQWFGNL VTMEWWNDIW LNEGFAKYME
401 LIAVNATYPE LQFDDYFLNV CFEVITKDSL NSSRPISKPA ETPTQIQEMF
451 DEVSYNKGAC ILNMLKDFLG EEKFQKGIIQ YLKKFSYRNA KNDDLWSSLS
501 NSCLESDFTS GGVCHSDPKM TSNMLAFLGE NAEVKEMMTT WTLQKGIPLL
551 VVKQDGCSLR LQQERFLQGV FQEDPEWRAL QERYLWHIPL TYSTSSSNVI
601 HRHILKSKTD TLDLPEKTSW VKFNVDSNGY YIVHYEGHGW DQLITQLNQN
651 HTLLRPKDRV GLIHDVFQLV GAGRLTLDKA LDMTYYLQHE TSSPALLEGL
701 SYLESFYHMM DRRNISDISE NLKRYLLQYF KPVIDRQSWS DKGSVWDRML
751 RSALLKLACD LNHAPCIQKA AELFSQWMES SGKLNIPTDV LKIVYSVGAQ
801 TTAGWNYLLE QYELSMSSAE QNKILYALST SKHQEKLLKL IELGMEGKVI
851 KTQNLAALLH AIARRPKGQQ LAWDFVRENW THLLKKFDLG SYDIRMIISG
901 TTAHFSSKDK LQEVKLFFES LEAQGSHLDI FQTVLETITK NIKWLEKNLP
951 TLRTWLMVNT
```

Residue Number Increasing Mass Decreasing Mass

Start - End	Observed	Mr(expt)	Mr(calc)	Delta	Miss	Sequence
162 - 171	1235.6139	1234.6066	1234.5692	0.0374	0	YYVAMDFQAK (No match)
172 - 181	1132.5270	1131.5197	1131.5236	-0.0039	0	LGDGFEGFYK (No match)
193 - 205	1460.7675	1459.7602	1459.7670	-0.0068	0	ILAVTDFEPTQAR (No match)
193 - 205	1460.7675	1459.7602	1459.7670	-0.0068	0	ILAVTDFEPTQAR (Ions score 27)
284 - 292	979.5142	978.5069	978.5022	0.0048	0	VSIYASPDK (No match)
658 - 674	1852.0179	1851.0106	1851.0114	-0.0008	1	DRVGLIHDVFQLVGAGR (No match)
660 - 674	1580.8829	1579.8756	1579.8834	-0.0077	0	VGLIHDVFQLVGAGR (Ions score 30)
660 - 674	1580.8829	1579.8756	1579.8834	-0.0077	0	VGLIHDVFQLVGAGR (No match)
725 - 736	1554.8600	1553.8527	1553.8605	-0.0078	0	YLLQYFKPVIDR (No match)
725 - 736	1554.8600	1553.8527	1553.8605	-0.0078	0	YLLQYFKPVIDR (Ions score 12)
852 - 864	1391.8101	1390.8028	1390.8044	-0.0016	0	TQNLAALLHAIAR (No match)
852 - 864	1391.8101	1390.8028	1390.8044	-0.0016	0	TQNLAALLHAIAR (Ions score 30)
886 - 895	1213.6223	1212.6150	1212.6138	0.0012	1	KFDLGSYDIR (No match)

Mascot: http://www.matrixscience.com/

Spot 104

{MATRIX SCIENCE} Mascot Search Results

Protein View

Match to: **gi|40807029** Score: **67** Expect: **0.041**
ERAP2 protein [Homo sapiens]

Nominal mass (M_r): **106086**; Calculated pI value: **6.37**
NCBI BLAST search of gi|40807029 against nr
Unformatted sequence string for pasting into other applications

Taxonomy: Homo sapiens

Fixed modifications: Carbamidomethyl (C)
Variable modifications: Oxidation (M)
Cleavage by Trypsin: cuts C-term side of KR unless next residue is P
Sequence Coverage: **7%**

Matched peptides shown in **Bold Red**

```
  1 MFHSSAMVNS HRKPMFNIHR GFYCLTAILP QICICSQFSV PSSYHFTEDP
 51 GAFPVATNGE RFPWQELRLP SVVIPLHYDL FVHPNLTSLD FVASEKIEVL
101 VSNATQFIIL HSKDLEITNA TLQSEEDSRY MKPGKELKVL SYPAHEQIAL
151 LVPEKLTPHL KYYVAMDFQA KLGDGFEGFY KSTYRTLGGE TRILAVTDFE
201 PTQARMAFPC FDEPLFKANF SIKIRRESRH IALSNMPKVS IYASPDKRNQ
251 THYALQASLK LLDFYEKYFD IYYPLSKLDL IAIPDFAPGA MENWGLITYR
301 ETSLLFDPKT SSASDKLWVT RVIAHELAHQ WFGNLVTMEW WNDIWLKEGF
351 AKYMELIAVN ATYPELQFDD YFLNVCFEVI TKDSLNSSRP ISKPAETPTQ
401 IQEMFDEVSY NKGACILNML KDFLGEEKFQ KGIIQYLKKF SYRNAKNDDL
451 WSSLSNSCLE SDFTSGGVCH SDPKMTSNML AFLGENAEVK EMMTTWTLQK
501 GIPLLVVKQD GCSLRLQQER FLQGVFQEDP EWRALQERYL WHIPLTYSTS
551 SSNVIHRHIL KSKTDTLDLP EKTSWVKFNV DSNGYYIVHY EGHGWDQLIT
601 QLNQNHTLLR PKDRVGLIHD VFQLVGAGRL TLDKALDMTY YLQHETSSPA
651 LLEGLSYLES FYHMMDRRNI SDISENLKRY LLQYFKPVID RQSWSDKGSV
701 WDRMLRSALL KLACDLNHAP CIQKAAELFS QWMESSGKLN IPTDVLKIVY
751 SVGAQTTAGW NYLLEQYELS MSSAEQNKIL YALSTSKHQE KLLKLIELGM
801 EGKVIKTQNL AALLHAIARR PKGQQLAWDF VRENWTHLLK KFDLGSYDIR
851 MIISGTTAHF SSKDKLQEVK LFFESLEAQG SHLDIFQTVL ETITKNIKWL
901 EKNLPTLRTW LMVNT
```

Residue Number Increasing Mass Decreasing Mass

Start - End	Observed	Mr(expt)	Mr(calc)	Delta	Miss	Sequence
62 - 68	975.4998	974.4925	974.4974	-0.0048	0	FPWQELR (No match)
193 - 205	1460.7726	1459.7653	1459.7670	-0.0017	0	ILAVTDFEPTQAR (Ions score 7)
193 - 205	1460.7726	1459.7653	1459.7670	-0.0017	0	ILAVTDFEPTQAR (No match)
615 - 629	1580.8895	1579.8822	1579.8834	-0.0011	0	VGLIHDVFQLVGAGR (Ions score 0)
615 - 629	1580.8895	1579.8822	1579.8834	-0.0011	0	VGLIHDVFQLVGAGR (No match)
680 - 691	1554.8663	1553.8590	1553.8605	-0.0015	0	YLLQYFKPVIDR (No match)
680 - 691	1554.8663	1553.8590	1553.8605	-0.0015	0	YLLQYFKPVIDR (Ions score 6)
804 - 819	1732.0985	1731.0912	1731.0518	0.0394	1	VIKTQNLAALLHAIAR (No match)
807 - 819	1391.8113	1390.8040	1390.8044	-0.0004	0	TQNLAALLHAIAR (No match)
807 - 819	1391.8113	1390.8040	1390.8044	-0.0004	0	TQNLAALLHAIAR (Ions score 5)
841 - 850	1213.6156	1212.6083	1212.6138	-0.0055	1	KFDLGSYDIR (No match)

Mascot: http://www.matrixscience.com/

Spot 106

Mascot Search Results

Protein View

Match to: **gi|40807029** Score: **79** Expect: **0.0025**
ERAP2 protein [Homo sapiens]

Nominal mass (M_r): **106086**; Calculated pI value: **6.37**
NCBI BLAST search of gi|40807029 against nr
Unformatted sequence string for pasting into other applications

Taxonomy: Homo sapiens

Fixed modifications: Carbamidomethyl (C)
Variable modifications: Oxidation (M)
Cleavage by Trypsin: cuts C-term side of KR unless next residue is P
Sequence Coverage: **7%**

Matched peptides shown in **Bold Red**

```
  1 MFHSSAMVNS HRKPMFNIHR GFYCLTAILP QICICSQFSV PSSYHFTEDP
 51 GAFPVATNGE RFPWQELRLP SVVIPLHYDL FVHPNLTSLD FVASEKIEVL
101 VSNATQFIIL HSKDLEITNA TLQSEEDSRY MKPGKELKVL SYPAHEQIAL
151 LVPEKLTPHL KYYVAMDFQA KLGDGFEGFY KSTYRTLGGE TRILAVTDFE
201 PTQARMAFPC FDEPLFKANF SIKIRRESRH IALSNMPKVS IYASPDKRNQ
251 THYALQASLK LLDFYEKYFD IYYPLSKLDL IAIPDFAPGA MENWGLITYR
301 ETSLLFDPKT SSASDKLWVT RVIAHELAHQ WFGNLVTMEW WNDIWLKEGF
351 AKYMELIAVN ATYPELQFDD YFLNVCFEVI TKDSLNSSRP ISKPAETPTQ
401 IQEMFDEVSY NKGACILNML KDFLGEEKFQ KGIIQYLKKF SYRNAKNDDL
451 WSSLSNSCLE SDFTSGGVCH SDPKMTSNML AFLGENAEVK EMMTTWTLQK
501 GIPLLVVKQD GCSLRLQQER FLQGVFQEDP EWRALQERYL WHIPLTYSTS
551 SSNVIHRHIL KSKTDTLDLP EKTSWVKFNV DSNGYYIVHY EGHGWDQLIT
601 QLNQNHTLLR PKDRVGLIHD VFQLVGAGRL TLDKALDMTY YLQHETSSPA
651 LLEGLSYLES FYHMMDRRNI SDISENLKRY LLQYFKPVID RQSWSDKGSV
701 WDRMLRSALL KLACDLNHAP CIQKAAELFS QWMESSGKLN IPTDVLKIVY
751 SVGAQTTAGW NYLLEQYELS MSSAEQNKIL YALSTSKHQE KLLKLIELGM
801 EGKVIKTQNL AALLHAIARR PKGQQLAWDF VRENWTHLLK KFDLGSYDIR
851 MIISGTTAHF SSKDKLQEVK LFFESLEAQG SHLDIFQTVL ETITKNIKWL
901 EKNLPTLRTW LMVNT
```

Residue Number Increasing Mass Decreasing Mass

Start - End	Observed	Mr(expt)	Mr(calc)	Delta	Miss	Sequence
193 - 205	1460.7401	1459.7328	1459.7670	-0.0342	0	ILAVTDFEPTQAR (No match)
193 - 205	1460.7401	1459.7328	1459.7670	-0.0342	0	ILAVTDFEPTQAR (Ions score 8)
613 - 629	1851.9606	1850.9533	1851.0114	-0.0581	1	DRVGLIHDVFQLVGAGR (No match)
615 - 629	1580.8510	1579.8437	1579.8834	-0.0396	0	VGLIHDVFQLVGAGR (No match)
615 - 629	1580.8510	1579.8437	1579.8834	-0.0396	0	VGLIHDVFQLVGAGR (Ions score 10)
680 - 691	1554.8290	1553.8217	1553.8605	-0.0388	0	YLLQYFKPVIDR (No match)
680 - 691	1554.8290	1553.8217	1553.8605	-0.0388	0	YLLQYFKPVIDR (Ions score 11)
807 - 819	1391.7800	1390.7727	1390.8044	-0.0317	0	TQNLAALLHAIAR (No match)
807 - 819	1391.7800	1390.7727	1390.8044	-0.0317	0	TQNLAALLHAIAR (Ions score 16)
841 - 850	1213.5936	1212.5863	1212.6138	-0.0275	1	KFDLGSYDIR (No match)

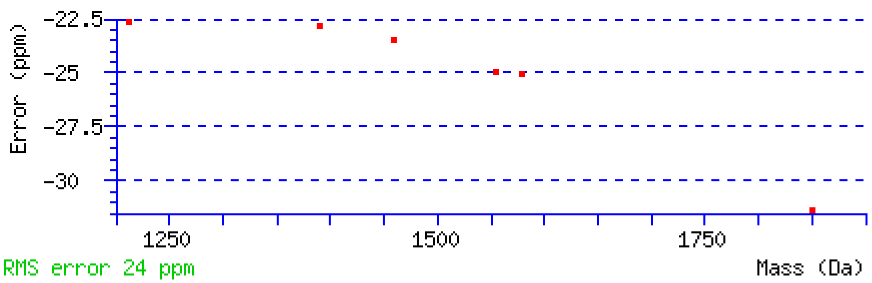

Mascot: http://www.matrixscience.com/

Spot 108

Mascot Search Results

Protein View

Match to: **gi|24657579** Score: **188** Expect: **3e-014**
Vinculin [Homo sapiens]

Nominal mass (M_r): **117234**; Calculated pI value: **5.83**
NCBI BLAST search of gi|24657579 against nr
Unformatted sequence string for pasting into other applications

Taxonomy: Homo sapiens
Links to retrieve other entries containing this sequence from NCBI Entrez:
gi|123996009 from synthetic construct

Fixed modifications: Carbamidomethyl (C)
Variable modifications: Oxidation (M)
Cleavage by Trypsin: cuts C-term side of KR unless next residue is P
Sequence Coverage: **7%**

Matched peptides shown in **Bold Red**

1 MPVFHTRTIE SILEPVAQQI SHLVIMHEEG EVDGK**AIPDL TAPVAAVQAA**
51 **VSNLVR**VGKE TVQTTEDQIL KRDMPPAFIK VENACTKLVQ AAQMLQSDPY
101 SVPARDYLID GSR**GILSGTS DLLLTFDEAE VRK**IIRVCKG ILEYLTVAEV
151 VETMEDLVTY TKNLGPGMTK MAKMIDERQQ ELTHQEHRVM LVNSMNTVKE
201 LLPVLISAMK IFVTTKNSKN QGIEEALKNR NFTLEKMSAE INEIIRVLQL
251 TSWDEDAWAS KDTEAMKRAL ASIDSKLNQA KGWLRDPSAS PGDAGEQAIR
301 QILDEAGKVG ELCAGKERRE ILGTCKMLGQ MTDQVADLRA RGQGSSPVAM
351 QKAQQVSQGL DVLTAKVENA ARKLEAMTNS KQSIAKKIDA AQNWLADPNG
401 GPEGEEQIRG ALAEARKIAE LCDDPKERDD ILRSLGEISA LTSKLADLRR
451 QGKGDSPEAR ALAKQVATAL QNLQTKTNRA VANSRPAKAA VHLEGKIEQA
501 QRWIDNPTVD DRGVGQAAIR GLVAEGHRLA NVMMGPYRQD LLAKCDRVDQ
551 LTAQLADLAA RGEGESPQAR ALASQLQDSL KDLKARMQEA MTQEVSDVFS
601 DTTTPIK**LLA VAATAPPDAP NREEVFDER**A ANFENHSGKL GATAEKAAAV
651 GTANKSTVEG IQASVKTARE LTPQVVSAAR ILLRNPGNQA AYEHFETMKN
701 QWIDNVEKMT GLVDEAIDTK SLLDASEEAI KKDLDKCKVA MANIQPQMLV
751 AGATSIARRA NRILLVAKRE VENSEDPKFR EAVKAASDEL SKTISPMVMD
801 AKAVAGNISD PGLQKSFLDS GYRILGAVAK VR**EAFQPQEP DFPPPPPDLE**
851 **QLR**LTDELAP PKPPLPEGEV PPPRPPPPEE KDEEFPEQKA GEVINQPMMM
901 AARQLHDEAR KWSSKGNDII AAAKRMALLM AEMSRLVRGG SGTKRALIQC
951 AKDIAKASDE VTRLAKEVAK QCTDKRIRTN LLQVCERIPT ISTQLKILST

1001 VKATMLGRTN ISDEESEQAT EMLVHNAQNL MQSVKETVRE AEAASIKIRT
1051 DAGFTLRWVR KTPWYQ

Residue Number Increasing Mass Decreasing Mass

Start - End	Observed	Mr(expt)	Mr(calc)	Delta	Miss	Sequence
36 - 56	2076.1821	2075.1748	2075.1738	0.0010	0	AIPDLTAPVAAVQAAVSNLVR (No match)
36 - 56	2076.1821	2075.1748	2075.1738	0.0010	0	AIPDLTAPVAAVQAAVSNLVR (Ions score 48)
114 - 133	2164.1584	2163.1511	2163.1422	0.0089	1	GILSGTSDLLLTFDEAEVRK (No match)
114 - 133	2164.1584	2163.1511	2163.1422	0.0089	1	GILSGTSDLLLTFDEAEVRK (Ions score 29)
608 - 629	2381.2236	2380.2163	2380.2022	0.0141	1	LLAVAATAPPDAPNREEVFDER (Ions score 20)
608 - 629	2381.2236	2380.2163	2380.2022	0.0141	1	LLAVAATAPPDAPNREEVFDER (No match)
833 - 853	2447.1975	2446.1902	2446.1804	0.0098	0	EAFQPQEPDFPPPPPDLEQLR (No match)
833 - 853	2447.1975	2446.1902	2446.1804	0.0098	0	EAFQPQEPDFPPPPPDLEQLR (Ions score 57)

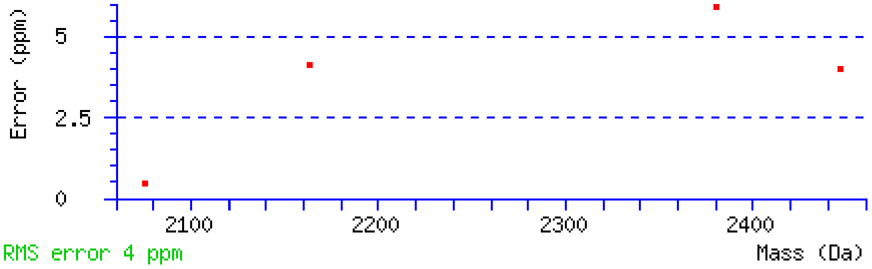

Mascot: http://www.matrixscience.com/

Spot 109

{MATRIX SCIENCE} Mascot Search Results

Protein View

Match to: **gi|109148542** Score: **93** Expect: **9.2e-005**
alanyl-tRNA synthetase [Homo sapiens]

Nominal mass (M_r): **107485**; Calculated pI value: **5.34**
NCBI BLAST search of gi|109148542 against nr
Unformatted sequence string for pasting into other applications

Taxonomy: Homo sapiens
Links to retrieve other entries containing this sequence from NCBI Entrez:
gi|115502460 from Homo sapiens
gi|15079238 from Homo sapiens
gi|119572224 from Homo sapiens
gi|123981462 from synthetic construct
gi|123996297 from synthetic construct

Fixed modifications: Carbamidomethyl (C)
Variable modifications: Oxidation (M)
Cleavage by Trypsin: cuts C-term side of KR unless next residue is P
Sequence Coverage: **3%**

Matched peptides shown in **Bold Red**

1 MDSTLTASEI RQR**FIDFFKR** NEHTYVHSSA TIPLDDPTLL FANAGMNQFK
51 PIFLNTIDPS HPMAKLSRAA NTQKCIRAGG KHNDLDDVGK DVYHHTFFEM
101 LGSWSFGDYF KELACKMALE LLTQEFGIPI ERLYVTYFGG DEAAGLEADL
151 ECKQIWQNLG LDDTKILPGN MKDNFWEMGD TGPCGPCSEI HYDRIGGRDA
201 AHLVNQDDPN VLEIWNLVFI QYNREADGIL KPLPKKSIDT GMGLERLVSV
251 LQNKMSNYDT DLFVPYFEAI QKGTGARPYT GKVGAEDADG IDMAYRVLAD
301 HARTITVALA DGGRPDNTGR GYVLRRILRR AVRYAHEKLN ASRGFFATLV
351 DVVVQSLGDA FPELKKDPDM VKDIINEEEV QFLKTLSRGR RILDRKIQSL
401 GDSKTIPGDT AWLLYDTYGF PVDLTGLIAE EKGLVVDMDG FEEERKLAQL
451 KSQGKGAGGE DLIMLDIYAI EELRARGLEV TDDSPKYNYH LDSSGSYVFE
501 NTVATVMALR REKMFVEEVS TGQECGVVLD KTCFYAEQGG QIYDEGYLVK
551 VDDSSEDKTE FTVKNAQVRG GYVLHIGTIY GDLKVGDQVW LFIDEPRRRP
601 IMSNHTATHI LNFALRSVLG EADQKGSLVA PDRLRFDFTA KGAMSTQQIK
651 KAEEIANEMI EAAKAVYTQD CPLAAAKAIQ GLR**AVFDETY PDPVR**VVSIG
701 VPVSELLDDP SGPAGSLTSV EFCGGTHLRN SSHAGAFVIV TEEAIAKGIR
751 RIVAVTGAEA QKALRKAESL KKCLSVMEAK VKAQTAPNKD VQREIADLGE

```
801 ALATAVIPQW QKDELRETLK SLKKVMDDLD RASKADVQKR VLEKTKQFID
851 SNPNQPLVIL EMESGASAKA LNEALKLFKM HSPQTSAMLF TVDNEAGKIT
901 CLCQVPQNAA NRGLKASEWV QQVSGLMDGK GGGKDVSAQA TGKNVGCLQE
951 ALQLATSFAQ LRLGDVKN
```

Residue Number Increasing Mass Decreasing Mass

Start - End	Observed	Mr(expt)	Mr(calc)	Delta	Miss	Sequence
14 - 20	972.5350	971.5277	971.5228	0.0049	1	FIDFFKR (No match)
684 - 695	1408.6689	1407.6616	1407.6670	-0.0054	0	AVFDETYPDPVR (No match)
684 - 695	1408.6689	1407.6616	1407.6670	-0.0054	0	AVFDETYPDPVR (Ions score 46)
899 - 912	1644.7826	1643.7753	1643.7872	-0.0118	0	ITCLCQVPQNAANR (No match)
899 - 912	1644.7826	1643.7753	1643.7872	-0.0118	0	ITCLCQVPQNAANR (Ions score 22)

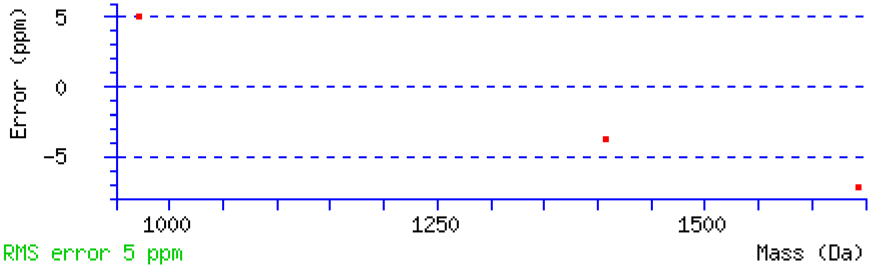

Mascot: http://www.matrixscience.com/

Spot 111

Protein View

Match to: **gi|109148542** Score: **160** Expect: **1.9e-011**
alanyl-tRNA synthetase [Homo sapiens]

Nominal mass (M_r): **107485**; Calculated pI value: **5.34**
NCBI BLAST search of gi|109148542 against nr
Unformatted sequence string for pasting into other applications

Taxonomy: Homo sapiens
Links to retrieve other entries containing this sequence from NCBI Entrez:
gi|115502460 from Homo sapiens
gi|15079238 from Homo sapiens
gi|119572224 from Homo sapiens
gi|123981462 from synthetic construct
gi|123996297 from synthetic construct

Fixed modifications: Carbamidomethyl (C)
Variable modifications: Oxidation (M)
Cleavage by Trypsin: cuts C-term side of KR unless next residue is P
Sequence Coverage: **5%**

Matched peptides shown in **Bold Red**

```
  1 MDSTLTASEI RQRFIDFFKR NEHTYVHSSA TIPLDDPTLL FANAGMNQFK
 51 PIFLNTIDPS HPMAKLSRAA NTQKCIRAGG KHNDLDDVGK DVYHHTFFEM
101 LGSWSFGDYF KELACKMALE LLTQEFGIPI ERLYVTYFGG DEAAGLEADL
151 ECKQIWQNLG LDDTKILPGN MKDNFWEMGD TGPCGPCSEI HYDRIGGRDA
201 AHLVNQDDPN VLEIWNLVFI QYNREADGIL KPLPKKSIDT GMGLERLVSV
251 LQNKMSNYDT DLFVPYFEAI QKGTGARPYT GKVGAEDADG IDMAYRVLAD
301 HARTITVALA DGGRPDNTGR GYVLRRILRR AVRYAHEKLN ASRGFFATLV
351 DVVVQSLGDA FPELKKDPDM VKDIINEEEV QFLKTLSRGR RILDRKIQSL
401 GDSKTIPGDT AWLLYDTYGF PVDLTGLIAE EKGLVVDMDG FEEERKLAQL
451 KSQGKGAGGE DLIMLDIYAI EELRARGLEV TDDSPKYNYH LDSSGSYVFE
501 NTVATVMALR REKMFVEEVS TGQECGVVLD KTCFYAEQGG QIYDEGYLVK
551 VDDSSEDKTE FTVKNAQVRG GYVLHIGTIY GDLKVGDQVW LFIDEPRRRP
601 IMSNHTATHI LNFALRSVLG EADQKGSLVA PDRLRFDFTA KGAMSTQQIK
651 KAEEIANEMI EAAKAVYTQD CPLAAAKAIQ GLRAVFDETY PDPVRVVSIG
701 VPVSELLDDP SGPAGSLTSV EFCGGTHLRN SSHAGAFVIV TEEAIAKGIR
751 RIVAVTGAEA QKALRKAESL KKCLSVMEAK VKAQTAPNKD VQREIADLGE
801 ALATAVIPQW QKDELRETLK SLKKVMDDLD RASKADVQKR VLEKTKQFID
851 SNPNQPLVIL EMESGASAKA LNEALKLFKM HSPQTSAMLF TVDNEAGKIT
901 CLCQVPQNAA NRGLKASEWV QQVSGLMDGK GGGKDVSAQA TGKNVGCLQE
```

951 ALQLATSFAQ LRLGDVKN

Residue Number Increasing Mass Decreasing Mass

Start - End	Observed	Mr(expt)	Mr(calc)	Delta	Miss	Sequence
14 - 20	972.5318	971.5245	971.5228	0.0017	1	FIDFFKR (Ions score 9)
14 - 20	972.5318	971.5245	971.5228	0.0017	1	FIDFFKR (No match)
304 - 320	1713.8839	1712.8766	1712.8805	-0.0039	0	TITVALADGGRPDNTGR (Ions score 29)
304 - 320	1713.8839	1712.8766	1712.8805	-0.0039	0	TITVALADGGRPDNTGR (No match)
684 - 695	1408.6746	1407.6673	1407.6670	0.0003	0	AVFDETYPDPVR (Ions score 39)
684 - 695	1408.6746	1407.6673	1407.6670	0.0003	0	AVFDETYPDPVR (No match)
899 - 912	1644.7925	1643.7852	1643.7872	-0.0019	0	ITCLCQVPQNAANR (No match)
899 - 912	1644.7925	1643.7852	1643.7872	-0.0019	0	ITCLCQVPQNAANR (Ions score 42)

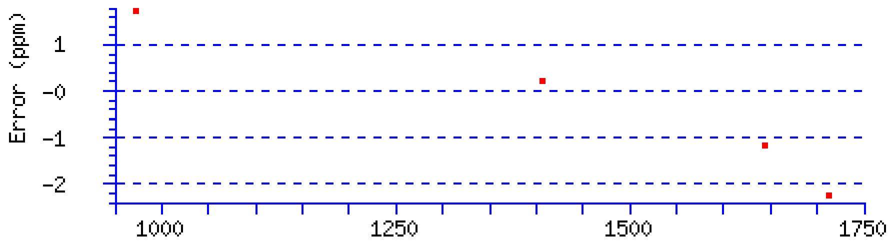

RMS error 1 ppm

Spot 112

Mascot Search Results

Protein View

Match to: **gi|292160** Score: **119** Expect: **2.4e-007**
heat shock protein 70

Nominal mass (M_r): **79858**; Calculated pI value: **5.13**
NCBI BLAST search of gi|292160 against nr
Unformatted sequence string for pasting into other applications

Taxonomy: Homo sapiens

Fixed modifications: Carbamidomethyl (C)
Variable modifications: Oxidation (M)
Cleavage by Trypsin: cuts C-term side of KR unless next residue is P
Sequence Coverage: **9%**

Matched peptides shown in **Bold Red**

```
  1 MSVVGIDLGF QSCYVAVARA GGIETIANEY SDRCTPACIS FGPKNRSIGA
 51 AAKSQVISNA KNTVQGFKRF HGRAFSDPFV EAEKSNLAYD IVQWPTGLTG
101 IKVTYMEEER NFTTEQVTAM LLSKLKETAE SVLKKPVVDC VVSVPCFYTD
151 AERRSVMDAT QIAGLNCLRL MNETTAVALA YGIYKQDLPR LEEKPRNVVF
201 VDMGHSAYQV SVCAFNRGKL KVLATAFDTT LGGRKFDEVL VNHFCEEFGK
251 KYKLDIKSKI RALLRLSQEC EKLKKLMSAN ASDLPLSIEC FMNDVDVSGT
301 MNRGKFLEMC NDLLARVEPP LRSVLEQTKL KKEDIYAVEI VGGATRIPAV
351 KEKISKFFGK ELSTTLNADE AVTRGCALQC AILSPAFKVR EFSITDVVPY
401 PISLRWNSPA EEGSSDCEVF SKNHAAPFSK VLTFYRKEPF TLEAYYSSPQ
451 DLPYPDPAIA QFSVQKVTPQ SDGSSSKVKV KVRVNVHGIF SVSSASLVEV
501 HKSEENEEPM ETDQNAKEEE KMQVDQEEPH VEEQQQQTPA ENKAESEEME
551 TSQAGSKDKK MDQPPQCQEG KSEDQYCGPA NRESAIWQID REMLNLYIEN
601 EGKMIMQDKL EKERNDAKNA VEEYVYEMRD KLSGEYEKFV SEDDRNSFTL
651 KLEDTENWLY EDGEDQPKQV YVDKLAELKN LGQPIKIRFQ ESEERPNYLK
701 N
```

Residue Number Increasing Mass Decreasing Mass

Start - End	Observed	Mr(expt)	Mr(calc)	Delta	Miss	Sequence

20 - 33	1495.7130	1494.7057	1494.6950	0.0107	0	AGGIETIANEYSDR	(No match)
20 - 33	1495.7130	1494.7057	1494.6950	0.0107	0	AGGIETIANEYSDR	(Ions score 45)
222 - 234	1321.7198	1320.7125	1320.7037	0.0088	0	VLATAFDTTLGGR	(No match)
391 - 405	1735.9338	1734.9265	1734.9192	0.0073	0	EFSITDVVPYPISLR	(Ions score 28)
391 - 405	1735.9338	1734.9265	1734.9192	0.0073	0	EFSITDVVPYPISLR	(No match)
619 - 629	1418.6329	1417.6256	1417.6183	0.0073	0	NAVEEYVYEMR Oxidation (M)	(No match)
619 - 629	1418.6329	1417.6256	1417.6183	0.0073	0	NAVEEYVYEMR Oxidation (M)	(No match)
639 - 651	1557.7637	1556.7564	1556.7470	0.0094	1	FVSEDDRNSFTLK	(No match)
639 - 651	1557.7637	1556.7564	1556.7470	0.0094	1	FVSEDDRNSFTLK	(No match)

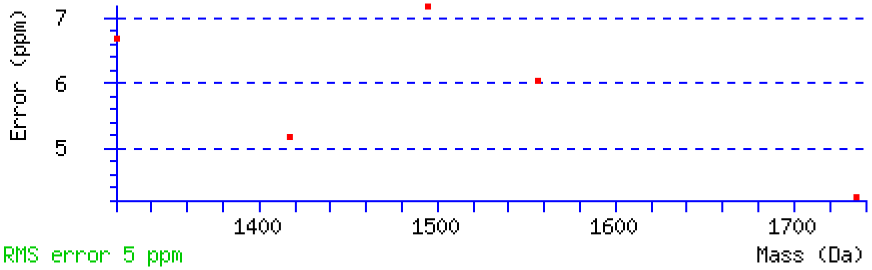

Mascot: http://www.matrixscience.com/

Spot 113

{MATRIX}{SCIENCE} Mascot Search Results

Protein View

Match to: **gi|109658548** Score: **447** Expect: **3.8e-040**
Coronin 7 [Homo sapiens]

Nominal mass (M_r): **101626**; Calculated pI value: **5.51**
NCBI BLAST search of gi|109658548 against nr
Unformatted sequence string for pasting into other applications

Taxonomy: Homo sapiens
Links to retrieve other entries containing this sequence from NCBI Entrez:
gi|109658696 from Homo sapiens
gi|119605713 from Homo sapiens

Fixed modifications: Carbamidomethyl (C)
Variable modifications: Oxidation (M)
Cleavage by Trypsin: cuts C-term side of KR unless next residue is P
Sequence Coverage: **28%**

Matched peptides shown in **Bold Red**

1 MNRFRVSK**FR** **HTEARPPRRE** **SWISDIR**AGT APSCRNHIK**S** **SCSLIAFNSD**
51 **RPGVLGIVPL** **QGQGEDK**RRV AHLGCHSDLV TDLDFSPFDD FLLATGSADR
101 TVKLWRLPGP GQALPSAPGV VLGPEDLPVE VLQFHPTSDG ILVSAAGTTV
151 KVWDAAKQQP LTELAAHGDL VQSAVWSRDG ALVGTACKDK QLRIFDPRTK
201 PR**ASQSTQAH** **ENSR**DSRLAW MGTWEHLVST GFNQMREREV KLWDTRFFSS
251 ALASLTLDTS LGCLVPLLDP DSGLLVLAGK GERQLYCYEV VPQQPALSPV
301 TQCVLESVLR GAALVPR**QAL** **AVMSCEVLRV** **LQLSDTAIVP** **IGYHVPR**KAV
351 EFHEDLFPDT AGCVPATDPH SWWAGDNQQV QKVSLNPACR PHPSFTSCLV
401 PPAEPLPDTA QPAVMETPVG DADASEGFSS PPSSLTSPST PSSLGPSLSS
451 TSGIGTSPSL R**SLQSLLGPS** **SKFRHAQGTV** **LHR**DSHITNL K**GLNLTTPGE**
501 **SDGFCANKLR** **VAVPLLSSGG** **QVAVLELR**KP GRLPDTALPT LQNGAAVTDL
551 AWDPFDPHR**L** **AVAGEDAR**IR LWR**VPAEGLE** **EVLTTPETVL** **TGHTEK**ICSL
601 R**FHPLAANVL** **ASSSYDLTVR** IWDLQAGADR LKLQGHQDQI FSLAWSPDGQ
651 QLATVCKDGR **VRVYRPRSGP** **EPLQEGPGPK** GGRGARIVWV CDGR**CLLVSG**
701 **FDSQSER**QLL LYEAEALAGG PLAVLGLDVA PSTLLPSYDP DTGLVLLTGK
751 GDTRVFLYEL LPESPFFLEC NSFTSPDPHK GLVLLPK**TEC** **DVR**EVELMRC
801 LR**LRQSSLEP** **VAFR**LPRVRK EFFQDDVFPD TAVIWEPVLS AEAWLQGANG
851 QPWLLSLQPP DMSPVSQAPR EAPAR**RAPSS** **AQYLEEK**SDQ QKKEELLNAM
901 VAKLGNREDP LPQDSFEGVD EDEWD

Residue Number Increasing Mass Decreasing Mass

Start - End	Observed	Mr(expt)	Mr(calc)	Delta	Miss	Sequence
9 - 18	1266.6770	1265.6697	1265.6741	-0.0044	1	FRHTEARPPR (No match)
11 - 18	963.5068	962.4995	962.5046	-0.0051	0	HTEARPPR (No match)
11 - 19	1119.6022	1118.5949	1118.6057	-0.0108	1	HTEARPPRR (No match)
20 - 27	1005.5034	1004.4961	1004.4927	0.0034	0	ESWISDIR (No match)
40 - 67	2944.4392	2943.4319	2943.4759	-0.0440	0	SSCSLIAFNSDRPGVLGIVPLQGQGEDK (No match)
203 - 214	1315.6018	1314.5945	1314.5912	0.0033	0	ASQSTQAHENSR (No match)
318 - 329	1392.6958	1391.6885	1391.6900	-0.0015	0	QALAVMSCEVLR Oxidation (M) (No match)
330 - 347	1978.0908	1977.0835	1977.1047	-0.0212	0	VLQLSDTAIVPIGYHVPR (No match)
330 - 347	1978.0908	1977.0835	1977.1047	-0.0212	0	VLQLSDTAIVPIGYHVPR (Ions score 93)
462 - 472	1116.5676	1115.5603	1115.6186	-0.0583	0	SLQSLLGPSSK (No match)
473 - 483	1321.7188	1320.7115	1320.7163	-0.0048	1	FRHAQGTVLHR (No match)
475 - 483	1018.5452	1017.5379	1017.5468	-0.0088	0	HAQGTVLHR (No match)
492 - 508	1780.8383	1779.8310	1779.8097	0.0213	0	GLNLTTPGESDGFCANK (No match)
492 - 510	2049.9797	2048.9724	2048.9949	-0.0224	1	GLNLTTPGESDGFCANKLR (No match)
511 - 528	1808.0455	1807.0382	1807.0566	-0.0184	0	VAVPLLSSGGQVAVLELR (Ions score 55)
511 - 528	1808.0455	1807.0382	1807.0566	-0.0184	0	VAVPLLSSGGQVAVLELR (No match)
560 - 568	901.4741	900.4668	900.4664	0.0004	0	LAVAGEDAR (No match)
574 - 596	2450.2512	2449.2439	2449.2587	-0.0148	0	VPAEGLEEVLTTPETVLTGHTEK (No match)
602 - 620	2061.0549	2060.0476	2060.0690	-0.0214	0	FHPLAANVLASSSYDLTVR (No match)
602 - 620	2061.0549	2060.0476	2060.0690	-0.0214	0	FHPLAANVLASSSYDLTVR (Ions score 35)
661 - 667	945.5675	944.5602	944.5668	-0.0065	1	VRVYRPR (No match)
668 - 680	1292.6378	1291.6305	1291.6408	-0.0102	0	SGPEPLQEGPGPK (No match)
695 - 707	1497.6863	1496.6790	1496.6929	-0.0139	0	CLLVSGFDSQSER (No match)
695 - 707	1497.6863	1496.6790	1496.6929	-0.0139	0	CLLVSGFDSQSER (Ions score 73)
788 - 793	779.3379	778.3306	778.3279	0.0027	0	TECDVR (No match)
803 - 814	1402.7660	1401.7587	1401.7728	-0.0141	1	LRQSSLEPVAFR (No match)
805 - 814	1133.5928	1132.5855	1132.5876	-0.0021	0	QSSLEPVAFR (No match)
876 - 887	1378.6868	1377.6795	1377.6888	-0.0093	1	RAPSSAQYLEEK (No match)

Mascot: http://www.matrixscience.com/

Spot 114

Mascot Search Results

Protein View

Match to: **gi|119605714** Score: **145** Expect: **6.1e-010**
hCG1787779, isoform CRA_b [Homo sapiens]

Nominal mass (M_r): **77412**; Calculated pI value: **5.04**
NCBI BLAST search of gi|119605714 against nr
Unformatted sequence string for pasting into other applications

Taxonomy: Homo sapiens
Links to retrieve other entries containing this sequence from NCBI Entrez:
gi|119605716 from Homo sapiens

Fixed modifications: Carbamidomethyl (C)
Variable modifications: Oxidation (M)
Cleavage by Trypsin: cuts C-term side of KR unless next residue is P
Sequence Coverage: **12%**

Matched peptides shown in **Bold Red**

```
  1 MGTWEHLVST GFNQMREREV KLWDTRFFSS ALASLTLDTS LGCLVPLLDP
 51 DSGLLVLAGK GERQLYCYEV VPQQPALSPV TQCVLESVLR GAALVPRQAL
101 AVMSCEVLRV LQLSDTAIVP IGYHVPRKAV EFHEDLFPDT AGCVPATDPH
151 SWWAGDNQQV QKVSLNPACR PHPSFTSCLV PPAEPLPDTA QPAVMETPVG
201 DADASEGFSS PPSSLTSPST PSSLGPSLSS TSGIGTSPSL RSLQSLLGPS
251 SKFRHAQGTV LHRDSHITNL KGLNLTTPGE SDGFCANKLR VAVPLLSSGG
301 QVAVLELRKP GRLPDTALPT LQNGAAVTDL AWDPFDPHRL AVAGEDARIR
351 LWRVPAEGLE EVLTTPETVL TGHTEKICSL RFHPLAANVL ASSSYDLTVR
401 IWDLQAGADR LKLQGHQDQI FSLAWSPDGQ QLATVCKDGR VRVYRPRSGP
451 EPLQEGPGPK GGRGARIVWV CDGRCLLVSG FDSQSERQLL LYEAEALAGG
501 PLAVLGLDVA PSTLLPSYDP DTGLVLLTGK GDTRVFLYEL LPESPFFLEC
551 NSFTSPDPHK GLVLLPKTEC DVREVELMRC LRLRQSSLEP VAFRLPRVRK
601 EFFQDDVFPD TAVIWEPVLS AEAWLQGANG QPWLLSLQPP DMSPVSQAPR
651 EAPARRAPSS AQYLEEKSDQ QKKEELLNAM VAKLGNREDP LPQDSFEGVD
701 EDEWD
```

Residue Number Increasing Mass Decreasing Mass

Start - End	Observed	Mr(expt)	Mr(calc)	Delta	Miss	Sequence
110 - 127	1978.1873	1977.1800	1977.1047	0.0753	0	VLQLSDTAIVPIGYHVPR (Ions score 33)
110 - 127	1978.1873	1977.1800	1977.1047	0.0753	0	VLQLSDTAIVPIGYHVPR (No match)
253 - 263	1321.7769	1320.7696	1320.7163	0.0533	1	FRHAQGTVLHR (No match)
291 - 308	1808.1357	1807.1284	1807.0566	0.0718	0	VAVPLLSSGGQVAVLELR (Ions score 27)
291 - 308	1808.1357	1807.1284	1807.0566	0.0718	0	VAVPLLSSGGQVAVLELR (No match)
382 - 400	2061.1589	2060.1516	2060.0690	0.0826	0	FHPLAANVLASSSYDLTVR (Ions score 16)
382 - 400	2061.1589	2060.1516	2060.0690	0.0826	0	FHPLAANVLASSSYDLTVR (No match)
475 - 487	1497.7557	1496.7484	1496.6929	0.0555	0	CLLVSGFDSQSER (No match)
475 - 487	1497.7557	1496.7484	1496.6929	0.0555	0	CLLVSGFDSQSER (Ions score 12)
583 - 594	1402.8369	1401.8296	1401.7728	0.0568	1	LRQSSLEPVAFR (No match)
585 - 594	1133.6416	1132.6343	1132.5876	0.0467	0	QSSLEPVAFR (No match)

Mascot: http://www.matrixscience.com/

Spot 118

Mascot Search Results

Protein View

Match to: **gi|4507677** Score: **836** Expect: **4.8e-079**
tumor rejection antigen (gp96) 1 [Homo sapiens]

Nominal mass (M_r): **92696**; Calculated pI value: **4.76**
NCBI BLAST search of gi|4507677 against nr
Unformatted sequence string for pasting into other applications

Taxonomy: Homo sapiens
Links to retrieve other entries containing this sequence from NCBI Entrez:
gi|119360 from Homo sapiens
gi|37261 from Homo sapiens
gi|44890631 from Homo sapiens
gi|119618130 from Homo sapiens

Fixed modifications: Carbamidomethyl (C)
Variable modifications: Oxidation (M)
Cleavage by Trypsin: cuts C-term side of KR unless next residue is P
Sequence Coverage: **51%**

Matched peptides shown in **Bold Red**

```
  1 MRALWVLGLC CVLLTFGSVR ADDEVDVDGT VEEDLGKSRE GSRTDDEVVQ
 51 REEEAIQLDG LNASQIRELR EKSEKFAFQA EVNRMMKLII NSLYKNKEIF
101 LRELISNASD ALDKIRLISL TDENALSGNE ELTVKIKCDK EKNLLHVTDT
151 GVGMTREELV KNLGTIAKSG TSEFLNKMTE AQEDGQSTSE LIGQFGVGFY
201 SAFLVADKVI VTSKHNNDTQ HIWESDSNEF SVIADPRGNT LGRGTTITLV
251 LKEEASDYLE LDTIKNLVKK YSQFINFPIY VWSSKTETVE EPMEEEEAAK
301 EEKEESDDEA AVEEEEEEKK PKTKKVEKTV WDWELMNDIK PIWQRPSKEV
351 EEDEYKAFYK SFSKESDDPM AYIHFTAEGE VTFKSILFVP TSAPRGLFDE
401 YGSKKSDYIK LYVRRVFITD DFHDMMPKYL NFVKGVVDSD DLPLNVSRET
451 LQQHKLLKVI RKKLVRKTLD MIKKIADDKY NDTFWKEFGT NIKLGVIEDH
501 SNRTRLAKLL RFQSSHHPTD ITSLDQYVER MKEKQDKIYF MAGSSRKEAE
551 SSPFVERLLK KGYEVIYLTE PVDEYCIQAL PEFDGKRFQN VAKEGVKFDE
601 SEKTKESREA VEKEFEPLLN WMKDKALKDK IEKAVVSQRL TESPCALVAS
651 QYGWSGNMER IMKAQAYQTG KDISTNYYAS QKKTFEINPR HPLIRDMLRR
701 IKEDEDDKTV LDLAVVLFET ATLRSGYLLP DTKAYGDRIE RMLRLSLNID
751 PDAKVEEEPE EEPEETAEDT TEDTEQDEDE EMDVGTDEEE ETAKESTAEK
801 DEL
```

Residue Number Increasing Mass Decreasing Mass

Start - End	Observed	Mr(expt)	Mr(calc)	Delta	Miss	Sequence
44 - 51	961.4530	960.4457	960.4512	-0.0055	0	TDDEVVQR (No match)
44 - 67	2728.3242	2727.3169	2727.3310	-0.0141	1	TDDEVVQREEEAIQLDGLNASQIR (No match)
52 - 67	1785.8612	1784.8539	1784.8904	-0.0365	0	EEEAIQLDGLNASQIR (No match)
76 - 84	1081.5348	1080.5275	1080.5352	-0.0076	0	FAFQAEVNR (Ions score 58)
76 - 84	1081.5348	1080.5275	1080.5352	-0.0076	0	FAFQAEVNR (No match)
88 - 95	963.5731	962.5658	962.5800	-0.0142	0	LIINSLYK (No match)
96 - 102	919.5294	918.5221	918.5286	-0.0065	1	NKEIFLR (No match)
103 - 116	1544.8108	1543.8035	1543.8205	-0.0170	1	ELISNASDALDKIR (No match)
117 - 135	2046.0455	2045.0382	2045.0528	-0.0145	0	LISLTDENALSGNEELTVK (No match)
143 - 156	1513.7639	1512.7566	1512.7718	-0.0152	0	NLLHVTDTGVGMTR (No match)
143 - 156	1529.7529	1528.7456	1528.7667	-0.0211	0	NLLHVTDTGVGMTR Oxidation (M) (No match)
143 - 161	2112.0764	2111.0691	2111.1044	-0.0353	1	NLLHVTDTGVGMTREELVK (No match)
143 - 161	2128.0784	2127.0711	2127.0993	-0.0282	1	NLLHVTDTGVGMTREELVK Oxidation (M) (No match)
169 - 177	982.5028	981.4955	981.4767	0.0189	0	SGTSEFLNK (No match)
304 - 322	2219.9480	2218.9407	2218.9600	-0.0193	1	EESDDEAAVEEEEEEKKPK (No match)
329 - 348	2542.2605	2541.2532	2541.2838	-0.0306	0	TVWDWELMNDIKPIWQRPSK (No match)
349 - 356	1040.4302	1039.4229	1039.4345	-0.0116	0	EVEEDEYK (No match)
365 - 384	2303.0154	2302.0081	2302.0099	-0.0018	0	ESDDPMAYIHFTAEGEVTFK Oxidation (M) (No match)
385 - 395	1187.6678	1186.6605	1186.6710	-0.0104	0	SILFVPTSAPR (No match)
385 - 395	1187.6678	1186.6605	1186.6710	-0.0104	0	SILFVPTSAPR (Ions score 88)
396 - 404	1015.4619	1014.4546	1014.4658	-0.0111	0	GLFDEYGSK (Ions score 45)
396 - 404	1015.4619	1014.4546	1014.4658	-0.0111	0	GLFDEYGSK (No match)
396 - 405	1143.5543	1142.5470	1142.5607	-0.0137	1	GLFDEYGSKK (No match)
415 - 428	1767.8047	1766.7974	1766.8120	-0.0146	1	RVFITDDFHDMMPK Oxidation (M) (No match)
415 - 428	1783.7983	1782.7910	1782.8069	-0.0159	1	RVFITDDFHDMMPK 2 Oxidation (M) (No match)
416 - 428	1611.7047	1610.6974	1610.7109	-0.0135	0	VFITDDFHDMMPK Oxidation (M) (No match)
416 - 428	1627.7017	1626.6944	1626.7058	-0.0114	0	VFITDDFHDMMPK 2 Oxidation (M) (No match)
416 - 434	2376.0476	2375.0403	2375.1330	-0.0926	1	VFITDDFHDMMPKYLNFVK Oxidation (M) (No match)
429 - 434	783.4333	782.4260	782.4326	-0.0066	0	YLNFVK (No match)
435 - 448	1485.7361	1484.7288	1484.7470	-0.0182	0	GVVDSDDLPLNVSR (No match)
435 - 448	1485.7361	1484.7288	1484.7470	-0.0182	0	GVVDSDDLPLNVSR (Ions score 112)
494 - 503	1139.5692	1138.5619	1138.5730	-0.0111	0	LGVIEDHSNR (No match)
494 - 505	1396.6803	1395.6730	1395.7218	-0.0488	1	LGVIEDHSNRTR (No match)
512 - 530	2260.0283	2259.0210	2259.0556	-0.0346	0	FQSSHHPTDITSLDQYVER (No match)
512 - 530	2260.0283	2259.0210	2259.0556	-0.0346	0	FQSSHHPTDITSLDQYVER (Ions score 108)
538 - 546	1031.4910	1030.4837	1030.4906	-0.0068	0	IYFMAGSSR (No match)
538 - 546	1047.4797	1046.4724	1046.4855	-0.0130	0	IYFMAGSSR Oxidation (M) (No match)
547 - 557	1278.6193	1277.6120	1277.6251	-0.0131	1	KEAESSPFVER (No match)
548 - 557	1150.5283	1149.5210	1149.5302	-0.0091	0	EAESSPFVER (No match)
562 - 587	3104.4753	3103.4680	3103.4847	-0.0167	1	GYEVIYLTEPVDEYCIQALPEFDGKR (No match)
594 - 603	1167.5415	1166.5342	1166.5455	-0.0112	1	EGVKFDESEK (No match)
598 - 605	983.4623	982.4550	982.4607	-0.0057	1	FDESEKTK (No match)
609 - 623	1878.9188	1877.9115	1877.9232	-0.0117	1	EAVEKEFEPLLNWMK Oxidation (M) (No match)

640 - 660	2356.0376	2355.0303	2355.0623	-0.0320	0	**LTESPCALVASQYGWSGNMER**	(No match)
640 - 660	2372.0488	2371.0415	2371.0572	-0.0157	0	**LTESPCALVASQYGWSGNMER**	Oxidation (M) (No match)
672 - 683	1417.6803	1416.6730	1416.6885	-0.0154	1	**DISTNYYASQKK**	(No match)
683 - 690	1004.5464	1003.5391	1003.5450	-0.0059	1	**KTFEINPR**	(No match)
684 - 690	876.4523	875.4450	875.4501	-0.0050	0	**TFEINPR**	(No match)
725 - 733	993.5141	992.5068	992.5178	-0.0110	0	**SGYLLPDTK**	(No match)
734 - 741	979.4870	978.4797	978.4882	-0.0085	1	**AYGDRIER**	(No match)
745 - 754	1085.5676	1084.5603	1084.5764	-0.0161	0	**LSLNIDPDAK**	(No match)
795 - 803	1021.4555	1020.4482	1020.4611	-0.0129	1	**ESTAEKDEL**	(No match)

Mascot: http://www.matrixscience.com/

Spot 119

Mascot Search Results

Protein View

Match to: **gi|24485** Score: **220** Expect: **1.9e-017**
unnamed protein product [Homo sapiens]

Nominal mass (M_r): **91182**; Calculated pI value: **5.76**
NCBI BLAST search of gi|24485 against nr
Unformatted sequence string for pasting into other applications

Taxonomy: Homo sapiens

Fixed modifications: Carbamidomethyl (C)
Variable modifications: Oxidation (M)
Cleavage by Trypsin: cuts C-term side of KR unless next residue is P
Sequence Coverage: **14%**

Matched peptides shown in **Bold Red**

```
  1 MVELNGNQPM EIKVLGPYTF SICDTSNFSD YIRGGIVSQV KVPKKISFKS
 51 LVASLAEPDF VVTDFAKFSR PAQLHIGFQA LHQFCAQHGR PPRPRNEEDA
101 AELVALAQAV NARALPAVQQ NNLDEDLIRK LAYVAAGDLA PINAFIGGLA
151 AQEVMKACSG KFMPIMQWLY FDALECLPED KEVLTEDKCL QRQNRYDGQV
201 AVFGSDLQEK LGKQKYFLVG AGAIGCELLK NFAMIGLGCG EGGEIIVTDM
251 DTIEKSNLNR QFLFRPWDVT KLKSDTAAAA VRQMNPHIRV TSHQNRVGPD
301 TERIYDDDFF QNLDGVANAL DNVDARMYMD RRCVYYRKPL LESGTLGTKG
351 NVQVVIPFLT ESYSSSQDPP EKSIPICTLK NFPNAIEHTL QWARDEFEGL
401 FKQPAENVNQ YLTDPKFVER TLRLAGTQPL EVLEAVQRSL VLQRPQTWAD
451 CVTWACHHWH TQYSNNIRQL LHNFPPDQLT SSGAPFWSGP KRCPHPLTFD
501 VNNPLHLDYV MAAANLFAQT YGLTGSQDRA AVATFLQSVQ VPEFTPKSGV
551 KIHVSDQELQ SANASVDDSR LEELKATLPS PDKLPGFKMY PIDFEKDDDS
601 NFHMDFIVAA SNLRAENYDI PSADRHKSKL IAGKIIPAIA TTTAAVVGLV
651 CLELYKVVQG HRQLDSYKNG FLNLALPFFG FSEPLAAPRH QYYNQEWTLW
701 DRFEVQGLQP NGEEMTLKQF LDYFKTEHKL EITMLSQGVS MLYSFFMPAA
751 KLKERLDQPM TEIVSRVSKR KLGRHVRALV LELCCNDESG EDVEVPYVRY
801 TIR
```

Residue Number Increasing Mass Decreasing Mass

Start - End	Observed	Mr(expt)	Mr(calc)	Delta	Miss	Sequence
14 - 33	2355.0923	2354.0850	2354.0888	-0.0038	0	VLGPYTFSICDTSNFSDYIR (No match)
96 - 113	1883.9370	1882.9297	1882.9384	-0.0086	0	NEEDAAELVALAQAVNAR (No match)
96 - 113	1883.9370	1882.9297	1882.9384	-0.0086	0	NEEDAAELVALAQAVNAR (Ions score 29)
114 - 129	1808.9458	1807.9385	1807.9428	-0.0042	0	ALPAVQQNNLDEDLIR (No match)
304 - 326	2600.1890	2599.1817	2599.1826	-0.0009	0	IYDDDFFQNLDGVANALDNVDAR (Ions score 108)
304 - 326	2600.1890	2599.1817	2599.1826	-0.0009	0	IYDDDFFQNLDGVANALDNVDAR (No match)
424 - 438	1623.8995	1622.8922	1622.8991	-0.0069	0	LAGTQPLEVLEAVQR (Ions score 29)
424 - 438	1623.8995	1622.8922	1622.8991	-0.0069	0	LAGTQPLEVLEAVQR (No match)
730 - 751	2512.2246	2511.2173	2511.2099	0.0075	0	LEITMLSQGVSMLYSFFMPAAK 3 Oxidation (M) (No match)

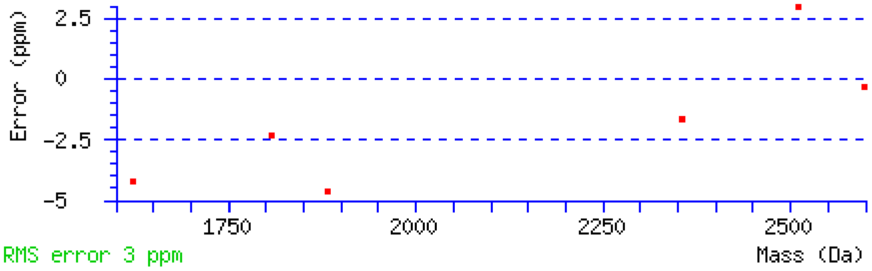

RMS error 3 ppm

Mascot: http://www.matrixscience.com/

Spot 120

Mascot Search Results

Protein View

Match to: **gi|24485** Score: **344** Expect: **7.7e-030**
unnamed protein product [Homo sapiens]

Nominal mass (M_r): **91182**; Calculated pI value: **5.76**
NCBI BLAST search of gi|24485 against nr
Unformatted sequence string for pasting into other applications

Taxonomy: Homo sapiens

Fixed modifications: Carbamidomethyl (C)
Variable modifications: Oxidation (M)
Cleavage by Trypsin: cuts C-term side of KR unless next residue is P
Sequence Coverage: **14%**

Matched peptides shown in **Bold Red**

```
  1 MVELNGNQPM EIKVLGPYTF SICDTSNFSD YIRGGIVSQV KVPKKISFKS
 51 LVASLAEPDF VVTDFAKFSR PAQLHIGFQA LHQFCAQHGR PPRPRNEEDA
101 AELVALAQAV NARALPAVQQ NNLDEDLIRK LAYVAAGDLA PINAFIGGLA
151 AQEVMKACSG KFMPIMQWLY FDALECLPED KEVLTEDKCL QRQNRYDGQV
201 AVFGSDLQEK LGKQKYFLVG AGAIGCELLK NFAMIGLGCG EGGEIIVTDM
251 DTIEKSNLNR QFLFRPWDVT KLKSDTAAAA VRQMNPHIRV TSHQNRVGPD
301 TERIYDDDFF QNLDGVANAL DNVDARMYMD RRCVYYRKPL LESGTLGTKG
351 NVQVVIPFLT ESYSSSQDPP EKSIPICTLK NFPNAIEHTL QWARDEFEGL
401 FKQPAENVNQ YLTDPKFVER TLRLAGTQPL EVLEAVQRSL VLQRPQTWAD
451 CVTWACHHWH TQYSNNIRQL LHNFPPDQLT SSGAPFWSGP KRCPHPLTFD
501 VNNPLHLDYV MAAANLFAQT YGLTGSQDRA AVATFLQSVQ VPEFTPKSGV
551 KIHVSDQELQ SANASVDDSR LEELKATLPS PDKLPGFKMY PIDFEKDDDS
601 NFHMDFIVAA SNLRAENYDI PSADRHKSKL IAGKIIPAIA TTTAAVVGLV
651 CLELYKVVQG HRQLDSYKNG FLNLALPFFG FSEPLAAPRH QYYNQEWTLW
701 DRFEVQGLQP NGEEMTLKQF LDYFKTEHKL EITMLSQGVS MLYSFFMPAA
751 KLKERLDQPM TEIVSRVSKR KLGRHVRALV LELCCNDESG EDVEVPYVRY
801 TIR
```

Residue Number Increasing Mass Decreasing Mass

Start	-	End	Observed	Mr(expt)	Mr(calc)	Delta	Miss	Sequence
14	-	33	2355.0962	2354.0889	2354.0888	0.0001	0	VLGPYTFSICDTSNFSDYIR (Ions score 90)
14	-	33	2355.0962	2354.0889	2354.0888	0.0001	0	VLGPYTFSICDTSNFSDYIR (No match)
96	-	113	1883.9464	1882.9391	1882.9384	0.0008	0	NEEDAAELVALAQAVNAR (Ions score 76)
96	-	113	1883.9464	1882.9391	1882.9384	0.0008	0	NEEDAAELVALAQAVNAR (No match)
114	-	129	1808.9536	1807.9463	1807.9428	0.0036	0	ALPAVQQNNLDEDLIR (No match)
114	-	130	1937.0500	1936.0427	1936.0377	0.0050	1	ALPAVQQNNLDEDLIRK (No match)
304	-	326	2600.2017	2599.1944	2599.1826	0.0118	0	IYDDDFFQNLDGVANALDNVDAR (No match)
304	-	326	2600.2017	2599.1944	2599.1826	0.0118	0	IYDDDFFQNLDGVANALDNVDAR (Ions score 72)
424	-	438	1623.9095	1622.9022	1622.8991	0.0031	0	LAGTQPLEVLEAVQR (Ions score 32)
424	-	438	1623.9095	1622.9022	1622.8991	0.0031	0	LAGTQPLEVLEAVQR (No match)
730	-	751	2512.2332	2511.2259	2511.2099	0.0161	0	LEITMLSQGVSMLYSFFMPAAK 3 Oxidation (M) (No match)

Mascot: http://www.matrixscience.com/

Spot 122

Mascot Search Results

Protein View

Match to: **gi|799177** Score: **277** Expect: **3.8e-023**
100 kDa coactivator

Nominal mass (M_r): **100313**; Calculated pI value: **6.62**
NCBI BLAST search of gi|799177 against nr
Unformatted sequence string for pasting into other applications

Taxonomy: Homo sapiens

Fixed modifications: Carbamidomethyl (C)
Variable modifications: Oxidation (M)
Cleavage by Trypsin: cuts C-term side of KR unless next residue is P
Sequence Coverage: **29%**

Matched peptides shown in **Bold Red**

```
  1 MVLSGCAIIV RGQPRGGPPP ERQINLSNIR AGNLARRAAA TQPDAKDTPD
 51 EPWAFPAREF LRKKLIGKEV CFTIENKTPQ GREYGMIYLG KDTNGENIAE
101 SLVAEGLATR REGMRANNPE QNRLSECEEQ AKAAKKGMWS EGNGSHTIRD
151 LKYTIENPRH FVDSHHQKPV NAIIEHVRDG SVVRALLLPD YYLVTVMLSG
201 IKCPTFRREA DGSETPEPFA AEAKFFTESR LLQRDVQIIL ESCHNQNIVG
251 TILHPNGNIT ELLLKEGFAR CVDWSIAVYT RGAEKLRAAE RFAKERRLRI
301 WRDYVAPTAN LDQKDKQFVA KVMQVLNADA IVVKLNSGDY KTIHLSSIRP
351 PRLEGENTQD KNKKLRPLYD IPYMFEAREF LRKKLIGKKV NVTVDYIRPA
401 SPATETVPAF SERTCATVTI GGINIAEALV SKGLATVIRY RQDDDQRSSH
451 YDELLAAEAR AIKNGKGLHS KKEVPIHRVA DISGDTQKAK QFLPFLQRAG
501 RSEAVVEYVF SGSRLKLYLP KETCLITFLL AGIECPRGAR NLPGLVQEGE
551 PFSEEATLFT KELVLQREVE VEVESMDKAG NFIGWLHIDG ANLSVLLVEH
601 ALSKVHFTAE RSSYYKSLLS AEEAAKQKKE KVWAHYEEQP VEEVMPVLEE
651 KERSASYKPV FVTEITDDLH FYVQDVETGT QFQKLMENMR NDIASHPPVE
701 GSYAPRRGEF CIAKFVDGEW YRARVEKVES PAKIHVFYID YGNREVLPST
751 RLGTLSPAFS TRVLPAQATE YAFAFIQVPQ DDDARTDAVD SVVRDIQNTQ
801 CLLNVEHLSA GCPHVTLQFA DSKGDVGLGL VKEGLVMVEV RKEKQFQKVI
851 TEYLNAQESA KSARLNLWRY GDFRADDADE FGYSR
```

Residue Number Increasing Mass Decreasing Mass

Start - End	Observed	Mr(expt)	Mr(calc)	Delta	Miss	Sequence
23 - 30	957.5529	956.5456	956.5403	0.0053	0	QINLSNIR (No match)
69 - 82	1678.8181	1677.8108	1677.8144	-0.0036	1	EVCFTIENKTPQGR (No match)
153 - 159	892.4623	891.4550	891.4450	0.0100	0	YTIENPR (No match)
160 - 178	2263.1768	2262.1695	2262.1769	-0.0074	0	HFVDSHHQKPVNAIIEHVR (No match)
225 - 230	786.3865	785.3792	785.3708	0.0085	0	FFTESR (No match)
342 - 352	1276.7462	1275.7389	1275.7411	-0.0022	0	TIHLSSIRPPR (No match)
365 - 378	1799.9089	1798.9016	1798.9076	-0.0059	0	LRPLYDIPYMFEAR Oxidation (M) (No match)
433 - 441	1048.5923	1047.5850	1047.6189	-0.0338	1	GLATVIRYR (No match)
440 - 447	1095.4812	1094.4739	1094.4741	-0.0002	1	YRQDDDQR (No match)
448 - 460	1461.6948	1460.6875	1460.6895	-0.0020	0	SSHYDELLAAEAR (No match)
491 - 498	1048.5923	1047.5850	1047.5865	-0.0015	0	QFLPFLQR (No match)
502 - 514	1429.6932	1428.6859	1428.6884	-0.0025	0	SEAVVEYVFSGSR (No match)
502 - 514	1429.6932	1428.6859	1428.6884	-0.0025	0	SEAVVEYVFSGSR (Ions score 10)
522 - 537	1892.9536	1891.9463	1891.9535	-0.0072	0	ETCLITFLLAGIECPR (No match)
541 - 561	2306.1453	2305.1380	2305.1477	-0.0097	0	NLPGLVQEGEPFSEEATLFTK (No match)
605 - 611	859.4485	858.4412	858.4348	0.0065	0	VHFTAER (No match)
691 - 706	1709.8181	1708.8108	1708.8168	-0.0060	0	NDIASHPPVEGSYAPR (Ions score 29)
691 - 706	1709.8181	1708.8108	1708.8168	-0.0060	0	NDIASHPPVEGSYAPR (No match)
707 - 714	980.5012	979.4939	979.4909	0.0030	1	RGEFCIAK (No match)
734 - 744	1396.7000	1395.6927	1395.6935	-0.0007	0	IHVFYIDYGNR (Ions score 22)
734 - 744	1396.7000	1395.6927	1395.6935	-0.0007	0	IHVFYIDYGNR (No match)
734 - 751	2179.1331	2178.1258	2178.1221	0.0037	1	IHVFYIDYGNREVLPSTR (No match)
752 - 762	1149.6289	1148.6216	1148.6189	0.0027	0	LGTLSPAFSTR (No match)
833 - 841	1031.5670	1030.5597	1030.5481	0.0117	0	EGLVMVEVR (No match)
833 - 841	1031.5670	1030.5597	1030.5481	0.0117	0	EGLVMVEVR (No match)
849 - 861	1465.7413	1464.7340	1464.7460	-0.0119	0	VITEYLNAQESAK (No match)
870 - 885	1883.7739	1882.7666	1882.7757	-0.0091	1	YGDFRADDADEFGYSR (No match)
875 - 885	1245.5004	1244.4931	1244.4945	-0.0014	0	ADDADEFGYSR (No match)

Mascot: http://www.matrixscience.com/

Spot 124

Protein View

Match to: **gi|15010550** Score: **282** Expect: **1.2e-023**
heat shock protein gp96 precursor [Homo sapiens]

Nominal mass (M_r): **90309**; Calculated pI value: **4.73**
NCBI BLAST search of gi|15010550 against nr
Unformatted sequence string for pasting into other applications

Taxonomy: Homo sapiens

Fixed modifications: Carbamidomethyl (C)
Variable modifications: Oxidation (M)
Cleavage by Trypsin: cuts C-term side of KR unless next residue is P
Sequence Coverage: **14%**

Matched peptides shown in **Bold Red**

```
  1 DDEVDVDGTV EEDLGKSREG SRTDDEVVQR EEEAIQLDGL NASQIRELRE
 51 KSEKFAFQAE VNRMMKLIIN SLYKNKEIFL RELISNASDA LDKIRLISLT
101 DENALSGNEE LTVKIKCDKE KNLLHVTDTG VGMTREELVK NLGTIAKSGT
151 SEFLNKMTEA QEDGQSSSEL IGQFGVGFYS AFLVADKVIV TSKHNNDTQH
201 IWESDSNEFS VIADPRGNTL GRGTTITLVL KEEASDYLEL DTIKNLVKKY
251 SQFINFPIYV WSSKTETVEE PMEEEEAAKE EKEESDDEAA VEEEEEEKKP
301 KTKKVEKTVW DWELMNDIKP IWQRPSKEVE EDEYKAFYKS FSKESDDPMA
351 YIHFTAEGEV TFKSILFVPT SAPRGLFDEY GSKKSDYIKL YVRRVFIPDD
401 FHDMMPKYLN FVKGVVDSDD LPLNVSRETL QQHKLLKVIR KKLVRKTLDM
451 IKKIADDKYN DTFWKEFGTN IKLGVIEDHS NRTRLAKLLR FQSSHHPTDI
501 TSLDQYVERM KEKQDKIYFM AGSSRKEAES SPFVERLLKK GYEVIYLTEP
551 VDEYCIQALP EFDGKRFQNV AKEGVKFDES EKTKESREAV EKEFEPLLNW
601 MKDKALKDKI EKAVVSQRLT ESPCALVASQ YGWSGNMERI MKAQAYQTGK
651 DISTNYYASQ KKTFEINPRH PLIRDMLRRI KEDEDDKTVL DLAVVLFETA
701 TLRSGYLLPD TKAYGDRIER MLRLSLNIDP DAKVEEEPEE EPEETAEDTT
751 EDTEQDEDEE MDVGTDEEEE TAKESTAEKD EF
```

Residue Number Increasing Mass Decreasing Mass

Start - End	Observed	Mr(expt)	Mr(calc)	Delta	Miss	Sequence
31 - 46	1785.9106	1784.9033	1784.8904	0.0129	0	**EEEAIQLDGLNASQIR** (No match)
55 - 63	1081.5620	1080.5547	1080.5352	0.0196	0	**FAFQAEVNR** (No match)

55 - 63	1081.5620	1080.5547	1080.5352	0.0196	0	**FAFQAEVNR** (Ions score 23)
75 - 81	919.5545	918.5472	918.5286	0.0186	1	**NKEIFLR** (No match)
82 - 95	1544.8419	1543.8346	1543.8205	0.0141	1	**ELISNASDALDKIR** (No match)
364 - 374	1187.6975	1186.6902	1186.6710	0.0193	0	**SILFVPTSAPR** (No match)
364 - 374	1187.6975	1186.6902	1186.6710	0.0193	0	**SILFVPTSAPR** (Ions score 32)
375 - 383	1015.4856	1014.4783	1014.4658	0.0126	0	**GLFDEYGSK** (No match)
375 - 383	1015.4856	1014.4783	1014.4658	0.0126	0	**GLFDEYGSK** (Ions score 19)
408 - 413	783.4723	782.4650	782.4326	0.0324	0	**YLNFVK** (No match)
414 - 427	1485.7656	1484.7583	1484.7470	0.0113	0	**GVVDSDDLPLNVSR** (Ions score 81)
414 - 427	1485.7656	1484.7583	1484.7470	0.0113	0	**GVVDSDDLPLNVSR** (No match)
491 - 509	2260.0601	2259.0528	2259.0556	-0.0028	0	**FQSSHHPTDITSLDQYVER** (No match)
491 - 509	2260.0601	2259.0528	2259.0556	-0.0028	0	**FQSSHHPTDITSLDQYVER** (Ions score 40)
713 - 720	979.5122	978.5049	978.4882	0.0167	1	**AYGDRIER** (No match)

Spot 126

Mascot Search Results

Protein View

Match to: **gi|48255891** Score: **168** Expect: **3e-012**
protein kinase C substrate 80K-H isoform 2 [Homo sapiens]

Nominal mass (M_r): **60110**; Calculated pI value: **4.34**
NCBI BLAST search of gi|48255891 against nr
Unformatted sequence string for pasting into other applications

Taxonomy: Homo sapiens
Links to retrieve other entries containing this sequence from NCBI Entrez:
gi|158261889 (no taxonomy information for this entry)

Fixed modifications: Carbamidomethyl (C)
Variable modifications: Oxidation (M)
Cleavage by Trypsin: cuts C-term side of KR unless next residue is P
Sequence Coverage: **28%**

Matched peptides shown in **Bold Red**

1 MLLPLLLLLP MCWAVEVKRP RGVSLTNHHF YDESKPFTCL DGSATIPFDQ
51 VNDDYCDCK**D** **GSDEPGTAAC** **PNGSFHCTNT** **GYKPLYIPSN** **R**VNDGVCDCC
101 DGTDEYNSGV ICENTCKEKG RKER**ESLQQM** **AEVTR**EGFRL KKILIEDWKK
151 AREEKQKKLI ELQAGKK**SLE** **DQVEMLRTVK** **EEAEKPER**EA KEQHQKLWEE
201 QLAAAK**AQQE** **QELAADAFK**E LDDDMDGTVS VTELQTHPEL DTDGDGALSE
251 AEAQALLSGD TQTDATSFYD RVWAAIRDKY R**SEALPTDLP** **APSAPDLTEP**
301 **K**EEQPPVPSS PTEEEEEEEE EEEEEAEEEE EEEDSEVQGE QPKPASPAEE
351 DK**MPPYDEQT** **QAFIDAAQEA** **RNKFEEAERS** **LKDMEESIR**N LEQEISFDFG
401 PNGEFAYLYS QCYELTTNEY VYRLCPFKLV SQKPKLGGSP TSLGTWGSWI
451 GPDHDKFSAM K**YEQGTGCWQ** **GPNR**STTVRL LCGKETMVTS TTEPSRCEYL
501 MELMTPAACP EPPPEAPTED DHDEL

Residue Number Increasing Mass Decreasing Mass

Start - End	Observed	Mr(expt)	Mr(calc)	Delta	Miss	Sequence	
60 - 91	3483.5120	3482.5047	3482.5255	-0.0208	0	DGSDEPGTAACPNGSFHCTNTGYKPLYIPSNR	(No match)
125 - 135	1291.6251	1290.6178	1290.6238	-0.0059	0	ESLQQMAEVTR	(No match)

125 - 135	1307.6252	1306.6179	1306.6187	-0.0007	0	ESLQQMAEVTR	Oxidation (M) (No match)
168 - 177	1219.6055	1218.5982	1218.5914	0.0068	0	SLEDQVEMLR	(No match)
168 - 177	1235.5927	1234.5854	1234.5863	-0.0009	0	SLEDQVEMLR	Oxidation (M) (No match)
168 - 177	1235.5927	1234.5854	1234.5863	-0.0009	0	SLEDQVEMLR	Oxidation (M) (Ions score 12)
178 - 188	1315.6880	1314.6807	1314.6779	0.0029	1	TVKEEAEKPER	(No match)
178 - 188	1315.6880	1314.6807	1314.6779	0.0029	1	TVKEEAEKPER	(Ions score 25)
207 - 219	1448.7007	1447.6934	1447.6943	-0.0008	0	AQQEQELAADAFK	(No match)
282 - 301	2049.0417	2048.0344	2048.0313	0.0031	0	SEALPTDLPAPSAPDLTEPK	(No match)
353 - 371	2180.9871	2179.9798	2179.9844	-0.0046	0	MPPYDEQTQAFIDAAQEAR	(No match)
353 - 371	2196.9988	2195.9915	2195.9793	0.0122	0	MPPYDEQTQAFIDAAQEAR	Oxidation (M) (No match)
372 - 379	1022.4950	1021.4877	1021.4828	0.0049	1	NKFEEAER	(Ions score 14)
372 - 379	1022.4950	1021.4877	1021.4828	0.0049	1	NKFEEAER	(No match)
380 - 389	1223.5948	1222.5875	1222.5863	0.0012	1	SLKDMEESIR	Oxidation (M) (No match)
462 - 474	1552.6581	1551.6508	1551.6524	-0.0016	0	YEQGTGCWQGPNR	(Ions score 24)
462 - 474	1552.6581	1551.6508	1551.6524	-0.0016	0	YEQGTGCWQGPNR	(No match)

Mascot: http://www.matrixscience.com/

Spot 127

Protein View

Match to: **gi|20521069** Score: **108** Expect: **3e-006**
KIAA0525 protein [Homo sapiens]

Nominal mass (M_r): **108595**; Calculated pI value: **5.86**
NCBI BLAST search of gi|20521069 against nr
Unformatted sequence string for pasting into other applications

Taxonomy: Homo sapiens

Fixed modifications: Carbamidomethyl (C)
Variable modifications: Oxidation (M)
Cleavage by Trypsin: cuts C-term side of KR unless next residue is P
Sequence Coverage: **4%**

Matched peptides shown in **Bold Red**

1 SKKMVFLPLK WSLATMSFLL SSLLALLTVS TPSWCQSTEA SPKRSDGTPF
51 PWNKIRLPEY VIPVHYDLLI HANLTTLTFW GTTKVEITAS QPTSTIILHS
101 HHLQISRATL RKGAGERLSE EPLQVLEHPP QEQIALLAPE PLLVGLPYTV
151 VIHYAGNLSE TFHGFYKSTY RTKEGELR**IL ASTQFEPTAA R**MAFPCFDEP
201 AFKASFSIKI RREPRHLAIS NMPLVKSVTV AEGLIEDHFD VTVKMSTYLV
251 AFIISDFESV SKITKSGVKV SVYAVPDKIN QADYALDAAV TLLEFYEDYF
301 SIPYPLPKQD LAAIPDFQSG AMENWGLTTY RESALLFDAE KSSASSKLGI
351 TVTVAHELAH QWFGNLVTME WWNDLWLNEG FAKFMEFVSV SVTHPELKVG
401 DYFFGKCFDA MEVDALNSSH PVSTPVENPA QIREMFDDVS YDKGACILNM
451 LREYLSADAF KSGIVQYLQK HSYKNTKNED LWDSMASICP TDGVKGMDGF
501 CSRSQHSSSS SHWHQEGVDV KTMMNTWTLQ R**GFPLITITV R**GRNVHMKQE
551 HYMKGSDGAP DTGYLWHVPL TFITSKSNMV HRFLLKTKTD VLILPEEVEW
601 IKFNVGMNGY YIVHYEDDGW DSLTGLLKGT HTAVSSNDRA SLINNAFQLV
651 SIGKLSIEKA LDLSLYLKHE TEIMPVFQGL NELIPMYKLM EKRDMNEVET
701 QFKAFLIRLL RDLIDKQTWT DEGSVSEQML RSELLLLACV HNYQPCVQRA
751 EGYFRKWKES NGNLSLPVDV TLAVFAVGAQ STEGWDFLYS KYQFSLSSTE
801 K**SQIEFALCR** TQNKEKLQWL LDESFKGDKI K**TQEFPQILT LIGR**NPVGYP
851 LAWQFLRKNW NKLVQKFELG SSSIAHMVMG TTNQFSTRTR LEEVKGFFSS
901 LKENGSQLRC VQQTIETIEE NIGWMDKNFD KIRVWLQSEK LEHDPEADAT
951 G

Residue Number Increasing Mass Decreasing Mass

Start - End	Observed	Mr(expt)	Mr(calc)	Delta	Miss	Sequence
179 - 191	1404.7208	1403.7135	1403.7408	-0.0273	0	ILASTQFEPTAAR (Ions score 30)
179 - 191	1404.7208	1403.7135	1403.7408	-0.0273	0	ILASTQFEPTAAR (No match)
532 - 541	1116.6552	1115.6479	1115.6702	-0.0223	0	GFPLITITVR (No match)
802 - 810	1123.5382	1122.5309	1122.5491	-0.0182	0	SQIEFALCR (No match)
802 - 810	1123.5382	1122.5309	1122.5491	-0.0182	0	SQIEFALCR (Ions score 8)
832 - 844	1515.8180	1514.8107	1514.8456	-0.0349	0	TQEFPQILTLIGR (No match)
832 - 844	1515.8180	1514.8107	1514.8456	-0.0349	0	TQEFPQILTLIGR (Ions score 35)

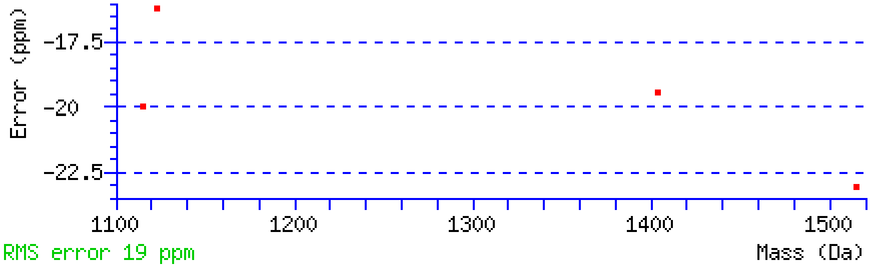

RMS error 19 ppm

Spot 129

Mascot Search Results

Protein View

Match to: **gi|2274968** Score: **111** Expect: **1.5e-006**
Glucosidase II [Homo sapiens]

Nominal mass (M_r): **107289**; Calculated pI value: **5.71**
NCBI BLAST search of gi|2274968 against nr
Unformatted sequence string for pasting into other applications

Taxonomy: Homo sapiens

Fixed modifications: Carbamidomethyl (C)
Variable modifications: Oxidation (M)
Cleavage by Trypsin: cuts C-term side of KR unless next residue is P
Sequence Coverage: **17%**

Matched peptides shown in **Bold Red**

1 MAAVAAVAAR RRRSWASLVL AFLGVCLGIT LAVDRSNFKT CEESSFCKRQ
51 R**SIRPGLSPY** **R**ALLDSLQLG PDSLTVHLIH EVTKVLLVLE LQGLQKNMTR
101 **FRIDELEPR**R PR**YRVPDVLV** **ADPPIAR**LSV SGRDENSVEL TMAEGPYK**II**
151 **LTARPFR**LDL LEDRSLLLSV NAR**GLLEFEH** **QR**APR**VSQGS** **KDPAEGDGAQ**
201 **PEETPR**DGDK PEETQGKAEK DEPGAWEETF KTHSDSKPYG PMSVGLDFSL
251 PGMEHVYGIP EHADNLR**LKV** **TEGGEPYR**LY NLDVFQYELY NPMALYGSVP
301 VLLAHNPHRD LGIFWLNAAE TWVDISSNTA GKTLFGK**MMD** **YLQGSGETPQ**
351 **TDVR**WMSETG IIDVFLLLGP SISDVFRQYA SLTGTQALPP LFSLGYHQSR
401 WNYRDEADVL EVDQGFDDHN LPCDVIWLDI EHADGKRYFT WDPSRFPQPR
451 TMLERLASKR RKLVAIVDPH IKVDSGYRVH EELRNLGLYV KTRDGSDYEG
501 WCWPGSAGYP DFTNPTMRAW WANMFSYDNY EGSAPNLFVW NDMNEPSVFN
551 GPEVTMLKDA QHYGGWEHRD VHNIYGLYVH MATADGLRQR **SGGMERPFVL**
601 **ARAFFAGSQR** FGAVWTGDNT AEWDHLKISI PMCLSLGLVG LSFCGADVGG
651 FFK**NPEPELL** **VR**WYQMGAYQ PFFRAHAHLD TGRREPWLLP SQHNDIIRDA
701 LGQRYSLLPF WYTLLYQAHR EGIPVMRPLW VQYPQDVTTF NIDDQYLLGD
751 ALLVHPVSDS GAHGVQVYLP GQGEVWYDIQ SYQKHHGPQT LYLPVTLSSI
801 PVFQRGGTIV PRWMRVRRSS ECMKDDPITL FVALSPQGTA QGELFLDDGY
851 TFNYQTR**QEF** **LLRR**FSFSGN TLVSSSADPE GHFETPIWIE R**VVIIGAGKP**
901 **AAVVLQTK**GS PESR**LSFQHD** **PETSVLVLR**K PGINVASDWS IHLR

Residue Number Increasing Mass Decreasing Mass

Start - End	Observed	Mr(expt)	Mr(calc)	Delta	Miss	Sequence
52 - 61	1145.6509	1144.6436	1144.6352	0.0084	0	SIRPGLSPYR (Ions score 1)
52 - 61	1145.6509	1144.6436	1144.6352	0.0084	0	SIRPGLSPYR (No match)
101 - 109	1174.6322	1173.6249	1173.6142	0.0108	1	FRIDELEPR (No match)
113 - 127	1680.9664	1679.9591	1679.9358	0.0233	1	YRVPDVLVADPPIAR (No match)
149 - 157	1086.6879	1085.6806	1085.6709	0.0097	0	IILTARPFR (No match)
174 - 182	1128.5881	1127.5808	1127.5723	0.0085	0	GLLEFEHQR (No match)
174 - 182	1128.5881	1127.5808	1127.5723	0.0085	0	GLLEFEHQR (Ions score 18)
186 - 206	2155.0085	2154.0012	2153.9824	0.0188	1	VSQGSKDPAEGDGAQPEETPR (No match)
268 - 278	1248.6748	1247.6675	1247.6509	0.0166	1	LKVTEGGEPYR (No match)
338 - 354	1959.8750	1958.8677	1958.8349	0.0328	0	MMDYLQGSGETPQTDVR 2 Oxidation (M) (No match)
591 - 602	1335.6953	1334.6880	1334.6764	0.0116	0	SGGMERPFVLAR Oxidation (M) (No match)
591 - 602	1335.6953	1334.6880	1334.6764	0.0116	0	SGGMERPFVLAR Oxidation (M) (No match)
603 - 610	883.4454	882.4381	882.4347	0.0034	0	AFFAGSQR (No match)
654 - 662	1066.6003	1065.5930	1065.5818	0.0112	0	NPEPELLVR (No match)
858 - 864	961.5665	960.5592	960.5504	0.0088	1	QEFLLRR (No match)
892 - 908	1664.0646	1663.0573	1663.0395	0.0178	0	VVIIGAGKPAAVVLQTK (No match)
915 - 929	1740.9484	1739.9411	1739.9206	0.0205	0	LSFQHDPETSVLVLR (No match)

Mascot: http://www.matrixscience.com/

Spot 130

{MATRIX SCIENCE} Mascot Search Results

Protein View

Match to: **gi|13699868** Score: **100** Expect: **2.1e-005**
methylenetetrahydrofolate dehydrogenase 1 [Homo sapiens]

Nominal mass (M_r): **102152**; Calculated pI value: **6.75**
NCBI BLAST search of gi|13699868 against nr
Unformatted sequence string for pasting into other applications

Taxonomy: Homo sapiens
Links to retrieve other entries containing this sequence from NCBI Entrez:
gi|14602585 from Homo sapiens
gi|119601263 from Homo sapiens
gi|119601264 from Homo sapiens
gi|123993917 from synthetic construct
gi|124000687 from synthetic construct

Fixed modifications: Carbamidomethyl (C)
Variable modifications: Oxidation (M)
Cleavage by Trypsin: cuts C-term side of KR unless next residue is P
Sequence Coverage: **6%**

Matched peptides shown in **Bold Red**

```
  1 MAPAEILNGK EISAQIRARL KNQVTQLKEQ VPGFTPRLAI LQVGNRDDSN
 51 LYINVKLKAA EEIGIKATHI KLPRTTTESE VMKYITSLNE DSTVHGFLVQ
101 LPLDSENSIN TEEVINAIAP EKDVDGLTSI NAGRLARGDL NDCFIPCTPK
151 GCLELIKETG VPIAGRHAVV VGRSKIVGAP MHDLLLWNNA TVTTCHSKTA
201 HLDEEVNKGD ILVVATGQPE MVKGEWIKPG AIVIDCGINY VPDDKKPNGR
251 KVVGDVAYDE AKERASFITP VPGGVGPMTV AMLMQSTVES AKRFLEKFKP
301 GKWMIQYNNL NLKTPVPSDI DISRSCKPKP IGKLAREIGL LSEEVELYGE
351 TKAKVLLSAL ERLKHRPDGK YVVVTGITPT PLGEGKSTTT IGLVQALGAH
401 LYQNVFACVR QPSQGPTFGI KGGAAGGGYS QVIPMEEFNL HLTGDIHAIT
451 AANNLVAAAI DARIFHELTQ TDKALFNRLV PSVNGVRRFS DIQIRRLKRL
501 GIEKTDPTTL TDEEINRFAR LDIDPETITW QRVLDTNDRF LRKITIGQAP
551 TEKGHTRTAQ FDISVASEIM AVLALTTSLE DMRERLGKMV VASSKKGEPV
601 SAEDLGVSGA LTVLMKDAIK PNLMQTLEGT PVFVHAGPFA NIAHGNSSII
651 ADQIALKLVG PEGFVVTEAG FGADIGMEKF FNIKCRYSGL CPHVVVLVAT
701 VRALKMHGGG PTVTAGLPLP KAYIQENLEL VEKGFSNLKK QIENARMFGI
751 PVVVAVNAFK TDTESELDLI SRLSREHGAF DAVKCTHWAE GGKGALALAQ
```

```
801 AVQRAAQAPS SFQLLYDLKL PVEDKIRIIA QKIYGADDIE LLPEAQHKAE
851 VYTKQGFGNL PICMAKTHLS LSHNPEQKGV PTGFILPIRD IRASVGAGFL
901 YPLVGTMSTM PGLPTRPCFY DIDLDPETEQ VNGLF
```

Residue Number Increasing Mass Decreasing Mass

Start - End	Observed	Mr(expt)	Mr(calc)	Delta	Miss	Sequence
38 - 56	2145.1812	2144.1739	2144.1589	0.0150	1	LAILQVGNRDDSNLYINVK (Ions score 3)
38 - 56	2145.1812	2144.1739	2144.1589	0.0150	1	LAILQVGNRDDSNLYINVK (No match)
151 - 166	1712.9583	1711.9510	1711.9290	0.0220	1	GCLELIKETGVPIAGR (No match)
151 - 166	1712.9583	1711.9510	1711.9290	0.0220	1	GCLELIKETGVPIAGR (Ions score 4)
687 - 702	1769.9908	1768.9835	1768.9657	0.0178	0	YSGLCPHVVVLVATVR (Ions score 29)
687 - 702	1769.9908	1768.9835	1768.9657	0.0178	0	YSGLCPHVVVLVATVR (No match)
879 - 889	1169.7203	1168.7130	1168.6968	0.0163	0	GVPTGFILPIR (No match)
879 - 889	1169.7203	1168.7130	1168.6968	0.0163	0	GVPTGFILPIR (No match)
879 - 892	1553.9319	1552.9246	1552.9089	0.0157	1	GVPTGFILPIRDIR (No match)
879 - 892	1553.9319	1552.9246	1552.9089	0.0157	1	GVPTGFILPIRDIR (Ions score 15)

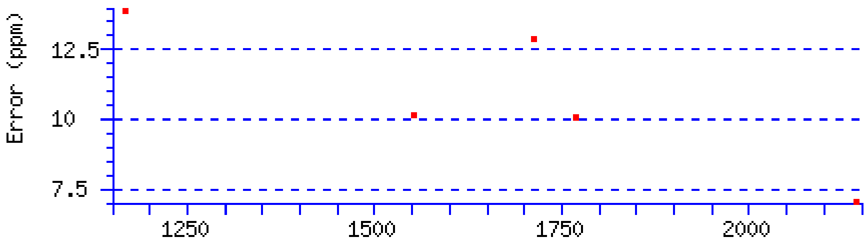

RMS error 11 ppm

Mascot: http://www.matrixscience.com/

Spot 131

{MATRIX SCIENCE} Mascot Search Results

Protein View

Match to: **gi|577295** Score: **78** Expect: **0.0032**
KIAA0088 [Homo sapiens]

Nominal mass (M_r): **107158**; Calculated pI value: **5.71**
NCBI BLAST search of gi|577295 against nr
Unformatted sequence string for pasting into other applications

Taxonomy: Homo sapiens

Fixed modifications: Carbamidomethyl (C)
Variable modifications: Oxidation (M)
Cleavage by Trypsin: cuts C-term side of KR unless next residue is P
Sequence Coverage: **8%**

Matched peptides shown in **Bold Red**

```
  1 AAVAAVAARR RRSWASLVLA FLGVCLGITL AVDRSNFKTC EESSFCKRQR
 51 SIRPGLSPYR ALLDSLQLGP DSLTVHLIHE VTKVLLVLEL QGLQKNMTRF
101 RIDELEPRRP RYRVPDVLVA DPPIARLSVS GRDENSVELT MAEGPYKIIL
151 TARPFRLDLL EDRSLLLSVN ARGLLEFEHQ RAPRVSQGSK DPAEGDGAQP
201 EETPRDGDKP EETQGKAEKD EPGAWEETFK THSDSKPYGP MSVGLDFSLP
251 GMEHVYGIPE HADNLRLKVT EGGEPYRLYN LDVFQYELYN PMALYGSVPV
301 LLAHNPHRDL GIFWLNAAET WVDISSNTAG KTLFGKMMDY LQGSGETPQT
351 DVRWMSETGI IDVFLLLGPS ISDVFRQYAS LTGTQALPPL FSLGYHQSRW
401 NYRDEADVLE VDQGFDDHNL PCDVIWLDIE HADGKRYFTW DPSRFPQPRT
451 MLERLASKRR KLVAIVDPHI KVDSGYRVHE ELRNLGLYVK TRDGSDYEGW
501 CWPGSAGYPD FTNPTMRAWW ANMFSYDNYE GSAPNLFVWN DMNEPSVFNG
551 PEVTMLKDAQ HYGGWEHRDV HNIYGLYVHM ATADGLRQRS GGMERPFVLA
601 RAFFAGSQRF GAVWTGDNTA EWDHLKISIP MCLSLGLVGL SFCGADVGGF
651 FKNPEPELLV RWYQMGAYQP FFRAHAHLDT GRREPWLLPS QHNDIIRDAL
701 GQRYSLLPFW YTLLYQAHRE GIPVMRPLWV QYPQDVTTFN IDDQYLLGDA
751 LLVHPVSDSG AHGVQVYLPG QGEVWYDIQS YQKHHGPQTL YLPVTLSSIP
801 VFQRGGTIVP RWMRVRRSSE CMKDDPITLF VALSPQGTAQ GELFLDDGYT
851 FNYQTRQEFL LRRFSFSGNT LVSSSADPEG HFETPIWIER VVIIGAGKPA
901 AVVLQTKGSP ESRLSFQHDP ETSVLVLRKP GINVASDWSI HLR
```

Residue Number Increasing Mass Decreasing Mass

Start - End	Observed	Mr(expt)	Mr(calc)	Delta	Miss	Sequence
51 - 60	1145.6494	1144.6421	1144.6352	0.0069	0	SIRPGLSPYR (No match)
100 - 108	1174.6230	1173.6157	1173.6142	0.0016	1	FRIDELEPR (No match)
112 - 126	1680.9452	1679.9379	1679.9358	0.0021	1	YRVPDVLVADPPIAR (No match)
173 - 181	1128.5870	1127.5797	1127.5723	0.0074	0	GLLEFEHQR (Ions score 3)
173 - 181	1128.5870	1127.5797	1127.5723	0.0074	0	GLLEFEHQR (No match)
784 - 804	2390.2917	2389.2844	2389.2906	-0.0062	0	HHGPQTLYLPVTLSSIPVFQR (No match)
784 - 804	2390.2917	2389.2844	2389.2906	-0.0062	0	HHGPQTLYLPVTLSSIPVFQR (Ions score 10)
914 - 928	1740.9296	1739.9223	1739.9206	0.0017	0	LSFQHDPETSVLVLR (Ions score 22)
914 - 928	1740.9296	1739.9223	1739.9206	0.0017	0	LSFQHDPETSVLVLR (No match)

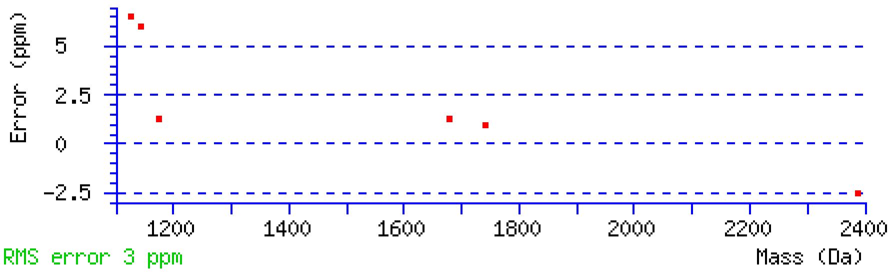

RMS error 3 ppm

Mascot: http://www.matrixscience.com/

Spot 132

Protein View

Match to: **gi|2274968** Score: **473** Expect: **9.6e-043**
Glucosidase II [Homo sapiens]

Nominal mass (M_r): **107289**; Calculated pI value: **5.71**
NCBI BLAST search of gi|2274968 against nr
Unformatted sequence string for pasting into other applications

Taxonomy: Homo sapiens

Fixed modifications: Carbamidomethyl (C)
Variable modifications: Oxidation (M)
Cleavage by Trypsin: cuts C-term side of KR unless next residue is P
Sequence Coverage: **38%**

Matched peptides shown in **Bold Red**

1 MAAVAAVAAR RRRSWASLVL AFLGVCLGIT LAVDRSNFK**T CEESSFCKR**Q

51 R**SIRPGLSPY RALLDSLQLG PDSLTVHLIH EVTKVLLVLE LQGLQK**NMTR

101 **FRIDELEPR**R PR**YRVPDVLV ADPPIAR**LSV SGRDENSVEL TMAEGPYK**II**

151 **LTARPFR**LDL LEDR**SLLLSV NARGLLEFEH QR**APR**VSQGS KDPAEGDGAQ**

201 **PEETPR**DGDK PEETQGK**AEK DEPGAWEETF K**THSDSKPYG PMSVGLDFSL

251 PGMEHVYGIP EHADNLR**LKV TEGGEPYR**LY NLDVFQYELY NPMALYGSVP

301 VLLAHNPHRD LGIFWLNAAE TWVDISSNTA GKTLFGK**MMD YLQGSGETPQ**

351 **TDVR**WMSETG IIDVFLLLGP SISDVFR**QYA SLTGTQALPP LFSLGYHQSR**

401 WNYRDEADVL EVDQGFDDHN LPCDVIWLDI EHADGKR**YFT WDPSRFPQPR**

451 TMLERLASKR RKLVAIVDPH IKVDSGYRVH EELRNLGLYV KTRDGSDYEG

501 WCWPGSAGYP DFTNPTMRAW WANMFSYDNY EGSAPNLFVW NDMNEPSVFN

551 GPEVTMLK**DA QHYGGWEHRD VHNIYGLYVH MATADGLR**QR **SGGMERPFVL**

601 **ARAFFAGSQR FGAVWTGDNT AEWDHLK**ISI PMCLSLGLVG LSFCGADVGG

651 FFK**NPEPELL VRWYQMGAYQ PFFR**AHAHLD TGR**REPWLLP SQHNDIIR**DA

701 LGQRYSLLPF WYTLLYQAHR EGIPVMRPLW VQYPQDVTTF NIDDQYLLGD

751 ALLVHPVSDS GAHGVQVYLP GQGEVWYDIQ SYQK**HHGPQT LYLPVTLSSI**

801 **PVFQR**GGTIV PRWMRVRRSS ECMKDDPITL FVALSPQGTA QGELFLDDGY

851 TFNYQTR**QEF LLRR**FSFSGN TLVSSSADPE GHFETPIWIE R**VVIIGAGKP**

901 **AAVVLQTK**GS PESR**LSFQHD PETSVLVLR**K PGINVASDWS IHLR

Residue Number Increasing Mass Decreasing Mass

Start - End	Observed	Mr(expt)	Mr(calc)	Delta	Miss	Sequence
40 - 49	1303.5449	1302.5376	1302.5332	0.0044	1	TCEESSFCKR (No match)
52 - 61	1145.6445	1144.6372	1144.6352	0.0020	0	SIRPGLSPYR (No match)
52 - 61	1145.6445	1144.6372	1144.6352	0.0020	0	SIRPGLSPYR (Ions score 1)
62 - 84	2499.3662	2498.3589	2498.3744	-0.0155	0	ALLDSLQLGPDSLTVHLIHEVTK (No match)
85 - 96	1352.8496	1351.8423	1351.8438	-0.0015	0	VLLVLELQGLQK (No match)
101 - 109	1174.6233	1173.6160	1173.6142	0.0019	1	FRIDELEPR (No match)
103 - 109	871.4545	870.4472	870.4447	0.0026	0	IDELEPR (No match)
113 - 127	1680.9431	1679.9358	1679.9358	0.0000	1	YRVPDVLVADPPIAR (No match)
149 - 157	1086.6777	1085.6704	1085.6709	-0.0005	0	IILTARPFR (No match)
165 - 173	972.5853	971.5780	971.5763	0.0017	0	SLLLSVNAR (No match)
174 - 182	1128.5820	1127.5747	1127.5723	0.0024	0	GLLEFEHQR (Ions score 34)
174 - 182	1128.5820	1127.5747	1127.5723	0.0024	0	GLLEFEHQR (No match)
186 - 206	2154.9866	2153.9793	2153.9824	-0.0031	1	VSQGSKDPAEGDGAQPEETPR (No match)
218 - 231	1636.7510	1635.7437	1635.7416	0.0021	1	AEKDEPGAWEETFK (No match)
268 - 278	1248.6599	1247.6526	1247.6509	0.0017	1	LKVTEGGEPYR (No match)
270 - 278	1007.4867	1006.4794	1006.4719	0.0075	0	VTEGGEPYR (No match)
338 - 354	1927.8595	1926.8522	1926.8451	0.0071	0	MMDYLQGSGETPQTDVR (No match)
338 - 354	1943.8528	1942.8455	1942.8400	0.0055	0	MMDYLQGSGETPQTDVR Oxidation (M) (No match)
378 - 400	2535.2822	2534.2749	2534.2917	-0.0168	0	QYASLTGTQALPPLFSLGYHQSR (No match)
438 - 445	1071.4930	1070.4857	1070.4821	0.0036	0	YFTWDPSR (No match)
438 - 450	1696.8802	1695.8729	1695.8157	0.0572	1	YFTWDPSRFPQPR (No match)
559 - 569	1355.5891	1354.5818	1354.5802	0.0016	0	DAQHYGGWEHR (No match)
559 - 569	1355.5891	1354.5818	1354.5802	0.0016	0	DAQHYGGWEHR (Ions score 31)
570 - 588	2145.0491	2144.0418	2144.0472	-0.0054	0	DVHNIYGLYVHMATADGLR (No match)
591 - 602	1319.6893	1318.6820	1318.6815	0.0005	0	SGGMERPFVLAR (No match)
591 - 602	1319.6893	1318.6820	1318.6815	0.0005	0	SGGMERPFVLAR (Ions score 24)
591 - 602	1335.6823	1334.6750	1334.6764	-0.0014	0	SGGMERPFVLAR Oxidation (M) (No match)
603 - 610	883.4452	882.4379	882.4347	0.0032	0	AFFAGSQR (No match)
611 - 627	1946.8859	1945.8786	1945.8958	-0.0172	0	FGAVWTGDNTAEWDHLK (No match)
654 - 662	1066.5935	1065.5862	1065.5818	0.0044	0	NPEPELLVR (No match)
663 - 674	1593.7343	1592.7270	1592.7234	0.0036	0	WYQMGAYQPFFR (No match)
663 - 674	1609.7267	1608.7194	1608.7183	0.0011	0	WYQMGAYQPFFR Oxidation (M) (No match)
684 - 698	1874.0024	1872.9951	1872.9958	-0.0007	1	REPWLLPSQHNDIIR (No match)
685 - 698	1717.9091	1716.9018	1716.8947	0.0071	0	EPWLLPSQHNDIIR (No match)
785 - 805	2390.2854	2389.2781	2389.2906	-0.0125	0	HHGPQTLYLPVTLSSIPVFQR (No match)
858 - 863	805.4559	804.4486	804.4493	-0.0007	0	QEFLLR (No match)
858 - 864	961.5549	960.5476	960.5504	-0.0028	1	QEFLLRR (No match)
892 - 908	1664.0491	1663.0418	1663.0395	0.0023	0	VVIIGAGKPAAVVLQTK (No match)
915 - 929	1740.9257	1739.9184	1739.9206	-0.0022	0	LSFQHDPETSVLVLR (Ions score 67)
915 - 929	1740.9257	1739.9184	1739.9206	-0.0022	0	LSFQHDPETSVLVLR (No match)

Spot 133

Mascot Search Results

Protein View

Match to: **gi|37182302** Score: **181** Expect: **1.5e-013**
ARTS-1 [Homo sapiens]

Nominal mass (M_r): **107645**; Calculated pI value: **5.92**
NCBI BLAST search of gi|37182302 against nr
Unformatted sequence string for pasting into other applications

Taxonomy: Homo sapiens

Fixed modifications: Carbamidomethyl (C)
Variable modifications: Oxidation (M)
Cleavage by Trypsin: cuts C-term side of KR unless next residue is P
Sequence Coverage: **12%**

Matched peptides shown in **Bold Red**

1 MVFLPLKWSL ATMSFLLSSL LALLTVSTPS WCQSTEASPK **RSDGTPFPWN**
51 **K**IRLPEYVIP VHYDLLIHAN LTTLTFWGTT K**VEITASQPT STIILHSHHL**
101 **QISR**ATLRKG AGERLSEEPL QVLEHPPQEQ IALLAPEPLL VGLPYTVVIH
151 YAGNLSETFH GFYKSTYRTK EGELR**ILAST QFEPTAAR**MA FPCFDEPAFK
201 ASFSIKIRRE PRHLAISNMP LVKSVTVAEG LIEDHFDVTV KMSTYLVAFI
251 ISDFESVSKI TKSGVKVSVY AVPDKINQAD YALDAAVTLL EFYEDYFSIP
301 YPLPKQDLAA IPDFQSGAME NWGLTTYRES ALLFDAEKSS ASSKLGITVT
351 VAHELAHQWF GNLVTMEWWN DLWLNEGFAK FMEFVSVSVT HPELKVGDYF
401 FGKCFDAMEV DALNSSHPVS TPVENPAQIR EMFDDVSYDK GACILNMLRE
451 YLSADAFKSG IVQYLQKHSY KNTKNEDLWD SMASICPTDG VKGMDGFCSR
501 SQHSSSSSHW HQEGVDVKTM MNTWTLQR**GF PLITITVR**GR NVHMKQEHYM
551 KGSDGAPDTG YLWHVPLTFI TSKSNMVHRF LLKTKTDVLI LPEEVEWIKF
601 NVGMNGYYIV HYEDDGWDSL TGLLKGTHTA VSSNDRASLI NNAFQLVSIG
651 KLSIEKALDL SLYLKHETEI MPVFQGLNEL IPMYKLMEKR DMNEVETQFK
701 AFLIRLLRDL IDKQTWTDEG SVSEQMLR**SE LLLLACVHNY QPCVQR**AEGY
751 FRKWKESNGN LSLPVDVTLA VFAVGAQSTE GWDFLYSKYQ FSLSSTEK**SQ**
801 **IEFALCR**TQN KEKLQWLLDE SFKGDKIK**TQ EFPQILTLIG RNPVGYPLAW**
851 **QFLR**KNWNKL VQKFELGSSS IAHMVMGTTN QFSTRTRLEE VKGFFSSLKE
901 NGSQLRCVQQ TIETIEENIG WMDKNFDKIR **VWLQSEKLER** M

Residue Number Increasing Mass Decreasing Mass

Start - End	Observed	Mr(expt)	Mr(calc)	Delta	Miss	Sequence
41 - 51	1304.6345	1303.6272	1303.6309	-0.0037	1	RSDGTPFPWNK (No match)
82 - 104	2568.3694	2567.3621	2567.3819	-0.0198	0	VEITASQPTSTIILHSHHLQISR (No match)
176 - 188	1404.7451	1403.7378	1403.7408	-0.0030	0	ILASTQFEPTAAR (No match)
176 - 188	1404.7451	1403.7378	1403.7408	-0.0030	0	ILASTQFEPTAAR (Ions score 37)
529 - 538	1116.6733	1115.6660	1115.6702	-0.0042	0	GFPLITITVR (No match)
529 - 538	1116.6733	1115.6660	1115.6702	-0.0042	0	GFPLITITVR (Ions score 6)
729 - 746	2200.0818	2199.0745	2199.0928	-0.0183	0	SELLLLACVHNYQPCVQR (No match)
799 - 807	1123.5565	1122.5492	1122.5491	0.0001	0	SQIEFALCR (Ions score 5)
799 - 807	1123.5565	1122.5492	1122.5491	0.0001	0	SQIEFALCR (No match)
829 - 841	1515.8450	1514.8377	1514.8456	-0.0079	0	TQEFPQILTLIGR (Ions score 37)
829 - 841	1515.8450	1514.8377	1514.8456	-0.0079	0	TQEFPQILTLIGR (No match)
842 - 854	1560.8269	1559.8196	1559.8248	-0.0052	0	NPVGYPLAWQFLR (Ions score 13)
842 - 854	1560.8269	1559.8196	1559.8248	-0.0052	0	NPVGYPLAWQFLR (No match)
931 - 940	1287.6835	1286.6762	1286.6982	-0.0220	1	VWLQSEKLER (No match)

Mascot: http://www.matrixscience.com/

Spot 134

{MATRIX SCIENCE} Mascot Search Results

Protein View

Match to: **gi|19879274** Score: **93** Expect: **9.9e-005**
adipocyte-derived leucine aminopeptidase 2 isoform a [Homo sapiens]

Nominal mass (M_r): **108264**; Calculated pI value: **5.78**
NCBI BLAST search of gi|19879274 against nr
Unformatted sequence string for pasting into other applications

Taxonomy: Homo sapiens
Links to retrieve other entries containing this sequence from NCBI Entrez:
gi|19879276 from Homo sapiens

Fixed modifications: Carbamidomethyl (C)
Variable modifications: Oxidation (M)
Cleavage by Trypsin: cuts C-term side of KR unless next residue is P
Sequence Coverage: **5%**

Matched peptides shown in **Bold Red**

```
  1 MVFLPLKWSL AIMSFLLSSL LALLTVSTPS WCQSTEASPK RSDGTPFPWN
 51 KIRLPEYVIP VHYDLLIHAN LTTLTFWGTT KVEITASQPT STIILHSHHL
101 QISRATLRKG AGERLSEEPL QVLEHPPQEQ IALLAPEPLL VGLPYTVVIH
151 YAGNLSETFH GFYKSTYRTK EGELRILAST QFEPTAARMA FPCFDEPAFK
201 ASFSIKIRRE PRHLAISNMP LVKSVTVAEG LIEDHFDVTV KMSTYLVAFI
251 ISDFESVSKI TKSGVKVSVY AVPDKINQAD YALDAAVTLL EFYEDYFSIP
301 YPLPKQDLAA IPDFQSGAME NWGLTTYRES ALLFDAEKSS ASSKLGITVT
351 VAHELAHQWF GNLVTMEWWN DLWLNEGFAK FMEFVSVSVT HPELKVGDYF
401 FGKCFDAMEV DALNSSHPVS TPVENPAQIR EMFDDVSYDK GACILNMLRE
451 YLSADAFKSG IVQYLQKHSY KNTKNEDLWD SMASICPTDG VKGMDGFCSR
501 SQHSSSSSHW HQEGVDVKTM MNTWTLQRGF PLITITVRGR NVHMKQEHYM
551 KGSDGAPDTG YLWHVPLTFI TSKSNMVHRF LLKTKTDVLI LPEEVEWIKF
601 NVGMNGYYIV HYEDDGWDSL TGLLKGTHTA VSSNDRASLI NNAFQLVSIG
651 KLSIEKALDL SLYLKHETEI MPVFQGLNEL IPMYKLMEKR DMNEVETQFK
701 AFLIRLLRDL IDKQTWTDEG SVSEQMLRSE LLLLACVHNY QPCVQRAEGY
751 FRKWKESNGN LSLPVDVTLA VFAVGAQSTE GWDFLYSKYQ FSLSSTEKSQ
801 IEFALCRTQN KEKLQWLLDE SFKGDKIKTQ EFPQILTLIG RNPVGYPLAW
851 QFLRKNWNKL VQKFELGSSS IAHMVMGTTN QFSTRTRLEE VKGFFSSLKE
901 NGSQLRCVQQ TIETIEENIG WMDKNFDKIR VWLQSEKLEH DPEADATG
```

Residue Number Increasing Mass Decreasing Mass

Start - End	Observed	Mr(expt)	Mr(calc)	Delta	Miss	Sequence
176 - 188	1404.7583	1403.7510	1403.7408	0.0102	0	ILASTQFEPTAAR (Ions score 3)
176 - 188	1404.7583	1403.7510	1403.7408	0.0102	0	ILASTQFEPTAAR (No match)
529 - 538	1116.6860	1115.6787	1115.6702	0.0085	0	GFPLITITVR (No match)
529 - 538	1116.6860	1115.6787	1115.6702	0.0085	0	GFPLITITVR (No match)
829 - 841	1515.8617	1514.8544	1514.8456	0.0088	0	TQEFPQILTLIGR (No match)
829 - 841	1515.8617	1514.8544	1514.8456	0.0088	0	TQEFPQILTLIGR (Ions score 62)
893 - 906	1569.8124	1568.8051	1568.7946	0.0105	1	GFFSSLKENGSQLR (No match)

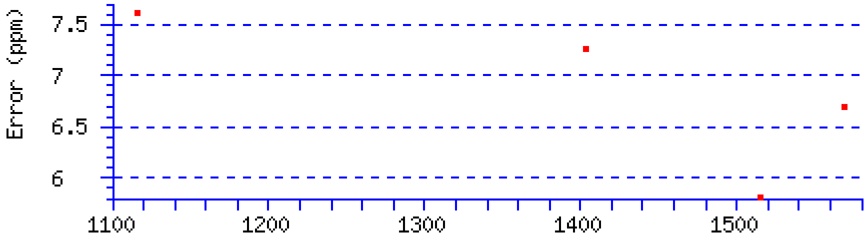

RMS error 6 ppm

Mascot: http://www.matrixscience.com/

Spot 135

{MATRIX SCIENCE} Mascot Search Results

Protein View

Match to: **gi|119625804** Score: **166** Expect: **4.8e-012**
moesin, isoform CRA_b [Homo sapiens]

Nominal mass (M_r): **66678**; Calculated pI value: **5.90**
NCBI BLAST search of gi|119625804 against nr
Unformatted sequence string for pasting into other applications

Taxonomy: Homo sapiens

Fixed modifications: Carbamidomethyl (C)
Variable modifications: Oxidation (M)
Cleavage by Trypsin: cuts C-term side of KR unless next residue is P
Sequence Coverage: **29%**

Matched peptides shown in **Bold Red**

```
  1 MDAELEFAIQ PNTTGKQLFD QVVKTIGLRE VWFFGLQYQD TKGFSTWLKL
 51 NKKVTAQDVR KESPLLFKFR AKFYPEDVSE ELIQDITQRL FFLQVKEGIL
101 NDDIYCPPET AVLLASYAVQ SKYGDFNKEV HKSGYLAGDK LLPQRVLEQH
151 KLNKDQWEER IQVWHEEHRG MLREDAVLEY LKIAQDLEMY GVNYFSIKNK
201 KGSELWLGVD ALGLNIYEQN DRLTPKIGFP WSEIRNISFN DKKFVIKPID
251 KKAPDFVFYA PRLRINKRIL ALCMGNHELY MRRRKPDTIE VQQMKAQARE
301 EKHQKQMERA MLENEKKKRE MAEKEKEKIE REKEELMERL KQIEEQTKKA
351 QQELEEQTRR ALELEQERKR AQSEAEKLAK ERQEAEEAKE ALLQASRDQK
401 KTQEQLALEM AELTARISQL EMARQKKESE AVEWQQKAQM VQEDLEKTRA
451 ELKTAMSTPH VAEPAENEQD EQDENGAEAS ADLRADAMAK DRSEEERTTE
501 AEKNERVQKH LKALTSELAN ARDESKKTAN DMIHAENMRL GRDKYKTLRQ
551 IRQGNTKQRI DEFESM
```

Residue Number Increasing Mass Decreasing Mass

Start - End	Observed	Mr(expt)	Mr(calc)	Delta	Miss	Sequence
17 - 24	976.5375	975.5302	975.5389	-0.0087	0	QLFDQVVK (No match)
62 - 70	1136.5719	1135.5646	1135.6389	-0.0743	1	ESPLLFKFR (No match)
71 - 89	2281.1033	2280.0960	2280.1273	-0.0313	1	AKFYPEDVSEELIQDITQR (No match)

73 - 89	2082.0063	2080.9990	2080.9953	0.0038	0	**FYPEDVSEELIQDITQR** (No match)
90 - 96	894.5416	893.5343	893.5374	-0.0031	0	**LFFLQVK** (No match)
123 - 132	1236.5707	1235.5634	1235.5934	-0.0300	1	**YGDFNKEVHK** (No match)
133 - 145	1417.7725	1416.7652	1416.7724	-0.0072	1	**SGYLAGDKLLPQR** (No match)
146 - 154	1108.5786	1107.5713	1107.6400	-0.0687	1	**VLEQHKLNK** (No match)
146 - 154	1108.5786	1107.5713	1107.6400	-0.0687	1	**VLEQHKLNK** (No match)
170 - 182	1552.8049	1551.7976	1551.7966	0.0010	1	**GMLREDAVLEYLK** Oxidation (M) (No match)
183 - 198	1906.9309	1905.9236	1905.9182	0.0055	0	**IAQDLEMYGVNYFSIK** Oxidation (M) (No match)
227 - 235	1104.5825	1103.5752	1103.5763	-0.0011	0	**IGFPWSEIR** (No match)
252 - 262	1310.6859	1309.6786	1309.6818	-0.0032	1	**KAPDFVFYAPR** (No match)
252 - 262	1310.6859	1309.6786	1309.6818	-0.0032	1	**KAPDFVFYAPR** (Ions score 4)
253 - 262	1182.5922	1181.5849	1181.5869	-0.0020	0	**APDFVFYAPR** (Ions score 33)
253 - 262	1182.5922	1181.5849	1181.5869	-0.0020	0	**APDFVFYAPR** (No match)
350 - 360	1387.6892	1386.6819	1386.6851	-0.0032	1	**AQQELEEQTRR** (No match)
361 - 368	987.5124	986.5051	986.5032	0.0019	0	**ALELEQER** (No match)
401 - 416	1847.9521	1846.9448	1846.9458	-0.0010	1	**KTQEQLALEMAELTAR** Oxidation (M) (No match)
498 - 506	1077.5184	1076.5111	1076.5098	0.0014	1	**TTEAEKNER** (No match)

Mascot: http://www.matrixscience.com/

Spot 136

Mascot Search Results

Protein View

Match to: **gi|37182302** Score: **222** Expect: **1.2e-017**
ARTS-1 [Homo sapiens]

Nominal mass (M_r): **107645**; Calculated pI value: **5.92**
NCBI BLAST search of gi|37182302 against nr
Unformatted sequence string for pasting into other applications

Taxonomy: Homo sapiens

Fixed modifications: Carbamidomethyl (C)
Variable modifications: Oxidation (M)
Cleavage by Trypsin: cuts C-term side of KR unless next residue is P
Sequence Coverage: **11%**

Matched peptides shown in **Bold Red**

1 MVFLPLKWSL ATMSFLLSSL LALLTVSTPS WCQSTEASPK **RSDGTPFPWN**
51 **K**IRLPEYVIP VHYDLLIHAN LTTLTFWGTT K**VEITASQPT STIILHSHHL**
101 **QISR**ATLRKG AGERLSEEPL QVLEHPPQEQ IALLAPEPLL VGLPYTVVIH
151 YAGNLSETFH GFYKSTYRTK EGELR**ILAST QFEPTAAR**MA FPCFDEPAFK
201 ASFSIKIRRE PRHLAISNMP LVKSVTVAEG LIEDHFDVTV KMSTYLVAFI
251 ISDFESVSKI TKSGVKVSVY AVPDKINQAD YALDAAVTLL EFYEDYFSIP
301 YPLPKQDLAA IPDFQSGAME NWGLTTYRES ALLFDAEKSS ASSKLGITVT
351 VAHELAHQWF GNLVTMEWWN DLWLNEGFAK FMEFVSVSVT HPELKVGDYF
401 FGKCFDAMEV DALNSSHPVS TPVENPAQIR EMFDDVSYDK GACILNMLRE
451 YLSADAFKSG IVQYLQKHSY KNTKNEDLWD SMASICPTDG VKGMDGFCSR
501 SQHSSSSSHW HQEGVDVKTM MNTWTLQR**GF PLITITVR**GR NVHMKQEHYM
551 KGSDGAPDTG YLWHVPLTFI TSKSNMVHRF LLKTKTDVLI LPEEVEWIKF
601 NVGMNGYYIV HYEDDGWDSL TGLLKGTHTA VSSNDRASLI NNAFQLVSIG
651 KLSIEKALDL SLYLKHETEI MPVFQGLNEL IPMYKLMEKR DMNEVETQFK
701 AFLIRLLRDL IDKQTWTDEG SVSEQMLR**SE LLLLACVHNY QPCVQR**AEGY
751 FRKWKESNGN LSLPVDVTLA VFAVGAQSTE GWDFLYSKYQ FSLSSTEK**SQ**
801 **IEFALCR**TQN KEKLQWLLDE SFKGDKIK**TQ EFPQILTLIG RNPVGYPLAW**
851 **QFLR**KNWNKL VQKFELGSSS IAHMVMGTTN QFSTRTRLEE VKGFFSSLKE
901 NGSQLRCVQQ TIETIEENIG WMDKNFDKIR VWLQSEKLER M

Residue Number Increasing Mass Decreasing Mass

Start - End	Observed	Mr(expt)	Mr(calc)	Delta	Miss	Sequence
41 - 51	1304.6261	1303.6188	1303.6309	-0.0121	1	RSDGTPFPWNK (No match)
82 - 104	2568.3572	2567.3499	2567.3819	-0.0320	0	VEITASQPTSTIILHSHHLQISR (No match)
176 - 188	1404.7334	1403.7261	1403.7408	-0.0147	0	ILASTQFEPTAAR (Ions score 43)
176 - 188	1404.7334	1403.7261	1403.7408	-0.0147	0	ILASTQFEPTAAR (No match)
529 - 538	1116.6677	1115.6604	1115.6702	-0.0098	0	GFPLITITVR (No match)
529 - 538	1116.6677	1115.6604	1115.6702	-0.0098	0	GFPLITITVR (Ions score 2)
729 - 746	2200.0671	2199.0598	2199.0928	-0.0330	0	SELLLLACVHNYQPCVQR (No match)
799 - 807	1123.5620	1122.5547	1122.5491	0.0056	0	SQIEFALCR (No match)
799 - 807	1123.5620	1122.5547	1122.5491	0.0056	0	SQIEFALCR (Ions score 10)
829 - 841	1515.8369	1514.8296	1514.8456	-0.0160	0	TQEFPQILTLIGR (No match)
829 - 841	1515.8369	1514.8296	1514.8456	-0.0160	0	TQEFPQILTLIGR (Ions score 73)
842 - 854	1560.8129	1559.8056	1559.8248	-0.0192	0	NPVGYPLAWQFLR (No match)
842 - 854	1560.8129	1559.8056	1559.8248	-0.0192	0	NPVGYPLAWQFLR (Ions score 17)

Mascot: http://www.matrixscience.com/

Spot 137

{MATRIX SCIENCE} Mascot Search Results

Protein View

Match to: **gi|13699868** Score: **288** Expect: **3e-024**
methylenetetrahydrofolate dehydrogenase 1 [Homo sapiens]

Nominal mass (M_r): **102152**; Calculated pI value: **6.75**
NCBI BLAST search of gi|13699868 against nr
Unformatted sequence string for pasting into other applications

Taxonomy: Homo sapiens
Links to retrieve other entries containing this sequence from NCBI Entrez:
gi|14602585 from Homo sapiens
gi|119601263 from Homo sapiens
gi|119601264 from Homo sapiens
gi|123993917 from synthetic construct
gi|124000687 from synthetic construct

Fixed modifications: Carbamidomethyl (C)
Variable modifications: Oxidation (M)
Cleavage by Trypsin: cuts C-term side of KR unless next residue is P
Sequence Coverage: **33%**

Matched peptides shown in **Bold Red**

```
  1 MAPAEILNGK EISAQIRARL KNQVTQLKEQ VPGFTPRLAI LQVGNRDDSN
 51 LYINVKLKAA EEIGIKATHI KLPRTTTESE VMKYITSLNE DSTVHGFLVQ
101 LPLDSENSIN TEEVINAIAP EKDVDGLTSI NAGRLARGDL NDCFIPCTPK
151 GCLELIKETG VPIAGRHAVV VGRSKIVGAP MHDLLLWNNA TVTTCHSKTA
201 HLDEEVNKGD ILVVATGQPE MVKGEWIKPG AIVIDCGINY VPDDKKPNGR
251 KVVGDVAYDE AKERASFITP VPGGVGPMTV AMLMQSTVES AKRFLEKFKP
301 GKWMIQYNNL NLKTPVPSDI DISRSCKPKP IGKLAREIGL LSEEVELYGE
351 TKAKVLLSAL ERLKHRPDGK YVVVTGITPT PLGEGKSTTT IGLVQALGAH
401 LYQNVFACVR QPSQGPTFGI KGGAAGGGYS QVIPMEEFNL HLTGDIHAIT
451 AANNLVAAAI DARIFHELTQ TDKALFNRLV PSVNGVRRFS DIQIRRLKRL
501 GIEKTDPTTL TDEEINRFAR LDIDPETITW QRVLDTNDRF LRKITIGQAP
551 TEKGHTRTAQ FDISVASEIM AVLALTTSLE DMRERLGKMV VASSKKGEPV
601 SAEDLGVSGA LTVLMKDAIK PNLMQTLEGT PVFVHAGPFA NIAHGNSSII
651 ADQIALKLVG PEGFVVTEAG FGADIGMEKF FNIKCRYSGL CPHVVVLVAT
701 VRALKMHGGG PTVTAGLPLP KAYIQENLEL VEKGFSNLKK QIENARMFGI
751 PVVVAVNAFK TDTESELDLI SRLSREHGAF DAVKCTHWAE GGKGALALAQ
```

```
801 AVQRAAQAPS SFQLLYDLKL PVEDKIRIIA QKIYGADDIE LLPEAQHKAE
851 VYTKQGFGNL PICMAKTHLS LSHNPEQKGV PTGFILPIRD IRASVGAGFL
901 YPLVGTMSTM PGLPTRPCFY DIDLDPETEQ VNGLF
```

Residue Number Increasing Mass Decreasing Mass

Start - End	Observed	Mr(expt)	Mr(calc)	Delta	Miss	Sequence
2 - 17	1709.9401	1708.9328	1708.9471	-0.0143	1	APAEILNGKEISAQIR (No match)
29 - 37	1030.5283	1029.5210	1029.5243	-0.0033	0	EQVPGFTPR (No match)
29 - 37	1030.5283	1029.5210	1029.5243	-0.0033	0	EQVPGFTPR (Ions score 7)
38 - 46	983.5823	982.5750	982.5923	-0.0173	0	LAILQVGNR (No match)
38 - 56	2145.1458	2144.1385	2144.1589	-0.0204	1	LAILQVGNRDDSNLYINVK (Ions score 18)
38 - 56	2145.1458	2144.1385	2144.1589	-0.0204	1	LAILQVGNRDDSNLYINVK (No match)
138 - 150	1536.6755	1535.6682	1535.6748	-0.0066	0	GDLNDCFIPCTPK (No match)
151 - 166	1712.9270	1711.9197	1711.9290	-0.0093	1	GCLELIKETGVPIAGR (No match)
252 - 264	1450.7164	1449.7091	1449.7099	-0.0008	1	VVGDVAYDEAKER (No match)
314 - 324	1199.6205	1198.6132	1198.6193	-0.0061	0	TPVPSDIDISR (No match)
337 - 352	1808.8910	1807.8837	1807.9090	-0.0253	0	EIGLLSEEVELYGETK (No match)
355 - 362	900.5397	899.5324	899.5440	-0.0115	0	VLLSALER (No match)
371 - 386	1630.9003	1629.8930	1629.8977	-0.0047	0	YVVVTGITPTPLGEGK (No match)
387 - 410	2619.3416	2618.3343	2618.3638	-0.0295	0	STTTIGLVQALGAHLYQNVFACVR (No match)
464 - 473	1231.6180	1230.6107	1230.6244	-0.0137	0	IFHELTQTDK (No match)
479 - 487	940.5566	939.5493	939.5501	-0.0008	0	LVPSVNGVR (No match)
489 - 496	1034.5708	1033.5635	1033.5668	-0.0033	1	FSDIQIRR (No match)
505 - 517	1504.7065	1503.6992	1503.7053	-0.0060	0	TDPTTLTDEEINR (No match)
533 - 539	832.4218	831.4145	831.4086	0.0059	0	VLDTNDR (No match)
687 - 702	1769.9587	1768.9514	1768.9657	-0.0143	0	YSGLCPHVVVLVATVR (No match)
687 - 702	1769.9587	1768.9514	1768.9657	-0.0143	0	YSGLCPHVVVLVATVR (Ions score 14)
706 - 721	1548.8129	1547.8056	1547.8129	-0.0073	0	MHGGGPTVTAGLPLPK Oxidation (M) (No match)
722 - 733	1448.7456	1447.7383	1447.7558	-0.0175	0	AYIQENLELVEK (No match)
794 - 804	1097.6377	1096.6304	1096.6352	-0.0048	0	GALALAQAVQR (Ions score 4)
794 - 804	1097.6377	1096.6304	1096.6352	-0.0048	0	GALALAQAVQR (No match)
805 - 819	1651.8584	1650.8511	1650.8617	-0.0105	0	AAQAPSSFQLLYDLK (No match)
833 - 848	1811.9104	1810.9031	1810.9101	-0.0069	0	IYGADDIELLPEAQHK (No match)
867 - 878	1390.6896	1389.6823	1389.7000	-0.0177	0	THLSLSHNPEQK (No match)
879 - 889	1169.7000	1168.6927	1168.6968	-0.0040	0	GVPTGFILPIR (Ions score 7)
879 - 889	1169.7000	1168.6927	1168.6968	-0.0040	0	GVPTGFILPIR (No match)

Mascot: http://www.matrixscience.com/

Spot 138

Mascot Search Results

Protein View

Match to: **gi|119594456** Score: **473** Expect: **9.6e-043**
glucosidase, alpha; neutral AB, isoform CRA_e [Homo sapiens]

Nominal mass (M_r): **107236**; Calculated pI value: **5.82**
NCBI BLAST search of gi|119594456 against nr
Unformatted sequence string for pasting into other applications

Taxonomy: Homo sapiens

Fixed modifications: Carbamidomethyl (C)
Variable modifications: Oxidation (M)
Cleavage by Trypsin: cuts C-term side of KR unless next residue is P
Sequence Coverage: **38%**

Matched peptides shown in **Bold Red**

1 MAAVAAVAAR RRRSWASLVL AFLGVCLGIT LAVDRSNFK**T** **CEESSFCKR**Q
51 R**SIRPGLSPY** **RALLDSLQLG** **PDSLTVHLIH** **EVTKVLLVLE** **LQGLQK**NMTR
101 **FRIDELEPR**R PR**YRVPDVLV** **ADPPIAR**LSV SGRDENSVEL TMAEGPYK**II**
151 **LTARPFR**LDL LEDR**SLLLSV** **NARGLLEFEH** **QR**APR**VSQGS** **KDPAEGDGAQ**
201 **PEETPR**DGDK AKETQGK**AEK** **DEPGAWEETF** **K**THSDSKPYG PMSVGLDFSL
251 PGMEHVYGIP EHADNLR**LKV** **TEGGEPYR**LY NLDVFQYELY NPMALYGSVP
301 VLLAHNPHRD LGIFWLNAAE TWVDISSNTA GKTLFGK**MMD** **YLQGSGETPQ**
351 **TDVR**WMSETG IIDVFLLLGP SISDVFR**QYA** **SLTGTQALPP** **LFSLGYHQSR**
401 WNYRDEADVL EVDQGFDDHN LPCDVIWLDI EHADGKR**YFT** **WDPSRFPQPR**
451 TMLERLASKR RKLVAIVDPH IKVDSGYRVH EELRNLGLYV KTRDGSDYEG
501 WCWPGSAGYP DFTNPTMRAW WANMFSYDNY EGSAPNLFVW NDMNEPSVFN
551 GPEVTMLK**DA** **QHYGGWEHRD** **VHNIYGLYVH** **MATADGLR**QR **SGGMERPFVL**
601 **ARAFFAGSQR** **FGAVWTGDNT** **AEWDHLK**ISI PMCLSLGLVG LSFCGADVGG
651 FFK**NPEPELL** **VRWYQMGAYQ** **PFFR**AHAHLD TGR**REPWLLP** **SQHNDIIR**DA
701 LGQRYSLLPF WYTLLYQAHR EGIPVMRPLW VQYPQDVTTF NIDDQYLLGD
751 ALLVHPVSDS GAHGVQVYLP GQGEVWYDIQ SYQK**HHGPQT** **LYLPVTLSSI**
801 **PVFQR**GGTIV PRWMRVRRSS ECMKDDPITL FVALSPQGTA QGELFLDDGH
851 TFNYQTR**QEF** **LLRR**FSFSGN TLVSSSADPE GHFETPIWIE R**VVIIGAGKP**
901 **AAVVLQTK**GS PESR**LSFQHD** **PETSVLVLR**K PGINVASDWS IHLR

Residue Number Increasing Mass Decreasing Mass

Start - End	Observed	Mr(expt)	Mr(calc)	Delta	Miss	Sequence
40 - 49	1303.5449	1302.5376	1302.5332	0.0044	1	TCEESSFCKR (No match)
52 - 61	1145.6445	1144.6372	1144.6352	0.0020	0	SIRPGLSPYR (No match)
52 - 61	1145.6445	1144.6372	1144.6352	0.0020	0	SIRPGLSPYR (Ions score 1)
62 - 84	2499.3662	2498.3589	2498.3744	-0.0155	0	ALLDSLQLGPDSLTVHLIHEVTK (No match)
85 - 96	1352.8496	1351.8423	1351.8438	-0.0015	0	VLLVLELQGLQK (No match)
101 - 109	1174.6233	1173.6160	1173.6142	0.0019	1	FRIDELEPR (No match)
103 - 109	871.4545	870.4472	870.4447	0.0026	0	IDELEPR (No match)
113 - 127	1680.9431	1679.9358	1679.9358	0.0000	1	YRVPDVLVADPPIAR (No match)
149 - 157	1086.6777	1085.6704	1085.6709	-0.0005	0	IILTARPFR (No match)
165 - 173	972.5853	971.5780	971.5763	0.0017	0	SLLLSVNAR (No match)
174 - 182	1128.5820	1127.5747	1127.5723	0.0024	0	GLLEFEHQR (Ions score 34)
174 - 182	1128.5820	1127.5747	1127.5723	0.0024	0	GLLEFEHQR (No match)
186 - 206	2154.9866	2153.9793	2153.9824	-0.0031	1	VSQGSKDPAEGDGAQPEETPR (No match)
218 - 231	1636.7510	1635.7437	1635.7416	0.0021	1	AEKDEPGAWEETFK (No match)
268 - 278	1248.6599	1247.6526	1247.6509	0.0017	1	LKVTEGGEPYR (No match)
270 - 278	1007.4867	1006.4794	1006.4719	0.0075	0	VTEGGEPYR (No match)
338 - 354	1927.8595	1926.8522	1926.8451	0.0071	0	MMDYLQGSGETPQTDVR (No match)
338 - 354	1943.8528	1942.8455	1942.8400	0.0055	0	MMDYLQGSGETPQTDVR Oxidation (M) (No match)
378 - 400	2535.2822	2534.2749	2534.2917	-0.0168	0	QYASLTGTQALPPLFSLGYHQSR (No match)
438 - 445	1071.4930	1070.4857	1070.4821	0.0036	0	YFTWDPSR (No match)
438 - 450	1696.8802	1695.8729	1695.8157	0.0572	1	YFTWDPSRFPQPR (No match)
559 - 569	1355.5891	1354.5818	1354.5802	0.0016	0	DAQHYGGWEHR (No match)
559 - 569	1355.5891	1354.5818	1354.5802	0.0016	0	DAQHYGGWEHR (Ions score 31)
570 - 588	2145.0491	2144.0418	2144.0472	-0.0054	0	DVHNIYGLYVHMATADGLR (No match)
591 - 602	1319.6893	1318.6820	1318.6815	0.0005	0	SGGMERPFVLAR (No match)
591 - 602	1319.6893	1318.6820	1318.6815	0.0005	0	SGGMERPFVLAR (Ions score 24)
591 - 602	1335.6823	1334.6750	1334.6764	-0.0014	0	SGGMERPFVLAR Oxidation (M) (No match)
603 - 610	883.4452	882.4379	882.4347	0.0032	0	AFFAGSQR (No match)
611 - 627	1946.8859	1945.8786	1945.8958	-0.0172	0	FGAVWTGDNTAEWDHLK (No match)
654 - 662	1066.5935	1065.5862	1065.5818	0.0044	0	NPEPELLVR (No match)
663 - 674	1593.7343	1592.7270	1592.7234	0.0036	0	WYQMGAYQPFFR (No match)
663 - 674	1609.7267	1608.7194	1608.7183	0.0011	0	WYQMGAYQPFFR Oxidation (M) (No match)
684 - 698	1874.0024	1872.9951	1872.9958	-0.0007	1	REPWLLPSQHNDIIR (No match)
685 - 698	1717.9091	1716.9018	1716.8947	0.0071	0	EPWLLPSQHNDIIR (No match)
785 - 805	2390.2854	2389.2781	2389.2906	-0.0125	0	HHGPQTLYLPVTLSSIPVFQR (No match)
858 - 863	805.4559	804.4486	804.4493	-0.0007	0	QEFLLR (No match)
858 - 864	961.5549	960.5476	960.5504	-0.0028	1	QEFLLRR (No match)
892 - 908	1664.0491	1663.0418	1663.0395	0.0023	0	VVIIGAGKPAAVVLQTK (No match)
915 - 929	1740.9257	1739.9184	1739.9206	-0.0022	0	LSFQHDPETSVLVLR (Ions score 67)
915 - 929	1740.9257	1739.9184	1739.9206	-0.0022	0	LSFQHDPETSVLVLR (No match)

Spot 139

Mascot Search Results

Protein View

Match to: **gi|13699868** Score: **297** Expect: **3.8e-025**
methylenetetrahydrofolate dehydrogenase 1 [Homo sapiens]

Nominal mass (M_r): **102152**; Calculated pI value: **6.75**
NCBI BLAST search of gi|13699868 against nr
Unformatted sequence string for pasting into other applications

Taxonomy: Homo sapiens
Links to retrieve other entries containing this sequence from NCBI Entrez:
gi|14602585 from Homo sapiens
gi|119601263 from Homo sapiens
gi|119601264 from Homo sapiens
gi|123993917 from synthetic construct
gi|124000687 from synthetic construct

Fixed modifications: Carbamidomethyl (C)
Variable modifications: Oxidation (M)
Cleavage by Trypsin: cuts C-term side of KR unless next residue is P
Sequence Coverage: **28%**

Matched peptides shown in **Bold Red**

```
  1 MAPAEILNGK EISAQIRARL KNQVTQLKEQ VPGFTPRLAI LQVGNRDDSN
 51 LYINVKLKAA EEIGIKATHI KLPRTTTESE VMKYITSLNE DSTVHGFLVQ
101 LPLDSENSIN TEEVINAIAP EKDVDGLTSI NAGRLARGDL NDCFIPCTPK
151 GCLELIKETG VPIAGRHAVV VGRSKIVGAP MHDLLLWNNA TVTTCHSKTA
201 HLDEEVNKGD ILVVATGQPE MVKGEWIKPG AIVIDCGINY VPDDKKPNGR
251 KVVGDVAYDE AKERASFITP VPGGVGPMTV AMLMQSTVES AKRFLEKFKP
301 GKWMIQYNNL NLKTPVPSDI DISRSCKPKP IGKLAREIGL LSEEVELYGE
351 TKAKVLLSAL ERLKHRPDGK YVVVTGITPT PLGEGKSTTT IGLVQALGAH
401 LYQNVFACVR QPSQGPTFGI KGGAAGGGYS QVIPMEEFNL HLTGDIHAIT
451 AANNLVAAAI DARIFHELTQ TDKALFNRLV PSVNGVRRFS DIQIRRLKRL
501 GIEKTDPTTL TDEEINRFAR LDIDPETITW QRVLDTNDRF LRKITIGQAP
551 TEKGHTRTAQ FDISVASEIM AVLALTTSLE DMRERLGKMV VASSKKGEPV
601 SAEDLGVSGA LTVLMKDAIK PNLMQTLEGT PVFVHAGPFA NIAHGNSSII
651 ADQIALKLVG PEGFVVTEAG FGADIGMEKF FNIKCRYSGL CPHVVVLVAT
701 VRALKMHGGG PTVTAGLPLP KAYIQENLEL VEKGFSNLKK QIENARMFGI
751 PVVVAVNAFK TDTESELDLI SRLSREHGAF DAVKCTHWAE GGKGALALAQ
```

```
801 AVQRAAQAPS SFQLLYDLKL PVEDKIRIIA QKIYGADDIE LLPEAQHKAE
851 VYTKQGFGNL PICMAKTHLS LSHNPEQKGV PTGFILPIRD IRASVGAGFL
901 YPLVGTMSTM PGLPTRPCFY DIDLDPETEQ VNGLF
```

Residue Number Increasing Mass Decreasing Mass

Start - End	Observed	Mr(expt)	Mr(calc)	Delta	Miss	Sequence
2 - 17	1710.0331	1709.0258	1708.9471	0.0787	1	APAEILNGKEISAQIR (Ions score 28)
2 - 17	1710.0331	1709.0258	1708.9471	0.0787	1	APAEILNGKEISAQIR (No match)
22 - 37	1842.0691	1841.0618	1840.9795	0.0823	1	NQVTQLKEQVPGFTPR (No match)
29 - 37	1030.5770	1029.5697	1029.5243	0.0454	0	EQVPGFTPR (No match)
29 - 37	1030.5770	1029.5697	1029.5243	0.0454	0	EQVPGFTPR (Ions score 5)
38 - 56	2145.2532	2144.2459	2144.1589	0.0870	1	LAILQVGNRDDSNLYINVK (No match)
151 - 166	1713.0195	1712.0122	1711.9290	0.0832	1	GCLELIKETGVPIAGR (No match)
252 - 264	1450.8021	1449.7948	1449.7099	0.0849	1	VVGDVAYDEAKER (No match)
314 - 324	1199.6821	1198.6748	1198.6193	0.0555	0	TPVPSDIDISR (No match)
355 - 362	900.5782	899.5709	899.5440	0.0270	0	VLLSALER (No match)
371 - 386	1630.9760	1629.9687	1629.8977	0.0710	0	YVVVTGITPTPLGEGK (No match)
479 - 487	940.6011	939.5938	939.5501	0.0437	0	LVPSVNGVR (No match)
489 - 496	1034.6213	1033.6140	1033.5668	0.0472	1	FSDIQIRR (No match)
505 - 517	1504.7917	1503.7844	1503.7053	0.0792	0	TDPTTLTDEEINR (No match)
521 - 532	1486.8218	1485.8145	1485.7463	0.0682	0	LDIDPETITWQR (No match)
687 - 702	1770.0549	1769.0476	1768.9657	0.0819	0	YSGLCPHVVVLVATVR (No match)
687 - 702	1770.0549	1769.0476	1768.9657	0.0819	0	YSGLCPHVVVLVATVR (Ions score 37)
722 - 733	1448.8252	1447.8179	1447.7558	0.0621	0	AYIQENLELVEK (No match)
747 - 760	1507.8976	1506.8903	1506.8268	0.0636	0	MFGIPVVVAVNAFK Oxidation (M) (No match)
794 - 804	1097.6934	1096.6861	1096.6352	0.0509	0	GALALAQAVQR (No match)
794 - 804	1097.6934	1096.6861	1096.6352	0.0509	0	GALALAQAVQR (Ions score 10)
805 - 819	1651.9457	1650.9384	1650.8617	0.0768	0	AAQAPSSFQLLYDLK (No match)
820 - 827	969.6137	968.6064	968.5654	0.0410	1	LPVEDKIR (No match)
833 - 848	1812.0018	1810.9945	1810.9101	0.0845	0	IYGADDIELLPEAQHK (No match)
879 - 889	1169.7567	1168.7494	1168.6968	0.0527	0	GVPTGFILPIR (Ions score 15)
879 - 889	1169.7567	1168.7494	1168.6968	0.0527	0	GVPTGFILPIR (No match)
879 - 892	1553.9822	1552.9749	1552.9089	0.0660	1	GVPTGFILPIRDIR (No match)

Mascot: http://www.matrixscience.com/

Spot 140

MATRIX SCIENCE Mascot Search Results

Protein View

Match to: **gi|12025678** Score: **432** Expect: **1.2e-038**
actinin, alpha 4 [Homo sapiens]

Nominal mass (M_r): **105245**; Calculated pI value: **5.27**
NCBI BLAST search of gi|12025678 against nr
Unformatted sequence string for pasting into other applications

Taxonomy: Homo sapiens
Links to retrieve other entries containing this sequence from NCBI Entrez:
gi|109124593 from Macaca mulatta
gi|13123943 from Homo sapiens
gi|13477151 from Homo sapiens
gi|119577214 from Homo sapiens
gi|123993527 from synthetic construct
gi|124000535 from synthetic construct

Fixed modifications: Carbamidomethyl (C)
Variable modifications: Oxidation (M)
Cleavage by Trypsin: cuts C-term side of KR unless next residue is P
Sequence Coverage: **32%**

Matched peptides shown in **Bold Red**

1 MVDYHAANQS YQYGPSSAGN GAGGGGSMGD YMAQEDDWDR DLLLDPAWEK
51 QQRK**TFTAWC NSHLRKAGTQ IENIDEDFRD GLK**LMLLLEV ISGERLPKPE
101 RGKMRVHKIN NVNKALDFIA SKGVKLVSIG AEEIVDGNAK MTLGMIWTII
151 LR**FAIQDISV EETSAKEGLL LWCQR**KTAPY K**NVNVQNFHI SWKDGLAFNA**
201 **LIHRHRPELI EYDKLR**KDDP VTNLNNAFEV AEKYLDIPK**M LDAEDIVNTA**
251 **RPDEKAIMTY VSSFYHAFSG AQK**AETAANR ICKVLAVNQE NEHLMEDYEK
301 LASDLLEWIR R**TIPWLEDRV PQK**TIQEMQQ K**LEDFRDYR**R VHKPPKVQEK
351 CQLEINFNTL QTKLR**LSNRP AFMPSEGK**MV SDINNGWQHL EQAEKGYEEW
401 LLNEIRRLER LDHLAEKFRQ KASIHEAWTD GKEAMLK**HRD YETATLSDIK**
451 ALIR**KHEAFE SDLAAHQDR**V EQIAAIAQEL NELDYYDSHN VNTRCQK**ICD**
501 **QWDALGSLTH SR**REALEKTE KQLEAIDQLH LEYAKRAAPF NNWMESAMED
551 LQDMFIVHTI EEIEGLISAH DQFK**STLPDA DRER**EAILAI HKEAQRIAES
601 NHIKLSGSNP YTTVTPQIIN SKWEKVQQLV PKRDHALLEE QSKQQSNEHL
651 RRQFASQANV VGPWIQTKME EIGRISIEMN GTLEDQLSHL KQYERSIVDY
701 KPNLDLLEQQ HQLIQEALIF DNK**HTNYTME HIRVGWEQLL TTIARTINEV**

```
751 ENQILTRDAK GISQEQMQEF RASFNHFDKD HGGALGPEEF KACLISLGYD
801 VENDRQGEAE FNRIMSLVDP NHSGLVTFQA FIDFMSRETT DTDTADQVIA
851 SFKVLAGDKN FITAEELRRE LPPDQAEYCI ARMAPYQGPD AVPGALDYKS
901 FSTALYGESD L
```

Residue Number Increasing Mass Decreasing Mass

Start - End	Observed	Mr(expt)	Mr(calc)	Delta	Miss	Sequence
55 - 65	1392.6846	1391.6773	1391.6404	0.0369	0	TFTAWCNSHLR (No match)
55 - 66	1520.7827	1519.7754	1519.7354	0.0400	1	TFTAWCNSHLRK (No match)
67 - 83	1920.9833	1919.9760	1919.9224	0.0536	1	AGTQIENIDEDFRDGLK (No match)
153 - 166	1537.8082	1536.8009	1536.7671	0.0338	0	FAIQDISVEETSAK (No match)
167 - 175	1174.6333	1173.6260	1173.5964	0.0296	0	EGLLLWCQR (Ions score 41)
167 - 175	1174.6333	1173.6260	1173.5964	0.0296	0	EGLLLWCQR (No match)
182 - 193	1485.8300	1484.8227	1484.7524	0.0703	0	NVNVQNFHISWK (No match)
194 - 204	1226.6958	1225.6885	1225.6567	0.0318	0	DGLAFNALIHR (No match)
205 - 214	1299.7024	1298.6951	1298.6619	0.0333	0	HRPELIEYDK (No match)
205 - 216	1568.8910	1567.8837	1567.8470	0.0367	1	HRPELIEYDKLR (No match)
240 - 255	1816.9170	1815.9097	1815.8672	0.0425	0	MLDAEDIVNTARPDEK (No match)
256 - 273	2008.0193	2007.0120	2006.9560	0.0561	0	AIMTYVSSFYHAFSGAQK (No match)
312 - 323	1481.8501	1480.8428	1480.8038	0.0391	1	TIPWLEDRVPQK (No match)
312 - 323	1481.8501	1480.8428	1480.8038	0.0391	1	TIPWLEDRVPQK (Ions score 30)
332 - 339	1113.5608	1112.5535	1112.5250	0.0285	1	LEDFRDYR (No match)
366 - 378	1433.7583	1432.7510	1432.7132	0.0378	0	LSNRPAFMPSEGK (No match)
438 - 450	1548.8020	1547.7947	1547.7579	0.0368	1	HRDYETATLSDIK (No match)
455 - 469	1753.8707	1752.8634	1752.8179	0.0455	1	KHEAFESDLAAHQDR (No match)
498 - 512	1758.8728	1757.8655	1757.8155	0.0500	0	ICDQWDALGSLTHSR (No match)
575 - 584	1159.6018	1158.5945	1158.5629	0.0316	1	STLPDADRER (No match)
724 - 733	1301.6500	1300.6427	1300.5982	0.0445	0	HTNYTMEHIR (No match)
734 - 745	1386.8080	1385.8007	1385.7666	0.0341	0	VGWEQLLTTIAR (No match)
746 - 757	1429.7987	1428.7914	1428.7572	0.0342	0	TINEVENQILTR (No match)
746 - 757	1429.7987	1428.7914	1428.7572	0.0342	0	TINEVENQILTR (Ions score 24)
761 - 771	1352.6611	1351.6538	1351.6190	0.0348	0	GISQEQMQEFR (No match)
761 - 771	1352.6611	1351.6538	1351.6190	0.0348	0	GISQEQMQEFR (Ions score 42)
761 - 771	1368.6554	1367.6481	1367.6139	0.0342	0	GISQEQMQEFR Oxidation (M) (No match)
772 - 791	2203.0686	2202.0613	2202.0129	0.0484	1	ASFNHFDKDHGGALGPEEFK (No match)
854 - 868	1675.9434	1674.9361	1674.8940	0.0421	1	VLAGDKNFITAEELR (No match)
870 - 882	1561.7706	1560.7633	1560.7242	0.0391	0	ELPPDQAEYCIAR (Ions score 75)
870 - 882	1561.7706	1560.7633	1560.7242	0.0391	0	ELPPDQAEYCIAR (No match)

Spot 141

{MATRIX SCIENCE} Mascot Search Results

Protein View

Match to: **gi|26000235** Score: **165** Expect: **6.1e-012**
Rap1-interacting adaptor molecule [Homo sapiens]

Nominal mass (M_r): **73496**; Calculated pI value: **5.40**
NCBI BLAST search of gi|26000235 against nr
Unformatted sequence string for pasting into other applications

Taxonomy: Homo sapiens

Fixed modifications: Carbamidomethyl (C)
Variable modifications: Oxidation (M)
Cleavage by Trypsin: cuts C-term side of KR unless next residue is P
Sequence Coverage: **12%**

Matched peptides shown in **Bold Red**

```
  1 MGESSEDIDQ MFSTLLGEMD LLTQSLGVDT LPPPDPNPPR AEFNYSVGFK
 51 DLNESLNALE DQDLDALMAD LVADISEAEQ RTIQAQKESL QNQHHSASLQ
101 ASIFSGAASL GYGTNVAATG ISQYEDDLPP PPADPVLDLP LPPPPPEPLS
151 QEEEEAQAKA DKIKLALEKL KEAKVKKLVV KVHMNDNSTK SLMVDERQLA
201 RDVLDNLFEK THCDCNVDWC LYEIYPELQI ERFFEDHENV VEVLSDWTRD
251 TENKILFLEK EEKYAVFKNP QNFYLDNRGK KESKETNEKM NAKNKESLLE
301 ESFCGTSIIV PELEGALYLK EDGKKSWKRR YFLLRASGIY YVPKGKTKTS
351 RDLACFIQFE NVNIYYGTQH KMKYKAPTDY CFVLKHPQIQ KESQYIKYLC
401 CDDTRTLNQW VMGIRIAKYG KTLYDNYQRA VAKAGLASRW TNLGTVNAAA
451 PAQPSTGPKT GTTQPNGQIP QATHSVSAVL QEAQRHAETS KDKKPALGNH
501 HDPAVPRAPH APKSSLPPPP PVRRSSDTSG SPATPLKAKG TGGGGLPAPP
551 DDFLPPPPPP PPLDDPELPP PPPDFMEPPP DFVPPPPPSY AGIAGSELPP
601 PPPPPAPAPA PVPDSARPPP AVAKRPPVPP KRQENPGHPG GAGGGEQDFM
651 SDLMKALQKK RGNVS
```

Residue Number Increasing Mass Decreasing Mass

Start - End	Observed	Mr(expt)	Mr(calc)	Delta	Miss	Sequence
264 - 278	1888.9911	1887.9838	1887.9267	0.0571	1	YAVFKNPQNFYLDNR (No match)

264 - 278	1888.9911	1887.9838	1887.9267	0.0571	1	YAVFKNPQNFYLDNR (Ions score 13)
269 - 278	1280.6425	1279.6352	1279.5945	0.0407	0	NPQNFYLDNR (No match)
269 - 278	1280.6425	1279.6352	1279.5945	0.0407	0	NPQNFYLDNR (Ions score 13)
330 - 335	867.5392	866.5319	866.5126	0.0193	1	RYFLLR (No match)
422 - 429	1072.5375	1071.5302	1071.4985	0.0317	0	TLYDNYQR (No match)
422 - 429	1072.5375	1071.5302	1071.4985	0.0317	0	TLYDNYQR (No match)
492 - 507	1751.9847	1750.9774	1750.9226	0.0548	1	DKKPALGNHHDPAVPR (No match)
492 - 507	1751.9847	1750.9774	1750.9226	0.0548	1	DKKPALGNHHDPAVPR (Ions score 30)
494 - 507	1508.8564	1507.8491	1507.8007	0.0484	0	KPALGNHHDPAVPR (No match)
514 - 523	1046.6348	1045.6275	1045.5920	0.0355	0	SSLPPPPPVR (No match)
514 - 524	1202.7362	1201.7289	1201.6931	0.0358	1	SSLPPPPPVRR (Ions score 33)
514 - 524	1202.7362	1201.7289	1201.6931	0.0358	1	SSLPPPPPVRR (No match)
632 - 655	2547.1941	2546.1868	2546.0913	0.0955	1	RQENPGHPGGAGGGEQDFMSDLMK 2 Oxidation (M) (No match)

Mascot: http://www.matrixscience.com/

Spot 143

MATRIX SCIENCE Mascot Search Results

Protein View

Match to: **gi|12025678** Score: **585** Expect: **6.1e-054**
actinin, alpha 4 [Homo sapiens]

Nominal mass (M_r): **105245**; Calculated pI value: **5.27**
NCBI BLAST search of gi|12025678 against nr
Unformatted sequence string for pasting into other applications

Taxonomy: Homo sapiens
Links to retrieve other entries containing this sequence from NCBI Entrez:
gi|109124593 from Macaca mulatta
gi|13123943 from Homo sapiens
gi|13477151 from Homo sapiens
gi|119577214 from Homo sapiens
gi|123993527 from synthetic construct
gi|124000535 from synthetic construct

Fixed modifications: Carbamidomethyl (C)
Variable modifications: Oxidation (M)
Cleavage by Trypsin: cuts C-term side of KR unless next residue is P
Sequence Coverage: **45%**

Matched peptides shown in **Bold Red**

1 MVDYHAANQS YQYGPSSAGN GAGGGGSMGD YMAQEDDWDR DLLLDPAWEK
51 QQRK**TFTAWC** **NSHLRKAGTQ** **IENIDEDFRD** **GLK**LMLLLEV ISGERLPKPE
101 RGKMRVHKIN NVNKALDFIA SKGVKLVSIG AEEIVDGNAK MTLGMIWTII
151 LR**FAIQDISV** **EETSAKEGLL** **LWCQR**KTAPY K**NVNVQNFHI** **SWKDGLAFNA**
201 **LIHRHRPELI** **EYDKLR**KDDP VTNLNNAFEV AEKYLDIPK**M** **LDAEDIVNTA**
251 **RPDEKAIMTY** **VSSFYHAFSG** **AQK**AETAANR ICK**VLAVNQE** **NEHLMEDYEK**
301 LASDLLEWIR R**TIPWLEDRV** **PQK**TIQEMQQ K**LEDFRDYR**R VHKPPKVQEK
351 **CQLEINFNTL** **QTK**LR**LSNRP** **AFMPSEGK**MV SDINNGWQHL EQAEKGYEEW
401 LLNEIRRLER LDHLAEKFRQ KASIHEAWTD GKEAMLK**HRD** **YETATLSDIK**
451 ALIR**KHEAFE** **SDLAAHQDR**V EQIAAIAQEL NELDYYDSHN VNTRCQK**ICD**
501 **QWDALGSLTH** **SR**REALEKTE K**QLEAIDQLH** **LEYAKR**AAPF NNWMESAMED
551 LQDMFIVHTI EEIEGLISAH DQFK**STLPDA** **DRER**EAILAI HKEAQRIAES
601 NHIK**LSGSNP** **YTTVTPQIIN** **SK**WEKVQQLV PKRDHALLEE QSK**QQSNEHL**
651 **RR**QFASQANV VGPWIQTKME EIGRISIEMN GTLEDQLSHL KQYERSIVDY
701 KPNLDLLEQQ HQLIQEALIF DNK**HTNYTME** **HIRVGWEQLL** **TTIARTINEV**

```
751 ENQILTRDAK GISQEQMQEF RASFNHFDKD HGGALGPEEF KACLISLGYD
801 VENDRQGEAE FNRIMSLVDP NHSGLVTFQA FIDFMSRETT DTDTADQVIA
851 SFKVLAGDKN FITAEELRRE LPPDQAEYCI ARMAPYQGPD AVPGALDYKS
901 FSTALYGESD L
```

Residue Number Increasing Mass Decreasing Mass

Start - End	Observed	Mr(expt)	Mr(calc)	Delta	Miss	Sequence
55 - 65	1392.6842	1391.6769	1391.6404	0.0365	0	TFTAWCNSHLR (No match)
55 - 66	1520.7809	1519.7736	1519.7354	0.0382	1	TFTAWCNSHLRK (No match)
67 - 83	1920.9883	1919.9810	1919.9224	0.0586	1	AGTQIENIDEDFRDGLK (No match)
153 - 166	1537.8077	1536.8004	1536.7671	0.0333	0	FAIQDISVEETSAK (No match)
167 - 175	1174.6332	1173.6259	1173.5964	0.0295	0	EGLLLWCQR (Ions score 46)
167 - 175	1174.6332	1173.6259	1173.5964	0.0295	0	EGLLLWCQR (No match)
182 - 193	1485.8289	1484.8216	1484.7524	0.0692	0	NVNVQNFHISWK (No match)
194 - 204	1226.6964	1225.6891	1225.6567	0.0324	0	DGLAFNALIHR (Ions score 41)
194 - 204	1226.6964	1225.6891	1225.6567	0.0324	0	DGLAFNALIHR (No match)
205 - 214	1299.7036	1298.6963	1298.6619	0.0345	0	HRPELIEYDK (No match)
205 - 216	1568.8937	1567.8864	1567.8470	0.0394	1	HRPELIEYDKLR (No match)
240 - 255	1816.9144	1815.9071	1815.8672	0.0399	0	MLDAEDIVNTARPDEK (No match)
256 - 273	2008.0116	2007.0043	2006.9560	0.0484	0	AIMTYVSSFYHAFSGAQK (No match)
284 - 300	2061.0144	2060.0071	2059.9520	0.0551	0	VLAVNQENEHLMEDYEK (No match)
312 - 319	1029.5713	1028.5640	1028.5291	0.0350	0	TIPWLEDR (No match)
312 - 323	1481.8496	1480.8423	1480.8038	0.0386	1	TIPWLEDRVPQK (No match)
332 - 339	1113.5597	1112.5524	1112.5250	0.0274	1	LEDFRDYR (No match)
351 - 363	1608.8411	1607.8338	1607.7977	0.0361	0	CQLEINFNTLQTK (No match)
366 - 378	1433.7584	1432.7511	1432.7132	0.0379	0	LSNRPAFMPSEGK (No match)
438 - 450	1548.8024	1547.7951	1547.7579	0.0372	1	HRDYETATLSDIK (No match)
455 - 469	1753.8715	1752.8642	1752.8179	0.0463	1	KHEAFESDLAAHQDR (No match)
498 - 512	1758.8685	1757.8612	1757.8155	0.0457	0	ICDQWDALGSLTHSR (No match)
522 - 536	1827.0170	1826.0097	1825.9686	0.0411	1	QLEAIDQLHLEYAKR (No match)
575 - 584	1159.6006	1158.5933	1158.5629	0.0304	1	STLPDADRER (No match)
605 - 622	1920.0547	1919.0474	1919.0000	0.0475	0	LSGSNPYTTVTPQIINSK (No match)
644 - 651	1011.5203	1010.5130	1010.4893	0.0237	0	QQSNEHLR (No match)
644 - 652	1167.6273	1166.6200	1166.5904	0.0296	1	QQSNEHLRR (No match)
724 - 733	1301.6466	1300.6393	1300.5982	0.0411	0	HTNYTMEHIR (No match)
734 - 745	1386.8058	1385.7985	1385.7666	0.0319	0	VGWEQLLTTIAR (No match)
746 - 757	1429.8003	1428.7930	1428.7572	0.0358	0	TINEVENQILTR (No match)
746 - 757	1429.8003	1428.7930	1428.7572	0.0358	0	TINEVENQILTR (Ions score 39)
761 - 771	1352.6599	1351.6526	1351.6190	0.0336	0	GISQEQMQEFR (No match)
761 - 771	1352.6599	1351.6526	1351.6190	0.0336	0	GISQEQMQEFR (Ions score 41)
761 - 771	1368.6542	1367.6469	1367.6139	0.0330	0	GISQEQMQEFR Oxidation (M) (No match)
772 - 791	2203.0706	2202.0633	2202.0129	0.0504	1	ASFNHFDKDHGGALGPEEFK (No match)
792 - 805	1624.8099	1623.8026	1623.7562	0.0464	0	ACLISLGYDVENDR (No match)
806 - 813	950.4553	949.4480	949.4253	0.0227	0	QGEAEFNR (No match)

854 - 868	1675.9427	1674.9354	1674.8940	0.0414	1	VLAGDKNFITAEELR (No match)
860 - 868	1092.5989	1091.5916	1091.5611	0.0306	0	NFITAEELR (No match)
870 - 882	1561.7706	1560.7633	1560.7242	0.0391	0	ELPPDQAEYCIAR (Ions score 70)
870 - 882	1561.7706	1560.7633	1560.7242	0.0391	0	ELPPDQAEYCIAR (No match)
883 - 899	1792.9027	1791.8954	1791.8501	0.0453	0	MAPYQGPDAVPGALDYK (No match)

Mascot: http://www.matrixscience.com/

Spot 144

MATRIX SCIENCE Mascot Search Results

Protein View

Match to: **gi|119577215** Score: **705** Expect: **6.1e-066**
actinin, alpha 4, isoform CRA_c [Homo sapiens]

Nominal mass (M_r): **104555**; Calculated pI value: **5.24**
NCBI BLAST search of gi|119577215 against nr
Unformatted sequence string for pasting into other applications

Taxonomy: Homo sapiens

Fixed modifications: Carbamidomethyl (C)
Variable modifications: Oxidation (M)
Cleavage by Trypsin: cuts C-term side of KR unless next residue is P
Sequence Coverage: **55%**

Matched peptides shown in **Bold Red**

1 MVDYHAANQS YQYGPSSAGN GAGGGGSMGD YMAQEDDWDR DLLLDPAWEK
51 QQRK**TFTAWC** **NSHLRKAGTQ** **IENIDEDFRD** **GLKLMLLLEV** **ISGER**LPKPE
101 RGKMRVHKIN NVNKALDFIA SKGVK**LVSIG** **AEEIVDGNAK** MTLGMIWTII
151 LR**FAIQDISV** **EETSAKEGLL** **LWCQR**KTAPY KNVNVQNFHI SWK**DGLAFNA**
201 **LIHRHRPELI** **EYDKLRKDDP** **VTNLNNAFEV** **AEK**YLDIPK**M** **LDAEDIVNTA**
251 **RPDEK**AIMTY VSSFYHAETA ANRICK**VLAV** **NQENEHLMED** **YEKLASDLLE**
301 **WIR**R**TIPWLE** **DRVPQK**TIQE MQQKLEDFRD YRRVHKPPKV QEK**CQLEINF**
351 **NTLQTK**LR**LS** **NRPAFMPSEG** **K**MVSDINNGW QHLEQAEK**GY** **EEWLLNEIR**R
401 LERLDHLAEK FRQKASIHEA WTDGKEAMLK **HRDYETATLS** **DIK**ALIR**KHE**
451 **AFESDLAAHQ** **DRVEQIAAIA** **QELNELDYYD** **SHNVNTR**CQK **ICDQWDALGS**
501 **LTHSR**REALE KTEK**QLEAID** **QLHLEYAK**RA APFNNWMESA MEDLQDMFIV
551 HTIEEIEGLI SAHDQFK**STL** **PDADRER**EAI LAIHKEAQRI AESNHIK**LSG**
601 **SNPYTTVTPQ** **IINSK**WEKVQ QLVPK**RDHAL** **LEEQSKQQSN** **EHLR**R**QFASQ**
651 **ANVVGPWIQT** **K**MEEIGR**ISI** **EMNGTLEDQL** **SHLK**QYERSI VDYKPNLDLL
701 EQQHQLIQEA LIFDNK**HTNY** **TMEHIRVGWE** **QLLTTIARTI** **NEVENQILTR**
751 DAK**GISQEQM** **QEFRASFNHF** **DKDHGGALGP** **EEFKACLISL** **GYDVENDRQG**
801 **EAEFNR**IMSL VDPNHSGLVT FQAFIDFMSR ETTDTDTADQ VIASFK**VLAG**
851 **DKNFITAEEL** R**RELPPDQAE** **YCIARMAPYQ** **GPDAVPGALD** **YK**SFSTALYG
901 ESDL

Residue Number Increasing Mass Decreasing Mass

Start - End	Observed	Mr(expt)	Mr(calc)	Delta	Miss	Sequence
55 - 65	1392.6261	1391.6188	1391.6404	-0.0216	0	TFTAWCNSHLR (No match)
55 - 66	1520.7153	1519.7080	1519.7354	-0.0274	1	TFTAWCNSHLRK (No match)
67 - 83	1920.9166	1919.9093	1919.9224	-0.0131	1	AGTQIENIDEDFRDGLK (No match)
84 - 95	1388.7573	1387.7500	1387.7744	-0.0244	0	LMLLLEVISGER Oxidation (M) (No match)
126 - 140	1514.7731	1513.7658	1513.7987	-0.0329	0	LVSIGAEEIVDGNAK (No match)
153 - 166	1537.7467	1536.7394	1536.7671	-0.0277	0	FAIQDISVEETSAK (No match)
167 - 175	1174.5851	1173.5778	1173.5964	-0.0186	0	EGLLLWCQR (No match)
194 - 204	1226.6426	1225.6353	1225.6567	-0.0214	0	DGLAFNALIHR (Ions score 48)
194 - 204	1226.6426	1225.6353	1225.6567	-0.0214	0	DGLAFNALIHR (No match)
205 - 214	1299.6475	1298.6402	1298.6619	-0.0216	0	HRPELIEYDK (No match)
205 - 216	1568.8252	1567.8179	1567.8470	-0.0291	1	HRPELIEYDKLR (No match)
217 - 233	1903.9133	1902.9060	1902.9322	-0.0262	1	KDDPVTNLNNAFEVAEK (No match)
240 - 255	1816.8466	1815.8393	1815.8672	-0.0279	0	MLDAEDIVNTARPDEK (No match)
240 - 255	1832.8669	1831.8596	1831.8621	-0.0025	0	MLDAEDIVNTARPDEK Oxidation (M) (No match)
277 - 293	2076.9470	2075.9397	2075.9469	-0.0072	0	VLAVNQENEHLMEDYEK Oxidation (M) (No match)
294 - 303	1215.6462	1214.6389	1214.6659	-0.0270	0	LASDLLEWIR (No match)
305 - 312	1029.5199	1028.5126	1028.5291	-0.0164	0	TIPWLEDR (No match)
305 - 316	1481.7858	1480.7785	1480.8038	-0.0252	1	TIPWLEDRVPQK (No match)
344 - 356	1608.7773	1607.7700	1607.7977	-0.0277	0	CQLEINFNTLQTK (No match)
359 - 371	1433.7012	1432.6939	1432.7132	-0.0193	0	LSNRPAFMPSEGK (No match)
359 - 371	1449.6930	1448.6857	1448.7081	-0.0224	0	LSNRPAFMPSEGK Oxidation (M) (No match)
389 - 399	1421.6859	1420.6786	1420.6986	-0.0200	0	GYEEWLLNEIR (No match)
431 - 443	1548.7416	1547.7343	1547.7579	-0.0236	1	HRDYETATLSDIK (No match)
448 - 462	1753.7998	1752.7925	1752.8179	-0.0254	1	KHEAFESDLAAHQDR (Ions score 64)
448 - 462	1753.7998	1752.7925	1752.8179	-0.0254	1	KHEAFESDLAAHQDR (No match)
463 - 487	2905.3401	2904.3328	2904.3889	-0.0561	0	VEQIAAIAQELNELDYYDSHNVNTR (No match)
491 - 505	1758.8005	1757.7932	1757.8155	-0.0223	0	ICDQWDALGSLTHSR (No match)
515 - 528	1670.8490	1669.8417	1669.8675	-0.0258	0	QLEAIDQLHLEYAK (No match)
568 - 577	1159.5518	1158.5445	1158.5629	-0.0184	1	STLPDADRER (No match)
598 - 615	1919.9800	1918.9727	1919.0000	-0.0272	0	LSGSNPYTTVTPQIINSK (No match)
626 - 636	1325.6600	1324.6527	1324.6735	-0.0208	1	RDHALLEEQSK (No match)
637 - 644	1011.4857	1010.4784	1010.4893	-0.0109	0	QQSNEHLR (No match)
646 - 661	1773.8842	1772.8769	1772.9209	-0.0440	0	QFASQANVVGPWIQTK (No match)
668 - 684	1927.9399	1926.9326	1926.9720	-0.0394	0	ISIEMNGTLEDQLSHLK (No match)
668 - 684	1943.9484	1942.9411	1942.9669	-0.0258	0	ISIEMNGTLEDQLSHLK Oxidation (M) (No match)
717 - 726	1301.6188	1300.6115	1300.5982	0.0133	0	HTNYTMEHIR (No match)
717 - 726	1317.5797	1316.5724	1316.5931	-0.0207	0	HTNYTMEHIR Oxidation (M) (No match)
727 - 738	1386.7488	1385.7415	1385.7666	-0.0251	0	VGWEQLLTTIAR (No match)
739 - 750	1429.7419	1428.7346	1428.7572	-0.0226	0	TINEVENQILTR (Ions score 23)
739 - 750	1429.7419	1428.7346	1428.7572	-0.0226	0	TINEVENQILTR (No match)
754 - 764	1352.6071	1351.5998	1351.6190	-0.0192	0	GISQEQMQEFR (No match)
754 - 764	1368.6008	1367.5935	1367.6139	-0.0204	0	GISQEQMQEFR Oxidation (M) (No match)
754 - 764	1368.6008	1367.5935	1367.6139	-0.0204	0	GISQEQMQEFR Oxidation (M) (Ions score 33)
765 - 784	2202.9941	2201.9868	2202.0129	-0.0261	1	ASFNHFDKDHGGALGPEEFK (No match)
773 - 784	1256.5765	1255.5692	1255.5832	-0.0140	0	DHGGALGPEEFK (No match)

785 - 798	1624.7399	1623.7326	1623.7562	-0.0236	0	**ACLISLGYDVENDR** (No match)
799 - 806	950.4183	949.4110	949.4253	-0.0143	0	**QGEAEFNR** (No match)
847 - 861	1675.8721	1674.8648	1674.8940	-0.0292	1	**VLAGDKNFITAEELR** (No match)
853 - 861	1092.5498	1091.5425	1091.5611	-0.0185	0	**NFITAEELR** (No match)
863 - 875	1561.7080	1560.7007	1560.7242	-0.0235	0	**ELPPDQAEYCIAR** (Ions score 74)
863 - 875	1561.7080	1560.7007	1560.7242	-0.0235	0	**ELPPDQAEYCIAR** (No match)
876 - 892	1808.8075	1807.8002	1807.8450	-0.0448	0	**MAPYQGPDAVPGALDYK** Oxidation (M) (No match)

Mascot: http://www.matrixscience.com/

Spot 146

{MATRIX}{SCIENCE} Mascot Search Results

Protein View

Match to: **gi|50513540** Score: **251** Expect: **1.5e-020**
Chain A, Moesin Ferm Domain Bound To Ebp50 C-Terminal Peptide

Nominal mass (M_r): **35010**; Calculated pI value: **9.03**
NCBI BLAST search of gi|50513540 against nr
Unformatted sequence string for pasting into other applications

Taxonomy: Homo sapiens

Fixed modifications: Carbamidomethyl (C)
Variable modifications: Oxidation (M)
Cleavage by Trypsin: cuts C-term side of KR unless next residue is P
Sequence Coverage: **17%**

Matched peptides shown in **Bold Red**

1 MPKTISVRVT TMDAELEFAI QPNTTGKQLF DQVVKTIGLR EVWFFGLQYQ
51 DTKGFSTWLK LNKKVTAQDV RKESPLLFKF R**AKFYPEDVS EELIQDITQR**
101 LFFLQVKEGI LNDDIYCPPE TAVLLASYAV QSKYGDFNKE VHKSGYLAGD
151 KLLPQR**VLEQ HKLNK**DQWEE RIQVWHEEHR **GMLREDAVLE YLK**IAQDLEM
201 YGVNYFSIKN KKGSELWLGV DALGLNIYEQ NDRLTPKIGF PWSEIRNISF
251 NDKKFVIKPI DK**KAPDFVFY APR**LRINKRI LALCMGNHEL YMRRRKP

Residue Number Increasing Mass Decreasing Mass

Start - End	Observed	Mr(expt)	Mr(calc)	Delta	Miss	Sequence
82 - 100	2281.1287	2280.1214	2280.1273	-0.0059	1	AKFYPEDVSEELIQDITQR (No match)
84 - 100	2081.9973	2080.9900	2080.9953	-0.0052	0	FYPEDVSEELIQDITQR (Ions score 104)
84 - 100	2081.9973	2080.9900	2080.9953	-0.0052	0	FYPEDVSEELIQDITQR (No match)
157 - 165	1108.5803	1107.5730	1107.6400	-0.0670	1	VLEQHKLNK (No match)
157 - 165	1108.5803	1107.5730	1107.6400	-0.0670	1	VLEQHKLNK (No match)
181 - 193	1552.7983	1551.7910	1551.7966	-0.0056	1	GMLREDAVLEYLK Oxidation (M) (No match)
263 - 273	1310.6891	1309.6818	1309.6818	-0.0000	1	KAPDFVFYAPR (No match)
263 - 273	1310.6891	1309.6818	1309.6818	-0.0000	1	KAPDFVFYAPR (Ions score 19)
264 - 273	1182.5935	1181.5862	1181.5869	-0.0007	0	APDFVFYAPR (Ions score 63)

264 - 273	1182.5935	1181.5862	1181.5869	-0.0007	0	APDFVFYAPR	(No match)

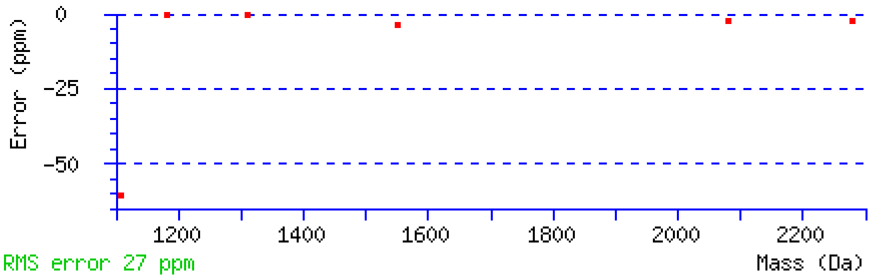

RMS error 27 ppm

Mascot: http://www.matrixscience.com/

Spot 147

{MATRIX SCIENCE} Mascot Search Results

Protein View

Match to: **gi|4503483** Score: **267** Expect: **3.8e-022**
eukaryotic translation elongation factor 2 [Homo sapiens]

Nominal mass (M_r): **96246;** Calculated pI value: **6.41**
NCBI BLAST search of gi|4503483 against nr
Unformatted sequence string for pasting into other applications

Taxonomy: Homo sapiens
Links to retrieve other entries containing this sequence from NCBI Entrez:
gi|119172 from Homo sapiens
gi|88909610 from Pongo pygmaeus
gi|31106 from Homo sapiens
gi|31108 from Homo sapiens
gi|60685056 from Homo sapiens
gi|116496673 from Homo sapiens
gi|119589680 from Homo sapiens
gi|119589681 from Homo sapiens

Fixed modifications: Carbamidomethyl (C)
Variable modifications: Oxidation (M)
Cleavage by Trypsin: cuts C-term side of KR unless next residue is P
Sequence Coverage: **15%**

Matched peptides shown in **Bold Red**

```
  1 MVNFTVDQIR AIMDKKANIR NMSVIAHVDH GKSTLTDSLV CKAGIIASAR
 51 AGETRFTDTR KDEQERCITI KSTAISLFYE LSENDLNFIK QSKDGAGFLI
101 NLIDSPGHVD FSSEVTAALR VTDGALVVVD CVSGVCVQTE TVLRQAIAER
151 IKPVLMMNKM DRALLELQLE PEELYQTFQR IVENVNVIIS TYGEGESGPM
201 GNIMIDPVLG TVGFGSGLHG WAFTLKQFAE MYVAKFAAKG EGQLGPAERA
251 KKVEDMMKKL WGDRYFDPAN GKFSKSATSP EGKKLPRTFC QLILDPIFKV
301 FDAIMNFKKE ETAKLIEKLD IKLDSEDKDK EGKPLLKAVM RRWLPAGDAL
351 LQMITIHLPS PVTAQKYRCE LLYEGPPDDE AAMGIKSCDP KGPLMMYISK
401 MVPTSDKGRF YAFGRVFSGL VSTGLKVRIM GPNYTPGKKE DLYLKPIQRT
451 ILMMGRYVEP IEDVPCGNIV GLVGVDQFLV KTGTITTFEH AHNMRVMKFS
501 VSPVVRVAVE AKNPADLPKL VEGLKRLAKS DPMVQCIIEE SGEHIIAGAG
551 ELHLEICLKD LEEDHACIPI KKSDPVVSYR ETVSEESNVL CLSKSPNKHN
601 RLYMKARPFP DGLAEDIDKG EVSARQELKQ RARYLAEKYE WDVAEARKIW
```

```
651 CFGPDGTGPN ILTDITKGVQ YLNEIKDSVV AGFQWATKEG ALCEENMRGV
701 RFDVHDVTLH ADAIHRGGGQ IIPTARRCLY ASVLTAQPRL MEPIYLVEIQ
751 CPEQVVGGIY GVLNRKRGHV FEESQVAGTP MFVVKAYLPV NESFGFTADL
801 RSNTGGQAFP QCVFDHWQIL PGDPFDNSSR PSQVVAETRK RKGLKEGIPA
851 LDNFLDKL
```

Residue Number Increasing Mass Decreasing Mass

Start - End	Observed	Mr(expt)	Mr(calc)	Delta	Miss	Sequence
2 - 10	1091.5818	1090.5745	1090.5771	-0.0025	0	VNFTVDQIR (Ions score 14)
2 - 10	1091.5818	1090.5745	1090.5771	-0.0025	0	VNFTVDQIR (No match)
61 - 66	804.3782	803.3709	803.3773	-0.0064	1	KDEQER (No match)
163 - 180	2220.1555	2219.1482	2219.1473	0.0009	0	ALLELQLEPEELYQTFQR (No match)
240 - 249	1013.5047	1012.4974	1012.4937	0.0037	0	GEGQLGPAER (Ions score 4)
240 - 249	1013.5047	1012.4974	1012.4937	0.0037	0	GEGQLGPAER (No match)
439 - 449	1402.8103	1401.8030	1401.7979	0.0051	1	KEDLYLKPIQR (No match)
440 - 449	1274.7134	1273.7061	1273.7030	0.0031	0	EDLYLKPIQR (No match)
482 - 495	1631.7656	1630.7583	1630.7522	0.0062	0	TGTITTFEHAHNMR Oxidation (M) (No match)
499 - 506	890.5063	889.4990	889.5021	-0.0031	0	FSVSPVVR (No match)
606 - 625	2143.0850	2142.0777	2142.0705	0.0073	1	ARPFPDGLAEDIDKGEVSAR (No match)
606 - 625	2143.0850	2142.0777	2142.0705	0.0073	1	ARPFPDGLAEDIDKGEVSAR (Ions score 15)
717 - 726	969.5450	968.5377	968.5403	-0.0025	0	GGGQIIPTAR (No match)
727 - 739	1534.8187	1533.8114	1533.8085	0.0029	1	RCLYASVLTAQPR (No match)
728 - 739	1378.7131	1377.7058	1377.7074	-0.0016	0	CLYASVLTAQPR (Ions score 52)
728 - 739	1378.7131	1377.7058	1377.7074	-0.0016	0	CLYASVLTAQPR (No match)
786 - 801	1799.8988	1798.8915	1798.8889	0.0026	0	AYLPVNESFGFTADLR (No match)
786 - 801	1799.8988	1798.8915	1798.8889	0.0026	0	AYLPVNESFGFTADLR (Ions score 71)

Mascot: http://www.matrixscience.com/

Spot 148

Mascot Search Results

Protein View

Match to: **gi|56549121** Score: **157** Expect: **3.8e-011**
dynamin 2 isoform 1 [Homo sapiens]

Nominal mass (M_r): **98345**; Calculated pI value: **7.04**
NCBI BLAST search of gi|56549121 against nr
Unformatted sequence string for pasting into other applications

Taxonomy: Homo sapiens
Links to retrieve other entries containing this sequence from NCBI Entrez:
gi|47117856 from Homo sapiens
gi|56969514 from Homo sapiens

Fixed modifications: Carbamidomethyl (C)
Variable modifications: Oxidation (M)
Cleavage by Trypsin: cuts C-term side of KR unless next residue is P
Sequence Coverage: **18%**

Matched peptides shown in **Bold Red**

```
  1 MGNRGMEELI PLVNKLQDAF SSIGQSCHLD LPQIAVVGGQ SAGKSSVLEN
 51 FVGRDFLPRG SGIVTRRPLI LQLIFSKTEH AEFLHCKSKK FTDFDEVRQE
101 IEAETDRVTG TNKGISPVPI NLRVYSPHVL NLTLIDLPGI TKVPVGDQPP
151 DIEYQIKDMI LQFISRESSL ILAVTPANMD LANSDALKLA KEVDPQGLRT
201 IGVITKLDLM DEGTDARDVL ENKLLPLRRG YIGVVNRSQK DIEGKKDIRA
251 ALAAERKFFL SHPAYRHMAD RMGTPHLQKT LNQQLTNHIR ESLPALRSKL
301 QSQLLSLEKE VEEYKNFRPD DPTRKTKALL QMVQQFGVDF EKRIEGSGDQ
351 VDTLELSGGA RINRIFHERF PFELVKMEFD EKDLRREISY AIKNIHGVRT
401 GLFTPDLAFE AIVKKQVVKL KEPCLKCVDL VIQELINTVR QCTSKLSSYP
451 RLREETERIV TTYIREREGR TKDQILLLID IEQSYINTNH EDFIGFANAQ
501 QRSTQLNKKR AIPNQGEILV IRRGWLTINN ISLMKGGSKE YWFVLTAESL
551 SWYKDEEEKE KKYMLPLDNL KIRDVEKGFM SNKHVFAIFN TEQRNVYKDL
601 RQIELACDSQ EDVDSWKASF LRAGVYPEKD QAENEDGAQE NTFSMDPQLE
651 RQVETIRNLV DSYVAIINKS IRDLMPKTIM HLMINNTKAF IHHELLAYLY
701 SSADQSSLME ESADQAQRRD DMLRMYHALK EALNIIGDIS TSTVSTPVPP
751 PVDDTWLQSA SSHSPTPQRR PVSSIHPPGR PPAVRGPTPG PPLIPVPVGA
801 AASFSAPPIP SRPGPQSVFA NSDLFPAPPQ IPSRPVRIPP GIPPGVPSRR
851 PPAAPSRPTI IRPAEPSLLD
```

Residue Number Increasing Mass Decreasing Mass

Start - End	Observed	Mr(expt)	Mr(calc)	Delta	Miss	Sequence
45 - 59	1736.0190	1735.0117	1734.9052	0.1065	1	SSVLENFVGRDFLPR (Ions score 15)
45 - 59	1736.0190	1735.0117	1734.9052	0.1065	1	SSVLENFVGRDFLPR (No match)
67 - 77	1327.9269	1326.9196	1326.8387	0.0809	0	RPLILQLIFSK (No match)
114 - 123	1065.7009	1064.6936	1064.6342	0.0595	0	GISPVPINLR (No match)
114 - 123	1065.7009	1064.6936	1064.6342	0.0595	0	GISPVPINLR (No match)
258 - 266	1137.6516	1136.6443	1136.5767	0.0677	0	FFLSHPAYR (No match)
280 - 290	1337.8129	1336.8056	1336.7211	0.0845	0	TLNQQLTNHIR (No match)
280 - 297	2104.2900	2103.2827	2103.1548	0.1279	1	TLNQQLTNHIRESLPALR (No match)
328 - 343	1925.1133	1924.1060	1923.9876	0.1184	1	ALLQMVQQFGVDFEKR Oxidation (M) (No match)
344 - 361	1803.9928	1802.9855	1802.8645	0.1210	0	IEGSGDQVDTLELSGGAR (No match)
365 - 376	1561.9451	1560.9378	1560.8452	0.0926	1	IFHERFPFELVK (No match)
511 - 522	1322.8611	1321.8538	1321.7717	0.0821	0	AIPNQGEILVIR (No match)
511 - 522	1322.8611	1321.8538	1321.7717	0.0821	0	AIPNQGEILVIR (No match)
511 - 523	1478.9712	1477.9639	1477.8728	0.0911	1	AIPNQGEILVIRR (No match)
511 - 523	1478.9712	1477.9639	1477.8728	0.0911	1	AIPNQGEILVIRR (Ions score 16)
584 - 594	1361.7778	1360.7705	1360.6887	0.0818	0	HVFAIFNTEQR (No match)
770 - 785	1723.0916	1722.0843	1721.9801	0.1042	0	RPVSSIHPPGRPPAVR (No match)
838 - 849	1186.7666	1185.7593	1185.6869	0.0724	0	IPPGIPPGVPSR (Ions score 22)
838 - 849	1186.7666	1185.7593	1185.6869	0.0724	0	IPPGIPPGVPSR (No match)

Mascot: http://www.matrixscience.com/

Spot 149

MATRIX SCIENCE Mascot Search Results

Protein View

Match to: **gi|119625804** Score: **198** Expect: **3e-015**
moesin, isoform CRA_b [Homo sapiens]

Nominal mass (M_r): **66678;** Calculated pI value: **5.90**
NCBI BLAST search of gi|119625804 against nr
Unformatted sequence string for pasting into other applications

Taxonomy: Homo sapiens

Fixed modifications: Carbamidomethyl (C)
Variable modifications: Oxidation (M)
Cleavage by Trypsin: cuts C-term side of KR unless next residue is P
Sequence Coverage: **30%**

Matched peptides shown in **Bold Red**

1 MDAELEFAIQ PNTTGK**QLFD QVVK**TIGLRE VWFFGLQYQD TKGFSTWLKL
51 NKKVTAQDVR KESPLLFKFR **AKFYPEDVSE ELIQDITQRL FFLQVK**EGIL
101 NDDIYCPPET AVLLASYAVQ SK**YGDFNKEV HK**SGYLAGDK LLPQRVLEQH
151 KLNKDQWEER **IQVWHEEHRG MLREDAVLEY LK**IAQDLEMY GVNYFSIKNK
201 KGSELWLGVD ALGLNIYEQN DRLTPK**IGFP WSEIR**NISFN DKKFVIKPID
251 K**KAPDFVFYA PR**LRINKR**IL ALCMGNHELY MR**R**RKPDTIE VQQMK**AQARE
301 EKHQKQMERA MLENEKKKRE MAEKEKEKIE R**EKEELMER**L KQIEEQTKK**A**
351 **QQELEEQTRR ALELEQER**KR AQSEAEKLAK ERQEAEEAKE ALLQASRDQK
401 **KTQEQLALEM AELTAR**ISQL EMARQKKESE AVEWQQKAQM VQEDLEKTRA
451 ELKTAMSTPH VAEPAENEQD EQDENGAEAS ADLRADAMAK DRSEEER**TTE**
501 **AEKNER**VQKH LKALTSELAN ARDESKKTAN DMIHAENMRL GRDKYKTLRQ
551 IRQGNTK**QRI DEFESM**

Residue Number Increasing Mass Decreasing Mass

Start - End	Observed	Mr(expt)	Mr(calc)	Delta	Miss	Sequence
17 - 24	976.5357	975.5284	975.5389	-0.0105	0	QLFDQVVK (No match)
71 - 89	2281.1047	2280.0974	2280.1273	-0.0299	1	AKFYPEDVSEELIQDITQR (No match)
73 - 89	2081.9902	2080.9829	2080.9953	-0.0123	0	FYPEDVSEELIQDITQR (No match)

90 - 96	894.5275	893.5202	893.5374	-0.0172	0	**LFFLQVK** (No match)
123 - 132	1236.5721	1235.5648	1235.5934	-0.0286	1	**YGDFNKEVHK** (No match)
161 - 169	1233.5936	1232.5863	1232.6050	-0.0187	0	**IQVWHEEHR** (No match)
170 - 182	1552.7904	1551.7831	1551.7966	-0.0135	1	**GMLREDAVLEYLK** Oxidation (M) (No match)
227 - 235	1104.5774	1103.5701	1103.5763	-0.0062	0	**IGFPWSEIR** (No match)
252 - 262	1310.6820	1309.6747	1309.6818	-0.0071	1	**KAPDFVFYAPR** (Ions score 8)
252 - 262	1310.6820	1309.6747	1309.6818	-0.0071	1	**KAPDFVFYAPR** (No match)
253 - 262	1182.5859	1181.5786	1181.5869	-0.0083	0	**APDFVFYAPR** (Ions score 42)
253 - 262	1182.5859	1181.5786	1181.5869	-0.0083	0	**APDFVFYAPR** (No match)
269 - 282	1752.8066	1751.7993	1751.8157	-0.0163	0	**ILALCMGNHELYMR** 2 Oxidation (M) (No match)
284 - 295	1488.7736	1487.7663	1487.7766	-0.0102	1	**RKPDTIEVQQMK** Oxidation (M) (No match)
332 - 339	1079.5046	1078.4973	1078.4964	0.0009	1	**EKEELMER** Oxidation (M) (No match)
350 - 360	1387.6818	1386.6745	1386.6851	-0.0106	1	**AQQELEEQTRR** (No match)
361 - 368	987.5052	986.4979	986.5032	-0.0053	0	**ALELEQER** (No match)
401 - 416	1847.9458	1846.9385	1846.9458	-0.0073	1	**KTQEQLALEMAELTAR** Oxidation (M) (No match)
402 - 416	1719.8473	1718.8400	1718.8508	-0.0108	0	**TQEQLALEMAELTAR** Oxidation (M) (No match)
498 - 506	1077.5087	1076.5014	1076.5098	-0.0083	1	**TTEAEKNER** (No match)
558 - 566	1170.4985	1169.4912	1169.5022	-0.0110	1	**QRIDEFESM** Oxidation (M) (No match)

Mascot: http://www.matrixscience.com/

Spot 151

MATRIX SCIENCE Mascot Search Results

Protein View

Match to: **gi|4503483** Score: **98** Expect: **3e-005**
eukaryotic translation elongation factor 2 [Homo sapiens]

Nominal mass (M_r): **96246;** Calculated pI value: **6.41**
NCBI BLAST search of gi|4503483 against nr
Unformatted sequence string for pasting into other applications

Taxonomy: Homo sapiens
Links to retrieve other entries containing this sequence from NCBI Entrez:
gi|119172 from Homo sapiens
gi|88909610 from Pongo pygmaeus
gi|31106 from Homo sapiens
gi|31108 from Homo sapiens
gi|60685056 from Homo sapiens
gi|116496673 from Homo sapiens
gi|119589680 from Homo sapiens
gi|119589681 from Homo sapiens

Fixed modifications: Carbamidomethyl (C)
Variable modifications: Oxidation (M)
Cleavage by Trypsin: cuts C-term side of KR unless next residue is P
Sequence Coverage: **10%**

Matched peptides shown in **Bold Red**

1 M**VNFTVDQIR** AIMDKKANIR NMSVIAHVDH GKSTLTDSLV CKAGIIASAR
51 AGETRFTDTR KDEQERCITI KSTAISLFYE LSENDLNFIK QSKDGAGFLI
101 NLIDSPGHVD FSSEVTAALR VTDGALVVVD CVSGVCVQTE TVLRQAIAER
151 IKPVLMMNKM DR**ALLELQLE PEELYQTFQR** IVENVNVIIS TYGEGESGPM
201 GNIMIDPVLG TVGFGSGLHG WAFTLKQFAE MYVAKFAAKG EGQLGPAERA
251 KKVEDMMKKL WGDRYFDPAN GKFSKSATSP EGKKLPRTFC QLILDPIFKV
301 FDAIMNFKKE ETAKLIEKLD IKLDSEDKDK EGKPLLKAVM RRWLPAGDAL
351 LQMITIHLPS PVTAQKYRCE LLYEGPPDDE AAMGIKSCDP KGPLMMYISK
401 MVPTSDKGR**F YAFGR**VFSGL VSTGLKVRIM GPNYTPGKK**E DLYLKPIQR**T
451 ILMMGRYVEP IEDVPCGNIV GLVGVDQFLV KTGTITTFEH AHNMRVMKFS
501 VSPVVRVAVE AKNPADLPKL VEGLKRLAKS DPMVQCIIEE SGEHIIAGAG
551 ELHLEICLKD LEEDHACIPI KKSDPVVSYR ETVSEESNVL CLSKSPNKHN
601 RLYMK**ARPFP DGLAEDIDKG EVSAR**QELKQ RARYLAEKYE WDVAEARKIW

```
651 CFGPDGTGPN ILTDITKGVQ YLNEIKDSVV AGFQWATKEG ALCEENMRGV
701 RFDVHDVTLH ADAIHRGGGQ IIPTARRCLY ASVLTAQPRL MEPIYLVEIQ
751 CPEQVVGGIY GVLNRKRGHV FEESQVAGTP MFVVKAYLPV NESFGFTADL
801 RSNTGGQAFP QCVFDHWQIL PGDPFDNSSR PSQVVAETRK RKGLKEGIPA
851 LDNFLDKL
```

Residue Number Increasing Mass Decreasing Mass

Start - End	Observed	Mr(expt)	Mr(calc)	Delta	Miss	Sequence
2 - 10	1091.5630	1090.5557	1090.5771	-0.0213	0	VNFTVDQIR (No match)
2 - 10	1091.5630	1090.5557	1090.5771	-0.0213	0	VNFTVDQIR (No match)
163 - 180	2220.1208	2219.1135	2219.1473	-0.0338	0	ALLELQLEPEELYQTFQR (Ions score 26)
163 - 180	2220.1208	2219.1135	2219.1473	-0.0338	0	ALLELQLEPEELYQTFQR (No match)
410 - 415	760.3719	759.3646	759.3703	-0.0057	0	FYAFGR (No match)
440 - 449	1274.6865	1273.6792	1273.7030	-0.0238	0	EDLYLKPIQR (No match)
606 - 625	2143.0449	2142.0376	2142.0705	-0.0328	1	ARPFPDGLAEDIDKGEVSAR (No match)
728 - 739	1378.6996	1377.6923	1377.7074	-0.0151	0	CLYASVLTAQPR (Ions score 6)
728 - 739	1378.6996	1377.6923	1377.7074	-0.0151	0	CLYASVLTAQPR (No match)
786 - 801	1799.8623	1798.8550	1798.8889	-0.0339	0	AYLPVNESFGFTADLR (Ions score 22)
786 - 801	1799.8623	1798.8550	1798.8889	-0.0339	0	AYLPVNESFGFTADLR (No match)

Mascot: http://www.matrixscience.com/

Spot 152

Mascot Search Results

Protein View

Match to: **gi|4503483** Score: **175** Expect: **6.1e-013**
eukaryotic translation elongation factor 2 [Homo sapiens]

Nominal mass (M_r): **96246;** Calculated pI value: **6.41**
NCBI BLAST search of gi|4503483 against nr
Unformatted sequence string for pasting into other applications

Taxonomy: Homo sapiens
Links to retrieve other entries containing this sequence from NCBI Entrez:
gi|119172 from Homo sapiens
gi|88909610 from Pongo pygmaeus
gi|31106 from Homo sapiens
gi|31108 from Homo sapiens
gi|60685056 from Homo sapiens
gi|116496673 from Homo sapiens
gi|119589680 from Homo sapiens
gi|119589681 from Homo sapiens

Fixed modifications: Carbamidomethyl (C)
Variable modifications: Oxidation (M)
Cleavage by Trypsin: cuts C-term side of KR unless next residue is P
Sequence Coverage: **17%**

Matched peptides shown in **Bold Red**

```
  1 MVNFTVDQIR AIMDKKANIR NMSVIAHVDH GKSTLTDSLV CKAGIIASAR
 51 AGETRFTDTR KDEQERCITI KSTAISLFYE LSENDLNFIK QSKDGAGFLI
101 NLIDSPGHVD FSSEVTAALR VTDGALVVVD CVSGVCVQTE TVLRQAIAER
151 IKPVLMMNKM DRALLELQLE PEELYQTFQR IVENVNVIIS TYGEGESGPM
201 GNIMIDPVLG TVGFGSGLHG WAFTLKQFAE MYVAKFAAKG EGQLGPAERA
251 KKVEDMMKKL WGDRYFDPAN GKFSKSATSP EGKKLPRTFC QLILDPIFKV
301 FDAIMNFKKE ETAKLIEKLD IKLDSEDKDK EGKPLLKAVM RRWLPAGDAL
351 LQMITIHLPS PVTAQKYRCE LLYEGPPDDE AAMGIKSCDP KGPLMMYISK
401 MVPTSDKGRF YAFGRVFSGL VSTGLKVRIM GPNYTPGKKE DLYLKPIQRT
451 ILMMGRYVEP IEDVPCGNIV GLVGVDQFLV KTGTITTFEH AHNMRVMKFS
501 VSPVVRVAVE AKNPADLPKL VEGLKRLAKS DPMVQCIIEE SGEHIIAGAG
551 ELHLEICLKD LEEDHACIPI KKSDPVVSYR ETVSEESNVL CLSKSPNKHN
601 RLYMKARPFP DGLAEDIDKG EVSARQELKQ RARYLAEKYE WDVAEARKIW
```

```
651 CFGPDGTGPN ILTDITKGVQ YLNEIKDSVV AGFQWATKEG ALCEENMRGV
701 RFDVHDVTLH ADAIHRGGGQ IIPTARRCLY ASVLTAQPRL MEPIYLVEIQ
751 CPEQVVGGIY GVLNRKRGHV FEESQVAGTP MFVVKAYLPV NESFGFTADL
801 RSNTGGQAFP QCVFDHWQIL PGDPFDNSSR PSQVVAETRK RKGLKEGIPA
851 LDNFLDKL
```

Residue Number Increasing Mass Decreasing Mass

Start - End	Observed	Mr(expt)	Mr(calc)	Delta	Miss	Sequence
2 - 10	1091.6063	1090.5990	1090.5771	0.0220	0	VNFTVDQIR (Ions score 3)
2 - 10	1091.6063	1090.5990	1090.5771	0.0220	0	VNFTVDQIR (No match)
61 - 66	804.4006	803.3933	803.3773	0.0160	1	KDEQER (No match)
163 - 180	2220.1851	2219.1778	2219.1473	0.0305	0	ALLELQLEPEELYQTFQR (No match)
240 - 249	1013.5199	1012.5126	1012.4937	0.0189	0	GEGQLGPAER (No match)
288 - 299	1494.8252	1493.8179	1493.7952	0.0227	0	TFCQLILDPIFK (No match)
439 - 449	1402.8378	1401.8305	1401.7979	0.0326	1	KEDLYLKPIQR (No match)
440 - 449	1274.7363	1273.7290	1273.7030	0.0260	0	EDLYLKPIQR (No match)
440 - 449	1274.7363	1273.7290	1273.7030	0.0260	0	EDLYLKPIQR (Ions score 8)
482 - 495	1615.7953	1614.7880	1614.7572	0.0308	0	TGTITTFEHAHNMR (No match)
482 - 495	1631.7922	1630.7849	1630.7522	0.0328	0	TGTITTFEHAHNMR Oxidation (M) (No match)
499 - 506	890.5273	889.5200	889.5021	0.0179	0	FSVSPVVR (No match)
606 - 625	2143.1165	2142.1092	2142.0705	0.0388	1	ARPFPDGLAEDIDKGEVSAR (No match)
717 - 726	969.5707	968.5634	968.5403	0.0232	0	GGGQIIPTAR (No match)
728 - 739	1378.7448	1377.7375	1377.7074	0.0301	0	CLYASVLTAQPR (Ions score 18)
728 - 739	1378.7448	1377.7375	1377.7074	0.0301	0	CLYASVLTAQPR (No match)
786 - 801	1799.9253	1798.9180	1798.8889	0.0291	0	AYLPVNESFGFTADLR (No match)
786 - 801	1799.9253	1798.9180	1798.8889	0.0291	0	AYLPVNESFGFTADLR (Ions score 55)

Mascot: http://www.matrixscience.com/

Spot 153

{MATRIX SCIENCE} Mascot Search Results

Protein View

Match to: **gi|61656607** Score: **480** Expect: **1.9e-043**
tumor rejection antigen (gp96) 1 [Homo sapiens]

Nominal mass (M_r): **92567;** Calculated pI value: **4.77**
NCBI BLAST search of gi|61656607 against nr
Unformatted sequence string for pasting into other applications

Taxonomy: Homo sapiens

Fixed modifications: Carbamidomethyl (C)
Variable modifications: Oxidation (M)
Cleavage by Trypsin: cuts C-term side of KR unless next residue is P
Sequence Coverage: **26%**

Matched peptides shown in **Bold Red**

```
  1 MRALWVLGLC CVLLTFGSVR ADDEVDVDGT VEEDLGKSRE GSRTDDEVVQ
 51 REEEAIQLDG LNASQIRELR EKSEKFAFQA EVNRMMKLII NSLYKNKEIF
101 LRELISNASD ALDKIRLISL TDENALSGNE ELTVKIKCDK EKNLLHVTDT
151 GVGMTREELV KNLGTIAKSG TSEFLNKMTE AQEDGQSTSE LIGQFGVGFY
201 SAFLVADKVI VTSKHNNDTQ HIWESDSNEF SVIADPRGNT LGRGTTITLV
251 LKEEASDYLE LDTIKNLVKK YSQFINFPIY VWSSKTETVE EPMEEEEAAK
301 EEKEESDDEA AVEEEEEEKK PKTKKVEKTV WDWELMNDIK PIWQRPSKEV
351 EEDEYKAFYK SFSKESDDPM AYIHFTAEGE VTFKSILFVP TSAPRGLFDE
401 YGSKKSDYIK LYVRRVFITD DFHDMMPKYL NFVKGVVDSD DLPLNVSRET
451 LQQHKLLKVI RKKLVRKTLD MIKKIADDKY NDTFWKEFGT NIKLGVIEDH
501 SNRTRLAKLL RFQSSHHPTD ITSLDQYVER MKEKQDKIYF MAGSSRKEAE
551 SSPFVERLLK KGYEVIYLTE PVDEYCIQAL PEFDGKRFQN VAKEGVKFDE
601 SEKTKESREA VEKEFEPLLN WMKDKALKDK IEKAVVSQRL TESPCALVAS
651 QYGWSGNMER IMKAQAYQTG KDISTNYYAS QKKTFEINPR HPLIRDMLRR
701 IKEDEDDKTV LDLAVVLFET ATLRSGYLLP DTKAYGDRIE RMLRLSLNID
751 PDAKVEEEPE EEPEETAEDT TEDTEQDEDE EMDVGTDEEE TAKESTAEKD
801 EL
```

Residue Number Increasing Mass Decreasing Mass

Start - End	Observed	Mr(expt)	Mr(calc)	Delta	Miss	Sequence
44 - 67	2728.4832	2727.4759	2727.3310	0.1449	1	TDDEVVQREEEAIQLDGLNASQIR (No match)
52 - 67	1785.9614	1784.9541	1784.8904	0.0637	0	EEEAIQLDGLNASQIR (No match)
76 - 84	1081.5927	1080.5854	1080.5352	0.0503	0	FAFQAEVNR (No match)
76 - 84	1081.5927	1080.5854	1080.5352	0.0503	0	FAFQAEVNR (Ions score 31)
96 - 102	919.5759	918.5686	918.5286	0.0400	1	NKEIFLR (No match)
103 - 116	1544.9044	1543.8971	1543.8205	0.0766	1	ELISNASDALDKIR (No match)
143 - 156	1529.8447	1528.8374	1528.7667	0.0707	0	NLLHVTDTGVGMTR Oxidation (M) (No match)
143 - 161	2128.2146	2127.2073	2127.0993	0.1080	1	NLLHVTDTGVGMTREELVK Oxidation (M) (No match)
385 - 395	1187.7346	1186.7273	1186.6710	0.0564	0	SILFVPTSAPR (Ions score 52)
385 - 395	1187.7346	1186.7273	1186.6710	0.0564	0	SILFVPTSAPR (No match)
396 - 404	1015.5124	1014.5051	1014.4658	0.0394	0	GLFDEYGSK (Ions score 42)
396 - 404	1015.5124	1014.5051	1014.4658	0.0394	0	GLFDEYGSK (No match)
396 - 405	1143.6206	1142.6133	1142.5607	0.0526	1	GLFDEYGSKK (No match)
415 - 428	1783.9052	1782.8979	1782.8069	0.0910	1	RVFITDDFHDMMPK 2 Oxidation (M) (No match)
416 - 428	1627.7953	1626.7880	1626.7058	0.0822	0	VFITDDFHDMMPK 2 Oxidation (M) (No match)
435 - 448	1485.8287	1484.8214	1484.7470	0.0744	0	GVVDSDDLPLNVSR (Ions score 66)
494 - 503	1139.6361	1138.6288	1138.5730	0.0558	0	LGVIEDHSNR (No match)
512 - 530	2260.1680	2259.1607	2259.0556	0.1051	0	FQSSHHPTDITSLDQYVER (Ions score 77)
512 - 530	2260.1680	2259.1607	2259.0556	0.1051	0	FQSSHHPTDITSLDQYVER (No match)
538 - 546	1047.5374	1046.5301	1046.4855	0.0447	0	IYFMAGSSR Oxidation (M) (No match)
547 - 557	1278.6978	1277.6905	1277.6251	0.0654	1	KEAESSPFVER (No match)
683 - 690	1004.6003	1003.5930	1003.5450	0.0480	1	KTFEINPR (No match)
684 - 690	876.4933	875.4860	875.4501	0.0360	0	TFEINPR (No match)
691 - 699	1150.5945	1149.5872	1149.6441	-0.0568	1	HPLIRDMLR (No match)
734 - 741	979.5357	978.5284	978.4882	0.0402	1	AYGDRIER (No match)
742 - 754	1485.8287	1484.8214	1484.8021	0.0194	1	MLRLSLNIDPDAK (No match)

Mascot: http://www.matrixscience.com/

Spot 154

Mascot Search Results

Protein View

Match to: **gi|148727247** Score: **114** Expect: **7.7e-007**
ubiquitin specific peptidase 5 isoform 2 [Homo sapiens]

Nominal mass (M_r): **94104;** Calculated pI value: **4.96**
NCBI BLAST search of gi|148727247 against nr
Unformatted sequence string for pasting into other applications

Taxonomy: Homo sapiens
Links to retrieve other entries containing this sequence from NCBI Entrez:
gi|1732411 from Homo sapiens
gi|13436149 from Homo sapiens
gi|119609131 from Homo sapiens
gi|119609132 from Homo sapiens
gi|123991812 from synthetic construct
gi|123999428 from synthetic construct

Fixed modifications: Carbamidomethyl (C)
Variable modifications: Oxidation (M)
Cleavage by Trypsin: cuts C-term side of KR unless next residue is P
Sequence Coverage: **11%**

Matched peptides shown in **Bold Red**

1 MAELSEEALL SVLPTIRVPK AGDRVHKDEC AFSFDTPESE GGLYICMNTF
51 LGFGKQYVER HFNKTGQRVY LHLRRTRRPK EEDPATGTGD PPRKKPTRLA
101 IGVEGGFDLS EEKFELDEDV K**IVILPDYLE IAR**DGLGGLP DIVRDRVTSA
151 VEALLSADSA SRKQEVQAWD GEVRQVSKHA FSLKQLDNPA RIPPCGWKCS
201 KCDMRENLWL NLTDGSILCG RR**YFDGSGGN NHAVEHYR**ET GYPLAVKLGT
251 ITPDGADVYS YDEDDMVLDP SLAEHLSHFG IDMLKMQKTD KTMTELEIDM
301 NQRIGEWELI QESGVPLKPL FGPGYTGIRN LGNSCYLNSV VQVLFSIPDF
351 QRKYVDKLEK IFQNAPTDPT QDFSTQVAK**L GHGLLSGEYS KPVPESGDGE**
401 **RVPEQK**EVQD GIAPRMFKAL IGKGHPEFST NRQQDAQEFF LHLINMVERN
451 CRSSENPNEV FRFLVEEKIK CLATEKVKYT QRVDYIMQLP VPMDAALNKE
501 ELLEYEEKKR QAEEEKMALP ELVRAQVPFS SCLEAYGAPE QVDDFWSTAL
551 QAKSVAVKTT RFASFPDYLV IQIKKFTFGL DWVPK**KLDVS IEMPEELDIS**
601 **QLR**GTGLQPG EEELPDIAPP LVTPDEPKAP MLDESVIIQL VEMGFPMDAC
651 RKAVYYTGNS GAEAAMNWVM SHMDDPDFAN PLILPGSSGP GSTSAAADPP
701 PEDCVTTIVS MGFSRDQALK ALRATNNSLE RAVDWIFSHI DDLDAEAAMD

751 ISEGRSAADS ISESVPVGPK VRDGPGKYQL FAFISHMGTS TMCGHYVCHI
801 KKEGRWVIYN DQK**VCASEKP PKDLGYIYFY QR**VAS

Residue Number Increasing Mass Decreasing Mass

Start - End	Observed	Mr(expt)	Mr(calc)	Delta	Miss	Sequence
122 - 133	1414.8411	1413.8338	1413.8231	0.0107	0	IVILPDYLEIAR (No match)
223 - 238	1822.7919	1821.7846	1821.7818	0.0028	0	YFDGSGGNNHAVEHYR (Ions score 37)
223 - 238	1822.7919	1821.7846	1821.7818	0.0028	0	YFDGSGGNNHAVEHYR (No match)
380 - 406	2865.4392	2864.4319	2864.4303	0.0016	1	LGHGLLSGEYSKPVPESGDGERVPEQK (No match)
586 - 603	2131.1021	2130.0948	2130.0878	0.0071	1	KLDVSIEMPEELDISQLR Oxidation (M) (Ions score 20)
586 - 603	2131.1021	2130.0948	2130.0878	0.0071	1	KLDVSIEMPEELDISQLR Oxidation (M) (No match)
814 - 832	2334.1619	2333.1546	2333.1514	0.0033	1	VCASEKPPKDLGYIYFYQR (No match)
823 - 832	1337.6573	1336.6500	1336.6451	0.0049	0	DLGYIYFYQR (No match)
823 - 832	1337.6573	1336.6500	1336.6451	0.0049	0	DLGYIYFYQR (Ions score 7)

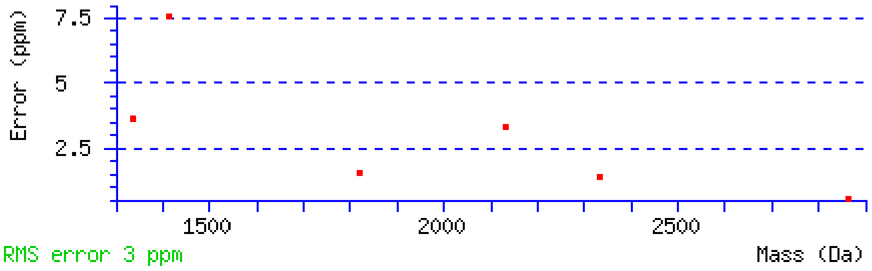

Mascot: http://www.matrixscience.com/

Spot 155

MATRIX SCIENCE Mascot Search Results

Protein View

Match to: **gi|3882215** Score: **84** Expect: **0.0008**
KIAA0747 protein [Homo sapiens]

Nominal mass (M_r): **120296**; Calculated pI value: **5.65**
NCBI BLAST search of gi|3882215 against nr
Unformatted sequence string for pasting into other applications

Taxonomy: Homo sapiens

Fixed modifications: Carbamidomethyl (C)
Variable modifications: Oxidation (M)
Cleavage by Trypsin: cuts C-term side of KR unless next residue is P
Sequence Coverage: **6%**

Matched peptides shown in **Bold Red**

1 AGPGAAGEAL AVLTSFGRRL LVLIPVYLAG AVGLSVGFVL FGLALYLGWR
51 RVRDEKERSL RAARQLLDDE EQLTAKTLYM SHRELPAWVS FPDVEKAEWL
101 NKIVAQVWPF LGQYMEKLLA ETVAPAVR**GS** **NPHLQTFTFT** **R**VELGEKPLR
151 IIGVKVHPGQ RKEQILLDLN ISYVGDVQID VEVKKYFCKA GVKGMQLHGV
201 LRVILEPLIG DLPFVGAVSM FFIRRPTLDI NWTGMTNLLD IPGLSSLSDT
251 MIMDSIAAFL VLPNR**LLVPL** **VPDLQDVAQL** **R**SPLPRGIIR IHLLAARGLS
301 SKDKYVKGLI EGKSDPYALV RLGTQTFCSR VIDEELNPQW GETYEVMVHE
351 VPGQEIEVEV FDKDPDKDDF LGRMKLDVGK VLQASVLDDW FPLQGGQGQV
401 HLRLEWLSLL SDAEKLEQVL QWNWGVSSRP DPPSAAILVV YLDRAQDLPM
451 VTSELYPPQL KKGNKEPNPM VQLSIQDVTQ ESK**AVYSTNC** **PVWEEAFR**FF
501 LQDPQSQELD VQVKDDSR**AL** **TLGALTLPLA** **R**LLTAPELIL DQWFQLSSSG
551 PNSRLYMKLV MRILYLDSSE ICFPTVPGCP GAWDVDSENP QRGSSVDAPP
601 RPCHTTPDSQ FGTEHVLRIH VLEAQDLIAK DRFLGGLVKG KSDPYVKLKL
651 AGRSFRSHVV REDLNPRWNE VFEVIVTSVP GQELEVEVFD KDLDKDDFLG
701 RCKVRLTTVL NSGFLDEWLT LEDVPSGRLH LRLERLTPRP TAAELEEVLQ
751 VNSLIQTQKS AELAAALLSI YMERAEDLPL RKGTKHLSPY ATLTVGDSSH
801 KTKTISQTSA PVWDESASFL IR**KPHTESLE** **LQVR**GEGTGV LGSLSLPLSE
851 LLVADQLCLD RWFTLSSGQG QVLLRAQLGI LVSQHSGVEA HSHSYSHSSS
901 SLSEEPELSG GPPHITSSAP ELRQRLTHVD SPLEAPAGPL GQVKLTLWYY
951 SEERKLVSIV HGCRSLRQNG RDPPDPYVSL LLLPDKNRGT KRRTSQKKRT
1001 LSPEFNERFE WELPLDEAQR RKLDVSVKSN SSFMSREREL LGKVQLDLAE
1051 TDLSQGVARW YDLMDNKDKG SS

Residue Number Increasing Mass Decreasing Mass

Start - End	Observed	Mr(expt)	Mr(calc)	Delta	Miss	Sequence
129 - 141	1505.7336	1504.7263	1504.7422	-0.0159	0	GSNPHLQTFTFTR (Ions score 12)
129 - 141	1505.7336	1504.7263	1504.7422	-0.0159	0	GSNPHLQTFTFTR (No match)
266 - 281	1789.0364	1788.0291	1788.0509	-0.0217	0	LLVPLVPDLQDVAQLR (Ions score 42)
266 - 281	1789.0364	1788.0291	1788.0509	-0.0217	0	LLVPLVPDLQDVAQLR (No match)
484 - 498	1828.8204	1827.8131	1827.8250	-0.0118	0	AVYSTNCPVWEEAFR (No match)
519 - 531	1309.8066	1308.7993	1308.8129	-0.0135	0	ALTLGALTLPLAR (Ions score 2)
519 - 531	1309.8066	1308.7993	1308.8129	-0.0135	0	ALTLGALTLPLAR (No match)
823 - 834	1436.7831	1435.7758	1435.7783	-0.0025	0	KPHTESLELQVR (No match)

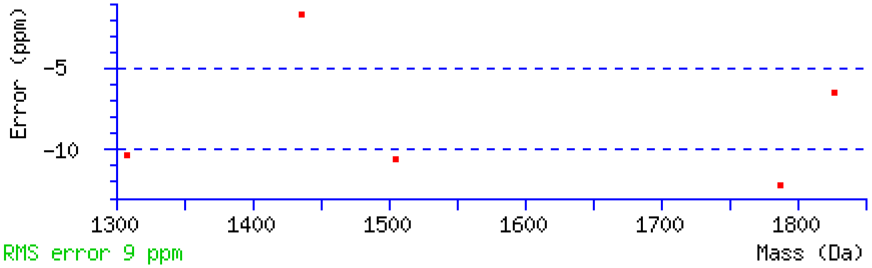

RMS error 9 ppm

Mascot: http://www.matrixscience.com/

Spot 156

MATRIX SCIENCE **Mascot Search Results**

Protein View

Match to: **gi|3882215** Score: **66** Expect: **0.051**
KIAA0747 protein [Homo sapiens]

Nominal mass (M_r): **120296**; Calculated pI value: **5.65**
NCBI BLAST search of gi|3882215 against nr
Unformatted sequence string for pasting into other applications

Taxonomy: Homo sapiens

Fixed modifications: Carbamidomethyl (C)
Variable modifications: Oxidation (M)
Cleavage by Trypsin: cuts C-term side of KR unless next residue is P
Sequence Coverage: **3%**

Matched peptides shown in **Bold Red**

1 AGPGAAGEAL AVLTSFGRRL LVLIPVYLAG AVGLSVGFVL FGLALYLGWR
51 RVRDEKERSL RAARQLLDDE EQLTAKTLYM SHRELPAWVS FPDVEKAEWL
101 NKIVAQVWPF LGQYMEKLLA ETVAPAVR**GS** **NPHLQTFTFT** **R**VELGEKPLR
151 IIGVKVHPGQ RKEQILLDLN ISYVGDVQID VEVKKYFCKA GVKGMQLHGV
201 LRVILEPLIG DLPFVGAVSM FFIRRPTLDI NWTGMTNLLD IPGLSSLSDT
251 MIMDSIAAFL VLPNR**LLVPL** **VPDLQDVAQL** **R**SPLPRGIIR IHLLAARGLS
301 SKDKYVKGLI EGKSDPYALV RLGTQTFCSR VIDEELNPQW GETYEVMVHE
351 VPGQEIEVEV FDKDPDKDDF LGRMKLDVGK VLQASVLDDW FPLQGGQGQV
401 HLRLEWLSLL SDAEKLEQVL QWNWGVSSRP DPPSAAILVV YLDRAQDLPM
451 VTSELYPPQL KKGNKEPNPM VQLSIQDVTQ ESKAVYSTNC PVWEEAFRFF
501 LQDPQSQELD VQVKDDSR**AL** **TLGALTLPLA** **R**LLTAPELIL DQWFQLSSSG
551 PNSRLYMKLV MRILYLDSSE ICFPTVPGCP GAWDVDSENP QRGSSVDAPP
601 RPCHTTPDSQ FGTEHVLRIH VLEAQDLIAK DRFLGGLVKG KSDPYVKLKL
651 AGRSFRSHVV REDLNPRWNE VFEVIVTSVP GQELEVEVFD KDLDKDDFLG
701 RCKVRLTTVL NSGFLDEWLT LEDVPSGRLH LRLERLTPRP TAAELEEVLQ
751 VNSLIQTQKS AELAAALLSI YMERAEDLPL RKGTKHLSPY ATLTVGDSSH
801 KTKTISQTSA PVWDESASFL IRKPHTESLE LQVRGEGTGV LGSLSLPLSE
851 LLVADQLCLD RWFTLSSGQG QVLLRAQLGI LVSQHSGVEA HSHSYSHSSS
901 SLSEEPELSG GPPHITSSAP ELRQRLTHVD SPLEAPAGPL GQVKLTLWYY
951 SEERKLVSIV HGCRSLRQNG RDPPDPYVSL LLLPDKNRGT KRRTSQKKRT
1001 LSPEFNERFE WELPLDEAQR RKLDVSVKSN SSFMSREREL LGKVQLDLAE
1051 TDLSQGVARW YDLMDNKDKG SS

Residue Number Increasing Mass Decreasing Mass

Start - End	Observed	Mr(expt)	Mr(calc)	Delta	Miss	Sequence
129 - 141	1505.7339	1504.7266	1504.7422	-0.0156	0	GSNPHLQTFTFTR (Ions score 43)
129 - 141	1505.7339	1504.7266	1504.7422	-0.0156	0	GSNPHLQTFTFTR (No match)
266 - 281	1789.0337	1788.0264	1788.0509	-0.0244	0	LLVPLVPDLQDVAQLR (No match)
519 - 531	1309.8038	1308.7965	1308.8129	-0.0163	0	ALTLGALTLPLAR (No match)

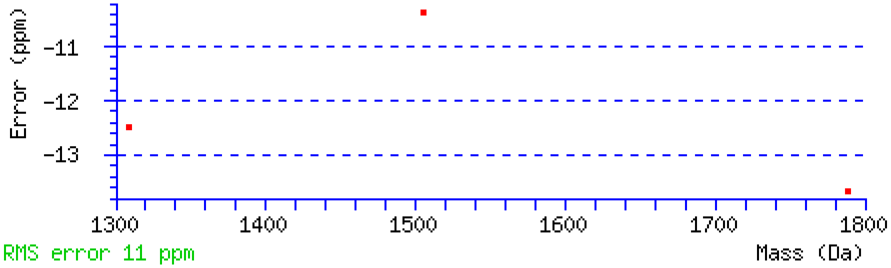

Mascot: http://www.matrixscience.com/

Spot 157

Mascot Search Results

Protein View

Match to: **gi|119625804** Score: **315** Expect: **6.1e-027**
moesin, isoform CRA_b [Homo sapiens]

Nominal mass (M_r): **66678;** Calculated pI value: **5.90**
NCBI BLAST search of gi|119625804 against nr
Unformatted sequence string for pasting into other applications

Taxonomy: Homo sapiens

Fixed modifications: Carbamidomethyl (C)
Variable modifications: Oxidation (M)
Cleavage by Trypsin: cuts C-term side of KR unless next residue is P
Sequence Coverage: **51%**

Matched peptides shown in **Bold Red**

```
  1 MDAELEFAIQ PNTTGKQLFD QVVKTIGLRE VWFFGLQYQD TKGFSTWLKL
 51 NKKVTAQDVR KESPLLFKFR AKFYPEDVSE ELIQDITQRL FFLQVKEGIL
101 NDDIYCPPET AVLLASYAVQ SKYGDFNKEV HKSGYLAGDK LLPQRVLEQH
151 KLNKDQWEER IQVWHEEHRG MLREDAVLEY LKIAQDLEMY GVNYFSIKNK
201 KGSELWLGVD ALGLNIYEQN DRLTPKIGFP WSEIRNISFN DKKFVIKPID
251 KKAPDFVFYA PRLRINKRIL ALCMGNHELY MRRRKPDTIE VQQMKAQARE
301 EKHQKQMERA MLENEKKKRE MAEKEKEKIE REKEELMERL KQIEEQTKKA
351 QQELEEQTRR ALELEQERKR AQSEAEKLAK ERQEAEEAKE ALLQASRDQK
401 KTQEQLALEM AELTARISQL EMARQKKESE AVEWQQKAQM VQEDLEKTRA
451 ELKTAMSTPH VAEPAENEQD EQDENGAEAS ADLRADAMAK DRSEEERTTE
501 AEKNERVQKH LKALTSELAN ARDESKKTAN DMIHAENMRL GRDKYKTLRQ
551 IRQGNTKQRI DEFESM
```

Residue Number Increasing Mass Decreasing Mass

Start - End	Observed	Mr(expt)	Mr(calc)	Delta	Miss	Sequence
17 - 24	976.5390	975.5317	975.5389	-0.0072	0	QLFDQVVK (No match)
54 - 60	788.4267	787.4194	787.4188	0.0006	0	VTAQDVR (No match)
62 - 70	1136.5747	1135.5674	1135.6389	-0.0715	1	ESPLLFKFR (No match)

71 - 89	2281.1235	2280.1162	2280.1273	-0.0111	1	AKFYPEDVSEELIQDITQR (No match)
73 - 89	2081.9871	2080.9798	2080.9953	-0.0154	0	FYPEDVSEELIQDITQR (No match)
90 - 96	894.5385	893.5312	893.5374	-0.0062	0	LFFLQVK (No match)
123 - 132	1236.5743	1235.5670	1235.5934	-0.0264	1	YGDFNKEVHK (No match)
133 - 145	1417.7773	1416.7700	1416.7724	-0.0024	1	SGYLAGDKLLPQR (No match)
146 - 154	1108.5763	1107.5690	1107.6400	-0.0710	1	VLEQHKLNK (No match)
146 - 154	1108.5763	1107.5690	1107.6400	-0.0710	1	VLEQHKLNK (No match)
161 - 169	1233.6023	1232.5950	1232.6050	-0.0100	0	IQVWHEEHR (No match)
170 - 182	1552.8010	1551.7937	1551.7966	-0.0029	1	GMLREDAVLEYLK Oxidation (M) (No match)
183 - 198	1906.9269	1905.9196	1905.9182	0.0015	0	IAQDLEMYGVNYFSIK Oxidation (M) (No match)
227 - 235	1104.5837	1103.5764	1103.5763	0.0001	0	IGFPWSEIR (No match)
252 - 262	1310.6869	1309.6796	1309.6818	-0.0022	1	KAPDFVFYAPR (No match)
252 - 262	1310.6869	1309.6796	1309.6818	-0.0022	1	KAPDFVFYAPR (Ions score 18)
253 - 262	1182.5917	1181.5844	1181.5869	-0.0025	0	APDFVFYAPR (Ions score 62)
253 - 262	1182.5917	1181.5844	1181.5869	-0.0025	0	APDFVFYAPR (No match)
269 - 282	1752.8247	1751.8174	1751.8157	0.0018	0	ILALCMGNHELYMR 2 Oxidation (M) (No match)
284 - 295	1488.7802	1487.7729	1487.7766	-0.0036	1	RKPDTIEVQQMK Oxidation (M) (No match)
332 - 339	1079.5024	1078.4951	1078.4964	-0.0013	1	EKEELMER Oxidation (M) (No match)
350 - 360	1387.6870	1386.6797	1386.6851	-0.0054	1	AQQELEEQTRR (No match)
361 - 368	987.5103	986.5030	986.5032	-0.0002	0	ALELEQER (No match)
383 - 397	1672.8472	1671.8399	1671.8427	-0.0028	1	QEAEEAKEALLQASR (No match)
401 - 416	1847.9521	1846.9448	1846.9458	-0.0010	1	KTQEQLALEMAELTAR Oxidation (M) (No match)
402 - 416	1719.8502	1718.8429	1718.8508	-0.0079	0	TQEQLALEMAELTAR Oxidation (M) (No match)
417 - 424	963.4893	962.4820	962.4855	-0.0034	0	ISQLEMAR Oxidation (M) (No match)
438 - 449	1463.7109	1462.7036	1462.7085	-0.0049	1	AQMVQEDLEKTR Oxidation (M) (No match)
498 - 506	1077.5138	1076.5065	1076.5098	-0.0032	1	TTEAEKNER (No match)
513 - 526	1504.7566	1503.7493	1503.7528	-0.0035	1	ALTSELANARDESK (No match)
527 - 539	1530.7073	1529.7000	1529.7079	-0.0078	1	KTANDMIHAENMR (No match)
558 - 566	1170.5018	1169.4945	1169.5022	-0.0077	1	QRIDEFESM Oxidation (M) (No match)

Mascot: http://www.matrixscience.com/

Spot 158

Mascot Search Results

Protein View

Match to: **gi|4503483** Score: **349** Expect: **2.4e-030**
eukaryotic translation elongation factor 2 [Homo sapiens]

Nominal mass (M_r): **96246**; Calculated pI value: **6.41**
NCBI BLAST search of gi|4503483 against nr
Unformatted sequence string for pasting into other applications

Taxonomy: Homo sapiens
Links to retrieve other entries containing this sequence from NCBI Entrez:
gi|119172 from Homo sapiens
gi|88909610 from Pongo pygmaeus
gi|31106 from Homo sapiens
gi|31108 from Homo sapiens
gi|60685056 from Homo sapiens
gi|116496673 from Homo sapiens
gi|119589680 from Homo sapiens
gi|119589681 from Homo sapiens

Fixed modifications: Carbamidomethyl (C)
Variable modifications: Oxidation (M)
Cleavage by Trypsin: cuts C-term side of KR unless next residue is P
Sequence Coverage: **29%**

Matched peptides shown in **Bold Red**

```
  1 MVNFTVDQIR AIMDKKANIR NMSVIAHVDH GKSTLTDSLV CKAGIIASAR
 51 AGETRFTDTR KDEQERCITI KSTAISLFYE LSENDLNFIK QSKDGAGFLI
101 NLIDSPGHVD FSSEVTAALR VTDGALVVVD CVSGVCVQTE TVLRQAIAER
151 IKPVLMMNKM DRALLELQLE PEELYQTFQR IVENVNVIIS TYGEGESGPM
201 GNIMIDPVLG TVGFGSGLHG WAFTLKQFAE MYVAKFAAKG EGQLGPAERA
251 KKVEDMMKKL WGDRYFDPAN GKFSKSATSP EGKKLPRTFC QLILDPIFKV
301 FDAIMNFKKE ETAKLIEKLD IKLDSEDKDK EGKPLLKAVM RRWLPAGDAL
351 LQMITIHLPS PVTAQKYRCE LLYEGPPDDE AAMGIKSCDP KGPLMMYISK
401 MVPTSDKGRF YAFGRVFSGL VSTGLKVRIM GPNYTPGKKE DLYLKPIQRT
451 ILMMGRYVEP IEDVPCGNIV GLVGVDQFLV KTGTITTFEH AHNMRVMKFS
501 VSPVVRVAVE AKNPADLPKL VEGLKRLAKS DPMVQCIIEE SGEHIIAGAG
551 ELHLEICLKD LEEDHACIPI KKSDPVVSYR ETVSEESNVL CLSKSPNKHN
601 RLYMKARPFP DGLAEDIDKG EVSARQELKQ RARYLAEKYE WDVAEARKIW
```

```
651 CFGPDGTGPN ILTDITKGVQ YLNEIKDSVV AGFQWATKEG ALCEENMRGV
701 RFDVHDVTLH ADAIHRGGGQ IIPTARRCLY ASVLTAQPRL MEPIYLVEIQ
751 CPEQVVGGIY GVLNRKRGHV FEESQVAGTP MFVVKAYLPV NESFGFTADL
801 RSNTGGQAFP QCVFDHWQIL PGDPFDNSSR PSQVVAETRK RKGLKEGIPA
851 LDNFLDKL
```

Residue Number Increasing Mass Decreasing Mass

Start - End	Observed	Mr(expt)	Mr(calc)	Delta	Miss	Sequence
2 - 10	1091.6116	1090.6043	1090.5771	0.0273	0	VNFTVDQIR (Ions score 15)
2 - 10	1091.6116	1090.6043	1090.5771	0.0273	0	VNFTVDQIR (No match)
61 - 66	804.4053	803.3980	803.3773	0.0207	1	KDEQER (No match)
163 - 180	2220.1924	2219.1851	2219.1473	0.0378	0	ALLELQLEPEELYQTFQR (No match)
240 - 249	1013.5313	1012.5240	1012.4937	0.0303	0	GEGQLGPAER (No match)
273 - 283	1138.5474	1137.5401	1137.5665	-0.0264	1	FSKSATSPEGK (No match)
288 - 299	1494.8385	1493.8312	1493.7952	0.0360	0	TFCQLILDPIFK (No match)
300 - 308	1084.5767	1083.5694	1083.5423	0.0272	0	VFDAIMNFK (No match)
369 - 386	2007.9445	2006.9372	2006.8965	0.0408	0	CELLYEGPPDDEAAMGIK (No match)
416 - 426	1107.6592	1106.6519	1106.6335	0.0184	0	VFSGLVSTGLK (No match)
439 - 449	1402.8390	1401.8317	1401.7979	0.0338	1	KEDLYLKPIQR (No match)
440 - 449	1274.7434	1273.7361	1273.7030	0.0331	0	EDLYLKPIQR (No match)
440 - 449	1274.7434	1273.7361	1273.7030	0.0331	0	EDLYLKPIQR (Ions score 17)
482 - 495	1615.8030	1614.7957	1614.7572	0.0385	0	TGTITTFEHAHNMR (No match)
482 - 495	1615.8030	1614.7957	1614.7572	0.0385	0	TGTITTFEHAHNMR (Ions score 16)
482 - 495	1631.7983	1630.7910	1630.7522	0.0389	0	TGTITTFEHAHNMR Oxidation (M) (No match)
499 - 506	890.5340	889.5267	889.5021	0.0246	0	FSVSPVVR (No match)
560 - 572	1567.8109	1566.8036	1566.7711	0.0325	1	DLEEDHACIPIKK (No match)
606 - 625	2143.1245	2142.1172	2142.0705	0.0468	1	ARPFPDGLAEDIDKGEVSAR (No match)
634 - 647	1742.8776	1741.8703	1741.8311	0.0393	1	YLAEKYEWDVAEAR (No match)
689 - 698	1208.5364	1207.5291	1207.4961	0.0330	0	EGALCEENMR (No match)
717 - 726	969.5756	968.5683	968.5403	0.0281	0	GGGQIIPTAR (No match)
727 - 739	1534.8517	1533.8444	1533.8085	0.0359	1	RCLYASVLTAQPR (No match)
728 - 739	1378.7480	1377.7407	1377.7074	0.0333	0	CLYASVLTAQPR (No match)
728 - 739	1378.7480	1377.7407	1377.7074	0.0333	0	CLYASVLTAQPR (Ions score 44)
768 - 785	1962.0237	1961.0164	1960.9716	0.0448	0	GHVFEESQVAGTPMFVVK (No match)
786 - 801	1799.9362	1798.9289	1798.8889	0.0400	0	AYLPVNESFGFTADLR (Ions score 63)
786 - 801	1799.9362	1798.9289	1798.8889	0.0400	0	AYLPVNESFGFTADLR (No match)

Mascot: http://www.matrixscience.com/

Spot 159

{MATRIX SCIENCE} Mascot Search Results

Protein View

Match to: **gi|48257098** Score: **207** Expect: **3.8e-016**
VCP protein [Homo sapiens]

Nominal mass (M_r): **71534;** Calculated pI value: **4.94**
NCBI BLAST search of gi|48257098 against nr
Unformatted sequence string for pasting into other applications

Taxonomy: Homo sapiens

Fixed modifications: Carbamidomethyl (C)
Variable modifications: Oxidation (M)
Cleavage by Trypsin: cuts C-term side of KR unless next residue is P
Sequence Coverage: **17%**

Matched peptides shown in **Bold Red**

1 FKVVETDPSP YCIVAPDTVI HCEGEPIKRE DEEESLNEVG YDDIGGCRKQ
51 LAQIK**EMVEL PLRHPALFK**A IGVKPPRGIL LYGPPGTGKT LIARAVANET
101 GAFFFLINGP EIMSKLAGES ESNLRKAFEE AEK**NAPAIIF IDELDAIAPK**
151 **R**EKTHGEVER RIVSQLLTLM DGLKQRAHVI VMAATNRPNS IDPALRRFGR
201 FDR**EVDIGIP DATGRLEILQ IHTK**NMKLAD DVDLEQVANE THGHVGADLA
251 ALCSEAALQA IRKKMDLIDL EDETIDAEVM NSLAVTMDDF RWALSQSNPS
301 ALRETVVEVP QVTWEDIGGL EDVKR**ELQEL VQYPVEHPDK FLK**FGMTPSK
351 **GVLFYGPPGC GK**TLLAKAIA NECQANFISI KGPELLTMWF GESEANVREI
401 FDKARQAAPC VLFFDELDSI AKARGGNIGD GGGAADRVIN QILTEMDGMS
451 TKKNVFIIGA TNRPDIIDPA ILRPGRLDQL IYIPLPDEKS RVAILKANLR
501 KSPVAKDVDL EFLAK**MTNGF SGADLTEICQ R**ACKLAIRES IESEIRRERE
551 RQTNPSAMEV EEDDPVPEIR RDHFEEAMRF ARRSVSDNDI R**KYEMFAQTL**
601 **QQSR**GFGSFR FPSGNQGGAG PSQGSGGGTG GSVYTEDNDD DLYG

Residue Number Increasing Mass Decreasing Mass

Start - End	Observed	Mr(expt)	Mr(calc)	Delta	Miss	Sequence
56 - 69	1695.9312	1694.9239	1694.9177	0.0062	1	**EMVELPLRHPALFK** Oxidation (M) (No match)
134 - 151	1967.1035	1966.0962	1966.0887	0.0075	1	**NAPAIIFIDELDAIAPKR** (Ions score 48)

134 - 151	1967.1035	1966.0962	1966.0887	0.0075	1	NAPAIIFIDELDAIAPKR (No match)
204 - 224	2318.2788	2317.2715	2317.2641	0.0074	1	EVDIGIPDATGRLEILQIHTK (Ions score 4)
204 - 224	2318.2788	2317.2715	2317.2641	0.0074	1	EVDIGIPDATGRLEILQIHTK (No match)
326 - 343	2212.1711	2211.1638	2211.1575	0.0063	1	ELQELVQYPVEHPDKFLK (No match)
326 - 343	2212.1711	2211.1638	2211.1575	0.0063	1	ELQELVQYPVEHPDKFLK (Ions score 49)
351 - 362	1251.6243	1250.6170	1250.6117	0.0053	0	GVLFYGPPGCGK (No match)
516 - 531	1815.8085	1814.8012	1814.7927	0.0085	0	MTNGFSGADLTEICQR Oxidation (M) (No match)
592 - 604	1645.8110	1644.8037	1644.7929	0.0108	1	KYEMFAQTLQQSR Oxidation (M) (Ions score 26)
592 - 604	1645.8110	1644.8037	1644.7929	0.0108	1	KYEMFAQTLQQSR Oxidation (M) (No match)

Mascot: http://www.matrixscience.com/

Spot 162

{MATRIX SCIENCE} Mascot Search Results

Protein View

Match to: **gi|119625804** Score: **646** Expect: **4.8e-060**
moesin, isoform CRA_b [Homo sapiens]

Nominal mass (M_r): **66678**; Calculated pI value: **5.90**
NCBI BLAST search of gi|119625804 against nr
Unformatted sequence string for pasting into other applications

Taxonomy: Homo sapiens

Fixed modifications: Carbamidomethyl (C)
Variable modifications: Oxidation (M)
Cleavage by Trypsin: cuts C-term side of KR unless next residue is P
Sequence Coverage: **48%**

Matched peptides shown in **Bold Red**

```
  1 MDAELEFAIQ PNTTGKQLFD QVVKTIGLRE VWFFGLQYQD TKGFSTWLKL
 51 NKKVTAQDVR KESPLLFKFR AKFYPEDVSE ELIQDITQRL FFLQVKEGIL
101 NDDIYCPPET AVLLASYAVQ SKYGDFNKEV HKSGYLAGDK LLPQRVLEQH
151 KLNKDQWEER IQVWHEEHRG MLREDAVLEY LKIAQDLEMY GVNYFSIKNK
201 KGSELWLGVD ALGLNIYEQN DRLTPKIGFP WSEIRNISFN DKKFVIKPID
251 KKAPDFVFYA PRLRINKRIL ALCMGNHELY MRRRKPDTIE VQQMKAQARE
301 EKHQKQMERA MLENEKKKRE MAEKEKEKIE REKEELMERL KQIEEQTKKA
351 QQELEEQTRR ALELEQERKR AQSEAEKLAK ERQEAEEAKE ALLQASRDQK
401 KTQEQLALEM AELTARISQL EMARQKKESE AVEWQQKAQM VQEDLEKTRA
451 ELKTAMSTPH VAEPAENEQD EQDENGAEAS ADLRADAMAK DRSEEERTTE
501 AEKNERVQKH LKALTSELAN ARDESKKTAN DMIHAENMRL GRDKYKTLRQ
551 IRQGNTKQRI DEFESM
```

Residue Number Increasing Mass Decreasing Mass

Start - End	Observed	Mr(expt)	Mr(calc)	Delta	Miss	Sequence
17 - 24	976.5388	975.5315	975.5389	-0.0074	0	QLFDQVVK (No match)
30 - 42	1660.7955	1659.7882	1659.7932	-0.0050	0	EVWFFGLQYQDTK (No match)
54 - 60	788.4311	787.4238	787.4188	0.0050	0	VTAQDVR (No match)

62 - 70	1136.5751	1135.5678	1135.6389	-0.0711	1	ESPLLFKFR	(No match)
73 - 89	2081.9905	2080.9832	2080.9953	-0.0120	0	FYPEDVSEELIQDITQR	(Ions score 131)
73 - 89	2081.9905	2080.9832	2080.9953	-0.0120	0	FYPEDVSEELIQDITQR	(No match)
90 - 96	894.5352	893.5279	893.5374	-0.0095	0	LFFLQVK	(No match)
146 - 154	1108.5822	1107.5749	1107.6400	-0.0651	1	VLEQHKLNK	(No match)
152 - 160	1217.5933	1216.5860	1216.5836	0.0024	1	LNKDQWEER	(No match)
155 - 160	862.3723	861.3650	861.3617	0.0034	0	DQWEER	(No match)
161 - 169	1233.6057	1232.5984	1232.6050	-0.0066	0	IQVWHEEHR	(No match)
161 - 169	1233.6057	1232.5984	1232.6050	-0.0066	0	IQVWHEEHR	(Ions score 47)
170 - 182	1536.8018	1535.7945	1535.8017	-0.0072	1	GMLREDAVLEYLK	(No match)
170 - 182	1552.8014	1551.7941	1551.7966	-0.0025	1	GMLREDAVLEYLK Oxidation (M)	(No match)
183 - 198	1890.9408	1889.9335	1889.9233	0.0103	0	IAQDLEMYGVNYFSIK	(No match)
183 - 198	1906.9547	1905.9474	1905.9182	0.0293	0	IAQDLEMYGVNYFSIK Oxidation (M)	(No match)
202 - 222	2362.1501	2361.1428	2361.1600	-0.0172	0	GSELWLGVDALGLNIYEQNDR	(No match)
227 - 235	1104.5822	1103.5749	1103.5763	-0.0014	0	IGFPWSEIR	(Ions score 41)
227 - 235	1104.5822	1103.5749	1103.5763	-0.0014	0	IGFPWSEIR	(No match)
252 - 262	1310.6862	1309.6789	1309.6818	-0.0029	1	KAPDFVFYAPR	(Ions score 68)
252 - 262	1310.6862	1309.6789	1309.6818	-0.0029	1	KAPDFVFYAPR	(No match)
253 - 262	1182.5920	1181.5847	1181.5869	-0.0022	0	APDFVFYAPR	(Ions score 71)
253 - 262	1182.5920	1181.5847	1181.5869	-0.0022	0	APDFVFYAPR	(No match)
269 - 282	1720.8431	1719.8358	1719.8259	0.0100	0	ILALCMGNHELYMR	(No match)
269 - 282	1736.8336	1735.8263	1735.8208	0.0056	0	ILALCMGNHELYMR Oxidation (M)	(No match)
269 - 282	1752.8323	1751.8250	1751.8157	0.0094	0	ILALCMGNHELYMR 2 Oxidation (M)	(No match)
284 - 295	1472.7800	1471.7727	1471.7817	-0.0089	1	RKPDTIEVQQMK	(No match)
284 - 295	1488.7776	1487.7703	1487.7766	-0.0062	1	RKPDTIEVQQMK Oxidation (M)	(No match)
332 - 339	1063.5117	1062.5044	1062.5015	0.0029	1	EKEELMER	(No match)
332 - 339	1079.5106	1078.5033	1078.4964	0.0069	1	EKEELMER Oxidation (M)	(No match)
350 - 359	1231.5908	1230.5835	1230.5840	-0.0005	0	AQQELEEQTR	(No match)
350 - 360	1387.6866	1386.6793	1386.6851	-0.0058	1	AQQELEEQTRR	(No match)
361 - 368	987.5112	986.5039	986.5032	0.0007	0	ALELEQER	(No match)
381 - 389	1089.5186	1088.5113	1088.5097	0.0016	1	ERQEAEEAK	(No match)
401 - 416	1831.9446	1830.9373	1830.9509	-0.0136	1	KTQEQLALEMAELTAR	(No match)
401 - 416	1847.9575	1846.9502	1846.9458	0.0044	1	KTQEQLALEMAELTAR Oxidation (M)	(No match)
402 - 416	1703.8518	1702.8445	1702.8559	-0.0114	0	TQEQLALEMAELTAR	(No match)
402 - 416	1719.8542	1718.8469	1718.8508	-0.0039	0	TQEQLALEMAELTAR Oxidation (M)	(No match)
417 - 424	947.4979	946.4906	946.4906	0.0001	0	ISQLEMAR	(No match)
417 - 424	963.4979	962.4906	962.4855	0.0052	0	ISQLEMAR Oxidation (M)	(No match)
491 - 497	920.4109	919.4036	919.3995	0.0041	1	DRSEEER	(No match)
498 - 506	1077.5186	1076.5113	1076.5098	0.0016	1	TTEAEKNER	(No match)
513 - 522	1045.5596	1044.5523	1044.5563	-0.0040	0	ALTSELANAR	(No match)
558 - 566	1154.5087	1153.5014	1153.5073	-0.0059	1	QRIDEFESM	(No match)
558 - 566	1170.5088	1169.5015	1169.5022	-0.0007	1	QRIDEFESM Oxidation (M)	(No match)

**Mascot:** http://www.matrixscience.com/

Spot 163

{MATRIX SCIENCE} Mascot Search Results

Protein View

Match to: **gi|119625804** Score: **546** Expect: **4.8e-050**
moesin, isoform CRA_b [Homo sapiens]

Nominal mass (M_r): **66678;** Calculated pI value: **5.90**
NCBI BLAST search of gi|119625804 against nr
Unformatted sequence string for pasting into other applications

Taxonomy: Homo sapiens

Fixed modifications: Carbamidomethyl (C)
Variable modifications: Oxidation (M)
Cleavage by Trypsin: cuts C-term side of KR unless next residue is P
Sequence Coverage: **42%**

Matched peptides shown in **Bold Red**

1 MDAELEFAIQ PNTTGK**QLFD QVVK**TIGLR**E VWFFGLQYQD TK**GFSTWLKL
51 NKKVTAQDVR K**ESPLLFKFR** AK**FYPEDVSE ELIQDITQRL FFLQVK**EGIL
101 NDDIYCPPET AVLLASYAVQ SKYGDFNKEV HKSGYLAGDK LLPQR**VLEQH**
151 **KLNKDQWEER IQVWHEEHRG MLREDAVLEY LK**IAQDLEMY GVNYFSIKNK
201 K**GSELWLGVD ALGLNIYEQN DR**LTPK**IGFP WSEIR**NISFN DKKFVIKPID
251 K**KAPDFVFYA PR**LRINKR**IL ALCMGNHELY MR**R**RKPDTIE VQQMK**AQARE
301 EKHQKQMERA MLENEKKKRE MAEKEKEKIE R**EKEELMER**L KQIEEQTKK**A**
351 **QQELEEQTRR ALELEQER**KR AQSEAEKLAK **ERQEAEEAK**E ALLQASRDQK
401 **KTQEQLALEM AELTAR**ISQL EMARQKKESE AVEWQQKAQM VQEDLEKTRA
451 ELKTAMSTPH VAEPAENEQD EQDENGAEAS ADLRADAMAK DRSEEER**TTE**
501 **AEKNER**VQKH LK**ALTSELAN AR**DESKKTAN DMIHAENMRL GRDKYKTLRQ
551 IRQGNTK**QRI DEFESM**

Residue Number Increasing Mass Decreasing Mass

Start - End	Observed	Mr(expt)	Mr(calc)	Delta	Miss	Sequence
17 - 24	976.5394	975.5321	975.5389	-0.0068	0	QLFDQVVK (No match)
30 - 42	1660.7970	1659.7897	1659.7932	-0.0035	0	EVWFFGLQYQDTK (No match)
62 - 70	1136.5686	1135.5613	1135.6389	-0.0776	1	ESPLLFKFR (No match)

73 - 89	2081.9863	2080.9790	2080.9953	-0.0162	0	FYPEDVSEELIQDITQR (No match)
73 - 89	2081.9863	2080.9790	2080.9953	-0.0162	0	FYPEDVSEELIQDITQR (Ions score 125)
90 - 96	894.5378	893.5305	893.5374	-0.0069	0	LFFLQVK (No match)
146 - 154	1108.5729	1107.5656	1107.6400	-0.0744	1	VLEQHKLNK (No match)
155 - 160	862.3727	861.3654	861.3617	0.0038	0	DQWEER (No match)
161 - 169	1233.6007	1232.5934	1232.6050	-0.0116	0	IQVWHEEHR (Ions score 25)
161 - 169	1233.6007	1232.5934	1232.6050	-0.0116	0	IQVWHEEHR (No match)
170 - 182	1536.8022	1535.7949	1535.8017	-0.0068	1	GMLREDAVLEYLK (No match)
170 - 182	1552.7948	1551.7875	1551.7966	-0.0091	1	GMLREDAVLEYLK Oxidation (M) (No match)
202 - 222	2362.1421	2361.1348	2361.1600	-0.0252	0	GSELWLGVDALGLNIYEQNDR (No match)
227 - 235	1104.5778	1103.5705	1103.5763	-0.0058	0	IGFPWSEIR (Ions score 43)
227 - 235	1104.5778	1103.5705	1103.5763	-0.0058	0	IGFPWSEIR (No match)
252 - 262	1310.6807	1309.6734	1309.6818	-0.0084	1	KAPDFVFYAPR (No match)
252 - 262	1310.6807	1309.6734	1309.6818	-0.0084	1	KAPDFVFYAPR (Ions score 56)
253 - 262	1182.5881	1181.5808	1181.5869	-0.0061	0	APDFVFYAPR (Ions score 68)
253 - 262	1182.5881	1181.5808	1181.5869	-0.0061	0	APDFVFYAPR (No match)
269 - 282	1736.8251	1735.8178	1735.8208	-0.0029	0	ILALCMGNHELYMR Oxidation (M) (No match)
284 - 295	1488.7772	1487.7699	1487.7766	-0.0066	1	RKPDTIEVQQMK Oxidation (M) (No match)
332 - 339	1063.5092	1062.5019	1062.5015	0.0004	1	EKEELMER (No match)
332 - 339	1079.5056	1078.4983	1078.4964	0.0019	1	EKEELMER Oxidation (M) (No match)
350 - 359	1231.5813	1230.5740	1230.5840	-0.0100	0	AQQELEEQTR (No match)
350 - 360	1387.6852	1386.6779	1386.6851	-0.0072	1	AQQELEEQTRR (No match)
361 - 368	987.5089	986.5016	986.5032	-0.0016	0	ALELEQER (No match)
381 - 389	1089.5176	1088.5103	1088.5097	0.0006	1	ERQEAEEAK (No match)
401 - 416	1831.9431	1830.9358	1830.9509	-0.0151	1	KTQEQLALEMAELTAR (No match)
401 - 416	1847.9491	1846.9418	1846.9458	-0.0040	1	KTQEQLALEMAELTAR Oxidation (M) (No match)
402 - 416	1703.8468	1702.8395	1702.8559	-0.0164	0	TQEQLALEMAELTAR (No match)
402 - 416	1719.8490	1718.8417	1718.8508	-0.0091	0	TQEQLALEMAELTAR Oxidation (M) (No match)
498 - 506	1077.5187	1076.5114	1076.5098	0.0017	1	TTEAEKNER (No match)
513 - 522	1045.5586	1044.5513	1044.5563	-0.0050	0	ALTSELANAR (No match)
558 - 566	1170.5048	1169.4975	1169.5022	-0.0047	1	QRIDEFESM Oxidation (M) (No match)

Mascot: http://www.matrixscience.com/

Spot 164

{MATRIX SCIENCE} Mascot Search Results

Protein View

Match to: **gi|119615215** Score: **148** Expect: **3e-010**
karyopherin (importin) beta 1, isoform CRA_b [Homo sapiens]

Nominal mass (M_r): **95094**; Calculated pI value: **4.66**
NCBI BLAST search of gi|119615215 against nr
Unformatted sequence string for pasting into other applications

Taxonomy: Homo sapiens

Fixed modifications: Carbamidomethyl (C)
Variable modifications: Oxidation (M)
Cleavage by Trypsin: cuts C-term side of KR unless next residue is P
Sequence Coverage: **20%**

Matched peptides shown in **Bold Red**

```
  1 MELITILEKT VSPDRLELEA AQKFLERAAV ENLPTFLVEL SRVLANPGNS
 51 QVARVAAGLQ IKNSLTSKDP DIKAQYQQRW LAIDANARRE VKNYVLQTLG
101 TETYRPSSAS QCVAGIACAE IPVNQWPELI PQLVANVTNP NSTEHMKEST
151 LEAIGYICQD IDPEQLQDKS NEILTAIIQG MRKEEPSNNV KLAATNALLN
201 SLEFTKANFD KESERHFIMQ VVCEATQCPD TRVRVAALQN LVKIMSLYYQ
251 YMETYMGPAL FAITIEAMKS DIDEVALQGI EFWSNVCDEE MDLAIEASEA
301 AEQGRPPEHT SKFYAKGALQ YLVPILTQTL TKQDENDDDD DWNPCKAAGV
351 CLMLLATCCE DDIVPHVLPF IKEHIKNPDW RYRDAAVMAF GCILEGPEPS
401 QLKPLVIQAM PTLIELMKDP SVVVRDTAAW TVGRICELLP EAAINDVYLA
451 PLLQCLIEGL SAEPRVASNV CWAFSSLAEA AYEAADVADD QEEPATYCLS
501 SSFELIVQKL LETTDRPDGH QNNLRSSAYE SLMEIVKNSA KDCYPAVQKT
551 TLVIMERLQQ VLQMESHIQS TSDRIQFNDL QSLLCATLQN VLRKVQHQDA
601 LQISDVVMAS LLRMFQSTAG SGGVQEDALM AVSTLVEVLG GEFLKYMEAF
651 KPFLGIGLKN YAEYQVCLAA VGLVGDLCRA LQSNIIPFCD EVMQLLLENL
701 GVKVVLNTLQ QASQAQVDKS DYDMVDYLNE LRESCLEAYT GIVQGLKGDQ
751 ENVHPDVMLV QPRVEFILSF IDHIAGDEDH TDGVVACAAG LIGDLCTAFG
801 KDVLKLVEAR PMIHELLTEG RRSKTNKAKT LATWATKELR KLKNQA
```

Residue Number Increasing Mass Decreasing Mass

Start - End	Observed	Mr(expt)	Mr(calc)	Delta	Miss	Sequence
10 - 23	1556.8900	1555.8827	1555.8205	0.0622	1	TVSPDRLELEAAQK (No match)
10 - 23	1556.8900	1555.8827	1555.8205	0.0622	1	TVSPDRLELEAAQK (No match)
28 - 42	1658.9778	1657.9705	1657.9038	0.0667	0	AAVENLPTFLVELSR (No match)
28 - 42	1658.9778	1657.9705	1657.9038	0.0667	0	AAVENLPTFLVELSR (Ions score 46)
43 - 54	1225.7150	1224.7077	1224.6574	0.0503	0	VLANPGNSQVAR (Ions score 8)
43 - 54	1225.7150	1224.7077	1224.6574	0.0503	0	VLANPGNSQVAR (No match)
170 - 182	1461.8296	1460.8223	1460.7657	0.0567	0	SNEILTAIIQGMR Oxidation (M) (No match)
192 - 206	1605.9497	1604.9424	1604.8773	0.0651	0	LAATNALLNSLEFTK (No match)
313 - 332	2268.3186	2267.3113	2267.2929	0.0185	1	FYAKGALQYLVPILTQTLTK (No match)
510 - 525	1879.0182	1878.0109	1877.9343	0.0766	0	LLETTDRPDGHQNNLR (Ions score 2)
510 - 525	1879.0182	1878.0109	1877.9343	0.0766	0	LLETTDRPDGHQNNLR (No match)
558 - 574	2016.0712	2015.0639	2014.9742	0.0898	0	LQQVLQMESHIQSTSDR Oxidation (M) (No match)
595 - 613	2139.2041	2138.1968	2138.1153	0.0815	0	VQHQDALQISDVVMASLLR Oxidation (M) (No match)
646 - 659	1629.9409	1628.9336	1628.8635	0.0701	0	YMEAFKPFLGIGLK Oxidation (M) (No match)
748 - 763	1849.9685	1848.9612	1848.8788	0.0824	0	GDQENVHPDVMLVQPR Oxidation (M) (No match)

Mascot: http://www.matrixscience.com/

Spot 165

MATRIX SCIENCE Mascot Search Results

Protein View

Match to: **gi|2134674** Score: **121** Expect: **1.5e-007**
55.11 protein homolog - human (fragment)

Nominal mass (M_r): **99994;** Calculated pI value: **5.14**
NCBI BLAST search of gi|2134674 against nr
Unformatted sequence string for pasting into other applications

Taxonomy: Homo sapiens
Links to retrieve other entries containing this sequence from NCBI Entrez:
gi|1008089 from Homo sapiens

Fixed modifications: Carbamidomethyl (C)
Variable modifications: Oxidation (M)
Cleavage by Trypsin: cuts C-term side of KR unless next residue is P
Sequence Coverage: **6%**

Matched peptides shown in **Bold Red**

```
  1 RVQPQQSPAA APGGTDEKPS GKERRDAGDK DKEQELSEED KQLQDELEML
 51 VERLGEKDTS LYRPALEELR RQIRSSTTSM TSVPKPLKFL RPHYGKLKEI
101 YENMAPGENK RFAADIISVL AMTMSGEREC LKYRLVGSQE ELASWGHEYV
151 RHLAGEVAKE WQELDDAEKV QREPLLTLVK EIVPYNMAHN AEHEACDLLM
201 EIEQVDMLEK DIDENAYAKV CLYLTSCVNY VPEPENSALL RCALGVFRKF
251 TRFPEALRLA LMLNDMELVE DIFTSCKDVV VQKQMAFMLG RHGVFLELSE
301 DVEEYEDLTE IMSNVQLNSN FLALARELDI MEPKVPDDIY KTHLENNRFG
351 GSGSQVDSAR MNLASSFVNG FVNAAFGQDK LLTDDGNKWL YKNKDHGMLS
401 AAASLGMILL WDVDGGLTQI DKYLYSSEDY IKSGALLACG IVNSGVRNEC
451 DPALALLSDY VLHNSNTMRL GSIFGLGLAY AGSNREDVLT LLLPVMGDSK
501 SSMEVAGVTA LACGMIAVGS CNGDVTSTIL QTIMEKSETE LKDTYARWLP
551 LGLGLNHLGK GEAIEAILAA LEVVSEPFRS FANTLVDVCA YAGSGNVLKV
601 QQLLHICSEH FDSKEKEEDK DKKEKKDKDK KEAPADMGAH QGVAVLGIAL
651 IAMGEEIGAE MALRTFGHLL RYGEPTLRRA VPLALALISV SNPRLNILDT
701 LSKFSHDADP EVSYNSIFAM GMVGSGTNNA RLAAMLRQLA QYHAKDPNNL
751 FMVRLAQGLT HLGKGTLTLC PYHSDRQLMS QVAVAGLLTV LVSFLDVRNI
801 ILGKSHYVLY GLVAAMQPRM LVTFDEELRP LPVSVRVGQA VDVVGQAGKP
851 KTITGFQTHT TPVLLAHGER AELATEEFLP VTPILEGFVI FGRTPIMISK
901
```

Residue Number Increasing Mass Decreasing Mass

Start - End	Observed	Mr(expt)	Mr(calc)	Delta	Miss	Sequence
89 - 96	1017.6290	1016.6217	1016.5555	0.0662	0	FLRPHYGK (No match)
89 - 96	1017.6290	1016.6217	1016.5555	0.0662	0	FLRPHYGK (No match)
680 - 694	1520.9368	1519.9295	1519.9085	0.0210	0	AVPLALALISVSNPR (Ions score 53)
680 - 694	1520.9368	1519.9295	1519.9085	0.0210	0	AVPLALALISVSNPR (No match)
805 - 819	1720.9171	1719.9098	1719.8766	0.0332	0	SHYVLYGLVAAMQPR Oxidation (M) (No match)
805 - 819	1720.9171	1719.9098	1719.8766	0.0332	0	SHYVLYGLVAAMQPR Oxidation (M) (No match)
852 - 870	2079.1189	2078.1116	2078.0908	0.0208	0	TITGFQTHTTPVLLAHGER (No match)
852 - 870	2079.1189	2078.1116	2078.0908	0.0208	0	TITGFQTHTTPVLLAHGER (Ions score 33)

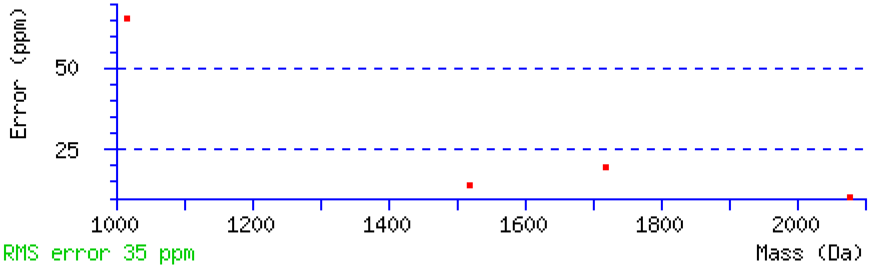

RMS error 35 ppm

Mascot: http://www.matrixscience.com/

Spot 167

{MATRIX}{SCIENCE} Mascot Search Results

Protein View

Match to: **gi|51100948** Score: **282** Expect: **1.2e-023**
MHC class I antigen [Homo sapiens]

Nominal mass (M_r): **31659**; Calculated pI value: **5.68**
NCBI BLAST search of gi|51100948 against nr
Unformatted sequence string for pasting into other applications

Taxonomy: Homo sapiens

Fixed modifications: Carbamidomethyl (C)
Variable modifications: Oxidation (M)
Cleavage by Trypsin: cuts C-term side of KR unless next residue is P
Sequence Coverage: **60%**

Matched peptides shown in **Bold Red**

```
  1 SHSMRYFSTS VSRPGRGEPR FIAVGYVDDT QFVRFDSDAA SQRMEPRAPW
 51 IEQEGPEYWD EETGKVKAHS QTDRENLRIA LRYYNQSEAG SHTLQMMFGC
101 DVGSDGRFLR GYHQYAYDGK DYIALKEDLR SWTAADMAAQ ITKRKWEAAH
151 VAEQQRAYLE GTCVDGLRRY LENGKETLQR TDPPKTHMTH HPISDHEATL
201 RCWALGFYPA EITLTWQRDG EDQTQDTELV ETRPAGDGTF QKWAAVVVPS
251 GEEQRYTCHV QHEGLPKPLT LRW
```

Residue Number Increasing Mass Decreasing Mass

Start - End	Observed	Mr(expt)	Mr(calc)	Delta	Miss	Sequence
6 - 16	1256.6957	1255.6884	1255.6309	0.0575	0	YFSTSVSRPGR (No match)
6 - 20	1695.9343	1694.9270	1694.8488	0.0782	1	YFSTSVSRPGRGEPR (No match)
6 - 20	1695.9343	1694.9270	1694.8488	0.0782	1	YFSTSVSRPGRGEPR (Ions score 14)
21 - 34	1629.9015	1628.8942	1628.8198	0.0744	0	FIAVGYVDDTQFVR (No match)
21 - 34	1629.9015	1628.8942	1628.8198	0.0744	0	FIAVGYVDDTQFVR (Ions score 44)
35 - 43	996.4835	995.4762	995.4308	0.0454	0	FDSDAASQR (No match)
68 - 78	1326.7079	1325.7006	1325.6436	0.0570	1	AHSQTDRENLR (No match)
111 - 126	1905.0076	1904.0003	1903.9104	0.0899	1	GYHQYAYDGKDYIALK (No match)
157 - 169	1509.8175	1508.8102	1508.7405	0.0697	1	AYLEGTCVDGLRR (No match)

170 - 180	1350.7625	1349.7552	1349.6939	0.0614	1	YLENGKETLQR (No match)
170 - 180	1350.7625	1349.7552	1349.6939	0.0614	1	YLENGKETLQR (Ions score 18)
181 - 201	2437.2786	2436.2713	2436.1604	0.1109	1	TDPPKTHMTHHPISDHEATLR Oxidation (M) (No match)
186 - 201	1898.9946	1897.9873	1897.8853	0.1020	0	THMTHHPISDHEATLR Oxidation (M) (No match)
219 - 242	2637.3030	2636.2957	2636.1837	0.1120	0	DGEDQTQDTELVETRPAGDGTFQK (Ions score 8)
219 - 242	2637.3030	2636.2957	2636.1837	0.1120	0	DGEDQTQDTELVETRPAGDGTFQK (No match)
243 - 255	1427.7917	1426.7844	1426.7204	0.0640	0	WAAVVVPSGEEQR (No match)
256 - 272	2049.1606	2048.1533	2048.0625	0.0908	0	YTCHVQHEGLPKPLTLR (No match)
256 - 272	2049.1606	2048.1533	2048.0625	0.0908	0	YTCHVQHEGLPKPLTLR (Ions score 35)

Mascot: http://www.matrixscience.com/

Spot 171

Mascot Search Results

Protein View

Match to: **gi|46249756** Score: **290** Expect: **1.9e-024**
PDCD6IP protein [Homo sapiens]

Nominal mass (M_r): **97385**; Calculated pI value: **6.13**
NCBI BLAST search of gi|46249756 against nr
Unformatted sequence string for pasting into other applications

Taxonomy: Homo sapiens

Fixed modifications: Carbamidomethyl (C)
Variable modifications: Oxidation (M)
Cleavage by Trypsin: cuts C-term side of KR unless next residue is P
Sequence Coverage: **32%**

Matched peptides shown in **Bold Red**

```
  1 MATFISMQLK KTSEVDLAKP LVKFIQQTYP SGGEEQAQYC RAAEELSKLR
 51 RAAVGRPLDK HEGALETLLR YYDQICSIEP KFPFSENQIC LTFTWKDAFD
101 KGSLFGGSVK LALASLGYEK SCVLFNCAAL ASQIAAEQNL DNDEGLKIAA
151 KHYQFASGAF LHIKETVLSA LSREPTVDIS PDTVGTLSLI MLAQAQEVFF
201 LKATRDKMKD AIIAKLANQA ADYFGDAFKQ CQYKDTLPKY FYFQEVFPVL
251 AAKHCIMQAN AEYHQSILAK QQKKFGEEIA RLQHAAELIK TVASRYDEYV
301 NVKDFSDKIN RALAAAKKDN DFIYHDRVPD LKDLDPIGKA TLVKSTPVNV
351 PISQKFTDLF EKMVPVSVQQ SLAAYNQRKA DLINRSIAQM REATTLANGV
401 LASLNLPAAI EDVSGDTVPQ SILTKSRSVI EQGGIQTVDQ LIKELPELLQ
451 RNREILDESL RLLDEEEATD NDLRAKFKER WQRTPSNELY KPLRAEGTNF
501 RTVLDKAVQA DGQVKECYQS HRDTIVLLCK PEPELNAAIP SANPAKTMQG
551 SEVVNVLKSL LSNLDEVKKE REGLENDLKS VNFDMTSKFL TALAQDGVIN
601 EEALSVTELD RVYGGLTTKV QESLKKQEGL LKNIQVSHQE FSKMKQSNNE
651 ANLREEVLKN LATAYDNFVE LVANLKEGTK FYNELTEILV RFQNKCSDIV
701 FARKTERDEL LKDLQQSIAR EPSAPSIPTP AYQSSPAGGH APTPPTPAPR
751 TMPPTKPQPP ARPPPPVLPA NRAPSATAPS PVGAGTAAPA PSQTPGSAPP
801 PQAQGPPYPT YPGYPGYCQM PMPMGYNPYA YGQYNMPYPP VYHQSPGQAP
851 YPGPQQPSYP FPQPPQQSYY PQQ
```

Residue Number Increasing Mass Decreasing Mass

Start - End	Observed	Mr(expt)	Mr(calc)	Delta	Miss	Sequence
2 - 11	1182.6440	1181.6367	1181.6478	-0.0111	1	ATFISMQLKK Oxidation (M) (No match)
11 - 23	1427.7816	1426.7743	1426.8395	-0.0651	1	KTSEVDLAKPLVK (No match)
24 - 41	2162.0527	2161.0454	2160.9534	0.0920	0	FIQQTYPSGGEEQAQYCR (No match)
24 - 41	2162.0527	2161.0454	2160.9534	0.0920	0	FIQQTYPSGGEEQAQYCR (Ions score 18)
52 - 70	2046.2343	2045.2270	2045.1381	0.0889	1	AAVGRPLDKHEGALETLLR (No match)
152 - 164	1518.8501	1517.8428	1517.7779	0.0650	0	HYQFASGAFLHIK (No match)
275 - 281	821.4438	820.4365	820.4079	0.0287	0	FGEEIAR (No match)
318 - 327	1322.6692	1321.6619	1321.6051	0.0569	1	KDNDFIYHDR (No match)
319 - 327	1194.5726	1193.5653	1193.5101	0.0552	0	DNDFIYHDR (No match)
363 - 378	1806.9989	1805.9916	1805.9093	0.0823	0	MVPVSVQQSLAAYNQR Oxidation (M) (Ions score 6)
363 - 378	1806.9989	1805.9916	1805.9093	0.0823	0	MVPVSVQQSLAAYNQR Oxidation (M) (No match)
428 - 443	1728.0293	1727.0220	1726.9464	0.0756	0	SVIEQGGIQTVDQLIK (No match)
444 - 451	997.6100	996.6027	996.5603	0.0424	0	ELPELLQR (No match)
452 - 461	1244.7115	1243.7042	1243.6520	0.0522	1	NREILDESLR (No match)
462 - 474	1532.7864	1531.7791	1531.7001	0.0790	0	LLDEEEATDNDLR (No match)
484 - 494	1317.7604	1316.7531	1316.7088	0.0443	0	TPSNELYKPLR (No match)
484 - 501	2093.1726	2092.1653	2092.0701	0.0953	1	TPSNELYKPLRAEGTNFR (No match)
589 - 611	2504.3948	2503.3875	2503.2805	0.1070	0	FLTALAQDGVINEEALSVTELDR (No match)
633 - 643	1316.7153	1315.7080	1315.6520	0.0560	0	NIQVSHQEFSK (No match)
646 - 659	1643.9086	1642.9013	1642.8274	0.0739	1	QSNNEANLREEVLK (No match)
681 - 691	1396.8096	1395.8023	1395.7397	0.0626	0	FYNELTEILVR (Ions score 47)
681 - 691	1396.8096	1395.8023	1395.7397	0.0626	0	FYNELTEILVR (No match)
721 - 750	2936.5591	2935.5518	2935.4464	0.1054	0	EPSAPSIPTPAYQSSPAGGHAPTPPTPAPR (No match)
721 - 750	2936.5591	2935.5518	2935.4464	0.1054	0	EPSAPSIPTPAYQSSPAGGHAPTPPTPAPR (Ions score 31)
751 - 772	2375.4138	2374.4065	2374.2943	0.1122	0	TMPPTKPQPPARPPPPVLPANR Oxidation (M) (No match)
751 - 772	2375.4138	2374.4065	2374.2943	0.1122	0	TMPPTKPQPPARPPPPVLPANR Oxidation (M) (No match)

Mascot: http://www.matrixscience.com/

Spot 174

{MATRIX SCIENCE} Mascot Search Results

Protein View

Match to: **gi|119625804** Score: **92** Expect: **0.00012**
moesin, isoform CRA_b [Homo sapiens]

Nominal mass (M_r): **66678**; Calculated pI value: **5.90**
NCBI BLAST search of gi|119625804 against nr
Unformatted sequence string for pasting into other applications

Taxonomy: Homo sapiens

Fixed modifications: Carbamidomethyl (C)
Variable modifications: Oxidation (M)
Cleavage by Trypsin: cuts C-term side of KR unless next residue is P
Sequence Coverage: **18%**

Matched peptides shown in **Bold Red**

```
  1 MDAELEFAIQ PNTTGKQLFD QVVKTIGLRE VWFFGLQYQD TKGFSTWLKL
 51 NKKVTAQDVR KESPLLFKFR AKFYPEDVSE ELIQDITQRL FFLQVKEGIL
101 NDDIYCPPET AVLLASYAVQ SKYGDFNKEV HKSGYLAGDK LLPQRVLEQH
151 KLNKDQWEER IQVWHEEHRG MLREDAVLEY LKIAQDLEMY GVNYFSIKNK
201 KGSELWLGVD ALGLNIYEQN DRLTPKIGFP WSEIRNISFN DKKFVIKPID
251 KKAPDFVFYA PRLRINKRIL ALCMGNHELY MRRRKPDTIE VQQMKAQARE
301 EKHQKQMERA MLENEKKKRE MAEKEKEKIE REKEELMERL KQIEEQTKKA
351 QQELEEQTRR ALELEQERKR AQSEAEKLAK ERQEAEEAKE ALLQASRDQK
401 KTQEQLALEM AELTARISQL EMARQKKESE AVEWQQKAQM VQEDLEKTRA
451 ELKTAMSTPH VAEPAENEQD EQDENGAEAS ADLRADAMAK DRSEEERTTE
501 AEKNERVQKH LKALTSELAN ARDESKKTAN DMIHAENMRL GRDKYKTLRQ
551 IRQGNTKQRI DEFESM
```

Residue Number Increasing Mass Decreasing Mass

Start - End	Observed	Mr(expt)	Mr(calc)	Delta	Miss	Sequence
71 - 89	2281.1162	2280.1089	2280.1273	-0.0184	1	AKFYPEDVSEELIQDITQR (No match)
73 - 89	2081.9851	2080.9778	2080.9953	-0.0174	0	FYPEDVSEELIQDITQR (No match)
146 - 154	1108.5785	1107.5712	1107.6400	-0.0688	1	VLEQHKLNK (No match)

146 - 154	1108.5785	1107.5712	1107.6400	-0.0688	1	VLEQHKLNK (No match)
170 - 182	1552.7853	1551.7780	1551.7966	-0.0186	1	GMLREDAVLEYLK Oxidation (M) (No match)
252 - 262	1310.6860	1309.6787	1309.6818	-0.0031	1	KAPDFVFYAPR (Ions score 7)
252 - 262	1310.6860	1309.6787	1309.6818	-0.0031	1	KAPDFVFYAPR (No match)
253 - 262	1182.5883	1181.5810	1181.5869	-0.0059	0	APDFVFYAPR (Ions score 23)
253 - 262	1182.5883	1181.5810	1181.5869	-0.0059	0	APDFVFYAPR (No match)
269 - 282	1752.8079	1751.8006	1751.8157	-0.0150	0	ILALCMGNHELYMR 2 Oxidation (M) (No match)
350 - 360	1387.6815	1386.6742	1386.6851	-0.0109	1	AQQELEEQTRR (No match)
401 - 416	1847.9279	1846.9206	1846.9458	-0.0252	1	KTQEQLALEMAELTAR Oxidation (M) (No match)
498 - 506	1077.5220	1076.5147	1076.5098	0.0050	1	TTEAEKNER (No match)

Mascot: http://www.matrixscience.com/

Spot 177

Mascot Search Results

Protein View

Match to: **gi|20149594** Score: **299** Expect: **2.4e-025**
heat shock 90kDa protein 1, beta [Homo sapiens]

Nominal mass (M_r): **83554;** Calculated pI value: **4.97**
NCBI BLAST search of gi|20149594 against nr
Unformatted sequence string for pasting into other applications

Taxonomy: Homo sapiens
Links to retrieve other entries containing this sequence from NCBI Entrez:
gi|17865718 from Homo sapiens
gi|386786 from Homo sapiens
gi|13436257 from Homo sapiens
gi|15215418 from Homo sapiens
gi|15680260 from Homo sapiens
gi|16876955 from Homo sapiens
gi|34304590 from Homo sapiens
gi|46249928 from Homo sapiens
gi|56204416 from Homo sapiens
gi|83699651 from Homo sapiens
gi|119624662 from Homo sapiens
gi|119624663 from Homo sapiens
gi|119624665 from Homo sapiens
gi|123991523 from synthetic construct
gi|123999414 from synthetic construct

Fixed modifications: Carbamidomethyl (C)
Variable modifications: Oxidation (M)
Cleavage by Trypsin: cuts C-term side of KR unless next residue is P
Sequence Coverage: **29%**

Matched peptides shown in **Bold Red**

```
  1 MPEEVHHGEE EVETFAFQAE IAQLMSLIIN TFYSNKEIFL RELISNASDA
 51 LDKIRYESLT DPSKLDSGKE LKIDIIPNPQ ERTLTLVDTG IGMTKADLIN
101 NLGTIAKSGT KAFMEALQAG ADISMIGQFG VGFYSAYLVA EKVVVITKHN
151 DDEQYAWESS AGGSFTVRAD HGEPIGRGTK VILHLKEDQT EYLEERRVKE
201 VVKKHSQFIG YPITLYLEKE REKEISDDEA EEEKGEKEEE DKDDEEKPKI
251 EDVGSDEEDD SGKDKKKKTK KIKEKYIDQE ELNKTKPIWT RNPDDITQEE
```

```
301 YGEFYKSLTN DWEDHLAVKH FSVEGQLEFR ALLFIPRRAP FDLFENKKKK
351 NNIKLYVRRV FIMDSCDELI PEYLNFIRGV VDSEDLPLNI SREMLQQSKI
401 LKVIRKNIVK KCLELFSELA EDKENYKKFY EAFSKNLKLG IHEDSTNRRR
451 LSELLRYHTS QSGDEMTSLS EYVSRMKETQ KSIYYITGES KEQVANSAFV
501 ERVRKRGFEV VYMTEPIDEY CVQQLKEFDG KSLVSVTKEG LELPEDEEEK
551 KKMEESKAKF ENLCKLMKEI LDKKVEKVTI SNRLVSSPCC IVTSTYGWTA
601 NMERIMKAQA LRDNSTMGYM MAKKHLEINP DHPIVETLRQ KAEADKNDKA
651 VKDLVVLLFE TALLSSGFSL EDPQTHSNRI YRMIKLGLGI DEDEVAAEEP
701 NAAVPDEIPP LEGDEDASRM EEVD
```

Residue Number Increasing Mass Decreasing Mass

Start - End	Observed	Mr(expt)	Mr(calc)	Delta	Miss	Sequence
42 - 55	1544.8777	1543.8704	1543.8205	0.0499	1	ELISNASDALDKIR (No match)
70 - 82	1564.9283	1563.9210	1563.8620	0.0590	1	ELKIDIIPNPQER (No match)
73 - 82	1194.6882	1193.6809	1193.6404	0.0405	0	IDIIPNPQER (No match)
73 - 82	1194.6882	1193.6809	1193.6404	0.0405	0	IDIIPNPQER (Ions score 57)
169 - 177	951.4939	950.4866	950.4569	0.0297	0	ADHGEPIGR (No match)
181 - 196	2015.1088	2014.1015	2014.0370	0.0645	1	VILHLKEDQTEYLEER (No match)
205 - 219	1809.0181	1808.0108	1807.9508	0.0600	0	HSQFIGYPITLYLEK (No match)
276 - 284	1151.5925	1150.5852	1150.5506	0.0347	0	YIDQEELNK (No match)
292 - 306	1847.8643	1846.8570	1846.7897	0.0674	0	NPDDITQEEYGEFYK (No match)
320 - 330	1348.7107	1347.7034	1347.6571	0.0464	0	HFSVEGQLEFR (No match)
320 - 330	1348.7107	1347.7034	1347.6571	0.0464	0	HFSVEGQLEFR (Ions score 28)
331 - 337	829.5525	828.5452	828.5221	0.0231	0	ALLFIPR (No match)
338 - 347	1236.6750	1235.6677	1235.6298	0.0379	1	RAPFDLFENK (No match)
338 - 347	1236.6750	1235.6677	1235.6298	0.0379	1	RAPFDLFENK (Ions score 17)
379 - 392	1513.8376	1512.8303	1512.7783	0.0520	0	GVVDSEDLPLNISR (Ions score 57)
379 - 392	1513.8376	1512.8303	1512.7783	0.0520	0	GVVDSEDLPLNISR (No match)
429 - 435	891.4487	890.4414	890.4174	0.0241	0	FYEAFSK (No match)
439 - 448	1141.5979	1140.5906	1140.5523	0.0383	0	LGIHEDSTNR (No match)
457 - 475	2193.0222	2192.0149	2191.9327	0.0822	0	YHTSQSGDEMTSLSEYVSR Oxidation (M) (No match)
482 - 502	2391.2493	2390.2420	2390.1753	0.0667	1	SIYYITGESKEQVANSAFVER (No match)
492 - 502	1249.6600	1248.6527	1248.6098	0.0429	0	EQVANSAFVER (No match)
605 - 612	946.5754	945.5681	945.5429	0.0252	1	IMKAQALR Oxidation (M) (No match)
624 - 639	1911.1044	1910.0971	1910.0373	0.0598	1	KHLEINPDHPIVETLR (No match)
625 - 639	1783.0090	1782.0017	1781.9424	0.0593	0	HLEINPDHPIVETLR (No match)

Mascot: http://www.matrixscience.com/

Spot 178

{MATRIX SCIENCE} Mascot Search Results

Protein View

Match to: **gi|62089310** Score: **118** Expect: **3e-007**
Phospholipase C, delta 1 variant [Homo sapiens]

Nominal mass (M_r): **88289;** Calculated pI value: **6.44**
NCBI BLAST search of gi|62089310 against nr
Unformatted sequence string for pasting into other applications

Taxonomy: Homo sapiens

Fixed modifications: Carbamidomethyl (C)
Variable modifications: Oxidation (M)
Cleavage by Trypsin: cuts C-term side of KR unless next residue is P
Sequence Coverage: **11%**

Matched peptides shown in **Bold Red**

```
  1 GAVATQVSPA VPLPPRRPSG MDSGRDFLTL HGLQDDEDLQ ALLKGSQLLK
 51 VKSSSWRRER FYKLQEDCKT IWQESRKVMR TPESQLFSIE DIQEVRMGHR
101 TEGLEKFARD VPEDRCFSIV FKDQRNTLDL IAPSPADAQH WVLGLHKIIH
151 HSGSMDQRQK LQHWIHSCLR KADKNKDNKM SFKELQNFLK ELNIQVDDSY
201 ARKIFRECDH SQTDSLEDEE IEAFYKMLTQ RVEIDRTFAE AAGSGETLSV
251 NQLVTFLQHQ QREEAAGPAL ALSLIERYEP SETAKAQRQM TKDGFLMYLL
301 SADGSAFSLA HRRVYQDMGQ PLSHYLVSSS HNTYLLEDQL AGPSSTEAYI
351 RALCKGCRCL ELDCWDGPNQ EPIIYHGYTF TSKILFCDVL RAIRDYAFKA
401 SPYPVILSLE NHCTLEQQRV MARHLHAILG PMLLNRPLDG VTNSLPSPEQ
451 LKGKILLKGK KLGGLLPPGG EGGPEATVVS DEDEAAEMED EAVRSRVQHK
501 PKEDKLRLAQ ELSDMVIYCK SVHFGGFSSP GTPGQAFYEM ASFSENRALR
551 LLQESGNGFV RHNVGHLSRI YPAGWRTDSS NYSPVEMWNG GCQIVALNFQ
601 TPGPEMDVYQ GRFQDNGACG YVLKPAFLRD PNGTFNPRAL AQGPWWARKR
651 LNIRVISGQQ LPKVNKNKNS IVDPKVTVEI HGVSRDVASR QTAVITNNGF
701 NPWWDTEFAF EVVVPDLALI RFLVEDYDAS SKNDFIGQST IPLNSLKQGY
751 RHVHLMSKNG DQHPSATLFV KISLQD
```

Residue Number Increasing Mass Decreasing Mass

Start - End	Observed	Mr(expt)	Mr(calc)	Delta	Miss	Sequence
70 - 76	919.4710	918.4637	918.4559	0.0078	0	TIWQESR (No match)
81 - 96	1890.9744	1889.9671	1889.9370	0.0301	0	TPESQLFSIEDIQEVR (No match)
81 - 96	1890.9744	1889.9671	1889.9370	0.0301	0	TPESQLFSIEDIQEVR (Ions score 16)
263 - 277	1539.8611	1538.8538	1538.8303	0.0235	0	EEAAGPALALSLIER (No match)
263 - 277	1539.8611	1538.8538	1538.8303	0.0235	0	EEAAGPALALSLIER (Ions score 6)
384 - 391	1035.5844	1034.5771	1034.5582	0.0189	0	ILFCDVLR (No match)
384 - 391	1035.5844	1034.5771	1034.5582	0.0189	0	ILFCDVLR (No match)
400 - 419	2355.1958	2354.1885	2354.1688	0.0197	0	ASPYPVILSLENHCTLEQQR (Ions score 52)
400 - 419	2355.1958	2354.1885	2354.1688	0.0197	0	ASPYPVILSLENHCTLEQQR (No match)
551 - 561	1219.6616	1218.6543	1218.6356	0.0187	0	LLQESGNGFVR (No match)
639 - 648	1155.6248	1154.6175	1154.5985	0.0191	0	ALAQGPWWAR (No match)

Mascot: http://www.matrixscience.com/

Spot 179

{MATRIX SCIENCE} Mascot Search Results

Protein View

Match to: **gi|31283** Score: **148** Expect: **3e-010**
unnamed protein product [Homo sapiens]

Nominal mass (M_r): **69470**; Calculated pI value: **5.94**
NCBI BLAST search of gi|31283 against nr
Unformatted sequence string for pasting into other applications

Taxonomy: Homo sapiens

Fixed modifications: Carbamidomethyl (C)
Variable modifications: Oxidation (M)
Cleavage by Trypsin: cuts C-term side of KR unless next residue is P
Sequence Coverage: **17%**

Matched peptides shown in **Bold Red**

```
  1 MPKPINVRVT TMDAELEFAI QPNTTGKQLF DQVVKTIGLR EVWYFGLHYV
 51 DNKGFPTWLK LDKKVSAQEV RKENPLQFKF RAKFYPEDVA EELIQDITQK
101 LFFLQVKEGI LSDEIYCPPE TAVLLGSYAV QAKFGDYNKE VHKSGYLSSE
151 RLIPQRVMDQ HKLTRDQWED RIQVWHAEHR GMLKDNAMLE YLKIAQDLEM
201 YGINYFEIKN KKGTDLWLGV DALGLNIYEK DDKLTPKIGF PWSEIRNISF
251 NDKKFVIKPI DKKAPDFVFY APRLRINKRI LQLCMGNHEL YMRRRKPDTI
301 EVQQMKAQAR EEKHQKQLER QQLETEKKRR ETVEREKEQM MREKEELMLR
351 LQDYEEKTKK AERELSEQIQ RALQLEEERK RAQEEAERLE ADRMAALRAK
401 EELERQAVDQ IKSQEQLAAE LAEYTAKIAL LEEARRRKED EVEEWQHRAK
451 EAQDDLVKTK EELHLVMTAP PPPPPPVYEP VSYHVQESLQ DEGAEPTGYS
501 AELSSEGIRD DRNEEKRITE AEKNERVQRQ LVTLSSELSQ ARDENKRTHN
551 DIIHNENMRQ GRDKYKTLRQ IRQGNTKQRI DEFEAL
```

Residue Number Increasing Mass Decreasing Mass

Start - End	Observed	Mr(expt)	Mr(calc)	Delta	Miss	Sequence
2 - 8	823.5213	822.5140	822.5075	0.0065	0	PKPINVR (No match)
28 - 35	976.5432	975.5359	975.5389	-0.0030	0	QLFDQVVK (No match)
101 - 107	894.5487	893.5414	893.5374	0.0040	0	LFFLQVK (No match)

172 - 180	1175.6101	1174.6028	1174.5995	0.0033	0	IQVWHAEHR	(No match)
238 - 246	1104.5847	1103.5774	1103.5763	0.0011	0	IGFPWSEIR	(Ions score 12)
238 - 246	1104.5847	1103.5774	1103.5763	0.0011	0	IGFPWSEIR	(No match)
263 - 273	1310.6974	1309.6901	1309.6818	0.0083	1	KAPDFVFYAPR	(Ions score 8)
263 - 273	1310.6974	1309.6901	1309.6818	0.0083	1	KAPDFVFYAPR	(No match)
264 - 273	1182.6006	1181.5933	1181.5869	0.0064	0	APDFVFYAPR	(Ions score 43)
264 - 273	1182.6006	1181.5933	1181.5869	0.0064	0	APDFVFYAPR	(No match)
280 - 293	1777.8578	1776.8505	1776.8473	0.0032	0	ILQLCMGNHELYMR	(No match)
280 - 293	1793.8533	1792.8460	1792.8422	0.0038	0	ILQLCMGNHELYMR	Oxidation (M) (No match)
364 - 371	1002.5297	1001.5224	1001.5141	0.0083	0	ELSEQIQR	(No match)
364 - 371	1002.5297	1001.5224	1001.5141	0.0083	0	ELSEQIQR	(No match)
372 - 379	987.5186	986.5113	986.5032	0.0081	0	ALQLEEER	(No match)
548 - 559	1493.7008	1492.6935	1492.6841	0.0094	0	THNDIIHNENMR	(No match)
548 - 559	1509.6880	1508.6807	1508.6790	0.0017	0	THNDIIHNENMR	Oxidation (M) (No match)
578 - 586	1120.5757	1119.5684	1119.5560	0.0124	1	QRIDEFEAL	(No match)

Mascot: http://www.matrixscience.com/

Spot 180

Mascot Search Results

Protein View

Match to: **gi|119631258** Score: **181** Expect: **1.5e-013**
signal transducer and activator of transcription 1, 91kDa, isoform CRA_c [Homo sapiens]

Nominal mass (M_r): **82379;** Calculated pI value: **6.06**
NCBI BLAST search of gi|119631258 against nr
Unformatted sequence string for pasting into other applications

Taxonomy: Homo sapiens

Fixed modifications: Carbamidomethyl (C)
Variable modifications: Oxidation (M)
Cleavage by Trypsin: cuts C-term side of KR unless next residue is P
Sequence Coverage: **7%**

Matched peptides shown in **Bold Red**

```
  1 MSQWYELQQL DSKFLEQVHQ LYDDSFPMEI RQYLAQWLEK QDWEHAANDV
 51 SFATIRFHDL LSQLDDQYSR FSLENNFLLQ HNIRKSKRNL QDNFQEDPIQ
101 MSMIIYSCLK EERKILENAQ RFNQAQSGNI QSTVMLDKQK ELDSKVRNVK
151 DKVMCIEHEI KSLEDLQDEY DFKCKTLQNR EQLLLKKMYL MLDNKRKEVV
201 HKIIELLNVT ELTQNALIND ELVEWKRRQQ SACIGGPPNA CLDQLQNWFT
251 IVAESLQQVR QQLKKLEELE QKYTYEHDPI TKNKQVLWDR TFSLFQQLIQ
301 SSFVVERQPC MPTHPQRPLV LKTGVQFTVK LRLLVKLQEL NYNLKVKVLF
351 DKDVNERNTV KGFRKFNILG THTKVMNMEE STNGSLAAEF RHLQLKEQKN
401 AGTRTNEGPL IVTEELHSLS FETQLCQPGL VIDLETTSLP VVVISNVSQL
451 PSGWASILWY NMLVAEPRNL SFFLTPPCAR WAQLSEVLSW QFSSVTKRGL
501 NVDQLNMLGE KLLGPNASPD GLIPWTRFCK ENINDKNFPF WLWIESILEL
551 IKKHLLPLWN DGCIMGFISK ERERALLKDQ QPGTFLLRFS ESSREGAITF
601 TWVERSQNGG EPDFHAVEPY TKKELSAVTF PDIIRNYKVM AAENIPENPL
651 KYLYPNIDKD HAFGKYYSRP KEAPEPMELD GPKGTGYIKT ELISVSEVTV
701 LG
```

Residue Number Increasing Mass Decreasing Mass

Start - End Observed Mr(expt) Mr(calc) Delta Miss Sequence

153 - 161	1174.6095	1173.6022	1173.5522	0.0501	0	VMCIEHEIK Oxidation (M) (No match)
348 - 357	1234.6278	1233.6205	1233.6353	-0.0148	1	VLFDKDVNER (Ions score 25)
348 - 357	1234.6278	1233.6205	1233.6353	-0.0148	1	VLFDKDVNER (No match)
469 - 480	1422.7047	1421.6974	1421.7125	-0.0151	0	NLSFFLTPPCAR (No match)
469 - 480	1422.7047	1421.6974	1421.7125	-0.0151	0	NLSFFLTPPCAR (Ions score 6)
579 - 588	1174.6095	1173.6022	1173.6142	-0.0120	0	DQQPGTFLLR (No match)
652 - 665	1680.8256	1679.8183	1679.8307	-0.0124	1	YLYPNIDKDHAFGK (Ions score 86)
652 - 665	1680.8256	1679.8183	1679.8307	-0.0124	1	YLYPNIDKDHAFGK (No match)

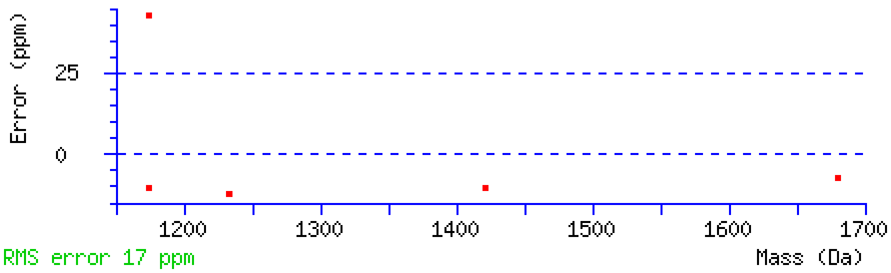

RMS error 17 ppm

Mascot: http://www.matrixscience.com/

Spot 181

MATRIX SCIENCE Mascot Search Results

Protein View

Match to: **gi|48145705** Score: **397** Expect: **3.8e-035**
HCLS1 [Homo sapiens]

Nominal mass (M_r): **54065;** Calculated pI value: **4.74**
NCBI BLAST search of gi|48145705 against nr
Unformatted sequence string for pasting into other applications

Taxonomy: Homo sapiens

Fixed modifications: Carbamidomethyl (C)
Variable modifications: Oxidation (M)
Cleavage by Trypsin: cuts C-term side of KR unless next residue is P
Sequence Coverage: **44%**

Matched peptides shown in **Bold Red**

```
  1 MWKSVVGHDV SVSVETQGDD WDTDPDFVND ISEKEQRWGA KTIEGSGRTE
 51 HINIHQLRNK VSEEHDVLRK KEMESGPKAS HGYGGRFGVE RDRMDKSAVG
101 HEYVAEVEKH SSQTDAAKGF GGKYGVERDR ADKSAVGFDY KGEVEKHTSQ
151 KDYSRGFGGR YGVEKDKWDK AALGYDYKGE TEKHESQRDY AKGFGGQYGI
201 QKDRVDKSAV GFNEMEAPTT AYKKTTPIEA ASSGARGLKA KFESMAEEKR
251 KREEEEKAQQ VARRQQERKA VTKRSPEAPQ PVIAMEEPAV PAPLPKKISS
301 EAWPPVGTPP SSESEPVRTS REHPVPLLPI RQTLPEDNEE PPALPPRTLE
351 GLQVEEEPVY EAEPEPEPEP EPEPENDYED VEEMDRHEQE DEPEGDYEEV
401 LEPEDSSFSS ALAGSSGCPA GAGAGAVALG ISAVALYDYQ GEGSDELSFD
451 PDDVITDIEM VDEGWWRGRC HGHFGLFPAN YVKLLD
```

Residue Number Increasing Mass Decreasing Mass

Start - End	Observed	Mr(expt)	Mr(calc)	Delta	Miss	Sequence
49 - 58	1260.6842	1259.6769	1259.6734	0.0035	0	TEHINIHQLR (No match)
49 - 58	1260.6842	1259.6769	1259.6734	0.0035	0	TEHINIHQLR (Ions score 46)
59 - 69	1325.6814	1324.6741	1324.6735	0.0007	1	NKVSEEHDVLR (No match)
61 - 69	1083.5505	1082.5432	1082.5356	0.0076	0	VSEEHDVLR (No match)
79 - 86	804.3819	803.3746	803.3674	0.0072	0	ASHGYGGR (No match)

97 - 109	1417.7007	1416.6934	1416.6884	0.0050	0	SAVGHEYVAEVEK (No match)
97 - 118	2343.1584	2342.1511	2342.1138	0.0374	1	SAVGHEYVAEVEKHSSQTDAAK (No match)
134 - 146	1428.7003	1427.6930	1427.6932	-0.0001	1	SAVGFDYKGEVEK (No match)
179 - 188	1200.5696	1199.5623	1199.5530	0.0093	1	GETEKHESQR (No match)
193 - 202	1054.5293	1053.5220	1053.5243	-0.0022	0	GFGGQYGIQK (No match)
193 - 202	1054.5293	1053.5220	1053.5243	-0.0022	0	GFGGQYGIQK (Ions score 43)
208 - 224	1859.8871	1858.8798	1858.8770	0.0028	1	SAVGFNEMEAPTTAYKK Oxidation (M) (No match)
225 - 236	1160.5991	1159.5918	1159.5833	0.0086	0	TTPIEAASSGAR (No match)
242 - 250	1142.5162	1141.5089	1141.5073	0.0016	1	FESMAEEKR Oxidation (M) (No match)
253 - 263	1316.6455	1315.6382	1315.6367	0.0015	1	EEEEKAQQVAR (No match)
274 - 296	2440.3086	2439.3013	2439.2831	0.0182	1	RSPEAPQPVIAMEEPAVPAPLPK Oxidation (M) (No match)
275 - 296	2284.2073	2283.2000	2283.1820	0.0180	0	SPEAPQPVIAMEEPAVPAPLPK Oxidation (M) (No match)
297 - 318	2337.1765	2336.1692	2336.1648	0.0045	1	KISSEAWPPVGTPPSSESEPVR (No match)
322 - 331	1170.7025	1169.6952	1169.6920	0.0032	0	EHPVPLLPIR (Ions score 42)
322 - 331	1170.7025	1169.6952	1169.6920	0.0032	0	EHPVPLLPIR (No match)
332 - 347	1802.8905	1801.8832	1801.8846	-0.0014	0	QTLPEDNEEPPALPPR (Ions score 93)
332 - 347	1802.8905	1801.8832	1801.8846	-0.0014	0	QTLPEDNEEPPALPPR (No match)
470 - 483	1646.7915	1645.7842	1645.7823	0.0019	0	CHGHFGLFPANYVK (No match)

Mascot: http://www.matrixscience.com/

Spot 182

{MATRIX} {SCIENCE} Mascot Search Results

Protein View

Match to: **gi|62738788** Score: **114** Expect: **7.7e-007**
Chain A, Structure Of Unphosphorylated Stat1

Nominal mass (M_r): **80465;** Calculated pI value: **6.26**
NCBI BLAST search of gi|62738788 against nr
Unformatted sequence string for pasting into other applications

Taxonomy: Homo sapiens
Links to retrieve other entries containing this sequence from NCBI Entrez:
gi|62738789 from Homo sapiens

Fixed modifications: Carbamidomethyl (C)
Variable modifications: Oxidation (M)
Cleavage by Trypsin: cuts C-term side of KR unless next residue is P
Sequence Coverage: **14%**

Matched peptides shown in **Bold Red**

```
  1 MSQWYELQQL DSKFLEQVHQ LYDDSFPMEI RQYLAQWLEK QDWEHAANDV
 51 SFATIRFHDL LSQLDDQYSR FSLENNFLLQ HNIRKSKRNL QDNFQEDPIQ
101 MSMIIYSCLK EERKILENAQ RFNQAQSGNI QSTVMLDKQK ELDSKVRNVK
151 DKVMCIEHEI KSLEDLQDEY DFKCKTLQNR EHETNGVAKS DQKQEQLLLK
201 KMYLMLDNKR KEVVHKIIEL LNVTELTQNA LINDELVEWK RRQQSACIGG
251 PPNACLDQLQ NWFTIVAESL QQVRQQLKKL EELEQKYTYE HDPITKNKQV
301 LWDRTFSLFQ QLIQSSFVVE RQPCMPTHPQ RPLVLKTGVQ FTVKLRLLVK
351 LQELNYNLKV KVLFDKDVNE RNTVKGFRKF NILGTHTKVM NMEESTNGSL
401 AAEFRHLQLK EQKNAGTRTN EGPLIVTEEL HSLSFETQLC QPGLVIDLET
451 TSLPVVVISN VSQLPSGWAS ILWYNMLVAE PRNLSFFLTP PCARWAQLSE
501 VLSWQFSSVT KRGLNVDQLN MLGEKLLGPN ASPDGLIPWT RFCKENINDK
551 NFPFWLWIES ILELIKKHLL PLWNDGCIMG FISKERERAL LKDQQPGTFL
601 LRFSESSREG AITFTWVERS QNGGEPDFHA VEPYTKKELS AVTFPDIIRN
651 YKVMAAENIP ENPLKYLYPN IDKDHAFGKY YSR
```

Residue Number Increasing Mass Decreasing Mass

Start - End	Observed	Mr(expt)	Mr(calc)	Delta	Miss	Sequence
57 - 70	1736.8368	1735.8295	1735.8165	0.0130	0	FHDLLSQLDDQYSR (No match)
71 - 84	1744.9213	1743.9140	1743.9056	0.0084	0	FSLENNFLLQHNIR (No match)
322 - 336	1801.9639	1800.9566	1800.9491	0.0075	0	QPCMPTHPQRPLVLK (No match)
362 - 371	1234.6561	1233.6488	1233.6353	0.0135	1	VLFDKDVNER (Ions score 18)
362 - 371	1234.6561	1233.6488	1233.6353	0.0135	1	VLFDKDVNER (No match)
483 - 494	1422.7310	1421.7237	1421.7125	0.0112	0	NLSFFLTPPCAR (Ions score 7)
483 - 494	1422.7310	1421.7237	1421.7125	0.0112	0	NLSFFLTPPCAR (No match)
589 - 602	1599.9326	1598.9253	1598.9144	0.0110	1	ALLKDQQPGTFLLR (Ions score 17)
589 - 602	1599.9326	1598.9253	1598.9144	0.0110	1	ALLKDQQPGTFLLR (No match)
593 - 602	1174.6309	1173.6236	1173.6142	0.0094	0	DQQPGTFLLR (No match)
609 - 619	1308.6724	1307.6651	1307.6509	0.0142	0	EGAITFTWVER (No match)
638 - 649	1360.7620	1359.7547	1359.7398	0.0150	0	ELSAVTFPDIIR (No match)

Mascot: http://www.matrixscience.com/

Spot 184

Mascot Search Results

Protein View

Match to: **gi|119599912** Score: **163** Expect: **9.6e-012**
hematopoietic cell-specific Lyn substrate 1, isoform CRA_b [Homo sapiens]

Nominal mass (M_r): **57793;** Calculated pI value: **4.80**
NCBI BLAST search of gi|119599912 against nr
Unformatted sequence string for pasting into other applications

Taxonomy: Homo sapiens

Fixed modifications: Carbamidomethyl (C)
Variable modifications: Oxidation (M)
Cleavage by Trypsin: cuts C-term side of KR unless next residue is P
Sequence Coverage: **19%**

Matched peptides shown in **Bold Red**

```
  1 MWKSVVGHDV SVSVETQGDD WDTDPDFVND ISEKEQRWGA KTIEGSGRTE
 51 HINIHQLRNK VSEEHDVLRK KEMESGPKAS HGYGGRFGVE RDRMDKSAVG
101 HEYVAEVEKH SSQTDAAKGF GGKYGVERDR ADKSAVGFDY KGEVEKHTSQ
151 KDYSRGFDGR YGVEKDKWDK AALGYDYKGE TEKHESQRDY AKGFGGQYGI
201 QKDRVDKSAV GFNEMEAPTT AYKKTTPIEA ASSGARGLKA KFESMAEEKR
251 KREEEEKAQQ VARRQQERKA VTKRSPEAPQ PVIAMEEPAV PAPLPKKISS
301 EAWPPVGTPP SSESEPVRTS REHPVPLLPI RQTLPEDNEE PPALPPRTLE
351 GLQVEEEPVY EAEPEPEPEP EPEPENDYED VEEMDRHEQE DEPEGDYEEV
401 LEPEDSSFSS ALAGSSGCPA GAGAGAVALG ISAVALYDYQ GEGSDELSFD
451 PDDVITDIEM VDEGWWRGRC HGHFGLFPAN YVKLLDFPVC LPGKVRLDCV
501 VCLIVLFAHF PSLKKYL
```

Residue Number Increasing Mass Decreasing Mass

Start - End	Observed	Mr(expt)	Mr(calc)	Delta	Miss	Sequence	
49 - 58	1260.7075	1259.7002	1259.6734	0.0268	0	TEHINIHQLR	(No match)
49 - 58	1260.7075	1259.7002	1259.6734	0.0268	0	TEHINIHQLR	(Ions score 22)
59 - 69	1325.6912	1324.6839	1324.6735	0.0105	1	NKVSEEHDVLR	(No match)
59 - 69	1325.6912	1324.6839	1324.6735	0.0105	1	NKVSEEHDVLR	(No match)

79 - 86	804.3872	803.3799	803.3674	0.0125	0	**ASHGYGGR** (No match)
193 - 202	1054.5435	1053.5362	1053.5243	0.0120	0	**GFGGQYGIQK** (No match)
193 - 202	1054.5435	1053.5362	1053.5243	0.0120	0	**GFGGQYGIQK** (No match)
274 - 296	2440.3557	2439.3484	2439.2831	0.0653	1	**RSPEAPQPVIAMEEPAVPAPLPK** Oxidation (M) (No match)
322 - 331	1170.7206	1169.7133	1169.6920	0.0213	0	**EHPVPLLPIR** (Ions score 18)
322 - 331	1170.7206	1169.7133	1169.6920	0.0213	0	**EHPVPLLPIR** (No match)
332 - 347	1802.9293	1801.9220	1801.8846	0.0374	0	**QTLPEDNEEPPALPPR** (Ions score 52)
332 - 347	1802.9293	1801.9220	1801.8846	0.0374	0	**QTLPEDNEEPPALPPR** (No match)
470 - 483	1646.8232	1645.8159	1645.7823	0.0336	0	**CHGHFGLFPANYVK** (No match)

Mascot: http://www.matrixscience.com/

Spot 185

{MATRIX SCIENCE} Mascot Search Results

Protein View

Match to: **gi|15277503** Score: **228** Expect: **3e-018**
ACTB protein [Homo sapiens]

Nominal mass (M_r): **40536;** Calculated pI value: **5.55**
NCBI BLAST search of gi|15277503 against nr
Unformatted sequence string for pasting into other applications

Taxonomy: Homo sapiens

Fixed modifications: Carbamidomethyl (C)
Variable modifications: Oxidation (M)
Cleavage by Trypsin: cuts C-term side of KR unless next residue is P
Sequence Coverage: **18%**

Matched peptides shown in **Bold Red**

```
  1 MCKAGFAGDD APRAVFPSIV GRPRHQGVMV GMGQKDSYVG DEAQSKRGIL
 51 TLKYPIEHGI VTNWDDMEKI WHHTFYNELR VAPEEHPVLL TEAPLNPKAN
101 LEKMTQIMFE TFNTPAMYVA IQAVLSLYAS GRTTGIVMDS GDGVTHTVPI
151 YEGYALPHAI LRLDLAGRDL TDYLMKILTE RGYSFTTTAE REIVRDIKEK
201 LCYVALDFEQ EMATAASSSS LEKSYELPDG QVITIGNERF RCPEALFQPS
251 FLGMESCGIH ETTFNSIMKC DVDIRKDLYA NTVLSGGTTM YPGIADRMQK
301 EITALAPSTM KIKIIAPPER KYSVWIGGSI LASLSTFQQM WISKQEYDES
351 GPSIVHRKCF
```

Residue Number Increasing Mass Decreasing Mass

Start - End	Observed	Mr(expt)	Mr(calc)	Delta	Miss	Sequence
4 - 13	976.4305	975.4232	975.4409	-0.0177	0	AGFAGDDAPR (No match)
14 - 24	1198.6810	1197.6737	1197.6982	-0.0244	0	AVFPSIVGRPR (Ions score 20)
14 - 24	1198.6810	1197.6737	1197.6982	-0.0244	0	AVFPSIVGRPR (No match)
81 - 98	1954.0334	1953.0261	1953.0571	-0.0309	0	VAPEEHPVLLTEAPLNPK (No match)
81 - 98	1954.0334	1953.0261	1953.0571	-0.0309	0	VAPEEHPVLLTEAPLNPK (Ions score 102)
182 - 191	1132.5056	1131.4983	1131.5196	-0.0213	0	GYSFTTTAER (No match)
224 - 239	1790.8600	1789.8527	1789.8846	-0.0318	0	SYELPDGQVITIGNER (Ions score 46)

224 - 239 1790.8600 1789.8527 1789.8846 -0.0318 0 SYELPDGQVITIGNER (No match)

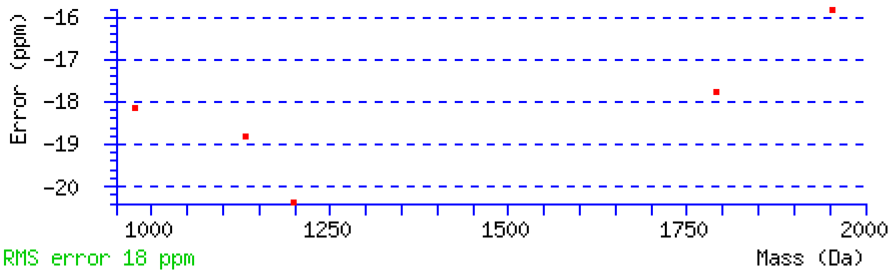

RMS error 18 ppm

Mascot: http://www.matrixscience.com/

Spot 187

{MATRIX SCIENCE} Mascot Search Results

Protein View

Match to: **gi|48145705** Score: **91** Expect: **0.00016**
HCLS1 [Homo sapiens]

Nominal mass (M_r): **54065**; Calculated pI value: **4.74**
NCBI BLAST search of gi|48145705 against nr
Unformatted sequence string for pasting into other applications

Taxonomy: Homo sapiens

Fixed modifications: Carbamidomethyl (C)
Variable modifications: Oxidation (M)
Cleavage by Trypsin: cuts C-term side of KR unless next residue is P
Sequence Coverage: **9%**

Matched peptides shown in **Bold Red**

```
  1 MWKSVVGHDV SVSVETQGDD WDTDPDFVND ISEKEQRWGA KTIEGSGRTE
 51 HINIHQLRNK VSEEHDVLRK KEMESGPKAS HGYGGRFGVE RDRMDKSAVG
101 HEYVAEVEKH SSQTDAAKGF GGKYGVERDR ADKSAVGFDY KGEVEKHTSQ
151 KDYSRGFGGR YGVEKDKWDK AALGYDYKGE TEKHESQRDY AKGFGGQYGI
201 QKDRVDKSAV GFNEMEAPTT AYKKTTPIEA ASSGARGLKA KFESMAEEKR
251 KREEEEKAQQ VARRQQERKA VTKRSPEAPQ PVIAMEEPAV PAPLPKKISS
301 EAWPPVGTPP SSESEPVRTS REHPVPLLPI RQTLPEDNEE PPALPPRTLE
351 GLQVEEEPVY EAEPEPEPEP EPEPENDYED VEEMDRHEQE DEPEGDYEEV
401 LEPEDSSFSS ALAGSSGCPA GAGAGAVALG ISAVALYDYQ GEGSDELSFD
451 PDDVITDIEM VDEGWWRGRC HGHFGLFPAN YVKLLD
```

Residue Number Increasing Mass Decreasing Mass

Start - End	Observed	Mr(expt)	Mr(calc)	Delta	Miss	Sequence	
49 - 58	1260.6868	1259.6795	1259.6734	0.0061	0	TEHINIHQLR	(Ions score 3)
49 - 58	1260.6868	1259.6795	1259.6734	0.0061	0	TEHINIHQLR	(No match)
193 - 202	1054.5259	1053.5186	1053.5243	-0.0056	0	GFGGQYGIQK	(No match)
193 - 202	1054.5259	1053.5186	1053.5243	-0.0056	0	GFGGQYGIQK	(No match)
322 - 331	1170.7034	1169.6961	1169.6920	0.0041	0	EHPVPLLPIR	(Ions score 15)

322 - 331	1170.7034	1169.6961	1169.6920	0.0041	0	**EHPVPLLPIR**	(No match)
332 - 347	1802.9056	1801.8983	1801.8846	0.0137	0	**QTLPEDNEEPPALPPR**	(No match)
332 - 347	1802.9056	1801.8983	1801.8846	0.0137	0	**QTLPEDNEEPPALPPR**	(Ions score 51)

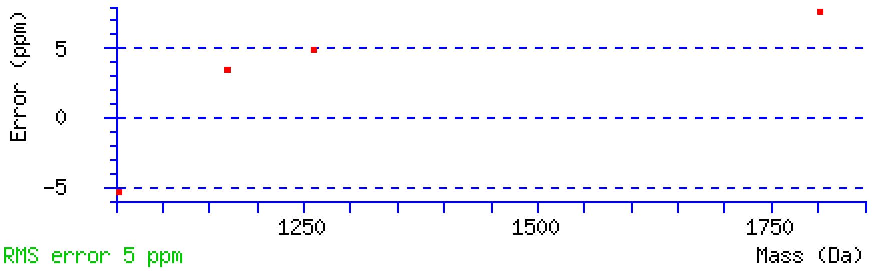

RMS error 5 ppm

Mascot: http://www.matrixscience.com/

Spot 188

{MATRIX SCIENCE} Mascot Search Results

Protein View

Match to: **gi|119631259** Score: **191** Expect: **1.5e-014**
signal transducer and activator of transcription 1, 91kDa, isoform CRA_d [Homo sapiens]

Nominal mass (M_r): **87076;** Calculated pI value: **5.80**
NCBI BLAST search of gi|119631259 against nr
Unformatted sequence string for pasting into other applications

Taxonomy: Homo sapiens

Fixed modifications: Carbamidomethyl (C)
Variable modifications: Oxidation (M)
Cleavage by Trypsin: cuts C-term side of KR unless next residue is P
Sequence Coverage: **13%**

Matched peptides shown in **Bold Red**

```
  1 MSQWYELQQL DSKFLEQVHQ LYDDSFPMEI RQYLAQWLEK QDWEHAANDV
 51 SFATIRFHDL LSQLDDQYSR FSLENNFLLQ HNIRKSKRNL QDNFQEDPIQ
101 MSMIIYSCLK EERKILENAQ RFNQAQSGNI QSTVMLDKQK ELDSKVRNVK
151 DKVMCIEHEI KSLEDLQDEY DFKCKTLQNR EHETNGVAKS DQKQEQLLLK
201 KMYLMLDNKR KEVVHKIIEL LNVTELTQNA LINDELVEWK RRQQSACIGG
251 PPNACLDQLQ NCLQQVRQQL KKLEELEQKY TYEHDPITKN KQVLWDRTFS
301 LFQQLIQSSF VVERQPCMPT HPQRPLVLKT GVQFTVKLRL LVKLQELNYN
351 LKVKVLFDKD VNERNTVKGF RKFNILGTHT KVMNMEESTN GSLAAEFRHL
401 QLKEQKNAGT RTNEGPLIVT EELHSLSFET QLCQPGLVID LETTSLPVVV
451 ISNVSQLPSG WASILWYNML VAEPRNLSFF LTPPCARWAQ LSEVLSWQFS
501 SVTKRGLNVD QLNMLGEKLL GPNASPDGLI PWTRFCKENI NDKNFPFWLW
551 IESILELIKK HLLPLWNDGC IMGFISKERE RALLKDQQPG TFLLRFSESS
601 REGAITFTWV ERSQNGGEPD FHAVEPYTKK ELSAVTFPDI IRNYKVMAAE
651 NIPENPLKYL YPNIDKDHAF GKYYSRPKEA PEPMELDGPK GTGYIKTELI
701 SVSEVHPSRL QTTDNLLPMS PEEFDEVSRI VGSVEFDSMM NTV
```

Residue Number Increasing Mass Decreasing Mass

Start - End	Observed	Mr(expt)	Mr(calc)	Delta	Miss	Sequence

57 - 70	1736.8413	1735.8340	1735.8165	0.0175	0	FHDLLSQLDDQYSR (No match)
71 - 84	1744.9275	1743.9202	1743.9056	0.0146	0	FSLENNFLLQHNIR (No match)
71 - 84	1744.9275	1743.9202	1743.9056	0.0146	0	FSLENNFLLQHNIR (Ions score 11)
71 - 85	1873.0237	1872.0164	1872.0005	0.0159	1	FSLENNFLLQHNIRK (No match)
355 - 364	1234.6541	1233.6468	1233.6353	0.0115	1	VLFDKDVNER (No match)
355 - 364	1234.6541	1233.6468	1233.6353	0.0115	1	VLFDKDVNER (Ions score 45)
476 - 487	1422.7373	1421.7300	1421.7125	0.0175	0	NLSFFLTPPCAR (Ions score 16)
476 - 487	1422.7373	1421.7300	1421.7125	0.0175	0	NLSFFLTPPCAR (No match)
582 - 595	1599.9377	1598.9304	1598.9144	0.0161	1	ALLKDQQPGTFLLR (Ions score 14)
582 - 595	1599.9377	1598.9304	1598.9144	0.0161	1	ALLKDQQPGTFLLR (No match)
586 - 595	1174.6333	1173.6260	1173.6142	0.0118	0	DQQPGTFLLR (No match)
602 - 612	1308.6741	1307.6668	1307.6509	0.0159	0	EGAITFTWVER (No match)
631 - 642	1360.7659	1359.7586	1359.7398	0.0189	0	ELSAVTFPDIIR (No match)
631 - 642	1360.7659	1359.7586	1359.7398	0.0189	0	ELSAVTFPDIIR (Ions score 25)
697 - 709	1453.7723	1452.7650	1452.7572	0.0078	0	TELISVSEVHPSR (No match)

Mascot: http://www.matrixscience.com/

Spot 189

Mascot Search Results

Protein View

Match to: **gi|62738788** Score: **78** Expect: **0.0029**
Chain A, Structure Of Unphosphorylated Stat1

Nominal mass (M_r): **80465**; Calculated pI value: **6.26**
NCBI BLAST search of gi|62738788 against nr
Unformatted sequence string for pasting into other applications

Taxonomy: Homo sapiens
Links to retrieve other entries containing this sequence from NCBI Entrez:
gi|62738789 from Homo sapiens

Fixed modifications: Carbamidomethyl (C)
Variable modifications: Oxidation (M)
Cleavage by Trypsin: cuts C-term side of KR unless next residue is P
Sequence Coverage: **13%**

Matched peptides shown in **Bold Red**

```
  1 MSQWYELQQL DSKFLEQVHQ LYDDSFPMEI RQYLAQWLEK QDWEHAANDV
 51 SFATIRFHDL LSQLDDQYSR FSLENNFLLQ HNIRKSKRNL QDNFQEDPIQ
101 MSMIIYSCLK EERKILENAQ RFNQAQSGNI QSTVMLDKQK ELDSKVRNVK
151 DKVMCIEHEI KSLEDLQDEY DFKCKTLQNR EHETNGVAKS DQKQEQLLLK
201 KMYLMLDNKR KEVVHKIIEL LNVTELTQNA LINDELVEWK RRQQSACIGG
251 PPNACLDQLQ NWFTIVAESL QQVRQQLKKL EELEQKYTYE HDPITKNKQV
301 LWDRTFSLFQ QLIQSSFVVE RQPCMPTHPQ RPLVLKTGVQ FTVKLRLLVK
351 LQELNYNLKV KVLFDKDVNE RNTVKGFRKF NILGTHTKVM NMEESTNGSL
401 AAEFRHLQLK EQKNAGTRTN EGPLIVTEEL HSLSFETQLC QPGLVIDLET
451 TSLPVVVISN VSQLPSGWAS ILWYNMLVAE PRNLSFFLTP PCARWAQLSE
501 VLSWQFSSVT KRGLNVDQLN MLGEKLLGPN ASPDGLIPWT RFCKENINDK
551 NFPFWLWIES ILELIKKHLL PLWNDGCIMG FISKERERAL LKDQQPGTFL
601 LRFSESSREG AITFTWVERS QNGGEPDFHA VEPYTKKELS AVTFPDIIRN
651 YKVMAAENIP ENPLKYLYPN IDKDHAFGKY YSR
```

Residue Number Increasing Mass Decreasing Mass

Start - End	Observed	Mr(expt)	Mr(calc)	Delta	Miss	Sequence
57 - 70	1736.8203	1735.8130	1735.8165	-0.0035	0	FHDLLSQLDDQYSR (No match)
71 - 84	1744.9094	1743.9021	1743.9056	-0.0035	0	FSLENNFLLQHNIR (No match)
362 - 371	1234.6447	1233.6374	1233.6353	0.0021	1	VLFDKDVNER (No match)
362 - 371	1234.6447	1233.6374	1233.6353	0.0021	1	VLFDKDVNER (Ions score 20)
483 - 494	1422.7198	1421.7125	1421.7125	0.0000	0	NLSFFLTPPCAR (No match)
483 - 494	1422.7198	1421.7125	1421.7125	0.0000	0	NLSFFLTPPCAR (No match)
589 - 602	1599.9276	1598.9203	1598.9144	0.0060	1	ALLKDQQPGTFLLR (No match)
593 - 602	1174.6276	1173.6203	1173.6142	0.0061	0	DQQPGTFLLR (No match)
638 - 649	1360.7517	1359.7444	1359.7398	0.0047	0	ELSAVTFPDIIR (No match)
666 - 679	1680.8306	1679.8233	1679.8307	-0.0074	1	YLYPNIDKDHAFGK (No match)

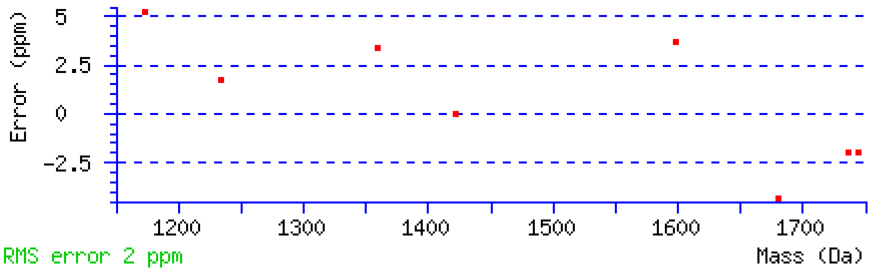

Mascot: http://www.matrixscience.com/

Spot 191

{MATRIX SCIENCE} Mascot Search Results

Protein View

Match to: **gi|148539876** Score: **70** Expect: **0.021**
beta adrenergic receptor kinase 1 [Homo sapiens]

Nominal mass (M_r): **80321;** Calculated pI value: **6.89**
NCBI BLAST search of gi|148539876 against nr
Unformatted sequence string for pasting into other applications

Taxonomy: Homo sapiens
Links to retrieve other entries containing this sequence from NCBI Entrez:
gi|126302521 from Homo sapiens
gi|179335 from Homo sapiens
gi|23272739 from Homo sapiens
gi|60552563 from Homo sapiens
gi|123980490 from synthetic construct
gi|123995305 from synthetic construct

Fixed modifications: Carbamidomethyl (C)
Variable modifications: Oxidation (M)
Cleavage by Trypsin: cuts C-term side of KR unless next residue is P
Sequence Coverage: **14%**

Matched peptides shown in **Bold Red**

```
  1 MADLEAVLAD VSYLMAMEKS KATPAARASK KILLPEPSIR SVMQKYLEDR
 51 GEVTFEKIFS QKLGYLLFRD FCLNHLEEAR PLVEFYEEIK KYEKLETEEE
101 RVARSREIFD SYIMKELLAC SHPFSKSATE HVQGHLGKKQ VPPDLFQPYI
151 EEICQNLRGD VFQKFIESDK FTRFCQWKNV ELNIHLTMND FSVHRIIGRG
201 GFGEVYGCRK ADTGKMYAMK CLDKKRIKMK QGETLALNER IMLSLVSTGD
251 CPFIVCMSYA FHTPDKLSFI LDLMNGGDLH YHLSQHGVFS EADMRFYAAE
301 IILGLEHMHN RFVVYRDLKP ANILLDEHGH VRISDLGLAC DFSKKKPHAS
351 VGTHGYMAPE VLQKGVAYDS SADWFSLGCM LFKLLRGHSP FRQHKTKDKH
401 EIDRMTLTMA VELPDSFSPE LRSLLEGLLQ RDVNRRLGCL GRGAQEVKES
451 PFFRSLDWQM VFLQKYPPPL IPPRGEVNAA DAFDIGSFDE EDTKGIKLLD
501 SDQELYRNFP LTISERWQQE VAETVFDTIN AETDRLEARK KAKNKQLGHE
551 EDYALGKDCI MHGYMSKMGN PFLTQWQRRY FYLFPNRLEW RGEGEAPQSL
601 LTMEEIQSVE ETQIKERKCL LLKIRGGKQF ILQCDSDPEL VQWKKELRDA
651 YREAQQLVQR VPKMKNKPRS PVVELSKVPL VQRGSANGL
```

Residue Number Increasing Mass Decreasing Mass

Start - End	Observed	Mr(expt)	Mr(calc)	Delta	Miss	Sequence
63 - 69	881.5306	880.5233	880.5170	0.0063	0	LGYLLFR (No match)
179 - 195	2039.0140	2038.0067	2038.0054	0.0013	0	NVELNIHLTMNDFSVHR (No match)
200 - 209	1101.4861	1100.4788	1100.4708	0.0080	0	GGFGEVYGCR (No match)
200 - 209	1101.4861	1100.4788	1100.4708	0.0080	0	GGFGEVYGCR (Ions score 4)
296 - 311	1929.9669	1928.9596	1928.9566	0.0030	0	FYAAEIILGLEHMHNR Oxidation (M) (No match)
317 - 332	1826.9851	1825.9778	1825.9798	-0.0020	0	DLKPANILLDEHGHVR (No match)
317 - 332	1826.9851	1825.9778	1825.9798	-0.0020	0	DLKPANILLDEHGHVR (Ions score 11)
423 - 431	1028.6166	1027.6093	1027.6025	0.0068	0	SLLEGLLQR (No match)
466 - 474	1049.6194	1048.6121	1048.6069	0.0052	0	YPPPLIPPR (No match)
466 - 474	1049.6194	1048.6121	1048.6069	0.0052	0	YPPPLIPPR (No match)
498 - 507	1251.6680	1250.6607	1250.6142	0.0465	0	LLDSDQELYR (No match)
580 - 587	1119.5654	1118.5581	1118.5549	0.0033	0	YFYLFPNR (No match)

Mascot: http://www.matrixscience.com/

Spot 193

{MATRIX SCIENCE} Mascot Search Results

Protein View

Match to: **gi|37930520** Score: **120** Expect: **1.9e-007**
cell migration-inducing gene 2 [Homo sapiens]

Nominal mass (M_r): **63691;** Calculated pI value: **5.46**
NCBI BLAST search of gi|37930520 against nr
Unformatted sequence string for pasting into other applications

Taxonomy: Homo sapiens

Fixed modifications: Carbamidomethyl (C)
Variable modifications: Oxidation (M)
Cleavage by Trypsin: cuts C-term side of KR unless next residue is P
Sequence Coverage: **22%**

Matched peptides shown in **Bold Red**

```
  1 MGCGLNKLEK RDEKRPGNIY STLKRPQVET KIDVSYEYRF LEFTTLSAAE
 51 LPGSSAVRLA SLRDLPAQLL ELYQQGFSLA ALHPFVQPTH EREKTPLEHI
101 FRAILIKKTD RSQKTDLHNE GYILELDCCS SLDHPTDQKL IPEFIKKIQE
151 AASQGLKFVG VIPQYHSSVN SAGSSAPVST ANSTEDARDA KNARGDHASL
201 ENEKPGTGDV CSAPAGRNQS PEPSSGPRGE VPLAKQPSSP SGEGDGGELS
251 PQGVSKTLDG PESNPLEVHE EPLSGKMEIF TLFNKPKSHQ KCRQYYPVTI
301 PLHVSKNGQT VSGLDANWLE HMSDHFRKGG MLVNAVFYLG IVNDSLHGLT
351 DGVFIFEAVS TEDSKTIQGY DAIVVEQWTV LEGVEVQTDY VPLLNSLAAY
401 GWQLTCVLPT PVVKTTSEGS ISTKQIVFLQ RPCLPQKIKK KESKFQWRFS
451 REEMHNRQMR KSKGKLSARD KQQAEENEKN LEDQSSKAGD MGNCVSGQQQ
501 EGGVSEEMKG PVQEDKGEQL SPGGLLCGVG VEGEAVQNGP ASHSRALVGI
551 CTGHSNPGED ARDGDAEEVR ELGTVEEN
```

Residue Number Increasing Mass Decreasing Mass

Start - End	Observed	Mr(expt)	Mr(calc)	Delta	Miss	Sequence
15 - 24	1148.6414	1147.6341	1147.6349	-0.0008	0	RPGNIYSTLK (No match)
32 - 39	1044.5017	1043.4944	1043.4923	0.0021	0	IDVSYEYR (No match)
40 - 58	1996.0287	1995.0214	1995.0312	-0.0098	0	FLEFTTLSAAELPGSSAVR (Ions score 26)

40 - 58	1996.0287	1995.0214	1995.0312	-0.0098	0	FLEFTTLSAAELPGSSAVR (No match)
93 - 102	1269.6876	1268.6803	1268.6877	-0.0073	1	EKTPLEHIFR (No match)
95 - 102	1012.5583	1011.5510	1011.5501	0.0009	0	TPLEHIFR (No match)
158 - 188	3135.5088	3134.5015	3134.4904	0.0112	0	FVGVIPQYHSSVNSAGSSAPVSTANSTEDAR (No match)
195 - 217	2325.0361	2324.0288	2324.0450	-0.0162	0	GDHASLENEKPGTGDVCSAPAGR (No match)
425 - 437	1626.9073	1625.9000	1625.9075	-0.0075	0	QIVFLQRPCLPQK (No match)
425 - 437	1626.9073	1625.9000	1625.9075	-0.0075	0	QIVFLQRPCLPQK (Ions score 15)
546 - 562	1753.8176	1752.8103	1752.8213	-0.0109	0	ALVGICTGHSNPGEDAR (No match)

Mascot: http://www.matrixscience.com/

Spot 194

{MATRIX}{SCIENCE} Mascot Search Results

Protein View

Match to: **gi|52145310** Score: **106** Expect: **4.8e-006**
hexose-6-phosphate dehydrogenase precursor [Homo sapiens]

Nominal mass (M_r): **89407;** Calculated pI value: **6.84**
NCBI BLAST search of gi|52145310 against nr
Unformatted sequence string for pasting into other applications

Taxonomy: Homo sapiens
Links to retrieve other entries containing this sequence from NCBI Entrez:
gi|108884809 from Homo sapiens
gi|66351381 from Homo sapiens
gi|119592019 from Homo sapiens

Fixed modifications: Carbamidomethyl (C)
Variable modifications: Oxidation (M)
Cleavage by Trypsin: cuts C-term side of KR unless next residue is P
Sequence Coverage: **19%**

Matched peptides shown in **Bold Red**

```
  1 MWNMLIVAMC LALLGCLQAQ ELQGHVSIIL LGATGDLAKK YLWQGLFQLY
 51 LDEAGRGHSF SFHGAALTAP KQGQELMAKA LESLSCPKDM APSHCAEHKD
101 QFLQLSQYRQ LKTAEDYQAL NKDIEAQLQH AGLREAGRIF YFSVPPFAYE
151 DIARNINSSC RPGPGAWLRV VLEKPFGHDH FSAQQLATEL GTFFQEEEMY
201 RVDHYLGKQA VAQILPFRDQ NRKALDGLWN RHHVERVEII MKETVDAEGR
251 TSFYEEYGVI RDVLQNHLTE VLTLVAMELP HNVSSAEAVL RHKLQVFQAL
301 RGLQRGSAVV GQYQSYSEQV RRELQKPDSF HSLTPTFAAV LVHIDNLRWE
351 GVPFILMSGK ALDERVGYAR ILFKNQACCV QSEKHWAAAQ SQCLPRQLVF
401 HIGHGDLGSP AVLVSRNLFR PSLPSSWKEM EGPPGLRLFG SPLSDYYAYS
451 PVRERDAHSV LLSHIFHGRK NFFITTENLL ASWNFWTPLL ESLAHKAPRL
501 YPGGAENGRL LDFEFSSGRL FFSQQQPEQL VPGPGPAPMP SDFQVLRAKY
551 RESPLVSAWS EELISKLAND IEATAVRAVR RFGQFHLALS GGSSPVALFQ
601 QLATAHYGFP WAHTHLWLVD ERCVPLSDPE SNFQGLQAHL LQHVRIPYYN
651 IHPMPVHLQQ RLCAEEDQGA QIYAREISAL VANSSFDLVL LGMGADGHTA
701 SLFPQSPTGL DGEQLVVLTT SPSQPHRRMS LSLPLINRAK KVAVLVMGRM
751 KREITTLVSR VGHEPKKWPI SGVLPHSGQL VWYMDYDAFL G
```

Residue Number Increasing Mass Decreasing Mass

Start - End	Observed	Mr(expt)	Mr(calc)	Delta	Miss	Sequence
113 - 134	2484.3779	2483.3706	2483.2404	0.1302	1	TAEDYQALNKDIEAQLQHAGLR (No match)
123 - 134	1350.7869	1349.7796	1349.7051	0.0745	0	DIEAQLQHAGLR (No match)
139 - 154	1935.0718	1934.0645	1933.9613	0.1032	0	IFYFSVPPFAYEDIAR (No match)
209 - 222	1655.9869	1654.9796	1654.8903	0.0893	1	QAVAQILPFRDQNR (No match)
292 - 301	1239.7987	1238.7914	1238.7247	0.0667	1	HKLQVFQALR (No match)
292 - 301	1239.7987	1238.7914	1238.7247	0.0667	1	HKLQVFQALR (No match)
294 - 301	974.6249	973.6176	973.5708	0.0468	0	LQVFQALR (No match)
306 - 321	1757.9454	1756.9381	1756.8379	0.1002	0	GSAVVGQYQSYSEQVR (No match)
306 - 322	1914.0562	1913.0489	1912.9390	0.1099	1	GSAVVGQYQSYSEQVRR (No match)
306 - 322	1914.0562	1913.0489	1912.9390	0.1099	1	GSAVVGQYQSYSEQVRR (No match)
349 - 360	1363.7288	1362.7215	1362.7005	0.0210	0	WEGVPFILMSGK (No match)
397 - 416	2102.2566	2101.2493	2101.1432	0.1062	0	QLVFHIGHGDLGSPAVLVSR (No match)
438 - 453	1834.9971	1833.9898	1833.8937	0.0961	0	LFGSPLSDYYAYSPVR (No match)
456 - 469	1588.9166	1587.9093	1587.8269	0.0824	0	DAHSVLLSHIFHGR (No match)
456 - 469	1588.9166	1587.9093	1587.8269	0.0824	0	DAHSVLLSHIFHGR (Ions score 1)
510 - 519	1170.6427	1169.6354	1169.5716	0.0638	0	LLDFEFSSGR (No match)
510 - 519	1170.6427	1169.6354	1169.5716	0.0638	0	LLDFEFSSGR (No match)

Mascot: http://www.matrixscience.com/

Spot 196

Mascot Search Results

Protein View

Match to: **gi|119606901** Score: **262** Expect: **1.2e-021**
phosphofructokinase, platelet, isoform CRA_a [Homo sapiens]

Nominal mass (M_r): **93725**; Calculated pI value: **8.64**
NCBI BLAST search of gi|119606901 against nr
Unformatted sequence string for pasting into other applications

Taxonomy: Homo sapiens

Fixed modifications: Carbamidomethyl (C)
Variable modifications: Oxidation (M)
Cleavage by Trypsin: cuts C-term side of KR unless next residue is P
Sequence Coverage: **26%**

Matched peptides shown in **Bold Red**

```
  1 MLLPTPAPAP APAPSRARAP SPAAPPIRSG PARHRPLALG PGGGAGGAAV
 51 PSQARAGRVP IACCAPGRAA PLGLLAMDAD DSRAPKGSLR KFLEHLSGAG
101 KAIGVLTSGG DAQGMNAAVR AVVRMGIYVG AKVYFIYEGY QGMVDGGSNI
151 AEADWESVSS ILQVGGTIIG SARCQAFRTR EGRLKAACNL LQRGITNLCV
201 IGGDGSLTGA NLFRKEWSGL LEELARNGQI DKEAVQKYAY LNVVGMVGSI
251 DNDFCGTDMT IGTDSALHRI IEVVDAIMTT AQSHQRTFVL EVMGRHCGYL
301 ALVSALACGA DWVFLPESPP EEGWEEQMCV KLSENRARKK RLNIIIVAEG
351 AIDTQNKPIT SEKIKELVVT QLGYDTRVTI LGHVQRGGTP SAFDRILASR
401 MGVEAVIALL EATPDTPACV VSLNGNHAVR LPLMECVQMT QDVQKAMDER
451 RFQDAVRLRG RSFAGNLNTY KRLAIKLPDD QIPKTNCNVA VINVGAPAAG
501 MNAAVRSAVR VGIADGHRML AIYDGFDGFA KGQIKEIGWT DVGGWTGQGG
551 SILGTKRVLP GKYLEEIATQ MRTHSINALL IIGGFEAYLG LLELSAAREK
601 HEEFCVPMVM VPATVSNNVP GSDFSIGADT ALNTITDTCD RIKQSASGTK
651 RRVFIIETMG GYCGYLANMG GLAAGADAAY IFEEPFDIRD LQSNVEHLTE
701 KMKTTIQRGL VLRNESCSEN YTTDFIYQLY SEEGKGVFDC RKNVLGHMQQ
751 GGAPSPFDRN FGTKISARAM EWITAKLKEA RGRGKKFTTD DSICVLGISK
801 RNVIFQPVAE LKKQTDFEHR IPKEQWWLKL RPLMKILAKY KASYDVSDSG
851 QLEHVQPWSV
```

Residue Number Increasing Mass Decreasing Mass

Start - End	Observed	Mr(expt)	Mr(calc)	Delta	Miss	Sequence
102 - 120	1787.9363	1786.9290	1786.8995	0.0295	0	AIGVLTSGGDAQGMNAAVR (No match)
186 - 193	945.5026	944.4953	944.4861	0.0092	0	AACNLLQR (No match)
194 - 214	2135.1172	2134.1099	2134.0840	0.0259	0	GITNLCVIGGDGSLTGANLFR (Ions score 20)
194 - 214	2135.1172	2134.1099	2134.0840	0.0259	0	GITNLCVIGGDGSLTGANLFR (No match)
194 - 215	2263.2112	2262.2039	2262.1789	0.0250	1	GITNLCVIGGDGSLTGANLFRK (No match)
216 - 226	1302.6893	1301.6820	1301.6615	0.0205	0	EWSGLLEELAR (No match)
270 - 286	1912.0214	1911.0141	1910.9884	0.0258	0	IIEVVDAIMTTAQSHQR (No match)
270 - 286	1912.0214	1911.0141	1910.9884	0.0258	0	IIEVVDAIMTTAQSHQR (Ions score 45)
270 - 286	1928.0229	1927.0156	1926.9833	0.0324	0	IIEVVDAIMTTAQSHQR Oxidation (M) (No match)
287 - 295	1067.5728	1066.5655	1066.5481	0.0175	0	TFVLEVMGR Oxidation (M) (No match)
364 - 377	1634.9357	1633.9284	1633.9039	0.0246	1	IKELVVTQLGYDTR (No match)
366 - 377	1393.7579	1392.7506	1392.7248	0.0258	0	ELVVTQLGYDTR (No match)
378 - 386	1022.6328	1021.6255	1021.6032	0.0223	0	VTILGHVQR (Ions score 7)
378 - 386	1022.6328	1021.6255	1021.6032	0.0223	0	VTILGHVQR (No match)
387 - 395	907.4474	906.4401	906.4195	0.0206	0	GGTPSAFDR (No match)
451 - 457	891.4943	890.4870	890.4722	0.0148	1	RFQDAVR (No match)
485 - 506	2170.1143	2169.1070	2169.0782	0.0288	0	TNCNVAVINVGAPAAGMNAAVR (No match)
485 - 506	2170.1143	2169.1070	2169.0782	0.0288	0	TNCNVAVINVGAPAAGMNAAVR (Ions score 12)
519 - 531	1447.7196	1446.7123	1446.6853	0.0271	0	MLAIYDGFDGFAK (No match)
563 - 572	1253.6433	1252.6360	1252.6121	0.0239	0	YLEEIATQMR (No match)
563 - 572	1269.6344	1268.6271	1268.6070	0.0201	0	YLEEIATQMR Oxidation (M) (No match)
736 - 742	881.4473	880.4400	880.4225	0.0176	1	GVFDCRK (No match)
743 - 759	1810.8954	1809.8881	1809.8580	0.0301	0	NVLGHMQQGGAPSPFDR (No match)
787 - 801	1711.9004	1710.8931	1710.8610	0.0321	1	FTTDDSICVLGISKR (No match)
802 - 813	1385.8385	1384.8312	1384.8078	0.0235	1	NVIFQPVAELKK (No match)
814 - 823	1270.6700	1269.6627	1269.6465	0.0162	1	QTDFEHRIPK (No match)

Mascot: http://www.matrixscience.com/

Spot 197

{MATRIX SCIENCE} Mascot Search Results

Protein View

```
Match to: gi|29791785 Score: 149 Expect: 2.4e-010
ERBB2IP protein [Homo sapiens]

Nominal mass (Mr): 72831; Calculated pI value: 4.80
NCBI BLAST search of gi|29791785 against nr
Unformatted sequence string for pasting into other applications

Taxonomy: Homo sapiens

Fixed modifications: Carbamidomethyl (C)
Variable modifications: Oxidation (M)
Cleavage by Trypsin: cuts C-term side of KR unless next residue is P
Sequence Coverage: 9%

Matched peptides shown in Bold Red

  1 MTTKRSLFVR LVPCRCLRGE EETVTTLDYS HCSLEQVPKE IFTFEKTLEE
 51 LYLDANQIEE LPKQLFNCQS LHKLSLPDND LTTLPASIAN LINLRELDVS
101 KNGIQEFPEN IKNCKVLTIV EASVNPISKL PDGFSQLLNL TQLYLNDAFL
151 EFLPANFGRL TKLQILELRE NQLKMLPKTM NRLTQLERLD LGSNEFTEVP
201 EVLEQLSGLK EFWMDANRLT FIPGFIGSLK QLTYLDVSKN NIEMVEEGIS
251 TCENLQDLLL SSNSLQQLPE TIGSLKNITT LKIDENQLMY LPDSIGGLIS
301 VEELDCSFNE VEALPSSIGQ LTNLRTFAAD HNYLQQLPPE IGSWKNITVL
351 FLHSNKLETL PEEMGDMQKL KVINLSDNRL KNLPFSFTKL QQLTAMWLSD
401 NQSKPLIPLQ KETDSETQKM VLTNYMFPQQ PRTEDVMFIS DNESFNPSLW
451 EEQRKQRAQV AFECDEDKDE REAPPREGNL KRYPTPYPDE LKNMVKTVQT
501 IVHRLKDEET NEDSGRDLKP HEDQQDINKD TSESTTTVKS KVDEREKYMI
551 GNSVQKISEP EAEISPGSLP VTANMKASEN LKHIVNHDDV FEESEELSSD
601 EEMKMAEMRP PLIETSINQP KVVALSNNKK KKK
```

Residue Number Increasing Mass Decreasing Mass

Start - End	Observed	Mr(expt)	Mr(calc)	Delta	Miss	Sequence	
102 - 112	1288.6240	1287.6167	1287.6458	-0.0291	0	NGIQEFPENIK	(No match)
102 - 112	1288.6240	1287.6167	1287.6458	-0.0291	0	NGIQEFPENIK	(Ions score 10)

420 - 432	1656.7614	1655.7541	1655.7799	-0.0258	0	MVLTNYMFPQQPR	2 Oxidation (M) (Ions score 6)
420 - 432	1656.7614	1655.7541	1655.7799	-0.0258	0	MVLTNYMFPQQPR	2 Oxidation (M) (No match)
458 - 471	1711.7056	1710.6983	1710.7155	-0.0171	1	AQVAFECDEDKDER	(Ions score 40)
458 - 471	1711.7056	1710.6983	1710.7155	-0.0171	1	AQVAFECDEDKDER	(No match)
482 - 492	1378.6763	1377.6690	1377.6928	-0.0238	1	RYPTPYPDELK	(No match)
505 - 516	1392.6021	1391.5948	1391.6164	-0.0216	1	LKDEETNEDSGR	(Ions score 37)
505 - 516	1392.6021	1391.5948	1391.6164	-0.0216	1	LKDEETNEDSGR	(No match)

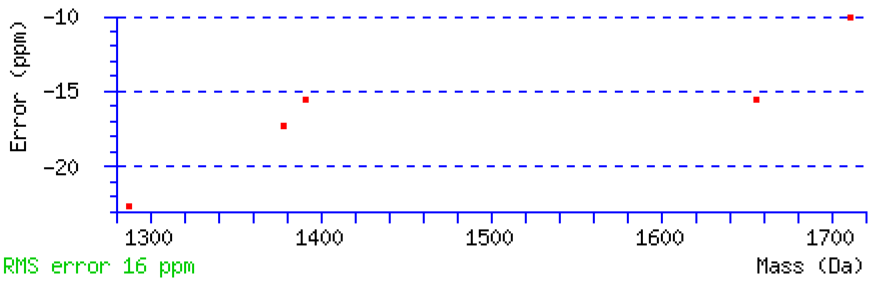

Mascot: http://www.matrixscience.com/

Spot 199

Mascot Search Results

Protein View

Match to: **gi|12669909** Score: **232** Expect: **1.2e-018**
acyl-CoA synthetase long-chain family member 4 isoform 2 [Homo sapiens]

Nominal mass (M_r): **80220**; Calculated pI value: **8.66**
NCBI BLAST search of gi|12669909 against nr
Unformatted sequence string for pasting into other applications

Taxonomy: Homo sapiens
Links to retrieve other entries containing this sequence from NCBI Entrez:
gi|13432172 from Homo sapiens
gi|119623076 from Homo sapiens
gi|119623078 from Homo sapiens

Fixed modifications: Carbamidomethyl (C)
Variable modifications: Oxidation (M)
Cleavage by Trypsin: cuts C-term side of KR unless next residue is P
Sequence Coverage: **31%**

Matched peptides shown in **Bold Red**

```
  1 MKLKLNVLTI ILLPVHLLIT IYSALIFIPW YFLTNAKKKN AMAKRIKAKP
 51 TSDKPGSPYR SVTHFDSLAV IDIPGADTLD KLFDHAVSKF GKKDSLGTRE
101 ILSEENEMQP NGKVFKKLIL GNYKWMNYLE VNRRVNNFGS GLTALGLKPK
151 NTIAIFCETR AEWMIAAQTC FKYNFPLVTL YATLGKEAVV HGLNESEASY
201 LITSVELLES KLKTALLDIS CVKHIIYVDN KAINKAEYPE GFEIHSMQSV
251 EELGSNPENL GIPPSRPTPS DMAIVMYTSG STGRPKGVMM HHSNLIAGMT
301 GQCERIPGLG PKDTYIGYLP LAHVLELTAE ISCFTYGCRI GYSSPLTLSD
351 QSSKIKKGSK GDCTVLKPTL MAAVPEIMDR IYKNVMSKVQ EMNYIQKTLF
401 KIGYDYKLEQ IKKGYDAPLC NLLLFKKVKA LLGGNVRMML SGGAPLSPQT
451 HRFMNVCFCC PIGQGYGLTE SCGAGTVTEV TDYTTGRVGA PLICCEIKLK
501 DWQEGGYTIN DKPNPRGEIV IGGQNISMGY FKNEEKTAED YSVDENGQRW
551 FCTGDIGEFH PDGCLQIIDR KKDLVKLQAG EYVSLGKVEA ALKNCPLIDN
601 ICAFAKSDQS YVISFVVPNQ KRLTLLAQQK GVEGTWVDIC NNPAMEAEIL
651 KEIREAANAM KLERFEIPIK VRLSPEPWTP ETGLVTDAFK LKRKELRNHY
701 LKDIERMYGG K
```

Residue Number Increasing Mass Decreasing Mass

Start - End	Observed	Mr(expt)	Mr(calc)	Delta	Miss	Sequence
48 - 60	1403.7582	1402.7509	1402.7204	0.0305	0	AKPTSDKPGSPYR (No match)
125 - 133	1240.6069	1239.5996	1239.5706	0.0290	0	WMNYLEVNR Oxidation (M) (No match)
134 - 150	1772.0529	1771.0456	1771.0103	0.0353	1	RVNNFGSGLTALGLKPK (No match)
135 - 150	1615.9449	1614.9376	1614.9092	0.0284	0	VNNFGSGLTALGLKPK (No match)
151 - 160	1224.6265	1223.6192	1223.5968	0.0224	0	NTIAIFCETR (Ions score 24)
151 - 160	1224.6265	1223.6192	1223.5968	0.0224	0	NTIAIFCETR (No match)
173 - 186	1599.8715	1598.8642	1598.8708	-0.0065	0	YNFPLVTLYATLGK (No match)
340 - 354	1582.8149	1581.8076	1581.7886	0.0191	0	IGYSSPLTLSDQSSK (No match)
361 - 380	2217.1450	2216.1377	2216.1003	0.0374	0	GDCTVLKPTLMAAVPEIMDR (No match)
414 - 427	1651.9237	1650.9164	1650.8803	0.0361	1	GYDAPLCNLLLFKK (No match)
438 - 452	1598.8101	1597.8028	1597.7704	0.0324	0	MMLSGGAPLSPQTHR Oxidation (M) (No match)
438 - 452	1598.8101	1597.8028	1597.7704	0.0324	0	MMLSGGAPLSPQTHR Oxidation (M) (Ions score 5)
488 - 498	1259.6716	1258.6643	1258.6413	0.0230	0	VGAPLICCEIK (No match)
499 - 516	2131.0745	2130.0672	2130.0493	0.0179	1	LKDWQEGGYTINDKPNPR (No match)
517 - 532	1712.9030	1711.8957	1711.8602	0.0355	0	GEIVIGGQNISMGYFK (No match)
577 - 593	1776.0251	1775.0178	1774.9828	0.0350	1	LQAGEYVSLGKVEAALK (No match)
594 - 606	1535.7650	1534.7577	1534.7272	0.0306	0	NCPLIDNICAFAK (No match)
607 - 621	1710.9060	1709.8987	1709.8624	0.0363	0	SDQSYVISFVVPNQK (No match)
607 - 621	1710.9060	1709.8987	1709.8624	0.0363	0	SDQSYVISFVVPNQK (Ions score 10)
607 - 622	1867.0049	1865.9976	1865.9635	0.0341	1	SDQSYVISFVVPNQKR (No match)
607 - 622	1867.0049	1865.9976	1865.9635	0.0341	1	SDQSYVISFVVPNQKR (Ions score 26)
665 - 672	1001.6289	1000.6216	1000.6069	0.0147	1	FEIPIKVR (No match)

Mascot: http://www.matrixscience.com/

Spot 202

Mascot Search Results

Protein View

Match to: **gi|22477159** Score: **91** Expect: **0.00016**
Sec23 homolog A (S. cerevisiae) [Homo sapiens]

Nominal mass (M_r): **87022**; Calculated pI value: **6.64**
NCBI BLAST search of gi|22477159 against nr
Unformatted sequence string for pasting into other applications

Taxonomy: Homo sapiens
Links to retrieve other entries containing this sequence from NCBI Entrez:
gi|123979998 from synthetic construct
gi|123993343 from synthetic construct
gi|123994759 from synthetic construct

Fixed modifications: Carbamidomethyl (C)
Variable modifications: Oxidation (M)
Cleavage by Trypsin: cuts C-term side of KR unless next residue is P
Sequence Coverage: **14%**

Matched peptides shown in **Bold Red**

```
  1 MTTYLEFIQQ NEERDGVRFS WNVWPSSRLE ATRMVVPVAA LFTPLKERPD
 51 LPPIQYEPVL CSRTTCRAVL NPLCQVDYRA KLWACNFCYQ RNQFPPSYAG
101 ISELNQPAEL LPQFSSIEYV VLRGPQMPLI FLYVVDTCME DEDLQALKES
151 MQMSLSLLPP TALVGLITFG RMVQVHELGC EGISKSYVFR GTKDLSAKQL
201 QEMLGLSKVP VTQATRGPQV QQPPPSNRFL QPVQKIDMNL TDLLGELQRD
251 PWPVPQGKRP LRSSGVALSI AVGLLECTFP NTGARIMMFI GGPATQGPGM
301 VVGDELKTPI RSWHDIDKDN AKYVKKGTKH FEALANRAAT TGHVIDIYAC
351 ALDQTGLLEM KCCPNLTGGY MVMGDSFNTS LFKQTFQRVF TKDMHGQFKM
401 GFGGTLEIKT SREIKISGAI GPCVSLNSKG PCVSENEIGT GGTCQWKICG
451 LSPTTTLAIY FEVVNQHNAP IPQGGRGAIQ FVTQYQHSSG QRRIRVTTIA
501 RNWADAQTQI QNIAASFDQE AAAILMARLA IYRAETEEGP DVLRWLDRQL
551 IRLCQKFGEY HKDDPSSFRF SETFSLYPQF MFHLRRSSFL QVFNNSPDES
601 SYYRHHFMRQ DLTQSLIMIQ PIMYAYSFSG PPEPVLLDSS SILADRILLM
651 DTFFQILIYH GETIAQWRKS GYQDMPEYEN FRHLLQAPVD DAQEILHSRF
701 PMPRYIDTEH GGSQARFLLS KVNPSQTHNN MYAWGQESGA PILTDDVSLQ
751 VFMDHLKKLA VSSAA
```

Residue Number Increasing Mass Decreasing Mass

Start - End	Observed	Mr(expt)	Mr(calc)	Delta	Miss	Sequence
68 - 79	1447.8292	1446.8219	1446.7289	0.0930	0	AVLNPLCQVDYR (No match)
217 - 228	1304.7523	1303.7450	1303.6633	0.0818	0	GPQVQQPPPSNR (No match)
217 - 228	1304.7523	1303.7450	1303.6633	0.0818	0	GPQVQQPPPSNR (No match)
477 - 492	1806.9983	1805.9910	1805.8808	0.1102	0	GAIQFVTQYQHSSGQR (Ions score 12)
477 - 492	1806.9983	1805.9910	1805.8808	0.1102	0	GAIQFVTQYQHSSGQR (No match)
557 - 569	1584.8094	1583.8021	1583.7004	0.1017	1	FGEYHKDDPSSFR (No match)
587 - 604	2140.1079	2139.1006	2138.9544	0.1462	0	SSFLQVFNNSPDESSYYR (Ions score 20)
587 - 604	2140.1079	2139.1006	2138.9544	0.1462	0	SSFLQVFNNSPDESSYYR (No match)
670 - 682	1651.7781	1650.7708	1650.6620	0.1089	0	SGYQDMPEYENFR Oxidation (M) (No match)
683 - 699	1942.1217	1941.1144	1941.0068	0.1076	0	HLLQAPVDDAQEILHSR (No match)
705 - 716	1333.7015	1332.6942	1332.6058	0.0884	0	YIDTEHGGSQAR (No match)

Mascot: http://www.matrixscience.com/

Spot 203

MATRIX SCIENCE Mascot Search Results

Protein View

Match to: **gi|556514** Score: **178** Expect: **3e-013**
acylamino acid-releasing enzyme [Homo sapiens]

Nominal mass (M_r): **82210**; Calculated pI value: **5.29**
NCBI BLAST search of gi|556514 against nr
Unformatted sequence string for pasting into other applications

Taxonomy: Homo sapiens

Fixed modifications: Carbamidomethyl (C)
Variable modifications: Oxidation (M)
Cleavage by Trypsin: cuts C-term side of KR unless next residue is P
Sequence Coverage: **18%**

Matched peptides shown in **Bold Red**

1 MER**QVLLSEP EEAAALYR**GL SR**QPALSAAC LGPEVTTQYG GQYR**TVHTEW
51 TQRDLERMEN IRFCRQYLVF HDGDSVVFAG PAGNSVETRG ELLSRESPSG
101 SMKAVLRKAG GTGPGEEKQF LEVWEKNRKL KSFNLSVLEK HGPVYEDDCF
151 GCLSWSHSET HLLYVAERKR PKAESFFQTK **ALDVSASDDE IAR**LKKPDQP
201 IKGDQFVFYE DWGENMVSKS IPVLCVLDVE SGNISVLEGV PENVSPGQAF
251 WAPGDAGVVF VGWWHEPFRL GIRFCTNRR**S ALYYVDLIGG K**CELLSDDSL
301 AVSSPRLSPD QCRIVYLQYP SLIPHHQCSQ LCLYDWYTK**V TSVVVDVVPR**
351 QLGENFSGIY CSLLPLGCWS ADSQR**VVFDS AQR**SRQDLFA VDTQVGTVTS
401 LTAGGSGGSW KLLTIDQDLM VAQFSTPSLP PTLKVGFLPS AGKEQSVLWV
451 SLEEAEPIPD IHWGIRVLQP PPEQENVQYA GLDFEAILLQ PGSPPDKTQV
501 PMVVMPHGGP HSSFVTAWML FPAMLCK**MGF AVLLVNYRGS TGFGQDSILS**
551 **LPGNVGHQDV K**DVQFAVEQV LQEEHFDASH VALMGGSHGG FISCHLIGQY
601 PETYRACVAR NPVINIASML GSTDIPDWCV VEAGFPFSSD CLPDLSVWAE
651 MLDKSPIRYI PQVK**TPLLLM LGQEDR**RVPF K**QGMEYYR**AL KTRNVPVRLL
701 LYPKSTHALS EVEVESDSFM NAVLWLRTHL GS

Residue Number Increasing Mass Decreasing Mass

Start - End	Observed	Mr(expt)	Mr(calc)	Delta	Miss	Sequence

4 - 18	1688.8582	1687.8509	1687.8780	-0.0271	0	QVLLSEPEEAAALYR (Ions score 40)
4 - 18	1688.8582	1687.8509	1687.8780	-0.0271	0	QVLLSEPEEAAALYR (No match)
23 - 44	2367.0935	2366.0862	2366.1324	-0.0462	0	QPALSAACLGPEVTTQYGGQYR (No match)
23 - 44	2367.0935	2366.0862	2366.1324	-0.0462	0	QPALSAACLGPEVTTQYGGQYR (Ions score 62)
181 - 193	1361.6404	1360.6331	1360.6470	-0.0139	0	ALDVSASDDEIAR (No match)
280 - 291	1298.6705	1297.6632	1297.6917	-0.0285	0	SALYYVDLIGGK (No match)
340 - 350	1169.6746	1168.6673	1168.6815	-0.0142	0	VTSVVVDVVPR (No match)
376 - 383	921.4692	920.4619	920.4715	-0.0096	0	VVFDSAQR (No match)
528 - 538	1298.6705	1297.6632	1297.6852	-0.0220	0	MGFAVLLVNYR Oxidation (M) (No match)
539 - 561	2313.1133	2312.1060	2312.1396	-0.0336	0	GSTGFGQDSILSLPGNVGHQDVK (No match)
665 - 676	1401.7163	1400.7090	1400.7333	-0.0243	0	TPLLLMLGQEDR Oxidation (M) (No match)
665 - 676	1401.7163	1400.7090	1400.7333	-0.0243	0	TPLLLMLGQEDR Oxidation (M) (Ions score 1)
682 - 688	962.3887	961.3814	961.3963	-0.0149	0	QGMEYYR Oxidation (M) (No match)

Mascot: http://www.matrixscience.com/

Spot 206

MATRIX SCIENCE Mascot Search Results

Protein View

Match to: **gi|46249758** Score: **387** Expect: **3.8e-034**
Ezrin [Homo sapiens]

Nominal mass (M_r): **69313**; Calculated pI value: **5.94**
NCBI BLAST search of gi|46249758 against nr
Unformatted sequence string for pasting into other applications

Taxonomy: Homo sapiens

Fixed modifications: Carbamidomethyl (C)
Variable modifications: Oxidation (M)
Cleavage by Trypsin: cuts C-term side of KR unless next residue is P
Sequence Coverage: **41%**

Matched peptides shown in **Bold Red**

```
  1 MPKPINVRVT TMDAELEFAI QPNTTGKQLF DQVVKTIGLR EVWYFGLHYV
 51 DNKGFPTWLK LDKKVSAQEV RKENPLQFKF RAKFYPEDVA EELIQDITQK
101 LFFLQVKEGI LSDEIYCPPE TAVLLGSYAV QAKFGDYNKE VHKSGYLSSE
151 RLIPQRVMDQ HKLTRDQWED RIQVWHAEHR GMLKDNAMLE YLKIAQDLEM
201 YGINYFEIKN KKGTDLWLGV DALGLNIYEK DDKLTPKIGF PWSEIRNISF
251 NDKKFVIKPI DKKAPDFVFY APRLRINKRI LQLCMGNHEL YMRRRKPDTI
301 EVQQMKAQAR EEKHQKQLER QQLETEKKRR ETVEREKEQM MREKEELMLR
351 LQDYEEKTKK AERELSEQIQ RALQLEEERK RAQEEAERLE ADRMAALRAK
401 EELERQAVDQ IKSQEQLAAE LAEYTAKIAL LEEARRRKED EVEEWQHRAK
451 EAQDDLVKTK EELHLVMTAP PPPPPPVYEP VSYHVQESLQ DEGAEPTGYS
501 AGLSSEGIRD DGNEEKRITE AEKNERVQRQ LLTLSSELSQ ARDENKRTHN
551 DIIHNENMRQ GRDKYKTLRQ IRQGNTKQRI DEFEAL
```

Residue Number Increasing Mass Decreasing Mass

Start - End	Observed	Mr(expt)	Mr(calc)	Delta	Miss	Sequence
2 - 8	823.5565	822.5492	822.5075	0.0417	0	PKPINVR (No match)
9 - 27	2082.1326	2081.1253	2080.9986	0.1267	0	VTTMDAELEFAIQPNTTGK Oxidation (M) (No match)
28 - 35	976.5887	975.5814	975.5389	0.0425	0	QLFDQVVK (No match)

54 - 63	1204.6450	1203.6377	1203.6651	-0.0274	1	GFPTWLKLDK (No match)
65 - 72	916.5742	915.5669	915.5137	0.0532	1	VSAQEVRK (No match)
101 - 107	894.5862	893.5789	893.5374	0.0415	0	LFFLQVK (No match)
144 - 151	898.4758	897.4685	897.4192	0.0494	0	SGYLSSER (No match)
194 - 209	1963.0725	1962.0652	1961.9444	0.1208	0	IAQDLEMYGINYFEIK Oxidation (M) (No match)
238 - 246	1104.6416	1103.6343	1103.5763	0.0580	0	IGFPWSEIR (No match)
238 - 246	1104.6416	1103.6343	1103.5763	0.0580	0	IGFPWSEIR (Ions score 35)
247 - 254	965.5527	964.5454	964.4977	0.0477	1	NISFNDKK (No match)
255 - 262	959.5705	958.5632	958.5851	-0.0219	0	FVIKPIDK (No match)
263 - 273	1310.7650	1309.7577	1309.6818	0.0759	1	KAPDFVFYAPR (Ions score 58)
263 - 273	1310.7650	1309.7577	1309.6818	0.0759	1	KAPDFVFYAPR (No match)
264 - 273	1182.6613	1181.6540	1181.5869	0.0671	0	APDFVFYAPR (Ions score 75)
264 - 273	1182.6613	1181.6540	1181.5869	0.0671	0	APDFVFYAPR (No match)
295 - 306	1488.8677	1487.8604	1487.7766	0.0839	1	RKPDTIEVQQMK Oxidation (M) (No match)
296 - 306	1332.7434	1331.7361	1331.6755	0.0607	0	KPDTIEVQQMK Oxidation (M) (No match)
343 - 350	1063.5990	1062.5917	1062.5379	0.0538	1	EKEELMLR Oxidation (M) (No match)
364 - 371	1002.5753	1001.5680	1001.5141	0.0539	0	ELSEQIQR (No match)
372 - 379	987.5635	986.5562	986.5032	0.0530	0	ALQLEEER (No match)
382 - 388	832.4328	831.4255	831.3722	0.0533	0	AQEEAER (No match)
382 - 393	1416.7526	1415.7453	1415.6640	0.0813	1	AQEEAERLEADR (No match)
399 - 405	874.5086	873.5013	873.4555	0.0458	1	AKEELER (No match)
413 - 427	1651.9137	1650.9064	1650.8100	0.0964	0	SQEQLAAELAEYTAK (No match)
428 - 435	914.5789	913.5716	913.5232	0.0484	0	IALLEEAR (No match)
428 - 436	1070.6886	1069.6813	1069.6243	0.0570	1	IALLEEARR (No match)
518 - 526	1089.6145	1088.6072	1088.5461	0.0611	1	ITEAEKNER (No match)
530 - 542	1445.8805	1444.8732	1444.7885	0.0847	0	QLLTLSSELSQAR (No match)
548 - 559	1509.7742	1508.7669	1508.6790	0.0879	0	THNDIIHNENMR Oxidation (M) (No match)
578 - 586	1120.6321	1119.6248	1119.5560	0.0688	1	QRIDEFEAL (Ions score 7)
578 - 586	1120.6321	1119.6248	1119.5560	0.0688	1	QRIDEFEAL (No match)

Mascot: http://www.matrixscience.com/

Spot 207

Mascot Search Results

Protein View

Match to: **gi|46249758** Score: **430** Expect: **1.9e-038**
Ezrin [Homo sapiens]

Nominal mass (M_r): **69313**; Calculated pI value: **5.94**
NCBI BLAST search of gi|46249758 against nr
Unformatted sequence string for pasting into other applications

Taxonomy: Homo sapiens

Fixed modifications: Carbamidomethyl (C)
Variable modifications: Oxidation (M)
Cleavage by Trypsin: cuts C-term side of KR unless next residue is P
Sequence Coverage: **40%**

Matched peptides shown in **Bold Red**

```
  1 MPKPINVRVT TMDAELEFAI QPNTTGKQLF DQVVKTIGLR EVWYFGLHYV
 51 DNKGFPTWLK LDKKVSAQEV RKENPLQFKF RAKFYPEDVA EELIQDITQK
101 LFFLQVKEGI LSDEIYCPPE TAVLLGSYAV QAKFGDYNKE VHKSGYLSSE
151 RLIPQRVMDQ HKLTRDQWED RIQVWHAEHR GMLKDNAMLE YLKIAQDLEM
201 YGINYFEIKN KKGTDLWLGV DALGLNIYEK DDKLTPKIGF PWSEIRNISF
251 NDKKFVIKPI DKKAPDFVFY APRLRINKRI LQLCMGNHEL YMRRRKPDTI
301 EVQQMKAQAR EEKHQKQLER QQLETEKKRR ETVEREKEQM MREKEELMLR
351 LQDYEEKTKK AERELSEQIQ RALQLEEERK RAQEEAERLE ADRMAALRAK
401 EELERQAVDQ IKSQEQLAAE LAEYTAKIAL LEEARRRKED EVEEWQHRAK
451 EAQDDLVKTK EELHLVMTAP PPPPPPVYEP VSYHVQESLQ DEGAEPTGYS
501 AGLSSEGIRD DGNEEKRITE AEKNERVQRQ LLTLSSELSQ ARDENKRTHN
551 DIIHNENMRQ GRDKYKTLRQ IRQGNTKQRI DEFEAL
```

Residue Number Increasing Mass Decreasing Mass

Start - End	Observed	Mr(expt)	Mr(calc)	Delta	Miss	Sequence
2 - 8	823.5357	822.5284	822.5075	0.0209	0	PKPINVR (No match)
28 - 35	976.5601	975.5528	975.5389	0.0139	0	QLFDQVVK (No match)
54 - 60	848.4498	847.4425	847.4592	-0.0167	0	GFPTWLK (No match)

65 - 72	916.5472	915.5399	915.5137	0.0262	1	**VSAQEVRK** (No match)
72 - 79	1003.5572	1002.5499	1002.5498	0.0001	1	**KENPLQFK** (No match)
101 - 107	894.5636	893.5563	893.5374	0.0189	0	**LFFLQVK** (No match)
134 - 143	1236.5461	1235.5388	1235.5934	-0.0546	1	**FGDYNKEVHK** (No match)
144 - 151	898.4495	897.4422	897.4192	0.0231	0	**SGYLSSER** (No match)
172 - 180	1175.6373	1174.6300	1174.5995	0.0305	0	**IQVWHAEHR** (No match)
172 - 180	1175.6373	1174.6300	1174.5995	0.0305	0	**IQVWHAEHR** (Ions score 5)
238 - 246	1104.6038	1103.5965	1103.5763	0.0202	0	**IGFPWSEIR** (Ions score 34)
238 - 246	1104.6038	1103.5965	1103.5763	0.0202	0	**IGFPWSEIR** (No match)
255 - 262	959.5500	958.5427	958.5851	-0.0424	0	**FVIKPIDK** (No match)
263 - 273	1310.7170	1309.7097	1309.6818	0.0279	1	**KAPDFVFYAPR** (No match)
263 - 273	1310.7170	1309.7097	1309.6818	0.0279	1	**KAPDFVFYAPR** (Ions score 42)
264 - 273	1182.6199	1181.6126	1181.5869	0.0257	0	**APDFVFYAPR** (Ions score 68)
264 - 273	1182.6199	1181.6126	1181.5869	0.0257	0	**APDFVFYAPR** (No match)
280 - 293	1777.8905	1776.8832	1776.8473	0.0359	0	**ILQLCMGNHELYMR** (No match)
280 - 293	1793.8915	1792.8842	1792.8422	0.0420	0	**ILQLCMGNHELYMR** Oxidation (M) (No match)
295 - 306	1472.8204	1471.8131	1471.7817	0.0315	1	**RKPDTIEVQQMK** (No match)
295 - 306	1488.8090	1487.8017	1487.7766	0.0252	1	**RKPDTIEVQQMK** Oxidation (M) (No match)
343 - 350	1047.5737	1046.5664	1046.5430	0.0234	1	**EKEELMLR** (No match)
364 - 371	1002.5446	1001.5373	1001.5141	0.0232	0	**ELSEQIQR** (No match)
372 - 379	987.5338	986.5265	986.5032	0.0233	0	**ALQLEEER** (No match)
382 - 388	832.4041	831.3968	831.3722	0.0246	0	**AQEEAER** (No match)
382 - 393	1416.7056	1415.6983	1415.6640	0.0343	1	**AQEEAERLEADR** (No match)
399 - 405	874.4820	873.4747	873.4555	0.0192	1	**AKEELER** (No match)
413 - 427	1651.8538	1650.8465	1650.8100	0.0365	0	**SQEQLAAELAEYTAK** (No match)
428 - 435	914.5527	913.5454	913.5232	0.0222	0	**IALLEEAR** (No match)
428 - 436	1070.6547	1069.6474	1069.6243	0.0231	1	**IALLEEARR** (No match)
518 - 526	1089.5800	1088.5727	1088.5461	0.0266	1	**ITEAEKNER** (No match)
530 - 542	1445.8275	1444.8202	1444.7885	0.0317	0	**QLLTLSSELSQAR** (No match)
548 - 559	1493.7255	1492.7182	1492.6841	0.0341	0	**THNDIIHNENMR** (No match)
548 - 559	1509.7201	1508.7128	1508.6790	0.0338	0	**THNDIIHNENMR** Oxidation (M) (No match)
578 - 586	1120.5908	1119.5835	1119.5560	0.0275	1	**QRIDEFEAL** (Ions score 12)
578 - 586	1120.5908	1119.5835	1119.5560	0.0275	1	**QRIDEFEAL** (No match)

Mascot: http://www.matrixscience.com/

Spot 208

MATRIX SCIENCE Mascot Search Results

Protein View

Match to: **gi|46249758** Score: **569** Expect: **2.4e-052**
Ezrin [Homo sapiens]

Nominal mass (M_r): **69313**; Calculated pI value: **5.94**
NCBI BLAST search of gi|46249758 against nr
Unformatted sequence string for pasting into other applications

Taxonomy: Homo sapiens

Fixed modifications: Carbamidomethyl (C)
Variable modifications: Oxidation (M)
Cleavage by Trypsin: cuts C-term side of KR unless next residue is P
Sequence Coverage: **42%**

Matched peptides shown in **Bold Red**

1 M**PKPINVR**VT TMDAELEFAI QPNTTGK**QLF** **DQVVK**TIGLR EVWYFGLHYV
51 DNK**GFPTWLK** LDKKVSAQEV RKENPLQFKF RAKFYPEDVA EELIQDITQK
101 **LFFLQVK**EGI LSDEIYCPPE TAVLLGSYAV QAK**FGDYNKE** **VHKSGYLSSE**
151 **R**LIPQRVMDQ HKLTR**DQWED** **RIQVWHAEHR** GMLKDNAMLE YLK**IAQDLEM**
201 **YGINYFEIK**N KKGTDLWLGV DALGLNIYEK DDKLTPK**IGF** **PWSEIR**NISF
251 NDKK**FVIKPI** **DKKAPDFVFY** **APR**LRINKR**I** **LQLCMGNHEL** **YMR**R**RKPDTI**
301 **EVQQMK**AQAR EEKHQKQLER QQLETEKKRR ETVEREK**EQM** **MREKEELMLR**
351 LQDYEEKTKK AER**ELSEQIQ** **RALQLEEER**K R**AQEEAERLE** **ADR**MAALR**AK**
401 **EELER**QAVDQ IK**SQEQLAAE** **LAEYTAKIAL** **LEEARR**RKED EVEEWQHRAK
451 EAQDDLVKTK EELHLVMTAP PPPPPPVYEP VSYHVQESLQ DEGAEPTGYS
501 AGLSSEGIRD DGNEEKR**ITE** **AEKNER**VQR**Q** **LLTLSSELSQ** **AR**DENKR**THN**
551 **DIIHNENMR**Q GRDKYKTLRQ IRQGNTK**QRI** **DEFEAL**

Residue Number Increasing Mass Decreasing Mass

Start - End	Observed	Mr(expt)	Mr(calc)	Delta	Miss	Sequence
2 - 8	823.5201	822.5128	822.5075	0.0053	0	PKPINVR (No match)
28 - 35	976.5505	975.5432	975.5389	0.0043	0	QLFDQVVK (No match)
54 - 60	848.4648	847.4575	847.4592	-0.0017	0	GFPTWLK (No match)

101 - 107	894.5544	893.5471	893.5374	0.0097	0	LFFLQVK (No match)
134 - 143	1236.5670	1235.5597	1235.5934	-0.0337	1	FGDYNKEVHK (No match)
144 - 151	898.4379	897.4306	897.4192	0.0115	0	SGYLSSER (No match)
166 - 171	848.3687	847.3614	847.3460	0.0154	0	DQWEDR (No match)
172 - 180	1175.6213	1174.6140	1174.5995	0.0145	0	IQVWHAEHR (No match)
172 - 180	1175.6213	1174.6140	1174.5995	0.0145	0	IQVWHAEHR (Ions score 13)
194 - 209	1946.9880	1945.9807	1945.9495	0.0312	0	IAQDLEMYGINYFEIK (No match)
238 - 246	1104.5925	1103.5852	1103.5763	0.0089	0	IGFPWSEIR (Ions score 43)
238 - 246	1104.5925	1103.5852	1103.5763	0.0089	0	IGFPWSEIR (No match)
255 - 262	959.5548	958.5475	958.5851	-0.0376	0	FVIKPIDK (No match)
263 - 273	1310.7034	1309.6961	1309.6818	0.0143	1	KAPDFVFYAPR (No match)
263 - 273	1310.7034	1309.6961	1309.6818	0.0143	1	KAPDFVFYAPR (Ions score 95)
264 - 273	1182.6066	1181.5993	1181.5869	0.0124	0	APDFVFYAPR (Ions score 60)
264 - 273	1182.6066	1181.5993	1181.5869	0.0124	0	APDFVFYAPR (No match)
280 - 293	1777.8750	1776.8677	1776.8473	0.0204	0	ILQLCMGNHELYMR (No match)
280 - 293	1793.8712	1792.8639	1792.8422	0.0217	0	ILQLCMGNHELYMR Oxidation (M) (No match)
295 - 306	1472.8081	1471.8008	1471.7817	0.0192	1	RKPDTIEVQQMK (No match)
295 - 306	1488.8005	1487.7932	1487.7766	0.0167	1	RKPDTIEVQQMK Oxidation (M) (No match)
338 - 344	951.4492	950.4419	950.4314	0.0106	1	EQMMREK (No match)
343 - 350	1047.5598	1046.5525	1046.5430	0.0095	1	EKEELMLR (No match)
364 - 371	1002.5323	1001.5250	1001.5141	0.0109	0	ELSEQIQR (No match)
372 - 379	987.5205	986.5132	986.5032	0.0100	0	ALQLEEER (No match)
382 - 388	832.3983	831.3910	831.3722	0.0188	0	AQEEAER (No match)
382 - 393	1416.6849	1415.6776	1415.6640	0.0136	1	AQEEAERLEADR (No match)
399 - 405	874.4733	873.4660	873.4555	0.0105	1	AKEELER (No match)
413 - 427	1651.8344	1650.8271	1650.8100	0.0171	0	SQEQLAAELAEYTAK (No match)
428 - 435	914.5399	913.5326	913.5232	0.0094	0	IALLEEAR (No match)
428 - 436	1070.6414	1069.6341	1069.6243	0.0098	1	IALLEEARR (No match)
518 - 526	1089.5649	1088.5576	1088.5461	0.0115	1	ITEAEKNER (No match)
530 - 542	1445.8125	1444.8052	1444.7885	0.0167	0	QLLTLSSELSQAR (No match)
548 - 559	1493.7115	1492.7042	1492.6841	0.0201	0	THNDIIHNENMR (No match)
548 - 559	1493.7115	1492.7042	1492.6841	0.0201	0	THNDIIHNENMR (Ions score 77)
548 - 559	1509.7046	1508.6973	1508.6790	0.0183	0	THNDIIHNENMR Oxidation (M) (No match)
578 - 586	1120.5800	1119.5727	1119.5560	0.0167	1	QRIDEFEAL (No match)

Mascot: http://www.matrixscience.com/

Spot 209

{MATRIX}{SCIENCE} **Mascot Search Results**

Protein View

Match to: **gi|27262655** Score: **299** Expect: **2.4e-025**
interleukin 16 isoform 1 precursor [Homo sapiens]

Nominal mass (M_r): **66948**; Calculated pI value: **5.67**
NCBI BLAST search of gi|27262655 against nr
Unformatted sequence string for pasting into other applications

Taxonomy: Homo sapiens
Links to retrieve other entries containing this sequence from NCBI Entrez:
gi|50403773 from Homo sapiens
gi|4322271 from Homo sapiens
gi|40786787 from Homo sapiens

Fixed modifications: Carbamidomethyl (C)
Variable modifications: Oxidation (M)
Cleavage by Trypsin: cuts C-term side of KR unless next residue is P
Sequence Coverage: **20%**

Matched peptides shown in **Bold Red**

```
  1 MDYSFDTTAE DPWVRISDCI KNLFSPIMSE NHGHMPLQPN ASLNEEEGTQ
 51 GHPDGTPPKL DTANGTPKVY KSADSSTVKK GPPVAPKPAW FRQSLKGLRN
101 RASDPRGLPD PALSTQPAPA SREHLGSHIR ASSSSSSIRQ RISSFETFGS
151 SQLPDKGAQR LSLQPSSGEA AKPLGKHEEG RFSGLLGRGA APTLVPQQPE
201 QVLSSGSPAA SEARDPGVSE SPPPGRQPNQ KTLPPGPDPL LRLLSTQAEE
251 SQGPVLKMPS QRARSFPLTR SQSCETKLLD EKTSKLYSIS SQVSSAVMKS
301 LLCLPSSISC AQTPCIPKEG ASPTSSSNED SAANGSAETS ALDTGFSLNL
351 SELREYTEGL TEAKEDDDGD HSSLQSGQSV ISLLSSEELK KLIEEVKVLD
401 EATLKQLDGI HVTILHKEEG AGLGFSLAGG ADLENKVITV HRVFPNGLAS
451 QEGTIQKGNE VLSINGKSLK GTTHHDALAI LRQAREPRQA VIVTRKLTPE
501 AMPDLNSSTD SAASASAASD VSVESTAEAT VCTVTLEKMS AGLGFSLEGG
551 KGSLHGDKPL TINRIFKGAA SEQSETVQPG DEILQLGGTA MQGLTRFEAW
601 NIIKALPDGP VTIVIRRKSL QSKETTAAGD S
```

Residue Number Increasing Mass Decreasing Mass

Start - End	Observed	Mr(expt)	Mr(calc)	Delta	Miss	Sequence
107 - 122	1577.8201	1576.8128	1576.8209	-0.0081	0	GLPDPALSTQPAPASR (No match)
142 - 160	2054.9978	2053.9905	2054.0068	-0.0163	1	ISSFETFGSSQLPDKGAQR (Ions score 85)
142 - 160	2054.9978	2053.9905	2054.0068	-0.0163	1	ISSFETFGSSQLPDKGAQR (No match)
189 - 214	2548.2800	2547.2727	2547.2928	-0.0201	0	GAAPTLVPQQPEQVLSSGSPAASEAR (No match)
189 - 214	2548.2800	2547.2727	2547.2928	-0.0201	0	GAAPTLVPQQPEQVLSSGSPAASEAR (Ions score 106)
215 - 231	1789.8721	1788.8648	1788.8754	-0.0106	1	DPGVSESPPPGRQPNQK (No match)
232 - 242	1175.6740	1174.6667	1174.6710	-0.0043	0	TLPPGPDPLLR (No match)
232 - 242	1175.6740	1174.6667	1174.6710	-0.0043	0	TLPPGPDPLLR (Ions score 22)
568 - 596	2960.4348	2959.4275	2959.4192	0.0083	0	GAASEQSETVQPGDEILQLGGTAMQGLTR Oxidation (M) (No match)
568 - 596	2960.4348	2959.4275	2959.4192	0.0083	0	GAASEQSETVQPGDEILQLGGTAMQGLTR Oxidation (M) (Ions score 10)
605 - 616	1250.7430	1249.7357	1249.7394	-0.0036	0	ALPDGPVTIVIR (No match)

Mascot: http://www.matrixscience.com/

Spot 210

{MATRIX}{SCIENCE} Mascot Search Results

Protein View

Match to: **gi|27262655** Score: **406** Expect: **4.8e-036**
interleukin 16 isoform 1 precursor [Homo sapiens]

Nominal mass (M_r): **66948**; Calculated pI value: **5.67**
NCBI BLAST search of gi|27262655 against nr
Unformatted sequence string for pasting into other applications

Taxonomy: Homo sapiens
Links to retrieve other entries containing this sequence from NCBI Entrez:
gi|50403773 from Homo sapiens
gi|4322271 from Homo sapiens
gi|40786787 from Homo sapiens

Fixed modifications: Carbamidomethyl (C)
Variable modifications: Oxidation (M)
Cleavage by Trypsin: cuts C-term side of KR unless next residue is P
Sequence Coverage: **40%**

Matched peptides shown in **Bold Red**

```
  1 MDYSFDTTAE DPWVRISDCI KNLFSPIMSE NHGHMPLQPN ASLNEEEGTQ
 51 GHPDGTPPKL DTANGTPKVY KSADSSTVKK GPPVAPKPAW FRQSLKGLRN
101 RASDPRGLPD PALSTQPAPA SREHLGSHIR ASSSSSSIRQ RISSFETFGS
151 SQLPDKGAQR LSLQPSSGEA AKPLGKHEEG RFSGLLGRGA APTLVPQQPE
201 QVLSSGSPAA SEARDPGVSE SPPPGRQPNQ KTLPPGPDPL LRLLSTQAEE
251 SQGPVLKMPS QRARSFPLTR SQSCETKLLD EKTSKLYSIS SQVSSAVMKS
301 LLCLPSSISC AQTPCIPKEG ASPTSSSNED SAANGSAETS ALDTGFSLNL
351 SELREYTEGL TEAKEDDDGD HSSLQSGQSV ISLLSSEELK KLIEEVKVLD
401 EATLKQLDGI HVTILHKEEG AGLGFSLAGG ADLENKVITV HRVFPNGLAS
451 QEGTIQKGNE VLSINGKSLK GTTHHDALAI LRQAREPRQA VIVTRKLTPE
501 AMPDLNSSTD SAASASAASD VSVESTAEAT VCTVTLEKMS AGLGFSLEGG
551 KGSLHGDKPL TINRIFKGAA SEQSETVQPG DEILQLGGTA MQGLTRFEAW
601 NIIKALPDGP VTIVIRRKSL QSKETTAAGD S
```

Residue Number Increasing Mass Decreasing Mass

Start - End	Observed	Mr(expt)	Mr(calc)	Delta	Miss	Sequence
80 - 92	1450.8778	1449.8705	1449.8244	0.0461	1	KGPPVAPKPAWFR (No match)
107 - 122	1577.8756	1576.8683	1576.8209	0.0474	0	GLPDPALSTQPAPASR (No match)
107 - 122	1577.8756	1576.8683	1576.8209	0.0474	0	GLPDPALSTQPAPASR (Ions score 21)
123 - 130	948.5278	947.5205	947.4937	0.0269	0	EHLGSHIR (No match)
142 - 160	2055.0715	2054.0642	2054.0068	0.0574	1	ISSFETFGSSQLPDKGAQR (No match)
142 - 160	2055.0715	2054.0642	2054.0068	0.0574	1	ISSFETFGSSQLPDKGAQR (Ions score 40)
161 - 181	2191.2097	2190.2024	2190.1392	0.0632	1	LSLQPSSGEAAKPLGKHEEGR (No match)
189 - 214	2548.3616	2547.3543	2547.2928	0.0615	0	GAAPTLVPQQPEQVLSSGSPAASEAR (No match)
189 - 214	2548.3616	2547.3543	2547.2928	0.0615	0	GAAPTLVPQQPEQVLSSGSPAASEAR (Ions score 73)
215 - 231	1789.9397	1788.9324	1788.8754	0.0570	1	DPGVSESPPPGRQPNQK (No match)
232 - 242	1175.7112	1174.7039	1174.6710	0.0329	0	TLPPGPDPLLR (Ions score 25)
232 - 242	1175.7112	1174.7039	1174.6710	0.0329	0	TLPPGPDPLLR (No match)
243 - 262	2215.2104	2214.2031	2214.1314	0.0718	1	LLSTQAEESQGPVLKMPSQR Oxidation (M) (No match)
392 - 405	1599.9052	1598.8979	1598.9130	-0.0151	1	LIEEVKVLDEATLK (No match)
443 - 457	1588.8799	1587.8726	1587.8256	0.0470	0	VFPNGLASQEGTIQK (No match)
468 - 482	1632.9653	1631.9580	1631.9107	0.0473	1	SLKGTTHHDALAILR (No match)
471 - 482	1304.7423	1303.7350	1303.6996	0.0354	0	GTTHHDALAILR (No match)
552 - 564	1407.8568	1406.8495	1406.7630	0.0866	0	GSLHGDKPLTINR (No match)
568 - 596	2960.5293	2959.5220	2959.4192	0.1028	0	GAASEQSETVQPGDEILQLGGTAMQGLTR Oxidation (M) (No match)
597 - 604	1020.5690	1019.5617	1019.5440	0.0178	0	FEAWNIIK (No match)
605 - 616	1250.7842	1249.7769	1249.7394	0.0376	0	ALPDGPVTIVIR (No match)
605 - 616	1250.7842	1249.7769	1249.7394	0.0376	0	ALPDGPVTIVIR (Ions score 43)
605 - 617	1406.8916	1405.8843	1405.8405	0.0439	1	ALPDGPVTIVIRR (No match)

Mascot: http://www.matrixscience.com/

Spot 211

{MATRIX SCIENCE} Mascot Search Results

Protein View

Match to: **gi|119596926** Score: **259** Expect: **2.4e-021**
leucine-rich repeats and calponin homology (CH) domain containing 4, isoform CRA_b [Homo sapiens]

Nominal mass (M_r): **70861;** Calculated pI value: **6.88**
NCBI BLAST search of gi|119596926 against nr
Unformatted sequence string for pasting into other applications

Taxonomy: Homo sapiens

Fixed modifications: Carbamidomethyl (C)
Variable modifications: Oxidation (M)
Cleavage by Trypsin: cuts C-term side of KR unless next residue is P
Sequence Coverage: **28%**

Matched peptides shown in **Bold Red**

```
  1 MAAAVAAPLA AGGEEAAATT SVPGSPGLPG RRSAERALEE AVATGTLNLS
 51 NRRLKHFPRG AARSYDLSDI TQADLSRNRF PEVPEAACQL VSLEGLSLYH
101 NCLRCLNPAL GNLTALTYLN LSRNQLSLLP PYICQLPLRV LIVSNNKLGA
151 LPPDIGTLGS LRQLDVSSNE LQSLPSELCG LSSLRDLNVR RNQLSTLPEE
201 LGDLPLVRLD FSCNRVSRIP VSFCRLRHLQ VILLDSNPLQ SPPAQVCLKG
251 KLHIFKYLST EAGQRGSALG DLAPSRPPSF SPCPAEDLFP GHRYDGGLDS
301 GFHSVDSGSK RWSGNESTDE FSELSFRISE LAREPRGPRE RKEDGSADGD
351 PVQIDFIDSH VPGEDEERGT VEEQRPPELS PGAGDRERAP SSRREEPAGE
401 ERRRPDTLQL WQERERRQQQ QSGAWGAPRK DSLLKPGLRA VVGGAAAVST
451 QAMHNGSPKS SASQAGAAAG QGAPAPAPAS QEPLPIAGPA TAPAPRPLGS
501 IQRPNSFLFR SSSQSGSGPS SPDSVLRPRR YPQVPDEKDL MTQLRQVLES
551 RLQRPLPEDL AEALASGVIL CQLANQLRPR SVPFIHVPSP AVPKLSALKA
601 RKNVESFLEA CRKMGVPEES LCQPHHILEE EGAPGRGLPY IAAVVHALLD
651 RP
```

Residue Number Increasing Mass Decreasing Mass

Start - End	Observed	Mr(expt)	Mr(calc)	Delta	Miss	Sequence
37 - 53	1815.0116	1814.0043	1813.9645	0.0398	1	ALEEAVATGTLNLSNRR (No match)

64 - 77	1583.7861	1582.7788	1582.7475	0.0314	0	SYDLSDITQADLSR (No match)
105 - 123	2104.1565	2103.1492	2103.1146	0.0346	0	CLNPALGNLTALTYLNLSR (No match)
124 - 139	1925.1024	1924.0951	1924.0604	0.0347	0	NQLSLLPPYICQLPLR (No match)
124 - 139	1925.1024	1924.0951	1924.0604	0.0347	0	NQLSLLPPYICQLPLR (Ions score 30)
148 - 162	1479.8838	1478.8765	1478.8456	0.0309	0	LGALPPDIGTLGSLR (Ions score 39)
148 - 162	1479.8838	1478.8765	1478.8456	0.0309	0	LGALPPDIGTLGSLR (No match)
192 - 208	1894.0632	1893.0559	1893.0207	0.0352	0	NQLSTLPEELGDLPLVR (No match)
192 - 208	1894.0632	1893.0559	1893.0207	0.0352	0	NQLSTLPEELGDLPLVR (Ions score 52)
209 - 215	911.4263	910.4190	910.3967	0.0224	0	LDFSCNR (No match)
219 - 225	878.4749	877.4676	877.4480	0.0197	0	IPVSFCR (No match)
257 - 265	1024.5380	1023.5307	1023.4985	0.0323	0	YLSTEAGQR (No match)
418 - 429	1313.6613	1312.6540	1312.6272	0.0268	0	QQQQSGAWGAPR (No match)
430 - 439	1126.7209	1125.7136	1125.6869	0.0267	1	KDSLLKPGLR (No match)
511 - 529	1887.9474	1886.9401	1886.9082	0.0320	0	SSSQSGSGPSSPDSVLRPR (No match)
581 - 594	1474.8679	1473.8606	1473.8343	0.0263	0	SVPFIHVPSPAVPK (No match)
603 - 613	1352.6920	1351.6847	1351.6554	0.0294	1	NVESFLEACRK (No match)

Mascot: http://www.matrixscience.com/

Spot 212

MATRIX SCIENCE **Mascot Search Results**

Protein View

Match to: **gi|16507237** Score: **157** Expect: **3.8e-011**
heat shock 70kDa protein 5 [Homo sapiens]

Nominal mass (M_r): **72402;** Calculated pI value: **5.07**
NCBI BLAST search of gi|16507237 against nr
Unformatted sequence string for pasting into other applications

Taxonomy: Homo sapiens
Links to retrieve other entries containing this sequence from NCBI Entrez:
gi|109112231 from Macaca mulatta
gi|114626688 from Pan troglodytes
gi|114626690 from Pan troglodytes
gi|14916999 from Homo sapiens
gi|7229462 from Homo sapiens
gi|6900104 from Homo sapiens
gi|18044381 from Homo sapiens
gi|86559365 from Homo sapiens
gi|119608026 from Homo sapiens
gi|123982716 from synthetic construct
gi|123997385 from synthetic construct

Fixed modifications: Carbamidomethyl (C)
Variable modifications: Oxidation (M)
Cleavage by Trypsin: cuts C-term side of KR unless next residue is P
Sequence Coverage: **24%**

Matched peptides shown in **Bold Red**

1 MKLSLVAAML LLLSAARAEE EDKKEDVGTV VGIDLGTTYS CVGVFKNGRV
51 EIIANDQGNR **ITPSYVAFTP EGER**LIGDAA K**NQLTSNPEN TVFDAKR**LIG
101 RTWNDPSVQQ DIKFLPFKVV EKKTKPYIQV DIGGGQTKTF APEEISAMVL
151 TKMKETAEAY LGKK**VTHAVV TVPAYFNDAQ R**QATKDAGTI AGLNVMR**IIN**
201 **EPTAAAIAYG LDKR**EGEKNI LVFDLGGGTF DVSLLTIDNG VFEVVATNGD
251 THLGGEDFDQ RVMEHFIKLY KKKTGKDVRK DNRAVQKLRR EVEKAKRALS
301 SQHQAR**IEIE SFYEGEDFSE TLTRAKFEEL NMDLFR**STMK PVQKVLEDSD
351 LK**KSDIDEIV LVGGSTR**IPK IQQLVK**EFFN GKEPSR**GINP DEAVYGAAV
401 QAGVLSGDQD TGDLVLLDVC PLTLGIETVG GVMTKLIPRN TVVPTKKSQI
451 FSTASDNQPT VTIKVYEGER PLTK**DNHLLG TFDLTGIPPA PRGVPQIEVT**

```
501 FEIDVNGILR VTAEDKGTGN KNKITITNDQ NRLTPEEIER MVNDAEKFAE
551 EDKKLKERID TRNELESYAY SLKNQIGDKE KLGGKLSSED KETMEKAVEE
601 KIEWLESHQD ADIEDFKAKK KELEEIVQPI ISKLYGSAGP PPTGEEDTAE
651 KDEL
```

Residue Number Increasing Mass Decreasing Mass

Start - End	Observed	Mr(expt)	Mr(calc)	Delta	Miss	Sequence
61 - 74	1566.7668	1565.7595	1565.7725	-0.0130	0	ITPSYVAFTPEGER (No match)
61 - 74	1566.7668	1565.7595	1565.7725	-0.0130	0	ITPSYVAFTPEGER (Ions score 2)
82 - 97	1833.9088	1832.9015	1832.9016	-0.0001	1	NQLTSNPENTVFDAKR (No match)
165 - 181	1887.9473	1886.9400	1886.9638	-0.0238	0	VTHAVVTVPAYFNDAQR (Ions score 8)
165 - 181	1887.9473	1886.9400	1886.9638	-0.0238	0	VTHAVVTVPAYFNDAQR (No match)
198 - 214	1815.9802	1814.9729	1814.9890	-0.0160	1	IINEPTAAAIAYGLDKR (No match)
198 - 214	1815.9802	1814.9729	1814.9890	-0.0160	1	IINEPTAAAIAYGLDKR (Ions score 7)
307 - 324	2164.9695	2163.9622	2163.9847	-0.0225	0	IEIESFYEGEDFSETLTR (No match)
325 - 336	1528.7310	1527.7237	1527.7391	-0.0154	1	AKFEELNMDLFR Oxidation (M) (No match)
353 - 367	1588.8397	1587.8324	1587.8467	-0.0143	1	KSDIDEIVLVGGSTR (No match)
377 - 386	1210.5769	1209.5696	1209.5778	-0.0081	1	EFFNGKEPSR (No match)
377 - 386	1210.5769	1209.5696	1209.5778	-0.0081	1	EFFNGKEPSR (Ions score 17)
475 - 492	1933.9933	1932.9860	1933.0057	-0.0197	0	DNHLLGTFDLTGIPPAPR (No match)
475 - 492	1933.9933	1932.9860	1933.0057	-0.0197	0	DNHLLGTFDLTGIPPAPR (Ions score 23)
493 - 510	1999.0710	1998.0637	1998.0785	-0.0148	0	GVPQIEVTFEIDVNGILR (No match)
533 - 540	986.5046	985.4973	985.5080	-0.0107	0	LTPEEIER (No match)

Mascot: http://www.matrixscience.com/

Spot 213

{MATRIX SCIENCE} Mascot Search Results

Protein View

Match to: **gi|119596926** Score: **152** Expect: **1.2e-010**
leucine-rich repeats and calponin homology (CH) domain containing 4, isoform CRA_b [Homo sapiens]

Nominal mass (M_r): **70861;** Calculated pI value: **6.88**
NCBI BLAST search of gi|119596926 against nr
Unformatted sequence string for pasting into other applications

Taxonomy: Homo sapiens

Fixed modifications: Carbamidomethyl (C)
Variable modifications: Oxidation (M)
Cleavage by Trypsin: cuts C-term side of KR unless next residue is P
Sequence Coverage: **14%**

Matched peptides shown in **Bold Red**

```
  1 MAAAVAPLA AGGEEAAATT SVPGSPGLPG RRSAERALEE AVATGTLNLS
 51 NRRLKHFPRG AARSYDLSDI TQADLSRNRF PEVPEAACQL VSLEGLSLYH
101 NCLRCLNPAL GNLTALTYLN LSRNQLSLLP PYICQLPLRV LIVSNNKLGA
151 LPPDIGTLGS LRQLDVSSNE LQSLPSELCG LSSLRDLNVR RNQLSTLPEE
201 LGDLPLVRLD FSCNRVSRIP VSFCRLRHLQ VILLDSNPLQ SPPAQVCLKG
251 KLHIFKYLST EAGQRGSALG DLAPSRPPSF SPCPAEDLFP GHRYDGGLDS
301 GFHSVDSGSK RWSGNESTDE FSELSFRISE LAREPRGPRE RKEDGSADGD
351 PVQIDFIDSH VPGEDEERGT VEEQRPPELS PGAGDRERAP SSRREEPAGE
401 ERRRPDTLQL WQERERRQQQ QSGAWGAPRK DSLLKPGLRA VVGGAAAVST
451 QAMHNGSPKS SASQAGAAAG QGAPAPAPAS QEPLPIAGPA TAPAPRPLGS
501 IQRPNSFLFR SSSQSGSGPS SPDSVLRPRR YPQVPDEKDL MTQLRQVLES
551 RLQRPLPEDL AEALASGVIL CQLANQLRPR SVPFIHVPSP AVPKLSALKA
601 RKNVESFLEA CRKMGVPEES LCQPHHILEE EGAPGRGLPY IAAVVHALLD
651 RP
```

Residue Number Increasing Mass Decreasing Mass

Start - End	Observed	Mr(expt)	Mr(calc)	Delta	Miss	Sequence	
64 - 77	1583.7419	1582.7346	1582.7475	-0.0128	0	SYDLSDITQADLSR	(No match)

105 - 123	2104.0840	2103.0767	2103.1146	-0.0379	0	CLNPALGNLTALTYLNLSR	(No match)
124 - 139	1925.0450	1924.0377	1924.0604	-0.0227	0	NQLSLLPPYICQLPLR	(No match)
124 - 139	1925.0450	1924.0377	1924.0604	-0.0227	0	NQLSLLPPYICQLPLR	(Ions score 31)
148 - 162	1479.8368	1478.8295	1478.8456	-0.0161	0	LGALPPDIGTLGSLR	(Ions score 40)
148 - 162	1479.8368	1478.8295	1478.8456	-0.0161	0	LGALPPDIGTLGSLR	(No match)
192 - 208	1894.0060	1892.9987	1893.0207	-0.0220	0	NQLSTLPEELGDLPLVR	(Ions score 22)
192 - 208	1894.0060	1892.9987	1893.0207	-0.0220	0	NQLSTLPEELGDLPLVR	(No match)
637 - 652	1704.9609	1703.9536	1703.9722	-0.0186	0	GLPYIAAVVHALLDRP	(No match)
637 - 652	1704.9609	1703.9536	1703.9722	-0.0186	0	GLPYIAAVVHALLDRP	(No match)

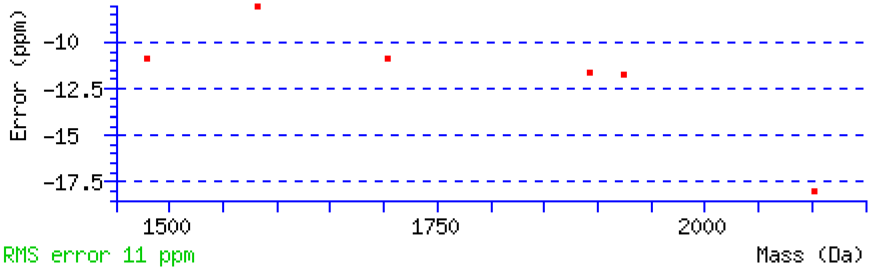

Mascot: http://www.matrixscience.com/

Spot 214

{MATRIX SCIENCE} Mascot Search Results

Protein View

Match to: **gi|7661880** Score: **142** Expect: **1.2e-009**
centaurin beta1 [Homo sapiens]

Nominal mass (M_r): **82397**; Calculated pI value: **7.60**
NCBI BLAST search of gi|7661880 against nr
Unformatted sequence string for pasting into other applications

Taxonomy: Homo sapiens
Links to retrieve other entries containing this sequence from NCBI Entrez:
gi|3183210 from Homo sapiens
gi|17391289 from Homo sapiens
gi|32879919 from Homo sapiens
gi|61359819 from synthetic construct
gi|61359827 from synthetic construct
gi|119610616 from Homo sapiens
gi|123981632 from synthetic construct

Fixed modifications: Carbamidomethyl (C)
Variable modifications: Oxidation (M)
Cleavage by Trypsin: cuts C-term side of KR unless next residue is P
Sequence Coverage: **16%**

Matched peptides shown in **Bold Red**

```
  1 MTVKLDFEEC LKDSPRFRAS IELVEAEVSE LETRLEKLLK LGTGLLESGR
 51 HYLAASRAFV VGICDLARLG PPEPMMAECL EKFTVSLNHK LDSHAELLDA
101 TQHTLQQQIQ TLVKEGLRGF REARRDFWRG AESLEAALTH NAEVPRRRAQ
151 EAEEAGAALR TARAGYRGRA LDYALQINVI EDKRKFDIME FVLRLVEAQA
201 THFQQGHEEL SRLSQYRKEL GAQLHQLVLN SAREKRDMEQ RHVLLKQKEL
251 GGEEPEPSLR EGPGGLVMEG HLFKRASNAF KTWSRRWFTI QSNQLVYQKK
301 YKDPVTVVVD DLRLCTVKLC PDSERRFCFE VVSTSKSCLL QADSERLLQL
351 WVSAVQSSIA SAFSQARLDD SPRGPGQGSG HLAIGSAATL GSGGMARGRE
401 PGGVGHVVAQ VQSVDGNAQC CDCREPAPEW ASINLGVTLC IQCSGIHRSL
451 GVHFSKVRSL TLDSWEPELV KLMCELGNVI INQIYEARVE AMAVKKPGPS
501 CSRQEKEAWI HAKYVEKKFL TKLPEIRGRR GGRGRPRGQP PVPPKPSIRP
551 RPGSLRSKPE PPSEDLGSLH PGALLFRASG HPPSLPTMAD ALAHGADVNW
601 VNGGQDNATP LIQATAANSL LACEFLLQNG ANVNQADSAG RGPLHHATIL
651 GHTGLACLFL KRGADLGARD SEGRDPLTIA METANADIVT LLRLAKMREA
```

701 EAAQGQAGDE TYLDIFRDFS LMASDDPEKL SRRSHDLHTL

Residue Number Increasing Mass Decreasing Mass

Start - End	Observed	Mr(expt)	Mr(calc)	Delta	Miss	Sequence
19 - 34	1774.9629	1773.9556	1773.8995	0.0561	0	ASIELVEAEVSELETR (No match)
58 - 68	1220.6798	1219.6725	1219.6383	0.0343	0	AFVVGICDLAR (No match)
130 - 146	1764.9449	1763.9376	1763.8801	0.0575	0	GAESLEAALTHNAEVPR (Ions score 32)
130 - 146	1764.9449	1763.9376	1763.8801	0.0575	0	GAESLEAALTHNAEVPR (No match)
185 - 194	1313.7178	1312.7105	1312.6849	0.0256	1	KFDIMEFVLR Oxidation (M) (No match)
186 - 194	1185.6260	1184.6187	1184.5899	0.0288	0	FDIMEFVLR Oxidation (M) (No match)
195 - 212	2080.0859	2079.0786	2079.0133	0.0653	0	LVEAQATHFQQGHEELSR (No match)
195 - 212	2080.0859	2079.0786	2079.0133	0.0653	0	LVEAQATHFQQGHEELSR (Ions score 17)
219 - 233	1648.9663	1647.9590	1647.9056	0.0534	0	ELGAQLHQLVLNSAR (No match)
219 - 233	1648.9663	1647.9590	1647.9056	0.0534	0	ELGAQLHQLVLNSAR (Ions score 30)
247 - 260	1568.8452	1567.8379	1567.7841	0.0538	1	QKELGGEEPEPSLR (No match)
557 - 577	2247.2456	2246.2383	2246.1694	0.0689	0	SKPEPPSEDLGSLHPGALLFR (No match)
557 - 577	2247.2456	2246.2383	2246.1694	0.0689	0	SKPEPPSEDLGSLHPGALLFR (Ions score 3)

Mascot: http://www.matrixscience.com/

Spot 215

{MATRIX}{SCIENCE} Mascot Search Results

Protein View

Match to: **gi|13529299** Score: **65** Expect: **0.055**
SEC23B protein [Homo sapiens]

Nominal mass (M_r): **87377**; Calculated pI value: **6.43**
NCBI BLAST search of gi|13529299 against nr
Unformatted sequence string for pasting into other applications

Taxonomy: Homo sapiens
Links to retrieve other entries containing this sequence from NCBI Entrez:
gi|123980500 from synthetic construct
gi|123995313 from synthetic construct

Fixed modifications: Carbamidomethyl (C)
Variable modifications: Oxidation (M)
Cleavage by Trypsin: cuts C-term side of KR unless next residue is P
Sequence Coverage: **17%**

Matched peptides shown in **Bold Red**

```
  1 MATYLEFIQQ NEERDGVRFS WNVWPSSRLE ATRMVVPLAC LLTPLKERPD
 51 LPPVQYEPVL CSRPTCKAVL NPLCQVDYRA KLWACNFCFQ RNQFPPAYGG
101 ISEVNQPAEL MPQFSTIEYV IQRGAQSPLI FLYVVDTCLE EDDLQALKES
151 LQMSLSLLPP DALVGLITFG RMVQVHELSC EGISKSYVFR GTKDLTAKQI
201 QDMLGLTKPA MPMQQARPAQ PQEHPFASSR FLQPVHKIDM NLTDLLGELQ
251 RDPWPVTQGK RPLRSTGVAL SIAVGLLEGT FPNTGARIML FTGGPPTQGP
301 GMVVGDELKI PIRSWHDIEK DNARFMKKAT KHYEMLANRT AANGHCIDIY
351 ACALDQTGLL EMKCCANLTG GYVVMGDSFN TSLFKQTFQR IFTKDFNGDF
401 RMAFGATLDV KTSRELKIAG AIGPCVSLNV KGLCVSENEL GVGGTSQWKI
451 CGLDPTSTLG IYFEVVNQHN TPIPQGGRGA IQFVTHYQHS STQRRIRVTT
501 IARNWADVQS QLRHIEAAFD QEAAAVLMAR LGVFRAESEE GPDVLRWLDR
551 QLIRLCQKFG QYNKEDPTSF RLSDSFSLYP QFMFHLRRSP FLQVFNNSPD
601 ESSYYRHHFA RQDLTQSLIM IQPILYSYSF HGPPEPVLLD SSSILADRIL
651 LMDTFFQIVI YLGETIAQWR KAGYQDMPEY ENFKHLLQAP LDDAQEILQA
701 RFPMPRYINT EHGGSQARFL LSKVNPSQTH NNLYAWGQET GAPILTDDVS
751 LQVFMDHLKK LAVSSAC
```

Residue Number Increasing Mass Decreasing Mass

Start - End	Observed	Mr(expt)	Mr(calc)	Delta	Miss	Sequence
2 - 14	1640.7538	1639.7465	1639.7841	-0.0376	0	ATYLEFIQQNEER (No match)
68 - 79	1447.7198	1446.7125	1446.7289	-0.0164	0	AVLNPLCQVDYR (No match)
265 - 287	2231.1792	2230.1719	2230.1956	-0.0237	0	STGVALSIAVGLLEGTFPNTGAR (No match)
479 - 494	1859.8921	1858.8848	1858.9074	-0.0226	0	GAIQFVTHYQHSSTQR (No match)
536 - 546	1201.5663	1200.5590	1200.5622	-0.0032	0	AESEEGPDVLR (No match)
559 - 571	1588.7224	1587.7151	1587.7317	-0.0166	1	FGQYNKEDPTSFR (No match)
559 - 571	1588.7224	1587.7151	1587.7317	-0.0166	1	FGQYNKEDPTSFR (Ions score 5)
589 - 606	2149.9492	2148.9419	2148.9752	-0.0333	0	SPFLQVFNNSPDESSYYR (No match)
685 - 701	1931.0182	1930.0109	1930.0272	-0.0163	0	HLLQAPLDDAQEILQAR (No match)
707 - 718	1332.6237	1331.6164	1331.6218	-0.0053	0	YINTEHGGSQAR (No match)

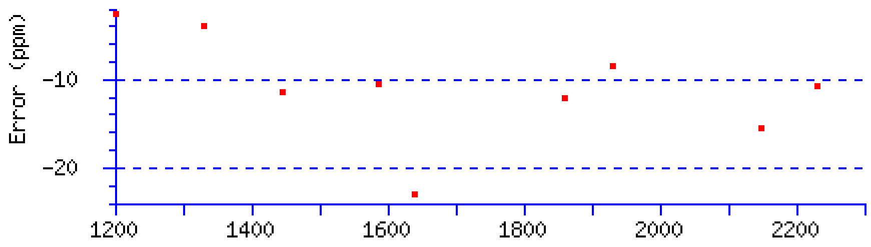

RMS error 12 ppm

Mascot: http://www.matrixscience.com/

Spot 216

{MATRIX SCIENCE} **Mascot Search Results**

Protein View

Match to: **gi|62896583** Score: **330** Expect: **1.9e-028**
Hypothetical protein FLJ42574 variant [Homo sapiens]

Nominal mass (M_r): **71794;** Calculated pI value: **6.12**
NCBI BLAST search of gi|62896583 against nr
Unformatted sequence string for pasting into other applications

Taxonomy: Homo sapiens

Fixed modifications: Carbamidomethyl (C)
Variable modifications: Oxidation (M)
Cleavage by Trypsin: cuts C-term side of KR unless next residue is P
Sequence Coverage: **30%**

Matched peptides shown in **Bold Red**

```
  1 SASAGCPGPG IGPQTKSSTE GEPGWRRASP VTQTSPIKHP LLKRQARMDY
 51 SFDTTAEDPW VRISDCIKNL FSPIMSENHG HMPLQPNASL NEEEGTQGHP
101 DGTPPRLDTA NGTPKVYKSA DSSTVKKGPP VAPKPAWFRQ SLKGLRNRAS
151 DPRGLPDPAL STQPAPASRE HLGSHIRASS SSSSIRQRIS SFETFGSSQL
201 PDKGAQRLSL QPSSGEAAKP LGKHEEGRFS GLLGRGAAPT LVPQQPEQVL
251 SSGSPAASEA RDPGVSESPP PGRQPNQKTL PPGPDPLLRL LSTQAEESQG
301 PVLKMPSQRA RSFPLTRSQS CETKLLDEKT SKLYSISSQV SSAVMKSLLC
351 LPSSISCAQT PCIPKEGASP TSSSNEDSAA NGSAETSALD TGFSLNLSEL
401 REYTEGLTEA KEDDDGDHSS LQSGQSVISL LSSEELKKLI EEAKVLDEAT
451 LKQLDGIHVT ILHKEEGAGL GFSLAGGADL ENKVITVHRV FPNGLASQEG
501 TIQKGNEVLS INGKSLKGTT HHDALAILRQ AREPRQAVIV TRKLTPEAMP
551 DLNSSTDSAA SASAASDVSV ESTEATVCTV TLEKMSAGLG FSLEGGKGSL
601 HGDKPLTINR IFKGAASEQS ETVQPGDEIL QLGGTAMQGL TRFEAWNIIK
651 ALPDGPVTIV IRRKSLQSKE TTAAGDS
```

Residue Number Increasing Mass Decreasing Mass

Start - End	Observed	Mr(expt)	Mr(calc)	Delta	Miss	Sequence
27 - 38	1284.7278	1283.7205	1283.7197	0.0008	1	RASPVTQTSPIK (No match)

154 - 169	1577.8768	1576.8695	1576.8209	0.0486	0	GLPDPALSTQPAPASR	(Ions score 19)
154 - 169	1577.8768	1576.8695	1576.8209	0.0486	0	GLPDPALSTQPAPASR	(No match)
170 - 177	948.5285	947.5212	947.4937	0.0276	0	EHLGSHIR	(No match)
189 - 207	2055.0713	2054.0640	2054.0068	0.0572	1	ISSFETFGSSQLPDKGAQR	(No match)
189 - 207	2055.0713	2054.0640	2054.0068	0.0572	1	ISSFETFGSSQLPDKGAQR	(Ions score 27)
208 - 228	2191.2051	2190.1978	2190.1392	0.0586	1	LSLQPSSGEAAKPLGKHEEGR	(No match)
236 - 261	2548.3640	2547.3567	2547.2928	0.0639	0	GAAPTLVPQQPEQVLSSGSPAASEAR	(Ions score 83)
236 - 261	2548.3640	2547.3567	2547.2928	0.0639	0	GAAPTLVPQQPEQVLSSGSPAASEAR	(No match)
262 - 278	1789.9381	1788.9308	1788.8754	0.0554	1	DPGVSESPPPGRQPNQK	(No match)
279 - 289	1175.7097	1174.7024	1174.6710	0.0314	0	TLPPGPDPLLR	(No match)
279 - 289	1175.7097	1174.7024	1174.6710	0.0314	0	TLPPGPDPLLR	(Ions score 8)
290 - 304	1599.9143	1598.9070	1598.8515	0.0555	0	LLSTQAEESQGPVLK	(No match)
290 - 309	2215.2056	2214.1983	2214.1314	0.0670	1	LLSTQAEESQGPVLKMPSQR Oxidation (M)	(No match)
490 - 504	1588.8782	1587.8709	1587.8256	0.0453	0	VFPNGLASQEGTIQK	(No match)
515 - 529	1632.9631	1631.9558	1631.9107	0.0451	1	SLKGTTHHDALAILR	(No match)
518 - 529	1304.7454	1303.7381	1303.6996	0.0385	0	GTTHHDALAILR	(No match)
598 - 610	1407.8552	1406.8479	1406.7630	0.0850	0	GSLHGDKPLTINR	(No match)
651 - 662	1250.7827	1249.7754	1249.7394	0.0361	0	ALPDGPVTIVIR	(Ions score 22)
651 - 662	1250.7827	1249.7754	1249.7394	0.0361	0	ALPDGPVTIVIR	(No match)
651 - 663	1406.8899	1405.8826	1405.8405	0.0422	1	ALPDGPVTIVIRR	(No match)

Mascot: http://www.matrixscience.com/

Spot 217

Mascot Search Results

Protein View

Match to: **gi|2114410** Score: **285** Expect: **6.1e-024**
interleukin-16 [Homo sapiens]

Nominal mass (M_r): **67047;** Calculated pI value: **5.74**
NCBI BLAST search of gi|2114410 against nr
Unformatted sequence string for pasting into other applications

Taxonomy: Homo sapiens

Fixed modifications: Carbamidomethyl (C)
Variable modifications: Oxidation (M)
Cleavage by Trypsin: cuts C-term side of KR unless next residue is P
Sequence Coverage: **13%**

Matched peptides shown in **Bold Red**

1 MDYSFDTTAE DPWVRISDCI KNLFSPIMSE NHGHMPLQPN ASLNEEEGTQ
51 GHPDGTPPKL DTANGTPKVY KSADSSTVKK GPPVAPKPAW FRQSLKGLRN
101 RASDPR**GLPD PALSTQPAPA SR**EHLGSHIR ASSSSSSIRQ R**ISSFETFGS**
151 **SQLPDKGAQR** LSLQPSSGEA AKPLGKHEEG RFSGLLGR**GA APTLVPQQPE**
201 **QVLSSGSPAA SEAR**DPGVSE SPPPRRQPNQ K**TLPPGPDPL LR**LLSTQAEE
251 SQGPVLKMPS QRARSFPLTR SQSCETKLLD EKTSKLYSIS SQVSSAVMKS
301 LLCLPSSISC AQTPCIPKEG ASPTSSSNED SAANGSAETS ALDTGFSLNL
351 SELREYTEGL TEAKEDDDGD HSSLQSGQSV ISLLSSEELK KLIEEVKVLD
401 EATLKQLDGI HVTILHKEEG AGLGFSLAGG ADLENKVITV HRVFPNGLAS
451 QEGTIQKGNE VLSINGKSLK GTTHHDALAI LRQAREPRQA VIVTRKLTPE
501 AMPDLNSSTD SAASASAASD VSVESTAEAT VCTVTLEKMS AGLGFSLEGG
551 KGSLHGDKPL TINRIFKGAA SEQSETVQPG DEILQLGGTA MQGLTRFEAW
601 NIIK**ALPDGP VTIVIR**RKSL QSKETTAAGD S

Residue Number Increasing Mass Decreasing Mass

Start - End	Observed	Mr(expt)	Mr(calc)	Delta	Miss	Sequence	
107 - 122	1577.8230	1576.8157	1576.8209	-0.0052	0	GLPDPALSTQPAPASR	(Ions score 25)
107 - 122	1577.8230	1576.8157	1576.8209	-0.0052	0	GLPDPALSTQPAPASR	(No match)

142 - 160	2055.0071	2053.9998	2054.0068	-0.0070	1	ISSFETFGSSQLPDKGAQR	(Ions score 86)
142 - 160	2055.0071	2053.9998	2054.0068	-0.0070	1	ISSFETFGSSQLPDKGAQR	(No match)
189 - 214	2548.2908	2547.2835	2547.2928	-0.0093	0	GAAPTLVPQQPEQVLSSGSPAASEAR	(No match)
189 - 214	2548.2908	2547.2835	2547.2928	-0.0093	0	GAAPTLVPQQPEQVLSSGSPAASEAR	(Ions score 65)
232 - 242	1175.6735	1174.6662	1174.6710	-0.0048	0	TLPPGPDPLLR	(No match)
232 - 242	1175.6735	1174.6662	1174.6710	-0.0048	0	TLPPGPDPLLR	(Ions score 22)
605 - 616	1250.7438	1249.7365	1249.7394	-0.0028	0	ALPDGPVTIVIR	(Ions score 23)
605 - 616	1250.7438	1249.7365	1249.7394	-0.0028	0	ALPDGPVTIVIR	(No match)

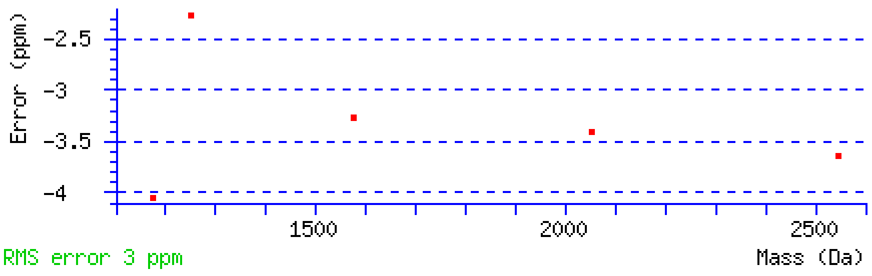

RMS error 3 ppm

Mascot: http://www.matrixscience.com/

Spot 218

MATRIX SCIENCE Mascot Search Results

Protein View

Match to: **gi|2114410** Score: **283** Expect: **9.6e-024**
interleukin-16 [Homo sapiens]

Nominal mass (M_r): **67047;** Calculated pI value: **5.74**
NCBI BLAST search of gi|2114410 against nr
Unformatted sequence string for pasting into other applications

Taxonomy: Homo sapiens

Fixed modifications: Carbamidomethyl (C)
Variable modifications: Oxidation (M)
Cleavage by Trypsin: cuts C-term side of KR unless next residue is P
Sequence Coverage: **13%**

Matched peptides shown in **Bold Red**

```
  1 MDYSFDTTAE DPWVRISDCI KNLFSPIMSE NHGHMPLQPN ASLNEEEGTQ
 51 GHPDGTPPKL DTANGTPKVY KSADSSTVKK GPPVAPKPAW FRQSLKGLRN
101 RASDPRGLPD PALSTQPAPA SREHLGSHIR ASSSSSSIRQ RISSFETFGS
151 SQLPDKGAQR LSLQPSSGEA AKPLGKHEEG RFSGLLGRGA APTLVPQQPE
201 QVLSSGSPAA SEARDPGVSE SPPPRRQPNQ KTLPPGPDPL LRLLSTQAEE
251 SQGPVLKMPS QRARSFPLTR SQSCETKLLD EKTSKLYSIS SQVSSAVMKS
301 LLCLPSSISC AQTPCIPKEG ASPTSSSNED SAANGSAETS ALDTGFSLNL
351 SELREYTEGL TEAKEDDDGD HSSLQSGQSV ISLLSSEELK KLIEEVKVLD
401 EATLKQLDGI HVTILHKEEG AGLGFSLAGG ADLENKVITV HRVFPNGLAS
451 QEGTIQKGNE VLSINGKSLK GTTHHDALAI LRQAREPRQA VIVTRKLTPE
501 AMPDLNSSTD SAASASAASD VSVESTAEAT VCTVTLEKMS AGLGFSLEGG
551 KGSLHGDKPL TINRIFKGAA SEQSETVQPG DEILQLGGTA MQGLTRFEAW
601 NIIKALPDGP VTIVIRRKSL QSKETTAAGD S
```

Residue Number Increasing Mass Decreasing Mass

Start - End	Observed	Mr(expt)	Mr(calc)	Delta	Miss	Sequence	
107 - 122	1577.8279	1576.8206	1576.8209	-0.0003	0	GLPDPALSTQPAPASR	(No match)
107 - 122	1577.8279	1576.8206	1576.8209	-0.0003	0	GLPDPALSTQPAPASR	(Ions score 19)

142 - 160	2055.0103	2054.0030	2054.0068	-0.0038	1	ISSFETFGSSQLPDKGAQR (Ions score 76)
142 - 160	2055.0103	2054.0030	2054.0068	-0.0038	1	ISSFETFGSSQLPDKGAQR (No match)
189 - 214	2548.2935	2547.2862	2547.2928	-0.0066	0	GAAPTLVPQQPEQVLSSGSPAASEAR (Ions score 83)
189 - 214	2548.2935	2547.2862	2547.2928	-0.0066	0	GAAPTLVPQQPEQVLSSGSPAASEAR (No match)
232 - 242	1175.6782	1174.6709	1174.6710	-0.0001	0	TLPPGPDPLLR (No match)
232 - 242	1175.6782	1174.6709	1174.6710	-0.0001	0	TLPPGPDPLLR (Ions score 23)
605 - 616	1250.7467	1249.7394	1249.7394	0.0001	0	ALPDGPVTIVIR (Ions score 23)
605 - 616	1250.7467	1249.7394	1249.7394	0.0001	0	ALPDGPVTIVIR (No match)

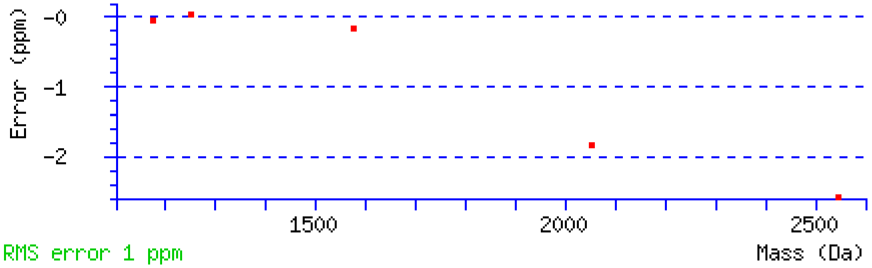

RMS error 1 ppm

Mascot: http://www.matrixscience.com/

Spot 220

{MATRIX SCIENCE} Mascot Search Results

Protein View

Match to: **gi|148539876** Score: **247** Expect: **3.8e-020**
beta adrenergic receptor kinase 1 [Homo sapiens]

Nominal mass (M_r): **80321;** Calculated pI value: **6.89**
NCBI BLAST search of gi|148539876 against nr
Unformatted sequence string for pasting into other applications

Taxonomy: Homo sapiens
Links to retrieve other entries containing this sequence from NCBI Entrez:
gi|126302521 from Homo sapiens
gi|179335 from Homo sapiens
gi|23272739 from Homo sapiens
gi|60552563 from Homo sapiens
gi|123980490 from synthetic construct
gi|123995305 from synthetic construct

Fixed modifications: Carbamidomethyl (C)
Variable modifications: Oxidation (M)
Cleavage by Trypsin: cuts C-term side of KR unless next residue is P
Sequence Coverage: **24%**

Matched peptides shown in **Bold Red**

```
  1 MADLEAVLAD VSYLMAMEKS KATPAARASK KILLPEPSIR SVMQKYLEDR
 51 GEVTFEKIFS QKLGYLLFRD FCLNHLEEAR PLVEFYEEIK KYEKLETEEE
101 RVARSREIFD SYIMKELLAC SHPFSKSATE HVQGHLGKKQ VPPDLFQPYI
151 EEICQNLRGD VFQKFIESDK FTRFCQWKNV ELNIHLTMND FSVHRIIGRG
201 GFGEVYGCRK ADTGKMYAMK CLDKKRIKMK QGETLALNER IMLSLVSTGD
251 CPFIVCMSYA FHTPDKLSFI LDLMNGGDLH YHLSQHGVFS EADMRFYAAE
301 IILGLEHMHN RFVVYRDLKP ANILLDEHGH VRISDLGLAC DFSKKKPHAS
351 VGTHGYMAPE VLQKGVAYDS SADWFSLGCM LFKLLRGHSP FRQHKTKDKH
401 EIDRMTLTMA VELPDSFSPE LRSLLEGLLQ RDVNRRLGCL GRGAQEVKES
451 PFFRSLDWQM VFLQKYPPPL IPPRGEVNAA DAFDIGSFDE EDTKGIKLLD
501 SDQELYRNFP LTISERWQQE VAETVFDTIN AETDRLEARK KAKNKQLGHE
551 EDYALGKDCI MHGYMSKMGN PFLTQWQRRY FYLFPNRLEW RGEGEAPQSL
601 LTMEEIQSVE ETQIKERKCL LLKIRGGKQF ILQCDSDPEL VQWKKELRDA
651 YREAQQLVQR VPKMKNKPRS PVVELSKVPL VQRGSANGL
```

Residue Number Increasing Mass Decreasing Mass

Start - End	Observed	Mr(expt)	Mr(calc)	Delta	Miss	Sequence
31 - 40	1165.7878	1164.7805	1164.7230	0.0575	1	KILLPEPSIR (No match)
32 - 40	1037.6848	1036.6775	1036.6280	0.0495	0	ILLPEPSIR (No match)
46 - 57	1485.7985	1484.7912	1484.7146	0.0766	1	YLEDRGEVTFEK (No match)
63 - 69	881.5597	880.5524	880.5170	0.0354	0	LGYLLFR (No match)
165 - 173	1142.6384	1141.6311	1141.5767	0.0544	1	FIESDKFTR (No match)
200 - 209	1101.5299	1100.5226	1100.4708	0.0518	0	GGFGEVYGCR (No match)
296 - 311	1930.0580	1929.0507	1928.9566	0.0941	0	FYAAEIILGLEHMHNR Oxidation (M) (No match)
317 - 332	1827.0731	1826.0658	1825.9798	0.0860	0	DLKPANILLDEHGHVR (Ions score 24)
317 - 332	1827.0731	1826.0658	1825.9798	0.0860	0	DLKPANILLDEHGHVR (No match)
405 - 422	2069.0984	2068.0911	2067.9856	0.1055	0	MTLTMAVELPDSFSPELR 2 Oxidation (M) (No match)
423 - 431	1028.6526	1027.6453	1027.6025	0.0428	0	SLLEGLLQR (No match)
466 - 474	1049.6644	1048.6571	1048.6069	0.0502	0	YPPPLIPPR (Ions score 11)
466 - 474	1049.6644	1048.6571	1048.6069	0.0502	0	YPPPLIPPR (No match)
498 - 507	1251.6810	1250.6737	1250.6142	0.0595	0	LLDSDQELYR (No match)
508 - 516	1076.6222	1075.6149	1075.5662	0.0488	0	NFPLTISER (No match)
579 - 587	1275.7231	1274.7158	1274.6560	0.0599	1	RYFYLFPNR (No match)
580 - 587	1119.6123	1118.6050	1118.5549	0.0502	0	YFYLFPNR (No match)
580 - 587	1119.6123	1118.6050	1118.5549	0.0502	0	YFYLFPNR (Ions score 20)
649 - 660	1476.8285	1475.8212	1475.7480	0.0732	1	DAYREAQQLVQR (No match)
653 - 660	971.5718	970.5645	970.5195	0.0450	0	EAQQLVQR (No match)
670 - 683	1551.0004	1549.9931	1549.9191	0.0740	1	SPVVELSKVPLVQR (No match)
670 - 683	1551.0004	1549.9931	1549.9191	0.0740	1	SPVVELSKVPLVQR (Ions score 34)

Mascot: http://www.matrixscience.com/

Spot 221

{MATRIX}{SCIENCE} Mascot Search Results

Protein View

Match to: **gi|22477159** Score: **228** Expect: **3e-018**
Sec23 homolog A (S. cerevisiae) [Homo sapiens]

Nominal mass (M_r): **87022;** Calculated pI value: **6.64**
NCBI BLAST search of gi|22477159 against nr
Unformatted sequence string for pasting into other applications

Taxonomy: Homo sapiens
Links to retrieve other entries containing this sequence from NCBI Entrez:
gi|123979998 from synthetic construct
gi|123993343 from synthetic construct
gi|123994759 from synthetic construct

Fixed modifications: Carbamidomethyl (C)
Variable modifications: Oxidation (M)
Cleavage by Trypsin: cuts C-term side of KR unless next residue is P
Sequence Coverage: **15%**

Matched peptides shown in **Bold Red**

```
  1 MTTYLEFIQQ NEERDGVRFS WNVWPSSRLE ATRMVVPVAA LFTPLKERPD
 51 LPPIQYEPVL CSRTTCRAVL NPLCQVDYRA KLWACNFCYQ RNQFPPSYAG
101 ISELNQPAEL LPQFSSIEYV VLRGPQMPLI FLYVVDTCME DEDLQALKES
151 MQMSLSLLPP TALVGLITFG RMVQVHELGC EGISKSYVFR GTKDLSAKQL
201 QEMLGLSKVP VTQATRGPQV QQPPPSNRFL QPVQKIDMNL TDLLGELQRD
251 PWPVPQGKRP LRSSGVALSI AVGLLECTFP NTGARIMMFI GGPATQGPGM
301 VVGDELKTPI RSWHDIDKDN AKYVKKGTKH FEALANRAAT TGHVIDIYAC
351 ALDQTGLLEM KCCPNLTGGY MVMGDSFNTS LFKQTFQRVF TKDMHGQFKM
401 GFGGTLEIKT SREIKISGAI GPCVSLNSKG PCVSENEIGT GGTCQWKICG
451 LSPTTTLAIY FEVVNQHNAP IPQGGRGAIQ FVTQYQHSSG QRRIRVTTIA
501 RNWADAQTQI QNIAASFDQE AAAILMARLA IYRAETEEGP DVLRWLDRQL
551 IRLCQKFGEY HKDDPSSFRF SETFSLYPQF MFHLRRSSFL QVFNNSPDES
601 SYYRHHFMRQ DLTQSLIMIQ PIMYAYSFSG PPEPVLLDSS SILADRILLM
651 DTFFQILIYH GETIAQWRKS GYQDMPEYEN FRHLLQAPVD DAQEILHSRF
701 PMPRYIDTEH GGSQARFLLS KVNPSQTHNN MYAWGQESGA PILTDDVSLQ
751 VFMDHLKKLA VSSAA
```

Residue Number Increasing Mass Decreasing Mass

Start - End	Observed	Mr(expt)	Mr(calc)	Delta	Miss	Sequence
47 - 63	2069.0129	2068.0056	2068.0411	-0.0355	0	ERPDLPPIQYEPVLCSR (No match)
68 - 79	1447.7207	1446.7134	1446.7289	-0.0155	0	AVLNPLCQVDYR (No match)
217 - 228	1304.6606	1303.6533	1303.6633	-0.0099	0	GPQVQQPPPSNR (No match)
217 - 228	1304.6606	1303.6533	1303.6633	-0.0099	0	GPQVQQPPPSNR (Ions score 4)
477 - 492	1806.8676	1805.8603	1805.8808	-0.0205	0	GAIQFVTQYQHSSGQR (Ions score 47)
477 - 492	1806.8676	1805.8603	1805.8808	-0.0205	0	GAIQFVTQYQHSSGQR (No match)
557 - 569	1584.6914	1583.6841	1583.7004	-0.0163	1	FGEYHKDDPSSFR (No match)
587 - 604	2139.9448	2138.9375	2138.9544	-0.0169	0	SSFLQVFNNSPDESSYYR (No match)
587 - 604	2139.9448	2138.9375	2138.9544	-0.0169	0	SSFLQVFNNSPDESSYYR (Ions score 46)
670 - 682	1651.6494	1650.6421	1650.6620	-0.0198	0	SGYQDMPEYENFR Oxidation (M) (No match)
670 - 682	1651.6494	1650.6421	1650.6620	-0.0198	0	SGYQDMPEYENFR Oxidation (M) (Ions score 41)
683 - 699	1941.9926	1940.9853	1941.0068	-0.0215	0	HLLQAPVDDAQEILHSR (No match)

Mascot: http://www.matrixscience.com/

Spot 223

{MATRIX SCIENCE} Mascot Search Results

Protein View

Match to: **gi|386758** Score: **599** Expect: **2.4e-055**
GRP78 precursor

Nominal mass (M_r): **72185**; Calculated pI value: **5.03**
NCBI BLAST search of gi|386758 against nr
Unformatted sequence string for pasting into other applications

Taxonomy: Homo sapiens
Links to retrieve other entries containing this sequence from NCBI Entrez:
gi|1143492 from Homo sapiens

Fixed modifications: Carbamidomethyl (C)
Variable modifications: Oxidation (M)
Cleavage by Trypsin: cuts C-term side of KR unless next residue is P
Sequence Coverage: **41%**

Matched peptides shown in **Bold Red**

```
  1 MKLSLVAAML LLLSAARAEE EDKKEDVGTV VGIDLGTTYS CVGVFKNGRV
 51 EIIANDQGNR ITPSYVAFTP EGERLIGDAA KNQLTSNPEN TVFDAKRLIG
101 RTWNDPSVQQ DIKFLPFKVV EKKTKPYIQV DIGGGQTKTF APEEISAMVL
151 TKMKETAEAY LGKKVTHAVV TVPAYFNDAQ RQATKDAGTI AGLNVMRIIN
201 EPTAAAIAYG LDKREGEKNI LVFDLGGGTF DVSLLTIDNG VFEVVATNGD
251 THLGGEDFDQ RVMEHFIKLY KKKTGKDVRK DNRAVQKLRR EVEKAKALSS
301 QHQARIEIES FYEGEDFSET LTRAKFEELN MDLFRSTMKP VQKVLEDSDL
351 KKSDIDEIVL VGGSTRIPKI QQLVKEFFNG KEPSRGINPD EAVAYGAAVQ
401 AGVLSGDQDT GDLVLLHVCP LTLGIETVGG VMTKLIPSNT VVPTKNSQIF
451 STASDNQPTV TIKVYEGERP LTKDNHLLGT FDLTGIPPAP RGVPQIEVTF
501 EIDVNGILRV TAEDKGTGNK NKITITNDQN RLTPEEIERM VNDAEKFAEE
551 DKKLKERIDT RNELESYAYS LKNQIGDKEK LGGKLSSEDK ETMEKAVEEK
601 IEWLESHQDA DIEDFKAKKK ELEEIVQPII SKLYGSAGPP PTGEEDTAEK
651 DEL
```

Residue Number Increasing Mass Decreasing Mass

Start - End	Observed	Mr(expt)	Mr(calc)	Delta	Miss	Sequence
47 - 60	1555.8912	1554.8839	1554.7862	0.0977	1	NGRVEIIANDQGNR (No match)
50 - 60	1228.7008	1227.6935	1227.6207	0.0728	0	VEIIANDQGNR (No match)
61 - 74	1566.8756	1565.8683	1565.7725	0.0958	0	ITPSYVAFTPEGER (Ions score 70)
61 - 74	1566.8756	1565.8683	1565.7725	0.0958	0	ITPSYVAFTPEGER (No match)
124 - 138	1604.9631	1603.9558	1603.8569	0.0989	0	TKPYIQVDIGGGQTK (No match)
139 - 152	1552.8846	1551.8773	1551.7854	0.0919	0	TFAPEEISAMVLTK Oxidation (M) (No match)
164 - 181	2016.1909	2015.1836	2015.0588	0.1248	1	KVTHAVVTVPAYFNDAQR (No match)
165 - 181	1888.0880	1887.0807	1886.9638	0.1169	0	VTHAVVTVPAYFNDAQR (No match)
165 - 181	1888.0880	1887.0807	1886.9638	0.1169	0	VTHAVVTVPAYFNDAQR (Ions score 64)
186 - 197	1233.6947	1232.6874	1232.6183	0.0692	0	DAGTIAGLNVMR Oxidation (M) (No match)
198 - 214	1816.1082	1815.1009	1814.9890	0.1120	1	IINEPTAAAIAYGLDKR (Ions score 62)
198 - 214	1816.1082	1815.1009	1814.9890	0.1120	1	IINEPTAAAIAYGLDKR (No match)
262 - 268	919.5204	918.5131	918.4633	0.0499	0	VMEHFIK Oxidation (M) (No match)
297 - 305	997.5767	996.5694	996.5101	0.0594	0	ALSSQHQAR (No match)
306 - 323	2165.1248	2164.1175	2163.9847	0.1328	0	IEIESFYEGEDFSETLTR (No match)
324 - 335	1528.8374	1527.8301	1527.7391	0.0910	1	AKFEELNMDLFR Oxidation (M) (Ions score 27)
324 - 335	1528.8374	1527.8301	1527.7391	0.0910	1	AKFEELNMDLFR Oxidation (M) (No match)
326 - 335	1329.6909	1328.6836	1328.6070	0.0766	0	FEELNMDLFR Oxidation (M) (No match)
336 - 351	1834.0238	1833.0165	1832.9553	0.0612	1	STMKPVQKVLEDSDLK Oxidation (M) (No match)
352 - 366	1588.9525	1587.9452	1587.8467	0.0985	1	KSDIDEIVLVGGSTR (No match)
353 - 366	1460.8491	1459.8418	1459.7518	0.0901	0	SDIDEIVLVGGSTR (No match)
376 - 385	1210.6583	1209.6510	1209.5778	0.0733	1	EFFNGKEPSR (No match)
464 - 473	1191.7095	1190.7022	1190.6295	0.0728	0	VYEGERPLTK (No match)
474 - 491	1934.1331	1933.1258	1933.0057	0.1201	0	DNHLLGTFDLTGIPPAPR (Ions score 75)
474 - 491	1934.1331	1933.1258	1933.0057	0.1201	0	DNHLLGTFDLTGIPPAPR (No match)
521 - 531	1316.7321	1315.7248	1315.6844	0.0404	1	NKITITNDQNR (No match)
523 - 531	1074.6179	1073.6106	1073.5465	0.0641	0	ITITNDQNR (No match)
532 - 539	986.5687	985.5614	985.5080	0.0534	0	LTPEEIER (No match)
621 - 632	1397.8694	1396.8621	1396.7813	0.0808	0	ELEEIVQPIISK (No match)
633 - 653	2176.1396	2175.1323	2174.9855	0.1469	1	LYGSAGPPPTGEEDTAEKDEL (No match)

Mascot: http://www.matrixscience.com/

Spot 225

MATRIX SCIENCE **Mascot Search Results**

Protein View

Match to: **gi|386758** Score: **551** Expect: **1.5e-050**
GRP78 precursor

Nominal mass (M_r): **72185;** Calculated pI value: **5.03**
NCBI BLAST search of gi|386758 against nr
Unformatted sequence string for pasting into other applications

Taxonomy: Homo sapiens
Links to retrieve other entries containing this sequence from NCBI Entrez:
gi|1143492 from Homo sapiens

Fixed modifications: Carbamidomethyl (C)
Variable modifications: Oxidation (M)
Cleavage by Trypsin: cuts C-term side of KR unless next residue is P
Sequence Coverage: **46%**

Matched peptides shown in **Bold Red**

```
  1 MKLSLVAAML LLLSAARAEE EDKKEDVGTV VGIDLGTTYS CVGVFKNGRV
 51 EIIANDQGNR ITPSYVAFTP EGERLIGDAA KNQLTSNPEN TVFDAKRLIG
101 RTWNDPSVQQ DIKFLPFKVV EKKTKPYIQV DIGGGQTKTF APEEISAMVL
151 TKMKETAEAY LGKKVTHAVV TVPAYFNDAQ RQATKDAGTI AGLNVMRIIN
201 EPTAAAIAYG LDKREGEKNI LVFDLGGGTF DVSLLTIDNG VFEVVATNGD
251 THLGGEDFDQ RVMEHFIKLY KKKTGKDVRK DNRAVQKLRR EVEKAKALSS
301 QHQARIEIES FYEGEDFSET LTRAKFEELN MDLFRSTMKP VQKVLEDSDL
351 KKSDIDEIVL VGGSTRIPKI QQLVKEFFNG KEPSRGINPD EAVAYGAAVQ
401 AGVLSGDQDT GDLVLLHVCP LTLGIETVGG VMTKLIPSNT VVPTKNSQIF
451 STASDNQPTV TIKVYEGERP LTKDNHLLGT FDLTGIPPAP RGVPQIEVTF
501 EIDVNGILRV TAEDKGTGNK NKITITNDQN RLTPEEIERM VNDAEKFAEE
551 DKKLKERIDT RNELESYAYS LKNQIGDKEK LGGKLSSEDK ETMEKAVEEK
601 IEWLESHQDA DIEDFKAKKK ELEEIVQPII SKLYGSAGPP PTGEEDTAEK
651 DEL
```

Residue Number Increasing Mass Decreasing Mass

Start - End	Observed	Mr(expt)	Mr(calc)	Delta	Miss	Sequence
47 - 60	1555.8782	1554.8709	1554.7862	0.0847	1	NGRVEIIANDQGNR (No match)
50 - 60	1228.6964	1227.6891	1227.6207	0.0684	0	VEIIANDQGNR (No match)
61 - 74	1566.8663	1565.8590	1565.7725	0.0865	0	ITPSYVAFTPEGER (No match)
61 - 74	1566.8663	1565.8590	1565.7725	0.0865	0	ITPSYVAFTPEGER (Ions score 55)
82 - 96	1677.9163	1676.9090	1676.8005	0.1085	0	NQLTSNPENTVFDAK (No match)
102 - 113	1430.7653	1429.7580	1429.6837	0.0743	0	TWNDPSVQQDIK (No match)
124 - 138	1604.9521	1603.9448	1603.8569	0.0879	0	TKPYIQVDIGGGQTK (No match)
139 - 152	1552.8721	1551.8648	1551.7854	0.0794	0	TFAPEEISAMVLTK Oxidation (M) (No match)
164 - 181	2016.1768	2015.1695	2015.0588	0.1107	1	KVTHAVVTVPAYFNDAQR (No match)
165 - 181	1888.0762	1887.0689	1886.9638	0.1051	0	VTHAVVTVPAYFNDAQR (Ions score 64)
165 - 181	1888.0762	1887.0689	1886.9638	0.1051	0	VTHAVVTVPAYFNDAQR (No match)
186 - 197	1233.6849	1232.6776	1232.6183	0.0594	0	DAGTIAGLNVMR Oxidation (M) (No match)
198 - 214	1816.0953	1815.0880	1814.9890	0.0991	1	IINEPTAAAIAYGLDKR (No match)
198 - 214	1816.0953	1815.0880	1814.9890	0.0991	1	IINEPTAAAIAYGLDKR (Ions score 41)
297 - 305	997.5690	996.5617	996.5101	0.0517	0	ALSSQHQAR (No match)
306 - 323	2165.1077	2164.1004	2163.9847	0.1157	0	IEIESFYEGEDFSETLTR (No match)
324 - 335	1528.8282	1527.8209	1527.7391	0.0818	1	AKFEELNMDLFR Oxidation (M) (No match)
324 - 335	1528.8282	1527.8209	1527.7391	0.0818	1	AKFEELNMDLFR Oxidation (M) (Ions score 24)
326 - 335	1329.6818	1328.6745	1328.6070	0.0675	0	FEELNMDLFR Oxidation (M) (No match)
336 - 351	1834.0132	1833.0059	1832.9553	0.0506	1	STMKPVQKVLEDSDLK Oxidation (M) (No match)
352 - 366	1588.9402	1587.9329	1587.8467	0.0862	1	KSDIDEIVLVGGSTR (No match)
353 - 366	1460.8387	1459.8314	1459.7518	0.0797	0	SDIDEIVLVGGSTR (No match)
376 - 385	1210.6490	1209.6417	1209.5778	0.0640	1	EFFNGKEPSR (No match)
464 - 473	1191.6979	1190.6906	1190.6295	0.0612	0	VYEGERPLTK (No match)
474 - 491	1934.1210	1933.1137	1933.0057	0.1080	0	DNHLLGTFDLTGIPPAPR (No match)
474 - 491	1934.1210	1933.1137	1933.0057	0.1080	0	DNHLLGTFDLTGIPPAPR (Ions score 51)
521 - 531	1316.7103	1315.7030	1315.6844	0.0186	1	NKITITNDQNR (No match)
523 - 531	1074.6115	1073.6042	1073.5465	0.0577	0	ITITNDQNR (No match)
532 - 539	986.5655	985.5582	985.5080	0.0502	0	LTPEEIER (No match)
558 - 572	1802.0006	1800.9933	1800.8893	0.1040	1	IDTRNELESYAYSLK (No match)
621 - 632	1397.8608	1396.8535	1396.7813	0.0722	0	ELEEIVQPIISK (No match)
633 - 653	2176.1265	2175.1192	2174.9855	0.1338	1	LYGSAGPPPTGEEDTAEKDEL (No match)

Mascot: http://www.matrixscience.com/

Spot 226

{MATRIX}{SCIENCE} Mascot Search Results

Protein View

Match to: **gi|16507237** Score: **720** Expect: **1.9e-067**
heat shock 70kDa protein 5 [Homo sapiens]

Nominal mass (M_r): **72402**; Calculated pI value: **5.07**
NCBI BLAST search of gi|16507237 against nr
Unformatted sequence string for pasting into other applications

Taxonomy: Homo sapiens
Links to retrieve other entries containing this sequence from NCBI Entrez:
gi|109112231 from Macaca mulatta
gi|114626688 from Pan troglodytes
gi|114626690 from Pan troglodytes
gi|14916999 from Homo sapiens
gi|7229462 from Homo sapiens
gi|6900104 from Homo sapiens
gi|18044381 from Homo sapiens
gi|86559365 from Homo sapiens
gi|119608026 from Homo sapiens
gi|123982716 from synthetic construct
gi|123997385 from synthetic construct

Fixed modifications: Carbamidomethyl (C)
Variable modifications: Oxidation (M)
Cleavage by Trypsin: cuts C-term side of KR unless next residue is P
Sequence Coverage: **53%**

Matched peptides shown in **Bold Red**

1 MKLSLVAAML LLLSAARAEE EDKKEDVGTV VGIDLGTTYS CVGVFK**NGRV**
51 **EIIANDQGNR ITPSYVAFTP EGER**LIGDAA K**NQLTSNPEN TVFDAKR**LIG
101 R**TWNDPSVQQ DIK**FLPFKVV EKK**TKPYIQV DIGGGQTKTF APEEISAMVL**
151 **TK**MKETAEAY LGK**KVTHAVV TVPAYFNDAQ** RQATK**DAGTI AGLNVMRIIN**
201 **EPTAAAIAYG LDKR**EGEKNI LVFDLGGGTF DVSLLTIDNG VFEVVATNGD
251 THLGGEDFDQ R**VMEHFIK**LY KKKTGKDVRK DNRAVQKLRR EVEKAKR**ALS**
301 **SQHQARIEIE SFYEGEDFSE TLTRAKFEEL NMDLFR**STMK PVQK**VLEDSD**
351 **LKKSDIDEIV LVGGSTR**IPK IQQLVK**EFFN GKEPSR**GINP DEAVAYGAAV
401 QAGVLSGDQD TGDLVLLDVC PLTLGIETVG GVMTKLIPRN TVVPTKK**SQI**
451 **FSTASDNQPT VTIKVYEGER PLTKDNHLLG TFDLTGIPPA PR**GVPQIEVT

```
501 FEIDVNGILR VTAEDKGTGN KNKITITNDQ NRLTPEEIER MVNDAEKFAE
551 EDKKLKERID TRNELESYAY SLKNQIGDKE KLGGKLSSED KETMEKAVEE
601 KIEWLESHQD ADIEDFKAKK KELEEIVQPI ISKLYGSAGP PPTGEEDTAE
651 KDEL
```

Residue Number Increasing Mass Decreasing Mass

Start - End	Observed	Mr(expt)	Mr(calc)	Delta	Miss	Sequence
47 - 60	1555.7788	1554.7715	1554.7862	-0.0147	1	NGRVEIIANDQGNR (No match)
50 - 60	1228.6204	1227.6131	1227.6207	-0.0076	0	VEIIANDQGNR (No match)
61 - 74	1566.7644	1565.7571	1565.7725	-0.0154	0	ITPSYVAFTPEGER (Ions score 54)
61 - 74	1566.7644	1565.7571	1565.7725	-0.0154	0	ITPSYVAFTPEGER (No match)
82 - 96	1677.7961	1676.7888	1676.8005	-0.0117	0	NQLTSNPENTVFDAK (No match)
82 - 97	1833.8905	1832.8832	1832.9016	-0.0184	1	NQLTSNPENTVFDAKR (No match)
102 - 113	1430.6754	1429.6681	1429.6837	-0.0156	0	TWNDPSVQQDIK (No match)
124 - 138	1604.8490	1603.8417	1603.8569	-0.0152	0	TKPYIQVDIGGGQTK (No match)
139 - 152	1536.7793	1535.7720	1535.7905	-0.0185	0	TFAPEEISAMVLTK (No match)
164 - 181	2016.0471	2015.0398	2015.0588	-0.0190	1	KVTHAVVTVPAYFNDAQR (No match)
165 - 181	1887.9514	1886.9441	1886.9638	-0.0197	0	VTHAVVTVPAYFNDAQR (No match)
165 - 181	1887.9514	1886.9441	1886.9638	-0.0197	0	VTHAVVTVPAYFNDAQR (Ions score 90)
186 - 197	1217.6206	1216.6133	1216.6234	-0.0100	0	DAGTIAGLNVMR (No match)
186 - 197	1233.6084	1232.6011	1232.6183	-0.0171	0	DAGTIAGLNVMR Oxidation (M) (No match)
198 - 214	1815.9786	1814.9713	1814.9890	-0.0176	1	IINEPTAAAIAYGLDKR (Ions score 93)
198 - 214	1815.9786	1814.9713	1814.9890	-0.0176	1	IINEPTAAAIAYGLDKR (No match)
262 - 268	903.4664	902.4591	902.4684	-0.0092	0	VMEHFIK (No match)
298 - 306	997.5153	996.5080	996.5101	-0.0020	0	ALSSQHQAR (No match)
307 - 324	2164.9700	2163.9627	2163.9847	-0.0220	0	IEIESFYEGEDFSETLTR (No match)
325 - 336	1512.7377	1511.7304	1511.7442	-0.0138	1	AKFEELNMDLFR (No match)
325 - 336	1528.7321	1527.7248	1527.7391	-0.0143	1	AKFEELNMDLFR Oxidation (M) (No match)
327 - 336	1313.6116	1312.6043	1312.6121	-0.0078	0	FEELNMDLFR (No match)
345 - 352	918.4724	917.4651	917.4705	-0.0054	0	VLEDSDLK (No match)
353 - 367	1588.8353	1587.8280	1587.8467	-0.0187	1	KSDIDEIVLVGGSTR (Ions score 64)
353 - 367	1588.8353	1587.8280	1587.8467	-0.0187	1	KSDIDEIVLVGGSTR (No match)
354 - 367	1460.7448	1459.7375	1459.7518	-0.0142	0	SDIDEIVLVGGSTR (No match)
377 - 386	1210.5760	1209.5687	1209.5778	-0.0090	1	EFFNGKEPSR (Ions score 67)
377 - 386	1210.5760	1209.5687	1209.5778	-0.0090	1	EFFNGKEPSR (No match)
448 - 464	1836.9087	1835.9014	1835.9265	-0.0250	0	SQIFSTASDNQPTVTIK (No match)
465 - 474	1191.6266	1190.6193	1190.6295	-0.0101	0	VYEGERPLTK (No match)
475 - 492	1933.9937	1932.9864	1933.0057	-0.0193	0	DNHLLGTFDLTGIPPAPR (No match)
524 - 532	1074.5476	1073.5403	1073.5465	-0.0062	0	ITITNDQNR (No match)
533 - 540	986.5092	985.5019	985.5080	-0.0061	0	LTPEEIER (No match)
548 - 554	866.4184	865.4111	865.4181	-0.0070	1	FAEEDKK (No match)
563 - 573	1316.6254	1315.6181	1315.6295	-0.0114	0	NELESYAYSLK (No match)
574 - 581	931.4764	930.4691	930.4770	-0.0079	1	NQIGDKEK (No match)
602 - 619	2174.0205	2173.0132	2173.0327	-0.0195	1	IEWLESHQDADIEDFKAK (No match)

622 - 633	1397.7734	1396.7661	1396.7813	-0.0152	0	ELEEIVQPIISK	(No match)
634 - 654	2175.9934	2174.9861	2174.9855	0.0007	1	LYGSAGPPPTGEEDTAEKDEL	(No match)

Mascot: http://www.matrixscience.com/

Spot 228

{MATRIX}{SCIENCE} Mascot Search Results

Protein View

Match to: **gi|119624227** Score: **103** Expect: **9.6e-006**
differentially expressed in FDCP 6 homolog (mouse), isoform CRA_b [Homo sapiens]

Nominal mass (M_r): **44508;** Calculated pI value: **6.63**
NCBI BLAST search of gi|119624227 against nr
Unformatted sequence string for pasting into other applications

Taxonomy: Homo sapiens

Fixed modifications: Carbamidomethyl (C)
Variable modifications: Oxidation (M)
Cleavage by Trypsin: cuts C-term side of KR unless next residue is P
Sequence Coverage: **15%**

Matched peptides shown in **Bold Red**

```
  1 MICPPRPPKV LGLQVLPDRD GKRCMFCVKT ATRTYEMSAS DTRQRQEWTA
 51 AIQMAIRLQA EGKTSLHKDL KQKRREQREQ RERRRAAKEE ELLRLQQLQE
101 EKERKLQELE LLQEAQRQAE RLLQEEEERR RSQHRELQQA LEGQLREAEQ
151 ARASMQAEME LKEEEAARQR QRIKELEEMQ QRLQEALQLE VKARRDEESV
201 RIAQTRLLEE EEEKLKQLMQ LKEEQERYIE RAQQEKEELQ QEMAQQSRSL
251 QQAQQQLEEV RQNRQRADED VEAAQRKLRQ ASTNVKHWNV QMNRLMHPIE
301 PGDKRPVTSS SFSGFQPPLL AHRDSSLKRL TRWGSQGNRT PSPNSNEQQK
351 SLNGGDEAPA PASTPQEDKL DPAPEN
```

Residue Number Increasing Mass Decreasing Mass

Start - End	Observed	Mr(expt)	Mr(calc)	Delta	Miss	Sequence
105 - 117	1597.8306	1596.8233	1596.8835	-0.0601	1	**KLQELELLQEAQR** (Ions score 22)
105 - 117	1597.8306	1596.8233	1596.8835	-0.0601	1	**KLQELELLQEAQR** (No match)
136 - 146	1284.6425	1283.6352	1283.6833	-0.0481	0	**ELQQALEGQLR** (No match)
136 - 146	1284.6425	1283.6352	1283.6833	-0.0481	0	**ELQQALEGQLR** (Ions score 9)
232 - 248	2076.8911	2075.8838	2075.9541	-0.0703	1	**AQQEKEELQQEMAQQSR** Oxidation (M) (Ions score 2)
232 - 248	2076.8911	2075.8838	2075.9541	-0.0703	1	**AQQEKEELQQEMAQQSR** Oxidation (M) (No match)
249 - 261	1556.7468	1555.7395	1555.7954	-0.0559	0	**SLQQAQQQLEEVR** (No match)

249 - 261	1556.7468	1555.7395	1555.7954	-0.0559	0	SLQQAQQQLEEVR	(Ions score 1)
249 - 264	1954.9354	1953.9281	1953.9980	-0.0699	1	SLQQAQQQLEEVRQNR	(No match)
249 - 264	1954.9354	1953.9281	1953.9980	-0.0699	1	SLQQAQQQLEEVRQNR	(Ions score 6)

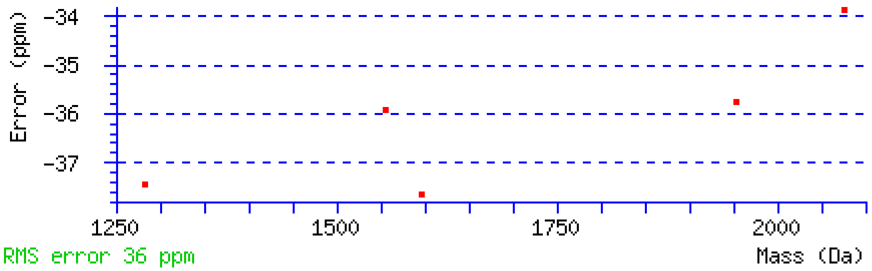

RMS error 36 ppm

Mascot: http://www.matrixscience.com/

Spot 229

{MATRIX SCIENCE} Mascot Search Results

Protein View

Match to: **gi|119581882** Score: **142** Expect: **1.2e-009**
lymphocyte cytosolic protein 2 (SH2 domain containing leukocyte protein of 76kDa), isoform CRA_a [H

Nominal mass (M_r): **59922**; Calculated pI value: **6.32**
NCBI BLAST search of gi|119581882 against nr
Unformatted sequence string for pasting into other applications

Taxonomy: Homo sapiens

Fixed modifications: Carbamidomethyl (C)
Variable modifications: Oxidation (M)
Cleavage by Trypsin: cuts C-term side of KR unless next residue is P
Sequence Coverage: **15%**

Matched peptides shown in **Bold Red**

```
  1 MALRNVPFRS EVLGWDPDSL ADYFKKLNYK DCEKAVKKYH IDGARFLNLT
 51 ENDIQKFPKL RVPILSKLSQ EINKNEERRS IFTRKPQVPR FPEETESHEE
101 DNGGWSSFEE DDYESPNDDQ DGEDDGDYES PNEEEEAPVE DDADYEPPPS
151 NDEEALQNSI LPAKPFPNSN SMYIDRPPSG KTPQQPPVPP QRPMAALPPP
201 PAGRNHSPLP PPQTNHEEPS RSRNHKTAKL PAPSIDRSTK PPLDRSLAPF
251 DREPFTLGKK PPFSDKPSIP AGRSLGEHLP KIQKPPLPPT TERHERSSPL
301 PGKKPPVPKH GWGPDRRENV MHQRPLPQPA LLPMSSNTFP SRSTKPSPMN
351 PLPSSHMPGA FSESNSSFPQ SASLPPYFSQ GPSNRPPIRA EGRNFPLPLP
401 NKPRPPSPAE EENSLNEEWY VSYITRPEAE AALRKINQDG TFLVRDSSKK
451 TTTNPYVLMV LYKDKVYNIQ IRYQKESQVY LLGTGLRGKE DFLSVSDIID
501 YFRKMPLLLI DGKNRGSRYQ CTLTHAAGYP
```

Residue Number Increasing Mass Decreasing Mass

Start - End	Observed	Mr(expt)	Mr(calc)	Delta	Miss	Sequence
205 - 221	1937.0475	1936.0402	1935.9187	0.1215	0	NHSPLPPPQTNHEEPSR (Ions score 19)
205 - 221	1937.0475	1936.0402	1935.9187	0.1215	0	NHSPLPPPQTNHEEPSR (No match)
246 - 259	1577.9379	1576.9306	1576.8249	0.1057	1	SLAPFDREPFTLGK (Ions score 13)
246 - 259	1577.9379	1576.9306	1576.8249	0.1057	1	SLAPFDREPFTLGK (No match)

260 - 273	1496.9214	1495.9141	1495.8147	0.0995	0	KPPFSDKPSIPAGR	(No match)
260 - 273	1496.9214	1495.9141	1495.8147	0.0995	0	KPPFSDKPSIPAGR	(Ions score 35)
435 - 445	1290.8025	1289.7952	1289.7091	0.0861	1	KINQDGTFLVR	(No match)
436 - 445	1162.6952	1161.6879	1161.6142	0.0738	0	INQDGTFLVR	(No match)
476 - 487	1335.8103	1334.8030	1334.7193	0.0837	0	ESQVYLLGTGLR	(No match)
476 - 487	1335.8103	1334.8030	1334.7193	0.0837	0	ESQVYLLGTGLR	(Ions score 5)
488 - 503	1904.0680	1903.0607	1902.9363	0.1245	1	GKEDFLSVSDIIDYFR	(No match)
488 - 503	1904.0680	1903.0607	1902.9363	0.1245	1	GKEDFLSVSDIIDYFR	(Ions score 10)

Mascot: http://www.matrixscience.com/

Spot 234

{MATRIX SCIENCE} Mascot Search Results

Protein View

Match to: **gi|41349456** Score: **125** Expect: **6.1e-008**
prolyl endopeptidase [Homo sapiens]

Nominal mass (M_r): **81560**; Calculated pI value: **5.53**
NCBI BLAST search of gi|41349456 against nr
Unformatted sequence string for pasting into other applications

Taxonomy: Homo sapiens
Links to retrieve other entries containing this sequence from NCBI Entrez:
gi|21040382 from Homo sapiens
gi|55665552 from Homo sapiens
gi|56068195 from Homo sapiens
gi|56204735 from Homo sapiens
gi|57208661 from Homo sapiens
gi|119568811 from Homo sapiens
gi|123993503 from synthetic construct
gi|124000465 from synthetic construct

Fixed modifications: Carbamidomethyl (C)
Variable modifications: Oxidation (M)
Cleavage by Trypsin: cuts C-term side of KR unless next residue is P
Sequence Coverage: **11%**

Matched peptides shown in **Bold Red**

```
  1 MLSLQYPDVY RDETAVQDYH GHKICDPYAW LEDPDSEQTK AFVEAQNKIT
 51 VPFLEQCPIR GLYKERMTEL YDYPKYSCHF KKGKRYFYFY NTGLQNQRVL
101 YVQDSLEGEA RVFLDPNILS DDGTVALRGY AFSEDGEYFA YGLSASGSDW
151 VTIKFMKVDG AKELPDVLER VKFSCMAWTH DGKGMFYNSY PQQDGKSDGT
201 ETSTNLHQKL YYHVLGTDQS EDILCAEFPD EPKWMGGAEL SDDGRYVLLS
251 IREGCDPVNR LWYCDLQQES SGIAGILKWV KLIDNFEGEY DYVTNEGTVF
301 TFKTNRQSPN YRVINIDFRD PEESKWKVLV PEHEKDVLEW IACVRSNFLV
351 LCYLHDVKNI LQLHDLTTGA LLKTFPLDVG SIVGYSGQKK DTEIFYQFTS
401 FLSPGIIYHC DLTKEELEPR VFREVTVKGI DASDYQTVQI FYPSKDGTKI
451 PMFIVHKKGI KLDGSHPAFL YGYGGFNISI TPNYSVSRLI FVRHMGGILA
501 VANIRGGGEY GETWHKGGIL ANKQNCFDDF QCAAEYLIKE GYTSPKRLTI
551 NGGSNGGLLV AACANQRPDL FGCVIAQVGV MDMLKFHKYT IGHAWTTDYG
601 CSDSKQHFEW LVKYSPLHNV KLPEADDIQY PSMLLLTADH DDRVVPLHSL
```

651 KFIATLQYIV GRSRKQSNPL LIHVDTKAGH GAGKPTAKVI EEVSDMFAFI
701 ARCLNVDWIP

Residue Number Increasing Mass Decreasing Mass

Start - End	Observed	Mr(expt)	Mr(calc)	Delta	Miss	Sequence
49 - 60	1472.7190	1471.7117	1471.7857	-0.0740	0	ITVPFLEQCPIR (Ions score 26)
49 - 60	1472.7190	1471.7117	1471.7857	-0.0740	0	ITVPFLEQCPIR (No match)
86 - 98	1713.7144	1712.7071	1712.7946	-0.0875	0	YFYFYNTGLQNQR (No match)
86 - 98	1713.7144	1712.7071	1712.7946	-0.0875	0	YFYFYNTGLQNQR (Ions score 26)
99 - 111	1478.6776	1477.6703	1477.7412	-0.0709	0	VLYVQDSLEGEAR (No match)
112 - 128	1844.8673	1843.8600	1843.9679	-0.1079	0	VFLDPNILSDDGTVALR (No match)
652 - 662	1280.6737	1279.6664	1279.7288	-0.0624	0	FIATLQYIVGR (No match)
652 - 662	1280.6737	1279.6664	1279.7288	-0.0624	0	FIATLQYIVGR (Ions score 9)
689 - 702	1642.7327	1641.7254	1641.8072	-0.0817	0	VIEEVSDMFAFIAR Oxidation (M) (No match)

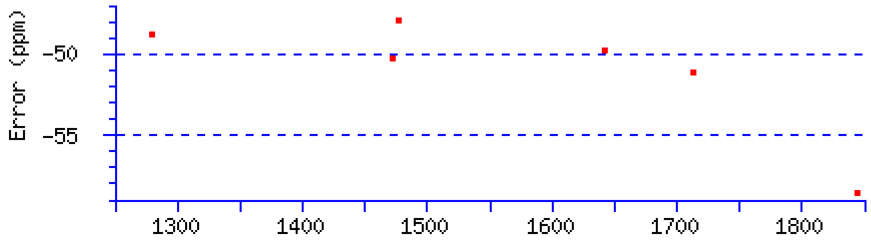

RMS error 50 ppm

Mascot: http://www.matrixscience.com/

Spot 237

MATRIX SCIENCE Mascot Search Results

Protein View

Match to: **gi|119625804** Score: **650** Expect: **1.9e-060**
moesin, isoform CRA_b [Homo sapiens]

Nominal mass (M_r): **66678**; Calculated pI value: **5.90**
NCBI BLAST search of gi|119625804 against nr
Unformatted sequence string for pasting into other applications

Taxonomy: Homo sapiens

Fixed modifications: Carbamidomethyl (C)
Variable modifications: Oxidation (M)
Cleavage by Trypsin: cuts C-term side of KR unless next residue is P
Sequence Coverage: **48%**

Matched peptides shown in **Bold Red**

```
  1 MDAELEFAIQ PNTTGKQLFD QVVKTIGLRE VWFFGLQYQD TKGFSTWLKL
 51 NKKVTAQDVR KESPLLFKFR AKFYPEDVSE ELIQDITQRL FFLQVKEGIL
101 NDDIYCPPET AVLLASYAVQ SKYGDFNKEV HKSGYLAGDK LLPQRVLEQH
151 KLNKDQWEER IQVWHEEHRG MLREDAVLEY LKIAQDLEMY GVNYFSIKNK
201 KGSELWLGVD ALGLNIYEQN DRLTPKIGFP WSEIRNISFN DKKFVIKPID
251 KKAPDFVFYA PRLRINKRIL ALCMGNHELY MRRRKPDTIE VQQMKAQARE
301 EKHQKQMERA MLENEKKKRE MAEKEKEKIE REKEELMERL KQIEEQTKKA
351 QQELEEQTRR ALELEQERKR AQSEAEKLAK ERQEAEEAKE ALLQASRDQK
401 KTQEQLALEM AELTARISQL EMARQKKESE AVEWQQKAQM VQEDLEKTRA
451 ELKTAMSTPH VAEPAENEQD EQDENGAEAS ADLRADAMAK DRSEEERTTE
501 AEKNERVQKH LKALTSELAN ARDESKKTAN DMIHAENMRL GRDKYKTLRQ
551 IRQGNTKQRI DEFESM
```

Residue Number Increasing Mass Decreasing Mass

Start - End	Observed	Mr(expt)	Mr(calc)	Delta	Miss	Sequence
17 - 24	976.5394	975.5321	975.5389	-0.0068	0	QLFDQVVK (No match)
30 - 42	1660.8077	1659.8004	1659.7932	0.0072	0	EVWFFGLQYQDTK (No match)
54 - 60	788.4310	787.4237	787.4188	0.0049	0	VTAQDVR (No match)

62 - 70	1136.5713	1135.5640	1135.6389	-0.0749	1	**ESPLLFKFR** (No match)
73 - 89	2081.9966	2080.9893	2080.9953	-0.0059	0	**FYPEDVSEELIQDITQR** (Ions score 127)
73 - 89	2081.9966	2080.9893	2080.9953	-0.0059	0	**FYPEDVSEELIQDITQR** (No match)
90 - 96	894.5364	893.5291	893.5374	-0.0083	0	**LFFLQVK** (No match)
146 - 154	1108.5802	1107.5729	1107.6400	-0.0671	1	**VLEQHKLNK** (No match)
152 - 160	1217.5940	1216.5867	1216.5836	0.0031	1	**LNKDQWEER** (No match)
155 - 160	862.3735	861.3662	861.3617	0.0046	0	**DQWEER** (No match)
161 - 169	1233.6055	1232.5982	1232.6050	-0.0068	0	**IQVWHEEHR** (No match)
161 - 169	1233.6055	1232.5982	1232.6050	-0.0068	0	**IQVWHEEHR** (Ions score 47)
170 - 182	1536.8046	1535.7973	1535.8017	-0.0044	1	**GMLREDAVLEYLK** (No match)
170 - 182	1552.8062	1551.7989	1551.7966	0.0023	1	**GMLREDAVLEYLK** Oxidation (M) (No match)
183 - 198	1890.9236	1889.9163	1889.9233	-0.0069	0	**IAQDLEMYGVNYFSIK** (No match)
183 - 198	1906.9253	1905.9180	1905.9182	-0.0001	0	**IAQDLEMYGVNYFSIK** Oxidation (M) (No match)
202 - 222	2362.1479	2361.1406	2361.1600	-0.0194	0	**GSELWLGVDALGLNIYEQNDR** (No match)
227 - 235	1104.5828	1103.5755	1103.5763	-0.0008	0	**IGFPWSEIR** (Ions score 45)
227 - 235	1104.5828	1103.5755	1103.5763	-0.0008	0	**IGFPWSEIR** (No match)
244 - 251	959.5272	958.5199	958.5851	-0.0652	0	**FVIKPIDK** (No match)
252 - 262	1310.6880	1309.6807	1309.6818	-0.0011	1	**KAPDFVFYAPR** (Ions score 74)
252 - 262	1310.6880	1309.6807	1309.6818	-0.0011	1	**KAPDFVFYAPR** (No match)
253 - 262	1182.5935	1181.5862	1181.5869	-0.0007	0	**APDFVFYAPR** (Ions score 69)
253 - 262	1182.5935	1181.5862	1181.5869	-0.0007	0	**APDFVFYAPR** (No match)
269 - 282	1720.8492	1719.8419	1719.8259	0.0161	0	**ILALCMGNHELYMR** (No match)
269 - 282	1736.8309	1735.8236	1735.8208	0.0029	0	**ILALCMGNHELYMR** Oxidation (M) (No match)
269 - 282	1752.8319	1751.8246	1751.8157	0.0090	0	**ILALCMGNHELYMR** 2 Oxidation (M) (No match)
284 - 295	1472.7882	1471.7809	1471.7817	-0.0007	1	**RKPDTIEVQQMK** (No match)
284 - 295	1488.7810	1487.7737	1487.7766	-0.0028	1	**RKPDTIEVQQMK** Oxidation (M) (No match)
332 - 339	1063.5154	1062.5081	1062.5015	0.0066	1	**EKEELMER** (No match)
332 - 339	1079.5144	1078.5071	1078.4964	0.0107	1	**EKEELMER** Oxidation (M) (No match)
350 - 359	1231.5928	1230.5855	1230.5840	0.0015	0	**AQQELEEQTR** (No match)
350 - 360	1387.6913	1386.6840	1386.6851	-0.0011	1	**AQQELEEQTRR** (No match)
361 - 368	987.5135	986.5062	986.5032	0.0030	0	**ALELEQER** (No match)
381 - 389	1089.5205	1088.5132	1088.5097	0.0035	1	**ERQEAEEAK** (No match)
401 - 416	1831.9521	1830.9448	1830.9509	-0.0061	1	**KTQEQLALEMAELTAR** (No match)
401 - 416	1847.9585	1846.9512	1846.9458	0.0054	1	**KTQEQLALEMAELTAR** Oxidation (M) (No match)
402 - 416	1703.8567	1702.8494	1702.8559	-0.0065	0	**TQEQLALEMAELTAR** (No match)
402 - 416	1719.8582	1718.8509	1718.8508	0.0001	0	**TQEQLALEMAELTAR** Oxidation (M) (No match)
417 - 424	947.4953	946.4880	946.4906	-0.0025	0	**ISQLEMAR** (No match)
417 - 424	963.4922	962.4849	962.4855	-0.0006	0	**ISQLEMAR** Oxidation (M) (No match)
498 - 506	1077.5164	1076.5091	1076.5098	-0.0006	1	**TTEAEKNER** (No match)
513 - 522	1045.5646	1044.5573	1044.5563	0.0010	0	**ALTSELANAR** (No match)
558 - 566	1154.5106	1153.5033	1153.5073	-0.0040	1	**QRIDEFESM** (No match)
558 - 566	1170.5114	1169.5041	1169.5022	0.0019	1	**QRIDEFESM** Oxidation (M) (No match)

**Mascot:** http://www.matrixscience.com/

Spot 238

{MATRIX SCIENCE} Mascot Search Results

Protein View

Match to: **gi|62898171** Score: **348** Expect: **3e-030**
L-plastin variant [Homo sapiens]

Nominal mass (M_r): **70785;** Calculated pI value: **5.20**
NCBI BLAST search of gi|62898171 against nr
Unformatted sequence string for pasting into other applications

Taxonomy: Homo sapiens

Fixed modifications: Carbamidomethyl (C)
Variable modifications: Oxidation (M)
Cleavage by Trypsin: cuts C-term side of KR unless next residue is P
Sequence Coverage: **37%**

Matched peptides shown in **Bold Red**

```
  1 MARGSVSDEE MMELREAFAK VDTDGNGYIS FNELNDLFKA ACLPLPGYRV
 51 REITENLMAT GDLDQDGRIS FDEFIKIFHG LKSTDVAKTF RKAINKKEGI
101 CAIGGTSEQS SVGTQHSYSE EEKYAFVNWI NKALENDPDC RHVIPMNPNT
151 NDLFNAVGDG IVLCKMINLS VPDTIDERTI NKKKLTPFTI QENLNLALNS
201 ASAIGCHVVN IGAEDLKEGK PYLVLGLLWQ VIKIGLFADI ELSRNEALIA
251 LLREGESLED LMKLSPEELL LRWANYHLEN AGCNKIGNFS TDIKDSKAYY
301 HLLEQVAPKG DEEGVPAVVI DMSGLREKDD IQRAECMLQQ AERLGCRQFV
351 TATDVVRGNP KLNLAFIANL FNRYPALHKP ENQDIDWGAL EGETREERTF
401 RNWMNSLGVN PRVNHLYGDL SDALVIFQLY EKIKVPVDWN RVNKPPYPKL
451 GGNMKKLENC NYAVELGKNQ AKFSLVGIGG QDLNEGNRTL TLALIWQLMR
501 RYTLNILEEI GGGQKVNDDI IVNWVNETLR EAEKSSSISS FKDPKISTSL
551 PVLDLIDAIQ PGSINYDLLK TENLNDDEKL NNAKYAISMA RKIGARVYAL
601 PEDLVEVNPK MVMTVFACLM GKGMKRV
```

Residue Number Increasing Mass Decreasing Mass

Start - End	Observed	Mr(expt)	Mr(calc)	Delta	Miss	Sequence
4 - 15	1414.6586	1413.6513	1413.5751	0.0762	0	GSVSDEEMMELR 2 Oxidation (M) (No match)
21 - 39	2161.1411	2160.1338	2160.0010	0.1328	0	VDTDGNGYISFNELNDLFK (No match)

40 - 49	1117.6439	1116.6366	1116.5750	0.0617	0	AACLPLPGYR (Ions score 22)
40 - 49	1117.6439	1116.6366	1116.5750	0.0617	0	AACLPLPGYR (No match)
52 - 68	1893.9702	1892.9629	1892.8421	0.1208	0	EITENLMATGDLDQDGR Oxidation (M) (No match)
69 - 76	998.5673	997.5600	997.5120	0.0480	0	ISFDEFIK (No match)
166 - 178	1518.8301	1517.8228	1517.7395	0.0833	0	MINLSVPDTIDER Oxidation (M) (Ions score 20)
166 - 178	1518.8301	1517.8228	1517.7395	0.0833	0	MINLSVPDTIDER Oxidation (M) (No match)
234 - 244	1233.7499	1232.7426	1232.6764	0.0662	0	IGLFADIELSR (No match)
245 - 253	1012.6671	1011.6598	1011.6076	0.0522	0	NEALIALLR (Ions score 33)
245 - 253	1012.6671	1011.6598	1011.6076	0.0522	0	NEALIALLR (No match)
264 - 272	1069.6836	1068.6763	1068.6179	0.0585	0	LSPEELLLR (No match)
298 - 309	1431.8390	1430.8317	1430.7557	0.0760	0	AYYHLLEQVAPK (No match)
310 - 326	1759.9584	1758.9511	1758.8457	0.1054	0	GDEEGVPAVVIDMSGLR Oxidation (M) (No match)
327 - 333	903.5015	902.4942	902.4457	0.0485	1	EKDDIQR (No match)
348 - 357	1135.6716	1134.6643	1134.6033	0.0611	0	QFVTATDVVR (No match)
362 - 373	1405.8760	1404.8687	1404.7877	0.0810	0	LNLAFIANLFNR (No match)
362 - 373	1405.8760	1404.8687	1404.7877	0.0810	0	LNLAFIANLFNR (Ions score 47)
402 - 412	1303.6930	1302.6857	1302.6138	0.0719	0	NWMNSLGVNPR Oxidation (M) (No match)
433 - 441	1126.6915	1125.6842	1125.6294	0.0548	1	IKVPVDWNR (No match)
473 - 488	1675.9352	1674.9279	1674.8324	0.0955	0	FSLVGIGGQDLNEGNR (No match)
473 - 488	1675.9352	1674.9279	1674.8324	0.0955	0	FSLVGIGGQDLNEGNR (Ions score 61)
502 - 515	1534.8912	1533.8839	1533.8038	0.0801	0	YTLNILEEIGGGQK (No match)
585 - 591	827.4414	826.4341	826.4007	0.0334	0	YAISMAR Oxidation (M) (No match)
597 - 610	1585.9384	1584.9311	1584.8398	0.0913	0	VYALPEDLVEVNPK (No match)

Mascot: http://www.matrixscience.com/

Spot 239

{MATRIX SCIENCE} Mascot Search Results

Protein View

Match to: **gi|14530105** Score: **201** Expect: **1.5e-015**
OVARIAN/Breast septin gamma [Homo sapiens]

Nominal mass (M_r): **64894;** Calculated pI value: **7.21**
NCBI BLAST search of gi|14530105 against nr
Unformatted sequence string for pasting into other applications

Taxonomy: Homo sapiens
Links to retrieve other entries containing this sequence from NCBI Entrez:
gi|119609873 from Homo sapiens

Fixed modifications: Carbamidomethyl (C)
Variable modifications: Oxidation (M)
Cleavage by Trypsin: cuts C-term side of KR unless next residue is P
Sequence Coverage: **18%**

Matched peptides shown in **Bold Red**

```
  1 MSDPAVNAQL DGIISDFEAL KRSFEVEEVE TPNSTPPRRV QTPLLRATVA
 51 SSTQKFQDLG VKNSEPSARH VDSLSQRSPK ASLRRVELSG PKAAEPVSRR
101 TELSIDISSK QVENAGAIGP SRFGLKRAEV LGHKTPEPAP RRTEITIVKP
151 QESAHRRMEP PASKVPEVPT APATDAAPKR VEIQMPKPAE APTAPSPAQT
201 LENSEPAPVS QLQSRLEPKP QPPVAEATPR SQEATEAAPS CVGDMADTPR
251 DAGLKQAPAS RNEKAPVDFG YVGIDSILEQ MRRKAMKQGF EFNIMVVGQS
301 GLGKSTLINT LFKSKISRKS VQPTSEERIP KTIEIKSITH DIEEKGVRMK
351 LTVIDTPGFG DHINNENCWQ PIMKFINDQY EKYLQEEVNI NRKKRIPDTR
401 VHCCLYFIPA TGHSLRPLDI EFMKRLSKVV NIVPVIAKAD TLTLEERVHF
451 KQRITADLLS NGIDVYPQKE FDEDSEDRLV NEKFREMIPF AVVGSDHEYQ
501 VNGKRILGRK TKWGTIEVEN TTHCEFAYLR DLLIRTHMQN IKDITSSIHF
551 EAYRVKRLNE GSSAMANGVE EKEPEAPEM
```

Residue Number Increasing Mass Decreasing Mass

Start - End	Observed	Mr(expt)	Mr(calc)	Delta	Miss	Sequence
23 - 38	1817.8997	1816.8924	1816.8479	0.0446	0	SFEVEEVETPNSTPPR (No match)

23 - 38	1817.8997	1816.8924	1816.8479	0.0446	0	SFEVEEVETPNSTPPR (Ions score 57)
23 - 39	1974.0070	1972.9997	1972.9490	0.0508	1	SFEVEEVETPNSTPPRR (No match)
39 - 46	982.6241	981.6168	981.6083	0.0085	1	RVQTPLLR (No match)
111 - 122	1198.6449	1197.6376	1197.6101	0.0275	0	QVENAGAIGPSR (No match)
142 - 156	1765.0083	1764.0010	1763.9642	0.0369	1	RTEITIVKPQESAHR (Ions score 2)
143 - 157	1765.0083	1764.0010	1763.9642	0.0369	1	TEITIVKPQESAHRR (No match)
216 - 230	1629.9366	1628.9293	1628.8885	0.0408	0	LEPKPQPPVAEATPR (Ions score 27)
216 - 230	1629.9366	1628.9293	1628.8885	0.0408	0	LEPKPQPPVAEATPR (No match)
375 - 382	1056.5187	1055.5114	1055.4923	0.0191	0	FINDQYEK (No match)
383 - 392	1277.6803	1276.6730	1276.6411	0.0319	0	YLQEEVNINR (No match)
429 - 438	1051.7227	1050.7154	1050.6800	0.0354	0	VVNIVPVIAK (No match)
543 - 554	1438.7328	1437.7255	1437.6888	0.0367	0	DITSSIHFEAYR (Ions score 7)
543 - 554	1438.7328	1437.7255	1437.6888	0.0367	0	DITSSIHFEAYR (No match)

Mascot: http://www.matrixscience.com/

Spot 240

{MATRIX SCIENCE} Mascot Search Results

Protein View

Match to: **gi|4505257** Score: **354** Expect: **7.7e-031**
moesin [Homo sapiens]

Nominal mass (M_r): **67892;** Calculated pI value: **6.08**
NCBI BLAST search of gi|4505257 against nr
Unformatted sequence string for pasting into other applications

Taxonomy: Homo sapiens
Links to retrieve other entries containing this sequence from NCBI Entrez:
gi|127234 from Homo sapiens
gi|188626 from Homo sapiens
gi|5419633 from Homo sapiens
gi|16878176 from Homo sapiens
gi|119625803 from Homo sapiens
gi|123984467 from synthetic construct
gi|123998433 from synthetic construct

Fixed modifications: Carbamidomethyl (C)
Variable modifications: Oxidation (M)
Cleavage by Trypsin: cuts C-term side of KR unless next residue is P
Sequence Coverage: **37%**

Matched peptides shown in **Bold Red**

1 **MPKTISVRVT TMDAELEFAI QPNTTGKQLF DQVVK**TIGLR EVWFFGLQYQ
51 DTKGFSTWLK LNKKVTAQDV RKESPLLFKF RAKFYPEDVS EELIQDITQR
101 **LFFLQVK**EGI LNDDIYCPPE TAVLLASYAV QSK**YGDFNKE VHK**SGYLAGD
151 KLLPQR**VLEQ HKLNK**DQWEE R**IQVWHEEHR GMLREDAVLE YLKIAQDLEM**
201 **YGVNYFSIK**N KKGSELWLGV DALGLNIYEQ NDRLTPK**IGF PWSEIR**NISF
251 NDKK**FVIKPI DKKAPDFVFY APR**LRINKR**I LALCMGNHEL YMR**R**RKPDTI**
301 **EVQQMK**AQAR EEKHQKQMER AMLENEKKKR EMAEKEKEKI ER**EKEELMER**
351 **LK**QIEEQTKK **AQQELEEQTR RALELEQER**K RAQSEAEKLA KERQEAEEAK
401 EALLQASRDQ K**KTQEQLALE MAELTAR**ISQ LEMARQKKES EAVEWQQKAQ
451 MVQEDLEKTR AELKTAMSTP HVAEPAENEQ DEQDENGAEA SADLRADAMA
501 K**DRSEEERTT EAEKNER**VQK HLKALTSELA NARDESKKTA NDMIHAENMR
551 LGRDKYKTLR QIRQGNTKQR IDEFESM

Residue Number Increasing Mass Decreasing Mass

Start - End	Observed	Mr(expt)	Mr(calc)	Delta	Miss	Sequence
1 - 8	947.5193	946.5120	946.5269	-0.0149	1	MPKTISVR Oxidation (M) (No match)
9 - 27	2066.0627	2065.0554	2065.0037	0.0517	0	VTTMDAELEFAIQPNTTGK (No match)
9 - 27	2082.0398	2081.0325	2080.9986	0.0339	0	VTTMDAELEFAIQPNTTGK Oxidation (M) (No match)
28 - 35	976.5630	975.5557	975.5389	0.0168	0	QLFDQVVK (No match)
101 - 107	894.5624	893.5551	893.5374	0.0177	0	LFFLQVK (No match)
134 - 143	1236.5770	1235.5697	1235.5934	-0.0237	1	YGDFNKEVHK (No match)
157 - 165	1108.6040	1107.5967	1107.6400	-0.0433	1	VLEQHKLNK (No match)
172 - 180	1233.6371	1232.6298	1232.6050	0.0248	0	IQVWHEEHR (No match)
172 - 180	1233.6371	1232.6298	1232.6050	0.0248	0	IQVWHEEHR (Ions score 31)
181 - 193	1536.8429	1535.8356	1535.8017	0.0339	1	GMLREDAVLEYLK (No match)
181 - 193	1552.8401	1551.8328	1551.7966	0.0362	1	GMLREDAVLEYLK Oxidation (M) (No match)
194 - 209	1890.9741	1889.9668	1889.9233	0.0436	0	IAQDLEMYGVNYFSIK (No match)
238 - 246	1104.6089	1103.6016	1103.5763	0.0253	0	IGFPWSEIR (Ions score 34)
238 - 246	1104.6089	1103.6016	1103.5763	0.0253	0	IGFPWSEIR (No match)
255 - 262	959.5455	958.5382	958.5851	-0.0469	0	FVIKPIDK (No match)
263 - 273	1310.7162	1309.7089	1309.6818	0.0271	1	KAPDFVFYAPR (No match)
263 - 273	1310.7162	1309.7089	1309.6818	0.0271	1	KAPDFVFYAPR (Ions score 21)
264 - 273	1182.6206	1181.6133	1181.5869	0.0264	0	APDFVFYAPR (Ions score 65)
264 - 273	1182.6206	1181.6133	1181.5869	0.0264	0	APDFVFYAPR (No match)
280 - 293	1720.8761	1719.8688	1719.8259	0.0430	0	ILALCMGNHELYMR (No match)
280 - 293	1720.8761	1719.8688	1719.8259	0.0430	0	ILALCMGNHELYMR (Ions score 4)
280 - 293	1736.8658	1735.8585	1735.8208	0.0378	0	ILALCMGNHELYMR Oxidation (M) (No match)
295 - 306	1472.8137	1471.8064	1471.7817	0.0248	1	RKPDTIEVQQMK (No match)
343 - 350	1079.5317	1078.5244	1078.4964	0.0280	1	EKEELMER Oxidation (M) (No match)
345 - 352	1063.5372	1062.5299	1062.5379	-0.0080	1	EELMERLK Oxidation (M) (No match)
361 - 371	1387.7213	1386.7140	1386.6851	0.0289	1	AQQELEEQTRR (No match)
372 - 379	987.5342	986.5269	986.5032	0.0237	0	ALELEQER (No match)
412 - 427	1831.9934	1830.9861	1830.9509	0.0352	1	KTQEQLALEMAELTAR (No match)
413 - 427	1703.9014	1702.8941	1702.8559	0.0382	0	TQEQLALEMAELTAR (No match)
502 - 508	920.4352	919.4279	919.3995	0.0284	1	DRSEEER (No match)
509 - 517	1077.5463	1076.5390	1076.5098	0.0293	1	TTEAEKNER (No match)

Mascot: http://www.matrixscience.com/

Spot 241

{MATRIX SCIENCE} Mascot Search Results

Protein View

Match to: **gi|119625804** Score: **573** Expect: **9.6e-053**
moesin, isoform CRA_b [Homo sapiens]

Nominal mass (M_r): **66678**; Calculated pI value: **5.90**
NCBI BLAST search of gi|119625804 against nr
Unformatted sequence string for pasting into other applications

Taxonomy: Homo sapiens

Fixed modifications: Carbamidomethyl (C)
Variable modifications: Oxidation (M)
Cleavage by Trypsin: cuts C-term side of KR unless next residue is P
Sequence Coverage: **46%**

Matched peptides shown in **Bold Red**

```
  1 MDAELEFAIQ PNTTGKQLFD QVVKTIGLRE VWFFGLQYQD TKGFSTWLKL
 51 NKKVTAQDVR KESPLLFKFR AKFYPEDVSE ELIQDITQRL FFLQVKEGIL
101 NDDIYCPPET AVLLASYAVQ SKYGDFNKEV HKSGYLAGDK LLPQRVLEQH
151 KLNKDQWEER IQVWHEEHRG MLREDAVLEY LKIAQDLEMY GVNYFSIKNK
201 KGSELWLGVD ALGLNIYEQN DRLTPKIGFP WSEIRNISFN DKKFVIKPID
251 KKAPDFVFYA PRLRINKRIL ALCMGNHELY MRRRKPDTIE VQQMKAQARE
301 EKHQKQMERA MLENEKKKRE MAEKEKEKIE REKEELMERL KQIEEQTKKA
351 QQELEEQTRR ALELEQERKR AQSEAEKLAK ERQEAEEAKE ALLQASRDQK
401 KTQEQLALEM AELTARISQL EMARQKKESE AVEWQQKAQM VQEDLEKTRA
451 ELKTAMSTPH VAEPAENEQD EQDENGAEAS ADLRADAMAK DRSEEERTTE
501 AEKNERVQKH LKALTSELAN ARDESKKTAN DMIHAENMRL GRDKYKTLRQ
551 IRQGNTKQRI DEFESM
```

Residue Number Increasing Mass Decreasing Mass

Start - End	Observed	Mr(expt)	Mr(calc)	Delta	Miss	Sequence	
17 - 24	976.5344	975.5271	975.5389	-0.0118	0	QLFDQVVK	(No match)
43 - 49	838.4374	837.4301	837.4384	-0.0083	0	GFSTWLK	(No match)
54 - 61	916.5175	915.5102	915.5137	-0.0035	1	VTAQDVRK	(No match)

71 - 89	2281.1057	2280.0984	2280.1273	-0.0289	1	AKFYPEDVSEELIQDITQR (No match)
73 - 89	2081.9788	2080.9715	2080.9953	-0.0237	0	FYPEDVSEELIQDITQR (No match)
90 - 96	894.5344	893.5271	893.5374	-0.0103	0	LFFLQVK (No match)
133 - 145	1417.7708	1416.7635	1416.7724	-0.0089	1	SGYLAGDKLLPQR (No match)
152 - 160	1217.5857	1216.5784	1216.5836	-0.0052	1	LNKDQWEER (No match)
155 - 160	862.3639	861.3566	861.3617	-0.0050	0	DQWEER (No match)
161 - 169	1233.6024	1232.5951	1232.6050	-0.0099	0	IQVWHEEHR (No match)
161 - 169	1233.6024	1232.5951	1232.6050	-0.0099	0	IQVWHEEHR (Ions score 39)
170 - 182	1536.7986	1535.7913	1535.8017	-0.0104	1	GMLREDAVLEYLK (No match)
170 - 182	1552.7870	1551.7797	1551.7966	-0.0169	1	GMLREDAVLEYLK Oxidation (M) (No match)
227 - 235	1104.5776	1103.5703	1103.5763	-0.0060	0	IGFPWSEIR (Ions score 41)
227 - 235	1104.5776	1103.5703	1103.5763	-0.0060	0	IGFPWSEIR (No match)
236 - 243	965.4965	964.4892	964.4977	-0.0085	1	NISFNDKK (No match)
252 - 262	1310.6799	1309.6726	1309.6818	-0.0092	1	KAPDFVFYAPR (Ions score 62)
252 - 262	1310.6799	1309.6726	1309.6818	-0.0092	1	KAPDFVFYAPR (No match)
253 - 262	1182.5887	1181.5814	1181.5869	-0.0055	0	APDFVFYAPR (Ions score 80)
253 - 262	1182.5887	1181.5814	1181.5869	-0.0055	0	APDFVFYAPR (No match)
269 - 282	1720.8207	1719.8134	1719.8259	-0.0124	0	ILALCMGNHELYMR (Ions score 52)
269 - 282	1720.8207	1719.8134	1719.8259	-0.0124	0	ILALCMGNHELYMR (No match)
269 - 282	1736.8140	1735.8067	1735.8208	-0.0140	0	ILALCMGNHELYMR Oxidation (M) (No match)
284 - 295	1472.7800	1471.7727	1471.7817	-0.0089	1	RKPDTIEVQQMK (No match)
332 - 339	1063.5038	1062.4965	1062.5015	-0.0050	1	EKEELMER (No match)
350 - 359	1231.5895	1230.5822	1230.5840	-0.0018	0	AQQELEEQTR (No match)
350 - 360	1387.6823	1386.6750	1386.6851	-0.0101	1	AQQELEEQTRR (No match)
361 - 368	987.5048	986.4975	986.5032	-0.0057	0	ALELEQER (No match)
381 - 389	1089.5139	1088.5066	1088.5097	-0.0031	1	ERQEAEEAK (No match)
383 - 397	1672.8369	1671.8296	1671.8427	-0.0131	1	QEAEEAKEALLQASR (No match)
390 - 397	887.4919	886.4846	886.4872	-0.0026	0	EALLQASR (No match)
401 - 416	1831.9401	1830.9328	1830.9509	-0.0181	1	KTQEQLALEMAELTAR (No match)
401 - 416	1847.9360	1846.9287	1846.9458	-0.0171	1	KTQEQLALEMAELTAR Oxidation (M) (No match)
402 - 416	1703.8480	1702.8407	1702.8559	-0.0152	0	TQEQLALEMAELTAR (No match)
402 - 416	1719.8367	1718.8294	1718.8508	-0.0214	0	TQEQLALEMAELTAR Oxidation (M) (No match)
417 - 424	947.4926	946.4853	946.4906	-0.0052	0	ISQLEMAR (No match)
417 - 424	963.4886	962.4813	962.4855	-0.0042	0	ISQLEMAR Oxidation (M) (No match)
491 - 497	920.4011	919.3938	919.3995	-0.0057	1	DRSEEER (No match)
498 - 506	1077.5111	1076.5038	1076.5098	-0.0059	1	TTEAEKNER (No match)
513 - 522	1045.5558	1044.5485	1044.5563	-0.0078	0	ALTSELANAR (No match)
527 - 539	1530.7059	1529.6986	1529.7079	-0.0092	1	KTANDMIHAENMR (No match)
528 - 539	1402.6149	1401.6076	1401.6129	-0.0053	0	TANDMIHAENMR (No match)
558 - 566	1154.5054	1153.4981	1153.5073	-0.0092	1	QRIDEFESM (No match)
558 - 566	1170.5006	1169.4933	1169.5022	-0.0089	1	QRIDEFESM Oxidation (M) (No match)

Mascot: http://www.matrixscience.com/

Spot 242

{MATRIX SCIENCE} Mascot Search Results

Protein View

Match to: **gi|119625804** Score: **459** Expect: **2.4e-041**
moesin, isoform CRA_b [Homo sapiens]

Nominal mass (M_r): **66678;** Calculated pI value: **5.90**
NCBI BLAST search of gi|119625804 against nr
Unformatted sequence string for pasting into other applications

Taxonomy: Homo sapiens

Fixed modifications: Carbamidomethyl (C)
Variable modifications: Oxidation (M)
Cleavage by Trypsin: cuts C-term side of KR unless next residue is P
Sequence Coverage: **42%**

Matched peptides shown in **Bold Red**

```
  1 MDAELEFAIQ PNTTGKQLFD QVVKTIGLRE VWFFGLQYQD TKGFSTWLKL
 51 NKKVTAQDVR KESPLLFKFR AKFYPEDVSE ELIQDITQRL FFLQVKEGIL
101 NDDIYCPPET AVLLASYAVQ SKYGDFNKEV HKSGYLAGDK LLPQRVLEQH
151 KLNKDQWEER IQVWHEEHRG MLREDAVLEY LKIAQDLEMY GVNYFSIKNK
201 KGSELWLGVD ALGLNIYEQN DRLTPKIGFP WSEIRNISFN DKKFVIKPID
251 KKAPDFVFYA PRLRINKRIL ALCMGNHELY MRRRKPDTIE VQQMKAQARE
301 EKHQKQMERA MLENEKKKRE MAEKEKEKIE REKEELMERL KQIEEQTKKA
351 QQELEEQTRR ALELEQERKR AQSEAEKLAK ERQEAEEAKE ALLQASRDQK
401 KTQEQLALEM AELTARISQL EMARQKKESE AVEWQQKAQM VQEDLEKTRA
451 ELKTAMSTPH VAEPAENEQD EQDENGAEAS ADLRADAMAK DRSEEERTTE
501 AEKNERVQKH LKALTSELAN ARDESKKTAN DMIHAENMRL GRDKYKTLRQ
551 IRQGNTKQRI DEFESM
```

Residue Number Increasing Mass Decreasing Mass

Start - End	Observed	Mr(expt)	Mr(calc)	Delta	Miss	Sequence
17 - 24	976.5596	975.5523	975.5389	0.0134	0	QLFDQVVK (No match)
43 - 49	838.4649	837.4576	837.4384	0.0192	0	GFSTWLK (No match)
71 - 89	2281.1692	2280.1619	2280.1273	0.0346	1	AKFYPEDVSEELIQDITQR (No match)

73 - 89	2082.0396	2081.0323	2080.9953	0.0371	0	FYPEDVSEELIQDITQR (No match)
90 - 96	894.5574	893.5501	893.5374	0.0127	0	LFFLQVK (No match)
123 - 132	1236.5601	1235.5528	1235.5934	-0.0406	1	YGDFNKEVHK (No match)
146 - 154	1108.6046	1107.5973	1107.6400	-0.0427	1	VLEQHKLNK (No match)
152 - 160	1217.6195	1216.6122	1216.5836	0.0286	1	LNKDQWEER (No match)
155 - 160	862.3934	861.3861	861.3617	0.0245	0	DQWEER (No match)
161 - 169	1233.6356	1232.6283	1232.6050	0.0233	0	IQVWHEEHR (Ions score 26)
161 - 169	1233.6356	1232.6283	1232.6050	0.0233	0	IQVWHEEHR (No match)
170 - 182	1536.8367	1535.8294	1535.8017	0.0277	1	GMLREDAVLEYLK (No match)
170 - 182	1552.8312	1551.8239	1551.7966	0.0273	1	GMLREDAVLEYLK Oxidation (M) (No match)
174 - 182	1079.5345	1078.5272	1078.5546	-0.0274	0	EDAVLEYLK (No match)
183 - 198	1890.9749	1889.9676	1889.9233	0.0444	0	IAQDLEMYGVNYFSIK (No match)
227 - 235	1104.6067	1103.5994	1103.5763	0.0231	0	IGFPWSEIR (No match)
227 - 235	1104.6067	1103.5994	1103.5763	0.0231	0	IGFPWSEIR (Ions score 33)
252 - 262	1310.7170	1309.7097	1309.6818	0.0279	1	KAPDFVFYAPR (Ions score 37)
252 - 262	1310.7170	1309.7097	1309.6818	0.0279	1	KAPDFVFYAPR (No match)
253 - 262	1182.6185	1181.6112	1181.5869	0.0243	0	APDFVFYAPR (Ions score 86)
253 - 262	1182.6185	1181.6112	1181.5869	0.0243	0	APDFVFYAPR (No match)
269 - 282	1720.8691	1719.8618	1719.8259	0.0360	0	ILALCMGNHELYMR (Ions score 15)
269 - 282	1720.8691	1719.8618	1719.8259	0.0360	0	ILALCMGNHELYMR (No match)
269 - 282	1736.8612	1735.8539	1735.8208	0.0332	0	ILALCMGNHELYMR Oxidation (M) (No match)
284 - 295	1472.8199	1471.8126	1471.7817	0.0310	1	RKPDTIEVQQMK (No match)
334 - 341	1063.5320	1062.5247	1062.5379	-0.0132	1	EELMERLK Oxidation (M) (No match)
350 - 359	1231.6520	1230.6447	1230.5840	0.0607	0	AQQELEEQTR (No match)
350 - 360	1387.7207	1386.7134	1386.6851	0.0283	1	AQQELEEQTRR (No match)
361 - 368	987.5297	986.5224	986.5032	0.0192	0	ALELEQER (No match)
401 - 416	1831.9923	1830.9850	1830.9509	0.0341	1	KTQEQLALEMAELTAR (No match)
402 - 416	1703.8995	1702.8922	1702.8559	0.0363	0	TQEQLALEMAELTAR (No match)
417 - 424	947.5137	946.5064	946.4906	0.0159	0	ISQLEMAR (No match)
417 - 424	963.5078	962.5005	962.4855	0.0150	0	ISQLEMAR Oxidation (M) (No match)
491 - 497	920.4300	919.4227	919.3995	0.0232	1	DRSEEER (No match)
498 - 506	1077.5413	1076.5340	1076.5098	0.0243	1	TTEAEKNER (No match)
528 - 539	1402.6519	1401.6446	1401.6129	0.0317	0	TANDMIHAENMR (No match)
558 - 566	1154.5353	1153.5280	1153.5073	0.0207	1	QRIDEFESM (No match)

Mascot: http://www.matrixscience.com/

Spot 244

MATRIX SCIENCE Mascot Search Results

Protein View

Match to: **gi|116805340** Score: **246** Expect: **4.8e-020**
glycyl-tRNA synthetase [Homo sapiens]

Nominal mass (M_r): **83854;** Calculated pI value: **6.61**
NCBI BLAST search of gi|116805340 against nr
Unformatted sequence string for pasting into other applications

Taxonomy: Homo sapiens

Fixed modifications: Carbamidomethyl (C)
Variable modifications: Oxidation (M)
Cleavage by Trypsin: cuts C-term side of KR unless next residue is P
Sequence Coverage: **23%**

Matched peptides shown in **Bold Red**

```
  1 MPSPRPVLLR GARAALLLLL PPRLLARPSL LLRRSLSAAS CPPISLPAAA
 51 SRSSMDGAGA EEVLAPLRLA VRQQGDLVRK LKEDKAPQVD VDKAVAELKA
101 RKRVLEAKEL ALQPKDDIVD RAKMEDTLKR RFFYDQAFAI YGGVSGLYDF
151 GPVGCALKNN IIQTWRQHFI QEEQILEIDC TMLTPEPVLK TSGHVDKFAD
201 FMVKDVKNGE CFRADHLLKA HLQKLMSDKK CSVEKKSEME SVLAQLDNYG
251 QQELADLFVN YNVKSPITGN DLSPPVSFNL MFKTFIGPGG NMPGYLRPET
301 AQGIFLNFKR LLEFNQGKLP FAAAQIGNSF RNEISPRSGL IRVREFTMAE
351 IEHFVDPSEK DHPKFQNVAD LHLYLYSAKA QVSGQSARKM RLGDAVEQGV
401 INNTVLGYFI GRIYLYLTKV GISPDKLRFR QHMENEMAHY ACDCWDAESK
451 TSYGWIEIVG CADRSCYDLS CHARATKVPL VAEKPLKEPK TVNVVQFEPS
501 KGAIGKAYKK DAKLVMEYLA ICDECYITEM EMLLNEKGEF TIETEGKTFQ
551 LTKDMINVKR FQKTLYVEEV VPNVIEPSFG LGRIMYTVFE HTFHVREGDE
601 QRTFFSFPAV VAPFKCSVLP LSQNQEFMPF VKELSEALTR HGVSHKVDDS
651 SGSIGRRYAR TDEIGVAFGV TIDFDTVNKT PHTATLRDRD SMRQIRAEIS
701 ELPSIVQDLA NGNITWADVE ARYPLFEGQE TGKKETIEE
```

Residue Number Increasing Mass Decreasing Mass

Start - End	Observed	Mr(expt)	Mr(calc)	Delta	Miss	Sequence

1 - 10	1165.7550	1164.7477	1164.6801	0.0676	0	MPSPRPVLLR (No match)
265 - 283	2080.0859	2079.0786	2079.0346	0.0440	0	SPITGNDLSPPVSFNLMFK Oxidation (M) (No match)
310 - 318	1104.6036	1103.5963	1103.6087	-0.0123	1	RLLEFNQGK (No match)
311 - 318	948.5179	947.5106	947.5076	0.0031	0	LLEFNQGK (No match)
311 - 331	2321.2668	2320.2595	2320.2327	0.0268	1	LLEFNQGKLPFAAAQIGNSFR (No match)
311 - 331	2321.2668	2320.2595	2320.2327	0.0268	1	LLEFNQGKLPFAAAQIGNSFR (Ions score 27)
319 - 331	1391.7697	1390.7624	1390.7357	0.0268	0	LPFAAAQIGNSFR (No match)
319 - 331	1391.7697	1390.7624	1390.7357	0.0268	0	LPFAAAQIGNSFR (Ions score 59)
365 - 379	1781.9594	1780.9521	1780.9147	0.0374	0	FQNVADLHLYLYSAK (No match)
392 - 412	2235.2090	2234.2017	2234.1694	0.0323	0	LGDAVEQGVINNTVLGYFIGR (No match)
413 - 419	913.5436	912.5363	912.5320	0.0043	0	IYLYLTK (No match)
420 - 428	984.5925	983.5852	983.5763	0.0089	1	VGISPDKLR (No match)
491 - 506	1673.9401	1672.9328	1672.9147	0.0181	1	TVNVVQFEPSKGAIGK (No match)
564 - 583	2218.2119	2217.2046	2217.1680	0.0366	0	TLYVEEVVPNVIEPSFGLGR (Ions score 35)
564 - 583	2218.2119	2217.2046	2217.1680	0.0366	0	TLYVEEVVPNVIEPSFGLGR (No match)
584 - 596	1695.8728	1694.8655	1694.8238	0.0417	0	IMYTVFEHTFHVR Oxidation (M) (No match)
603 - 615	1457.8052	1456.7979	1456.7754	0.0225	0	TFFSFPAVVAPFK (No match)
723 - 734	1396.7344	1395.7271	1395.7034	0.0238	1	YPLFEGQETGKK (No match)

Mascot: http://www.matrixscience.com/

Spot 246

{MATRIX SCIENCE} Mascot Search Results

Protein View

Match to: **gi|119625804** Score: **718** Expect: **3e-067**
moesin, isoform CRA_b [Homo sapiens]

Nominal mass (M_r): **66678**; Calculated pI value: **5.90**
NCBI BLAST search of gi|119625804 against nr
Unformatted sequence string for pasting into other applications

Taxonomy: Homo sapiens

Fixed modifications: Carbamidomethyl (C)
Variable modifications: Oxidation (M)
Cleavage by Trypsin: cuts C-term side of KR unless next residue is P
Sequence Coverage: **49%**

Matched peptides shown in **Bold Red**

```
  1 MDAELEFAIQ PNTTGKQLFD QVVKTIGLRE VWFFGLQYQD TKGFSTWLKL
 51 NKKVTAQDVR KESPLLFKFR AKFYPEDVSE ELIQDITQRL FFLQVKEGIL
101 NDDIYCPPET AVLLASYAVQ SKYGDFNKEV HKSGYLAGDK LLPQRVLEQH
151 KLNKDQWEER IQVWHEEHRG MLREDAVLEY LKIAQDLEMY GVNYFSIKNK
201 KGSELWLGVD ALGLNIYEQN DRLTPKIGFP WSEIRNISFN DKKFVIKPID
251 KKAPDFVFYA PRLRINKRIL ALCMGNHELY MRRRKPDTIE VQQMKAQARE
301 EKHQKQMERA MLENEKKKRE MAEKEKEKIE REKEELMERL KQIEEQTKKA
351 QQELEEQTRR ALELEQERKR AQSEAEKLAK ERQEAEEAKE ALLQASRDQK
401 KTQEQLALEM AELTARISQL EMARQKKESE AVEWQQKAQM VQEDLEKTRA
451 ELKTAMSTPH VAEPAENEQD EQDENGAEAS ADLRADAMAK DRSEEERTTE
501 AEKNERVQKH LKALTSELAN ARDESKKTAN DMIHAENMRL GRDKYKTLRQ
551 IRQGNTKQRI DEFESM
```

Residue Number Increasing Mass Decreasing Mass

Start - End	Observed	Mr(expt)	Mr(calc)	Delta	Miss	Sequence	
17 - 24	976.5382	975.5309	975.5389	-0.0080	0	QLFDQVVK	(No match)
43 - 49	838.4446	837.4373	837.4384	-0.0011	0	GFSTWLK	(No match)
54 - 61	916.5223	915.5150	915.5137	0.0013	1	VTAQDVRK	(No match)

71 - 89	2281.1328	2280.1255	2280.1273	-0.0018	1	**AKFYPEDVSEELIQDITQR** (No match)
73 - 89	2082.0012	2080.9939	2080.9953	-0.0013	0	**FYPEDVSEELIQDITQR** (No match)
90 - 96	894.5415	893.5342	893.5374	-0.0032	0	**LFFLQVK** (No match)
133 - 145	1417.7821	1416.7748	1416.7724	0.0024	1	**SGYLAGDKLLPQR** (No match)
152 - 160	1217.5936	1216.5863	1216.5836	0.0027	1	**LNKDQWEER** (No match)
155 - 160	862.3705	861.3632	861.3617	0.0016	0	**DQWEER** (No match)
161 - 169	1233.6108	1232.6035	1232.6050	-0.0015	0	**IQVWHEEHR** (No match)
161 - 169	1233.6108	1232.6035	1232.6050	-0.0015	0	**IQVWHEEHR** (Ions score 41)
170 - 182	1536.8109	1535.8036	1535.8017	0.0019	1	**GMLREDAVLEYLK** (No match)
170 - 182	1552.8071	1551.7998	1551.7966	0.0032	1	**GMLREDAVLEYLK** Oxidation (M) (No match)
227 - 235	1104.5844	1103.5771	1103.5763	0.0008	0	**IGFPWSEIR** (No match)
227 - 235	1104.5844	1103.5771	1103.5763	0.0008	0	**IGFPWSEIR** (Ions score 44)
236 - 243	965.5015	964.4942	964.4977	-0.0035	1	**NISFNDKK** (No match)
252 - 262	1310.6901	1309.6828	1309.6818	0.0010	1	**KAPDFVFYAPR** (Ions score 93)
252 - 262	1310.6901	1309.6828	1309.6818	0.0010	1	**KAPDFVFYAPR** (No match)
253 - 262	1182.5968	1181.5895	1181.5869	0.0026	0	**APDFVFYAPR** (Ions score 90)
253 - 262	1182.5968	1181.5895	1181.5869	0.0026	0	**APDFVFYAPR** (No match)
269 - 282	1720.8374	1719.8301	1719.8259	0.0043	0	**ILALCMGNHELYMR** (No match)
269 - 282	1736.8319	1735.8246	1735.8208	0.0039	0	**ILALCMGNHELYMR** Oxidation (M) (No match)
284 - 295	1472.7899	1471.7826	1471.7817	0.0010	1	**RKPDTIEVQQMK** (No match)
284 - 295	1488.7852	1487.7779	1487.7766	0.0014	1	**RKPDTIEVQQMK** Oxidation (M) (No match)
332 - 339	1063.5107	1062.5034	1062.5015	0.0019	1	**EKEELMER** (No match)
350 - 359	1231.5950	1230.5877	1230.5840	0.0037	0	**AQQELEEQTR** (No match)
350 - 360	1387.6920	1386.6847	1386.6851	-0.0004	1	**AQQELEEQTRR** (No match)
361 - 368	987.5114	986.5041	986.5032	0.0009	0	**ALELEQER** (No match)
381 - 389	1089.5227	1088.5154	1088.5097	0.0057	1	**ERQEAEEAK** (No match)
383 - 397	1672.8533	1671.8460	1671.8427	0.0033	1	**QEAEEAKEALLQASR** (No match)
390 - 397	887.4940	886.4867	886.4872	-0.0005	0	**EALLQASR** (No match)
401 - 416	1831.9574	1830.9501	1830.9509	-0.0008	1	**KTQEQLALEMAELTAR** (No match)
401 - 416	1831.9574	1830.9501	1830.9509	-0.0008	1	**KTQEQLALEMAELTAR** (Ions score 115)
401 - 416	1847.9542	1846.9469	1846.9458	0.0011	1	**KTQEQLALEMAELTAR** Oxidation (M) (No match)
402 - 416	1703.8650	1702.8577	1702.8559	0.0018	0	**TQEQLALEMAELTAR** (No match)
402 - 416	1719.8574	1718.8501	1718.8508	-0.0007	0	**TQEQLALEMAELTAR** Oxidation (M) (No match)
417 - 424	947.4993	946.4920	946.4906	0.0015	0	**ISQLEMAR** (No match)
417 - 424	963.4920	962.4847	962.4855	-0.0008	0	**ISQLEMAR** Oxidation (M) (No match)
438 - 449	1447.7285	1446.7212	1446.7136	0.0076	1	**AQMVQEDLEKTR** (No match)
491 - 497	920.4064	919.3991	919.3995	-0.0004	1	**DRSEEER** (No match)
498 - 506	1077.5162	1076.5089	1076.5098	-0.0008	1	**TTEAEKNER** (No match)
513 - 522	1045.5643	1044.5570	1044.5563	0.0007	0	**ALTSELANAR** (No match)
513 - 526	1504.7632	1503.7559	1503.7528	0.0031	1	**ALTSELANARDESK** (No match)
527 - 539	1530.7207	1529.7134	1529.7079	0.0056	1	**KTANDMIHAENMR** (No match)
528 - 539	1402.6224	1401.6151	1401.6129	0.0022	0	**TANDMIHAENMR** (No match)
558 - 566	1154.5143	1153.5070	1153.5073	-0.0003	1	**QRIDEFESM** (No match)
558 - 566	1170.5090	1169.5017	1169.5022	-0.0005	1	**QRIDEFESM** Oxidation (M) (No match)

Mascot: http://www.matrixscience.com/

Spot 247

{MATRIX SCIENCE} Mascot Search Results

Protein View

Match to: **gi|4505257** Score: **566** Expect: **4.8e-052**
moesin [Homo sapiens]

Nominal mass (M_r): **67892;** Calculated pI value: **6.08**
NCBI BLAST search of gi|4505257 against nr
Unformatted sequence string for pasting into other applications

Taxonomy: Homo sapiens
Links to retrieve other entries containing this sequence from NCBI Entrez:
gi|127234 from Homo sapiens
gi|188626 from Homo sapiens
gi|5419633 from Homo sapiens
gi|16878176 from Homo sapiens
gi|119625803 from Homo sapiens
gi|123984467 from synthetic construct
gi|123998433 from synthetic construct

Fixed modifications: Carbamidomethyl (C)
Variable modifications: Oxidation (M)
Cleavage by Trypsin: cuts C-term side of KR unless next residue is P
Sequence Coverage: **57%**

Matched peptides shown in **Bold Red**

```
  1 MPKTISVRVT TMDAELEFAI QPNTTGKQLF DQVVKTIGLR EVWFFGLQYQ
 51 DTKGFSTWLK LNKKVTAQDV RKESPLLFKF RAKFYPEDVS EELIQDITQR
101 LFFLQVKEGI LNDDIYCPPE TAVLLASYAV QSKYGDFNKE VHKSGYLAGD
151 KLLPQRVLEQ HKLNKDQWEE RIQVWHEEHR GMLREDAVLE YLKIAQDLEM
201 YGVNYFSIKN KKGSELWLGV DALGLNIYEQ NDRLTPKIGF PWSEIRNISF
251 NDKKFVIKPI DKKAPDFVFY APRLRINKRI LALCMGNHEL YMRRRKPDTI
301 EVQQMKAQAR EEKHQKQMER AMLENEKKKR EMAEKEKEKI EREKEELMER
351 LKQIEEQTKK AQQELEEQTR RALELEQERK RAQSEAEKLA KERQEAEEAK
401 EALLQASRDQ KKTQEQLALE MAELTARISQ LEMARQKKES EAVEWQQKAQ
451 MVQEDLEKTR AELKTAMSTP HVAEPAENEQ DEQDENGAEA SADLRADAMA
501 KDRSEEERTT EAEKNERVQK HLKALTSELA NARDESKKTA NDMIHAENMR
551 LGRDKYKTLR QIRQGNTKQR IDEFESM
```

Residue Number Increasing Mass Decreasing Mass

Start - End	Observed	Mr(expt)	Mr(calc)	Delta	Miss	Sequence
1 - 8	947.5184	946.5111	946.5269	-0.0158	1	MPKTISVR Oxidation (M) (No match)
9 - 27	2066.0569	2065.0496	2065.0037	0.0459	0	VTTMDAELEFAIQPNTTGK (No match)
9 - 27	2082.0330	2081.0257	2080.9986	0.0271	0	VTTMDAELEFAIQPNTTGK Oxidation (M) (No match)
28 - 35	976.5596	975.5523	975.5389	0.0134	0	QLFDQVVK (No match)
41 - 53	1660.8381	1659.8308	1659.7932	0.0376	0	EVWFFGLQYQDTK (No match)
54 - 60	838.4604	837.4531	837.4384	0.0147	0	GFSTWLK (No match)
65 - 72	916.5468	915.5395	915.5137	0.0258	1	VTAQDVRK (No match)
82 - 100	2281.1699	2280.1626	2280.1273	0.0353	1	AKFYPEDVSEELIQDITQR (No match)
101 - 107	894.5579	893.5506	893.5374	0.0132	0	LFFLQVK (No match)
134 - 143	1236.5563	1235.5490	1235.5934	-0.0444	1	YGDFNKEVHK (No match)
144 - 156	1417.8109	1416.8036	1416.7724	0.0312	1	SGYLAGDKLLPQR (No match)
157 - 165	1108.6024	1107.5951	1107.6400	-0.0449	1	VLEQHKLNK (No match)
163 - 171	1217.6190	1216.6117	1216.5836	0.0281	1	LNKDQWEER (No match)
166 - 171	862.3915	861.3842	861.3617	0.0226	0	DQWEER (No match)
172 - 180	1233.6329	1232.6256	1232.6050	0.0206	0	IQVWHEEHR (Ions score 36)
172 - 180	1233.6329	1232.6256	1232.6050	0.0206	0	IQVWHEEHR (No match)
181 - 193	1536.8400	1535.8327	1535.8017	0.0310	1	GMLREDAVLEYLK (No match)
181 - 193	1552.8372	1551.8299	1551.7966	0.0333	1	GMLREDAVLEYLK Oxidation (M) (No match)
185 - 193	1079.5337	1078.5264	1078.5546	-0.0282	0	EDAVLEYLK (No match)
194 - 209	1890.9644	1889.9571	1889.9233	0.0339	0	IAQDLEMYGVNYFSIK (No match)
238 - 246	1104.6058	1103.5985	1103.5763	0.0222	0	IGFPWSEIR (Ions score 41)
238 - 246	1104.6058	1103.5985	1103.5763	0.0222	0	IGFPWSEIR (No match)
247 - 254	965.5192	964.5119	964.4977	0.0142	1	NISFNDKK (No match)
263 - 273	1310.7166	1309.7093	1309.6818	0.0275	1	KAPDFVFYAPR (Ions score 51)
263 - 273	1310.7166	1309.7093	1309.6818	0.0275	1	KAPDFVFYAPR (No match)
264 - 273	1182.6195	1181.6122	1181.5869	0.0253	0	APDFVFYAPR (Ions score 80)
264 - 273	1182.6195	1181.6122	1181.5869	0.0253	0	APDFVFYAPR (No match)
280 - 293	1720.8698	1719.8625	1719.8259	0.0367	0	ILALCMGNHELYMR (No match)
280 - 293	1720.8698	1719.8625	1719.8259	0.0367	0	ILALCMGNHELYMR (Ions score 22)
280 - 293	1736.8674	1735.8601	1735.8208	0.0394	0	ILALCMGNHELYMR Oxidation (M) (No match)
295 - 306	1472.8198	1471.8125	1471.7817	0.0309	1	RKPDTIEVQQMK (No match)
295 - 306	1488.8140	1487.8067	1487.7766	0.0302	1	RKPDTIEVQQMK Oxidation (M) (No match)
345 - 352	1063.5305	1062.5232	1062.5379	-0.0147	1	EELMERLK Oxidation (M) (No match)
361 - 371	1387.7206	1386.7133	1386.6851	0.0282	1	AQQELEEQTRR (No match)
372 - 379	987.5313	986.5240	986.5032	0.0208	0	ALELEQER (No match)
394 - 408	1672.8811	1671.8738	1671.8427	0.0311	1	QEAEEAKEALLQASR (No match)
412 - 427	1831.9894	1830.9821	1830.9509	0.0312	1	KTQEQLALEMAELTAR (No match)
412 - 427	1847.9875	1846.9802	1846.9458	0.0344	1	KTQEQLALEMAELTAR Oxidation (M) (No match)
413 - 427	1703.8966	1702.8893	1702.8559	0.0334	0	TQEQLALEMAELTAR (No match)
413 - 427	1719.8884	1718.8811	1718.8508	0.0303	0	TQEQLALEMAELTAR Oxidation (M) (No match)
428 - 435	963.5083	962.5010	962.4855	0.0156	0	ISQLEMAR Oxidation (M) (No match)
502 - 508	920.4259	919.4186	919.3995	0.0191	1	DRSEEER (No match)
509 - 517	1077.5406	1076.5333	1076.5098	0.0236	1	TTEAEKNER (No match)
524 - 533	1045.5856	1044.5783	1044.5563	0.0220	0	ALTSELANAR (No match)
539 - 550	1402.6550	1401.6477	1401.6129	0.0348	0	TANDMIHAENMR (No match)

569 - 577	1154.5349	1153.5276	1153.5073	0.0203	1	**QRIDEFESM**	(No match)
569 - 577	1170.5331	1169.5258	1169.5022	0.0236	1	**QRIDEFESM**	Oxidation (M) (No match)

Mascot: http://www.matrixscience.com/

Spot 248

{MATRIX SCIENCE} Mascot Search Results

Protein View

Match to: **gi|14625824** Score: **329** Expect: **2.4e-028**
moesin/anaplastic lymphoma kinase fusion protein [Homo sapiens]

Nominal mass (M_r): **62004**; Calculated pI value: **7.61**
NCBI BLAST search of gi|14625824 against nr
Unformatted sequence string for pasting into other applications

Taxonomy: Homo sapiens

Fixed modifications: Carbamidomethyl (C)
Variable modifications: Oxidation (M)
Cleavage by Trypsin: cuts C-term side of KR unless next residue is P
Sequence Coverage: **16%**

Matched peptides shown in **Bold Red**

```
  1 MPKTISVRVT TMDAELEFAI QPNTTGKQLF DQVVKTIGLR EVWFFGLQYQ
 51 DTKGFSTWLK LNKKVTAQDV RKESPLLFKF RAKFYPEDVS EELIQDITQR
101 LFFLQVKEGI LNDDIYCPPE TAVLLASYAV QSKYGDFNKE VHKSGYLAGD
151 KLLPQRVLEQ HKLNKDQWEE RIQVWHEEHR GMLREDAVLE YLKIAQDLEM
201 YGVNYFSIKN KKGSELWLGV DALGLNIYEQ NDRLTPKIGF PWSEIRNISF
251 NDKKFVIKPI DKKAPDFVFY APRLRINKRI LALCMGNHEL YMRRRKPDTI
301 EVQQMKAQAR EEKHQKQMER AMLENEKKKR EMAEKEKEKI EREKEELMER
351 LKQIEEQTKK AQQELEEQTR RALELEQERK RAQSEAEKLA KERQEAEEAK
401 EALLQASRDQ KKTQEQLALE MAELTARISQ LEMARQKKES EAVEWQQKQE
451 LQAMQMELQS PEYKLSKLRT STIMTDYNPN YCFAGKTSSI SDLKEVPRKN
501 ITLIRGLGHG AFGEVYEGQV SGMPNDP
```

Residue Number Increasing Mass Decreasing Mass

Start - End	Observed	Mr(expt)	Mr(calc)	Delta	Miss	Sequence
82 - 100	2281.0669	2280.0596	2280.1273	-0.0677	1	AKFYPEDVSEELIQDITQR (Ions score 113)
84 - 100	2081.9426	2080.9353	2080.9953	-0.0599	0	FYPEDVSEELIQDITQR (Ions score 42)
84 - 100	2081.9426	2080.9353	2080.9953	-0.0599	0	FYPEDVSEELIQDITQR (No match)
181 - 193	1552.7565	1551.7492	1551.7966	-0.0474	1	GMLREDAVLEYLK Oxidation (M) (No match)

238 - 246	1104.5509	1103.5436	1103.5763	-0.0327	0	IGFPWSEIR (No match)
263 - 273	1310.6512	1309.6439	1309.6818	-0.0379	1	KAPDFVFYAPR (No match)
263 - 273	1310.6512	1309.6439	1309.6818	-0.0379	1	KAPDFVFYAPR (Ions score 23)
264 - 273	1182.5574	1181.5501	1181.5869	-0.0368	0	APDFVFYAPR (Ions score 62)
264 - 273	1182.5574	1181.5501	1181.5869	-0.0368	0	APDFVFYAPR (No match)
412 - 427	1847.9001	1846.8928	1846.9458	-0.0530	1	KTQEQLALEMAELTAR Oxidation (M) (No match)
449 - 467	2281.0669	2280.0596	2280.1130	-0.0533	1	QELQAMQMELQSPEYKLSK (No match)

Mascot: http://www.matrixscience.com/

Spot 249

{MATRIX}{SCIENCE} Mascot Search Results

Protein View

Match to: **gi|14625824** Score: **268** Expect: **3e-022**
moesin/anaplastic lymphoma kinase fusion protein [Homo sapiens]

Nominal mass (M_r): **62004;** Calculated pI value: **7.61**
NCBI BLAST search of gi|14625824 against nr
Unformatted sequence string for pasting into other applications

Taxonomy: Homo sapiens

Fixed modifications: Carbamidomethyl (C)
Variable modifications: Oxidation (M)
Cleavage by Trypsin: cuts C-term side of KR unless next residue is P
Sequence Coverage: **14%**

Matched peptides shown in **Bold Red**

```
  1 MPKTISVRVT TMDAELEFAI QPNTTGKQLF DQVVKTIGLR EVWFFGLQYQ
 51 DTKGFSTWLK LNKKVTAQDV RKESPLLFKF RAKFYPEDVS EELIQDITQR
101 LFFLQVKEGI LNDDIYCPPE TAVLLASYAV QSKYGDFNKE VHKSGYLAGD
151 KLLPQRVLEQ HKLNKDQWEE RIQVWHEEHR GMLREDAVLE YLKIAQDLEM
201 YGVNYFSIKN KKGSELWLGV DALGLNIYEQ NDRLTPKIGF PWSEIRNISF
251 NDKKFVIKPI DKKAPDFVFY APRLRINKRI LALCMGNHEL YMRRRKPDTI
301 EVQQMKAQAR EEKHQKQMER AMLENEKKKR EMAEKEKEKI EREKEELMER
351 LKQIEEQTKK AQQELEEQTR RALELEQERK RAQSEAEKLA KERQEAEEAK
401 EALLQASRDQ KKTQEQLALE MAELTARISQ LEMARQKKES EAVEWQQKQE
451 LQAMQMELQS PEYKLSKLRT STIMTDYNPN YCFAGKTSSI SDLKEVPRKN
501 ITLIRGLGHG AFGEVYEGQV SGMPNDP
```

Residue Number Increasing Mass Decreasing Mass

Start - End	Observed	Mr(expt)	Mr(calc)	Delta	Miss	Sequence
82 - 100	2281.0583	2280.0510	2280.1273	-0.0763	1	AKFYPEDVSEELIQDITQR (Ions score 44)
84 - 100	2081.9316	2080.9243	2080.9953	-0.0709	0	FYPEDVSEELIQDITQR (Ions score 66)
84 - 100	2081.9316	2080.9243	2080.9953	-0.0709	0	FYPEDVSEELIQDITQR (No match)
181 - 193	1552.7522	1551.7449	1551.7966	-0.0517	1	GMLREDAVLEYLK Oxidation (M) (No match)

263 - 273	1310.6426	1309.6353	1309.6818	-0.0465	1	KAPDFVFYAPR (No match)
263 - 273	1310.6426	1309.6353	1309.6818	-0.0465	1	KAPDFVFYAPR (Ions score 19)
264 - 273	1182.5509	1181.5436	1181.5869	-0.0433	0	APDFVFYAPR (Ions score 71)
264 - 273	1182.5509	1181.5436	1181.5869	-0.0433	0	APDFVFYAPR (No match)
412 - 427	1847.8925	1846.8852	1846.9458	-0.0606	1	KTQEQLALEMAELTAR Oxidation (M) (No match)
449 - 467	2281.0583	2280.0510	2280.1130	-0.0619	1	QELQAMQMELQSPEYKLSK (No match)

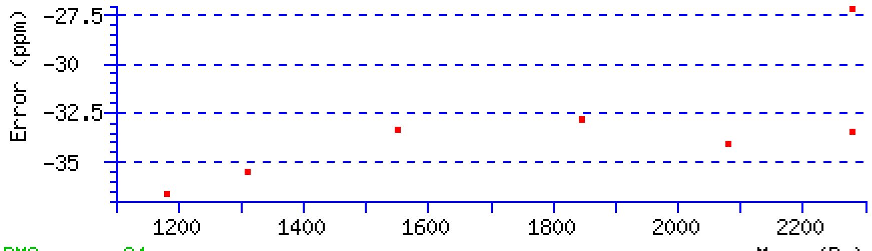

RMS error 34 ppm

Mascot: http://www.matrixscience.com/

Spot 250

Mascot Search Results

Protein View

Match to: **gi|14625824** Score: **361** Expect: **1.5e-031**
moesin/anaplastic lymphoma kinase fusion protein [Homo sapiens]

Nominal mass (M_r): **62004;** Calculated pI value: **7.61**
NCBI BLAST search of gi|14625824 against nr
Unformatted sequence string for pasting into other applications

Taxonomy: Homo sapiens

Fixed modifications: Carbamidomethyl (C)
Variable modifications: Oxidation (M)
Cleavage by Trypsin: cuts C-term side of KR unless next residue is P
Sequence Coverage: **29%**

Matched peptides shown in **Bold Red**

```
  1 MPKTISVRVT TMDAELEFAI QPNTTGKQLF DQVVKTIGLR EVWFFGLQYQ
 51 DTKGFSTWLK LNKKVTAQDV RKESPLLFKF RAKFYPEDVS EELIQDITQR
101 LFFLQVKEGI LNDDIYCPPE TAVLLASYAV QSKYGDFNKE VHKSGYLAGD
151 KLLPQRVLEQ HKLNKDQWEE RIQVWHEEHR GMLREDAVLE YLKIAQDLEM
201 YGVNYFSIKN KKGSELWLGV DALGLNIYEQ NDRLTPKIGF PWSEIRNISF
251 NDKKFVIKPI DKKAPDFVFY APRLRINKRI LALCMGNHEL YMRRRKPDTI
301 EVQQMKAQAR EEKHQKQMER AMLENEKKKR EMAEKEKEKI EREKEELMER
351 LKQIEEQTKK AQQELEEQTR RALELEQERK RAQSEAEKLA KERQEAEEAK
401 EALLQASRDQ KKTQEQLALE MAELTARISQ LEMARQKKES EAVEWQQKQE
451 LQAMQMELQS PEYKLSKLRT STIMTDYNPN YCFAGKTSSI SDLKEVPRKN
501 ITLIRGLGHG AFGEVYEGQV SGMPNDP
```

Residue Number Increasing Mass Decreasing Mass

Start - End	Observed	Mr(expt)	Mr(calc)	Delta	Miss	Sequence
28 - 35	976.5081	975.5008	975.5389	-0.0381	0	QLFDQVVK (No match)
84 - 100	2081.9507	2080.9434	2080.9953	-0.0518	0	FYPEDVSEELIQDITQR (No match)
144 - 156	1417.7397	1416.7324	1416.7724	-0.0400	1	SGYLAGDKLLPQR (No match)
172 - 180	1233.5718	1232.5645	1232.6050	-0.0405	0	IQVWHEEHR (Ions score 30)

181 - 193	1552.7590	1551.7517	1551.7966	-0.0449	1	**GMLREDAVLEYLK** Oxidation (M) (No match)
238 - 246	1104.5508	1103.5435	1103.5763	-0.0328	0	**IGFPWSEIR** (No match)
238 - 246	1104.5508	1103.5435	1103.5763	-0.0328	0	**IGFPWSEIR** (Ions score 38)
263 - 273	1310.6503	1309.6430	1309.6818	-0.0388	1	**KAPDFVFYAPR** (No match)
263 - 273	1310.6503	1309.6430	1309.6818	-0.0388	1	**KAPDFVFYAPR** (Ions score 56)
264 - 273	1182.5576	1181.5503	1181.5869	-0.0366	0	**APDFVFYAPR** (Ions score 89)
264 - 273	1182.5576	1181.5503	1181.5869	-0.0366	0	**APDFVFYAPR** (No match)
295 - 306	1488.7415	1487.7342	1487.7766	-0.0423	1	**RKPDTIEVQQMK** Oxidation (M) (No match)
361 - 371	1387.6516	1386.6443	1386.6851	-0.0408	1	**AQQELEEQTRR** (No match)
372 - 379	987.4825	986.4752	986.5032	-0.0280	0	**ALELEQER** (No match)
412 - 427	1847.9083	1846.9010	1846.9458	-0.0448	1	**KTQEQLALEMAELTAR** Oxidation (M) (No match)
413 - 427	1719.8167	1718.8094	1718.8508	-0.0414	0	**TQEQLALEMAELTAR** Oxidation (M) (No match)
439 - 448	1233.5718	1232.5645	1232.5673	-0.0028	0	**ESEAVEWQQK** (No match)
449 - 467	2281.0764	2280.0691	2280.1130	-0.0438	1	**QELQAMQMELQSPEYKLSK** (No match)

Mascot: http://www.matrixscience.com/

Spot 251

{MATRIX}{SCIENCE} **Mascot Search Results**

Protein View

Match to: **gi|8569616** Score: **130** Expect: **1.9e-008**
Chain A, Crystal Structure Of The Moesin Ferm DomainTAIL DOMAIN Complex

Nominal mass (M_r): **34554;** Calculated pI value: **8.92**
NCBI BLAST search of gi|8569616 against nr
Unformatted sequence string for pasting into other applications

Taxonomy: Homo sapiens
Links to retrieve other entries containing this sequence from NCBI Entrez:
gi|8569618 from Homo sapiens

Fixed modifications: Carbamidomethyl (C)
Variable modifications: Oxidation (M)
Cleavage by Trypsin: cuts C-term side of KR unless next residue is P
Sequence Coverage: **34%**

Matched peptides shown in **Bold Red**

```
  1 TISVRVTTXD AELEFAIQPN TTGKQLFDQV VKTIGLREVW FFGLQYQDTK
 51 GFSTWLKLNK KVTAQDVRKE SPLLFKFRAK FYPEDVSEEL IQDITQRLFF
101 LQVKEGILND DIYCPPETAV LLASYAVQSK YGDFNKEVHK SGYLAGDKLL
151 PQRVLEQHKL NKDQWEERIQ VWHEEHRGXL REDAVLEYLK IAQDLEXYGV
201 NYFSIKNKKG SELWLGVDAL GLNIYEQNDR LTPKIGFPWS EIRNISFNDK
251 KFVIKPIDKK APDFVFYAPR LRINKRILAL CXGNHELYXR RRKP
```

Residue Number Increasing Mass Decreasing Mass

Start - End	Observed	Mr(expt)	Mr(calc)	Delta	Miss	Sequence
6 - 24	2082.1523	2081.1450	2081.0316	0.1134	0	VTTFDAELEFAIQPNTTGK (Ions score 7)
25 - 32	976.6057	975.5984	975.5389	0.0595	0	QLFDQVVK (No match)
51 - 57	838.4995	837.4922	837.4384	0.0538	0	GFSTWLK (No match)
70 - 78	1136.6545	1135.6472	1135.6389	0.0083	1	ESPLLFKFR (No match)
98 - 104	894.5967	893.5894	893.5374	0.0520	0	LFFLQVK (No match)
154 - 162	1108.6571	1107.6498	1107.6400	0.0098	1	VLEQHKLNK (No match)
169 - 177	1233.6934	1232.6861	1232.6050	0.0811	0	IQVWHEEHR (No match)

182 - 190	1079.5803	1078.5730	1078.5546	0.0184	0	EDAVLEYLK	(No match)
235 - 243	1104.6599	1103.6526	1103.5763	0.0763	0	IGFPWSEIR	(Ions score 45)
235 - 243	1104.6599	1103.6526	1103.5763	0.0763	0	IGFPWSEIR	(No match)
244 - 251	965.5646	964.5573	964.4977	0.0596	1	NISFNDKK	(No match)
252 - 259	959.5834	958.5761	958.5851	-0.0090	0	FVIKPIDK	(No match)

Mascot: http://www.matrixscience.com/

Spot 253

Mascot Search Results

Protein View

Match to: **gi|20127408** Score: **126** Expect: **4.8e-008**
mitochondrial trifunctional protein, alpha subunit precursor [Homo sapiens]

Nominal mass (M_r): **83688**; Calculated pI value: **9.16**
NCBI BLAST search of gi|20127408 against nr
Unformatted sequence string for pasting into other applications

Taxonomy: Homo sapiens
Links to retrieve other entries containing this sequence from NCBI Entrez:
gi|20141376 from Homo sapiens
gi|14328041 from Homo sapiens
gi|119621108 from Homo sapiens
gi|123984943 from synthetic construct
gi|123998787 from synthetic construct

Fixed modifications: Carbamidomethyl (C)
Variable modifications: Oxidation (M)
Cleavage by Trypsin: cuts C-term side of KR unless next residue is P
Sequence Coverage: **7%**

Matched peptides shown in **Bold Red**

1 MVACRAIGIL SRFSAFRILR SRGYICRNFT GSSALLTRTH INYGVKGDVA
51 VVRINSPNSK VNTLSKELHS EFSEVMNEIW ASDQIRSAVL ISSKPGCFIA
101 GADINMLAAC KTLQEVTQLS QEAQRIVEKL EKSTKPIVAA INGSCLGGGL
151 EVAISCQYRI ATKDRK**TVLG** **TPEVLLGALP** **GAGGTQR**LPK MVGVPAALDM
201 MLTGRSIRAD RAKKMGLVDQ LVEPLGPGLK PPEERTIEYL EEVAITFAKG
251 LADKKISPKR DKGLVEKLTA YAMTIPFVRQ QVYKKVEEKV RKQTKGLYPA
301 PLKIIDVVKT GIEQGSDAGY LCESQKFGEL VMTKESKALM GLYHGQVLCK
351 KNKFGAPQKD VKHLAILGAG LMGAGIAQVS VDKGLKTILK DATLTALDRG
401 QQQVFKGLND KVKKKALTSF ERDSIFSNLT GQLDYQGFEK ADMVIEAVFE
451 DLSLKHRVLK EVEAVIPDHC IFASNTSALP ISEIAAVSKR PEKVIGMHYF
501 SPVDKMQLLE IITTEKTSKD TSASAVAVGL KQGKVIIVVK **DGPGFYTTR**C
551 LAPMMSEVIR ILQEGVDPKK LDSLTTSFGF PVGAATLVDE VGVDVAK**HVA**
601 **EDLGK**VFGER FGGGNPELLT QMVSKGFLGR KSGK**GFYIYQ** **EGVK**RKDLNS
651 DMDSILASLK LPPKSEVSSD EDIQFRLVTR FVNEAVMCLQ EGILATPAEG
701 DIGAVFGLGF PPCLGGPFR**F** **VDLYGAQK**IV DRLKKYEAAY GKQFTPCQLL
751 ADHANSPNKK FYQ

Residue Number Increasing Mass Decreasing Mass

Start - End	Observed	Mr(expt)	Mr(calc)	Delta	Miss	Sequence
167 - 187	2007.0778	2006.0705	2006.1159	-0.0454	0	TVLGTPEVLLGALPGAGGTQR (No match)
167 - 187	2007.0778	2006.0705	2006.1159	-0.0454	0	TVLGTPEVLLGALPGAGGTQR (Ions score 49)
541 - 549	1013.4338	1012.4265	1012.4614	-0.0348	0	DGPGFYTTR (No match)
598 - 605	868.4597	867.4524	867.4450	0.0074	0	HVAEDLGK (No match)
635 - 644	1203.5565	1202.5492	1202.5971	-0.0479	0	GFYIYQEGVK (No match)
635 - 644	1203.5565	1202.5492	1202.5971	-0.0479	0	GFYIYQEGVK (Ions score 33)
720 - 728	1040.4978	1039.4905	1039.5338	-0.0433	0	FVDLYGAQK (No match)

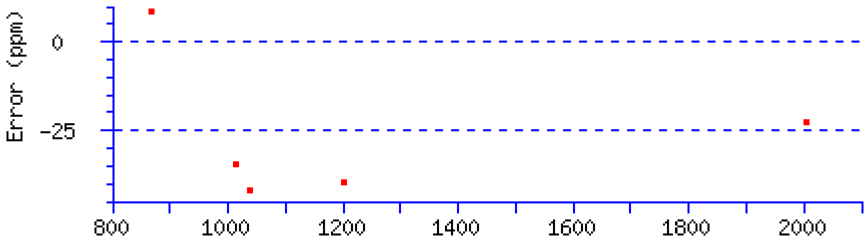

RMS error 32 ppm

Mascot: http://www.matrixscience.com/

Spot 256

{MATRIX}{SCIENCE} Mascot Search Results

Protein View

Match to: **gi|119612222** Score: **270** Expect: **1.9e-022**
poly(A) binding protein, cytoplasmic 1, isoform CRA_c [Homo sapiens]

Nominal mass (M_r): **47647;** Calculated pI value: **9.04**
NCBI BLAST search of gi|119612222 against nr
Unformatted sequence string for pasting into other applications

Taxonomy: Homo sapiens
Links to retrieve other entries containing this sequence from NCBI Entrez:
gi|119612227 from Homo sapiens

Fixed modifications: Carbamidomethyl (C)
Variable modifications: Oxidation (M)
Cleavage by Trypsin: cuts C-term side of KR unless next residue is P
Sequence Coverage: **15%**

Matched peptides shown in **Bold Red**

```
  1 MNPSAPSYPM ASLYVGDLHP DVTEAMLYEK FSPAGPILSI RVCRDMITRR
 51 SLGYAYVNFQ QPADAERALD TMNFDVIKGK PVRIMWSQRD PSLRKSGVGN
101 IFIKNLDKSI DNKALYDTFS AFGNILSCKV VCDENGSKGY GFVHFETQEA
151 AERAIEKMNG MLLNDRKVFV GRFKSRKERE AELGARAKEF TNVYIKNFGE
201 DMDDERLKDL FGKFGPALSV KVMTDESGKS KGFGFVSFER HEDAQKAVDE
251 MNGKELNGKQ IYVGRAQKKV ERQTELKRKF EQMKQDRITR YQGVNLYVKN
301 LDDGIDDERL RKEFSPFGTI TSAKVMMEGG RSKGFGFVCF SSPEEATKAV
351 TEMNGRIVAT KPLYVALAQR KEERQAHLTN QYMQRMASVR AVPNPASTSF
401 RLLHGSYPTD SEPCCILSS
```

Residue Number Increasing Mass Decreasing Mass

Start - End	Observed	Mr(expt)	Mr(calc)	Delta	Miss	Sequence
31 - 41	1157.6250	1156.6177	1156.6604	-0.0427	0	FSPAGPILSIR (No match)
31 - 41	1157.6250	1156.6177	1156.6604	-0.0427	0	FSPAGPILSIR (No match)
51 - 67	1928.8379	1927.8306	1927.9064	-0.0757	0	SLGYAYVNFQQPADAER (Ions score 69)
51 - 67	1928.8379	1927.8306	1927.9064	-0.0757	0	SLGYAYVNFQQPADAER (No match)

139 - 153	1740.7310	1739.7237	1739.7903	-0.0665	0	GYGFVHFETQEAAER	(Ions score 63)
139 - 153	1740.7310	1739.7237	1739.7903	-0.0665	0	GYGFVHFETQEAAER	(No match)
232 - 240	1045.4701	1044.4628	1044.5028	-0.0400	0	GFGFVSFER	(Ions score 17)
232 - 240	1045.4701	1044.4628	1044.5028	-0.0400	0	GFGFVSFER	(No match)
357 - 370	1542.8770	1541.8697	1541.9293	-0.0596	0	IVATKPLYVALAQR	(Ions score 51)
357 - 370	1542.8770	1541.8697	1541.9293	-0.0596	0	IVATKPLYVALAQR	(No match)

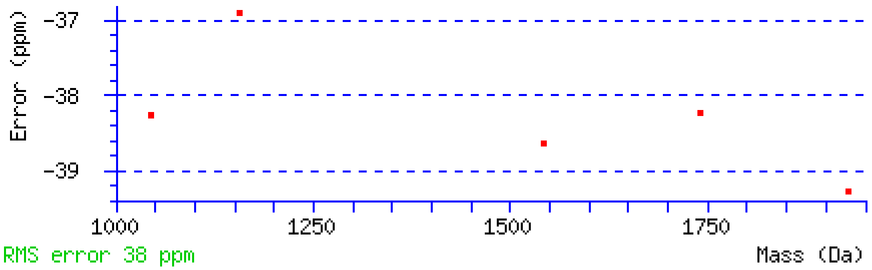

RMS error 38 ppm

Mascot: http://www.matrixscience.com/

Spot 259

{MATRIX SCIENCE} Mascot Search Results

Protein View

Match to: **gi|5729877** Score: **517** Expect: **3.8e-047**
heat shock 70kDa protein 8 isoform 1 [Homo sapiens]

Nominal mass (M_r): **71082**; Calculated pI value: **5.37**
NCBI BLAST search of gi|5729877 against nr
Unformatted sequence string for pasting into other applications

Taxonomy: Homo sapiens
Links to retrieve other entries containing this sequence from NCBI Entrez:
gi|126352504 from Equus caballus
gi|57085907 from Canis lupus familiaris
gi|109109056 from Macaca mulatta
gi|114640901 from Pan troglodytes
gi|123648 from Homo sapiens
gi|52000695 from Saguinus oedipus
gi|146324912 from Equus caballus
gi|7672663 from Saguinus oedipus
gi|13273304 from Homo sapiens
gi|32467 from Homo sapiens
gi|16740593 from Homo sapiens
gi|16741727 from Homo sapiens
gi|18043726 from Homo sapiens
gi|55729181 from Pongo pygmaeus
gi|119587943 from Homo sapiens
gi|119587944 from Homo sapiens
gi|123980484 from synthetic construct
gi|123995299 from synthetic construct
gi|124377696 from Equus caballus

Fixed modifications: Carbamidomethyl (C)
Variable modifications: Oxidation (M)
Cleavage by Trypsin: cuts C-term side of KR unless next residue is P
Sequence Coverage: **46%**

Matched peptides shown in **Bold Red**

1 MSK**GPAVGID LGTTYSCVGV FQHGKVEIIA NDQGNRTTPS YVAFTDTER**L
51 IGDAAK**NQVA MNPTNTVFDA KR**LIGR**RFDD AVVQSDMKHW PFMVVNDAGR**

```
101 PKVQVEYKGE TKSFYPEEVS SMVLTKMKEI AEAYLGKTVT NAVVTVPAYF
151 NDSQRQATKD AGTIAGLNVL RIINEPTAAA IAYGLDKKVG AERNVLIFDL
201 GGGTFDVSIL TIEDGIFEVK STAGDTHLGG EDFDNRMVNH FIAEFKRKHK
251 KDISENKRAV RRLRTACERA KRTLSSSTQA SIEIDSLYEG IDFYTSITRA
301 RFEELNADLF RGTLDPVEKA LRDAKLDKSQ IHDIVLVGGS TRIPKIQKLL
351 QDFFNGKELN KSINPDEAVA YGAAVQAAIL SGDKSENVQD LLLLDVTPLS
401 LGIETAGGVM TVLIKRNTTI PTKQTQTFTT YSDNQPGVLI QVYEGERAMT
451 KDNNLLGKFE LTGIPPAPRG VPQIEVTFDI DANGILNVSA VDKSTGKENK
501 ITITNDKGRL SKEDIERMVQ EAEKYKAEDE KQRDKVSSKN SLESYAFNMK
551 ATVEDEKLQG KINDEDKQKI LDKCNEIINW LDKNQTAEKE EFEHQQKELE
601 KVCNPIITKL YQSAGGMPGG MPGGFPGGGA PPSGGASSGP TIEEVD
```

Residue Number Increasing Mass Decreasing Mass

Start - End	Observed	Mr(expt)	Mr(calc)	Delta	Miss	Sequence
4 - 25	2263.1465	2262.1392	2262.1102	0.0290	0	GPAVGIDLGTTYSCVGVFQHGK (No match)
26 - 36	1228.6436	1227.6363	1227.6207	0.0156	0	VEIIANDQGNR (No match)
37 - 49	1487.7139	1486.7066	1486.6940	0.0127	0	TTPSYVAFTDTER (Ions score 24)
37 - 49	1487.7139	1486.7066	1486.6940	0.0127	0	TTPSYVAFTDTER (No match)
57 - 72	1805.9117	1804.9044	1804.8890	0.0155	1	NQVAMNPTNTVFDAKR (No match)
57 - 72	1821.9065	1820.8992	1820.8839	0.0154	1	NQVAMNPTNTVFDAKR Oxidation (M) (No match)
77 - 88	1410.6885	1409.6812	1409.6609	0.0204	1	RFDDAVVQSDMK (No match)
89 - 102	1653.8458	1652.8385	1652.8245	0.0140	0	HWPFMVVNDAGRPK (No match)
89 - 102	1669.8423	1668.8350	1668.8194	0.0156	0	HWPFMVVNDAGRPK Oxidation (M) (No match)
127 - 137	1252.6479	1251.6406	1251.6532	-0.0126	1	MKEIAEAYLGK (No match)
138 - 155	1982.0122	1981.0049	1980.9904	0.0145	0	TVTNAVVTVPAYFNDSQR (Ions score 84)
138 - 155	1982.0122	1981.0049	1980.9904	0.0145	0	TVTNAVVTVPAYFNDSQR (No match)
160 - 171	1199.6859	1198.6786	1198.6669	0.0117	0	DAGTIAGLNVLR (Ions score 67)
160 - 171	1199.6859	1198.6786	1198.6669	0.0117	0	DAGTIAGLNVLR (No match)
172 - 188	1788.0065	1786.9992	1786.9828	0.0164	1	IINEPTAAAIAYGLDKK (No match)
221 - 236	1691.7418	1690.7345	1690.7182	0.0163	0	STAGDTHLGGEDFDNR (No match)
221 - 236	1691.7418	1690.7345	1690.7182	0.0163	0	STAGDTHLGGEDFDNR (Ions score 109)
237 - 246	1235.6340	1234.6267	1234.6168	0.0099	0	MVNHFIAEFK (No match)
237 - 246	1251.6288	1250.6215	1250.6117	0.0098	0	MVNHFIAEFK Oxidation (M) (No match)
237 - 247	1391.7393	1390.7320	1390.7179	0.0141	1	MVNHFIAEFKR (No match)
237 - 247	1407.7322	1406.7249	1406.7128	0.0121	1	MVNHFIAEFKR Oxidation (M) (No match)
252 - 258	861.4385	860.4312	860.4352	-0.0039	1	DISENKR (No match)
273 - 299	2997.4529	2996.4456	2996.4502	-0.0046	0	TLSSSTQASIEIDSLYEGIDFYTSITR (No match)
300 - 311	1480.7682	1479.7609	1479.7469	0.0140	1	ARFEELNADLFR (No match)
302 - 311	1253.6312	1252.6239	1252.6087	0.0152	0	FEELNADLFR (No match)
326 - 342	1838.0308	1837.0235	1837.0057	0.0178	1	LDKSQIHDIVLVGGSTR (No match)
349 - 357	1081.5745	1080.5672	1080.5603	0.0069	0	LLQDFFNGK (No match)
424 - 447	2774.3347	2773.3274	2773.3194	0.0080	0	QTQTFTTYSDNQPGVLIQVYEGER (No match)
510 - 517	989.5375	988.5302	988.5189	0.0113	1	LSKEDIER (No match)
540 - 550	1303.6062	1302.5989	1302.5914	0.0075	0	NSLESYAFNMK (No match)

584 - 597	1745.8322	1744.8249	1744.8016	0.0233	1	NQTAEKEEFEHQQK	(No match)

Mascot: http://www.matrixscience.com/

Spot 260

Mascot Search Results

Protein View

Match to: **gi|5729877** Score: **382** Expect: **1.2e-033**
heat shock 70kDa protein 8 isoform 1 [Homo sapiens]

Nominal mass (M_r): **71082**; Calculated pI value: **5.37**
NCBI BLAST search of gi|5729877 against nr
Unformatted sequence string for pasting into other applications

Taxonomy: Homo sapiens
Links to retrieve other entries containing this sequence from NCBI Entrez:
gi|126352504 from Equus caballus
gi|57085907 from Canis lupus familiaris
gi|109109056 from Macaca mulatta
gi|114640901 from Pan troglodytes
gi|123648 from Homo sapiens
gi|52000695 from Saguinus oedipus
gi|146324912 from Equus caballus
gi|7672663 from Saguinus oedipus
gi|13273304 from Homo sapiens
gi|32467 from Homo sapiens
gi|16740593 from Homo sapiens
gi|16741727 from Homo sapiens
gi|18043726 from Homo sapiens
gi|55729181 from Pongo pygmaeus
gi|119587943 from Homo sapiens
gi|119587944 from Homo sapiens
gi|123980484 from synthetic construct
gi|123995299 from synthetic construct
gi|124377696 from Equus caballus

Fixed modifications: Carbamidomethyl (C)
Variable modifications: Oxidation (M)
Cleavage by Trypsin: cuts C-term side of KR unless next residue is P
Sequence Coverage: **37%**

Matched peptides shown in **Bold Red**

1 MSK**GPAVGID LGTTYSCVGV FQHGKVEIIA NDQGNRTTPS YVAFTDTER**L
51 IGDAAK**NQVA MNPTNTVFDA KR**LIGRRFDD AVVQSDMK**HW PFMVVNDAGR**

```
101 PKVQVEYKGE TKSFYPEEVS SMVLTKMKEI AEAYLGKTVT NAVVTVPAYF
151 NDSQRQATKD AGTIAGLNVL RIINEPTAAA IAYGLDKKVG AERNVLIFDL
201 GGGTFDVSIL TIEDGIFEVK STAGDTHLGG EDFDNRMVNH FIAEFKRKHK
251 KDISENKRAV RRLRTACERA KRTLSSSTQA SIEIDSLYEG IDFYTSITRA
301 RFEELNADLF RGTLDPVEKA LRDAKLDKSQ IHDIVLVGGS TRIPKIQKLL
351 QDFFNGKELN KSINPDEAVA YGAAVQAAIL SGDKSENVQD LLLLDVTPLS
401 LGIETAGGVM TVLIKRNTTI PTKQTQTFTT YSDNQPGVLI QVYEGERAMT
451 KDNNLLGKFE LTGIPPAPRG VPQIEVTFDI DANGILNVSA VDKSTGKENK
501 ITITNDKGRL SKEDIERMVQ EAEKYKAEDE KQRDKVSSKN SLESYAFNMK
551 ATVEDEKLQG KINDEDKQKI LDKCNEIINW LDKNQTAEKE EFEHQQKELE
601 KVCNPIITKL YQSAGGMPGG MPGGFPGGGA PPSGGASSGP TIEEVD
```

Residue Number Increasing Mass Decreasing Mass

Start - End	Observed	Mr(expt)	Mr(calc)	Delta	Miss	Sequence
4 - 25	2263.1399	2262.1326	2262.1102	0.0224	0	GPAVGIDLGTTYSCVGVFQHGK (No match)
26 - 36	1228.6511	1227.6438	1227.6207	0.0231	0	VEIIANDQGNR (No match)
37 - 49	1487.7124	1486.7051	1486.6940	0.0112	0	TTPSYVAFTDTER (Ions score 13)
37 - 49	1487.7124	1486.7051	1486.6940	0.0112	0	TTPSYVAFTDTER (No match)
57 - 72	1805.9138	1804.9065	1804.8890	0.0176	1	NQVAMNPTNTVFDAKR (No match)
89 - 102	1653.8442	1652.8369	1652.8245	0.0124	0	HWPFMVVNDAGRPK (No match)
89 - 102	1669.8430	1668.8357	1668.8194	0.0163	0	HWPFMVVNDAGRPK Oxidation (M) (No match)
138 - 155	1982.0077	1981.0004	1980.9904	0.0100	0	TVTNAVVTVPAYFNDSQR (No match)
138 - 155	1982.0077	1981.0004	1980.9904	0.0100	0	TVTNAVVTVPAYFNDSQR (Ions score 76)
160 - 171	1199.6859	1198.6786	1198.6669	0.0117	0	DAGTIAGLNVLR (Ions score 40)
160 - 171	1199.6859	1198.6786	1198.6669	0.0117	0	DAGTIAGLNVLR (No match)
172 - 188	1788.0042	1786.9969	1786.9828	0.0141	1	IINEPTAAAIAYGLDKK (No match)
221 - 236	1691.7391	1690.7318	1690.7182	0.0136	0	STAGDTHLGGEDFDNR (No match)
221 - 236	1691.7391	1690.7318	1690.7182	0.0136	0	STAGDTHLGGEDFDNR (Ions score 74)
237 - 246	1235.6331	1234.6258	1234.6168	0.0090	0	MVNHFIAEFK (No match)
237 - 246	1251.6300	1250.6227	1250.6117	0.0110	0	MVNHFIAEFK Oxidation (M) (No match)
237 - 247	1391.7408	1390.7335	1390.7179	0.0156	1	MVNHFIAEFKR (No match)
300 - 311	1480.7672	1479.7599	1479.7469	0.0130	1	ARFEELNADLFR (No match)
302 - 311	1253.6309	1252.6236	1252.6087	0.0149	0	FEELNADLFR (No match)
326 - 342	1838.0271	1837.0198	1837.0057	0.0141	1	LDKSQIHDIVLVGGSTR (No match)
349 - 357	1081.5715	1080.5642	1080.5603	0.0039	0	LLQDFFNGK (No match)
424 - 447	2774.3293	2773.3220	2773.3194	0.0026	0	QTQTFTTYSDNQPGVLIQVYEGER (No match)
510 - 517	989.5417	988.5344	988.5189	0.0155	1	LSKEDIER (No match)
540 - 550	1303.6063	1302.5990	1302.5914	0.0076	0	NSLESYAFNMK (No match)
584 - 597	1745.8342	1744.8269	1744.8016	0.0253	1	NQTAEKEEFEHQQK (No match)

**Mascot:** http://www.matrixscience.com/

Spot 261

{MATRIX}{SCIENCE} Mascot Search Results

Protein View

Match to: **gi|35218** Score: **353** Expect: **9.6e-031**
unnamed protein product [Homo sapiens]

Nominal mass (M_r): **76154;** Calculated pI value: **5.41**
NCBI BLAST search of gi|35218 against nr
Unformatted sequence string for pasting into other applications

Taxonomy: Homo sapiens

Fixed modifications: Carbamidomethyl (C)
Variable modifications: Oxidation (M)
Cleavage by Trypsin: cuts C-term side of KR unless next residue is P
Sequence Coverage: **48%**

Matched peptides shown in **Bold Red**

```
  1 MAKPAQGAKY RGSIHDFPGF DPNQDAEALY TAMKGFGSDK EAILDIITSR
 51 SNRQRQEVCQ SYKSLYGKDL IADLKYELTG KFERLIVGLM RPPAYCDAKE
101 IKDAISGIGT DEKCLIEILA SRTNEQMHQL VAAYKDAYER DLEADIIGDT
151 SGHFQKMLVV LLQGTREEDD VVSEDLVQQD VQDLYEAGEL KWGTDEAQFI
201 YILGNRSKQH LRLVFDEYLK TTGKPIEASI RGELSGDFEK LMLAVVKCIR
251 STPEYFAERL FKAMKGLGTR DNTLIRIMVS RSELDMLDIR EIFRTKYEKS
301 LYSMIKNDTS GEYKKTLLKL SGGDDDAAGQ FFPEAAQVAY QMWELSAVAR
351 VELKGTVRPA NDFNPDADAK ALRKAMKGLG TDEDTIIDII THRSNVQRQQ
401 IRQTFKSHFG RDLMTDLKSE ISGDLARLIL GLMMPPAHYD AKQLKKAMEG
451 AGTDEKALIE ILATRTNAEI RAINEAYKED YHKSLEDALS SDTSGHFRRI
501 LISLATGHRE EGGENLDQAR EDAQVAAEIL EIADTPSGDK TSLETRFMTI
551 LCTRSYPHLR RVFQEFIKMT NYDVEHTIKK EMSGDVRDAF VAIVQSVKNK
601 PLFFADKLYK SMKGAGTDDK TLTRIMVSRS EIDLLNIRRE FIEKYDKSLH
651 QAIEGDTSGD FLKALLALCG GED
```

Residue Number Increasing Mass Decreasing Mass

Start - End	Observed	Mr(expt)	Mr(calc)	Delta	Miss	Sequence	
35 - 50	1721.9521	1720.9448	1720.8995	0.0453	1	GFGSDKEAILDIITSR	(Ions score 25)

35 - 50	1721.9521	1720.9448	1720.8995	0.0453	1	**GFGSDKEAILDIITSR** (No match)
54 - 63	1325.6638	1324.6565	1324.6193	0.0372	1	**QRQEVCQSYK** (No match)
85 - 99	1719.9343	1718.9270	1718.8847	0.0423	0	**LIVGLMRPPAYCDAK** Oxidation (M) (No match)
114 - 122	1074.6233	1073.6160	1073.5903	0.0258	0	**CLIEILASR** (No match)
123 - 135	1548.7861	1547.7788	1547.7402	0.0387	0	**TNEQMHQLVAAYK** Oxidation (M) (No match)
141 - 156	1745.8927	1744.8854	1744.8267	0.0587	0	**DLEADIIGDTSGHFQK** (No match)
157 - 166	1145.6963	1144.6890	1144.6638	0.0253	0	**MLVVLLQGTR** Oxidation (M) (No match)
213 - 220	1026.5687	1025.5614	1025.5433	0.0181	0	**LVFDEYLK** (No match)
221 - 231	1172.6967	1171.6894	1171.6560	0.0334	0	**TTGKPIEASIR** (No match)
251 - 259	1099.5342	1098.5269	1098.4981	0.0288	0	**STPEYFAER** (No match)
251 - 259	1099.5342	1098.5269	1098.4981	0.0288	0	**STPEYFAER** (Ions score 9)
355 - 370	1687.8522	1686.8449	1686.7961	0.0488	0	**GTVRPANDFNPDADAK** (Ions score 12)
355 - 370	1687.8522	1686.8449	1686.7961	0.0488	0	**GTVRPANDFNPDADAK** (No match)
378 - 393	1768.9543	1767.9470	1767.9003	0.0468	0	**GLGTDEDTIIDIITHR** (No match)
378 - 393	1768.9543	1767.9470	1767.9003	0.0468	0	**GLGTDEDTIIDIITHR** (Ions score 39)
403 - 411	1107.5591	1106.5518	1106.5621	-0.0102	1	**QTFKSHFGR** (No match)
412 - 427	1779.9224	1778.9151	1778.8720	0.0431	1	**DLMTDLKSEISGDLAR** Oxidation (M) (No match)
457 - 465	999.6425	998.6352	998.6124	0.0228	0	**ALIEILATR** (No match)
472 - 483	1480.7811	1479.7738	1479.6993	0.0745	1	**AINEAYKEDYHK** (No match)
484 - 498	1621.7920	1620.7847	1620.7379	0.0468	0	**SLEDALSSDTSGHFR** (No match)
500 - 520	2279.2378	2278.2305	2278.1665	0.0641	1	**ILISLATGHREEGGENLDQAR** (No match)
521 - 546	2759.4177	2758.4104	2758.3508	0.0596	1	**EDAQVAAEILEIADTPSGDKTSLETR** (No match)
547 - 554	1057.5767	1056.5694	1056.5096	0.0598	0	**FMTILCTR** Oxidation (M) (No match)
555 - 561	928.5297	927.5224	927.5038	0.0186	1	**SYPHLRR** (No match)
561 - 568	1066.6300	1065.6227	1065.5970	0.0257	1	**RVFQEFIK** (No match)
561 - 568	1066.6300	1065.6227	1065.5970	0.0257	1	**RVFQEFIK** (No match)
562 - 568	910.5176	909.5103	909.4959	0.0144	0	**VFQEFIK** (No match)
588 - 598	1176.6890	1175.6817	1175.6550	0.0268	0	**DAFVAIVQSVK** (No match)
599 - 607	1079.6068	1078.5995	1078.5811	0.0185	0	**NKPLFFADK** (No match)
630 - 638	1072.6223	1071.6150	1071.5924	0.0226	0	**SEIDLLNIR** (No match)
630 - 639	1228.7321	1227.7248	1227.6935	0.0313	1	**SEIDLLNIRR** (No match)
648 - 663	1717.9008	1716.8935	1716.8318	0.0617	0	**SLHQAIEGDTSGDFLK** (No match)

Mascot: http://www.matrixscience.com/

Spot 262

{MATRIX SCIENCE} Mascot Search Results

Protein View

Match to: **gi|35218** Score: **524** Expect: **7.7e-048**
unnamed protein product [Homo sapiens]

Nominal mass (M_r): **76154;** Calculated pI value: **5.41**
NCBI BLAST search of gi|35218 against nr
Unformatted sequence string for pasting into other applications

Taxonomy: Homo sapiens

Fixed modifications: Carbamidomethyl (C)
Variable modifications: Oxidation (M)
Cleavage by Trypsin: cuts C-term side of KR unless next residue is P
Sequence Coverage: **63%**

Matched peptides shown in **Bold Red**

```
  1 MAKPAQGAKY RGSIHDFPGF DPNQDAEALY TAMKGFGSDK EAILDIITSR
 51 SNRQRQEVCQ SYKSLYGKDL IADLKYELTG KFERLIVGLM RPPAYCDAKE
101 IKDAISGIGT DEKCLIEILA SRTNEQMHQL VAAYKDAYER DLEADIIGDT
151 SGHFQKMLVV LLQGTREEDD VVSEDLVQQD VQDLYEAGEL KWGTDEAQFI
201 YILGNRSKQH LRLVFDEYLK TTGKPIEASI RGELSGDFEK LMLAVVKCIR
251 STPEYFAERL FKAMKGLGTR DNTLIRIMVS RSELDMLDIR EIFRTKYEKS
301 LYSMIKNDTS GEYKKTLLKL SGGDDDAAGQ FFPEAAQVAY QMWELSAVAR
351 VELKGTVRPA NDFNPDADAK ALRKAMKGLG TDEDTIIDII THRSNVQRQQ
401 IRQTFKSHFG RDLMTDLKSE ISGDLARLIL GLMMPPAHYD AKQLKKAMEG
451 AGTDEKALIE ILATRTNAEI RAINEAYKED YHKSLEDALS SDTSGHFRRI
501 LISLATGHRE EGGENLDQAR EDAQVAAEIL EIADTPSGDK TSLETRFMTI
551 LCTRSYPHLR RVFQEFIKMT NYDVEHTIKK EMSGDVRDAF VAIVQSVKNK
601 PLFFADKLYK SMKGAGTDDK TLTRIMVSRS EIDLLNIRRE FIEKYDKSLH
651 QAIEGDTSGD FLKALLALCG GED
```

Residue Number Increasing Mass Decreasing Mass

Start - End	Observed	Mr(expt)	Mr(calc)	Delta	Miss	Sequence
10 - 34	2859.3601	2858.3528	2858.2969	0.0559	1	YRGSIHDFPGFDPNQDAEALYTAMK Oxidation (M) (No match)

12 - 34	2540.1863	2539.1790	2539.1325	0.0465	0	GSIHDFPGFDPNQDAEALYTAMK Oxidation (M) (No match)
35 - 50	1721.9216	1720.9143	1720.8995	0.0148	1	GFGSDKEAILDIITSR (Ions score 25)
35 - 50	1721.9216	1720.9143	1720.8995	0.0148	1	GFGSDKEAILDIITSR (No match)
54 - 63	1325.6379	1324.6306	1324.6193	0.0113	1	QRQEVCQSYK (No match)
85 - 99	1719.9059	1718.8986	1718.8847	0.0139	0	LIVGLMRPPAYCDAK Oxidation (M) (No match)
114 - 122	1074.6012	1073.5939	1073.5903	0.0037	0	CLIEILASR (No match)
123 - 135	1548.7633	1547.7560	1547.7402	0.0159	0	TNEQMHQLVAAYK Oxidation (M) (No match)
123 - 140	2183.0493	2182.0420	2182.0112	0.0308	1	TNEQMHQLVAAYKDAYER Oxidation (M) (No match)
141 - 156	1745.8579	1744.8506	1744.8267	0.0239	0	DLEADIIGDTSGHFQK (No match)
157 - 166	1145.6742	1144.6669	1144.6638	0.0032	0	MLVVLLQGTR Oxidation (M) (No match)
167 - 191	2865.3694	2864.3621	2864.3086	0.0535	0	EEDDVVSEDLVQQDVQDLYEAGELK (No match)
192 - 206	1782.8983	1781.8910	1781.8736	0.0174	0	WGTDEAQFIYILGNR (No match)
213 - 220	1026.5498	1025.5425	1025.5433	-0.0008	0	LVFDEYLK (No match)
221 - 231	1172.6748	1171.6675	1171.6560	0.0115	0	TTGKPIEASIR (No match)
251 - 259	1099.5121	1098.5048	1098.4981	0.0067	0	STPEYFAER (No match)
251 - 259	1099.5121	1098.5048	1098.4981	0.0067	0	STPEYFAER (Ions score 29)
251 - 262	1487.7197	1486.7124	1486.7456	-0.0331	1	STPEYFAERLFK (No match)
282 - 290	1107.5416	1106.5343	1106.5277	0.0066	0	SELDMLDIR Oxidation (M) (No match)
355 - 370	1687.8210	1686.8137	1686.7961	0.0176	0	GTVRPANDFNPDADAK (Ions score 12)
355 - 370	1687.8210	1686.8137	1686.7961	0.0176	0	GTVRPANDFNPDADAK (No match)
378 - 393	1768.9244	1767.9171	1767.9003	0.0169	0	GLGTDEDTIIDIITHR (Ions score 58)
378 - 393	1768.9244	1767.9171	1767.9003	0.0169	0	GLGTDEDTIIDIITHR (No match)
412 - 427	1779.8905	1778.8832	1778.8720	0.0112	1	DLMTDLKSEISGDLAR Oxidation (M) (No match)
428 - 442	1701.8877	1700.8804	1700.8629	0.0175	0	LILGLMMPPAHYDAK 2 Oxidation (M) (No match)
457 - 465	999.6231	998.6158	998.6124	0.0034	0	ALIEILATR (No match)
472 - 483	1480.7515	1479.7442	1479.6993	0.0449	1	AINEAYKEDYHK (No match)
484 - 498	1621.7633	1620.7560	1620.7379	0.0181	0	SLEDALSSDTSGHFR (No match)
484 - 499	1777.8673	1776.8600	1776.8390	0.0210	1	SLEDALSSDTSGHFRR (No match)
500 - 509	1080.6407	1079.6334	1079.6451	-0.0117	0	ILISLATGHR (No match)
500 - 520	2279.1985	2278.1912	2278.1665	0.0248	1	ILISLATGHREEGGENLDQAR (No match)
521 - 546	2759.3796	2758.3723	2758.3508	0.0215	1	EDAQVAAEILEIADTPSGDKTSLETR (No match)
547 - 554	1057.5542	1056.5469	1056.5096	0.0373	0	FMTILCTR Oxidation (M) (No match)
555 - 561	928.5143	927.5070	927.5038	0.0032	1	SYPHLRR (No match)
561 - 568	1066.6100	1065.6027	1065.5970	0.0057	1	RVFQEFIK (No match)
561 - 568	1066.6100	1065.6027	1065.5970	0.0057	1	RVFQEFIK (Ions score 5)
562 - 568	910.5003	909.4930	909.4959	-0.0029	0	VFQEFIK (No match)
581 - 587	809.3423	808.3350	808.3385	-0.0034	0	EMSGDVR Oxidation (M) (No match)
588 - 598	1176.6646	1175.6573	1175.6550	0.0024	0	DAFVAIVQSVK (No match)
599 - 607	1079.5879	1078.5806	1078.5811	-0.0004	0	NKPLFFADK (No match)
599 - 610	1483.8383	1482.8310	1482.8234	0.0076	1	NKPLFFADKLYK (No match)
630 - 638	1072.6040	1071.5967	1071.5924	0.0043	0	SEIDLLNIR (No match)
630 - 639	1228.7081	1227.7008	1227.6935	0.0073	1	SEIDLLNIRR (No match)
648 - 663	1717.8678	1716.8605	1716.8318	0.0287	0	SLHQAIEGDTSGDFLK (No match)

Spot 263

{MATRIX}{SCIENCE} Mascot Search Results

Protein View

Match to: **gi|35218** Score: **552** Expect: **1.2e-050**
unnamed protein product [Homo sapiens]

Nominal mass (M_r): **76154;** Calculated pI value: **5.41**
NCBI BLAST search of gi|35218 against nr
Unformatted sequence string for pasting into other applications

Taxonomy: Homo sapiens

Fixed modifications: Carbamidomethyl (C)
Variable modifications: Oxidation (M)
Cleavage by Trypsin: cuts C-term side of KR unless next residue is P
Sequence Coverage: **59%**

Matched peptides shown in **Bold Red**

1 MAKPAQGAKY R**GSIHDFPGF** **DPNQDAEALY** **TAMKGFGSDK** **EAILDIITSR**
51 SNR**QRQEVCQ** **SYK**SLYGKDL IADLKYELTG KFER**LIVGLM** **RPPAYCDAK**E
101 IKDAISGIGT DEK**CLIEILA** **SRTNEQMHQL** **VAAYK**DAYER **DLEADIIGDT**
151 **SGHFQKMLVV** **LLQGTREEDD** **VVSEDLVQQD** **VQDLYEAGEL** **K**WGTDEAQFI
201 YILGNRSKQH LR**LVFDEYLK** **TTGKPIEASI** **R**GELSGDFEK LMLAVVKCIR
251 **STPEYFAER**L FKAMKGLGTR DNTLIRIMVS RSELDMLDIR EIFRTKYEKS
301 LYSMIKNDTS GEYKKTLLKL SGGDDDAAGQ FFPEAAQVAY QMWELSAVAR
351 VELK**GTVRPA** **NDFNPDADAK** ALRKAMK**GLG** **TDEDTIIDII** **THR**SNVQRQQ
401 IR**QTFKSHFG** **RDLMTDLKSE** **ISGDLARLIL** **GLMMPPAHYD** **AK**QLKKAMEG
451 AGTDEK**ALIE** **ILATR**TNAEI R**AINEAYKED** **YHKSLEDALS** **SDTSGHFR**R**I**
501 **LISLATGHRE** **EGGENLDQAR** **EDAQVAAEIL** **EIADTPSGDK** **TSLETRFMTI**
551 **LCTRSYPHLR** **RVFQEFIK**MT NYDVEHTIKK **EMSGDVRDAF** **VAIVQSVKNK**
601 **PLFFADKLYK** SMKGAGTDDK TLTRIMVSR**S** **EIDLLNIRR**E FIEKYDK**SLH**
651 **QAIEGDTSGD** **FLK**ALLALCG GED

Residue Number Increasing Mass Decreasing Mass

Start - End	Observed	Mr(expt)	Mr(calc)	Delta	Miss	Sequence
12 - 34	2540.2346	2539.2273	2539.1325	0.0948	0	GSIHDFPGFDPNQDAEALYTAMK Oxidation (M) (No match)

35 - 50	1721.9497	1720.9424	1720.8995	0.0429	1	**GFGSDKEAILDIITSR** (No match)
35 - 50	1721.9497	1720.9424	1720.8995	0.0429	1	**GFGSDKEAILDIITSR** (Ions score 18)
54 - 63	1325.6588	1324.6515	1324.6193	0.0322	1	**QRQEVCQSYK** (No match)
85 - 99	1719.9324	1718.9251	1718.8847	0.0404	0	**LIVGLMRPPAYCDAK** Oxidation (M) (No match)
114 - 122	1074.6207	1073.6134	1073.5903	0.0232	0	**CLIEILASR** (No match)
123 - 135	1548.7859	1547.7786	1547.7402	0.0385	0	**TNEQMHQLVAAYK** Oxidation (M) (No match)
141 - 156	1745.8837	1744.8764	1744.8267	0.0497	0	**DLEADIIGDTSGHFQK** (No match)
157 - 166	1145.6919	1144.6846	1144.6638	0.0209	0	**MLVVLLQGTR** Oxidation (M) (No match)
167 - 191	2865.4036	2864.3963	2864.3086	0.0877	0	**EEDDVVSEDLVQQDVQDLYEAGELK** (No match)
213 - 220	1026.5664	1025.5591	1025.5433	0.0158	0	**LVFDEYLK** (Ions score 36)
213 - 220	1026.5664	1025.5591	1025.5433	0.0158	0	**LVFDEYLK** (No match)
221 - 231	1172.6914	1171.6841	1171.6560	0.0281	0	**TTGKPIEASIR** (No match)
251 - 259	1099.5314	1098.5241	1098.4981	0.0260	0	**STPEYFAER** (Ions score 27)
251 - 259	1099.5314	1098.5241	1098.4981	0.0260	0	**STPEYFAER** (No match)
355 - 370	1687.8492	1686.8419	1686.7961	0.0458	0	**GTVRPANDFNPDADAK** (No match)
355 - 370	1687.8492	1686.8419	1686.7961	0.0458	0	**GTVRPANDFNPDADAK** (Ions score 7)
378 - 393	1768.9524	1767.9451	1767.9003	0.0449	0	**GLGTDEDTIIDIITHR** (No match)
378 - 393	1768.9524	1767.9451	1767.9003	0.0449	0	**GLGTDEDTIIDIITHR** (Ions score 56)
403 - 411	1107.5586	1106.5513	1106.5621	-0.0107	1	**QTFKSHFGR** (No match)
412 - 427	1779.9202	1778.9129	1778.8720	0.0409	1	**DLMTDLKSEISGDLAR** Oxidation (M) (No match)
419 - 427	947.4991	946.4918	946.4719	0.0199	0	**SEISGDLAR** (No match)
428 - 442	1701.9158	1700.9085	1700.8629	0.0456	0	**LILGLMMPPAHYDAK** 2 Oxidation (M) (No match)
457 - 465	999.6421	998.6348	998.6124	0.0224	0	**ALIEILATR** (No match)
472 - 483	1480.7532	1479.7459	1479.6993	0.0466	1	**AINEAYKEDYHK** (No match)
484 - 498	1621.7881	1620.7808	1620.7379	0.0429	0	**SLEDALSSDTSGHFR** (No match)
500 - 509	1080.6541	1079.6468	1079.6451	0.0017	0	**ILISLATGHR** (No match)
500 - 520	2279.2302	2278.2229	2278.1665	0.0565	1	**ILISLATGHREEGGENLDQAR** (No match)
510 - 520	1217.5732	1216.5659	1216.5319	0.0340	0	**EEGGENLDQAR** (No match)
521 - 540	2072.0657	2071.0584	2070.9956	0.0628	0	**EDAQVAAEILEIADTPSGDK** (No match)
521 - 546	2759.4175	2758.4102	2758.3508	0.0594	1	**EDAQVAAEILEIADTPSGDKTSLETR** (No match)
547 - 554	1057.5731	1056.5658	1056.5096	0.0562	0	**FMTILCTR** Oxidation (M) (No match)
555 - 561	928.5279	927.5206	927.5038	0.0168	1	**SYPHLRR** (No match)
561 - 568	1066.6300	1065.6227	1065.5970	0.0257	1	**RVFQEFIK** (No match)
562 - 568	910.5151	909.5078	909.4959	0.0119	0	**VFQEFIK** (No match)
581 - 587	809.3582	808.3509	808.3385	0.0125	0	**EMSGDVR** Oxidation (M) (No match)
588 - 598	1176.6833	1175.6760	1175.6550	0.0211	0	**DAFVAIVQSVK** (No match)
599 - 607	1079.6091	1078.6018	1078.5811	0.0208	0	**NKPLFFADK** (No match)
599 - 610	1483.8684	1482.8611	1482.8234	0.0377	1	**NKPLFFADKLYK** (No match)
630 - 638	1072.6223	1071.6150	1071.5924	0.0226	0	**SEIDLLNIR** (No match)
630 - 639	1228.7310	1227.7237	1227.6935	0.0302	1	**SEIDLLNIRR** (No match)
648 - 663	1717.8949	1716.8876	1716.8318	0.0558	0	**SLHQAIEGDTSGDFLK** (No match)

Spot 264

Mascot Search Results

Protein View

Match to: **gi|118600975** Score: **170** Expect: **1.9e-012**
syntaxin binding protein 3 [Homo sapiens]

Nominal mass (M_r): **68633;** Calculated pI value: **7.98**
NCBI BLAST search of gi|118600975 against nr
Unformatted sequence string for pasting into other applications

Taxonomy: Homo sapiens
Links to retrieve other entries containing this sequence from NCBI Entrez:
gi|3820482 from Homo sapiens
gi|28838618 from Homo sapiens
gi|55665678 from Homo sapiens
gi|55960045 from Homo sapiens
gi|119576734 from Homo sapiens
gi|158261291 (no taxonomy information for this entry)

Fixed modifications: Carbamidomethyl (C)
Variable modifications: Oxidation (M)
Cleavage by Trypsin: cuts C-term side of KR unless next residue is P
Sequence Coverage: **13%**

Matched peptides shown in **Bold Red**

```
  1 MAPPVAERGL KSVVWQKIKA TVFDDCKKEG EWKIMLLDEF TTKLLASCCK
 51 MTDLLEEGIT VVENIYKNRE PVRQMKALYF ITPTSKSVDC FLHDFASKSE
101 NKYKAAYIYF TDFCPDNLFN KIKASCSKSI RRCKEINISF IPHESQVYTL
151 DVPDAFYYCY SPDPGNAKGK DAIMETMADQ IVTVCATLDE NPGVRYKSKP
201 LDNASKLAQL VEKKLEDYYK IDEKSLIKGK THSQLLIIDR GFDPVSTVLH
251 ELTFQAMAYD LLPIENDTYK YKTDGKEKEA ILEEEDDLWV RIRHRHIAVV
301 LEEIPKLMKE ISSTKKATEG KTSLSALTQL MKKMPHFRKQ ITKQVVHLNL
351 AEDCMNKFKL NIEKLCKTEQ DLALGTDAEG QKVKDSMRVL LPVLLNKNHD
401 NCDKIRAILL YIFSINGTTE ENLDRLIQNV KIENESDMIR NWSYLGVPIV
451 PQSQQGKPLR KDRSAEETFQ LSRWTPFIKD IMEDAIDNRL DSKEWPYCSQ
501 CPAVWNGSGA VSARQKPRAN YLEDRKNGSK LIVFVIGGIT YSEVRCAYEV
551 SQAHKSCEVI IGSTHVLTPK KLLDDIKMLN KPKDKVSLIK DE
```

Residue Number Increasing Mass Decreasing Mass

Start - End	Observed	Mr(expt)	Mr(calc)	Delta	Miss	Sequence
105 - 121	2098.9075	2097.9002	2097.9505	-0.0503	0	AAYIYFTDFCPDNLFNK (Ions score 41)
105 - 121	2098.9075	2097.9002	2097.9505	-0.0503	0	AAYIYFTDFCPDNLFNK (No match)
231 - 240	1195.6538	1194.6465	1194.6720	-0.0255	0	THSQLLIIDR (No match)
231 - 240	1195.6538	1194.6465	1194.6720	-0.0255	0	THSQLLIIDR (Ions score 37)
389 - 397	1008.6570	1007.6497	1007.6742	-0.0245	0	VLLPVLLNK (No match)
407 - 425	2182.0803	2181.0730	2181.1317	-0.0586	0	AILLYIFSINGTTEENLDR (No match)
464 - 473	1167.5398	1166.5325	1166.5567	-0.0242	0	SAEETFQLSR (No match)
531 - 545	1665.9169	1664.9096	1664.9500	-0.0404	0	LIVFVIGGITYSEVR (Ions score 56)
531 - 545	1665.9169	1664.9096	1664.9500	-0.0404	0	LIVFVIGGITYSEVR (No match)

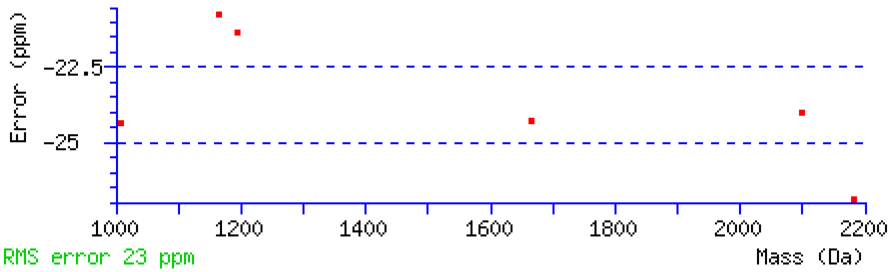

RMS error 23 ppm

Mascot: http://www.matrixscience.com/

Spot 265

{MATRIX}{SCIENCE} Mascot Search Results

Protein View

Match to: **gi|1658374** Score: **77** Expect: **0.0043**
lysosomal acid alpha-mannosidase [Homo sapiens]

Nominal mass (M_r): **111986**; Calculated pI value: **6.65**
NCBI BLAST search of gi|1658374 against nr
Unformatted sequence string for pasting into other applications

Taxonomy: Homo sapiens

Fixed modifications: Carbamidomethyl (C)
Variable modifications: Oxidation (M)
Cleavage by Trypsin: cuts C-term side of KR unless next residue is P
Sequence Coverage: **3%**

Matched peptides shown in **Bold Red**

```
  1 MSRALRPPLP PLCFFLLLLA AAGARAGGYE TCPTVQPNML NVHLLPHTHD
 51 DVGWLKTVDQ YFYGIKNDIQ HAGVQYILDS VISALLADPT RRFIYVEIAF
101 FSRWWHQQTN ATQEVVRDLV RQGRLEFANG GWVMNDEAAT HYGAIVDQMT
151 LGLRFLEDTF GNDGRPRVAW HIDPFGHSRE QASLFAQMGF DGFFFGRLDY
201 QDKWVRMQKL EMEQVWRAST SLKPPTADLF TGVLPNGYNP PRNLCWDVLC
251 VDQPLVEDPR SPEYNAKELV DYFLNVATAQ GRYYRTNHIV MTMGSDFQYE
301 NANMWFKNLD KLIQLVNAQQ AKGSSVHVLY STPACYLWEL NKANLTWSVK
351 HDDFFPYADG HHQFWTGYFS SRPALKRYER LSYNFLQVCN QLEALVGLAA
401 NVGPYGSGDS APLNEAMAVL QHHDAVSGTS RQHVANDYAR QLAAGWGPCE
451 VLLSNALARL RGFKDHFTFC QQLNISICPL SQTAARFQVI VYNPLGRKVN
501 WMVRLPVSEG VFVVKDPNGR TVPSDVVIFP SSDSQAHPPE LLFSASLPAL
551 GFSTYSVAQV PRWKPQARAP QPIPRRSWSP ALTIENEHIR ATFDPDTGLL
601 MEIMNMNQQL LLPVRQTFFW YNASIGDNES DQASGAYIFR PNQQKPLPVS
651 RWAQIHLVKT PLVQEVHQNF SAWCSQVVRL YPGQRHLELE WSVGPIPVGD
701 TWGKEVISRF DTPLETKGRF YTDSNGREIL ERRRDYRPTW KLNQTEPVAG
751 NYYPVNTRIY ITDGNMQLTV LTDRSQGGSS LRDGSLELMV HRRLLKDDGR
801 GVSEPLMENG SGAWVRGRHL VLLDTAQAAA AGHRLLAEQE VLAPQVVLAP
851 GGGAAYNLGA PPRTQFSGLR RDLPPSVHLL TLASWGPEMV LLRLEHQFAV
901 GEDSGRNLSA PVTLNLRDLF STFTITRLQE TTLVANQLRE AASRLKWTTN
951 TGPTPHQTPY QLDPANITLE PMEIRTFLAS VQWKEVDG
```

Residue Number Increasing Mass Decreasing Mass

Start - End	Observed	Mr(expt)	Mr(calc)	Delta	Miss	Sequence
155 - 167	1523.7239	1522.7166	1522.7164	0.0002	0	FLEDTFGNDGRPR (No match)
155 - 167	1523.7239	1522.7166	1522.7164	0.0002	0	FLEDTFGNDGRPR (Ions score 27)
268 - 282	1695.8741	1694.8668	1694.8627	0.0041	0	ELVDYFLNVATAQGR (No match)
487 - 497	1305.7249	1304.7176	1304.7240	-0.0064	0	FQVIVYNPLGR (No match)
487 - 497	1305.7249	1304.7176	1304.7240	-0.0064	0	FQVIVYNPLGR (Ions score 28)

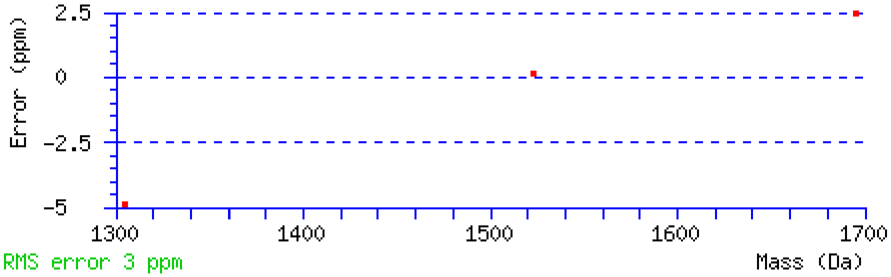

Mascot: http://www.matrixscience.com/

Spot 266

Mascot Search Results

Protein View

Match to: **gi|4504965** Score: **838** Expect: **3e-079**
L-plastin [Homo sapiens]

Nominal mass (M_r): **70815**; Calculated pI value: **5.20**
NCBI BLAST search of gi|4504965 against nr
Unformatted sequence string for pasting into other applications

Taxonomy: Homo sapiens
Links to retrieve other entries containing this sequence from NCBI Entrez:
gi|114651523 from Pan troglodytes
gi|114651525 from Pan troglodytes
gi|114651527 from Pan troglodytes
gi|114651529 from Pan troglodytes
gi|114651531 from Pan troglodytes
gi|114651533 from Pan troglodytes
gi|114651535 from Pan troglodytes
gi|114651537 from Pan troglodytes
gi|114651539 from Pan troglodytes
gi|114651541 from Pan troglodytes
gi|114651543 from Pan troglodytes
gi|114651545 from Pan troglodytes
gi|114651547 from Pan troglodytes
gi|114651549 from Pan troglodytes
gi|114651551 from Pan troglodytes
gi|1346733 from Homo sapiens
gi|189502 from Homo sapiens
gi|14043359 from Homo sapiens
gi|16307448 from Homo sapiens
gi|123988880 from synthetic construct
gi|123999207 from synthetic construct

Fixed modifications: Carbamidomethyl (C)
Variable modifications: Oxidation (M)
Cleavage by Trypsin: cuts C-term side of KR unless next residue is P
Sequence Coverage: **65%**

Matched peptides shown in **Bold Red**

```
  1 MARGSVSDEE MMELREAFAK VDTDGNGYIS FNELNDLFKA ACLPLPGYRV
 51 REITENLMAT GDLDQDGRIS FDEFIKIFHG LKSTDVAKTF RKAINKKEGI
101 CAIGGTSEQS SVGTQHSYSE EEKYAFVNWI NKALENDPDC RHVIPMNPNT
151 NDLFNAVGDG IVLCKMINLS VPDTIDERTI NKKKLTPFTI QENLNLALNS
201 ASAIGCHVVN IGAEDLKEGK PYLVLGLLWQ VIKIGLFADI ELSRNEALIA
251 LLREGESLED LMKLSPEELL LRWANYHLEN AGCNKIGNFS TDIKDSKAYY
301 HLLEQVAPKG DEEGVPAVVI DMSGLREKDD IQRAECMLQQ AERLGCRQFV
351 TATDVVRGNP KLNLAFIANL FNRYPALHKP ENQDIDWGAL EGETREERTF
401 RNWMNSLGVN PRVNHLYSDL SDALVIFQLY EKIKVPVDWN RVNKPPYPKL
451 GGNMKKLENC NYAVELGKNQ AKFSLVGIGG QDLNEGNRTL TLALIWQLMR
501 RYTLNILEEI GGGQKVNDDI IVNWVNETLR EAEKSSSISS FKDPKISTSL
551 PVLDLIDAIQ PGSINYDLLK TENLNDDEKL NNAKYAISMA RKIGARVYAL
601 PEDLVEVNPK MVMTVFACLM GKGMKRV
```

Residue Number Increasing Mass Decreasing Mass

Start - End	Observed	Mr(expt)	Mr(calc)	Delta	Miss	Sequence
4 - 15	1382.5691	1381.5618	1381.5853	-0.0235	0	GSVSDEEMMELR (No match)
4 - 15	1398.5630	1397.5557	1397.5802	-0.0245	0	GSVSDEEMMELR Oxidation (M) (No match)
4 - 15	1414.5487	1413.5414	1413.5751	-0.0337	0	GSVSDEEMMELR 2 Oxidation (M) (No match)
21 - 39	2161.0007	2159.9934	2160.0010	-0.0076	0	VDTDGNGYISFNELNDLFK (No match)
40 - 49	1117.5648	1116.5575	1116.5750	-0.0174	0	AACLPLPGYR (No match)
40 - 49	1117.5648	1116.5575	1116.5750	-0.0174	0	AACLPLPGYR (Ions score 31)
52 - 68	1877.8274	1876.8201	1876.8472	-0.0271	0	EITENLMATGDLDQDGR (No match)
52 - 68	1893.8331	1892.8258	1892.8421	-0.0163	0	EITENLMATGDLDQDGR Oxidation (M) (No match)
69 - 76	998.4991	997.4918	997.5120	-0.0202	0	ISFDEFIK (No match)
98 - 123	2770.2219	2769.2146	2769.2034	0.0112	0	EGICAIGGTSEQSSVGTQHSYSEEEK (No match)
124 - 132	1154.5771	1153.5698	1153.5920	-0.0221	0	YAFVNWINK (No match)
133 - 141	1089.4447	1088.4374	1088.4556	-0.0182	0	ALENDPDCR (No match)
142 - 165	2638.3247	2637.3174	2637.3042	0.0132	0	HVIPMNPNTNDLFNAVGDGIVLCK (No match)
166 - 178	1502.7274	1501.7201	1501.7446	-0.0245	0	MINLSVPDTIDER (No match)
166 - 178	1518.7247	1517.7174	1517.7395	-0.0221	0	MINLSVPDTIDER Oxidation (M) (No match)
234 - 244	1233.6626	1232.6553	1232.6764	-0.0211	0	IGLFADIELSR (No match)
245 - 253	1012.6002	1011.5929	1011.6076	-0.0147	0	NEALIALLR (No match)
264 - 272	1069.6088	1068.6015	1068.6179	-0.0163	0	LSPEELLLR (No match)
273 - 285	1576.6741	1575.6668	1575.6888	-0.0220	0	WANYHLENAGCNK (No match)
298 - 309	1431.7382	1430.7309	1430.7557	-0.0248	0	AYYHLLEQVAPK (No match)
310 - 326	1743.8307	1742.8234	1742.8508	-0.0274	0	GDEEGVPAVVIDMSGLR (Ions score 139)
310 - 326	1743.8307	1742.8234	1742.8508	-0.0274	0	GDEEGVPAVVIDMSGLR (No match)
310 - 326	1759.8330	1758.8257	1758.8457	-0.0200	0	GDEEGVPAVVIDMSGLR Oxidation (M) (No match)
327 - 333	903.4408	902.4335	902.4457	-0.0122	1	EKDDIQR (No match)
334 - 343	1235.5776	1234.5703	1234.5434	0.0269	0	AECMLQQAER (No match)
334 - 343	1251.5245	1250.5172	1250.5383	-0.0211	0	AECMLQQAER Oxidation (M) (No match)
348 - 357	1135.5917	1134.5844	1134.6033	-0.0188	0	QFVTATDVVR (No match)

362 - 373	1405.7727	1404.7654	1404.7877	-0.0223	0	**LNLAFIANLFNR** (No match)
362 - 373	1405.7727	1404.7654	1404.7877	-0.0223	0	**LNLAFIANLFNR** (Ions score 79)
374 - 395	2539.1838	2538.1765	2538.2138	-0.0373	0	**YPALHKPENQDIDWGALEGETR** (No match)
402 - 412	1287.6030	1286.5957	1286.6189	-0.0232	0	**NWMNSLGVNPR** (No match)
402 - 412	1287.6030	1286.5957	1286.6189	-0.0232	0	**NWMNSLGVNPR** (Ions score 98)
402 - 412	1303.5969	1302.5896	1302.6138	-0.0242	0	**NWMNSLGVNPR** Oxidation (M) (No match)
413 - 432	2367.2141	2366.2068	2366.2157	-0.0089	0	**VNHLYSDLSDALVIFQLYEK** (No match)
433 - 441	1126.6174	1125.6101	1125.6294	-0.0193	1	**IKVPVDWNR** (No match)
435 - 441	885.4465	884.4392	884.4504	-0.0112	0	**VPVDWNR** (No match)
456 - 468	1537.7635	1536.7562	1536.7605	-0.0043	1	**KLENCNYAVELGK** (No match)
473 - 488	1675.8131	1674.8058	1674.8324	-0.0266	0	**FSLVGIGGQDLNEGNR** (Ions score 122)
473 - 488	1675.8131	1674.8058	1674.8324	-0.0266	0	**FSLVGIGGQDLNEGNR** (No match)
489 - 500	1458.8239	1457.8166	1457.8428	-0.0262	0	**TLTLALIWQLMR** (No match)
489 - 500	1474.8195	1473.8122	1473.8377	-0.0255	0	**TLTLALIWQLMR** Oxidation (M) (No match)
501 - 515	1690.8832	1689.8759	1689.9049	-0.0290	1	**RYTLNILEEIGGGQK** (No match)
502 - 515	1534.7896	1533.7823	1533.8038	-0.0215	0	**YTLNILEEIGGGQK** (No match)
516 - 530	1799.8992	1798.8919	1798.9213	-0.0294	0	**VNDDIIVNWVNETLR** (No match)
585 - 591	811.4052	810.3979	810.4058	-0.0079	0	**YAISMAR** (No match)
597 - 610	1585.8247	1584.8174	1584.8398	-0.0224	0	**VYALPEDLVEVNPK** (No match)

Mascot: http://www.matrixscience.com/

Spot 267

{MATRIX SCIENCE} Mascot Search Results

Protein View

Match to: **gi|62898171** Score: **427** Expect: **3.8e-038**
L-plastin variant [Homo sapiens]

Nominal mass (M_r): **70785**; Calculated pI value: **5.20**
NCBI BLAST search of gi|62898171 against nr
Unformatted sequence string for pasting into other applications

Taxonomy: Homo sapiens

Fixed modifications: Carbamidomethyl (C)
Variable modifications: Oxidation (M)
Cleavage by Trypsin: cuts C-term side of KR unless next residue is P
Sequence Coverage: **41%**

Matched peptides shown in **Bold Red**

```
  1 MARGSVSDEE MMELREAFAK VDTDGNGYIS FNELNDLFKA ACLPLPGYRV
 51 REITENLMAT GDLDQDGRIS FDEFIKIFHG LKSTDVAKTF RKAINKKEGI
101 CAIGGTSEQS SVGTQHSYSE EEKYAFVNWI NKALENDPDC RHVIPMNPNT
151 NDLFNAVGDG IVLCKMINLS VPDTIDERTI NKKKLTPFTI QENLNLALNS
201 ASAIGCHVVN IGAEDLKEGK PYLVLGLLWQ VIKIGLFADI ELSRNEALIA
251 LLREGESLED LMKLSPEELL LRWANYHLEN AGCNKIGNFS TDIKDSKAYY
301 HLLEQVAPKG DEEGVPAVVI DMSGLREKDD IQRAECMLQQ AERLGCRQFV
351 TATDVVRGNP KLNLAFIANL FNRYPALHKP ENQDIDWGAL EGETREERTF
401 RNWMNSLGVN PRVNHLYGDL SDALVIFQLY EKIKVPVDWN RVNKPPYPKL
451 GGNMKKLENC NYAVELGKNQ AKFSLVGIGG QDLNEGNRTL TLALIWQLMR
501 RYTLNILEEI GGGQKVNDDI IVNWVNETLR EAEKSSSISS FKDPKISTSL
551 PVLDLIDAIQ PGSINYDLLK TENLNDDEKL NNAKYAISMA RKIGARVYAL
601 PEDLVEVNPK MVMTVFACLM GKGMKRV
```

Residue Number Increasing Mass Decreasing Mass

Start - End	Observed	Mr(expt)	Mr(calc)	Delta	Miss	Sequence
4 - 15	1414.6520	1413.6447	1413.5751	0.0696	0	GSVSDEEMMELR 2 Oxidation (M) (No match)
21 - 39	2161.1294	2160.1221	2160.0010	0.1211	0	VDTDGNGYISFNELNDLFK (No match)

40 - 49	1117.6396	1116.6323	1116.5750	0.0574	0	**AACLPLPGYR**	(Ions score 21)
40 - 49	1117.6396	1116.6323	1116.5750	0.0574	0	**AACLPLPGYR**	(No match)
50 - 68	2149.1433	2148.1360	2148.0116	0.1244	1	**VREITENLMATGDLDQDGR**	Oxidation (M) (No match)
52 - 68	1893.9567	1892.9494	1892.8421	0.1073	0	**EITENLMATGDLDQDGR**	Oxidation (M) (No match)
69 - 76	998.5613	997.5540	997.5120	0.0420	0	**ISFDEFIK**	(No match)
133 - 141	1089.5182	1088.5109	1088.4556	0.0553	0	**ALENDPDCR**	(No match)
166 - 178	1518.8228	1517.8155	1517.7395	0.0760	0	**MINLSVPDTIDER**	Oxidation (M) (No match)
234 - 244	1233.7474	1232.7401	1232.6764	0.0637	0	**IGLFADIELSR**	(No match)
245 - 253	1012.6636	1011.6563	1011.6076	0.0487	0	**NEALIALLR**	(No match)
254 - 272	2217.2542	2216.2469	2216.1245	0.1224	1	**EGESLEDLMKLSPEELLLR**	Oxidation (M) (No match)
264 - 272	1069.6772	1068.6699	1068.6179	0.0521	0	**LSPEELLLR**	(No match)
264 - 272	1069.6772	1068.6699	1068.6179	0.0521	0	**LSPEELLLR**	(Ions score 23)
298 - 309	1431.8346	1430.8273	1430.7557	0.0716	0	**AYYHLLEQVAPK**	(No match)
310 - 326	1759.9463	1758.9390	1758.8457	0.0933	0	**GDEEGVPAVVIDMSGLR**	Oxidation (M) (Ions score 22)
310 - 326	1759.9463	1758.9390	1758.8457	0.0933	0	**GDEEGVPAVVIDMSGLR**	Oxidation (M) (No match)
327 - 333	903.4916	902.4843	902.4457	0.0386	1	**EKDDIQR**	(No match)
334 - 343	1251.6021	1250.5948	1250.5383	0.0565	0	**AECMLQQAER**	Oxidation (M) (No match)
348 - 357	1135.6642	1134.6569	1134.6033	0.0537	0	**QFVTATDVVR**	(No match)
362 - 373	1405.8698	1404.8625	1404.7877	0.0748	0	**LNLAFIANLFNR**	(No match)
362 - 373	1405.8698	1404.8625	1404.7877	0.0748	0	**LNLAFIANLFNR**	(Ions score 61)
402 - 412	1303.6849	1302.6776	1302.6138	0.0638	0	**NWMNSLGVNPR**	Oxidation (M) (No match)
473 - 488	1675.9282	1674.9209	1674.8324	0.0885	0	**FSLVGIGGQDLNEGNR**	(Ions score 63)
473 - 488	1675.9282	1674.9209	1674.8324	0.0885	0	**FSLVGIGGQDLNEGNR**	(No match)
501 - 515	1690.9969	1689.9896	1689.9049	0.0847	1	**RYTLNILEEIGGGQK**	(No match)
502 - 515	1534.8815	1533.8742	1533.8038	0.0704	0	**YTLNILEEIGGGQK**	(No match)
585 - 591	827.4422	826.4349	826.4007	0.0342	0	**YAISMAR**	Oxidation (M) (No match)
597 - 610	1585.9281	1584.9208	1584.8398	0.0810	0	**VYALPEDLVEVNPK**	(No match)

Mascot: http://www.matrixscience.com/

Spot 268

{MATRIX SCIENCE} Mascot Search Results

Protein View

Match to: **gi|4504965** Score: **585** Expect: **6.1e-054**
L-plastin [Homo sapiens]

Nominal mass (M_r): **70815;** Calculated pI value: **5.20**
NCBI BLAST search of gi|4504965 against nr
Unformatted sequence string for pasting into other applications

Taxonomy: Homo sapiens
Links to retrieve other entries containing this sequence from NCBI Entrez:
gi|114651523 from Pan troglodytes
gi|114651525 from Pan troglodytes
gi|114651527 from Pan troglodytes
gi|114651529 from Pan troglodytes
gi|114651531 from Pan troglodytes
gi|114651533 from Pan troglodytes
gi|114651535 from Pan troglodytes
gi|114651537 from Pan troglodytes
gi|114651539 from Pan troglodytes
gi|114651541 from Pan troglodytes
gi|114651543 from Pan troglodytes
gi|114651545 from Pan troglodytes
gi|114651547 from Pan troglodytes
gi|114651549 from Pan troglodytes
gi|114651551 from Pan troglodytes
gi|1346733 from Homo sapiens
gi|189502 from Homo sapiens
gi|14043359 from Homo sapiens
gi|16307448 from Homo sapiens
gi|123988880 from synthetic construct
gi|123999207 from synthetic construct

Fixed modifications: Carbamidomethyl (C)
Variable modifications: Oxidation (M)
Cleavage by Trypsin: cuts C-term side of KR unless next residue is P
Sequence Coverage: **53%**

Matched peptides shown in **Bold Red**

1 MAR**GSVSDEE MMELR**EAFAK **VDTDGNGYIS FNELNDLFKA ACLPLPGYR**V

51 REITENLMAT GDLDQDGR**IS FDEFIK**IFHG LKSTDVAKTF RKAINKKEGI

101 CAIGGTSEQS SVGTQHSYSE EEKYAFVNWI NK**ALENDPDC RHVIPMNPNT**

151 **NDLFNAVGDG IVLCKMINLS VPDTIDER**TI NKKKLTPFTI QENLNLALNS

201 ASAIGCHVVN IGAEDLKEGK PYLVLGLLWQ VIK**IGLFADI ELSRNEALIA**

251 **LLR**EGESLED LMK**LSPEELL LR**WANYHLEN AGCNKIGNFS TDIKDSK**AYY**

301 **HLLEQVAPKG DEEGVPAVVI DMSGLREKDD IQRAECMLQQ AER**LGCR**QFV**

351 **TATDVVR**GNP K**LNLAFIANL FNRYPALHKP ENQDIDWGAL EGETR**EERTF

401 R**NWMNSLGVN PRVNHLYSDL SDALVIFQLY EKIKVPVDWN R**VNKPPYPKL

451 GGNMK**KLENC NYAVELGK**NQ AK**FSLVGIGG QDLNEGNR**TL TLALIWQLMR

501 **RYTLNILEEI GGGQKVNDDI IVNWVNETLR** EAEKSSSISS FKDPKISTSL

551 PVLDLIDAIQ PGSINYDLLK TENLNDDEKL NNAK**YAISMA R**KIGAR**VYAL**

601 **PEDLVEVNPK** MVMTVFACLM GKGMKRV

Residue Number Increasing Mass Decreasing Mass

Start - End	Observed	Mr(expt)	Mr(calc)	Delta	Miss	Sequence
4 - 15	1382.5713	1381.5640	1381.5853	-0.0213	0	GSVSDEEMMELR (No match)
4 - 15	1398.5637	1397.5564	1397.5802	-0.0238	0	GSVSDEEMMELR Oxidation (M) (No match)
21 - 39	2160.9790	2159.9717	2160.0010	-0.0293	0	VDTDGNGYISFNELNDLFK (No match)
40 - 49	1117.5673	1116.5600	1116.5750	-0.0149	0	AACLPLPGYR (Ions score 27)
40 - 49	1117.5673	1116.5600	1116.5750	-0.0149	0	AACLPLPGYR (No match)
69 - 76	998.5004	997.4931	997.5120	-0.0189	0	ISFDEFIK (No match)
133 - 141	1089.4452	1088.4379	1088.4556	-0.0177	0	ALENDPDCR (No match)
142 - 165	2654.2639	2653.2566	2653.2991	-0.0425	0	HVIPMNPNTNDLFNAVGDGIVLCK Oxidation (M) (No match)
166 - 178	1502.7283	1501.7210	1501.7446	-0.0236	0	MINLSVPDTIDER (No match)
166 - 178	1518.7257	1517.7184	1517.7395	-0.0211	0	MINLSVPDTIDER Oxidation (M) (No match)
234 - 244	1233.6678	1232.6605	1232.6764	-0.0159	0	IGLFADIELSR (No match)
245 - 253	1012.6008	1011.5935	1011.6076	-0.0141	0	NEALIALLR (No match)
264 - 272	1069.6089	1068.6016	1068.6179	-0.0162	0	LSPEELLLR (No match)
298 - 309	1431.7374	1430.7301	1430.7557	-0.0256	0	AYYHLLEQVAPK (No match)
310 - 326	1743.8300	1742.8227	1742.8508	-0.0281	0	GDEEGVPAVVIDMSGLR (Ions score 77)
310 - 326	1743.8300	1742.8227	1742.8508	-0.0281	0	GDEEGVPAVVIDMSGLR (No match)
310 - 326	1759.8284	1758.8211	1758.8457	-0.0246	0	GDEEGVPAVVIDMSGLR Oxidation (M) (No match)
327 - 333	903.4378	902.4305	902.4457	-0.0152	1	EKDDIQR (No match)
334 - 343	1235.5365	1234.5292	1234.5434	-0.0142	0	AECMLQQAER (No match)
334 - 343	1251.5281	1250.5208	1250.5383	-0.0175	0	AECMLQQAER Oxidation (M) (No match)
348 - 357	1135.5933	1134.5860	1134.6033	-0.0172	0	QFVTATDVVR (No match)
362 - 373	1405.7739	1404.7666	1404.7877	-0.0211	0	LNLAFIANLFNR (Ions score 68)
362 - 373	1405.7739	1404.7666	1404.7877	-0.0211	0	LNLAFIANLFNR (No match)
374 - 395	2539.1670	2538.1597	2538.2138	-0.0541	0	YPALHKPENQDIDWGALEGETR (No match)
402 - 412	1287.6038	1286.5965	1286.6189	-0.0224	0	NWMNSLGVNPR (No match)
402 - 412	1303.5999	1302.5926	1302.6138	-0.0212	0	NWMNSLGVNPR Oxidation (M) (No match)
413 - 432	2367.1799	2366.1726	2366.2157	-0.0431	0	VNHLYSDLSDALVIFQLYEK (No match)
433 - 441	1126.6200	1125.6127	1125.6294	-0.0167	1	IKVPVDWNR (No match)

456 - 468	1537.7623	1536.7550	1536.7605	-0.0055	1	**KLENCNYAVELGK**	(No match)
473 - 488	1675.8130	1674.8057	1674.8324	-0.0267	0	**FSLVGIGGQDLNEGNR**	(Ions score 107)
473 - 488	1675.8130	1674.8057	1674.8324	-0.0267	0	**FSLVGIGGQDLNEGNR**	(No match)
501 - 515	1690.8838	1689.8765	1689.9049	-0.0284	1	**RYTLNILEEIGGGQK**	(No match)
502 - 515	1534.7836	1533.7763	1533.8038	-0.0275	0	**YTLNILEEIGGGQK**	(No match)
516 - 530	1799.8970	1798.8897	1798.9213	-0.0316	0	**VNDDIIVNWVNETLR**	(No match)
585 - 591	811.3981	810.3908	810.4058	-0.0150	0	**YAISMAR**	(No match)
597 - 610	1585.8195	1584.8122	1584.8398	-0.0276	0	**VYALPEDLVEVNPK**	(No match)

Mascot: http://www.matrixscience.com/

Spot 269

{MATRIX SCIENCE} Mascot Search Results

Protein View

Match to: **gi|4504965** Score: **440** Expect: **1.9e-039**
L-plastin [Homo sapiens]

Nominal mass (M_r): **70815;** Calculated pI value: **5.20**
NCBI BLAST search of gi|4504965 against nr
Unformatted sequence string for pasting into other applications

Taxonomy: Homo sapiens
Links to retrieve other entries containing this sequence from NCBI Entrez:
gi|114651523 from Pan troglodytes
gi|114651525 from Pan troglodytes
gi|114651527 from Pan troglodytes
gi|114651529 from Pan troglodytes
gi|114651531 from Pan troglodytes
gi|114651533 from Pan troglodytes
gi|114651535 from Pan troglodytes
gi|114651537 from Pan troglodytes
gi|114651539 from Pan troglodytes
gi|114651541 from Pan troglodytes
gi|114651543 from Pan troglodytes
gi|114651545 from Pan troglodytes
gi|114651547 from Pan troglodytes
gi|114651549 from Pan troglodytes
gi|114651551 from Pan troglodytes
gi|1346733 from Homo sapiens
gi|189502 from Homo sapiens
gi|14043359 from Homo sapiens
gi|16307448 from Homo sapiens
gi|123988880 from synthetic construct
gi|123999207 from synthetic construct

Fixed modifications: Carbamidomethyl (C)
Variable modifications: Oxidation (M)
Cleavage by Trypsin: cuts C-term side of KR unless next residue is P
Sequence Coverage: **48%**

Matched peptides shown in **Bold Red**

1 MAR**GSVSDEE** **MMELR**EAFAK **VDTDGNGYIS** **FNELNDLFKA** **ACLPLPGYRV**
51 **REITENLMAT** **GDLDQDGRIS** **FDEFIK**IFHG LKSTDVAKTF RKAINKKEGI
101 CAIGGTSEQS SVGTQHSYSE EEKYAFVNWI NK**ALENDPDC** **R**HVIPMNPNT
151 NDLFNAVGDG IVLCK**MINLS** **VPDTIDER**TI NKKKLTPFTI QENLNLALNS
201 ASAIGCHVVN IGAEDLKEGK PYLVLGLLWQ VIK**IGLFADI** **ELSRNEALIA**
251 **LLREGESLED** **LMKLSPEELL** **LR**WANYHLEN AGCNKIGNFS TDIKDSK**AYY**
301 **HLLEQVAPKG** **DEEGVPAVVI** **DMSGLREKDD** **IQRAECMLQQ** **AER**LGCR**QFV**
351 **TATDVVR**GNP K**LNLAFIANL** **FNR**YPALHKP ENQDIDWGAL EGETREERTF
401 R**NWMNSLGVN** **PRVNHLYSDL** **SDALVIFQLY** **EK**IKVPVDWN R**VNKPPYPK**L
451 GGNMKKLENC NYAVELGKNQ AK**FSLVGIGG** **QDLNEGNR**TL TLALIWQLMR
501 **RYTLNILEEI** **GGGQKVNDDI** **IVNWVNETLR** EAEKSSSISS FKDPKISTSL
551 PVLDLIDAIQ PGSINYDLLK TENLNDDEKL NNAK**YAISMA** **R**KIGAR**VYAL**
601 **PEDLVEVNPK** MVMTVFACLM GKGMKRV

Residue Number Increasing Mass Decreasing Mass

Start - End	Observed	Mr(expt)	Mr(calc)	Delta	Miss	Sequence
4 - 15	1414.6150	1413.6077	1413.5751	0.0326	0	GSVSDEEMMELR 2 Oxidation (M) (No match)
21 - 39	2161.0713	2160.0640	2160.0010	0.0630	0	VDTDGNGYISFNELNDLFK (No match)
40 - 49	1117.6085	1116.6012	1116.5750	0.0263	0	AACLPLPGYR (No match)
50 - 68	2149.0903	2148.0830	2148.0116	0.0714	1	VREITENLMATGDLDQDGR Oxidation (M) (No match)
52 - 68	1893.9015	1892.8942	1892.8421	0.0521	0	EITENLMATGDLDQDGR Oxidation (M) (No match)
69 - 76	998.5353	997.5280	997.5120	0.0160	0	ISFDEFIK (No match)
133 - 141	1089.4868	1088.4795	1088.4556	0.0239	0	ALENDPDCR (No match)
166 - 178	1518.7831	1517.7758	1517.7395	0.0363	0	MINLSVPDTIDER Oxidation (M) (No match)
234 - 244	1233.7128	1232.7055	1232.6764	0.0291	0	IGLFADIELSR (No match)
245 - 253	1012.6366	1011.6293	1011.6076	0.0217	0	NEALIALLR (No match)
254 - 272	2217.1917	2216.1844	2216.1245	0.0599	1	EGESLEDLMKLSPEELLLR Oxidation (M) (No match)
264 - 272	1069.6481	1068.6408	1068.6179	0.0230	0	LSPEELLLR (Ions score 28)
264 - 272	1069.6481	1068.6408	1068.6179	0.0230	0	LSPEELLLR (No match)
298 - 309	1431.7946	1430.7873	1430.7557	0.0316	0	AYYHLLEQVAPK (No match)
298 - 326	3172.6785	3171.6712	3171.5909	0.0803	1	AYYHLLEQVAPKGDEEGVPAVVIDMSGLR Oxidation (M) (No match)
310 - 326	1759.8995	1758.8922	1758.8457	0.0465	0	GDEEGVPAVVIDMSGLR Oxidation (M) (No match)
310 - 326	1759.8995	1758.8922	1758.8457	0.0465	0	GDEEGVPAVVIDMSGLR Oxidation (M) (Ions score 19)
327 - 333	903.4694	902.4621	902.4457	0.0164	1	EKDDIQR (No match)
334 - 343	1251.5759	1250.5686	1250.5383	0.0303	0	AECMLQQAER Oxidation (M) (No match)
348 - 357	1135.6344	1134.6271	1134.6033	0.0239	0	QFVTATDVVR (No match)
362 - 373	1405.8301	1404.8228	1404.7877	0.0351	0	LNLAFIANLFNR (No match)
362 - 373	1405.8301	1404.8228	1404.7877	0.0351	0	LNLAFIANLFNR (Ions score 57)
402 - 412	1303.6508	1302.6435	1302.6138	0.0297	0	NWMNSLGVNPR Oxidation (M) (No match)
413 - 432	2367.2837	2366.2764	2366.2157	0.0607	0	VNHLYSDLSDALVIFQLYEK (No match)
442 - 449	942.5533	941.5460	941.5334	0.0126	0	VNKPPYPK (No match)
473 - 488	1675.8821	1674.8748	1674.8324	0.0424	0	FSLVGIGGQDLNEGNR (No match)
473 - 488	1675.8821	1674.8748	1674.8324	0.0424	0	FSLVGIGGQDLNEGNR (Ions score 63)
501 - 515	1690.9523	1689.9450	1689.9049	0.0401	1	RYTLNILEEIGGGQK (No match)

502 - 515	1534.8424	1533.8351	1533.8038	0.0313	0	**YTLNILEEIGGGQK** (No match)
516 - 530	1799.9683	1798.9610	1798.9213	0.0397	0	**VNDDIIVNWVNETLR** (No match)
585 - 591	827.4174	826.4101	826.4007	0.0094	0	**YAISMAR** Oxidation (M) (No match)
597 - 610	1585.8872	1584.8799	1584.8398	0.0401	0	**VYALPEDLVEVNPK** (No match)

Mascot: http://www.matrixscience.com/

Spot 272

MATRIX SCIENCE Mascot Search Results

Protein View

Match to: **gi|62087548** Score: **105** Expect: **6.1e-006**
L-plastin variant [Homo sapiens]

Nominal mass (M_r): **56196;** Calculated pI value: **5.21**
NCBI BLAST search of gi|62087548 against nr
Unformatted sequence string for pasting into other applications

Taxonomy: Homo sapiens

Fixed modifications: Carbamidomethyl (C)
Variable modifications: Oxidation (M)
Cleavage by Trypsin: cuts C-term side of KR unless next residue is P
Sequence Coverage: **3%**

Matched peptides shown in **Bold Red**

```
  1 KMARGSVSDE EMMELREAFA KVDTDGNGYI SFNELNDLFK AACLPLPGYR
 51 VREITENLMA TGDLDQDGRI SFDEFIKIFH GLKSTDVAKT FRKAINKKEG
101 ICAIGGTSEQ SSVGTQHSYS EEEKYAFVNW INKALENDPD CRHVIPMNPN
151 TNDLFNAVGD GIVLCKMINL SVPDTIDERT INKKKLTPFT IQENLNLALN
201 SASAIGCHVV NIGAEDLKEG KPYLVLGLLW QVIKIGLFAD IELSRNEALI
251 ALLREGESLE DLMKLSPEEL LLRWANYHLE NAGCNKIGNF STDIKDSKAY
301 YHLLEQVAPK GDEEGVPAVV IDMSGLREKD DIQRAECMLQ QAERLGCRQF
351 VTATDVVRGN PKLNLAFIAN LFNRYPALHK PENQDIDWGA LEGETREERT
401 FRNWMNSLGV NPRVNHLYSD LSDALVIFQL YEKIKVPVDW NRVNKPPYPK
451 LGGNMKKLEN CNYAVELGKN QAKFSLVGIG GQDLNEGNRT LTLALIGS
```

Residue Number Increasing Mass Decreasing Mass

Start - End	Observed	Mr(expt)	Mr(calc)	Delta	Miss	Sequence	
474 - 489	1675.7822	1674.7749	1674.8324	-0.0575	0	FSLVGIGGQDLNEGNR	(No match)
474 - 489	1675.7822	1674.7749	1674.8324	-0.0575	0	FSLVGIGGQDLNEGNR	(Ions score 97)

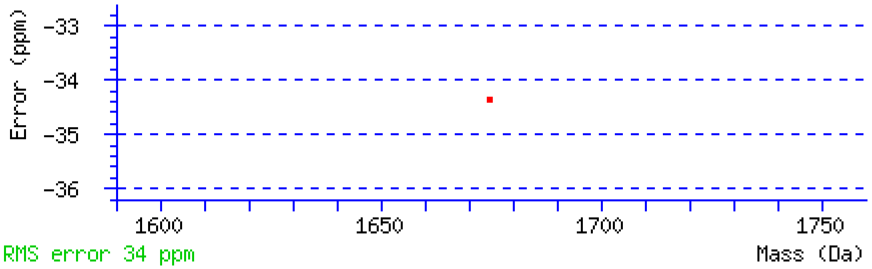

Mascot: http://www.matrixscience.com/

Spot 273

{MATRIX}{SCIENCE} Mascot Search Results

Protein View

Match to: **gi|119631462** Score: **172** Expect: **1.2e-012**
alkylglycerone phosphate synthase, isoform CRA_a [Homo sapiens]

Nominal mass (M_r): **60803**; Calculated pI value: **6.01**
NCBI BLAST search of gi|119631462 against nr
Unformatted sequence string for pasting into other applications

Taxonomy: Homo sapiens

Fixed modifications: Carbamidomethyl (C)
Variable modifications: Oxidation (M)
Cleavage by Trypsin: cuts C-term side of KR unless next residue is P
Sequence Coverage: **15%**

Matched peptides shown in **Bold Red**

```
  1 MGLPTFKEWI QNTLGVNVEH KTTSKASLNP SDTPPSVVNE DFLHDLKETN
 51 ISYSQEADDR VFRAHGHCLH EIFLLREGMF ERIPDIVLWP TCHDDVVKIV
101 NLACKYNLCI IPIGGGTSVS YGLMCPADET RTIISLDTSQ MNRILWVDEN
151 NLTAHVEAGI TGQELERQLK ESGYCTGHEP DSLEFSTVGG WVSTRASGMK
201 KNIYGNIEDL VVHIKMVTPR GIIEKSCQGP RMSTGPDIHH FIMGSEGTLG
251 VITEATIKIR PVPEYQKYGS VAFPNFEQGV ACLREIAKQR CAPASIRLMD
301 NKQFQFGHAL KPQVSSIFTS FLDGLKKFYI TKFKGFDPNQ LSVATLLFEG
351 DREKVLQHEK QVYDIAAKFG GLAAGEDNGQ RGYLLTYVIA YIRDLALEYY
401 VLGESFETSA PWDRVVDLCR NVKERITREC KEKGVQFAPF STCRVTQTYD
451 AGACIYFYFA FNYRGISDPL TVFEQTEAAA REEILANGGS LSHHHGVGKL
501 RKQWLKESIS DVGFGMLKSV KEYVDPNNIF GNRNLL
```

Residue Number Increasing Mass Decreasing Mass

Start - End	Observed	Mr(expt)	Mr(calc)	Delta	Miss	Sequence
64 - 76	1602.8068	1601.7995	1601.8248	-0.0253	0	AHGHCLHEIFLLR (No match)
64 - 76	1602.8068	1601.7995	1601.8248	-0.0253	0	AHGHCLHEIFLLR (Ions score 6)
268 - 284	1914.8837	1913.8764	1913.9093	-0.0329	0	YGSVAFPNFEQGVACLR (No match)
268 - 284	1914.8837	1913.8764	1913.9093	-0.0329	0	YGSVAFPNFEQGVACLR (Ions score 31)

382 - 393	1444.7976	1443.7903	1443.8125	-0.0222	0	GYLLTYVIAYIR	(No match)
382 - 393	1444.7976	1443.7903	1443.8125	-0.0222	0	GYLLTYVIAYIR	(No match)
434 - 444	1269.5914	1268.5841	1268.5971	-0.0130	0	GVQFAPFSTCR	(Ions score 10)
434 - 444	1269.5914	1268.5841	1268.5971	-0.0130	0	GVQFAPFSTCR	(No match)
465 - 481	1804.8799	1803.8726	1803.9002	-0.0276	0	GISDPLTVFEQTEAAAR	(No match)
465 - 481	1804.8799	1803.8726	1803.9002	-0.0276	0	GISDPLTVFEQTEAAAR	(Ions score 46)
522 - 533	1437.6556	1436.6483	1436.6684	-0.0200	0	EYVDPNNIFGNR	(No match)

Mascot: http://www.matrixscience.com/

Spot 274

{MATRIX SCIENCE} Mascot Search Results

Protein View

Match to: **gi|31455611** Score: **161** Expect: **1.5e-011**
zeta-chain associated protein kinase 70kDa isoform 1 [Homo sapiens]

Nominal mass (M_r): **70796;** Calculated pI value: **7.78**
NCBI BLAST search of gi|31455611 against nr
Unformatted sequence string for pasting into other applications

Taxonomy: Homo sapiens
Links to retrieve other entries containing this sequence from NCBI Entrez:
gi|1177044 from Homo sapiens
gi|31753147 from Homo sapiens

Fixed modifications: Carbamidomethyl (C)
Variable modifications: Oxidation (M)
Cleavage by Trypsin: cuts C-term side of KR unless next residue is P
Sequence Coverage: **10%**

Matched peptides shown in **Bold Red**

```
  1 MPDPAAHLPF FYGSISRAEA EEHLKLAGMA DGLFLLRQCL RSLGGYVLSL
 51 VHDVRFHHFP IERQLNGTYA IAGGKAHCGP AELCEFYSRD PDGLPCNLRK
101 PCNRPSGLEP QPGVFDCLRD AMVRDYVRQT WKLEGEALEQ AIISQAPQVE
151 KLIATTAHER MPWYHSSLTR EEAERKLYSG AQTDGKFLLR PRKEQGTYAL
201 SLIYGKTVYH YLISQDKAGK YCIPEGTKFD TLWQLVEYLK LKADGLIYCL
251 KEACPNSSAS NASGAAAPTL PAHPSTLTHP QRRIDTLNSD GYTPEPARIT
301 SPDKPRPMPM DTSVYESPYS DPEELKDKKL FLKRDNLLIA DIELGCGNFG
351 SVRQGVYRMR KKQIDVAIKV LKQGTEKADT EEMMREAQIM HQLDNPYIVR
401 LIGVCQAEAL MLVMEMAGGG PLHKFLVGKR EEIPVSNVAE LLHQVSMGMK
451 YLEEKNFVHR DLAARNVLLV NRHYAKISDF GLSKALGADD SYYTARSAGK
501 WPLKWYAPEC INFRKFSSRS DVWSYGVTMW EALSYGQKPY KKMKGPEVMA
551 FIEQGKRMEC PPECPPELYA LMSDCWIYKW EDRPDFLTVE QRMACYYSL
601 ASKVEGPPGS TQKAEAACA
```

Residue Number Increasing Mass Decreasing Mass

Start - End	Observed	Mr(expt)	Mr(calc)	Delta	Miss	Sequence
2 - 17	1774.8453	1773.8380	1773.8838	-0.0458	0	PDPAAHLPFFYGSISR (No match)
2 - 17	1774.8453	1773.8380	1773.8838	-0.0458	0	PDPAAHLPFFYGSISR (Ions score 48)
42 - 55	1514.7933	1513.7860	1513.8252	-0.0392	0	SLGGYVLSLVHDVR (Ions score 45)
42 - 55	1514.7933	1513.7860	1513.8252	-0.0392	0	SLGGYVLSLVHDVR (No match)
56 - 63	1082.5217	1081.5144	1081.5457	-0.0313	0	FHHFPIER (No match)
56 - 63	1082.5217	1081.5144	1081.5457	-0.0313	0	FHHFPIER (Ions score 5)
76 - 89	1696.6769	1695.6696	1695.7133	-0.0437	0	AHCGPAELCEFYSR (No match)
76 - 89	1696.6769	1695.6696	1695.7133	-0.0437	0	AHCGPAELCEFYSR (No match)
386 - 400	1842.8696	1841.8623	1841.9094	-0.0470	0	EAQIMHQLDNPYIVR Oxidation (M) (Ions score 5)
386 - 400	1842.8696	1841.8623	1841.9094	-0.0470	0	EAQIMHQLDNPYIVR Oxidation (M) (No match)

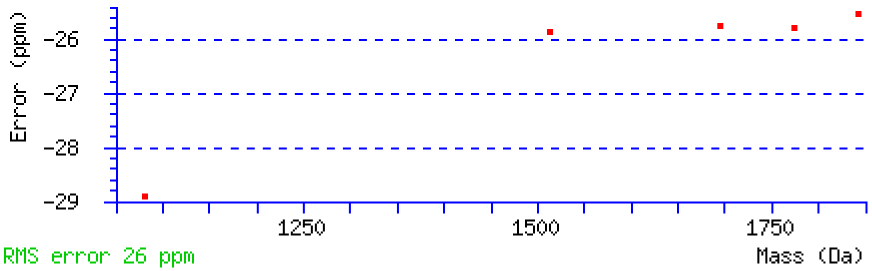

RMS error 26 ppm

Mascot: http://www.matrixscience.com/

Spot 275

{MATRIX SCIENCE} Mascot Search Results

Protein View

Match to: **gi|4504965** Score: **544** Expect: **7.7e-050**
L-plastin [Homo sapiens]

Nominal mass (M_r): **70815;** Calculated pI value: **5.20**
NCBI BLAST search of gi|4504965 against nr
Unformatted sequence string for pasting into other applications

Taxonomy: Homo sapiens
Links to retrieve other entries containing this sequence from NCBI Entrez:
gi|114651523 from Pan troglodytes
gi|114651525 from Pan troglodytes
gi|114651527 from Pan troglodytes
gi|114651529 from Pan troglodytes
gi|114651531 from Pan troglodytes
gi|114651533 from Pan troglodytes
gi|114651535 from Pan troglodytes
gi|114651537 from Pan troglodytes
gi|114651539 from Pan troglodytes
gi|114651541 from Pan troglodytes
gi|114651543 from Pan troglodytes
gi|114651545 from Pan troglodytes
gi|114651547 from Pan troglodytes
gi|114651549 from Pan troglodytes
gi|114651551 from Pan troglodytes
gi|1346733 from Homo sapiens
gi|189502 from Homo sapiens
gi|14043359 from Homo sapiens
gi|16307448 from Homo sapiens
gi|123988880 from synthetic construct
gi|123999207 from synthetic construct

Fixed modifications: Carbamidomethyl (C)
Variable modifications: Oxidation (M)
Cleavage by Trypsin: cuts C-term side of KR unless next residue is P
Sequence Coverage: **50%**

Matched peptides shown in **Bold Red**

1 MARGSVSDEE MMELREAFAK VDTDGNGYIS FNELNDLFKA ACLPLPGYRV
51 REITENLMAT GDLDQDGRIS FDEFIKIFHG LKSTDVAKTF RKAINKKEGI
101 CAIGGTSEQS SVGTQHSYSE EEKYAFVNWI NKALENDPDC RHVIPMNPNT
151 NDLFNAVGDG IVLCKMINLS VPDTIDERTI NKKKLTPFTI QENLNLALNS
201 ASAIGCHVVN IGAEDLKEGK PYLVLGLLWQ VIKIGLFADI ELSRNEALIA
251 LLREGESLED LMKLSPEELL LRWANYHLEN AGCNKIGNFS TDIKDSKAYY
301 HLLEQVAPKG DEEGVPAVVI DMSGLREKDD IQRAECMLQQ AERLGCRQFV
351 TATDVVRGNP KLNLAFIANL FNRYPALHKP ENQDIDWGAL EGETREERTF
401 RNWMNSLGVN PRVNHLYSDL SDALVIFQLY EKIKVPVDWN RVNKPPYPKL
451 GGNMKKLENC NYAVELGKNQ AKFSLVGIGG QDLNEGNRTL TLALIWQLMR
501 RYTLNILEEI GGGQKVNDDI IVNWVNETLR EAEKSSSISS FKDPKISTSL
551 PVLDLIDAIQ PGSINYDLLK TENLNDDEKL NNAKYAISMA RKIGARVYAL
601 PEDLVEVNPK MVMTVFACLM GKGMKRV

Residue Number Increasing Mass Decreasing Mass

Start - End	Observed	Mr(expt)	Mr(calc)	Delta	Miss	Sequence
4 - 15	1382.5858	1381.5785	1381.5853	-0.0068	0	GSVSDEEMMELR (No match)
4 - 15	1398.5765	1397.5692	1397.5802	-0.0110	0	GSVSDEEMMELR Oxidation (M) (No match)
21 - 39	2160.9937	2159.9864	2160.0010	-0.0146	0	VDTDGNGYISFNELNDLFK (No match)
40 - 49	1117.5757	1116.5684	1116.5750	-0.0065	0	AACLPLPGYR (No match)
40 - 49	1117.5757	1116.5684	1116.5750	-0.0065	0	AACLPLPGYR (Ions score 21)
69 - 76	998.5115	997.5042	997.5120	-0.0078	0	ISFDEFIK (No match)
133 - 141	1089.4546	1088.4473	1088.4556	-0.0083	0	ALENDPDCR (No match)
166 - 178	1502.7408	1501.7335	1501.7446	-0.0111	0	MINLSVPDTIDER (No match)
166 - 178	1518.7379	1517.7306	1517.7395	-0.0089	0	MINLSVPDTIDER Oxidation (M) (No match)
234 - 244	1233.6768	1232.6695	1232.6764	-0.0069	0	IGLFADIELSR (No match)
245 - 253	1012.6078	1011.6005	1011.6076	-0.0071	0	NEALIALLR (No match)
264 - 272	1069.6160	1068.6087	1068.6179	-0.0091	0	LSPEELLLR (No match)
298 - 309	1431.7496	1430.7423	1430.7557	-0.0134	0	AYYHLLEQVAPK (No match)
310 - 326	1743.8417	1742.8344	1742.8508	-0.0164	0	GDEEGVPAVVIDMSGLR (No match)
310 - 326	1743.8417	1742.8344	1742.8508	-0.0164	0	GDEEGVPAVVIDMSGLR (Ions score 86)
310 - 326	1759.8400	1758.8327	1758.8457	-0.0130	0	GDEEGVPAVVIDMSGLR Oxidation (M) (No match)
327 - 333	903.4495	902.4422	902.4457	-0.0035	1	EKDDIQR (No match)
334 - 343	1235.5448	1234.5375	1234.5434	-0.0059	0	AECMLQQAER (No match)
334 - 343	1251.5342	1250.5269	1250.5383	-0.0114	0	AECMLQQAER Oxidation (M) (No match)
348 - 357	1135.6036	1134.5963	1134.6033	-0.0069	0	QFVTATDVVR (No match)
362 - 373	1405.7837	1404.7764	1404.7877	-0.0113	0	LNLAFIANLFNR (No match)
362 - 373	1405.7837	1404.7764	1404.7877	-0.0113	0	LNLAFIANLFNR (Ions score 59)
374 - 395	2539.1895	2538.1822	2538.2138	-0.0316	0	YPALHKPENQDIDWGALEGETR (No match)
402 - 412	1287.6140	1286.6067	1286.6189	-0.0122	0	NWMNSLGVNPR (No match)
402 - 412	1303.6075	1302.6002	1302.6138	-0.0136	0	NWMNSLGVNPR Oxidation (M) (No match)
413 - 432	2367.1858	2366.1785	2366.2157	-0.0372	0	VNHLYSDLSDALVIFQLYEK (No match)
433 - 441	1126.6254	1125.6181	1125.6294	-0.0113	1	IKVPVDWNR (No match)
442 - 449	942.5325	941.5252	941.5334	-0.0082	0	VNKPPYPK (No match)

456 - 468	1537.7688	1536.7615	1536.7605	0.0010	1	KLENCNYAVELGK (No match)
473 - 488	1675.8231	1674.8158	1674.8324	-0.0166	0	FSLVGIGGQDLNEGNR (Ions score 91)
473 - 488	1675.8231	1674.8158	1674.8324	-0.0166	0	FSLVGIGGQDLNEGNR (No match)
501 - 515	1690.8960	1689.8887	1689.9049	-0.0162	1	RYTLNILEEIGGGQK (No match)
502 - 515	1534.7966	1533.7893	1533.8038	-0.0145	0	YTLNILEEIGGGQK (No match)
516 - 530	1799.9142	1798.9069	1798.9213	-0.0144	0	VNDDIIVNWVNETLR (No match)
585 - 591	811.4099	810.4026	810.4058	-0.0032	0	YAISMAR (No match)
585 - 591	827.3998	826.3925	826.4007	-0.0082	0	YAISMAR Oxidation (M) (No match)
597 - 610	1585.8331	1584.8258	1584.8398	-0.0140	0	VYALPEDLVEVNPK (No match)

Mascot: http://www.matrixscience.com/

Spot 276

Mascot Search Results

Protein View

Match to: **gi|31417921** Score: **376** Expect: **4.8e-033**
TKT protein [Homo sapiens]

Nominal mass (M_r): **50335;** Calculated pI value: **8.02**
NCBI BLAST search of gi|31417921 against nr
Unformatted sequence string for pasting into other applications

Taxonomy: Homo sapiens

Fixed modifications: Carbamidomethyl (C)
Variable modifications: Oxidation (M)
Cleavage by Trypsin: cuts C-term side of KR unless next residue is P
Sequence Coverage: **38%**

Matched peptides shown in **Bold Red**

```
  1 MAFASIYKLD NLVAILDINR LGQSDPAPLQ HQMDIYQKRC EAFGWHAIIV
 51 DGHSVEELCK AFGQAKHQPT AIIAKTFKGR GITGVEDKES WHGKPLPKNM
101 AEQIIQEIYS QIQSKKKILA TPPQEDAPSV DIANIRMPSL PSYKVGDKIA
151 TRKAYGQALA KLGHASDRII ALDGDTKNST FSEIFKKEHP DRFIECYIAE
201 QNMVSIAVGC ATRNRTVPFC STFAAFFTRA FDQIRMAAIS ESNINLCGSH
251 CGVSIGEDGP SQMALEDLAM FRSVPTSTVF YPSDGVATEK AVELAANTKG
301 ICFIRTSRPE NAIIYNNNED FQVGQAKVVL KSKDDQVTVI GAGVTLHEAL
351 AAAELLKKEK INIRVLDPFT IKPLDRKLIL DSARATKGRI LTVEDHYYEG
401 GIGEAVSSAV VGEPGITVTH LAVNRVPRSG KPAELLKMFG IDRDAIAQAV
451 RGLITKA
```

Residue Number Increasing Mass Decreasing Mass

Start - End	Observed	Mr(expt)	Mr(calc)	Delta	Miss	Sequence
9 - 20	1368.7648	1367.7575	1367.7772	-0.0197	0	LDNLVAILDINR (Ions score 57)
9 - 20	1368.7648	1367.7575	1367.7772	-0.0197	0	LDNLVAILDINR (No match)
21 - 38	2068.9819	2067.9746	2068.0047	-0.0301	0	LGQSDPAPLQHQMDIYQK (No match)
21 - 38	2084.9827	2083.9754	2083.9996	-0.0242	0	LGQSDPAPLQHQMDIYQK Oxidation (M) (No match)
99 - 115	2038.9875	2037.9802	2038.0040	-0.0238	0	NMAEQIIQEIYSQIQSK Oxidation (M) (No match)

118 - 136	2020.0342	2019.0269	2019.0636	-0.0367	0	ILATPPQEDAPSVDIANIR (No match)
118 - 136	2020.0342	2019.0269	2019.0636	-0.0367	0	ILATPPQEDAPSVDIANIR (Ions score 69)
178 - 187	1200.6066	1199.5993	1199.6186	-0.0193	1	NSTFSEIFKK (No match)
193 - 213	2448.0957	2447.0884	2447.1283	-0.0398	0	FIECYIAEQNMVSIAVGCATR Oxidation (M) (No match)
216 - 229	1651.7672	1650.7599	1650.7864	-0.0265	0	TVPFCSTFAAFFTR (Ions score 71)
216 - 229	1651.7672	1650.7599	1650.7864	-0.0265	0	TVPFCSTFAAFFTR (No match)
273 - 290	1884.8965	1883.8892	1883.9152	-0.0260	0	SVPTSTVFYPSDGVATEK (No match)
306 - 327	2508.1628	2507.1555	2507.2040	-0.0485	0	TSRPENAIIYNNNEDFQVGQAK (Ions score 17)
306 - 327	2508.1628	2507.1555	2507.2040	-0.0485	0	TSRPENAIIYNNNEDFQVGQAK (No match)
365 - 376	1413.7905	1412.7832	1412.8027	-0.0195	0	VLDPFTIKPLDR (No match)
365 - 376	1413.7905	1412.7832	1412.8027	-0.0195	0	VLDPFTIKPLDR (Ions score 31)
365 - 377	1541.8800	1540.8727	1540.8977	-0.0249	1	VLDPFTIKPLDRK (No match)
438 - 451	1562.7887	1561.7814	1561.8034	-0.0220	1	MFGIDRDAIAQAVR (No match)
438 - 451	1578.7792	1577.7719	1577.7983	-0.0264	1	MFGIDRDAIAQAVR Oxidation (M) (No match)

Mascot: http://www.matrixscience.com/

Spot 277

{MATRIX}{SCIENCE} Mascot Search Results

Protein View

Match to: **gi|149242795** Score: **363** Expect: **9.6e-032**
Chain A, Autoinhibited Intact Human Zap-70

Nominal mass (M_r): **70122**; Calculated pI value: **7.80**
NCBI BLAST search of gi|149242795 against nr
Unformatted sequence string for pasting into other applications

Taxonomy: Homo sapiens

Fixed modifications: Carbamidomethyl (C)
Variable modifications: Oxidation (M)
Cleavage by Trypsin: cuts C-term side of KR unless next residue is P
Sequence Coverage: **46%**

Matched peptides shown in **Bold Red**

```
  1 MPDPAAHLPF FYGSISRAEA EEHLKLAGMA DGLFLLRQCL RSLGGYVLSL
 51 VHDVRFHHFP IERQLNGTYA IAGGKAHCGP AELCEFYSRD PDGLPCNLRK
101 PCNRPSGLEP QPGVFDCLRD AMVRDYVRQT WKLEGEALEQ AIISQAPQVE
151 KLIATTAHER MPWYHSSLTR EEAERKLYSG AQTDGKFLLR PRKEQGTYAL
201 SLIYGKTVYH YLISQDKAGK YCIPEGTKFD TLWQLVEYLK LKADGLIYCL
251 KEACPNSSAS NASGAAAPTL PAHPSTLTHP QRRIDTLNSD GYTPEPARIT
301 SPDKPRPMPM DTSVFESPFS DPEELKDKKL FLKRDNLLIA DIELGCGNFG
351 SVRQGVYRMR KKQIDVAIKV LKQGTEKADT EEMMREAQIM HQLDNPYIVR
401 LIGVCQAEAL MLVMEMAGGG PLHKFLVGKR EEIPVSNVAE LLHQVSMGMK
451 YLEEKNFVHR NLAARNVLLV NRHYAKISDF GLSKALGADD SYYTARSAGK
501 WPLKWYAPEC INFRKFSSRS DVWSYGVTMW EALSYGQKPY KKMKGPEVMA
551 FIEQGKRMEC PPECPPELYA LMSDCWIYKW EDRPDFLTVE QRMRACYYSL
601 ASKVEGGSAL EVA
```

Residue Number Increasing Mass Decreasing Mass

Start - End	Observed	Mr(expt)	Mr(calc)	Delta	Miss	Sequence	
2 - 17	1774.8734	1773.8661	1773.8838	-0.0177	0	PDPAAHLPFFYGSISR	(Ions score 38)
2 - 17	1774.8734	1773.8661	1773.8838	-0.0177	0	PDPAAHLPFFYGSISR	(No match)

26 - 37	1292.6902	1291.6829	1291.6958	-0.0128	0	**LAGMADGLFLLR** Oxidation (M) (No match)
42 - 55	1514.8225	1513.8152	1513.8252	-0.0100	0	**SLGGYVLSLVHDVR** (No match)
42 - 55	1514.8225	1513.8152	1513.8252	-0.0100	0	**SLGGYVLSLVHDVR** (Ions score 42)
56 - 63	1082.5491	1081.5418	1081.5457	-0.0039	0	**FHHFPIER** (Ions score 26)
56 - 63	1082.5491	1081.5418	1081.5457	-0.0039	0	**FHHFPIER** (No match)
76 - 89	1696.7053	1695.6980	1695.7133	-0.0153	0	**AHCGPAELCEFYSR** (No match)
76 - 89	1696.7053	1695.6980	1695.7133	-0.0153	0	**AHCGPAELCEFYSR** (Ions score 24)
90 - 99	1156.5376	1155.5303	1155.5342	-0.0039	0	**DPDGLPCNLR** (No match)
100 - 119	2327.1121	2326.1048	2326.1310	-0.0262	0	**KPCNRPSGLEPQPGVFDCLR** (No match)
133 - 151	2053.0745	2052.0672	2052.0738	-0.0066	0	**LEGEALEQAIISQAPQVEK** (No match)
152 - 160	1011.5626	1010.5553	1010.5509	0.0045	0	**LIATTAHER** (No match)
187 - 192	801.5065	800.4992	800.5020	-0.0028	0	**FLLRPR** (No match)
193 - 206	1570.8286	1569.8213	1569.8402	-0.0189	1	**KEQGTYALSLIYGK** (No match)
207 - 217	1366.6874	1365.6801	1365.6928	-0.0127	0	**TVYHYLISQDK** (No match)
241 - 251	1293.6975	1292.6902	1292.7162	-0.0260	1	**LKADGLIYCLK** (No match)
252 - 282	3098.4365	3097.4292	3097.4635	-0.0343	0	**EACPNSSASNASGAAAPTLPAHPSTLTHPQR** (No match)
283 - 298	1804.8733	1803.8660	1803.8751	-0.0091	1	**RIDTLNSDGYTPEPAR** (No match)
284 - 298	1648.7723	1647.7650	1647.7740	-0.0090	0	**IDTLNSDGYTPEPAR** (No match)
335 - 353	2063.0061	2061.9988	2062.0152	-0.0164	0	**DNLLIADIELGCGNFGSVR** (No match)
386 - 400	1826.9037	1825.8964	1825.9145	-0.0180	0	**EAQIMHQLDNPYIVR** (No match)
386 - 400	1842.9031	1841.8958	1841.9094	-0.0135	0	**EAQIMHQLDNPYIVR** Oxidation (M) (No match)
386 - 400	1842.9031	1841.8958	1841.9094	-0.0135	0	**EAQIMHQLDNPYIVR** Oxidation (M) (Ions score 14)
466 - 472	827.5104	826.5031	826.5024	0.0007	0	**NVLLVNR** (No match)
485 - 496	1302.5869	1301.5796	1301.5887	-0.0091	0	**ALGADDSYYTAR** (No match)
505 - 514	1355.6130	1354.6057	1354.6128	-0.0071	0	**WYAPECINFR** (No match)
580 - 592	1690.8015	1689.7942	1689.8110	-0.0168	0	**WEDRPDFLTVEQR** (No match)

Mascot: http://www.matrixscience.com/

Spot 278

{MATRIX}{SCIENCE} Mascot Search Results

Protein View

Match to: **gi|5123454** Score: **422** Expect: **1.2e-037**
heat shock 70kDa protein 1A [Homo sapiens]

Nominal mass (M_r): **70280**; Calculated pI value: **5.48**
NCBI BLAST search of gi|5123454 against nr
Unformatted sequence string for pasting into other applications

Taxonomy: Homo sapiens
Links to retrieve other entries containing this sequence from NCBI Entrez:
gi|4529893 from Homo sapiens
gi|55962551 from Homo sapiens

Fixed modifications: Carbamidomethyl (C)
Variable modifications: Oxidation (M)
Cleavage by Trypsin: cuts C-term side of KR unless next residue is P
Sequence Coverage: **39%**

Matched peptides shown in **Bold Red**

```
  1 MAKAAAIGID LGTTYSCVGV FQHGKVEIIA NDQGNRTTPS YVAFTDTERL
 51 IGDAAKNQVA LNPQNTVFDA KRLIGRKFGD PVVQSDMKHW PFQVINDGDK
101 PKVQVSYKGD TKAFYPEEIS SMVLTKMKEI AEAYLGYPVT NAVITVPAYF
151 NDSQRQATKD AGVIAGLNVL RIINEPTAAA IAYGLDRTGK GERNVLIFDL
201 GGGTFDVSIL TIDDGIFEVK ATAGDTHLGG EDFDNRLVNH FVEEFKRKHK
251 KDISQNKRAV RRLRTACERA KRTLSSSTQA SLEIDSLFEG IDFYTSITRA
301 RFEELCSDLF RSTLEPVEKA LRDAKLDKAQ IHDLVLVGGS TRIPKVQKLL
351 QDFFNGRDLN KSINPDEAVA YGAAVQAAIL MGDKSENVQD LLLLDVAPLS
401 LGLETAGGVM TALIKRNSTI PTKQTQIFTT YSDNQPGVLI QVYEGERAMT
451 KDNNLLGRFE LSGIPPAPRG VPQIEVTFDI DANGILNVTA TDKSTGKANK
501 ITITNDKGRL SKEEIERMVQ EAEKYKAEDE VQRERVSAKN ALESYAFNMK
551 SAVEDEGLKG KISEADKKKV LDKCQEVISW LDANTLAEKD EFEHKRKELE
601 QVCNPIISGL YQGAGGPGPG GFGAQGPKGG SGSGPTIEEV D
```

Residue Number Increasing Mass Decreasing Mass

Start - End	Observed	Mr(expt)	Mr(calc)	Delta	Miss	Sequence
4 - 25	2265.1309	2264.1236	2264.1259	-0.0022	0	AAAIGIDLGTTYSCVGVFQHGK (No match)
26 - 36	1228.6298	1227.6225	1227.6207	0.0018	0	VEIIANDQGNR (No match)
37 - 49	1487.7000	1486.6927	1486.6940	-0.0012	0	TTPSYVAFTDTER (No match)
57 - 71	1658.8440	1657.8367	1657.8423	-0.0056	0	NQVALNPQNTVFDAK (No match)
57 - 72	1814.9435	1813.9362	1813.9434	-0.0072	1	NQVALNPQNTVFDAKR (No match)
89 - 102	1680.8265	1679.8192	1679.8419	-0.0227	0	HWPFQVINDGDKPK (No match)
113 - 126	1630.7996	1629.7923	1629.7959	-0.0036	0	AFYPEEISSMVLTK Oxidation (M) (No match)
160 - 171	1197.6884	1196.6811	1196.6876	-0.0065	0	DAGVIAGLNVLR (Ions score 47)
160 - 171	1197.6884	1196.6811	1196.6876	-0.0065	0	DAGVIAGLNVLR (No match)
172 - 187	1687.8945	1686.8872	1686.8940	-0.0068	0	IINEPTAAAIAYGLDR (No match)
172 - 187	1687.8945	1686.8872	1686.8940	-0.0068	0	IINEPTAAAIAYGLDR (Ions score 39)
221 - 236	1675.7700	1674.7627	1674.7233	0.0394	0	ATAGDTHLGGEDFDNR (Ions score 38)
221 - 236	1675.7700	1674.7627	1674.7233	0.0394	0	ATAGDTHLGGEDFDNR (No match)
237 - 246	1261.6531	1260.6458	1260.6502	-0.0044	0	LVNHFVEEFK (No match)
237 - 247	1417.7573	1416.7500	1416.7513	-0.0013	1	LVNHFVEEFKR (Ions score 47)
237 - 247	1417.7573	1416.7500	1416.7513	-0.0013	1	LVNHFVEEFKR (No match)
300 - 311	1542.7335	1541.7262	1541.7296	-0.0034	1	ARFEELCSDLFR (No match)
302 - 311	1315.5996	1314.5923	1314.5914	0.0009	0	FEELCSDLFR (No match)
326 - 342	1822.0101	1821.0028	1821.0108	-0.0079	1	LDKAQIHDLVLVGGSTR (No match)
329 - 342	1465.8101	1464.8028	1464.8048	-0.0020	0	AQIHDLVLVGGSTR (No match)
329 - 345	1803.9213	1802.9140	1803.0366	-0.1226	1	AQIHDLVLVGGSTRIPK (No match)
349 - 357	1109.5742	1108.5669	1108.5665	0.0004	0	LLQDFFNGR (Ions score 25)
349 - 357	1109.5742	1108.5669	1108.5665	0.0004	0	LLQDFFNGR (No match)
424 - 447	2786.3689	2785.3616	2785.3558	0.0058	0	QTQIFTTYSDNQPGVLIQVYEGER (No match)
498 - 507	1117.5851	1116.5778	1116.6138	-0.0360	1	ANKITITNDK (No match)
501 - 509	1017.5992	1016.5919	1016.5614	0.0305	1	ITITNDKGR (No match)
510 - 517	1003.5437	1002.5364	1002.5345	0.0019	1	LSKEEIER (No match)
525 - 533	1137.5591	1136.5518	1136.5461	0.0057	1	YKAEDEVQR (No match)
540 - 550	1303.6008	1302.5935	1302.5914	0.0022	0	NALESYAFNMK Oxidation (M) (No match)

Mascot: http://www.matrixscience.com/

Spot 280

MATRIX SCIENCE Mascot Search Results

Protein View

Match to: **gi|35386** Score: **163** Expect: **9.6e-012**
unnamed protein product [Homo sapiens]

Nominal mass (M_r): **62495;** Calculated pI value: **8.05**
NCBI BLAST search of gi|35386 against nr
Unformatted sequence string for pasting into other applications

Taxonomy: Homo sapiens
Links to retrieve other entries containing this sequence from NCBI Entrez:
gi|226180 from Homo sapiens

Fixed modifications: Carbamidomethyl (C)
Variable modifications: Oxidation (M)
Cleavage by Trypsin: cuts C-term side of KR unless next residue is P
Sequence Coverage: **13%**

Matched peptides shown in **Bold Red**

```
  1 MAARLLLLGI LLLLLPLPVP APCHTAARSE CKRSHKFVPG AWLAGEGVDV
 51 TSLRRSGSFP VDTQRFLRPD GTCTLCENAL QEGTLQRLPL ALTNWRAQGS
101 GCQRHVTRAK VSSTEAVARD AARSIRNDWK VGLDVTPKPT SNVHVSVAGS
151 HSQAANFAAQ KTHQDQYSFS TDTVECRFYS FHVVHTPPLH PDFKRALGDL
201 PHHFNASTQP AYLRLISNYG THFIRAVELG GRISALTALR TCELALEGLT
251 DNEVEDCLTV EAQVNIGIHG SISAEAKACE EKKKKHKMTA SFHQTYRERH
301 SEVVGGHHTS INDLLFGIQA GPEQYSAWVN SVPGSPGLVD YTLEPLHVLL
351 DSQDPRREAL RRALSQYLTD RARWRDCSRP CPPGRQKSPR DPCQCVCHGS
401 AVTTQDCCPR QRGLAQLEVT FIQAWSLWGD WFTATDAYVK LFFGGQELRT
451 STVWDNNNPI WSVRLDFGDV LLATGGPLRL QVWDQDSGRD DDLLGTCDQA
501 PKSGSHEVRC NLNHGHLKFR YHARCLPHLG GGTCLDYVPQ MLLGEPPGNR
551 SGAVW
```

Residue Number Increasing Mass Decreasing Mass

Start - End	Observed	Mr(expt)	Mr(calc)	Delta	Miss	Sequence	
162 - 177	1973.8103	1972.8030	1972.8221	-0.0191	0	THQDQYSFSTDTVECR	(No match)

162 - 177	1973.8103	1972.8030	1972.8221	-0.0191	0	THQDQYSFSTDTVECR	(Ions score 44)
178 - 194	2068.0183	2067.0110	2067.0366	-0.0256	0	FYSFHVVHTPPLHPDFK	(No match)
215 - 225	1320.6992	1319.6919	1319.6986	-0.0066	0	LISNYGTHFIR	(Ions score 7)
215 - 225	1320.6992	1319.6919	1319.6986	-0.0066	0	LISNYGTHFIR	(No match)
363 - 371	1066.5612	1065.5539	1065.5454	0.0085	0	ALSQYLTDR	(No match)
441 - 449	1066.5612	1065.5539	1065.5607	-0.0067	0	LFFGGQELR	(No match)
465 - 479	1543.8326	1542.8253	1542.8405	-0.0152	0	LDFGDVLLATGGPLR	(No match)
465 - 479	1543.8326	1542.8253	1542.8405	-0.0152	0	LDFGDVLLATGGPLR	(Ions score 42)

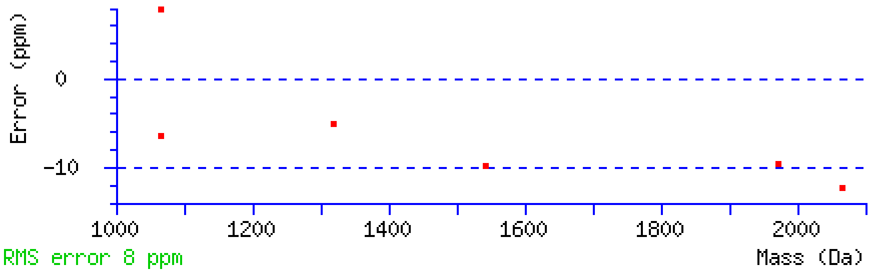

Mascot: http://www.matrixscience.com/

Spot 281

MATRIX SCIENCE Mascot Search Results

Protein View

Match to: **gi|118600975** Score: **198** Expect: **3e-015**
syntaxin binding protein 3 [Homo sapiens]

Nominal mass (M_r): **68633;** Calculated pI value: **7.98**
NCBI BLAST search of gi|118600975 against nr
Unformatted sequence string for pasting into other applications

Taxonomy: Homo sapiens
Links to retrieve other entries containing this sequence from NCBI Entrez:
gi|3820482 from Homo sapiens
gi|28838618 from Homo sapiens
gi|55665678 from Homo sapiens
gi|55960045 from Homo sapiens
gi|119576734 from Homo sapiens
gi|158261291 (no taxonomy information for this entry)

Fixed modifications: Carbamidomethyl (C)
Variable modifications: Oxidation (M)
Cleavage by Trypsin: cuts C-term side of KR unless next residue is P
Sequence Coverage: **17%**

Matched peptides shown in **Bold Red**

```
  1 MAPPVAERGL KSVVWQKIKA TVFDDCKKEG EWKIMLLDEF TTKLLASCCK
 51 MTDLLEEGIT VVENIYKNRE PVRQMKALYF ITPTSKSVDC FLHDFASKSE
101 NKYKAAYIYF TDFCPDNLFN KIKASCSKSI RRCKEINISF IPHESQVYTL
151 DVPDAFYYCY SPDPGNAKGK DAIMETMADQ IVTVCATLDE NPGVRYKSKP
201 LDNASKLAQL VEKKLEDYYK IDEKSLIKGK THSQLLIIDR GFDPVSTVLH
251 ELTFQAMAYD LLPIENDTYK YKTDGKEKEA ILEEEDDLWV RIRHRHIAVV
301 LEEIPKLMKE ISSTKKATEG KTSLSALTQL MKKMPHFRKQ ITKQVVHLNL
351 AEDCMNKFKL NIEKLCKTEQ DLALGTDAEG QKVKDSMRVL LPVLLNKNHD
401 NCDKIRAILL YIFSINGTTE ENLDRLIQNV KIENESDMIR NWSYLGVPIV
451 PQSQQGKPLR KDRSAEETFQ LSRWTPFIKD IMEDAIDNRL DSKEWPYCSQ
501 CPAVWNGSGA VSARQKPRAN YLEDRKNGSK LIVFVIGGIT YSEVRCAYEV
551 SQAHKSCEVI IGSTHVLTPK KLLDDIKMLN KPKDKVSLIK DE
```

Residue Number Increasing Mass Decreasing Mass

Start - End	Observed	Mr(expt)	Mr(calc)	Delta	Miss	Sequence
105 - 121	2099.0161	2098.0088	2097.9505	0.0583	0	AAYIYFTDFCPDNLFNK (No match)
231 - 240	1195.7096	1194.7023	1194.6720	0.0303	0	THSQLLIIDR (Ions score 20)
231 - 240	1195.7096	1194.7023	1194.6720	0.0303	0	THSQLLIIDR (No match)
279 - 291	1616.8257	1615.8184	1615.7729	0.0455	0	EAILEEEDDLWVR (No match)
294 - 306	1540.9307	1539.9234	1539.8885	0.0350	1	HRHIAVVLEEIPK (No match)
389 - 397	1008.6922	1007.6849	1007.6742	0.0107	0	VLLPVLLNK (No match)
407 - 425	2182.1953	2181.1880	2181.1317	0.0564	0	AILLYIFSINGTTEENLDR (No match)
407 - 425	2182.1953	2181.1880	2181.1317	0.0564	0	AILLYIFSINGTTEENLDR (Ions score 38)
464 - 473	1167.5933	1166.5860	1166.5567	0.0293	0	SAEETFQLSR (No match)
464 - 473	1167.5933	1166.5860	1166.5567	0.0293	0	SAEETFQLSR (No match)
531 - 545	1666.0012	1664.9939	1664.9500	0.0439	0	LIVFVIGGITYSEVR (No match)
531 - 545	1666.0012	1664.9939	1664.9500	0.0439	0	LIVFVIGGITYSEVR (Ions score 67)

Mascot: http://www.matrixscience.com/

Spot 284

{MATRIX SCIENCE} Mascot Search Results

Protein View

Match to: **gi|4507909** Score: **148** Expect: **3e-010**
Wiskott-Aldrich syndrome protein [Homo sapiens]

Nominal mass (M_r): **53108**; Calculated pI value: **6.18**
NCBI BLAST search of gi|4507909 against nr
Unformatted sequence string for pasting into other applications

Taxonomy: Homo sapiens
Links to retrieve other entries containing this sequence from NCBI Entrez:
gi|1722836 from Homo sapiens
gi|695151 from Homo sapiens
gi|854673 from Homo sapiens
gi|15215303 from Homo sapiens
gi|119571143 from Homo sapiens
gi|119571144 from Homo sapiens
gi|157929230 (no taxonomy information for this entry)
gi|1096899 from Homo sapiens

Fixed modifications: Carbamidomethyl (C)
Variable modifications: Oxidation (M)
Cleavage by Trypsin: cuts C-term side of KR unless next residue is P
Sequence Coverage: **7%**

Matched peptides shown in **Bold Red**

1 MSGGPMGGRP GGRGAPAVQQ NIPSTLLQDH ENQRLFEMLG RKCLTLATAV
51 VQLYLALPPG AEHWTKEHCG AVCFVKDNPQ KSYFIRLYGL QAGRLLWEQE
101 LYSQLVYSTP TPFFHTFAGD DCQAGLNFAD EDEAQAFRAL VQEKIQKRNQ
151 RQSGDRRQLP PPPTPANEER RGGLPPLPLH PGGDQGGPPV GPLSLGLATV
201 DIQNPDITSS RYRGLPAPGP SPADKKRSGK KKISKADIGA PSGFKHVSHV
251 GWDPQNGFDV NNLDPDLRSL FSRAGISEAQ LTDAETSKLI YDFIEDQGGL
301 EAVRQEMRR**Q** **EPLPPPPPPS** **RGGNQLPRPP** **IVGGNK**GRSG PLPPVPLGIA
351 PPPPTPRGPP PPGR**GGPPPP** **PPPATGR**SGP LPPPPPGAGG PPMPPPPPPP
401 PPPPSSGNGP APPPLPPALV PAGGLAPGGG RGALLDQIRQ GIQLNKTPGA
451 PESSALQPPP QSSEGLVGAL MHVMQKRSRA IHSSDEGEDQ AGDEDEDDEW
501 DD

Residue Number Increasing Mass Decreasing Mass

Start - End	Observed	Mr(expt)	Mr(calc)	Delta	Miss	Sequence
310 - 321	1311.6823	1310.6750	1310.6983	-0.0232	0	QEPLPPPPPPSR (Ions score 37)
310 - 321	1311.6823	1310.6750	1310.6983	-0.0232	0	QEPLPPPPPPSR (No match)
322 - 336	1503.8138	1502.8065	1502.8317	-0.0251	0	GGNQLPRPPIVGGNK (Ions score 32)
322 - 336	1503.8138	1502.8065	1502.8317	-0.0251	0	GGNQLPRPPIVGGNK (No match)
365 - 377	1197.6211	1196.6138	1196.6302	-0.0163	0	GGPPPPPPPATGR (No match)
365 - 377	1197.6211	1196.6138	1196.6302	-0.0163	0	GGPPPPPPPATGR (Ions score 51)

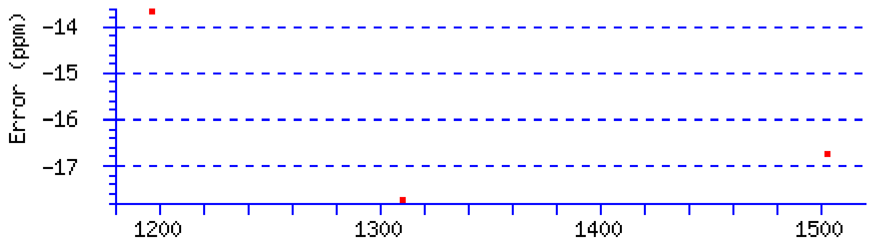

RMS error 16 ppm

Mascot: http://www.matrixscience.com/

Spot 286

MATRIX SCIENCE Mascot Search Results

Protein View

Match to: **gi|62897681** Score: **351** Expect: **1.5e-030**
calreticulin precursor variant [Homo sapiens]

Nominal mass (M_r): **47061**; Calculated pI value: **4.30**
NCBI BLAST search of gi|62897681 against nr
Unformatted sequence string for pasting into other applications

Taxonomy: Homo sapiens

Fixed modifications: Carbamidomethyl (C)
Variable modifications: Oxidation (M)
Cleavage by Trypsin: cuts C-term side of KR unless next residue is P
Sequence Coverage: **36%**

Matched peptides shown in **Bold Red**

```
  1 LGLAVAEPAV YFKEQFLDGD GWTSRWIESK HKSDFGKFVL SSGKFYGDEE
 51 KDKGLQTSQD ARFYALSASF EPFSNKGQTL VVQFTVKHEQ NIDCGGGYVK
101 LFPNSLDQTD MHGDSEYNIM FGPDICGPGT KKVHVIFNYK GKNVLINKDI
151 RCKDDEFTHL YTLIVRPDNT YEVKIDNSQV ESGSLEDDWD FLPPKKIKDP
201 DASKPEDWDE RAKIDDPTDS KPEDWDKPEH IPDPDAKKPE DWDEEMDGEW
251 EPPVIQNPEY KGEWKPRQID NPDYKGTWIH PEIDNPEYSP DPSIYAYDNF
301 GVLGLDLWQV KSGTIFDNFL ITNDEAYAEE FGNETWGVTK AAEKQMKDKQ
351 DEEQRLKEEE EDKKRKEEEE AEDKGDDEDK DEDEEDEEDK EEDEEEDVPG
401 QAKDEL
```

Residue Number Increasing Mass Decreasing Mass

Start - End	Observed	Mr(expt)	Mr(calc)	Delta	Miss	Sequence
14 - 25	1410.6162	1409.6089	1409.6211	-0.0122	0	EQFLDGDGWTSR (No match)
14 - 25	1410.6162	1409.6089	1409.6211	-0.0122	0	EQFLDGDGWTSR (Ions score 45)
45 - 53	1130.4929	1129.4856	1129.4927	-0.0071	1	FYGDEEKDK (No match)
54 - 62	975.4792	974.4719	974.4781	-0.0062	0	GLQTSQDAR (No match)
63 - 76	1607.7618	1606.7545	1606.7667	-0.0122	0	FYALSASFEPFSNK (No match)

88 - 100	1476.6428	1475.6355	1475.6463	-0.0107	0	HEQNIDCGGGYVK (No match)
132 - 140	1147.6289	1146.6216	1146.6549	-0.0333	1	KVHVIFNYK (No match)
133 - 140	1019.5570	1018.5497	1018.5599	-0.0102	0	VHVIFNYK (No match)
152 - 174	2856.3472	2855.3399	2855.3799	-0.0400	1	CKDDEFTHLYTLIVRPDNTYEVK (No match)
152 - 174	2856.3472	2855.3399	2855.3799	-0.0400	1	CKDDEFTHLYTLIVRPDNTYEVK (Ions score 42)
175 - 196	2519.2097	2518.2024	2518.1863	0.0161	1	IDNSQVESGSLEDDWDFLPPKK (No match)
197 - 211	1800.8239	1799.8166	1799.8326	-0.0159	1	IKDPDASKPEDWDER (Ions score 93)
197 - 211	1800.8239	1799.8166	1799.8326	-0.0159	1	IKDPDASKPEDWDER (No match)
262 - 267	772.4116	771.4043	771.4027	0.0016	0	GEWKPR (No match)
268 - 275	992.4636	991.4563	991.4610	-0.0047	0	QIDNPDYK (No match)
348 - 355	1047.4664	1046.4591	1046.4628	-0.0037	1	DKQDEEQR (No match)
348 - 355	1047.4664	1046.4591	1046.4628	-0.0037	1	DKQDEEQR (Ions score 14)
350 - 355	804.3890	803.3817	803.3409	0.0408	0	QDEEQR (No match)

Mascot: http://www.matrixscience.com/

Spot 287

{MATRIX}{SCIENCE} **Mascot Search Results**

Protein View

Match to: **gi|62897681** Score: **458** Expect: **3e-041**
calreticulin precursor variant [Homo sapiens]

Nominal mass (M_r): **47061;** Calculated pI value: **4.30**
NCBI BLAST search of gi|62897681 against nr
Unformatted sequence string for pasting into other applications

Taxonomy: Homo sapiens

Fixed modifications: Carbamidomethyl (C)
Variable modifications: Oxidation (M)
Cleavage by Trypsin: cuts C-term side of KR unless next residue is P
Sequence Coverage: **37%**

Matched peptides shown in **Bold Red**

```
  1 LGLAVAEPAV YFKEQFLDGD GWTSRWIESK HKSDFGKFVL SSGKFYGDEE
 51 KDKGLQTSQD ARFYALSASF EPFSNKGQTL VVQFTVKHEQ NIDCGGGYVK
101 LFPNSLDQTD MHGDSEYNIM FGPDICGPGT KKVHVIFNYK GKNVLINKDI
151 RCKDDEFTHL YTLIVRPDNT YEVKIDNSQV ESGSLEDDWD FLPPKKIKDP
201 DASKPEDWDE RAKIDDPTDS KPEDWDKPEH IPDPDAKKPE DWDEEMDGEW
251 EPPVIQNPEY KGEWKPRQID NPDYKGTWIH PEIDNPEYSP DPSIYAYDNF
301 GVLGLDLWQV KSGTIFDNFL ITNDEAYAEE FGNETWGVTK AAEKQMKDKQ
351 DEEQRLKEEE EDKKRKEEEE AEDKGDDEDK DEDEEDEEDK EEDEEEDVPG
401 QAKDEL
```

Residue Number Increasing Mass Decreasing Mass

Start - End	Observed	Mr(expt)	Mr(calc)	Delta	Miss	Sequence
14 - 25	1410.6205	1409.6132	1409.6211	-0.0079	0	**EQFLDGDGWTSR** (No match)
14 - 25	1410.6205	1409.6132	1409.6211	-0.0079	0	**EQFLDGDGWTSR** (Ions score 59)
45 - 53	1130.4987	1129.4914	1129.4927	-0.0013	1	**FYGDEEKDK** (No match)
54 - 62	975.4823	974.4750	974.4781	-0.0031	0	**GLQTSQDAR** (No match)
63 - 76	1607.7631	1606.7558	1606.7667	-0.0109	0	**FYALSASFEPFSNK** (No match)
63 - 87	2808.4233	2807.4160	2807.4533	-0.0373	1	**FYALSASFEPFSNKGQTLVVQFTVK** (No match)

77 - 87	1219.6936	1218.6863	1218.6972	-0.0108	0	GQTLVVQFTVK (No match)
88 - 100	1476.6469	1475.6396	1475.6463	-0.0066	0	HEQNIDCGGGYVK (No match)
132 - 140	1147.6530	1146.6457	1146.6549	-0.0092	1	KVHVIFNYK (No match)
132 - 140	1147.6530	1146.6457	1146.6549	-0.0092	1	KVHVIFNYK (Ions score 40)
133 - 140	1019.5632	1018.5559	1018.5599	-0.0040	0	VHVIFNYK (Ions score 49)
133 - 140	1019.5632	1018.5559	1018.5599	-0.0040	0	VHVIFNYK (No match)
152 - 174	2856.3574	2855.3501	2855.3799	-0.0298	1	CKDDEFTHLYTLIVRPDNTYEVK (No match)
152 - 174	2856.3574	2855.3501	2855.3799	-0.0298	1	CKDDEFTHLYTLIVRPDNTYEVK (Ions score 31)
175 - 196	2519.2100	2518.2027	2518.1863	0.0164	1	IDNSQVESGSLEDDWDFLPPKK (No match)
197 - 211	1800.8271	1799.8198	1799.8326	-0.0127	1	IKDPDASKPEDWDER (No match)
197 - 211	1800.8271	1799.8198	1799.8326	-0.0127	1	IKDPDASKPEDWDER (Ions score 94)
262 - 267	772.4120	771.4047	771.4027	0.0020	0	GEWKPR (No match)
348 - 355	1047.4713	1046.4640	1046.4628	0.0012	1	DKQDEEQR (No match)
350 - 355	804.3964	803.3891	803.3409	0.0482	0	QDEEQR (No match)

Mascot: http://www.matrixscience.com/

Spot 291

{MATRIX SCIENCE} Mascot Search Results

Protein View

Match to: **gi|119576735** Score: **121** Expect: **1.5e-007**
syntaxin binding protein 3, isoform CRA_b [Homo sapiens]

Nominal mass (M_r): **38947**; Calculated pI value: **8.86**
NCBI BLAST search of gi|119576735 against nr
Unformatted sequence string for pasting into other applications

Taxonomy: Homo sapiens

Fixed modifications: Carbamidomethyl (C)
Variable modifications: Oxidation (M)
Cleavage by Trypsin: cuts C-term side of KR unless next residue is P
Sequence Coverage: **10%**

Matched peptides shown in **Bold Red**

```
  1 MIYYQLRMIH TSKYKTDGKE KEAILEEEDD LWVRIRHRHI AVVLEEIPKL
 51 MKEISSTKKA TEGKTSLSAL TQLMKKMPHF RKQITKQVVH LNLAEDCMNK
101 FKLNIEKLCK TEQDLALGTD AEGQKVKDSM RVLLPVLLNK NHDNCDKIRA
151 ILLYIFSING TTEENLDRLI QNVKIENESD MIRNWSYLGV PIVPQSQQGK
201 PLRKDRSAEE TFQLSRWTPF IKDIMEDAID NRLDSKEWPY CSQCPAVWNG
251 SGAVSARQKP RANYLEDRKN GSKLIVFVIG GITYSEVRCA YEVSQAHKSC
301 EVIIGSTHVL TPKKLLDDIK MLNKPKDKVS LIKDE
```

Residue Number Increasing Mass Decreasing Mass

Start - End	Observed	Mr(expt)	Mr(calc)	Delta	Miss	Sequence	
150 - 168	2182.1042	2181.0969	2181.1317	-0.0347	0	AILLYIFSINGTTEENLDR	(No match)
150 - 168	2182.1042	2181.0969	2181.1317	-0.0347	0	AILLYIFSINGTTEENLDR	(Ions score 20)
274 - 288	1665.9258	1664.9185	1664.9500	-0.0315	0	LIVFVIGGITYSEVR	(No match)
274 - 288	1665.9258	1664.9185	1664.9500	-0.0315	0	LIVFVIGGITYSEVR	(Ions score 76)

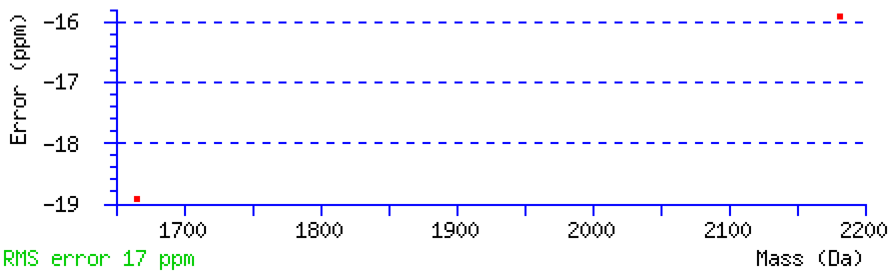

Mascot: http://www.matrixscience.com/

Spot 292

Mascot Search Results

Protein View

Match to: **gi|55958705** Score: **246** Expect: **4.8e-020**
novel protein [Homo sapiens]

Nominal mass (M_r): **36102**; Calculated pI value: **9.13**
NCBI BLAST search of gi|55958705 against nr
Unformatted sequence string for pasting into other applications

Taxonomy: Homo sapiens
Links to retrieve other entries containing this sequence from NCBI Entrez:
gi|55959436 from Homo sapiens
gi|55959495 from Homo sapiens

Fixed modifications: Carbamidomethyl (C)
Variable modifications: Oxidation (M)
Cleavage by Trypsin: cuts C-term side of KR unless next residue is P
Sequence Coverage: **21%**

Matched peptides shown in **Bold Red**

```
  1 MPALSTGSGS DTGLYELLAA LPAQLQPHVD SQEDLTFLWD MFGEKSLHSL
 51 VKHFQERRLA LRRPEILVQP LKVSNRKSSG FRKSFRLSRK DKKTNKSMYE
101 CKKSDQYDTA DVPTYEEVTP YRRQTNEKYR LVVLVGPVGV GLNELKRKLL
151 ISDTQHYGVT VPHTTRARRS QESDGVEYIF ISKHLFETDV QNNKFIEYGE
201 YKNNYYGTSI DSVRSVLAKN KVCLLDVQPH TVKHLRTLEF KPYVIFIKPP
251 SIERLRETRK NAKIISSRDD QGAAKPFTEE DFQEMIKSAQ IMESQYGHLF
301 DKIIINDDLT VA
```

Residue Number Increasing Mass Decreasing Mass

Start - End	Observed	Mr(expt)	Mr(calc)	Delta	Miss	Sequence
59 - 72	1646.0123	1645.0050	1645.0402	-0.0352	1	LALRRPEILVQPLK (No match)
63 - 72	1192.7235	1191.7162	1191.7339	-0.0177	0	RPEILVQPLK (No match)
63 - 72	1192.7235	1191.7162	1191.7339	-0.0177	0	RPEILVQPLK (Ions score 34)
131 - 146	1605.9550	1604.9477	1604.9864	-0.0387	0	LVVLVGPVGVGLNELK (No match)

131 - 146	1605.9550	1604.9477	1604.9864	-0.0387	0	LVVLVGPVGVGLNELK	(Ions score 35)
131 - 147	1762.0552	1761.0479	1761.0875	-0.0396	1	LVVLVGPVGVGLNELKR	(No match)
131 - 147	1762.0552	1761.0479	1761.0875	-0.0396	1	LVVLVGPVGVGLNELKR	(Ions score 42)
149 - 166	2038.0245	2037.0172	2037.0643	-0.0471	0	LLISDTQHYGVTVPHTTR	(Ions score 31)
149 - 166	2038.0245	2037.0172	2037.0643	-0.0471	0	LLISDTQHYGVTVPHTTR	(No match)
237 - 254	2177.1892	2176.1819	2176.2295	-0.0476	0	TLEFKPYVIFIKPPSIER	(No match)
237 - 254	2177.1892	2176.1819	2176.2295	-0.0476	0	TLEFKPYVIFIKPPSIER	(Ions score 17)

Mascot: http://www.matrixscience.com/

Spot 294

{MATRIX}{SCIENCE} **Mascot Search Results**

Protein View

Match to: **gi|62897901** Score: **321** Expect: **1.5e-027**
syntaxin binding protein 3 variant [Homo sapiens]

Nominal mass (M_r): **68599**; Calculated pI value: **7.98**
NCBI BLAST search of gi|62897901 against nr
Unformatted sequence string for pasting into other applications

Taxonomy: Homo sapiens

Fixed modifications: Carbamidomethyl (C)
Variable modifications: Oxidation (M)
Cleavage by Trypsin: cuts C-term side of KR unless next residue is P
Sequence Coverage: **28%**

Matched peptides shown in **Bold Red**

```
  1 MAPPVAERGL KSVVWQKIKA TVFDDCKKEG EWKIMLLDEF TTKLLASCCK
 51 MTDLLEEGIT VVENIYKNRE PVRQMKALYF ITPTSKSVDC FLHDFASKSE
101 NKYKAAYIYF TDFCPDNLFN KIKASCSKSI RRCKEINISF IPHESQVYTL
151 DVPDAFYYCY SPDPGNAKGK DAIMETMADQ IVTVCATLDE NPGVRYKSKP
201 LDNASKLAQL VEKKLEDYYK IDEKSLIKGK THSQLLIIDR GIDPVSTVLH
251 ELTFQAMAYD LLPIENDTYK YKTDGKEKEA ILEEEDDLWV RIRHRHIAVV
301 LEEIPKLMKE ISSTKKATEG KTSLSALTQL MKKMPHFRKQ ITKQVVHLNL
351 AEDCMNKFKL NIEKLCKTEQ DLALGTDAEG QKVKDSMRVL LPVLLNKNHD
401 NCDKIRAILL YIFSINGTTE ENLDRLIQNV KIENESDMIR NWSYLGVPIV
451 PQSQQGKPLR KDRSAEETFQ LSRWTPFIKD IMEDAIDNRL DSKEWPYCSQ
501 CPAVWNGSGA VSARQKPRAN YLEDRKNGSK LIVFVIGGIT YSEVRCAYEV
551 SQAHKSCEVI IGSTHVLTPK KLLDDIKMLN KPKDKVSLIK DE
```

Residue Number Increasing Mass Decreasing Mass

Start - End	Observed	Mr(expt)	Mr(calc)	Delta	Miss	Sequence
34 - 43	1226.6499	1225.6426	1225.6264	0.0162	0	IMLLDEFTTK Oxidation (M) (No match)
77 - 86	1140.6428	1139.6355	1139.6226	0.0129	0	ALYFITPTSK (No match)
87 - 98	1425.6732	1424.6659	1424.6394	0.0265	0	SVDCFLHDFASK (No match)

105 - 121	2099.0051	2097.9978	2097.9505	0.0473	0	**AAYIYFTDFCPDNLFNK** (No match)
229 - 240	1380.8198	1379.8125	1379.7885	0.0241	1	**GKTHSQLLIIDR** (No match)
231 - 240	1195.6971	1194.6898	1194.6720	0.0178	0	**THSQLLIIDR** (No match)
231 - 240	1195.6971	1194.6898	1194.6720	0.0178	0	**THSQLLIIDR** (Ions score 29)
294 - 306	1540.9268	1539.9195	1539.8885	0.0311	1	**HRHIAVVLEEIPK** (No match)
296 - 306	1247.7484	1246.7411	1246.7285	0.0127	0	**HIAVVLEEIPK** (No match)
334 - 339	831.4353	830.4280	830.4221	0.0059	1	**MPHFRK** Oxidation (M) (No match)
389 - 397	1008.6865	1007.6792	1007.6742	0.0050	0	**VLLPVLLNK** (Ions score 24)
389 - 397	1008.6865	1007.6792	1007.6742	0.0050	0	**VLLPVLLNK** (No match)
407 - 425	2182.1746	2181.1673	2181.1317	0.0357	0	**AILLYIFSINGTTEENLDR** (Ions score 36)
407 - 425	2182.1746	2181.1673	2181.1317	0.0357	0	**AILLYIFSINGTTEENLDR** (No match)
432 - 440	1122.5245	1121.5172	1121.5022	0.0150	0	**IENESDMIR** Oxidation (M) (No match)
462 - 473	1438.7183	1437.7110	1437.6848	0.0263	1	**DRSAEETFQLSR** (No match)
464 - 473	1167.5845	1166.5772	1166.5567	0.0205	0	**SAEETFQLSR** (No match)
464 - 473	1167.5845	1166.5772	1166.5567	0.0205	0	**SAEETFQLSR** (No match)
480 - 489	1207.5446	1206.5373	1206.5186	0.0187	0	**DIMEDAIDNR** Oxidation (M) (No match)
531 - 545	1665.9879	1664.9806	1664.9500	0.0306	0	**LIVFVIGGITYSEVR** (No match)
531 - 545	1665.9879	1664.9806	1664.9500	0.0306	0	**LIVFVIGGITYSEVR** (Ions score 64)
556 - 570	1640.8920	1639.8847	1639.8603	0.0244	0	**SCEVIIGSTHVLTPK** (No match)

Mascot: http://www.matrixscience.com/

Spot 297

{MATRIX SCIENCE} Mascot Search Results

Protein View

Match to: **gi|62897087** Score: **257** Expect: **3.8e-021**
WD repeat-containing protein 1 isoform 1 variant [Homo sapiens]

Nominal mass (M_r): **66870;** Calculated pI value: **6.17**
NCBI BLAST search of gi|62897087 against nr
Unformatted sequence string for pasting into other applications

Taxonomy: Homo sapiens

Fixed modifications: Carbamidomethyl (C)
Variable modifications: Oxidation (M)
Cleavage by Trypsin: cuts C-term side of KR unless next residue is P
Sequence Coverage: **35%**

Matched peptides shown in **Bold Red**

```
  1 MPYEIKKVFA SLPQVERGVS KIIGGDPKGN NFLYTNGKCV ILRNIDNPAL
 51 ADIYTEHAHQ VVVAKYAPSG FYIASGDVSG KLRIWDTTQK EHLLKYEYQP
101 FAGKIKDIAW TEDSKRIAVV GEGREKFGAV FLWDSGSSVG EITGHNKVIN
151 SVDIKQSRPY RLATGSDDNC AAFFEGPPFK FKFTVGDHSR FVNCVRFSPD
201 GNRFATASAD GQIYIYDGKT GEKVCALGGS KAHDGGIYAI SWSPDSTHLL
251 SASGDKTSKI WDVSVNSVVS TFPMGSTVLD QQLGCLWQKD HLLSVSLSGY
301 INYLDRNNPS KPLHVIKGHS KSIQCLTVHK NGGKSYIYSG SHDGHINYWD
351 SETGENDSFA GKGHTNQVSR MTVDESGQLI SCSMDDTVRY TSLMLRDYSG
401 QGVVKLDVQP KCVAVGPGGY AVVVCIGQIV LLKDQRKCFS IDNPGYEPEV
451 VAVHPGGDTV AIGGVDGNVR LYSILGTTLK DEGKLLEAKG PVTDVAYSHD
501 GAFLAVCDAS KVVTVFSVAD GYSENNVFYG HHAKIVCLAW SPDNEHFASG
551 GMDMMVYVWT LSDPETRVKI QDAHRLHHVS SLAWLDEHTL FTTSHDASVK
601 EWTITY
```

Residue Number Increasing Mass Decreasing Mass

Start - End	Observed	Mr(expt)	Mr(calc)	Delta	Miss	Sequence	
7 - 17	1273.7719	1272.7646	1272.7189	0.0457	1	KVFASLPQVER	(No match)
8 - 17	1145.6720	1144.6647	1144.6240	0.0407	0	VFASLPQVER	(Ions score 22)

8 - 17	1145.6720	1144.6647	1144.6240	0.0407	0	VFASLPQVER (No match)
29 - 38	1127.5817	1126.5744	1126.5406	0.0338	0	GNNFLYTNGK (No match)
44 - 65	2418.3376	2417.3303	2417.2338	0.0965	0	NIDNPALADIYTEHAHQVVVAK (Ions score 13)
44 - 65	2418.3376	2417.3303	2417.2338	0.0965	0	NIDNPALADIYTEHAHQVVVAK (No match)
66 - 81	1618.8331	1617.8258	1617.7674	0.0584	0	YAPSGFYIASGDVSGK (No match)
96 - 104	1102.5521	1101.5448	1101.5130	0.0318	0	YEYQPFAGK (No match)
117 - 124	800.4818	799.4745	799.4551	0.0194	0	IAVVGEGR (No match)
156 - 161	806.4445	805.4372	805.4194	0.0178	0	QSRPYR (No match)
162 - 180	2043.9921	2042.9848	2042.9043	0.0805	0	LATGSDDNCAAFFEGPPFK (No match)
181 - 190	1193.6484	1192.6411	1192.5988	0.0423	1	FKFTVGDHSR (No match)
183 - 190	918.4752	917.4679	917.4355	0.0324	0	FTVGDHSR (No match)
204 - 219	1719.8876	1718.8803	1718.8151	0.0652	0	FATASADGQIYIYDGK (No match)
290 - 306	1965.0778	1964.0705	1964.0003	0.0702	0	DHLLSVSLSGYINYLDR (Ions score 56)
290 - 306	1965.0778	1964.0705	1964.0003	0.0702	0	DHLLSVSLSGYINYLDR (No match)
363 - 370	898.4823	897.4750	897.4416	0.0334	0	GHTNQVSR (No match)
390 - 396	899.4856	898.4783	898.4582	0.0201	0	YTSLMLR Oxidation (M) (No match)
471 - 484	1537.9043	1536.8970	1536.8399	0.0572	1	LYSILGTTLKDEGK (No match)
490 - 511	2280.1484	2279.1411	2279.0528	0.0884	0	GPVTDVAYSHDGAFLAVCDASK (No match)
512 - 534	2540.3091	2539.3018	2539.2131	0.0888	0	VVTVFSVADGYSENNVFYGHHAK (No match)

Mascot: http://www.matrixscience.com/

Spot 298

Protein View

Match to: **gi|62897087** Score: **495** Expect: **6.1e-045**
WD repeat-containing protein 1 isoform 1 variant [Homo sapiens]

Nominal mass (M_r): **66870**; Calculated pI value: **6.17**
NCBI BLAST search of gi|62897087 against nr
Unformatted sequence string for pasting into other applications

Taxonomy: Homo sapiens

Fixed modifications: Carbamidomethyl (C)
Variable modifications: Oxidation (M)
Cleavage by Trypsin: cuts C-term side of KR unless next residue is P
Sequence Coverage: **39%**

Matched peptides shown in **Bold Red**

```
  1 MPYEIKKVFA SLPQVERGVS KIIGGDPKGN NFLYTNGKCV ILRNIDNPAL
 51 ADIYTEHAHQ VVVAKYAPSG FYIASGDVSG KLRIWDTTQK EHLLKYEYQP
101 FAGKIKDIAW TEDSKRIAVV GEGREKFGAV FLWDSGSSVG EITGHNKVIN
151 SVDIKQSRPY RLATGSDDNC AAFFEGPPFK FKFTVGDHSR FVNCVRFSPD
201 GNRFATASAD GQIYIYDGKT GEKVCALGGS KAHDGGIYAI SWSPDSTHLL
251 SASGDKTSKI WDVSVNSVVS TFPMGSTVLD QQLGCLWQKD HLLSVSLSGY
301 INYLDRNNPS KPLHVIKGHS KSIQCLTVHK NGGKSYIYSG SHDGHINYWD
351 SETGENDSFA GKGHTNQVSR MTVDESGQLI SCSMDDTVRY TSLMLRDYSG
401 QGVVKLDVQP KCVAVGPGGY AVVVCIGQIV LLKDQRKCFS IDNPGYEPEV
451 VAVHPGGDTV AIGGVDGNVR LYSILGTTLK DEGKLLEAKG PVTDVAYSHD
501 GAFLAVCDAS KVVTVFSVAD GYSENNVFYG HHAKIVCLAW SPDNEHFASG
551 GMDMMVYVWT LSDPETRVKI QDAHRLHHVS SLAWLDEHTL FTTSHDASVK
601 EWTITY
```

Residue Number Increasing Mass Decreasing Mass

Start - End	Observed	Mr(expt)	Mr(calc)	Delta	Miss	Sequence
7 - 17	1273.7297	1272.7224	1272.7189	0.0035	1	KVFASLPQVER (No match)
8 - 17	1145.6318	1144.6245	1144.6240	0.0005	0	VFASLPQVER (No match)
8 - 17	1145.6318	1144.6245	1144.6240	0.0005	0	VFASLPQVER (Ions score 25)
29 - 38	1127.5449	1126.5376	1126.5406	-0.0030	0	GNNFLYTNGK (No match)
44 - 65	2418.2434	2417.2361	2417.2338	0.0023	0	NIDNPALADIYTEHAHQVVVAK (Ions score 69)

44 - 65	2418.2434	2417.2361	2417.2338	0.0023	0	**NIDNPALADIYTEHAHQVVVAK** (No match)
66 - 81	1618.7723	1617.7650	1617.7674	-0.0024	0	**YAPSGFYIASGDVSGK** (Ions score 77)
66 - 81	1618.7723	1617.7650	1617.7674	-0.0024	0	**YAPSGFYIASGDVSGK** (No match)
96 - 104	1102.5167	1101.5094	1101.5130	-0.0036	0	**YEYQPFAGK** (No match)
96 - 104	1102.5167	1101.5094	1101.5130	-0.0036	0	**YEYQPFAGK** (Ions score 24)
117 - 124	800.4620	799.4547	799.4551	-0.0004	0	**IAVVGEGR** (No match)
156 - 161	806.4282	805.4209	805.4194	0.0015	0	**QSRPYR** (No match)
162 - 180	2043.9131	2042.9058	2042.9043	0.0015	0	**LATGSDDNCAAFFEGPPFK** (Ions score 108)
162 - 180	2043.9131	2042.9058	2042.9043	0.0015	0	**LATGSDDNCAAFFEGPPFK** (No match)
181 - 190	1193.6083	1192.6010	1192.5988	0.0022	1	**FKFTVGDHSR** (No match)
183 - 190	918.4445	917.4372	917.4355	0.0017	0	**FTVGDHSR** (No match)
204 - 219	1719.8259	1718.8186	1718.8151	0.0035	0	**FATASADGQIYIYDGK** (No match)
290 - 306	1965.0054	1963.9981	1964.0003	-0.0022	0	**DHLLSVSLSGYINYLDR** (No match)
363 - 370	898.4521	897.4448	897.4416	0.0032	0	**GHTNQVSR** (No match)
371 - 389	2143.9250	2142.9177	2142.9231	-0.0054	0	**MTVDESGQLISCSMDDTVR** (No match)
371 - 389	2159.9333	2158.9260	2158.9180	0.0080	0	**MTVDESGQLISCSMDDTVR** Oxidation (M) (No match)
390 - 396	899.4608	898.4535	898.4582	-0.0047	0	**YTSLMLR** Oxidation (M) (No match)
471 - 484	1537.8459	1536.8386	1536.8399	-0.0012	1	**LYSILGTTLKDEGK** (No match)
490 - 511	2280.0640	2279.0567	2279.0528	0.0040	0	**GPVTDVAYSHDGAFLAVCDASK** (No match)
512 - 534	2540.2236	2539.2163	2539.2131	0.0033	0	**VVTVFSVADGYSENNVFYGHHAK** (No match)

Spot 299

Protein View

Match to: **gi|62897087** Score: **296** Expect: **4.8e-025**
WD repeat-containing protein 1 isoform 1 variant [Homo sapiens]

Nominal mass (M_r): **66870**; Calculated pI value: **6.17**
NCBI BLAST search of gi|62897087 against nr
Unformatted sequence string for pasting into other applications

Taxonomy: Homo sapiens

Fixed modifications: Carbamidomethyl (C)
Variable modifications: Oxidation (M)
Cleavage by Trypsin: cuts C-term side of KR unless next residue is P
Sequence Coverage: **35%**

Matched peptides shown in **Bold Red**

```
  1 MPYEIKKVFA SLPQVERGVS KIIGGDPKGN NFLYTNGKCV ILRNIDNPAL
 51 ADIYTEHAHQ VVVAKYAPSG FYIASGDVSG KLRIWDTTQK EHLLKYEYQP
101 FAGKIKDIAW TEDSKRIAVV GEGREKFGAV FLWDSGSSVG EITGHNKVIN
151 SVDIKQSRPY RLATGSDDNC AAFFEGPPFK FKFTVGDHSR FVNCVRFSPD
201 GNRFATASAD GQIYIYDGKT GEKVCALGGS KAHDGGIYAI SWSPDSTHLL
251 SASGDKTSKI WDVSVNSVVS TFPMGSTVLD QQLGCLWQKD HLLSVSLSGY
301 INYLDRNNPS KPLHVIKGHS KSIQCLTVHK NGGKSYIYSG SHDGHINYWD
351 SETGENDSFA GKGHTNQVSR MTVDESGQLI SCSMDDTVRY TSLMLRDYSG
401 QGVVKLDVQP KCVAVGPGGY AVVVCIGQIV LLKDQRKCFS IDNPGYEPEV
451 VAVHPGGDTV AIGGVDGNVR LYSILGTTLK DEGKLLEAKG PVTDVAYSHD
501 GAFLAVCDAS KVVTVFSVAD GYSENNVFYG HHAKIVCLAW SPDNEHFASG
551 GMDMMVYVWT LSDPETRVKI QDAHRLHHVS SLAWLDEHTL FTTSHDASVK
601 EWTITY
```

Residue Number Increasing Mass Decreasing Mass

Start - End	Observed	Mr(expt)	Mr(calc)	Delta	Miss	Sequence	
7 - 17	1273.7892	1272.7819	1272.7189	0.0630	1	KVFASLPQVER	(No match)
7 - 17	1273.7892	1272.7819	1272.7189	0.0630	1	KVFASLPQVER	(No match)
8 - 17	1145.6844	1144.6771	1144.6240	0.0531	0	VFASLPQVER	(Ions score 13)
8 - 17	1145.6844	1144.6771	1144.6240	0.0531	0	VFASLPQVER	(No match)
29 - 38	1127.5942	1126.5869	1126.5406	0.0463	0	GNNFLYTNGK	(No match)

44 - 65	2418.3533	2417.3460	2417.2338	0.1122	0	NIDNPALADIYTEHAHQVVVAK (Ions score 19)
44 - 65	2418.3533	2417.3460	2417.2338	0.1122	0	NIDNPALADIYTEHAHQVVVAK (No match)
66 - 81	1618.8510	1617.8437	1617.7674	0.0763	0	YAPSGFYIASGDVSGK (No match)
66 - 81	1618.8510	1617.8437	1617.7674	0.0763	0	YAPSGFYIASGDVSGK (Ions score 37)
96 - 104	1102.5637	1101.5564	1101.5130	0.0434	0	YEYQPFAGK (Ions score 33)
96 - 104	1102.5637	1101.5564	1101.5130	0.0434	0	YEYQPFAGK (No match)
117 - 124	800.4901	799.4828	799.4551	0.0277	0	IAVVGEGR (No match)
156 - 161	806.4594	805.4521	805.4194	0.0327	0	QSRPYR (No match)
162 - 180	2044.0072	2042.9999	2042.9043	0.0956	0	LATGSDDNCAAFFEGPPFK (No match)
181 - 190	1193.6625	1192.6552	1192.5988	0.0564	1	FKFTVGDHSR (No match)
183 - 190	918.4823	917.4750	917.4355	0.0395	0	FTVGDHSR (No match)
204 - 219	1719.9017	1718.8944	1718.8151	0.0793	0	FATASADGQIYIYDGK (No match)
290 - 306	1965.0991	1964.0918	1964.0003	0.0915	0	DHLLSVSLSGYINYLDR (No match)
363 - 370	898.4885	897.4812	897.4416	0.0396	0	GHTNQVSR (No match)
390 - 396	899.4963	898.4890	898.4582	0.0308	0	YTSLMLR Oxidation (M) (No match)
471 - 484	1537.9188	1536.9115	1536.8399	0.0717	1	LYSILGTTLKDEGK (No match)
490 - 511	2280.1699	2279.1626	2279.0528	0.1099	0	GPVTDVAYSHDGAFLAVCDASK (No match)
512 - 534	2540.3413	2539.3340	2539.2131	0.1210	0	VVTVFSVADGYSENNVFYGHHAK (No match)

Spot 300

Protein View

Match to: **gi|119613095** Score: **176** Expect: **4.8e-013**
WD repeat domain 1, isoform CRA_d [Homo sapiens]

Nominal mass (M_r): **58579;** Calculated pI value: **6.41**
NCBI BLAST search of gi|119613095 against nr
Unformatted sequence string for pasting into other applications

Taxonomy: Homo sapiens

Fixed modifications: Carbamidomethyl (C)
Variable modifications: Oxidation (M)
Cleavage by Trypsin: cuts C-term side of KR unless next residue is P
Sequence Coverage: **21%**

Matched peptides shown in **Bold Red**

```
  1 MPYEIKKVFA SLPQVERGVS KIIGGDPKGN NFLYTNGKCV ILRNIDNPAL
 51 ADIYTEHAHQ VVVAKYAPSG FYIASGDVSG KLRIWDTTQK EHLLKYEYQP
101 FAGKIKDIAW TEDSKRIAVV GEGREKFGAV FLWDSGSSVG EITGHNKVIN
151 SVDIKQSRPY RLATGSDDNC AAFFEGPPFK FKFTVGDHSR FVNCVRFSPD
201 GNRFATASAD GQIYIYDGKT GEKVCALGGS KAHDGGIYAI SWSPDSTHLL
251 SASGDKTSKI WDVSVNSVVS TFPMGSTVLD QQLGCLWQKD HLLSVSLSGY
301 INYLDRNNPS KPLHVIKGHS KSIQCLTVHK NGGKSYIYSG SHDGHINYWD
351 SETGENDSFA GKGHTNQVSR MTVDESGQLI SCSMDDTVRY TSLMLRDYSG
401 QGVVKLDVQP KCVAVGPGGY AVVVCIGQIV LLKDQRKCFS IDNPGYEPEV
451 VAVHPGGDTV AIGGVDGNVR LYSILGTTLK DEGKLLEAKG PVTDVAYSHD
501 GAFLAVCDAS KVVTVFSVAD GYSENNVFYG HHEK
```

Residue Number Increasing Mass Decreasing Mass

Start - End	Observed	Mr(expt)	Mr(calc)	Delta	Miss	Sequence
7 - 17	1273.8113	1272.8040	1272.7189	0.0851	1	KVFASLPQVER (No match)
8 - 17	1145.7041	1144.6968	1144.6240	0.0728	0	VFASLPQVER (No match)
8 - 17	1145.7041	1144.6968	1144.6240	0.0728	0	VFASLPQVER (No match)
44 - 65	2418.3979	2417.3906	2417.2338	0.1568	0	NIDNPALADIYTEHAHQVVVAK (No match)
44 - 65	2418.3979	2417.3906	2417.2338	0.1568	0	NIDNPALADIYTEHAHQVVVAK (Ions score 3)
66 - 81	1618.8810	1617.8737	1617.7674	0.1063	0	YAPSGFYIASGDVSGK (No match)
96 - 104	1102.5845	1101.5772	1101.5130	0.0642	0	YEYQPFAGK (No match)

162 - 180	2044.0532	2043.0459	2042.9043	0.1416	0	LATGSDDNCAAFFEGPPFK (No match)
162 - 180	2044.0532	2043.0459	2042.9043	0.1416	0	LATGSDDNCAAFFEGPPFK (Ions score 63)
204 - 219	1719.9360	1718.9287	1718.8151	0.1136	0	FATASADGQIYIYDGK (No match)
290 - 306	1965.1315	1964.1242	1964.0003	0.1239	0	DHLLSVSLSGYINYLDR (Ions score 16)
290 - 306	1965.1315	1964.1242	1964.0003	0.1239	0	DHLLSVSLSGYINYLDR (No match)
390 - 396	899.5074	898.5001	898.4582	0.0419	0	YTSLMLR Oxidation (M) (No match)

Spot 302

Protein View

Match to: **gi|12804033** Score: **616** Expect: **4.8e-057**
STXBP2 protein [Homo sapiens]

Nominal mass (M_r): **66553**; Calculated pI value: **6.11**
NCBI BLAST search of gi|12804033 against nr
Unformatted sequence string for pasting into other applications

Taxonomy: Homo sapiens
Links to retrieve other entries containing this sequence from NCBI Entrez:
gi|30582669 from Homo sapiens
gi|60655927 from synthetic construct
gi|119589424 from Homo sapiens

Fixed modifications: Carbamidomethyl (C)
Variable modifications: Oxidation (M)
Cleavage by Trypsin: cuts C-term side of KR unless next residue is P
Sequence Coverage: **55%**

Matched peptides shown in **Bold Red**

```
  1 MAPSGLKAVV GEKILSGVIR SVKKDGEWKV LIMDHPSMRI LSSCCKMSDI
 51 LAEGITIVED INKRREPIPS LEAIYLLSPT EKALIKDFQG TPTFTYKAAH
101 IFFTDTCPEP LFSELGRSRL AKVVKTLKEI HLAFLPYEAQ VFSLDAPHST
151 YNLYCPFRAE ERTRQLEVLA QQIATLCATL QEYPAIRYRK GPEDTAQLAH
201 AVLAKLNAFK ADTPSLGEGP EKTRSQLLIM DRAADPVSPL LHELTFQAMA
251 YDLLDIEQDT YRYETTGLSE AREKAVLLDE DDDLWVELRH MHIADVSKKV
301 TELLRTFCES KRLTTDKANI KDLSQILKKM PQYQKELNKY STHLHLADDC
351 MKHFKGSVEK LCSVEQDLAM GSDAEGEKIK DSMKLIVPVL LDAAVPAYDK
401 IRVLLLYILL RNGVSEENLA KLIQHANVQA HSSLIRNLEQ LGGTVTNPGG
451 SGTSSRLEPR ERMEPTYQLS RWTPVIKDVM EDAVEDRLDR NLWPFVSDPA
501 PTASSQAAVS ARFGHWHKNK AGIEARAGPR LIVYVMGGVA MSEMRAAYEV
551 TRATEGKWEV LIGSSHILTP TRFLDDLKAL DKKLEDIALP
```

Residue Number Increasing Mass Decreasing Mass

Start - End	Observed	Mr(expt)	Mr(calc)	Delta	Miss	Sequence
14 - 20	757.4891	756.4818	756.4857	-0.0039	0	ILSGVIR (No match)
30 - 39	1198.5963	1197.5890	1197.5998	-0.0108	0	VLIMDHPSMR (No match)

30 - 39	1214.5911	1213.5838	1213.5947	-0.0109	0	**VLIMDHPSMR** Oxidation (M) (No match)
30 - 39	1230.5834	1229.5761	1229.5896	-0.0135	0	**VLIMDHPSMR** 2 Oxidation (M) (No match)
47 - 64	2017.0355	2016.0282	2016.0561	-0.0279	1	**MSDILAEGITIVEDINKR** (No match)
47 - 64	2033.0468	2032.0395	2032.0510	-0.0115	1	**MSDILAEGITIVEDINKR** Oxidation (M) (No match)
65 - 82	2056.1040	2055.0967	2055.1251	-0.0284	1	**REPIPSLEAIYLLSPTEK** (No match)
65 - 82	2056.1040	2055.0967	2055.1251	-0.0284	1	**REPIPSLEAIYLLSPTEK** (Ions score 43)
66 - 82	1900.0238	1899.0165	1899.0240	-0.0075	0	**EPIPSLEAIYLLSPTEK** (No match)
87 - 97	1304.6019	1303.5946	1303.6084	-0.0138	0	**DFQGTPTFTYK** (No match)
98 - 117	2308.0730	2307.0657	2307.0993	-0.0336	0	**AAHIFFTDTCPEPLFSELGR** (No match)
98 - 117	2308.0730	2307.0657	2307.0993	-0.0336	0	**AAHIFFTDTCPEPLFSELGR** (Ions score 105)
165 - 187	2629.3418	2628.3345	2628.3945	-0.0599	0	**QLEVLAQQIATLCATLQEYPAIR** (No match)
190 - 205	1648.8834	1647.8761	1647.8943	-0.0182	1	**KGPEDTAQLAHAVLAK** (No match)
225 - 232	975.5229	974.5156	974.5219	-0.0063	0	**SQLLIMDR** (No match)
225 - 232	991.5130	990.5057	990.5168	-0.0111	0	**SQLLIMDR** Oxidation (M) (No match)
263 - 272	1126.5320	1125.5247	1125.5302	-0.0054	0	**YETTGLSEAR** (No match)
275 - 289	1800.8766	1799.8693	1799.8941	-0.0248	0	**AVLLDEDDDLWVELR** (No match)
299 - 305	858.5336	857.5263	857.5334	-0.0071	1	**KVTELLR** (No match)
340 - 352	1590.6901	1589.6828	1589.6966	-0.0138	0	**YSTHLHLADDCMK** (No match)
340 - 352	1606.6909	1605.6836	1605.6915	-0.0079	0	**YSTHLHLADDCMK** Oxidation (M) (No match)
385 - 400	1696.9672	1695.9599	1695.9810	-0.0211	0	**LIVPVLLDAAVPAYDK** (No match)
385 - 402	1966.1488	1965.1415	1965.1662	-0.0247	1	**LIVPVLLDAAVPAYDKIR** (No match)
403 - 411	1115.7472	1114.7399	1114.7477	-0.0078	0	**VLLLYILLR** (No match)
422 - 436	1686.9175	1685.9102	1685.9325	-0.0223	0	**LIQHANVQAHSSLIR** (Ions score 46)
422 - 436	1686.9175	1685.9102	1685.9325	-0.0223	0	**LIQHANVQAHSSLIR** (No match)
437 - 456	1931.9128	1930.9055	1930.9344	-0.0288	0	**NLEQLGGTVTNPGGSGTSSR** (Ions score 116)
437 - 456	1931.9128	1930.9055	1930.9344	-0.0288	0	**NLEQLGGTVTNPGGSGTSSR** (No match)
461 - 471	1409.6628	1408.6555	1408.6769	-0.0213	1	**ERMEPTYQLSR** (No match)
461 - 471	1425.6649	1424.6576	1424.6718	-0.0141	1	**ERMEPTYQLSR** Oxidation (M) (No match)
463 - 471	1124.5327	1123.5254	1123.5332	-0.0077	0	**MEPTYQLSR** (No match)
491 - 512	2272.1150	2271.1077	2271.1283	-0.0206	0	**NLWPFVSDPAPTASSQAAVSAR** (No match)
513 - 518	811.3924	810.3851	810.3925	-0.0074	0	**FGHWHK** (No match)
531 - 545	1703.8319	1702.8246	1702.8091	0.0155	0	**LIVYVMGGVAMSEMR** 3 Oxidation (M) (No match)
546 - 552	809.4100	808.4027	808.4079	-0.0051	0	**AAYEVTR** (No match)
553 - 572	2195.1511	2194.1438	2194.1745	-0.0307	1	**ATEGKWEVLIGSSHILTPTR** (No match)
558 - 572	1708.9136	1707.9063	1707.9308	-0.0244	0	**WEVLIGSSHILTPTR** (No match)
573 - 578	750.4013	749.3940	749.3959	-0.0019	0	**FLDDLK** (No match)

Spot 303

Protein View

Match to: **gi|9257257** Score: **150** Expect: **1.9e-010**
WD repeat-containing protein 1 isoform 1 [Homo sapiens]

Nominal mass (M_r): **66836;** Calculated pI value: **6.17**
NCBI BLAST search of gi|9257257 against nr
Unformatted sequence string for pasting into other applications

Taxonomy: Homo sapiens
Links to retrieve other entries containing this sequence from NCBI Entrez:
gi|12643636 from Homo sapiens
gi|3420179 from Homo sapiens
gi|5103673 from Homo sapiens
gi|12803341 from Homo sapiens
gi|158256660 (no taxonomy information for this entry)

Fixed modifications: Carbamidomethyl (C)
Variable modifications: Oxidation (M)
Cleavage by Trypsin: cuts C-term side of KR unless next residue is P
Sequence Coverage: **31%**

Matched peptides shown in **Bold Red**

```
  1 MPYEIKKVFA SLPQVERGVS KIIGGDPKGN NFLYTNGKCV ILRNIDNPAL
 51 ADIYTEHAHQ VVVAKYAPSG FYIASGDVSG KLRIWDTTQK EHLLKYEYQP
101 FAGKIKDIAW TEDSKRIAVV GEGREKFGAV FLWDSGSSVG EITGHNKVIN
151 SVDIKQSRPY RLATGSDDNC AAFFEGPPFK FKFTIGDHSR FVNCVRFSPD
201 GNRFATASAD GQIYIYDGKT GEKVCALGGS KAHDGGIYAI SWSPDSTHLL
251 SASGDKTSKI WDVSVNSVVS TFPMGSTVLD QQLGCLWQKD HLLSVSLSGY
301 INYLDRNNPS KPLHVIKGHS KSIQCLTVHK NGGKSYIYSG SHDGHINYWD
351 SETGENDSFA GKGHTNQVSR MTVDESGQLI SCSMDDTVRY TSLMLRDYSG
401 QGVVKLDVQP KCVAVGPGGY AVVVCIGQIV LLKDQRKCFS IDNPGYEPEV
451 VAVHPGGDTV AIGGVDGNVR LYSILGTTLK DEGKLLEAKG PVTDVAYSHD
501 GAFLAVCDAS KVVTVFSVAD GYSENNVFYG HHAKIVCLAW SPDNEHFASG
551 GMDMMVYVWT LSDPETRVKI QDAHRLHHVS SLAWLDEHTL VTTSHDASVK
601 EWTITY
```

Residue Number Increasing Mass Decreasing Mass

Start - End	Observed	Mr(expt)	Mr(calc)	Delta	Miss	Sequence
7 - 17	1273.7069	1272.6996	1272.7189	-0.0193	1	**KVFASLPQVER** (No match)
8 - 17	1145.6184	1144.6111	1144.6240	-0.0129	0	**VFASLPQVER** (Ions score 10)
8 - 17	1145.6184	1144.6111	1144.6240	-0.0129	0	**VFASLPQVER** (No match)
44 - 65	2418.2097	2417.2024	2417.2338	-0.0314	0	**NIDNPALADIYTEHAHQVVVAK** (No match)
66 - 81	1618.7523	1617.7450	1617.7674	-0.0224	0	**YAPSGFYIASGDVSGK** (No match)
96 - 104	1102.5026	1101.4953	1101.5130	-0.0177	0	**YEYQPFAGK** (No match)
156 - 161	806.4163	805.4090	805.4194	-0.0104	0	**QSRPYR** (No match)
162 - 180	2043.8881	2042.8808	2042.9043	-0.0235	0	**LATGSDDNCAAFFEGPPFK** (No match)
183 - 196	1707.7462	1706.7389	1706.8310	-0.0921	1	**FTIGDHSRFVNCVR** (No match)
191 - 196	794.3926	793.3853	793.3904	-0.0051	0	**FVNCVR** (No match)
197 - 203	792.3564	791.3491	791.3562	-0.0070	0	**FSPDGNR** (No match)
204 - 219	1719.8062	1718.7989	1718.8151	-0.0162	0	**FATASADGQIYIYDGK** (No match)
290 - 306	1964.9646	1963.9573	1964.0003	-0.0430	0	**DHLLSVSLSGYINYLDR** (No match)
390 - 396	899.4525	898.4452	898.4582	-0.0130	0	**YTSLMLR** Oxidation (M) (No match)
490 - 511	2280.0352	2279.0279	2279.0528	-0.0248	0	**GPVTDVAYSHDGAFLAVCDASK** (No match)
512 - 534	2540.1868	2539.1795	2539.2131	-0.0335	0	**VVTVFSVADGYSENNVFYGHHAK** (No match)

Spot 304

Protein View

Match to: **gi|12652891** Score: **657** Expect: **3.8e-061**
WD repeat domain 1 [Homo sapiens]

Nominal mass (M_r): **66822**; Calculated pI value: **6.17**
NCBI BLAST search of gi|12652891 against nr
Unformatted sequence string for pasting into other applications

Taxonomy: Homo sapiens
Links to retrieve other entries containing this sequence from NCBI Entrez:
gi|62897353 from Homo sapiens
gi|119613092 from Homo sapiens

Fixed modifications: Carbamidomethyl (C)
Variable modifications: Oxidation (M)
Cleavage by Trypsin: cuts C-term side of KR unless next residue is P
Sequence Coverage: **59%**

Matched peptides shown in **Bold Red**

```
  1 MPYEIKKVFA SLPQVERGVS KIIGGDPKGN NFLYTNGKCV ILRNIDNPAL
 51 ADIYTEHAHQ VVVAKYAPSG FYIASGDVSG KLRIWDTTQK EHLLKYEYQP
101 FAGKIKDIAW TEDSKRIAVV GEGREKFGAV FLWDSGSSVG EITGHNKVIN
151 SVDIKQSRPY RLATGSDDNC AAFFEGPPFK FKFTVGDHSR FVNCVRFSPD
201 GNRFATASAD GQIYIYDGKT GEKVCALGGS KAHDGGIYAI SWSPDSTHLL
251 SASGDKTSKI WDVSVNSVVS TFPMGSTVLD QQLGCLWQKD HLLSVSLSGY
301 INYLDRNNPS KPLHVIKGHS KSIQCLTVHK NGGKSYIYSG SHDGHINYWD
351 SETGENDSFA GKGHTNQVSR MTVDESGQLI SCSMDDTVRY TSLMLRDYSG
401 QGVVKLDVQP KCVAVGPGGY AVVVCIGQIV LLKDQRKCFS IDNPGYEPEV
451 VAVHPGGDTV AIGGVDGNVR LYSILGTTLK DEGKLLEAKG PVTDVAYSHD
501 GAFLAVCDAS KVVTVFSVAD GYSENNVFYG HHAKIVCLAW SPDNEHFASG
551 GMDMMVYVWT LSDPETRVKI QDAHRLHHVS SLAWLDEHTL VTTSHDASVK
601 EWTITY
```

Residue Number Increasing Mass Decreasing Mass

Start - End	Observed	Mr(expt)	Mr(calc)	Delta	Miss	Sequence	
7 - 17	1273.7061	1272.6988	1272.7189	-0.0201	1	KVFASLPQVER	(Ions score 31)
7 - 17	1273.7061	1272.6988	1272.7189	-0.0201	1	KVFASLPQVER	(No match)

8 - 17	1145.6128	1144.6055	1144.6240	-0.0185	0	**VFASLPQVER** (Ions score 29)
8 - 17	1145.6128	1144.6055	1144.6240	-0.0185	0	**VFASLPQVER** (No match)
29 - 38	1127.5297	1126.5224	1126.5406	-0.0182	0	**GNNFLYTNGK** (No match)
44 - 65	2418.2170	2417.2097	2417.2338	-0.0241	0	**NIDNPALADIYTEHAHQVVVAK** (No match)
44 - 65	2418.2170	2417.2097	2417.2338	-0.0241	0	**NIDNPALADIYTEHAHQVVVAK** (Ions score 123)
66 - 81	1618.7499	1617.7426	1617.7674	-0.0248	0	**YAPSGFYIASGDVSGK** (No match)
96 - 104	1102.4985	1101.4912	1101.5130	-0.0218	0	**YEYQPFAGK** (No match)
96 - 104	1102.4985	1101.4912	1101.5130	-0.0218	0	**YEYQPFAGK** (Ions score 41)
117 - 124	800.4523	799.4450	799.4551	-0.0101	0	**IAVVGEGR** (No match)
156 - 161	806.4138	805.4065	805.4194	-0.0129	0	**QSRPYR** (No match)
162 - 180	2043.8899	2042.8826	2042.9043	-0.0217	0	**LATGSDDNCAAFFEGPPFK** (No match)
181 - 190	1193.5868	1192.5795	1192.5988	-0.0193	1	**FKFTVGDHSR** (No match)
183 - 190	918.4323	917.4250	917.4355	-0.0105	0	**FTVGDHSR** (No match)
191 - 196	794.3875	793.3802	793.3904	-0.0102	0	**FVNCVR** (No match)
197 - 203	792.3552	791.3479	791.3562	-0.0082	0	**FSPDGNR** (No match)
204 - 219	1719.8003	1718.7930	1718.8151	-0.0221	0	**FATASADGQIYIYDGK** (No match)
232 - 256	2585.2241	2584.2168	2584.2193	-0.0025	0	**AHDGGIYAISWSPDSTHLLSASGDK** (No match)
290 - 306	1964.9651	1963.9578	1964.0003	-0.0425	0	**DHLLSVSLSGYINYLDR** (No match)
290 - 306	1964.9651	1963.9578	1964.0003	-0.0425	0	**DHLLSVSLSGYINYLDR** (Ions score 125)
335 - 362	3136.2676	3135.2603	3135.3118	-0.0514	0	**SYIYSGSHDGHINYWDSETGENDSFAGK** (No match)
363 - 370	898.4391	897.4318	897.4416	-0.0098	0	**GHTNQVSR** (No match)
371 - 389	2143.8940	2142.8867	2142.9231	-0.0364	0	**MTVDESGQLISCSMDDTVR** (No match)
371 - 389	2159.9082	2158.9009	2158.9180	-0.0171	0	**MTVDESGQLISCSMDDTVR** Oxidation (M) (No match)
390 - 396	883.4728	882.4655	882.4633	0.0022	0	**YTSLMLR** (No match)
390 - 396	899.4468	898.4395	898.4582	-0.0187	0	**YTSLMLR** Oxidation (M) (No match)
438 - 470	3397.5833	3396.5760	3396.6043	-0.0283	0	**CFSIDNPGYEPEVVAVHPGGDTVAIGGVDGNVR** (No match)
471 - 484	1537.8174	1536.8101	1536.8399	-0.0297	1	**LYSILGTTLKDEGK** (No match)
490 - 511	2280.0393	2279.0320	2279.0528	-0.0207	0	**GPVTDVAYSHDGAFLAVCDASK** (No match)
512 - 534	2540.1831	2539.1758	2539.2131	-0.0372	0	**VVTVFSVADGYSENNVFYGHHAK** (No match)
576 - 600	2783.3750	2782.3677	2782.4038	-0.0361	0	**LHHVSSLAWLDEHTLVTTSHDASVK** (No match)

Spot 305

Protein View

Match to: **gi|62087940** Score: **166** Expect: **4.8e-012**
Syntaxin binding protein 1 variant [Homo sapiens]

Nominal mass (M_r): **47476;** Calculated pI value: **6.11**
NCBI BLAST search of gi|62087940 against nr
Unformatted sequence string for pasting into other applications

Taxonomy: Homo sapiens

Fixed modifications: Carbamidomethyl (C)
Variable modifications: Oxidation (M)
Cleavage by Trypsin: cuts C-term side of KR unless next residue is P
Sequence Coverage: **21%**

Matched peptides shown in **Bold Red**

```
  1 ATLKEYPAVR YRGEYKDNAL LAQLIQDKLD AYKADDPTMG EGPDKARSQL
 51 LILDRGFDPS SPVLHELTFQ AMSYDLLPIE NDVYKYETSG IGEARVKEVL
101 LDEDDDLWIA LRHKHIAEVS QEVTRSLKDF SSSKRMNTGE KTTMRDLSQM
151 LKKMPQYQKE LSKYSTHLHL AEDCMKHYQG TVDKLCRVEQ DLAMGTDAEG
201 EKIKDPMRAI VPILLDANVS TYDKIRIILL YIFLKNGITE ENLNKLIQHA
251 QIPPEDSEII TNMAHLGVPI VTDSTLRRRS KPERKERISE QTYQLSRWTP
301 IIKDIMEDTI EDKLDTKHYP YISTRSSASF STTAVSARYG HWHKNKAPGE
351 YRSGPRLIIF ILGGVSLNEM RCAYEVTQAN GKWEVLIGST HILTPQKLLD
401 TLKKLNKTDE EISS
```

Residue Number Increasing Mass Decreasing Mass

Start - End	Observed	Mr(expt)	Mr(calc)	Delta	Miss	Sequence
48 - 55	957.5718	956.5645	956.5654	-0.0009	0	SQLLILDR (No match)
98 - 112	1814.8950	1813.8877	1813.9097	-0.0220	0	EVLLDEDDDLWIALR (No match)
98 - 112	1814.8950	1813.8877	1813.9097	-0.0220	0	EVLLDEDDDLWIALR (Ions score 60)
209 - 226	2001.1243	2000.1170	2000.1306	-0.0135	1	AIVPILLDANVSTYDKIR (No match)
227 - 235	1135.7393	1134.7320	1134.7416	-0.0096	0	IILLYIFLK (No match)
288 - 297	1224.6196	1223.6123	1223.6146	-0.0023	0	ISEQTYQLSR (No match)
318 - 325	1036.5276	1035.5203	1035.5137	0.0066	0	HYPYISTR (No match)
318 - 325	1036.5276	1035.5203	1035.5137	0.0066	0	HYPYISTR (Ions score 15)
339 - 344	827.4091	826.4018	826.3874	0.0144	0	YGHWHK (No match)

357 - 371	1690.9348	1689.9275	1689.9487	-0.0211	0	LIIFILGGVSLNEMR	Oxidation (M)	(Ions score 6)
357 - 371	1690.9348	1689.9275	1689.9487	-0.0211	0	LIIFILGGVSLNEMR	Oxidation (M)	(No match)

Spot 306

Protein View

Match to: **gi|119583453** Score: **167** Expect: **3.8e-012**
asparaginyl-tRNA synthetase, isoform CRA_b [Homo sapiens]

Nominal mass (M_r): **63687**; Calculated pI value: **5.90**
NCBI BLAST search of gi|119583453 against nr
Unformatted sequence string for pasting into other applications

Taxonomy: Homo sapiens

Fixed modifications: Carbamidomethyl (C)
Variable modifications: Oxidation (M)
Cleavage by Trypsin: cuts C-term side of KR unless next residue is P
Sequence Coverage: **6%**

Matched peptides shown in **Bold Red**

```
  1 MVLELYVSDR EGSDATGDGT KEKPFKTGLK ALMTVGKEPF PTIYVDSQKE
 51 NERWNVISKS QLKNIKKMWH REQMKSESRE KKEAEDSLRR EKNLEEAKKI
101 TIKNDPSLPE PKCVKIGALE GYRGQRVKVF GWVHRLRRQG KNLMFLVLRD
151 GTGYLQCVLA DELCQCYNGV LLSTESSVAV YGMLNLTPKG KQAPGGHELS
201 CDFWELIGLA PAGGADNLIN EESDVDVQLN NRHMMIRGEN MSKILKARSM
251 VTRCFRDHFF DRGYYEVTPP TLVQTQVEGG ATLFKLDYFG EEAFLTQSSQ
301 LYLETCLPAL GDVFCIAQSY RAEQSRTRRH LAEYTHVEAE CPFLTFDDLL
351 NRLEDLVCDV VDRILKSPAG SIVHELNPNF QPPKRPFKRM NYSDAIVWLK
401 EHDVKKEDGT FYEFGEDIPE APERLMTDTI NEPILLCRFP VEIKSFYMQR
451 CPEDSRLTES VDVLMPNVGE IVGGSMRIFD SEEILAGYKR EGIDPTPYYW
501 YTDQRKYGTC PHGGYGLGLE RFLTWILNRY HIRDVCLYPR FVQRCTP
```

Residue Number Increasing Mass Decreasing Mass

Start - End	Observed	Mr(expt)	Mr(calc)	Delta	Miss	Sequence
142 - 149	1021.5506	1020.5433	1020.5790	-0.0356	0	NLMFLVLR Oxidation (M) (No match)
142 - 149	1021.5506	1020.5433	1020.5790	-0.0356	0	NLMFLVLR Oxidation (M) (No match)
478 - 490	1540.7561	1539.7488	1539.7932	-0.0444	1	IFDSEEILAGYKR (No match)
478 - 490	1540.7561	1539.7488	1539.7932	-0.0444	1	IFDSEEILAGYKR (Ions score 69)
507 - 521	1636.7089	1635.7016	1635.7463	-0.0447	0	YGTCPHGGYGLGLER (Ions score 64)
507 - 521	1636.7089	1635.7016	1635.7463	-0.0447	0	YGTCPHGGYGLGLER (No match)

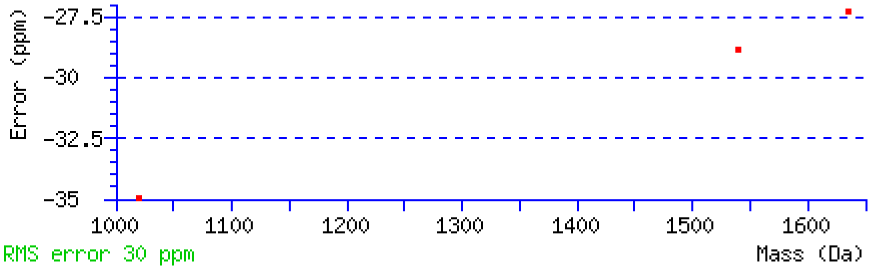

Error (ppm)
-27.5
-30
-32.5
-35
1000
1100
1200
1300
1400
1500
1600
RMS error 30 ppm
Mass (Da)

Spot 307

Protein View

Match to: **gi|671527** Score: **223** Expect: **9.6e-018**
gamma subunit of CCT chaperonin [Homo sapiens]

Nominal mass (M_r): **60862;** Calculated pI value: **6.23**
NCBI BLAST search of gi|671527 against nr
Unformatted sequence string for pasting into other applications

Taxonomy: Homo sapiens

Fixed modifications: Carbamidomethyl (C)
Variable modifications: Oxidation (M)
Cleavage by Trypsin: cuts C-term side of KR unless next residue is P
Sequence Coverage: **20%**

Matched peptides shown in **Bold Red**

```
  1 MGHRPVLVLS QNTKRESGRK VQSGNINAAK TIADIIRTCL GPKSMMKMLL
 51 DPMGGIVMTN DGNAILREIQ VQHPAAKSMI EISRTQDEEV GDGTTSVIIL
101 AGEMLSVAEH FLEQQMHPTV VISAYRKALD DMISTLKKIS IPVDISDSDM
151 MLNIINSSIT TKAISRWSSL ACNIALDAVK MVQFEENGRK EIDIKKYARV
201 EKIPGGIIED SCVLRGVMIN KDVTHPRMRR YIKNPRIVLL DSSLEYKKGG
251 SQTDIEITRE EDFTRILQME EEYIQQLCED IIQLKPDVVI TEKGISDLAQ
301 HYLMRANITA IRRVRKTDNN RIARACGARI VSRPEELRED DVGTGAGLLE
351 IKKIGDEYFT FITDCKDPKA CTILLRGASK EILSEVERNL QDAMQVCRNV
401 LLDPQLVPGG GASEMAVAHA LTEKSKAMTG VEQWPYRAVA QALEVIPRTL
451 IQNCGASTIR LLTSLRAKHT QENCETWGVN GETGTLVDMK ELGIWEPLAV
501 KLQTYKTAVE TAVLLLRIDD IVSGHKKKGD DQSRQGGAPD AGQE
```

Residue Number Increasing Mass Decreasing Mass

Start - End	Observed	Mr(expt)	Mr(calc)	Delta	Miss	Sequence
181 - 190	1237.5853	1236.5780	1236.5920	-0.0140	1	MVQFEENGRK (No match)
200 - 215	1784.9276	1783.9203	1783.9501	-0.0298	1	VEKIPGGIIEDSCVLR (No match)
200 - 215	1784.9276	1783.9203	1783.9501	-0.0298	1	VEKIPGGIIEDSCVLR (Ions score 57)
203 - 215	1428.7332	1427.7259	1427.7442	-0.0183	0	IPGGIIEDSCVLR (No match)
294 - 305	1403.6978	1402.6905	1402.7027	-0.0122	0	GISDLAQHYLMR (No match)
294 - 305	1419.6869	1418.6796	1418.6976	-0.0180	0	GISDLAQHYLMR Oxidation (M) (No match)

294 - 305	1419.6869	1418.6796	1418.6976	-0.0180	0	**GISDLAQHYLMR** Oxidation (M) (No match)
330 - 352	2496.2954	2495.2881	2495.3230	-0.0349	1	**IVSRPEELREDDVGTGAGLLEIK** (No match)
370 - 376	846.4746	845.4673	845.4793	-0.0120	0	**ACTILLR** (No match)
427 - 437	1353.6128	1352.6055	1352.6183	-0.0127	0	**AMTGVEQWPYR** Oxidation (M) (No match)
438 - 448	1166.6987	1165.6914	1165.6818	0.0096	0	**AVAQALEVIPR** (Ions score 32)
438 - 448	1166.6987	1165.6914	1165.6818	0.0096	0	**AVAQALEVIPR** (No match)
449 - 460	1333.6736	1332.6663	1332.6819	-0.0156	0	**TLIQNCGASTIR** (No match)
449 - 460	1333.6736	1332.6663	1332.6819	-0.0156	0	**TLIQNCGASTIR** (No match)
507 - 517	1185.7084	1184.7011	1184.7128	-0.0117	0	**TAVETAVLLLR** (No match)
507 - 517	1185.7084	1184.7011	1184.7128	-0.0117	0	**TAVETAVLLLR** (Ions score 45)

Spot 308

Protein View

Match to: **gi|6272557** Score: **169** Expect: **2.4e-012**
ERO1L [Homo sapiens]

Nominal mass (M_r): **45319**; Calculated pI value: **5.38**
NCBI BLAST search of gi|6272557 against nr
Unformatted sequence string for pasting into other applications

Taxonomy: Homo sapiens

Fixed modifications: Carbamidomethyl (C)
Variable modifications: Oxidation (M)
Cleavage by Trypsin: cuts C-term side of KR unless next residue is P
Sequence Coverage: **33%**

Matched peptides shown in **Bold Red**

```
  1 PCPFWNDISQ CGRRDCAVKP CQSDEVPDGI KSASYKYSEE ANNLIEECEQ
 51 AERLGAVDES LSEETQKAVL QWTKHDDSSD NFCEADDIQS PEAEYVDLLL
101 NPERYTGYKG PDAWKIWNVI YEENCFKPQT IKRPLNPLAS GQGTSEENTF
151 YSWLEGLCVE KRAFYRLISG LHASINVHLS ARYLLQETWL EKKWGHNITE
201 FQQRFDGILT EGEGPRRLKN LYFLYLIELR ALSKVLPFFE RPDFQLFTGN
251 KIQDEENKML LLEILHEIKS FPLHFDENSF FAGDKKEAHK LKEDFRLHFR
301 NISRIMDCVG CFKCRLWGKL QTQGLGTALK ILFSEKLIAN MPESGPSYEF
351 HLTRQEIVSL FNAFGRISTS VKELENFRNL LQNIH
```

Residue Number Increasing Mass Decreasing Mass

Start - End	Observed	Mr(expt)	Mr(calc)	Delta	Miss	Sequence
37 - 53	2083.9983	2082.9910	2082.8799	0.1111	0	YSEEANNLIEECEQAER (No match)
37 - 53	2083.9983	2082.9910	2082.8799	0.1111	0	YSEEANNLIEECEQAER (Ions score 25)
167 - 182	1688.0492	1687.0419	1686.9529	0.0891	0	LISGLHASINVHLSAR (No match)
205 - 216	1290.6985	1289.6912	1289.6251	0.0661	0	FDGILTEGEGPR (No match)
205 - 217	1446.8057	1445.7984	1445.7262	0.0722	1	FDGILTEGEGPRR (No match)
220 - 230	1456.8971	1455.8898	1455.8125	0.0773	0	NLYFLYLIELR (No match)
235 - 251	2055.1721	2054.1648	2054.0625	0.1024	0	VLPFFERPDFQLFTGNK (No match)
235 - 251	2055.1721	2054.1648	2054.0625	0.1024	0	VLPFFERPDFQLFTGNK (No match)
270 - 286	1986.0436	1985.0363	1984.9318	0.1045	1	SFPLHFDENSFFAGDKK (No match)

270 - 286	1986.0436	1985.0363	1984.9318	0.1045	1	**SFPLHFDENSFFAGDKK** (No match)
297 - 304	1042.5721	1041.5648	1041.5831	-0.0183	1	**LHFRNISR** (No match)
337 - 354	2078.1147	2077.1074	2076.9938	0.1136	0	**LIANMPESGPSYEFHLTR** Oxidation (M) (No match)
355 - 366	1380.7985	1379.7912	1379.7197	0.0716	0	**QEIVSLFNAFGR** (Ions score 39)
355 - 366	1380.7985	1379.7912	1379.7197	0.0716	0	**QEIVSLFNAFGR** (No match)

Spot 309

Protein View

Match to: **gi|119617964** Score: **193** Expect: **9.6e-015**
leukotriene A4 hydrolase, isoform CRA_b [Homo sapiens]

Nominal mass (M_r): **63481**; Calculated pI value: **5.71**
NCBI BLAST search of gi|119617964 against nr
Unformatted sequence string for pasting into other applications

Taxonomy: Homo sapiens

Fixed modifications: Carbamidomethyl (C)
Variable modifications: Oxidation (M)
Cleavage by Trypsin: cuts C-term side of KR unless next residue is P
Sequence Coverage: **14%**

Matched peptides shown in **Bold Red**

```
  1 MPEIVDTCSL ASPASVCRTK HLHLRCSVDF TRRTLTGTAA LTVQSQEDNL
 51 RSLVLDTKDL TIEKVVINGQ EVKYALGERQ SYKGSPMEIS LPIALSKNQE
101 IVIEISFETS PKSSALQWLT PEQTSGKEHP YLFSQCQAIH CRAILPCQDT
151 PSVKLTYTAE VSVPKELVAL MSAIRDGETP DPEDPSRKIY KFIQKVPIPC
201 YLIALVVGAL ESRQIGPRTL VWSEKEQVEK SAYEFSETES MLKIAEDLGG
251 PYVWGQYDLL VLPPSFPYGG MENPCLTFVT PTLLAGDKSL SNVIAHEISH
301 SWTGNLVTNK TWDHFWLNEG HTVYLERHIC GRLFGEKFRH FNALGGWGEL
351 QNSVKTFGET HPFTKLVVDL TDIDPDVAYS SVPYEKGFAL LFYLEQLLGG
401 PEIFLGFLKA YVEKFSYKSI TTDDWKDFLY SYFKDKVDVL NQVDWNAWLY
451 SPGLPPIKPN YDMTLTNACI ALSQRWITAK EDDLNSFNAT DLKDLSSHQL
501 NEFLAQTLQR APLPLGHIKR MQEVYNFNAI NNSEIRFRYI ILTCLCGRKQ
551 KTKLL
```

Residue Number Increasing Mass Decreasing Mass

Start - End	Observed	Mr(expt)	Mr(calc)	Delta	Miss	Sequence	
34 - 51	1917.9773	1916.9700	1916.9803	-0.0103	0	TLTGTAALTVQSQEDNLR	(No match)
34 - 51	1917.9773	1916.9700	1916.9803	-0.0103	0	TLTGTAALTVQSQEDNLR	(Ions score 22)
98 - 112	1733.8821	1732.8748	1732.8883	-0.0134	0	NQEIVIEISFETSPK	(No match)
98 - 112	1733.8821	1732.8748	1732.8883	-0.0134	0	NQEIVIEISFETSPK	(Ions score 36)
128 - 142	1945.8674	1944.8601	1944.8723	-0.0122	0	EHPYLFSQCQAIHCR	(No match)

128 - 142	1945.8674	1944.8601	1944.8723	-0.0122	0	EHPYLFSQCQAIHCR	(Ions score 17)
494 - 510	2000.0079	1999.0006	1999.0123	-0.0116	0	DLSSHQLNEFLAQTLQR	(Ions score 31)
494 - 510	2000.0079	1999.0006	1999.0123	-0.0116	0	DLSSHQLNEFLAQTLQR	(No match)
521 - 536	1957.8944	1956.8871	1956.8999	-0.0128	0	MQEVYNFNAINNSEIR	Oxidation (M) (Ions score 18)
521 - 536	1957.8944	1956.8871	1956.8999	-0.0128	0	MQEVYNFNAINNSEIR	Oxidation (M) (No match)

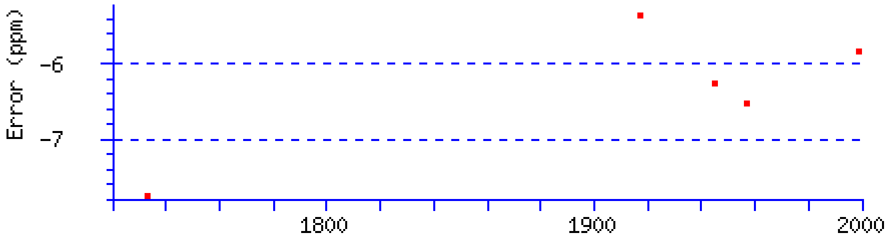

RMS error 6 ppm

Spot 310

Protein View

Match to: **gi|48145697** Score: **232** Expect: **1.2e-018**
CPNE1 [Homo sapiens]

Nominal mass (M_r): **59677**; Calculated pI value: **5.52**
NCBI BLAST search of gi|48145697 against nr
Unformatted sequence string for pasting into other applications

Taxonomy: Homo sapiens

Fixed modifications: Carbamidomethyl (C)
Variable modifications: Oxidation (M)
Cleavage by Trypsin: cuts C-term side of KR unless next residue is P
Sequence Coverage: **19%**

Matched peptides shown in **Bold Red**

```
  1 MAHCVTLVQL SISCDHLIDK DIGSKSDPLC VLLQDVGGGS WAELGRTERV
 51 RNCSSPEFSK TLQLEYRFET VQKLRFGIYD IDNKTPELRD DDFLGGAECS
101 LGQIVSSQVL TLPLMLKPGK PAGRGTITVS AQELKDNRVV TMEVEARNLD
151 KKDFLGKSDP FLEFFRQGDG KWHLVYRSEV IKNNLNPTWK RFSVPVQHFC
201 GGNPSTPIQV QCSDYDSDGS HDLIGTFHTS LAQLQAVPAE FECIHPEKQQ
251 KKKSYKNSGT IRVKICRVET EYSFLDYVMG GCQINFTVGV DFTGSNGDPS
301 SPDSLHYLSP TGVNEYLMAL WSVGSVVQDY DSDKLFPAFG FGAQVPPDWQ
351 VSHEFALNFN PSNPYCVGIQ GIVDAYRQAL PQVRLYGPTN FAPIINHVAR
401 FAAQAAHQGT ASQYFMLLLL TDGAVTDVEA TREAVVRASN LPMSVIIVGV
451 GGADFEAMEQ LDADGGPLHT RSGQAAARDI VQFVPYRRFQ NAPREALAQT
501 VLAEVPTQLV SYFRAQGWAP LKPLPPSAKD PAQAPQA
```

Residue Number Increasing Mass Decreasing Mass

Start - End	Observed	Mr(expt)	Mr(calc)	Delta	Miss	Sequence
61 - 67	922.5195	921.5122	921.4919	0.0203	0	TLQLEYR (No match)
76 - 89	1680.9092	1679.9019	1679.8518	0.0501	1	FGIYDIDNKTPELR (Ions score 47)
76 - 89	1680.9092	1679.9019	1679.8518	0.0501	1	FGIYDIDNKTPELR (No match)
125 - 138	1531.8566	1530.8493	1530.8001	0.0492	1	GTITVSAQELKDNR (No match)
158 - 166	1157.5898	1156.5825	1156.5553	0.0273	0	SDPFLEFFR (Ions score 5)
158 - 166	1157.5898	1156.5825	1156.5553	0.0273	0	SDPFLEFFR (No match)

172 - 177	873.4910	872.4837	872.4657	0.0181	0	**WHLVYR** (No match)
378 - 384	811.4908	810.4835	810.4711	0.0124	0	**QALPQVR** (No match)
385 - 400	1783.0156	1782.0083	1781.9576	0.0507	0	**LYGPTNFAPIINHVAR** (No match)
385 - 400	1783.0156	1782.0083	1781.9576	0.0507	0	**LYGPTNFAPIINHVAR** (Ions score 50)
479 - 487	1136.6394	1135.6321	1135.6025	0.0296	0	**DIVQFVPYR** (Ions score 22)
479 - 487	1136.6394	1135.6321	1135.6025	0.0296	0	**DIVQFVPYR** (No match)
479 - 488	1292.7446	1291.7373	1291.7036	0.0337	1	**DIVQFVPYRR** (No match)
479 - 488	1292.7446	1291.7373	1291.7036	0.0337	1	**DIVQFVPYRR** (Ions score 22)
495 - 514	2235.2561	2234.2488	2234.1946	0.0542	0	**EALAQTVLAEVPTQLVSYFR** (No match)

Spot 311

Protein View

Match to: **gi|119586051** Score: **348** Expect: **3e-030**
ERO1-like (S. cerevisiae), isoform CRA_b [Homo sapiens]

Nominal mass (M_r): **54839**; Calculated pI value: **5.55**
NCBI BLAST search of gi|119586051 against nr
Unformatted sequence string for pasting into other applications

Taxonomy: Homo sapiens

Fixed modifications: Carbamidomethyl (C)
Variable modifications: Oxidation (M)
Cleavage by Trypsin: cuts C-term side of KR unless next residue is P
Sequence Coverage: **36%**

Matched peptides shown in **Bold Red**

```
  1 MGRGWGFLFG LLGAVWLLSS GHGEEQPPET AAQRCFCQVS GYLDDCTCDV
 51 ETIDRFNNYR LFPRLQKLLE SDYFRYYKVN LKRPCPFWND ISQCGRRDCA
101 VKPCQSDEVP DGIKSASYKY SEEANNLIEE CEQAERLGAV DESLSEETQK
151 AVLQWTKHDD SSDNFCEADD IQSPEAEYVD LLLNPERYTG YKGPDAWKIW
201 NVIYEENCFK PQTIKRPLNP LASGQENTFY SWLEGLCVEK RAFYRLISGL
251 HASINVHLSA RYLLQETWLE KKWGHNITEF QQRFDGILTE GEGPRRLKNL
301 YFLYLIELRA LSKVLPFFER PDFQLFTGNK IQDEENKMLL LEIHEIKSF
351 PLHFDENSFF AGDKKEAHKL KEDFRLHFRN ISRIMDCVGC FKCRLWGKLQ
401 TQGLGTALKI LFSEKLIANM PESGPSYEFH LTRQEIVSLF NAFGRISTSV
451 KELENFRNLL QNIH
```

Residue Number Increasing Mass Decreasing Mass

Start - End	Observed	Mr(expt)	Mr(calc)	Delta	Miss	Sequence
68 - 75	1042.5173	1041.5100	1041.5131	-0.0030	0	LLESDYFR (No match)
83 - 96	1792.7802	1791.7729	1791.7933	-0.0204	0	RPCPFWNDISQCGR (No match)
98 - 114	1917.8529	1916.8456	1916.8608	-0.0151	0	DCAVKPCQSDEVPDGIK (No match)
120 - 136	2083.8647	2082.8574	2082.8799	-0.0225	0	YSEEANNLIEECEQAER (Ions score 76)
120 - 136	2083.8647	2082.8574	2082.8799	-0.0225	0	YSEEANNLIEECEQAER (No match)
246 - 261	1687.9479	1686.9406	1686.9529	-0.0122	0	LISGLHASINVHLSAR (No match)
284 - 295	1290.6241	1289.6168	1289.6251	-0.0083	0	FDGILTEGEGPR (Ions score 14)

284 - 295	1290.6241	1289.6168	1289.6251	-0.0083	0	FDGILTEGEGPR (No match)
284 - 296	1446.7241	1445.7168	1445.7262	-0.0094	1	FDGILTEGEGPRR (No match)
297 - 309	1697.9890	1696.9817	1696.9915	-0.0098	1	LKNLYFLYLIELR (No match)
299 - 309	1456.8076	1455.8003	1455.8125	-0.0122	0	NLYFLYLIELR (No match)
314 - 330	2055.0474	2054.0401	2054.0625	-0.0223	0	VLPFFERPDFQLFTGNK (Ions score 9)
314 - 330	2055.0474	2054.0401	2054.0625	-0.0223	0	VLPFFERPDFQLFTGNK (No match)
349 - 364	1857.8317	1856.8244	1856.8369	-0.0125	0	SFPLHFDENSFFAGDK (No match)
349 - 365	1985.9249	1984.9176	1984.9318	-0.0142	1	SFPLHFDENSFFAGDKK (No match)
416 - 433	2061.9902	2060.9829	2060.9989	-0.0160	0	LIANMPESGPSYEFHLTR (No match)
416 - 433	2077.9971	2076.9898	2076.9938	-0.0040	0	LIANMPESGPSYEFHLTR Oxidation (M) (No match)
434 - 445	1380.7170	1379.7097	1379.7197	-0.0099	0	QEIVSLFNAFGR (No match)
434 - 445	1380.7170	1379.7097	1379.7197	-0.0099	0	QEIVSLFNAFGR (Ions score 80)
452 - 457	807.4097	806.4024	806.3922	0.0102	0	ELENFR (No match)

Spot 312

Protein View

Match to: **gi|119586051** Score: **304** Expect: **7.7e-026**
ERO1-like (S. cerevisiae), isoform CRA_b [Homo sapiens]

Nominal mass (M_r): **54839**; Calculated pI value: **5.55**
NCBI BLAST search of gi|119586051 against nr
Unformatted sequence string for pasting into other applications

Taxonomy: Homo sapiens

Fixed modifications: Carbamidomethyl (C)
Variable modifications: Oxidation (M)
Cleavage by Trypsin: cuts C-term side of KR unless next residue is P
Sequence Coverage: **36%**

Matched peptides shown in **Bold Red**

```
  1 MGRGWGFLFG LLGAVWLLSS GHGEEQPPET AAQRCFCQVS GYLDDCTCDV
 51 ETIDRFNNYR LFPRLQKLLE SDYFRYYKVN LKRPCPFWND ISQCGRRDCA
101 VKPCQSDEVP DGIKSASYKY SEEANNLIEE CEQAERLGAV DESLSEETQK
151 AVLQWTKHDD SSDNFCEADD IQSPEAEYVD LLLNPERYTG YKGPDAWKIW
201 NVIYEENCFK PQTIKRPLNP LASGQENTFY SWLEGLCVEK RAFYRLISGL
251 HASINVHLSA RYLLQETWLE KKWGHNITEF QQRFDGILTE GEGPRRLKNL
301 YFLYLIELRA LSKVLPFFER PDFQLFTGNK IQDEENKMLL LEILHEIKSF
351 PLHFDENSFF AGDKKEAHKL KEDFRLHFRN ISRIMDCVGC FKCRLWGKLQ
401 TQGLGTALKI LFSEKLIANM PESGPSYEFH LTRQEIVSLF NAFGRISTSV
451 KELENFRNLL QNIH
```

Residue Number Increasing Mass Decreasing Mass

Start - End	Observed	Mr(expt)	Mr(calc)	Delta	Miss	Sequence
68 - 75	1042.5195	1041.5122	1041.5131	-0.0008	0	LLESDYFR (No match)
83 - 96	1792.7971	1791.7898	1791.7933	-0.0035	0	RPCPFWNDISQCGR (No match)
98 - 114	1917.8578	1916.8505	1916.8608	-0.0102	0	DCAVKPCQSDEVPDGIK (No match)
120 - 136	2083.8804	2082.8731	2082.8799	-0.0068	0	YSEEANNLIEECEQAER (Ions score 60)
120 - 136	2083.8804	2082.8731	2082.8799	-0.0068	0	YSEEANNLIEECEQAER (No match)
246 - 261	1687.9550	1686.9477	1686.9529	-0.0051	0	LISGLHASINVHLSAR (No match)
284 - 295	1290.6299	1289.6226	1289.6251	-0.0025	0	FDGILTEGEGPR (No match)

284 - 295	1290.6299	1289.6226	1289.6251	-0.0025	0	FDGILTEGEGPR	(Ions score 23)
284 - 296	1446.7274	1445.7201	1445.7262	-0.0061	1	FDGILTEGEGPRR	(No match)
297 - 309	1697.9923	1696.9850	1696.9915	-0.0065	1	LKNLYFLYLIELR	(No match)
299 - 309	1456.8160	1455.8087	1455.8125	-0.0038	0	NLYFLYLIELR	(No match)
314 - 330	2055.0579	2054.0506	2054.0625	-0.0118	0	VLPFFERPDFQLFTGNK	(No match)
314 - 330	2055.0579	2054.0506	2054.0625	-0.0118	0	VLPFFERPDFQLFTGNK	(Ions score 8)
349 - 365	1985.9309	1984.9236	1984.9318	-0.0082	1	SFPLHFDENSFFAGDKK	(No match)
416 - 433	2061.9941	2060.9868	2060.9989	-0.0121	0	LIANMPESGPSYEFHLTR	(No match)
416 - 433	2077.9980	2076.9907	2076.9938	-0.0031	0	LIANMPESGPSYEFHLTR Oxidation (M)	(No match)
434 - 445	1380.7240	1379.7167	1379.7197	-0.0029	0	QEIVSLFNAFGR	(No match)
434 - 445	1380.7240	1379.7167	1379.7197	-0.0029	0	QEIVSLFNAFGR	(Ions score 63)
452 - 457	807.4108	806.4035	806.3922	0.0113	0	ELENFR	(No match)

Spot 313

Protein View

Match to: **gi|4503377** Score: **206** Expect: **4.8e-016**
dihydropyrimidinase-like 2 [Homo sapiens]

Nominal mass (M_r): **62711**; Calculated pI value: **5.95**
NCBI BLAST search of gi|4503377 against nr
Unformatted sequence string for pasting into other applications

Taxonomy: Homo sapiens
Links to retrieve other entries containing this sequence from NCBI Entrez:
gi|3122051 from Homo sapiens
gi|75054993 from Pongo pygmaeus
gi|1244400 from Homo sapiens
gi|1330240 from Homo sapiens
gi|2967519 from Homo sapiens
gi|6467213 from Homo sapiens
gi|33991634 from Homo sapiens
gi|45501286 from Homo sapiens
gi|55729384 from Pongo pygmaeus
gi|119583977 from Homo sapiens
gi|119583978 from Homo sapiens
gi|158256010 (no taxonomy information for this entry)

Fixed modifications: Carbamidomethyl (C)
Variable modifications: Oxidation (M)
Cleavage by Trypsin: cuts C-term side of KR unless next residue is P
Sequence Coverage: **13%**

Matched peptides shown in **Bold Red**

1 MSYQGKKNIP RITSDRLLIK GGKIVNDDQS FYADIYMEDG LIKQIGENLI
51 VPGGVKTIEA HSR**MVIPGGI DVHTR**FQMPD QGMTSADDFF QGTKAALAGG
101 TTMIIDHVVP EPGTSLLAAF DQWREWADSK SCCDYSLHVD ISEWHKGIQE
151 EMEALVKDHG VNSFLVYMAF KDRFQLTDCQ IYEVLSVIR**D IGAIAQVHAE**
201 **NGDIIAEEQQ R**ILDLGITGP EGHVLSRPEE VEAEAVNRAI TIANQTNCPL
251 YITKVMSKSS AEVIAQARKK GTVVYGEPIT ASLGTDGSHY WSKNWAKAAA
301 FVTSPPLSPD PTTPDFLNSL LSCGDLQVTG SAHCTFNTAQ KAVGKDNFTL
351 IPEGTNGTEE RMSVIWDKAV VTGKMDENQF VAVTSTNAAK **VFNLYPR**KGR
401 IAVGSDADLV IWDPDSVKTI SAKTHNSSLE YNIFEGMECR GSPLVVISQG
451 K**IVLEDGTLH VTEGSGR**YIP RKPFPDFVYK RIKARSRLAE LRGVPRGLYD
501 GPVCEVSVTP KTVTPASSAK TSPAKQQAPP VR**NLHQSGFS LSGAQIDDNI**
551 **PR**RTTQRIVA PPGGRANITS LG

Residue Number Increasing Mass Decreasing Mass

Start - End	Observed	Mr(expt)	Mr(calc)	Delta	Miss	Sequence
64 - 75	1310.6356	1309.6283	1309.6812	-0.0529	0	MVIPGGIDVHTR Oxidation (M) (No match)
190 - 211	2377.0847	2376.0774	2376.1669	-0.0894	0	DIGAIAQVHAENGDIIAEEQQR (Ions score 53)
190 - 211	2377.0847	2376.0774	2376.1669	-0.0894	0	DIGAIAQVHAENGDIIAEEQQR (No match)
391 - 397	908.4634	907.4561	907.4915	-0.0354	0	VFNLYPR (No match)
452 - 467	1682.8090	1681.8017	1681.8634	-0.0617	0	IVLEDGTLHVTEGSGR (No match)
452 - 467	1682.8090	1681.8017	1681.8634	-0.0617	0	IVLEDGTLHVTEGSGR (Ions score 64)
533 - 552	2168.9883	2167.9810	2168.0610	-0.0799	0	NLHQSGFSLSGAQIDDNIPR (Ions score 37)
533 - 552	2168.9883	2167.9810	2168.0610	-0.0799	0	NLHQSGFSLSGAQIDDNIPR (No match)

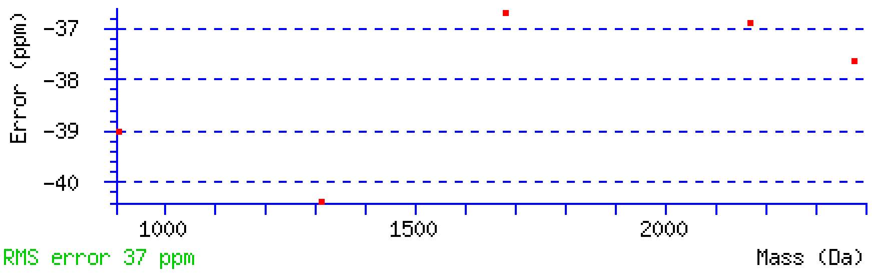

Spot 314

Protein View

Match to: **gi|119612414** Score: **68** Expect: **0.029**
syntrophin, beta 1 (dystrophin-associated protein A1, 59kDa, basic component 1) [Homo sapiens]

Nominal mass (M_r): **58383**; Calculated pI value: **8.81**
NCBI BLAST search of gi|119612414 against nr
Unformatted sequence string for pasting into other applications

Taxonomy: Homo sapiens

Fixed modifications: Carbamidomethyl (C)
Variable modifications: Oxidation (M)
Cleavage by Trypsin: cuts C-term side of KR unless next residue is P
Sequence Coverage: **3%**

Matched peptides shown in **Bold Red**

```
  1 MAVAASAAAA GPAGAGGGRA QRSGLLEVLV RDRWHKVLVN LSEDALVLSS
 51 EEGAAAYNGI GTATNGSFCR GAGAGHPGAG GAQPPDSPAG VRTAFTDLPE
101 QVPESISNQK RGVKVLKQEL GGLGISIKGG KENKMPILIS KIFKGLAADQ
151 TQALYVGDAI LSVNGADLRD ATHDEAVQAL KRAGKEVLLE VKYMREATPY
201 VKKGSPVSEI GWETPPPESP RLGGSTSDPP SSQSFSFHRD RKSIPLKMCY
251 VTRSMALADP ENRQLEIHSP DAKHTVILRS KDSATAQAWF SAIHSNVNDL
301 LTRVIAEVRE QLGKTGIAGS REIRHLGWLA EKVPGESKKQ WKPALVVLTE
351 KDLLIYDSMP RRKEAWFSPV HTYPLLATRL VHSGPGKGSP QAGVDLSFAT
401 RTGTRQGIET HLFRAETSRD LSHWTRSIVQ GCHNSAELIA EISTACTYKN
451 QECRLTIHYE NGFSITTEPQ EGAFPKTIIQ SPYEKLKMSS DDGIRMLYLD
501 FGGKDGEIQL DLHSCPKPIV FIIHSFLSAK ITRLGLVA
```

Residue Number Increasing Mass Decreasing Mass

Start - End	Observed	Mr(expt)	Mr(calc)	Delta	Miss	Sequence	
23 - 31	985.5681	984.5608	984.5967	-0.0359	0	SGLLEVLVR	(No match)
406 - 414	1100.5730	1099.5657	1099.5774	-0.0117	0	QGIETHLFR	(No match)
406 - 414	1100.5730	1099.5657	1099.5774	-0.0117	0	QGIETHLFR	(Ions score 44)

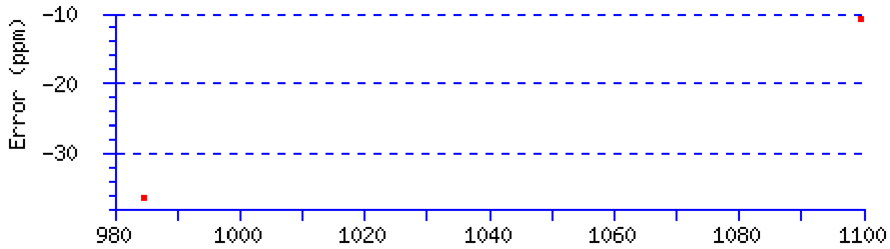

RMS error 22 ppm

Spot 315

Protein View

Match to: **gi|119617726** Score: **112** Expect: **1.2e-006**
nucleosome assembly protein 1-like 1, isoform CRA_d [Homo sapiens]

Nominal mass (M_r): **40154;** Calculated pI value: **4.60**
NCBI BLAST search of gi|119617726 against nr
Unformatted sequence string for pasting into other applications

Taxonomy: Homo sapiens

Fixed modifications: Carbamidomethyl (C)
Variable modifications: Oxidation (M)
Cleavage by Trypsin: cuts C-term side of KR unless next residue is P
Sequence Coverage: **11%**

Matched peptides shown in **Bold Red**

```
  1 MMQNPQILAA LQERLDGLVE TPTGYIESLP RVVKRRVNAL KNLQVKCAQI
 51 EAKFYEEVHD LERKYAVLYQ PLFDKRFEII NAIYEPTEEE CEWKPDEEDE
101 ISEELKEKAK IEDEKKDEEK EDPKGIPEFW LTVFKNVDLL SDMVQEHDEP
151 ILKHLKDIKV KFSDAGQPMS FVLEFHFEPN EYFTNEVLTK TYRMRSEPDD
201 SDPFSFDGPE IMGCTGCQID WKKGKNVTLK TIKKKQKHKG RGTVRTVTKT
251 VSNDSFFNFF APPEVPESGD LDDDAEAILA ADFEIGHFLR ERIIPRSVLY
301 FTGEAIEDDD DDYDEEGEEA DEGYQLFEEV KSCSKLFQRW LQ
```

Residue Number Increasing Mass Decreasing Mass

Start - End	Observed	Mr(expt)	Mr(calc)	Delta	Miss	Sequence
15 - 31	1859.9432	1858.9359	1858.9676	-0.0317	0	LDGLVETPTGYIESLPR (Ions score 56)
15 - 31	1859.9432	1858.9359	1858.9676	-0.0317	0	LDGLVETPTGYIESLPR (No match)
54 - 63	1336.6150	1335.6077	1335.6095	-0.0017	0	FYEEVHDLER (No match)
54 - 63	1336.6150	1335.6077	1335.6095	-0.0017	0	FYEEVHDLER (Ions score 6)
65 - 76	1512.7957	1511.7884	1511.8136	-0.0252	1	YAVLYQPLFDKR (No match)
65 - 76	1512.7957	1511.7884	1511.8136	-0.0252	1	YAVLYQPLFDKR (Ions score 26)

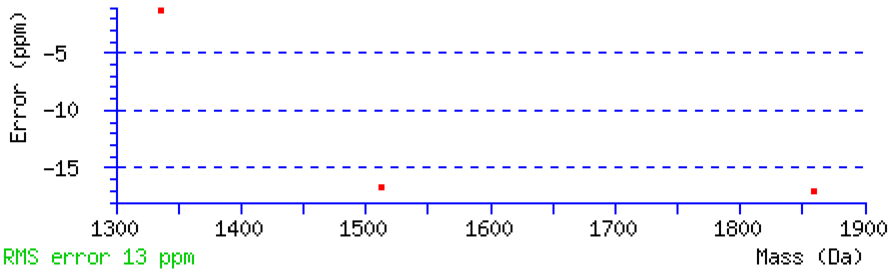

Error (ppm)
-5
-10
-15
1300
1400
1500
1600
1700
1800
1900
RMS error 13 ppm
Mass (Da)

Spot 316

Protein View

Match to: **gi|149243188** Score: **227** Expect: **3.8e-018**
Chain A, Structure Of A Complex Between The A Subunit Of Protein Phosphatase 2a And The Small T Ant

Nominal mass (M_r): **65232**; Calculated pI value: **5.10**
NCBI BLAST search of gi|149243188 against nr
Unformatted sequence string for pasting into other applications

Taxonomy: Homo sapiens
Links to retrieve other entries containing this sequence from NCBI Entrez:
gi|149243189 from Homo sapiens

Fixed modifications: Carbamidomethyl (C)
Variable modifications: Oxidation (M)
Cleavage by Trypsin: cuts C-term side of KR unless next residue is P
Sequence Coverage: **26%**

Matched peptides shown in **Bold Red**

1 LYPIAVLIDE LR**NEDVQLR**L NSIK**KLSTIA LALGVER**TRS ELLPFLTDTI
51 YDEDEVLLAL AEQLGTFTTL VGGPEYVHCL LPPLESLATV EETVVRDKAV
101 ESLR**AISHEH SPSDLEAHFV PLVK**RLAGGD WFTSR**TSACG LFSVCYPR**VS
151 SAVKAELRQY FRNLCSDDTP MVRRAAASKL GEFAKVLELD NVK**SEIIPMF**
201 **SNLASDEQDS VR**LLAVEACV NIAQLLPQED LEALVMPTLR QAAEDKSWRV
251 R**YMVADKFTE LQK**AVGPEIT K**TDLVPAFQN LMK**DCEAEVR AAASHKVKEF
301 CENLSADCRE NVIMSQILPC IKELVSDANQ HVKSALASVI MGLSPILGKD
351 NTIEHLLPLF LAQLKDECPE VR**LNIISNLD CVNEVIGIR**Q LSQSLLPAIV
401 ELAEDAKWRV RLAIIEYMPL LAGQLGVEFF DEKLNSLCMA WLVDHVYAIR
451 EAATSNLKKL VEKFGKEWAH ATIIPK**VLAM SGDPNYLHR**M TTLFCINVLS
501 EVCGQDITTK **HMLPTVLR**MA GDPVANVRFN VAKSLQK**IGP ILDNSTLQSE**
551 **VKPILEK**LTQ DQDVDVKYFA QEALTVLSLA

Residue Number Increasing Mass Decreasing Mass

Start - End	Observed	Mr(expt)	Mr(calc)	Delta	Miss	Sequence
13 - 19	873.4677	872.4604	872.4352	0.0253	0	NEDVQLR (No match)
25 - 37	1370.8948	1369.8875	1369.8292	0.0583	1	KLSTIALALGVER (No match)
26 - 37	1242.7906	1241.7833	1241.7343	0.0491	0	LSTIALALGVER (No match)

26 - 37	1242.7906	1241.7833	1241.7343	0.0491	0	**LSTIALALGVER** (Ions score 28)
105 - 124	2213.2200	2212.2127	2212.1276	0.0851	0	**AISHEHSPSDLEAHFVPLVK** (No match)
105 - 124	2213.2200	2212.2127	2212.1276	0.0851	0	**AISHEHSPSDLEAHFVPLVK** (Ions score 26)
136 - 148	1517.7539	1516.7466	1516.6802	0.0664	0	**TSACGLFSVCYPR** (No match)
194 - 212	2154.0913	2153.0840	2152.9946	0.0894	0	**SEIIPMFSNLASDEQDSVR** Oxidation (M) (Ions score 9)
194 - 212	2154.0913	2153.0840	2152.9946	0.0894	0	**SEIIPMFSNLASDEQDSVR** Oxidation (M) (No match)
252 - 263	1488.7876	1487.7803	1487.7330	0.0474	1	**YMVADKFTELQK** Oxidation (M) (No match)
272 - 283	1392.7731	1391.7658	1391.7118	0.0540	0	**TDLVPAFQNLMK** Oxidation (M) (No match)
373 - 389	1942.1248	1941.1175	1941.0353	0.0823	0	**LNIISNLDCVNEVIGIR** (No match)
477 - 489	1488.7876	1487.7803	1487.7190	0.0613	0	**VLAMSGDPNYLHR** Oxidation (M) (Ions score 8)
511 - 518	982.5881	981.5808	981.5429	0.0379	0	**HMLPTVLR** Oxidation (M) (No match)
538 - 557	2194.3252	2193.3179	2193.2256	0.0924	0	**IGPILDNSTLQSEVKPILEK** (No match)
538 - 557	2194.3252	2193.3179	2193.2256	0.0924	0	**IGPILDNSTLQSEVKPILEK** (Ions score 24)

Spot 317

Protein View

Match to: **gi|62898990** Score: **202** Expect: **1.2e-015**
protein tyrosine phosphatase, non-receptor type 6 isoform 1 variant [Homo sapiens]

Nominal mass (M_r): **67966**; Calculated pI value: **7.65**
NCBI BLAST search of gi|62898990 against nr
Unformatted sequence string for pasting into other applications

Taxonomy: Homo sapiens

Fixed modifications: Carbamidomethyl (C)
Variable modifications: Oxidation (M)
Cleavage by Trypsin: cuts C-term side of KR unless next residue is P
Sequence Coverage: **32%**

Matched peptides shown in **Bold Red**

```
  1 MVRWFHRDLS GLDAETLLKG RGVHGSFLAR PSRKNQGDFS LSVRVGDQVT
 51 HIRIQNSGDF YDLYGGEKFA TLTELVEYYT QQQGVLQDRD GTIIHLKYPL
101 NCSDPTSERW YHGHMSGGQA ETLLQAKGEP WTFLVRESLS QPGDFVLSVL
151 SDQPKAGPGS PLRVTHIKVM CEGGRYTVGG LETFDSLTDL VEHFKKTGIE
201 EASGAFVYLR QPYYATRVNA ADIENRVLEL NKKQESEDTA KAGFWEEFES
251 LQKQEVKNLH QRLEGQRPEN KGKNRYKNIL PFDHSRVILQ GRDSNIPGSD
301 YINANYIKNQ LLGPDENAKT YIASQGCLEA TVNDFWQMAW QENSRVIVMT
351 TREVEKGRNK CVPYWPEVGM QRAYGPYSVT NCGEHDTTEY KLRTLQVSPL
401 DNGDLIREIW HYQYLSWPDH GVPSEPGGIL SFLDQINQRQ ESLPHAGPII
451 VHCSAGIGRT GTIIVIDMLM ENISTKGLDC DIDIQKTIQM VRAQRSGMVQ
501 TEAQYKFIYV AIAQFFETTK KKLEVLQSQK GQESEYGNIT YPPAMKNAHA
551 KASRTSSKHK EDVYENLHTK NKREEKVKKQ RSADKEKSKG SLKRK
```

Residue Number Increasing Mass Decreasing Mass

Start - End	Observed	Mr(expt)	Mr(calc)	Delta	Miss	Sequence
35 - 44	1122.5698	1121.5625	1121.5465	0.0160	0	NQGDFSLSVR (No match)
45 - 53	1024.5590	1023.5517	1023.5461	0.0056	0	VGDQVTHIR (No match)
54 - 68	1705.7909	1704.7836	1704.7630	0.0206	0	IQNSGDFYDLYGGEK (Ions score 8)
54 - 68	1705.7909	1704.7836	1704.7630	0.0206	0	IQNSGDFYDLYGGEK (No match)
69 - 89	2502.2732	2501.2659	2501.2437	0.0222	0	FATLTELVEYYTQQQGVLQDR (No match)

98 - 109	1438.6449	1437.6376	1437.6194	0.0182	0	YPLNCSDPTSER	(No match)
98 - 109	1438.6449	1437.6376	1437.6194	0.0182	0	YPLNCSDPTSER	(No match)
128 - 136	1104.5939	1103.5866	1103.5763	0.0103	0	GEPWTFLVR	(No match)
137 - 155	2046.0679	2045.0606	2045.0316	0.0290	0	ESLSQPGDFVLSVLSDQPK	(No match)
197 - 210	1512.7876	1511.7803	1511.7619	0.0184	0	TGIEEASGAFVYLR	(No match)
197 - 210	1512.7876	1511.7803	1511.7619	0.0184	0	TGIEEASGAFVYLR	(Ions score 25)
211 - 217	898.4436	897.4363	897.4344	0.0019	0	QPYYATR	(No match)
276 - 286	1389.7463	1388.7390	1388.7200	0.0190	1	YKNILPFDHSR	(No match)
278 - 286	1098.5797	1097.5724	1097.5618	0.0107	0	NILPFDHSR	(No match)
278 - 286	1098.5797	1097.5724	1097.5618	0.0107	0	NILPFDHSR	(Ions score 13)
293 - 308	1783.8793	1782.8720	1782.8424	0.0296	0	DSNIPGSDYINANYIK	(No match)
394 - 407	1540.8544	1539.8471	1539.8256	0.0215	0	TLQVSPLDNGDLIR	(No match)
394 - 407	1540.8544	1539.8471	1539.8256	0.0215	0	TLQVSPLDNGDLIR	(Ions score 17)
440 - 459	2099.1082	2098.1009	2098.0741	0.0268	0	QESLPHAGPIIVHCSAGIGR	(No match)
531 - 546	1800.8431	1799.8358	1799.8035	0.0323	0	GQESEYGNITYPPAMK	Oxidation (M) (No match)

Spot 318

Protein View

Match to: **gi|41281398** Score: **105** Expect: **6.1e-006**
soc-2 suppressor of clear homolog [Homo sapiens]

Nominal mass (M_r): **65304;** Calculated pI value: **8.65**
NCBI BLAST search of gi|41281398 against nr
Unformatted sequence string for pasting into other applications

Taxonomy: Homo sapiens
Links to retrieve other entries containing this sequence from NCBI Entrez:
gi|114632804 from Pan troglodytes
gi|149689670 from Equus caballus
gi|14423936 from Homo sapiens
gi|3252979 from Homo sapiens
gi|3293320 from Homo sapiens
gi|29792199 from Homo sapiens
gi|55661683 from Homo sapiens
gi|119569933 from Homo sapiens

Fixed modifications: Carbamidomethyl (C)
Variable modifications: Oxidation (M)
Cleavage by Trypsin: cuts C-term side of KR unless next residue is P
Sequence Coverage: **5%**

Matched peptides shown in **Bold Red**

```
  1 MSSSLGKEKD SKEKDPKVPS AKEREKEAKA SGGFGKESKE KEPKTKGKDA
 51 KDGKKDSSAA QPGVAFSVDN TIKRPNPAPG TRKKSSNAEV IKELNKCREE
101 NSMRLDLSKR SIHILPSSIK ELTQLTELYL YSNKLQSLPA EVGCLVNLMT
151 LALSENSLTS LPDSLDNLKK LRMLDLRHNK LREIPSVVYR LDSLTTLYLR
201 FNRITTVEKD IKNLSKLSML SIRENKIKQL PAEIGELCNL ITLDVAHNQL
251 EHLPKEIGNC TQITNLDLQH NELLDLPDTI GNLSSLSRLG LRYNRLSAIP
301 RSLAKCSALE ELNLENNNIS TLPESLLSSL VKLNSLTLAR NCFQLYPVGG
351 PSQFSTIYSL NMEHNRINKI PFGIFSRAKV LSKLNMKDNQ LTSLPLDFGT
401 WTSMVELNLA TNQLTKIPED VSGLVSLEVL ILSNNLLKKL PHGLGNLRKL
451 RELDLEENKL ESLPNEIAYL KDLQKLVLTN NQLTTLPRGI GHLTNLTHLG
501 LGENLLTHLP EEIGTLENLE ELYLNDNPNL HSLPFELALC SKLSIMSIEN
551 CPLSHLPPQI VAGGPSFIIQ FLKMQGPYRA MV
```

Residue Number Increasing Mass Decreasing Mass

Start - End	Observed	Mr(expt)	Mr(calc)	Delta	Miss	Sequence
1 - 9	982.5544	981.5471	981.4800	0.0671	1	MSSSLGKEK Oxidation (M) (No match)
370 - 377	936.5132	935.5059	935.5228	-0.0169	0	IPFGIFSR (No match)
476 - 488	1482.8365	1481.8292	1481.8565	-0.0273	0	LVLTNNQLTTLPR (No match)
476 - 488	1482.8365	1481.8292	1481.8565	-0.0273	0	LVLTNNQLTTLPR (Ions score 75)

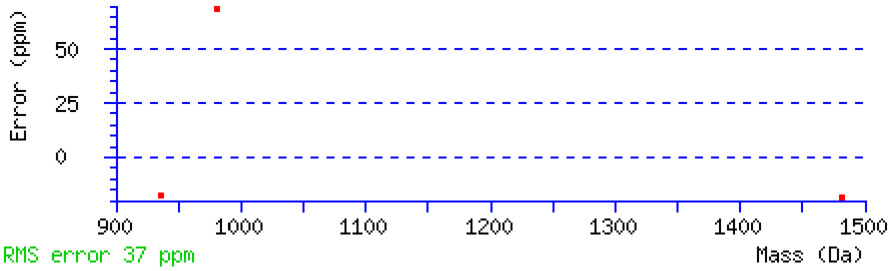

Spot 319

Protein View

Match to: **gi|4503377** Score: **479** Expect: **2.4e-043**
dihydropyrimidinase-like 2 [Homo sapiens]

Nominal mass (M_r): **62711**; Calculated pI value: **5.95**
NCBI BLAST search of gi|4503377 against nr
Unformatted sequence string for pasting into other applications

Taxonomy: Homo sapiens
Links to retrieve other entries containing this sequence from NCBI Entrez:
gi|3122051 from Homo sapiens
gi|75054993 from Pongo pygmaeus
gi|1244400 from Homo sapiens
gi|1330240 from Homo sapiens
gi|2967519 from Homo sapiens
gi|6467213 from Homo sapiens
gi|33991634 from Homo sapiens
gi|45501286 from Homo sapiens
gi|55729384 from Pongo pygmaeus
gi|119583977 from Homo sapiens
gi|119583978 from Homo sapiens
gi|158256010 (no taxonomy information for this entry)

Fixed modifications: Carbamidomethyl (C)
Variable modifications: Oxidation (M)
Cleavage by Trypsin: cuts C-term side of KR unless next residue is P
Sequence Coverage: **40%**

Matched peptides shown in **Bold Red**

1 MSYQGKKNIP RITSDRLLIK GGKIVNDDQS FYADIYMEDG LIK**QIGENLI**
51 **VPGGVK**TIEA HSR**MVIPGGI DVHTRFQMPD QGMTSADDFF QGTK**AALAGG
101 TTMIIDHVVP EPGTSLLAAF DQWREWADSK SCCDYSLHVD ISEWHKGIQE
151 EMEALVK**DHG VNSFLVYMAF K**DR**FQLTDCQ IYEVLSVIRD IGAIAQVHAE**
201 **NGDIIAEEQQ RILDLGITGP EGHVLSRPEE VEAEAVNRAI TIANQTNCPL**
251 **YITK**VMSK**SS AEVIAQAR**KK GTVVYGEPIT ASLGTDGSHY WSKNWAKAAA
301 FVTSPPLSPD PTTPDFLNSL LSCGDLQVTG SAHCTFNTAQ KAVGK**DNFTL**
351 **IPEGTNGTEE R**MSVIWDKAV VTGKMDENQF VAVTSTNAAK **VFNLYPR**KGR
401 IAVGSDADLV IWDPDSVKTI SAKTHNSSLE YNIFEGMECR GSPLVVISQG
451 K**IVLEDGTLH VTEGSGR**YIP R**KPFPDFVYK R**IKARSRLAE LRGVPR**GLYD**
501 **GPVCEVSVTP K**TVTPASSAK TSPAKQQAPP VR**NLHQSGFS LSGAQIDDNI**
551 **PRR**TTQRIVA PPGGRANITS LG

Residue Number Increasing Mass Decreasing Mass

Start - End	Observed	Mr(expt)	Mr(calc)	Delta	Miss	Sequence
44 - 56	1323.8278	1322.8205	1322.7557	0.0648	0	QIGENLIVPGGVK (No match)
64 - 75	1310.7562	1309.7489	1309.6812	0.0677	0	MVIPGGIDVHTR Oxidation (M) (No match)
76 - 94	2183.0396	2182.0323	2181.8983	0.1341	0	FQMPDQGMTSADDFFQGTK 2 Oxidation (M) (No match)
158 - 171	1643.8831	1642.8758	1642.7813	0.0945	0	DHGVNSFLVYMAFK Oxidation (M) (No match)
174 - 189	1984.1272	1983.1199	1983.0135	0.1064	0	FQLTDCQIYEVLSVIR (Ions score 64)
174 - 189	1984.1272	1983.1199	1983.0135	0.1064	0	FQLTDCQIYEVLSVIR (No match)
190 - 211	2377.2996	2376.2923	2376.1669	0.1255	0	DIGAIAQVHAENGDIIAEEQQR (No match)
190 - 211	2377.2996	2376.2923	2376.1669	0.1255	0	DIGAIAQVHAENGDIIAEEQQR (Ions score 90)
212 - 238	2900.6709	2899.6636	2899.5038	0.1598	0	ILDLGITGPEGHVLSRPEEVEAEAVNR (No match)
239 - 254	1821.0615	1820.0542	1819.9502	0.1040	0	AITIANQTNCPLYITK (No match)
259 - 268	1031.6044	1030.5971	1030.5407	0.0565	0	SSAEVIAQAR (No match)
346 - 361	1792.9351	1791.9278	1791.8275	0.1004	0	DNFTLIPEGTNGTEER (No match)
391 - 397	908.5433	907.5360	907.4915	0.0445	0	VFNLYPR (No match)
452 - 467	1682.9681	1681.9608	1681.8634	0.0974	0	IVLEDGTLHVTEGSGR (No match)
452 - 467	1682.9681	1681.9608	1681.8634	0.0974	0	IVLEDGTLHVTEGSGR (Ions score 56)
472 - 480	1140.6676	1139.6603	1139.6015	0.0589	0	KPFPDFVYK (No match)
472 - 481	1296.7811	1295.7738	1295.7026	0.0712	1	KPFPDFVYKR (No match)
472 - 481	1296.7811	1295.7738	1295.7026	0.0712	1	KPFPDFVYKR (Ions score 34)
497 - 511	1620.8849	1619.8776	1619.7864	0.0912	0	GLYDGPVCEVSVTPK (No match)
533 - 552	2169.1877	2168.1804	2168.0610	0.1195	0	NLHQSGFSLSGAQIDDNIPR (Ions score 39)
533 - 552	2169.1877	2168.1804	2168.0610	0.1195	0	NLHQSGFSLSGAQIDDNIPR (No match)
533 - 553	2325.3022	2324.2949	2324.1621	0.1328	1	NLHQSGFSLSGAQIDDNIPRR (No match)

Spot 320

Protein View

Match to: **gi|62089240** Score: **144** Expect: **7.7e-010**
copine I variant [Homo sapiens]

Nominal mass (M_r): **57352**; Calculated pI value: **5.49**
NCBI BLAST search of gi|62089240 against nr
Unformatted sequence string for pasting into other applications

Taxonomy: Homo sapiens

Fixed modifications: Carbamidomethyl (C)
Variable modifications: Oxidation (M)
Cleavage by Trypsin: cuts C-term side of KR unless next residue is P
Sequence Coverage: **11%**

Matched peptides shown in **Bold Red**

```
  1 GVNWMAHCVT LVQLSISCDH LIDKDIGSKS DPLCVLLQDV GGGSWAELGR
 51 TERVRNCSSP EFSKTLQLEY RFETVQKLRF GIYDIDNKTP ELRDDDFLGG
101 AECSLGQIVS SQVLTLPLML KPGKPAGRGT ITDFLGKSDP FLEFFRQGDG
151 KWHLVYRSEV IKNNLNPTWK RFSVPVQHFC GGNPSTPIQV QCSDYDSDGS
201 HDLIGTFHTS LAQLQAVPAE FECIHPEKQQ KKKSYKNSGT IRVKICRVET
251 EYSFLDYVMG GCQINFTVGV DFTGSNGDPS SPDSLHYLSP TGVNEYLMAL
301 WSVGSVVQDY DSDKLFPAFG FGAQVPPDWQ VSHEFALNFN PSNPYCAGIQ
351 GIVDAYRQAL PQVRLYGPTN FAPIINHVAR FAAQAAHQGT ASQYFMLLLL
401 TDGAVTDVEA TREAVVRASN LPMSVIIVGV GGADFEAMEQ LDADGGPLHT
451 RSGQAAARDI VQFVPYRRFQ NAPREALAQT VLAEVPTQLV SYFRAQGWAP
501 LKPLPPSAKD PAQAPQA
```

Residue Number Increasing Mass Decreasing Mass

Start - End	Observed	Mr(expt)	Mr(calc)	Delta	Miss	Sequence
138 - 146	1157.5514	1156.5441	1156.5553	-0.0111	0	SDPFLEFFR (No match)
138 - 146	1157.5514	1156.5441	1156.5553	-0.0111	0	SDPFLEFFR (No match)
152 - 157	873.4694	872.4621	872.4657	-0.0035	0	WHLVYR (No match)
365 - 380	1782.9412	1781.9339	1781.9576	-0.0237	0	LYGPTNFAPIINHVAR (No match)
365 - 380	1782.9412	1781.9339	1781.9576	-0.0237	0	LYGPTNFAPIINHVAR (Ions score 40)
459 - 467	1136.5990	1135.5917	1135.6025	-0.0108	0	DIVQFVPYR (No match)

459 - 467	1136.5990	1135.5917	1135.6025	-0.0108	0	DIVQFVPYR	(Ions score 15)
459 - 468	1292.6847	1291.6774	1291.7036	-0.0262	1	DIVQFVPYRR	(No match)
475 - 494	2235.1689	2234.1616	2234.1946	-0.0330	0	EALAQTVLAEVPTQLVSYFR	(Ions score 29)
475 - 494	2235.1689	2234.1616	2234.1946	-0.0330	0	EALAQTVLAEVPTQLVSYFR	(No match)

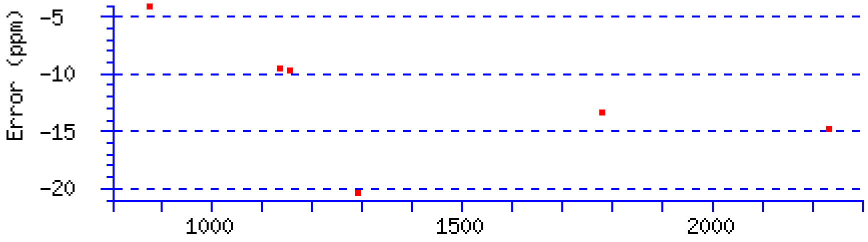

RMS error 12 ppm

Spot 321

Protein View

Match to: **gi|4507909** Score: **141** Expect: **1.5e-009**
Wiskott-Aldrich syndrome protein [Homo sapiens]

Nominal mass (M_r): **53108;** Calculated pI value: **6.18**
NCBI BLAST search of gi|4507909 against nr
Unformatted sequence string for pasting into other applications

Taxonomy: Homo sapiens
Links to retrieve other entries containing this sequence from NCBI Entrez:
gi|1722836 from Homo sapiens
gi|695151 from Homo sapiens
gi|854673 from Homo sapiens
gi|15215303 from Homo sapiens
gi|119571143 from Homo sapiens
gi|119571144 from Homo sapiens
gi|157929230 (no taxonomy information for this entry)
gi|1096899 from Homo sapiens

Fixed modifications: Carbamidomethyl (C)
Variable modifications: Oxidation (M)
Cleavage by Trypsin: cuts C-term side of KR unless next residue is P
Sequence Coverage: **13%**

Matched peptides shown in **Bold Red**

```
  1 MSGGPMGGRP GGRGAPAVQQ NIPSTLLQDH ENQRLFEMLG RKCLTLATAV
 51 VQLYLALPPG AEHWTKEHCG AVCFVKDNPQ KSYFIRLYGL QAGRLLWEQE
101 LYSQLVYSTP TPFFHTFAGD DCQAGLNFAD EDEAQAFRAL VQEKIQKRNQ
151 RQSGDRRQLP PPPTPANEER RGGLPPLPLH PGGDQGGPPV GPLSLGLATV
201 DIQNPDITSS RYRGLPAPGP SPADKKRSGK KKISKADIGA PSGFKHVSHV
251 GWDPQNGFDV NNLDPDLRSL FSRAGISEAQ LTDAETSKLI YDFIEDQGGL
301 EAVRQEMRRQ EPLPPPPPPS RGGNQLPRPP IVGGNKGRSG PLPPVPLGIA
351 PPPPTPRGPP PPGRGGPPPP PPPATGRSGP LPPPPPGAGG PPMPPPPPPP
401 PPPPSSGNGP APPPLPPALV PAGGLAPGGG RGALLDQIRQ GIQLNKTPGA
451 PESSALQPPP QSSEGLVGAL MHVMQKRSRA IHSSDEGEDQ AGDEDEDDEW
501 DD
```

Residue Number Increasing Mass Decreasing Mass

Start - End	Observed	Mr(expt)	Mr(calc)	Delta	Miss	Sequence
42 - 66	2780.4070	2779.3997	2779.5094	-0.1097	1	KCLTLATAVVQLYLALPPGAEHWTK (No match)
309 - 321	1467.7861	1466.7788	1466.7994	-0.0205	1	RQEPLPPPPPPSR (No match)
310 - 321	1311.6857	1310.6784	1310.6983	-0.0198	0	QEPLPPPPPPSR (No match)
310 - 321	1311.6857	1310.6784	1310.6983	-0.0198	0	QEPLPPPPPPSR (Ions score 32)
322 - 336	1503.8156	1502.8083	1502.8317	-0.0233	0	GGNQLPRPPIVGGNK (Ions score 24)
322 - 336	1503.8156	1502.8083	1502.8317	-0.0233	0	GGNQLPRPPIVGGNK (No match)
365 - 377	1197.6239	1196.6166	1196.6302	-0.0135	0	GGPPPPPPPATGR (Ions score 33)
365 - 377	1197.6239	1196.6166	1196.6302	-0.0135	0	GGPPPPPPPATGR (No match)

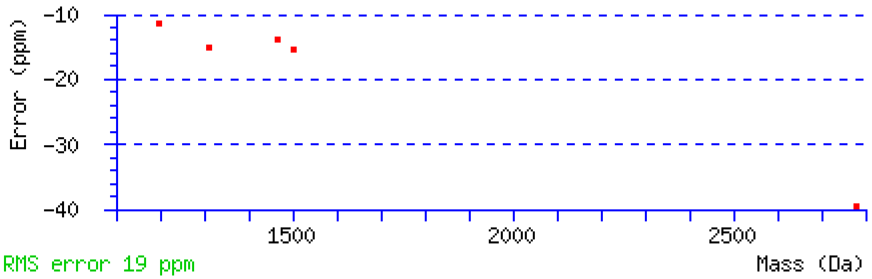

Spot 322

Protein View

Match to: **gi|18105063** Score: **284** Expect: **7.7e-024**
vacuolar protein sorting 45A [Homo sapiens]

Nominal mass (M_r): **65435**; Calculated pI value: **8.41**
NCBI BLAST search of gi|18105063 against nr
Unformatted sequence string for pasting into other applications

Taxonomy: Homo sapiens
Links to retrieve other entries containing this sequence from NCBI Entrez:
gi|23396937 from Homo sapiens
gi|9294733 from Homo sapiens
gi|10434974 from Homo sapiens
gi|15277875 from Homo sapiens
gi|22382109 from Homo sapiens
gi|55959177 from Homo sapiens
gi|119573969 from Homo sapiens
gi|119573970 from Homo sapiens

Fixed modifications: Carbamidomethyl (C)
Variable modifications: Oxidation (M)
Cleavage by Trypsin: cuts C-term side of KR unless next residue is P
Sequence Coverage: **11%**

Matched peptides shown in **Bold Red**

```
  1 MNVVFAVKQY ISKMIEDSGP GMKVLLMDKE TTGIVSMVYT QSEILQKEVY
 51 LFERIDSQNR EIMKHLKAIC FLRPTKENVD YIIQELRRPK YTIYFIYFSN
101 VISKSDVKSL AEADEQEVVA EVQEFYGDYI AVNPHLFSLN ILGCCQGRNW
151 DPAQLSRTTQ GLTALLLSLK KCPMIRYQLS SEAAKRLAEC VKQVITKEYE
201 LFEFRRTEVP PLLLILDRCD DAITPLLNQW TYQAMVHELL GINNNRIDLS
251 RVPGISKDLR EVVLSAENDE FYANNMYLNF AEIGSNIKNL MEDFQKKKPK
301 EQQKLESIAD MKAFVENYPQ FKKMSGTVSK HVTVVGELSR LVSERNLLEV
351 SEVEQELACQ NDHSSALQNI KRLLQNPKVT EFDAARLVML YALHYERHSS
401 NSLPGLMMDL RNKGVSEKYR KLVSAVVEYG GKRVRGSDLF SPKDAVAITK
451 QFLKGLKGVE NVYTQHQPFL HETLDHLIKG RLKENLYPYL GPSTLRDRPQ
501 DIIVFVIGGA TYEEALTVYN LNRTTPGVRI VLGGTTVHNT KSFLEEVLAS
551 GLHSRSKESS QVTSRSASRR
```

Residue Number Increasing Mass Decreasing Mass

Start - End	Observed	Mr(expt)	Mr(calc)	Delta	Miss	Sequence
48 - 54	955.4709	954.4636	954.4810	-0.0174	0	EVYLFER (No match)
77 - 87	1391.6919	1390.6846	1390.7092	-0.0246	0	ENVDYIIQELR (Ions score 47)
77 - 87	1391.6919	1390.6846	1390.7092	-0.0246	0	ENVDYIIQELR (No match)
198 - 206	1288.6104	1287.6031	1287.6247	-0.0216	1	EYELFEFRR (No match)
198 - 206	1288.6104	1287.6031	1287.6247	-0.0216	1	EYELFEFRR (Ions score 6)
387 - 397	1423.7089	1422.7016	1422.7329	-0.0313	0	LVMLYALHYER Oxidation (M) (Ions score 14)
387 - 397	1423.7089	1422.7016	1422.7329	-0.0313	0	LVMLYALHYER Oxidation (M) (No match)
482 - 496	1763.9384	1762.9311	1762.9617	-0.0306	1	LKENLYPYLGPSTLR (Ions score 79)
482 - 496	1763.9384	1762.9311	1762.9617	-0.0306	1	LKENLYPYLGPSTLR (No match)
542 - 555	1544.7823	1543.7750	1543.7994	-0.0244	0	SFLEEVLASGLHSR (Ions score 50)
542 - 555	1544.7823	1543.7750	1543.7994	-0.0244	0	SFLEEVLASGLHSR (No match)

Spot 323 a

Protein View

Match to: **gi|5803181** Score: **156** Expect: **4.8e-011**
stress-induced-phosphoprotein 1 (Hsp70/Hsp90-organizing protein) [Homo sapiens]

Nominal mass (M_r): **63227**; Calculated pI value: **6.40**
NCBI BLAST search of gi|5803181 against nr
Unformatted sequence string for pasting into other applications

Taxonomy: Homo sapiens
Links to retrieve other entries containing this sequence from NCBI Entrez:
gi|114638257 from Pan troglodytes
gi|400042 from Homo sapiens
gi|184565 from Homo sapiens
gi|12804257 from Homo sapiens
gi|49168510 from Homo sapiens
gi|54696882 from Homo sapiens
gi|54696884 from Homo sapiens
gi|61356792 from synthetic construct
gi|61356797 from synthetic construct
gi|119594602 from Homo sapiens
gi|119594604 from Homo sapiens
gi|123993239 from synthetic construct
gi|157928502 (no taxonomy information for this entry)

Fixed modifications: Carbamidomethyl (C)
Variable modifications: Oxidation (M)
Cleavage by Trypsin: cuts C-term side of KR unless next residue is P
Sequence Coverage: **31%**

Matched peptides shown in **Bold Red**

1 MEQVNELKEK GNKALSVGNI DDALQCYSEA IK**LDPHNHVL YSNR**SAAYAK
51 KGDYQKAYED GCKTVDLKPD WGKGYSR**KAA ALEFLNRFEE AK**R**TYEEGLK**
101 **HEANNPQLKE GLQNMEAR**LA ER**KFMNPFNM PNLYQK**LESD PRTR**TLLSDP**
151 **TYRELIEQLR** NKPSDLGTKL QDPRIMTTLS VLLGVDLGSM DEEEEIATPP
201 PPPPPKKETK PEPMEEDLPE NKKQALKEKE LGNDAYKKKD FDTALKHYDK
251 AK**ELDPTNMT YITNQAAVYF EK**GDYNKCRE LCEKAIEVGR ENREDYRQIA
301 KAYAR**IGNSY FKEEKYKDAI HFYNK**SLAEH RTPDVLKKCQ QAEK**ILKEQE**
351 **RLAYINPDLA LEEK**NKGNEC FQKGDYPQAM KHYTEAIKRN PKDAKLYSNR
401 AACYTKLLEF QLALKDCEEC IQLEPTFIKG YTRKAAALEA MKDYTKAMDV
451 YQKALDLDSS CKEAADGYQR CMMAQYNRHD SPEDVKRR**AM ADPEVQQIMS**
501 **DPAMR**LILEQ MQKDPQALSE HLKNPVIAQK IQK**LMDVGLI AIR**

Residue Number Increasing Mass Decreasing Mass

Start - End	Observed	Mr(expt)	Mr(calc)	Delta	Miss	Sequence
33 - 44	1464.7632	1463.7559	1463.7269	0.0290	0	LDPHNHVLYSNR (Ions score 17)
33 - 44	1464.7632	1463.7559	1463.7269	0.0290	0	LDPHNHVLYSNR (No match)
78 - 87	1132.6698	1131.6625	1131.6400	0.0226	1	KAAALEFLNR (No match)
78 - 87	1132.6698	1131.6625	1131.6400	0.0226	1	KAAALEFLNR (No match)
79 - 87	1004.5715	1003.5642	1003.5450	0.0192	0	AAALEFLNR (No match)
79 - 92	1608.8754	1607.8681	1607.8307	0.0375	1	AAALEFLNRFEEAK (No match)
94 - 109	1870.9758	1869.9685	1869.9220	0.0465	1	TYEEGLKHEANNPQLK (No match)
110 - 118	1063.5012	1062.4939	1062.4763	0.0176	0	EGLQNMEAR Oxidation (M) (No match)
123 - 136	1803.8961	1802.8888	1802.8483	0.0405	1	KFMNPFNMPNLYQK 2 Oxidation (M) (Ions score 4)
123 - 136	1803.8961	1802.8888	1802.8483	0.0405	1	KFMNPFNMPNLYQK 2 Oxidation (M) (No match)
124 - 136	1675.8005	1674.7932	1674.7534	0.0399	0	FMNPFNMPNLYQK 2 Oxidation (M) (No match)
145 - 153	1065.5730	1064.5657	1064.5502	0.0155	0	TLLSDPTYR (No match)
154 - 160	900.5266	899.5193	899.5076	0.0117	0	ELIEQLR (No match)
253 - 272	2364.1775	2363.1702	2363.0991	0.0712	0	ELDPTNMTYITNQAAVYFEK Oxidation (M) (No match)
306 - 315	1214.6300	1213.6227	1213.5978	0.0249	1	IGNSYFKEEK (No match)
316 - 325	1298.6763	1297.6690	1297.6455	0.0236	1	YKDAIHFYNK (No match)
345 - 351	915.5372	914.5299	914.5185	0.0114	1	ILKEQER (No match)
352 - 364	1488.8220	1487.8147	1487.7871	0.0276	0	LAYINPDLALEEK (No match)
489 - 505	1937.8887	1936.8814	1936.8328	0.0486	0	AMADPEVQQIMSDPAMR 3 Oxidation (M) (No match)
534 - 543	1116.6605	1115.6532	1115.6372	0.0160	0	LMDVGLIAIR Oxidation (M) (No match)
534 - 543	1116.6605	1115.6532	1115.6372	0.0160	0	LMDVGLIAIR Oxidation (M) (Ions score 2)

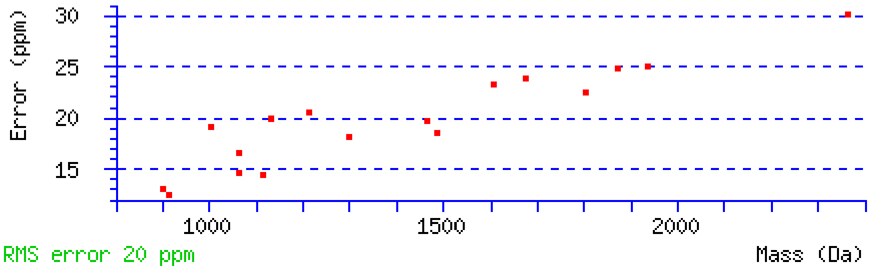

Spot 323 b

Protein View

Match to: **gi|12804207** Score: **191** Expect: **1.5e-014**
WAS protein [Homo sapiens]

Nominal mass (M_r): **54422**; Calculated pI value: **6.36**
NCBI BLAST search of gi|12804207 against nr
Unformatted sequence string for pasting into other applications

Taxonomy: Homo sapiens

Fixed modifications: Carbamidomethyl (C)
Variable modifications: Oxidation (M)
Cleavage by Trypsin: cuts C-term side of KR unless next residue is P
Sequence Coverage: **18%**

Matched peptides shown in **Bold Red**

```
  1 RASPEKTRAE STMSGGPMGG RPGGRGAPAV QQNIPSTLLQ DHENQRLFEM
 51 LGRKCLTLAT AVVQLYLALP PGAEHWTKEH CGAVCFVKDN PQKSYFIRLY
101 GLQAGRLLWE QELYSQLVYS TPTPFFHTFA GDDCQAGLNF ADEDEAQAFR
151 ALVQEKIQKR NQRQSGDRRQ LPPPPTPANE ERRGGLPPLP LHPGGDQGGP
201 PVGPLSLGLA TVDIQNPDIT SSRYRGLPAP GPSPADKKRS GKKKISKADI
251 GAPSGFKHVS HVGWDPQNGF DVNNLDPDLR SLFSRAGISE AQLTDAETSK
301 LIYDFIEDQG GLEAVRQEMR RQEPLPPPPP PSRGGNQLPR PPIVGGNKGR
351 SGPLPPVPLG IAPPPPTPRG PPPPGRGGPP PPPPPATGRS GPLPPPPPGA
401 GGPPMPPPPP PPPPPPSSGN GPAPPPLPPA LVPAGGLAPG GGRGALLDQI
451 RQGIQLNKTP GAPESSALQP PPQSSEGLVG ALMHVMQKRS RAIHSSDEGE
501 DQAGDEDEDD EWDD
```

Residue Number Increasing Mass Decreasing Mass

Start - End	Observed	Mr(expt)	Mr(calc)	Delta	Miss	Sequence
26 - 46	2316.1384	2315.1311	2315.1618	-0.0306	0	GAPAVQQNIPSTLLQDHENQR (No match)
26 - 46	2316.1384	2315.1311	2315.1618	-0.0306	0	GAPAVQQNIPSTLLQDHENQR (Ions score 56)
301 - 316	1837.9102	1836.9029	1836.9257	-0.0228	0	LIYDFIEDQGGLEAVR (No match)
321 - 333	1467.7797	1466.7724	1466.7994	-0.0269	1	RQEPLPPPPPPSR (No match)
334 - 348	1503.8220	1502.8147	1502.8317	-0.0169	0	GGNQLPRPPIVGGNK (Ions score 34)
334 - 348	1503.8220	1502.8147	1502.8317	-0.0169	0	GGNQLPRPPIVGGNK (No match)

351 - 369	1860.0515	1859.0442	1859.0668	-0.0226	0	**SGPLPPVPLGIAPPPPTPR**	(Ions score 39)
351 - 369	1860.0515	1859.0442	1859.0668	-0.0226	0	**SGPLPPVPLGIAPPPPTPR**	(No match)
377 - 389	1197.6265	1196.6192	1196.6302	-0.0109	0	**GGPPPPPPPATGR**	(No match)

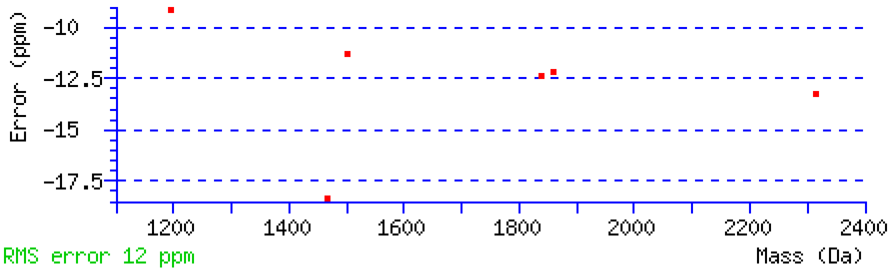

RMS error 12 ppm

Spot 324

Protein View

Match to: **gi|557900** Score: **95** Expect: **5.9e-005**
protein tyrosine phosphatase 1C

Nominal mass (M_r): **67924;** Calculated pI value: **7.27**
NCBI BLAST search of gi|557900 against nr
Unformatted sequence string for pasting into other applications

Taxonomy: Homo sapiens

Fixed modifications: Carbamidomethyl (C)
Variable modifications: Oxidation (M)
Cleavage by Trypsin: cuts C-term side of KR unless next residue is P
Sequence Coverage: **9%**

Matched peptides shown in **Bold Red**

```
  1 MVRWFLRDLS GLDAETLLKG RGVHGSFLAR PSRKNQGDFS LSVRVGDQVT
 51 HIRIQNSGDF YDLYGGEKFA TLTELVEYYT QQQGVLQDRD GTIIHLKYPL
101 NCSDPTSERW YHGHMSGGQA ETLLQAKGEP WTFLVRESLS QPGDFELSVL
151 SDQPKAGPGS PLRVTHIKVM CEGGRYTVGG LETFDSLTDL VEHFKKTGIE
201 EASGAFVYLR QPYYATRVNA ADIENRVLEL NKKQESEDTA KAGFWEEFES
251 LQKQEVKNLH QRLEGQRPEN KGKNRYKNIL PFDHSRVILQ GRDSNIPGSD
301 YINANYIKNQ LLGPDENAKT YIASQGCLEA TVNDFWQMAW QENSRVIVMT
351 TREVEKGRNK CVPYWPEVGM QRAYGPYSVT NCGEHDTTEY KLRTLQVSPL
401 DNGDLIREIW HYQYLSWPDH GVPSEPGGVL SFLDQINQRQ ESLPHAGPII
451 VHCSAGIGRT GTIIVIDMLM ENISTKGLDC DIDIQKTIQM VRAQRSGMVQ
501 TEAQYKFIYV AIAQFIETTK KKLEVLQSQK GQESEYGNIT YPPAMKNAHA
551 KASRTSSKHK EDVYENLHTK NKREEKVKKQ RSADKEKSKG SLKRK
```

Residue Number Increasing Mass Decreasing Mass

Start - End	Observed	Mr(expt)	Mr(calc)	Delta	Miss	Sequence
98 - 109	1438.6117	1437.6044	1437.6194	-0.0150	0	YPLNCSDPTSER (No match)
98 - 109	1438.6117	1437.6044	1437.6194	-0.0150	0	YPLNCSDPTSER (No match)
197 - 210	1512.7542	1511.7469	1511.7619	-0.0150	0	TGIEEASGAFVYLR (Ions score 26)
254 - 262	1151.7076	1150.7003	1150.6207	0.0797	1	QEVKNLHQR (No match)
276 - 286	1389.7164	1388.7091	1388.7200	-0.0109	1	YKNILPFDHSR (No match)

276 - 286	1389.7164	1388.7091	1388.7200	-0.0109	1	YKNILPFDHSR	(Ions score 23)
559 - 570	1512.7542	1511.7469	1511.7368	0.0101	1	HKEDVYENLHTK	(No match)

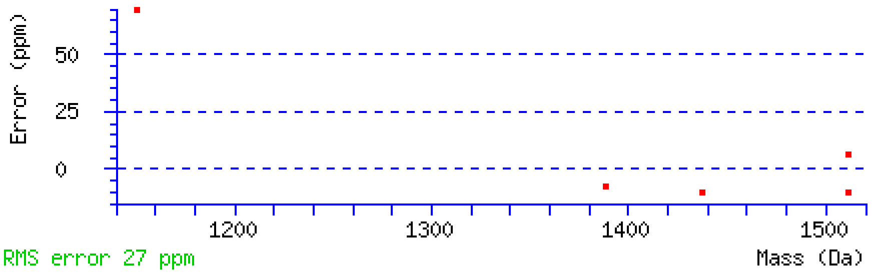

RMS error 27 ppm

Spot 325

Protein View

Match to: **gi|82407989** Score: **115** Expect: **6.1e-007**
Chain A, Crystal Structure Of Human Tyrosine Phosphatase Shp-1

Nominal mass (M_r): **60638**; Calculated pI value: **6.05**
NCBI BLAST search of gi|82407989 against nr
Unformatted sequence string for pasting into other applications

Taxonomy: Homo sapiens

Fixed modifications: Carbamidomethyl (C)
Variable modifications: Oxidation (M)
Cleavage by Trypsin: cuts C-term side of KR unless next residue is P
Sequence Coverage: **23%**

Matched peptides shown in **Bold Red**

```
  1 MVRWFHRDLS GLDAETLLKG RGVHGSFLAR PSRKNQGDFS LSVRVGDQVT
 51 HIRIQNSGDF YDLYGGEKFA TLTELVEYYT QQQGVLQDRD GTIIHLKYPL
101 NCSDPTSERW YHGHMSGGQA ETLLQAKGEP WTFLVRESLS QPGDFVLSVL
151 SDQPKAGPGS PLRVTHIKVM CEGGRYTVGG LETFDSLTDL VEHFKKTGIE
201 EASGAFVYLR QPYYATRVNA ADIENRVLEL NKKQESEDTA KAGFWEEFES
251 LQKQEVKNLH QRLEGQRPEN KGKNRYKNIL PFDHSRVILQ GRDSNIPGSD
301 YINANYIKNQ LLGPDENAKT YIASQGCLEA TVNDFWQMAW QENSRVIVMT
351 TREVEKGRNK CVPYWPEVGM QRAYGPYSVT NCGEHDTTEY KLRTLQVSPL
401 DNGDLIREIW HYQYLSWPDH GVPSEPGGVL SFLDQINQRQ ESLPHAGPII
451 VHCSAGIGRT GTIIVIDMLM ENISTKGLDC DIDIQKTIQM VRAQRSGMVQ
501 TEAQYKFIYV AIAQFIETTK KKLEVLQSQK GQ
```

Residue Number Increasing Mass Decreasing Mass

Start - End	Observed	Mr(expt)	Mr(calc)	Delta	Miss	Sequence
35 - 44	1122.5826	1121.5753	1121.5465	0.0288	0	NQGDFSLSVR (No match)
54 - 68	1705.8043	1704.7970	1704.7630	0.0340	0	IQNSGDFYDLYGGEK (No match)
69 - 89	2502.2871	2501.2798	2501.2437	0.0361	0	FATLTELVEYYTQQQGVLQDR (No match)
69 - 89	2502.2871	2501.2798	2501.2437	0.0361	0	FATLTELVEYYTQQQGVLQDR (No match)
98 - 109	1438.6522	1437.6449	1437.6194	0.0255	0	YPLNCSDPTSER (No match)
98 - 109	1438.6522	1437.6449	1437.6194	0.0255	0	YPLNCSDPTSER (No match)

128 - 136	1104.5961	1103.5888	1103.5763	0.0125	0	**GEPWTFLVR**	(No match)
197 - 210	1512.7964	1511.7891	1511.7619	0.0272	0	**TGIEEASGAFVYLR**	(Ions score 11)
197 - 210	1512.7964	1511.7891	1511.7619	0.0272	0	**TGIEEASGAFVYLR**	(No match)
276 - 286	1389.7500	1388.7427	1388.7200	0.0227	1	**YKNILPFDHSR**	(No match)
278 - 286	1098.5848	1097.5775	1097.5618	0.0158	0	**NILPFDHSR**	(No match)
394 - 407	1540.8590	1539.8517	1539.8256	0.0261	0	**TLQVSPLDNGDLIR**	(Ions score 14)
394 - 407	1540.8590	1539.8517	1539.8256	0.0261	0	**TLQVSPLDNGDLIR**	(No match)
440 - 459	2099.1130	2098.1057	2098.0741	0.0316	0	**QESLPHAGPIIVHCSAGIGR**	(No match)

Spot 326

Protein View

Match to: **gi|119590931** Score: **401** Expect: **1.5e-035**
5-aminoimidazole-4-carboxamide ribonucleotide formyltransferase/IMP cyclohydrolase, isoform CRA_c [

Nominal mass (M_r): **65075;** Calculated pI value: **6.27**
NCBI BLAST search of gi|119590931 against nr
Unformatted sequence string for pasting into other applications

Taxonomy: Homo sapiens

Fixed modifications: Carbamidomethyl (C)
Variable modifications: Oxidation (M)
Cleavage by Trypsin: cuts C-term side of KR unless next residue is P
Sequence Coverage: **44%**

Matched peptides shown in **Bold Red**

```
  1 MAPGQLALFS VSDKTGLVEF ARNLTALGLN LVASGGTAKA LRDAGLAVRD
 51 VSELTGFPEM LGGRVKTLHP AVHAGILARN IPEDNADMAR LDFNLIRVVA
101 CNLYPFVKTV ASPGVSVEEA VEQIDIGGVT LLRAAAKNHA RVTVVCEPED
151 YVVVSTEMQS SESKDTSLET RRQLALKAFT HTAQYDEAIS DYFRKQYSKG
201 VSQMPLRYGM NPHQTPAQLY TLQPKLPITV LNGAPGFINL CDALNAWQLV
251 KELKEALGIP AAASFKHVSP AGAAVGIPLS EDEAKVCMVY DLYKTLTPIS
301 AAYARARGAD RMSSFGDFVA LSDVCDVPTA KIISREVSDG IIAPGYEEEA
351 LTILSKKKNG NYCVLQMDQS YKPDENEVRT LFGLHLSQKR NNGVVDKSLF
401 SNVVTKNKDL PESALRDLIV ATIAVKYTQS NSVCYAKNGQ VIGIGAGQQS
451 RIHCTRLAGD KANYWWLRHH PQVLSMKFKT GVKRAEISNA IDQYVTGTIG
501 EDEDLIKWKA LFEEVPELLT EAEKKEWVEK LTEVSISSDA FFPFRDNVDR
551 AKRSGVAYIA APSGSAADKV VIEACDELGI ILAHTNLRLF HH
```

Residue Number Increasing Mass Decreasing Mass

Start - End	Observed	Mr(expt)	Mr(calc)	Delta	Miss	Sequence
2 - 14	1332.7183	1331.7110	1331.7085	0.0026	0	APGQLALFSVSDK (No match)
2 - 22	2206.2012	2205.1939	2205.1793	0.0147	1	APGQLALFSVSDKTGLVEFAR (No match)
15 - 22	892.5000	891.4927	891.4814	0.0114	0	TGLVEFAR (No match)
23 - 39	1599.9213	1598.9140	1598.8991	0.0149	0	NLTALGLNLVASGGTAK (No match)
50 - 64	1623.7816	1622.7743	1622.7609	0.0134	0	DVSELTGFPEMLGGR Oxidation (M) (No match)

50 - 64	1623.7816	1622.7743	1622.7609	0.0134	0	DVSELTGFPEMLGGR Oxidation (M) (Ions score 18)
67 - 79	1355.8009	1354.7936	1354.7833	0.0103	0	TLHPAVHAGILAR (No match)
67 - 79	1355.8009	1354.7936	1354.7833	0.0103	0	TLHPAVHAGILAR (Ions score 22)
80 - 90	1261.5570	1260.5497	1260.5404	0.0093	0	NIPEDNADMAR Oxidation (M) (No match)
91 - 97	890.5095	889.5022	889.5021	0.0001	0	LDFNLIR (No match)
98 - 108	1309.7046	1308.6973	1308.6900	0.0074	0	VVACNLYPFVK (No match)
178 - 194	2034.9335	2033.9262	2033.9119	0.0144	0	AFTHTAQYDEAISDYFR (Ions score 45)
178 - 194	2034.9335	2033.9262	2033.9119	0.0144	0	AFTHTAQYDEAISDYFR (No match)
178 - 195	2163.0215	2162.0142	2162.0068	0.0074	1	AFTHTAQYDEAISDYFRK (No match)
200 - 207	903.4692	902.4619	902.4643	-0.0024	0	GVSQMPLR Oxidation (M) (No match)
208 - 225	2103.0574	2102.0501	2102.0255	0.0247	0	YGMNPHQTPAQLYTLQPK Oxidation (M) (No match)
267 - 285	1847.9844	1846.9771	1846.9424	0.0347	0	HVSPAGAAVGIPLSEDEAK (No match)
295 - 305	1163.6481	1162.6408	1162.6346	0.0062	0	TLTPISAAYAR (No match)
295 - 305	1163.6481	1162.6408	1162.6346	0.0062	0	TLTPISAAYAR (Ions score 8)
336 - 356	2234.1746	2233.1673	2233.1365	0.0309	0	EVSDGIIAPGYEEEALTILSK (No match)
380 - 389	1143.6421	1142.6348	1142.6447	-0.0099	0	TLFGLHLSQK (No match)
380 - 390	1299.7681	1298.7608	1298.7459	0.0150	1	TLFGLHLSQKR (No match)
407 - 416	1142.6244	1141.6171	1141.6091	0.0080	1	NKDLPESALR (No match)
438 - 451	1384.7437	1383.7364	1383.7218	0.0146	0	NGQVIGIGAGQQSR (No match)
438 - 451	1384.7437	1383.7364	1383.7218	0.0146	0	NGQVIGIGAGQQSR (Ions score 31)
510 - 525	1846.0033	1844.9960	1844.9771	0.0190	1	ALFEEVPELLTEAEKK (No match)
531 - 545	1715.8811	1714.8738	1714.8566	0.0173	0	LTEVSISSDAFFPFR (No match)
531 - 550	2315.1631	2314.1558	2314.1229	0.0329	1	LTEVSISSDAFFPFRDNVDR (No match)

Spot 327

Protein View

Match to: **gi|12804207** Score: **174** Expect: **7.7e-013**
WAS protein [Homo sapiens]

Nominal mass (M_r): **54422**; Calculated pI value: **6.36**
NCBI BLAST search of gi|12804207 against nr
Unformatted sequence string for pasting into other applications

Taxonomy: Homo sapiens

Fixed modifications: Carbamidomethyl (C)
Variable modifications: Oxidation (M)
Cleavage by Trypsin: cuts C-term side of KR unless next residue is P
Sequence Coverage: **13%**

Matched peptides shown in **Bold Red**

```
  1 RASPEKTRAE STMSGGPMGG RPGGRGAPAV QQNIPSTLLQ DHENQRLFEM
 51 LGRKCLTLAT AVVQLYLALP PGAEHWTKEH CGAVCFVKDN PQKSYFIRLY
101 GLQAGRLLWE QELYSQLVYS TPTPFFHTFA GDDCQAGLNF ADEDEAQAFR
151 ALVQEKIQKR NQRQSGDRRQ LPPPPTPANE ERRGGLPPLP LHPGGDQGGP
201 PVGPLSLGLA TVDIQNPDIT SSRYRGLPAP GPSPADKKRS GKKKISKADI
251 GAPSGFKHVS HVGWDPQNGF DVNNLDPDLR SLFSRAGISE AQLTDAETSK
301 LIYDFIEDQG GLEAVRQEMR RQEPLPPPPP PSRGGNQLPR PPIVGGNKGR
351 SGPLPPVPLG IAPPPPTPRG PPPPGRGGPP PPPPPATGRS GPLPPPPPGA
401 GGPPMPPPPP PPPPPPSSGN GPAPPPLPPA LVPAGGLAPG GGRGALLDQI
451 RQGIQLNKTP GAPESSALQP PPQSSEGLVG ALMHVMQKRS RAIHSSDEGE
501 DQAGDEDEDD EWDD
```

Residue Number Increasing Mass Decreasing Mass

Start - End	Observed	Mr(expt)	Mr(calc)	Delta	Miss	Sequence
26 - 46	2316.1313	2315.1240	2315.1618	-0.0377	0	GAPAVQQNIPSTLLQDHENQR (No match)
26 - 46	2316.1313	2315.1240	2315.1618	-0.0377	0	GAPAVQQNIPSTLLQDHENQR (Ions score 25)
334 - 348	1503.8193	1502.8120	1502.8317	-0.0196	0	GGNQLPRPPIVGGNK (No match)
334 - 348	1503.8193	1502.8120	1502.8317	-0.0196	0	GGNQLPRPPIVGGNK (Ions score 23)
351 - 369	1860.0468	1859.0395	1859.0668	-0.0273	0	SGPLPPVPLGIAPPPPTPR (Ions score 44)
351 - 369	1860.0468	1859.0395	1859.0668	-0.0273	0	SGPLPPVPLGIAPPPPTPR (No match)

377 - 389	1197.6221	1196.6148	1196.6302	-0.0153	0	GGPPPPPPPATGR	(No match)
377 - 389	1197.6221	1196.6148	1196.6302	-0.0153	0	GGPPPPPPPATGR	(Ions score 39)

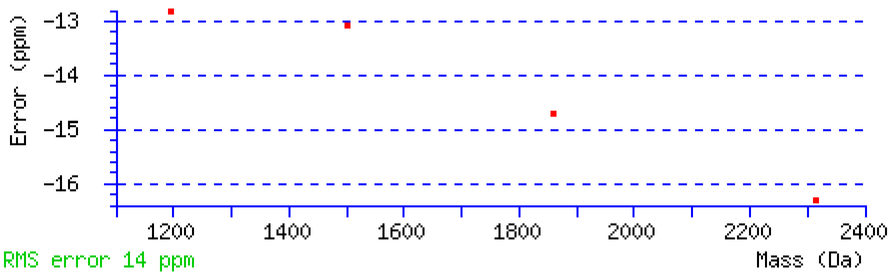

RMS error 14 ppm

Spot 328

Protein View

Match to: **gi|62896585** Score: **103** Expect: **9.6e-006**
adenylyl cyclase-associated protein variant [Homo sapiens]

Nominal mass (M_r): **51899**; Calculated pI value: **8.07**
NCBI BLAST search of gi|62896585 against nr
Unformatted sequence string for pasting into other applications

Taxonomy: Homo sapiens

Fixed modifications: Carbamidomethyl (C)
Variable modifications: Oxidation (M)
Cleavage by Trypsin: cuts C-term side of KR unless next residue is P
Sequence Coverage: **18%**

Matched peptides shown in **Bold Red**

```
  1 MADMQNLVER LERAVGRLEA VSHTSDMHRG YADSPSKAGA APYVQAFDSL
 51 LAGPVAEYLK ISKEIGGDVQ KHAEMVHTGL KLERALLVTA SQCQQPAENK
101 LSDLLAPISE QIKEVITFRE KNRGSKLFNH LSAVSESIQA LGWVAMAPKP
151 GPYVKEMNDA AMFYTNRVLK EYKDVDKKHV DWVKAYLSIW TELQAYIKEF
201 HTTGLAWSKT GPVAKELSGL PSGPSAGSGP PPPPPGPPPP PVSTSSGSDE
251 SASRSALFAQ INQGESITHA LKHVSDDMKT HKNPALKAQS GPVRSGPKPF
301 SAPKPQTSPS PKRATKKEPA VLELEGKKWR VENQENVSNL VIEDTELKQV
351 AYIYKCVNTT LQIKGKINSI TVDSCKKLGL VFDDVVGIVE IINSKDVKVQ
401 VMGKVPTISI NKTDGCHAYL SKNSLDCEIV SAKSSEMNVL IPTEGGDFNE
451 FPVPEQFKTL WNGQKLVTTV TEIAG
```

Residue Number Increasing Mass Decreasing Mass

Start - End	Observed	Mr(expt)	Mr(calc)	Delta	Miss	Sequence
38 - 60	2351.2214	2350.2141	2350.2208	-0.0067	0	AGAAPYVQAFDSLLAGPVAEYLK (No match)
85 - 100	1757.8821	1756.8748	1756.8777	-0.0029	0	ALLVTASQCQQPAENK (No match)
156 - 167	1462.6047	1461.5974	1461.6017	-0.0042	0	EMNDAAMFYTNR (Ions score 11)
156 - 167	1462.6047	1461.5974	1461.6017	-0.0042	0	EMNDAAMFYTNR (No match)
156 - 167	1478.5938	1477.5865	1477.5966	-0.0100	0	EMNDAAMFYTNR Oxidation (M) (No match)
156 - 167	1478.5938	1477.5865	1477.5966	-0.0100	0	EMNDAAMFYTNR Oxidation (M) (Ions score 1)
156 - 167	1494.5889	1493.5816	1493.5915	-0.0098	0	EMNDAAMFYTNR 2 Oxidation (M) (No match)

255 - 272	1928.0116	1927.0043	1927.0163	-0.0119	0	SALFAQINQGESITHALK	(No match)
255 - 272	1928.0116	1927.0043	1927.0163	-0.0119	0	SALFAQINQGESITHALK	(Ions score 50)
331 - 348	2073.0266	2072.0193	2072.0273	-0.0079	0	VENQENVSNLVIEDTELK	(No match)

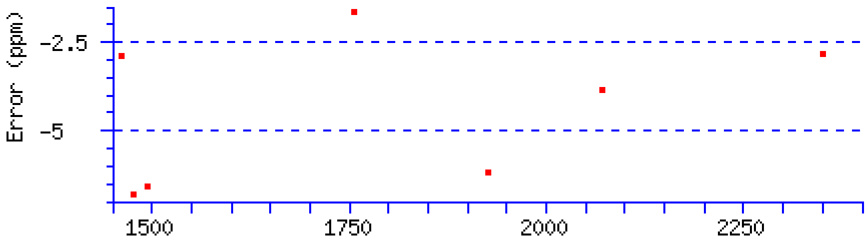

RMS error 5 ppm

Spot 329

Protein View

Match to: **gi|340217** Score: **94** Expect: **8.4e-005**
cytovillin 2

Nominal mass (M_r): **68233;** Calculated pI value: **5.80**
NCBI BLAST search of gi|340217 against nr
Unformatted sequence string for pasting into other applications

Taxonomy: Homo sapiens

Fixed modifications: Carbamidomethyl (C)
Variable modifications: Oxidation (M)
Cleavage by Trypsin: cuts C-term side of KR unless next residue is P
Sequence Coverage: **11%**

Matched peptides shown in **Bold Red**

```
  1 MDAELEFAIQ PNTTGKQLFD QVVKTIGLRE VWYFGLHYVD NKGFPTWLKL
 51 DKKVSAQEVR KENPLQFKFR AKFYPEDVAE ELIQDITQKL FFLQVKEGIL
101 SDEIYCPPET AVLLGSYAVQ AKFGDYNKEV HKSGYLSSER LIPQRVMDQH
151 KLTRDQWEDR IQVWHAEHRG MLKDNAMLEY LKIAQDLEMY GINYFEIKNK
201 KGTDLWLGVD ALGLNIYEKD DKLTPKIGFP WSEIRNISFN DKKFVIKPID
251 KKAPDFVFYA PRLRINKRIL QLCMGNHELY MRRRKPDTIE VQQMKAQARE
301 EKHQKQLERQ QLETEKKRRE TVEREKEQMM REKEELMLRL QDYEEKTKKA
351 ERELSEQIQR ALQLEEERKR AQEEAERLEA DRMAALRAKE ELERQAVDQI
401 KSQEQLAAEL AEYTAKIALL EEARRRKEDE VEEWQHRAKE AQDDLVKTKE
451 ELHLVMTAPP PPPPPVYEPV SYHVQESLQD EGAEPTGYSA ELSSEGIRDD
501 RNEEKRITEA EKNERVQRQL VTLSSELSQA RDENKRTHND IIHNENMRQG
551 RDKYKTLRQI RQGNTKQRID EFEAL
```

Residue Number Increasing Mass Decreasing Mass

Start - End	Observed	Mr(expt)	Mr(calc)	Delta	Miss	Sequence
17 - 24	976.5690	975.5617	975.5389	0.0228	0	QLFDQVVK (No match)
90 - 96	894.5745	893.5672	893.5374	0.0298	0	LFFLQVK (No match)
252 - 262	1310.7422	1309.7349	1309.6818	0.0531	1	KAPDFVFYAPR (No match)
252 - 262	1310.7422	1309.7349	1309.6818	0.0531	1	KAPDFVFYAPR (Ions score 8)
253 - 262	1182.6418	1181.6345	1181.5869	0.0476	0	APDFVFYAPR (Ions score 47)

253 - 262	1182.6418	1181.6345	1181.5869	0.0476	0	APDFVFYAPR (No match)
353 - 360	1002.5547	1001.5474	1001.5141	0.0333	0	ELSEQIQR (No match)
361 - 368	987.5477	986.5404	986.5032	0.0372	0	ALQLEEER (No match)
402 - 416	1651.8860	1650.8787	1650.8100	0.0687	0	SQEQLAAELAEYTAK (No match)
567 - 575	1120.6233	1119.6160	1119.5560	0.0600	1	QRIDEFEAL (No match)

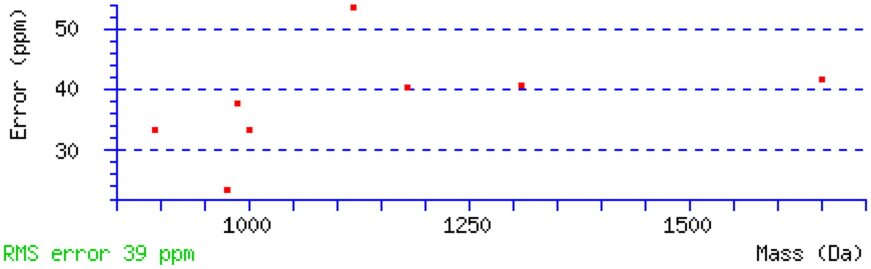

RMS error 39 ppm

Spot 331

Protein View

Match to: **gi|36796** Score: **162** Expect: **1.2e-011**
t-complex polypeptide 1 [Homo sapiens]

Nominal mass (M_r): **60869;** Calculated pI value: **6.03**
NCBI BLAST search of gi|36796 against nr
Unformatted sequence string for pasting into other applications

Taxonomy: Homo sapiens

Fixed modifications: Carbamidomethyl (C)
Variable modifications: Oxidation (M)
Cleavage by Trypsin: cuts C-term side of KR unless next residue is P
Sequence Coverage: **10%**

Matched peptides shown in **Bold Red**

```
  1 MEGPLSVFGD RSTGETIRSQ NVMAAASIAN IVKSSLGPVG LDKMLVDDIG
 51 DVTITNDGAT ILKLLEVEHP AAKVLCELAD LQDKEVGDGT TSVVIIAAEL
101 LKNADELVKQ KIHPTSVISG YRLACKEAVR YINENLIVNT DELGRDCLIN
151 AAKTSMSSKI IGINGDFFAN MVVDAVLAIK YTDIRGQPRY PVNSVNILKA
201 HGRSQMESML ISGYALNCVV GSQGMPKRIV NAKIACLDFS LQKTKMKLGV
251 QVVITDPEKL DQIRQRESDI TKERIQKILA TGANVILTTG GIDDMCLKYF
301 VEAGAMAVRR VLKRDLKRIA KASGATILST LANLEGEETF EAAMLGQAEE
351 VVQERICDDE LILIKNTKAR TSASIILRGA NDFMCDEMER SLHDALCVVK
401 RVLESKSVVP GGGAVEAALS IYLENYATSM GSREQLAIAE FARSLLVIPN
451 TLAVNAAQDS TDLVAKLRAF HNEAQVNPER KNLKWIGLDL SNGKPRDNKQ
501 AGVFEPTIVK VKSLKFATEA AITILRIDDL IKLHPEILRI KHGSYEDAVH
551 SGALND
```

Residue Number Increasing Mass Decreasing Mass

Start - End	Observed	Mr(expt)	Mr(calc)	Delta	Miss	Sequence
112 - 122	1229.6299	1228.6226	1228.6564	-0.0337	0	IHPTSVISGYR (Ions score 12)
131 - 145	1762.8617	1761.8544	1761.8896	-0.0352	0	YINENLIVNTDELGR (No match)
131 - 145	1762.8617	1761.8544	1761.8896	-0.0352	0	YINENLIVNTDELGR (Ions score 75)
299 - 309	1229.6299	1228.6226	1228.5910	0.0317	0	YFVEAGAMAVR Oxidation (M) (No match)
434 - 443	1147.6028	1146.5955	1146.6033	-0.0077	0	EQLAIAEFAR (Ions score 2)

434 - 443	1147.6028	1146.5955	1146.6033	-0.0077	0	EQLAIAEFAR	(No match)
469 - 480	1411.6538	1410.6465	1410.6640	-0.0174	0	AFHNEAQVNPER	(No match)
469 - 481	1539.7422	1538.7349	1538.7589	-0.0240	1	AFHNEAQVNPERK	(Ions score 16)
469 - 481	1539.7422	1538.7349	1538.7589	-0.0240	1	AFHNEAQVNPERK	(No match)

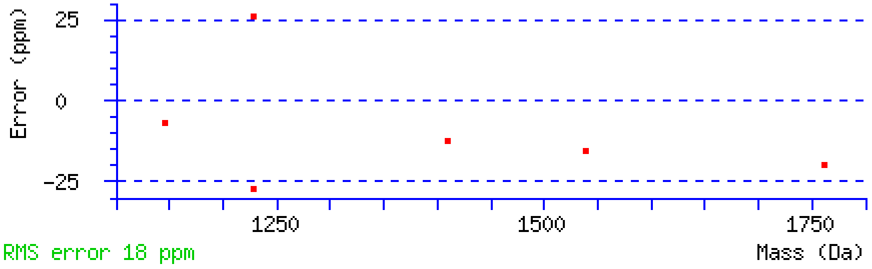

Spot 332

Protein View

Match to: **gi|33150628** Score: **172** Expect: **1.2e-012**
DCHT2 [Homo sapiens]

Nominal mass (M_r): **32518**; Calculated pI value: **6.88**
NCBI BLAST search of gi|33150628 against nr
Unformatted sequence string for pasting into other applications

Taxonomy: Homo sapiens

Fixed modifications: Carbamidomethyl (C)
Variable modifications: Oxidation (M)
Cleavage by Trypsin: cuts C-term side of KR unless next residue is P
Sequence Coverage: **21%**

Matched peptides shown in **Bold Red**

```
  1 MKVLMLTLQN DPPSLETGVQ DKEMLKKYGK SFRKMISLCL QKDPEKRPTA
 51 AELLRHKFFQ KAKNKEFLQE KTLQRAPTIS ERARKVRRVP GSGGRLHKTE
101 DGGWEWSDDE FDEESEEGKA AISQLRSPRV RESISNSELF PTTDPVGTLL
151 QVPEQISAHL PQPAGQIATQ PTQVSLPPTA EPAKTAQALS SGSGSQETKI
201 PISLVLRLRN SKKELNDIRF EFTPGRDTAE GVSQELISAG LVDGRDLVIV
251 AANLQKIVEE PQSNRSVTFK LASGVEGSDI PDDGKLIGFA QLSIS
```

Residue Number Increasing Mass Decreasing Mass

Start - End	Observed	Mr(expt)	Mr(calc)	Delta	Miss	Sequence
43 - 55	1495.7719	1494.7646	1494.8154	-0.0508	1	DPEKRPTAAELLR (Ions score 30)
43 - 55	1495.7719	1494.7646	1494.8154	-0.0508	1	DPEKRPTAAELLR (No match)
200 - 207	910.5771	909.5698	909.6011	-0.0313	0	IPISLVLR (No match)
214 - 226	1593.7471	1592.7398	1592.7946	-0.0548	1	ELNDIRFEFTPGR (Ions score 6)
214 - 226	1593.7471	1592.7398	1592.7946	-0.0548	1	ELNDIRFEFTPGR (No match)
227 - 245	1916.8944	1915.8871	1915.9486	-0.0615	0	DTAEGVSQELISAGLVDGR (No match)
227 - 245	1916.8944	1915.8871	1915.9486	-0.0615	0	DTAEGVSQELISAGLVDGR (Ions score 54)
257 - 265	1071.5061	1070.4988	1070.5356	-0.0368	0	IVEEPQSNR (No match)
257 - 265	1071.5061	1070.4988	1070.5356	-0.0368	0	IVEEPQSNR (Ions score 1)

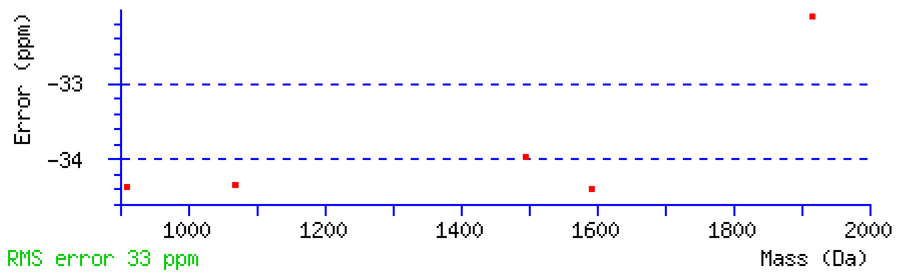

Error (ppm)
-33
-34
1000
1200
1400
1600
1800
2000
RMS error 33 ppm
Mass (Da)

Spot 335

Protein View

Match to: **gi|62897169** Score: **482** Expect: **1.2e-043**
nucleobindin 1 variant [Homo sapiens]

Nominal mass (M_r): **53874;** Calculated pI value: **5.15**
NCBI BLAST search of gi|62897169 against nr
Unformatted sequence string for pasting into other applications

Taxonomy: Homo sapiens

Fixed modifications: Carbamidomethyl (C)
Variable modifications: Oxidation (M)
Cleavage by Trypsin: cuts C-term side of KR unless next residue is P
Sequence Coverage: **46%**

Matched peptides shown in **Bold Red**

```
  1 MPPSGPRGTL LLLPLLLLLL LRAVLAVPLE RGAPNKEETP ATESPDTGLY
 51 YHRYLQEVID VLETDGHFRE KLQAANAEDI KSGKLSRELD FVSHHVRTRL
101 DELKRQEVSR LRMLLKAKMD AEQDPNVQVD HLNLLKQFEH LDPQNQHTFE
151 ARDLELLIQT ATRDLAQYDA AHHEEFKRYE MLKEHERRRY LESLGEEQRK
201 EAERKLEEQQ RRHREHPKVN VPGSQAQLKE VWEELDGLDP NRFNPKTFFI
251 LHDINSDGVL DEQELEALFT KELEKVYDPK NEEDDMREME EERLRMREHV
301 MKNVDTNQDR LVTLEEFLAS TQRKEFGDTG EGWETVEMHP AYTEEELRRF
351 EEELAAREAE LNAKAQRLSQ ETEALGRSQG RLEAQKRELQ QAVLHMEQRK
401 QQQQQQQGHK APAAHPEGQL KFHPDTDDVP VPAPAGDQKE VDTSEKKLLE
451 RLPEVEVPQH L
```

Residue Number Increasing Mass Decreasing Mass

Start - End	Observed	Mr(expt)	Mr(calc)	Delta	Miss	Sequence
54 - 69	1933.9718	1932.9645	1932.9581	0.0065	0	YLQEVIDVLETDGHFR (No match)
54 - 69	1933.9718	1932.9645	1932.9581	0.0065	0	YLQEVIDVLETDGHFR (Ions score 88)
88 - 97	1238.6357	1237.6284	1237.6203	0.0081	0	ELDFVSHHVR (No match)
88 - 97	1238.6357	1237.6284	1237.6203	0.0081	0	ELDFVSHHVR (Ions score 27)
137 - 152	1996.9313	1995.9240	1995.9187	0.0053	0	QFEHLDPQNQHTFEAR (No match)
153 - 163	1272.7244	1271.7171	1271.7085	0.0086	0	DLELLIQTATR (No match)
153 - 163	1272.7244	1271.7171	1271.7085	0.0086	0	DLELLIQTATR (Ions score 30)

164 - 178	1829.8596	1828.8523	1828.8492	0.0031	1	**DLAQYDAAHHEEFKR** (No match)
164 - 178	1829.8596	1828.8523	1828.8492	0.0031	1	**DLAQYDAAHHEEFKR** (Ions score 70)
179 - 187	1250.5883	1249.5810	1249.5761	0.0050	1	**YEMLKEHER** Oxidation (M) (No match)
190 - 199	1223.6046	1222.5973	1222.5829	0.0144	0	**YLESLGEEQR** (No match)
190 - 200	1351.6929	1350.6856	1350.6779	0.0078	1	**YLESLGEEQRK** (No match)
205 - 211	930.5084	929.5011	929.4930	0.0081	1	**KLEEQQR** (No match)
213 - 218	803.4321	802.4248	802.4198	0.0050	1	**HREHPK** (No match)
272 - 280	1120.5870	1119.5797	1119.5811	-0.0014	1	**ELEKVYDPK** (No match)
303 - 310	961.4446	960.4373	960.4261	0.0113	0	**NVDTNQDR** (No match)
311 - 323	1506.8232	1505.8159	1505.8089	0.0070	0	**LVTLEEFLASTQR** (Ions score 42)
311 - 323	1506.8232	1505.8159	1505.8089	0.0070	0	**LVTLEEFLASTQR** (No match)
311 - 324	1634.9196	1633.9123	1633.9039	0.0085	1	**LVTLEEFLASTQRK** (No match)
350 - 357	964.4798	963.4725	963.4661	0.0064	0	**FEEELAAR** (No match)
368 - 377	1103.5817	1102.5744	1102.5618	0.0126	0	**LSQETEALGR** (No match)
388 - 399	1481.8267	1480.8194	1480.7456	0.0738	0	**ELQQAVLHMEQR** (No match)
388 - 399	1497.7557	1496.7484	1496.7405	0.0079	0	**ELQQAVLHMEQR** Oxidation (M) (No match)
400 - 410	1365.7042	1364.6969	1364.6909	0.0061	1	**KQQQQQQQGHK** (No match)
411 - 421	1118.6010	1117.5937	1117.5880	0.0058	0	**APAAHPEGQLK** (No match)
422 - 439	1905.9005	1904.8932	1904.8904	0.0028	0	**FHPDTDDVPVPAPAGDQK** (No match)
448 - 461	1671.9514	1670.9441	1670.9355	0.0086	1	**LLERLPEVEVPQHL** (No match)
452 - 461	1160.6239	1159.6166	1159.6237	-0.0070	0	**LPEVEVPQHL** (No match)

Spot 337

Protein View

Match to: **gi|190786** Score: **85** Expect: **0.00065**
prolyl 4-hydroxylase alpha subunit (EC 1.14.11.2)

Nominal mass (M_r): **61075**; Calculated pI value: **5.70**
NCBI BLAST search of gi|190786 against nr
Unformatted sequence string for pasting into other applications

Taxonomy: Homo sapiens

Fixed modifications: Carbamidomethyl (C)
Variable modifications: Oxidation (M)
Cleavage by Trypsin: cuts C-term side of KR unless next residue is P
Sequence Coverage: **6%**

Matched peptides shown in **Bold Red**

```
  1 MIWYILIIGI LLPQSLAHPG FFTSIGQMTD LIHTEKDLVT SLKDYIKAEE
 51 DKLEQIKKWA EKLDRLTSTA TKDPEGFVGH PVNAFKLMKR LNTEWSELEN
101 LVLKDMSDGF ISNLTIQRPV LSNDEDQVGA AKALLRLQDT YNLDTDTISK
151 GNLPGVKHKS FLTAEDCFEL GKVAYTEADY YHTELWMEQA LRQLDEGEIS
201 TIDKVSVLDY LSYAVYQQGD LDKALLLTKK LLELDPEHQR ANGNLKYFEY
251 IMAKEKDVNK SASDDQSDQK TTPKKKGVAV DYLPERQKYE MLCRGEGIKM
301 TPRRQKKLFC RYHDGNRNPK FILAPAKQED EWDKPRIIRF HDIISDAEIE
351 IVKDLAKPRL SRATVHDPET GKLTTAQYRV SKSAWLSGYE NPVVSRINMR
401 IQDLTGLDVS TAEELQVANY GVGGQYEPHF DFARKDEPDA FKELGTGNRI
451 ATWLFYMSDV SAGGATVFPE VGASVWPKKG TAVFWYNLFA SGEGDYSTRH
501 AACPVLVGNK WVSNKWLHER GQEFRRPCTL SELE
```

Residue Number Increasing Mass Decreasing Mass

Start - End	Observed	Mr(expt)	Mr(calc)	Delta	Miss	Sequence
340 - 359	2309.2209	2308.2136	2308.2426	-0.0290	1	**FHDIISDAEIEIVKDLAKPR** (No match)
340 - 359	2309.2209	2308.2136	2308.2426	-0.0290	1	**FHDIISDAEIEIVKDLAKPR** (Ions score 67)
363 - 379	1887.9424	1886.9351	1886.9486	-0.0135	1	**ATVHDPETGKLTTAQYR** (No match)
363 - 379	1887.9424	1886.9351	1886.9486	-0.0135	1	**ATVHDPETGKLTTAQYR** (No match)

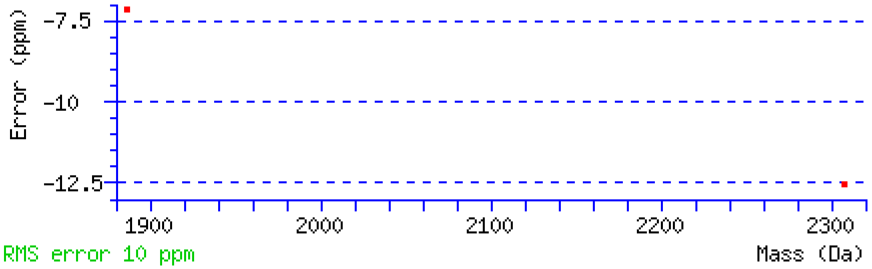

RMS error 10 ppm

Spot 338

Protein View

Match to: **gi|306890** Score: **413** Expect: **9.6e-037**
chaperonin (HSP60)

Nominal mass (M_r): **61157**; Calculated pI value: **5.70**
NCBI BLAST search of gi|306890 against nr
Unformatted sequence string for pasting into other applications

Taxonomy: Homo sapiens

Fixed modifications: Carbamidomethyl (C)
Variable modifications: Oxidation (M)
Cleavage by Trypsin: cuts C-term side of KR unless next residue is P
Sequence Coverage: **43%**

Matched peptides shown in **Bold Red**

```
  1 MLRLPTVFRQ MRPVSRVLAP HLTRAYAKDV KFGADARALM LQGVDLLADA
 51 VAVTMGPKGR TVIIEQGWGS PKVTKDGVTV AKSIDLKDKY KNIGAKLVQD
101 VANNTNEEAG DGTTTATVLA RSIAKEGFEK ISKGANPVEI RRGVMLAVDA
151 VIAELKKQSK PVTTPEEIAQ VATISANGDK EIGNIISDAM KKVGRKGVIT
201 VKDGKTLNDE LEIIEGMKFD RGYISPYFIN TSKGQKCEFQ DAYVLLSEKK
251 ISSIQSIVPA LEIANAHRKP LVIIAEDVDG EALSTLVLNR LKVGLQVVAV
301 KAPGFGDNRK NQLKDMAIAT GGAVFGEEGL TLNLEDVQPH DLGKVGEVIV
351 TKDDAMLLKG KGDKAQIEKR IQEIIEQLDV TTSEYEKEKL NERLAKLSDG
401 VAVLKVGGTS DVEVNEKKDR VTDALNATRA AVEEGIVLGG GCALLRCIPA
451 LDSLTPANED QKIGIEIIKR TLKIPAMTIA KNAGVEGSLI VEKIMQSSSE
501 VGYDAMAGDF VNMVEKGIID PTKVVRTALL DAAGVASLLT TAEVVVTEIP
551 KEEKDPGMGA MGGMGGGMGG GMF
```

Residue Number Increasing Mass Decreasing Mass

Start - End	Observed	Mr(expt)	Mr(calc)	Delta	Miss	Sequence
38 - 58	2145.2114	2144.2041	2144.1220	0.0821	0	ALMLQGVDLLADAVAVTMGPK 2 Oxidation (M) (No match)
97 - 121	2560.3225	2559.3152	2559.2412	0.0741	0	LVQDVANNTNEEAGDGTTTATVLAR (No match)
97 - 121	2560.3225	2559.3152	2559.2412	0.0741	0	LVQDVANNTNEEAGDGTTTATVLAR (Ions score 48)
134 - 141	855.4918	854.4845	854.4610	0.0236	0	GANPVEIR (No match)
134 - 142	1011.5931	1010.5858	1010.5621	0.0238	1	GANPVEIRR (No match)

143 - 157	1572.9565	1571.9492	1571.8956	0.0537	1	**GVMLAVDAVIAELKK** Oxidation (M) (No match)
206 - 221	1939.0168	1938.0095	1937.9404	0.0692	1	**TLNDELEIIEGMKFDR** Oxidation (M) (No match)
206 - 221	1939.0168	1938.0095	1937.9404	0.0692	1	**TLNDELEIIEGMKFDR** Oxidation (M) (Ions score 6)
222 - 233	1389.7469	1388.7396	1388.6976	0.0421	0	**GYISPYFINTSK** (Ions score 40)
222 - 233	1389.7469	1388.7396	1388.6976	0.0421	0	**GYISPYFINTSK** (No match)
237 - 249	1601.7986	1600.7913	1600.7442	0.0471	0	**CEFQDAYVLLSEK** (No match)
250 - 268	2047.2363	2046.2290	2046.1585	0.0705	1	**KISSIQSIVPALEIANAHR** (No match)
251 - 268	1919.1368	1918.1295	1918.0635	0.0660	0	**ISSIQSIVPALEIANAHR** (Ions score 39)
251 - 268	1919.1368	1918.1295	1918.0635	0.0660	0	**ISSIQSIVPALEIANAHR** (No match)
269 - 290	2365.4177	2364.4104	2364.3263	0.0841	0	**KPLVIIAEDVDGEALSTLVLNR** (No match)
302 - 309	833.4081	832.4008	832.3827	0.0181	0	**APGFGDNR** (No match)
302 - 310	961.5177	960.5104	960.4777	0.0328	1	**APGFGDNRK** (No match)
371 - 387	2038.0966	2037.0893	2037.0153	0.0740	0	**IQEIIEQLDVTTSEYEK** (No match)
371 - 389	2295.2524	2294.2451	2294.1529	0.0923	1	**IQEIIEQLDVTTSEYEKEK** (No match)
406 - 417	1233.6334	1232.6261	1232.5884	0.0377	0	**VGGTSDVEVNEK** (No match)
406 - 418	1361.7432	1360.7359	1360.6833	0.0526	1	**VGGTSDVEVNEKK** (No match)
421 - 429	960.5385	959.5312	959.5036	0.0277	0	**VTDALNATR** (No match)
430 - 446	1684.9633	1683.9560	1683.8977	0.0583	0	**AAVEEGIVLGGGCALLR** (Ions score 56)
430 - 446	1684.9633	1683.9560	1683.8977	0.0583	0	**AAVEEGIVLGGGCALLR** (No match)
463 - 470	941.6409	940.6336	940.6069	0.0267	1	**IGIEIIKR** (No match)
494 - 516	2556.2048	2555.1975	2555.0865	0.1110	0	**IMQSSSEVGYDAMAGDFVNMVEK** 3 Oxidation (M) (No match)

Spot 339

Protein View

Match to: **gi|24307939** Score: **262** Expect: **1.2e-021**
chaperonin containing TCP1, subunit 5 (epsilon) [Homo sapiens]

Nominal mass (M_r): **60089**; Calculated pI value: **5.45**
NCBI BLAST search of gi|24307939 against nr
Unformatted sequence string for pasting into other applications

Taxonomy: Homo sapiens
Links to retrieve other entries containing this sequence from NCBI Entrez:
gi|109076723 from Macaca mulatta
gi|114598964 from Pan troglodytes
gi|1351211 from Homo sapiens
gi|75076500 from Macaca fascicularis
gi|16306837 from Homo sapiens
gi|23273788 from Homo sapiens
gi|67969647 from Macaca fascicularis
gi|119628477 from Homo sapiens
gi|123984647 from synthetic construct
gi|123998627 from synthetic construct
gi|158259729 (no taxonomy information for this entry)

Fixed modifications: Carbamidomethyl (C)
Variable modifications: Oxidation (M)
Cleavage by Trypsin: cuts C-term side of KR unless next residue is P
Sequence Coverage: **32%**

Matched peptides shown in **Bold Red**

1 MASMGTLAFD EYGRPFLIIK DQDRKSRLMG LEALKSHIMA AKAVANTMRT
51 SLGPNGLDKM MVDKDGDVTV TNDGATILSM MDVDHQIAKL MVELSKSQDD
101 EIGDGTTGVV VLAGALLEEA EQLLDRGIHP IR**IADGYEQA** **AR**VAIEHLDK
151 **ISDSVLVDIK** **DTEPLIQTAK** TTLGSKVVNS CHR**QMAEIAV** **NAVLTVADME**
201 **R**R**DVDFELIK** VEGKVGGRLE DTKLIK**GVIV** **DKDFSHPQMP** **K**KVEDAK**IAI**
251 **LTCPFEPPKP** **K**TKHKLDVTS VEDYKALQKY EK**EKFEEMIQ** **QIK**ETGANLA
301 ICQWGFDDEA NHLLLQNNLP AVR**WVGGPEI** **ELIAIATGGR** IVPR**FSELTA**
351 **EKLGFAGLVQ** **EISFGTTK**DK MLVIEQCKNS R**AVTIFIR**GG NK**MIIEEAKR**
401 **SLHDALCVIR** NLIRDNRVVY GGGAAEISCA LAVSQEADKC PTLEQYAMRA
451 FADALEVIPM ALSENSGMNP IQTMTEVRAR QVKEMNPALG IDCLHKGTND
501 MKQQHVIETL IGK**KQQISLA** **TQMVR**MILKI DDIRKPGESE E

Residue Number Increasing Mass Decreasing Mass

Start - End	Observed	Mr(expt)	Mr(calc)	Delta	Miss	Sequence
133 - 142	1093.5370	1092.5297	1092.5199	0.0098	0	IADGYEQAAR (Ions score 23)
133 - 142	1093.5370	1092.5297	1092.5199	0.0098	0	IADGYEQAAR (No match)
151 - 170	2185.2307	2184.2234	2184.1889	0.0346	1	ISDSVLVDIKDTEPLIQTAK (No match)
184 - 201	1992.9996	1991.9923	1991.9655	0.0268	0	QMAEIAVNAVLTVADMER 2 Oxidation (M) (No match)
203 - 210	978.5098	977.5025	977.5069	-0.0044	0	DVDFELIK (No match)
227 - 241	1713.8850	1712.8777	1712.8555	0.0222	1	GVIVDKDFSHPQMPK Oxidation (M) (Ions score 11)
227 - 241	1713.8850	1712.8777	1712.8555	0.0222	1	GVIVDKDFSHPQMPK Oxidation (M) (No match)
248 - 261	1610.9153	1609.9080	1609.8901	0.0179	0	IAILTCPFEPPKPK (Ions score 26)
248 - 261	1610.9153	1609.9080	1609.8901	0.0179	0	IAILTCPFEPPKPK (No match)
283 - 293	1438.7363	1437.7290	1437.7173	0.0117	1	EKFEEMIQQIK Oxidation (M) (No match)
324 - 340	1738.9666	1737.9593	1737.9413	0.0181	0	WVGGPEIELIAIATGGR (No match)
345 - 352	924.4854	923.4781	923.4600	0.0182	0	FSELTAEK (No match)
353 - 368	1667.9176	1666.9103	1666.8929	0.0174	0	LGFAGLVQEISFGTTK (No match)
353 - 368	1667.9176	1666.9103	1666.8929	0.0174	0	LGFAGLVQEISFGTTK (Ions score 52)
382 - 388	819.5094	818.5021	818.5014	0.0008	0	AVTIFIR (No match)
393 - 400	1005.5344	1004.5271	1004.5324	-0.0053	1	MIIEEAKR Oxidation (M) (No match)
401 - 410	1183.6349	1182.6276	1182.6179	0.0097	0	SLHDALCVIR (No match)
514 - 525	1418.7915	1417.7842	1417.7711	0.0131	1	KQQISLATQMVR Oxidation (M) (No match)
515 - 525	1290.6925	1289.6852	1289.6761	0.0091	0	QQISLATQMVR Oxidation (M) (No match)

Spot 340

Protein View

Match to: **gi|4503015** Score: **93** Expect: **9.9e-005**
copine III [Homo sapiens]

Nominal mass (M_r): **60947;** Calculated pI value: **5.60**
NCBI BLAST search of gi|4503015 against nr
Unformatted sequence string for pasting into other applications

Taxonomy: Homo sapiens
Links to retrieve other entries containing this sequence from NCBI Entrez:
gi|10719946 from Homo sapiens
gi|5670328 from Homo sapiens
gi|42744574 from Homo sapiens
gi|119612047 from Homo sapiens
gi|119612048 from Homo sapiens

Fixed modifications: Carbamidomethyl (C)
Variable modifications: Oxidation (M)
Cleavage by Trypsin: cuts C-term side of KR unless next residue is P
Sequence Coverage: **8%**

Matched peptides shown in **Bold Red**

```
  1 MAAQCVTKVA LNVSCANLLD KDIGSKSDPL CVLFLNTSGQ QWYEVERTER
 51 IKNCLNPQFS KTFIIDYYFE VVQKLKFGVY DIDNKTIELS DDDFLGECEC
101 TLGQIVSSKK LTRPLVMKTG RPAGKGSITI SAEEIKDNRV VLFEMEARKL
151 DNKDLFGKSD PYLEFHKQTS DGNWLMVHRT EVVKNNLNPV WRPFKISLNS
201 LCYGDMDKTI KVECYDYDND GSHDLIGTFQ TTMTKLKEAS RSSPVEFECI
251 NEKKRQKKKS YKNSGVISVK QCEITVECTF LDYIMGGCQL NFTVGVDFTG
301 SNGDPRSPDS LHYISPNGVN EYLTALWSVG LVIQDYDADK MFPAFGFGAQ
351 IPPQWQVSHE FPMNFNPSNP YCNGIQGIVE AYRSCLPQIK LYGPTNFSPI
401 INHVARFAAA ATQQQTASQY FVLLIITDGV ITDLDETRQA IVNASRLPMS
451 IIIVGVGGAD FSAMEFLDGD GGSLRSPLGE VAIRDIVQFV PFRQFQNAPK
501 EALAQCVLAE IPQQVVGYFN TYKLLPPKNP ATKQQKQ
```

Residue Number Increasing Mass Decreasing Mass

Start - End	Observed	Mr(expt)	Mr(calc)	Delta	Miss	Sequence

126 - 139	1532.8273	1531.8200	1531.7841	0.0359	1	**GSITISAEEIKDNR**	(Ions score 10)
126 - 139	1532.8273	1531.8200	1531.7841	0.0359	1	**GSITISAEEIKDNR**	(No match)
140 - 148	1109.5894	1108.5821	1108.5586	0.0235	0	**VVLFEMEAR** Oxidation (M)	(No match)
140 - 148	1109.5894	1108.5821	1108.5586	0.0235	0	**VVLFEMEAR** Oxidation (M)	(Ions score 4)
391 - 406	1799.0050	1797.9977	1797.9525	0.0452	0	**LYGPTNFSPIINHVAR**	(No match)
391 - 406	1799.0050	1797.9977	1797.9525	0.0452	0	**LYGPTNFSPIINHVAR**	(Ions score 20)
485 - 493	1120.6411	1119.6338	1119.6076	0.0262	0	**DIVQFVPFR**	(Ions score 29)
485 - 493	1120.6411	1119.6338	1119.6076	0.0262	0	**DIVQFVPFR**	(No match)

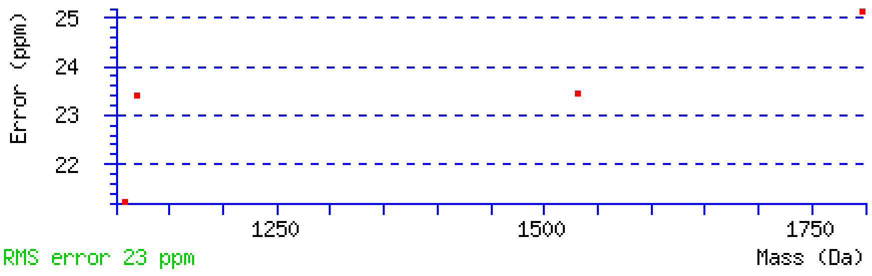

RMS error 23 ppm

Spot 341

Protein View

Match to: **gi|30240932** Score: **236** Expect: **4.8e-019**
EH-domain containing 1 [Homo sapiens]

Nominal mass (M_r): **60646;** Calculated pI value: **6.35**
NCBI BLAST search of gi|30240932 against nr
Unformatted sequence string for pasting into other applications

Taxonomy: Homo sapiens
Links to retrieve other entries containing this sequence from NCBI Entrez:
gi|75070798 (no taxonomy information for this entry)
gi|18202945 from Homo sapiens
gi|55728132 from Pongo pygmaeus
gi|85396897 from Homo sapiens
gi|85397978 from Homo sapiens
gi|119594724 from Homo sapiens
gi|119594725 from Homo sapiens
gi|119594726 from Homo sapiens

Fixed modifications: Carbamidomethyl (C)
Variable modifications: Oxidation (M)
Cleavage by Trypsin: cuts C-term side of KR unless next residue is P
Sequence Coverage: **26%**

Matched peptides shown in **Bold Red**

```
  1 MFSWVSKDAR RKKEPELFQT VAEGLRQLYA QKLLPLEEHY RFHEFHSPAL
 51 EDADFDNKPM VLLVGQYSTG KTTFIRHLIE QDFPGMRIGP EPTTDSFIAV
101 MHGPTEGVVP GNALVVDPRR PFRKLNAFGN AFLNRFMCAQ LPNPVLDSIS
151 IIDTPGILSG EKQRISRGYD FAAVLEWFAE RVDRIILLFD AHKLDISDEF
201 SEVIKALKNH EDKIRVVLNK ADQIETQQLM RVYGALMWSL GKIINTPEVV
251 RVYIGSFWSH PLLIPDNRKL FEAEEQDLFK DIQSLPRNAA LRKLNDLIKR
301 ARLAKVHAYI ISSLKKEMPN VFGKESKKKE LVNNLGEIYQ KIEREHQISP
351 GDFPSLRKMQ ELLQTQDFSK FQALKPKLLD TVDDMLANDI ARLMVMVRQE
401 ESLMPSQVVK GGAFDGTMNG PFGHGYGEGA GEGIDDVEWV VGKDKPTYDE
451 IFYTLSPVNG KITGANAKKE MVKSKLPNTV LGKIWKLADV DKDGLLDDEE
501 FALANHLIKV KLEGHELPAD LPPHLVPPSK RRHE
```

Residue Number Increasing Mass Decreasing Mass

Start - End	Observed	Mr(expt)	Mr(calc)	Delta	Miss	Sequence
14 - 26	1488.7231	1487.7158	1487.7619	-0.0461	0	EPELFQTVAEGLR (No match)
33 - 41	1169.5931	1168.5858	1168.6240	-0.0382	0	LLPLEEHYR (Ions score 17)
33 - 41	1169.5931	1168.5858	1168.6240	-0.0382	0	LLPLEEHYR (No match)
77 - 87	1342.6136	1341.6063	1341.6499	-0.0436	0	HLIEQDFPGMR (No match)
77 - 87	1342.6136	1341.6063	1341.6499	-0.0436	0	HLIEQDFPGMR (Ions score 17)
77 - 87	1358.6068	1357.5995	1357.6448	-0.0453	0	HLIEQDFPGMR Oxidation (M) (Ions score 6)
77 - 87	1358.6068	1357.5995	1357.6448	-0.0453	0	HLIEQDFPGMR Oxidation (M) (No match)
124 - 135	1364.7028	1363.6955	1363.7360	-0.0405	1	KLNAFGNAFLNR (No match)
125 - 135	1236.6132	1235.6059	1235.6410	-0.0351	0	LNAFGNAFLNR (Ions score 32)
125 - 135	1236.6132	1235.6059	1235.6410	-0.0351	0	LNAFGNAFLNR (No match)
243 - 251	1040.5823	1039.5750	1039.6025	-0.0275	0	IINTPEVVR (No match)
270 - 287	2178.0288	2177.0215	2177.1004	-0.0788	1	LFEAEEQDLFKDIQSLPR (No match)
342 - 357	1880.9031	1879.8958	1879.9540	-0.0582	1	IEREHQISPGDFPSLR (No match)
345 - 357	1482.6847	1481.6774	1481.7262	-0.0488	0	EHQISPGDFPSLR (Ions score 42)
345 - 357	1482.6847	1481.6774	1481.7262	-0.0488	0	EHQISPGDFPSLR (No match)
345 - 358	1610.7750	1609.7677	1609.8212	-0.0535	1	EHQISPGDFPSLRK (No match)
378 - 392	1674.7786	1673.7713	1673.8294	-0.0581	0	LLDTVDDMLANDIAR (No match)
378 - 392	1690.7679	1689.7606	1689.8243	-0.0637	0	LLDTVDDMLANDIAR Oxidation (M) (No match)
444 - 461	2086.9661	2085.9588	2086.0258	-0.0670	0	DKPTYDEIFYTLSPVNGK (No match)
510 - 530	2273.1938	2272.1865	2272.2579	-0.0713	1	VKLEGHELPADLPPHLVPPSK (No match)

Spot 342

Protein View

Match to: **gi|119594723** Score: **420** Expect: **1.9e-037**
EH-domain containing 1, isoform CRA_a [Homo sapiens]

Nominal mass (M_r): **61945**; Calculated pI value: **6.25**
NCBI BLAST search of gi|119594723 against nr
Unformatted sequence string for pasting into other applications

Taxonomy: Homo sapiens
Links to retrieve other entries containing this sequence from NCBI Entrez:
gi|119594727 from Homo sapiens

Fixed modifications: Carbamidomethyl (C)
Variable modifications: Oxidation (M)
Cleavage by Trypsin: cuts C-term side of KR unless next residue is P
Sequence Coverage: **30%**

Matched peptides shown in **Bold Red**

```
  1 MEQPGTAASP VSGSMFSWVS KDARRKKEPE LFQTVAEGLR QLYAQKLLPL
 51 EEHYRFHEFH SPALEDADFD NKPMVLLVGQ YSTGKTTFIR HLIEQDFPGM
101 RIGPEPTTDS FIAVMHGPTE GVVPGNALVV DPRRPFRKLN AFGNAFLNRF
151 MCAQLPNPVL DSISIIDTPG ILSGEKQRIS RGYDFAAVLE WFAERVDRII
201 LLFDAHKLDI SDEFSEVIKA LKNHEDKIRV VLNKADQIET QQLMRVYGAL
251 MWSLGKIINT PEVVRVYIGS FWSHPLLIPD NRKLFEAEEQ DLFKDIQSLP
301 RNAALRKLND LIKRARLAKV HAYIISSLKK EMPNVFGKES KKKELVNNLG
351 EIYQKIEREH QISPGDFPSL RKMQELLQTQ DFSKFQALKP KLLDTVDDML
401 ANDIARLMVM VRQEESLMPS QVVKGGAFDG TMNGPFGHGY GEGAGEGIDD
451 VEWVVGKDKP TYDEIFYTLS PVNGKITGAN AKKEMVKSKL PNTVLGKIWK
501 LADVDKDGLL DDEEFALANH LIKVKLEGHE LPADLPPHLV PPSKRRHE
```

Residue Number Increasing Mass Decreasing Mass

Start - End	Observed	Mr(expt)	Mr(calc)	Delta	Miss	Sequence
27 - 40	1616.7972	1615.7899	1615.8569	-0.0670	1	KEPELFQTVAEGLR (No match)
28 - 40	1488.7056	1487.6983	1487.7619	-0.0636	0	EPELFQTVAEGLR (No match)
41 - 55	1900.9833	1899.9760	1900.0206	-0.0446	1	QLYAQKLLPLEEHYR (No match)
47 - 55	1169.5818	1168.5745	1168.6240	-0.0495	0	LLPLEEHYR (Ions score 45)

47 - 55	1169.5818	1168.5745	1168.6240	-0.0495	0	**LLPLEEHYR** (No match)
91 - 101	1358.5862	1357.5789	1357.6448	-0.0659	0	**HLIEQDFPGMR** Oxidation (M) (No match)
138 - 149	1364.6863	1363.6790	1363.7360	-0.0570	1	**KLNAFGNAFLNR** (Ions score 42)
138 - 149	1364.6863	1363.6790	1363.7360	-0.0570	1	**KLNAFGNAFLNR** (No match)
139 - 149	1236.5984	1235.5911	1235.6410	-0.0499	0	**LNAFGNAFLNR** (No match)
139 - 149	1236.5984	1235.5911	1235.6410	-0.0499	0	**LNAFGNAFLNR** (Ions score 78)
235 - 245	1348.5934	1347.5861	1347.6452	-0.0591	0	**ADQIETQQLMR** Oxidation (M) (No match)
235 - 256	2554.2146	2553.2073	2553.2719	-0.0646	1	**ADQIETQQLMRVYGALMWSLGK** Oxidation (M) (No match)
257 - 265	1040.5663	1039.5590	1039.6025	-0.0435	0	**IINTPEVVR** (No match)
284 - 294	1368.6143	1367.6070	1367.6608	-0.0538	0	**LFEAEEQDLFK** (No match)
284 - 301	2178.0200	2177.0127	2177.1004	-0.0876	1	**LFEAEEQDLFKDIQSLPR** (No match)
359 - 371	1482.6714	1481.6641	1481.7262	-0.0621	0	**EHQISPGDFPSLR** (Ions score 52)
359 - 371	1482.6714	1481.6641	1481.7262	-0.0621	0	**EHQISPGDFPSLR** (No match)
359 - 372	1610.7639	1609.7566	1609.8212	-0.0646	1	**EHQISPGDFPSLRK** (No match)
392 - 406	1690.7517	1689.7444	1689.8243	-0.0799	0	**LLDTVDDMLANDIAR** Oxidation (M) (No match)
458 - 475	2086.9512	2085.9439	2086.0258	-0.0819	0	**DKPTYDEIFYTLSPVNGK** (No match)
526 - 545	2202.1138	2201.1065	2201.1956	-0.0891	1	**LEGHELPADLPPHLVPPSKR** (No match)

Spot 343

Protein View

Match to: **gi|67464392** Score: **480** Expect: **1.9e-043**
Chain A, Structure Of Human Muscle Pyruvate Kinase (Pkm2)

Nominal mass (M_r): **60277**; Calculated pI value: **8.22**
NCBI BLAST search of gi|67464392 against nr
Unformatted sequence string for pasting into other applications

Taxonomy: Homo sapiens

Fixed modifications: Carbamidomethyl (C)
Variable modifications: Oxidation (M)
Cleavage by Trypsin: cuts C-term side of KR unless next residue is P
Sequence Coverage: **48%**

Matched peptides shown in **Bold Red**

```
  1 MGSSHHHHHH SSGLVPRGSK PHSEAGTAFI QTQQLHAAMA DTFLEHMCRL
 51 DIDSPPITAR NTGIICTIGP ASRSVETLKE MIKSGMNVAR LNFSHGTHEY
101 HAETIKNVRT ATESFASDPI LYRPVAVALD TKGPEIRTGL IKGSGTAEVE
151 LKKGATLKIT LDNAYMEKCD ENILWLDYKN ICKVVEVGSK IYVDDGLISL
201 QVKQKGADFL VTEVENGGSL GSKKGVNLPG AAVDLPAVSE KDIQDLKFGV
251 EQDVDMVFAS FIRKASDVHE VRKVLGEKGK NIKIISKIEN HEGVRRFDEI
301 LEASDGIMVA RGDLGIEIPA EKVFLAQKMM IGRCNRAGKP VICATQMLES
351 MIKKPRPTRA EGSDVANAVL DGADCIMLSG ETAKGDYPLE AVRMQHLIAR
401 EAEAAIYHLQ LFEELRRLAP ITSDPTEATA VGAVEASFKC CSGAIIVLTK
451 SGRSAHQVAR YRPRAPIIAV TRNPQTARQA HLYRGIFPVL CKDPVQEAWA
501 EDVDLRVNFA MNVGKARGFF KKGDVVIVLT GWRPGSGFTN TMRVVPVP
```

Residue Number Increasing Mass Decreasing Mass

Start - End	Observed	Mr(expt)	Mr(calc)	Delta	Miss	Sequence
50 - 60	1197.6346	1196.6273	1196.6401	-0.0128	0	LDIDSPPITAR (Ions score 51)
50 - 60	1197.6346	1196.6273	1196.6401	-0.0128	0	LDIDSPPITAR (No match)
61 - 73	1359.6962	1358.6889	1358.6976	-0.0087	0	NTGIICTIGPASR (No match)
61 - 73	1359.6962	1358.6889	1358.6976	-0.0087	0	NTGIICTIGPASR (Ions score 41)
91 - 106	1883.8850	1882.8777	1882.8961	-0.0184	0	LNFSHGTHEYHAETIK (No match)
91 - 106	1883.8850	1882.8777	1882.8961	-0.0184	0	LNFSHGTHEYHAETIK (Ions score 97)

110 - 132	2465.2678	2464.2605	2464.2849	-0.0244	0	**TATESFASDPILYRPVAVALDTK** (Ions score 11)
110 - 132	2465.2678	2464.2605	2464.2849	-0.0244	0	**TATESFASDPILYRPVAVALDTK** (No match)
110 - 137	3017.5723	3016.5650	3016.5869	-0.0218	1	**TATESFASDPILYRPVAVALDTKGPEIR** (No match)
169 - 179	1468.6563	1467.6490	1467.6704	-0.0214	0	**CDENILWLDYK** (No match)
191 - 203	1462.8003	1461.7930	1461.8078	-0.0148	0	**IYVDDGLISLQVK** (No match)
206 - 223	1779.8624	1778.8551	1778.8685	-0.0134	0	**GADFLVTEVENGGSLGSK** (No match)
225 - 247	2349.2556	2348.2483	2348.2586	-0.0103	1	**GVNLPGAAVDLPAVSEKDIQDLK** (No match)
248 - 263	1859.8834	1858.8761	1858.8923	-0.0162	0	**FGVEQDVDMVFASFIR** (No match)
248 - 263	1875.8787	1874.8714	1874.8872	-0.0158	0	**FGVEQDVDMVFASFIR** Oxidation (M) (No match)
288 - 295	953.4693	952.4620	952.4726	-0.0106	0	**IENHEGVR** (No match)
296 - 311	1837.8976	1836.8903	1836.9039	-0.0136	1	**RFDEILEASDGIMVAR** Oxidation (M) (No match)
312 - 322	1141.5966	1140.5893	1140.6026	-0.0133	0	**GDLGIEIPAEK** (No match)
337 - 353	1892.9491	1891.9418	1891.9569	-0.0151	0	**AGKPVICATQMLESMIK** Oxidation (M) (No match)
337 - 353	1908.9500	1907.9427	1907.9518	-0.0091	0	**AGKPVICATQMLESMIK** 2 Oxidation (M) (No match)
385 - 393	1019.5054	1018.4981	1018.5083	-0.0102	0	**GDYPLEAVR** (No match)
394 - 400	868.5040	867.4967	867.4749	0.0219	0	**MQHLIAR** (No match)
394 - 400	884.4864	883.4791	883.4698	0.0094	0	**MQHLIAR** Oxidation (M) (No match)
401 - 416	1931.9681	1930.9608	1930.9788	-0.0180	0	**EAEAAIYHLQLFEELR** (No match)
401 - 417	2088.0679	2087.0606	2087.0799	-0.0193	1	**EAEAAIYHLQLFEELRR** (No match)
401 - 417	2088.0679	2087.0606	2087.0799	-0.0193	1	**EAEAAIYHLQLFEELRR** (Ions score 40)
418 - 439	2175.1069	2174.0996	2174.1106	-0.0110	0	**LAPITSDPTEATAVGAVEASFK** (No match)
465 - 472	840.5217	839.5144	839.5228	-0.0084	0	**APIIAVTR** (No match)

Spot 344

Protein View

Match to: **gi|4827050** Score: **115** Expect: **6.1e-007**
ubiquitin specific protease 14 isoform a [Homo sapiens]

Nominal mass (M_r): **56489**; Calculated pI value: **5.20**
NCBI BLAST search of gi|4827050 against nr
Unformatted sequence string for pasting into other applications

Taxonomy: Homo sapiens
Links to retrieve other entries containing this sequence from NCBI Entrez:
gi|1729927 from Homo sapiens
gi|940182 from Homo sapiens
gi|13097696 from Homo sapiens
gi|30583205 from Homo sapiens
gi|60655633 from synthetic construct
gi|60655635 from synthetic construct
gi|123994173 from synthetic construct
gi|124126877 from synthetic construct

Fixed modifications: Carbamidomethyl (C)
Variable modifications: Oxidation (M)
Cleavage by Trypsin: cuts C-term side of KR unless next residue is P
Sequence Coverage: **12%**

Matched peptides shown in **Bold Red**

```
  1 MPLYSVTVKW GKEKFEGVEL NTDEPPMVFK AQLFALTGVQ PARQKVMVKG
 51 GTLKDDDWGN IKIKNGMTLL MMGSADALPE EPSAKTVFVE DMTEEQLASA
101 MELPCGLTNL GNTCYMNATV QCIRSVPELK DALKRYAGAL RASGEMASAQ
151 YITAALRDLF DSMDKTSSSI PPIILLQFLH MAFPQFAEKG EQGQYLQQDA
201 NECWIQMMRV LQQKLEAIED DSVKETDSSS ASAATPSKKK SLIDQFFGVE
251 FETTMKCTES EEEEVTKGKE NQLQLSCFIN QEVKYLFTGL KLRLQEEITK
301 QSPTLQRNAL YIKSSKISRL PAYLTIQMVR FFYKEKESVN AKVLKDVKFP
351 LMLDMYELCT PELQEKMVSF RSKFKDLEDK KVNQQPNTSD KKSSPQKEVK
401 YEPFSFADDI GSNNCGYYDL QAVLTHQGRS SSSGHYVSWV KRKQDEWIKF
451 DDDKVSIVTP EDILRLSGGG DWHIAYVLLY GPRRVEIMEE ESEQ
```

Residue Number Increasing Mass Decreasing Mass

Start - End	Observed	Mr(expt)	Mr(calc)	Delta	Miss	Sequence
31 - 43	1371.7622	1370.7549	1370.7670	-0.0120	0	**AQLFALTGVQPAR** (Ions score 69)
31 - 43	1371.7622	1370.7549	1370.7670	-0.0120	0	**AQLFALTGVQPAR** (No match)
65 - 85	2211.0938	2210.0865	2209.9904	0.0961	0	**NGMTLLMMGSADALPEEPSAK** 3 Oxidation (M) (No match)
320 - 330	1320.7205	1319.7132	1319.7271	-0.0139	0	**LPAYLTIQMVR** Oxidation (M) (No match)
320 - 330	1320.7205	1319.7132	1319.7271	-0.0139	0	**LPAYLTIQMVR** Oxidation (M) (No match)
466 - 483	1974.0081	1973.0008	1973.0158	-0.0150	0	**LSGGGDWHIAYVLLYGPR** (No match)

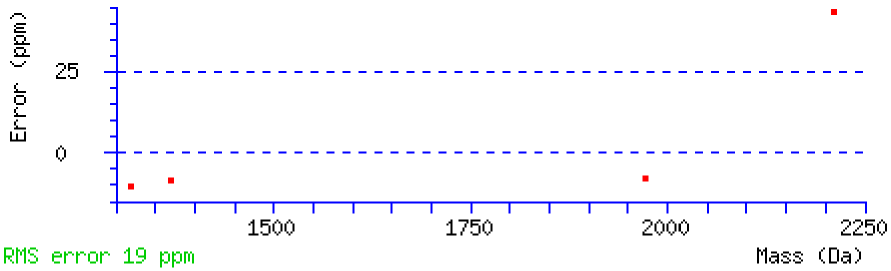

RMS error 19 ppm

Spot 345

Protein View

Match to: **gi|20070125** Score: **519** Expect: **2.4e-047**
prolyl 4-hydroxylase, beta subunit precursor [Homo sapiens]

Nominal mass (M_r): **57480**; Calculated pI value: **4.76**
NCBI BLAST search of gi|20070125 against nr
Unformatted sequence string for pasting into other applications

Taxonomy: Homo sapiens
Links to retrieve other entries containing this sequence from NCBI Entrez:
gi|2507460 from Homo sapiens
gi|190384 from Homo sapiens
gi|14790033 from Homo sapiens
gi|20810352 from Homo sapiens
gi|48735337 from Homo sapiens
gi|119610096 from Homo sapiens
gi|119610098 from Homo sapiens
gi|119610101 from Homo sapiens

Fixed modifications: Carbamidomethyl (C)
Variable modifications: Oxidation (M)
Cleavage by Trypsin: cuts C-term side of KR unless next residue is P
Sequence Coverage: **45%**

Matched peptides shown in **Bold Red**

```
  1 MLRRALLCLA VAALVRADAP EEEDHVLVLR KSNFAEALAA HKYLLVEFYA
 51 PWCGHCKALA PEYAKAAGKL KAEGSEIRLA KVDATEESDL AQQYGVRGYP
101 TIKFFRNGDT ASPKEYTAGR EADDIVNWLK KRTGPAATTL PDGAAAESLV
151 ESSEVAVIGF FKDVESDSAK QFLQAAEAID DIPFGITSNS DVFSKYQLDK
201 DGVVLFKKFD EGRNNFEGEV TKENLLDFIK HNQLPLVIEF TEQTAPKIFG
251 GEIKTHILLF LPKSVSDYDG KLSNFKTAAE SFKGKILFIF IDSDHTDNQR
301 ILEFFGLKKE ECPAVRLITL EEEMTKYKPE SEELTAERIT EFCHRFLEGK
351 IKPHLMSQEL PEDWDKQPVK VLVGKNFEDV AFDEKKNVFV EFYAPWCGHC
401 KQLAPIWDKL GETYKDHENI VIAKMDSTAN EVEAVKVHSF PTLKFFPASA
451 DRTVIDYNGE RTLDGFKKFL ESGGQDGAGD DDDLEDLEEA EEPDMEEDDD
501 QKAVKDEL
```

Residue Number Increasing Mass Decreasing Mass

Start - End	Observed	Mr(expt)	Mr(calc)	Delta	Miss	Sequence
31 - 42	1286.6719	1285.6646	1285.6778	-0.0132	1	KSNFAEALAAHK (No match)
32 - 42	1158.5782	1157.5709	1157.5829	-0.0119	0	SNFAEALAAHK (No match)
70 - 78	1002.5483	1001.5410	1001.5505	-0.0095	1	LKAEGSEIR (No match)
82 - 97	1780.8131	1779.8058	1779.8275	-0.0216	0	VDATEESDLAQQYGVR (Ions score 96)
82 - 97	1780.8131	1779.8058	1779.8275	-0.0216	0	VDATEESDLAQQYGVR (No match)
163 - 170	850.3723	849.3650	849.3716	-0.0065	0	DVESDSAK (No match)
196 - 207	1424.7635	1423.7562	1423.7710	-0.0148	1	YQLDKDGVVLFK (No match)
208 - 213	751.3708	750.3635	750.3660	-0.0025	1	KFDEGR (No match)
209 - 222	1641.7256	1640.7183	1640.7430	-0.0246	1	FDEGRNNFEGEVTK (No match)
214 - 230	2009.9945	2008.9872	2009.0105	-0.0232	1	NNFEGEVTKENLLDFIK (No match)
231 - 247	1965.0266	1964.0193	1964.0367	-0.0173	0	HNQLPLVIEFTEQTAPK (No match)
248 - 254	763.4216	762.4143	762.4275	-0.0132	0	IFGGEIK (No match)
255 - 263	1081.6593	1080.6520	1080.6695	-0.0175	0	THILLFLPK (No match)
264 - 271	870.3735	869.3662	869.3766	-0.0104	0	SVSDYDGK (No match)
286 - 300	1833.8925	1832.8852	1832.9057	-0.0204	0	ILFIFIDSDHTDNQR (Ions score 66)
286 - 300	1833.8925	1832.8852	1832.9057	-0.0204	0	ILFIFIDSDHTDNQR (No match)
301 - 308	966.5519	965.5446	965.5585	-0.0139	0	ILEFFGLK (No match)
301 - 309	1094.6479	1093.6406	1093.6535	-0.0129	1	ILEFFGLKK (No match)
309 - 316	988.5078	987.5005	987.4807	0.0198	1	KEECPAVR (No match)
317 - 326	1206.5938	1205.5865	1205.6213	-0.0348	0	LITLEEEMTK (No match)
317 - 326	1222.6143	1221.6070	1221.6162	-0.0092	0	LITLEEEMTK Oxidation (M) (No match)
327 - 338	1451.6865	1450.6792	1450.6939	-0.0147	0	YKPESEELTAER (No match)
327 - 338	1451.6865	1450.6792	1450.6939	-0.0147	0	YKPESEELTAER (Ions score 42)
339 - 345	962.4466	961.4393	961.4440	-0.0046	0	ITEFCHR (No match)
376 - 385	1213.5233	1212.5160	1212.5298	-0.0138	0	NFEDVAFDEK (No match)
376 - 386	1341.6149	1340.6076	1340.6248	-0.0171	1	NFEDVAFDEKK (No match)
410 - 424	1729.8936	1728.8863	1728.9046	-0.0182	1	LGETYKDHENIVIAK (No match)
437 - 444	928.5146	927.5073	927.5178	-0.0104	0	VHSFPTLK (No match)
445 - 452	910.4369	909.4296	909.4344	-0.0048	0	FFPASADR (No match)
453 - 461	1066.5089	1065.5016	1065.5090	-0.0074	0	TVIDYNGER (No match)
453 - 461	1066.5089	1065.5016	1065.5090	-0.0074	0	TVIDYNGER (Ions score 8)

Spot 346

Protein View

Match to: **gi|67464392** Score: **677** Expect: **3.8e-063**
Chain A, Structure Of Human Muscle Pyruvate Kinase (Pkm2)

Nominal mass (M_r): **60277**; Calculated pI value: **8.22**
NCBI BLAST search of gi|67464392 against nr
Unformatted sequence string for pasting into other applications

Taxonomy: Homo sapiens

Fixed modifications: Carbamidomethyl (C)
Variable modifications: Oxidation (M)
Cleavage by Trypsin: cuts C-term side of KR unless next residue is P
Sequence Coverage: **62%**

Matched peptides shown in **Bold Red**

1 MGSSHHHHHH SSGLVPRGSK PHSEAGTAFI QTQQLHAAMA DTFLEHMCR**L**
51 **DIDSPPITAR NTGIICTIGP ASR**SVETLKE MIKSGMNVAR **LNFSHGTHEY**
101 **HAETIK**NVR**T ATESFASDPI LYRPVAVALD TKGPEIR**TGL IKGSGTAEVE
151 LKKGATLK**IT LDNAYMEKCD ENILWLDYK**N ICKVVEVGSK **IYVDDGLISL**
201 **QVK**QK**GADFL VTEVENGGSL GSK**K**GVNLPG AAVDLPAVSE KDIQDLKFGV**
251 **EQDVDMVFAS FIR**K**ASDVHE VRK**VLGEKGK NIKIISK**IEN HEGVRRFDEI**
301 **LEASDGIMVA RGDLGIEIPA EK**VFLAQKMM IGRCNR**AGKP VICATQMLES**
351 **MIK**KPRPTR**A EGSDVANAVL DGADCIMLSG ETAKGDYPLE AVRMQHLIAR**
401 **EAEAAIYHLQ LFEELRRLAP ITSDPTEATA VGAVEASFK**C CSGAIIVLTK
451 SGRSAHQVAR YRPR**APIIAV TR**NPQTARQA HLYRGIFPVL CK**DPVQEAWA**
501 **EDVDLR**VNFA MNVGKARGFF K**KGDVVIVLT GWRPGSGFTN TMR**VVPVP

Residue Number Increasing Mass Decreasing Mass

Start - End	Observed	Mr(expt)	Mr(calc)	Delta	Miss	Sequence
50 - 60	1197.6315	1196.6242	1196.6401	-0.0159	0	LDIDSPPITAR (No match)
50 - 60	1197.6315	1196.6242	1196.6401	-0.0159	0	LDIDSPPITAR (Ions score 68)
61 - 73	1359.6908	1358.6835	1358.6976	-0.0141	0	NTGIICTIGPASR (No match)
61 - 73	1359.6908	1358.6835	1358.6976	-0.0141	0	NTGIICTIGPASR (Ions score 73)
91 - 106	1883.8785	1882.8712	1882.8961	-0.0249	0	LNFSHGTHEYHAETIK (Ions score 111)
91 - 106	1883.8785	1882.8712	1882.8961	-0.0249	0	LNFSHGTHEYHAETIK (No match)

110 - 132	2465.2598	2464.2525	2464.2849	-0.0324	0	**TATESFASDPILYRPVAVALDTK** (Ions score 37)
110 - 132	2465.2598	2464.2525	2464.2849	-0.0324	0	**TATESFASDPILYRPVAVALDTK** (No match)
110 - 137	3017.5747	3016.5674	3016.5869	-0.0194	1	**TATESFASDPILYRPVAVALDTKGPEIR** (No match)
159 - 168	1213.5724	1212.5651	1212.5696	-0.0045	0	**ITLDNAYMEK** Oxidation (M) (No match)
169 - 179	1468.6576	1467.6503	1467.6704	-0.0201	0	**CDENILWLDYK** (No match)
191 - 203	1462.7971	1461.7898	1461.8078	-0.0180	0	**IYVDDGLISLQVK** (No match)
206 - 223	1779.8518	1778.8445	1778.8685	-0.0240	0	**GADFLVTEVENGGSLGSK** (No match)
225 - 247	2349.2512	2348.2439	2348.2586	-0.0147	1	**GVNLPGAAVDLPAVSEKDIQDLK** (No match)
248 - 263	1859.8734	1858.8661	1858.8923	-0.0262	0	**FGVEQDVDMVFASFIR** (No match)
248 - 263	1875.8700	1874.8627	1874.8872	-0.0245	0	**FGVEQDVDMVFASFIR** Oxidation (M) (No match)
265 - 273	1040.5377	1039.5304	1039.5410	-0.0106	1	**ASDVHEVRK** (No match)
288 - 295	953.4691	952.4618	952.4726	-0.0108	0	**IENHEGVR** (No match)
296 - 311	1821.8905	1820.8832	1820.9090	-0.0258	1	**RFDEILEASDGIMVAR** (No match)
296 - 311	1837.8920	1836.8847	1836.9039	-0.0192	1	**RFDEILEASDGIMVAR** Oxidation (M) (No match)
297 - 311	1681.7872	1680.7799	1680.8028	-0.0229	0	**FDEILEASDGIMVAR** Oxidation (M) (No match)
312 - 322	1141.5929	1140.5856	1140.6026	-0.0170	0	**GDLGIEIPAEK** (No match)
337 - 353	1876.9012	1875.8939	1875.9620	-0.0681	0	**AGKPVICATQMLESMIK** (No match)
337 - 353	1892.9415	1891.9342	1891.9569	-0.0227	0	**AGKPVICATQMLESMIK** Oxidation (M) (No match)
337 - 353	1908.9404	1907.9331	1907.9518	-0.0187	0	**AGKPVICATQMLESMIK** 2 Oxidation (M) (No match)
360 - 384	2494.1345	2493.1272	2493.1362	-0.0090	0	**AEGSDVANAVLDGADCIMLSGETAK** (No match)
385 - 393	1019.5036	1018.4963	1018.5083	-0.0120	0	**GDYPLEAVR** (No match)
394 - 400	868.4888	867.4815	867.4749	0.0067	0	**MQHLIAR** (No match)
394 - 400	884.4713	883.4640	883.4698	-0.0057	0	**MQHLIAR** Oxidation (M) (No match)
401 - 416	1931.9614	1930.9541	1930.9788	-0.0247	0	**EAEAAIYHLQLFEELR** (No match)
401 - 417	2088.0603	2087.0530	2087.0799	-0.0269	1	**EAEAAIYHLQLFEELRR** (Ions score 83)
401 - 417	2088.0603	2087.0530	2087.0799	-0.0269	1	**EAEAAIYHLQLFEELRR** (No match)
418 - 439	2175.0991	2174.0918	2174.1106	-0.0188	0	**LAPITSDPTEATAVGAVEASFK** (No match)
465 - 472	840.5223	839.5150	839.5228	-0.0078	0	**APIIAVTR** (No match)
493 - 506	1642.7510	1641.7437	1641.7634	-0.0197	0	**DPVQEAWAEDVDLR** (No match)
522 - 543	2391.2339	2390.2266	2390.2528	-0.0262	1	**KGDVVIVLTGWRPGSGFTNTMR** (No match)

Spot 347

Protein View

Match to: **gi|119598292** Score: **335** Expect: **6.1e-029**
pyruvate kinase, muscle, isoform CRA_c [Homo sapiens]

Nominal mass (M_r): **60773;** Calculated pI value: **7.95**
NCBI BLAST search of gi|119598292 against nr
Unformatted sequence string for pasting into other applications

Taxonomy: Homo sapiens

Fixed modifications: Carbamidomethyl (C)
Variable modifications: Oxidation (M)
Cleavage by Trypsin: cuts C-term side of KR unless next residue is P
Sequence Coverage: **32%**

Matched peptides shown in **Bold Red**

```
  1 MTQPFNYSAF ERIIFAGTSA AMSKPHSEAG TAFIQTQQLH AAMADTFLEH
 51 MCRLDIDSPP ITARNTGIIC TIGPASRSVE TLKEMIKSGM NVARLNFSHG
101 THEYHAETIK NVRTATESFA SDPILYRPVA VALDTKGPEI RTGLIKGSGT
151 AEVELKKGAT LKITLDNAYM EKCDENILWL DYKNICKVVE VGSKIYVDDG
201 LISLQVKQKG ADFLVTEVEN GGSLGSKKGV NLPGAAVDLP AVSEKDIQDL
251 KFGVEQDVDM VFASFIRKAS DVHEVRKVLG EKGKNIKIIS KIENHEGVRR
301 FDEILEASDG IMVARGDLGI EIPAEKVFLA QKMMIGRCNR AGKPVICATQ
351 MLESMIKKPR PTRAEGSDVA NAVLDGADCI MLSGETAKGD YPLEAVRMQH
401 LIAREAEAAI YHLQLFEELR RLAPITSDPT EATAVGAVEA SFKCCSGAII
451 VLTKSGRSAH QVARYRPRAP IIAVTRNPQT ARQAHLYRGI FPVLCKDPVQ
501 EAWAEDVDLR VNFAMNVGKA RGFFKKGDVV IVLTGWRPGS GFTNTMRVVP
551 VP
```

Residue Number Increasing Mass Decreasing Mass

Start - End	Observed	Mr(expt)	Mr(calc)	Delta	Miss	Sequence
2 - 12	1359.6566	1358.6493	1358.6255	0.0239	0	TQPFNYSAFER (No match)
54 - 64	1197.6093	1196.6020	1196.6401	-0.0381	0	LDIDSPPITAR (Ions score 46)
65 - 77	1359.6566	1358.6493	1358.6976	-0.0483	0	NTGIICTIGPASR (Ions score 31)
95 - 110	1883.8214	1882.8141	1882.8961	-0.0820	0	LNFSHGTHEYHAETIK (Ions score 26)
95 - 110	1883.8214	1882.8141	1882.8961	-0.0820	0	LNFSHGTHEYHAETIK (No match)

114 - 136	2465.1765	2464.1692	2464.2849	-0.1157	0	TATESFASDPILYRPVAVALDTK (Ions score 19)
114 - 136	2465.1765	2464.1692	2464.2849	-0.1157	0	TATESFASDPILYRPVAVALDTK (No match)
163 - 172	1197.6093	1196.6020	1196.5747	0.0273	0	ITLDNAYMEK (No match)
195 - 207	1462.7654	1461.7581	1461.8078	-0.0497	0	IYVDDGLISLQVK (No match)
210 - 227	1779.8032	1778.7959	1778.8685	-0.0726	0	GADFLVTEVENGGSLGSK (No match)
292 - 299	953.4454	952.4381	952.4726	-0.0345	0	IENHEGVR (No match)
300 - 315	1837.8372	1836.8299	1836.9039	-0.0740	1	RFDEILEASDGIMVAR Oxidation (M) (No match)
389 - 397	1019.4901	1018.4828	1018.5083	-0.0255	0	GDYPLEAVR (No match)
405 - 420	1931.9066	1930.8993	1930.9788	-0.0795	0	EAEAAIYHLQLFEELR (No match)
405 - 421	2087.9924	2086.9851	2087.0799	-0.0948	1	EAEAAIYHLQLFEELRR (No match)
405 - 421	2087.9924	2086.9851	2087.0799	-0.0948	1	EAEAAIYHLQLFEELRR (Ions score 38)
469 - 476	840.5112	839.5039	839.5228	-0.0189	0	APIIAVTR (No match)
483 - 488	787.4067	786.3994	786.4136	-0.0142	0	QAHLYR (No match)

Spot 348

Protein View

Match to: **gi|20070125** Score: **653** Expect: **9.6e-061**
prolyl 4-hydroxylase, beta subunit precursor [Homo sapiens]

Nominal mass (M_r): **57480**; Calculated pI value: **4.76**
NCBI BLAST search of gi|20070125 against nr
Unformatted sequence string for pasting into other applications

Taxonomy: Homo sapiens
Links to retrieve other entries containing this sequence from NCBI Entrez:
gi|2507460 from Homo sapiens
gi|190384 from Homo sapiens
gi|14790033 from Homo sapiens
gi|20810352 from Homo sapiens
gi|48735337 from Homo sapiens
gi|119610096 from Homo sapiens
gi|119610098 from Homo sapiens
gi|119610101 from Homo sapiens

Fixed modifications: Carbamidomethyl (C)
Variable modifications: Oxidation (M)
Cleavage by Trypsin: cuts C-term side of KR unless next residue is P
Sequence Coverage: **57%**

Matched peptides shown in **Bold Red**

1 MLRRALLCLA VAALVRADAP EEEDHVLVLR **KSNFAEALAA HK**YLLVEFYA
51 PWCGHCK**ALA PEYAK**AAGK**L KAEGSEIR**LA K**VDATEESDL AQQYGVR**GYP
101 TIKFFRNGDT ASPKEYTAGR EADDIVNWLK KR**TGPAATTL PDGAAAESLV**
151 **ESSEVAVIGF FKDVESDSAK** QFLQAAEAID DIPFGITSNS DVFSK**YQLDK**
201 **DGVVLFKKFD EGRNNFEGEV TKENLLDFIK HNQLPLVIEF TEQTAPKIFG**
251 **GEIKTHILLF LPKSVSDYDG K**LSNFKTAAE SFK**GKILFIF IDSDHTDNQR**
301 **ILEFFGLKKE ECPAVRLITL EEEMTKYKPE SEELTAERIT EFCHR**FLEGK
351 **IKPHLMSQEL PEDWDKQPVK** VLVGK**NFEDV AFDEKK**NVFV EFYAPWCGHC
401 KQLAPIWDK**L GETYKDHENI VIAK**MDSTAN EVEAVK**VHSF PTLKFFPASA**
451 **DRTVIDYNGE R**TLDGFKKFL ESGGQDGAGD DDDLEDLEEA EEPDMEEDDD
501 QKAVKDEL

Residue Number Increasing Mass Decreasing Mass

Start - End	Observed	Mr(expt)	Mr(calc)	Delta	Miss	Sequence
31 - 42	1286.6685	1285.6612	1285.6778	-0.0166	1	KSNFAEALAAHK (No match)
32 - 42	1158.5774	1157.5701	1157.5829	-0.0127	0	SNFAEALAAHK (No match)
58 - 65	862.4541	861.4468	861.4596	-0.0127	0	ALAPEYAK (No match)
70 - 78	1002.5441	1001.5368	1001.5505	-0.0137	1	LKAEGSEIR (Ions score 37)
70 - 78	1002.5441	1001.5368	1001.5505	-0.0137	1	LKAEGSEIR (No match)
72 - 78	761.3715	760.3642	760.3715	-0.0073	0	AEGSEIR (No match)
82 - 97	1780.8148	1779.8075	1779.8275	-0.0199	0	VDATEESDLAQQYGVR (No match)
82 - 97	1780.8148	1779.8075	1779.8275	-0.0199	0	VDATEESDLAQQYGVR (Ions score 104)
133 - 162	2935.4768	2934.4695	2934.4861	-0.0166	0	TGPAATTLPDGAAAESLVESSEVAVIGFFK (No match)
163 - 170	850.3703	849.3630	849.3716	-0.0085	0	DVESDSAK (No match)
196 - 207	1424.7646	1423.7573	1423.7710	-0.0137	1	YQLDKDGVVLFK (No match)
208 - 213	751.3725	750.3652	750.3660	-0.0008	1	KFDEGR (No match)
214 - 230	2010.0049	2008.9976	2009.0105	-0.0128	1	NNFEGEVTKENLLDFIK (No match)
231 - 247	1965.0304	1964.0231	1964.0367	-0.0135	0	HNQLPLVIEFTEQTAPK (No match)
248 - 254	763.4280	762.4207	762.4275	-0.0068	0	IFGGEIK (No match)
248 - 263	1826.0775	1825.0702	1825.0865	-0.0163	1	IFGGEIKTHILLFLPK (No match)
255 - 263	1081.6613	1080.6540	1080.6695	-0.0155	0	THILLFLPK (No match)
264 - 271	870.3718	869.3645	869.3766	-0.0121	0	SVSDYDGK (No match)
284 - 300	2018.9919	2017.9846	2018.0221	-0.0375	1	GKILFIFIDSDHTDNQR (No match)
286 - 300	1833.8931	1832.8858	1832.9057	-0.0198	0	ILFIFIDSDHTDNQR (No match)
286 - 300	1833.8931	1832.8858	1832.9057	-0.0198	0	ILFIFIDSDHTDNQR (Ions score 71)
301 - 308	966.5527	965.5454	965.5585	-0.0131	0	ILEFFGLK (No match)
301 - 309	1094.6490	1093.6417	1093.6535	-0.0118	1	ILEFFGLKK (No match)
309 - 316	988.5079	987.5006	987.4807	0.0199	1	KEECPAVR (No match)
317 - 326	1206.5905	1205.5832	1205.6213	-0.0381	0	LITLEEEMTK (No match)
317 - 326	1222.6117	1221.6044	1221.6162	-0.0118	0	LITLEEEMTK Oxidation (M) (No match)
327 - 338	1451.6882	1450.6809	1450.6939	-0.0130	0	YKPESEELTAER (Ions score 77)
327 - 338	1451.6882	1450.6809	1450.6939	-0.0130	0	YKPESEELTAER (No match)
339 - 345	962.4444	961.4371	961.4440	-0.0068	0	ITEFCHR (No match)
351 - 370	2434.2554	2433.2481	2433.2362	0.0119	1	IKPHLMSQELPEDWDKQPVK Oxidation (M) (No match)
376 - 385	1213.5243	1212.5170	1212.5298	-0.0128	0	NFEDVAFDEK (No match)
376 - 386	1341.6190	1340.6117	1340.6248	-0.0130	1	NFEDVAFDEKK (No match)
410 - 424	1729.8959	1728.8886	1728.9046	-0.0159	1	LGETYKDHENIVIAK (No match)
437 - 444	928.5138	927.5065	927.5178	-0.0112	0	VHSFPTLK (No match)
445 - 452	910.4350	909.4277	909.4344	-0.0067	0	FFPASADR (No match)
453 - 461	1066.5098	1065.5025	1065.5090	-0.0065	0	TVIDYNGER (No match)

Spot 349

Protein View

Match to: **gi|20070125** Score: **448** Expect: **3e-040**
prolyl 4-hydroxylase, beta subunit precursor [Homo sapiens]

Nominal mass (M_r): **57480**; Calculated pI value: **4.76**
NCBI BLAST search of gi|20070125 against nr
Unformatted sequence string for pasting into other applications

Taxonomy: Homo sapiens
Links to retrieve other entries containing this sequence from NCBI Entrez:
gi|2507460 from Homo sapiens
gi|190384 from Homo sapiens
gi|14790033 from Homo sapiens
gi|20810352 from Homo sapiens
gi|48735337 from Homo sapiens
gi|119610096 from Homo sapiens
gi|119610098 from Homo sapiens
gi|119610101 from Homo sapiens

Fixed modifications: Carbamidomethyl (C)
Variable modifications: Oxidation (M)
Cleavage by Trypsin: cuts C-term side of KR unless next residue is P
Sequence Coverage: **36%**

Matched peptides shown in **Bold Red**

1 MLRRALLCLA VAALVRADAP EEEDHVLVLR KSNFAEALAA HKYLLVEFYA
51 PWCGHCKALA PEYAKAAGK**L KAEGSEIR**LA K**VDATEESDL AQQYGVR**GYP
101 TIKFFRNGDT ASPKEYTAGR EADDIVNWLK KRTGPAATTL PDGAAAESLV
151 ESSEVAVIGF FKDVESDSAK QFLQAAEAID DIPFGITSNS DVFSK**YQLDK**
201 **DGVVLFKKFD EGRNNFEGEV TKENLLDFIK HNQLPLVIEF TEQTAPK**IFG
251 GEIK**THILLF LPKSVSDYDG K**LSNFKTAAE SFKGK**ILFIF IDSDHTDNQR**
301 **ILEFFGLKKE ECPAVR**LITL EEEMTK**YKPE SEELTAERIT EFCHR**FLEGK
351 IKPHLMSQEL PEDWDKQPVK VLVGK**NFEDV AFDEKK**NVFV EFYAPWCGHC
401 KQLAPIWDK**L GETYKDHENI VIAK**MDSTAN EVEAVKVHSF PTLK**FFPASA**
451 **DRTVIDYNGE R**TLDGFKKFL ESGGQDGAGD DDDLEDLEEA EEPDMEEDDD
501 QKAVKDEL

Residue Number Increasing Mass Decreasing Mass

Start - End	Observed	Mr(expt)	Mr(calc)	Delta	Miss	Sequence
70 - 78	1002.5817	1001.5744	1001.5505	0.0239	1	LKAEGSEIR (No match)
82 - 97	1780.8550	1779.8477	1779.8275	0.0203	0	VDATEESDLAQQYGVR (No match)
82 - 97	1780.8550	1779.8477	1779.8275	0.0203	0	VDATEESDLAQQYGVR (Ions score 107)
196 - 207	1424.7996	1423.7923	1423.7710	0.0213	1	YQLDKDGVVLFK (No match)
208 - 213	751.4083	750.4010	750.3660	0.0350	1	KFDEGR (No match)
214 - 230	2010.0485	2009.0412	2009.0105	0.0308	1	NNFEGEVTKENLLDFIK (No match)
231 - 247	1965.0629	1964.0556	1964.0367	0.0190	0	HNQLPLVIEFTEQTAPK (Ions score 88)
231 - 247	1965.0629	1964.0556	1964.0367	0.0190	0	HNQLPLVIEFTEQTAPK (No match)
255 - 263	1081.6885	1080.6812	1080.6695	0.0117	0	THILLFLPK (No match)
264 - 271	870.4061	869.3988	869.3766	0.0222	0	SVSDYDGK (No match)
286 - 300	1833.9316	1832.9243	1832.9057	0.0187	0	ILFIFIDSDHTDNQR (No match)
286 - 300	1833.9316	1832.9243	1832.9057	0.0187	0	ILFIFIDSDHTDNQR (Ions score 49)
301 - 308	966.5848	965.5775	965.5585	0.0190	0	ILEFFGLK (No match)
309 - 316	988.5378	987.5305	987.4807	0.0498	1	KEECPAVR (No match)
327 - 338	1451.7256	1450.7183	1450.6939	0.0244	0	YKPESEELTAER (Ions score 18)
327 - 338	1451.7256	1450.7183	1450.6939	0.0244	0	YKPESEELTAER (No match)
339 - 345	962.4773	961.4700	961.4440	0.0261	0	ITEFCHR (No match)
376 - 386	1341.6549	1340.6476	1340.6248	0.0229	1	NFEDVAFDEKK (No match)
410 - 424	1729.9305	1728.9232	1728.9046	0.0187	1	LGETYKDHENIVIAK (No match)
445 - 452	910.4706	909.4633	909.4344	0.0289	0	FFPASADR (No match)
453 - 461	1066.5450	1065.5377	1065.5090	0.0287	0	TVIDYNGER (No match)

Spot 350

Protein View

Match to: **gi|67464392** Score: **433** Expect: **9.6e-039**
Chain A, Structure Of Human Muscle Pyruvate Kinase (Pkm2)

Nominal mass (M_r): **60277**; Calculated pI value: **8.22**
NCBI BLAST search of gi|67464392 against nr
Unformatted sequence string for pasting into other applications

Taxonomy: Homo sapiens

Fixed modifications: Carbamidomethyl (C)
Variable modifications: Oxidation (M)
Cleavage by Trypsin: cuts C-term side of KR unless next residue is P
Sequence Coverage: **37%**

Matched peptides shown in **Bold Red**

```
  1 MGSSHHHHHH SSGLVPRGSK PHSEAGTAFI QTQQLHAAMA DTFLEHMCRL
 51 DIDSPPITAR NTGIICTIGP ASRSVETLKE MIKSGMNVAR LNFSHGTHEY
101 HAETIKNVRT ATESFASDPI LYRPVAVALD TKGPEIRTGL IKGSGTAEVE
151 LKKGATLKIT LDNAYMEKCD ENILWLDYKN ICKVVEVGSK IYVDDGLISL
201 QVKQKGADFL VTEVENGGSL GSKKGVNLPG AAVDLPAVSE KDIQDLKFGV
251 EQDVDMVFAS FIRKASDVHE VRKVLGEKGK NIKIISKIEN HEGVRRFDEI
301 LEASDGIMVA RGDLGIEIPA EKVFLAQKMM IGRCNRAGKP VICATQMLES
351 MIKKPRPTRA EGSDVANAVL DGADCIMLSG ETAKGDYPLE AVRMQHLIAR
401 EAEAAIYHLQ LFEELRRLAP ITSDPTEATA VGAVEASFKC CSGAIIVLTK
451 SGRSAHQVAR YRPRAPIIAV TRNPQTARQA HLYRGIFPVL CKDPVQEAWA
501 EDVDLRVNFA MNVGKARGFF KKGDVVIVLT GWRPGSGFTN TMRVVPVP
```

Residue Number Increasing Mass Decreasing Mass

Start - End	Observed	Mr(expt)	Mr(calc)	Delta	Miss	Sequence
50 - 60	1197.6868	1196.6795	1196.6401	0.0394	0	LDIDSPPITAR (No match)
50 - 60	1197.6868	1196.6795	1196.6401	0.0394	0	LDIDSPPITAR (Ions score 61)
61 - 73	1359.7466	1358.7393	1358.6976	0.0417	0	NTGIICTIGPASR (No match)
61 - 73	1359.7466	1358.7393	1358.6976	0.0417	0	NTGIICTIGPASR (Ions score 51)
91 - 106	1883.9440	1882.9367	1882.8961	0.0406	0	LNFSHGTHEYHAETIK (Ions score 42)
91 - 106	1883.9440	1882.9367	1882.8961	0.0406	0	LNFSHGTHEYHAETIK (No match)

110 - 132	2465.3418	2464.3345	2464.2849	0.0496	0	**TATESFASDPILYRPVAVALDTK** (Ions score 8)
110 - 132	2465.3418	2464.3345	2464.2849	0.0496	0	**TATESFASDPILYRPVAVALDTK** (No match)
191 - 203	1462.8486	1461.8413	1461.8078	0.0335	0	**IYVDDGLISLQVK** (No match)
206 - 223	1779.9170	1778.9097	1778.8685	0.0412	0	**GADFLVTEVENGGSLGSK** (No match)
248 - 263	1859.9446	1858.9373	1858.8923	0.0450	0	**FGVEQDVDMVFASFIR** (No match)
248 - 263	1875.9365	1874.9292	1874.8872	0.0420	0	**FGVEQDVDMVFASFIR** Oxidation (M) (No match)
288 - 295	953.5211	952.5138	952.4726	0.0412	0	**IENHEGVR** (No match)
296 - 311	1821.9586	1820.9513	1820.9090	0.0423	1	**RFDEILEASDGIMVAR** (No match)
296 - 311	1837.9576	1836.9503	1836.9039	0.0464	1	**RFDEILEASDGIMVAR** Oxidation (M) (No match)
385 - 393	1019.5575	1018.5502	1018.5083	0.0419	0	**GDYPLEAVR** (No match)
394 - 400	884.5102	883.5029	883.4698	0.0332	0	**MQHLIAR** Oxidation (M) (No match)
401 - 416	1932.0304	1931.0231	1930.9788	0.0443	0	**EAEAAIYHLQLFEELR** (No match)
401 - 417	2088.1306	2087.1233	2087.0799	0.0434	1	**EAEAAIYHLQLFEELRR** (No match)
401 - 417	2088.1306	2087.1233	2087.0799	0.0434	1	**EAEAAIYHLQLFEELRR** (Ions score 59)
418 - 439	2175.1731	2174.1658	2174.1106	0.0552	0	**LAPITSDPTEATAVGAVEASFK** (No match)
465 - 472	840.5685	839.5612	839.5228	0.0384	0	**APIIAVTR** (No match)
479 - 484	787.4653	786.4580	786.4136	0.0444	0	**QAHLYR** (No match)

Spot 351

Protein View

Match to: **gi|119613095** Score: **354** Expect: **7.7e-031**
WD repeat domain 1, isoform CRA_d [Homo sapiens]

Nominal mass (M_r): **58579**; Calculated pI value: **6.41**
NCBI BLAST search of gi|119613095 against nr
Unformatted sequence string for pasting into other applications

Taxonomy: Homo sapiens

Fixed modifications: Carbamidomethyl (C)
Variable modifications: Oxidation (M)
Cleavage by Trypsin: cuts C-term side of KR unless next residue is P
Sequence Coverage: **17%**

Matched peptides shown in **Bold Red**

```
  1 MPYEIKKVFA SLPQVERGVS KIIGGDPKGN NFLYTNGKCV ILRNIDNPAL
 51 ADIYTEHAHQ VVVAKYAPSG FYIASGDVSG KLRIWDTTQK EHLLKYEYQP
101 FAGKIKDIAW TEDSKRIAVV GEGREKFGAV FLWDSGSSVG EITGHNKVIN
151 SVDIKQSRPY RLATGSDDNC AAFFEGPPFK FKFTVGDHSR FVNCVRFSPD
201 GNRFATASAD GQIYIYDGKT GEKVCALGGS KAHDGGIYAI SWSPDSTHLL
251 SASGDKTSKI WDVSVNSVVS TFPMGSTVLD QQLGCLWQKD HLLSVSLSGY
301 INYLDRNNPS KPLHVIKGHS KSIQCLTVHK NGGKSYIYSG SHDGHINYWD
351 SETGENDSFA GKGHTNQVSR MTVDESGQLI SCSMDDTVRY TSLMLRDYSG
401 QGVVKLDVQP KCVAVGPGGY AVVVCIGQIV LLKDQRKCFS IDNPGYEPEV
451 VAVHPGGDTV AIGGVDGNVR LYSILGTTLK DEGKLLEAKG PVTDVAYSHD
501 GAFLAVCDAS KVVTVFSVAD GYSENNVFYG HHEK
```

Residue Number Increasing Mass Decreasing Mass

Start - End	Observed	Mr(expt)	Mr(calc)	Delta	Miss	Sequence
7 - 17	1273.7278	1272.7205	1272.7189	0.0016	1	KVFASLPQVER (No match)
8 - 17	1145.6316	1144.6243	1144.6240	0.0003	0	VFASLPQVER (Ions score 5)
8 - 17	1145.6316	1144.6243	1144.6240	0.0003	0	VFASLPQVER (No match)
44 - 65	2418.2444	2417.2371	2417.2338	0.0033	0	NIDNPALADIYTEHAHQVVVAK (Ions score 104)
44 - 65	2418.2444	2417.2371	2417.2338	0.0033	0	NIDNPALADIYTEHAHQVVVAK (No match)
66 - 81	1618.7726	1617.7653	1617.7674	-0.0021	0	YAPSGFYIASGDVSGK (Ions score 36)

66 - 81	1618.7726	1617.7653	1617.7674	-0.0021	0	YAPSGFYIASGDVSGK (No match)
96 - 104	1102.5143	1101.5070	1101.5130	-0.0060	0	YEYQPFAGK (No match)
96 - 104	1102.5143	1101.5070	1101.5130	-0.0060	0	YEYQPFAGK (Ions score 10)
162 - 180	2043.9137	2042.9064	2042.9043	0.0021	0	LATGSDDNCAAFFEGPPFK (No match)
162 - 180	2043.9137	2042.9064	2042.9043	0.0021	0	LATGSDDNCAAFFEGPPFK (Ions score 111)
290 - 306	1965.0092	1964.0019	1964.0003	0.0016	0	DHLLSVSLSGYINYLDR (No match)

Spot 352

Protein View

Match to: **gi|984305** Score: **196** Expect: **4.8e-015**
hPAK65

Nominal mass (M_r): **55166**; Calculated pI value: **5.97**
NCBI BLAST search of gi|984305 against nr
Unformatted sequence string for pasting into other applications

Taxonomy: Homo sapiens

Fixed modifications: Carbamidomethyl (C)
Variable modifications: Oxidation (M)
Cleavage by Trypsin: cuts C-term side of KR unless next residue is P
Sequence Coverage: **11%**

Matched peptides shown in **Bold Red**

```
  1 SANHSLKPLP SVPEEKKPRH KIISIFSGTE KGSKKKEKER PEISPPSDFE
 51 HTIHVGFDTV TGEFTGMPEQ WARLLQTSNI TKLEQKKNPQ AVLDVLKFYD
101 SNTVKQKYLS FTPPEKDGFP SGTPALNAKG TEAPAVVTEE EDDDEETAPP
151 VIAPRPDHTK SIYTRSVIDP VPAPVGDSHV DGAAKSLDKQ KKKTKMTDEE
201 IMEKLRTIVS IGDPKKKYTR YEKIGQGASG TVFTATDVAL GQEVAIKQIN
251 LQKQPKKELI INEILVMKEL KNPNIVNFLD SYLVGDELFV VMEYLAGRSL
301 TDVVTETCMD EAQIAAVCRE CLQALEFLHA NQVIHRDIKS DNVLLGMEGS
351 VKLTDFGFCA QITPEQSKRS TMVGTPYWMA PEVVTRKAYG PKVDIWSLGI
401 MAIEMVEGEP PYLNENPLRA LYLIATNGTP ELQNPEKLSP IFRDFLNRCL
451 EMDVEKRGSA KELLQHPFLK LAKPLSSLTP LIMAAKEAMK SNR
```

Residue Number Increasing Mass Decreasing Mass

Start - End	Observed	Mr(expt)	Mr(calc)	Delta	Miss	Sequence	
108 - 129	2337.2158	2336.2085	2336.1688	0.0397	1	YLSFTPPEKDGFPSGTPALNAK	(No match)
108 - 129	2337.2158	2336.2085	2336.1688	0.0397	1	YLSFTPPEKDGFPSGTPALNAK	(Ions score 64)
320 - 336	2078.0918	2077.0845	2077.0527	0.0319	0	ECLQALEFLHANQVIHR	(Ions score 82)
320 - 336	2078.0918	2077.0845	2077.0527	0.0319	0	ECLQALEFLHANQVIHR	(No match)
353 - 369	1998.0051	1996.9978	1996.9676	0.0302	1	LTDFGFCAQITPEQSKR	(No match)
353 - 369	1998.0051	1996.9978	1996.9676	0.0302	1	LTDFGFCAQITPEQSKR	(Ions score 18)

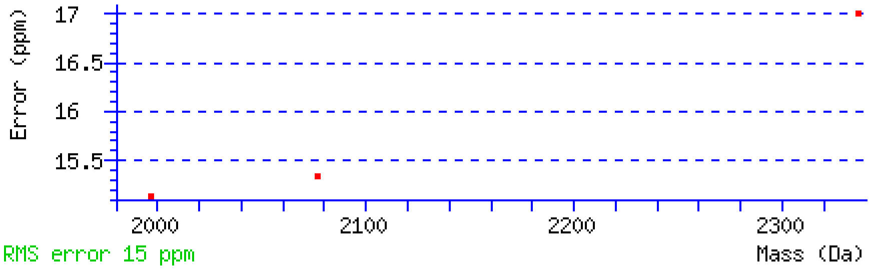

Error (ppm)
17
16.5
16
15.5
2000
2100
2200
2300
RMS error 15 ppm
Mass (Da)

Spot 353

Protein View

Match to: **gi|62897707** Score: **265** Expect: **6.1e-022**
coronin, actin binding protein, 1C variant [Homo sapiens]

Nominal mass (M_r): **53931**; Calculated pI value: **6.65**
NCBI BLAST search of gi|62897707 against nr
Unformatted sequence string for pasting into other applications

Taxonomy: Homo sapiens

Fixed modifications: Carbamidomethyl (C)
Variable modifications: Oxidation (M)
Cleavage by Trypsin: cuts C-term side of KR unless next residue is P
Sequence Coverage: **17%**

Matched peptides shown in **Bold Red**

```
  1 MRRVVRQSKF RHVFGQAVKN DQCYDDIRVS RVTWDSSFCA VNPRFVAIII
 51 EASGGGAFLV LPLHKTGRID KSYPTVCGHT GPVLDIDWCP HNDQAIASGS
101 EDCTVMVWQI PENGLTLSLT EPVVILEGHS KRVGIVAWHP TARNVLLSAG
151 CDNAIIIWNV GTGEALINLD DMHSDMIYNV SWNRNGSLIC TASKDKKVRV
201 IDPRKQEIVA EKEKAHEGAR PMRAIFLADG NVFTTGFSRM SERQLALWNP
251 KNMQEPIALH EMDTSNGVLL PFYDPDTSII YLCGKGDSSI RYFEITDESP
301 YVHYLNTFSS KEPQRGMGYM PKRGLDVNKC EIARFFKLHE RKCEPIIMTV
351 PRKSDLFQDD LYPDTAGPEA ALEAEEWFEG KNADPILISL KHGYIPGKNR
401 DLKVVKKNIL DSKPTANKKC DLISIPKKTT DTASVQNEAK LDEILKEIKS
451 IKDTICNQDE RIFKLEQQMA KIAA
```

Residue Number Increasing Mass Decreasing Mass

Start - End	Observed	Mr(expt)	Mr(calc)	Delta	Miss	Sequence
10 - 19	1188.6349	1187.6276	1187.6563	-0.0287	1	FRHVFGQAVK (Ions score 27)
10 - 19	1188.6349	1187.6276	1187.6563	-0.0287	1	FRHVFGQAVK (No match)
20 - 28	1198.4512	1197.4439	1197.4720	-0.0281	0	NDQCYDDIR (No match)
32 - 44	1538.6687	1537.6614	1537.6983	-0.0369	0	VTWDSSFCAVNPR (No match)
224 - 239	1715.8346	1714.8273	1714.8678	-0.0405	0	AIFLADGNVFTTGFSR (Ions score 40)
224 - 239	1715.8346	1714.8273	1714.8678	-0.0405	0	AIFLADGNVFTTGFSR (No match)
292 - 311	2440.0911	2439.0838	2439.1270	-0.0432	0	YFEITDESPYVHYLNTFSSK (Ions score 52)

292 - 311	2440.0911	2439.0838	2439.1270	-0.0432	0	YFEITDESPYVHYLNTFSSK	(No match)
292 - 315	2950.3184	2949.3111	2949.3820	-0.0709	1	YFEITDESPYVHYLNTFSSKEPQR	(No match)
292 - 315	2950.3184	2949.3111	2949.3820	-0.0709	1	YFEITDESPYVHYLNTFSSKEPQR	(Ions score 61)
343 - 353	1359.6760	1358.6687	1358.7050	-0.0363	1	CEPIIMTVPRK Oxidation (M)	(No match)

Spot 354

Protein View

Match to: **gi|33286420** Score: **368** Expect: **3e-032**
pyruvate kinase 3 isoform 2 [Homo sapiens]

Nominal mass (M_r): **58538;** Calculated pI value: **7.60**
NCBI BLAST search of gi|33286420 against nr
Unformatted sequence string for pasting into other applications

Taxonomy: Homo sapiens
Links to retrieve other entries containing this sequence from NCBI Entrez:
gi|33286422 from Homo sapiens
gi|119598290 from Homo sapiens
gi|119598296 from Homo sapiens

Fixed modifications: Carbamidomethyl (C)
Variable modifications: Oxidation (M)
Cleavage by Trypsin: cuts C-term side of KR unless next residue is P
Sequence Coverage: **43%**

Matched peptides shown in **Bold Red**

```
  1 MSKPHSEAGT AFIQTQQLHA AMADTFLEHM CRLDIDSPPI TARNTGIICT
 51 IGPASRSVET LKEMIKSGMN VARLNFSHGT HEYHAETIKN VRTATESFAS
101 DPILYRPVAV ALDTKGPEIR TGLIKGSGTA EVELKKGATL KITLDNAYME
151 KCDENILWLD YKNICKVVEV GSKIYVDDGL ISLQVKQKGA DFLVTEVENG
201 GSLGSKKGVN LPGAAVDLPA VSEKDIQDLK FGVEQDVDMV FASFIRKASD
251 VHEVRKVLGE KGKNIKIISK IENHEGVRRF DEILEASDGI MVARGDLGIE
301 IPAEKVFLAQ KMMIGRCNRA GKPVICATQM LESMIKKPRP TRAEGSDVAN
351 AVLDGADCIM LSGETAKGDY PLEAVRMQHL IAREAEAAMF HRKLFEELVR
401 ASSHSTDLME AMAMGSVEAS YKCLAAALIV LTESGRSAHQ VARYRPRAPI
451 IAVTRNPQTA RQAHLYRGIF PVLCKDPVQE AWAEDVDLRV NFAMNVGKAR
501 GFFKKGDVVI VLTGWRPGSG FTNTMRVVPV P
```

Residue Number Increasing Mass Decreasing Mass

Start - End	Observed	Mr(expt)	Mr(calc)	Delta	Miss	Sequence
33 - 43	1197.6300	1196.6227	1196.6401	-0.0174	0	LDIDSPPITAR (No match)
33 - 43	1197.6300	1196.6227	1196.6401	-0.0174	0	LDIDSPPITAR (Ions score 46)
44 - 56	1359.6864	1358.6791	1358.6976	-0.0185	0	NTGIICTIGPASR (Ions score 19)
44 - 56	1359.6864	1358.6791	1358.6976	-0.0185	0	NTGIICTIGPASR (No match)

74 - 89	1883.8745	1882.8672	1882.8961	-0.0289	0	**LNFSHGTHEYHAETIK** (Ions score 23)
74 - 89	1883.8745	1882.8672	1882.8961	-0.0289	0	**LNFSHGTHEYHAETIK** (No match)
93 - 115	2465.2515	2464.2442	2464.2849	-0.0407	0	**TATESFASDPILYRPVAVALDTK** (Ions score 17)
93 - 115	2465.2515	2464.2442	2464.2849	-0.0407	0	**TATESFASDPILYRPVAVALDTK** (No match)
93 - 120	3017.5750	3016.5677	3016.5869	-0.0191	1	**TATESFASDPILYRPVAVALDTKGPEIR** (No match)
174 - 186	1462.7897	1461.7824	1461.8078	-0.0254	0	**IYVDDGLISLQVK** (No match)
189 - 206	1779.8452	1778.8379	1778.8685	-0.0306	0	**GADFLVTEVENGGSLGSK** (No match)
231 - 246	1875.8646	1874.8573	1874.8872	-0.0299	0	**FGVEQDVDMVFASFIR** Oxidation (M) (No match)
248 - 256	1040.5375	1039.5302	1039.5410	-0.0108	1	**ASDVHEVRK** (No match)
271 - 278	953.4725	952.4652	952.4726	-0.0074	0	**IENHEGVR** (No match)
279 - 294	1837.8832	1836.8759	1836.9039	-0.0280	1	**RFDEILEASDGIMVAR** Oxidation (M) (No match)
320 - 336	1908.9260	1907.9187	1907.9518	-0.0331	0	**AGKPVICATQMLESMIK** 2 Oxidation (M) (No match)
337 - 342	754.4558	753.4485	753.4609	-0.0124	0	**KPRPTR** (No match)
368 - 376	1019.5068	1018.4995	1018.5083	-0.0088	0	**GDYPLEAVR** (No match)
377 - 383	884.4689	883.4616	883.4698	-0.0081	0	**MQHLIAR** Oxidation (M) (No match)
384 - 392	1077.4613	1076.4540	1076.4709	-0.0168	0	**EAEAAMFHR** Oxidation (M) (No match)
393 - 400	1033.5952	1032.5879	1032.5967	-0.0088	1	**KLFEELVR** (No match)
394 - 400	905.5048	904.4975	904.5018	-0.0042	0	**LFEELVR** (No match)
423 - 436	1473.7853	1472.7780	1472.8020	-0.0240	0	**CLAAALIVLTESGR** (Ions score 46)
423 - 436	1473.7853	1472.7780	1472.8020	-0.0240	0	**CLAAALIVLTESGR** (No match)
448 - 455	840.5221	839.5148	839.5228	-0.0080	0	**APIIAVTR** (No match)
462 - 467	787.4153	786.4080	786.4136	-0.0056	0	**QAHLYR** (No match)

Spot 355

Protein View

Match to: **gi|179950** Score: **105** Expect: **6.1e-006**
catalase

Nominal mass (M_r): **51550**; Calculated pI value: **7.83**
NCBI BLAST search of gi|179950 against nr
Unformatted sequence string for pasting into other applications

Taxonomy: Homo sapiens

Fixed modifications: Carbamidomethyl (C)
Variable modifications: Oxidation (M)
Cleavage by Trypsin: cuts C-term side of KR unless next residue is P
Sequence Coverage: **13%**

Matched peptides shown in **Bold Red**

```
  1 KGAGAFGYFE VTHDITKYSK AKVFEHIGKK TPIAVRFSTV AGESGSADTV
 51 RDPRGFAVKF YTEDGNWDLV GNNTPIFFIR DPILFPSFIH SQKRNPQTHL
101 KDPDMVWDFW SLRPESLHQV SFLFSDRGIP DGHRHMNGYG SHTFKLVNAN
151 GEAVYCKFHY KTDQGIKNLS VEDAARLSQE DPDYGIRDLF NAIATGKYPS
201 WTFYIQVMTF NQAETFPFNP FDLTKVWPHK DYPLIPVGKL VLNRNPVNYF
251 AEVEQIAFDP SNMPPGIEAS PDKMLQGRLF AYPDTHRHRL GPNYLHIPVN
301 CPYRARVANY QRDGPMCMQD NQGGAPNYYP NSFGAPEQQP SALEHSIQYS
351 GEVRRFNTAN DDNVTQVRAF YVNVLNEEQR KRLCENIAGH LKDAQIFIQK
401 KAVKNFTEVH PDYGSHIQAL LDKYNAEKPK NAIHTFVQSG SHLAAREKAN
451 L
```

Residue Number Increasing Mass Decreasing Mass

Start - End	Observed	Mr(expt)	Mr(calc)	Delta	Miss	Sequence
177 - 187	1292.6066	1291.5993	1291.6044	-0.0051	0	LSQEDPDYGIR (No match)
279 - 287	1119.5514	1118.5441	1118.5509	-0.0067	0	LFAYPDTHR (No match)
279 - 287	1119.5514	1118.5441	1118.5509	-0.0067	0	LFAYPDTHR (Ions score 9)
290 - 304	1812.8848	1811.8775	1811.9140	-0.0365	0	LGPNYLHIPVNCPYR (No match)
290 - 304	1812.8848	1811.8775	1811.9140	-0.0365	0	LGPNYLHIPVNCPYR (Ions score 10)
369 - 380	1481.7173	1480.7100	1480.7310	-0.0209	0	AFYVNVLNEEQR (No match)
369 - 380	1481.7173	1480.7100	1480.7310	-0.0209	0	AFYVNVLNEEQR (Ions score 26)

**431 - 446**	**1708.8572**	**1707.8499**	**1707.8804**	**-0.0305**	**0**	**NAIHTFVQSGSHLAAR**	(No match)
**431 - 446**	**1708.8572**	**1707.8499**	**1707.8804**	**-0.0305**	**0**	**NAIHTFVQSGSHLAAR**	(No match)

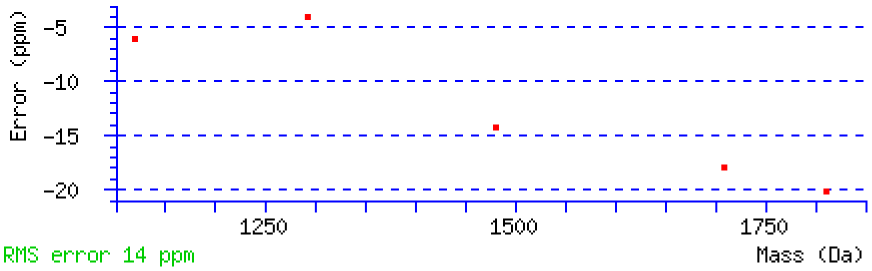

RMS error 14 ppm

Spot 356

Protein View

Match to: **gi|189998** Score: **212** Expect: **1.2e-016**
M2-type pyruvate kinase

Nominal mass (M_r): **58447;** Calculated pI value: **7.95**
NCBI BLAST search of gi|189998 against nr
Unformatted sequence string for pasting into other applications

Taxonomy: Homo sapiens

Fixed modifications: Carbamidomethyl (C)
Variable modifications: Oxidation (M)
Cleavage by Trypsin: cuts C-term side of KR unless next residue is P
Sequence Coverage: **12%**

Matched peptides shown in **Bold Red**

```
  1 MSKPHSEAGT AFIQTQQLHA AMADTFLEHM CRLDIDSPPI TARNTGIICT
 51 IGPASRSVET LKEMIKSGMN VARLNFSHGT HEYHAETIKN VRTATESFAS
101 DPILYRPVAV ALDTKGPEIR TGLIKGSGTA EVELKKGATL KITLDNAYME
151 KCDENILWLD YKNICKVVEV GSKIYVDDGL ISLQVKQKGA DFLVTEVENG
201 GSLGSKKGVN LPGAAVDLPA VSEKDIQDLK FGVEQDVDMV FASFIRKASD
251 VHEVRKVLGE KGKNIKIISK IENHEGVRRF DEILEASDGI MVARGDLGIE
301 IPAEKVFLAQ KMMIGRCNRA GKPVICATQM LESMIKKPRP TRAEGSDVAN
351 AVLDGADCIM LSGETAKGDY PLEAVRMQNL IAREAEAAIY HLQLFEELRR
401 LAPITSDPTE ATAVGAVEAS FKCCSGAIIV LTKSGRSAHQ VARYRPRAPI
451 IAVTRNPQTA RQAHLYRGIF PVLCKDPVQE AWAEDVDLRV NFAMNVGKAR
501 GFFKKGDVVI VLTGWRPGSG FTNTMRVVPV P
```

Residue Number Increasing Mass Decreasing Mass

Start - End	Observed	Mr(expt)	Mr(calc)	Delta	Miss	Sequence
44 - 56	1359.7113	1358.7040	1358.6976	0.0064	0	NTGIICTIGPASR (No match)
44 - 56	1359.7113	1358.7040	1358.6976	0.0064	0	NTGIICTIGPASR (Ions score 19)
74 - 89	1883.9098	1882.9025	1882.8961	0.0064	0	LNFSHGTHEYHAETIK (Ions score 68)
74 - 89	1883.9098	1882.9025	1882.8961	0.0064	0	LNFSHGTHEYHAETIK (No match)
93 - 115	2465.2981	2464.2908	2464.2849	0.0059	0	TATESFASDPILYRPVAVALDTK (No match)
93 - 115	2465.2981	2464.2908	2464.2849	0.0059	0	TATESFASDPILYRPVAVALDTK (Ions score 38)

384 - 400	2088.0935	2087.0862	2087.0799	0.0063	1	EAEAAIYHLQLFEELRR	(Ions score 39)
384 - 400	2088.0935	2087.0862	2087.0799	0.0063	1	EAEAAIYHLQLFEELRR	(No match)

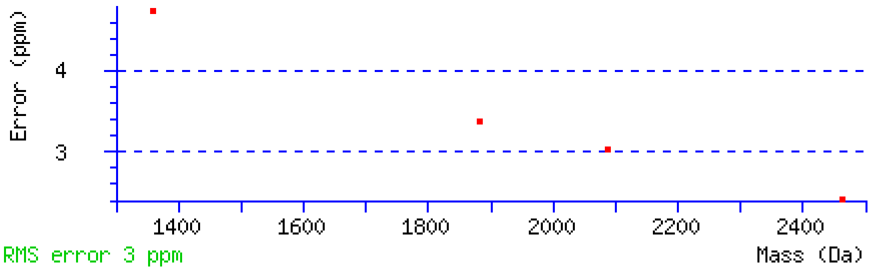

Spot 357

Protein View

Match to: **gi|48762932** Score: **314** Expect: **7.7e-027**
chaperonin containing TCP1, subunit 8 (theta) [Homo sapiens]

Nominal mass (M_r): **60153**; Calculated pI value: **5.42**
NCBI BLAST search of gi|48762932 against nr
Unformatted sequence string for pasting into other applications

Taxonomy: Homo sapiens
Links to retrieve other entries containing this sequence from NCBI Entrez:
gi|9988062 from Homo sapiens
gi|66773868 from Pongo pygmaeus
gi|7717311 from Homo sapiens
gi|47940653 from Homo sapiens
gi|55728862 from Pongo pygmaeus
gi|66267606 from Homo sapiens
gi|119630330 from Homo sapiens

Fixed modifications: Carbamidomethyl (C)
Variable modifications: Oxidation (M)
Cleavage by Trypsin: cuts C-term side of KR unless next residue is P
Sequence Coverage: **15%**

Matched peptides shown in **Bold Red**

1 MALHVPKAPG FAQMLKEGAK **HFSGLEEAVY R**NIQACKELA QTTRTAYGPN
51 GMNKMVINHL EK**LFVTNDAA TILR**ELEVQH PAAKMIVMAS HMQEQEVGDG
101 TNFVLVFAGA LLELAEELLR **IGLSVSEVIE GYEIACR**KAH EILPNLVCCS
151 AK**NLRDIDEV SSLLR**TSIMS KQYGNEVFLA K**LIAQACVSI FPDSGHFNVD**
201 **NIR**VCKILGS GISSSSVLHG MVFKKETEGD VTSVKDAKIA VYSCPFDGMI
251 TETKGTVLIK TAEELMNFSK GEENLMDAQV KAIADTGANV VVTGGKVADM
301 ALHYANKYNI MLVRLNSKWD LRRLCKTVGA TALPRLTPPV LEEMGHCDSV
351 YLSEVGDTQV VVFKHEKEDG AISTIVLRGS TDNLMDDIER AVDDGVNTFK
401 VLTRDKRLVP GGGATEIELA KQITSYGETC PGLEQYAIK**K FAEAFEAIPR**
451 ALAENSGVKA NEVISKLYAV HQEGNKNVGL DIEAEVPAVK DMLEAGILDT
501 YLGKYWAIKL ATNAAVTVLR VDQIIMAKPA GGPKPPSGKK DWDDDQND

Residue Number Increasing Mass Decreasing Mass

Start - End	Observed	Mr(expt)	Mr(calc)	Delta	Miss	Sequence
21 - 31	1307.6061	1306.5988	1306.6305	-0.0317	0	HFSGLEEAVYR (Ions score 47)
21 - 31	1307.6061	1306.5988	1306.6305	-0.0317	0	HFSGLEEAVYR (No match)
63 - 74	1333.7156	1332.7083	1332.7401	-0.0318	0	LFVTNDAATILR (No match)
63 - 74	1333.7156	1332.7083	1332.7401	-0.0318	0	LFVTNDAATILR (Ions score 20)
121 - 137	1894.9138	1893.9065	1893.9505	-0.0440	0	IGLSVSEVIEGYEIACR (No match)
121 - 137	1894.9138	1893.9065	1893.9505	-0.0440	0	IGLSVSEVIEGYEIACR (Ions score 25)
153 - 165	1529.7909	1528.7836	1528.8209	-0.0372	1	NLRDIDEVSSLLR (No match)
182 - 203	2473.1758	2472.1685	2472.2219	-0.0534	0	LIAQACVSIFPDSGHFNVDNIR (Ions score 82)
182 - 203	2473.1758	2472.1685	2472.2219	-0.0534	0	LIAQACVSIFPDSGHFNVDNIR (No match)
440 - 450	1278.6506	1277.6433	1277.6767	-0.0334	1	KFAEAFEAIPR (No match)
441 - 450	1150.5562	1149.5489	1149.5818	-0.0329	0	FAEAFEAIPR (Ions score 37)
441 - 450	1150.5562	1149.5489	1149.5818	-0.0329	0	FAEAFEAIPR (No match)

Spot 358

Protein View

Match to: **gi|48762932** Score: **183** Expect: **9.6e-014**
chaperonin containing TCP1, subunit 8 (theta) [Homo sapiens]

Nominal mass (M_r): **60153**; Calculated pI value: **5.42**
NCBI BLAST search of gi|48762932 against nr
Unformatted sequence string for pasting into other applications

Taxonomy: Homo sapiens
Links to retrieve other entries containing this sequence from NCBI Entrez:
gi|9988062 from Homo sapiens
gi|66773868 from Pongo pygmaeus
gi|7717311 from Homo sapiens
gi|47940653 from Homo sapiens
gi|55728862 from Pongo pygmaeus
gi|66267606 from Homo sapiens
gi|119630330 from Homo sapiens

Fixed modifications: Carbamidomethyl (C)
Variable modifications: Oxidation (M)
Cleavage by Trypsin: cuts C-term side of KR unless next residue is P
Sequence Coverage: **13%**

Matched peptides shown in **Bold Red**

```
  1 MALHVPKAPG FAQMLKEGAK HFSGLEEAVY RNIQACKELA QTTRTAYGPN
 51 GMNKMVINHL EKLFVTNDAA TILRELEVQH PAAKMIVMAS HMQEQEVGDG
101 TNFVLVFAGA LLELAEELLR IGLSVSEVIE GYEIACRKAH EILPNLVCCS
151 AKNLRDIDEV SSLLRTSIMS KQYGNEVFLA KLIAQACVSI FPDSGHFNVD
201 NIRVCKILGS GISSSSVLHG MVFKKETEGD VTSVKDAKIA VYSCPFDGMI
251 TETKGTVLIK TAEELMNFSK GEENLMDAQV KAIADTGANV VVTGGKVADM
301 ALHYANKYNI MLVRLNSKWD LRRLCKTVGA TALPRLTPPV LEEMGHCDSV
351 YLSEVGDTQV VVFKHEKEDG AISTIVLRGS TDNLMDDIER AVDDGVNTFK
401 VLTRDKRLVP GGGATEIELA KQITSYGETC PGLEQYAIKK FAEAFEAIPR
451 ALAENSGVKA NEVISKLYAV HQEGNKNVGL DIEAEVPAVK DMLEAGILDT
501 YLGKYWAIKL ATNAAVTVLR VDQIIMAKPA GGPKPPSGKK DWDDDQND
```

Residue Number Increasing Mass Decreasing Mass

Start - End	Observed	Mr(expt)	Mr(calc)	Delta	Miss	Sequence
21 - 31	1307.6096	1306.6023	1306.6305	-0.0282	0	HFSGLEEAVYR (No match)
21 - 31	1307.6096	1306.6023	1306.6305	-0.0282	0	HFSGLEEAVYR (Ions score 10)
63 - 74	1333.7159	1332.7086	1332.7401	-0.0315	0	LFVTNDAATILR (Ions score 5)
63 - 74	1333.7159	1332.7086	1332.7401	-0.0315	0	LFVTNDAATILR (No match)
121 - 137	1894.9159	1893.9086	1893.9505	-0.0419	0	IGLSVSEVIEGYEIACR (No match)
121 - 137	1894.9159	1893.9086	1893.9505	-0.0419	0	IGLSVSEVIEGYEIACR (Ions score 18)
182 - 203	2473.1733	2472.1660	2472.2219	-0.0559	0	LIAQACVSIFPDSGHFNVDNIR (No match)
182 - 203	2473.1733	2472.1660	2472.2219	-0.0559	0	LIAQACVSIFPDSGHFNVDNIR (Ions score 44)
441 - 450	1150.5597	1149.5524	1149.5818	-0.0294	0	FAEAFEAIPR (Ions score 23)
441 - 450	1150.5597	1149.5524	1149.5818	-0.0294	0	FAEAFEAIPR (No match)

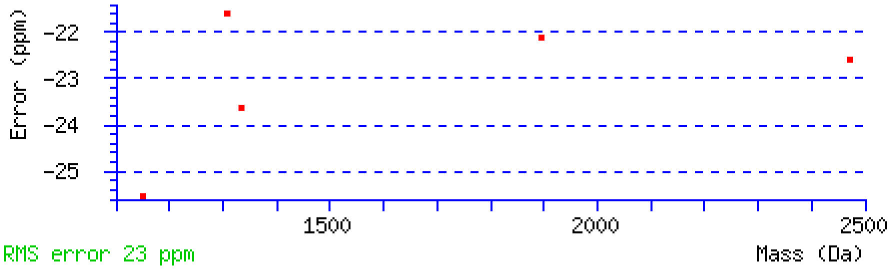

RMS error 23 ppm

Spot 360

Protein View

Match to: **gi|5902134** Score: **360** Expect: **1.9e-031**
coronin, actin binding protein, 1A [Homo sapiens]

Nominal mass (M_r): **51678;** Calculated pI value: **6.25**
NCBI BLAST search of gi|5902134 against nr
Unformatted sequence string for pasting into other applications

Taxonomy: Homo sapiens
Links to retrieve other entries containing this sequence from NCBI Entrez:
gi|1706004 from Homo sapiens
gi|20271119 from Homo sapiens
gi|927649 from Homo sapiens
gi|1136140 from Homo sapiens
gi|82571468 from Homo sapiens
gi|116497053 from Homo sapiens
gi|116497211 from Homo sapiens
gi|119600312 from Homo sapiens
gi|119600313 from Homo sapiens
gi|119600314 from Homo sapiens
gi|119600316 from Homo sapiens

Fixed modifications: Carbamidomethyl (C)
Variable modifications: Oxidation (M)
Cleavage by Trypsin: cuts C-term side of KR unless next residue is P
Sequence Coverage: **37%**

Matched peptides shown in **Bold Red**

1 MSRQVVRSSK **FRHVFGQPAK ADQCYEDVRV SQTTWDSGFC AVNPKFVALI**
51 **CEASGGGAFL VLPLGK**TGRV DKNAPTVCGH TAPVLDIAWC PHNDNVIASG
101 SEDCTVMVWE IPDGGLMLPL REPVVTLEGH TKRVGIVAWH TTAQNVLLSA
151 GCDNVIMVWD VGTGAAMLTL GPEVHPDTIY SVDWSR**DGGL ICTSCR**DKRV
201 RIIEPRKGTV VAEK**DRPHEG TRPVR**AVFVS EGK**ILTTGFS R**MSER**QVALW**
251 **DTK**HLEEPLS LQELDTSSGV LLPFFDPDTN IVYLCGKGDS SIR**YFEITSE**
301 **APFLHYLSMF SSK**ESQRGMG YMPKR**GLEVN KCEIAR**FYKL HER**RCEPIAM**
351 **TVPRKSDLFQ EDLYPPTAGP DPALTAEEWL GGRDAGPLLI SLK**DGYVPPK
401 SRELRVNRGL DTGRRRAAPE ASGTPSSDAV SRLEEEMRKL QATVQELQKR
451 LDRLEETVQA K

Residue Number Increasing Mass Decreasing Mass

Start - End	Observed	Mr(expt)	Mr(calc)	Delta	Miss	Sequence
11 - 20	1186.6456	1185.6383	1185.6406	-0.0023	1	FRHVFGQPAK (No match)
13 - 20	883.5141	882.5068	882.4711	0.0357	0	HVFGQPAK (No match)
21 - 29	1155.4708	1154.4635	1154.4662	-0.0027	0	ADQCYEDVR (No match)
21 - 29	1155.4708	1154.4635	1154.4662	-0.0027	0	ADQCYEDVR (Ions score 12)
30 - 45	1796.8220	1795.8147	1795.8199	-0.0052	0	VSQTTWDSGFCAVNPK (No match)
30 - 45	1796.8220	1795.8147	1795.8199	-0.0052	0	VSQTTWDSGFCAVNPK (Ions score 99)
46 - 66	2119.1624	2118.1551	2118.1546	0.0005	0	FVALICEASGGGAFLVLPLGK (No match)
187 - 196	1138.4954	1137.4881	1137.4906	-0.0025	0	DGGLICTSCR (No match)
187 - 196	1138.4954	1137.4881	1137.4906	-0.0025	0	DGGLICTSCR (Ions score 40)
215 - 225	1319.6868	1318.6795	1318.6854	-0.0059	0	DRPHEGTRPVR (Ions score 39)
215 - 225	1319.6868	1318.6795	1318.6854	-0.0059	0	DRPHEGTRPVR (No match)
234 - 241	894.5016	893.4943	893.4970	-0.0027	0	ILTTGFSR (No match)
246 - 253	960.5037	959.4964	959.5076	-0.0112	0	QVALWDTK (No match)
294 - 313	2397.1311	2396.1238	2396.1398	-0.0160	0	YFEITSEAPFLHYLSMFSSK (No match)
294 - 313	2413.1414	2412.1341	2412.1347	-0.0006	0	YFEITSEAPFLHYLSMFSSK Oxidation (M) (No match)
326 - 336	1288.6676	1287.6603	1287.6604	-0.0001	1	GLEVNKCEIAR (No match)
344 - 354	1329.6722	1328.6649	1328.6693	-0.0044	1	RCEPIAMTVPR (No match)
344 - 354	1329.6722	1328.6649	1328.6693	-0.0044	1	RCEPIAMTVPR (Ions score 9)
344 - 354	1345.6663	1344.6590	1344.6642	-0.0052	1	RCEPIAMTVPR Oxidation (M) (No match)
345 - 354	1173.5657	1172.5584	1172.5682	-0.0098	0	CEPIAMTVPR (No match)
345 - 354	1189.5687	1188.5614	1188.5631	-0.0017	0	CEPIAMTVPR Oxidation (M) (No match)
355 - 383	3173.5051	3172.4978	3172.5352	-0.0374	1	KSDLFQEDLYPPTAGPDPALTAEEWLGGR (No match)
384 - 393	1026.6140	1025.6067	1025.6120	-0.0053	0	DAGPLLISLK (No match)

Spot 361

Protein View

Match to: **gi|4557014** Score: **274** Expect: **7.7e-023**
catalase [Homo sapiens]

Nominal mass (M_r): **59947;** Calculated pI value: **6.90**
NCBI BLAST search of gi|4557014 against nr
Unformatted sequence string for pasting into other applications

Taxonomy: Homo sapiens
Links to retrieve other entries containing this sequence from NCBI Entrez:
gi|115702 from Homo sapiens
gi|5542444 from Homo sapiens
gi|5542445 from Homo sapiens
gi|5542446 from Homo sapiens
gi|5542447 from Homo sapiens
gi|9257059 from Homo sapiens
gi|9257060 from Homo sapiens
gi|9257061 from Homo sapiens
gi|9257062 from Homo sapiens
gi|29721 from Homo sapiens
gi|1228085 from Homo sapiens
gi|5102626 from Homo sapiens
gi|42716352 from Homo sapiens
gi|85566897 from Homo sapiens
gi|85567275 from Homo sapiens
gi|111493936 from Homo sapiens
gi|119588576 from Homo sapiens
gi|119588577 from Homo sapiens
gi|158256602 (no taxonomy information for this entry)

Fixed modifications: Carbamidomethyl (C)
Variable modifications: Oxidation (M)
Cleavage by Trypsin: cuts C-term side of KR unless next residue is P
Sequence Coverage: **29%**

Matched peptides shown in **Bold Red**

```
  1 MADSRDPASD QMQHWKEQRA AQKADVLTTG AGNPVGDKLN VITVGPRGPL
 51 LVQDVVFTDE MAHFDRERIP ERVVHAKGAG AFGYFEVTHD ITKYSKAKVF
101 EHIGKKTPIA VRFSTVAGES GSADTVRDPR GFAVKFYTED GNWDLVGNNT
151 PIFFIRDPIL FPSFIHSQKR NPQTHLKDPD MVWDFWSLRP ESLHQVSFLF
201 SDRGIPDGHR HMNGYGSHTF KLVNANGEAV YCKFHYKTDQ GIKNLSVEDA
```

```
251 ARLSQEDPDY GIRDLFNAIA TGKYPSWTFY IQVMTFNQAE TFPFNPFDLT
301 KVWPHKDYPL IPVGKLVLNR NPVNYFAEVE QIAFDPSNMP PGIEASPDKM
351 LQGRLFAYPD THRHRLGPNY LHIPVNCPYR ARVANYQRDG PMCMQDNQGG
401 APNYYPNSFG APEQQPSALE HSIQYSGEVR RFNTANDDNV TQVRAFYVNV
451 LNEEQRKRLC ENIAGHLKDA QIFIQKKAVK NFTEVHPDYG SHIQALLDKY
501 NAEKPKNAIH TFVQSGSHLA AREKANL
```

Residue Number Increasing Mass Decreasing Mass

Start - End	Observed	Mr(expt)	Mr(calc)	Delta	Miss	Sequence
24 - 47	2364.2859	2363.2786	2363.2808	-0.0021	1	ADVLTTGAGNPVGDKLNVITVGPR (Ions score 61)
24 - 47	2364.2859	2363.2786	2363.2808	-0.0021	1	ADVLTTGAGNPVGDKLNVITVGPR (No match)
39 - 47	968.5666	967.5593	967.5814	-0.0221	0	LNVITVGPR (No match)
48 - 66	2205.0737	2204.0664	2204.0571	0.0093	0	GPLLVQDVVFTDEMAHFDR Oxidation (M) (No match)
78 - 93	1712.8368	1711.8295	1711.8205	0.0090	0	GAGAFGYFEVTHDITK (No match)
113 - 130	1851.8912	1850.8839	1850.8758	0.0081	1	FSTVAGESGSADTVRDPR (No match)
253 - 263	1292.6129	1291.6056	1291.6044	0.0012	0	LSQEDPDYGIR (No match)
355 - 363	1119.5571	1118.5498	1118.5509	-0.0010	0	LFAYPDTHR (Ions score 22)
355 - 363	1119.5571	1118.5498	1118.5509	-0.0010	0	LFAYPDTHR (No match)
366 - 380	1812.9254	1811.9181	1811.9140	0.0041	0	LGPNYLHIPVNCPYR (No match)
366 - 380	1812.9254	1811.9181	1811.9140	0.0041	0	LGPNYLHIPVNCPYR (Ions score 23)
432 - 444	1493.7006	1492.6933	1492.6906	0.0027	0	FNTANDDNVTQVR (No match)
445 - 456	1481.7397	1480.7324	1480.7310	0.0015	0	AFYVNVLNEEQR (No match)
445 - 456	1481.7397	1480.7324	1480.7310	0.0015	0	AFYVNVLNEEQR (Ions score 59)
507 - 522	1708.8918	1707.8845	1707.8804	0.0041	0	NAIHTFVQSGSHLAAR (No match)
507 - 522	1708.8918	1707.8845	1707.8804	0.0041	0	NAIHTFVQSGSHLAAR (Ions score 7)

Spot 362

Protein View

Match to: **gi|340021** Score: **479** Expect: **2.4e-043**
alpha-tubulin

Nominal mass (M_r): **50804;** Calculated pI value: **4.94**
NCBI BLAST search of gi|340021 against nr
Unformatted sequence string for pasting into other applications

Taxonomy: Homo sapiens

Fixed modifications: Carbamidomethyl (C)
Variable modifications: Oxidation (M)
Cleavage by Trypsin: cuts C-term side of KR unless next residue is P
Sequence Coverage: **45%**

Matched peptides shown in **Bold Red**

```
  1 MRECISIHVG QAGVQIGNAC WELYCLEHGI QPDGQMPSDK TIGGGDDSFN
 51 TFFSETGAGK HVPRAVFVDL EPTVIDEVRT GTYRQLFHPE QLITGKEDAA
101 NNYARGHYTI GKEIIDLVLD RIRKLADQCT RLQGFLVFHS FGGGTGSGFT
151 SLLMERLSVD YGKKSKLEFS IYPAPQVSTA VVEPYNSILT THTTLEHSDC
201 AFMVDNEAIY DICRRNLDIE RPTYTNLNRL ISQIVSSITA SLRFDGALNV
251 DLTEFQTNLV PYPRIHFPLA TYAPVISAEK AYHEQLSVAD ITNACFEPAN
301 QMVKCDPGHG KYMACCLLYR GDVVPKDVNA AIATIKTKRT IQFVDWCPTG
351 FKVGINYQPP TVVPGGDLAK VQRAVCMLSN TTAIAEAWAR LDHKFDLMYA
401 KRAFVHWYVG EGMEEGEFSE AREDMAALEK DYEEVGVDSV EGEGEEEGEE
451 Y
```

Residue Number Increasing Mass Decreasing Mass

Start - End	Observed	Mr(expt)	Mr(calc)	Delta	Miss	Sequence
41 - 60	2007.8905	2006.8832	2006.8857	-0.0024	0	TIGGGDDSFNTFFSETGAGK (No match)
65 - 79	1701.8990	1700.8917	1700.8984	-0.0067	0	AVFVDLEPTVIDEVR (Ions score 84)
65 - 79	1701.8990	1700.8917	1700.8984	-0.0067	0	AVFVDLEPTVIDEVR (No match)
85 - 96	1410.7649	1409.7576	1409.7666	-0.0090	0	QLFHPEQLITGK (Ions score 81)
85 - 96	1410.7649	1409.7576	1409.7666	-0.0090	0	QLFHPEQLITGK (No match)
85 - 105	2415.1921	2414.1848	2414.1978	-0.0130	1	QLFHPEQLITGKEDAANNYAR (No match)
97 - 105	1023.4465	1022.4392	1022.4417	-0.0025	0	EDAANNYAR (No match)

113 - 121	1085.6112	1084.6039	1084.6128	-0.0089	0	**EIIDLVLDR** (No match)
216 - 229	1718.8761	1717.8688	1717.8747	-0.0059	0	**NLDIERPTYTNLNR** (Ions score 34)
216 - 229	1718.8761	1717.8688	1717.8747	-0.0059	0	**NLDIERPTYTNLNR** (No match)
230 - 243	1487.8765	1486.8692	1486.8718	-0.0026	0	**LISQIVSSITASLR** (Ions score 78)
230 - 243	1487.8765	1486.8692	1486.8718	-0.0026	0	**LISQIVSSITASLR** (No match)
244 - 264	2409.1897	2408.1824	2408.2011	-0.0187	0	**FDGALNVDLTEFQTNLVPYPR** (No match)
265 - 280	1756.9548	1755.9475	1755.9559	-0.0084	0	**IHFPLATYAPVISAEK** (No match)
312 - 320	1249.5491	1248.5418	1248.5453	-0.0035	0	**YMACCLLYR** (Ions score 36)
312 - 320	1249.5491	1248.5418	1248.5453	-0.0035	0	**YMACCLLYR** (No match)
312 - 320	1265.5413	1264.5340	1264.5402	-0.0062	0	**YMACCLLYR** Oxidation (M) (No match)
353 - 370	1824.9813	1823.9740	1823.9781	-0.0040	0	**VGINYQPPTVVPGGDLAK** (No match)
374 - 390	1864.8959	1863.8886	1863.8971	-0.0085	0	**AVCMLSNTTAIAEAWAR** (No match)
374 - 390	1880.8969	1879.8896	1879.8920	-0.0024	0	**AVCMLSNTTAIAEAWAR** Oxidation (M) (No match)
391 - 401	1380.6913	1379.6840	1379.6907	-0.0067	1	**LDHKFDLMYAK** (No match)
391 - 401	1396.6910	1395.6837	1395.6856	-0.0019	1	**LDHKFDLMYAK** Oxidation (M) (No match)
403 - 422	2330.0071	2328.9998	2329.0109	-0.0110	0	**AFVHWYVGEGMEEGEFSEAR** (No match)

Spot 363

Protein View

Match to: **gi|62897609** Score: **460** Expect: **1.9e-041**
tubulin alpha 6 variant [Homo sapiens]

Nominal mass (M_r): **50476**; Calculated pI value: **5.00**
NCBI BLAST search of gi|62897609 against nr
Unformatted sequence string for pasting into other applications

Taxonomy: Homo sapiens

Fixed modifications: Carbamidomethyl (C)
Variable modifications: Oxidation (M)
Cleavage by Trypsin: cuts C-term side of KR unless next residue is P
Sequence Coverage: **34%**

Matched peptides shown in **Bold Red**

```
  1 MRECISIHVG QAGVQIGNAC WELYCLEHGI QPDGQMPSDK TIGGGDDSFN
 51 TFFSETGAGK HVPRAVFVDL EPTVIDEVRT GTYRQLFHPE QLITGKEDAA
101 NNYARGHYTI GKEIIDLVLD RIRKLADQCT GLQGFLVFHS FGGGTGSGFT
151 SLLMERLSVD YGKKSKLEFS IYPAPQVSTA VVEPYNSILT THTTLEHSDC
201 AFMVDNEAIY DICRRNLDIE RPTYTNLNRL ISQIVSSITA SLRFDGALNV
251 DLTEFQTNLV PYPRIHFPLA TYAPVISAEK AYHEQLTVAE ITNACFEPAN
301 QMVKCDPRHG KYMACCLLYR GDVVPKDVNA AIATIKTKRT IQFVDWCPTG
351 FKVGINYQPP TVVPGGDLAK VQRAVCMLSN TTAVAEAWAR LDHKFDLMYA
401 KRAFVHWYVG EGMEEGEFSE AREDMAALEK DYEGVGADSA DGEDEGEEY
```

Residue Number Increasing Mass Decreasing Mass

Start - End	Observed	Mr(expt)	Mr(calc)	Delta	Miss	Sequence
41 - 60	1007.8882	2006.8809	2006.8857	-0.0047	0	TIGGGDDSFNTFFSETGAGK (No match)
65 - 79	1701.8851	1700.8778	1700.8984	-0.0206	0	AVFVDLEPTVIDEVR (Ions score 84)
65 - 79	1701.8851	1700.8778	1700.8984	-0.0206	0	AVFVDLEPTVIDEVR (No match)
85 - 96	1410.7520	1409.7447	1409.7666	-0.0219	0	QLFHPEQLITGK (Ions score 91)
85 - 96	1410.7520	1409.7447	1409.7666	-0.0219	0	QLFHPEQLITGK (No match)
85 - 105	2415.1799	2414.1726	2414.1978	-0.0252	1	QLFHPEQLITGKEDAANNYAR (No match)
97 - 105	1023.4388	1022.4315	1022.4417	-0.0102	0	EDAANNYAR (No match)
113 - 121	1085.6050	1084.5977	1084.6128	-0.0151	0	EIIDLVLDR (No match)

216 - 229	1718.8646	1717.8573	1717.8747	-0.0174	0	NLDIERPTYTNLNR	(Ions score 22)
216 - 229	1718.8646	1717.8573	1717.8747	-0.0174	0	NLDIERPTYTNLNR	(No match)
230 - 243	1487.8604	1486.8531	1486.8718	-0.0187	0	LISQIVSSITASLR	(No match)
230 - 243	1487.8604	1486.8531	1486.8718	-0.0187	0	LISQIVSSITASLR	(Ions score 79)
244 - 264	2409.1863	2408.1790	2408.2011	-0.0221	0	FDGALNVDLTEFQTNLVPYPR	(No match)
265 - 280	1756.9427	1755.9354	1755.9559	-0.0205	0	IHFPLATYAPVISAEK	(No match)
312 - 320	1249.5403	1248.5330	1248.5453	-0.0123	0	YMACCLLYR	(No match)
312 - 320	1265.5259	1264.5186	1264.5402	-0.0216	0	YMACCLLYR Oxidation (M)	(Ions score 14)
312 - 320	1265.5259	1264.5186	1264.5402	-0.0216	0	YMACCLLYR Oxidation (M)	(No match)
353 - 370	1824.9647	1823.9574	1823.9781	-0.0206	0	VGINYQPPTVVPGGDLAK	(No match)

Spot 364

Protein View

Match to: **gi|37589898** Score: **223** Expect: **9.6e-018**
PPP5C protein [Homo sapiens]

Nominal mass (M_r): **55632**; Calculated pI value: **5.84**
NCBI BLAST search of gi|37589898 against nr
Unformatted sequence string for pasting into other applications

Taxonomy: Homo sapiens
Links to retrieve other entries containing this sequence from NCBI Entrez:
gi|38197276 from Homo sapiens

Fixed modifications: Carbamidomethyl (C)
Variable modifications: Oxidation (M)
Cleavage by Trypsin: cuts C-term side of KR unless next residue is P
Sequence Coverage: **11%**

Matched peptides shown in **Bold Red**

```
  1 MAEGERTECA EPPRDEPPAD GALKRAEELK TQANDYFKAK DYENAIKFYS
 51 QAIELNPSNA IYYGNRSLAY LRTECYGYAL GDATRAIELD KKYIKGYYRR
101 AASNMALGKF RAALRDYETV VKVKPHDKDA KMKYQECNKI VKQKAFERAI
151 AGDEHKRSVV DSLDIESMTI EDEYSGPKLE DGKVTISFMK ELMQWYKDQK
201 KLHRKCAYQI LVQVKEVLSK LSTLVETTLK ETEKITVCGD THGQFYDLLN
251 IFELNGLPSE TNPYIFNGDF VDRGSFSVEV ILTLFGFKLL YPDHFHLLRG
301 NHETDNMNQI YGFEGEVKAK YTAQMYELFS EVFEWLPLAQ CINGKVLIMH
351 GGLFSEDGVT LDDIRKIERN RQPPDSGPMC DLLWSDPQPQ NGRSISKRGV
401 SCQFGPDVTK AFLEENNLDY IIRSHEVKAE GYEVAHGGRC VTVFSAPNYC
451 DQMGNKASYI HLQGSDLRPQ FHQFTAVGRP SSGS
```

Residue Number Increasing Mass Decreasing Mass

Start - End	Observed	Mr(expt)	Mr(calc)	Delta	Miss	Sequence
48 - 66	2220.0452	2219.0379	2219.0646	-0.0267	0	FYSQAIELNPSNAIYYGNR (Ions score 78)
48 - 66	2220.0452	2219.0379	2219.0646	-0.0267	0	FYSQAIELNPSNAIYYGNR (No match)
73 - 85	1476.6277	1475.6204	1475.6350	-0.0146	0	TECYGYALGDATR (Ions score 36)
73 - 85	1476.6277	1475.6204	1475.6350	-0.0146	0	TECYGYALGDATR (No match)
289 - 299	1423.7689	1422.7616	1422.7772	-0.0155	0	LLYPDHFHLLR (No match)

289 - 299	1423.7689	1422.7616	1422.7772	-0.0155	0	LLYPDHFHLLR	(Ions score 13)
411 - 423	1609.8059	1608.7986	1608.8147	-0.0161	0	AFLEENNLDYIIR	(No match)
411 - 423	1609.8059	1608.7986	1608.8147	-0.0161	0	AFLEENNLDYIIR	(Ions score 40)

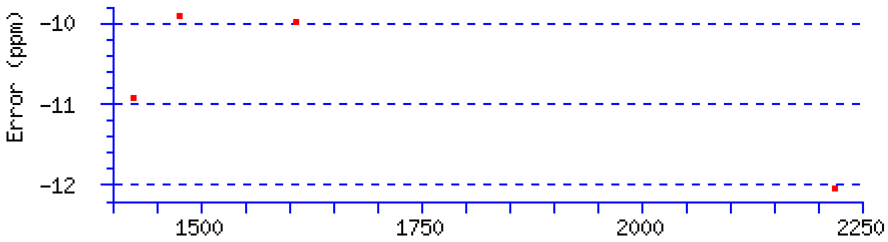

RMS error 10 ppm

Spot 365

Protein View

Match to: **gi|119576769** Score: **103** Expect: **9.6e-006**
seryl-tRNA synthetase, isoform CRA_a [Homo sapiens]

Nominal mass (M_r): **53844;** Calculated pI value: **5.98**
NCBI BLAST search of gi|119576769 against nr
Unformatted sequence string for pasting into other applications

Taxonomy: Homo sapiens

Fixed modifications: Carbamidomethyl (C)
Variable modifications: Oxidation (M)
Cleavage by Trypsin: cuts C-term side of KR unless next residue is P
Sequence Coverage: **15%**

Matched peptides shown in **Bold Red**

```
  1 MVLDLDLFRV DKGGDPALIR ETQEKRFKDP GLVDQLVKAD SEWRRCRFRA
 51 DNLNKLKNLC SKTIGEKMKK KEPVGDDESV PENVLSFDDL TADALANLKV
101 SQIKKVRLLI DEAILKCDAE RIKLEAERFE NLREIGNLLH PSVPISNDEG
151 VLVFLEQALI QYALRTLGSR GYIPIYTPFF MRKEVMQEVA QLSQFDEELY
201 KVIGKGSEKS DDNSYDEKYL IATSEQPIAA LHRDEWLRPE DLPIKYAGLS
251 TCFRQEVGSH GRDTRGIFRV HQFEKIEQFV YSSPHDNKSW EMFEEMITTA
301 EEFYQSLGIP YHIVNIVSGS LNHAASKKLD LEAWFPGSGA FRELVSCSNC
351 TDYQARRLRI RYGQTKKMMD KVEFVHMLNA TMCATTRTIC AILENYQTEK
401 GITVPEKLKE FMPPGLQELI PFVKPAPIEQ EPSKKQKKQH EGSKKKAAAR
451 DVTLENRLQN MEVTDA
```

Residue Number Increasing Mass Decreasing Mass

Start - End	Observed	Mr(expt)	Mr(calc)	Delta	Miss	Sequence
2 - 9	990.5684	989.5611	989.5545	0.0066	0	VLDLDLFR (No match)
27 - 38	1358.7543	1357.7470	1357.7605	-0.0135	1	FKDPGLVDQLVK (No match)
171 - 182	1504.7510	1503.7437	1503.7584	-0.0147	0	GYIPIYTPFFMR (No match)
171 - 182	1520.7465	1519.7392	1519.7533	-0.0141	0	GYIPIYTPFFMR Oxidation (M) (No match)
219 - 233	1682.9006	1681.8933	1681.9151	-0.0218	0	YLIATSEQPIAALHR (No match)
219 - 233	1682.9006	1681.8933	1681.9151	-0.0218	0	YLIATSEQPIAALHR (Ions score 51)
246 - 254	1074.5016	1073.4943	1073.4964	-0.0020	0	YAGLSTCFR (No match)

246 - 254	1074.5016	1073.4943	1073.4964	-0.0020	0	**YAGLSTCFR**	(No match)
343 - 356	1702.6899	1701.6826	1701.7086	-0.0260	0	**ELVSCSNCTDYQAR**	(No match)
343 - 356	1702.6899	1701.6826	1701.7086	-0.0260	0	**ELVSCSNCTDYQAR**	(No match)

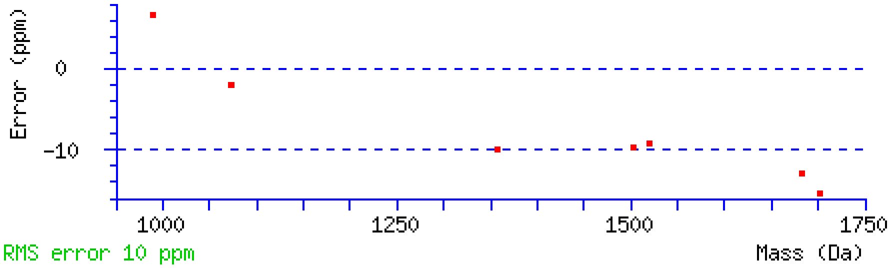

RMS error 10 ppm

Spot 366

Protein View

Match to: **gi|62898948** Score: **127** Expect: **3.8e-008**
tyrosyl-tRNA synthetase variant [Homo sapiens]

Nominal mass (M_r): **59467;** Calculated pI value: **6.79**
NCBI BLAST search of gi|62898948 against nr
Unformatted sequence string for pasting into other applications

Taxonomy: Homo sapiens

Fixed modifications: Carbamidomethyl (C)
Variable modifications: Oxidation (M)
Cleavage by Trypsin: cuts C-term side of KR unless next residue is P
Sequence Coverage: **18%**

Matched peptides shown in **Bold Red**

```
  1 MGDAPSPEEK LHLITRNLQE VLGEEKLKEI LKERELKIYW GTATTGKPHV
 51 AYFVPMSKIA DFLKAGCEVT ILFADLHAYL DNMKAPWELL ELRVSYYENV
101 IKAMLESIGV PLEKLKFIKG TDYQLSKEYT LDVYRLSSVV TQRDSKKAGA
151 EVVKQVEHPL LSGLLYPGLQ ALDEEYLKVD AQFGGIDQRK IFTFAEKYLP
201 ALGYSKRVHL MNPMVPGLTG SKMSSSEEES KIDLLDRKED VKKKLKKAFC
251 EPGNVENNGV LSFIKHVLFP LKSEFVILRD EKWGGNKTYT AYVDLEKDFA
301 AEVVHPGDLK NSVEVALNKL LDPIREKFNT PALKKLASAA YPDPSKQKPM
351 AKGPAKNSEP EEVIPSRLDI RVGKIITVEK HPDADSLYVE KIDVGEAEPR
401 TVVSGLVQFV PKEELQDRLV VVLCNLKPQK MRGVESQGML LCASIEGINR
451 QVEPLDPPAG SAPGEHVFVK GYEKGQPDEE LKPKKKVFEK LQADFKISEE
501 CIAQWKQTNF MTKLGSISCK SLKGGNIS
```

Residue Number Increasing Mass Decreasing Mass

Start - End	Observed	Mr(expt)	Mr(calc)	Delta	Miss	Sequence
128 - 135	1058.5457	1057.5384	1057.5080	0.0304	0	EYTLDVYR (No match)
128 - 135	1058.5457	1057.5384	1057.5080	0.0304	0	EYTLDVYR (No match)
179 - 189	1205.6238	1204.6165	1204.5836	0.0329	0	VDAQFGGIDQR (Ions score 13)
179 - 189	1205.6238	1204.6165	1204.5836	0.0329	0	VDAQFGGIDQR (No match)
266 - 279	1698.0583	1697.0510	1697.0028	0.0483	1	HVLFPLKSEFVILR (No match)
320 - 327	983.6022	982.5949	982.5811	0.0138	1	LLDPIREK (No match)

357 - 367	1256.6481	1255.6408	1255.6044	0.0364	0	**NSEPEEVIPSR** (No match)
357 - 367	1256.6481	1255.6408	1255.6044	0.0364	0	**NSEPEEVIPSR** (Ions score 19)
401 - 418	2044.1639	2043.1566	2043.1000	0.0567	1	**TVVSGLVQFVPKEELQDR** (No match)
401 - 418	2044.1639	2043.1566	2043.1000	0.0567	1	**TVVSGLVQFVPKEELQDR** (Ions score 14)
419 - 430	1410.8876	1409.8803	1409.8428	0.0376	0	**LVVVLCNLKPQK** (No match)
433 - 450	1949.9941	1948.9868	1948.9346	0.0523	0	**GVESQGMLLCASIEGINR** Oxidation (M) (No match)

Spot 368

Protein View

Match to: **gi|33286420** Score: **327** Expect: **3.8e-028**
pyruvate kinase 3 isoform 2 [Homo sapiens]

Nominal mass (M_r): **58538;** Calculated pI value: **7.60**
NCBI BLAST search of gi|33286420 against nr
Unformatted sequence string for pasting into other applications

Taxonomy: Homo sapiens
Links to retrieve other entries containing this sequence from NCBI Entrez:
gi|33286422 from Homo sapiens
gi|119598290 from Homo sapiens
gi|119598296 from Homo sapiens

Fixed modifications: Carbamidomethyl (C)
Variable modifications: Oxidation (M)
Cleavage by Trypsin: cuts C-term side of KR unless next residue is P
Sequence Coverage: **37%**

Matched peptides shown in **Bold Red**

```
  1 MSKPHSEAGT AFIQTQQLHA AMADTFLEHM CRLDIDSPPI TARNTGIICT
 51 IGPASRSVET LKEMIKSGMN VARLNFSHGT HEYHAETIKN VRTATESFAS
101 DPILYRPVAV ALDTKGPEIR TGLIKGSGTA EVELKKGATL KITLDNAYME
151 KCDENILWLD YKNICKVVEV GSKIYVDDGL ISLQVKQKGA DFLVTEVENG
201 GSLGSKKGVN LPGAAVDLPA VSEKDIQDLK FGVEQDVDMV FASFIRKASD
251 VHEVRKVLGE KGKNIKIISK IENHEGVRRF DEILEASDGI MVARGDLGIE
301 IPAEKVFLAQ KMMIGRCNRA GKPVICATQM LESMIKKPRP TRAEGSDVAN
351 AVLDGADCIM LSGETAKGDY PLEAVRMQHL IAREAEAAMF HRKLFEELVR
401 ASSHSTDLME AMAMGSVEAS YKCLAAALIV LTESGRSAHQ VARYRPRAPI
451 IAVTRNPQTA RQAHLYRGIF PVLCKDPVQE AWAEDVDLRV NFAMNVGKAR
501 GFFKKGDVVI VLTGWRPGSG FTNTMRVVPV P
```

Residue Number Increasing Mass Decreasing Mass

Start - End	Observed	Mr(expt)	Mr(calc)	Delta	Miss	Sequence	
33 - 43	1197.6235	1196.6162	1196.6401	-0.0239	0	LDIDSPPITAR	(No match)
33 - 43	1197.6235	1196.6162	1196.6401	-0.0239	0	LDIDSPPITAR	(Ions score 32)

44 - 56	1359.6766	1358.6693	1358.6976	-0.0283	0	NTGIICTIGPASR (No match)
44 - 56	1359.6766	1358.6693	1358.6976	-0.0283	0	NTGIICTIGPASR (Ions score 34)
74 - 89	1883.8643	1882.8570	1882.8961	-0.0391	0	LNFSHGTHEYHAETIK (No match)
74 - 89	1883.8643	1882.8570	1882.8961	-0.0391	0	LNFSHGTHEYHAETIK (Ions score 33)
93 - 115	2465.2375	2464.2302	2464.2849	-0.0547	0	TATESFASDPILYRPVAVALDTK (Ions score 13)
93 - 115	2465.2375	2464.2302	2464.2849	-0.0547	0	TATESFASDPILYRPVAVALDTK (No match)
174 - 186	1462.7849	1461.7776	1461.8078	-0.0302	0	IYVDDGLISLQVK (No match)
189 - 206	1779.8369	1778.8296	1778.8685	-0.0389	0	GADFLVTEVENGGSLGSK (No match)
231 - 246	1859.8584	1858.8511	1858.8923	-0.0412	0	FGVEQDVDMVFASFIR (No match)
231 - 246	1875.8558	1874.8485	1874.8872	-0.0387	0	FGVEQDVDMVFASFIR Oxidation (M) (No match)
271 - 278	953.4619	952.4546	952.4726	-0.0180	0	IENHEGVR (No match)
279 - 294	1837.8763	1836.8690	1836.9039	-0.0349	1	RFDEILEASDGIMVAR Oxidation (M) (No match)
320 - 336	1892.9285	1891.9212	1891.9569	-0.0357	0	AGKPVICATQMLESMIK Oxidation (M) (No match)
320 - 336	1908.9127	1907.9054	1907.9518	-0.0464	0	AGKPVICATQMLESMIK 2 Oxidation (M) (No match)
368 - 376	1019.4946	1018.4873	1018.5083	-0.0210	0	GDYPLEAVR (No match)
384 - 392	1061.4637	1060.4564	1060.4760	-0.0195	0	EAEAAMFHR (No match)
393 - 400	1033.5928	1032.5855	1032.5967	-0.0112	1	KLFEELVR (No match)
394 - 400	905.4927	904.4854	904.5018	-0.0163	0	LFEELVR (No match)
423 - 436	1473.7773	1472.7700	1472.8020	-0.0320	0	CLAAALIVLTESGR (Ions score 58)
423 - 436	1473.7773	1472.7700	1472.8020	-0.0320	0	CLAAALIVLTESGR (No match)
448 - 455	840.5133	839.5060	839.5228	-0.0168	0	APIIAVTR (No match)

Spot 370

Mascot Search Results

Protein View

Match to: **gi|34740335** Score: **465** Expect: **6.1e-042**
tubulin, alpha 1B [Mus musculus]

Nominal mass (M_r): **50804;** Calculated pI value: **4.94**
NCBI BLAST search of gi|34740335 against nr
Unformatted sequence string for pasting into other applications

Taxonomy: Mus musculus
Links to retrieve other entries containing this sequence from NCBI Entrez:
gi|57013276 from Homo sapiens
gi|112984124 from Rattus norvegicus
gi|113205626 from Sus scrofa
gi|109096464 from Macaca mulatta
gi|109096466 from Macaca mulatta
gi|109096468 from Macaca mulatta
gi|109096470 from Macaca mulatta
gi|109096472 from Macaca mulatta
gi|109096474 from Macaca mulatta
gi|109096476 from Macaca mulatta
gi|109096478 from Macaca mulatta
gi|109096480 from Macaca mulatta
gi|126343940 from Monodelphis domestica
gi|149632555 from Ornithorhynchus anatinus
gi|55976173 from Rattus norvegicus
gi|55977471 from Meriones unguiculatus
gi|55977472 from Cricetulus griseus
gi|55977474 from Homo sapiens
gi|55977764 from Mus musculus
gi|75075906 from Macaca fascicularis
gi|116256086 from Sus scrofa
gi|304530 from Cricetulus griseus
gi|3420929 from Homo sapiens
gi|4151197 from Meriones unguiculatus
gi|12653815 from Homo sapiens
gi|12654585 from Homo sapiens
gi|12805487 from Mus musculus
gi|13623541 from Homo sapiens

gi|13623707 from Homo sapiens
gi|14198110 from Mus musculus
gi|14250446 from Homo sapiens
gi|14424572 from Homo sapiens
gi|14550518 from Homo sapiens
gi|14550524 from Homo sapiens
gi|14550526 from Homo sapiens
gi|14714705 from Homo sapiens
gi|15079477 from Homo sapiens
gi|16198427 from Homo sapiens
gi|16877501 from Homo sapiens
gi|21410072 from Homo sapiens
gi|26344862 from Mus musculus
gi|38014707 from Rattus norvegicus
gi|39645091 from Mus musculus
gi|47938360 from Homo sapiens
gi|49903131 from Rattus norvegicus
gi|52789271 from Mus musculus
gi|67970890 from Macaca fascicularis
gi|68226670 from Mus musculus
gi|74139440 from Mus musculus
gi|74139472 from Mus musculus
gi|74139544 from Mus musculus
gi|74139677 from Mus musculus
gi|74142152 from Mus musculus
gi|74143974 from Mus musculus
gi|74202338 from Mus musculus
gi|74213777 from Mus musculus
gi|74213863 from Mus musculus
gi|74216755 from Mus musculus
gi|74220058 from Mus musculus
gi|80474380 from Mus musculus
gi|80478451 from Mus musculus
gi|81174755 from Sus scrofa
gi|89089750 from Homo sapiens
gi|90077354 from Macaca fascicularis
gi|119578452 from Homo sapiens
gi|123981190 from synthetic construct
gi|123996027 from synthetic construct
gi|148672206 from Mus musculus
gi|148878019 from Bos taurus

Fixed modifications: Carbamidomethyl (C)
Variable modifications: Oxidation (M)
Cleavage by Trypsin: cuts C-term side of KR unless next residue is P
Sequence Coverage: **49%**

Matched peptides shown in **Bold Red**

```
  1 MRECISIHVG QAGVQIGNAC WELYCLEHGI QPDGQMPSDK TIGGGDDSFN
 51 TFFSETGAGK HVPRAVFVDL EPTVIDEVRT GTYRQLFHPE QLITGKEDAA
101 NNYARGHYTI GKEIIDLVLD RIRKLADQCT GLQGFLVFHS FGGGTGSGFT
151 SLLMERLSVD YGKKSKLEFS IYPAPQVSTA VVEPYNSILT THTTLEHSDC
201 AFMVDNEAIY DICRRNLDIE RPTYTNLNRL ISQIVSSITA SLRFDGALNV
251 DLTEFQTNLV PYPRIHFPLA TYAPVISAEK AYHEQLSVAE ITNACFEPAN
301 QMVKCDPRHG KYMACCLLYR GDVVPKDVNA AIATIKTKRS IQFVDWCPTG
351 FKVGINYQPP TVVPGGDLAK VQRAVCMLSN TTAIAEAWAR LDHKFDLMYA
401 KRAFVHWYVG EGMEEGEFSE AREDMAALEK DYEEVGVDSV EGEGEEEGEE
451 Y
```

Residue Number Increasing Mass Decreasing Mass

Start - End	Observed	Mr(expt)	Mr(calc)	Delta	Miss	Sequence
41 - 60	2007.8831	2006.8758	2006.8857	-0.0098	0	TIGGGDDSFNTFFSETGAGK (No match)
65 - 79	1701.8901	1700.8828	1700.8984	-0.0156	0	AVFVDLEPTVIDEVR (No match)
65 - 79	1701.8901	1700.8828	1700.8984	-0.0156	0	AVFVDLEPTVIDEVR (Ions score 75)
85 - 96	1410.7563	1409.7490	1409.7666	-0.0176	0	QLFHPEQLITGK (No match)
85 - 105	2415.1804	2414.1731	2414.1978	-0.0247	1	QLFHPEQLITGKEDAANNYAR (No match)
97 - 105	1023.4401	1022.4328	1022.4417	-0.0089	0	EDAANNYAR (No match)
106 - 121	1841.9935	1840.9862	1841.0046	-0.0184	1	GHYTIGKEIIDLVLDR (No match)
113 - 121	1085.6084	1084.6011	1084.6128	-0.0117	0	EIIDLVLDR (No match)
216 - 229	1718.8701	1717.8628	1717.8747	-0.0119	0	NLDIERPTYTNLNR (No match)
216 - 229	1718.8701	1717.8628	1717.8747	-0.0119	0	NLDIERPTYTNLNR (Ions score 13)
230 - 243	1487.8658	1486.8585	1486.8718	-0.0133	0	LISQIVSSITASLR (No match)
230 - 243	1487.8658	1486.8585	1486.8718	-0.0133	0	LISQIVSSITASLR (Ions score 61)
244 - 264	2409.1780	2408.1707	2408.2011	-0.0304	0	FDGALNVDLTEFQTNLVPYPR (No match)
265 - 280	1756.9463	1755.9390	1755.9559	-0.0169	0	IHFPLATYAPVISAEK (No match)
265 - 280	1756.9463	1755.9390	1755.9559	-0.0169	0	IHFPLATYAPVISAEK (Ions score 64)
312 - 320	1249.5438	1248.5365	1248.5453	-0.0088	0	YMACCLLYR (No match)
312 - 320	1249.5438	1248.5365	1248.5453	-0.0088	0	YMACCLLYR (Ions score 28)
312 - 320	1265.5342	1264.5269	1264.5402	-0.0133	0	YMACCLLYR Oxidation (M) (No match)
340 - 352	1584.7322	1583.7249	1583.7442	-0.0193	0	SIQFVDWCPTGFK (No match)
353 - 370	1824.9662	1823.9589	1823.9781	-0.0191	0	VGINYQPPTVVPGGDLAK (No match)
374 - 390	1880.8772	1879.8699	1879.8920	-0.0221	0	AVCMLSNTTAIAEAWAR Oxidation (M) (No match)
391 - 401	1380.6862	1379.6789	1379.6907	-0.0118	1	LDHKFDLMYAK (No match)
391 - 401	1396.6887	1395.6814	1395.6856	-0.0042	1	LDHKFDLMYAK Oxidation (M) (No match)
403 - 422	2345.9956	2344.9883	2345.0058	-0.0174	0	AFVHWYVGEGMEEGEFSEAR Oxidation (M) (No match)

Spot 371

Mascot Search Results

Protein View

Match to: **gi|5902134** Score: **485** Expect: **6.1e-044**
coronin, actin binding protein, 1A [Homo sapiens]

Nominal mass (M_r): **51678;** Calculated pI value: **6.25**
NCBI BLAST search of gi|5902134 against nr
Unformatted sequence string for pasting into other applications

Taxonomy: Homo sapiens
Links to retrieve other entries containing this sequence from NCBI Entrez:
gi|1706004 from Homo sapiens
gi|20271119 from Homo sapiens
gi|927649 from Homo sapiens
gi|1136140 from Homo sapiens
gi|82571468 from Homo sapiens
gi|116497053 from Homo sapiens
gi|116497211 from Homo sapiens
gi|119600312 from Homo sapiens
gi|119600313 from Homo sapiens
gi|119600314 from Homo sapiens
gi|119600316 from Homo sapiens

Fixed modifications: Carbamidomethyl (C)
Variable modifications: Oxidation (M)
Cleavage by Trypsin: cuts C-term side of KR unless next residue is P
Sequence Coverage: **42%**

Matched peptides shown in **Bold Red**

1 MSRQVVRSSK **FRHVFGQPAK ADQCYEDVRV SQTTWDSGFC AVNPKFVALI**
51 **CEASGGGAFL VLPLGK**TGRV DKNAPTVCGH TAPVLDIAWC PHNDNVIASG
101 SEDCTVMVWE IPDGGLMLPL REPVVTLEGH TKRVGIVAWH TTAQNVLLSA
151 GCDNVIMVWD VGTGAAMLTL GPEVHPDTIY SVDWSR**DGGL ICTSCR**DKRV
201 RIIEPRK**GTV VAEKDRPHEG TRPVRAVFVS EGKILTTGFS R**MSER**QVALW**
251 **DTK**HLEEPLS LQELDTSSGV LLPFFDPDTN IVYLCGKGDS SIR**YFEITSE**
301 **APFLHYLSMF SSK**ESQRGMG YMPK**RGLEVN KCEIAR**FYKL HER**RCEPIAM**
351 **TVPRKSDLFQ EDLYPPTAGP DPALTAEEWL GGRDAGPLLI SLKDGYVPPK**
401 SRELRVNRGL DTGRRRAPE ASGTPSSDAV SRLEEEMRKL QATVQELQKR

451 LDRLEETVQA K

Residue Number Increasing Mass Decreasing Mass

Start - End	Observed	Mr(expt)	Mr(calc)	Delta	Miss	Sequence
11 - 20	1186.6122	1185.6049	1185.6406	-0.0357	1	FRHVFGQPAK (Ions score 34)
11 - 20	1186.6122	1185.6049	1185.6406	-0.0357	1	FRHVFGQPAK (No match)
13 - 20	883.4766	882.4693	882.4711	-0.0018	0	HVFGQPAK (No match)
13 - 29	2019.8672	2018.8599	2018.9268	-0.0669	1	HVFGQPAKADQCYEDVR (No match)
21 - 29	1155.4386	1154.4313	1154.4662	-0.0349	0	ADQCYEDVR (No match)
21 - 29	1155.4386	1154.4313	1154.4662	-0.0349	0	ADQCYEDVR (Ions score 19)
30 - 45	1796.7700	1795.7627	1795.8199	-0.0572	0	VSQTTWDSGFCAVNPK (Ions score 127)
30 - 45	1796.7700	1795.7627	1795.8199	-0.0572	0	VSQTTWDSGFCAVNPK (No match)
46 - 66	2119.0996	2118.0923	2118.1546	-0.0623	0	FVALICEASGGGAFLVLPLGK (No match)
187 - 196	1138.4634	1137.4561	1137.4906	-0.0345	0	DGGLICTSCR (No match)
208 - 225	2004.0129	2003.0056	2003.0660	-0.0604	1	GTVVAEKDRPHEGTRPVR (No match)
215 - 225	1319.6517	1318.6444	1318.6854	-0.0410	0	DRPHEGTRPVR (Ions score 39)
215 - 225	1319.6517	1318.6444	1318.6854	-0.0410	0	DRPHEGTRPVR (No match)
226 - 233	836.4249	835.4176	835.4439	-0.0263	0	AVFVSEGK (No match)
226 - 241	1711.8851	1710.8778	1710.9304	-0.0525	1	AVFVSEGKILTTGFSR (No match)
234 - 241	894.4769	893.4696	893.4970	-0.0274	0	ILTTGFSR (No match)
246 - 253	960.4816	959.4743	959.5076	-0.0333	0	QVALWDTK (No match)
294 - 313	2397.0647	2396.0574	2396.1398	-0.0824	0	YFEITSEAPFLHYLSMFSSK (No match)
294 - 313	2413.0645	2412.0572	2412.1347	-0.0775	0	YFEITSEAPFLHYLSMFSSK Oxidation (M) (No match)
325 - 331	815.4540	814.4467	814.4660	-0.0193	1	RGLEVNK (No match)
326 - 336	1288.6333	1287.6260	1287.6604	-0.0344	1	GLEVNKCEIAR (No match)
344 - 354	1329.6356	1328.6283	1328.6693	-0.0410	1	RCEPIAMTVPR (Ions score 23)
344 - 354	1329.6356	1328.6283	1328.6693	-0.0410	1	RCEPIAMTVPR (No match)
344 - 354	1345.6300	1344.6227	1344.6642	-0.0415	1	RCEPIAMTVPR Oxidation (M) (No match)
345 - 354	1173.5364	1172.5291	1172.5682	-0.0391	0	CEPIAMTVPR (No match)
345 - 354	1189.5408	1188.5335	1188.5631	-0.0296	0	CEPIAMTVPR Oxidation (M) (No match)
355 - 383	3173.4106	3172.4033	3172.5352	-0.1319	1	KSDLFQEDLYPPTAGPDPALTAEEWLGGR (No match)
384 - 393	1026.5854	1025.5781	1025.6120	-0.0339	0	DAGPLLISLK (No match)
384 - 400	1782.9478	1781.9405	1781.9927	-0.0521	1	DAGPLLISLKDGYVPPK (No match)

Mascot: http://www.matrixscience.com/

Spot 372

{MATRIX}{SCIENCE} Mascot Search Results

Protein View

Match to: **gi|5902134** Score: **476** Expect: **4.8e-043**
coronin, actin binding protein, 1A [Homo sapiens]

Nominal mass (M_r): **51678**; Calculated pI value: **6.25**
NCBI BLAST search of gi|5902134 against nr
Unformatted sequence string for pasting into other applications

Taxonomy: Homo sapiens
Links to retrieve other entries containing this sequence from NCBI Entrez:
gi|1706004 from Homo sapiens
gi|20271119 from Homo sapiens
gi|927649 from Homo sapiens
gi|1136140 from Homo sapiens
gi|82571468 from Homo sapiens
gi|116497053 from Homo sapiens
gi|116497211 from Homo sapiens
gi|119600312 from Homo sapiens
gi|119600313 from Homo sapiens
gi|119600314 from Homo sapiens
gi|119600316 from Homo sapiens

Fixed modifications: Carbamidomethyl (C)
Variable modifications: Oxidation (M)
Cleavage by Trypsin: cuts C-term side of KR unless next residue is P
Sequence Coverage: **40%**

Matched peptides shown in **Bold Red**

```
  1 MSRQVVRSSK FRHVFGQPAK ADQCYEDVRV SQTTWDSGFC AVNPKFVALI
 51 CEASGGGAFL VLPLGKTGRV DKNAPTVCGH TAPVLDIAWC PHNDNVIASG
101 SEDCTVMVWE IPDGGLMLPL REPVVTLEGH TKRVGIVAWH TTAQNVLLSA
151 GCDNVIMVWD VGTGAAMLTL GPEVHPDTIY SVDWSRDGGL ICTSCRDKRV
201 RIIEPRKGTV VAEKDRPHEG TRPVRAVFVS EGKILTTGFS RMSERQVALW
251 DTKHLEEPLS LQELDTSSGV LLPFFDPDTN IVYLCGKGDS SIRYFEITSE
301 APFLHYLSMF SSKESQRGMG YMPKRGLEVN KCEIARFYKL HERRCEPIAM
351 TVPRKSDLFQ EDLYPPTAGP DPALTAEEWL GGRDAGPLLI SLKDGYVPPK
401 SRELRVNRGL DTGRRRAAPE ASGTPSSDAV SRLEEEMRKL QATVQELQKR
```

451 LDRLEETVQA K

Residue Number Increasing Mass Decreasing Mass

Start - End	Observed	Mr(expt)	Mr(calc)	Delta	Miss	Sequence
11 - 20	1186.6041	1185.5968	1185.6406	-0.0438	1	FRHVFGQPAK (No match)
11 - 20	1186.6041	1185.5968	1185.6406	-0.0438	1	FRHVFGQPAK (Ions score 37)
13 - 29	2019.8557	2018.8484	2018.9268	-0.0784	1	HVFGQPAKADQCYEDVR (No match)
21 - 29	1155.4316	1154.4243	1154.4662	-0.0419	0	ADQCYEDVR (Ions score 15)
21 - 29	1155.4316	1154.4243	1154.4662	-0.0419	0	ADQCYEDVR (No match)
30 - 45	1796.7573	1795.7500	1795.8199	-0.0699	0	VSQTTWDSGFCAVNPK (No match)
30 - 45	1796.7573	1795.7500	1795.8199	-0.0699	0	VSQTTWDSGFCAVNPK (Ions score 113)
46 - 66	2119.0884	2118.0811	2118.1546	-0.0735	0	FVALICEASGGGAFLVLPLGK (No match)
187 - 196	1138.4559	1137.4486	1137.4906	-0.0420	0	DGGLICTSCR (Ions score 43)
187 - 196	1138.4559	1137.4486	1137.4906	-0.0420	0	DGGLICTSCR (No match)
200 - 206	882.5228	881.5155	881.5446	-0.0291	1	VRIIEPR (No match)
208 - 225	2003.9985	2002.9912	2003.0660	-0.0748	1	GTVVAEKDRPHEGTRPVR (No match)
215 - 225	1319.6390	1318.6317	1318.6854	-0.0537	0	DRPHEGTRPVR (Ions score 63)
215 - 225	1319.6390	1318.6317	1318.6854	-0.0537	0	DRPHEGTRPVR (No match)
234 - 241	894.4703	893.4630	893.4970	-0.0340	0	ILTTGFSR (No match)
246 - 253	960.4738	959.4665	959.5076	-0.0411	0	QVALWDTK (No match)
294 - 313	2397.0564	2396.0491	2396.1398	-0.0907	0	YFEITSEAPFLHYLSMFSSK (No match)
294 - 313	2413.0557	2412.0484	2412.1347	-0.0863	0	YFEITSEAPFLHYLSMFSSK Oxidation (M) (No match)
326 - 336	1288.6208	1287.6135	1287.6604	-0.0469	1	GLEVNKCEIAR (No match)
344 - 354	1329.6248	1328.6175	1328.6693	-0.0518	1	RCEPIAMTVPR (No match)
344 - 354	1345.6190	1344.6117	1344.6642	-0.0525	1	RCEPIAMTVPR Oxidation (M) (No match)
345 - 354	1173.5228	1172.5155	1172.5682	-0.0527	0	CEPIAMTVPR (No match)
345 - 354	1189.5342	1188.5269	1188.5631	-0.0362	0	CEPIAMTVPR Oxidation (M) (No match)
355 - 383	3173.4048	3172.3975	3172.5352	-0.1377	1	KSDLFQEDLYPPTAGPDPALTAEEWLGGR (No match)
384 - 393	1026.5742	1025.5669	1025.6120	-0.0451	0	DAGPLLISLK (No match)

Mascot: http://www.matrixscience.com/

Spot 373

Mascot Search Results

Protein View

Match to: **gi|23510364** Score: **331** Expect: **1.5e-028**
protein-tyrosine kinase fyn isoform c [Homo sapiens]

Nominal mass (M_r): **54821**; Calculated pI value: **5.85**
NCBI BLAST search of gi|23510364 against nr
Unformatted sequence string for pasting into other applications

Taxonomy: Homo sapiens
Links to retrieve other entries containing this sequence from NCBI Entrez:
gi|114608948 from Pan troglodytes
gi|114608950 from Pan troglodytes
gi|21618480 from Homo sapiens

Fixed modifications: Carbamidomethyl (C)
Variable modifications: Oxidation (M)
Cleavage by Trypsin: cuts C-term side of KR unless next residue is P
Sequence Coverage: **27%**

Matched peptides shown in **Bold Red**

```
  1 MGCVQCKDKE ATKLTEERDG SLNQSSGYRY GTDPTPQHYP SFGVTSIPNY
 51 NNFHAAGGQG LTVFGGVNSS SHTGTLRTRG GTGVTLFVAL YDYEARTEDD
101 LSFHKGEKFQ ILNSSEGDWW EARSLTTGET GYIPSNYVAP VDSIQAEEWY
151 FGKLGRKDAE RQLLSFGNPR GTFLIRESET TKGAYSLSIR DWDDMKGDHV
201 KHYKIRKLDN GGYYITTRAQ FETLQQLVQH YSGTWNGNTK VAIKTLKPGT
251 MSPESFLEEA QIMKKLKHDK LVQLYAVVSE EPIYIVTEYM NKGSLLDFLK
301 DGEGRALKLP NLVDMAAQVA AGMAYIERMN YIHRDLRSAN ILVGNGLICK
351 IADFGLARLI EDNEYTARQG AKFPIKWTAP EAALYGRFTI KSDVWSFGIL
401 LTELVTKGRV PYPGMNNREV LEQVERGYRM PCPQDCPISL HELMIHCWKK
451 DPEERPTFEY LQSFLEDYFT ATEPQYQPGE NL
```

Residue Number Increasing Mass Decreasing Mass

Start - End	Observed	Mr(expt)	Mr(calc)	Delta	Miss	Sequence
19 - 29	1183.5304	1182.5231	1182.5265	-0.0034	0	DGSLNQSSGYR (No match)
80 - 96	1831.8999	1830.8926	1830.9151	-0.0225	0	GGTGVTLFVALYDYEAR (No match)
109 - 123	1837.8334	1836.8261	1836.8430	-0.0169	0	FQILNSSEGDWWEAR (No match)
162 - 170	1031.5551	1030.5478	1030.5559	-0.0081	0	QLLSFGNPR (Ions score 21)
162 - 170	1031.5551	1030.5478	1030.5559	-0.0081	0	QLLSFGNPR (No match)
183 - 190	866.4777	865.4704	865.4657	0.0047	0	GAYSLSIR (No match)
207 - 218	1400.7073	1399.7000	1399.7095	-0.0095	1	KLDNGGYYITTR (Ions score 35)
207 - 218	1400.7073	1399.7000	1399.7095	-0.0095	1	KLDNGGYYITTR (No match)
208 - 218	1272.6112	1271.6039	1271.6146	-0.0106	0	LDNGGYYITTR (No match)
293 - 305	1406.6534	1405.6461	1405.7201	-0.0739	1	GSLLDFLKDGEGR (No match)
351 - 358	862.4677	861.4604	861.4708	-0.0104	0	IADFGLAR (No match)
359 - 368	1223.5811	1222.5738	1222.5829	-0.0091	0	LIEDNEYTAR (Ions score 39)
359 - 368	1223.5811	1222.5738	1222.5829	-0.0091	0	LIEDNEYTAR (No match)
377 - 387	1234.6132	1233.6059	1233.6142	-0.0082	0	WTAPEAALYGR (No match)
377 - 387	1234.6132	1233.6059	1233.6142	-0.0082	0	WTAPEAALYGR (Ions score 66)
408 - 418	1276.6095	1275.6022	1275.6142	-0.0119	1	GRVPYPGMNNR Oxidation (M) (No match)
410 - 418	1063.4910	1062.4837	1062.4916	-0.0079	0	VPYPGMNNR Oxidation (M) (No match)
419 - 426	1001.5156	1000.5083	1000.5189	-0.0105	0	EVLEQVER (No match)

Mascot: http://www.matrixscience.com/

Spot 374

Mascot Search Results

Protein View

Match to: **gi|1002923** Score: **145** Expect: **6.1e-010**
coronin-like protein

Nominal mass (M_r): **51722**; Calculated pI value: **6.12**
NCBI BLAST search of gi|1002923 against nr
Unformatted sequence string for pasting into other applications

Taxonomy: Homo sapiens

Fixed modifications: Carbamidomethyl (C)
Variable modifications: Oxidation (M)
Cleavage by Trypsin: cuts C-term side of KR unless next residue is P
Sequence Coverage: **22%**

Matched peptides shown in **Bold Red**

```
  1 MSRQVVRTSK FRHVFGQPAK ADQCYEDVRV SQTTWDSGFC AVNPKFVALI
 51 CEASGGGAFL VLPLGKTGRV DKNAPTVCGH TAPVLDIAWC PHNDNVIASG
101 SEDCTVMVWE IPDGGLMLPL REPVVTLEGH TKRVGIVAWH TTAQNVLLSA
151 GCDNVIMVWD VGTGAAMLTL GPEVHPDTIY SVDWSRDGGL ICTSCRDKRV
201 RIIEPRKGTV VAEKDRPHEG TRPVRAVFVS EGKILTTGFS RMSEWQVALW
251 DTKHLEEPLS LQELDTSSGV LLPFFDPDTN IVYLCGKGDS SIRYFEITSE
301 APFLHYLSMF SSKESQRGMG YMPKRGLEVN KCEIARFYKL HERRCEPIAM
351 TVPRKSDLFQ EDLYPPTAGP DPALTAEEWL GGRDAGPLLI SLKDGYVPPK
401 SRELRVNRGL DTGRRRAAPE ASGTPSSDAV SRLEEEMRKL QATVQELQKR
451 LDRLEETVQA K
```

Residue Number Increasing Mass Decreasing Mass

Start - End	Observed	Mr(expt)	Mr(calc)	Delta	Miss	Sequence	
11 - 20	1186.6057	1185.5984	1185.6406	-0.0422	1	FRHVFGQPAK	(No match)
11 - 20	1186.6057	1185.5984	1185.6406	-0.0422	1	FRHVFGQPAK	(Ions score 15)
21 - 29	1155.4320	1154.4247	1154.4662	-0.0415	0	ADQCYEDVR	(No match)

21 - 29	1155.4320	1154.4247	1154.4662	-0.0415	0	ADQCYEDVR	(No match)
30 - 45	1796.7594	1795.7521	1795.8199	-0.0678	0	VSQTTWDSGFCAVNPK	(No match)
46 - 66	2119.0823	2118.0750	2118.1546	-0.0796	0	FVALICEASGGGAFLVLPLGK	(No match)
187 - 196	1138.4564	1137.4491	1137.4906	-0.0415	0	DGGLICTSCR	(Ions score 16)
187 - 196	1138.4564	1137.4491	1137.4906	-0.0415	0	DGGLICTSCR	(No match)
200 - 206	882.5237	881.5164	881.5446	-0.0282	1	VRIIEPR	(No match)
215 - 225	1319.6445	1318.6372	1318.6854	-0.0482	0	DRPHEGTRPVR	(Ions score 27)
215 - 225	1319.6445	1318.6372	1318.6854	-0.0482	0	DRPHEGTRPVR	(No match)
234 - 241	894.4770	893.4697	893.4970	-0.0273	0	ILTTGFSR	(No match)
344 - 354	1329.6298	1328.6225	1328.6693	-0.0468	1	RCEPIAMTVPR	(No match)
344 - 354	1345.6191	1344.6118	1344.6642	-0.0524	1	RCEPIAMTVPR	Oxidation (M) (No match)
345 - 354	1173.5289	1172.5216	1172.5682	-0.0466	0	CEPIAMTVPR	(No match)

Mascot: http://www.matrixscience.com/

Spot 375

Mascot Search Results

Protein View

Match to: **gi|1002923** Score: **257** Expect: **3.8e-021**
coronin-like protein

Nominal mass (M_r): **51722**; Calculated pI value: **6.12**
NCBI BLAST search of gi|1002923 against nr
Unformatted sequence string for pasting into other applications

Taxonomy: Homo sapiens

Fixed modifications: Carbamidomethyl (C)
Variable modifications: Oxidation (M)
Cleavage by Trypsin: cuts C-term side of KR unless next residue is P
Sequence Coverage: **35%**

Matched peptides shown in **Bold Red**

1 MSRQVVRTSK **FRHVFGQPAK ADQCYEDVRV SQTTWDSGFC AVNPKFVALI**
51 **CEASGGGAFL VLPLGK**TGRV DKNAPTVCGH TAPVLDIAWC PHNDNVIASG
101 SEDCTVMVWE IPDGGLMLPL REPVVTLEGH TKRVGIVAWH TTAQNVLLSA
151 GCDNVIMVWD VGTGAAMLTL GPEVHPDTIY SVDWSR**DGGL ICTSCR**DKRV
201 RIIEPRKGTV VAEK**DRPHEG TRPVRAVFVS EGKILTTGFS R**MSEWQVALW
251 DTKHLEEPLS LQELDTSSGV LLPFFDPDTN IVYLCGKGDS SIR**YFEITSE**
301 **APFLHYLSMF SSK**ESQRGMG YMPKR**GLEVN KCEIARFYKL HERRCEPIAM**
351 **TVPR**KSDLFQ EDLYPPTAGP DPALTAEEWL GGR**DAGPLLI SLKDGYVPPK**
401 SR**ELRVNR**GL DTGRRRAAPE ASGTPSSDAV SRLEEEMRKL QATVQELQKR
451 LDRLEETVQA K

Residue Number Increasing Mass Decreasing Mass

Start - End	Observed	Mr(expt)	Mr(calc)	Delta	Miss	Sequence
11 - 20	1186.6298	1185.6225	1185.6406	-0.0181	1	FRHVFGQPAK (Ions score 31)
11 - 20	1186.6298	1185.6225	1185.6406	-0.0181	1	FRHVFGQPAK (No match)
13 - 29	2019.8809	2018.8736	2018.9268	-0.0532	1	HVFGQPAKADQCYEDVR (No match)

13 - 29	2019.8809	2018.8736	2018.9268	-0.0532	1	**HVFGQPAKADQCYEDVR** (Ions score 27)
21 - 29	1155.4609	1154.4536	1154.4662	-0.0126	0	**ADQCYEDVR** (No match)
30 - 45	1796.7877	1795.7804	1795.8199	-0.0395	0	**VSQTTWDSGFCAVNPK** (No match)
46 - 66	2119.1243	2118.1170	2118.1546	-0.0376	0	**FVALICEASGGGAFLVLPLGK** (No match)
187 - 196	1138.4861	1137.4788	1137.4906	-0.0118	0	**DGGLICTSCR** (No match)
215 - 225	1319.6674	1318.6601	1318.6854	-0.0253	0	**DRPHEGTRPVR** (No match)
215 - 225	1319.6674	1318.6601	1318.6854	-0.0253	0	**DRPHEGTRPVR** (Ions score 19)
226 - 241	1711.9001	1710.8928	1710.9304	-0.0376	1	**AVFVSEGKILTTGFSR** (No match)
234 - 241	894.4996	893.4923	893.4970	-0.0047	0	**ILTTGFSR** (No match)
294 - 313	2413.0923	2412.0850	2412.1347	-0.0497	0	**YFEITSEAPFLHYLSMFSSK** Oxidation (M) (No match)
326 - 336	1288.6512	1287.6439	1287.6604	-0.0165	1	**GLEVNKCEIAR** (No match)
337 - 343	992.5230	991.5157	991.5239	-0.0082	1	**FYKLHER** (No match)
344 - 354	1329.6580	1328.6507	1328.6693	-0.0186	1	**RCEPIAMTVPR** (No match)
344 - 354	1345.6443	1344.6370	1344.6642	-0.0272	1	**RCEPIAMTVPR** Oxidation (M) (No match)
384 - 400	1782.9576	1781.9503	1781.9927	-0.0423	1	**DAGPLLISLKDGYVPPK** (No match)
403 - 408	786.4523	785.4450	785.4507	-0.0057	1	**ELRVNR** (No match)

Mascot: http://www.matrixscience.com/

Spot 376

Mascot Search Results

Protein View

Match to: **gi|5453607** Score: **237** Expect: **3.8e-019**
chaperonin containing TCP1, subunit 7 isoform a [Homo sapiens]

Nominal mass (M_r): **59842;** Calculated pI value: **7.55**
NCBI BLAST search of gi|5453607 against nr
Unformatted sequence string for pasting into other applications

Taxonomy: Homo sapiens
Links to retrieve other entries containing this sequence from NCBI Entrez:
gi|3041738 from Homo sapiens
gi|2559010 from Homo sapiens
gi|17939554 from Homo sapiens
gi|49168508 from Homo sapiens
gi|56789228 from Homo sapiens
gi|62630157 from Homo sapiens
gi|119620145 from Homo sapiens
gi|123993577 from synthetic construct
gi|123999578 from synthetic construct
gi|158257354 (no taxonomy information for this entry)

Fixed modifications: Carbamidomethyl (C)
Variable modifications: Oxidation (M)
Cleavage by Trypsin: cuts C-term side of KR unless next residue is P
Sequence Coverage: **19%**

Matched peptides shown in **Bold Red**

1 MMPTPVILLK **EGTDSSQGIP QLVSNISACQ VIAEAVR**TTL GPRGMDKLIV
51 DGRGKATISN DGATILKLLD VVHPAAKTLV DIAKSQDAEV GDGTTSVTLL
101 AAEFLK**QVKP YVEEGLHPQI IIR**AFRTATQ LAVNKIKEIA VTVKKADKVE
151 QRKLLEKCAM TALSSKLISQ QKAFFAKMVV DAVMMLDDLL QLKMIGIKKV
201 QGGALEDSQL VAGVAFKK**TF SYAGFEMQPK** KYHNPKIALL NVELELKAEK
251 DNAEIRVHTV EDYQAIVDAE WNILYDKLEK IHHSGAKVVL SK**LPIGDVAT**
301 **QYFADR**DMFC AGRVPEEDLK RTMMACGGSI QTSVNALSAD VLGR**CQVFEE**
351 **TQIGGERYNF FTGCPK**AKTC TFILR**GGAEQ FMEETER**SLH DAIMIVRRAI
401 KNDSVVAGGG AIEMELSKYL RDYSRTIPGK QQLLIGAYAK ALEIIPRQLC
451 DNAGFDATNI LNKLRARHAQ GGTWYGVDIN NEDIADNFEA FVWEPAMVRI

501 NALTAASEAA CLIVSVDETI KNPRSTVDAP TAAGRGRGRG RPH

Residue Number Increasing Mass Decreasing Mass

Start - End	Observed	Mr(expt)	Mr(calc)	Delta	Miss	Sequence
11 - 37	2829.4092	2828.4019	2828.3973	0.0046	0	EGTDSSQGIPQLVSNISACQVIAEAVR (No match)
11 - 37	2829.4092	2828.4019	2828.3973	0.0046	0	EGTDSSQGIPQLVSNISACQVIAEAVR (Ions score 103)
107 - 123	2019.1372	2018.1299	2018.1312	-0.0013	0	QVKPYVEEGLHPQIIIR (No match)
107 - 123	2019.1372	2018.1299	2018.1312	-0.0013	0	QVKPYVEEGLHPQIIIR (Ions score 23)
219 - 230	1421.6344	1420.6271	1420.6332	-0.0061	0	TFSYAGFEMQPK Oxidation (M) (No match)
293 - 306	1565.7937	1564.7864	1564.7885	-0.0021	0	LPIGDVATQYFADR (No match)
293 - 306	1565.7937	1564.7864	1564.7885	-0.0021	0	LPIGDVATQYFADR (Ions score 42)
345 - 357	1552.6946	1551.6873	1551.6987	-0.0114	0	CQVFEETQIGGER (No match)
358 - 366	1133.5026	1132.4953	1132.5011	-0.0058	0	YNFFTGCPK (No match)
376 - 387	1399.5760	1398.5687	1398.5721	-0.0034	0	GGAEQFMEETER Oxidation (M) (No match)

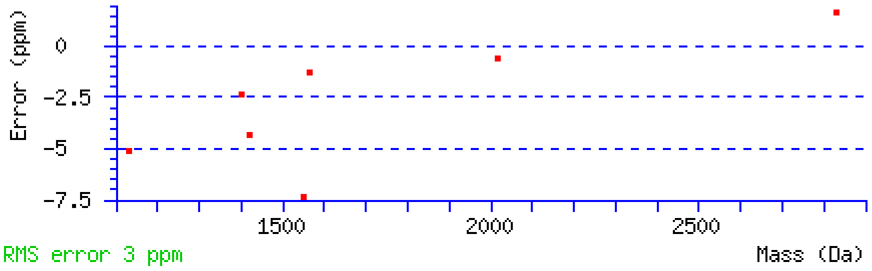

Mascot: http://www.matrixscience.com/

Spot 377

Mascot Search Results

Protein View

Match to: **gi|48146451** Score: **191** Expect: **1.5e-014**
HIP-55 [Homo sapiens]

Nominal mass (M_r): **48449**; Calculated pI value: **5.01**
NCBI BLAST search of gi|48146451 against nr
Unformatted sequence string for pasting into other applications

Taxonomy: Homo sapiens

Fixed modifications: Carbamidomethyl (C)
Variable modifications: Oxidation (M)
Cleavage by Trypsin: cuts C-term side of KR unless next residue is P
Sequence Coverage: **28%**

Matched peptides shown in **Bold Red**

```
  1 MAANLSRNGP ALQEAYVRVV TEKSPTDWAL FTYEGNSNDI RVAGTGEGGL
 51 EEMVEELNSG KVMYAFCRVK DPNSGLPKFV LINWTGEGVN DVRKGACASH
101 VSTMASFLKG AHVTINARAE EDVEPECIME KVAKASGANY SFHKESGRFQ
151 DVGPQAPVGS VYQKTNAVSE IKRVGKDSFW AKAEKEEENR RLEEKRRAEE
201 AQRQLEQERR ERELREAARR EQRYQEQGGE ASPQRTWEQQ QEVVSRNRNE
251 QESAVHPREI FKQKERAMST TSISSPQPGK LRSPFLQKQL TQPETHFGRE
301 PAAAISRPRA DLPAEEPAPS TPPCLVQAEE EAVYEEPPEQ ETFYEQPPLV
351 QQQGAGSEHI DHHIQGQGLS GQGLCARALY DYQAADDTEI SFDPENLITG
401 IEVIDEGWWR GYGPDGHFGM FPANYVELID
```

Residue Number Increasing Mass Decreasing Mass

Start - End	Observed	Mr(expt)	Mr(calc)	Delta	Miss	Sequence
8 - 18	1217.6130	1216.6057	1216.6200	-0.0142	0	NGPALQEAYVR (Ions score 53)
8 - 18	1217.6130	1216.6057	1216.6200	-0.0142	0	NGPALQEAYVR (No match)
42 - 61	2021.9276	2020.9203	2020.9258	-0.0055	0	VAGTGEGGLEEMVEELNSGK Oxidation (M) (No match)
62 - 68	962.4039	961.3966	961.4149	-0.0183	0	VMYAFCR Oxidation (M) (No match)

79 - 93	1718.8713	1717.8640	1717.8787	-0.0147	0	FVLINWTGEGVNDVR (Ions score 0)
79 - 93	1718.8713	1717.8640	1717.8787	-0.0147	0	FVLINWTGEGVNDVR (No match)
79 - 94	1846.9576	1845.9503	1845.9736	-0.0233	1	FVLINWTGEGVNDVRK (No match)
110 - 118	938.5029	937.4956	937.5093	-0.0137	0	GAHVTINAR (No match)
204 - 210	958.4918	957.4845	957.4991	-0.0146	1	QLEQERR (No match)
224 - 235	1349.5956	1348.5883	1348.6007	-0.0124	0	YQEQGGEASPQR (No match)
247 - 258	1436.6774	1435.6701	1435.6916	-0.0215	1	NRNEQESAVHPR (No match)
281 - 288	988.5743	987.5670	987.5865	-0.0195	1	LRSPFLQK (No match)
289 - 299	1313.6486	1312.6413	1312.6524	-0.0110	0	QLTQPETHFGR (No match)
289 - 309	2362.2241	2361.2168	2361.2301	-0.0133	1	QLTQPETHFGREPAAAISRPR (Ions score 31)
289 - 309	2362.2241	2361.2168	2361.2301	-0.0133	1	QLTQPETHFGREPAAAISRPR (No match)
300 - 309	1067.5857	1066.5784	1066.5883	-0.0099	0	EPAAAISRPR (No match)

Mascot: http://www.matrixscience.com/

Spot 378

Mascot Search Results

Protein View

Match to: **gi|119597640** Score: **138** Expect: **3e-009**
protein disulfide isomerase family A, member 3, isoform CRA_a [Homo sapiens]

Nominal mass (M_r): **54454**; Calculated pI value: **6.78**
NCBI BLAST search of gi|119597640 against nr
Unformatted sequence string for pasting into other applications

Taxonomy: Homo sapiens

Fixed modifications: Carbamidomethyl (C)
Variable modifications: Oxidation (M)
Cleavage by Trypsin: cuts C-term side of KR unless next residue is P
Sequence Coverage: **9%**

Matched peptides shown in **Bold Red**

```
  1 MRLRRLALFP GVALLLAAAR LAAASDFFAP WCGHCKRLAP EYEAAATRLK
 51 GIVPLAKVDC TANTNTCNKY GVSGYPTLKI FRDGEEAGAY DGPRTADGIV
101 SHLKKQAGPA SVPLRTEEEF KKFISDKDAS IVGFFDDSFS EAHSEFLKAA
151 SNLRDNYRFA HTNVESLVNE YDDNGEGIIL FRPSHLTNKF EDKTVAYTEQ
201 KMTSGKIKKF IQENIFGICP HMTEDNKDLI QGKDLLIAYY DVDYEKNAKG
251 SNYWRNRVMM VAKKFLDAGH KLNFAVASRK TFSHELSDFG LESTAGEIPV
301 VAIRTAKGEK FVMQEEFSRD GKALERFLQD YFDGNLKRYL KSEPIPESND
351 GPVKVVVAEN FDEIVNNENK DVLIEFYAPW CGHCKNLEPK YKELGEKLSK
401 DPNIVIAKMD ATANDVPSPY EVRGFPTIYF SPANKKLNPK KYEGGRELSD
451 FISYLQREAT NPPVIQEEKP KKKKKAQEDL
```

Residue Number Increasing Mass Decreasing Mass

Start - End	Observed	Mr(expt)	Mr(calc)	Delta	Miss	Sequence
190 - 201	1458.6825	1457.6752	1457.7038	-0.0285	1	**FEDKTVAYTEQK** (No match)
190 - 201	1458.6825	1457.6752	1457.7038	-0.0285	1	**FEDKTVAYTEQK** (No match)
311 - 319	1188.4875	1187.4802	1187.5280	-0.0478	0	**FVMQEEFSR** Oxidation (M) (No match)

327 - 338	1515.7050	1514.6977	1514.7517	-0.0540	1	FLQDYFDGNLKR	(No match)
327 - 338	1515.7050	1514.6977	1514.7517	-0.0540	1	FLQDYFDGNLKR	(Ions score 65)
447 - 457	1370.6478	1369.6405	1369.6877	-0.0472	0	ELSDFISYLQR	(Ions score 42)
447 - 457	1370.6478	1369.6405	1369.6877	-0.0472	0	ELSDFISYLQR	(No match)

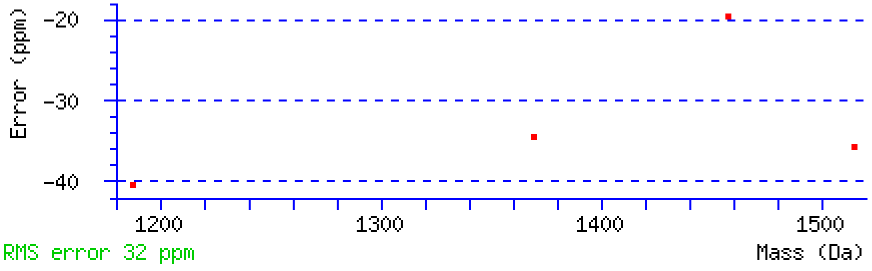

Mascot: http://www.matrixscience.com/

Spot 379

MATRIX SCIENCE Mascot Search Results

Protein View

Match to: **gi|119597640** Score: **443** Expect: **9.6e-040**
protein disulfide isomerase family A, member 3, isoform CRA_a [Homo sapiens]

Nominal mass (M_r): **54454**; Calculated pI value: **6.78**
NCBI BLAST search of gi|119597640 against nr
Unformatted sequence string for pasting into other applications

Taxonomy: Homo sapiens

Fixed modifications: Carbamidomethyl (C)
Variable modifications: Oxidation (M)
Cleavage by Trypsin: cuts C-term side of KR unless next residue is P
Sequence Coverage: **28%**

Matched peptides shown in **Bold Red**

```
  1 MRLRRLALFP GVALLLAAAR LAAASDFFAP WCGHCKRLAP EYEAAATRLK
 51 GIVPLAKVDC TANTNTCNKY GVSGYPTLKI FRDGEEAGAY DGPRTADGIV
101 SHLKKQAGPA SVPLRTEEEF KKFISDKDAS IVGFFDDSFS EAHSEFLKAA
151 SNLRDNYRFA HTNVESLVNE YDDNGEGIIL FRPSHLTNKF EDKTVAYTEQ
201 KMTSGKIKKF IQENIFGICP HMTEDNKDLI QGKDLLIAYY DVDYEKNAKG
251 SNYWRNRVMM VAKKFLDAGH KLNFAVASRK TFSHELSDFG LESTAGEIPV
301 VAIRTAKGEK FVMQEEFSRD GKALERFLQD YFDGNLKRYL KSEPIPESND
351 GPVKVVVAEN FDEIVNNENK DVLIEFYAPW CGHCKNLEPK YKELGEKLSK
401 DPNIVIAKMD ATANDVPSPY EVRGFPTIYF SPANKKLNPK KYEGGRELSD
451 FISYLQREAT NPPVIQEEKP KKKKKAQEDL
```

Residue Number Increasing Mass Decreasing Mass

Start - End	Observed	Mr(expt)	Mr(calc)	Delta	Miss	Sequence
38 - 48	1191.5824	1190.5751	1190.5931	-0.0180	0	LAPEYEAAATR (No match)
83 - 94	1236.5068	1235.4995	1235.5054	-0.0059	0	DGEEAGAYDGPR (No match)
234 - 246	1619.7727	1618.7654	1618.7766	-0.0112	0	DLLIAYYDVDYEK (No match)

265 - 279	1645.8698	1644.8625	1644.8735	-0.0110	1	**FLDAGHKLNFAVASR** (No match)
280 - 304	2703.3765	2702.3692	2702.3914	-0.0222	1	**KTFSHELSDFGLESTAGEIPVVAIR** (No match)
281 - 304	2575.2861	2574.2788	2574.2965	-0.0177	0	**TFSHELSDFGLESTAGEIPVVAIR** (No match)
281 - 304	2575.2861	2574.2788	2574.2965	-0.0177	0	**TFSHELSDFGLESTAGEIPVVAIR** (Ions score 95)
311 - 319	1188.5240	1187.5167	1187.5280	-0.0113	0	**FVMQEEFSR** Oxidation (M) (Ions score 18)
311 - 319	1188.5240	1187.5167	1187.5280	-0.0113	0	**FVMQEEFSR** Oxidation (M) (No match)
327 - 338	1515.7479	1514.7406	1514.7517	-0.0111	1	**FLQDYFDGNLKR** (No match)
327 - 338	1515.7479	1514.7406	1514.7517	-0.0111	1	**FLQDYFDGNLKR** (Ions score 80)
409 - 423	1680.7397	1679.7324	1679.7460	-0.0136	0	**MDATANDVPSPYEVR** Oxidation (M) (No match)
409 - 423	1680.7397	1679.7324	1679.7460	-0.0136	0	**MDATANDVPSPYEVR** Oxidation (M) (Ions score 47)
424 - 435	1341.6675	1340.6602	1340.6764	-0.0162	0	**GFPTIYFSPANK** (No match)
424 - 436	1469.7642	1468.7569	1468.7714	-0.0145	1	**GFPTIYFSPANKK** (No match)
447 - 457	1370.6853	1369.6780	1369.6877	-0.0097	0	**ELSDFISYLQR** (No match)
447 - 457	1370.6853	1369.6780	1369.6877	-0.0097	0	**ELSDFISYLQR** (Ions score 44)

Mascot: http://www.matrixscience.com/

Spot 381

Mascot Search Results

Protein View

Match to: **gi|119597640** Score: **190** Expect: **1.9e-014**
protein disulfide isomerase family A, member 3, isoform CRA_a [Homo sapiens]

Nominal mass (M_r): **54454**; Calculated pI value: **6.78**
NCBI BLAST search of gi|119597640 against nr
Unformatted sequence string for pasting into other applications

Taxonomy: Homo sapiens

Fixed modifications: Carbamidomethyl (C)
Variable modifications: Oxidation (M)
Cleavage by Trypsin: cuts C-term side of KR unless next residue is P
Sequence Coverage: **20%**

Matched peptides shown in **Bold Red**

```
  1 MRLRRLALFP GVALLLAAAR LAAASDFFAP WCGHCKRLAP EYEAAATRLK
 51 GIVPLAKVDC TANTNTCNKY GVSGYPTLKI FRDGEEAGAY DGPRTADGIV
101 SHLKKQAGPA SVPLRTEEEF KKFISDKDAS IVGFFDDSFS EAHSEFLKAA
151 SNLRDNYRFA HTNVESLVNE YDDNGEGIIL FRPSHLTNKF EDKTVAYTEQ
201 KMTSGKIKKF IQENIFGICP HMTEDNKDLI QGKDLLIAYY DVDYEKNAKG
251 SNYWRNRVMM VAKKFLDAGH KLNFAVASRK TFSHELSDFG LESTAGEIPV
301 VAIRTAKGEK FVMQEEFSRD GKALERFLQD YFDGNLKRYL KSEPIPESND
351 GPVKVVVAEN FDEIVNNENK DVLIEFYAPW CGHCKNLEPK YKELGEKLSK
401 DPNIVIAKMD ATANDVPSPY EVRGFPTIYF SPANKKLNPK KYEGGRELSD
451 FISYLQREAT NPPVIQEEKP KKKKKAQEDL
```

Residue Number Increasing Mass Decreasing Mass

Start - End	Observed	Mr(expt)	Mr(calc)	Delta	Miss	Sequence
38 - 48	1191.5837	1190.5764	1190.5931	-0.0167	0	LAPEYEAAATR (No match)
80 - 94	1652.7445	1651.7372	1651.7590	-0.0217	1	IFRDGEEAGAYDGPR (No match)
83 - 94	1236.5005	1235.4932	1235.5054	-0.0122	0	DGEEAGAYDGPR (Ions score 23)

83 - 94	1236.5005	1235.4932	1235.5054	-0.0122	0	**DGEEAGAYDGPR** (No match)
265 - 279	1645.8616	1644.8543	1644.8735	-0.0192	1	**FLDAGHKLNFAVASR** (No match)
311 - 319	1172.5273	1171.5200	1171.5331	-0.0131	0	**FVMQEEFSR** (Ions score 4)
311 - 319	1172.5273	1171.5200	1171.5331	-0.0131	0	**FVMQEEFSR** (No match)
311 - 319	1188.5120	1187.5047	1187.5280	-0.0233	0	**FVMQEEFSR** Oxidation (M) (No match)
311 - 319	1188.5120	1187.5047	1187.5280	-0.0233	0	**FVMQEEFSR** Oxidation (M) (Ions score 11)
327 - 338	1515.7388	1514.7315	1514.7517	-0.0202	1	**FLQDYFDGNLKR** (No match)
327 - 338	1515.7388	1514.7315	1514.7517	-0.0202	1	**FLQDYFDGNLKR** (Ions score 31)
409 - 423	1664.7335	1663.7262	1663.7511	-0.0249	0	**MDATANDVPSPYEVR** (No match)
409 - 423	1680.7338	1679.7265	1679.7460	-0.0195	0	**MDATANDVPSPYEVR** Oxidation (M) (No match)
424 - 435	1341.6589	1340.6516	1340.6764	-0.0248	0	**GFPTIYFSPANK** (No match)
447 - 457	1370.6743	1369.6670	1369.6877	-0.0207	0	**ELSDFISYLQR** (No match)
447 - 457	1370.6743	1369.6670	1369.6877	-0.0207	0	**ELSDFISYLQR** (Ions score 32)

Mascot: http://www.matrixscience.com/

Spot 385

Mascot Search Results

Protein View

Match to: **gi|114656687** Score: **443** Expect: **9.6e-040**
PREDICTED: protein disulfide isomerase-associated 3 isoform 1 [Pan troglodytes]

Nominal mass (M_r): **55328;** Calculated pI value: **6.42**
NCBI BLAST search of gi|114656687 against nr
Unformatted sequence string for pasting into other applications

Taxonomy: Pan troglodytes
Links to retrieve other entries containing this sequence from NCBI Entrez:
gi|119597641 from Homo sapiens

Fixed modifications: Carbamidomethyl (C)
Variable modifications: Oxidation (M)
Cleavage by Trypsin: cuts C-term side of KR unless next residue is P
Sequence Coverage: **54%**

Matched peptides shown in **Bold Red**

```
  1 MTLWILPKFK AVDFSPSLAA YTHTWLLPRF LEILIQCGHC KRLAPEYEAA
 51 ATRLKGIVPL AKVDCTANTN TCNKYGVSGY PTLKIFRDGE EAGAYDGPRT
101 ADGIVSHLKK QAGPASVPLR TEEEFKKFIS DKDASIVGFF DDSFSEAHSE
151 FLKAASNLRD NYRFAHTNVE SLVNEYDDNG EGIILFRPSH LTNKFEDKTV
201 AYTEQKMTSG KIKKFIQENI FGICPHMTED NKDLIQGKDL LIAYYDVDYE
251 KNAKGSNYWR NRVMMVAKKF LDAGHKLNFA VASRKTFSHE LSDFGLESTA
301 GEIPVVAIRT AKGEKFVMQE EFSRDGKALE RFLQDYFDGN LKRYLKSEPI
351 PESNDGPVKV VVAENFDEIV NNENKDVLIE FYAPWCGHCK NLEPKYKELG
401 EKLSKDPNIV IAKMDATAND VPSPYEVRGF PTIYFSPANK KLNPKKYEGG
451 RELSDFISYL QREATNPPVI QEEKPKKKKK AQEDL
```

Residue Number Increasing Mass Decreasing Mass

Start - End	Observed	Mr(expt)	Mr(calc)	Delta	Miss	Sequence	
42 - 53	1347.7131	1346.7058	1346.6942	0.0116	1	RLAPEYEAAATR	(No match)

43 - 53	1191.5959	1190.5886	1190.5931	-0.0045	0	LAPEYEAAATR (No match)
75 - 84	1084.5679	1083.5606	1083.5600	0.0006	0	YGVSGYPTLK (No match)
85 - 99	1652.7826	1651.7753	1651.7590	0.0164	1	IFRDGEEAGAYDGPR (No match)
88 - 99	1236.5201	1235.5128	1235.5054	0.0074	0	DGEEAGAYDGPR (Ions score 53)
88 - 99	1236.5201	1235.5128	1235.5054	0.0074	0	DGEEAGAYDGPR (No match)
111 - 120	995.5646	994.5573	994.5559	0.0014	0	QAGPASVPLR (No match)
111 - 126	1758.9237	1757.9164	1757.8947	0.0217	1	QAGPASVPLRTEEEFK (No match)
128 - 153	2938.4077	2937.4004	2937.3708	0.0297	1	FISDKDASIVGFFDDSFSEAHSEFLK (No match)
164 - 194	3529.7268	3528.7195	3528.7272	-0.0077	0	FAHTNVESLVNEYDDNGEGIILFRPSHLTNK (No match)
199 - 206	939.4772	938.4699	938.4709	-0.0009	0	TVAYTEQK (No match)
239 - 251	1619.7983	1618.7910	1618.7766	0.0144	0	DLLIAYYDVDYEK (No match)
270 - 284	1645.8954	1644.8881	1644.8735	0.0146	1	FLDAGHKLNFAVASR (No match)
277 - 284	877.4929	876.4856	876.4817	0.0039	0	LNFAVASR (No match)
285 - 309	2703.4089	2702.4016	2702.3914	0.0102	1	KTFSHELSDFGLESTAGEIPVVAIR (No match)
286 - 309	2575.3162	2574.3089	2574.2965	0.0124	0	TFSHELSDFGLESTAGEIPVVAIR (No match)
316 - 324	1188.5371	1187.5298	1187.5280	0.0018	0	FVMQEEFSR Oxidation (M) (Ions score 17)
316 - 324	1188.5371	1187.5298	1187.5280	0.0018	0	FVMQEEFSR Oxidation (M) (No match)
332 - 342	1359.6691	1358.6618	1358.6506	0.0112	0	FLQDYFDGNLK (No match)
332 - 343	1515.7720	1514.7647	1514.7517	0.0130	1	FLQDYFDGNLKR (Ions score 55)
332 - 343	1515.7720	1514.7647	1514.7517	0.0130	1	FLQDYFDGNLKR (No match)
347 - 359	1368.6782	1367.6709	1367.6568	0.0141	0	SEPIPESNDGPVK (No match)
396 - 402	866.4607	865.4534	865.4545	-0.0010	1	YKELGEK (No match)
414 - 428	1680.7689	1679.7616	1679.7460	0.0156	0	MDATANDVPSPYEVR Oxidation (M) (No match)
429 - 440	1341.6888	1340.6815	1340.6764	0.0051	0	GFPTIYFSPANK (No match)
429 - 441	1469.7870	1468.7797	1468.7714	0.0083	1	GFPTIYFSPANKK (No match)
452 - 462	1370.7063	1369.6990	1369.6877	0.0113	0	ELSDFISYLQR (No match)
452 - 462	1370.7063	1369.6990	1369.6877	0.0113	0	ELSDFISYLQR (Ions score 54)
463 - 476	1579.8444	1578.8371	1578.8253	0.0119	0	EATNPPVIQEEKPK (No match)

Mascot: http://www.matrixscience.com/

Spot 386

Mascot Search Results

Protein View

Match to: **gi|14488680** Score: **201** Expect: **1.5e-015**
Chain A, Crystal Structure Of Human Phosphoglucose IsomeraseNEUROLEUKINAUTOCRINE MOTILITY FACTORMAT

Nominal mass (M_r): **63204;** Calculated pI value: **8.44**
NCBI BLAST search of gi|14488680 against nr
Unformatted sequence string for pasting into other applications

Taxonomy: Homo sapiens

Fixed modifications: Carbamidomethyl (C)
Variable modifications: Oxidation (M)
Cleavage by Trypsin: cuts C-term side of KR unless next residue is P
Sequence Coverage: **35%**

Matched peptides shown in **Bold Red**

```
  1 AALTRDPQFQ KLQQWYREHR SELNLRRLFD ANKDRFNHFS LTLNTNHGHI
 51 LVDYSKNLVT EDVMRMLVDL AKSRGVEAAR ERMFNGEKIN YTEGRAVLHV
101 ALRNRSNTPI LVDGKDVMPE VNKVLDKMKS FCQRVRSGDW KGYTGKTITD
151 VINIGIGGSD LGPLMVTEAL KPYSSGGPRV WYVSNIDGTH IAKTLAQLNP
201 ESSLFIIASK TFTTQETITN AETAKEWFLQ AAKDPSAVAK HFVALSTNTT
251 KVKEFGIDPQ NMFEFWDWVG GRYSLWSAIG LSIALHVGFD NFEQLLSGAH
301 WMDQHFRTTP LEKNAPVLLA LLGIWYINCF GCETHAMLPY DQYLHRFAAY
351 FQQGDMESNG KYITKSGTRV DHQTGPIVWG EPGTNGQHAF YQLIHQGTKM
401 IPCDFLIPVQ TQHPIRKGLH HKILLANFLA QTEALMRGKS TEEARKELQA
451 AGKSPEDLER LLPHKVFEGN RPTNSIVFTK LTPFMLGALV AMYEHKIFVQ
501 GIIWDINSFD QWGVELGKQL AKKIEPELDG SAQVTSHDAS TNGLINFIKQ
551 QREARVQ
```

Residue Number Increasing Mass Decreasing Mass

Start - End	Observed	Mr(expt)	Mr(calc)	Delta	Miss	Sequence
12 - 17	893.4711	892.4638	892.4555	0.0083	0	LQQWYR (No match)

28 - 35	978.5079	977.5006	977.4930	0.0076	1	LFDANKDR (No match)
36 - 56	2457.2366	2456.2293	2456.2236	0.0057	0	FNHFSLTLNTNHGHILVDYSK (No match)
57 - 65	1092.5375	1091.5302	1091.5281	0.0022	0	NLVTEDVMR Oxidation (M) (No match)
89 - 95	852.4307	851.4234	851.4137	0.0097	0	INYTEGR (No match)
96 - 103	878.5627	877.5554	877.5497	0.0057	0	AVLHVALR (No match)
106 - 123	1972.0178	1971.0105	1970.9982	0.0123	1	SNTPILVDGKDVMPEVNK Oxidation (M) (No match)
194 - 210	1832.0214	1831.0141	1831.0090	0.0051	0	TLAQLNPESSLFIIASK (Ions score 32)
194 - 210	1832.0214	1831.0141	1831.0090	0.0051	0	TLAQLNPESSLFIIASK (No match)
362 - 369	925.4558	924.4485	924.5028	-0.0543	1	YITKSGTR (No match)
400 - 416	2065.0864	2064.0791	2064.0649	0.0143	0	MIPCDFLIPVQTQHPIR (No match)
400 - 416	2081.0679	2080.0606	2080.0598	0.0009	0	MIPCDFLIPVQTQHPIR Oxidation (M) (No match)
400 - 417	2209.1775	2208.1702	2208.1547	0.0155	1	MIPCDFLIPVQTQHPIRK Oxidation (M) (No match)
423 - 437	1703.9528	1702.9455	1702.9440	0.0016	0	ILLANFLAQTEALMR (No match)
423 - 437	1719.9471	1718.9398	1718.9389	0.0010	0	ILLANFLAQTEALMR Oxidation (M) (No match)
423 - 437	1719.9471	1718.9398	1718.9389	0.0010	0	ILLANFLAQTEALMR Oxidation (M) (No match)
454 - 460	845.4116	844.4043	844.3926	0.0117	0	SPEDLER (No match)
466 - 480	1708.9015	1707.8942	1707.8943	-0.0001	0	VFEGNRPTNSIVFTK (No match)
466 - 480	1708.9015	1707.8942	1707.8943	-0.0001	0	VFEGNRPTNSIVFTK (Ions score 19)
481 - 496	1852.9430	1851.9357	1851.9262	0.0095	0	LTPFMLGALVAMYEHK 2 Oxidation (M) (No match)
523 - 549	2884.4929	2883.4856	2883.4613	0.0243	1	KIEPELDGSAQVTSHDASTNGLINFIK (No match)

Mascot: http://www.matrixscience.com/

Spot 388

Mascot Search Results

Protein View

Match to: **gi|18088719** Score: **466** Expect: **4.8e-042**
Tubulin, beta [Homo sapiens]

Nominal mass (M_r): **50096;** Calculated pI value: **4.75**
NCBI BLAST search of gi|18088719 against nr
Unformatted sequence string for pasting into other applications

Taxonomy: Homo sapiens

Fixed modifications: Carbamidomethyl (C)
Variable modifications: Oxidation (M)
Cleavage by Trypsin: cuts C-term side of KR unless next residue is P
Sequence Coverage: **52%**

Matched peptides shown in **Bold Red**

1 **MREIVHIQAG QCGNQIGAK**F WEVISDEHGI DPTGTYHGDS DLQLDR**ISVY**
51 **YNEATGGK**YV PR**AILVDLEP GTMDSVRSGP FGQIFRPDNF VFGQSGAGNN**
101 **WAKGHYTEGA ELVDSVLDVV RK**EAESCDCL QGFQLTHSLG GGTGSGMGTL
151 LISK**IREEYP DR**IMNTFSVV PSPKVSDTVV EPYNATLSVH QLVENTDETY
201 CIDNEALYDI CFRTLK**LTTP TYGDLNHLVS ATMSGVTTCL RFPGQLNADL**
251 **RKLAVNMVPF PRLHFFMPGF APLTSR**GSQQ YR**ALTVPELT QQVFDAK**DMM
301 AACDPRHGR**Y LTVAAVFR**GR MSMKEVDEQM LNVQNK**NSSY FVEWIPNNVK**
351 **TAVCDIPPR**G LK**MAVTFIGN STAIQELFKR ISEQFTAMFR R**KAFLHWYTG
401 EGMDEMEFTE AESNMNDLVS EYQQYQDATA EEEEDFGEEA EEEA

Residue Number Increasing Mass Decreasing Mass

Start - End	Observed	Mr(expt)	Mr(calc)	Delta	Miss	Sequence
1 - 19	2110.0154	2109.0081	2109.0571	-0.0490	1	**MREIVHIQAGQCGNQIGAK** (No match)
1 - 19	2126.0215	2125.0142	2125.0520	-0.0378	1	**MREIVHIQAGQCGNQIGAK** Oxidation (M) (No match)
3 - 19	1822.8905	1821.8832	1821.9155	-0.0323	0	**EIVHIQAGQCGNQIGAK** (No match)
47 - 58	1301.6147	1300.6074	1300.6299	-0.0224	0	**ISVYYNEATGGK** (No match)

63 - 77	1615.8080	1614.8007	1614.8287	-0.0279	0	**AILVDLEPGTMDSVR** (No match)
63 - 77	1631.7998	1630.7925	1630.8236	-0.0310	0	**AILVDLEPGTMDSVR** Oxidation (M) (No match)
78 - 103	2798.2483	2797.2410	2797.3360	-0.0949	0	**SGPFGQIFRPDNFVFGQSGAGNNWAK** (No match)
104 - 121	1958.9412	1957.9339	1957.9744	-0.0405	0	**GHYTEGAELVDSVLDVVR** (No match)
104 - 122	2087.0308	2086.0235	2086.0694	-0.0459	1	**GHYTEGAELVDSVLDVVRK** (No match)
155 - 162	1077.5189	1076.5116	1076.5250	-0.0134	1	**IREEYPDR** (No match)
217 - 241	2708.2646	2707.2573	2707.3309	-0.0736	0	**LTTPTYGDLNHLVSATMSGVTTCLR** (No match)
242 - 251	1130.5844	1129.5771	1129.5880	-0.0108	0	**FPGQLNADLR** (Ions score 62)
242 - 251	1130.5844	1129.5771	1129.5880	-0.0108	0	**FPGQLNADLR** (No match)
242 - 252	1258.6749	1257.6676	1257.6829	-0.0153	1	**FPGQLNADLRK** (No match)
252 - 262	1271.7144	1270.7071	1270.7219	-0.0148	1	**KLAVNMVPFPR** (No match)
252 - 262	1287.7020	1286.6947	1286.7168	-0.0221	1	**KLAVNMVPFPR** Oxidation (M) (No match)
253 - 262	1143.6243	1142.6170	1142.6270	-0.0100	0	**LAVNMVPFPR** (No match)
253 - 262	1159.6124	1158.6051	1158.6219	-0.0168	0	**LAVNMVPFPR** Oxidation (M) (No match)
263 - 276	1620.8060	1619.7987	1619.8282	-0.0295	0	**LHFFMPGFAPLTSR** (No match)
263 - 276	1620.8060	1619.7987	1619.8282	-0.0295	0	**LHFFMPGFAPLTSR** (Ions score 20)
263 - 276	1636.8004	1635.7931	1635.8231	-0.0300	0	**LHFFMPGFAPLTSR** Oxidation (M) (Ions score 32)
263 - 276	1636.8004	1635.7931	1635.8231	-0.0300	0	**LHFFMPGFAPLTSR** Oxidation (M) (No match)
283 - 297	1659.8615	1658.8542	1658.8879	-0.0337	0	**ALTVPELTQQVFDAK** (No match)
310 - 318	1039.5847	1038.5774	1038.5862	-0.0087	0	**YLTVAAVFR** (No match)
310 - 318	1039.5847	1038.5774	1038.5862	-0.0087	0	**YLTVAAVFR** (Ions score 41)
337 - 350	1696.7983	1695.7910	1695.8256	-0.0346	0	**NSSYFVEWIPNNVK** (No match)
351 - 359	1028.5121	1027.5048	1027.5120	-0.0072	0	**TAVCDIPPR** (No match)
363 - 380	2026.0317	2025.0244	2025.0717	-0.0472	1	**MAVTFIGNSTAIQELFKR** (No match)
363 - 380	2042.0374	2041.0301	2041.0666	-0.0364	1	**MAVTFIGNSTAIQELFKR** Oxidation (M) (No match)
381 - 390	1229.5847	1228.5774	1228.5910	-0.0136	0	**ISEQFTAMFR** (Ions score 59)
381 - 390	1229.5847	1228.5774	1228.5910	-0.0136	0	**ISEQFTAMFR** (No match)
381 - 390	1245.5731	1244.5658	1244.5859	-0.0201	0	**ISEQFTAMFR** Oxidation (M) (No match)
381 - 391	1385.6780	1384.6707	1384.6921	-0.0214	1	**ISEQFTAMFRR** (No match)
381 - 391	1401.6714	1400.6641	1400.6870	-0.0229	1	**ISEQFTAMFRR** Oxidation (M) (No match)

Mascot: http://www.matrixscience.com/

Spot 389

{MATRIX SCIENCE} Mascot Search Results

Protein View

Match to: **gi|114656687** Score: **298** Expect: **3e-025**
PREDICTED: protein disulfide isomerase-associated 3 isoform 1 [Pan troglodytes]

Nominal mass (M_r): **55328**; Calculated pI value: **6.42**
NCBI BLAST search of gi|114656687 against nr
Unformatted sequence string for pasting into other applications

Taxonomy: Pan troglodytes
Links to retrieve other entries containing this sequence from NCBI Entrez:
gi|119597641 from Homo sapiens

Fixed modifications: Carbamidomethyl (C)
Variable modifications: Oxidation (M)
Cleavage by Trypsin: cuts C-term side of KR unless next residue is P
Sequence Coverage: **30%**

Matched peptides shown in **Bold Red**

```
  1 MTLWILPKFK AVDFSPSLAA YTHTWLLPRF LEILIQCGHC KRLAPEYEAA
 51 ATRLKGIVPL AKVDCTANTN TCNKYGVSGY PTLKIFRDGE EAGAYDGPRT
101 ADGIVSHLKK QAGPASVPLR TEEEFKKFIS DKDASIVGFF DDSFSEAHSE
151 FLKAASNLRD NYRFAHTNVE SLVNEYDDNG EGIILFRPSH LTNKFEDKTV
201 AYTEQKMTSG KIKKFIQENI FGICPHMTED NKDLIQGKDL LIAYYDVDYE
251 KNAKGSNYWR NRVMMVAKKF LDAGHKLNFA VASRKTFSHE LSDFGLESTA
301 GEIPVVAIRT AKGEKFVMQE EFSRDGKALE RFLQDYFDGN LKRYLKSEPI
351 PESNDGPVKV VVAENFDEIV NNENKDVLIE FYAPWCGHCK NLEPKYKELG
401 EKLSKDPNIV IAKMDATAND VPSPYEVRGF PTIYFSPANK KLNPKKYEGG
451 RELSDFISYL QREATNPPVI QEEKPKKKKK AQEDL
```

Residue Number Increasing Mass Decreasing Mass

Start - End	Observed	Mr(expt)	Mr(calc)	Delta	Miss	Sequence	
43 - 53	1191.6127	1190.6054	1190.5931	0.0123	0	LAPEYEAAATR	(No match)

75 - 84	1084.5750	1083.5677	1083.5600	0.0077	0	YGVSGYPTLK (No match)
85 - 99	1652.7985	1651.7912	1651.7590	0.0323	1	IFRDGEEAGAYDGPR (No match)
88 - 99	1236.5352	1235.5279	1235.5054	0.0225	0	DGEEAGAYDGPR (Ions score 40)
88 - 99	1236.5352	1235.5279	1235.5054	0.0225	0	DGEEAGAYDGPR (No match)
239 - 251	1619.8168	1618.8095	1618.7766	0.0329	0	DLLIAYYDVDYEK (No match)
270 - 284	1645.9141	1644.9068	1644.8735	0.0333	1	FLDAGHKLNFAVASR (No match)
285 - 309	2703.4333	2702.4260	2702.3914	0.0346	1	KTFSHELSDFGLESTAGEIPVVAIR (No match)
286 - 309	2575.3430	2574.3357	2574.2965	0.0392	0	TFSHELSDFGLESTAGEIPVVAIR (No match)
316 - 324	1188.5510	1187.5437	1187.5280	0.0157	0	FVMQEEFSR Oxidation (M) (No match)
316 - 324	1188.5510	1187.5437	1187.5280	0.0157	0	FVMQEEFSR Oxidation (M) (Ions score 9)
332 - 343	1515.7878	1514.7805	1514.7517	0.0288	1	FLQDYFDGNLKR (Ions score 52)
332 - 343	1515.7878	1514.7805	1514.7517	0.0288	1	FLQDYFDGNLKR (No match)
414 - 428	1680.7880	1679.7807	1679.7460	0.0347	0	MDATANDVPSPYEVR Oxidation (M) (No match)
429 - 440	1341.7004	1340.6931	1340.6764	0.0167	0	GFPTIYFSPANK (No match)
429 - 441	1469.8027	1468.7954	1468.7714	0.0240	1	GFPTIYFSPANKK (No match)
452 - 462	1370.7196	1369.7123	1369.6877	0.0246	0	ELSDFISYLQR (Ions score 48)
452 - 462	1370.7196	1369.7123	1369.6877	0.0246	0	ELSDFISYLQR (No match)

Mascot: http://www.matrixscience.com/

Spot 390

Mascot Search Results

Protein View

Match to: **gi|14488680** Score: **378** Expect: **3e-033**
Chain A, Crystal Structure Of Human Phosphoglucose IsomeraseNEUROLEUKINAUTOCRINE MOTILITY FACTORMAT

Nominal mass (M_r): **63204;** Calculated pI value: **8.44**
NCBI BLAST search of gi|14488680 against nr
Unformatted sequence string for pasting into other applications

Taxonomy: Homo sapiens

Fixed modifications: Carbamidomethyl (C)
Variable modifications: Oxidation (M)
Cleavage by Trypsin: cuts C-term side of KR unless next residue is P
Sequence Coverage: **47%**

Matched peptides shown in **Bold Red**

```
  1 AALTRDPQFQ KLQQWYREHR SELNLRRLFD ANKDRFNHFS LTLNTNHGHI
 51 LVDYSKNLVT EDVMRMLVDL AKSRGVEAAR ERMFNGEKIN YTEGRAVLHV
101 ALRNRSNTPI LVDGKDVMPE VNKVLDKMKS FCQRVRSGDW KGYTGKTITD
151 VINIGIGGSD LGPLMVTEAL KPYSSGGPRV WYVSNIDGTH IAKTLAQLNP
201 ESSLFIIASK TFTTQETITN AETAKEWFLQ AAKDPSAVAK HFVALSTNTT
251 KVKEFGIDPQ NMFEFWDWVG GRYSLWSAIG LSIALHVGFD NFEQLLSGAH
301 WMDQHFRTTP LEKNAPVLLA LLGIWYINCF GCETHAMLPY DQYLHRFAAY
351 FQQGDMESNG KYITKSGTRV DHQTGPIVWG EPGTNGQHAF YQLIHQGTKM
401 IPCDFLIPVQ TQHPIRKGLH HKILLANFLA QTEALMRGKS TEEARKELQA
451 AGKSPEDLER LLPHKVFEGN RPTNSIVFTK LTPFMLGALV AMYEHKIFVQ
501 GIIWDINSFD QWGVELGKQL AKKIEPELDG SAQVTSHDAS TNGLINFIKQ
551 QREARVQ
```

Residue Number Increasing Mass Decreasing Mass

Start - End	Observed	Mr(expt)	Mr(calc)	Delta	Miss	Sequence
12 - 17	893.4319	892.4246	892.4555	-0.0309	0	LQQWYR (No match)

18 - 26	1153.5724	1152.5651	1152.5999	-0.0348	1	EHRSELNLR (No match)
28 - 35	978.4720	977.4647	977.4930	-0.0283	1	LFDANKDR (No match)
34 - 56	2728.2593	2727.2520	2727.3517	-0.0996	1	DRFNHFSLTLNTNHGHILVDYSK (No match)
36 - 56	2457.1577	2456.1504	2456.2236	-0.0732	0	FNHFSLTLNTNHGHILVDYSK (No match)
57 - 65	1076.5081	1075.5008	1075.5332	-0.0323	0	NLVTEDVMR (No match)
57 - 65	1092.5055	1091.4982	1091.5281	-0.0298	0	NLVTEDVMR Oxidation (M) (No match)
75 - 82	887.4722	886.4649	886.4620	0.0029	1	GVEAARER (No match)
89 - 95	852.3942	851.3869	851.4137	-0.0268	0	INYTEGR (No match)
96 - 103	878.5311	877.5238	877.5497	-0.0259	0	AVLHVALR (No match)
147 - 179	3328.6321	3327.6248	3327.7383	-0.1135	0	TITDVINIGIGGSDLGPLMVTEALKPYSSGGPR (No match)
180 - 193	1602.7769	1601.7696	1601.8201	-0.0505	0	VWYVSNIDGTHIAK (No match)
194 - 210	1831.9600	1830.9527	1831.0090	-0.0563	0	TLAQLNPESSLFIIASK (No match)
226 - 233	992.4852	991.4779	991.5127	-0.0347	0	EWFLQAAK (No match)
254 - 272	2329.9575	2328.9502	2329.0262	-0.0759	0	EFGIDPQNMFEFWDWVGGR (No match)
400 - 416	2065.0083	2064.0010	2064.0649	-0.0638	0	MIPCDFLIPVQTQHPIR (Ions score 73)
400 - 416	2065.0083	2064.0010	2064.0649	-0.0638	0	MIPCDFLIPVQTQHPIR (No match)
400 - 416	2081.0039	2079.9966	2080.0598	-0.0631	0	MIPCDFLIPVQTQHPIR Oxidation (M) (No match)
400 - 417	2193.0955	2192.0882	2192.1598	-0.0716	1	MIPCDFLIPVQTQHPIRK (No match)
400 - 417	2193.0955	2192.0882	2192.1598	-0.0716	1	MIPCDFLIPVQTQHPIRK (Ions score 54)
400 - 417	2209.0969	2208.0896	2208.1547	-0.0651	1	MIPCDFLIPVQTQHPIRK Oxidation (M) (No match)
423 - 437	1703.8978	1702.8905	1702.9440	-0.0534	0	ILLANFLAQTEALMR (No match)
423 - 437	1719.8943	1718.8870	1718.9389	-0.0518	0	ILLANFLAQTEALMR Oxidation (M) (No match)
454 - 460	845.3782	844.3709	844.3926	-0.0217	0	SPEDLER (No match)
466 - 480	1708.8475	1707.8402	1707.8943	-0.0541	0	VFEGNRPTNSIVFTK (No match)
466 - 480	1708.8475	1707.8402	1707.8943	-0.0541	0	VFEGNRPTNSIVFTK (Ions score 16)
481 - 496	1820.8889	1819.8816	1819.9364	-0.0548	0	LTPFMLGALVAMYEHK (No match)
523 - 549	2884.4011	2883.3938	2883.4613	-0.0675	1	KIEPELDGSAQVTSHDASTNGLINFIK (No match)

Mascot: http://www.matrixscience.com/

Spot 392

{MATRIX SCIENCE} Mascot Search Results

Protein View

Match to: **gi|15277503** Score: **271** Expect: **1.5e-022**
ACTB protein [Homo sapiens]

Nominal mass (M_r): **40536**; Calculated pI value: **5.55**
NCBI BLAST search of gi|15277503 against nr
Unformatted sequence string for pasting into other applications

Taxonomy: Homo sapiens

Fixed modifications: Carbamidomethyl (C)
Variable modifications: Oxidation (M)
Cleavage by Trypsin: cuts C-term side of KR unless next residue is P
Sequence Coverage: **34%**

Matched peptides shown in **Bold Red**

```
  1 MCKAGFAGDD APRAVFPSIV GRPRHQGVMV GMGQKDSYVG DEAQSKRGIL
 51 TLKYPIEHGI VTNWDDMEKI WHHTFYNELR VAPEEHPVLL TEAPLNPKAN
101 LEKMTQIMFE TFNTPAMYVA IQAVLSLYAS GRTTGIVMDS GDGVTHTVPI
151 YEGYALPHAI LRLDLAGRDL TDYLMKILTE RGYSFTTTAE REIVRDIKEK
201 LCYVALDFEQ EMATAASSSS LEKSYELPDG QVITIGNERF RCPEALFQPS
251 FLGMESCGIH ETTFNSIMKC DVDIRKDLYA NTVLSGGTTM YPGIADRMQK
301 EITALAPSTM KIKIIAPPER KYSVWIGGSI LASLSTFQQM WISKQEYDES
351 GPSIVHRKCF
```

Residue Number Increasing Mass Decreasing Mass

Start - End	Observed	Mr(expt)	Mr(calc)	Delta	Miss	Sequence
4 - 13	976.4375	975.4302	975.4409	-0.0107	0	AGFAGDDAPR (No match)
14 - 24	1198.6871	1197.6798	1197.6982	-0.0183	0	AVFPSIVGRPR (Ions score 71)
14 - 24	1198.6871	1197.6798	1197.6982	-0.0183	0	AVFPSIVGRPR (No match)
70 - 80	1515.7174	1514.7101	1514.7418	-0.0317	0	IWHHTFYNELR (No match)
81 - 98	1954.0377	1953.0304	1953.0571	-0.0266	0	VAPEEHPVLLTEAPLNPK (No match)

163 - 176	1623.8678	1622.8605	1622.8338	0.0268	1	**LDLAGRDLTDYLMK** (No match)
163 - 176	1623.8678	1622.8605	1622.8338	0.0268	1	**LDLAGRDLTDYLMK** (No match)
182 - 191	1132.5129	1131.5056	1131.5196	-0.0140	0	**GYSFTTTAER** (No match)
224 - 239	1790.8615	1789.8542	1789.8846	-0.0303	0	**SYELPDGQVITIGNER** (No match)
224 - 239	1790.8615	1789.8542	1789.8846	-0.0303	0	**SYELPDGQVITIGNER** (Ions score 116)
277 - 297	2215.0447	2214.0374	2214.0626	-0.0252	0	**DLYANTVLSGGTTMYPGIADR** (No match)
277 - 297	2231.0422	2230.0349	2230.0575	-0.0226	0	**DLYANTVLSGGTTMYPGIADR** Oxidation (M) (No match)
345 - 357	1516.6920	1515.6847	1515.6953	-0.0106	0	**QEYDESGPSIVHR** (No match)

Mascot: http://www.matrixscience.com/

Spot 393

{MATRIX SCIENCE} Mascot Search Results

Protein View

Match to: **gi|14488680** Score: **136** Expect: **4.8e-009**
Chain A, Crystal Structure Of Human Phosphoglucose IsomeraseNEUROLEUKINAUTOCRINE MOTILITY FACTORMAT

Nominal mass (M_r): **63204**; Calculated pI value: **8.44**
NCBI BLAST search of gi|14488680 against nr
Unformatted sequence string for pasting into other applications

Taxonomy: Homo sapiens

Fixed modifications: Carbamidomethyl (C)
Variable modifications: Oxidation (M)
Cleavage by Trypsin: cuts C-term side of KR unless next residue is P
Sequence Coverage: **23%**

Matched peptides shown in **Bold Red**

```
  1 AALTRDPQFQ KLQQWYREHR SELNLRRLFD ANKDRFNHFS LTLNTNHGHI
 51 LVDYSKNLVT EDVMRMLVDL AKSRGVEAAR ERMFNGEKIN YTEGRAVLHV
101 ALRNRSNTPI LVDGKDVMPE VNKVLDKMKS FCQRVRSGDW KGYTGKTITD
151 VINIGIGGSD LGPLMVTEAL KPYSSGGPRV WYVSNIDGTH IAKTLAQLNP
201 ESSLFIIASK TFTTQETITN AETAKEWFLQ AAKDPSAVAK HFVALSTNTT
251 KVKEFGIDPQ NMFEFWDWVG GRYSLWSAIG LSIALHVGFD NFEQLLSGAH
301 WMDQHFRTTP LEKNAPVLLA LLGIWYINCF GCETHAMLPY DQYLHRFAAY
351 FQQGDMESNG KYITKSGTRV DHQTGPIVWG EPGTNGQHAF YQLIHQGTKM
401 IPCDFLIPVQ TQHPIRKGLH HKILLANFLA QTEALMRGKS TEEARKELQA
451 AGKSPEDLER LLPHKVFEGN RPTNSIVFTK LTPFMLGALV AMYEHKIFVQ
501 GIIWDINSFD QWGVELGKQL AKKIEPELDG SAQVTSHDAS TNGLINFIKQ
551 QREARVQ
```

Residue Number Increasing Mass Decreasing Mass

Start - End	Observed	Mr(expt)	Mr(calc)	Delta	Miss	Sequence
12 - 17	893.4348	892.4275	892.4555	-0.0280	0	LQQWYR (No match)

28 - 35	978.4780	977.4707	977.4930	-0.0223	1	LFDANKDR (No match)
36 - 56	2457.1890	2456.1817	2456.2236	-0.0419	0	FNHFSLTLNTNHGHILVDYSK (No match)
57 - 65	1092.5122	1091.5049	1091.5281	-0.0231	0	NLVTEDVMR Oxidation (M) (No match)
89 - 95	852.3946	851.3873	851.4137	-0.0264	0	INYTEGR (No match)
96 - 103	878.5260	877.5187	877.5497	-0.0310	0	AVLHVALR (No match)
194 - 210	1831.9803	1830.9730	1831.0090	-0.0360	0	TLAQLNPESSLFIIASK (No match)
194 - 210	1831.9803	1830.9730	1831.0090	-0.0360	0	TLAQLNPESSLFIIASK (Ions score 28)
423 - 437	1719.9099	1718.9026	1718.9389	-0.0362	0	ILLANFLAQTEALMR Oxidation (M) (Ions score 13)
423 - 437	1719.9099	1718.9026	1718.9389	-0.0362	0	ILLANFLAQTEALMR Oxidation (M) (No match)
454 - 460	845.3813	844.3740	844.3926	-0.0186	0	SPEDLER (No match)
466 - 480	1708.8669	1707.8596	1707.8943	-0.0347	0	VFEGNRPTNSIVFTK (Ions score 6)
466 - 480	1708.8669	1707.8596	1707.8943	-0.0347	0	VFEGNRPTNSIVFTK (No match)
481 - 496	1852.9014	1851.8941	1851.9262	-0.0321	0	LTPFMLGALVAMYEHK 2 Oxidation (M) (No match)

Mascot: http://www.matrixscience.com/

Spot 394

Mascot Search Results

Protein View

Match to: **gi|62896585** Score: **296** Expect: **4.8e-025**
adenylyl cyclase-associated protein variant [Homo sapiens]

Nominal mass (M_r): **51899**; Calculated pI value: **8.07**
NCBI BLAST search of gi|62896585 against nr
Unformatted sequence string for pasting into other applications

Taxonomy: Homo sapiens

Fixed modifications: Carbamidomethyl (C)
Variable modifications: Oxidation (M)
Cleavage by Trypsin: cuts C-term side of KR unless next residue is P
Sequence Coverage: **36%**

Matched peptides shown in **Bold Red**

```
  1 MADMQNLVER LERAVGRLEA VSHTSDMHRG YADSPSKAGA APYVQAFDSL
 51 LAGPVAEYLK ISKEIGGDVQ KHAEMVHTGL KLERALLVTA SQCQQPAENK
101 LSDLLAPISE QIKEVITFRE KNRGSKLFNH LSAVSESIQA LGWVAMAPKP
151 GPYVKEMNDA AMFYTNRVLK EYKDVDKKHV DWVKAYLSIW TELQAYIKEF
201 HTTGLAWSKT GPVAKELSGL PSGPSAGSGP PPPPPGPPPP PVSTSSGSDE
251 SASRSALFAQ INQGESITHA LKHVSDDMKT HKNPALKAQS GPVRSGPKPF
301 SAPKPQTSPS PKRATKKEPA VLELEGKKWR VENQENVSNL VIEDTELKQV
351 AYIYKCVNTT LQIKGKINSI TVDSCKKLGL VFDDVVGIVE IINSKDVKVQ
401 VMGKVPTISI NKTDGCHAYL SKNSLDCEIV SAKSSEMNVL IPTEGGDFNE
451 FPVPEQFKTL WNGQKLVTTV TEIAG
```

Residue Number Increasing Mass Decreasing Mass

Start - End	Observed	Mr(expt)	Mr(calc)	Delta	Miss	Sequence
38 - 60	2351.1748	2350.1675	2350.2208	-0.0533	0	AGAAPYVQAFDSLLAGPVAEYLK (No match)
101 - 119	2172.1672	2171.1599	2171.2201	-0.0602	1	LSDLLAPISEQIKEVITFR (No match)
156 - 167	1494.5657	1493.5584	1493.5915	-0.0330	0	EMNDAAMFYTNR 2 Oxidation (M) (No match)

216 - 254	3590.5906	3589.5833	3589.7171	-0.1338	0	ELSGLPSGPSAGSGPPPPPPGPPPPPVSTSSGSDESASR	(No match)
216 - 254	3590.5906	3589.5833	3589.7171	-0.1338	0	ELSGLPSGPSAGSGPPPPPPGPPPPPVSTSSGSDESASR	(Ions score 58)
255 - 272	1927.9797	1926.9724	1927.0163	-0.0438	0	SALFAQINQGESITHALK	(Ions score 82)
255 - 272	1927.9797	1926.9724	1927.0163	-0.0438	0	SALFAQINQGESITHALK	(No match)
295 - 312	1837.9425	1836.9352	1836.9733	-0.0381	0	SGPKPFSAPKPQTSPSPK	(No match)
295 - 312	1837.9425	1836.9352	1836.9733	-0.0381	0	SGPKPFSAPKPQTSPSPK	(Ions score 71)
331 - 348	2072.9893	2071.9820	2072.0273	-0.0452	0	VENQENVSNLVIEDTELK	(No match)
434 - 458	2827.2627	2826.2554	2826.3057	-0.0503	0	SSEMNVLIPTEGGDFNEFPVPEQFK Oxidation (M)	(No match)

Mascot: http://www.matrixscience.com/

Spot 395

Mascot Search Results

Protein View

Match to: **gi|62896585** Score: **755** Expect: **6.1e-071**
adenylyl cyclase-associated protein variant [Homo sapiens]

Nominal mass (M_r): **51899**; Calculated pI value: **8.07**
NCBI BLAST search of gi|62896585 against nr
Unformatted sequence string for pasting into other applications

Taxonomy: Homo sapiens

Fixed modifications: Carbamidomethyl (C)
Variable modifications: Oxidation (M)
Cleavage by Trypsin: cuts C-term side of KR unless next residue is P
Sequence Coverage: **58%**

Matched peptides shown in **Bold Red**

```
  1 MADMQNLVER LERAVGRLEA VSHTSDMHRG YADSPSKAGA APYVQAFDSL
 51 LAGPVAEYLK ISKEIGGDVQ KHAEMVHTGL KLERALLVTA SQCQQPAENK
101 LSDLLAPISE QIKEVITFRE KNRGSKLFNH LSAVSESIQA LGWVAMAPKP
151 GPYVKEMNDA AMFYTNRVLK EYKDVDKKHV DWVKAYLSIW TELQAYIKEF
201 HTTGLAWSKT GPVAKELSGL PSGPSAGSGP PPPPPGPPPP PVSTSSGSDE
251 SASRSALFAQ INQGESITHA LKHVSDDMKT HKNPALKAQS GPVRSGPKPF
301 SAPKPQTSPS PKRATKKEPA VLELEGKKWR VENQENVSNL VIEDTELKQV
351 AYIYKCVNTT LQIKGKINSI TVDSCKKLGL VFDDVVGIVE IINSKDVKVQ
401 VMGKVPTISI NKTDGCHAYL SKNSLDCEIV SAKSSEMNVL IPTEGGDFNE
451 FPVPEQFKTL WNGQKLVTTV TEIAG
```

Residue Number Increasing Mass Decreasing Mass

Start - End	Observed	Mr(expt)	Mr(calc)	Delta	Miss	Sequence
18 - 29	1382.6371	1381.6298	1381.6408	-0.0110	0	LEAVSHTSDMHR (No match)
38 - 60	2351.1741	2350.1668	2350.2208	-0.0540	0	AGAAPYVQAFDSLLAGPVAEYLK (No match)
72 - 84	1520.7693	1519.7620	1519.7929	-0.0309	1	HAEMVHTGLKLER (No match)

85 - 100	1757.8422	1756.8349	1756.8777	-0.0428	0	**ALLVTASQCQQPAENK** (No match)
101 - 113	1426.7786	1425.7713	1425.8078	-0.0365	0	**LSDLLAPISEQIK** (No match)
101 - 119	2172.1665	2171.1592	2171.2201	-0.0609	1	**LSDLLAPISEQIKEVITFR** (Ions score 161)
101 - 119	2172.1665	2171.1592	2171.2201	-0.0609	1	**LSDLLAPISEQIKEVITFR** (No match)
156 - 167	1462.5739	1461.5666	1461.6017	-0.0350	0	**EMNDAAMFYTNR** (Ions score 88)
156 - 167	1462.5739	1461.5666	1461.6017	-0.0350	0	**EMNDAAMFYTNR** (No match)
156 - 167	1478.5665	1477.5592	1477.5966	-0.0373	0	**EMNDAAMFYTNR** Oxidation (M) (No match)
156 - 167	1494.5620	1493.5547	1493.5915	-0.0367	0	**EMNDAAMFYTNR** 2 Oxidation (M) (No match)
185 - 198	1698.8716	1697.8643	1697.9028	-0.0385	0	**AYLSIWTELQAYIK** (No match)
199 - 209	1276.6018	1275.5945	1275.6247	-0.0302	0	**EFHTTGLAWSK** (No match)
216 - 254	3590.6135	3589.6062	3589.7171	-0.1109	0	**ELSGLPSGPSAGSGPPPPPPGPPPPPVSTSSGSDESASR** (No match)
216 - 254	3590.6135	3589.6062	3589.7171	-0.1109	0	**ELSGLPSGPSAGSGPPPPPPGPPPPPVSTSSGSDESASR** (Ions score 162)
255 - 272	1927.9764	1926.9691	1927.0163	-0.0471	0	**SALFAQINQGESITHALK** (No match)
255 - 272	1927.9764	1926.9691	1927.0163	-0.0471	0	**SALFAQINQGESITHALK** (Ions score 132)
273 - 279	831.3593	830.3520	830.3592	-0.0072	0	**HVSDDMK** (No match)
295 - 312	1837.9370	1836.9297	1836.9733	-0.0436	0	**SGPKPFSAPKPQTSPSPK** (No match)
331 - 348	2072.9824	2071.9751	2072.0273	-0.0521	0	**VENQENVSNLVIEDTELK** (No match)
349 - 355	884.4604	883.4531	883.4803	-0.0272	0	**QVAYIYK** (No match)
405 - 422	2003.9622	2002.9549	2003.0146	-0.0596	1	**VPTISINKTDGCHAYLSK** (No match)
434 - 458	2811.2817	2810.2744	2810.3108	-0.0364	0	**SSEMNVLIPTEGGDFNEFPVPEQFK** (No match)
459 - 465	846.4211	845.4138	845.4395	-0.0257	0	**TLWNGQK** (No match)

Mascot: http://www.matrixscience.com/

Spot 396

{MATRIX SCIENCE} Mascot Search Results

Protein View

Match to: **gi|6984209** Score: **272** Expect: **1.2e-022**
tyrosine kinase LCK [Homo sapiens]

Nominal mass (M_r): **56959**; Calculated pI value: **5.34**
NCBI BLAST search of gi|6984209 against nr
Unformatted sequence string for pasting into other applications

Taxonomy: Homo sapiens
Links to retrieve other entries containing this sequence from NCBI Entrez:
gi|56204147 from Homo sapiens

Fixed modifications: Carbamidomethyl (C)
Variable modifications: Oxidation (M)
Cleavage by Trypsin: cuts C-term side of KR unless next residue is P
Sequence Coverage: **15%**

Matched peptides shown in **Bold Red**

```
  1 MENIDVCENC HYPIVPLDGK GTLLIRNGSE VRDPLVTYEG SNPPASPLQD
 51 NLVIALHSYE PSHDGDLGFE KGEQLRILEQ SGEWWKAQSL TTGQEGFIPF
101 NFVAKANSLE PEPWFFKNLS RKDAERQLLA PGNTHGSFLI RESESTAGSF
151 SLSVRDFDQN QGEVVKHYKI RNLDNGGFYI SPRITFPGLH ELVRHYTNAS
201 DGLCTRLSRP CQTQKPQKPW WEDEWEVPRE TLKLVERLGA GQFGEVWMGY
251 YNGHTKVAVK SLKQGSMSPD AFLAEANLMK QLQHQRLVRL YAVVTQEPIY
301 IITEYMENGS LVDFLKTPSG IKLTINKLLD MAAQIAEGMA FIEERNYIHR
351 DLRAANILVS DTLSCKIADF GLARLIEDNE YTAREGAKFP IKWTAPEAIN
401 YGTFTIKSDV WSFGILLTEI VTHGRIPYPG MTNPEVIQNL ERGYRMVRPD
451 NCPEELYQLM RLCWKERPED RPTFDYLRSV LEDFFTATEG QYQPQP
```

Residue Number Increasing Mass Decreasing Mass

Start - End	Observed	Mr(expt)	Mr(calc)	Delta	Miss	Sequence	
127 - 141	1623.8695	1622.8622	1622.8892	-0.0270	0	QLLAPGNTHGSFLIR	(No match)

127 - 141	1623.8695	1622.8622	1622.8892	-0.0270	0	QLLAPGNTHGSFLIR	(Ions score 56)
172 - 183	1352.6406	1351.6333	1351.6520	-0.0187	0	NLDNGGFYISPR	(No match)
172 - 183	1352.6406	1351.6333	1351.6520	-0.0187	0	NLDNGGFYISPR	(Ions score 78)
184 - 194	1281.7137	1280.7064	1280.7240	-0.0176	0	ITFPGLHELVR	(Ions score 46)
184 - 194	1281.7137	1280.7064	1280.7240	-0.0176	0	ITFPGLHELVR	(No match)
375 - 384	1223.5752	1222.5679	1222.5829	-0.0150	0	LIEDNEYTAR	(No match)
426 - 442	1986.9707	1985.9634	1985.9880	-0.0246	0	IPYPGMTNPEVIQNLER Oxidation (M)	(No match)
466 - 478	1693.8042	1692.7969	1692.8219	-0.0250	0	ERPEDRPTFDYLR	(Ions score 32)
466 - 478	1693.8042	1692.7969	1692.8219	-0.0250	0	ERPEDRPTFDYLR	(No match)

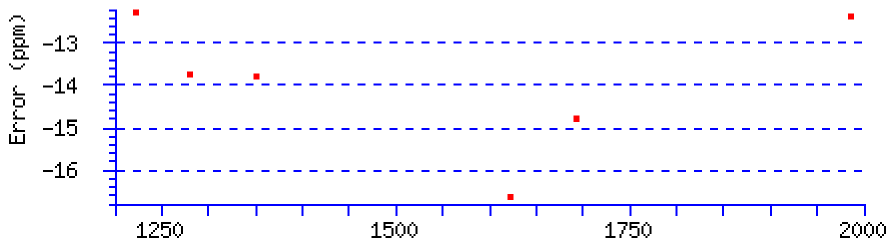

RMS error 14 ppm

Mascot: http://www.matrixscience.com/

Spot 397

{MATRIX SCIENCE} Mascot Search Results

Protein View

Match to: **gi|62896585** Score: **644** Expect: **7.7e-060**
adenylyl cyclase-associated protein variant [Homo sapiens]

Nominal mass (M_r): **51899**; Calculated pI value: **8.07**
NCBI BLAST search of gi|62896585 against nr
Unformatted sequence string for pasting into other applications

Taxonomy: Homo sapiens

Fixed modifications: Carbamidomethyl (C)
Variable modifications: Oxidation (M)
Cleavage by Trypsin: cuts C-term side of KR unless next residue is P
Sequence Coverage: **64%**

Matched peptides shown in **Bold Red**

```
  1 MADMQNLVER LERAVGRLEA VSHTSDMHRG YADSPSKAGA APYVQAFDSL
 51 LAGPVAEYLK ISKEIGGDVQ KHAEMVHTGL KLERALLVTA SQCQQPAENK
101 LSDLLAPISE QIKEVITFRE KNRGSKLFNH LSAVSESIQA LGWVAMAPKP
151 GPYVKEMNDA AMFYTNRVLK EYKDVDKKHV DWVKAYLSIW TELQAYIKEF
201 HTTGLAWSKT GPVAKELSGL PSGPSAGSGP PPPPPGPPPP PVSTSSGSDE
251 SASRSALFAQ INQGESITHA LKHVSDDMKT HKNPALKAQS GPVRSGPKPF
301 SAPKPQTSPS PKRATKKEPA VLELEGKKWR VENQENVSNL VIEDTELKQV
351 AYIYKCVNTT LQIKGKINSI TVDSCKKLGL VFDDVVGIVE IINSKDVKVQ
401 VMGKVPTISI NKTDGCHAYL SKNSLDCEIV SAKSSEMNVL IPTEGGDFNE
451 FPVPEQFKTL WNGQKLVTTV TEIAG
```

Residue Number Increasing Mass Decreasing Mass

Start - End	Observed	Mr(expt)	Mr(calc)	Delta	Miss	Sequence
18 - 29	1382.6078	1381.6005	1381.6408	-0.0403	0	LEAVSHTSDMHR (No match)
38 - 60	2351.1433	2350.1360	2350.2208	-0.0848	0	AGAAPYVQAFDSLLAGPVAEYLK (No match)
72 - 81	1122.5258	1121.5185	1121.5651	-0.0466	0	HAEMVHTGLK (No match)

72 - 84	1520.7449	1519.7376	1519.7929	-0.0553	1	HAEMVHTGLKLER (No match)
85 - 100	1757.8231	1756.8158	1756.8777	-0.0619	0	ALLVTASQCQQPAENK (No match)
101 - 113	1426.7603	1425.7530	1425.8078	-0.0548	0	LSDLLAPISEQIK (No match)
101 - 119	2172.1401	2171.1328	2171.2201	-0.0873	1	LSDLLAPISEQIKEVITFR (No match)
101 - 119	2172.1401	2171.1328	2171.2201	-0.0873	1	LSDLLAPISEQIKEVITFR (Ions score 120)
127 - 155	3110.5173	3109.5100	3109.6422	-0.1322	0	LFNHLSAVSESIQALGWVAMAPKPGPYVK (No match)
156 - 167	1462.5531	1461.5458	1461.6017	-0.0558	0	EMNDAAMFYTNR (Ions score 70)
156 - 167	1462.5531	1461.5458	1461.6017	-0.0558	0	EMNDAAMFYTNR (No match)
156 - 167	1478.5488	1477.5415	1477.5966	-0.0550	0	EMNDAAMFYTNR Oxidation (M) (No match)
156 - 167	1494.5392	1493.5319	1493.5915	-0.0595	0	EMNDAAMFYTNR 2 Oxidation (M) (No match)
185 - 198	1698.8442	1697.8369	1697.9028	-0.0659	0	AYLSIWTELQAYIK (No match)
199 - 209	1276.5819	1275.5746	1275.6247	-0.0501	0	EFHTTGLAWSK (No match)
216 - 254	3590.5500	3589.5427	3589.7171	-0.1744	0	ELSGLPSGPSAGSGPPPPPPGPPPPPVSTSSGSDESASR (No match)
216 - 254	3590.5500	3589.5427	3589.7171	-0.1744	0	ELSGLPSGPSAGSGPPPPPPGPPPPPVSTSSGSDESASR (Ions score 94)
255 - 272	1927.9486	1926.9413	1927.0163	-0.0749	0	SALFAQINQGESITHALK (No match)
255 - 272	1927.9486	1926.9413	1927.0163	-0.0749	0	SALFAQINQGESITHALK (Ions score 98)
273 - 279	831.3431	830.3358	830.3592	-0.0234	0	HVSDDMK (No match)
295 - 312	1837.9137	1836.9064	1836.9733	-0.0669	0	SGPKPFSAPKPQTSPSPK (No match)
331 - 348	2072.9543	2071.9470	2072.0273	-0.0802	0	VENQENVSNLVIEDTELK (No match)
349 - 355	884.4519	883.4446	883.4803	-0.0357	0	QVAYIYK (No match)
405 - 422	2003.9419	2002.9346	2003.0146	-0.0799	1	VPTISINKTDGCHAYLSK (No match)
413 - 422	1151.4733	1150.4660	1150.5077	-0.0417	0	TDGCHAYLSK (No match)
434 - 458	2811.2209	2810.2136	2810.3108	-0.0972	0	SSEMNVLIPTEGGDFNEFPVPEQFK (No match)
459 - 465	846.4055	845.3982	845.4395	-0.0413	0	TLWNGQK (No match)

Mascot: http://www.matrixscience.com/

Spot 398

Mascot Search Results

Protein View

Match to: **gi|14625824** Score: **368** Expect: **3e-032**
moesin/anaplastic lymphoma kinase fusion protein [Homo sapiens]

Nominal mass (M_r): **62004**; Calculated pI value: **7.61**
NCBI BLAST search of gi|14625824 against nr
Unformatted sequence string for pasting into other applications

Taxonomy: Homo sapiens

Fixed modifications: Carbamidomethyl (C)
Variable modifications: Oxidation (M)
Cleavage by Trypsin: cuts C-term side of KR unless next residue is P
Sequence Coverage: **19%**

Matched peptides shown in **Bold Red**

```
  1 MPKTISVRVT TMDAELEFAI QPNTTGKQLF DQVVKTIGLR EVWFFGLQYQ
 51 DTKGFSTWLK LNKKVTAQDV RKESPLLFKF RAKFYPEDVS EELIQDITQR
101 LFFLQVKEGI LNDDIYCPPE TAVLLASYAV QSKYGDFNKE VHKSGYLAGD
151 KLLPQRVLEQ HKLNKDQWEE RIQVWHEEHR GMLREDAVLE YLKIAQDLEM
201 YGVNYFSIKN KKGSELWLGV DALGLNIYEQ NDRLTPKIGF PWSEIRNISF
251 NDKKFVIKPI DKKAPDFVFY APRLRINKRI LALCMGNHEL YMRRRKPDTI
301 EVQQMKAQAR EEKHQKQMER AMLENEKKKR EMAEKEKEKI EREKEELMER
351 LKQIEEQTKK AQQELEEQTR RALELEQERK RAQSEAEKLA KERQEAEEAK
401 EALLQASRDQ KKTQEQLALE MAELTARISQ LEMARQKKES EAVEWQQKQE
451 LQAMQMELQS PEYKLSKLRT STIMTDYNPN YCFAGKTSSI SDLKEVPRKN
501 ITLIRGLGHG AFGEVYEGQV SGMPNDP
```

Residue Number Increasing Mass Decreasing Mass

Start - End	Observed	Mr(expt)	Mr(calc)	Delta	Miss	Sequence
28 - 35	976.5088	975.5015	975.5389	-0.0374	0	QLFDQVVK (No match)
84 - 100	2081.9595	2080.9522	2080.9953	-0.0430	0	FYPEDVSEELIQDITQR (No match)

84 - 100	2081.9595	2080.9522	2080.9953	-0.0430	0	**FYPEDVSEELIQDITQR** (Ions score 105)
172 - 180	1233.5779	1232.5706	1232.6050	-0.0344	0	**IQVWHEEHR** (Ions score 26)
181 - 193	1552.7583	1551.7510	1551.7966	-0.0456	1	**GMLREDAVLEYLK** Oxidation (M) (No match)
238 - 246	1104.5538	1103.5465	1103.5763	-0.0298	0	**IGFPWSEIR** (Ions score 37)
238 - 246	1104.5538	1103.5465	1103.5763	-0.0298	0	**IGFPWSEIR** (No match)
263 - 273	1310.6514	1309.6441	1309.6818	-0.0377	1	**KAPDFVFYAPR** (No match)
263 - 273	1310.6514	1309.6441	1309.6818	-0.0377	1	**KAPDFVFYAPR** (Ions score 27)
264 - 273	1182.5629	1181.5556	1181.5869	-0.0313	0	**APDFVFYAPR** (Ions score 58)
264 - 273	1182.5629	1181.5556	1181.5869	-0.0313	0	**APDFVFYAPR** (No match)
361 - 371	1387.6517	1386.6444	1386.6851	-0.0407	1	**AQQELEEQTRR** (No match)
412 - 427	1831.9198	1830.9125	1830.9509	-0.0384	1	**KTQEQLALEMAELTAR** (No match)
412 - 427	1847.9087	1846.9014	1846.9458	-0.0444	1	**KTQEQLALEMAELTAR** Oxidation (M) (No match)
413 - 427	1703.8225	1702.8152	1702.8559	-0.0407	0	**TQEQLALEMAELTAR** (No match)
413 - 427	1719.8253	1718.8180	1718.8508	-0.0328	0	**TQEQLALEMAELTAR** Oxidation (M) (No match)
439 - 448	1233.5779	1232.5706	1232.5673	0.0033	0	**ESEAVEWQQK** (No match)

Mascot: http://www.matrixscience.com/

Spot 399

Mascot Search Results

Protein View

Match to: **gi|187034** Score: **533** Expect: **9.6e-049**
lymphocyte-specific protein tyrosine kinase

Nominal mass (M_r): **58574;** Calculated pI value: **5.34**
NCBI BLAST search of gi|187034 against nr
Unformatted sequence string for pasting into other applications

Taxonomy: Homo sapiens

Fixed modifications: Carbamidomethyl (C)
Variable modifications: Oxidation (M)
Cleavage by Trypsin: cuts C-term side of KR unless next residue is P
Sequence Coverage: **53%**

Matched peptides shown in **Bold Red**

```
  1 MGCGCSSHPE DDWMENIDVC ENCHYPIVRL DGKGRLLIRN GSEVRDPLVT
 51 YEGSNPPASP LQDNLVIALH SYEPSHDGDL GFEKGEPLRI LEQSGEWWKA
101 QSLTTGQEGF IPFNFVAKAN SLEPEPWFFK NLSRKDAERQ LLAPGNTHGS
151 FLIRESESTA GSFSLSVRDF DQNQGEVVKH YKIRNLDNGG FYISPRITFP
201 GLHELVRHYT NASDGLCTRL SRPCQTQKPQ KPWWEDEWEV PRETLKLVER
251 LGAAQFGEVW MGYYNGHTKV AVKSLKQGSM SPDAFLAEAN LMKQLQHQRL
301 VRLYAVVTQE PIYIITEYME NGSLVDFLKT PSGIKLTINK LLDMAAQIAE
351 GMAFIEERNY IHRDLRAANI LVSDTLSCKI ADFGLARLIE DNEYTAREGA
401 KFPIKWTAPE AINYGTFTIK SDVWSFGILL TEIVTHGRIP YPGMTNPEVI
451 QNLERGYRMV RPDNCPEELY QLMRLCWKER PEDRPTFDYL RSVLEDFFTA
501 TEGQYQPQP
```

Residue Number Increasing Mass Decreasing Mass

Start - End	Observed	Mr(expt)	Mr(calc)	Delta	Miss	Sequence
90 - 99	1275.6134	1274.6061	1274.6295	-0.0234	0	ILEQSGEWWK (No match)
100 - 118	2055.0286	2054.0213	2054.0472	-0.0259	0	AQSLTTGQEGFIPFNFVAK (No match)

119 - 130	1464.6893	1463.6820	1463.7085	-0.0264	0	ANSLEPEPWFFK	(No match)
140 - 154	1623.8756	1622.8683	1622.8892	-0.0209	0	QLLAPGNTHGSFLIR	(Ions score 74)
140 - 154	1623.8756	1622.8683	1622.8892	-0.0209	0	QLLAPGNTHGSFLIR	(No match)
155 - 168	1456.6715	1455.6642	1455.6841	-0.0199	0	ESESTAGSFSLSVR	(No match)
169 - 179	1278.5736	1277.5663	1277.5887	-0.0224	0	DFDQNQGEVVK	(No match)
185 - 196	1352.6433	1351.6360	1351.6520	-0.0160	0	NLDNGGFYISPR	(Ions score 89)
185 - 196	1352.6433	1351.6360	1351.6520	-0.0160	0	NLDNGGFYISPR	(No match)
197 - 207	1281.7122	1280.7049	1280.7240	-0.0191	0	ITFPGLHELVR	(Ions score 65)
197 - 207	1281.7122	1280.7049	1280.7240	-0.0191	0	ITFPGLHELVR	(No match)
247 - 269	2626.2043	2625.1970	2625.2797	-0.0827	1	LVERLGAAQFGEVWMGYYNGHTK	(No match)
274 - 293	2138.0154	2137.0081	2137.0547	-0.0466	1	SLKQGSMSPDAFLAEANLMK	(No match)
294 - 299	809.4169	808.4096	808.4304	-0.0207	0	QLQHQR	(No match)
341 - 358	2007.9639	2006.9566	2006.9805	-0.0238	0	LLDMAAQIAEGMAFIEER	(No match)
341 - 358	2023.9646	2022.9573	2022.9754	-0.0180	0	LLDMAAQIAEGMAFIEER Oxidation (M)	(No match)
380 - 387	862.4607	861.4534	861.4708	-0.0174	0	IADFGLAR	(No match)
388 - 397	1223.5728	1222.5655	1222.5829	-0.0174	0	LIEDNEYTAR	(No match)
402 - 420	2197.1497	2196.1424	2196.1619	-0.0194	1	FPIKWTAPEAINYGTFTIK	(No match)
421 - 438	2030.0424	2029.0351	2029.0632	-0.0281	0	SDVWSFGILLTEIVTHGR	(Ions score 44)
421 - 438	2030.0424	2029.0351	2029.0632	-0.0281	0	SDVWSFGILLTEIVTHGR	(No match)
439 - 455	1970.9795	1969.9722	1969.9931	-0.0209	0	IPYPGMTNPEVIQNLER	(No match)
459 - 474	2050.9309	2049.9236	2049.9434	-0.0198	0	MVRPDNCPEELYQLMR	(No match)
479 - 491	1693.8119	1692.8046	1692.8219	-0.0173	0	ERPEDRPTFDYLR	(No match)
479 - 491	1693.8119	1692.8046	1692.8219	-0.0173	0	ERPEDRPTFDYLR	(Ions score 34)

Mascot: http://www.matrixscience.com/

Spot 400

{MATRIX SCIENCE} Mascot Search Results

Protein View

Match to: **gi|119600805** Score: **130** Expect: **1.9e-008**
annexin A11, isoform CRA_a [Homo sapiens]

Nominal mass (M_r): **26961**; Calculated pI value: **6.92**
NCBI BLAST search of gi|119600805 against nr
Unformatted sequence string for pasting into other applications

Taxonomy: Homo sapiens

Fixed modifications: Carbamidomethyl (C)
Variable modifications: Oxidation (M)
Cleavage by Trypsin: cuts C-term side of KR unless next residue is P
Sequence Coverage: **27%**

Matched peptides shown in **Bold Red**

```
  1 MKTPVLFDIY EIKEAIKGVG TDEACLIEIL ASRSNEHIRE LNRAYKAEFK
 51 KTLEEAIRSD TSGHFQRLLI SLSQGNRDES TNVDMSLAQR DAQELYAAGE
101 NRLGTDESKF NAVLCSRSRA HLVAVFNEYQ RMTGRDIEKS ICREMSGDLE
151 EGMLAVVKCL KNTPAFFAER LNKAMRGAGT KDRTLIRIMV SRSETDLLDI
201 RSEYKRMYGK SLYHDISGDT SGDYRKILLK ICGGND
```

Residue Number Increasing Mass Decreasing Mass

Start - End	Observed	Mr(expt)	Mr(calc)	Delta	Miss	Sequence
18 - 33	1703.8964	1702.8891	1702.8559	0.0332	0	GVGTDEACLIEILASR (Ions score 14)
18 - 33	1703.8964	1702.8891	1702.8559	0.0332	0	GVGTDEACLIEILASR (No match)
91 - 102	1336.6364	1335.6291	1335.6054	0.0237	0	DAQELYAAGENR (Ions score 0)
91 - 102	1336.6364	1335.6291	1335.6054	0.0237	0	DAQELYAAGENR (No match)
120 - 131	1446.7715	1445.7642	1445.7415	0.0228	0	AHLVAVFNEYQR (No match)
120 - 131	1446.7715	1445.7642	1445.7415	0.0228	0	AHLVAVFNEYQR (Ions score 33)
162 - 170	1052.5275	1051.5202	1051.5086	0.0116	0	NTPAFFAER (Ions score 15)
162 - 170	1052.5275	1051.5202	1051.5086	0.0116	0	NTPAFFAER (No match)

211 - 226	1813.8689	1812.8616	1812.8278	0.0338	1	SLYHDISGDTSGDYRK	(No match)
211 - 226	1813.8689	1812.8616	1812.8278	0.0338	1	SLYHDISGDTSGDYRK	(Ions score 3)

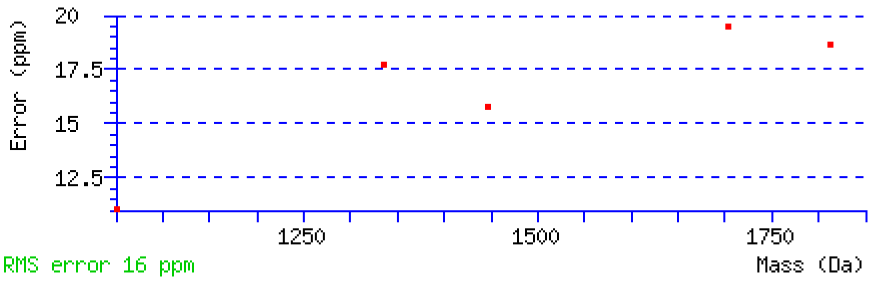

Mascot: http://www.matrixscience.com/

Spot 401

Mascot Search Results

Protein View

Match to: **gi|6984209** Score: **533** Expect: **9.6e-049**
tyrosine kinase LCK [Homo sapiens]

Nominal mass (M_r): **56959**; Calculated pI value: **5.34**
NCBI BLAST search of gi|6984209 against nr
Unformatted sequence string for pasting into other applications

Taxonomy: Homo sapiens
Links to retrieve other entries containing this sequence from NCBI Entrez:
gi|56204147 from Homo sapiens

Fixed modifications: Carbamidomethyl (C)
Variable modifications: Oxidation (M)
Cleavage by Trypsin: cuts C-term side of KR unless next residue is P
Sequence Coverage: **43%**

Matched peptides shown in **Bold Red**

```
  1 MENIDVCENC HYPIVPLDGK GTLLIRNGSE VRDPLVTYEG SNPPASPLQD
 51 NLVIALHSYE PSHDGDLGFE KGEQLRILEQ SGEWWKAQSL TTGQEGFIPF
101 NFVAKANSLE PEPWFFKNLS RKDAERQLLA PGNTHGSFLI RESESTAGSF
151 SLSVRDFDQN QGEVVKHYKI RNLDNGGFYI SPRITFPGLH ELVRHYTNAS
201 DGLCTRLSRP CQTQKPQKPW WEDEWEVPRE TLKLVERLGA GQFGEVWMGY
251 YNGHTKVAVK SLKQGSMSPD AFLAEANLMK QLQHQRLVRL YAVVTQEPIY
301 IITEYMENGS LVDFLKTPSG IKLTINKLLD MAAQIAEGMA FIEERNYIHR
351 DLRAANILVS DTLSCKIADF GLARLIEDNE YTAREGAKFP IKWTAPEAIN
401 YGTFTIKSDV WSFGILLTEI VTHGRIPYPG MTNPEVIQNL ERGYRMVRPD
451 NCPEELYQLM RLCWKERPED RPTFDYLRSV LEDFFTATEG QYQPQP
```

Residue Number Increasing Mass Decreasing Mass

Start - End	Observed	Mr(expt)	Mr(calc)	Delta	Miss	Sequence	
87 - 105	2054.9817	2053.9744	2054.0472	-0.0728	0	AQSLTTGQEGFIPFNFVAK	(No match)

106 - 117	1464.6576	1463.6503	1463.7085	-0.0581	0	ANSLEPEPWFFK (No match)
127 - 141	1623.8330	1622.8257	1622.8892	-0.0635	0	QLLAPGNTHGSFLIR (Ions score 96)
127 - 141	1623.8330	1622.8257	1622.8892	-0.0635	0	QLLAPGNTHGSFLIR (No match)
142 - 155	1456.6368	1455.6295	1455.6841	-0.0546	0	ESESTAGSFSLSVR (No match)
156 - 166	1278.5441	1277.5368	1277.5887	-0.0519	0	DFDQNQGEVVK (No match)
170 - 183	1621.7897	1620.7824	1620.8372	-0.0547	1	IRNLDNGGFYISPR (No match)
172 - 183	1352.6091	1351.6018	1351.6520	-0.0502	0	NLDNGGFYISPR (Ions score 88)
172 - 183	1352.6091	1351.6018	1351.6520	-0.0502	0	NLDNGGFYISPR (No match)
184 - 194	1281.6840	1280.6767	1280.7240	-0.0473	0	ITFPGLHELVR (Ions score 61)
184 - 194	1281.6840	1280.6767	1280.7240	-0.0473	0	ITFPGLHELVR (No match)
195 - 206	1394.5668	1393.5595	1393.6044	-0.0449	0	HYTNASDGLCTR (No match)
238 - 256	2130.8916	2129.8843	2129.9628	-0.0785	0	LGAGQFGEVWMGYYNGHTK Oxidation (M) (No match)
281 - 286	809.4138	808.4065	808.4304	-0.0238	0	QLQHQR (No match)
328 - 345	2007.8547	2006.8474	2006.9805	-0.1330	0	LLDMAAQIAEGMAFIEER (No match)
328 - 345	2023.9059	2022.8986	2022.9754	-0.0767	0	LLDMAAQIAEGMAFIEER Oxidation (M) (No match)
328 - 345	2039.9022	2038.8949	2038.9703	-0.0754	0	LLDMAAQIAEGMAFIEER 2 Oxidation (M) (No match)
367 - 374	862.4477	861.4404	861.4708	-0.0304	0	IADFGLAR (No match)
375 - 384	1223.5463	1222.5390	1222.5829	-0.0439	0	LIEDNEYTAR (No match)
375 - 384	1223.5463	1222.5390	1222.5829	-0.0439	0	LIEDNEYTAR (Ions score 57)
426 - 442	1970.9257	1969.9184	1969.9931	-0.0747	0	IPYPGMTNPEVIQNLER (No match)
426 - 442	1986.9200	1985.9127	1985.9880	-0.0753	0	IPYPGMTNPEVIQNLER Oxidation (M) (No match)
446 - 461	2066.8782	2065.8709	2065.9383	-0.0674	0	MVRPDNCPEELYQLMR Oxidation (M) (No match)
446 - 461	2082.8713	2081.8640	2081.9332	-0.0692	0	MVRPDNCPEELYQLMR 2 Oxidation (M) (No match)
466 - 478	1693.7645	1692.7572	1692.8219	-0.0647	0	ERPEDRPTFDYLR (Ions score 30)
466 - 478	1693.7645	1692.7572	1692.8219	-0.0647	0	ERPEDRPTFDYLR (No match)

Mascot: http://www.matrixscience.com/

Spot 402

{MATRIX SCIENCE} Mascot Search Results

Protein View

Match to: **gi|15277503** Score: **294** Expect: **7.7e-025**
ACTB protein [Homo sapiens]

Nominal mass (M_r): **40536**; Calculated pI value: **5.55**
NCBI BLAST search of gi|15277503 against nr
Unformatted sequence string for pasting into other applications

Taxonomy: Homo sapiens

Fixed modifications: Carbamidomethyl (C)
Variable modifications: Oxidation (M)
Cleavage by Trypsin: cuts C-term side of KR unless next residue is P
Sequence Coverage: **31%**

Matched peptides shown in **Bold Red**

```
  1 MCKAGFAGDD APRAVFPSIV GRPRHQGVMV GMGQKDSYVG DEAQSKRGIL
 51 TLKYPIEHGI VTNWDDMEKI WHHTFYNELR VAPEEHPVLL TEAPLNPKAN
101 LEKMTQIMFE TFNTPAMYVA IQAVLSLYAS GRTTGIVMDS GDGVTHTVPI
151 YEGYALPHAI LRLDLAGRDL TDYLMKILTE RGYSFTTTAE REIVRDIKEK
201 LCYVALDFEQ EMATAASSSS LEKSYELPDG QVITIGNERF RCPEALFQPS
251 FLGMESCGIH ETTFNSIMKC DVDIRKDLYA NTVLSGGTTM YPGIADRMQK
301 EITALAPSTM KIKIIAPPER KYSVWIGGSI LASLSTFQQM WISKQEYDES
351 GPSIVHRKCF
```

Residue Number Increasing Mass Decreasing Mass

Start - End	Observed	Mr(expt)	Mr(calc)	Delta	Miss	Sequence
4 - 13	976.4325	975.4252	975.4409	-0.0157	0	AGFAGDDAPR (No match)
14 - 24	1198.6761	1197.6688	1197.6982	-0.0293	0	AVFPSIVGRPR (Ions score 35)
14 - 24	1198.6761	1197.6688	1197.6982	-0.0293	0	AVFPSIVGRPR (No match)
81 - 98	1954.0226	1953.0153	1953.0571	-0.0417	0	VAPEEHPVLLTEAPLNPK (No match)
163 - 176	1623.8522	1622.8449	1622.8338	0.0112	1	LDLAGRDLTDYLMK (No match)

182 - 191	1132.5073	1131.5000	1131.5196	-0.0196	0	GYSFTTTAER	(No match)
182 - 191	1132.5073	1131.5000	1131.5196	-0.0196	0	GYSFTTTAER	(No match)
224 - 239	1790.8510	1789.8437	1789.8846	-0.0408	0	SYELPDGQVITIGNER	(No match)
224 - 239	1790.8510	1789.8437	1789.8846	-0.0408	0	SYELPDGQVITIGNER	(Ions score 102)
277 - 297	2231.0300	2230.0227	2230.0575	-0.0348	0	DLYANTVLSGGTTMYPGIADR	Oxidation (M) (No match)
345 - 357	1516.6831	1515.6758	1515.6953	-0.0195	0	QEYDESGPSIVHR	(No match)
345 - 357	1516.6831	1515.6758	1515.6953	-0.0195	0	QEYDESGPSIVHR	(Ions score 62)

Mascot: http://www.matrixscience.com/

Spot 403

{MATRIX SCIENCE} Mascot Search Results

Protein View

Match to: **gi|5453595** Score: **193** Expect: **9.6e-015**
adenylyl cyclase-associated protein [Homo sapiens]

Nominal mass (M_r): **51926**; Calculated pI value: **8.07**
NCBI BLAST search of gi|5453595 against nr
Unformatted sequence string for pasting into other applications

Taxonomy: Homo sapiens
Links to retrieve other entries containing this sequence from NCBI Entrez:
gi|157649073 (no taxonomy information for this entry)
gi|114555781 from Pan troglodytes
gi|114555783 from Pan troglodytes
gi|114555785 from Pan troglodytes
gi|178084 from Homo sapiens
gi|179920 from Homo sapiens
gi|15530330 from Homo sapiens
gi|30583143 from Homo sapiens
gi|48146935 from Homo sapiens
gi|61362546 from synthetic construct
gi|63102451 from Homo sapiens
gi|119627644 from Homo sapiens
gi|119627646 from Homo sapiens
gi|119627648 from Homo sapiens
gi|119627649 from Homo sapiens
gi|119627650 from Homo sapiens
gi|123994329 from synthetic construct

Fixed modifications: Carbamidomethyl (C)
Variable modifications: Oxidation (M)
Cleavage by Trypsin: cuts C-term side of KR unless next residue is P
Sequence Coverage: **10%**

Matched peptides shown in **Bold Red**

1 MADMQNLVER LERAVGRLEA VSHTSDMHRG YADSPSKAGA APYVQAFDSL
51 LAGPVAEYLK ISKEIGGDVQ KHAEMVHTGL KLERALLVTA SQCQQPAENK
101 LSDLLAPISE QIKEVITFRE KNRGSKLFNH LSAVSESIQA LGWVAMAPKP

```
151 GPYVKEMNDA AMFYTNRVLK EYKDVDKKHV DWVKAYLSIW TELQAYIKEF
201 HTTGLAWSKT GPVAKELSGL PSGPSAGSGP PPPPPGPPPP PVSTSSGSDE
251 SASRSALFAQ INQGESITHA LKHVSDDMKT HKNPALKAQS GPVRSGPKPF
301 SAPKPQTSPS PKRATKKEPA VLELEGKKWR VENQENVSNL VIEDTELKQV
351 AYIYKCVNTT LQIKGKINSI TVDNCKKLGL VFDDVVGIVE IINSKDVKVQ
401 VMGKVPTISI NKTDGCHAYL SKNSLDCEIV SAKSSEMNVL IPTEGGDFNE
451 FPVPEQFKTL WNGQKLVTTV TEIAG
```

Residue Number Increasing Mass Decreasing Mass

Start - End	Observed	Mr(expt)	Mr(calc)	Delta	Miss	Sequence
156 - 167	1494.6039	1493.5966	1493.5915	0.0052	0	EMNDAAMFYTNR 2 Oxidation (M) (Ions score 17)
156 - 167	1494.6039	1493.5966	1493.5915	0.0052	0	EMNDAAMFYTNR 2 Oxidation (M) (No match)
255 - 272	1928.0353	1927.0280	1927.0163	0.0118	0	SALFAQINQGESITHALK (Ions score 80)
255 - 272	1928.0353	1927.0280	1927.0163	0.0118	0	SALFAQINQGESITHALK (No match)
331 - 348	2073.0542	2072.0469	2072.0273	0.0197	0	VENQENVSNLVIEDTELK (No match)
331 - 348	2073.0542	2072.0469	2072.0273	0.0197	0	VENQENVSNLVIEDTELK (Ions score 69)

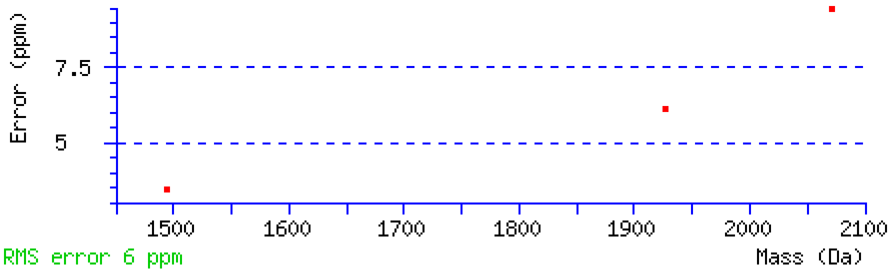

Mascot: http://www.matrixscience.com/

Spot 404

Mascot Search Results

Protein View

Match to: **gi|5453595** Score: **501** Expect: **1.5e-045**
adenylyl cyclase-associated protein [Homo sapiens]

Nominal mass (M_r): **51926**; Calculated pI value: **8.07**
NCBI BLAST search of gi|5453595 against nr
Unformatted sequence string for pasting into other applications

Taxonomy: Homo sapiens
Links to retrieve other entries containing this sequence from NCBI Entrez:
gi|157649073 (no taxonomy information for this entry)
gi|114555781 from Pan troglodytes
gi|114555783 from Pan troglodytes
gi|114555785 from Pan troglodytes
gi|178084 from Homo sapiens
gi|179920 from Homo sapiens
gi|15530330 from Homo sapiens
gi|30583143 from Homo sapiens
gi|48146935 from Homo sapiens
gi|61362546 from synthetic construct
gi|63102451 from Homo sapiens
gi|119627644 from Homo sapiens
gi|119627646 from Homo sapiens
gi|119627648 from Homo sapiens
gi|119627649 from Homo sapiens
gi|119627650 from Homo sapiens
gi|123994329 from synthetic construct

Fixed modifications: Carbamidomethyl (C)
Variable modifications: Oxidation (M)
Cleavage by Trypsin: cuts C-term side of KR unless next residue is P
Sequence Coverage: **53%**

Matched peptides shown in **Bold Red**

```
  1 MADMQNLVER LERAVGRLEA VSHTSDMHRG YADSPSKAGA APYVQAFDSL
 51 LAGPVAEYLK ISKEIGGDVQ KHAEMVHTGL KLERALLVTA SQCQQPAENK
101 LSDLLAPISE QIKEVITFRE KNRGSKLFNH LSAVSESIQA LGWVAMAPKP
```

```
151 GPYVKEMNDA AMFYTNRVLK EYKDVDKKHV DWVKAYLSIW TELQAYIKEF
201 HTTGLAWSKT GPVAKELSGL PSGPSAGSGP PPPPPGPPPP PVSTSSGSDE
251 SASRSALFAQ INQGESITHA LKHVSDDMKT HKNPALKAQS GPVRSGPKPF
301 SAPKPQTSPS PKRATKKEPA VLELEGKKWR VENQENVSNL VIEDTELKQV
351 AYIYKCVNTT LQIKGKINSI TVDNCKKLGL VFDDVVGIVE IINSKDVKVQ
401 VMGKVPTISI NKTDGCHAYL SKNSLDCEIV SAKSSEMNVL IPTEGGDFNE
451 FPVPEQFKTL WNGQKLVTTV TEIAG
```

Residue Number Increasing Mass Decreasing Mass

Start - End	Observed	Mr(expt)	Mr(calc)	Delta	Miss	Sequence
18 - 29	1382.6042	1381.5969	1381.6408	-0.0439	0	LEAVSHTSDMHR (No match)
38 - 60	2351.1384	2350.1311	2350.2208	-0.0897	0	AGAAPYVQAFDSLLAGPVAEYLK (No match)
72 - 81	1122.5231	1121.5158	1121.5651	-0.0493	0	HAEMVHTGLK (No match)
72 - 84	1520.7329	1519.7256	1519.7929	-0.0673	1	HAEMVHTGLKLER (No match)
85 - 100	1757.8090	1756.8017	1756.8777	-0.0760	0	ALLVTASQCQQPAENK (No match)
101 - 113	1426.7493	1425.7420	1425.8078	-0.0658	0	LSDLLAPISEQIK (No match)
101 - 119	2172.1321	2171.1248	2171.2201	-0.0953	1	LSDLLAPISEQIKEVITFR (Ions score 133)
101 - 119	2172.1321	2171.1248	2171.2201	-0.0953	1	LSDLLAPISEQIKEVITFR (No match)
127 - 155	3110.5110	3109.5037	3109.6422	-0.1385	0	LFNHLSAVSESIQALGWVAMAPKPGPYVK (No match)
156 - 167	1462.5470	1461.5397	1461.6017	-0.0619	0	EMNDAAMFYTNR (Ions score 60)
156 - 167	1462.5470	1461.5397	1461.6017	-0.0619	0	EMNDAAMFYTNR (No match)
156 - 167	1478.5398	1477.5325	1477.5966	-0.0640	0	EMNDAAMFYTNR Oxidation (M) (No match)
185 - 198	1698.8450	1697.8377	1697.9028	-0.0651	0	AYLSIWTELQAYIK (No match)
199 - 209	1276.5739	1275.5666	1275.6247	-0.0581	0	EFHTTGLAWSK (No match)
255 - 272	1927.9407	1926.9334	1927.0163	-0.0828	0	SALFAQINQGESITHALK (No match)
255 - 272	1927.9407	1926.9334	1927.0163	-0.0828	0	SALFAQINQGESITHALK (Ions score 106)
273 - 279	831.3393	830.3320	830.3592	-0.0272	0	HVSDDMK (No match)
331 - 348	2072.9465	2071.9392	2072.0273	-0.0880	0	VENQENVSNLVIEDTELK (No match)
377 - 395	2058.1055	2057.0982	2057.1771	-0.0789	1	KLGLVFDDVVGIVEIINSK (No match)
405 - 422	2003.9410	2002.9337	2003.0146	-0.0808	1	VPTISINKTDGCHAYLSK (No match)
434 - 458	2811.2075	2810.2002	2810.3108	-0.1106	0	SSEMNVLIPTEGGDFNEFPVPEQFK (No match)

Mascot: http://www.matrixscience.com/

Spot 406

Mascot Search Results

Protein View

Match to: **gi|119627645** Score: **183** Expect: **9.6e-014**
CAP, adenylate cyclase-associated protein 1 (yeast), isoform CRA_b [Homo sapiens]

Nominal mass (M_r): **51357;** Calculated pI value: **7.64**
NCBI BLAST search of gi|119627645 against nr
Unformatted sequence string for pasting into other applications

Taxonomy: Homo sapiens

Fixed modifications: Carbamidomethyl (C)
Variable modifications: Oxidation (M)
Cleavage by Trypsin: cuts C-term side of KR unless next residue is P
Sequence Coverage: **10%**

Matched peptides shown in **Bold Red**

```
  1 MADMQNLVER LERAVGRLEA VSHTSDMHRG YADTPYVQAF DSLLAGPVAE
 51 YLKISKEIGG DVQKHAEMVH TGLKLERALL VTASQCQQPA ENKLSDLLAP
101 ISEQIKEVIT FREKNRGSKL FNHLSAVSES IQALGWVAMA PKPGPYVKEM
151 NDAAMFYTNR VLKEYKDVDK KHVDWVKAYL SIWTELQAYI KEFHTTGLAW
201 SKTGPVAKEL SGLPSGPSAG SGPPPPPPGP PPPPVSTSSG SDESASRSAL
251 FAQINQGESI THALKHVSDD MKTHKNPALK AQSGPVRSGP KPFSAPKPQT
301 SPSPKRATKK EPAVLELEGK KWRVENQENV SNLVIEDTEL KQVAYIYKCV
351 NTTLQIKGKI NSITVDNCKK LGLVFDDVVG IVEIINSKDV KVQVMGKVPT
401 ISINKTDGCH AYLSKNSLDC EIVSAKSSEM NVLIPTEGGD FNEFPVPEQF
451 KTLWNGQKLV TTVTEIAG
```

Residue Number Increasing Mass Decreasing Mass

Start - End	Observed	Mr(expt)	Mr(calc)	Delta	Miss	Sequence
149 - 160	1494.6089	1493.6016	1493.5915	0.0102	0	EMNDAAMFYTNR 2 Oxidation (M) (Ions score 5)
149 - 160	1494.6089	1493.6016	1493.5915	0.0102	0	EMNDAAMFYTNR 2 Oxidation (M) (No match)
248 - 265	1928.0420	1927.0347	1927.0163	0.0185	0	SALFAQINQGESITHALK (Ions score 84)

248 - 265	1928.0420	1927.0347	1927.0163	0.0185	0	SALFAQINQGESITHALK	(No match)
324 - 341	2073.0659	2072.0586	2072.0273	0.0314	0	VENQENVSNLVIEDTELK	(No match)
324 - 341	2073.0659	2072.0586	2072.0273	0.0314	0	VENQENVSNLVIEDTELK	(Ions score 67)

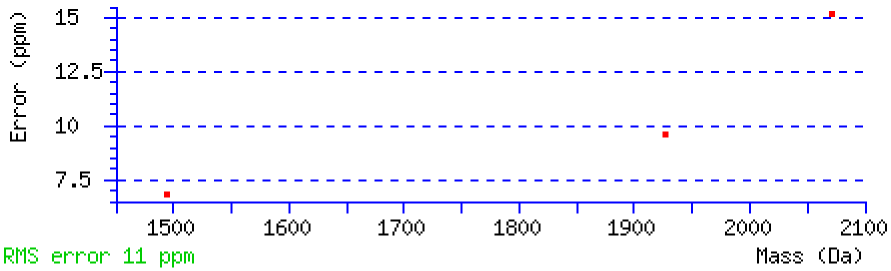

RMS error 11 ppm

Mascot: http://www.matrixscience.com/

Spot 407

Mascot Search Results

Protein View

Match to: **gi|18088719** Score: **659** Expect: **2.4e-061**
Tubulin, beta [Homo sapiens]

Nominal mass (M_r): **50096**; Calculated pI value: **4.75**
NCBI BLAST search of gi|18088719 against nr
Unformatted sequence string for pasting into other applications

Taxonomy: Homo sapiens

Fixed modifications: Carbamidomethyl (C)
Variable modifications: Oxidation (M)
Cleavage by Trypsin: cuts C-term side of KR unless next residue is P
Sequence Coverage: **66%**

Matched peptides shown in **Bold Red**

```
  1 MREIVHIQAG QCGNQIGAKF WEVISDEHGI DPTGTYHGDS DLQLDRISVY
 51 YNEATGGKYV PRAILVDLEP GTMDSVRSGP FGQIFRPDNF VFGQSGAGNN
101 WAKGHYTEGA ELVDSVLDVV RKEAESCDCL QGFQLTHSLG GGTGSGMGTL
151 LISKIREEYP DRIMNTFSVV PSPKVSDTVV EPYNATLSVH QLVENTDETY
201 CIDNEALYDI CFRTLKLTTP TYGDLNHLVS ATMSGVTTCL RFPGQLNADL
251 RKLAVNMVPF PRLHFFMPGF APLTSRGSQQ YRALTVPELT QQVFDAKDMM
301 AACDPRHGRY LTVAAVFRGR MSMKEVDEQM LNVQNKNSSY FVEWIPNNVK
351 TAVCDIPPRG LKMAVTFIGN STAIQELFKR ISEQFTAMFR RKAFLHWYTG
401 EGMDEMEFTE AESNMNDLVS EYQQYQDATA EEEEDFGEEA EEEA
```

Residue Number Increasing Mass Decreasing Mass

Start - End	Observed	Mr(expt)	Mr(calc)	Delta	Miss	Sequence
1 - 19	2110.0273	2109.0200	2109.0571	-0.0371	1	MREIVHIQAGQCGNQIGAK (No match)
3 - 19	1822.8944	1821.8871	1821.9155	-0.0284	0	EIVHIQAGQCGNQIGAK (No match)
20 - 46	3102.3496	3101.3423	3101.4002	-0.0579	0	FWEVISDEHGIDPTGTYHGDSDLQLDR (No match)
47 - 58	1301.6128	1300.6055	1300.6299	-0.0243	0	ISVYYNEATGGK (No match)

63 - 77	1615.8090	1614.8017	1614.8287	-0.0269	0	AILVDLEPGTMDSVR (No match)
63 - 77	1631.8066	1630.7993	1630.8236	-0.0242	0	AILVDLEPGTMDSVR Oxidation (M) (No match)
78 - 103	2798.2737	2797.2664	2797.3360	-0.0695	0	SGPFGQIFRPDNFVFGQSGAGNNWAK (No match)
104 - 121	1958.9480	1957.9407	1957.9744	-0.0337	0	GHYTEGAELVDSVLDVVR (No match)
104 - 122	2087.0393	2086.0320	2086.0694	-0.0374	1	GHYTEGAELVDSVLDVVRK (No match)
155 - 162	1077.5110	1076.5037	1076.5250	-0.0213	1	IREEYPDR (No match)
163 - 174	1319.6835	1318.6762	1318.6955	-0.0192	0	IMNTFSVVPSPK (No match)
217 - 241	2708.2761	2707.2688	2707.3309	-0.0621	0	LTTPTYGDLNHLVSATMSGVTTCLR (No match)
242 - 251	1130.5784	1129.5711	1129.5880	-0.0168	0	FPGQLNADLR (Ions score 83)
242 - 251	1130.5784	1129.5711	1129.5880	-0.0168	0	FPGQLNADLR (No match)
242 - 252	1258.6733	1257.6660	1257.6829	-0.0169	1	FPGQLNADLRK (No match)
252 - 262	1271.7120	1270.7047	1270.7219	-0.0172	1	KLAVNMVPFPR (No match)
252 - 262	1287.7035	1286.6962	1286.7168	-0.0206	1	KLAVNMVPFPR Oxidation (M) (No match)
253 - 262	1143.6185	1142.6112	1142.6270	-0.0158	0	LAVNMVPFPR (Ions score 51)
253 - 262	1143.6185	1142.6112	1142.6270	-0.0158	0	LAVNMVPFPR (No match)
253 - 262	1159.6097	1158.6024	1158.6219	-0.0195	0	LAVNMVPFPR Oxidation (M) (No match)
263 - 276	1620.8099	1619.8026	1619.8282	-0.0256	0	LHFFMPGFAPLTSR (No match)
263 - 276	1620.8099	1619.8026	1619.8282	-0.0256	0	LHFFMPGFAPLTSR (Ions score 38)
263 - 276	1636.8033	1635.7960	1635.8231	-0.0271	0	LHFFMPGFAPLTSR Oxidation (M) (No match)
283 - 297	1659.8688	1658.8615	1658.8879	-0.0264	0	ALTVPELTQQVFDAK (No match)
283 - 306	2723.2966	2722.2893	2722.2764	0.0129	1	ALTVPELTQQVFDAKDMMAACDPR Oxidation (M) (No match)
310 - 318	1039.5790	1038.5717	1038.5862	-0.0144	0	YLTVAAVFR (Ions score 57)
310 - 318	1039.5790	1038.5717	1038.5862	-0.0144	0	YLTVAAVFR (No match)
325 - 336	1446.6663	1445.6590	1445.6820	-0.0230	0	EVDEQMLNVQNK (No match)
337 - 350	1696.8042	1695.7969	1695.8256	-0.0287	0	NSSYFVEWIPNNVK (No match)
351 - 359	1028.5057	1027.4984	1027.5120	-0.0136	0	TAVCDIPPR (No match)
363 - 379	1869.9518	1868.9445	1868.9706	-0.0260	0	MAVTFIGNSTAIQELFK (No match)
363 - 380	2026.0435	2025.0362	2025.0717	-0.0354	1	MAVTFIGNSTAIQELFKR (No match)
363 - 380	2042.0477	2041.0404	2041.0666	-0.0261	1	MAVTFIGNSTAIQELFKR Oxidation (M) (No match)
381 - 390	1229.5818	1228.5745	1228.5910	-0.0165	0	ISEQFTAMFR (Ions score 69)
381 - 390	1229.5818	1228.5745	1228.5910	-0.0165	0	ISEQFTAMFR (No match)
381 - 390	1245.5728	1244.5655	1244.5859	-0.0204	0	ISEQFTAMFR Oxidation (M) (No match)
381 - 391	1385.6798	1384.6725	1384.6921	-0.0196	1	ISEQFTAMFRR (No match)
381 - 391	1401.6664	1400.6591	1400.6870	-0.0279	1	ISEQFTAMFRR Oxidation (M) (No match)

Mascot: http://www.matrixscience.com/

Spot 408

Mascot Search Results

Protein View

Match to: **gi|5771523** Score: **411** Expect: **1.5e-036**
3-phosphoglycerate dehydrogenase [Homo sapiens]

Nominal mass (M_r): **57370**; Calculated pI value: **6.29**
NCBI BLAST search of gi|5771523 against nr
Unformatted sequence string for pasting into other applications

Taxonomy: Homo sapiens
Links to retrieve other entries containing this sequence from NCBI Entrez:
gi|2674062 from Homo sapiens

Fixed modifications: Carbamidomethyl (C)
Variable modifications: Oxidation (M)
Cleavage by Trypsin: cuts C-term side of KR unless next residue is P
Sequence Coverage: **38%**

Matched peptides shown in **Bold Red**

```
  1 MAFANLRKVL ISDSLDPCCR KILQEGGLQV VEKQNLSKEE LIAELQDCEG
 51 LIVRSATKVT ADVINAAEKL QVVGRAGTGV DNVDLEAATR KGILVMNTPN
101 GNSLSAAELT CGMIMCLARQ IPQATASMKD GKWERKKFMG TELNGKTLGI
151 LGLGRIGREV ATRMQSFGMK TIGYDPIISP EVSASFGVQQ LPLEEIWPLC
201 DFITVHTPLL PSTTGLLNDN TFAQCKKGVR VVNCARGGIV DEGALLRALQ
251 SGQCAGAALD VFTEEPPRDR ALVDHENVIS CPHLGASTKE AQSRCGEEIA
301 VQFVDMVKGK SLTGVVNAQA LTSAFSPHTK PWIGLAEALG TLMRAWAGSP
351 KGTIQVITQG TSLKNAGNCL SPAVIVGLLK EASKQADVNL VNAKLLVKEA
401 GLNVTTSHSP AAPGEQGFGE CLLAVALAGA PYQAVGLVQG TTPVLQGLNG
451 AVFRPEVPLR RDLPLLLFRT QTSDPAMLPT MIGLLAEAGV RLLSYQTSLV
501 SDGETWHVMG ISSLLPSLEA WKQHVTEAFQ FHF
```

Residue Number Increasing Mass Decreasing Mass

Start - End	Observed	Mr(expt)	Mr(calc)	Delta	Miss Sequence

9 - 20	1434.6536	1433.6463	1433.6643	-0.0179	0	**VLISDSLDPCCR** (No match)
9 - 20	1434.6536	1433.6463	1433.6643	-0.0179	0	**VLISDSLDPCCR** (Ions score 7)
9 - 21	1562.7495	1561.7422	1561.7592	-0.0170	1	**VLISDSLDPCCRK** (No match)
39 - 54	1886.9318	1885.9245	1885.9454	-0.0209	0	**EELIAELQDCEGLIVR** (Ions score 78)
39 - 54	1886.9318	1885.9245	1885.9454	-0.0209	0	**EELIAELQDCEGLIVR** (No match)
55 - 69	1517.8184	1516.8111	1516.8096	0.0015	1	**SATKVTADVINAAEK** (No match)
76 - 90	1488.7190	1487.7117	1487.7215	-0.0098	0	**AGTGVDNVDLEAATR** (No match)
147 - 155	899.5600	898.5527	898.5599	-0.0072	0	**TLGILGLGR** (No match)
237 - 247	1099.6011	1098.5938	1098.6032	-0.0094	0	**GGIVDEGALLR** (No match)
237 - 247	1099.6011	1098.5938	1098.6032	-0.0094	0	**GGIVDEGALLR** (Ions score 45)
248 - 270	2488.1851	2487.1778	2487.1812	-0.0033	1	**ALQSGQCAGAALDVFTEEPPRDR** (No match)
271 - 289	2048.0120	2047.0047	2047.0156	-0.0109	0	**ALVDHENVISCPHLGASTK** (No match)
271 - 289	2048.0120	2047.0047	2047.0156	-0.0109	0	**ALVDHENVISCPHLGASTK** (Ions score 114)
295 - 308	1640.7574	1639.7501	1639.7585	-0.0084	0	**CGEEIAVQFVDMVK** Oxidation (M) (No match)
352 - 364	1345.7427	1344.7354	1344.7612	-0.0258	0	**GTIQVITQGTSLK** (No match)
365 - 380	1625.8782	1624.8709	1624.8970	-0.0260	0	**NAGNCLSPAVIVGLLK** (No match)
462 - 469	986.5931	985.5858	985.5960	-0.0102	0	**DLPLLLFR** (No match)
470 - 491	2272.1348	2271.1275	2271.1602	-0.0327	0	**TQTSDPAMLPTMIGLLAEAGVR** (No match)
470 - 491	2288.1501	2287.1428	2287.1552	-0.0123	0	**TQTSDPAMLPTMIGLLAEAGVR** Oxidation (M) (No match)
470 - 491	2304.1650	2303.1577	2303.1501	0.0077	0	**TQTSDPAMLPTMIGLLAEAGVR** 2 Oxidation (M) (No match)
523 - 533	1390.6754	1389.6681	1389.6465	0.0216	0	**QHVTEAFQFHF** (No match)

Mascot: http://www.matrixscience.com/

Spot 409

{MATRIX SCIENCE} Mascot Search Results

Protein View

Match to: **gi|108773793** Score: **490** Expect: **1.9e-044**
glucose-6-phosphate dehydrogenase isoform b [Homo sapiens]

Nominal mass (M_r): **59675**; Calculated pI value: **6.39**
NCBI BLAST search of gi|108773793 against nr
Unformatted sequence string for pasting into other applications

Taxonomy: Homo sapiens
Links to retrieve other entries containing this sequence from NCBI Entrez:
gi|116242483 from Homo sapiens
gi|16596511 from Homo sapiens
gi|12653141 from Homo sapiens
gi|119593088 from Homo sapiens
gi|119593092 from Homo sapiens
gi|123982808 from synthetic construct
gi|123997479 from synthetic construct

Fixed modifications: Carbamidomethyl (C)
Variable modifications: Oxidation (M)
Cleavage by Trypsin: cuts C-term side of KR unless next residue is P
Sequence Coverage: **55%**

Matched peptides shown in **Bold Red**

1 MAEQVALSR**T QVCGILR**EEL FQGDAFHQSD THIFIIMGAS GDLAKKKIYP
51 TIWWLFR**DGL LPENTFIVGY AR**SRLTVADI RKQSEPFFK**A TPEEKLKLED**
101 **FFARNSYVAG QYDDAASYQR LNSHMNALHL GSQANR**LFYL ALPPTVYEAV
151 TK**NIHESCMS QIGWNRIIVE KPFGR**DLQSS DR**LSNHISSL FR**EDQIYRID
201 HYLGK**EMVQN LMVLR**FANR**I FGPIWNRDNI ACVILTFKEP FGTEGRGGYF**
251 **DEFGIIR**DVM QNHLLQMLCL VAMEKPASTN SDDVRDEKVK VLKCISEVQA
301 NNVVLGQYVG NPDGEGEATK **GYLDDPTVPR GSTTATFAAV VLYVENERWD**
351 **GVPFILR**CGK ALNERKAEVR **LQFHDVAGDI FHQQCKRNEL VIR**VQPNEAV
401 YTKMMTK**KPG MFFNPEESEL DLTYGNR**YKN VK**LPDAYERL ILDVFCGSQM**
451 **HFVR**SDELRE AWR**IFTPLLH QIELEKPKPI PYIYGSR**GPT EADELMK**RVG**
501 **FQYEGTYK**WV NPHKL

Residue Number Increasing Mass Decreasing Mass

Start - End	Observed	Mr(expt)	Mr(calc)	Delta	Miss	Sequence
10 - 17	946.5006	945.4933	945.5065	-0.0132	0	TQVCGILR (No match)
58 - 72	1664.8401	1663.8328	1663.8569	-0.0241	0	DGLLPENTFIVGYAR (Ions score 79)
58 - 72	1664.8401	1663.8328	1663.8569	-0.0241	0	DGLLPENTFIVGYAR (No match)
90 - 97	915.4921	914.4848	914.5072	-0.0224	1	ATPEEKLK (No match)
96 - 104	1138.6094	1137.6021	1137.6182	-0.0161	1	LKLEDFFAR (No match)
98 - 104	897.4418	896.4345	896.4392	-0.0046	0	LEDFFAR (No match)
105 - 120	1807.7625	1806.7552	1806.7808	-0.0256	0	NSYVAGQYDDAASYQR (No match)
105 - 120	1807.7625	1806.7552	1806.7808	-0.0256	0	NSYVAGQYDDAASYQR (Ions score 94)
121 - 136	1762.8485	1761.8412	1761.8692	-0.0280	0	LNSHMNALHLGSQANR (No match)
121 - 136	1778.8461	1777.8388	1777.8641	-0.0253	0	LNSHMNALHLGSQANR Oxidation (M) (No match)
153 - 166	1747.7412	1746.7339	1746.7566	-0.0227	0	NIHESCMSQIGWNR Oxidation (M) (No match)
167 - 175	1058.6235	1057.6162	1057.6283	-0.0121	0	IIVEKPFGR (Ions score 11)
167 - 175	1058.6235	1057.6162	1057.6283	-0.0121	0	IIVEKPFGR (No match)
183 - 192	1173.6211	1172.6138	1172.6302	-0.0163	0	LSNHISSLFR (No match)
206 - 215	1248.6198	1247.6125	1247.6366	-0.0240	0	EMVQNLMVLR Oxidation (M) (No match)
206 - 215	1264.6147	1263.6074	1263.6315	-0.0240	0	EMVQNLMVLR 2 Oxidation (M) (No match)
220 - 227	1002.5299	1001.5226	1001.5446	-0.0220	0	IFGPIWNR (No match)
228 - 246	2167.0530	2166.0457	2166.0779	-0.0321	1	DNIACVILTFKEPFGTEGR (No match)
247 - 257	1273.6046	1272.5973	1272.6138	-0.0165	0	GGYFDEFGIIR (No match)
247 - 257	1273.6046	1272.5973	1272.6138	-0.0165	0	GGYFDEFGIIR (Ions score 43)
321 - 330	1132.5513	1131.5440	1131.5560	-0.0120	0	GYLDDPTVPR (No match)
331 - 348	1927.9489	1926.9416	1926.9686	-0.0270	0	GSTTATFAAVVLYVENER (No match)
349 - 357	1102.5704	1101.5631	1101.5971	-0.0339	0	WDGVPFILR (No match)
371 - 386	1942.9021	1941.8948	1941.9155	-0.0207	0	LQFHDVAGDIFHQQCK (No match)
371 - 387	2099.0000	2097.9927	2098.0166	-0.0239	1	LQFHDVAGDIFHQQCKR (No match)
387 - 393	899.5068	898.4995	898.5348	-0.0353	1	RNELVIR (No match)
408 - 427	2344.0491	2343.0418	2343.0841	-0.0422	0	KPGMFFNPEESELDLTYGNR (No match)
408 - 427	2360.0708	2359.0635	2359.0790	-0.0154	0	KPGMFFNPEESELDLTYGNR Oxidation (M) (No match)
433 - 439	863.4158	862.4085	862.4184	-0.0099	0	LPDAYER (No match)
440 - 454	1821.8853	1820.8780	1820.9065	-0.0285	0	LILDVFCGSQMHFVR (No match)
440 - 454	1837.8871	1836.8798	1836.9014	-0.0216	0	LILDVFCGSQMHFVR Oxidation (M) (No match)
464 - 487	2852.5469	2851.5396	2851.5999	-0.0603	0	IFTPLLHQIELEKPKPIPYIYGSR (No match)
498 - 508	1347.6536	1346.6463	1346.6618	-0.0155	1	RVGFQYEGTYK (No match)
499 - 508	1191.5613	1190.5540	1190.5607	-0.0067	0	VGFQYEGTYK (No match)

Mascot: http://www.matrixscience.com/

Spot 411

Protein View

Match to: **gi|340368** Score: **480** Expect: **1.9e-043**
transfer RNA-Trp synthetase

Nominal mass (M_r): **53396**; Calculated pI value: **5.73**
NCBI BLAST search of gi|340368 against nr
Unformatted sequence string for pasting into other applications

Taxonomy: Homo sapiens

Fixed modifications: Carbamidomethyl (C)
Variable modifications: Oxidation (M)
Cleavage by Trypsin: cuts C-term side of KR unless next residue is P
Sequence Coverage: **46%**

Matched peptides shown in **Bold Red**

```
  1 MPNSEPASLL ELFNSIATQG ELVRSLKAGN ASKDEIDSAV KMLVSLKMSY
 51 KAAAGEDYKA DCPPGNPAPT SNHGPDATEA EEDFVDPWTV QTSSAKGIDY
101 DKLIVRFGSS KIDKELINRI ERATGQRPHH FLRRGIFFSH RDMNQVLDAY
151 ENKKPFYLYT GRGPSSEAMH VGHLIPFIFT KWLQDVFNVP LVIQMTDDEK
201 YLWKDLTLDQ AYGDAVENAK DIIACGFDIN KTFIFSDLDY MGMSSGFYKN
251 VVKIQKHVTF NQVKGIFGFT DSDCIGKISF PAIQAAPSFS NSFPQIFRDR
301 TDIQCLIPCA IDQDPYFRMT RDVAPRIGYP KPALLHSTFF PALQGAQTKM
351 SASDPNSSIF LTDTAKQIKT KVNKHAFSGG RDTIEEHRQF GGNCDVDVSF
401 MYLTFFLEDD DKLEQIRKDY TSGAMLTGEL KKALIEVLQP LIAEHQARRK
451 EVTDEIVKEF MTPRKLSFDF Q
```

Residue Number Increasing Mass Decreasing Mass

Start - End	Observed	Mr(expt)	Mr(calc)	Delta	Miss	Sequence
2 - 24	2485.2686	2484.2613	2484.2859	-0.0246	0	PNSEPASLLELFNSIATQGELVR (No match)
2 - 24	2485.2686	2484.2613	2484.2859	-0.0246	0	PNSEPASLLELFNSIATQGELVR (Ions score 38)
97 - 106	1191.6360	1190.6287	1190.6659	-0.0371	1	GIDYDKLIVR (No match)
123 - 133	1319.6678	1318.6605	1318.7006	-0.0401	0	ATGQRPHHFLR (No match)
123 - 133	1319.6678	1318.6605	1318.7006	-0.0401	0	ATGQRPHHFLR (Ions score 22)
134 - 141	1019.5203	1018.5130	1018.5460	-0.0330	1	RGIFFSHR (No match)
135 - 141	863.4216	862.4143	862.4449	-0.0306	0	GIFFSHR (No match)

154 - 162	1144.5808	1143.5735	1143.6076	-0.0341	0	**KPFYLYTGR** (No match)
163 - 181	2068.0090	2067.0017	2067.0611	-0.0594	0	**GPSSEAMHVGHLIPFIFTK** (No match)
163 - 181	2068.0090	2067.0017	2067.0611	-0.0594	0	**GPSSEAMHVGHLIPFIFTK** (Ions score 46)
163 - 181	2084.0037	2082.9964	2083.0560	-0.0596	0	**GPSSEAMHVGHLIPFIFTK** Oxidation (M) (No match)
221 - 231	1265.5881	1264.5808	1264.6121	-0.0313	0	**DIIACGFDINK** (No match)
232 - 253	2565.1714	2564.1641	2564.1966	-0.0325	1	**TFIFSDLDYMGMSSGFYKNVVK** Oxidation (M) (No match)
257 - 264	972.4902	971.4829	971.5188	-0.0359	0	**HVTFNQVK** (No match)
265 - 277	1416.6028	1415.5955	1415.6391	-0.0435	0	**GIFGFTDSDCIGK** (No match)
278 - 298	2325.1313	2324.1240	2324.1953	-0.0712	0	**ISFPAIQAAPSFSNSFPQIFR** (No match)
278 - 298	2325.1313	2324.1240	2324.1953	-0.0712	0	**ISFPAIQAAPSFSNSFPQIFR** (Ions score 83)
301 - 318	2224.9805	2223.9732	2224.0293	-0.0560	0	**TDIQCLIPCAIDQDPYFR** (No match)
350 - 366	1784.7909	1783.7836	1783.8298	-0.0462	0	**MSASDPNSSIFLTDTAK** (No match)
433 - 448	1800.9803	1799.9730	1800.0257	-0.0527	0	**ALIEVLQPLIAEHQAR** (No match)
433 - 448	1800.9803	1799.9730	1800.0257	-0.0527	0	**ALIEVLQPLIAEHQAR** (Ions score 72)
451 - 464	1693.7975	1692.7902	1692.8392	-0.0490	1	**EVTDEIVKEFMTPR** (No match)

Spot 412

Protein View

Match to: **gi|66361514** Score: **418** Expect: **3e-037**
Chain A, X-Ray Structure Of A Deletion Variant Of Human Glucose 6- Phosphate Dehydrogenase Complexe

Nominal mass (M_r): **56688**; Calculated pI value: **6.69**
NCBI BLAST search of gi|66361514 against nr
Unformatted sequence string for pasting into other applications

Taxonomy: Homo sapiens
Links to retrieve other entries containing this sequence from NCBI Entrez:
gi|66361517 from Homo sapiens
gi|66361518 from Homo sapiens

Fixed modifications: Carbamidomethyl (C)
Variable modifications: Oxidation (M)
Cleavage by Trypsin: cuts C-term side of KR unless next residue is P
Sequence Coverage: **38%**

Matched peptides shown in **Bold Red**

```
  1 VQSDTHIFII MGASGDLAKK KIYPTIWWLF RDGLLPENTF IVGYARSRLT
 51 VADIRKQSEP FFKATPEEKL KLEDFFARNS YVAGQYDDAA SYQRLNSHMN
101 ALHLGSQANR LFYLALPPTV YEAVTKNIHE SCMSQIGWNR IIVEKPFGRD
151 LQSSDRLSNH ISSLFREDQI YRIDHYLGKE MVQNLMVLRF ANRIFGPIWN
201 RDNIACVILT FKEPFGTEGR GGYFDEFGII RDVMQNHLLQ MLCLVAMEKP
251 ASTNSDDVRD EKVKVLKCIS EVQANNVVLG QYVGNPDGEG EATKGYLDDP
301 TVPRGSTTAT FAAVVLYVEN ERWDGVPFIL RCGKALNERK AEVRLQFHDV
351 AGDIFHQQCK RNELVIRVQP NEAVYTKMMT KKPGMFFNPE ESELDLTYGN
401 RYKNVKLPDA YERLILDVFC GSQMHFVRSD ELREAWRIFT PLLHQIELEK
451 PKPIPYIYGS RGPTEADELM KRVGFQYEGT YKWVNPHKL
```

Residue Number Increasing Mass Decreasing Mass

Start - End	Observed	Mr(expt)	Mr(calc)	Delta	Miss	Sequence
32 - 46	1664.8462	1663.8389	1663.8569	-0.0180	0	DGLLPENTFIVGYAR (Ions score 101)
32 - 46	1664.8462	1663.8389	1663.8569	-0.0180	0	DGLLPENTFIVGYAR (No match)
64 - 71	915.4834	914.4761	914.5072	-0.0311	1	ATPEEKLK (No match)
70 - 78	1138.6116	1137.6043	1137.6182	-0.0139	1	LKLEDFFAR (No match)

70 - 78	1138.6116	1137.6043	1137.6182	-0.0139	1	LKLEDFFAR (Ions score 21)
79 - 94	1807.7688	1806.7615	1806.7808	-0.0193	0	NSYVAGQYDDAASYQR (Ions score 63)
79 - 94	1807.7688	1806.7615	1806.7808	-0.0193	0	NSYVAGQYDDAASYQR (No match)
95 - 110	1762.8577	1761.8504	1761.8692	-0.0188	0	LNSHMNALHLGSQANR (No match)
95 - 110	1778.8496	1777.8423	1777.8641	-0.0218	0	LNSHMNALHLGSQANR Oxidation (M) (No match)
141 - 149	1058.6243	1057.6170	1057.6283	-0.0113	0	IIVEKPFGR (No match)
141 - 149	1058.6243	1057.6170	1057.6283	-0.0113	0	IIVEKPFGR (Ions score 12)
157 - 166	1173.6239	1172.6166	1172.6302	-0.0135	0	LSNHISSLFR (No match)
194 - 201	1002.5379	1001.5306	1001.5446	-0.0140	0	IFGPIWNR (No match)
202 - 220	2167.0710	2166.0637	2166.0779	-0.0141	1	DNIACVILTFKEPFGTEGR (No match)
221 - 231	1273.6100	1272.6027	1272.6138	-0.0111	0	GGYFDEFGIIR (Ions score 38)
221 - 231	1273.6100	1272.6027	1272.6138	-0.0111	0	GGYFDEFGIIR (No match)
295 - 304	1132.5510	1131.5437	1131.5560	-0.0123	0	GYLDDPTVPR (No match)
345 - 360	1942.9058	1941.8985	1941.9155	-0.0170	0	LQFHDVAGDIFHQQCK (No match)
382 - 401	2360.0674	2359.0601	2359.0790	-0.0188	0	KPGMFFNPEESELDLTYGNR Oxidation (M) (No match)
407 - 413	863.4210	862.4137	862.4184	-0.0047	0	LPDAYER (No match)
414 - 428	1837.8898	1836.8825	1836.9014	-0.0189	0	LILDVFCGSQMHFVR Oxidation (M) (No match)

Spot 413

Protein View

Match to: **gi|21040528** Score: **240** Expect: **1.9e-019**
ATPase, H+ transporting, lysosomal 56/58kDa, V1 subunit B2 [Homo sapiens]

Nominal mass (M_r): **56735**; Calculated pI value: **5.66**
NCBI BLAST search of gi|21040528 against nr
Unformatted sequence string for pasting into other applications

Taxonomy: Homo sapiens

Fixed modifications: Carbamidomethyl (C)
Variable modifications: Oxidation (M)
Cleavage by Trypsin: cuts C-term side of KR unless next residue is P
Sequence Coverage: **17%**

Matched peptides shown in **Bold Red**

```
  1 MALRAMRGIV NGAAPELPVP TGGPAVGAQE QALAVSRNYL SQPRLTYKTV
 51 SGVNGPLVIL DHVKFPRYAE IVHLTLPDGT KRSGQVLEVS GSKAVVQVFE
101 GTSGIDAKKT SCEFTGDILR TPVSEDMLGR VFNGSGKPID RGPVVLAEDF
151 LDIMGQPINP QCRIYPEEMI RTGISAIDGM NSIARGQKIP IFSAAGLPHN
201 EIAAQICRQA GLVKKSKDVV DYSEENFAIV FAAMGVNMET ARFFKSDFEE
251 NGSMDNVCLF LNLANDPTIE RIITPRLALT TAEFLAYQCE KHVLVILTDM
301 SSYAEALREV SAAREEVPGR RGFPGYMYTD LATIYERAGR VGGRNGSITQ
351 IPILTMPNDD ITHPIPDLTG YITEGQIYVD RQLHNRQIYP PINVLPSLSR
401 LMKSAIGEGM TRKDHADVSN QLYACYAIGK DVQAMKAVVG EEALTSDDLL
451 YLEFLQKFER NFIAQGPYEN RTVFETLDIG WQLLRIFPKE MLKRIPQSTL
501 SEFYPRDSAK H
```

Residue Number Increasing Mass Decreasing Mass

Start - End	Observed	Mr(expt)	Mr(calc)	Delta	Miss	Sequence
49 - 67	2048.1162	2047.1089	2047.1577	-0.0488	1	**TVSGVNGPLVILDHVKFPR** (No match)
68 - 82	1712.8882	1711.8809	1711.9257	-0.0447	1	**YAEIVHLTLPDGTKR** (No match)
322 - 337	1912.8390	1911.8317	1911.8712	-0.0395	0	**GFPGYMYTDLATIYER** Oxidation (M) (No match)
322 - 337	1912.8390	1911.8317	1911.8712	-0.0395	0	**GFPGYMYTDLATIYER** Oxidation (M) (Ions score 23)
387 - 400	1596.8715	1595.8642	1595.9035	-0.0392	0	**QIYPPINVLPSLSR** (No match)
387 - 400	1596.8715	1595.8642	1595.9035	-0.0392	0	**QIYPPINVLPSLSR** (Ions score 56)

461 - 471	1308.6018	1307.5945	1307.6258	-0.0313	0	NFIAQGPYENR	(Ions score 25)
461 - 471	1308.6018	1307.5945	1307.6258	-0.0313	0	NFIAQGPYENR	(No match)
494 - 506	1593.7999	1592.7926	1592.8310	-0.0384	1	RIPQSTLSEFYPR	(No match)
495 - 506	1437.7035	1436.6962	1436.7299	-0.0337	0	IPQSTLSEFYPR	(No match)
495 - 506	1437.7035	1436.6962	1436.7299	-0.0337	0	IPQSTLSEFYPR	(Ions score 48)

Spot 414

Protein View

Match to: **gi|522193** Score: **341** Expect: **1.5e-029**
vacuolar H+-ATPase 56,000 subunit

Nominal mass (M_r): **56792;** Calculated pI value: **5.57**
NCBI BLAST search of gi|522193 against nr
Unformatted sequence string for pasting into other applications

Taxonomy: Homo sapiens

Fixed modifications: Carbamidomethyl (C)
Variable modifications: Oxidation (M)
Cleavage by Trypsin: cuts C-term side of KR unless next residue is P
Sequence Coverage: **38%**

Matched peptides shown in **Bold Red**

```
  1 MALRAMRGIV NGAAPELPVP TGGPAVGARE QALAVSRNYL SQPRLTYKTV
 51 SGVNGPLVIL DHVKFPRYAE IVHLTLPDGT KRSGQVLEVS GSKAVVQVFE
101 GTSGIDAKKT SCEFTGDILR TPVSEDMLGR VFNGSGKPID RGPVVLAEDF
151 LDIMGQPINP QCRIYPEEMI QTGISAIDGM NSIARGQKIP IFSAAGLPHN
201 EIAAQICRQA GLVKKSKDVV DYSEENFAIV FAAMGVNMET ARFFKSDFEE
251 NGSMDNVCLF LNLANDPTIE RIITPRLALT TAEFLAYQCE KHVLVILTDM
301 SSYAEALREV SAAREEVPGR RGFPGYMYTD LATIYERAGR VEGRNGSITQ
351 IPILTMPNDD ITHPIPDLTG YITEGLIYVD RQLHNRQIYP PINVLPSLSR
401 LMKSAIGEGM TRKDHADVSN QLYACYAIGK DVQAMKAVVG EEALTSDDLL
451 YLEFLQKFER NFIAQGPYEN RTVFETLDIG WQLLRIFPKE MLKRIPQSTL
501 SEFYPRDSAK H
```

Residue Number Increasing Mass Decreasing Mass

Start - End	Observed	Mr(expt)	Mr(calc)	Delta	Miss	Sequence
8 - 29	2000.0565	1999.0492	1999.0849	-0.0357	0	GIVNGAAPELPVPTGGPAVGAR (No match)
8 - 29	2000.0565	1999.0492	1999.0849	-0.0357	0	GIVNGAAPELPVPTGGPAVGAR (Ions score 56)
38 - 44	877.4337	876.4264	876.4453	-0.0189	0	NYLSQPR (No match)
49 - 64	1647.9182	1646.9109	1646.9355	-0.0245	0	TVSGVNGPLVILDHVK (No match)
68 - 82	1712.9073	1711.9000	1711.9257	-0.0256	1	YAEIVHLTLPDGTKR (No match)
68 - 82	1712.9073	1711.9000	1711.9257	-0.0256	1	YAEIVHLTLPDGTKR (Ions score 34)

94 - 108	1520.7714	1519.7641	1519.7881	-0.0240	0	**AVVQVFEGTSGIDAK** (No match)
109 - 120	1426.6855	1425.6782	1425.6922	-0.0139	1	**KTSCEFTGDILR** (No match)
164 - 185	2441.1440	2440.1367	2440.1613	-0.0246	0	**IYPEEMIQTGISAIDGMNSIAR** 2 Oxidation (M) (No match)
189 - 208	2178.1145	2177.1072	2177.1415	-0.0342	0	**IPIFSAAGLPHNEIAAQICR** (No match)
322 - 337	1912.8523	1911.8450	1911.8712	-0.0262	0	**GFPGYMYTDLATIYER** Oxidation (M) (No match)
387 - 400	1596.8843	1595.8770	1595.9035	-0.0264	0	**QIYPPINVLPSLSR** (No match)
387 - 400	1596.8843	1595.8770	1595.9035	-0.0264	0	**QIYPPINVLPSLSR** (Ions score 32)
461 - 471	1308.6119	1307.6046	1307.6258	-0.0212	0	**NFIAQGPYENR** (No match)
461 - 471	1308.6119	1307.6046	1307.6258	-0.0212	0	**NFIAQGPYENR** (Ions score 33)
472 - 485	1690.8949	1689.8876	1689.9090	-0.0213	0	**TVFETLDIGWQLLR** (No match)
494 - 506	1593.8132	1592.8059	1592.8310	-0.0251	1	**RIPQSTLSEFYPR** (No match)
495 - 506	1437.7135	1436.7062	1436.7299	-0.0237	0	**IPQSTLSEFYPR** (No match)
495 - 506	1437.7135	1436.7062	1436.7299	-0.0237	0	**IPQSTLSEFYPR** (Ions score 21)

Spot 415

Protein View

Match to: **gi|4557317** Score: **558** Expect: **3e-051**
annexin A11 [Homo sapiens]

Nominal mass (M_r): **54697**; Calculated pI value: **7.53**
NCBI BLAST search of gi|4557317 against nr
Unformatted sequence string for pasting into other applications

Taxonomy: Homo sapiens
Links to retrieve other entries containing this sequence from NCBI Entrez:
gi|22165431 from Homo sapiens
gi|22165433 from Homo sapiens
gi|1703322 from Homo sapiens
gi|457129 from Homo sapiens
gi|8671171 from Homo sapiens
gi|8671173 from Homo sapiens
gi|8671175 from Homo sapiens
gi|14043153 from Homo sapiens
gi|55960545 from Homo sapiens
gi|57162311 from Homo sapiens
gi|119600806 from Homo sapiens
gi|119600807 from Homo sapiens
gi|119600808 from Homo sapiens
gi|119600809 from Homo sapiens
gi|119600810 from Homo sapiens
gi|119600811 from Homo sapiens
gi|123993655 from synthetic construct
gi|123999943 from synthetic construct

Fixed modifications: Carbamidomethyl (C)
Variable modifications: Oxidation (M)
Cleavage by Trypsin: cuts C-term side of KR unless next residue is P
Sequence Coverage: **38%**

Matched peptides shown in **Bold Red**

1 MSYPGYPPPP GGYPPAAPGG GPWGGAAYPP PPSMPPIGLD NVATYAGQFN
51 QDYLSGMAAN MSGTFGGANM PNLYPGAPGA GYPPVPPGGF GQPPSAQQPV
101 PPYGMYPPPG GNPPSRMPSY PPYPGAPVPG QPMPPPGQQP PGAYPGQPPV
151 TYPGQPPVPL PGQQQPVPSY PGYPGSGTVT PAVPPTQFGS R**GTITDAPGF**
201 **DPLRDAEVLR** KAMK**GFGTDE QAIIDCLGSR** SNK**QRQQILL SFK**TAYGKDL

251 IKDLKSELSG NFEKTILALM KTPVLFDIYE IK**EAIKGVGT DEACLIEILA**
301 **SR**SNEHIREL NRAYKAEFKK **TLEEAIRSDT SGHFQRLLIS LSQGNRDEST**
351 **NVDMSLAQRD AQELYAAGEN RLGTDESKFN AVLCSRSRAH LVAVFNEYQR**
401 MTGRDIEKSI CR**EMSGDLEE GMLAVVK**CLK **NTPAFFAER**L NKAMRGAGTK
451 DRTLIRIMVS R**SETDLLDIR** SEYKRMYGK**S LYHDISGDTS GDYRK**ILLKI
501 CGGND

Residue Number Increasing Mass Decreasing Mass

Start - End	Observed	Mr(expt)	Mr(calc)	Delta	Miss	Sequence
192 - 204	1359.6776	1358.6703	1358.6830	-0.0127	0	GTITDAPGFDPLR (No match)
192 - 210	2043.0165	2042.0092	2042.0432	-0.0340	1	GTITDAPGFDPLRDAEVLR (No match)
215 - 230	1738.7729	1737.7656	1737.7991	-0.0335	0	GFGTDEQAIIDCLGSR (No match)
234 - 243	1260.7192	1259.7119	1259.7350	-0.0230	1	QRQQILLSFK (No match)
236 - 243	976.5594	975.5521	975.5753	-0.0231	0	QQILLSFK (No match)
283 - 302	2145.0740	2144.0667	2144.1146	-0.0479	1	EAIKGVGTDEACLIEILASR (No match)
287 - 302	1703.8306	1702.8233	1702.8559	-0.0326	0	GVGTDEACLIEILASR (Ions score 60)
287 - 302	1703.8306	1702.8233	1702.8559	-0.0326	0	GVGTDEACLIEILASR (No match)
321 - 327	831.4360	830.4287	830.4497	-0.0210	0	TLEEAIR (No match)
328 - 336	1034.4465	1033.4392	1033.4577	-0.0185	0	SDTSGHFQR (No match)
328 - 336	1034.4465	1033.4392	1033.4577	-0.0185	0	SDTSGHFQR (Ions score 65)
337 - 346	1100.6223	1099.6150	1099.6349	-0.0199	0	LLISLSQGNR (No match)
337 - 359	2547.2300	2546.2227	2546.2758	-0.0531	1	LLISLSQGNRDESTNVDMSLAQR (No match)
360 - 371	1336.5869	1335.5796	1335.6054	-0.0258	0	DAQELYAAGENR (No match)
360 - 371	1336.5869	1335.5796	1335.6054	-0.0258	0	DAQELYAAGENR (Ions score 53)
372 - 386	1696.7999	1695.7926	1695.8250	-0.0323	1	LGTDESKFNAVLCSR (No match)
379 - 386	966.4609	965.4536	965.4752	-0.0216	0	FNAVLCSR (No match)
387 - 400	1689.8475	1688.8402	1688.8746	-0.0344	1	SRAHLVAVFNEYQR (No match)
389 - 400	1446.7201	1445.7128	1445.7415	-0.0286	0	AHLVAVFNEYQR (Ions score 75)
389 - 400	1446.7201	1445.7128	1445.7415	-0.0286	0	AHLVAVFNEYQR (No match)
413 - 427	1607.7369	1606.7296	1606.7582	-0.0286	0	EMSGDLEEGMLAVVK (No match)
431 - 439	1052.4937	1051.4864	1051.5086	-0.0222	0	NTPAFFAER (No match)
431 - 439	1052.4937	1051.4864	1051.5086	-0.0222	0	NTPAFFAER (Ions score 36)
462 - 470	1061.5234	1060.5161	1060.5400	-0.0239	0	SETDLLDIR (No match)
480 - 495	1813.7966	1812.7893	1812.8278	-0.0385	1	SLYHDISGDTSGDYRK (No match)

Spot 417

Protein View

Match to: **gi|115527074** Score: **211** Expect: **1.5e-016**
src kinase associated phosphoprotein 1 isoform 1 [Homo sapiens]

Nominal mass (M_r): **41692**; Calculated pI value: **4.47**
NCBI BLAST search of gi|115527074 against nr
Unformatted sequence string for pasting into other applications

Taxonomy: Homo sapiens
Links to retrieve other entries containing this sequence from NCBI Entrez:
gi|119615155 from Homo sapiens
gi|119615156 from Homo sapiens

Fixed modifications: Carbamidomethyl (C)
Variable modifications: Oxidation (M)
Cleavage by Trypsin: cuts C-term side of KR unless next residue is P
Sequence Coverage: **18%**

Matched peptides shown in **Bold Red**

```
  1 MQAAALPEEI RWLLEDAEEF LAEGLRNENL SAVARDHRDH ILRGFQQIKA
 51 RYYWDFQPQG GDIGQDSSDD NHSGTLGLSL TSDAPFLSDY QDEGMEDIVK
101 GAQELDNVIK QGYLEKKSKD HSFFGSEWQK RWCVVSRGLF YYYANEKSKQ
151 PKGTFLIKGY GVRMAPHLRR DSKKESCFEL TSQDRRSYEF TATSPAEARD
201 WVDQISFLLK DLSSLTIPYE EDEEEEEKEE TYDDIDGFDS PSCGSQCRPT
251 ILPGSVGIKE PTEEKEEEDI YEVLPDEEHD LEEDESGTRR KGVDYASYYQ
301 GLWDCHGDQP DELSFQRGDL IRILSKEYNM YGWWVGELNS LVGIVPKEYL
351 TTAFEVEER
```

Residue Number Increasing Mass Decreasing Mass

Start - End	Observed	Mr(expt)	Mr(calc)	Delta	Miss	Sequence
36 - 43	1061.5385	1060.5312	1060.5526	-0.0214	1	DHRDHILR (No match)
120 - 130	1367.6924	1366.6851	1366.5942	0.0910	0	DHSFFGSEWQK (No match)
138 - 147	1267.5698	1266.5625	1266.5920	-0.0295	0	GLFYYYANEK (No match)
138 - 147	1267.5698	1266.5625	1266.5920	-0.0295	0	GLFYYYANEK (Ions score 26)
175 - 186	1527.6669	1526.6596	1526.6783	-0.0187	1	ESCFELTSQDRR (No match)
175 - 186	1527.6669	1526.6596	1526.6783	-0.0187	1	ESCFELTSQDRR (No match)

187 - 199	1429.6376	1428.6303	1428.6521	-0.0217	0	SYEFTATSPAEAR	(No match)
187 - 199	1429.6376	1428.6303	1428.6521	-0.0217	0	SYEFTATSPAEAR	(Ions score 8)
348 - 359	1486.6794	1485.6721	1485.6987	-0.0265	0	EYLTTAFEVEER	(Ions score 99)
348 - 359	1486.6794	1485.6721	1485.6987	-0.0265	0	EYLTTAFEVEER	(No match)

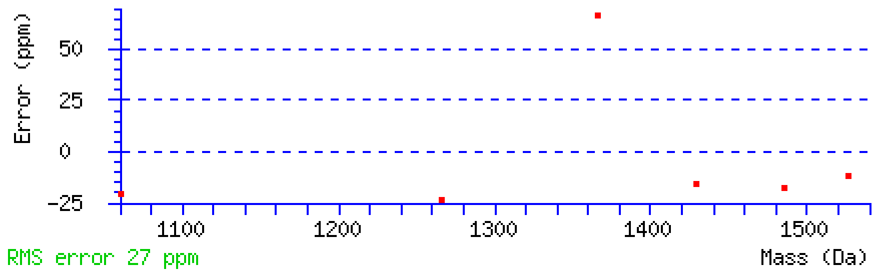

Spot 418

Protein View

Match to: **gi|54632179** Score: **165** Expect: **6.1e-012**
aging-associated gene 12 [Homo sapiens]

Nominal mass (M_r): **50022**; Calculated pI value: **6.91**
NCBI BLAST search of gi|54632179 against nr
Unformatted sequence string for pasting into other applications

Taxonomy: Homo sapiens

Fixed modifications: Carbamidomethyl (C)
Variable modifications: Oxidation (M)
Cleavage by Trypsin: cuts C-term side of KR unless next residue is P
Sequence Coverage: **15%**

Matched peptides shown in **Bold Red**

```
  1 MTLKASEGES GGSMHTALSD LYLEHLLQKR SRPEAVSHPL NTVTEDMYTN
 51 GSPAPGSPAQ VKGQEVRKVR LIQFEKVTEE PMGITLKLNE KQSCTVARIL
101 HGGMIHRQGS LHVGDEILEI NGTNVTNHSV DQLQKAMKET KGMISLKVIP
151 NQQSRLPALQ EAGLKFATGD IIQIINKDDS NWWRGRVEGS SKESAGLIPS
201 PELQEWRVAS MAQSAPSEAP SCSPFGKKKK YKDKYLAKHS SIFDQLDVVS
251 YEEVVRLPAF KRKTLVLIGA SGVGRSHIKN ALLSQNPEKF VYPVPYTTRP
301 PRKSEEDGKE YHFISTEEMT RNISANEFLE FGSYQGNMFG TKFETVHQIH
351 KQNKIAILDI EPQTLKIVRT AELSPFIVFI APTDQGTQTE ALQQLQKDSE
401 AIRSQYAHYF DLSLVNNGVD ETLKKLQEAF DQACSSPQWV PVSWVY
```

Residue Number Increasing Mass Decreasing Mass

Start - End	Observed	Mr(expt)	Mr(calc)	Delta	Miss	Sequence
99 - 107	1049.5481	1048.5408	1048.5600	-0.0191	0	ILHGGMIHR Oxidation (M) (No match)
99 - 107	1049.5481	1048.5408	1048.5600	-0.0191	0	ILHGGMIHR Oxidation (M) (No match)
148 - 155	941.5040	940.4967	940.5090	-0.0123	0	VIPNQQSR (No match)
239 - 256	2122.0173	2121.0100	2121.0378	-0.0277	0	HSSIFDQLDVVSYEEVVR (No match)
239 - 256	2122.0173	2121.0100	2121.0378	-0.0277	0	HSSIFDQLDVVSYEEVVR (Ions score 87)
264 - 275	1142.6752	1141.6679	1141.6818	-0.0139	0	TLVLIGASGVGR (No match)
290 - 302	1592.8390	1591.8317	1591.8510	-0.0193	0	FVYPVPYTTRPPR (Ions score 14)
290 - 302	1592.8390	1591.8317	1591.8510	-0.0193	0	FVYPVPYTTRPPR (No match)

343 - 351	1138.5546	1137.5473	1137.5930	-0.0457	0	FETVHQIHK	(No match)
343 - 351	1138.5546	1137.5473	1137.5930	-0.0457	0	FETVHQIHK	(No match)

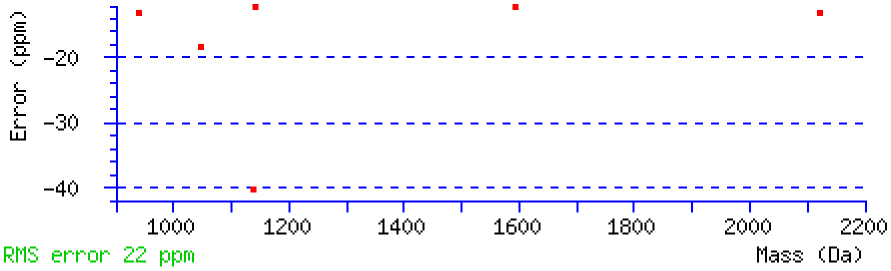

Spot 420

Protein View

Match to: **gi|5902134** Score: **420** Expect: **1.9e-037**
coronin, actin binding protein, 1A [Homo sapiens]

Nominal mass (M_r): **51678**; Calculated pI value: **6.25**
NCBI BLAST search of gi|5902134 against nr
Unformatted sequence string for pasting into other applications

Taxonomy: Homo sapiens
Links to retrieve other entries containing this sequence from NCBI Entrez:
gi|1706004 from Homo sapiens
gi|20271119 from Homo sapiens
gi|927649 from Homo sapiens
gi|1136140 from Homo sapiens
gi|82571468 from Homo sapiens
gi|116497053 from Homo sapiens
gi|116497211 from Homo sapiens
gi|119600312 from Homo sapiens
gi|119600313 from Homo sapiens
gi|119600314 from Homo sapiens
gi|119600316 from Homo sapiens

Fixed modifications: Carbamidomethyl (C)
Variable modifications: Oxidation (M)
Cleavage by Trypsin: cuts C-term side of KR unless next residue is P
Sequence Coverage: **34%**

Matched peptides shown in **Bold Red**

```
  1 MSRQVVRSSK FRHVFGQPAK ADQCYEDVRV SQTTWDSGFC AVNPKFVALI
 51 CEASGGGAFL VLPLGKTGRV DKNAPTVCGH TAPVLDIAWC PHNDNVIASG
101 SEDCTVMVWE IPDGGLMLPL REPVVTLEGH TKRVGIVAWH TTAQNVLLSA
151 GCDNVIMVWD VGTGAAMLTL GPEVHPDTIY SVDWSRDGGL ICTSCRDKRV
201 RIIEPRKGTV VAEKDRPHEG TRPVRAVFVS EGKILTTGFS RMSERQVALW
251 DTKHLEEPLS LQELDTSSGV LLPFFDPDTN IVYLCGKGDS SIRYFEITSE
301 APFLHYLSMF SSKESQRGMG YMPKRGLEVN KCEIARFYKL HERRCEPIAM
351 TVPRKSDLFQ EDLYPPTAGP DPALTAEEWL GGRDAGPLLI SLKDGYVPPK
401 SRELRVNRGL DTGRRRAAPE ASGTPSSDAV SRLEEEMRKL QATVQELQKR
451 LDRLEETVQA K
```

Residue Number Increasing Mass Decreasing Mass

Start - End	Observed	Mr(expt)	Mr(calc)	Delta	Miss	Sequence
11 - 20	1186.6267	1185.6194	1185.6406	-0.0212	1	FRHVFGQPAK (Ions score 31)
11 - 20	1186.6267	1185.6194	1185.6406	-0.0212	1	FRHVFGQPAK (No match)
13 - 20	883.4887	882.4814	882.4711	0.0103	0	HVFGQPAK (No match)
21 - 29	1155.4518	1154.4445	1154.4662	-0.0217	0	ADQCYEDVR (Ions score 43)
21 - 29	1155.4518	1154.4445	1154.4662	-0.0217	0	ADQCYEDVR (No match)
30 - 45	1796.7991	1795.7918	1795.8199	-0.0281	0	VSQTTWDSGFCAVNPK (No match)
187 - 196	1138.4761	1137.4688	1137.4906	-0.0218	0	DGGLICTSCR (Ions score 51)
187 - 196	1138.4761	1137.4688	1137.4906	-0.0218	0	DGGLICTSCR (No match)
215 - 225	1319.6660	1318.6587	1318.6854	-0.0267	0	DRPHEGTRPVR (No match)
215 - 225	1319.6660	1318.6587	1318.6854	-0.0267	0	DRPHEGTRPVR (Ions score 36)
234 - 241	894.4844	893.4771	893.4970	-0.0199	0	ILTTGFSR (No match)
246 - 253	960.4890	959.4817	959.5076	-0.0259	0	QVALWDTK (No match)
294 - 313	2397.1101	2396.1028	2396.1398	-0.0370	0	YFEITSEAPFLHYLSMFSSK (Ions score 89)
294 - 313	2397.1101	2396.1028	2396.1398	-0.0370	0	YFEITSEAPFLHYLSMFSSK (No match)
294 - 313	2413.1204	2412.1131	2412.1347	-0.0216	0	YFEITSEAPFLHYLSMFSSK Oxidation (M) (No match)
326 - 336	1288.6459	1287.6386	1287.6604	-0.0218	1	GLEVNKCEIAR (No match)
337 - 343	992.4838	991.4765	991.5239	-0.0474	1	FYKLHER (No match)
344 - 354	1329.6522	1328.6449	1328.6693	-0.0244	1	RCEPIAMTVPR (No match)
344 - 354	1345.6453	1344.6380	1344.6642	-0.0262	1	RCEPIAMTVPR Oxidation (M) (No match)
345 - 354	1173.5538	1172.5465	1172.5682	-0.0217	0	CEPIAMTVPR (No match)
345 - 354	1189.5577	1188.5504	1188.5631	-0.0127	0	CEPIAMTVPR Oxidation (M) (No match)
355 - 383	3173.4797	3172.4724	3172.5352	-0.0628	1	KSDLFQEDLYPPTAGPDPALTAEEWLGGR (No match)
384 - 393	1026.5952	1025.5879	1025.6120	-0.0241	0	DAGPLLISLK (No match)

Spot 421

Protein View

Match to: **gi|62896645** Score: **306** Expect: **4.8e-026**
sorting nexin 17 variant [Homo sapiens]

Nominal mass (M_r): **53121**; Calculated pI value: **7.07**
NCBI BLAST search of gi|62896645 against nr
Unformatted sequence string for pasting into other applications

Taxonomy: Homo sapiens

Fixed modifications: Carbamidomethyl (C)
Variable modifications: Oxidation (M)
Cleavage by Trypsin: cuts C-term side of KR unless next residue is P
Sequence Coverage: **25%**

Matched peptides shown in **Bold Red**

```
  1 MHFSIPETES RSGDSGGSAY VAYNIHVNGV LHCRVRYSQL LGLHEQLRKE
 51 YGANVLPAFP PKKLFSLTPA EVEQRREQLE KYMQAVRQDP LLGSSETFNS
101 FLRRAQQETQ QVPTEEVSLE VLLSNGQKVL VNVLTSDQTE DVLEAVAAKL
151 DLPDDLIGYF SLFLVREKED GAFSFVRKLQ EFELPYVSVT SLRSQEYKIV
201 LRKSYWDSAY DDDVMENRVG LNLLYAQTVS DIERGWILVT KEQHRQLKSL
251 QEKVSKKEFL RLAQTLRHYG YLRFDACVAD FPEKDCPVVV SAGNSELSLQ
301 LRLPGQQLRE GSFRVTRMRC WRVTSSVPLP SGSTSSPGRG RGEVRLELAF
351 EYLMSKDRLQ WVTITSPQAI MMSICLQSMV DELMVKKSGG SIRKMLRRRV
401 GGTLRRSDSQ QAVKSPPLLE SPDATRESVV KLSSKLSAVS LRGIGSPSTD
451 ASASDVHGNF AFEGIGDEDL
```

Residue Number Increasing Mass Decreasing Mass

Start - End	Observed	Mr(expt)	Mr(calc)	Delta	Miss	Sequence
37 - 48	1456.7305	1455.7232	1455.7834	-0.0601	0	YSQLLGLHEQLR (No match)
37 - 49	1584.8156	1583.8083	1583.8783	-0.0700	1	YSQLLGLHEQLRK (No match)
63 - 75	1517.7673	1516.7600	1516.8249	-0.0649	1	KLFSLTPAEVEQR (Ions score 47)
63 - 75	1517.7673	1516.7600	1516.8249	-0.0649	1	KLFSLTPAEVEQR (No match)
64 - 75	1389.6786	1388.6713	1388.7299	-0.0586	0	LFSLTPAEVEQR (No match)
64 - 75	1389.6786	1388.6713	1388.7299	-0.0586	0	LFSLTPAEVEQR (Ions score 26)
88 - 103	1810.8163	1809.8090	1809.8897	-0.0806	0	QDPLLGSSETFNSFLR (No match)

88 - 104	1966.9015	1965.8942	1965.9908	-0.0965	1	QDPLLGSSETFNSFLRR (No match)
167 - 177	1284.5680	1283.5607	1283.6145	-0.0538	1	EKEDGAFSFVR (No match)
167 - 177	1284.5680	1283.5607	1283.6145	-0.0538	1	EKEDGAFSFVR (Ions score 33)
178 - 193	1908.9564	1907.9491	1908.0356	-0.0865	1	KLQEFELPYVSVTSLR (No match)
178 - 193	1908.9564	1907.9491	1908.0356	-0.0865	1	KLQEFELPYVSVTSLR (Ions score 69)
179 - 193	1780.8699	1779.8626	1779.9406	-0.0780	0	LQEFELPYVSVTSLR (No match)
219 - 234	1790.8890	1789.8817	1789.9573	-0.0756	0	VGLNLLYAQTVSDIER (No match)
318 - 322	808.3727	807.3654	807.3632	0.0022	1	MRCWR (No match)
323 - 339	1615.7611	1614.7538	1614.8213	-0.0674	0	VTSSVPLPSGSTSSPGR (No match)
415 - 426	1282.6056	1281.5983	1281.6564	-0.0581	0	SPPLLESPDATR (No match)

Spot 422

Protein View

Match to: **gi|33876034** Score: **69** Expect: **0.024**
HEXA protein [Homo sapiens]

Nominal mass (M_r): **47407**; Calculated pI value: **4.88**
NCBI BLAST search of gi|33876034 against nr
Unformatted sequence string for pasting into other applications

Taxonomy: Homo sapiens

Fixed modifications: Carbamidomethyl (C)
Variable modifications: Oxidation (M)
Cleavage by Trypsin: cuts C-term side of KR unless next residue is P
Sequence Coverage: **10%**

Matched peptides shown in **Bold Red**

```
  1 NDDQCLLLSE TVWGALRGLE TFSQLVWKSA EGTFFINKTE IEDFPRFPHR
 51 GLLLDTSRHY LPLSSILDTL DVMAYNKLNV FHWHLVDDPS FPYESFTFPE
101 LMRKGSYNPV THIYTAQDVK EVIEYARLRG IRVLAEFDTP GHTLSWGPGI
151 PGLLTPCYSG SEPSGTFGPV NPSLNNTYEF MSTFFLEVSS VFPDFYLHLG
201 GDEVDFTCWK SNPEIQDFMR KKGFGEDFKQ LESFYIQTLL DIVSSYGKGY
251 VVWQEVFDNK VKIQPDTIIQ VWREDIPVNY MKELELVTKA GFRALLSAPW
301 YLNRISYGPD WKDFYVVEPL AFEGTPEQKA LVIGGEACMW GEYVDNTNLV
351 PRLWPRAGAV AERLWSNKLT SDLTFAYERL SHFRCELLRR GVQAQPLNVG
401 FCEQEFEQT
```

Residue Number Increasing Mass Decreasing Mass

Start - End	Observed	Mr(expt)	Mr(calc)	Delta	Miss	Sequence
18 - 28	1307.6942	1306.6869	1306.6921	-0.0052	0	GLETFSQLVWK (No match)
18 - 28	1307.6942	1306.6869	1306.6921	-0.0052	0	GLETFSQLVWK (No match)
39 - 50	1543.7473	1542.7400	1542.7579	-0.0179	1	TEIEDFPRFPHR (Ions score 8)
39 - 50	1543.7473	1542.7400	1542.7579	-0.0179	1	TEIEDFPRFPHR (No match)
211 - 220	1252.5419	1251.5346	1251.5553	-0.0207	0	SNPEIQDFMR Oxidation (M) (No match)
369 - 379	1315.6384	1314.6311	1314.6455	-0.0144	0	LTSDLTFAYER (Ions score 15)
369 - 379	1315.6384	1314.6311	1314.6455	-0.0144	0	LTSDLTFAYER (No match)

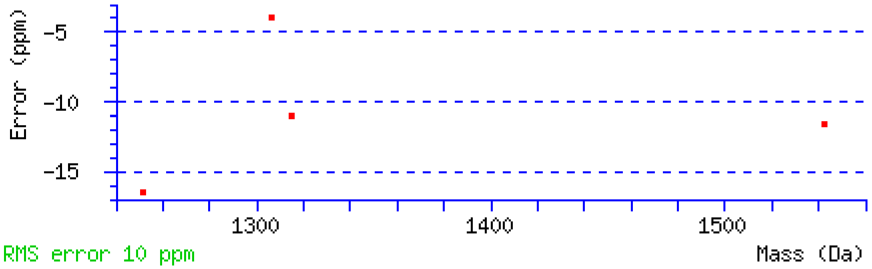

Error (ppm)
-5
-10
-15
1300
1400
1500
RMS error 10 ppm
Mass (Da)

Spot 424

Protein View

Match to: **gi|5453603** Score: **431** Expect: **1.5e-038**
chaperonin containing TCP1, subunit 2 [Homo sapiens]

Nominal mass (M_r): **57794;** Calculated pI value: **6.01**
NCBI BLAST search of gi|5453603 against nr
Unformatted sequence string for pasting into other applications

Taxonomy: Homo sapiens
Links to retrieve other entries containing this sequence from NCBI Entrez:
gi|6094436 from Homo sapiens
gi|75076361 from Macaca fascicularis
gi|2559012 from Homo sapiens
gi|4090929 from Homo sapiens
gi|54696794 from Homo sapiens
gi|67969939 from Macaca fascicularis
gi|109730511 from Homo sapiens
gi|109731722 from Homo sapiens
gi|119617635 from Homo sapiens

Fixed modifications: Carbamidomethyl (C)
Variable modifications: Oxidation (M)
Cleavage by Trypsin: cuts C-term side of KR unless next residue is P
Sequence Coverage: **42%**

Matched peptides shown in **Bold Red**

```
  1 MASLSLAPVN IFKAGADEER AETARLTSFI GAIAIGDLVK STLGPKGMDK
 51 ILLSSGRDAS LMVTNDGATI LKNIGVDNPA AKVLVDMSRV QDDEVGDGTT
101 SVTVLAAELL REAESLIAKK IHPQTIIAGW REATKAAREA LLSSAVDHGS
151 DEVKFRQDLM NIAGTTLSSK LLTHHKDHFT KLAVEAVLRL KGSGNLEAIH
201 IIKKLGGSLA DSYLDEGFLL DKKIGVNQPK RIENAKILIA NTGMDTDKIK
251 IFGSRVRVDS TAKVAEIEHA EKEKMKEKVE RILKHGINCF INRQLIYNYP
301 EQLFGAAGVM AIEHADFAGV ERLALVTGGE IASTFDHPEL VKLGSCKLIE
351 EVMIGEDKLI HFSGVALGEA CTIVLRGATQ QILDEAERSL HDALCVLAQT
401 VKDSRTVYGG GCSEMLMAHA VTQLANRTPG KEAVAMESYA KALRMLPTII
451 ADNAGYDSAD LVAQLRAAHS EGNTTAGLDM REGTIGDMAI LGITESFQVK
501 RQVLLSAAEA AEVILRVDNI IKAAPRKRVP DHHPC
```

Residue Number Increasing Mass Decreasing Mass

Start - End	Observed	Mr(expt)	Mr(calc)	Delta	Miss	Sequence
14 - 25	1275.5499	1274.5426	1274.5850	-0.0424	1	AGADEERAETAR (No match)
14 - 25	1275.5499	1274.5426	1274.5850	-0.0424	1	AGADEERAETAR (Ions score 18)
26 - 40	1517.8384	1516.8311	1516.8864	-0.0553	0	LTSFIGAIAIGDLVK (No match)
83 - 89	835.4023	834.3950	834.4269	-0.0319	0	VLVDMSR Oxidation (M) (No match)
90 - 111	2288.0688	2287.0615	2287.1543	-0.0927	0	VQDDEVGDGTTSVTVLAAELLR (Ions score 116)
90 - 111	2288.0688	2287.0615	2287.1543	-0.0927	0	VQDDEVGDGTTSVTVLAAELLR (No match)
139 - 156	1959.9044	1958.8971	1958.9697	-0.0726	1	EALLSSAVDHGSDEVKFR (No match)
155 - 170	1797.8517	1796.8444	1796.9090	-0.0646	1	FRQDLMNIAGTTLSSK Oxidation (M) (No match)
182 - 189	870.5126	869.5053	869.5334	-0.0281	0	LAVEAVLR (No match)
205 - 222	1912.9221	1911.9148	1911.9465	-0.0317	0	LGGSLADSYLDEGFLLDK (No match)
205 - 223	2040.9785	2039.9712	2040.0414	-0.0702	1	LGGSLADSYLDEGFLLDKK (No match)
237 - 248	1307.6771	1306.6698	1306.6438	0.0260	0	ILIANTGMDTDK Oxidation (M) (No match)
282 - 293	1484.7651	1483.7578	1483.8081	-0.0503	1	ILKHGINCFINR (No match)
285 - 293	1130.5167	1129.5094	1129.5450	-0.0356	0	HGINCFINR (No match)
285 - 293	1130.5167	1129.5094	1129.5450	-0.0356	0	HGINCFINR (Ions score 30)
323 - 342	2097.0452	2096.0379	2096.1153	-0.0774	0	LALVTGGEIASTFDHPELVK (Ions score 69)
323 - 342	2097.0452	2096.0379	2096.1153	-0.0774	0	LALVTGGEIASTFDHPELVK (No match)
377 - 388	1330.6179	1329.6106	1329.6524	-0.0418	0	GATQQILDEAER (No match)
377 - 388	1330.6179	1329.6106	1329.6524	-0.0418	0	GATQQILDEAER (Ions score 17)
445 - 466	2363.1147	2362.1074	2362.1838	-0.0764	0	MLPTIIADNAGYDSADLVAQLR Oxidation (M) (No match)
482 - 501	2181.0400	2180.0327	2180.1146	-0.0819	1	EGTIGDMAILGITESFQVKR Oxidation (M) (No match)
502 - 516	1582.8625	1581.8552	1581.9089	-0.0537	0	QVLLSAAEAAEVILR (No match)

Spot 426

Protein View

Match to: **gi|4757810** Score: **423** Expect: **9.6e-038**
ATP synthase, H+ transporting, mitochondrial F1 complex, alpha subunit precursor [Homo sapiens]

Nominal mass (M_r): **59828**; Calculated pI value: **9.16**
NCBI BLAST search of gi|4757810 against nr
Unformatted sequence string for pasting into other applications

Taxonomy: Homo sapiens
Links to retrieve other entries containing this sequence from NCBI Entrez:
gi|50345984 from Homo sapiens
gi|158514235 (no taxonomy information for this entry)
gi|114517 from Homo sapiens
gi|28938 from Homo sapiens
gi|34468 from Homo sapiens
gi|559317 from Homo sapiens
gi|559325 from Homo sapiens
gi|16359160 from Homo sapiens
gi|17939539 from Homo sapiens
gi|30583257 from Homo sapiens
gi|40352910 from Homo sapiens
gi|45709753 from Homo sapiens
gi|61362466 from synthetic construct
gi|61362474 from synthetic construct
gi|119621876 from Homo sapiens
gi|123995069 from synthetic construct
gi|124126803 from synthetic construct
gi|146741384 from Pan troglodytes verus
gi|1090507 from Homo sapiens

Fixed modifications: Carbamidomethyl (C)
Variable modifications: Oxidation (M)
Cleavage by Trypsin: cuts C-term side of KR unless next residue is P
Sequence Coverage: **46%**

Matched peptides shown in **Bold Red**

1 MLSVRVAAAV VRALPRRAGL VSRNALGSSF IAARNFHASN THLQKTGTAE
51 MSSILEER**IL GADTSVDLEE TGRVLSIGDG IAR**VHGLR**NV QAEEMVEFSS**
101 **GLKGMSLNLE PDNVGVVVFG NDK**LIKEGDI VK**RTGAIVDV PVGEELLGR**V
151 VDALGNAIDG KGPIGSKTRR R**VGLKAPGII PRISVREPMQ TGIKAVDSLV**

```
201 PIGRGQRELI IGDRQTGKTS IAIDTIINQK RFNDGSDEKK KLYCIYVAIG
251 QKRSTVAQLV KRLTDADAMK YTIVVSATAS DAAPLQYLAP YSGCSMGEYF
301 RDNGKHALII YDDLSKQAVA YRQMSLLLRR PPGREAYPGD VFYLHSRLLE
351 RAAKMNDAFG GGSLTALPVI ETQAGDVSAY IPTNVISITD GQIFLETELF
401 YKGIRPAINV GLSVSRVGSA AQTRAMKQVA GTMKLELAQY REVAAFAQFG
451 SDLDAATQQL LSRGVRLTEL LKQGQYSPMA IEEQVAVIYA GVRGYLDKLE
501 PSKITKFENA FLSHVVSQHQ ALLGTIRADG KISEQSDAKL KEIVTNFLAG
551 FEA
```

Residue Number Increasing Mass Decreasing Mass

Start - End	Observed	Mr(expt)	Mr(calc)	Delta	Miss	Sequence
59 - 73	1575.7379	1574.7306	1574.7787	-0.0481	0	ILGADTSVDLEETGR (No match)
74 - 83	1000.5486	999.5413	999.5712	-0.0299	0	VLSIGDGIAR (No match)
89 - 103	1683.7369	1682.7296	1682.7821	-0.0524	0	NVQAEEMVEFSSGLK Oxidation (M) (No match)
104 - 123	2119.9780	2118.9707	2119.0255	-0.0547	0	GMSLNLEPDNVGVVVFGNDK Oxidation (M) (No match)
133 - 149	1780.9344	1779.9271	1779.9842	-0.0571	1	RTGAIVDVPVGEELLGR (No match)
134 - 149	1624.8370	1623.8297	1623.8831	-0.0534	0	TGAIVDVPVGEELLGR (Ions score 57)
134 - 149	1624.8370	1623.8297	1623.8831	-0.0534	0	TGAIVDVPVGEELLGR (No match)
172 - 182	1120.6887	1119.6814	1119.7127	-0.0313	1	VGLKAPGIIPR (No match)
183 - 194	1374.6971	1373.6898	1373.7336	-0.0438	1	ISVREPMQTGIK Oxidation (M) (No match)
195 - 204	1026.5671	1025.5598	1025.5869	-0.0271	0	AVDSLVPIGR (No match)
208 - 214	815.4433	814.4360	814.4548	-0.0188	0	ELIIGDR (No match)
219 - 231	1472.8018	1471.7945	1471.8358	-0.0413	1	TSIAIDTIINQKR (No match)
242 - 253	1483.7657	1482.7584	1482.8016	-0.0432	1	LYCIYVAIGQKR (No match)
323 - 329	876.4692	875.4619	875.4898	-0.0279	0	QMSLLLR Oxidation (M) (No match)
335 - 347	1553.6910	1552.6837	1552.7310	-0.0472	0	EAYPGDVFYLHSR (No match)
335 - 347	1553.6910	1552.6837	1552.7310	-0.0472	0	EAYPGDVFYLHSR (Ions score 49)
403 - 416	1438.8049	1437.7976	1437.8415	-0.0439	0	GIRPAINVGLSVSR (Ions score 2)
403 - 416	1438.8049	1437.7976	1437.8415	-0.0439	0	GIRPAINVGLSVSR (No match)
428 - 441	1623.8002	1622.7929	1622.8450	-0.0520	1	QVAGTMKLELAQYR Oxidation (M) (No match)
435 - 441	892.4651	891.4578	891.4814	-0.0235	0	LELAQYR (No match)
442 - 463	2338.0757	2337.0684	2337.1600	-0.0916	0	EVAAFAQFGSDLDAATQQLLSR (No match)
442 - 463	2338.0757	2337.0684	2337.1600	-0.0916	0	EVAAFAQFGSDLDAATQQLLSR (Ions score 81)
473 - 493	2325.0774	2324.0701	2324.1470	-0.0768	0	QGQYSPMAIEEQVAVIYAGVR Oxidation (M) (No match)
504 - 527	2709.3655	2708.3582	2708.4761	-0.1179	1	ITKFENAFLSHVVSQHQALLGTIR (No match)
507 - 527	2367.1624	2366.1551	2366.2494	-0.0943	0	FENAFLSHVVSQHQALLGTIR (No match)
507 - 527	2367.1624	2366.1551	2366.2494	-0.0943	0	FENAFLSHVVSQHQALLGTIR (Ions score 23)

Spot 428

Protein View

Match to: **gi|4757766** Score: **108** Expect: **3e-006**
Rho GTPase activating protein 1 [Homo sapiens]

Nominal mass (M_r): **50461**; Calculated pI value: **5.85**
NCBI BLAST search of gi|4757766 against nr
Unformatted sequence string for pasting into other applications

Taxonomy: Homo sapiens
Links to retrieve other entries containing this sequence from NCBI Entrez:
gi|3024550 from Homo sapiens
gi|312212 from Homo sapiens
gi|17390260 from Homo sapiens
gi|119588389 from Homo sapiens
gi|119588390 from Homo sapiens
gi|123994939 from synthetic construct

Fixed modifications: Carbamidomethyl (C)
Variable modifications: Oxidation (M)
Cleavage by Trypsin: cuts C-term side of KR unless next residue is P
Sequence Coverage: **11%**

Matched peptides shown in **Bold Red**

```
  1 MDPLSELQDD LTLDDTSEAL NQLKLASIDE KNWPSDEMPD FPKSDDSKSS
 51 SPELVTHLKW DDPYYDIARH QIVEVAGDDK YGRKIIVFSA CRMPPSHQLD
101 HSKLLGYLKH TLDQYVESDY TLLYLHHGLT SDNKPSLSWL RDAYREFDRK
151 YKKNIKALYI VHPTMFIKTL LILFKPLISF KFGQKIFYVN YLSELSEHVK
201 LEQLGIPRQV LKYDDFLKST QKSPATAPKP MPPRPPLPNQ QFGVSLQHLQ
251 EKNPEQEPIP IVLRETVAYL QAHALTTEGI FRRSANTQVV REVQQKYNMG
301 LPVDFDQYNE LHLPAVILKT FLRELPEPLL TFDLYPHVVG FLNIDESQRV
351 PATLQVLQTL PEENYQVLRF LTAFLVQISA HSDQNKMTNT NLAVVFGPNL
401 LWAKDAAITL KAINPINTFT KFLLDHQGEL FPSPDPSGL
```

Residue Number Increasing Mass Decreasing Mass

Start - End	Observed	Mr(expt)	Mr(calc)	Delta	Miss	Sequence	
60 - 69	1313.5493	1312.5420	1312.5724	-0.0304	0	WDDPYYDIAR	(No match)

60 - 69	1313.5493	1312.5420	1312.5724	-0.0304	0	**WDDPYYDIAR** (Ions score 25)
85 - 92	965.5020	964.4947	964.5164	-0.0216	0	**IIVFSACR** (No match)
253 - 264	1404.7494	1403.7421	1403.7772	-0.0351	0	**NPEQEPIPIVLR** (Ions score 34)
253 - 264	1404.7494	1403.7421	1403.7772	-0.0351	0	**NPEQEPIPIVLR** (No match)
350 - 369	2311.1995	2310.1922	2310.2583	-0.0660	0	**VPATLQVLQTLPEENYQVLR** (No match)
350 - 369	2311.1995	2310.1922	2310.2583	-0.0660	0	**VPATLQVLQTLPEENYQVLR** (Ions score 4)

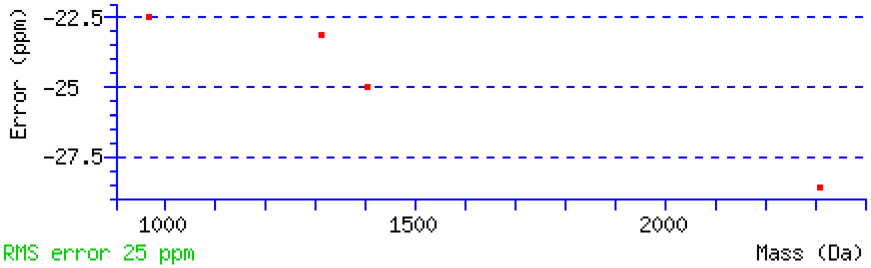

RMS error 25 ppm

Spot 429

Protein View

Match to: **gi|62702281** Score: **173** Expect: **9.6e-013**
unknown [Homo sapiens]

Nominal mass (M_r): **49362**; Calculated pI value: **8.78**
NCBI BLAST search of gi|62702281 against nr
Unformatted sequence string for pasting into other applications

Taxonomy: Homo sapiens

Fixed modifications: Carbamidomethyl (C)
Variable modifications: Oxidation (M)
Cleavage by Trypsin: cuts C-term side of KR unless next residue is P
Sequence Coverage: **16%**

Matched peptides shown in **Bold Red**

```
  1 MSRFVQDLSK AMSQDGASQF QEVIRQELEL SVKKELEKIL TTASSHEFEH
 51 TKKDLDGFRK LFHRFLQEKG PSVDWGKIQR PPEDSIQPYE KIKARGLPDN
101 ISSVLNKLVV VKLNGGLGTS MGCKGPKSLI GVRNENTFLD LTVQQIEHLN
151 KTYNTDVPLV LMNSFNTDED TKKILQKYNH CRVKIYTFNQ SRYPRINKES
201 LLPVAKDVSY SGENTEAWYP PGHGDIYASF YNSGLLDTFI GEGKEYIFVS
251 NIDNLGATVD LYILNHLMNP PNGKRCEFVM EVTNKTRADV KGGTLTQYEG
301 KLRLVEIAQV PKAHVDEFKS VSKFKIFNTN NLWISLAAVK RLQEQNAIDM
351 EIIVNAKTLD GGLNVIQLET AVGAAIKSFE NSLGINVPRS RFLPVKTTSD
401 LLLVMSNLYS LNAGSLTMSE KREFPTVPLV KLGSSFTK
```

Residue Number Increasing Mass Decreasing Mass

Start - End	Observed	Mr(expt)	Mr(calc)	Delta	Miss	Sequence
78 - 91	1699.8519	1698.8446	1698.8577	-0.0130	0	IQRPPEDSIQPYEK (Ions score 31)
78 - 91	1699.8519	1698.8446	1698.8577	-0.0130	0	IQRPPEDSIQPYEK (No match)
134 - 151	2156.0835	2155.0762	2155.0909	-0.0147	0	NENTFLDLTVQQIEHLNK (No match)
183 - 192	1255.6785	1254.6712	1254.6720	-0.0008	1	VKIYTFNQSR (No match)
185 - 192	1028.5116	1027.5043	1027.5087	-0.0043	0	IYTFNQSR (No match)
185 - 192	1028.5116	1027.5043	1027.5087	-0.0043	0	IYTFNQSR (Ions score 30)
358 - 377	1983.0984	1982.0911	1982.1047	-0.0136	0	TLDGGLNVIQLETAVGAAIK (No match)
378 - 389	1332.6829	1331.6756	1331.6833	-0.0077	0	SFENSLGINVPR (Ions score 37)

378 - 389	1332.6829	1331.6756	1331.6833	-0.0077	0	SFENSLGINVPR	(No match)

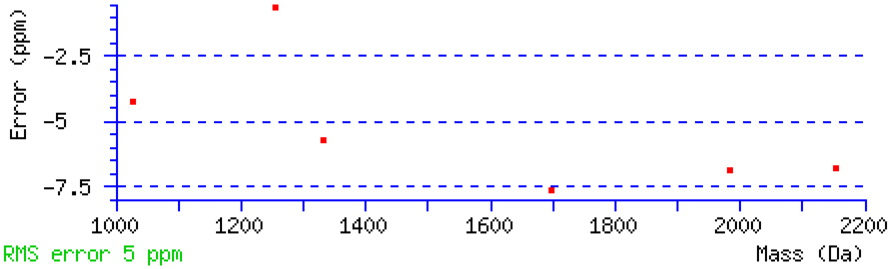

RMS error 5 ppm

Spot 430

Protein View

Match to: **gi|8922699** Score: **415** Expect: **6.1e-037**
CNDP dipeptidase 2 (metallopeptidase M20 family) [Homo sapiens]

Nominal mass (M_r): **53088**; Calculated pI value: **5.58**
NCBI BLAST search of gi|8922699 against nr
Unformatted sequence string for pasting into other applications

Taxonomy: Homo sapiens
Links to retrieve other entries containing this sequence from NCBI Entrez:
gi|7023109 from Homo sapiens

Fixed modifications: Carbamidomethyl (C)
Variable modifications: Oxidation (M)
Cleavage by Trypsin: cuts C-term side of KR unless next residue is P
Sequence Coverage: **34%**

Matched peptides shown in **Bold Red**

```
  1 MAALTTLFKY IDENQDRYIK KLAKWVAIQS VSAWPEKRGE IRRMMEVAAA
 51 DVKQLGGSVE LVDIGKQKLP DGSEIPLPPI LLGRLGSDPQ KKTVCIYGHL
101 DVQPAALEDG WDSEPFTLVE RDGKLYGGGS TDDKGPVAGW INALEAYQKT
151 GQEIPVNVRF CLEGMEESGS EGLDELIFAR KDTFFKDVDY VCISDNYWLG
201 KKKPCITYGL RGICYFFIEV ECSNKDLHSG VYGGSVHEAM TDLILLMGSL
251 VDKRGNILIP GINEAVAAVT EEEHKLYDDI DFDIEEFAKD VGAQILLHSH
301 KKDILMHRWR YPSLSLHGIE GAFSGSGAKT VIPRKVVGKF SIRLVPNMTP
351 EVVGEQVTSY LTKKFAELRS PNEFKVYMGH GGKPWVSDFS HPHYLAGRRA
401 MKTVFGVEPD LTREGGSIPV TLTFQEATGK NVMLLPVGSA DDGAHSQNEK
451 LNRYNYIEGT KMLAAYLYEV SQLKD
```

Residue Number Increasing Mass Decreasing Mass

Start - End	Observed	Mr(expt)	Mr(calc)	Delta	Miss	Sequence
10 - 17	1052.4207	1051.4134	1051.4570	-0.0436	0	YIDENQDR (No match)
67 - 84	1943.0667	1942.0594	1942.1251	-0.0657	1	QKLPDGSEIPLPPILLGR (No match)
67 - 84	1943.0667	1942.0594	1942.1251	-0.0657	1	QKLPDGSEIPLPPILLGR (Ions score 113)
69 - 84	1686.9207	1685.9134	1685.9715	-0.0581	0	LPDGSEIPLPPILLGR (No match)
69 - 84	1686.9207	1685.9134	1685.9715	-0.0581	0	LPDGSEIPLPPILLGR (Ions score 93)

150 - 159	1112.5690	1111.5617	1111.5985	-0.0368	0	TGQEIPVNVR	(Ions score 34)
150 - 159	1112.5690	1111.5617	1111.5985	-0.0368	0	TGQEIPVNVR	(No match)
202 - 211	1235.6395	1234.6322	1234.6856	-0.0533	1	KKPCITYGLR	(No match)
203 - 211	1107.5659	1106.5586	1106.5906	-0.0320	0	KPCITYGLR	(No match)
255 - 275	2204.0747	2203.0674	2203.1483	-0.0809	0	GNILIPGINEAVAAVTEEEHK	(No match)
311 - 329	1877.8750	1876.8677	1876.9318	-0.0641	0	YPSLSLHGIEGAFSGSGAK	(No match)
344 - 363	2221.0488	2220.0415	2220.1347	-0.0932	0	LVPNMTPEVVGEQVTSYLTK Oxidation (M)	(No match)
403 - 413	1233.6024	1232.5951	1232.6400	-0.0449	0	TVFGVEPDLTR	(Ions score 32)
403 - 413	1233.6024	1232.5951	1232.6400	-0.0449	0	TVFGVEPDLTR	(No match)
414 - 430	1734.8184	1733.8111	1733.8835	-0.0724	0	EGGSIPVTLTFQEATGK	(No match)
431 - 450	2097.9114	2096.9041	2096.9796	-0.0755	0	NVMLLPVGSADDGAHSQNEK Oxidation (M)	(No match)
451 - 461	1370.6610	1369.6537	1369.6989	-0.0452	1	LNRYNYIEGTK	(No match)

Spot 431

Protein View

Match to: **gi|55274010** Score: **119** Expect: **2.4e-007**
ENC-1AS [Homo sapiens]

Nominal mass (M_r): **38519**; Calculated pI value: **6.02**
NCBI BLAST search of gi|55274010 against nr
Unformatted sequence string for pasting into other applications

Taxonomy: Homo sapiens

Fixed modifications: Carbamidomethyl (C)
Variable modifications: Oxidation (M)
Cleavage by Trypsin: cuts C-term side of KR unless next residue is P
Sequence Coverage: **9%**

Matched peptides shown in **Bold Red**

```
  1 MAFNKFNVLH WHIVDDQSFP YQSITFPELS NKGSYSLSHV YTPNDVRMVI
 51 EYARLRGIRV LPEFDTPGHT LSWGKGQKDL LTPCYSRQNK LDSFGPINPT
101 LNTTYSFLTT FFKEISEVFP DQFIHLGGDE VEFKCWESNP KIQDFMRQKG
151 FGTDFKKLES FYIQKVLDII ATINKGSIVW QEVFDDKAKL APGTIVEVWK
201 DSAYPEELSR VTASGFPVIL SAPWYLDLIS YGQDWRKYYK VEPLDFGGTQ
251 KQKQLFIGGE ACLWGEYVDA TNLTPRLWPR ASAVGERLWS SKDVRDMDDA
301 YDRLTRHRCR MVERGIAAQP LYAGYCNHEN M
```

Residue Number Increasing Mass Decreasing Mass

Start - End	Observed	Mr(expt)	Mr(calc)	Delta	Miss	Sequence
33 - 47	1694.7942	1693.7869	1693.8059	-0.0190	0	GSYSLSHVYTPNDVR (No match)
33 - 47	1694.7942	1693.7869	1693.8059	-0.0190	0	GSYSLSHVYTPNDVR (Ions score 66)
79 - 87	1124.5242	1123.5169	1123.5332	-0.0162	0	DLLTPCYSR (No match)
79 - 87	1124.5242	1123.5169	1123.5332	-0.0162	0	DLLTPCYSR (Ions score 17)
142 - 147	825.3876	824.3803	824.3850	-0.0047	0	IQDFMR Oxidation (M) (No match)

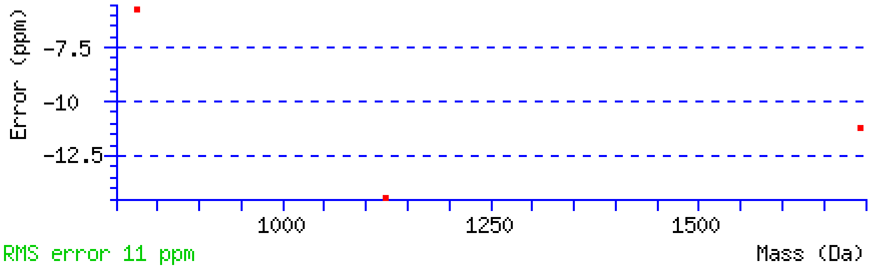
Error (ppm)
-7.5
-10
-12.5
1000
1250
1500
RMS error 11 ppm
Mass (Da)

Spot 432

Protein View

Match to: **gi|37588925** Score: **320** Expect: **1.9e-027**
LAP3 protein [Homo sapiens]

Nominal mass (M_r): **54754;** Calculated pI value: **6.80**
NCBI BLAST search of gi|37588925 against nr
Unformatted sequence string for pasting into other applications

Taxonomy: Homo sapiens

Fixed modifications: Carbamidomethyl (C)
Variable modifications: Oxidation (M)
Cleavage by Trypsin: cuts C-term side of KR unless next residue is P
Sequence Coverage: **39%**

Matched peptides shown in **Bold Red**

```
  1 LAVRRFGSRS LSTADMTKGL VLGIYSKEKE DDVPQFTSAG ENFDKLLAGK
 51 LRETLNISGP PLKAGKTRTF YGLHQDFPSV VLVGLGKKAA GIDEQENWHE
101 GKENIRAAVA AGCRQIQDLE LSSVEVDPCG DAQAAAEGAV LGLYEYDDLK
151 QKKKMAVSAK LYGSGDQEAW QKGVLFASGQ NLARQLMETP ANEMTPTRFA
201 EIIEKNLKSA SSKTEVHIRP KSWIEEQAMG SFLSVAKGSD EPPVFLEIHY
251 KGSPNANEPP LVFVGKGITF DSGGISIKAS ANMDLMRADM GGAATICSAI
301 VSAAKLNLPI NIIGLAPLCE NMPSGKANKP GDVVRAKNGK TIQVDNTDAE
351 GRLILADALC YAHTFNPKVI LNAATLTGAM DVALGSGATG VFTNSSWLWN
401 KLFEASIETG DRVWRMPLFE HYTRQVVDCQ LADVNNIGKY RSAGACTAAA
451 FLKEFVTHPK WAHLDIAGVM TNKDEVPYLR KGMTGRPTRT LIEFLLRFSQ
501 DNA
```

Residue Number Increasing Mass Decreasing Mass

Start - End	Observed	Mr(expt)	Mr(calc)	Delta	Miss	Sequence
28 - 45	2055.8359	2054.8286	2054.9068	-0.0782	1	EKEDDVPQFTSAGENFDK (No match)
51 - 63	1437.7899	1436.7826	1436.8351	-0.0524	1	LRETLNISGPPLK (No match)
69 - 87	2077.0239	2076.0166	2076.1043	-0.0877	0	TFYGLHQDFPSVVLVGLGK (No match)
173 - 184	1232.6304	1231.6231	1231.6672	-0.0441	0	GVLFASGQNLAR (Ions score 41)
173 - 184	1232.6304	1231.6231	1231.6672	-0.0441	0	GVLFASGQNLAR (No match)
185 - 198	1650.6808	1649.6735	1649.7389	-0.0654	0	QLMETPANEMTPTR 2 Oxidation (M) (No match)

199 - 205	849.4415	848.4342	848.4643	-0.0301	0	**FAEIIEK**	(No match)
214 - 221	979.5276	978.5203	978.5610	-0.0407	0	**TEVHIRPK**	(No match)
238 - 251	1630.7478	1629.7405	1629.8038	-0.0633	0	**GSDEPPVFLEIHYK**	(Ions score 65)
238 - 251	1630.7478	1629.7405	1629.8038	-0.0633	0	**GSDEPPVFLEIHYK**	(No match)
252 - 266	1525.7367	1524.7294	1524.7935	-0.0641	0	**GSPNANEPPLVFVGK**	(No match)
267 - 278	1194.5837	1193.5764	1193.6291	-0.0527	0	**GITFDSGGISIK**	(No match)
327 - 335	955.4970	954.4897	954.5246	-0.0349	0	**ANKPGDVVR**	(No match)
341 - 352	1318.5718	1317.5645	1317.6160	-0.0515	0	**TIQVDNTDAEGR**	(No match)
353 - 368	1846.8800	1845.8727	1845.9447	-0.0720	0	**LILADALCYAHTFNPK**	(No match)
402 - 412	1237.5701	1236.5628	1236.5986	-0.0357	0	**LFEASIETGDR**	(No match)
416 - 424	1209.5231	1208.5158	1208.5648	-0.0490	0	**MPLFEHYTR** Oxidation (M)	(Ions score 32)
416 - 424	1209.5231	1208.5158	1208.5648	-0.0490	0	**MPLFEHYTR** Oxidation (M)	(No match)
490 - 497	1004.5760	1003.5687	1003.6066	-0.0378	0	**TLIEFLLR**	(Ions score 18)
490 - 497	1004.5760	1003.5687	1003.6066	-0.0378	0	**TLIEFLLR**	(No match)

Spot 433

Protein View

Match to: **gi|57209813** Score: **161** Expect: **1.5e-011**
tubulin, beta polypeptide [Homo sapiens]

Nominal mass (M_r): **48135**; Calculated pI value: **4.70**
NCBI BLAST search of gi|57209813 against nr
Unformatted sequence string for pasting into other applications

Taxonomy: Homo sapiens
Links to retrieve other entries containing this sequence from NCBI Entrez:
gi|123270825 from Homo sapiens
gi|123281140 from Homo sapiens
gi|123293908 from Homo sapiens

Fixed modifications: Carbamidomethyl (C)
Variable modifications: Oxidation (M)
Cleavage by Trypsin: cuts C-term side of KR unless next residue is P
Sequence Coverage: **33%**

Matched peptides shown in **Bold Red**

```
  1 MFWEVISDEH GIDPTGTYHG DSDLQLDRIS VVYYNEATGGK YVPRAILVDL
 51 EPGTMDSVRS GPFGQIFRPD NFVFGQSGAG NNWAKGHYTE GAELVDSVLD
101 VVRKEAESCD CLQGFQLTHS LGGGTGSGMG TLLISKIREE YPDRIMNTFS
151 VVPSPKVSDT VVEPYNATLS VHQLVENTDE TYCIDNEALY DICFRTLKLT
201 TPTYGDLNHL VSATMSGVTT CLRFPGQLNA DLRKLAVNMV PFPRLHFFMP
251 GFAPLTSRGS QQYRALTVPE LTQQVFDAKN MMAACDPRHG RYLTVAAVFR
301 GRMSMKEVDE QMLNVQNKNS SYFVEWIPNN VKTAVCDIPP RGLKMAVTFI
351 GNSTAIQELF KRISEQFTAM FRRKAFLHWY TGEGMDEMEF TEAESNMNDL
401 VSEYQQYQDA TAEEEEDFGE EAEEEA
```

Residue Number Increasing Mass Decreasing Mass

Start - End	Observed	Mr(expt)	Mr(calc)	Delta	Miss	Sequence
29 - 40	1301.6257	1300.6184	1300.6299	-0.0114	0	ISVYYNEATGGK (No match)
45 - 59	1631.8202	1630.8129	1630.8236	-0.0106	0	AILVDLEPGTMDSVR Oxidation (M) (No match)
86 - 103	1958.9620	1957.9547	1957.9744	-0.0197	0	GHYTEGAELVDSVLDVVR (No match)
86 - 104	2087.0654	2086.0581	2086.0694	-0.0113	1	GHYTEGAELVDSVLDVVRK (No match)

137 - 144	1077.5264	1076.5191	1076.5250	-0.0059	1	**IREEYPDR** (No match)
224 - 233	1130.5920	1129.5847	1129.5880	-0.0032	0	**FPGQLNADLR** (Ions score 6)
224 - 233	1130.5920	1129.5847	1129.5880	-0.0032	0	**FPGQLNADLR** (No match)
224 - 234	1258.6864	1257.6791	1257.6829	-0.0038	1	**FPGQLNADLRK** (No match)
224 - 234	1258.6864	1257.6791	1257.6829	-0.0038	1	**FPGQLNADLRK** (No match)
235 - 244	1159.6161	1158.6088	1158.6219	-0.0131	0	**LAVNMVPFPR** Oxidation (M) (Ions score 1)
235 - 244	1159.6161	1158.6088	1158.6219	-0.0131	0	**LAVNMVPFPR** Oxidation (M) (No match)
245 - 258	1636.8209	1635.8136	1635.8231	-0.0095	0	**LHFFMPGFAPLTSR** Oxidation (M) (No match)
245 - 258	1636.8209	1635.8136	1635.8231	-0.0095	0	**LHFFMPGFAPLTSR** Oxidation (M) (Ions score 6)
265 - 279	1659.8756	1658.8683	1658.8879	-0.0196	0	**ALTVPELTQQVFDAK** (No match)
292 - 300	1039.5913	1038.5840	1038.5862	-0.0021	0	**YLTVAAVFR** (No match)
292 - 300	1039.5913	1038.5840	1038.5862	-0.0021	0	**YLTVAAVFR** (Ions score 4)
345 - 362	2042.0646	2041.0573	2041.0666	-0.0092	1	**MAVTFIGNSTAIQELFKR** Oxidation (M) (No match)
363 - 372	1245.5850	1244.5777	1244.5859	-0.0082	0	**ISEQFTAMFR** Oxidation (M) (No match)

Spot 434

Protein View

Match to: **gi|7705981** Score: **221** Expect: **1.5e-017**
NESH protein [Homo sapiens]

Nominal mass (M_r): **39293**; Calculated pI value: **4.99**
NCBI BLAST search of gi|7705981 against nr
Unformatted sequence string for pasting into other applications

Taxonomy: Homo sapiens
Links to retrieve other entries containing this sequence from NCBI Entrez:
gi|50400674 from Homo sapiens
gi|6939771 from Homo sapiens
gi|14043609 from Homo sapiens
gi|119615093 from Homo sapiens
gi|123992828 from synthetic construct
gi|123999638 from synthetic construct

Fixed modifications: Carbamidomethyl (C)
Variable modifications: Oxidation (M)
Cleavage by Trypsin: cuts C-term side of KR unless next residue is P
Sequence Coverage: **19%**

Matched peptides shown in **Bold Red**

1 MAELQQLQEF EIPTGREALR GNHSALLRVA DYCEDNYVQA TDKRKALEET
51 MAFTTQALAS VAYQVGNLAG HTLR**MLDLQG AALR**QVEARV STLGQMVNMH
101 MEKVARREIG TLATVQRLPP GQK**VIAPENL PPLTPYCRRP LNFGCLDDIG**
151 **HGIK**DLSTQL SRTGTLSRKS IK**APATPASA TLGRPPRIPE PVHLPVVPDG**
201 **R**LSAASSASS LASAGSAEGV GGAPTPKGQA APPAPPLPSS LDPPPPPAAV
251 EVFQRPPTLE ELSPPPPDEE LPLPLDLPPP PPLDGDELGL PPPPPGFGPD
301 EPSWVPASYL EKVVTLYPYT SQKDNELSFS EGTVICVTRR YSDGWCEGVS
351 SEGTGFFPGN YVEPSC

Residue Number Increasing Mass Decreasing Mass

Start - End	Observed	Mr(expt)	Mr(calc)	Delta	Miss	Sequence
75 - 84	1103.5743	1102.5670	1102.5804	-0.0134	0	**MLDLQGAALR** Oxidation (M) (No match)
124 - 138	1739.8972	1738.8899	1738.9076	-0.0177	0	**VIAPENLPPLTPYCR** (No match)

124 - 138	1739.8972	1738.8899	1738.9076	-0.0177	0	VIAPENLPPLTPYCR	(Ions score 44)
139 - 154	1811.9047	1810.8974	1810.9148	-0.0173	0	RPLNFGCLDDIGHGIK	(Ions score 0)
139 - 154	1811.9047	1810.8974	1810.9148	-0.0173	0	RPLNFGCLDDIGHGIK	(No match)
173 - 187	1462.7974	1461.7901	1461.8052	-0.0150	0	APATPASATLGRPPR	(Ions score 31)
173 - 187	1462.7974	1461.7901	1461.8052	-0.0150	0	APATPASATLGRPPR	(No match)
188 - 201	1524.8372	1523.8299	1523.8459	-0.0160	0	IPEPVHLPVVPDGR	(Ions score 77)
188 - 201	1524.8372	1523.8299	1523.8459	-0.0160	0	IPEPVHLPVVPDGR	(No match)

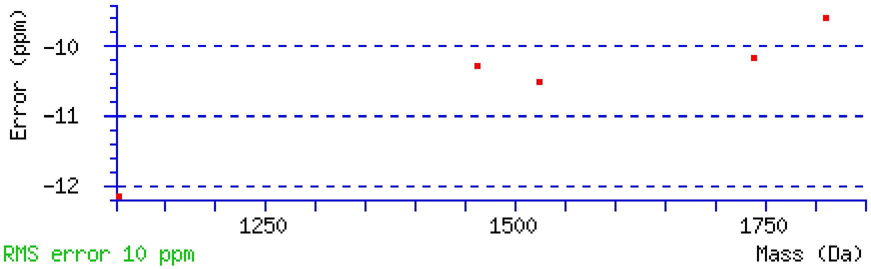

RMS error 10 ppm

Spot 435

Protein View

Match to: **gi|119586960** Score: **109** Expect: **2.4e-006**
CNDP dipeptidase 2 (metallopeptidase M20 family), isoform CRA_e [Homo sapiens]

Nominal mass (M_r): **19432**; Calculated pI value: **6.07**
NCBI BLAST search of gi|119586960 against nr
Unformatted sequence string for pasting into other applications

Taxonomy: Homo sapiens

Fixed modifications: Carbamidomethyl (C)
Variable modifications: Oxidation (M)
Cleavage by Trypsin: cuts C-term side of KR unless next residue is P
Sequence Coverage: **10%**

Matched peptides shown in **Bold Red**

```
  1 MAALTTLFKY IDENQDRYIK KLAKWVAIQS VSAWPEKRGE IRRMMEVAAA
 51 DVKQLGGSVE LVDIGKQKLP DGSEIPLPPI LLGRLGSDPQ KKTVCIYGHL
101 DVQPAALEDG WDSEPFTLVE RDERHPHAPM AVPVSVPPWH RRRLLWVWGQ
151 DRDSQEGGWQ VLHQARAEHD S
```

Residue Number Increasing Mass Decreasing Mass

Start - End	Observed	Mr(expt)	Mr(calc)	Delta	Miss	Sequence
67 - 84	1943.0636	1942.0563	1942.1251	-0.0688	1	QKLPDGSEIPLPPILLGR (No match)
67 - 84	1943.0636	1942.0563	1942.1251	-0.0688	1	QKLPDGSEIPLPPILLGR (Ions score 49)
69 - 84	1686.9177	1685.9104	1685.9715	-0.0611	0	LPDGSEIPLPPILLGR (No match)
69 - 84	1686.9177	1685.9104	1685.9715	-0.0611	0	LPDGSEIPLPPILLGR (Ions score 34)

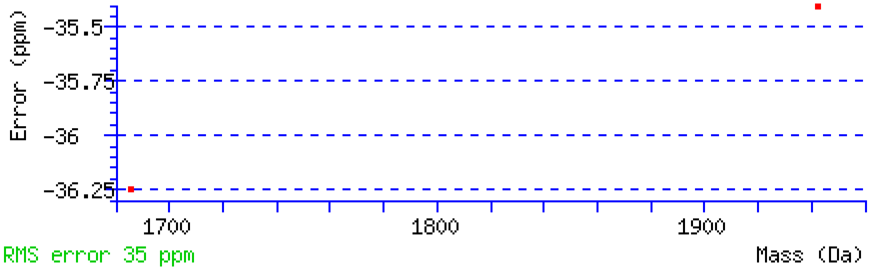

RMS error 35 ppm

Spot 437

Protein View

Match to: **gi|20151189** Score: **276** Expect: **4.8e-023**
Chain A, Structure Of Human Glutamate Dehydrogenase-Apo Form

Nominal mass (M_r): **56315**; Calculated pI value: **6.71**
NCBI BLAST search of gi|20151189 against nr
Unformatted sequence string for pasting into other applications

Taxonomy: Homo sapiens
Links to retrieve other entries containing this sequence from NCBI Entrez:
gi|20151190 from Homo sapiens
gi|20151191 from Homo sapiens
gi|20151192 from Homo sapiens
gi|20151193 from Homo sapiens
gi|20151194 from Homo sapiens

Fixed modifications: Carbamidomethyl (C)
Variable modifications: Oxidation (M)
Cleavage by Trypsin: cuts C-term side of KR unless next residue is P
Sequence Coverage: **29%**

Matched peptides shown in **Bold Red**

```
  1 SEAVADREDD PNFFKMVEGF FDRGASIVED KLVEDLRTRE SEEQKRNRVR
 51 GILRIIKPCN HVLSLSFPIR RDDGSWEVIE GYRAQHSQHR TPCKGGIRYS
101 TDVSVDEVKA LASLMTYKCA VVDVPFGGAK AGVKINPKNY TDNELEKITR
151 RFTMELAKKG FIGPGIDVPA PDMSTGEREM SWIADTYAST IGHYDINAHA
201 CVTGKPISQG GIHGRISATG RGVFHGIENF INEASYMSIL GMTPGFGDKT
251 FVVQGFGNVG LHSMRYLHRF GAKCIAVGES DGSIWNPDGI DPKELEDFKL
301 QHGSILGFPK AKPYEGSILE ADCDILIPAA SEKQLTKSNA PRVKAKIIAE
351 GANGPTTPEA DKIFLERNIM VIPDLYLNAG GVTVSYFEWL KNLNHVSYGR
401 LTFKYERDSN YHLLMSVQES LERKFGKHGG TIPIVPTAEF QDRISGASEK
451 DIVHSGLAYT MERSARQIMR TAMKYNLGLD LRTAAYVNAI EKVFKVYNEA
501 GVTFT
```

Residue Number Increasing Mass Decreasing Mass

Start - End	Observed	Mr(expt)	Mr(calc)	Delta	Miss	Sequence

1 - 15	1739.8896	1738.8823	1738.7798	0.1026	1	SEAVADREDDPNFFK (No match)
16 - 23	1016.4688	1015.4615	1015.4433	0.0183	0	MVEGFFDR Oxidation (M) (No match)
55 - 70	1894.1260	1893.1187	1893.0658	0.0529	0	IIKPCNHVLSLSFPIR (No match)
55 - 70	1894.1260	1893.1187	1893.0658	0.0529	0	IIKPCNHVLSLSFPIR (Ions score 6)
160 - 178	1931.9716	1930.9643	1930.9094	0.0549	0	GFIGPGIDVPAPDMSTGER Oxidation (M) (Ions score 29)
160 - 178	1931.9716	1930.9643	1930.9094	0.0549	0	GFIGPGIDVPAPDMSTGER Oxidation (M) (No match)
250 - 265	1764.9344	1763.9271	1763.8776	0.0495	0	TFVVQGFGNVGLHSMR Oxidation (M) (No match)
347 - 367	2242.2351	2241.2278	2241.1640	0.0638	1	IIAEGANGPTTPEADKIFLER (Ions score 99)
347 - 367	2242.2351	2241.2278	2241.1640	0.0638	1	IIAEGANGPTTPEADKIFLER (No match)
392 - 400	1059.5574	1058.5501	1058.5257	0.0244	0	NLNHVSYGR (No match)
408 - 423	1936.9594	1935.9521	1935.8996	0.0525	0	DSNYHLLMSVQESLER Oxidation (M) (No match)
428 - 443	1737.9362	1736.9289	1736.8845	0.0444	0	HGGTIPIVPTAEFQDR (Ions score 26)
428 - 443	1737.9362	1736.9289	1736.8845	0.0444	0	HGGTIPIVPTAEFQDR (No match)
451 - 463	1507.7568	1506.7495	1506.7136	0.0359	0	DIVHSGLAYTMER Oxidation (M) (No match)

Spot 439

Protein View

Match to: **gi|3211984** Score: **155** Expect: **6.1e-011**
adenylosuccinate lyase [Homo sapiens]

Nominal mass (M_r): **48981**; Calculated pI value: **7.93**
NCBI BLAST search of gi|3211984 against nr
Unformatted sequence string for pasting into other applications

Taxonomy: Homo sapiens
Links to retrieve other entries containing this sequence from NCBI Entrez:
gi|47678267 from Homo sapiens
gi|109451010 from synthetic construct
gi|109451588 from synthetic construct
gi|119580779 from Homo sapiens

Fixed modifications: Carbamidomethyl (C)
Variable modifications: Oxidation (M)
Cleavage by Trypsin: cuts C-term side of KR unless next residue is P
Sequence Coverage: **21%**

Matched peptides shown in **Bold Red**

```
  1 MAAGGDHGSP DSYRSPLASR YASPEMCFVF SDRYKFRTWR QLWLWLAEAE
 51 QTLGLPITDE QIQEMKSNLE NIDFKMAAEE EKRLRHDVMA HVHTFGHCCP
101 KAAGIIHLGA TSCYVGDNTD LIILRNALDL LLPKLARVIS RLADFAKERA
151 SLPTLGFTHF QPAQLTTVGK RCCLWIQDLC MDLQNLKRVR DDLRFRGVKG
201 TTGTQASFLQ LFEGDDHKVE QLDKMVTEKA GFKRAFIITG QTYTRKVDIE
251 VLSVLASLGA SVHKICTDIR LLANLKEMEE PFEKQQIGSS AMPYKRNPMR
301 SERCCSLARH LMTLVMDPLQ TASVQWFERT LDDSANRRIC LAEAFLTADT
351 ILNTLQNISE GLVVYPKVIE RRIRQELPFM ATENIIMAMV KAGGSRQVQR
401 FLEEEVYPLL KPYESVMKVK AELCL
```

Residue Number Increasing Mass Decreasing Mass

Start - End	Observed	Mr(expt)	Mr(calc)	Delta	Miss	Sequence
21 - 33	1624.6543	1623.6470	1623.6697	-0.0227	0	YASPEMCFVFSDR Oxidation (M) (No match)
21 - 35	1915.8027	1914.7954	1914.8280	-0.0326	1	YASPEMCFVFSDRYK Oxidation (M) (No match)
102 - 125	2543.2842	2542.2769	2542.3213	-0.0444	0	AAGIIHLGATSCYVGDNTDLIILR (No match)

150 - 170	2214.1646	2213.1573	2213.1844	-0.0271	0	**ASLPTLGFTHFQPAQLTTVGK**	(Ions score 56)
150 - 171	2370.2493	2369.2420	2369.2855	-0.0435	1	**ASLPTLGFTHFQPAQLTTVGKR**	(No match)
235 - 245	1270.6594	1269.6521	1269.6717	-0.0196	0	**AFIITGQTYTR**	(Ions score 23)
235 - 245	1270.6594	1269.6521	1269.6717	-0.0196	0	**AFIITGQTYTR**	(No match)
401 - 418	2214.1646	2213.1573	2213.1329	0.0244	0	**FLEEEVYPLLKPYESVMK**	(No match)

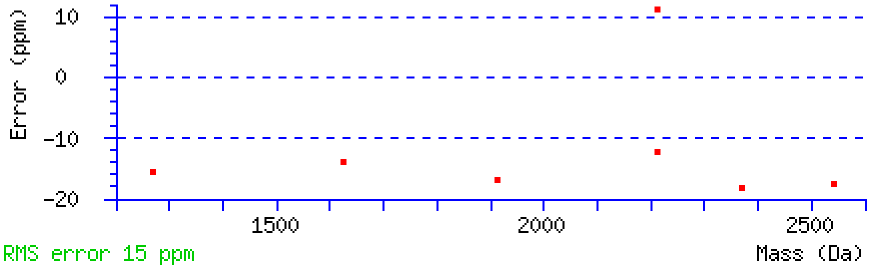

RMS error 15 ppm

Spot 441

Protein View

Match to: **gi|4757766** Score: **416** Expect: **4.8e-037**
Rho GTPase activating protein 1 [Homo sapiens]

Nominal mass (M_r): **50461**; Calculated pI value: **5.85**
NCBI BLAST search of gi|4757766 against nr
Unformatted sequence string for pasting into other applications

Taxonomy: Homo sapiens
Links to retrieve other entries containing this sequence from NCBI Entrez:
gi|3024550 from Homo sapiens
gi|312212 from Homo sapiens
gi|17390260 from Homo sapiens
gi|119588389 from Homo sapiens
gi|119588390 from Homo sapiens
gi|123994939 from synthetic construct

Fixed modifications: Carbamidomethyl (C)
Variable modifications: Oxidation (M)
Cleavage by Trypsin: cuts C-term side of KR unless next residue is P
Sequence Coverage: **39%**

Matched peptides shown in **Bold Red**

```
  1 MDPLSELQDD LTLDDTSEAL NQLKLASIDE KNWPSDEMPD FPKSDDSKSS
 51 SPELVTHLKW DDPYYDIARH QIVEVAGDDK YGRKIIVFSA CRMPPSHQLD
101 HSKLLGYLKH TLDQYVESDY TLLYLHHGLT SDNKPSLSWL RDAYREFDRK
151 YKKNIKALYI VHPTMFIKTL LILFKPLISF KFGQKIFYVN YLSELSEHVK
201 LEQLGIPRQV LKYDDFLKST QKSPATAPKP MPPRPPLPNQ QFGVSLQHLQ
251 EKNPEQEPIP IVLRETVAYL QAHALTTEGI FRRSANTQVV REVQQKYNMG
301 LPVDFDQYNE LHLPAVILKT FLRELPEPLL TFDLYPHVVG FLNIDESQRV
351 PATLQVLQTL PEENYQVLRF LTAFLVQISA HSDQNKMTNT NLAVVFGPNL
401 LWAKDAAITL KAINPINTFT KFLLDHQGEL FPSPDPSGL
```

Residue Number Increasing Mass Decreasing Mass

Start - End	Observed	Mr(expt)	Mr(calc)	Delta	Miss	Sequence
60 - 69	1313.5537	1312.5464	1312.5724	-0.0260	0	WDDPYYDIAR (Ions score 45)

60 - 69	1313.5537	1312.5464	1312.5724	-0.0260	0	**WDDPYYDIAR** (No match)
70 - 83	1586.7609	1585.7536	1585.7848	-0.0312	1	**HQIVEVAGDDKYGR** (No match)
84 - 92	1093.6039	1092.5966	1092.6113	-0.0147	1	**KIIVFSACR** (No match)
85 - 92	965.5060	964.4987	964.5164	-0.0176	0	**IIVFSACR** (No match)
157 - 168	1432.7704	1431.7631	1431.7948	-0.0317	0	**ALYIVHPTMFIK** (No match)
157 - 168	1448.7571	1447.7498	1447.7897	-0.0399	0	**ALYIVHPTMFIK** Oxidation (M) (No match)
169 - 181	1532.9438	1531.9365	1531.9741	-0.0376	0	**TLLILFKPLISFK** (No match)
186 - 200	1840.9124	1839.9051	1839.9406	-0.0355	0	**IFYVNYLSELSEHVK** (No match)
201 - 208	925.5319	924.5246	924.5392	-0.0146	0	**LEQLGIPR** (No match)
253 - 264	1404.7576	1403.7503	1403.7772	-0.0269	0	**NPEQEPIPIVLR** (Ions score 57)
253 - 264	1404.7576	1403.7503	1403.7772	-0.0269	0	**NPEQEPIPIVLR** (No match)
265 - 282	2020.0050	2018.9977	2019.0425	-0.0447	0	**ETVAYLQAHALTTEGIFR** (No match)
265 - 282	2020.0050	2018.9977	2019.0425	-0.0447	0	**ETVAYLQAHALTTEGIFR** (Ions score 37)
265 - 283	2176.1021	2175.0948	2175.1436	-0.0487	1	**ETVAYLQAHALTTEGIFRR** (No match)
324 - 349	3041.4707	3040.4634	3040.5545	-0.0911	0	**ELPEPLLTFDLYPHVVGFLNIDESQR** (Ions score 21)
324 - 349	3041.4707	3040.4634	3040.5545	-0.0911	0	**ELPEPLLTFDLYPHVVGFLNIDESQR** (No match)
350 - 369	2311.2124	2310.2051	2310.2583	-0.0531	0	**VPATLQVLQTLPEENYQVLR** (Ions score 60)
350 - 369	2311.2124	2310.2051	2310.2583	-0.0531	0	**VPATLQVLQTLPEENYQVLR** (No match)
370 - 386	1918.9623	1917.9550	1917.9948	-0.0398	0	**FLTAFLVQISAHSDQNK** (No match)

Spot 442

Protein View

Match to: **gi|48255968** Score: **321** Expect: **1.5e-027**
UDP-glucose pyrophosphorylase 2 isoform b [Homo sapiens]

Nominal mass (M_r): **55813**; Calculated pI value: **7.69**
NCBI BLAST search of gi|48255968 against nr
Unformatted sequence string for pasting into other applications

Taxonomy: Homo sapiens
Links to retrieve other entries containing this sequence from NCBI Entrez:
gi|114577657 from Pan troglodytes
gi|114577659 from Pan troglodytes
gi|114577661 from Pan troglodytes
gi|114577663 from Pan troglodytes
gi|114577665 from Pan troglodytes
gi|114577667 from Pan troglodytes
gi|12804193 from Homo sapiens
gi|55730161 from Pongo pygmaeus

Fixed modifications: Carbamidomethyl (C)
Variable modifications: Oxidation (M)
Cleavage by Trypsin: cuts C-term side of KR unless next residue is P
Sequence Coverage: **28%**

Matched peptides shown in **Bold Red**

```
  1 MSQDGASQFQ EVIRQELELS VKKELEKILT TASSHEFEHT KKDLDGFRKL
 51 FHRFLQEKGP SVDWGKIQRP PEDSIQPYEK IKARGLPDNI SSVLNKLVVV
101 KLNGGLGTSM GCKGPKSLIG VRNENTFLDL TVQQIEHLNK TYNTDVPLVL
151 MNSFNTDEDT KKILQKYNHC RVKIYTFNQS RYPRINKESL LPVAKDVSYS
201 GENTEAWYPP GHGDIYASFY NSGLLDTFIG EGKEYIFVSN IDNLGATVDL
251 YILNHLMNPP NGKRCEFVME VTNKTRADVK GGTLTQYEGK LRLVEIAQVP
301 KAHVDEFKSV SKFKIFNTNN LWISLAAVKR LQEQNAIDME IIVNAKTLDG
351 GLNVIQLETA VGAAIKSFEN SLGINVPRSR FLPVKTTSDL LLVMSNLYSL
401 NAGSLTMSEK REFPTVPLVK LGSSFTKVQD YLRRFESIPD MLELDHLTVS
451 GDVTFGKNVS LKGTVIIIAN HGDRIDIPPG AVLENKIVSG NLRILDH
```

Residue Number Increasing Mass Decreasing Mass

Start - End	Observed	Mr(expt)	Mr(calc)	Delta	Miss	Sequence
43 - 49	850.4730	849.4657	849.4344	0.0313	1	DLDGFRK (No match)
67 - 80	1699.8384	1698.8311	1698.8577	-0.0265	0	IQRPPEDSIQPYEK (No match)
67 - 80	1699.8384	1698.8311	1698.8577	-0.0265	0	IQRPPEDSIQPYEK (Ions score 33)
123 - 140	2156.0613	2155.0540	2155.0909	-0.0369	0	NENTFLDLTVQQIEHLNK (No match)
141 - 161	2433.1733	2432.1660	2432.1053	0.0607	0	TYNTDVPLVLMNSFNTDEDTK Oxidation (M) (No match)
141 - 162	2561.2527	2560.2454	2560.2002	0.0452	1	TYNTDVPLVLMNSFNTDEDTKK Oxidation (M) (No match)
172 - 181	1255.6622	1254.6549	1254.6720	-0.0171	1	VKIYTFNQSR (No match)
174 - 181	1028.4998	1027.4925	1027.5087	-0.0161	0	IYTFNQSR (No match)
174 - 181	1028.4998	1027.4925	1027.5087	-0.0161	0	IYTFNQSR (Ions score 19)
331 - 346	1828.9144	1827.9071	1827.9400	-0.0329	0	LQEQNAIDMEIIVNAK (No match)
347 - 366	1983.0759	1982.0686	1982.1047	-0.0361	0	TLDGGLNVIQLETAVGAAIK (Ions score 60)
347 - 366	1983.0759	1982.0686	1982.1047	-0.0361	0	TLDGGLNVIQLETAVGAAIK (No match)
367 - 378	1332.6696	1331.6623	1331.6833	-0.0210	0	SFENSLGINVPR (No match)
367 - 378	1332.6696	1331.6623	1331.6833	-0.0210	0	SFENSLGINVPR (Ions score 47)
463 - 474	1265.7020	1264.6947	1264.6887	0.0060	0	GTVIIIANHGDR (No match)
463 - 486	2512.3452	2511.3379	2511.3808	-0.0429	1	GTVIIIANHGDRIDIPPGAVLENK (No match)

Spot 443

Protein View

Match to: **gi|89574029** Score: **526** Expect: **4.8e-048**
mitochondrial ATP synthase, H+ transporting F1 complex beta subunit [Homo sapiens]

Nominal mass (M_r): **48083**; Calculated pI value: **4.95**
NCBI BLAST search of gi|89574029 against nr
Unformatted sequence string for pasting into other applications

Taxonomy: Homo sapiens

Fixed modifications: Carbamidomethyl (C)
Variable modifications: Oxidation (M)
Cleavage by Trypsin: cuts C-term side of KR unless next residue is P
Sequence Coverage: **55%**

Matched peptides shown in **Bold Red**

```
  1 AVIGAVVDVQ FDEGLPPILN ALEVQGRETR LVLEVAQHLG ESTVRTIAMD
 51 GTEGLVRGQK VLDSGAPIKI PVGPETLGRI MNVIGEPIDE RGPIKTKQFA
101 PIHAEAPEFM EMSVEQEILV TGIKVVDLLA PYAKGGKIGL FGGAGVGKTV
151 LIMELINNVA KAHGGYSVFA GVGERTREGN DLYHEMIESG VINLKDATSK
201 VALVYGQMNE PPGARARVAL TGLTVAEYFR DQEGQDVLLF IDNIFRFTQA
251 GSEVSALLGR IPSAVGYQPT LATDMGTMQE RITTTKKGSI TSVQAIYVPA
301 DDLTDPAPAT TFAHLDATTV LSRAIAELGI YPAVDPLDST SRIMDPNIVG
351 SEHYDVARGV QKILQDYKSL QDIIAILGMD ELSEEDKLTV SRARKIQRFL
401 SQPFQVAEVF TGHMGKLVPL KETIKGFQQI LAGEYDHLPE QAFYM
```

Residue Number Increasing Mass Decreasing Mass

Start - End	Observed	Mr(expt)	Mr(calc)	Delta	Miss	Sequence
31 - 45	1650.8579	1649.8506	1649.9100	-0.0594	0	LVLEVAQHLGESTVR (No match)
31 - 45	1650.8579	1649.8506	1649.9100	-0.0594	0	LVLEVAQHLGESTVR (Ions score 74)
46 - 57	1278.5929	1277.5856	1277.6285	-0.0429	0	TIAMDGTEGLVR Oxidation (M) (No match)
61 - 79	1919.0212	1918.0139	1918.0887	-0.0747	1	VLDSGAPIKIPVGPETLGR (Ions score 90)
61 - 79	1919.0212	1918.0139	1918.0887	-0.0747	1	VLDSGAPIKIPVGPETLGR (No match)
80 - 91	1401.6521	1400.6448	1400.6969	-0.0521	0	IMNVIGEPIDER Oxidation (M) (No match)
80 - 95	1796.8926	1795.8853	1795.9501	-0.0648	1	IMNVIGEPIDERGPIK Oxidation (M) (No match)
125 - 134	1088.5919	1087.5846	1087.6277	-0.0431	0	VVDLLAPYAK (No match)

138 - 148	975.5240	974.5167	974.5548	-0.0381	0	IGLFGGAGVGK (No match)
149 - 161	1473.7772	1472.7699	1472.8272	-0.0573	0	TVLIMELINNVAK Oxidation (M) (No match)
162 - 175	1406.6337	1405.6264	1405.6738	-0.0473	0	AHGGYSVFAGVGER (No match)
162 - 175	1406.6337	1405.6264	1405.6738	-0.0473	0	AHGGYSVFAGVGER (Ions score 100)
176 - 195	2334.0520	2333.0447	2333.1321	-0.0873	1	TREGNDLYHEMIESGVINLK Oxidation (M) (No match)
201 - 215	1617.7495	1616.7422	1616.7980	-0.0558	0	VALVYGQMNEPPGAR Oxidation (M) (Ions score 28)
201 - 215	1617.7495	1616.7422	1616.7980	-0.0558	0	VALVYGQMNEPPGAR Oxidation (M) (No match)
218 - 230	1439.7335	1438.7262	1438.7819	-0.0557	0	VALTGLTVAEYFR (No match)
247 - 260	1435.7045	1434.6972	1434.7466	-0.0494	0	FTQAGSEVSALLGR (Ions score 36)
247 - 260	1435.7045	1434.6972	1434.7466	-0.0494	0	FTQAGSEVSALLGR (No match)
261 - 281	2297.9941	2296.9868	2297.0667	-0.0799	0	IPSAVGYQPTLATDMGTMQER 2 Oxidation (M) (No match)
324 - 342	1987.9561	1986.9488	1987.0262	-0.0773	0	AIAELGIYPAVDPLDSTSR (No match)
343 - 358	1831.7982	1830.7909	1830.8570	-0.0661	0	IMDPNIVGSEHYDVAR Oxidation (M) (No match)
399 - 416	2038.9281	2037.9208	2037.9981	-0.0773	0	FLSQPFQVAEVFTGHMGK Oxidation (M) (No match)

Spot 444

Protein View

Match to: **gi|33150556** Score: **158** Expect: **3e-011**
B6 [Homo sapiens]

Nominal mass (M_r): **44905**; Calculated pI value: **8.91**
NCBI BLAST search of gi|33150556 against nr
Unformatted sequence string for pasting into other applications

Taxonomy: Homo sapiens

Fixed modifications: Carbamidomethyl (C)
Variable modifications: Oxidation (M)
Cleavage by Trypsin: cuts C-term side of KR unless next residue is P
Sequence Coverage: **13%**

Matched peptides shown in **Bold Red**

```
  1 MATSEQSICQ ARASVMVYDD TSKKWVPIKP GQQGFSRINI YHNTASNTFR
 51 VVGVKLQDQQ VVINYSIVKG LKYNQATPTF HQWRDARQVY GLNFASKEEA
101 TTFSNAMLFA LNIMNSQEGG PSSQRQVQNG PSPDEMDIQR RQVMEQHQQQ
151 RQESLERRTS ATGPILPPGH PSSAASAPVS CSGPPPPPPP PVPPPPPTGAT
201 PPPPPPLPAG GAQGSSHDES SMSGLAAAIA GAKLRRVQRP EDASGGSSPS
251 GTSKSDANRA SSGGGGGGLM EEMNKLLAKR RKAASQSDKP AEKKEDESQM
301 EDPSTSPSPG TRAASQPPNS SEAGRKPWER NNSVEKPVSS ILSRTPSVAK
351 SPEAKSPLQS QPHSRMKPAG SVNDMALDAF DLDRMKQEIL EEVVRELHKV
401 KEEIIDAIRQ ELSGISTT
```

Residue Number Increasing Mass Decreasing Mass

Start - End	Observed	Mr(expt)	Mr(calc)	Delta	Miss	Sequence	
38 - 50	1550.7063	1549.6990	1549.7637	-0.0647	0	INIYHNTASNTFR	(Ions score 42)
38 - 50	1550.7063	1549.6990	1549.7637	-0.0647	0	INIYHNTASNTFR	(No match)
56 - 69	1646.8356	1645.8283	1645.9039	-0.0755	0	LQDQQVVINYSIVK	(No match)
56 - 69	1646.8356	1645.8283	1645.9039	-0.0755	0	LQDQQVVINYSIVK	(Ions score 55)
356 - 365	1136.5325	1135.5252	1135.5734	-0.0482	0	SPLQSQPHSR	(No match)
356 - 365	1136.5325	1135.5252	1135.5734	-0.0482	0	SPLQSQPHSR	(No match)
366 - 384	2097.8740	2096.8667	2096.9506	-0.0839	0	MKPAGSVNDMALDAFDLDR	2 Oxidation (M) (No match)
366 - 384	2097.8740	2096.8667	2096.9506	-0.0839	0	MKPAGSVNDMALDAFDLDR	2 Oxidation (M) (Ions score 18)

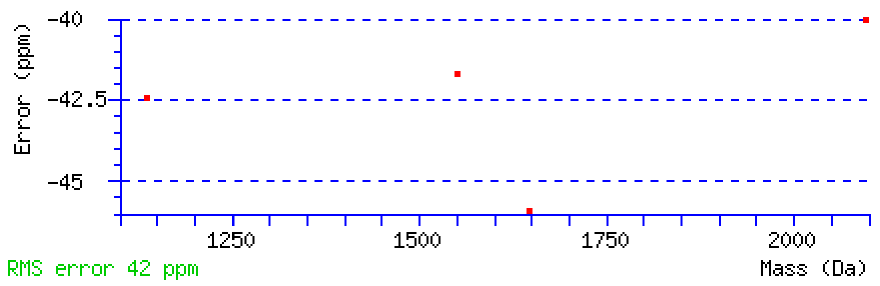
-40
-42.5
-45
Error (ppm)
1250
1500
1750
2000
RMS error 42 ppm
Mass (Da)

Spot 445

Protein View

Match to: **gi|6563206** Score: **323** Expect: **9.6e-028**
Ena-VASP-like protein [Homo sapiens]

Nominal mass (M_r): **44696**; Calculated pI value: **8.91**
NCBI BLAST search of gi|6563206 against nr
Unformatted sequence string for pasting into other applications

Taxonomy: Homo sapiens

Fixed modifications: Carbamidomethyl (C)
Variable modifications: Oxidation (M)
Cleavage by Trypsin: cuts C-term side of KR unless next residue is P
Sequence Coverage: **51%**

Matched peptides shown in **Bold Red**

```
  1 MSEQSICQAR ASVMVYDDTS KKWVPIKPGQ QGFSRINIYH NTASNTFRVV
 51 GVKLQDQQVV INYSIVKGLK YNQATPTFHQ WRDARQVYGL NFASKEEATT
101 FSNAMLFALN IMNSQEGGPS SQRQVQNGPS PDEMDIQRRQ VMEQHQQQRQ
151 ESLERRTSAT GPILPPGHPS SAASAPVSCS GPPPPPPPPV PPPPTGATPP
201 SPPPLPAGGA QGSSHDESSM SGLAAAIAGA KLRRVQRPED ASGGSSPSGT
251 SKSDANRASS GGGGGGLMEE MNKLLAKRRK AASQSDKPAE KKEDESQMED
301 PSTSPSPGTR AASQPPNSSE AGRKPWERSN SVEKPVSSIL SRTPSVAKSP
351 EAKSPLQSQP HSRMKPAGSV NDMALDAFDL DRMKQEILEE VVRELHKVKE
401 EIIDAIRQEL SGISTT
```

Residue Number Increasing Mass Decreasing Mass

Start - End	Observed	Mr(expt)	Mr(calc)	Delta	Miss	Sequence
23 - 35	1499.8146	1498.8073	1498.8044	0.0029	0	WVPIKPGQQGFSR (No match)
36 - 48	1550.7732	1549.7659	1549.7637	0.0022	0	INIYHNTASNTFR (Ions score 48)
36 - 48	1550.7732	1549.7659	1549.7637	0.0022	0	INIYHNTASNTFR (No match)
49 - 67	2129.2849	2128.2776	2128.2255	0.0521	1	VVGVKLQDQQVVINYSIVK (No match)
54 - 67	1646.9146	1645.9073	1645.9039	0.0035	0	LQDQQVVINYSIVK (Ions score 65)
54 - 67	1646.9146	1645.9073	1645.9039	0.0035	0	LQDQQVVINYSIVK (No match)
71 - 82	1548.7446	1547.7373	1547.7269	0.0104	0	YNQATPTFHQWR (No match)
86 - 95	1126.5896	1125.5823	1125.5818	0.0005	0	QVYGLNFASK (No match)

124 - 138	1729.7861	1728.7788	1728.7737	0.0052	0	**QVQNGPSPDEMDIQR** Oxidation (M) (No match)
124 - 139	1885.8864	1884.8791	1884.8748	0.0044	1	**QVQNGPSPDEMDIQRR** Oxidation (M) (No match)
140 - 149	1327.6177	1326.6104	1326.6098	0.0006	0	**QVMEQHQQQR** Oxidation (M) (No match)
150 - 155	761.3911	760.3838	760.3715	0.0123	0	**QESLER** (No match)
234 - 252	1902.9288	1901.9215	1901.9191	0.0025	1	**RVQRPEDASGGSSPSGTSK** (No match)
235 - 252	1746.8344	1745.8271	1745.8179	0.0092	0	**VQRPEDASGGSSPSGTSK** (No match)
292 - 310	2093.9102	2092.9029	2092.8855	0.0175	1	**KEDESQMEDPSTSPSPGTR** Oxidation (M) (No match)
311 - 323	1271.6039	1270.5966	1270.5901	0.0065	0	**AASQPPNSSEAGR** (No match)
329 - 342	1502.8243	1501.8170	1501.8100	0.0071	0	**SNSVEKPVSSILSR** (No match)
354 - 363	1136.5868	1135.5795	1135.5734	0.0061	0	**SPLQSQPHSR** (No match)
364 - 382	2097.9609	2096.9536	2096.9506	0.0030	0	**MKPAGSVNDMALDAFDLDR** 2 Oxidation (M) (No match)
383 - 393	1389.7440	1388.7367	1388.7333	0.0034	1	**MKQEILEEVVR** Oxidation (M) (No match)
398 - 407	1185.6852	1184.6779	1184.6764	0.0015	1	**VKEEIIDAIR** (No match)

Spot 446

Protein View

Match to: **gi|119602091** Score: **171** Expect: **1.5e-012**
Enah/Vasp-like, isoform CRA_b [Homo sapiens]

Nominal mass (M_r): **40952**; Calculated pI value: **9.55**
NCBI BLAST search of gi|119602091 against nr
Unformatted sequence string for pasting into other applications

Taxonomy: Homo sapiens

Fixed modifications: Carbamidomethyl (C)
Variable modifications: Oxidation (M)
Cleavage by Trypsin: cuts C-term side of KR unless next residue is P
Sequence Coverage: **12%**

Matched peptides shown in **Bold Red**

```
  1 MSEQSICQAR ASVMVYDDTS KKWVPIKPGQ QGFSRINIYH NTASNTFRVV
 51 GVKLQDQQVV INYSIVKGLK YNQATPTFHQ WRDARQVYGL NFASKEEATT
101 FSNAMLFALN IMNSQEGGPS SQRQVQNGPS PDEMDIQRRQ VMEQHQQQRQ
151 ESLERRTSAT GPILPPGHPS SAASAPVSCS GPPPPPPPPV PPPPTGATPP
201 PPPPLPAGGA QGSSHDESSM SGLAAAIAGA KLRRVQRPED ASGGSSPSGT
251 SKSDANRASS GGGGGGLMEE MNKLLAKRRK AASQSDKPAE KKEDESQMED
301 PSTSPSPGTR AASQPPNSSE AGRKPWERSN SVEKPVSSIL SRTPSVAKSP
351 EAKSPLQSQP HSRYRTTLLL TCPPGFGAPL SPVP
```

Residue Number Increasing Mass Decreasing Mass

Start - End	Observed	Mr(expt)	Mr(calc)	Delta	Miss	Sequence
36 - 48	1550.7552	1549.7479	1549.7637	-0.0158	0	INIYHNTASNTFR (No match)
36 - 48	1550.7552	1549.7479	1549.7637	-0.0158	0	INIYHNTASNTFR (Ions score 50)
54 - 67	1646.8942	1645.8869	1645.9039	-0.0169	0	LQDQQVVINYSIVK (No match)
54 - 67	1646.8942	1645.8869	1645.9039	-0.0169	0	LQDQQVVINYSIVK (Ions score 79)
140 - 149	1327.5997	1326.5924	1326.6098	-0.0174	0	QVMEQHQQQR Oxidation (M) (No match)
354 - 363	1136.5725	1135.5652	1135.5734	-0.0082	0	SPLQSQPHSR (No match)
354 - 363	1136.5725	1135.5652	1135.5734	-0.0082	0	SPLQSQPHSR (No match)

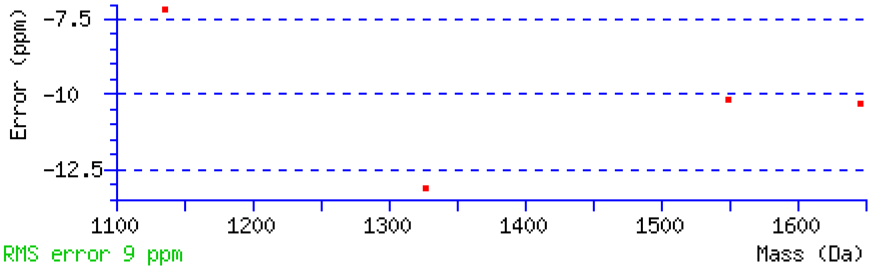

Error (ppm)
-7.5
-10
-12.5
1100
1200
1300
1400
1500
1600
RMS error 9 ppm
Mass (Da)

Spot 447

Protein View

Match to: **gi|25090276** Score: **393** Expect: **9.6e-035**
Ena/VASP-like protein (Ena/vasodilator-stimulated phosphoprotein-like)

Nominal mass (M_r): **44706**; Calculated pI value: **8.91**
NCBI BLAST search of gi|25090276 against nr
Unformatted sequence string for pasting into other applications

Taxonomy: Homo sapiens
Links to retrieve other entries containing this sequence from NCBI Entrez:
gi|18645127 from Homo sapiens
gi|119602090 from Homo sapiens
gi|119602093 from Homo sapiens
gi|158260463 (no taxonomy information for this entry)

Fixed modifications: Carbamidomethyl (C)
Variable modifications: Oxidation (M)
Cleavage by Trypsin: cuts C-term side of KR unless next residue is P
Sequence Coverage: **53%**

Matched peptides shown in **Bold Red**

```
  1 MSEQSICQAR ASVMVYDDTS KKWVPIKPGQ QGFSRINIYH NTASNTFRVV
 51 GVKLQDQQVV INYSIVKGLK YNQATPTFHQ WRDARQVYGL NFASKEEATT
101 FSNAMLFALN IMNSQEGGPS SQRQVQNGPS PDEMDIQRRQ VMEQHQQQRQ
151 ESLERRTSAT GPILPPGHPS SAASAPVSCS GPPPPPPPPV PPPPTGATPP
201 PPPPLPAGGA QGSSHDESSM SGLAAAIAGA KLRRVQRPED ASGGSSPSGT
251 SKSDANRASS GGGGGGLMEE MNKLLAKRRK AASQSDKPAE KKEDESQMED
301 PSTSPSPGTR AASQPPNSSE AGRKPWERSN SVEKPVSSIL SRTPSVAKSP
351 EAKSPLQSQP HSRMKPAGSV NDMALDAFDL DRMKQEILEE VVRELHKVKE
401 EIIDAIRQEL SGISTT
```

Residue Number Increasing Mass Decreasing Mass

Start - End	Observed	Mr(expt)	Mr(calc)	Delta	Miss	Sequence	
23 - 35	1499.8119	1498.8046	1498.8044	0.0002	0	WVPIKPGQQGFSR	(Ions score 55)
23 - 35	1499.8119	1498.8046	1498.8044	0.0002	0	WVPIKPGQQGFSR	(No match)
36 - 48	1550.7605	1549.7532	1549.7637	-0.0105	0	INIYHNTASNTFR	(No match)

54 - 67	1646.9059	1645.8986	1645.9039	-0.0052	0	LQDQQVVINYSIVK (No match)
71 - 82	1548.7345	1547.7272	1547.7269	0.0003	0	YNQATPTFHQWR (No match)
71 - 82	1548.7345	1547.7272	1547.7269	0.0003	0	YNQATPTFHQWR (Ions score 35)
86 - 95	1126.5874	1125.5801	1125.5818	-0.0017	0	QVYGLNFASK (No match)
96 - 123	3030.3713	3029.3640	3029.3858	-0.0218	0	EEATTFSNAMLFALNIMNSQEGGPSSQR (No match)
124 - 138	1713.7850	1712.7777	1712.7788	-0.0010	0	QVQNGPSPDEMDIQR (No match)
124 - 139	1869.8907	1868.8834	1868.8799	0.0036	1	QVQNGPSPDEMDIQRR (No match)
140 - 149	1311.6232	1310.6159	1310.6149	0.0010	0	QVMEQHQQQR (No match)
140 - 149	1311.6232	1310.6159	1310.6149	0.0010	0	QVMEQHQQQR (Ions score 49)
140 - 149	1327.6154	1326.6081	1326.6098	-0.0017	0	QVMEQHQQQR Oxidation (M) (No match)
234 - 252	1902.9207	1901.9134	1901.9191	-0.0056	1	RVQRPEDASGGSSPSGTSK (No match)
292 - 310	2077.8909	2076.8836	2076.8906	-0.0069	1	KEDESQMEDPSTSPSPGTR (No match)
311 - 323	1271.6044	1270.5971	1270.5901	0.0070	0	AASQPPNSSEAGR (No match)
329 - 342	1502.8167	1501.8094	1501.8100	-0.0005	0	SNSVEKPVSSILSR (No match)
354 - 363	1136.5823	1135.5750	1135.5734	0.0016	0	SPLQSQPHSR (Ions score 40)
354 - 363	1136.5823	1135.5750	1135.5734	0.0016	0	SPLQSQPHSR (No match)
364 - 382	2065.9595	2064.9522	2064.9608	-0.0086	0	MKPAGSVNDMALDAFDLDR (No match)
383 - 393	1373.7489	1372.7416	1372.7384	0.0033	1	MKQEILEEVVR (No match)

Spot 448

Protein View

Match to: **gi|12804709** Score: **550** Expect: **1.9e-050**
Lymphocyte-specific protein 1 [Homo sapiens]

Nominal mass (M_r): **37427**; Calculated pI value: **4.69**
NCBI BLAST search of gi|12804709 against nr
Unformatted sequence string for pasting into other applications

Taxonomy: Homo sapiens
Links to retrieve other entries containing this sequence from NCBI Entrez:
gi|62897025 from Homo sapiens

Fixed modifications: Carbamidomethyl (C)
Variable modifications: Oxidation (M)
Cleavage by Trypsin: cuts C-term side of KR unless next residue is P
Sequence Coverage: **59%**

Matched peptides shown in **Bold Red**

```
  1 MAEASSDPGA EEREELLGPT AQWSVEDEEE AVHEQCQHER DRQLQAQDEE
 51 GGGHVPERPK QEMLLSLKPS EAPELDEDEG FGDWSQRPEQ RQQHEGAQGT
101 LDSGEPPQCR SPEGEQEDRP GLHAYEKEDS DEVHLEELSL SKEGPGPEDT
151 VQDNLGAAGA EEEQEEHQKC QQPRTPSPLV LEGTIEQSSP PLSPTTKLID
201 RTESLNRSIE KSNSVKKSQP DLPISKIDQW LEQYTQAIET AGRTPKLARQ
251 ASIELPSMAV ASTKSRWETG EVQAQSAAKT PSCKDIVAGD MSKKSLWEQK
301 GGSKTSSTIK STPSGKRYKF VATGHGKYEK VLVEGGPAP
```

Residue Number Increasing Mass Decreasing Mass

Start - End	Observed	Mr(expt)	Mr(calc)	Delta	Miss	Sequence
14 - 40	3235.3838	3234.3765	3234.4159	-0.0394	0	EELLGPTAQWSVEDEEEAVHEQCQHER (No match)
41 - 60	2246.0862	2245.0789	2245.0835	-0.0046	1	DRQLQAQDEEGGGHVPERPK (No match)
43 - 60	1974.9556	1973.9483	1973.9554	-0.0071	0	QLQAQDEEGGGHVPERPK (No match)
43 - 60	1974.9556	1973.9483	1973.9554	-0.0071	0	QLQAQDEEGGGHVPERPK (Ions score 136)
92 - 110	2094.9192	2093.9119	2093.9184	-0.0065	0	QQHEGAQGTLDSGEPPQCR (No match)
92 - 110	2094.9192	2093.9119	2093.9184	-0.0065	0	QQHEGAQGTLDSGEPPQCR (Ions score 70)
111 - 127	1941.8879	1940.8806	1940.8864	-0.0057	0	SPEGEQEDRPGLHAYEK (No match)
111 - 127	1941.8879	1940.8806	1940.8864	-0.0057	0	SPEGEQEDRPGLHAYEK (Ions score 52)

111 - 142	3652.6604	3651.6531	3651.6811	-0.0280	1	SPEGEQEDRPGLHAYEKEDSDEVHLEELSLSK (No match)
143 - 169	2864.2593	2863.2520	2863.2379	0.0141	0	EGPGPEDTVQDNLGAAGAEEEQEEHQK (No match)
198 - 207	1216.6639	1215.6566	1215.6571	-0.0005	1	LIDRTESLNR (No match)
227 - 243	2021.9871	2020.9798	2020.9854	-0.0055	0	IDQWLEQYTQAIETAGR (Ions score 129)
227 - 243	2021.9871	2020.9798	2020.9854	-0.0055	0	IDQWLEQYTQAIETAGR (No match)
250 - 264	1532.7955	1531.7882	1531.7915	-0.0033	0	QASIELPSMAVASTK (No match)
265 - 279	1647.8115	1646.8042	1646.8012	0.0030	1	SRWETGEVQAQSAAK (No match)
267 - 279	1404.6801	1403.6728	1403.6680	0.0048	0	WETGEVQAQSAAK (No match)
294 - 300	918.5080	917.5007	917.4970	0.0037	1	KSLWEQK (No match)
318 - 327	1107.5750	1106.5677	1106.5872	-0.0195	1	YKFVATGHGK (No match)
320 - 330	1236.6417	1235.6344	1235.6298	0.0046	1	FVATGHGKYEK (No match)

Spot 449

Protein View

Match to: **gi|37674289** Score: **124** Expect: **7.7e-008**
hypothetical protein LOC221960 [Homo sapiens]

Nominal mass (M_r): **56286**; Calculated pI value: **6.07**
NCBI BLAST search of gi|37674289 against nr
Unformatted sequence string for pasting into other applications

Taxonomy: Homo sapiens
Links to retrieve other entries containing this sequence from NCBI Entrez:
gi|56699480 from Homo sapiens
gi|67460432 from Homo sapiens
gi|14603353 from Homo sapiens
gi|37574290 from Homo sapiens
gi|37674418 from Homo sapiens
gi|124376164 from Homo sapiens
gi|124376870 from Homo sapiens
gi|158258084 (no taxonomy information for this entry)

Fixed modifications: Carbamidomethyl (C)
Variable modifications: Oxidation (M)
Cleavage by Trypsin: cuts C-term side of KR unless next residue is P
Sequence Coverage: **12%**

Matched peptides shown in **Bold Red**

```
  1 MAAAAAGAGS GPWAAQEKQF PPALLSFFIY NPRFGPREGQ EENKILFYHP
 51 NEVEKNEKIR NVGLCEAIVQ FTRTFSPSKP AKSLHTQKNR QFFNEPEENF
101 WMVMVVRNPI IEKQSKDGKP VIEYQEEELL DKVYSSVLRQ CYSMYKLFNG
151 TFLKAMEDGG VKLLKERLEK FFHRYLQTLH LQSCDLLDIF GGISFFPLDK
201 MTYLKIQSFI NRMEESLNIV KYTAFLYNDQ LIWSGLEQDD MRILYKYLTT
251 SLFPRHIEPE LAGRDSPIRA EMPGNLQHYG RFLTGPLNLN DPDAKCRFPK
301 IFVNTDDTYE ELHLIVYKAM SAAVCFMIDA SVHPTLDFCR RLDSIVGPQL
351 TVLASDICEQ FNINKRMSGS EKEPQFKFIY FNHMNLAEKS TVHMRKTPSV
401 SLTSVHPDLM KILGDINSDF TRVDEDEEII VKAMSDYWVV GKKSDRRELY
451 VILNQKNANL IEVNEEVKKL CATQFNNIFF LD
```

Residue Number Increasing Mass Decreasing Mass

Start - End	Observed	Mr(expt)	Mr(calc)	Delta	Miss	Sequence
19 - 33	1809.9670	1808.9597	1808.9613	-0.0016	0	QFPPALLSFFIYNPR (Ions score 7)
19 - 33	1809.9670	1808.9597	1808.9613	-0.0016	0	QFPPALLSFFIYNPR (No match)
45 - 55	1388.6687	1387.6614	1387.7135	-0.0521	0	ILFYHPNEVEK (Ions score 23)
61 - 73	1506.7732	1505.7659	1505.7660	-0.0001	0	NVGLCEAIVQFTR (Ions score 42)
61 - 73	1506.7732	1505.7659	1505.7660	-0.0001	0	NVGLCEAIVQFTR (No match)
247 - 255	1097.6000	1096.5927	1096.5917	0.0011	0	YLTTSLFPR (Ions score 1)
247 - 255	1097.6000	1096.5927	1096.5917	0.0011	0	YLTTSLFPR (No match)
270 - 281	1388.6687	1387.6614	1387.6302	0.0312	0	AEMPGNLQHYGR Oxidation (M) (No match)

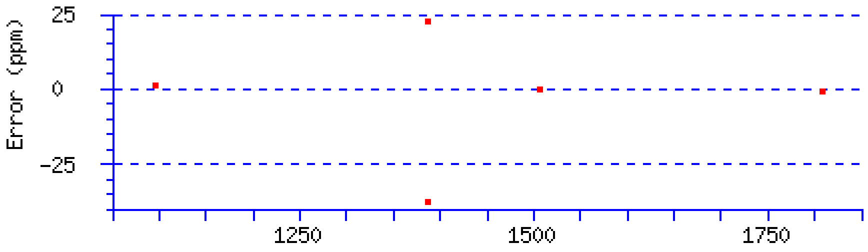

RMS error 15 ppm

Spot 450

Protein View

Match to: **gi|37674289** Score: **209** Expect: **2.4e-016**
hypothetical protein LOC221960 [Homo sapiens]

Nominal mass (M_r): **56286**; Calculated pI value: **6.07**
NCBI BLAST search of gi|37674289 against nr
Unformatted sequence string for pasting into other applications

Taxonomy: Homo sapiens
Links to retrieve other entries containing this sequence from NCBI Entrez:
gi|56699480 from Homo sapiens
gi|67460432 from Homo sapiens
gi|14603353 from Homo sapiens
gi|37574290 from Homo sapiens
gi|37674418 from Homo sapiens
gi|124376164 from Homo sapiens
gi|124376870 from Homo sapiens
gi|158258084 (no taxonomy information for this entry)

Fixed modifications: Carbamidomethyl (C)
Variable modifications: Oxidation (M)
Cleavage by Trypsin: cuts C-term side of KR unless next residue is P
Sequence Coverage: **15%**

Matched peptides shown in **Bold Red**

```
  1 MAAAAAGAGS GPWAAQEKQF PPALLSFFIY NPRFGPREGQ EENKILFYHP
 51 NEVEKNEKIR NVGLCEAIVQ FTRTFSPSKP AKSLHTQKNR QFFNEPEENF
101 WMVMVVRNPI IEKQSKDGKP VIEYQEEELL DKVYSSVLRQ CYSMYKLFNG
151 TFLKAMEDGG VKLLKERLEK FFHRYLQTLH LQSCDLLDIF GGISFFPLDK
201 MTYLKIQSFI NRMEESLNIV KYTAFLYNDQ LIWSGLEQDD MRILYKYLTT
251 SLFPRHIEPE LAGRDSPIRA EMPGNLQHYG RFLTGPLNLN DPDAKCRFPK
301 IFVNTDDTYE ELHLIVYKAM SAAVCFMIDA SVHPTLDFCR RLDSIVGPQL
351 TVLASDICEQ FNINKRMSGS EKEPQFKFIY FNHMNLAEKS TVHMRKTPSV
401 SLTSVHPDLM KILGDINSDF TRVDEDEEII VKAMSDYWVV GKKSDRRELY
451 VILNQKNANL IEVNEEVKKL CATQFNNIFF LD
```

Residue Number Increasing Mass Decreasing Mass

Start - End	Observed	Mr(expt)	Mr(calc)	Delta	Miss	Sequence
19 - 33	1809.9497	1808.9424	1808.9613	-0.0189	0	QFPPALLSFFIYNPR (No match)
19 - 33	1809.9497	1808.9424	1808.9613	-0.0189	0	QFPPALLSFFIYNPR (Ions score 33)
45 - 55	1388.6613	1387.6540	1387.7135	-0.0595	0	ILFYHPNEVEK (Ions score 36)
61 - 73	1506.7579	1505.7506	1505.7660	-0.0154	0	NVGLCEAIVQFTR (No match)
61 - 73	1506.7579	1505.7506	1505.7660	-0.0154	0	NVGLCEAIVQFTR (Ions score 40)
206 - 212	877.4878	876.4805	876.4817	-0.0012	0	IQSFINR (No match)
247 - 255	1097.5924	1096.5851	1096.5917	-0.0065	0	YLTTSLFPR (Ions score 26)
247 - 255	1097.5924	1096.5851	1096.5917	-0.0065	0	YLTTSLFPR (No match)
256 - 264	1021.5371	1020.5298	1020.5352	-0.0054	0	HIEPELAGR (No match)
270 - 281	1388.6613	1387.6540	1387.6302	0.0238	0	AEMPGNLQHYGR Oxidation (M) (No match)

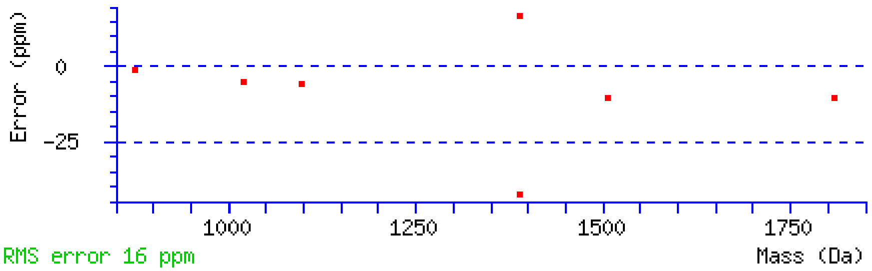

RMS error 16 ppm

Spot 451

Protein View

Match to: **gi|4759140** Score: **76** Expect: **0.0051**
solute carrier family 9 (sodium/hydrogen exchanger), isoform 3 regulator 1 [Homo sapiens]

Nominal mass (M_r): **39130**; Calculated pI value: **5.55**
NCBI BLAST search of gi|4759140 against nr
Unformatted sequence string for pasting into other applications

Taxonomy: Homo sapiens
Links to retrieve other entries containing this sequence from NCBI Entrez:
gi|41688557 from Homo sapiens
gi|2920585 from Homo sapiens
gi|3220019 from Homo sapiens
gi|12655175 from Homo sapiens
gi|13097186 from Homo sapiens
gi|15079985 from Homo sapiens
gi|31419627 from Homo sapiens
gi|119609596 from Homo sapiens

Fixed modifications: Carbamidomethyl (C)
Variable modifications: Oxidation (M)
Cleavage by Trypsin: cuts C-term side of KR unless next residue is P
Sequence Coverage: **12%**

Matched peptides shown in **Bold Red**

```
  1 MSADAAAGAP LPRLCCLEKG PNGYGFHLHG EKGKLGQYIR LVEPGSPAEK
 51 AGLLAGDRLV EVNGENVEKE THQQVVSRIR AALNAVRLLV VDPETDEQLQ
101 KLGVQVREEL LRAQEAPGQA EPPAAAEVQG AGNENEPREA DKSHPEQREL
151 RPRLCTMKKG PSGYGFNLHS DKSKPGQFIR SVDPDSPAEA SGLRAQDRIV
201 EVNGVCMEGK QHGDVVSAIR AGGDETKLLV VDRETDEFFK KCRVIPSQEH
251 LNGPLPVPFT NGEIQKENSR EALAEAALES PRPALVRSAS SDTSEELNSQ
301 DSPPKQDSTA PSSTSSSDPI LDFNISLAMA KERAHQKRSS KRAPQMDWSK
351 KNELFSNL
```

Residue Number Increasing Mass Decreasing Mass

Start - End	Observed	Mr(expt)	Mr(calc)	Delta	Miss	Sequence

113 - 138	2588.2043	2587.1970	2587.1898	0.0073	0	AQEAPGQAEPPAAAEVQGAGNENEPR	(No match)
113 - 138	2588.2043	2587.1970	2587.1898	0.0073	0	AQEAPGQAEPPAAAEVQGAGNENEPR	(Ions score 33)
271 - 287	1792.9633	1791.9560	1791.9842	-0.0282	0	EALAEAALESPRPALVR	(No match)
271 - 287	1792.9633	1791.9560	1791.9842	-0.0282	0	EALAEAALESPRPALVR	(Ions score 24)

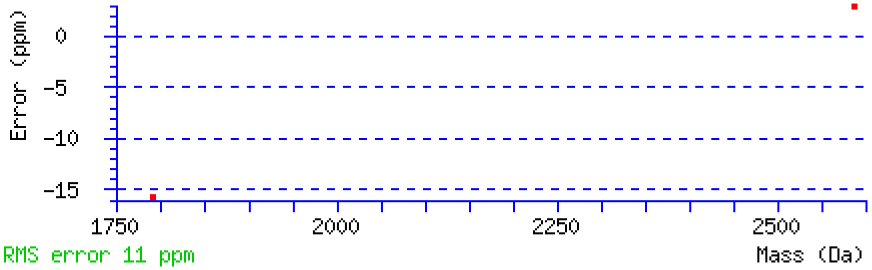

Spot 453

Protein View

Match to: **gi|6857820** Score: **221** Expect: **1.5e-017**
phosphatidylinositol-5-phosphate 4-kinase, type II, alpha [Homo sapiens]

Nominal mass (M_r): **46424;** Calculated pI value: **6.50**
NCBI BLAST search of gi|6857820 against nr
Unformatted sequence string for pasting into other applications

Taxonomy: Homo sapiens
Links to retrieve other entries containing this sequence from NCBI Entrez:
gi|73948884 from Canis lupus familiaris
gi|18266879 from Homo sapiens
gi|6760472 from Homo sapiens
gi|17390058 from Homo sapiens
gi|55664519 from Homo sapiens
gi|55665197 from Homo sapiens
gi|57160667 from Homo sapiens
gi|119606546 from Homo sapiens
gi|119606547 from Homo sapiens

Fixed modifications: Carbamidomethyl (C)
Variable modifications: Oxidation (M)
Cleavage by Trypsin: cuts C-term side of KR unless next residue is P
Sequence Coverage: **23%**

Matched peptides shown in **Bold Red**

1 MATPGNLGSS VLASKTKTKK KHFVAQKVKL FRASDPLLSV LMWGVNHSIN
51 ELSHVQIPVM LMPDDFKAYS KIKVDNHLFN KENMPSHFK**F KEYCPMVFR**N
101 LRER**FGIDDQ DFQNSLTRSA PLPNDSQAR**S GAR**FHTSYDK R**YIIKTITSE
151 DVAEMHNILK KYHQYIVECH GITLLPQFLG MYR**LNVDGVE IYVIVTRNVF**
201 **SHR**LSVYRKY DLKGSTVARE ASDKEKAKEL PTLKDNDFIN EGQKIYIDDN
251 NKKVFLEKLK KDVEFLAQLK **LMDYSLLVGI HDVER**AEQEE VECEENDGEE
301 EGESDGTHPV GTPPDSPGNT LNSSPPLAPG EFDPNIDVYG IKCHENSPRK
351 EVYFMAIIDI LTHYDAKKKA AHAAKTVK**HG AGAEISTVNP EQYSKR**FLDF
401 IGHILT

Residue Number Increasing Mass Decreasing Mass

Start - End	Observed	Mr(expt)	Mr(calc)	Delta	Miss	Sequence
90 - 99	1392.6320	1391.6247	1391.6366	-0.0118	1	FKEYCPMVFR Oxidation (M) (No match)
105 - 118	1655.7489	1654.7416	1654.7587	-0.0170	0	FGIDDQDFQNSLTR (No match)
105 - 118	1655.7489	1654.7416	1654.7587	-0.0170	0	FGIDDQDFQNSLTR (Ions score 87)
119 - 129	1155.5702	1154.5629	1154.5680	-0.0050	0	SAPLPNDSQAR (No match)
134 - 141	1053.5132	1052.5059	1052.5039	0.0020	1	FHTSYDKR (No match)
134 - 141	1053.5132	1052.5059	1052.5039	0.0020	1	FHTSYDKR (No match)
184 - 197	1589.8745	1588.8672	1588.8824	-0.0151	0	LNVDGVEIYVIVTR (No match)
184 - 197	1589.8745	1588.8672	1588.8824	-0.0151	0	LNVDGVEIYVIVTR (Ions score 55)
198 - 203	759.3912	758.3839	758.3823	0.0016	0	NVFSHR (No match)
271 - 285	1775.8811	1774.8738	1774.8923	-0.0185	0	LMDYSLLVGIHDVER Oxidation (M) (No match)
379 - 395	1787.8629	1786.8556	1786.8485	0.0071	0	HGAGAEISTVNPEQYSK (No match)
379 - 396	1943.9473	1942.9400	1942.9496	-0.0096	1	HGAGAEISTVNPEQYSKR (No match)

Spot 454

Protein View

Match to: **gi|6857820** Score: **184** Expect: **7.7e-014**
phosphatidylinositol-5-phosphate 4-kinase, type II, alpha [Homo sapiens]

Nominal mass (M_r): **46424**; Calculated pI value: **6.50**
NCBI BLAST search of gi|6857820 against nr
Unformatted sequence string for pasting into other applications

Taxonomy: Homo sapiens
Links to retrieve other entries containing this sequence from NCBI Entrez:
gi|73948884 from Canis lupus familiaris
gi|18266879 from Homo sapiens
gi|6760472 from Homo sapiens
gi|17390058 from Homo sapiens
gi|55664519 from Homo sapiens
gi|55665197 from Homo sapiens
gi|57160667 from Homo sapiens
gi|119606546 from Homo sapiens
gi|119606547 from Homo sapiens

Fixed modifications: Carbamidomethyl (C)
Variable modifications: Oxidation (M)
Cleavage by Trypsin: cuts C-term side of KR unless next residue is P
Sequence Coverage: **7%**

Matched peptides shown in **Bold Red**

1 MATPGNLGSS VLASKTKTKK KHFVAQKVKL FRASDPLLSV LMWGVNHSIN
51 ELSHVQIPVM LMPDDFKAYS KIKVDNHLFN KENMPSHFKF KEYCPMVFRN
101 LRER**FGIDDQ DFQNSLTR**SA PLPNDSQARS GARFHTSYDK RYIIKTITSE
151 DVAEMHNILK KYHQYIVECH GITLLPQFLG MYRLNVDGVE IYVIVTRNVF
201 SHRLSVYRKY DLKGSTVARE ASDKEKAKEL PTLKDNDFIN EGQKIYIDDN
251 NKKVFLEKLK KDVEFLAQLK **LMDYSLLVGI HDVER**AEQEE VECEENDGEE
301 EGESDGTHPV GTPPDSPGNT LNSSPPLAPG EFDPNIDVYG IKCHENSPRK
351 EVYFMAIIDI LTHYDAKKKA AHAAKTVKHG AGAEISTVNP EQYSKRFLDF
401 IGHILT

Residue Number Increasing Mass Decreasing Mass

Start - End	Observed	Mr(expt)	Mr(calc)	Delta	Miss	Sequence
105 - 118	1655.7853	1654.7780	1654.7587	0.0194	0	FGIDDQDFQNSLTR (No match)
105 - 118	1655.7853	1654.7780	1654.7587	0.0194	0	FGIDDQDFQNSLTR (Ions score 111)
271 - 285	1775.9192	1774.9119	1774.8923	0.0196	0	LMDYSLLVGIHDVER Oxidation (M) (No match)
271 - 285	1775.9192	1774.9119	1774.8923	0.0196	0	LMDYSLLVGIHDVER Oxidation (M) (Ions score 52)

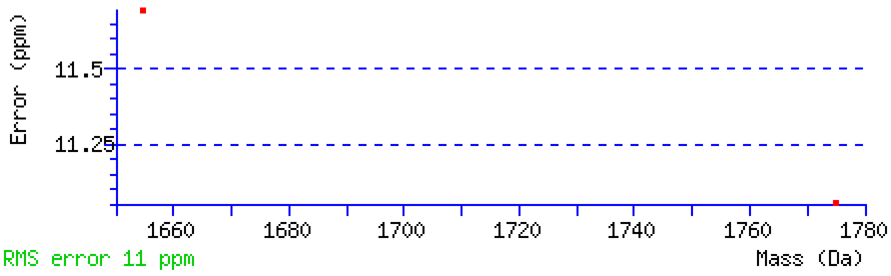

Spot 455

Protein View

Match to: **gi|41406050** Score: **262** Expect: **1.2e-021**
docking protein 2 [Homo sapiens]

Nominal mass (M_r): **45750**; Calculated pI value: **5.78**
NCBI BLAST search of gi|41406050 against nr
Unformatted sequence string for pasting into other applications

Taxonomy: Homo sapiens
Links to retrieve other entries containing this sequence from NCBI Entrez:
gi|143220234 from Homo sapiens
gi|21618483 from Homo sapiens
gi|119584143 from Homo sapiens
gi|124000597 from synthetic construct
gi|124126789 from synthetic construct

Fixed modifications: Carbamidomethyl (C)
Variable modifications: Oxidation (M)
Cleavage by Trypsin: cuts C-term side of KR unless next residue is P
Sequence Coverage: **18%**

Matched peptides shown in **Bold Red**

```
  1 MGDGAVKQGF LYLQQQQTFG KKWRRFGASL YGGSDCALAR LELQEGPEKP
 51 RRCEAARKVI RLSDCLRVAE AGGEASSPRD TSAFFLETKE RLYLLAAPAA
101 ERGDWVQAIC LLAFPGQRKE LSGPEGKQSR PCMEENELYS SAVTVGPHKE
151 FAVTMRPTEA SERCHLRGSY TLRAGESALE LWGGPEPGTQ LYDWPYRFLR
201 RFGRDKVTFS FEAGRRCVSG EGNFEFETRQ GNEIFLALEE AISAQKNAAP
251 ATPQPQPATI PASLPRPDSP YSRPHDSLPP PSPTTPVPAP RPRGQEGEYA
301 VPFDAVARSL GKNFRGILAV PPQLLADPLY DSIEETLPPR PDHIYDEPEG
351 VAALSLYDSP QEPRGEAWRR QATADRDPAG LQHVQPAGQD FSASGWQPGT
401 EYDNVVLKKG PK
```

Residue Number Increasing Mass Decreasing Mass

Start - End	Observed	Mr(expt)	Mr(calc)	Delta	Miss	Sequence	
26 - 40	1544.7014	1543.6941	1543.7088	-0.0147	0	FGASLYGGSDCALAR	(No match)
26 - 40	1544.7014	1543.6941	1543.7088	-0.0147	0	FGASLYGGSDCALAR	(Ions score 33)

41 - 51	1295.6840	1294.6767	1294.6880	-0.0113	0	LELQEGPEKPR	(No match)
41 - 51	1295.6840	1294.6767	1294.6880	-0.0113	0	LELQEGPEKPR	(Ions score 17)
92 - 102	1187.6653	1186.6580	1186.6709	-0.0129	0	LYLLAAPAAER	(Ions score 25)
92 - 102	1187.6653	1186.6580	1186.6709	-0.0129	0	LYLLAAPAAER	(No match)
207 - 216	1169.5907	1168.5834	1168.5988	-0.0154	1	VTFSFEAGRR	(No match)
217 - 229	1531.6364	1530.6291	1530.6408	-0.0117	0	CVSGEGNFEFETR	(Ions score 44)
217 - 229	1531.6364	1530.6291	1530.6408	-0.0117	0	CVSGEGNFEFETR	(No match)
294 - 308	1608.7496	1607.7423	1607.7579	-0.0156	0	GQEGEYAVPFDAVAR	(Ions score 58)
294 - 308	1608.7496	1607.7423	1607.7579	-0.0156	0	GQEGEYAVPFDAVAR	(No match)

Spot 457

Protein View

Match to: **gi|6857820** Score: **554** Expect: **7.7e-051**
phosphatidylinositol-5-phosphate 4-kinase, type II, alpha [Homo sapiens]

Nominal mass (M_r): **46424**; Calculated pI value: **6.50**
NCBI BLAST search of gi|6857820 against nr
Unformatted sequence string for pasting into other applications

Taxonomy: Homo sapiens
Links to retrieve other entries containing this sequence from NCBI Entrez:
gi|73948884 from Canis lupus familiaris
gi|18266879 from Homo sapiens
gi|6760472 from Homo sapiens
gi|17390058 from Homo sapiens
gi|55664519 from Homo sapiens
gi|55665197 from Homo sapiens
gi|57160667 from Homo sapiens
gi|119606546 from Homo sapiens
gi|119606547 from Homo sapiens

Fixed modifications: Carbamidomethyl (C)
Variable modifications: Oxidation (M)
Cleavage by Trypsin: cuts C-term side of KR unless next residue is P
Sequence Coverage: **46%**

Matched peptides shown in **Bold Red**

1 MATPGNLGSS VLASKTKTKK KHFVAQKVKL FRASDPLLSV LMWGVNHSIN
51 ELSHVQIPVM LMPDDFKAYS KIKVDNHLFN KENMPSHFK**F KEYCPMVFR**N
101 LRER**FGIDDQ DFQNSLTRSA PLPNDSQAR**S GAR**FHTSYDK R**YIIK**TITSE**
151 **DVAEMHNILK KYHQYIVECH GITLLPQFLG MYRLNVDGVE IYVIVTR**NVF
201 SHRLSVYRKY DLKGSTVARE ASDKEKAK**EL PTLKDNDFIN EGQK**IYIDDN
251 NKKVFLEKLK K**DVEFLAQLK LMDYSLLVGI HDVER**AEQEE VECEENDGEE
301 EGESDGTHPV GTPPDSPGNT LNSSPPLAPG EFDPNIDVYG IK**CHENSPR**K
351 **EVYFMAIIDI LTHYDAKK**KA AHAAKTVK**HG AGAEISTVNP EQYSKRFLDF**
401 **IGHILT**

Residue Number Increasing Mass Decreasing Mass

Start - End	Observed	Mr(expt)	Mr(calc)	Delta	Miss	Sequence
90 - 99	1376.6346	1375.6273	1375.6416	-0.0143	1	FKEYCPMVFR (No match)
90 - 99	1376.6346	1375.6273	1375.6416	-0.0143	1	FKEYCPMVFR (Ions score 47)
90 - 99	1392.6335	1391.6262	1391.6366	-0.0103	1	FKEYCPMVFR Oxidation (M) (No match)
92 - 99	1101.4775	1100.4702	1100.4783	-0.0081	0	EYCPMVFR (No match)
105 - 118	1655.7535	1654.7462	1654.7587	-0.0124	0	FGIDDQDFQNSLTR (No match)
105 - 118	1655.7535	1654.7462	1654.7587	-0.0124	0	FGIDDQDFQNSLTR (Ions score 92)
119 - 129	1155.5688	1154.5615	1154.5680	-0.0064	0	SAPLPNDSQAR (No match)
134 - 141	1053.5039	1052.4966	1052.5039	-0.0073	1	FHTSYDKR (Ions score 11)
134 - 141	1053.5039	1052.4966	1052.5039	-0.0073	1	FHTSYDKR (No match)
146 - 161	1828.9323	1827.9250	1827.9400	-0.0150	1	TITSEDVAEMHNILKK (No match)
162 - 183	2738.3213	2737.3140	2737.3508	-0.0368	0	YHQYIVECHGITLLPQFLGMYR (No match)
184 - 197	1589.8752	1588.8679	1588.8824	-0.0144	0	LNVDGVEIYVIVTR (Ions score 97)
184 - 197	1589.8752	1588.8679	1588.8824	-0.0144	0	LNVDGVEIYVIVTR (No match)
229 - 244	1860.9207	1859.9134	1859.9264	-0.0130	1	ELPTLKDNDFINEGQK (No match)
262 - 270	1062.5740	1061.5667	1061.5757	-0.0089	0	DVEFLAQLK (No match)
271 - 285	1759.8916	1758.8843	1758.8974	-0.0131	0	LMDYSLLVGIHDVER (Ions score 65)
271 - 285	1759.8916	1758.8843	1758.8974	-0.0131	0	LMDYSLLVGIHDVER (No match)
271 - 285	1775.8823	1774.8750	1774.8923	-0.0173	0	LMDYSLLVGIHDVER Oxidation (M) (No match)
343 - 349	899.3704	898.3631	898.3715	-0.0084	0	CHENSPR (No match)
351 - 368	2170.1113	2169.1040	2169.1179	-0.0139	1	EVYFMAIIDILTHYDAKK (No match)
379 - 395	1787.8441	1786.8368	1786.8485	-0.0117	0	HGAGAEISTVNPEQYSK (No match)
379 - 396	1943.9407	1942.9334	1942.9496	-0.0162	1	HGAGAEISTVNPEQYSKR (No match)
397 - 406	1175.6290	1174.6217	1174.6386	-0.0169	0	FLDFIGHILT (No match)

Spot 458

Protein View

Match to: **gi|1710248** Score: **733** Expect: **9.6e-069**
protein disulfide isomerase-related protein 5 [Homo sapiens]

Nominal mass (M_r): **46512**; Calculated pI value: **4.95**
NCBI BLAST search of gi|1710248 against nr
Unformatted sequence string for pasting into other applications

Taxonomy: Homo sapiens

Fixed modifications: Carbamidomethyl (C)
Variable modifications: Oxidation (M)
Cleavage by Trypsin: cuts C-term side of KR unless next residue is P
Sequence Coverage: **38%**

Matched peptides shown in **Bold Red**

1 **LYSSSDDVIE LTPSNFNR**EV IQSDSLWLVE FYAPWCGHCQ RLTPEWKKAA
51 TALKDVVK**VG AVDADKHHSL GGQYGVQGFP TIK**IFGSNK**N RPEDYQGGRT**
101 **GEAIVDAALS ALR**QLVKDRL GGRSGGYSSG KQGRSDSSSK KDVIELTDDS
151 FDKNVLDSED VWMVEFYAPW CGHCKNLEPE WAAAASEVKE QTKGRVK**LAA**
201 **VDATVNQVLA SRYGIRGFPT IKIFQKGESP VDYDGGR**TRS DIVSRALDLF
251 SDNAPPPELL EIINEDIAKR **TCEEHQLCVV AVLPHILDTG AAGR**NSYLEV
301 LLKLADKYKK KMWGWLWTEA GAQSELETAL GIGGFGYPAM AAINARKMKF
351 ALLK**GSFSEQ GINEFLR**ELS FGR**GSTAPVG GGAFPTIVER** EPWDGRDGEL
401 PVEDDIDLSD VELDDLGKDE L

Residue Number Increasing Mass Decreasing Mass

Start - End	Observed	Mr(expt)	Mr(calc)	Delta	Miss	Sequence
1 - 18	2056.9458	2055.9385	2055.9749	-0.0363	0	LYSSSDDVIELTPSNFNR (Ions score 160)
1 - 18	2056.9458	2055.9385	2055.9749	-0.0363	0	LYSSSDDVIELTPSNFNR (No match)
59 - 83	2581.2817	2580.2744	2580.3084	-0.0339	1	VGAVDADKHHSLGGQYGVQGFPTIK (No match)
59 - 83	2581.2817	2580.2744	2580.3084	-0.0339	1	VGAVDADKHHSLGGQYGVQGFPTIK (Ions score 127)
67 - 83	1825.9026	1824.8953	1824.9270	-0.0317	0	HHSLGGQYGVQGFPTIK (No match)
90 - 99	1191.5214	1190.5141	1190.5428	-0.0287	0	NRPEDYQGGR (No match)
100 - 113	1386.7289	1385.7216	1385.7514	-0.0297	0	TGEAIVDAALSALR (No match)
198 - 212	1527.8186	1526.8113	1526.8416	-0.0303	0	LAAVDATVNQVLASR (Ions score 84)

198 - 212	1527.8186	1526.8113	1526.8416	-0.0303	0	**LAAVDATVNQVLASR** (No match)
213 - 222	1151.6915	1150.6842	1150.6498	0.0344	1	**YGIRGFPTIK** (No match)
223 - 237	1667.7706	1666.7633	1666.7950	-0.0317	1	**IFQKGESPVDYDGGR** (No match)
227 - 237	1151.4723	1150.4650	1150.4890	-0.0240	0	**GESPVDYDGGR** (No match)
271 - 294	2646.2742	2645.2669	2645.3053	-0.0384	0	**TCEEHQLCVVAVLPHILDTGAAGR** (No match)
355 - 367	1483.6882	1482.6809	1482.7102	-0.0293	0	**GSFSEQGINEFLR** (Ions score 87)
355 - 367	1483.6882	1482.6809	1482.7102	-0.0293	0	**GSFSEQGINEFLR** (No match)
374 - 390	1615.8123	1614.8050	1614.8365	-0.0314	0	**GSTAPVGGGAFPTIVER** (Ions score 82)
374 - 390	1615.8123	1614.8050	1614.8365	-0.0314	0	**GSTAPVGGGAFPTIVER** (No match)

Spot 459

Protein View

Match to: **gi|1710248** Score: **515** Expect: **6.1e-047**
protein disulfide isomerase-related protein 5 [Homo sapiens]

Nominal mass (M_r): **46512**; Calculated pI value: **4.95**
NCBI BLAST search of gi|1710248 against nr
Unformatted sequence string for pasting into other applications

Taxonomy: Homo sapiens

Fixed modifications: Carbamidomethyl (C)
Variable modifications: Oxidation (M)
Cleavage by Trypsin: cuts C-term side of KR unless next residue is P
Sequence Coverage: **36%**

Matched peptides shown in **Bold Red**

```
  1 LYSSSDDVIE LTPSNFNREV IQSDSLWLVE FYAPWCGHCQ RLTPEWKKAA
 51 TALKDVVKVG AVDADKHHSL GGQYGVQGFP TIKIFGSNKN RPEDYQGGRT
101 GEAIVDAALS ALRQLVKDRL GGRSGGYSSG KQGRSDSSSK KDVIELTDDS
151 FDKNVLDSED VWMVEFYAPW CGHCKNLEPE WAAAASEVKE QTKGRVKLAA
201 VDATVNQVLA SRYGIRGFPT IKIFQKGESP VDYDGGRTRS DIVSRALDLF
251 SDNAPPPELL EIINEDIAKR TCEEHQLCVV AVLPHILDTG AAGRNSYLEV
301 LLKLADKYKK KMWGWLWTEA GAQSELETAL GIGGFGYPAM AAINARKMKF
351 ALLKGSFSEQ GINEFLRELS FGRGSTAPVG GGAFPTIVER EPWDGRDGEL
401 PVEDDIDLSD VELDDLGKDE L
```

Residue Number Increasing Mass Decreasing Mass

Start - End	Observed	Mr(expt)	Mr(calc)	Delta	Miss	Sequence
1 - 18	2056.9902	2055.9829	2055.9749	0.0081	0	LYSSSDDVIELTPSNFNR (No match)
59 - 83	2581.3188	2580.3115	2580.3084	0.0032	1	VGAVDADKHHSLGGQYGVQGFPTIK (No match)
90 - 99	1191.5516	1190.5443	1190.5428	0.0015	0	NRPEDYQGGR (No match)
90 - 99	1191.5516	1190.5443	1190.5428	0.0015	0	NRPEDYQGGR (Ions score 46)
100 - 113	1386.7668	1385.7595	1385.7514	0.0082	0	TGEAIVDAALSALR (Ions score 99)
100 - 113	1386.7668	1385.7595	1385.7514	0.0082	0	TGEAIVDAALSALR (No match)
196 - 212	1755.0188	1754.0115	1754.0049	0.0066	1	VKLAAVDATVNQVLASR (No match)
198 - 212	1527.8563	1526.8490	1526.8416	0.0074	0	LAAVDATVNQVLASR (Ions score 78)

198 - 212	1527.8563	1526.8490	1526.8416	0.0074	0	LAAVDATVNQVLASR	(No match)
223 - 237	1667.8179	1666.8106	1666.7950	0.0156	1	IFQKGESPVDYDGGR	(No match)
227 - 237	1151.5040	1150.4967	1150.4890	0.0077	0	GESPVDYDGGR	(No match)
271 - 294	2646.2881	2645.2808	2645.3053	-0.0245	0	TCEEHQLCVVAVLPHILDTGAAGR	(No match)
355 - 367	1483.7267	1482.7194	1482.7102	0.0092	0	GSFSEQGINEFLR	(No match)
355 - 367	1483.7267	1482.7194	1482.7102	0.0092	0	GSFSEQGINEFLR	(Ions score 69)
374 - 390	1615.8524	1614.8451	1614.8365	0.0087	0	GSTAPVGGGAFPTIVER	(Ions score 69)
374 - 390	1615.8524	1614.8451	1614.8365	0.0087	0	GSTAPVGGGAFPTIVER	(No match)

Spot 460

Protein View

Match to: **gi|4759140** Score: **525** Expect: **6.1e-048**
solute carrier family 9 (sodium/hydrogen exchanger), isoform 3 regulator 1 [Homo sapiens]

Nominal mass (M_r): **39130;** Calculated pI value: **5.55**
NCBI BLAST search of gi|4759140 against nr
Unformatted sequence string for pasting into other applications

Taxonomy: Homo sapiens
Links to retrieve other entries containing this sequence from NCBI Entrez:
gi|41688557 from Homo sapiens
gi|2920585 from Homo sapiens
gi|3220019 from Homo sapiens
gi|12655175 from Homo sapiens
gi|13097186 from Homo sapiens
gi|15079985 from Homo sapiens
gi|31419627 from Homo sapiens
gi|119609596 from Homo sapiens

Fixed modifications: Carbamidomethyl (C)
Variable modifications: Oxidation (M)
Cleavage by Trypsin: cuts C-term side of KR unless next residue is P
Sequence Coverage: **51%**

Matched peptides shown in **Bold Red**

1 MSADAAAGAP LPRLCCLEK**G PNGYGFHLHG EKGKLGQYIR** LVEPGSPAEK
51 AGLLAGDR**LV EVNGENVEKE THQQVVSR**IR AALNAVR**LLV VDPETDEQLQ**
101 **K**LGVQVREEL LR**AQEAPGQA EPPAAAEVQG AGNENEPREA DKSHPEQR**EL
151 RPRLCTMK**KG PSGYGFNLHS DKSKPGQFIR SVDPDSPAEA SGLR**AQDRIV
201 EVNGVCMEGK **QHGDVVSAIR** AGGDETKLLV VDRETDEFFK KCR**VIPSQEH**
251 **LNGPLPVPFT NGEIQK**ENSR **EALAEAALES PRPALVR**SAS SDTSEELNSQ
301 DSPPKQDSTA PSSTSSSDPI LDFNISLAMA KERAHQKRSS K**RAPQMDWSK**
351 KNELFSNL

Residue Number Increasing Mass Decreasing Mass

Start - End	Observed	Mr(expt)	Mr(calc)	Delta	Miss	Sequence

20 - 32	1412.6654	1411.6581	1411.6632	-0.0051	0	GPNGYGFHLHGEK	(Ions score 74)
20 - 32	1412.6654	1411.6581	1411.6632	-0.0051	0	GPNGYGFHLHGEK	(No match)
33 - 40	934.5375	933.5302	933.5395	-0.0093	1	GKLGQYIR	(No match)
59 - 78	2294.1538	2293.1465	2293.1661	-0.0196	1	LVEVNGENVEKETHQQVVSR	(No match)
88 - 101	1626.8475	1625.8402	1625.8512	-0.0109	0	LLVVDPETDEQLQK	(No match)
113 - 138	2588.1746	2587.1673	2587.1898	-0.0224	0	AQEAPGQAEPPAAAEVQGAGNENEPR	(Ions score 135)
113 - 138	2588.1746	2587.1673	2587.1898	-0.0224	0	AQEAPGQAEPPAAAEVQGAGNENEPR	(No match)
139 - 148	1196.5585	1195.5512	1195.5581	-0.0069	1	EADKSHPEQR	(No match)
139 - 148	1196.5585	1195.5512	1195.5581	-0.0069	1	EADKSHPEQR	(Ions score 79)
159 - 172	1506.7277	1505.7204	1505.7262	-0.0058	1	KGPSGYGFNLHSDK	(No match)
173 - 180	932.5237	931.5164	931.5239	-0.0075	0	SKPGQFIR	(No match)
181 - 194	1400.6594	1399.6521	1399.6579	-0.0058	0	SVDPDSPAEASGLR	(No match)
211 - 220	1081.5681	1080.5608	1080.5676	-0.0067	0	QHGDVVSAIR	(No match)
244 - 266	2514.3208	2513.3135	2513.3277	-0.0142	0	VIPSQEHLNGPLPVPFTNGEIQK	(No match)
271 - 287	1792.9821	1791.9748	1791.9842	-0.0094	0	EALAEAALESPRPALVR	(No match)
271 - 287	1792.9821	1791.9748	1791.9842	-0.0094	0	EALAEAALESPRPALVR	(Ions score 59)
342 - 350	1118.5317	1117.5244	1117.5338	-0.0094	1	RAPQMDWSK	(No match)

Spot 461

Protein View

Match to: **gi|4504169** Score: **274** Expect: **7.7e-023**
glutathione synthetase [Homo sapiens]

Nominal mass (M_r): **52523;** Calculated pI value: **5.67**
NCBI BLAST search of gi|4504169 against nr
Unformatted sequence string for pasting into other applications

Taxonomy: Homo sapiens
Links to retrieve other entries containing this sequence from NCBI Entrez:
gi|1346191 from Homo sapiens
gi|5542519 from Homo sapiens
gi|886284 from Homo sapiens
gi|1236350 from Homo sapiens
gi|8248826 from Homo sapiens
gi|14043991 from Homo sapiens
gi|66841737 from Homo sapiens
gi|119596645 from Homo sapiens
gi|119596646 from Homo sapiens
gi|123993125 from synthetic construct
gi|124000117 from synthetic construct

Fixed modifications: Carbamidomethyl (C)
Variable modifications: Oxidation (M)
Cleavage by Trypsin: cuts C-term side of KR unless next residue is P
Sequence Coverage: **16%**

Matched peptides shown in **Bold Red**

1 MATNWGSLLQ DKQQLEELAR QAVDRALAEG VLLRTSQEPT SSEVVSYAPF
51 TLFPSLVPSA LLEQAYAVQM DFNLLVDAVS QNAAFLEQTL SSTIKQDDFT
101 ARLFDIHKQV LK**EGIAQTVF LGLNR**SDYMF QRSADGSPAL K**QIEINTISA**
151 **SFGGLASR**TP AVHRHVLSVL SKTKEAGKIL SNNPSKGLAL GIAKAWELYG
201 SPNALVLLIA QEKERNIFDQ RAIENELLAR NIHVIRRTFE DISEKGSLDQ
251 DRR**LFVDGQE IAVVYFR**DGY MPRQYSLQNW EARLLLERSH AAKCPDIATQ
301 LAGTKKVQQE LSRPGMLEML LPGQPEAVAR LRATFAGLYS LDVGEEGDQA
351 IAEALAAPSR FVLKPQREGG GNNLYGEEMV QALKQLKDSE ERASYILMEK
401 **IEPEPFENCL LRPGSPARVV QCISELGIFG VYVR**QEKTLV MNKHVGHLLR
451 TKAIEHADGG VAAGVAVLDN PYPV

Residue Number Increasing Mass Decreasing Mass

Start - End	Observed	Mr(expt)	Mr(calc)	Delta	Miss	Sequence
113 - 125	1417.7666	1416.7593	1416.7724	-0.0131	0	EGIAQTVFLGLNR (Ions score 53)
113 - 125	1417.7666	1416.7593	1416.7724	-0.0131	0	EGIAQTVFLGLNR (No match)
142 - 158	1763.9104	1762.9031	1762.9213	-0.0182	0	QIEINTISASFGGLASR (No match)
254 - 267	1655.8630	1654.8557	1654.8718	-0.0161	0	LFVDGQEIAVVYFR (No match)
254 - 267	1655.8630	1654.8557	1654.8718	-0.0161	0	LFVDGQEIAVVYFR (Ions score 77)
401 - 418	2082.0220	2081.0147	2081.0363	-0.0216	0	IEPEPFENCLLRPGSPAR (Ions score 59)
401 - 418	2082.0220	2081.0147	2081.0363	-0.0216	0	IEPEPFENCLLRPGSPAR (No match)
419 - 434	1838.9628	1837.9555	1837.9759	-0.0204	0	VVQCISELGIFGVYVR (No match)
419 - 434	1838.9628	1837.9555	1837.9759	-0.0204	0	VVQCISELGIFGVYVR (Ions score 25)

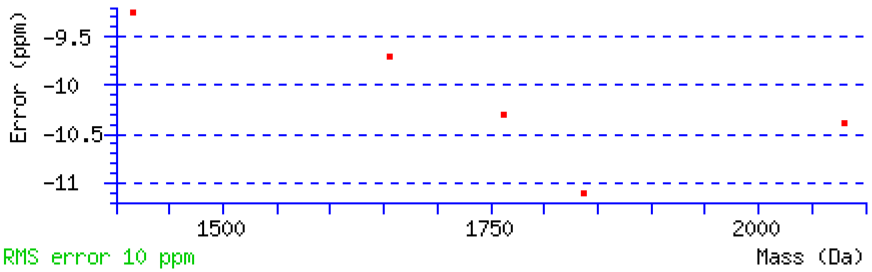

Spot 462

Protein View

Match to: **gi|48734966** Score: **182** Expect: **1.2e-013**
Eukaryotic translation elongation factor 1 alpha 1 [Homo sapiens]

Nominal mass (M_r): **50433**; Calculated pI value: **9.10**
NCBI BLAST search of gi|48734966 against nr
Unformatted sequence string for pasting into other applications

Taxonomy: Homo sapiens

Fixed modifications: Carbamidomethyl (C)
Variable modifications: Oxidation (M)
Cleavage by Trypsin: cuts C-term side of KR unless next residue is P
Sequence Coverage: **24%**

Matched peptides shown in **Bold Red**

```
  1 MGKEKTHINI VVIGHVDSGK STTTGHLIYK CGGIDKRTIE KFEKEAAEMG
 51 KGSFKYAWVL DKLKAERERG ITIDISLWKF ETSKYYVTII DAPGHRDFIK
101 NMITGTSQAD CAVLIVAAGV GEFEAGISKN GQTREHALLA YTLGVKQLIV
151 GVNKMDSTEP PYSQKRYEEI VKEVSTYIKK IGYNPDTVAF VPISGWNGDN
201 MLEPSANMPW FKGWKVTRKD GNASGTTLLE ALDCILPPTR PTDKPLRLPL
251 QDVYKIGGIG TVPVGRVETG VLKPGMVVTF APVNVTTEVK SVEIHHEALS
301 EALPGDNVGF NVKNVSVKDV RRGNVAGDSK NDPPMEAAGF TAQVIILNHP
351 GQISAGYAPV LDCHTAHIAC KFAELKEKID RRSGKKLEDG PKFLKSGDAA
401 IVDMVPGKPM CVESFSDYPP LGRFAVRDMR QTVAVGVIKA VDKKAAGAGK
451 VTKSAQKAQK AK
```

Residue Number Increasing Mass Decreasing Mass

Start - End	Observed	Mr(expt)	Mr(calc)	Delta	Miss	Sequence
6 - 20	1588.8612	1587.8539	1587.8732	-0.0193	0	THINIVVIGHVDSGK (No match)
85 - 96	1404.7104	1403.7031	1403.7197	-0.0166	0	YYVTIIDAPGHR (No match)
85 - 96	1404.7104	1403.7031	1403.7197	-0.0166	0	YYVTIIDAPGHR (Ions score 53)
85 - 100	1907.9780	1906.9707	1906.9941	-0.0233	1	YYVTIIDAPGHRDFIK (No match)
135 - 146	1314.7225	1313.7152	1313.7343	-0.0190	0	EHALLAYTLGVK (No match)
256 - 266	1025.5994	1024.5921	1024.6028	-0.0107	0	IGGIGTVPVGR (Ions score 42)
256 - 266	1025.5994	1024.5921	1024.6028	-0.0107	0	IGGIGTVPVGR (No match)

267 - 290	2531.3765	2530.3692	2530.3715	-0.0023	0	**VETGVLKPGMVVTFAPVNVTTEVK**	Oxidation (M) (No match)
396 - 423	3027.4258	3026.4185	3026.3823	0.0362	0	**SGDAAIVDMVPGKPMCVESFSDYPPLGR**	2 Oxidation (M) (No match)
431 - 439	914.5475	913.5402	913.5596	-0.0194	0	**QTVAVGVIK**	(No match)

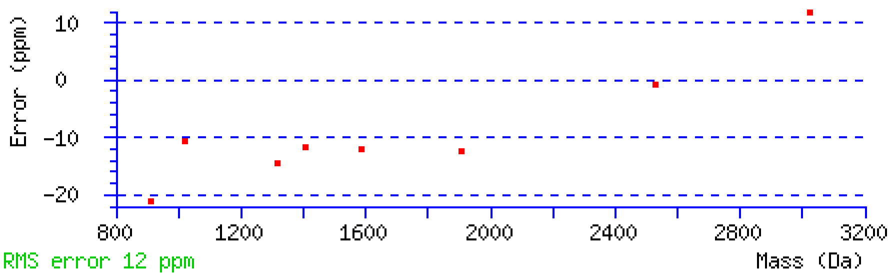

RMS error 12 ppm

Spot 463

Protein View

Match to: **gi|7245509** Score: **264** Expect: **7.7e-022**
Chain A, Human Bleomycin Hydrolase.

Nominal mass (M_r): **52869;** Calculated pI value: **6.00**
NCBI BLAST search of gi|7245509 against nr
Unformatted sequence string for pasting into other applications

Taxonomy: Homo sapiens
Links to retrieve other entries containing this sequence from NCBI Entrez:
gi|7546635 from Homo sapiens
gi|7546636 from Homo sapiens

Fixed modifications: Carbamidomethyl (C)
Variable modifications: Oxidation (M)
Cleavage by Trypsin: cuts C-term side of KR unless next residue is P
Sequence Coverage: **31%**

Matched peptides shown in **Bold Red**

```
  1 SSSGLNSEKV AALIQKLNSD PQFVLAQNVG TTHDLLDICL KRATVQRAQH
 51 VFQHAVPQEG KPITNQKSSG RCWIFSCLNV MRLPFMKKLN IEEFEFSQSY
101 LFFWDKVERC YFFLSAFVDT AQRKEPEDGR LVQFLLMNPA NDGGQWDMLV
151 NIVEKYGVIP KKCFPESYTT EATRRMNDIL NHKMREFCIR LRNLVHSGAT
201 KGEISATQDV MMEEIFRVVC ICLGNPPETF TWEYRDKDKN YQKIGPITPL
251 EFYREHVKPL FNMEDKICLV NDPRPQHKHN KLYTVEYLSN MVGGRKTLYN
301 NQPIDFLKKM VAASIKDGEA VWFGCDVGKH FNSKLGLSDM NLYDHELVFG
351 VSLKNMNKAE RLTFGESLMT HAMTFTAVSE KDDQDGAFTK WRVENSWGED
401 HGHKGYLCMT DEWFSEYVYE VVVDRKHVPE EVLAVLEQEP IILPAWDPMG
451 ALA
```

Residue Number Increasing Mass Decreasing Mass

Start - End	Observed	Mr(expt)	Mr(calc)	Delta	Miss	Sequence
48 - 67	2257.1592	2256.1519	2256.1763	-0.0243	0	AQHVFQHAVPQEGKPITNQK (No match)
48 - 67	2257.1592	2256.1519	2256.1763	-0.0243	0	AQHVFQHAVPQEGKPITNQK (Ions score 53)
72 - 82	1501.6553	1500.6480	1500.6676	-0.0195	0	CWIFSCLNVMR Oxidation (M) (No match)
110 - 123	1724.7886	1723.7813	1723.8028	-0.0214	0	CYFFLSAFVDTAQR (No match)

110 - 123	1724.7886	1723.7813	1723.8028	-0.0214	0	**CYFFLSAFVDTAQR** (Ions score 6)
162 - 174	1589.7111	1588.7038	1588.7191	-0.0153	1	**KCFPESYTTEATR** (No match)
163 - 174	1461.6179	1460.6106	1460.6242	-0.0135	0	**CFPESYTTEATR** (No match)
202 - 217	1887.8323	1886.8250	1886.8389	-0.0139	0	**GEISATQDVMMEEIFR** 2 Oxidation (M) (No match)
218 - 235	2241.0251	2240.0178	2240.0394	-0.0216	0	**VVCICLGNPPETFTWEYR** (No match)
244 - 254	1305.7046	1304.6973	1304.7128	-0.0155	0	**IGPITPLEFYR** (No match)
244 - 254	1305.7046	1304.6973	1304.7128	-0.0155	0	**IGPITPLEFYR** (Ions score 41)
267 - 278	1476.7531	1475.7458	1475.7667	-0.0209	0	**ICLVNDPRPQHK** (No match)
267 - 278	1476.7531	1475.7458	1475.7667	-0.0209	0	**ICLVNDPRPQHK** (Ions score 16)
282 - 295	1601.7686	1600.7613	1600.7919	-0.0305	0	**LYTVEYLSNMVGGR** (No match)
282 - 295	1617.7700	1616.7627	1616.7868	-0.0240	0	**LYTVEYLSNMVGGR** Oxidation (M) (No match)
282 - 295	1617.7700	1616.7627	1616.7868	-0.0240	0	**LYTVEYLSNMVGGR** Oxidation (M) (Ions score 8)
282 - 296	1745.8219	1744.8146	1744.8817	-0.0671	1	**LYTVEYLSNMVGGRK** Oxidation (M) (No match)
297 - 309	1593.8477	1592.8404	1592.8562	-0.0158	1	**TLYNNQPIDFLKK** (No match)

Spot 464

Protein View

Match to: **gi|5031573** Score: **715** Expect: **6.1e-067**
ARP3 actin-related protein 3 homolog [Homo sapiens]

Nominal mass (M_r): **47797**; Calculated pI value: **5.61**
NCBI BLAST search of gi|5031573 against nr
Unformatted sequence string for pasting into other applications

Taxonomy: Homo sapiens
Links to retrieve other entries containing this sequence from NCBI Entrez:
gi|27806335 from Bos taurus
gi|114580542 from Pan troglodytes
gi|47117646 from Bos taurus
gi|47117647 from Homo sapiens
gi|62899710 from Pongo pygmaeus
gi|17943199 from Bos taurus
gi|56966172 from Bos taurus
gi|56966192 from Bos taurus
gi|149243009 from Bos taurus
gi|149243024 from Bos taurus
gi|149243031 from Bos taurus
gi|149243038 from Bos taurus
gi|149243045 from Bos taurus
gi|149243052 from Bos taurus
gi|149243059 from Bos taurus
gi|217422 from Bos taurus
gi|2282032 from Homo sapiens
gi|5805246 from Homo sapiens
gi|27882036 from Homo sapiens
gi|55730253 from Pongo pygmaeus
gi|56403671 from Pongo pygmaeus
gi|62702302 from Homo sapiens
gi|119615585 from Homo sapiens
gi|119615586 from Homo sapiens
gi|148744289 from Bos taurus

Fixed modifications: Carbamidomethyl (C)
Variable modifications: Oxidation (M)
Cleavage by Trypsin: cuts C-term side of KR unless next residue is P
Sequence Coverage: **54%**

Matched peptides shown in **Bold Red**

```
  1 MAGRLPACVV DCGTGYTKLG YAGNTEPQFI IPSCIAIKES AKVGDQAQRR
 51 VMKGVDDLDF FIGDEAIEKP TYATKWPIRH GIVEDWDLME RFMEQVIFKY
101 LRAEPEDHYF LLTEPPLNTP ENREYTAEIM FESFNVPGLY IAVQAVLALA
151 ASWTSRQVGE RTLTGTVIDS GDGVTHVIPV AEGYVIGSCI KHIPIAGRDI
201 TYFIQQLLRD REVGIPPEQS LETAKAVKER YSYVCPDLVK EFNKYDTDGS
251 KWIKQYTGIN AISKKEFSID VGYERFLGPE IFFHPEFANP DFTQPISEVV
301 DEVIQNCPID VRRPLYKNIV LSGGSTMFRD FGRRLQRDLK RTVDARLKLS
351 EELSGGRLKP KPIDVQVITH HMQRYAVWFG GSMLASTPEF YQVCHTKKDY
401 EEIGPSICRH NPVFGVMS
```

Residue Number Increasing Mass Decreasing Mass

Start - End	Observed	Mr(expt)	Mr(calc)	Delta	Miss	Sequence
5 - 18	1540.7019	1539.6946	1539.7061	-0.0115	0	LPACVVDCGTGYTK (No match)
19 - 38	2192.1379	2191.1306	2191.1346	-0.0040	0	LGYAGNTEPQFIIPSCIAIK (No match)
54 - 75	2444.1919	2443.1846	2443.1794	0.0052	0	GVDDLDFFIGDEAIEKPTYATK (No match)
92 - 99	1057.5240	1056.5167	1056.5313	-0.0146	0	FMEQVIFK Oxidation (M) (No match)
103 - 123	2482.1782	2481.1709	2481.1812	-0.0102	0	AEPEDHYFLLTEPPLNTPENR (Ions score 144)
103 - 123	2482.1782	2481.1709	2481.1812	-0.0102	0	AEPEDHYFLLTEPPLNTPENR (No match)
162 - 191	3058.5938	3057.5865	3057.5691	0.0174	0	TLTGTVIDSGDGVTHVIPVAEGYVIGSCIK (No match)
199 - 209	1409.7671	1408.7598	1408.7714	-0.0116	0	DITYFIQQLLR (Ions score 97)
199 - 209	1409.7671	1408.7598	1408.7714	-0.0116	0	DITYFIQQLLR (No match)
210 - 225	1768.8955	1767.8882	1767.9002	-0.0120	1	DREVGIPPEQSLETAK (Ions score 63)
210 - 225	1768.8955	1767.8882	1767.9002	-0.0120	1	DREVGIPPEQSLETAK (No match)
212 - 225	1497.7599	1496.7526	1496.7722	-0.0195	0	EVGIPPEQSLETAK (No match)
231 - 240	1243.5901	1242.5828	1242.5954	-0.0126	0	YSYVCPDLVK (No match)
241 - 251	1303.5707	1302.5634	1302.5728	-0.0093	1	EFNKYDTDGSK (No match)
265 - 275	1342.6506	1341.6433	1341.6564	-0.0131	1	KEFSIDVGYER (No match)
266 - 275	1214.5591	1213.5518	1213.5614	-0.0096	0	EFSIDVGYER (Ions score 99)
266 - 275	1214.5591	1213.5518	1213.5614	-0.0096	0	EFSIDVGYER (No match)
318 - 329	1297.6404	1296.6331	1296.6496	-0.0164	0	NIVLSGGSTMFR Oxidation (M) (No match)
347 - 357	1188.6514	1187.6441	1187.6509	-0.0068	1	LKLSEELSGGR (No match)
349 - 357	947.4783	946.4710	946.4719	-0.0009	0	LSEELSGGR (No match)
358 - 374	2056.1384	2055.1311	2055.1411	-0.0100	0	LKPKPIDVQVITHHMQR Oxidation (M) (Ions score 25)
358 - 374	2056.1384	2055.1311	2055.1411	-0.0100	0	LKPKPIDVQVITHHMQR Oxidation (M) (No match)
398 - 409	1466.6846	1465.6773	1465.6871	-0.0097	1	KDYEEIGPSICR (No match)
399 - 409	1338.5883	1337.5810	1337.5921	-0.0111	0	DYEEIGPSICR (No match)

Spot 465

Protein View

Match to: **gi|5031573** Score: **729** Expect: **2.4e-068**
ARP3 actin-related protein 3 homolog [Homo sapiens]

Nominal mass (M_r): **47797**; Calculated pI value: **5.61**
NCBI BLAST search of gi|5031573 against nr
Unformatted sequence string for pasting into other applications

Taxonomy: Homo sapiens
Links to retrieve other entries containing this sequence from NCBI Entrez:
gi|27806335 from Bos taurus
gi|114580542 from Pan troglodytes
gi|47117646 from Bos taurus
gi|47117647 from Homo sapiens
gi|62899710 from Pongo pygmaeus
gi|17943199 from Bos taurus
gi|56966172 from Bos taurus
gi|56966192 from Bos taurus
gi|149243009 from Bos taurus
gi|149243024 from Bos taurus
gi|149243031 from Bos taurus
gi|149243038 from Bos taurus
gi|149243045 from Bos taurus
gi|149243052 from Bos taurus
gi|149243059 from Bos taurus
gi|217422 from Bos taurus
gi|2282032 from Homo sapiens
gi|5805246 from Homo sapiens
gi|27882036 from Homo sapiens
gi|55730253 from Pongo pygmaeus
gi|56403671 from Pongo pygmaeus
gi|62702302 from Homo sapiens
gi|119615585 from Homo sapiens
gi|119615586 from Homo sapiens
gi|148744289 from Bos taurus

Fixed modifications: Carbamidomethyl (C)
Variable modifications: Oxidation (M)
Cleavage by Trypsin: cuts C-term side of KR unless next residue is P
Sequence Coverage: **64%**

Matched peptides shown in **Bold Red**

```
  1 MAGRLPACVV DCGTGYTKLG YAGNTEPQFI IPSCIAIKES AKVGDQAQRR
 51 VMKGVDDLDF FIGDEAIEKP TYATKWPIRH GIVEDWDLME RFMEQVIFKY
101 LRAEPEDHYF LLTEPPLNTP ENREYTAEIM FESFNVPGLY IAVQAVLALA
151 ASWTSRQVGE RTLTGTVIDS GDGVTHVIPV AEGYVIGSCI KHIPIAGRDI
201 TYFIQQLLRD REVGIPPEQS LETAKAVKER YSYVCPDLVK EFNKYDTDGS
251 KWIKQYTGIN AISKKEFSID VGYERFLGPE IFFHPEFANP DFTQPISEVV
301 DEVIQNCPID VRRPLYKNIV LSGGSTMFRD FGRRLQRDLK RTVDARLKLS
351 EELSGGRLKP KPIDVQVITH HMQRYAVWFG GSMLASTPEF YQVCHTKKDY
401 EEIGPSICRH NPVFGVMS
```

Residue Number Increasing Mass Decreasing Mass

Start - End	Observed	Mr(expt)	Mr(calc)	Delta	Miss	Sequence
5 - 18	1540.7085	1539.7012	1539.7061	-0.0049	0	LPACVVDCGTGYTK (No match)
19 - 38	2192.1360	2191.1287	2191.1346	-0.0059	0	LGYAGNTEPQFIIPSCIAIK (No match)
54 - 75	2444.1794	2443.1721	2443.1794	-0.0073	0	GVDDLDFFIGDEAIEKPTYATK (No match)
80 - 91	1499.6927	1498.6854	1498.6874	-0.0020	0	HGIVEDWDLMER (No match)
80 - 91	1515.6801	1514.6728	1514.6823	-0.0095	0	HGIVEDWDLMER Oxidation (M) (No match)
92 - 99	1041.5367	1040.5294	1040.5364	-0.0070	0	FMEQVIFK (No match)
92 - 99	1057.5313	1056.5240	1056.5313	-0.0073	0	FMEQVIFK Oxidation (M) (No match)
103 - 123	2482.1677	2481.1604	2481.1812	-0.0207	0	AEPEDHYFLLTEPPLNTPENR (No match)
103 - 123	2482.1677	2481.1604	2481.1812	-0.0207	0	AEPEDHYFLLTEPPLNTPENR (Ions score 136)
162 - 191	3058.5657	3057.5584	3057.5691	-0.0107	0	TLTGTVIDSGDGVTHVIPVAEGYVIGSCIK (No match)
199 - 209	1409.7733	1408.7660	1408.7714	-0.0054	0	DITYFIQQLLR (Ions score 90)
199 - 209	1409.7733	1408.7660	1408.7714	-0.0054	0	DITYFIQQLLR (No match)
210 - 225	1768.8973	1767.8900	1767.9002	-0.0102	1	DREVGIPPEQSLETAK (Ions score 44)
210 - 225	1768.8973	1767.8900	1767.9002	-0.0102	1	DREVGIPPEQSLETAK (No match)
212 - 225	1497.7692	1496.7619	1496.7722	-0.0102	0	EVGIPPEQSLETAK (No match)
231 - 240	1243.5942	1242.5869	1242.5954	-0.0085	0	YSYVCPDLVK (No match)
241 - 251	1303.5874	1302.5801	1302.5728	0.0074	1	EFNKYDTDGSK (No match)
265 - 275	1342.6550	1341.6477	1341.6564	-0.0087	1	KEFSIDVGYER (No match)
266 - 275	1214.5646	1213.5573	1213.5614	-0.0041	0	EFSIDVGYER (No match)
266 - 275	1214.5646	1213.5573	1213.5614	-0.0041	0	EFSIDVGYER (Ions score 95)
318 - 329	1281.6587	1280.6514	1280.6546	-0.0032	0	NIVLSGGSTMFR (No match)
318 - 329	1297.6472	1296.6399	1296.6496	-0.0096	0	NIVLSGGSTMFR Oxidation (M) (No match)
347 - 357	1188.6541	1187.6468	1187.6509	-0.0041	1	LKLSEELSGGR (No match)
349 - 357	947.4789	946.4716	946.4719	-0.0003	0	LSEELSGGR (No match)
358 - 374	2040.1390	2039.1317	2039.1462	-0.0145	0	LKPKPIDVQVITHHMQR (No match)
358 - 374	2056.1340	2055.1267	2055.1411	-0.0144	0	LKPKPIDVQVITHHMQR Oxidation (M) (No match)
375 - 397	2679.2156	2678.2083	2678.2297	-0.0214	0	YAVWFGGSMLASTPEFYQVCHTK (No match)
398 - 409	1466.6898	1465.6825	1465.6871	-0.0045	1	KDYEEIGPSICR (No match)
399 - 409	1338.5947	1337.5874	1337.5921	-0.0047	0	DYEEIGPSICR (Ions score 65)
399 - 409	1338.5947	1337.5874	1337.5921	-0.0047	0	DYEEIGPSICR (No match)
410 - 418	987.4649	986.4576	986.4643	-0.0067	0	HNPVFGVMS (No match)
410 - 418	1003.4630	1002.4557	1002.4592	-0.0035	0	HNPVFGVMS Oxidation (M) (No match)

Spot 466

Protein View

Match to: **gi|119586320** Score: **112** Expect: **1.2e-006**
sorting nexin 6, isoform CRA_c [Homo sapiens]

Nominal mass (M_r): **36942**; Calculated pI value: **5.75**
NCBI BLAST search of gi|119586320 against nr
Unformatted sequence string for pasting into other applications

Taxonomy: Homo sapiens

Fixed modifications: Carbamidomethyl (C)
Variable modifications: Oxidation (M)
Cleavage by Trypsin: cuts C-term side of KR unless next residue is P
Sequence Coverage: **35%**

Matched peptides shown in **Bold Red**

```
  1 MRACAGPRLG AAMMEGLDDG PDFLSEEDRG LKAINVDLQS DAALQVDISD
 51 ALSERDKVKF TVHTKSSLPN FKQNEFSVVR QHEEFIWLHD SFVENEDYAG
101 YIIPPAPPRP DFDASREKLQ KLGEGEGSMT KEEFTKMKQE LEAEYLAIFK
151 KTVAMHEVFL CRVAAHPILR RDLNFHVFLE YNQDLSVRGK NKKEKLEDFF
201 KNMVKSADGV IVSGVKDVDD FFEHERTFLL EYHNRVKDAS AKSDRMTRSH
251 KSAADDYNRI GSSLYALGTQ DSTDICKFFL KVSELFDKTR SLALSPKLEC
301 SGTILAHCNL CLLGSKNRST SVC
```

Residue Number Increasing Mass Decreasing Mass

Start - End	Observed	Mr(expt)	Mr(calc)	Delta	Miss	Sequence
33 - 55	2443.1343	2442.1270	2442.2237	-0.0967	0	AINVDLQSDAALQVDISDALSER (No match)
66 - 80	1751.8533	1750.8460	1750.9001	-0.0541	1	SSLPNFKQNEFSVVR (No match)
73 - 80	978.4728	977.4655	977.4930	-0.0275	0	QNEFSVVR (No match)
152 - 162	1378.6172	1377.6099	1377.6533	-0.0434	0	TVAMHEVFLCR Oxidation (M) (No match)
163 - 171	1032.6089	1031.6016	1031.6352	-0.0336	1	VAAHPILRR (No match)
172 - 188	2108.9688	2107.9615	2108.0326	-0.0711	0	DLNFHVFLEYNQDLSVR (No match)
206 - 226	2321.0208	2320.0135	2320.0970	-0.0835	1	SADGVIVSGVKDVDDFFEHER (No match)
217 - 226	1308.5121	1307.5048	1307.5418	-0.0370	0	DVDDFFEHER (No match)
217 - 235	2482.0886	2481.0813	2481.1349	-0.0535	1	DVDDFFEHERTFLLEYHNR (No match)
217 - 235	2482.0886	2481.0813	2481.1349	-0.0535	1	DVDDFFEHERTFLLEYHNR (No match)

227 - 235	1192.5768	1191.5695	1191.6036	-0.0341	0	TFLLEYHNR	(Ions score 21)
227 - 235	1192.5768	1191.5695	1191.6036	-0.0341	0	TFLLEYHNR	(No match)
282 - 290	1094.5564	1093.5491	1093.5767	-0.0276	1	VSELFDKTR	(No match)

Spot 467

Protein View

Match to: **gi|119606836** Score: **384** Expect: **7.7e-034**
GDP dissociation inhibitor 2, isoform CRA_a [Homo sapiens]

Nominal mass (M_r): **48680**; Calculated pI value: **7.51**
NCBI BLAST search of gi|119606836 against nr
Unformatted sequence string for pasting into other applications

Taxonomy: Homo sapiens

Fixed modifications: Carbamidomethyl (C)
Variable modifications: Oxidation (M)
Cleavage by Trypsin: cuts C-term side of KR unless next residue is P
Sequence Coverage: **43%**

Matched peptides shown in **Bold Red**

```
  1 MSVNGKKVLH MDRNPYYGGE SASITPLEDL YKRFKIPGSP PESMGRGRDW
 51 NVDLIPKFLM ANGQLVKMLL YTEVTRYLDF KVTEGSFVYK GGKIYKVPST
101 EAEALASSLM GLFEKRRFRK FLVYVANFDE KDPRTFEGID PKKTTMRDVY
151 KKFDLGQDVI DFTGHALALY RTDDYLDQPC YETINRIKLY SESLARYGKS
201 PYLYPLYGLG ELPQGFARLS AIYGGTYMLN KPIEEIIVQN GKVIGVKSEG
251 EIARCKQLIC DPSYVKDRVE KVGQVIRVIC ILSHPIKNTN DANSCQIIIP
301 QNQVNRKSDI YVCMISFAHN VAAQGKYIAI VSTTVETKEP EKEIRPALEL
351 LEPIEQKFVS ISDLLVPKDL GTESQIFISR TYDATTHFET TCDDIKNIYK
401 RMTGSEFDFE EMKRKKNDIY GED
```

Residue Number Increasing Mass Decreasing Mass

Start - End	Observed	Mr(expt)	Mr(calc)	Delta	Miss	Sequence
14 - 33	2273.0730	2272.0657	2272.1011	-0.0354	1	NPYYGGESASITPLEDLYKR (No match)
34 - 46	1418.6807	1417.6734	1417.7023	-0.0289	1	FKIPGSPPESMGR Oxidation (M) (No match)
34 - 46	1418.6807	1417.6734	1417.7023	-0.0289	1	FKIPGSPPESMGR Oxidation (M) (Ions score 7)
47 - 57	1312.6786	1311.6713	1311.6935	-0.0222	1	GRDWNVDLIPK (No match)
68 - 76	1141.5701	1140.5628	1140.5849	-0.0220	0	MLLYTEVTR Oxidation (M) (No match)
121 - 134	1712.8367	1711.8294	1711.8569	-0.0275	1	FLVYVANFDEKDPR (No match)
172 - 186	1902.7833	1901.7760	1901.8101	-0.0341	0	TDDYLDQPCYETINR (No match)
172 - 186	1902.7833	1901.7760	1901.8101	-0.0341	0	TDDYLDQPCYETINR (Ions score 56)

187 - 196	1179.6573	1178.6500	1178.6659	-0.0158	1	**IKLYSESLAR** (No match)
200 - 218	2141.0691	2140.0618	2140.0992	-0.0374	0	**SPYLYPLYGLGELPQGFAR** (No match)
200 - 218	2141.0691	2140.0618	2140.0992	-0.0374	0	**SPYLYPLYGLGELPQGFAR** (Ions score 75)
288 - 306	2199.0227	2198.0154	2198.0498	-0.0343	0	**NTNDANSCQIIIPQNQVNR** (No match)
288 - 306	2199.0227	2198.0154	2198.0498	-0.0343	0	**NTNDANSCQIIIPQNQVNR** (Ions score 59)
343 - 357	1777.9803	1776.9730	1776.9985	-0.0255	0	**EIRPALELLEPIEQK** (No match)
369 - 380	1365.6793	1364.6720	1364.6935	-0.0215	0	**DLGTESQIFISR** (Ions score 39)
369 - 380	1365.6793	1364.6720	1364.6935	-0.0215	0	**DLGTESQIFISR** (No match)
381 - 396	1917.7933	1916.7860	1916.8098	-0.0238	0	**TYDATTHFETTCDDIK** (No match)
402 - 414	1638.6556	1637.6483	1637.6701	-0.0218	1	**MTGSEFDFEEMKR** 2 Oxidation (M) (No match)

Spot 468

Protein View

Match to: **gi|48255968** Score: **290** Expect: **1.9e-024**
UDP-glucose pyrophosphorylase 2 isoform b [Homo sapiens]

Nominal mass (M_r): **55813;** Calculated pI value: **7.69**
NCBI BLAST search of gi|48255968 against nr
Unformatted sequence string for pasting into other applications

Taxonomy: Homo sapiens
Links to retrieve other entries containing this sequence from NCBI Entrez:
gi|114577657 from Pan troglodytes
gi|114577659 from Pan troglodytes
gi|114577661 from Pan troglodytes
gi|114577663 from Pan troglodytes
gi|114577665 from Pan troglodytes
gi|114577667 from Pan troglodytes
gi|12804193 from Homo sapiens
gi|55730161 from Pongo pygmaeus

Fixed modifications: Carbamidomethyl (C)
Variable modifications: Oxidation (M)
Cleavage by Trypsin: cuts C-term side of KR unless next residue is P
Sequence Coverage: **38%**

Matched peptides shown in **Bold Red**

1 MSQDGASQFQ EVIRQELELS VKKELEK**ILT TASSHEFEHT KK**DLDGFRKL
51 FHRFLQEKGP SVDWGK**IQRP PEDSIQPYEK** IKARGLPDNI SSVLNKLVVV
101 KLNGGLGTSM GCKGPKSLIG VR**NENTFLDL TVQQIEHLNK TYNTDVPLVL**
151 **MNSFNTDEDT KK**ILQKYNHC R**VKIYTFNQS R**YPRINKESL LPVAKDVSYS
201 GENTEAWYPP GHGDIYASFY NSGLLDTFIG EGKEYIFVSN IDNLGATVDL
251 YILNHLMNPP NGKRCEFVME VTNKTRADVK GGTLTQYEGK LR**LVEIAQVP**
301 **K**AHVDEFKSV SKFKIFNTNN LWISLAAVKR **LQEQNAIDME IIVNAKTLDG**
351 **GLNVIQLETA VGAAIKSFEN SLGINVPR**SR FLPVKTTSDL LLVMSNLYSL
401 NAGSLTMSEK REFPTVPLVK LGSSFTKVQD YLR**RFESIPD MLELDHLTVS**
451 **GDVTFGK**NVS LK**GTVIIIAN HGDRIDIPPG AVLENKIVSG NLR**ILDH

Residue Number Increasing Mass Decreasing Mass

Start - End	Observed	Mr(expt)	Mr(calc)	Delta	Miss	Sequence
28 - 42	1728.8477	1727.8404	1727.8842	-0.0438	1	ILTTASSHEFEHTKK (No match)
67 - 80	1699.8207	1698.8134	1698.8577	-0.0442	0	IQRPPEDSIQPYEK (No match)
67 - 80	1699.8207	1698.8134	1698.8577	-0.0442	0	IQRPPEDSIQPYEK (Ions score 20)
123 - 140	2156.0439	2155.0366	2155.0909	-0.0543	0	NENTFLDLTVQQIEHLNK (No match)
123 - 140	2156.0439	2155.0366	2155.0909	-0.0543	0	NENTFLDLTVQQIEHLNK (Ions score 62)
141 - 161	2433.1550	2432.1477	2432.1053	0.0424	0	TYNTDVPLVLMNSFNTDEDTK Oxidation (M) (No match)
141 - 162	2561.2285	2560.2212	2560.2002	0.0210	1	TYNTDVPLVLMNSFNTDEDTKK Oxidation (M) (No match)
172 - 181	1255.6466	1254.6393	1254.6720	-0.0327	1	VKIYTFNQSR (No match)
174 - 181	1028.4866	1027.4793	1027.5087	-0.0293	0	IYTFNQSR (No match)
174 - 181	1028.4866	1027.4793	1027.5087	-0.0293	0	IYTFNQSR (Ions score 14)
293 - 301	996.5646	995.5573	995.6015	-0.0441	0	LVEIAQVPK (No match)
331 - 346	1844.8976	1843.8903	1843.9349	-0.0446	0	LQEQNAIDMEIIVNAK Oxidation (M) (No match)
347 - 366	1983.0629	1982.0556	1982.1047	-0.0491	0	TLDGGLNVIQLETAVGAAIK (No match)
367 - 378	1332.6566	1331.6493	1331.6833	-0.0340	0	SFENSLGINVPR (No match)
367 - 378	1332.6566	1331.6493	1331.6833	-0.0340	0	SFENSLGINVPR (Ions score 44)
434 - 457	2722.2815	2721.2742	2721.3319	-0.0577	1	RFESIPDMLELDHLTVSGDVTFGK Oxidation (M) (No match)
463 - 486	2512.3130	2511.3057	2511.3808	-0.0751	1	GTVIIIANHGDRIDIPPGAVLENK (No match)
475 - 486	1265.7139	1264.7066	1264.7026	0.0040	0	IDIPPGAVLENK (No match)
475 - 493	2005.0485	2004.0412	2004.1367	-0.0954	1	IDIPPGAVLENKIVSGNLR (No match)

Spot 469

Protein View

Match to: **gi|119606836** Score: **676** Expect: **4.8e-063**
GDP dissociation inhibitor 2, isoform CRA_a [Homo sapiens]

Nominal mass (M_r): **48680**; Calculated pI value: **7.51**
NCBI BLAST search of gi|119606836 against nr
Unformatted sequence string for pasting into other applications

Taxonomy: Homo sapiens

Fixed modifications: Carbamidomethyl (C)
Variable modifications: Oxidation (M)
Cleavage by Trypsin: cuts C-term side of KR unless next residue is P
Sequence Coverage: **53%**

Matched peptides shown in **Bold Red**

```
  1 MSVNGKKVLH MDRNPYYGGE SASITPLEDL YKRFKIPGSP PESMGRGRDW
 51 NVDLIPKFLM ANGQLVKMLL YTEVTRYLDF KVTEGSFVYK GGKIYKVPST
101 EAEALASSLM GLFEKRRFRK FLVYVANFDE KDPRTFEGID PKKTTMRDVY
151 KKFDLGQDVI DFTGHALALY RTDDYLDQPC YETINRIKLY SESLARYGKS
201 PYLYPLYGLG ELPQGFARLS AIYGGTYMLN KPIEEIIVQN GKVIGVKSEG
251 EIARCKQLIC DPSYVKDRVE KVGQVIRVIC ILSHPIKNTN DANSCQIIIP
301 QNQVNRKSDI YVCMISFAHN VAAQGKYIAI VSTTVETKEP EKEIRPALEL
351 LEPIEQKFVS ISDLLVPKDL GTESQIFISR TYDATTHFET TCDDIKNIYK
401 RMTGSEFDFE EMKRKKNDIY GED
```

Residue Number Increasing Mass Decreasing Mass

Start - End	Observed	Mr(expt)	Mr(calc)	Delta	Miss	Sequence
14 - 33	2273.0283	2272.0210	2272.1011	-0.0801	1	NPYYGGESASITPLEDLYKR (No match)
14 - 33	2273.0283	2272.0210	2272.1011	-0.0801	1	NPYYGGESASITPLEDLYKR (Ions score 101)
34 - 46	1418.6522	1417.6449	1417.7023	-0.0574	1	FKIPGSPPESMGR Oxidation (M) (No match)
58 - 67	1136.5630	1135.5557	1135.6059	-0.0502	0	FLMANGQLVK Oxidation (M) (No match)
68 - 76	1141.5454	1140.5381	1140.5849	-0.0467	0	MLLYTEVTR Oxidation (M) (No match)
97 - 115	1995.9272	1994.9199	1994.9870	-0.0670	0	VPSTEAEALASSLMGLFEK Oxidation (M) (No match)
121 - 134	1712.7994	1711.7921	1711.8569	-0.0648	1	FLVYVANFDEKDPR (No match)
121 - 134	1712.7994	1711.7921	1711.8569	-0.0648	1	FLVYVANFDEKDPR (Ions score 27)

172 - 186	1902.7484	1901.7411	1901.8101	-0.0690	0	TDDYLDQPCYETINR (Ions score 88)
172 - 186	1902.7484	1901.7411	1901.8101	-0.0690	0	TDDYLDQPCYETINR (No match)
187 - 196	1179.6359	1178.6286	1178.6659	-0.0372	1	IKLYSESLAR (No match)
189 - 196	938.4662	937.4589	937.4868	-0.0279	0	LYSESLAR (No match)
200 - 218	2141.0281	2140.0208	2140.0992	-0.0784	0	SPYLYPLYGLGELPQGFAR (Ions score 134)
200 - 218	2141.0281	2140.0208	2140.0992	-0.0784	0	SPYLYPLYGLGELPQGFAR (No match)
219 - 242	2667.3486	2666.3413	2666.3988	-0.0575	0	LSAIYGGTYMLNKPIEEIIVQNGK Oxidation (M) (No match)
288 - 306	2198.9795	2197.9722	2198.0498	-0.0775	0	NTNDANSCQIIIPQNQVNR (No match)
343 - 357	1777.9398	1776.9325	1776.9985	-0.0659	0	EIRPALELLEPIEQK (Ions score 80)
343 - 357	1777.9398	1776.9325	1776.9985	-0.0659	0	EIRPALELLEPIEQK (No match)
369 - 380	1365.6497	1364.6424	1364.6935	-0.0511	0	DLGTESQIFISR (No match)
381 - 396	1917.7485	1916.7412	1916.8098	-0.0686	0	TYDATTHFETTCDDIK (No match)
402 - 414	1638.6140	1637.6067	1637.6701	-0.0634	1	MTGSEFDFEEMKR 2 Oxidation (M) (No match)

Spot 470

Protein View

Match to: **gi|5454140** Score: **109** Expect: **2.4e-006**
tumor susceptibility gene 101 [Homo sapiens]

Nominal mass (M_r): **44088;** Calculated pI value: **6.06**
NCBI BLAST search of gi|5454140 against nr
Unformatted sequence string for pasting into other applications

Taxonomy: Homo sapiens
Links to retrieve other entries containing this sequence from NCBI Entrez:
gi|114636476 from Pan troglodytes
gi|9789790 from Homo sapiens
gi|3184258 from Homo sapiens
gi|60813968 from synthetic construct
gi|61355217 from synthetic construct
gi|119588791 from Homo sapiens

Fixed modifications: Carbamidomethyl (C)
Variable modifications: Oxidation (M)
Cleavage by Trypsin: cuts C-term side of KR unless next residue is P
Sequence Coverage: **9%**

Matched peptides shown in **Bold Red**

```
  1 MAVSESQLKK MVSKYKYRDL TVRETVNVIT LYKDLKPVLD SYVFNDGSSR
 51 ELMNLTGTIP VPYRGNTYNI PICLWLLDTY PYNPPICFVK PTSSMTIKTG
101 KHVDANGKIY LPYLHEWKHP QSDLLGLIQV MIVVFGDEPP VFSRPISASY
151 PPYQATGPPN TSYMPGMPGG ISPYPSGYPP NPSGYPGCPY PPGGPYPATT
201 SSQYPSQPPV TTVGPSRDGT ISEDTIRASL ISAVSDKLRW RMKEEMDRAQ
251 AELNALKRTE EDLKKGHQKL EEMVTRLDQE VAEVDKNIEL LKKKDEELSS
301 ALEKMENQSE NNDIDEVIIP TAPLYKQILN LYAEENAIED TIFYLGEALR
351 RGVIDLDVFL KHVRLLSRKQ FQLRALMQKA RKTAGLSDLY
```

Residue Number Increasing Mass Decreasing Mass

Start - End	Observed	Mr(expt)	Mr(calc)	Delta	Miss	Sequence	
34 - 50	1911.9202	1910.9129	1910.9373	-0.0244	0	DLKPVLDSYVFNDGSSR	(No match)
34 - 50	1911.9202	1910.9129	1910.9373	-0.0244	0	DLKPVLDSYVFNDGSSR	(Ions score 78)

51 - 64	1619.8214	1618.8141	1618.8388	-0.0247	0	**ELMNLTGTIPVPYR** Oxidation (M) (No match)
51 - 64	1619.8214	1618.8141	1618.8388	-0.0247	0	**ELMNLTGTIPVPYR** Oxidation (M) (Ions score 9)
369 - 374	819.4792	818.4719	818.4762	-0.0043	1	**KQFQLR** (No match)

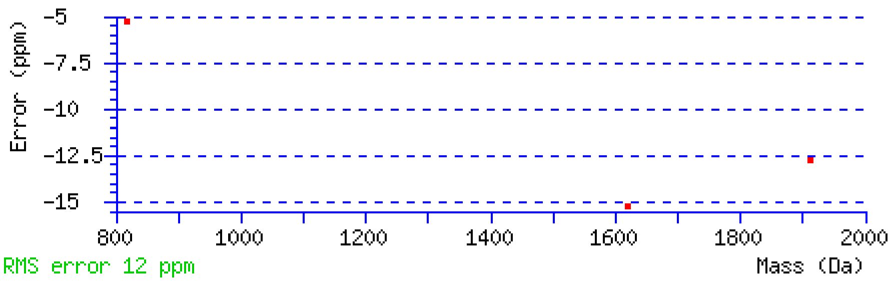

RMS error 12 ppm

Spot 471

Protein View

Match to: **gi|4758078** Score: **482** Expect: **1.2e-043**
c-src tyrosine kinase [Homo sapiens]

Nominal mass (M_r): **51242;** Calculated pI value: **6.62**
NCBI BLAST search of gi|4758078 against nr
Unformatted sequence string for pasting into other applications

Taxonomy: Homo sapiens
Links to retrieve other entries containing this sequence from NCBI Entrez:
gi|729887 from Homo sapiens
gi|30256 from Homo sapiens
gi|30315 from Homo sapiens
gi|6077093 from Homo sapiens
gi|49456875 from Homo sapiens
gi|66841739 from Homo sapiens
gi|77415510 from Homo sapiens
gi|85396953 from Homo sapiens
gi|85397984 from Homo sapiens
gi|119619717 from Homo sapiens
gi|119619718 from Homo sapiens

Fixed modifications: Carbamidomethyl (C)
Variable modifications: Oxidation (M)
Cleavage by Trypsin: cuts C-term side of KR unless next residue is P
Sequence Coverage: **43%**

Matched peptides shown in **Bold Red**

1 MSAIQAAWPS GTECIAKYNF HGTAEQDLPF CKGDVLTIVA VTKDPNWYKA
51 KNKVGR**EGII PANYVQK**REG VKAGTK**LSLM PWFHGK**ITRE QAER**LLYPPE**
101 **TGLFLVREST NYPGDYTLCV SCDGKVEHYR** IMYHASKLSI DEEVYFENLM
151 QLVEHYTSDA DGLCTRLIKP K**VMEGTVAAQ DEFYR**SGWAL NMKELKLLQT
201 IGK**GEFGDVM LGDYR**GNKVA VK**CIKNDATA QAFLAEASVM TQLRHSNLVQ**
251 **LLGVIVEEKG GLYIVTEYMA KGSLVDYLR**S RGRSVLGGDC LLK**FSLDVCE**
301 **AMEYLEGNNF VHR**DLAARNV LVSEDNVAKV SDFGLTKEAS STQDTGKLPV
351 K**WTAPEALR**E KKFSTKSDVW SFGILLWEIY SFGRVPYPR**I PLKDVVPR**VE
401 KGYKMDAPDG CPPAVYEVMK **NCWHLDAAMR PSFLQLR**EQL EHIKTHELHL
451

Residue Number Increasing Mass Decreasing Mass

Start - End	Observed	Mr(expt)	Mr(calc)	Delta	Miss	Sequence
57 - 67	1231.6453	1230.6380	1230.6608	-0.0227	0	EGIIPANYVQK (Ions score 22)
77 - 86	1215.6296	1214.6223	1214.6270	-0.0047	0	LSLMPWFHGK (No match)
77 - 86	1231.6453	1230.6380	1230.6219	0.0161	0	LSLMPWFHGK Oxidation (M) (No match)
95 - 107	1517.8726	1516.8653	1516.8653	0.0000	0	LLYPPETGLFLVR (No match)
95 - 107	1517.8726	1516.8653	1516.8653	0.0000	0	LLYPPETGLFLVR (Ions score 81)
108 - 130	2750.1687	2749.1614	2749.1748	-0.0133	1	ESTNYPGDYTLCVSCDGKVEHYR (No match)
172 - 185	1615.7456	1614.7383	1614.7347	0.0036	0	VMEGTVAAQDEFYR (No match)
172 - 185	1631.7311	1630.7238	1630.7296	-0.0058	0	VMEGTVAAQDEFYR Oxidation (M) (No match)
204 - 215	1358.6083	1357.6010	1357.5972	0.0038	0	GEFGDVMLGDYR (No match)
204 - 215	1374.5973	1373.5900	1373.5921	-0.0021	0	GEFGDVMLGDYR Oxidation (M) (No match)
223 - 244	2438.2009	2437.1936	2437.2093	-0.0157	1	CIKNDATAQAFLAEASVMTQLR (No match)
226 - 244	2037.0049	2035.9976	2035.9996	-0.0020	0	NDATAQAFLAEASVMTQLR (No match)
226 - 244	2037.0049	2035.9976	2035.9996	-0.0020	0	NDATAQAFLAEASVMTQLR (Ions score 71)
226 - 244	2053.0022	2051.9949	2051.9945	0.0004	0	NDATAQAFLAEASVMTQLR Oxidation (M) (No match)
245 - 259	1677.9543	1676.9470	1676.9460	0.0010	0	HSNLVQLLGVIVEEK (No match)
260 - 271	1344.6844	1343.6771	1343.6795	-0.0023	0	GGLYIVTEYMAK (No match)
272 - 279	922.4949	921.4876	921.4919	-0.0043	0	GSLVDYLR (No match)
294 - 313	2430.0754	2429.0681	2429.0779	-0.0098	0	FSLDVCEAMEYLEGNNFVHR (Ions score 102)
294 - 313	2430.0754	2429.0681	2429.0779	-0.0098	0	FSLDVCEAMEYLEGNNFVHR (No match)
294 - 313	2446.0828	2445.0755	2445.0728	0.0027	0	FSLDVCEAMEYLEGNNFVHR Oxidation (M) (No match)
352 - 359	943.4987	942.4914	942.4923	-0.0009	0	WTAPEALR (No match)
390 - 398	1036.6522	1035.6449	1035.6440	0.0009	1	IPLKDVVPR (Ions score 40)
390 - 398	1036.6522	1035.6449	1035.6440	0.0009	1	IPLKDVVPR (No match)
421 - 437	2115.0383	2114.0310	2114.0302	0.0008	0	NCWHLDAAMRPSFLQLR (No match)

Spot 472

Protein View

Match to: **gi|13375746** Score: **66** Expect: **0.048**
hypothetical protein LOC79624 [Homo sapiens]

Nominal mass (M_r): **51539**; Calculated pI value: **5.48**
NCBI BLAST search of gi|13375746 against nr
Unformatted sequence string for pasting into other applications

Taxonomy: Homo sapiens
Links to retrieve other entries containing this sequence from NCBI Entrez:
gi|74752737 from Homo sapiens
gi|10434672 from Homo sapiens
gi|55859570 from Homo sapiens
gi|119568130 from Homo sapiens

Fixed modifications: Carbamidomethyl (C)
Variable modifications: Oxidation (M)
Cleavage by Trypsin: cuts C-term side of KR unless next residue is P
Sequence Coverage: **12%**

Matched peptides shown in **Bold Red**

```
  1 MAVVPASLSG QDVGSFAYLT IKDRIPQILT KVIDTLHRHK SEFFEKHGEE
 51 GVEAEKKAIS LLSKLRNELQ TDKPFIPLVE KFVDTDIWNQ YLEYQQSLLN
101 ESDGKSRWFY SPWLLVECYM YRRIHEAIIQ SPPIDYFDVF KESKEQNFYG
151 SQESIIALCT HLQQLIRTIE DLDENQLKDE FFKLLQISLW GNKCDLSLSG
201 GESSSQNTNV LNSLEDLKPF ILLNDMEHLW SLLSNCKKTR EKASATRVYI
251 VLDNSGFELV TDLILADFLL SSELATEVHF YGKTIPWFVS DTTIHDFNWL
301 IEQVKHSNHK WMSKCGADWE EYIKMGKWVY HNHIFWTLPH EYCAMPQVAP
351 DLYAELQKAH LILFKGDLNY RKLTGDRKWE FSVPFHQALN GFHPAPLCTI
401 RTLKAEIQVG LQPGQGEQLL ASEPSWWTTG KYGIFQYDGP L
```

Residue Number Increasing Mass Decreasing Mass

Start - End	Observed	Mr(expt)	Mr(calc)	Delta	Miss	Sequence
65 - 81	2040.1118	2039.1045	2039.1414	-0.0369	1	LRNELQTDKPFIPLVEK (No match)
65 - 81	2040.1118	2039.1045	2039.1414	-0.0369	1	LRNELQTDKPFIPLVEK (Ions score 24)
145 - 167	2748.3142	2747.3069	2747.3700	-0.0631	0	EQNFYGSQESIIALCTHLQQLIR (No match)

145 - 167	2748.3142	2747.3069	2747.3700	-0.0631	0	EQNFYGSQESIIALCTHLQQLIR (Ions score 14)
168 - 183	1983.9133	1982.9060	1982.9472	-0.0412	1	TIEDLDENQLKDEFFK (No match)

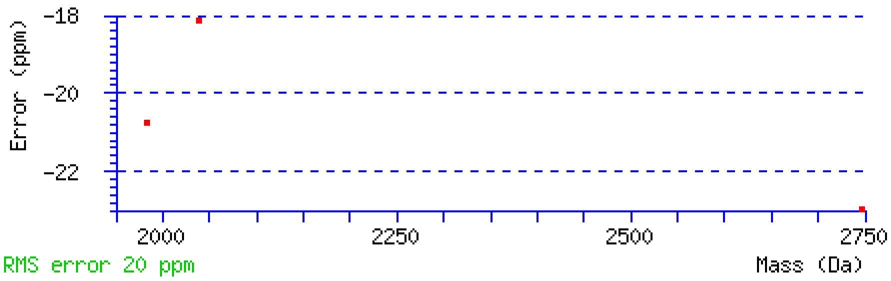

Spot 473

Protein View

Match to: **gi|4759140** Score: **391** Expect: **1.5e-034**
solute carrier family 9 (sodium/hydrogen exchanger), isoform 3 regulator 1 [Homo sapiens]

Nominal mass (M_r): **39130;** Calculated pI value: **5.55**
NCBI BLAST search of gi|4759140 against nr
Unformatted sequence string for pasting into other applications

Taxonomy: Homo sapiens
Links to retrieve other entries containing this sequence from NCBI Entrez:
gi|41688557 from Homo sapiens
gi|2920585 from Homo sapiens
gi|3220019 from Homo sapiens
gi|12655175 from Homo sapiens
gi|13097186 from Homo sapiens
gi|15079985 from Homo sapiens
gi|31419627 from Homo sapiens
gi|119609596 from Homo sapiens

Fixed modifications: Carbamidomethyl (C)
Variable modifications: Oxidation (M)
Cleavage by Trypsin: cuts C-term side of KR unless next residue is P
Sequence Coverage: **40%**

Matched peptides shown in **Bold Red**

```
  1 MSADAAAGAP LPRLCCLEKG PNGYGFHLHG EKGKLGQYIR LVEPGSPAEK
 51 AGLLAGDRLV EVNGENVEKE THQQVVSRIR AALNAVRLLV VDPETDEQLQ
101 KLGVQVREEL LRAQEAPGQA EPPAAAEVQG AGNENEPREA DKSHPEQREL
151 RPRLCTMKKG PSGYGFNLHS DKSKPGQFIR SVDPDSPAEA SGLRAQDRIV
201 EVNGVCMEGK QHGDVVSAIR AGGDETKLLV VDRETDEFFK KCRVIPSQEH
251 LNGPLPVPFT NGEIQKENSR EALAEAALES PRPALVRSAS SDTSEELNSQ
301 DSPPKQDSTA PSSTSSSDPI LDFNISLAMA KERAHQKRSS KRAPQMDWSK
351 KNELFSNL
```

Residue Number Increasing Mass Decreasing Mass

Start - End Observed Mr(expt) Mr(calc) Delta Miss Sequence

20 - 32	1412.6522	1411.6449	1411.6632	-0.0183	0	GPNGYGFHLHGEK (No match)
20 - 32	1412.6522	1411.6449	1411.6632	-0.0183	0	GPNGYGFHLHGEK (Ions score 53)
59 - 78	2294.1384	2293.1311	2293.1661	-0.0350	1	LVEVNGENVEKETHQQVVSR (No match)
70 - 78	1083.5475	1082.5402	1082.5468	-0.0066	0	ETHQQVVSR (No match)
88 - 101	1626.8315	1625.8242	1625.8512	-0.0269	0	LLVVDPETDEQLQK (No match)
113 - 138	2588.1641	2587.1568	2587.1898	-0.0329	0	AQEAPGQAEPPAAAEVQGAGNENEPR (No match)
113 - 138	2588.1641	2587.1568	2587.1898	-0.0329	0	AQEAPGQAEPPAAAEVQGAGNENEPR (Ions score 111)
139 - 148	1196.5540	1195.5467	1195.5581	-0.0114	1	EADKSHPEQR (Ions score 39)
139 - 148	1196.5540	1195.5467	1195.5581	-0.0114	1	EADKSHPEQR (No match)
159 - 172	1506.7142	1505.7069	1505.7262	-0.0193	1	KGPSGYGFNLHSDK (No match)
173 - 180	932.5246	931.5173	931.5239	-0.0066	0	SKPGQFIR (No match)
181 - 194	1400.6437	1399.6364	1399.6579	-0.0215	0	SVDPDSPAEASGLR (No match)
211 - 220	1081.5603	1080.5530	1080.5676	-0.0145	0	QHGDVVSAIR (No match)
271 - 287	1792.9637	1791.9564	1791.9842	-0.0278	0	EALAEAALESPRPALVR (No match)
271 - 287	1792.9637	1791.9564	1791.9842	-0.0278	0	EALAEAALESPRPALVR (Ions score 60)

Spot 474

Protein View

Match to: **gi|49168546** Score: **208** Expect: **3e-016**
LPXN [Homo sapiens]

Nominal mass (M_r): **44619**; Calculated pI value: **5.62**
NCBI BLAST search of gi|49168546 against nr
Unformatted sequence string for pasting into other applications

Taxonomy: Homo sapiens

Fixed modifications: Carbamidomethyl (C)
Variable modifications: Oxidation (M)
Cleavage by Trypsin: cuts C-term side of KR unless next residue is P
Sequence Coverage: **16%**

Matched peptides shown in **Bold Red**

```
  1 MDELDALLEE LERSTLQDSD EYSNPAPLPL DQHSRKETNL DETSEILSIQ
 51 DNTSPLPAQL VYTTNIQELN VYSEAQEPKE SPPPSKTSAA AQLDELMAHM
101 TEMQAKVAVR ADAGKKHLPD KQDHKASLDS MLGGLEQELQ DLGIATVPKG
151 HCASCQKPIA GKVIHALGQS WHPEHFVCTH CKEEIGSSPF FERSGLAYCP
201 NDYHQLFSPR CAYCAAPILD KVLTAMNQTW HPEHFFCSHC GEVFGAEGFH
251 EKDKKPYCRK DFLAMFSPKC GGCNRPVLEN YLSAMDTVWH PECFVCGDCF
301 TSFSTGSFFE LDGRPFCELH YHHRRGTLCH GCGQPITGRC ISAMGYKFHP
351 EHFVCAFCLT QLSKGIFREQ NDKTYCQPCF NKLFPL
```

Residue Number Increasing Mass Decreasing Mass

Start - End	Observed	Mr(expt)	Mr(calc)	Delta	Miss	Sequence
14 - 35	2470.0425	2469.0352	2469.1408	-0.1056	0	STLQDSDEYSNPAPLPLDQHSR (Ions score 72)
14 - 35	2470.0425	2469.0352	2469.1408	-0.1056	0	STLQDSDEYSNPAPLPLDQHSR (No match)
183 - 193	1297.5491	1296.5418	1296.5986	-0.0567	0	EEIGSSPFFER (No match)
183 - 193	1297.5491	1296.5418	1296.5986	-0.0567	0	EEIGSSPFFER (Ions score 27)
194 - 210	2024.8417	2023.8344	2023.9210	-0.0866	0	SGLAYCPNDYHQLFSPR (No match)
194 - 210	2024.8417	2023.8344	2023.9210	-0.0866	0	SGLAYCPNDYHQLFSPR (Ions score 54)
326 - 339	1513.6353	1512.6280	1512.6925	-0.0645	0	GTLCHGCGQPITGR (Ions score 5)
326 - 339	1513.6353	1512.6280	1512.6925	-0.0645	0	GTLCHGCGQPITGR (No match)

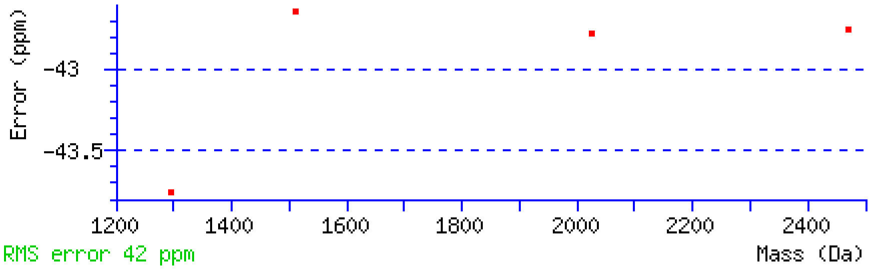
Error (ppm)
-43
-43.5
1200
1400
1600
1800
2000
2200
2400
RMS error 42 ppm
Mass (Da)

Spot 475

Protein View

Match to: **gi|4758078** Score: **647** Expect: **3.8e-060**
c-src tyrosine kinase [Homo sapiens]

Nominal mass (M_r): **51242;** Calculated pI value: **6.62**
NCBI BLAST search of gi|4758078 against nr
Unformatted sequence string for pasting into other applications

Taxonomy: Homo sapiens
Links to retrieve other entries containing this sequence from NCBI Entrez:
gi|729887 from Homo sapiens
gi|30256 from Homo sapiens
gi|30315 from Homo sapiens
gi|6077093 from Homo sapiens
gi|49456875 from Homo sapiens
gi|66841739 from Homo sapiens
gi|77415510 from Homo sapiens
gi|85396953 from Homo sapiens
gi|85397984 from Homo sapiens
gi|119619717 from Homo sapiens
gi|119619718 from Homo sapiens

Fixed modifications: Carbamidomethyl (C)
Variable modifications: Oxidation (M)
Cleavage by Trypsin: cuts C-term side of KR unless next residue is P
Sequence Coverage: **45%**

Matched peptides shown in **Bold Red**

1 MSAIQAAWPS GTECIAKYNF HGTAEQDLPF CKGDVLTIVA VTKDPNWYKA
51 KNKVGR**EGII PANYVQKR**EG VKAGTK**LSLM PWFHGK**ITRE QAER**LLYPPE**
101 **TGLFLVR**EST NYPGDYTLCV SCDGKVEHYR IMYHASKLSI DEEVYFENLM
151 QLVEHYTSDA DGLCTRLIKP K**VMEGTVAAQ DEFYRSGWAL NMK**ELK**LLQT**
201 **IGKGEFGDVM LGDYR**GNKVA VK**CIKNDATA QAFLAEASVM TQLRHSNLVQ**
251 **LLGVIVEEKG GLYIVTEYMA KGSLVDYLR**S RGRSVLGGDC LLK**FSLDVCE**
301 **AMEYLEGNNF VHR**DLAARNV LVSEDNVAKV SDFGLTKEAS STQDTGKLPV
351 K**WTAPEALRE K**KFSTKSDVW SFGILLWEIY SFGRVPYPR**I PLKDVVPR**VE
401 KGYK**MDAPDG CPPAVYEVMK NCWHLDAAMR PSFLQLR**EQL EHIKTHELHL
451

Residue Number Increasing Mass Decreasing Mass

Start - End	Observed	Mr(expt)	Mr(calc)	Delta	Miss	Sequence
57 - 67	1231.6548	1230.6475	1230.6608	-0.0132	0	EGIIPANYVQK (No match)
57 - 68	1387.7753	1386.7680	1386.7619	0.0062	1	EGIIPANYVQKR (No match)
77 - 86	1215.6431	1214.6358	1214.6270	0.0088	0	LSLMPWFHGK (No match)
95 - 107	1517.8893	1516.8820	1516.8653	0.0167	0	LLYPPETGLFLVR (Ions score 79)
95 - 107	1517.8893	1516.8820	1516.8653	0.0167	0	LLYPPETGLFLVR (No match)
172 - 185	1615.7603	1614.7530	1614.7347	0.0183	0	VMEGTVAAQDEFYR (Ions score 80)
172 - 185	1615.7603	1614.7530	1614.7347	0.0183	0	VMEGTVAAQDEFYR (No match)
172 - 185	1631.7588	1630.7515	1630.7296	0.0219	0	VMEGTVAAQDEFYR Oxidation (M) (No match)
186 - 193	906.4496	905.4423	905.4429	-0.0006	0	SGWALNMK (No match)
197 - 215	2112.0942	2111.0869	2111.0720	0.0149	1	LLQTIGKGEFGDVMLGDYR (No match)
204 - 215	1358.6201	1357.6128	1357.5972	0.0156	0	GEFGDVMLGDYR (No match)
204 - 215	1374.6090	1373.6017	1373.5921	0.0096	0	GEFGDVMLGDYR Oxidation (M) (No match)
223 - 244	2438.2351	2437.2278	2437.2093	0.0185	1	CIKNDATAQAFLAEASVMTQLR (No match)
226 - 244	2037.0339	2036.0266	2035.9996	0.0270	0	NDATAQAFLAEASVMTQLR (Ions score 99)
226 - 244	2037.0339	2036.0266	2035.9996	0.0270	0	NDATAQAFLAEASVMTQLR (No match)
245 - 259	1677.9718	1676.9645	1676.9460	0.0185	0	HSNLVQLLGVIVEEK (No match)
260 - 271	1344.6949	1343.6876	1343.6795	0.0082	0	GGLYIVTEYMAK (No match)
272 - 279	922.5016	921.4943	921.4919	0.0024	0	GSLVDYLR (No match)
294 - 313	2430.1025	2429.0952	2429.0779	0.0173	0	FSLDVCEAMEYLEGNNFVHR (No match)
294 - 313	2430.1025	2429.0952	2429.0779	0.0173	0	FSLDVCEAMEYLEGNNFVHR (Ions score 101)
352 - 359	943.5010	942.4937	942.4923	0.0014	0	WTAPEALR (No match)
352 - 361	1200.6494	1199.6421	1199.6298	0.0123	1	WTAPEALREK (No match)
390 - 398	1036.6591	1035.6518	1035.6440	0.0078	1	IPLKDVVPR (Ions score 53)
390 - 398	1036.6591	1035.6518	1035.6440	0.0078	1	IPLKDVVPR (No match)
405 - 420	1779.8026	1778.7953	1778.7677	0.0276	0	MDAPDGCPPAVYEVMK (No match)
421 - 437	2115.0676	2114.0603	2114.0302	0.0301	0	NCWHLDAAMRPSFLQLR (No match)

Spot 476

Protein View

Match to: **gi|182118** Score: **294** Expect: **7.7e-025**
gamma enolase

Nominal mass (M_r): **44568;** Calculated pI value: **4.94**
NCBI BLAST search of gi|182118 against nr
Unformatted sequence string for pasting into other applications

Taxonomy: Homo sapiens

Fixed modifications: Carbamidomethyl (C)
Variable modifications: Oxidation (M)
Cleavage by Trypsin: cuts C-term side of KR unless next residue is P
Sequence Coverage: **21%**

Matched peptides shown in **Bold Red**

```
  1 GCGLFRAAVP SGASTGIYEA LELRDGDKQR YLGKGVLKAV DHINSTIAPA
 51 LISSGLSVVE QEKLDNLMLE LDGTENKSKF GANAILGVSL AVCKAGAAER
101 NLPLYRHIAQ LAGNSDLILP VPAFNVINGG SHAGNKLAMQ EFMILPVGAE
151 SFRDAMRLGA EVYHTLKGVI KDKYGKDATN VGDEGGFAPN ILENSEALEL
201 VKEAIDKAGY TEKIVIGMDV AASEFYRDGK YDLDFKSPTD PSRYITGDQL
251 GALYQDFVRD YPVVSIEDPF DQDDWAAWSK FTANVGIQIV GDDLTVTNPK
301 RIERAVEEKA CNCLLLKVNQ IGSVTEAIQA CKLAQENGWG VMVSHRSGET
351 EDTFIADLVV GLCTGQIKTG APCRSERLAK YNQLMRIEEE LGDEARFAGH
401 NFRNPSVL
```

Residue Number Increasing Mass Decreasing Mass

Start - End	Observed	Mr(expt)	Mr(calc)	Delta	Miss	Sequence
7 - 24	1804.9386	1803.9313	1803.9366	-0.0053	0	AAVPSGASTGIYEALELR (No match)
7 - 24	1804.9386	1803.9313	1803.9366	-0.0053	0	AAVPSGASTGIYEALELR (Ions score 92)
137 - 153	1970.9679	1969.9606	1969.9641	-0.0034	0	LAMQEFMILPVGAESFR 2 Oxidation (M) (No match)
137 - 153	1970.9679	1969.9606	1969.9641	-0.0034	0	LAMQEFMILPVGAESFR 2 Oxidation (M) (Ions score 30)
154 - 167	1603.7573	1602.7500	1602.8188	-0.0687	1	DAMRLGAEVYHTLK (No match)
154 - 167	1603.7573	1602.7500	1602.8188	-0.0687	1	DAMRLGAEVYHTLK (No match)
214 - 227	1586.7784	1585.7711	1585.7809	-0.0098	0	IVIGMDVAASEFYR Oxidation (M) (No match)
214 - 227	1586.7784	1585.7711	1585.7809	-0.0098	0	IVIGMDVAASEFYR Oxidation (M) (Ions score 4)

244 - 259	1858.9270	1857.9197	1857.9260	-0.0063	0	**YITGDQLGALYQDFVR**	(No match)
244 - 259	1858.9270	1857.9197	1857.9260	-0.0063	0	**YITGDQLGALYQDFVR**	(Ions score 99)
397 - 403	848.4137	847.4064	847.4089	-0.0024	0	**FAGHNFR**	(No match)

Spot 477

Protein View

Match to: **gi|5031699** Score: **296** Expect: **4.8e-025**
flotillin 1 [Homo sapiens]

Nominal mass (M_r): **47554;** Calculated pI value: **7.08**
NCBI BLAST search of gi|5031699 against nr
Unformatted sequence string for pasting into other applications

Taxonomy: Homo sapiens
Links to retrieve other entries containing this sequence from NCBI Entrez:
gi|26006960 from Homo sapiens
gi|3599573 from Homo sapiens
gi|12654619 from Homo sapiens
gi|15277227 from Homo sapiens
gi|27544399 from Homo sapiens
gi|30582993 from Homo sapiens
gi|55961565 from Homo sapiens
gi|55961676 from Homo sapiens
gi|57209816 from Homo sapiens
gi|60655509 from synthetic construct
gi|60655511 from synthetic construct
gi|86197962 from Homo sapiens
gi|114306780 from Homo sapiens
gi|119623731 from Homo sapiens
gi|119623732 from Homo sapiens
gi|123293910 from Homo sapiens
gi|123994279 from synthetic construct
gi|124126967 from synthetic construct

Fixed modifications: Carbamidomethyl (C)
Variable modifications: Oxidation (M)
Cleavage by Trypsin: cuts C-term side of KR unless next residue is P
Sequence Coverage: **22%**

Matched peptides shown in **Bold Red**

```
  1 MFFTCGPNEA MVVSGFCRSP PVMVAGGRVF VLPCIQQIQR ISLNTLTLNV
 51 KSEKVYTRHG VPISVTGIAQ VKIQGQNKEM LAAACQMFLG KTEAEIAHIA
101 LETLEGHQRA IMAHMTVEEI YKDRQKFSEQ VFKVASSDLV NMGISVVSYT
151 LKDIHDDQDY LHSLGKARTA QVQKDARIGE AEAKRDAGIR EAKAKQEKVS
201 AQYLSEIEMA KAQRDYELKK AAYDIEVNTR RAQADLAYQL QVAKTKQQIE
251 EQRVQVQVVE RAQQVAVQEQ EIARREKELE ARVRKPAEAE RYKLERLAEA
```

```
301 EKSQLIMQAE AEAASVRMRG EAEAFAIGAR ARAEAEQMAK KAEAFQLYQE
351 AAQLDMLLEK LPQVAEEISG PLTSANKITL VSSGSGTMGA AKVTGEVLDI
401 LTRLPESVER LTGVSISQVN HKPLRTA
```

Residue Number Increasing Mass Decreasing Mass

Start - End	Observed	Mr(expt)	Mr(calc)	Delta	Miss	Sequence
29 - 40	1500.8492	1499.8419	1499.8282	0.0137	0	VFVLPCIQQIQR (No match)
29 - 40	1500.8492	1499.8419	1499.8282	0.0137	0	VFVLPCIQQIQR (Ions score 58)
92 - 109	2018.0538	2017.0465	2017.0228	0.0237	0	TEAEIAHIALETLEGHQR (No match)
92 - 109	2018.0538	2017.0465	2017.0228	0.0237	0	TEAEIAHIALETLEGHQR (Ions score 42)
262 - 274	1469.7828	1468.7755	1468.7633	0.0122	0	AQQVAVQEQEIAR (No match)
262 - 274	1469.7828	1468.7755	1468.7633	0.0122	0	AQQVAVQEQEIAR (Ions score 63)
303 - 317	1619.8208	1618.8135	1618.7984	0.0151	0	SQLIMQAEAEAASVR Oxidation (M) (No match)
318 - 330	1394.6917	1393.6844	1393.6771	0.0073	1	MRGEAEAFAIGAR Oxidation (M) (No match)
393 - 403	1215.7070	1214.6997	1214.6870	0.0127	0	VTGEVLDILTR (No match)
411 - 425	1648.9652	1647.9579	1647.9420	0.0160	0	LTGVSISQVNHKPLR (Ions score 44)
411 - 425	1648.9652	1647.9579	1647.9420	0.0160	0	LTGVSISQVNHKPLR (No match)

Spot 478

Protein View

Match to: **gi|62896593** Score: **469** Expect: **2.4e-042**
enolase 1 variant [Homo sapiens]

Nominal mass (M_r): **47453**; Calculated pI value: **7.01**
NCBI BLAST search of gi|62896593 against nr
Unformatted sequence string for pasting into other applications

Taxonomy: Homo sapiens

Fixed modifications: Carbamidomethyl (C)
Variable modifications: Oxidation (M)
Cleavage by Trypsin: cuts C-term side of KR unless next residue is P
Sequence Coverage: **40%**

Matched peptides shown in **Bold Red**

```
  1 MSILKIHARE IFDSRGNPTV EVDLFTSKGL FRAAVPSGAS TGIYEALELR
 51 DNDKTRYMGK GVSKAVEHIN KTIAPALASK KLNVTEQEKI DKLMIEMDGT
101 ENKSKFGANA ILGVSLAVCK AGAVEKGVPL YRHIADLAGN SEVILPVPAF
151 NVINGGSHAG NKLAMQEFMI LPVGAANFRE AMRIGAEVYH NLKNVIKEKY
201 GKDATNVGDE GGFAPNILEN KEGLELLKTA IGKAGYTDKV VIGMDVAASE
251 FFRSGKYDLD FKSPDDPSRY ISPDQLADLY KSFIKDYPVV SIEDPFDQDD
301 WGAWQKFTAS AGIQVVGDDL TVTNPKRIAK AVNEKSCNCL LLKVNQIGSV
351 TESLQACKLA QANGWGVMVS HRSGETEDTF IADLVVGLCT GQIKTGAPCR
401 SERLAKYNQL LRIEEELGSK AKFAGRNFRN PLAK
```

Residue Number Increasing Mass Decreasing Mass

Start - End	Observed	Mr(expt)	Mr(calc)	Delta	Miss	Sequence
16 - 28	1406.7117	1405.7044	1405.7088	-0.0044	0	GNPTVEVDLFTSK (No match)
33 - 50	1804.9362	1803.9289	1803.9366	-0.0077	0	AAVPSGASTGIYEALELR (Ions score 65)
33 - 50	1804.9362	1803.9289	1803.9366	-0.0077	0	AAVPSGASTGIYEALELR (No match)
163 - 179	1907.9795	1906.9722	1906.9797	-0.0075	0	LAMQEFMILPVGAANFR (No match)
163 - 179	1907.9795	1906.9722	1906.9797	-0.0075	0	LAMQEFMILPVGAANFR (Ions score 82)
163 - 179	1923.9746	1922.9673	1922.9746	-0.0073	0	LAMQEFMILPVGAANFR Oxidation (M) (No match)
163 - 179	1939.9618	1938.9545	1938.9695	-0.0150	0	LAMQEFMILPVGAANFR 2 Oxidation (M) (No match)
184 - 193	1143.6068	1142.5995	1142.6083	-0.0088	0	IGAEVYHNLK (No match)

234 - 253	2176.0576	2175.0503	2175.0669	-0.0166	1	**AGYTDKVVIGMDVAASEFFR** (Ions score 85)
234 - 253	2176.0576	2175.0503	2175.0669	-0.0166	1	**AGYTDKVVIGMDVAASEFFR** (No match)
240 - 253	1540.7704	1539.7631	1539.7755	-0.0123	0	**VVIGMDVAASEFFR** (No match)
240 - 253	1540.7704	1539.7631	1539.7755	-0.0123	0	**VVIGMDVAASEFFR** (Ions score 24)
270 - 281	1425.7218	1424.7145	1424.7187	-0.0042	0	**YISPDQLADLYK** (No match)
307 - 326	2033.0442	2032.0369	2032.0476	-0.0107	0	**FTASAGIQVVGDDLTVTNPK** (No match)
344 - 358	1633.8169	1632.8096	1632.8141	-0.0044	0	**VNQIGSVTESLQACK** (No match)
359 - 372	1525.7594	1524.7521	1524.7619	-0.0098	0	**LAQANGWGVMVSHR** (No match)
359 - 372	1525.7594	1524.7521	1524.7619	-0.0098	0	**LAQANGWGVMVSHR** (Ions score 45)
359 - 372	1541.7717	1540.7644	1540.7568	0.0076	0	**LAQANGWGVMVSHR** Oxidation (M) (No match)
373 - 394	2353.1453	2352.1380	2352.1518	-0.0138	0	**SGETEDTFIADLVVGLCTGQIK** (No match)
407 - 412	806.4479	805.4406	805.4446	-0.0040	0	**YNQLLR** (No match)
427 - 434	959.5338	958.5265	958.5348	-0.0083	1	**NFRNPLAK** (No match)

Spot 479

Protein View

Match to: **gi|62896593** Score: **176** Expect: **4.8e-013**
enolase 1 variant [Homo sapiens]

Nominal mass (M_r): **47453;** Calculated pI value: **7.01**
NCBI BLAST search of gi|62896593 against nr
Unformatted sequence string for pasting into other applications

Taxonomy: Homo sapiens

Fixed modifications: Carbamidomethyl (C)
Variable modifications: Oxidation (M)
Cleavage by Trypsin: cuts C-term side of KR unless next residue is P
Sequence Coverage: **34%**

Matched peptides shown in **Bold Red**

```
  1 MSILKIHARE IFDSRGNPTV EVDLFTSKGL FRAAVPSGAS TGIYEALELR
 51 DNDKTRYMGK GVSKAVEHIN KTIAPALASK KLNVTEQEKI DKLMIEMDGT
101 ENKSKFGANA ILGVSLAVCK AGAVEKGVPL YRHIADLAGN SEVILPVPAF
151 NVINGGSHAG NKLAMQEFMI LPVGAANFRE AMRIGAEVYH NLKNVIKEKY
201 GKDATNVGDE GGFAPNILEN KEGLELLKTA IGKAGYTDKV VIGMDVAASE
251 FFRSGKYDLD FKSPDDPSRY ISPDQLADLY KSFIKDYPVV SIEDPFDQDD
301 WGAWQKFTAS AGIQVVGDDL TVTNPKRIAK AVNEKSCNCL LLKVNQIGSV
351 TESLQACKLA QANGWGVMVS HRSGETEDTF IADLVVGLCT GQIKTGAPCR
401 SERLAKYNQL LRIEEELGSK AKFAGRNFRN PLAK
```

Residue Number Increasing Mass Decreasing Mass

Start - End	Observed	Mr(expt)	Mr(calc)	Delta	Miss	Sequence
33 - 50	1804.8630	1803.8557	1803.9366	-0.0809	0	AAVPSGASTGIYEALELR (Ions score 70)
33 - 50	1804.8630	1803.8557	1803.9366	-0.0809	0	AAVPSGASTGIYEALELR (No match)
93 - 105	1527.7643	1526.7570	1526.6956	0.0614	1	LMIEMDGTENKSK 2 Oxidation (M) (No match)
163 - 179	1939.8905	1938.8832	1938.9695	-0.0863	0	LAMQEFMILPVGAANFR 2 Oxidation (M) (Ions score 10)
163 - 179	1939.8905	1938.8832	1938.9695	-0.0863	0	LAMQEFMILPVGAANFR 2 Oxidation (M) (No match)
184 - 193	1143.5653	1142.5580	1142.6083	-0.0503	0	IGAEVYHNLK (No match)
203 - 228	2743.2493	2742.2420	2742.3711	-0.1290	1	DATNVGDEGGFAPNILENKEGLELLK (No match)
234 - 253	2191.9885	2190.9812	2191.0618	-0.0806	1	AGYTDKVVIGMDVAASEFFR Oxidation (M) (No match)

240 - 253	1556.7096	1555.7023	1555.7704	-0.0680	0	**VVIGMDVAASEFFR**	Oxidation (M) (No match)
240 - 253	1556.7096	1555.7023	1555.7704	-0.0680	0	**VVIGMDVAASEFFR**	Oxidation (M) (Ions score 2)
270 - 281	1425.6625	1424.6552	1424.7187	-0.0635	0	**YISPDQLADLYK**	(No match)
307 - 326	2032.9681	2031.9608	2032.0476	-0.0868	0	**FTASAGIQVVGDDLTVTNPK**	(No match)
407 - 412	806.4189	805.4116	805.4446	-0.0330	0	**YNQLLR**	(No match)
427 - 434	959.5029	958.4956	958.5348	-0.0392	1	**NFRNPLAK**	(No match)

Spot 480

Protein View

Match to: **gi|67464043** Score: **507** Expect: **3.8e-046**
Chain O, Crsytal Structure Of Human Liver Gapdh

Nominal mass (M_r): **36483;** Calculated pI value: **8.58**
NCBI BLAST search of gi|67464043 against nr
Unformatted sequence string for pasting into other applications

Taxonomy: Homo sapiens
Links to retrieve other entries containing this sequence from NCBI Entrez:
gi|67464044 from Homo sapiens
gi|67464045 from Homo sapiens
gi|67464046 from Homo sapiens

Fixed modifications: Carbamidomethyl (C)
Variable modifications: Oxidation (M)
Cleavage by Trypsin: cuts C-term side of KR unless next residue is P
Sequence Coverage: **27%**

Matched peptides shown in **Bold Red**

```
  1 GSHMGKVKVG VNGFGRIGRL VTRAAFNSGK VDIVAINDPF IDLNYMVYMF
 51 QYDSTHGKFH GTVKAENGKL VINGNPITIF QERDPSKIKW GDAGAEYVVE
101 STGVFTTMEK AGAHLQGGAK RVIISAPSAD APMFVMGVNH EKYDNSLKII
151 SNASCTTNCL APLAKVIHDN FGIVEGLMTT VHAITATQKT VDGPSGKLWR
201 DGRGALQNII PASTGAAKAV GKVIPELNGK LTGMAFRVPT ANVSVVDLTC
251 RLEKPAKYDD IKKVVKQASE GPLKGILGYT EHQVVSSDFN SDTHSSTFDA
301 GAGIALNDHF VKLISWYDNE FGYSNRVVDL MAHMASKE
```

Residue Number Increasing Mass Decreasing Mass

Start - End	Observed	Mr(expt)	Mr(calc)	Delta	Miss	Sequence
9 - 16	805.4379	804.4306	804.4241	0.0065	0	VGVNGFGR (No match)
70 - 83	1613.9177	1612.9104	1612.8936	0.0168	0	LVINGNPITIFQER (Ions score 70)
70 - 83	1613.9177	1612.9104	1612.8936	0.0168	0	LVINGNPITIFQER (No match)
70 - 87	2041.1256	2040.1183	2040.1003	0.0180	1	LVINGNPITIFQERDPSK (Ions score 59)
70 - 87	2041.1256	2040.1183	2040.1003	0.0180	1	LVINGNPITIFQERDPSK (No match)
149 - 165	1833.9360	1832.9287	1832.9124	0.0163	0	IISNASCTTNCLAPLAK (Ions score 108)

149 - 165	1833.9360	1832.9287	1832.9124	0.0163	0	**IISNASCTTNCLAPLAK**	(No match)
204 - 218	1411.8003	1410.7930	1410.7830	0.0100	0	**GALQNIIPASTGAAK**	(No match)
231 - 237	811.4191	810.4118	810.4058	0.0061	0	**LTGMAFR** Oxidation (M)	(No match)
238 - 251	1530.8109	1529.8036	1529.7871	0.0165	0	**VPTANVSVVDLTCR**	(No match)
238 - 251	1530.8109	1529.8036	1529.7871	0.0165	0	**VPTANVSVVDLTCR**	(Ions score 81)
313 - 326	1763.8225	1762.8152	1762.7950	0.0202	0	**LISWYDNEFGYSNR**	(No match)
313 - 326	1763.8225	1762.8152	1762.7950	0.0202	0	**LISWYDNEFGYSNR**	(Ions score 83)

Spot 482

Protein View

Match to: **gi|62088934** Score: **139** Expect: **2.4e-009**
CDC10 protein variant [Homo sapiens]

Nominal mass (M_r): **44084;** Calculated pI value: **7.73**
NCBI BLAST search of gi|62088934 against nr
Unformatted sequence string for pasting into other applications

Taxonomy: Homo sapiens

Fixed modifications: Carbamidomethyl (C)
Variable modifications: Oxidation (M)
Cleavage by Trypsin: cuts C-term side of KR unless next residue is P
Sequence Coverage: **13%**

Matched peptides shown in **Bold Red**

```
  1 VNSSTMVAQQ KNLEGYVGFA NLPNQVYRKS VKRGFEFTLM VVGESGLGKS
 51 TLINSLFLTD LYSPEYPGPS HRIKKTVQVE QSKVLIKEGG VQLLLTIVDT
101 PGFGDAVDNS NCWQPVIDYI DSKFEDYLNA ESRVNRRQMP DNRVQCCLYF
151 IAPSGHGLKP LDIEFMKRLH EKVNIIPLIA KADTLTPEEC QQFKKQIMKE
201 IQEHKIKIYE FPETDDEEEN KLVKKIKDRL PLAVVGSNTI IEVNGKRVRG
251 RQYPWGVAEV ENGEHCDFTI LRNMLIRTHM QDLKDVTNNV HYENYRSRKL
301 AAVTYNGVDN NKNKGQLTKS PLAQMEEERR EHVAKMKKME MEMEQVFEMK
351 VKEKVQKLKD SEAEVISLIS HLLADIVFLF K
```

Residue Number Increasing Mass Decreasing Mass

Start - End	Observed	Mr(expt)	Mr(calc)	Delta	Miss	Sequence
12 - 28	1953.9781	1952.9708	1952.9744	-0.0035	0	NLEGYVGFANLPNQVYR (Ions score 39)
12 - 28	1953.9781	1952.9708	1952.9744	-0.0035	0	NLEGYVGFANLPNQVYR (No match)
50 - 72	2607.3010	2606.2937	2606.3016	-0.0079	0	STLINSLFLTDLYSPEYPGPSHR (No match)
50 - 72	2607.3010	2606.2937	2606.3016	-0.0079	0	STLINSLFLTDLYSPEYPGPSHR (Ions score 48)
285 - 296	1523.6940	1522.6867	1522.6800	0.0067	0	DVTNNVHYENYR (No match)
285 - 296	1523.6940	1522.6867	1522.6800	0.0067	0	DVTNNVHYENYR (Ions score 25)

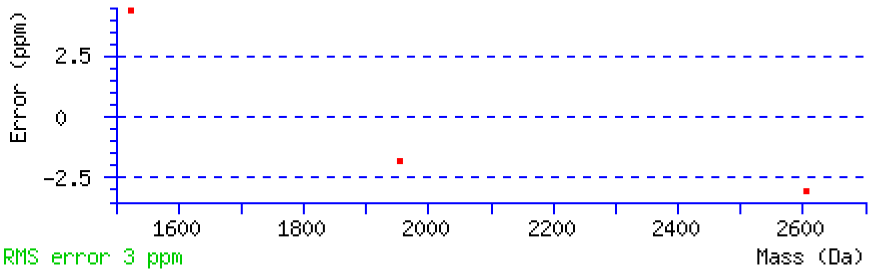

Error (ppm)
2.5
0
-2.5
1600
1800
2000
2200
2400
2600
RMS error 3 ppm
Mass (Da)

Spot 483

Protein View

Match to: **gi|31873302** Score: **386** Expect: **4.8e-034**
hypothetical protein [Homo sapiens]

Nominal mass (M_r): **47405**; Calculated pI value: **7.57**
NCBI BLAST search of gi|31873302 against nr
Unformatted sequence string for pasting into other applications

Taxonomy: Homo sapiens
Links to retrieve other entries containing this sequence from NCBI Entrez:
gi|117646030 from synthetic construct

Fixed modifications: Carbamidomethyl (C)
Variable modifications: Oxidation (M)
Cleavage by Trypsin: cuts C-term side of KR unless next residue is P
Sequence Coverage: **25%**

Matched peptides shown in **Bold Red**

```
  1 MSILKIHARE IFDSRGNPTV EVDLFTSKGL FRAAVPSGAS TGIYEALELR
 51 DNDKARYMGK GVSKAVEHIN KTIAPALVSK KLNVTEQEKI DKLMIEMDGT
101 ENKSKFGANA ILGVSLAVCK AGAVEKGVPL YRHIADLAGN SEVILPVPAF
151 NVINGGSHAG NKLAMQEFMI LPVGAANFRE AMRIGAGVYH NLKNVIKEKY
201 GKDATNVGDE GGFAPNILEN KEGLELLKTA IGKAGYTDKV VIGMDVAASE
251 FFRSGKYDLD FKSPDDPSRY ISPDQLADLY KSFIKDYPVV SIEDPFDQDD
301 WGAWQKFTAI AGIQVVGDDL TVTNPKRIAK AVNEKSCNCL LLKVNQIGSV
351 TESLQACKLA QANGWGVMVS HRSGETEDTF IADLVVGLCT GQIKTGAPCR
401 SERLAKYNQL LRIEEELGSK AKFAGRNFRN PLAK
```

Residue Number Increasing Mass Decreasing Mass

Start - End	Observed	Mr(expt)	Mr(calc)	Delta	Miss	Sequence
33 - 50	1804.9922	1803.9849	1803.9366	0.0483	0	AAVPSGASTGIYEALELR (Ions score 133)
33 - 50	1804.9922	1803.9849	1803.9366	0.0483	0	AAVPSGASTGIYEALELR (No match)
133 - 162	3011.6990	3010.6917	3010.5623	0.1294	0	HIADLAGNSEVILPVPAFNVINGGSHAGNK (No match)
133 - 162	3011.6990	3010.6917	3010.5623	0.1294	0	HIADLAGNSEVILPVPAFNVINGGSHAGNK (Ions score 108)
163 - 179	1940.0327	1939.0254	1938.9695	0.0559	0	LAMQEFMILPVGAANFR 2 Oxidation (M) (No match)
163 - 179	1940.0327	1939.0254	1938.9695	0.0559	0	LAMQEFMILPVGAANFR 2 Oxidation (M) (Ions score 23)

234 - 253	2192.1582	2191.1509	2191.0618	0.0891	1	**AGYTDKVVIGMDVAASEFFR** Oxidation (M) (No match)
240 - 253	1556.8142	1555.8069	1555.7704	0.0366	0	**VVIGMDVAASEFFR** Oxidation (M) (No match)
240 - 253	1556.8142	1555.8069	1555.7704	0.0366	0	**VVIGMDVAASEFFR** Oxidation (M) (Ions score 15)
270 - 281	1425.7595	1424.7522	1424.7187	0.0335	0	**YISPDQLADLYK** (No match)
407 - 412	806.4730	805.4657	805.4446	0.0211	0	**YNQLLR** (No match)
427 - 434	959.5673	958.5600	958.5348	0.0252	1	**NFRNPLAK** (No match)

Spot 484

Protein View

Match to: **gi|62088934** Score: **288** Expect: **3e-024**
CDC10 protein variant [Homo sapiens]

Nominal mass (M_r): **44084**; Calculated pI value: **7.73**
NCBI BLAST search of gi|62088934 against nr
Unformatted sequence string for pasting into other applications

Taxonomy: Homo sapiens

Fixed modifications: Carbamidomethyl (C)
Variable modifications: Oxidation (M)
Cleavage by Trypsin: cuts C-term side of KR unless next residue is P
Sequence Coverage: **16%**

Matched peptides shown in **Bold Red**

```
  1 VNSSTMVAQQ KNLEGYVGFA NLPNQVYRKS VKRGFEFTLM VVGESGLGKS
 51 TLINSLFLTD LYSPEYPGPS HRIKKTVQVE QSKVLIKEGG VQLLLTIVDT
101 PGFGDAVDNS NCWQPVIDYI DSKFEDYLNA ESRVNRRQMP DNRVQCCLYF
151 IAPSGHGLKP LDIEFMKRLH EKVNIIPLIA KADTLTPEEC QQFKKQIMKE
201 IQEHKIKIYE FPETDDEEEN KLVKKIKDRL PLAVVGSNTI IEVNGKRVRG
251 RQYPWGVAEV ENGEHCDFTI LRNMLIRTHM QDLKDVTNNV HYENYRSRKL
301 AAVTYNGVDN NKNKGQLTKS PLAQMEEERR EHVAKMKKME MEMEQVFEMK
351 VKEKVQKLKD SEAEVISLIS HLLADIVFLF K
```

Residue Number Increasing Mass Decreasing Mass

Start - End	Observed	Mr(expt)	Mr(calc)	Delta	Miss	Sequence
124 - 133	1243.5045	1242.4972	1242.5516	-0.0544	0	FEDYLNAESR (No match)
124 - 133	1243.5045	1242.4972	1242.5516	-0.0544	0	FEDYLNAESR (Ions score 73)
182 - 194	1566.6506	1565.6433	1565.7031	-0.0598	0	ADTLTPEECQQFK (No match)
182 - 195	1694.7311	1693.7238	1693.7981	-0.0743	1	ADTLTPEECQQFKK (No match)
208 - 224	2097.8918	2096.8845	2096.9789	-0.0944	1	IYEFPETDDEEENKLVK (Ions score 77)
208 - 224	2097.8918	2096.8845	2096.9789	-0.0944	1	IYEFPETDDEEENKLVK (No match)
285 - 296	1523.6188	1522.6115	1522.6800	-0.0685	0	DVTNNVHYENYR (No match)
285 - 296	1523.6188	1522.6115	1522.6800	-0.0685	0	DVTNNVHYENYR (Ions score 92)
320 - 330	1361.5833	1360.5760	1360.6405	-0.0644	1	SPLAQMEEERR Oxidation (M) (No match)

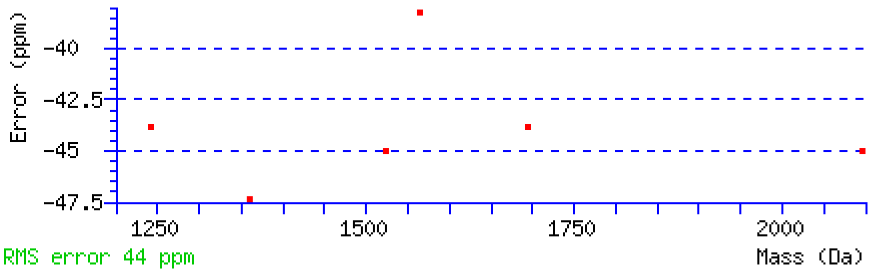

Error (ppm)
-40
-42.5
-45
-47.5
1250
1500
1750
2000
RMS error 44 ppm
Mass (Da)

Spot 485

Protein View

Match to: **gi|15277503** Score: **272** Expect: **1.2e-022**
ACTB protein [Homo sapiens]

Nominal mass (M_r): **40536**; Calculated pI value: **5.55**
NCBI BLAST search of gi|15277503 against nr
Unformatted sequence string for pasting into other applications

Taxonomy: Homo sapiens

Fixed modifications: Carbamidomethyl (C)
Variable modifications: Oxidation (M)
Cleavage by Trypsin: cuts C-term side of KR unless next residue is P
Sequence Coverage: **34%**

Matched peptides shown in **Bold Red**

```
  1 MCKAGFAGDD APRAVFPSIV GRPRHQGVMV GMGQKDSYVG DEAQSKRGIL
 51 TLKYPIEHGI VTNWDDMEKI WHHTFYNELR VAPEEHPVLL TEAPLNPKAN
101 LEKMTQIMFE TFNTPAMYVA IQAVLSLYAS GRTTGIVMDS GDGVTHTVPI
151 YEGYALPHAI LRLDLAGRDL TDYLMKILTE RGYSFTTTAE REIVRDIKEK
201 LCYVALDFEQ EMATAASSSS LEKSYELPDG QVITIGNERF RCPEALFQPS
251 FLGMESCGIH ETTFNSIMKC DVDIRKDLYA NTVLSGGTTM YPGIADRMQK
301 EITALAPSTM KIKIIAPPER KYSVWIGGSI LASLSTFQQM WISKQEYDES
351 GPSIVHRKCF
```

Residue Number Increasing Mass Decreasing Mass

Start - End	Observed	Mr(expt)	Mr(calc)	Delta	Miss	Sequence
4 - 13	976.4002	975.3929	975.4409	-0.0480	0	AGFAGDDAPR (No match)
14 - 24	1198.6486	1197.6413	1197.6982	-0.0568	0	AVFPSIVGRPR (Ions score 59)
14 - 24	1198.6486	1197.6413	1197.6982	-0.0568	0	AVFPSIVGRPR (No match)
36 - 47	1354.5619	1353.5546	1353.6160	-0.0614	1	DSYVGDEAQSKR (No match)
81 - 98	1953.9670	1952.9597	1953.0571	-0.0973	0	VAPEEHPVLLTEAPLNPK (Ions score 107)
81 - 98	1953.9670	1952.9597	1953.0571	-0.0973	0	VAPEEHPVLLTEAPLNPK (No match)
133 - 162	3199.4724	3198.4651	3198.6019	-0.1367	0	TTGIVMDSGDGVTHTVPIYEGYALPHAILR Oxidation (M) (No match)
182 - 191	1132.4714	1131.4641	1131.5196	-0.0555	0	GYSFTTTAER (No match)
182 - 191	1132.4714	1131.4641	1131.5196	-0.0555	0	GYSFTTTAER (Ions score 29)

276 - 297	2359.0491	2358.0418	2358.1525	-0.1107	1	**KDLYANTVLSGGTTMYPGIADR** Oxidation (M) (No match)
312 - 320	1036.6058	1035.5985	1035.6440	-0.0455	1	**IKIIAPPER** (No match)
314 - 321	923.5245	922.5172	922.5600	-0.0427	1	**IIAPPERK** (No match)

Spot 486

Protein View

Match to: **gi|5031699** Score: **704** Expect: **7.7e-066**
flotillin 1 [Homo sapiens]

Nominal mass (M_r): **47554;** Calculated pI value: **7.08**
NCBI BLAST search of gi|5031699 against nr
Unformatted sequence string for pasting into other applications

Taxonomy: Homo sapiens
Links to retrieve other entries containing this sequence from NCBI Entrez:
gi|26006960 from Homo sapiens
gi|3599573 from Homo sapiens
gi|12654619 from Homo sapiens
gi|15277227 from Homo sapiens
gi|27544399 from Homo sapiens
gi|30582993 from Homo sapiens
gi|55961565 from Homo sapiens
gi|55961676 from Homo sapiens
gi|57209816 from Homo sapiens
gi|60655509 from synthetic construct
gi|60655511 from synthetic construct
gi|86197962 from Homo sapiens
gi|114306780 from Homo sapiens
gi|119623731 from Homo sapiens
gi|119623732 from Homo sapiens
gi|123293910 from Homo sapiens
gi|123994279 from synthetic construct
gi|124126967 from synthetic construct

Fixed modifications: Carbamidomethyl (C)
Variable modifications: Oxidation (M)
Cleavage by Trypsin: cuts C-term side of KR unless next residue is P
Sequence Coverage: **63%**

Matched peptides shown in **Bold Red**

```
  1 MFFTCGPNEA MVVSGFCRSP PVMVAGGRVF VLPCIQQIQR ISLNTLTLNV
 51 KSEKVYTRHG VPISVTGIAQ VKIQGQNKEM LAAACQMFLG KTEAEIAHIA
101 LETLEGHQRA IMAHMTVEEI YKDRQKFSEQ VFKVASSDLV NMGISVVSYT
151 LKDIHDDQDY LHSLGKARTA QVQKDARIGE AEAKRDAGIR EAKAKQEKVS
201 AQYLSEIEMA KAQRDYELKK AAYDIEVNTR RAQADLAYQL QVAKTKQQIE
251 EQRVQVQVVE RAQQVAVQEQ EIARREKELE ARVRKPAEAE RYKLERLAEA
```

```
301 EKSQLIMQAE AEAASVRMRG EAEAFAIGAR ARAEAEQMAK KAEAFQLYQE
351 AAQLDMLLEK LPQVAEEISG PLTSANKITL VSSGSGTMGA AKVTGEVLDI
401 LTRLPESVER LTGVSISQVN HKPLRTA
```

Residue Number Increasing Mass Decreasing Mass

Start - End	Observed	Mr(expt)	Mr(calc)	Delta	Miss	Sequence
1 - 18	2109.8425	2108.8352	2108.8940	-0.0588	0	MFFTCGPNEAMVVSGFCR (No match)
1 - 18	2125.8567	2124.8494	2124.8889	-0.0395	0	MFFTCGPNEAMVVSGFCR Oxidation (M) (No match)
1 - 18	2141.8611	2140.8538	2140.8838	-0.0300	0	MFFTCGPNEAMVVSGFCR 2 Oxidation (M) (No match)
19 - 28	970.4918	969.4845	969.5065	-0.0220	0	SPPVMVAGGR (No match)
19 - 28	986.4833	985.4760	985.5014	-0.0254	0	SPPVMVAGGR Oxidation (M) (No match)
29 - 40	1500.7958	1499.7885	1499.8282	-0.0397	0	VFVLPCIQQIQR (No match)
29 - 40	1500.7958	1499.7885	1499.8282	-0.0397	0	VFVLPCIQQIQR (Ions score 72)
59 - 72	1405.7786	1404.7713	1404.8088	-0.0375	0	HGVPISVTGIAQVK (No match)
79 - 91	1469.7301	1468.7228	1468.6876	0.0352	0	EMLAAACQMFLGK (No match)
92 - 109	2017.9730	2016.9657	2017.0228	-0.0571	0	TEAEIAHIALETLEGHQR (No match)
92 - 109	2017.9730	2016.9657	2017.0228	-0.0571	0	TEAEIAHIALETLEGHQR (Ions score 128)
110 - 124	1822.8400	1821.8327	1821.8753	-0.0426	1	AIMAHMTVEEIYKDR Oxidation (M) (No match)
110 - 124	1838.8379	1837.8306	1837.8702	-0.0396	1	AIMAHMTVEEIYKDR 2 Oxidation (M) (No match)
153 - 166	1655.7249	1654.7176	1654.7587	-0.0411	0	DIHDDQDYLHSLGK (No match)
199 - 211	1484.6888	1483.6815	1483.7228	-0.0413	0	VSAQYLSEIEMAK Oxidation (M) (No match)
221 - 230	1151.5780	1150.5707	1150.5618	0.0089	0	AAYDIEVNTR (No match)
221 - 231	1307.6355	1306.6282	1306.6629	-0.0347	1	AAYDIEVNTRR (No match)
231 - 244	1574.8234	1573.8161	1573.8576	-0.0415	1	RAQADLAYQLQVAK (No match)
245 - 253	1159.5803	1158.5730	1158.5993	-0.0262	1	TKQQIEEQR (No match)
247 - 253	930.4452	929.4379	929.4566	-0.0187	0	QQIEEQR (No match)
254 - 261	956.5313	955.5240	955.5450	-0.0210	0	VQVQVVER (No match)
262 - 274	1469.7301	1468.7228	1468.7633	-0.0405	0	AQQVAVQEQEIAR (Ions score 78)
262 - 275	1625.8291	1624.8218	1624.8644	-0.0426	1	AQQVAVQEQEIARR (No match)
283 - 291	1055.5702	1054.5629	1054.5883	-0.0253	1	VRKPAEAER (No match)
303 - 317	1603.7690	1602.7617	1602.8035	-0.0418	0	SQLIMQAEAEAASVR (No match)
303 - 317	1619.7616	1618.7543	1618.7984	-0.0441	0	SQLIMQAEAEAASVR Oxidation (M) (No match)
318 - 330	1378.6530	1377.6457	1377.6822	-0.0365	1	MRGEAEAFAIGAR (No match)
318 - 330	1394.6450	1393.6377	1393.6771	-0.0394	1	MRGEAEAFAIGAR Oxidation (M) (No match)
320 - 330	1091.5255	1090.5182	1090.5406	-0.0224	0	GEAEAFAIGAR (No match)
342 - 360	2211.0554	2210.0481	2210.0929	-0.0447	0	AEAFQLYQEAAQLDMLLEK (No match)
393 - 403	1215.6689	1214.6616	1214.6870	-0.0254	0	VTGEVLDILTR (No match)
393 - 403	1215.6689	1214.6616	1214.6870	-0.0254	0	VTGEVLDILTR (Ions score 65)
404 - 410	829.4308	828.4235	828.4341	-0.0106	0	LPESVER (No match)
411 - 425	1648.9048	1647.8975	1647.9420	-0.0444	0	LTGVSISQVNHKPLR (No match)
411 - 425	1648.9048	1647.8975	1647.9420	-0.0444	0	LTGVSISQVNHKPLR (Ions score 74)

Spot 487

Protein View

Match to: **gi|62896593** Score: **597** Expect: **3.8e-055**
enolase 1 variant [Homo sapiens]

Nominal mass (M_r): **47453;** Calculated pI value: **7.01**
NCBI BLAST search of gi|62896593 against nr
Unformatted sequence string for pasting into other applications

Taxonomy: Homo sapiens

Fixed modifications: Carbamidomethyl (C)
Variable modifications: Oxidation (M)
Cleavage by Trypsin: cuts C-term side of KR unless next residue is P
Sequence Coverage: **55%**

Matched peptides shown in **Bold Red**

```
  1 MSILKIHARE IFDSRGNPTV EVDLFTSKGL FRAAVPSGAS TGIYEALELR
 51 DNDKTRYMGK GVSKAVEHIN KTIAPALASK KLNVTEQEKI DKLMIEMDGT
101 ENKSKFGANA ILGVSLAVCK AGAVEKGVPL YRHIADLAGN SEVILPVPAF
151 NVINGGSHAG NKLAMQEFMI LPVGAANFRE AMRIGAEVYH NLKNVIKEKY
201 GKDATNVGDE GGFAPNILEN KEGLELLKTA IGKAGYTDKV VIGMDVAASE
251 FFRSGKYDLD FKSPDDPSRY ISPDQLADLY KSFIKDYPVV SIEDPFDQDD
301 WGAWQKFTAS AGIQVVGDDL TVTNPKRIAK AVNEKSCNCL LLKVNQIGSV
351 TESLQACKLA QANGWGVMVS HRSGETEDTF IADLVVGLCT GQIKTGAPCR
401 SERLAKYNQL LRIEEELGSK AKFAGRNFRN PLAK
```

Residue Number Increasing Mass Decreasing Mass

Start - End	Observed	Mr(expt)	Mr(calc)	Delta	Miss	Sequence
16 - 28	1406.7009	1405.6936	1405.7088	-0.0152	0	GNPTVEVDLFTSK (No match)
33 - 50	1804.9224	1803.9151	1803.9366	-0.0215	0	AAVPSGASTGIYEALELR (Ions score 113)
33 - 50	1804.9224	1803.9151	1803.9366	-0.0215	0	AAVPSGASTGIYEALELR (No match)
33 - 54	2277.1108	2276.1035	2276.1284	-0.0248	1	AAVPSGASTGIYEALELRDNDK (No match)
106 - 120	1519.8132	1518.8059	1518.8227	-0.0168	0	FGANAILGVSLAVCK (No match)
133 - 162	3011.5256	3010.5183	3010.5623	-0.0440	0	HIADLAGNSEVILPVPAFNVINGGSHAGNK (No match)
163 - 179	1907.9701	1906.9628	1906.9797	-0.0169	0	LAMQEFMILPVGAANFR (No match)
163 - 179	1907.9701	1906.9628	1906.9797	-0.0169	0	LAMQEFMILPVGAANFR (Ions score 85)

163 - 179	1923.9628	1922.9555	1922.9746	-0.0191	0	**LAMQEFMILPVGAANFR** Oxidation (M) (No match)
163 - 179	1939.9585	1938.9512	1938.9695	-0.0183	0	**LAMQEFMILPVGAANFR** 2 Oxidation (M) (No match)
184 - 193	1143.5984	1142.5911	1142.6083	-0.0172	0	**IGAEVYHNLK** (No match)
203 - 221	1960.9001	1959.8928	1959.9173	-0.0245	0	**DATNVGDEGGFAPNILENK** (No match)
234 - 253	2176.0508	2175.0435	2175.0669	-0.0234	1	**AGYTDKVVIGMDVAASEFFR** (No match)
234 - 253	2176.0508	2175.0435	2175.0669	-0.0234	1	**AGYTDKVVIGMDVAASEFFR** (Ions score 108)
234 - 253	2192.0500	2191.0427	2191.0618	-0.0191	1	**AGYTDKVVIGMDVAASEFFR** Oxidation (M) (No match)
240 - 253	1540.7622	1539.7549	1539.7755	-0.0205	0	**VVIGMDVAASEFFR** (Ions score 21)
240 - 253	1540.7622	1539.7549	1539.7755	-0.0205	0	**VVIGMDVAASEFFR** (No match)
240 - 253	1556.7479	1555.7406	1555.7704	-0.0297	0	**VVIGMDVAASEFFR** Oxidation (M) (No match)
270 - 281	1425.7087	1424.7014	1424.7187	-0.0173	0	**YISPDQLADLYK** (No match)
307 - 326	2033.0342	2032.0269	2032.0476	-0.0207	0	**FTASAGIQVVGDDLTVTNPK** (No match)
344 - 358	1633.8093	1632.8020	1632.8141	-0.0120	0	**VNQIGSVTESLQACK** (No match)
359 - 372	1525.7546	1524.7473	1524.7619	-0.0146	0	**LAQANGWGVMVSHR** (Ions score 45)
359 - 372	1525.7546	1524.7473	1524.7619	-0.0146	0	**LAQANGWGVMVSHR** (No match)
359 - 372	1541.7594	1540.7521	1540.7568	-0.0047	0	**LAQANGWGVMVSHR** Oxidation (M) (No match)
373 - 394	2353.1318	2352.1245	2352.1518	-0.0273	0	**SGETEDTFIADLVVGLCTGQIK** (No match)
407 - 412	806.4424	805.4351	805.4446	-0.0095	0	**YNQLLR** (No match)
427 - 434	959.5318	958.5245	958.5348	-0.0103	1	**NFRNPLAK** (No match)

Spot 489

Protein View

Match to: **gi|9836652** Score: **145** Expect: **6.1e-010**
BSCv [Homo sapiens]

Nominal mass (M_r): **47887**; Calculated pI value: **5.78**
NCBI BLAST search of gi|9836652 against nr
Unformatted sequence string for pasting into other applications

Taxonomy: Homo sapiens

Fixed modifications: Carbamidomethyl (C)
Variable modifications: Oxidation (M)
Cleavage by Trypsin: cuts C-term side of KR unless next residue is P
Sequence Coverage: **12%**

Matched peptides shown in **Bold Red**

```
  1 HEVVSAGEAG ARTMSEADGL RQRRPLRPQV VTDDDGQAPE AKDGSSFSGR
 51 VFRVTFLMLA VSLTVPLLGA MMLLESPIDP QPLSFKEPPL LLGVLHPNTK
101 LRQAERLFEN QLVGPESIAH IGDVMFTGTA DGRVVKLENG EIETIARFGS
151 GPCKTRDDEP VCGRPLGIRA GPNGTLFVAD AYKGLFEVNP WKREVKLLLS
201 SETPIEGKNM SFVNDLTVTQ DGRKIYFTDS SSKWQRRDYL LLVMEGTDDG
251 RLLEYDTVTR EVKVLLDQLR FPNGVQLSPA EDFVLVAETT MARIRRVYVS
301 GLMKGGADLF VENMPGFPDN IRPSSSGGYW VGMSTIRPNP GFSMLDFLSE
351 RPWIKRMIFK LFSQETVMKF VPRYSLVLEL SDSGAFRRSL HDPDGLVATY
401 ISEVHEHDGH LYLGSFRSPF LCRLSLQAV
```

Residue Number Increasing Mass Decreasing Mass

Start - End	Observed	Mr(expt)	Mr(calc)	Delta	Miss	Sequence
24 - 42	2092.1089	2091.1016	2091.0708	0.0308	0	RPLRPQVVTDDDGQAPEAK (Ions score 17)
24 - 42	2092.1089	2091.1016	2091.0708	0.0308	0	RPLRPQVVTDDDGQAPEAK (No match)
87 - 100	1527.9066	1526.8993	1526.8820	0.0173	0	EPPLLLGVLHPNTK (No match)
87 - 100	1527.9066	1526.8993	1526.8820	0.0173	0	EPPLLLGVLHPNTK (Ions score 70)
155 - 169	1740.9058	1739.8985	1739.8736	0.0249	1	TRDDEPVCGRPLGIR (No match)
155 - 169	1740.9058	1739.8985	1739.8736	0.0249	1	TRDDEPVCGRPLGIR (Ions score 13)
264 - 270	856.5344	855.5271	855.5178	0.0094	0	VLLDQLR (No match)

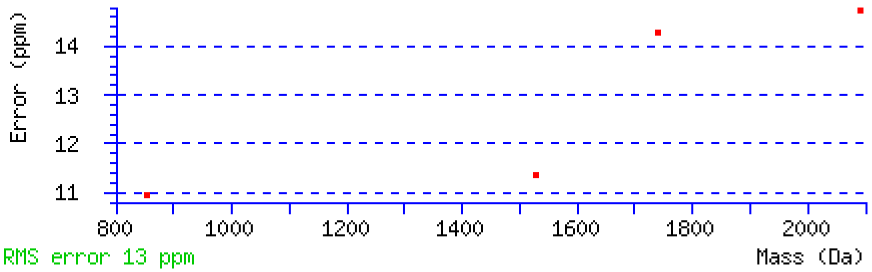

Error (ppm)
14
13
12
11
800
1000
1200
1400
1600
1800
2000
RMS error 13 ppm
Mass (Da)

Spot 490

Protein View

Match to: **gi|33879698** Score: **135** Expect: **6.1e-009**
PA2G4 protein [Homo sapiens]

Nominal mass (M_r): **41996;** Calculated pI value: **7.14**
NCBI BLAST search of gi|33879698 against nr
Unformatted sequence string for pasting into other applications

Taxonomy: Homo sapiens
Links to retrieve other entries containing this sequence from NCBI Entrez:
gi|47939640 from Homo sapiens

Fixed modifications: Carbamidomethyl (C)
Variable modifications: Oxidation (M)
Cleavage by Trypsin: cuts C-term side of KR unless next residue is P
Sequence Coverage: **18%**

Matched peptides shown in **Bold Red**

```
  1 MSGEDEQQEQ TIAEDLVVTK YKMGGDIANR VLRSLVEASS SGVSVLSLCE
 51 KGDAMIMEET GKIFKKEKEM KKGIAFPTSI SVNNCVCHFS PLKSDQDYIL
101 KEGDLVKIDL GVHVDGFIAN VAHTFVVDVA QGTQVTGRKA DVIKAAHLCA
151 EAALRLVKPG NQNTQVTEAW NKVAHSFNCT PIEGMLSHQL KQHVIDGEKT
201 IIQNPTDQQK KDHEKAEFEV HEVYAVDVLV SSGEGKAKDA GQRTTIYKRD
251 PSKQYGLKMK TSRAFFSEVE RRFDAMPFTL RAFEDEKKAR MGVVECAKHE
301 LLQPFNVLYE KEGEFVAQFK FTVLLMPNGP MRITSGPFEP DLYKSEMEVQ
351 DAELKALLQS SASRKTQKKK KKK
```

Residue Number Increasing Mass Decreasing Mass

Start - End	Observed	Mr(expt)	Mr(calc)	Delta	Miss	Sequence
21 - 30	1140.5516	1139.5443	1139.5393	0.0050	1	**YKMGGDIANR** Oxidation (M) (No match)
173 - 191	2184.9697	2183.9624	2184.0455	-0.0831	0	**VAHSFNCTPIEGMLSHQLK** Oxidation (M) (No match)
200 - 210	1285.6838	1284.6765	1284.6674	0.0092	0	**TIIQNPTDQQK** (No match)
264 - 271	984.4513	983.4440	983.4712	-0.0272	0	**AFFSEVER** (No match)
264 - 272	1140.5516	1139.5443	1139.5723	-0.0280	1	**AFFSEVERR** (Ions score 11)
272 - 281	1269.6088	1268.6015	1268.6335	-0.0320	1	**RFDAMPFTLR** Oxidation (M) (No match)
272 - 281	1269.6088	1268.6015	1268.6335	-0.0320	1	**RFDAMPFTLR** Oxidation (M) (Ions score 12)

273 - 281	1113.5101	1112.5028	1112.5324	-0.0296	0	**FDAMPFTLR** Oxidation (M) (Ions score 20)
273 - 281	1113.5101	1112.5028	1112.5324	-0.0296	0	**FDAMPFTLR** Oxidation (M) (No match)
321 - 332	1407.6763	1406.6690	1406.7050	-0.0359	0	**FTVLLMPNGPMR** 2 Oxidation (M) (No match)
321 - 332	1407.6763	1406.6690	1406.7050	-0.0359	0	**FTVLLMPNGPMR** 2 Oxidation (M) (Ions score 24)

Spot 491

Mascot Search Results

Protein View

Match to: **gi|40889612** Score: **66** Expect: **0.046**
Chain A, Crystal Structure Of An Active Fragment Of Human Tryptophanyl-Trna Synthetase With Cytokin

Nominal mass (M_r): **49731**; Calculated pI value: **6.10**
NCBI BLAST search of gi|40889612 against nr
Unformatted sequence string for pasting into other applications

Taxonomy: Homo sapiens
Links to retrieve other entries containing this sequence from NCBI Entrez:
gi|40889613 from Homo sapiens

Fixed modifications: Carbamidomethyl (C)
Variable modifications: Oxidation (M)
Cleavage by Trypsin: cuts C-term side of KR unless next residue is P
Sequence Coverage: **5%**

Matched peptides shown in **Bold Red**

```
  1 MSYKAAAGED YKADCPPGNP APTSNHGPDA TEAEEDFVDP WTVQTSSAKG
 51 IDYDKLIVRF GSSKIDKELI NRIERATGQR PHHFLRRGIF FSHRDXNQVL
101 DAYENKKPFY LYTGRGPSSE AXHVGHLIPF IFTKWLQDVF NVPLVIQXTD
151 DEKYLWKDLT LDQAYGDAVE NAKDIIACGF DINKTFIFSD LDYXGXSSGF
201 YKNVVKIQKH VTFNQVKGIF GFTDSDCIGK ISFPAIQAAP SFSNSFPQIF
251 RDRTDIQCLI PCAIDQDPYF RXTRDVAPRI GYPKPALLHS TFFPALQGAQ
301 TKXSASDPNS SIFLTDTAKQ IKTKVNKHAF SGGRDTIEEH RQFGGNCDVD
351 VSFXYLTFFL EDDDKLEQIR KDYTSGAXLT GELKKALIEV LQPLIAEHQA
401 RRKEVTDEIV KEFXTPRKLS FDFQKLAAAL EHHHHHH
```

Residue Number Increasing Mass Decreasing Mass

Start - End	Observed	Mr(expt)	Mr(calc)	Delta	Miss	Sequence
88 - 94	863.4366	862.4293	862.4449	-0.0156	0	GIFFSHR (No match)
386 - 401	1800.9940	1799.9867	1800.0257	-0.0390	0	ALIEVLQPLIAEHQAR (No match)
386 - 401	1800.9940	1799.9867	1800.0257	-0.0390	0	ALIEVLQPLIAEHQAR (Ions score 52)

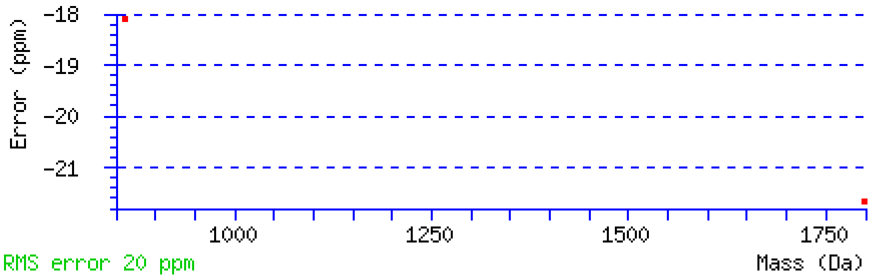

Mascot: http://www.matrixscience.com/

Spot 492

Mascot Search Results

Protein View

Match to: **gi|62896593** Score: **458** Expect: **3e-041**
enolase 1 variant [Homo sapiens]

Nominal mass (M_r): **47453**; Calculated pI value: **7.01**
NCBI BLAST search of gi|62896593 against nr
Unformatted sequence string for pasting into other applications

Taxonomy: Homo sapiens

Fixed modifications: Carbamidomethyl (C)
Variable modifications: Oxidation (M)
Cleavage by Trypsin: cuts C-term side of KR unless next residue is P
Sequence Coverage: **45%**

Matched peptides shown in **Bold Red**

```
  1 MSILKIHARE IFDSRGNPTV EVDLFTSKGL FRAAVPSGAS TGIYEALELR
 51 DNDKTRYMGK GVSKAVEHIN KTIAPALASK KLNVTEQEKI DKLMIEMDGT
101 ENKSKFGANA ILGVSLAVCK AGAVEKGVPL YRHIADLAGN SEVILPVPAF
151 NVINGGSHAG NKLAMQEFMI LPVGAANFRE AMRIGAEVYH NLKNVIKEKY
201 GKDATNVGDE GGFAPNILEN KEGLELLKTA IGKAGYTDKV VIGMDVAASE
251 FFRSGKYDLD FKSPDDPSRY ISPDQLADLY KSFIKDYPVV SIEDPFDQDD
301 WGAWQKFTAS AGIQVVGDDL TVTNPKRIAK AVNEKSCNCL LLKVNQIGSV
351 TESLQACKLA QANGWGVMVS HRSGETEDTF IADLVVGLCT GQIKTGAPCR
401 SERLAKYNQL LRIEEELGSK AKFAGRNFRN PLAK
```

Residue Number Increasing Mass Decreasing Mass

Start - End	Observed	Mr(expt)	Mr(calc)	Delta	Miss	Sequence
16 - 28	1406.7135	1405.7062	1405.7088	-0.0026	0	GNPTVEVDLFTSK (No match)
33 - 50	1804.9489	1803.9416	1803.9366	0.0050	0	AAVPSGASTGIYEALELR (Ions score 115)
33 - 50	1804.9489	1803.9416	1803.9366	0.0050	0	AAVPSGASTGIYEALELR (No match)
133 - 162	3011.6213	3010.6140	3010.5623	0.0517	0	HIADLAGNSEVILPVPAFNVINGGSHAGNK (No match)
163 - 179	1939.9865	1938.9792	1938.9695	0.0097	0	LAMQEFMILPVGAANFR 2 Oxidation (M) (No match)
163 - 179	1939.9865	1938.9792	1938.9695	0.0097	0	LAMQEFMILPVGAANFR 2 Oxidation (M) (Ions score 28)

184 - 193	1143.6145	1142.6072	1142.6083	-0.0011	0	IGAEVYHNLK (No match)
203 - 221	1960.9364	1959.9291	1959.9173	0.0118	0	DATNVGDEGGFAPNILENK (No match)
234 - 253	2192.1060	2191.0987	2191.0618	0.0369	1	AGYTDKVVIGMDVAASEFFR Oxidation (M) (No match)
240 - 253	1556.7764	1555.7691	1555.7704	-0.0012	0	VVIGMDVAASEFFR Oxidation (M) (Ions score 26)
240 - 253	1556.7764	1555.7691	1555.7704	-0.0012	0	VVIGMDVAASEFFR Oxidation (M) (No match)
254 - 262	1072.5305	1071.5232	1071.5236	-0.0004	1	SGKYDLDFK (No match)
270 - 281	1425.7246	1424.7173	1424.7187	-0.0014	0	YISPDQLADLYK (Ions score 60)
270 - 281	1425.7246	1424.7173	1424.7187	-0.0014	0	YISPDQLADLYK (No match)
307 - 326	2033.0703	2032.0630	2032.0476	0.0154	0	FTASAGIQVVGDDLTVTNPK (No match)
307 - 327	2189.1697	2188.1624	2188.1487	0.0137	1	FTASAGIQVVGDDLTVTNPKR (No match)
344 - 358	1633.8246	1632.8173	1632.8141	0.0033	0	VNQIGSVTESLQACK (No match)
407 - 412	806.4550	805.4477	805.4446	0.0031	0	YNQLLR (No match)
427 - 434	959.5460	958.5387	958.5348	0.0039	1	NFRNPLAK (No match)

Mascot: http://www.matrixscience.com/

Spot 495

Mascot Search Results

Protein View

Match to: **gi|27436897** Score: **238** Expect: **3e-019**
tapasin isoform 3 precursor [Homo sapiens]

Nominal mass (M_r): **44145;** Calculated pI value: **6.35**
NCBI BLAST search of gi|27436897 against nr
Unformatted sequence string for pasting into other applications

Taxonomy: Homo sapiens
Links to retrieve other entries containing this sequence from NCBI Entrez:
gi|119624120 from Homo sapiens

Fixed modifications: Carbamidomethyl (C)
Variable modifications: Oxidation (M)
Cleavage by Trypsin: cuts C-term side of KR unless next residue is P
Sequence Coverage: **19%**

Matched peptides shown in **Bold Red**

```
  1 MKSLSLLLAV ALGLATAVSA GPAVIECWFV EDASGKGLAK RPGALLLRQG
 51 PGEPPPRPDL DPELYLSVHD PAGALQAAFR RYPRGAPAPH CEMSRFVPLP
101 ASAKWASGLT PAQNCPRALD GAWLMVSISS PVLSLSSLLR PQPEPQQEPV
151 LITMATVVLT VLTHTPAPRV RLGQDALLDL SFAYMPPTSE AASSLAPGPP
201 PFGLEWRRQH LGKGHLLLAA TPGLNGQMPA AQEGAVAFAA WDDDEPWGPW
251 TGNGTFWLPT VQPFQEGTYL ATIHLPYLQG QVTLELAVYK PPKVSLMPAT
301 LARAAPGEAP PELLCLVSHF YPSGGLEVEW ELRGGPGGRS QKAEGQRWLS
351 ALRHHSDGSV SLSGHLQPPP VTTEQHGARY ACRIHHPSLP ASGRSAEVTL
401 EVAGKSWELC GI
```

Residue Number Increasing Mass Decreasing Mass

Start - End	Observed	Mr(expt)	Mr(calc)	Delta	Miss	Sequence
41 - 48	895.5657	894.5584	894.5763	-0.0178	0	RPGALLLR (No match)
85 - 95	1228.4956	1227.4883	1227.5124	-0.0241	0	GAPAPHCEMSR Oxidation (M) (Ions score 5)
85 - 95	1228.4956	1227.4883	1227.5124	-0.0241	0	GAPAPHCEMSR Oxidation (M) (No match)
105 - 117	1457.6665	1456.6592	1456.6881	-0.0289	0	WASGLTPAQNCPR (No match)

105 - 117	1457.6665	1456.6592	1456.6881	-0.0289	0	**WASGLTPAQNCPR** (Ions score 31)
294 - 303	1074.5735	1073.5662	1073.5903	-0.0240	0	**VSLMPATLAR** Oxidation (M) (No match)
354 - 379	2731.2764	2730.2691	2730.3222	-0.0531	0	**HHSDGSVSLSGHLQPPPVTTEQHGAR** (Ions score 107)
354 - 379	2731.2764	2730.2691	2730.3222	-0.0531	0	**HHSDGSVSLSGHLQPPPVTTEQHGAR** (No match)
384 - 394	1171.6121	1170.6048	1170.6257	-0.0209	0	**IHHPSLPASGR** (Ions score 30)
384 - 394	1171.6121	1170.6048	1170.6257	-0.0209	0	**IHHPSLPASGR** (No match)

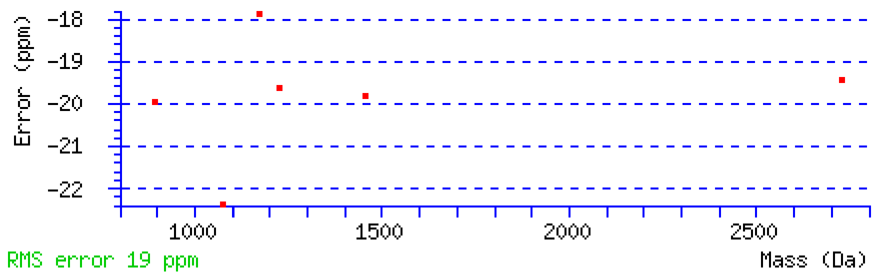

Mascot: http://www.matrixscience.com/

Spot 496

Mascot Search Results

Protein View

Match to: **gi|62896593** Score: **755** Expect: **6.1e-071**
enolase 1 variant [Homo sapiens]

Nominal mass (M_r): **47453**; Calculated pI value: **7.01**
NCBI BLAST search of gi|62896593 against nr
Unformatted sequence string for pasting into other applications

Taxonomy: Homo sapiens

Fixed modifications: Carbamidomethyl (C)
Variable modifications: Oxidation (M)
Cleavage by Trypsin: cuts C-term side of KR unless next residue is P
Sequence Coverage: **59%**

Matched peptides shown in **Bold Red**

```
  1 MSILKIHARE IFDSRGNPTV EVDLFTSKGL FRAAVPSGAS TGIYEALELR
 51 DNDKTRYMGK GVSKAVEHIN KTIAPALASK KLNVTEQEKI DKLMIEMDGT
101 ENKSKFGANA ILGVSLAVCK AGAVEKGVPL YRHIADLAGN SEVILPVPAF
151 NVINGGSHAG NKLAMQEFMI LPVGAANFRE AMRIGAEVYH NLKNVIKEKY
201 GKDATNVGDE GGFAPNILEN KEGLELLKTA IGKAGYTDKV VIGMDVAASE
251 FFRSGKYDLD FKSPDDPSRY ISPDQLADLY KSFIKDYPVV SIEDPFDQDD
301 WGAWQKFTAS AGIQVVGDDL TVTNPKRIAK AVNEKSCNCL LLKVNQIGSV
351 TESLQACKLA QANGWGVMVS HRSGETEDTF IADLVVGLCT GQIKTGAPCR
401 SERLAKYNQL LRIEEELGSK AKFAGRNFRN PLAK
```

Residue Number Increasing Mass Decreasing Mass

Start - End	Observed	Mr(expt)	Mr(calc)	Delta	Miss	Sequence
16 - 28	1406.7202	1405.7129	1405.7088	0.0041	0	GNPTVEVDLFTSK (No match)
33 - 50	1804.9476	1803.9403	1803.9366	0.0037	0	AAVPSGASTGIYEALELR (Ions score 146)
33 - 50	1804.9476	1803.9403	1803.9366	0.0037	0	AAVPSGASTGIYEALELR (No match)
33 - 54	2277.1316	2276.1243	2276.1284	-0.0040	1	AAVPSGASTGIYEALELRDNDK (No match)
106 - 120	1519.8293	1518.8220	1518.8227	-0.0007	0	FGANAILGVSLAVCK (No match)
133 - 162	3011.5671	3010.5598	3010.5623	-0.0025	0	HIADLAGNSEVILPVPAFNVINGGSHAGNK (No match)

163 - 179	1907.9937	1906.9864	1906.9797	0.0067	0	LAMQEFMILPVGAANFR (Ions score 85)
163 - 179	1907.9937	1906.9864	1906.9797	0.0067	0	LAMQEFMILPVGAANFR (No match)
163 - 179	1923.9915	1922.9842	1922.9746	0.0096	0	LAMQEFMILPVGAANFR Oxidation (M) (No match)
163 - 179	1939.9821	1938.9748	1938.9695	0.0053	0	LAMQEFMILPVGAANFR 2 Oxidation (M) (No match)
184 - 193	1143.6190	1142.6117	1142.6083	0.0034	0	IGAEVYHNLK (No match)
203 - 221	1960.9250	1959.9177	1959.9173	0.0004	0	DATNVGDEGGFAPNILENK (No match)
234 - 253	2176.0754	2175.0681	2175.0669	0.0012	1	AGYTDKVVIGMDVAASEFFR (No match)
234 - 253	2176.0754	2175.0681	2175.0669	0.0012	1	AGYTDKVVIGMDVAASEFFR (Ions score 171)
234 - 253	2192.0754	2191.0681	2191.0618	0.0063	1	AGYTDKVVIGMDVAASEFFR Oxidation (M) (No match)
240 - 253	1540.7864	1539.7791	1539.7755	0.0037	0	VVIGMDVAASEFFR (Ions score 51)
240 - 253	1540.7864	1539.7791	1539.7755	0.0037	0	VVIGMDVAASEFFR (No match)
240 - 253	1556.7697	1555.7624	1555.7704	-0.0079	0	VVIGMDVAASEFFR Oxidation (M) (No match)
254 - 262	1072.5344	1071.5271	1071.5236	0.0035	1	SGKYDLDFK (No match)
270 - 281	1425.7279	1424.7206	1424.7187	0.0019	0	YISPDQLADLYK (No match)
307 - 326	2033.0634	2032.0561	2032.0476	0.0085	0	FTASAGIQVVGDDLTVTNPK (No match)
336 - 343	1007.5187	1006.5114	1006.4939	0.0175	0	SCNCLLLK (No match)
344 - 358	1633.8257	1632.8184	1632.8141	0.0044	0	VNQIGSVTESLQACK (No match)
359 - 372	1525.7758	1524.7685	1524.7619	0.0066	0	LAQANGWGVMVSHR (Ions score 67)
359 - 372	1525.7758	1524.7685	1524.7619	0.0066	0	LAQANGWGVMVSHR (No match)
373 - 394	2353.1614	2352.1541	2352.1518	0.0023	0	SGETEDTFIADLVVGLCTGQIK (No match)
407 - 412	806.4526	805.4453	805.4446	0.0007	0	YNQLLR (No match)
427 - 434	959.5427	958.5354	958.5348	0.0006	1	NFRNPLAK (No match)

Mascot: http://www.matrixscience.com/

Spot 497

Mascot Search Results

Protein View

Match to: **gi|119594430** Score: **154** Expect: **7.7e-011**
eukaryotic translation elongation factor 1 gamma, isoform CRA_b [Homo sapiens]

Nominal mass (M_r): **44869**; Calculated pI value: **6.07**
NCBI BLAST search of gi|119594430 against nr
Unformatted sequence string for pasting into other applications

Taxonomy: Homo sapiens

Fixed modifications: Carbamidomethyl (C)
Variable modifications: Oxidation (M)
Cleavage by Trypsin: cuts C-term side of KR unless next residue is P
Sequence Coverage: **16%**

Matched peptides shown in **Bold Red**

```
  1 MAAGTLYTYP ENWRAFKALI AAQYSGAQVR VLSAPPHFHF GQTNRTPEFL
 51 RKFPAGKVPA FEGDDGFCVF ESNAIAYYVS NEELRGSTPE AAAQVVQWVS
101 FADSDIVPPA STWVFPTLGI MHHNKQVLEP SFRQAFPNTN RWFLTCINQP
151 QFRAVLGEVK LCEKMAQFDA KKFAETQPKK DTPRKEKGSR EEKQKPQAER
201 KEEKKAAAPA PEEEMDECEQ ALAAEPKAKD PFAHLPKSTF VLDEFKRKYS
251 NEDTLSVALP YFWEHFDKDG WSLWYSEYRF PEELTQTFMS CNLITGMFQR
301 LDKLRKNAFA SVILFGTNNS SSISGVWVFR GQELAFPLSP DWQVDYESYT
351 WRKLDPGSEE TQTLVREYFS WEGAFQHVGK AFNQGKIFK
```

Residue Number Increasing Mass Decreasing Mass

Start - End	Observed	Mr(expt)	Mr(calc)	Delta	Miss	Sequence
18 - 30	1347.7441	1346.7368	1346.7306	0.0062	0	ALIAAQYSGAQVR (Ions score 27)
18 - 30	1347.7441	1346.7368	1346.7306	0.0062	0	ALIAAQYSGAQVR (No match)
31 - 45	1707.8704	1706.8631	1706.8641	-0.0009	0	VLSAPPHFHFGQTNR (Ions score 35)
31 - 45	1707.8704	1706.8631	1706.8641	-0.0009	0	VLSAPPHFHFGQTNR (No match)
142 - 153	1609.7975	1608.7902	1608.7871	0.0031	0	WFLTCINQPQFR (No match)
142 - 153	1609.7975	1608.7902	1608.7871	0.0031	0	WFLTCINQPQFR (Ions score 10)
238 - 247	1241.6589	1240.6516	1240.6451	0.0065	1	STFVLDEFKR (Ions score 24)

238 - 247	1241.6589	1240.6516	1240.6451	0.0065	1	STFVLDEFKR	(No match)
354 - 366	1444.7559	1443.7486	1443.7205	0.0281	0	LDPGSEETQTLVR	(No match)

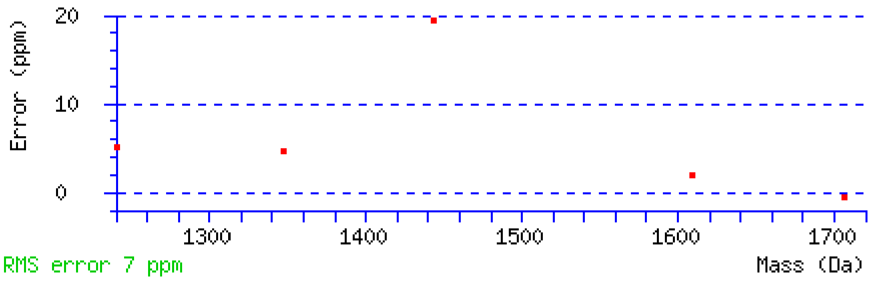

Mascot: http://www.matrixscience.com/

Spot 498

Mascot Search Results

Protein View

Match to: **gi|76879893** Score: **82** Expect: **0.0013**
putative protein product of Nbla10058 [Homo sapiens]

Nominal mass (M_r): **48986;** Calculated pI value: **5.85**
NCBI BLAST search of gi|76879893 against nr
Unformatted sequence string for pasting into other applications

Taxonomy: Homo sapiens

Fixed modifications: Carbamidomethyl (C)
Variable modifications: Oxidation (M)
Cleavage by Trypsin: cuts C-term side of KR unless next residue is P
Sequence Coverage: **11%**

Matched peptides shown in **Bold Red**

```
  1 MPDYLGADQR KTKEVEKDDK PIRALDEGDI ALLKTYGQST YSRQIKQVED
 51 DIQQLLKKIN ELTGIKESDT GLAPPALWDL AADKQTLQSE QPLQVARCTK
101 IINADSEDPK YIINVKQFAK FVVDLSDQVA PTDIEEGMRV GVDRNKYQIH
151 IPLPPKIDPT VTMMQVEEKP DVTYSDVGGC KEQIEKLREV VETPLLHPER
201 FVNLGIEPPK GVLLFGPPGT GKTLCARAVA NRTDACFIRV IGSELVQKYV
251 GEGARMVREL FEMARTKKAC LIFFDEIDAI GGARFDDGAG GDNEVQRTML
301 ELINQLDGFD PRGNIKVLMA TNRPDTLDPA LMRPGRLDRK IEFSLPDLEG
351 RTHIFKIHAR SMSVERDIRF ELLARLCPNS TGAEIRSVCT EAGMFAIRAR
401 RKIATEKDFL EAVNKVIKSY AKFSATPRYM TYN
```

Residue Number Increasing Mass Decreasing Mass

Start - End	Observed	Mr(expt)	Mr(calc)	Delta	Miss	Sequence
101 - 116	1831.8899	1830.8826	1830.9727	-0.0900	1	IINADSEDPKYIINVK (No match)
285 - 297	1379.5168	1378.5095	1378.5749	-0.0653	0	FDDGAGGDNEVQR (No match)
285 - 297	1379.5168	1378.5095	1378.5749	-0.0653	0	FDDGAGGDNEVQR (Ions score 27)
340 - 351	1403.6836	1402.6763	1402.7456	-0.0692	1	KIEFSLPDLEGR (No match)
340 - 351	1403.6836	1402.6763	1402.7456	-0.0692	1	KIEFSLPDLEGR (Ions score 25)
341 - 351	1275.5983	1274.5910	1274.6506	-0.0596	0	IEFSLPDLEGR (No match)

367 - 375	1132.5922	1131.5849	1131.6400	-0.0551	1	DIRFELLAR	(No match)
367 - 375	1132.5922	1131.5849	1131.6400	-0.0551	1	DIRFELLAR	(No match)

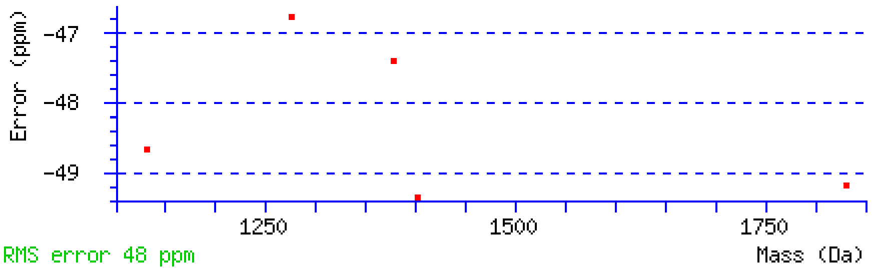

Mascot: http://www.matrixscience.com/

Spot 499

Mascot Search Results

Protein View

Match to: **gi|119578931** Score: **68** Expect: **0.029**
DnaJ (Hsp40) homolog, subfamily A, member 1, isoform CRA_d [Homo sapiens]

Nominal mass (M_r): **42864;** Calculated pI value: **7.53**
NCBI BLAST search of gi|119578931 against nr
Unformatted sequence string for pasting into other applications

Taxonomy: Homo sapiens

Fixed modifications: Carbamidomethyl (C)
Variable modifications: Oxidation (M)
Cleavage by Trypsin: cuts C-term side of KR unless next residue is P
Sequence Coverage: **9%**

Matched peptides shown in **Bold Red**

```
  1 MVKETTYYDV LGVKPNATQE ELKKAYRKLA LKYHPDKNPN EGEKFKQISQ
 51 AYEVLSDAKK RELYDKGGRM QRERRGKNVV HQLSVTLEDL YNGATRKLAL
101 QKNVICDKCE GRGGKKGAVE CCPNCRGTGM QIRIHQIGPG MVQQIQSVCM
151 ECQGHGERIS PKDRCKSCNG RKIVREKKIL EVHIDKGMKD GQKITFHGEG
201 DQEPGLEPGD IIIVLDQKDH AVFTRRGEDL FMCMDIQLVE ALCGFQKPIS
251 TLDNRTIVIT SHPGQIVKHG DIKCVLNEGM PIYRRPYEKG RLIIEFKVNF
301 PENGFLSPDK LSLLEKLLPE RKEVEETDEM DQVELVDFDP NQERRRHYNG
351 EAYEDDEHHP RGGVQCQTS
```

Residue Number Increasing Mass Decreasing Mass

Start - End	Observed	Mr(expt)	Mr(calc)	Delta	Miss	Sequence
78 - 96	2129.0566	2128.0493	2128.0912	-0.0419	0	NVVHQLSVTLEDLYNGATR (No match)
78 - 96	2129.0566	2128.0493	2128.0912	-0.0419	0	NVVHQLSVTLEDLYNGATR (Ions score 6)
347 - 361	1868.7223	1867.7150	1867.7509	-0.0359	0	HYNGEAYEDDEHHPR (No match)
347 - 361	1868.7223	1867.7150	1867.7509	-0.0359	0	HYNGEAYEDDEHHPR (Ions score 41)

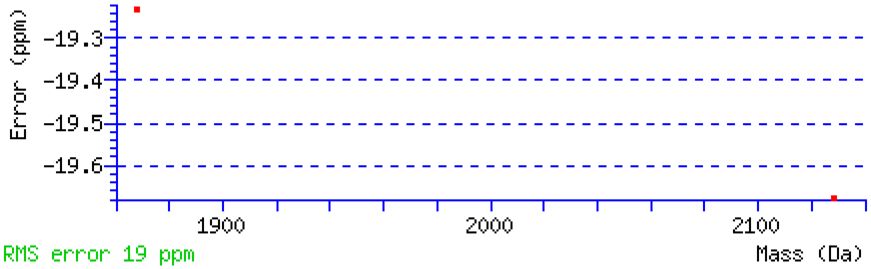

RMS error 19 ppm

Mascot: http://www.matrixscience.com/

Spot 500

MATRIX SCIENCE Mascot Search Results

Protein View

Match to: **gi|15277503** Score: **498** Expect: **3e-045**
ACTB protein [Homo sapiens]

Nominal mass (M_r): **40536;** Calculated pI value: **5.55**
NCBI BLAST search of gi|15277503 against nr
Unformatted sequence string for pasting into other applications

Taxonomy: Homo sapiens

Fixed modifications: Carbamidomethyl (C)
Variable modifications: Oxidation (M)
Cleavage by Trypsin: cuts C-term side of KR unless next residue is P
Sequence Coverage: **42%**

Matched peptides shown in **Bold Red**

```
  1 MCKAGFAGDD APRAVFPSIV GRPRHQGVMV GMGQKDSYVG DEAQSKRGIL
 51 TLKYPIEHGI VTNWDDMEKI WHHTFYNELR VAPEEHPVLL TEAPLNPKAN
101 LEKMTQIMFE TFNTPAMYVA IQAVLSLYAS GRTTGIVMDS GDGVTHTVPI
151 YEGYALPHAI LRLDLAGRDL TDYLMKILTE RGYSFTTTAE REIVRDIKEK
201 LCYVALDFEQ EMATAASSSS LEKSYELPDG QVITIGNERF RCPEALFQPS
251 FLGMESCGIH ETTFNSIMKC DVDIRKDLYA NTVLSGGTTM YPGIADRMQK
301 EITALAPSTM KIKIIAPPER KYSVWIGGSI LASLSTFQQM WISKQEYDES
351 GPSIVHRKCF
```

Residue Number Increasing Mass Decreasing Mass

Start - End	Observed	Mr(expt)	Mr(calc)	Delta	Miss	Sequence
4 - 13	976.4517	975.4444	975.4409	0.0035	0	AGFAGDDAPR (No match)
14 - 24	1198.7021	1197.6948	1197.6982	-0.0033	0	AVFPSIVGRPR (No match)
14 - 24	1198.7021	1197.6948	1197.6982	-0.0033	0	AVFPSIVGRPR (Ions score 54)
36 - 47	1354.6256	1353.6183	1353.6160	0.0023	1	DSYVGDEAQSKR (No match)
70 - 80	1515.7430	1514.7357	1514.7418	-0.0061	0	IWHHTFYNELR (No match)
70 - 80	1515.7430	1514.7357	1514.7418	-0.0061	0	IWHHTFYNELR (Ions score 74)
81 - 98	1954.0548	1953.0475	1953.0571	-0.0095	0	VAPEEHPVLLTEAPLNPK (No match)

81 - 98	1954.0548	1953.0475	1953.0571	-0.0095	0	**VAPEEHPVLLTEAPLNPK** (Ions score 122)
133 - 162	3199.6074	3198.6001	3198.6019	-0.0017	0	**TTGIVMDSGDGVTHTVPIYEGYALPHAILR** Oxidation (M) (No match)
182 - 191	1132.5282	1131.5209	1131.5196	0.0013	0	**GYSFTTTAER** (No match)
224 - 239	1790.8818	1789.8745	1789.8846	-0.0100	0	**SYELPDGQVITIGNER** (No match)
224 - 239	1790.8818	1789.8745	1789.8846	-0.0100	0	**SYELPDGQVITIGNER** (Ions score 119)
277 - 297	2215.0557	2214.0484	2214.0626	-0.0142	0	**DLYANTVLSGGTTMYPGIADR** (No match)
277 - 297	2231.0537	2230.0464	2230.0575	-0.0111	0	**DLYANTVLSGGTTMYPGIADR** Oxidation (M) (No match)
345 - 357	1516.7263	1515.7190	1515.6953	0.0237	0	**QEYDESGPSIVHR** (No match)
345 - 358	1644.7922	1643.7849	1643.7903	-0.0054	1	**QEYDESGPSIVHRK** (No match)

Mascot: http://www.matrixscience.com/

Spot 501

Mascot Search Results

Protein View

Match to: **gi|37183214** Score: **256** Expect: **4.8e-021**
TXNDC4 [Homo sapiens]

Nominal mass (M_r): **47297;** Calculated pI value: **5.13**
NCBI BLAST search of gi|37183214 against nr
Unformatted sequence string for pasting into other applications

Taxonomy: Homo sapiens

Fixed modifications: Carbamidomethyl (C)
Variable modifications: Oxidation (M)
Cleavage by Trypsin: cuts C-term side of KR unless next residue is P
Sequence Coverage: **26%**

Matched peptides shown in **Bold Red**

```
  1 MHPAVFLSLP DLRCSLLLLV TWVFTPVTTE ITSLATENID EILNNADVAL
 51 VNFYADWCRF SQMLHPIFEE ASDVIKEEFP NENQVVFARV DCDQHSDIAQ
101 RYRISKYPTL KLFRNGMMMK REYRGQRSVK ALADYIRQQK SDPIQEIRDL
151 AEITTLDRSK RNIIGYFEQK DSDNYRVFER VANILHDDCA FLSAFGDVSK
201 PERYSGDNII YKPPGHSAPD MVYLGAMTNF DVTYNWIQDK CVPLVREITF
251 ENGEELTEEG LPFLILFHMK EDTESLEIFQ NEVARQLISE KGTINFLHAD
301 CDKFRHPLLH IQKTPADCPV IAIDSFRHMY VFGDFKDVLI PGKLKQFVFD
351 LHSGKLHREF HHGPDPTDTA PGEQAQDVAS SPPESSFQKL APSEYRYTLL
401 RDRDEL
```

Residue Number Increasing Mass Decreasing Mass

Start - End	Observed	Mr(expt)	Mr(calc)	Delta	Miss	Sequence
77 - 89	1578.7045	1577.6972	1577.7473	-0.0501	0	EEFPNENQVVFAR (No match)
90 - 101	1443.5992	1442.5919	1442.6208	-0.0289	0	VDCDQHSDIAQR (No match)
162 - 170	1111.5278	1110.5205	1110.5709	-0.0504	0	NIIGYFEQK (No match)
181 - 203	2561.1555	2560.1482	2560.2379	-0.0897	0	VANILHDDCAFLSAFGDVSKPER (No match)
271 - 285	1779.7772	1778.7699	1778.8322	-0.0623	0	EDTESLEIFQNEVAR (No match)
271 - 285	1779.7772	1778.7699	1778.8322	-0.0623	0	EDTESLEIFQNEVAR (Ions score 102)

292 - 305	1693.7588	1692.7515	1692.8042	-0.0526	1	GTINFLHADCDKFR	(No match)
304 - 313	1288.7222	1287.7149	1287.7564	-0.0414	1	FRHPLLHIQK	(Ions score 9)
304 - 313	1288.7222	1287.7149	1287.7564	-0.0414	1	FRHPLLHIQK	(No match)
314 - 327	1561.7095	1560.7022	1560.7606	-0.0584	0	TPADCPVIAIDSFR	(No match)
314 - 327	1561.7095	1560.7022	1560.7606	-0.0584	0	TPADCPVIAIDSFR	(Ions score 63)

Mascot: http://www.matrixscience.com/

Spot 502

Mascot Search Results

Protein View

Match to: **gi|37183214** Score: **535** Expect: **6.1e-049**
TXNDC4 [Homo sapiens]

Nominal mass (M_r): **47297;** Calculated pI value: **5.13**
NCBI BLAST search of gi|37183214 against nr
Unformatted sequence string for pasting into other applications

Taxonomy: Homo sapiens

Fixed modifications: Carbamidomethyl (C)
Variable modifications: Oxidation (M)
Cleavage by Trypsin: cuts C-term side of KR unless next residue is P
Sequence Coverage: **28%**

Matched peptides shown in **Bold Red**

```
  1 MHPAVFLSLP DLRCSLLLLV TWVFTPVTTE ITSLATENID EILNNADVAL
 51 VNFYADWCRF SQMLHPIFEE ASDVIKEEFP NENQVVFARV DCDQHSDIAQ
101 RYRISKYPTL KLFRNGMMMK REYRGQRSVK ALADYIRQQK SDPIQEIRDL
151 AEITTLDRSK RNIIGYFEQK DSDNYRVFER VANILHDDCA FLSAFGDVSK
201 PERYSGDNII YKPPGHSAPD MVYLGAMTNF DVTYNWIQDK CVPLVREITF
251 ENGEELTEEG LPFLILFHMK EDTESLEIFQ NEVARQLISE KGTINFLHAD
301 CDKFRHPLLH IQKTPADCPV IAIDSFRHMY VFGDFKDVLI PGKLKQFVFD
351 LHSGKLHREF HHGPDPTDTA PGEQAQDVAS SPPESSFQKL APSEYRYTLL
401 RDRDEL
```

Residue Number Increasing Mass Decreasing Mass

Start - End	Observed	Mr(expt)	Mr(calc)	Delta	Miss	Sequence
77 - 89	1578.7496	1577.7423	1577.7473	-0.0050	0	EEFPNENQVVFAR (No match)
77 - 89	1578.7496	1577.7423	1577.7473	-0.0050	0	EEFPNENQVVFAR (Ions score 66)
90 - 101	1443.6259	1442.6186	1442.6208	-0.0022	0	VDCDQHSDIAQR (No match)
131 - 137	821.4491	820.4418	820.4443	-0.0024	0	ALADYIR (No match)
162 - 170	1111.5667	1110.5594	1110.5709	-0.0115	0	NIIGYFEQK (No match)
181 - 203	2561.2397	2560.2324	2560.2379	-0.0055	0	VANILHDDCAFLSAFGDVSKPER (No match)

181 - 203	2561.2397	2560.2324	2560.2379	-0.0055	0	**VANILHDDCAFLSAFGDVSKPER**	(Ions score 150)
271 - 285	1779.8313	1778.8240	1778.8322	-0.0082	0	**EDTESLEIFQNEVAR**	(No match)
271 - 285	1779.8313	1778.8240	1778.8322	-0.0082	0	**EDTESLEIFQNEVAR**	(Ions score 116)
292 - 305	1693.8081	1692.8008	1692.8042	-0.0033	1	**GTINFLHADCDKFR**	(No match)
304 - 313	1288.7573	1287.7500	1287.7564	-0.0063	1	**FRHPLLHIQK**	(No match)
304 - 313	1288.7573	1287.7500	1287.7564	-0.0063	1	**FRHPLLHIQK**	(Ions score 20)
314 - 327	1561.7579	1560.7506	1560.7606	-0.0100	0	**TPADCPVIAIDSFR**	(No match)
314 - 327	1561.7579	1560.7506	1560.7606	-0.0100	0	**TPADCPVIAIDSFR**	(Ions score 49)

Mascot: http://www.matrixscience.com/

Spot 503

MATRIX SCIENCE Mascot Search Results

Protein View

Match to: **gi|119577769** Score: **333** Expect: **9.6e-029**
vasodilator-stimulated phosphoprotein, isoform CRA_c [Homo sapiens]

Nominal mass (M_r): **39848**; Calculated pI value: **9.05**
NCBI BLAST search of gi|119577769 against nr
Unformatted sequence string for pasting into other applications

Taxonomy: Homo sapiens

Fixed modifications: Carbamidomethyl (C)
Variable modifications: Oxidation (M)
Cleavage by Trypsin: cuts C-term side of KR unless next residue is P
Sequence Coverage: **22%**

Matched peptides shown in **Bold Red**

```
  1 MSETVICSSR ATVMLYDDGN KRWLPAGTGP QAFSRVQIYH NPTANSFRVV
 51 GRKMQPDQQV VINCAIVRGV KYNQATPNFH QWRDARQVWG LNFGSKEDAA
101 QFAAGMASAL EALEGGGPPP PPALPTWSVP NGPSPEEVEQ QKRQQPGPSE
151 HIERRVSNAG GPPAPPAGGP PPPPGPPPPP GPPPPPGLPP SGVPAAAHGA
201 GGGPPPAPPL PAAQGPGGGG AGAPGLAAAI AGAKLRKVSK EEASGGPTAP
251 KAESGRSGGG GLMEEMNAML ARRRKATQVG EKTPKDESAN QEEPEARVPA
301 QSESVRRPWE KNSTTLPRMK SSSSVTTSET QPCTPSSSDY SDLQRVKQEL
351 LEEVKKELQK VKEEIIEAFV QELRKRGSP
```

Residue Number Increasing Mass Decreasing Mass

Start - End	Observed	Mr(expt)	Mr(calc)	Delta	Miss	Sequence
11 - 22	1382.6840	1381.6767	1381.6660	0.0108	1	ATVMLYDDGNKR (No match)
11 - 22	1398.6816	1397.6743	1397.6609	0.0135	1	ATVMLYDDGNKR Oxidation (M) (No match)
23 - 35	1387.7266	1386.7193	1386.7044	0.0149	0	WLPAGTGPQAFSR (No match)
23 - 35	1387.7266	1386.7193	1386.7044	0.0149	0	WLPAGTGPQAFSR (Ions score 56)
36 - 48	1546.7893	1545.7820	1545.7688	0.0133	0	VQIYHNPTANSFR (No match)
36 - 48	1546.7893	1545.7820	1545.7688	0.0133	0	VQIYHNPTANSFR (Ions score 72)
53 - 68	1899.0197	1898.0124	1897.9866	0.0259	1	KMQPDQQVVINCAIVR (No match)

54 - 68	1770.9150	1769.9077	1769.8916	0.0161	0	MQPDQQVVINCAIVR (No match)
54 - 68	1786.9059	1785.8986	1785.8865	0.0121	0	MQPDQQVVINCAIVR Oxidation (M) (No match)
72 - 83	1561.7472	1560.7399	1560.7222	0.0178	0	YNQATPNFHQWR (No match)
72 - 83	1561.7472	1560.7399	1560.7222	0.0178	0	YNQATPNFHQWR (Ions score 41)
144 - 154	1277.6368	1276.6295	1276.6160	0.0136	0	QQPGPSEHIER (Ions score 44)
144 - 154	1277.6368	1276.6295	1276.6160	0.0136	0	QQPGPSEHIER (No match)
144 - 155	1433.7389	1432.7316	1432.7171	0.0146	1	QQPGPSEHIERR (No match)
298 - 306	972.5232	971.5159	971.5036	0.0124	0	VPAQSESVR (No match)

Mascot: http://www.matrixscience.com/

Spot 504

Mascot Search Results

Protein View

Match to: **gi|4809279** Score: **212** Expect: **1.2e-016**
annexin VII isoform 2 [Homo sapiens]

Nominal mass (M_r): **52991**; Calculated pI value: **5.52**
NCBI BLAST search of gi|4809279 against nr
Unformatted sequence string for pasting into other applications

Taxonomy: Homo sapiens
Links to retrieve other entries containing this sequence from NCBI Entrez:
gi|55960473 from Homo sapiens
gi|58864775 from Homo sapiens
gi|119574879 from Homo sapiens

Fixed modifications: Carbamidomethyl (C)
Variable modifications: Oxidation (M)
Cleavage by Trypsin: cuts C-term side of KR unless next residue is P
Sequence Coverage: **18%**

Matched peptides shown in **Bold Red**

```
  1 MSYPGYPPTG YPPFPGYPPA GQESSFPPSG QYPYPSGFPP MGGGAYPQVP
 51 SSGYPGAGGY PAPGGYPAPG GYPGAPQPGG APSYPGVPPG QGFGVPPGGA
101 GFSGYPQPPS QSYGGGPAQV PLPGGFPGGQ MPSQYPGGQP TYPSQINTDS
151 FSSYPVFSPV SLDYSSEPAT VTQVTQGTIR PAANFDAIRD AEILRKAMKG
201 FGTDEQAIVD VVANRSNDQR QKIKAAFKTS YGKDLIKDLK SELSGNMEEL
251 ILALFMPPTY YDAWSLRKAM QGAGTQERVL IEILCTRTNQ EIREIVRCYQ
301 SEFGRDLEKD IRSDTSGHFE RLLVSMCQGN RDENQSINHQ MAQEDAQRLY
351 QAGEGRLGTD ESCFNMILAT RSFPQLRATM EAYSRMANRD LLSSVSREFS
401 GYVESGLKTI LQCALNRPAF FAERLYYAMK GAGTDDSTLV RIVVTRSEID
451 LVQIKQMFAQ MYQKTLGTMI AGDTSGDYRR LLLAIVGQ
```

Residue Number Increasing Mass Decreasing Mass

Start - End	Observed	Mr(expt)	Mr(calc)	Delta	Miss	Sequence
200 - 215	1690.8379	1689.8306	1689.8321	-0.0015	0	GFGTDEQAIVDVVANR (No match)

200 - 215	1690.8379	1689.8306	1689.8321	-0.0015	0	**GFGTDEQAIVDVVANR** (Ions score 100)
279 - 287	1116.6423	1115.6350	1115.6372	-0.0022	0	**VLIEILCTR** (No match)
298 - 305	1046.4305	1045.4232	1045.4287	-0.0055	0	**CYQSEFGR** (Ions score 4)
298 - 305	1046.4305	1045.4232	1045.4287	-0.0055	0	**CYQSEFGR** (No match)
332 - 348	2029.8685	2028.8612	2028.8555	0.0057	0	**DENQSINHQMAQEDAQR** Oxidation (M) (No match)
357 - 371	1743.8021	1742.7948	1742.7967	-0.0019	0	**LGTDESCFNMILATR** Oxidation (M) (No match)
357 - 371	1743.8021	1742.7948	1742.7967	-0.0019	0	**LGTDESCFNMILATR** Oxidation (M) (Ions score 12)
398 - 408	1215.5801	1214.5728	1214.5818	-0.0090	0	**EFSGYVESGLK** (No match)
409 - 424	1906.9974	1905.9901	1905.9883	0.0019	0	**TILQCALNRPAFFAER** (No match)
409 - 424	1906.9974	1905.9901	1905.9883	0.0019	0	**TILQCALNRPAFFAER** (Ions score 14)

Mascot: http://www.matrixscience.com/

Spot 505

Mascot Search Results

Protein View

Match to: **gi|4503529** Score: **97** Expect: **3.7e-005**
eukaryotic translation initiation factor 4A isoform 1 [Homo sapiens]

Nominal mass (M_r): **46353;** Calculated pI value: **5.32**
NCBI BLAST search of gi|4503529 against nr
Unformatted sequence string for pasting into other applications

Taxonomy: Homo sapiens
Links to retrieve other entries containing this sequence from NCBI Entrez:
gi|21450625 from Mus musculus
gi|40786436 from Rattus norvegicus
gi|77735407 from Bos taurus
gi|154147660 from Sus scrofa
gi|109113112 from Macaca mulatta
gi|149724255 from Equus caballus
gi|46397463 from Homo sapiens
gi|46397464 from Mus musculus
gi|109892471 from Bos taurus
gi|219403 from Homo sapiens
gi|2943740 from Mus musculus
gi|16307020 from Homo sapiens
gi|26346290 from Mus musculus
gi|29612606 from Mus musculus
gi|39793992 from Rattus norvegicus
gi|49522221 from Homo sapiens
gi|54696622 from Homo sapiens
gi|54696624 from Homo sapiens
gi|58864806 from Mus musculus
gi|61357581 from synthetic construct
gi|61357587 from synthetic construct
gi|74138684 from Mus musculus
gi|74138757 from Mus musculus
gi|74139346 from Mus musculus
gi|74139356 from Mus musculus
gi|74139407 from Mus musculus
gi|74141945 from Mus musculus
gi|74151432 from Mus musculus
gi|74151508 from Mus musculus

gi|74177669 from Mus musculus
gi|74185163 from Mus musculus
gi|74188940 from Mus musculus
gi|74189138 from Mus musculus
gi|74189153 from Mus musculus
gi|74193609 from Mus musculus
gi|74198159 from Mus musculus
gi|74201277 from Mus musculus
gi|74204464 from Mus musculus
gi|74208421 from Mus musculus
gi|74211475 from Mus musculus
gi|74220076 from Mus musculus
gi|74220399 from Mus musculus
gi|74268155 from Bos taurus
gi|85792232 from Sus scrofa
gi|90076216 from Macaca fascicularis
gi|119610573 from Homo sapiens
gi|119610574 from Homo sapiens
gi|123992423 from synthetic construct
gi|123999458 from synthetic construct
gi|149053069 from Rattus norvegicus
gi|227238 from Oryctolagus cuniculus

Fixed modifications: Carbamidomethyl (C)
Variable modifications: Oxidation (M)
Cleavage by Trypsin: cuts C-term side of KR unless next residue is P
Sequence Coverage: **16%**

Matched peptides shown in **Bold Red**

```
  1 MSASQDSRSR DNGPDGMEPE GVIESNWNEI VDSFDDMNLS ESLLRGIYAY
 51 GFEKPSAIQQ RAILPCIKGY DVIAQAQSGT GKTATFAISI LQQIELDLKA
101 TQALVLAPTR ELAQQIQKVV MALGDYMGAS CHACIGGTNV RAEVQKLQME
151 APHIIVGTPG RVFDMLNRRY LSPKYIKMFV LDEADEMLSR GFKDQIYDIF
201 QKLNSNTQVV LLSATMPSDV LEVTKKFMRD PIRILVKKEE LTLEGIRQFY
251 INVEREEWKL DTLCDLYETL TITQAVIFIN TRRKVDWLTE KMHARDFTVS
301 AMHGDMDQKE RDVIMREFRS GSSRVLITTD LLARGIDVQQ VSLVINYDLP
351 TNRENYIHRI GRGGRFGRKG VAINMVTEED KRTLRDIETF YNTSIEEMPL
401 NVADLI
```

Residue Number Increasing Mass Decreasing Mass

Start - End	Observed	Mr(expt)	Mr(calc)	Delta	Miss	Sequence	
46 - 61	1827.9003	1826.8930	1826.9314	-0.0384	0	GIYAYGFEKPSAIQQR	(No match)
46 - 61	1827.9003	1826.8930	1826.9314	-0.0384	0	GIYAYGFEKPSAIQQR	(Ions score 45)

147 - 161	1634.8346	1633.8273	1633.8609	-0.0336	0	**LQMEAPHIIVGTPGR**	Oxidation (M) (Ions score 7)
147 - 161	1634.8346	1633.8273	1633.8609	-0.0336	0	**LQMEAPHIIVGTPGR**	Oxidation (M) (No match)
162 - 169	1066.5236	1065.5163	1065.5389	-0.0226	1	**VFDMLNRR**	Oxidation (M) (No match)
248 - 255	1068.5297	1067.5224	1067.5399	-0.0175	0	**QFYINVER**	(No match)
248 - 255	1068.5297	1067.5224	1067.5399	-0.0175	0	**QFYINVER**	(No match)
335 - 353	2144.0776	2143.0703	2143.1273	-0.0569	0	**GIDVQQVSLVINYDLPTNR**	(No match)

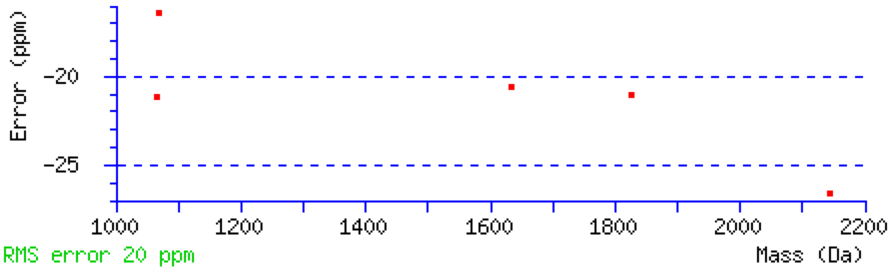

RMS error 20 ppm

Mascot: http://www.matrixscience.com/

Spot 506

{MATRIX SCIENCE} Mascot Search Results

Protein View

Match to: **gi|5453754** Score: **192** Expect: **1.2e-014**
NCK adaptor protein 1 [Homo sapiens]

Nominal mass (M_r): **43065**; Calculated pI value: **6.06**
NCBI BLAST search of gi|5453754 against nr
Unformatted sequence string for pasting into other applications

Taxonomy: Homo sapiens
Links to retrieve other entries containing this sequence from NCBI Entrez:
gi|109049236 from Macaca mulatta
gi|109049239 from Macaca mulatta
gi|114589375 from Pan troglodytes
gi|114589377 from Pan troglodytes
gi|114589379 from Pan troglodytes
gi|114589381 from Pan troglodytes
gi|114589383 from Pan troglodytes
gi|127962 from Homo sapiens
gi|35015 from Homo sapiens
gi|13623577 from Homo sapiens
gi|119599511 from Homo sapiens
gi|119599514 from Homo sapiens
gi|119599515 from Homo sapiens
gi|123992836 from synthetic construct

Fixed modifications: Carbamidomethyl (C)
Variable modifications: Oxidation (M)
Cleavage by Trypsin: cuts C-term side of KR unless next residue is P
Sequence Coverage: **21%**

Matched peptides shown in **Bold Red**

```
  1 MAEEVVVVAK FDYVAQQEQE LDIKKNERLW LLDDSKSWWR VRNSMNKTGF
 51 VPSNYVERKN SARKASIVKN LKDTLGIGKV KRKPSVPDSA SPADDSFVDP
101 GERLYDLNMP AYVKFNYMAE REDELSLIKG TKVIVMEKCS DGWWRGSYNG
151 QVGWFPSNYV TEEGDSPLGD HVGSLSEKLA AVVNNLNTGQ VLHVVQALYP
201 FSSSNDEELN FEKGDVMDVI EKPENDPEWW KCRKINGMVG LVPKNYVTVM
251 QNNPLTSGLE PSPPQCDYIR PSLTGKFAGN PWYYGKVTRH QAEMALNERG
301 HEGDFLIRDS ESSPNDFSVS LKAQGKNKHF KVQLKETVYC IGQRKFSTME
```

351 ELVEHYKKAP IFTSEQGEKL YLVKHLS

Residue Number Increasing Mass Decreasing Mass

Start - End	Observed	Mr(expt)	Mr(calc)	Delta	Miss	Sequence
11 - 25	1853.8741	1852.8668	1852.9206	-0.0538	1	FDYVAQQEQELDIKK (No match)
41 - 47	864.4778	863.4705	863.4283	0.0422	1	VRNSMNK Oxidation (M) (No match)
48 - 58	1268.5900	1267.5827	1267.6196	-0.0369	0	TGFVPSNYVER (Ions score 20)
48 - 58	1268.5900	1267.5827	1267.6196	-0.0369	0	TGFVPSNYVER (No match)
82 - 103	2329.0422	2328.0349	2328.0981	-0.0632	1	RKPSVPDSASPADDSFVDPGER (No match)
82 - 103	2329.0422	2328.0349	2328.0981	-0.0632	1	RKPSVPDSASPADDSFVDPGER (Ions score 34)
115 - 121	946.3875	945.3802	945.4014	-0.0212	0	FNYMAER Oxidation (M) (No match)
300 - 308	1043.4971	1042.4898	1042.5195	-0.0297	0	GHEGDFLIR (Ions score 19)
300 - 308	1043.4971	1042.4898	1042.5195	-0.0297	0	GHEGDFLIR (No match)
336 - 344	1125.5006	1124.4933	1124.5284	-0.0351	0	ETVYCIGQR (Ions score 30)
336 - 344	1125.5006	1124.4933	1124.5284	-0.0351	0	ETVYCIGQR (No match)

Mascot: http://www.matrixscience.com/

Spot 507

Mascot Search Results

Protein View

Match to: **gi|19743875** Score: **235** Expect: **6.1e-019**
fumarate hydratase precursor [Homo sapiens]

Nominal mass (M_r): **54773**; Calculated pI value: **8.85**
NCBI BLAST search of gi|19743875 against nr
Unformatted sequence string for pasting into other applications

Taxonomy: Homo sapiens
Links to retrieve other entries containing this sequence from NCBI Entrez:
gi|1730117 from Homo sapiens
gi|1545996 from Homo sapiens
gi|4097195 from Homo sapiens
gi|13111881 from Homo sapiens
gi|16924307 from Homo sapiens
gi|32880021 from Homo sapiens
gi|55959536 from Homo sapiens
gi|55960685 from Homo sapiens
gi|55961217 from Homo sapiens
gi|60655361 from synthetic construct
gi|60655363 from synthetic construct
gi|119590498 from Homo sapiens
gi|157929048 (no taxonomy information for this entry)

Fixed modifications: Carbamidomethyl (C)
Variable modifications: Oxidation (M)
Cleavage by Trypsin: cuts C-term side of KR unless next residue is P
Sequence Coverage: **21%**

Matched peptides shown in **Bold Red**

1 MYRALRLLAR SRPLVRAPAA ALASAPGLGG AAVPSFWPPN AARMASQNSF
51 RIEYDTFGEL K**VPNDKYYGA QTVR**STMNFK IGGVTERMPT PVIK**AFGILK**
101 **R**AAAEVNQDY GLDPKIANAI MKAADEVAEG KLNDHFPLVV WQTGSGTQTN
151 MNVNEVISNR AIEMLGGELG SK**IPVHPNDH VNK**SQSSNDT FPTAMHIAAA
201 IEVHEVLLPG LQKLHDALDA KSK**EFAQIIK** IGR**THTQDAV PLTLGQEFSG**
251 **YVQQVK**YAMT RIKAAMPR**IY ELAAGGTAVG TGLNTR**IGFA EKVAAKVAAL
301 TGLPFVTAPN KFEALAAHDA LVELSGAMNT TACSLMKIAN DIRFLGSGPR
351 SGLGELILPE NEPGSSIMPG KVNPTQCEAM TMVAAQVMGN HVAVTVGGSN

401 GHFELNVFKP MMIKNVLHSA R**LLGDASVSF TENCVVGIQA NTER**INKLMN
451 ESLMLVTALN PHIGYDKAAK **IAKTAHK**NGS TLKETAIELG YLTAEQFDEW
501 VKPKDMLGPK

Residue Number Increasing Mass Decreasing Mass

Start - End	Observed	Mr(expt)	Mr(calc)	Delta	Miss	Sequence
62 - 74	1510.7388	1509.7315	1509.7575	-0.0260	1	VPNDKYYGAQTVR (No match)
95 - 101	804.4954	803.4881	803.5017	-0.0136	1	AFGILKR (No match)
173 - 183	1269.6437	1268.6364	1268.6625	-0.0261	0	IPVHPNDHVNK (No match)
224 - 230	848.5075	847.5002	847.4803	0.0199	0	EFAQIIK (No match)
234 - 256	2546.2324	2545.2251	2545.2812	-0.0561	0	THTQDAVPLTLGQEFSGYVQQVK (No match)
269 - 286	1763.8959	1762.8886	1762.9213	-0.0326	0	IYELAAGGTAVGTGLNTR (Ions score 93)
269 - 286	1763.8959	1762.8886	1762.9213	-0.0326	0	IYELAAGGTAVGTGLNTR (No match)
422 - 444	2480.1506	2479.1433	2479.2012	-0.0579	0	LLGDASVSFTENCVVGIQANTER (Ions score 54)
422 - 444	2480.1506	2479.1433	2479.2012	-0.0579	0	LLGDASVSFTENCVVGIQANTER (No match)
471 - 477	768.5172	767.5099	767.4653	0.0446	1	IAKTAHK (No match)

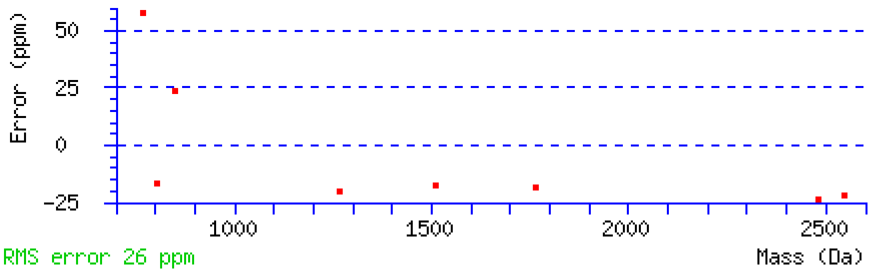

Mascot: http://www.matrixscience.com/

Spot 508

Mascot Search Results

Protein View

Match to: **gi|15277503** Score: **554** Expect: **7.7e-051**
ACTB protein [Homo sapiens]

Nominal mass (M_r): **40536**; Calculated pI value: **5.55**
NCBI BLAST search of gi|15277503 against nr
Unformatted sequence string for pasting into other applications

Taxonomy: Homo sapiens

Fixed modifications: Carbamidomethyl (C)
Variable modifications: Oxidation (M)
Cleavage by Trypsin: cuts C-term side of KR unless next residue is P
Sequence Coverage: **51%**

Matched peptides shown in **Bold Red**

```
  1 MCKAGFAGDD APRAVFPSIV GRPRHQGVMV GMGQKDSYVG DEAQSKRGIL
 51 TLKYPIEHGI VTNWDDMEKI WHHTFYNELR VAPEEHPVLL TEAPLNPKAN
101 LEKMTQIMFE TFNTPAMYVA IQAVLSLYAS GRTTGIVMDS GDGVTHTVPI
151 YEGYALPHAI LRLDLAGRDL TDYLMKILTE RGYSFTTTAE REIVRDIKEK
201 LCYVALDFEQ EMATAASSSS LEKSYELPDG QVITIGNERF RCPEALFQPS
251 FLGMESCGIH ETTFNSIMKC DVDIRKDLYA NTVLSGGTTM YPGIADRMQK
301 EITALAPSTM KIKIIAPPER KYSVWIGGSI LASLSTFQQM WISKQEYDES
351 GPSIVHRKCF
```

Residue Number Increasing Mass Decreasing Mass

Start - End	Observed	Mr(expt)	Mr(calc)	Delta	Miss	Sequence
4 - 13	976.4567	975.4494	975.4409	0.0085	0	AGFAGDDAPR (No match)
14 - 24	1198.7137	1197.7064	1197.6982	0.0083	0	AVFPSIVGRPR (Ions score 58)
14 - 24	1198.7137	1197.7064	1197.6982	0.0083	0	AVFPSIVGRPR (No match)
36 - 47	1354.6354	1353.6281	1353.6160	0.0121	1	DSYVGDEAQSKR (No match)
70 - 80	1515.7598	1514.7525	1514.7418	0.0107	0	IWHHTFYNELR (No match)
70 - 80	1515.7598	1514.7525	1514.7418	0.0107	0	IWHHTFYNELR (Ions score 82)
81 - 98	1954.0854	1953.0781	1953.0571	0.0211	0	VAPEEHPVLLTEAPLNPK (Ions score 94)

81 - 98	1954.0854	1953.0781	1953.0571	0.0211	0	**VAPEEHPVLLTEAPLNPK** (No match)
133 - 162	3183.6641	3182.6568	3182.6069	0.0499	0	**TTGIVMDSGDGVTHTVPIYEGYALPHAILR** (No match)
133 - 162	3199.6975	3198.6902	3198.6019	0.0884	0	**TTGIVMDSGDGVTHTVPIYEGYALPHAILR** Oxidation (M) (No match)
182 - 191	1132.5363	1131.5290	1131.5196	0.0094	0	**GYSFTTTAER** (No match)
201 - 223	2566.2234	2565.2161	2565.1614	0.0547	0	**LCYVALDFEQEMATAASSSSLEK** Oxidation (M) (No match)
224 - 239	1790.9100	1789.9027	1789.8846	0.0182	0	**SYELPDGQVITIGNER** (No match)
224 - 239	1790.9100	1789.9027	1789.8846	0.0182	0	**SYELPDGQVITIGNER** (Ions score 116)
276 - 297	2343.1951	2342.1878	2342.1576	0.0302	1	**KDLYANTVLSGGTTMYPGIADR** (No match)
276 - 297	2359.1926	2358.1853	2358.1525	0.0328	1	**KDLYANTVLSGGTTMYPGIADR** Oxidation (M) (No match)
277 - 297	2215.0923	2214.0850	2214.0626	0.0224	0	**DLYANTVLSGGTTMYPGIADR** (No match)
277 - 297	2231.0933	2230.0860	2230.0575	0.0285	0	**DLYANTVLSGGTTMYPGIADR** Oxidation (M) (No match)
277 - 297	2231.0933	2230.0860	2230.0575	0.0285	0	**DLYANTVLSGGTTMYPGIADR** Oxidation (M) (Ions score 44)
314 - 321	923.5744	922.5671	922.5600	0.0072	1	**IIAPPERK** (No match)
345 - 358	1644.8120	1643.8047	1643.7903	0.0144	1	**QEYDESGPSIVHRK** (No match)

Mascot: http://www.matrixscience.com/

Spot 509

Mascot Search Results

Protein View

Match to: **gi|13489054** Score: **129** Expect: **2.4e-008**
mitogen-activated protein kinase kinase 2 [Homo sapiens]

Nominal mass (M_r): **44681**; Calculated pI value: **6.12**
NCBI BLAST search of gi|13489054 against nr
Unformatted sequence string for pasting into other applications

Taxonomy: Homo sapiens
Links to retrieve other entries containing this sequence from NCBI Entrez:
gi|547915 from Homo sapiens
gi|12653403 from Homo sapiens
gi|17391417 from Homo sapiens
gi|119589668 from Homo sapiens
gi|123993851 from synthetic construct
gi|123996769 from synthetic construct

Fixed modifications: Carbamidomethyl (C)
Variable modifications: Oxidation (M)
Cleavage by Trypsin: cuts C-term side of KR unless next residue is P
Sequence Coverage: **19%**

Matched peptides shown in **Bold Red**

```
  1 MLARRKPVLP ALTINPTIAE GPSPTSEGAS EANLVDLQKK LEELELDEQQ
 51 KKRLEAFLTQ KAKVGELKDD DFERISELGA GNGGVVTKVQ HRPSGLIMAR
101 KLIHLEIKPA IRNQIIRELQ VLHECNSPYI VGFYGAFYSD GEISICMEHM
151 DGGSLDQVLK EAKRIPEEIL GKVSIAVLRG LAYLREKHQI MHRDVKPSNI
201 LVNSRGEIKL CDFGVSGQLI DSMANSFVGT RSYMAPERLQ GTHYSVQSDI
251 WSMGLSLVEL AVGRYPIPPP DAKELEAIFG RPVVDGEEGE PHSISPRPRP
301 PGRPVSGHGM DSRPAMAIFE LLDYIVNEPP PKLPNGVFTP DFQEFVNKCL
351 IKNPAERADL KMLTNHTFIK RSEVEEVDFA GWLCKTLRLN QPGTPTRTAV
401
```

Residue Number Increasing Mass Decreasing Mass

Start - End	Observed	Mr(expt)	Mr(calc)	Delta	Miss	Sequence
53 - 61	1105.6150	1104.6077	1104.6291	-0.0214	1	RLEAFLTQK (No match)
53 - 61	1105.6150	1104.6077	1104.6291	-0.0214	1	RLEAFLTQK (No match)
64 - 74	1322.6071	1321.5998	1321.6149	-0.0151	1	VGELKDDDFER (No match)
89 - 100	1364.6951	1363.6878	1363.7506	-0.0628	0	VQHRPSGLIMAR (No match)
89 - 100	1364.6951	1363.6878	1363.7506	-0.0628	0	VQHRPSGLIMAR (No match)
89 - 100	1380.7241	1379.7168	1379.7455	-0.0287	0	VQHRPSGLIMAR Oxidation (M) (No match)
89 - 100	1380.7241	1379.7168	1379.7455	-0.0287	0	VQHRPSGLIMAR Oxidation (M) (Ions score 10)
101 - 112	1430.8995	1429.8922	1429.9132	-0.0210	1	KLIHLEIKPAIR (Ions score 34)
164 - 172	1054.6067	1053.5994	1053.6182	-0.0188	1	RIPEEILGK (No match)
173 - 185	1430.8995	1429.8922	1429.8768	0.0154	1	VSIAVLRGLAYLR (No match)
194 - 205	1341.7278	1340.7205	1340.7412	-0.0206	0	DVKPSNILVNSR (No match)
194 - 205	1341.7278	1340.7205	1340.7412	-0.0206	0	DVKPSNILVNSR (No match)

Mascot: http://www.matrixscience.com/

Spot 510

Mascot Search Results

Protein View

Match to: **gi|119622488** Score: **166** Expect: **4.8e-012**
isocitrate dehydrogenase 2 (NADP+), mitochondrial, isoform CRA_b [Homo sapiens]

Nominal mass (M_r): **48071;** Calculated pI value: **8.26**
NCBI BLAST search of gi|119622488 against nr
Unformatted sequence string for pasting into other applications

Taxonomy: Homo sapiens

Fixed modifications: Carbamidomethyl (C)
Variable modifications: Oxidation (M)
Cleavage by Trypsin: cuts C-term side of KR unless next residue is P
Sequence Coverage: **31%**

Matched peptides shown in **Bold Red**

```
  1 MRCVSLLLDA DKRIKVAKPV VEMDGDEMTR IIWQFIKEKL ILPHVDIQLK
 51 YFDLGLPNRD QTDDQVTIDS ALATQKYSVA VKCATITPDE ARVEEFKLKK
101 MWKSPNGTIR NILGGTVFRE PIICKNIPRL VPGWTKPITI GRHAHGDQYK
151 ATDFVADRAG TFKMVFTPKD GSGVKEWEVY NFPAGGVGMG MYNTDESISG
201 FAHSCFQYAI QKKWPLYMST KNTILKAYDG RFKDIFQEIF DKHYKTDFDK
251 NKIWYEHRLI DDMVAQVLKS SGGFVWACKN YDGDVQSDIL AQGFGSLGLM
301 TSVLVCPDGK TIEAEAAHGT VTRHYREHQK GRPTSTNPIA SIFAWTRGLE
351 HRGKLDGNQD LIRFAQMLEK VCVETVESGA MTKDLAGCIH GLSNVKLNEH
401 FLNTTDFLDT IKSNLDRALG RQ
```

Residue Number Increasing Mass Decreasing Mass

Start - End	Observed	Mr(expt)	Mr(calc)	Delta	Miss	Sequence
40 - 50	1288.7885	1287.7812	1287.7914	-0.0102	0	LILPHVDIQLK (Ions score 26)
40 - 50	1288.7885	1287.7812	1287.7914	-0.0102	0	LILPHVDIQLK (No match)
51 - 59	1094.5613	1093.5540	1093.5556	-0.0016	0	YFDLGLPNR (No match)
51 - 59	1094.5613	1093.5540	1093.5556	-0.0016	0	YFDLGLPNR (Ions score 11)
51 - 76	2924.4229	2923.4156	2923.4199	-0.0043	1	YFDLGLPNRDQTDDQVTIDSALATQK (No match)
111 - 119	976.5561	975.5488	975.5501	-0.0013	0	NILGGTVFR (No match)

111 - 125	1716.9331	1715.9258	1715.9392	-0.0134	1	NILGGTVFREPIICK (No match)
111 - 125	1716.9331	1715.9258	1715.9392	-0.0134	1	NILGGTVFREPIICK (Ions score 0)
151 - 158	894.4360	893.4287	893.4243	0.0045	0	ATDFVADR (No match)
232 - 242	1429.7208	1428.7135	1428.7289	-0.0153	1	FKDIFQEIFDK (No match)
311 - 323	1355.6941	1354.6868	1354.6840	0.0028	0	TIEAEAAHGTVTR (No match)
353 - 363	1228.6603	1227.6530	1227.6571	-0.0041	1	GKLDGNQDLIR (No match)
353 - 363	1228.6603	1227.6530	1227.6571	-0.0041	1	GKLDGNQDLIR (Ions score 13)
355 - 370	1890.9729	1889.9656	1889.9669	-0.0012	1	LDGNQDLIRFAQMLEK (No match)
384 - 396	1383.6932	1382.6859	1382.6976	-0.0116	0	DLAGCIHGLSNVK (No match)
397 - 412	1920.9558	1919.9485	1919.9628	-0.0143	0	LNEHFLNTTDFLDTIK (No match)

Mascot: http://www.matrixscience.com/

Spot 511

Mascot Search Results

Protein View

Match to: **gi|58761500** Score: **134** Expect: **7.7e-009**
GTP-binding protein PTD004 isoform 1 [Homo sapiens]

Nominal mass (M_r): **44943**; Calculated pI value: **7.64**
NCBI BLAST search of gi|58761500 against nr
Unformatted sequence string for pasting into other applications

Taxonomy: Homo sapiens
Links to retrieve other entries containing this sequence from NCBI Entrez:
gi|109100089 from Macaca mulatta
gi|109100091 from Macaca mulatta
gi|114581806 from Pan troglodytes
gi|25453240 from Homo sapiens
gi|146387021 from Homo sapiens
gi|33150754 from Homo sapiens
gi|5531833 from Homo sapiens
gi|15277484 from Homo sapiens
gi|20810193 from Homo sapiens
gi|60688488 from Homo sapiens
gi|119631556 from Homo sapiens

Fixed modifications: Carbamidomethyl (C)
Variable modifications: Oxidation (M)
Cleavage by Trypsin: cuts C-term side of KR unless next residue is P
Sequence Coverage: **9%**

Matched peptides shown in **Bold Red**

```
  1 MPPKKGGDGI KPPPIIGRFG TSLKIGIVGL PNVGKSTFFN VLTNSQASAE
 51 NFPFCTIDPN ESRVPVPDER FDFLCQYHKP ASKIPAFLNV VDIAGLVKGA
101 HNGQGLGNAF LSHISACDGI FHLTRAFEDD DITHVEGSVD PIRDIEIIHE
151 ELQLKDEEMI GPIIDKLEKV AVRGGDKKLK PEYDIMCKVK SWVIDQKKPV
201 RFYHDWNDKE IEVLNKHLFL TSKPMVYLVN LSEKDYIRKK NKWLIKIKEW
251 VDKYDPGALV IPFSGALELK LQELSAEERQ KYLEANMTQS ALPKIIKAGF
301 AALQLEYFFT AGPDEVRAWT IRKGTKAPQA AGKIHTDFEK GFIMAEVMKY
351 EDFKEEGSEN AVKAAGKYRQ QGRNYIVEDG DIIFFKFNTP QQPKKK
```

Residue Number Increasing Mass Decreasing Mass

Start - End	Observed	Mr(expt)	Mr(calc)	Delta	Miss	Sequence
5 - 18	1404.7645	1403.7572	1403.8248	-0.0676	1	KGGDGIKPPPIIGR (Ions score 36)
5 - 18	1404.7645	1403.7572	1403.8248	-0.0676	1	KGGDGIKPPPIIGR (No match)
6 - 18	1276.6765	1275.6692	1275.7298	-0.0606	0	GGDGIKPPPIIGR (No match)
6 - 18	1276.6765	1275.6692	1275.7298	-0.0606	0	GGDGIKPPPIIGR (Ions score 17)
84 - 98	1568.8628	1567.8555	1567.9337	-0.0781	0	IPAFLNVVDIAGLVK (No match)
84 - 98	1568.8628	1567.8555	1567.9337	-0.0781	0	IPAFLNVVDIAGLVK (Ions score 51)
271 - 279	1074.4922	1073.4849	1073.5352	-0.0503	0	LQELSAEER (No match)

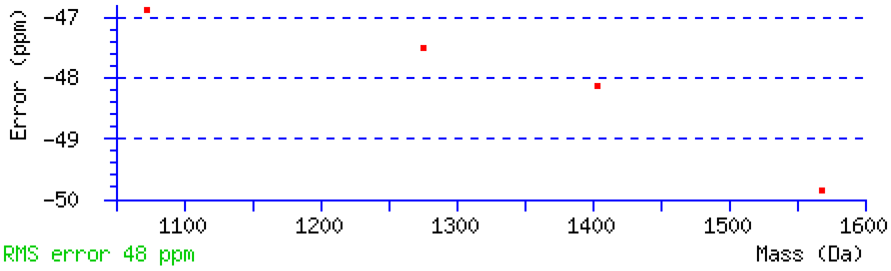

Mascot: http://www.matrixscience.com/

Spot 512

Mascot Search Results

Protein View

Match to: **gi|34452679** Score: **196** Expect: **4.8e-015**
growth-inhibiting protein 1 [Homo sapiens]

Nominal mass (M_r): **34670**; Calculated pI value: **5.69**
NCBI BLAST search of gi|34452679 against nr
Unformatted sequence string for pasting into other applications

Taxonomy: Homo sapiens

Fixed modifications: Carbamidomethyl (C)
Variable modifications: Oxidation (M)
Cleavage by Trypsin: cuts C-term side of KR unless next residue is P
Sequence Coverage: **13%**

Matched peptides shown in **Bold Red**

```
  1 MRLFGGNFAH QASVARVVGQ QGRGRAGIEA SLDVQYLMSA GANISTWVYS
 51 SPGRHEGQEP FLQWLMLLSN ESALPHVHTV SYGDDEDSLS SAYIQRVNTE
101 LMKAAARGLT LLFASGDSGA GCWSVSGRHQ FRPTFPASSP YVTTVGGTSF
151 QEPFLITNEI VDYISGGGFS NVFPRPSYQE EAVTKFLSSS PHLPPSSYFN
201 ASGRAYPDVA ALSDGYWVVS NRVPIPWVSG TSASTPVFGG ILSLINEHRI
251 LSGRPPLGFL NPRLYQQHGA GLFDVTRGCH ESCLDEEVEG QGFCSGPGWD
301 PVTGWGTPNF PALLKTLLNP
```

Residue Number Increasing Mass Decreasing Mass

Start - End	Observed	Mr(expt)	Mr(calc)	Delta	Miss	Sequence	
3 - 16	1474.6951	1473.6878	1473.7476	-0.0598	0	LFGGNFAHQASVAR	(Ions score 69)
3 - 16	1474.6951	1473.6878	1473.7476	-0.0598	0	LFGGNFAHQASVAR	(No match)
250 - 263	1536.8381	1535.8308	1535.8936	-0.0627	0	ILSGRPPLGFLNPR	(Ions score 35)
250 - 263	1536.8381	1535.8308	1535.8936	-0.0627	0	ILSGRPPLGFLNPR	(No match)
264 - 277	1604.7548	1603.7475	1603.8106	-0.0631	0	LYQQHGAGLFDVTR	(Ions score 64)
264 - 277	1604.7548	1603.7475	1603.8106	-0.0631	0	LYQQHGAGLFDVTR	(No match)

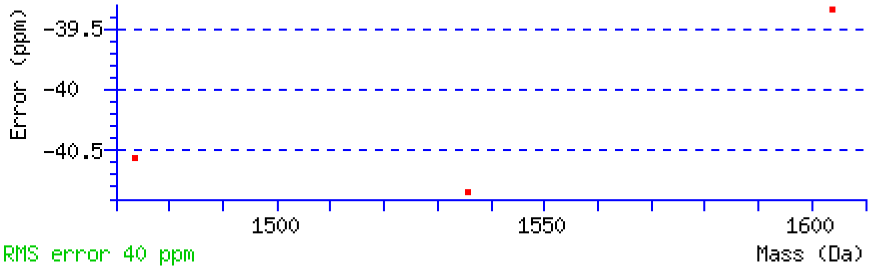

Mascot: http://www.matrixscience.com/

Spot 513

Mascot Search Results

Protein View

Match to: **gi|178277** Score: **189** Expect: **2.4e-014**
S-adenosylhomocysteine hydrolase

Nominal mass (M_r): **48254**; Calculated pI value: **6.03**
NCBI BLAST search of gi|178277 against nr
Unformatted sequence string for pasting into other applications

Taxonomy: Homo sapiens

Fixed modifications: Carbamidomethyl (C)
Variable modifications: Oxidation (M)
Cleavage by Trypsin: cuts C-term side of KR unless next residue is P
Sequence Coverage: **31%**

Matched peptides shown in **Bold Red**

```
  1 MSDKLPYKVA DIGLAAWGRK ALDIAENEMP GLMRMRERYS ASKPLKGARI
 51 AGCLHMTVET AVLIETLVTL GAEVQWSSCN IFSTQNHAAA AIAKAGIPVY
101 AWKGETDEEY LWCIEQTLYF KDGPLNMILD DGGDLTNLIH TKYPQLLPGI
151 RGISEETTTG VHNLYKMMAN GILKVPAINV NDSVTKSKFD NLYGCRESLI
201 DGIKRATDVM IAGKVAVVAG YGDVGKGCAQ ALRGFGARVI ITEIDPINAL
251 QAAMEGYEVT TMDEACQEGN IFVTTTGCID IILGRHFEQM KDDAIVCNIG
301 HFDVEIDVKW LNENAVEKVN IKPQVDRYRL KNGRRIILLA EGRLVNLGCA
351 MGHPSFVMSN SFTNQVMAQI ELWTHPDKYP VGVHFLPKKL DEAVAEAHLG
401 KLNVKLTKLT EKQAQYLGMS CDGPFKPDHY RY
```

Residue Number Increasing Mass Decreasing Mass

Start - End	Observed	Mr(expt)	Mr(calc)	Delta	Miss	Sequence
5 - 19	1629.8010	1628.7937	1628.9038	-0.1101	1	LPYKVADIGLAAWGR (No match)
9 - 19	1128.5994	1127.5921	1127.6087	-0.0165	0	VADIGLAAWGR (Ions score 16)
9 - 19	1128.5994	1127.5921	1127.6087	-0.0165	0	VADIGLAAWGR (No match)
21 - 34	1575.7444	1574.7371	1574.7432	-0.0061	0	ALDIAENEMPGLMR Oxidation (M) (No match)
21 - 34	1591.7369	1590.7296	1590.7381	-0.0085	0	ALDIAENEMPGLMR 2 Oxidation (M) (No match)
122 - 142	2268.0884	2267.0811	2267.1103	-0.0292	0	DGPLNMILDDGGDLTNLIHTK Oxidation (M) (No match)

143 - 151	1056.6071	1055.5998	1055.6127	-0.0129	0	YPQLLPGIR	(Ions score 20)
143 - 151	1056.6071	1055.5998	1055.6127	-0.0129	0	YPQLLPGIR	(No match)
152 - 166	1648.7917	1647.7844	1647.8103	-0.0259	0	GISEETTTGVHNLYK	(No match)
187 - 196	1259.5859	1258.5786	1258.5764	0.0022	1	SKFDNLYGCR	(No match)
187 - 196	1259.5859	1258.5786	1258.5764	0.0022	1	SKFDNLYGCR	(Ions score 19)
189 - 196	1044.4480	1043.4407	1043.4494	-0.0087	0	FDNLYGCR	(No match)
227 - 233	775.3874	774.3801	774.3806	-0.0005	0	GCAQALR	(No match)
319 - 327	1068.6057	1067.5984	1067.6087	-0.0103	0	VNIKPQVDR	(No match)
335 - 343	1040.6404	1039.6331	1039.6501	-0.0170	1	RIILLAEGR	(No match)
336 - 343	884.5503	883.5430	883.5490	-0.0060	0	IILLAEGR	(No match)
379 - 388	1156.6226	1155.6153	1155.6440	-0.0287	0	YPVGVHFLPK	(No match)
413 - 431	2286.0005	2284.9932	2284.9993	-0.0061	0	QAQYLGMSCDGPFKPDHYR	Oxidation (M) (No match)

Mascot: http://www.matrixscience.com/

Spot 514

{MATRIX SCIENCE} Mascot Search Results

Protein View

Match to: **gi|62087652** Score: **209** Expect: **2.4e-016**
proteasome 26S ATPase subunit 5 variant [Homo sapiens]

Nominal mass (M_r): **38907**; Calculated pI value: **6.07**
NCBI BLAST search of gi|62087652 against nr
Unformatted sequence string for pasting into other applications

Taxonomy: Homo sapiens

Fixed modifications: Carbamidomethyl (C)
Variable modifications: Oxidation (M)
Cleavage by Trypsin: cuts C-term side of KR unless next residue is P
Sequence Coverage: **27%**

Matched peptides shown in **Bold Red**

```
  1 KSYEMELEEG KAGSGLRQYY LSKIEELQLI VNDKSQNLRR LQAQRNELNA
 51 KVRLLREELQ LLQEQGSYVG EVVRAMDKKK VLVKVHPEGK FVVDVDKNID
101 INDVTPNCRV ALRNDSYTLH KILPNKVDPL VSLMMVEKVP DSTYEMIGGL
151 DKQIKEIKEV IELPVKHPEL FEALGIAQPK GVLLYGPPGT GKTLLARAVA
201 HHTDCTFIRV SGSELVQKFI GEGARMVREL FVMAREHAPS IIFMDEIDSI
251 GSSRLEGGSG GDSEVQRTML ELLNQLDGFE ATKNIKVIMA TNRIDILDSA
301 LLRPGRIDRK IEFPPPNEEA RLDLCLSHYY PQAFSKGSPL LVYPS
```

Residue Number Increasing Mass Decreasing Mass

Start - End	Observed	Mr(expt)	Mr(calc)	Delta	Miss	Sequence
54 - 74	2458.3564	2457.3491	2457.3226	0.0265	1	LLREELQLLQEQGSYVGEVVR (No match)
54 - 74	2458.3564	2457.3491	2457.3226	0.0265	1	LLREELQLLQEQGSYVGEVVR (No match)
91 - 109	2233.1033	2232.0960	2232.0844	0.0116	1	FVVDVDKNIDINDVTPNCR (No match)
91 - 109	2233.1033	2232.0960	2232.0844	0.0116	1	FVVDVDKNIDINDVTPNCR (Ions score 64)
198 - 209	1427.7324	1426.7251	1426.6775	0.0476	0	AVAHHTDCTFIR (No match)
236 - 254	2120.0093	2119.0020	2118.9891	0.0129	0	EHAPSIIFMDEIDSIGSSR Oxidation (M) (No match)
294 - 306	1438.8499	1437.8426	1437.8303	0.0123	0	IDILDSALLRPGR (No match)
294 - 306	1438.8499	1437.8426	1437.8303	0.0123	0	IDILDSALLRPGR (Ions score 16)

310 - 321	1426.7468	1425.7395	1425.7252	0.0144	1	KIEFPPPNEEAR	(Ions score 11)
310 - 321	1426.7468	1425.7395	1425.7252	0.0144	1	KIEFPPPNEEAR	(No match)
311 - 321	1298.6522	1297.6449	1297.6302	0.0147	0	IEFPPPNEEAR	(Ions score 22)
311 - 321	1298.6522	1297.6449	1297.6302	0.0147	0	IEFPPPNEEAR	(No match)

Mascot: http://www.matrixscience.com/

Spot 516

{MATRIX SCIENCE} Mascot Search Results

Protein View

Match to: **gi|15277503** Score: **485** Expect: **6.1e-044**
ACTB protein [Homo sapiens]

Nominal mass (M_r): **40536**; Calculated pI value: **5.55**
NCBI BLAST search of gi|15277503 against nr
Unformatted sequence string for pasting into other applications

Taxonomy: Homo sapiens

Fixed modifications: Carbamidomethyl (C)
Variable modifications: Oxidation (M)
Cleavage by Trypsin: cuts C-term side of KR unless next residue is P
Sequence Coverage: **32%**

Matched peptides shown in **Bold Red**

```
  1 MCKAGFAGDD APRAVFPSIV GRPRHQGVMV GMGQKDSYVG DEAQSKRGIL
 51 TLKYPIEHGI VTNWDDMEKI WHHTFYNELR VAPEEHPVLL TEAPLNPKAN
101 LEKMTQIMFE TFNTPAMYVA IQAVLSLYAS GRTTGIVMDS GDGVTHTVPI
151 YEGYALPHAI LRLDLAGRDL TDYLMKILTE RGYSFTTTAE REIVRDIKEK
201 LCYVALDFEQ EMATAASSSS LEKSYELPDG QVITIGNERF RCPEALFQPS
251 FLGMESCGIH ETTFNSIMKC DVDIRKDLYA NTVLSGGTTM YPGIADRMQK
301 EITALAPSTM KIKIIAPPER KYSVWIGGSI LASLSTFQQM WISKQEYDES
351 GPSIVHRKCF
```

Residue Number Increasing Mass Decreasing Mass

Start - End	Observed	Mr(expt)	Mr(calc)	Delta	Miss	Sequence
4 - 13	976.4369	975.4296	975.4409	-0.0113	0	AGFAGDDAPR (No match)
14 - 24	1198.6869	1197.6796	1197.6982	-0.0185	0	AVFPSIVGRPR (Ions score 58)
14 - 24	1198.6869	1197.6796	1197.6982	-0.0185	0	AVFPSIVGRPR (No match)
70 - 80	1515.7275	1514.7202	1514.7418	-0.0216	0	IWHHTFYNELR (No match)
81 - 98	1954.0366	1953.0293	1953.0571	-0.0277	0	VAPEEHPVLLTEAPLNPK (Ions score 117)
81 - 98	1954.0366	1953.0293	1953.0571	-0.0277	0	VAPEEHPVLLTEAPLNPK (No match)
182 - 191	1132.5109	1131.5036	1131.5196	-0.0160	0	GYSFTTTAER (No match)

182 - 191	1132.5109	1131.5036	1131.5196	-0.0160	0	GYSFTTTAER (Ions score 14)
224 - 239	1790.8618	1789.8545	1789.8846	-0.0300	0	SYELPDGQVITIGNER (Ions score 98)
224 - 239	1790.8618	1789.8545	1789.8846	-0.0300	0	SYELPDGQVITIGNER (No match)
277 - 297	2215.0515	2214.0442	2214.0626	-0.0184	0	DLYANTVLSGGTTMYPGIADR (No match)
277 - 297	2231.0488	2230.0415	2230.0575	-0.0160	0	DLYANTVLSGGTTMYPGIADR Oxidation (M) (No match)
314 - 320	795.4639	794.4566	794.4650	-0.0084	0	IIAPPER (No match)
345 - 357	1516.6929	1515.6856	1515.6953	-0.0097	0	QEYDESGPSIVHR (No match)
345 - 357	1516.6929	1515.6856	1515.6953	-0.0097	0	QEYDESGPSIVHR (Ions score 84)
345 - 358	1644.7828	1643.7755	1643.7903	-0.0148	1	QEYDESGPSIVHRK (No match)

Mascot: http://www.matrixscience.com/

Spot 517

{MATRIX SCIENCE} Mascot Search Results

Protein View

Match to: **gi|119572383** Score: **253** Expect: **9.6e-021**
Tu translation elongation factor, mitochondrial, isoform CRA_b [Homo sapiens]

Nominal mass (M_r): **37775**; Calculated pI value: **6.14**
NCBI BLAST search of gi|119572383 against nr
Unformatted sequence string for pasting into other applications

Taxonomy: Homo sapiens

Fixed modifications: Carbamidomethyl (C)
Variable modifications: Oxidation (M)
Cleavage by Trypsin: cuts C-term side of KR unless next residue is P
Sequence Coverage: **17%**

Matched peptides shown in **Bold Red**

```
  1 MTTMAAATLL RATPHFSGLA AGRTFLLQGL LRLLKAPALP LLCRGLAVEA
 51 KKTYVRDKPH VNVGTIGHVD HGKTTLTAAI TKILAEGGGA KFKKYEEIDN
101 APEERARGIT INAAHVEYST AARHYAHTDC PGHADYVKNM ITGTAPLDGC
151 ILVVAANDGP MPQTREHLLL ARQIGVEHVV VYVNKADAVQ DSEMVELVEL
201 EIRELLTEFG YKGEETPVIV GSALCALEGR DPELGLKSVQ KLLDAVDTYI
251 PVPARDLEKP FLLPVEAVYS VPGRGTVVTG TLERGILKKG DECELLGHSK
301 NIRTVVTGLG SQSGEQGLAE GWPYPGRSLV PSLCLTFAFL LPAPFPFPS
```

Residue Number Increasing Mass Decreasing Mass

Start - End	Observed	Mr(expt)	Mr(calc)	Delta	Miss	Sequence
94 - 105	1492.6986	1491.6913	1491.6841	0.0072	1	KYEEIDNAPEER (No match)
95 - 105	1364.6038	1363.5965	1363.5891	0.0074	0	YEEIDNAPEER (Ions score 34)
95 - 105	1364.6038	1363.5965	1363.5891	0.0074	0	YEEIDNAPEER (No match)
108 - 123	1673.8654	1672.8581	1672.8532	0.0049	0	GITINAAHVEYSTAAR (Ions score 46)
108 - 123	1673.8654	1672.8581	1672.8532	0.0049	0	GITINAAHVEYSTAAR (No match)
242 - 255	1542.8615	1541.8542	1541.8453	0.0089	0	LLDAVDTYIPVPAR (No match)
242 - 255	1542.8615	1541.8542	1541.8453	0.0089	0	LLDAVDTYIPVPAR (Ions score 36)
256 - 274	2129.1709	2128.1636	2128.1567	0.0069	0	DLEKPFLLPVEAVYSVPGR (No match)

256 - 274 2129.1709 2128.1636 2128.1567 0.0069 0 DLEKPFLLPVEAVYSVPGR (Ions score 74)

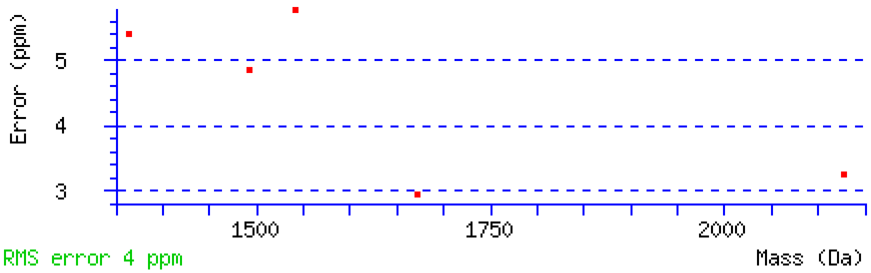

Mascot: http://www.matrixscience.com/

Spot 518

MATRIX SCIENCE Mascot Search Results

Protein View

Match to: **gi|15277503** Score: **393** Expect: **9.6e-035**
ACTB protein [Homo sapiens]

Nominal mass (M_r): **40536**; Calculated pI value: **5.55**
NCBI BLAST search of gi|15277503 against nr
Unformatted sequence string for pasting into other applications

Taxonomy: Homo sapiens

Fixed modifications: Carbamidomethyl (C)
Variable modifications: Oxidation (M)
Cleavage by Trypsin: cuts C-term side of KR unless next residue is P
Sequence Coverage: **30%**

Matched peptides shown in **Bold Red**

1 MCK**AGFAGDD APRAVFPSIV GRPR**HQGVMV GMGQKDSYVG DEAQSKRGIL
51 TLKYPIEHGI VTNWDDMEK**I WHHTFYNELR VAPEEHPVLL TEAPLNPK**AN
101 LEKMTQIMFE TFNTPAMYVA IQAVLSLYAS GRTTGIVMDS GDGVTHTVPI
151 YEGYALPHAI LRLDLAGRDL TDYLMKILTE R**GYSFTTTAE R**EIVRDIKEK
201 LCYVALDFEQ EMATAASSSS LEK**SYELPDG QVITIGNER**F RCPEALFQPS
251 FLGMESCGIH ETTFNSIMKC DVDIRK**DLYA NTVLSGGTTM YPGIADR**MQK
301 EITALAPSTM KIKIIAPPER KYSVWIGGSI LASLSTFQQM WISK**QEYDES**
351 **GPSIVHR**KCF

Residue Number Increasing Mass Decreasing Mass

Start - End	Observed	Mr(expt)	Mr(calc)	Delta	Miss	Sequence
4 - 13	976.4237	975.4164	975.4409	-0.0245	0	AGFAGDDAPR (No match)
14 - 24	1198.6692	1197.6619	1197.6982	-0.0362	0	AVFPSIVGRPR (No match)
14 - 24	1198.6692	1197.6619	1197.6982	-0.0362	0	AVFPSIVGRPR (Ions score 53)
70 - 80	1515.7010	1514.6937	1514.7418	-0.0481	0	IWHHTFYNELR (Ions score 27)
70 - 80	1515.7010	1514.6937	1514.7418	-0.0481	0	IWHHTFYNELR (No match)
81 - 98	1954.0072	1952.9999	1953.0571	-0.0571	0	VAPEEHPVLLTEAPLNPK (Ions score 115)
81 - 98	1954.0072	1952.9999	1953.0571	-0.0571	0	VAPEEHPVLLTEAPLNPK (No match)

182 - 191	1132.4961	1131.4888	1131.5196	-0.0308	0	GYSFTTTAER (No match)
182 - 191	1132.4961	1131.4888	1131.5196	-0.0308	0	GYSFTTTAER (Ions score 8)
224 - 239	1790.8346	1789.8273	1789.8846	-0.0572	0	SYELPDGQVITIGNER (Ions score 105)
224 - 239	1790.8346	1789.8273	1789.8846	-0.0572	0	SYELPDGQVITIGNER (No match)
277 - 297	2215.0049	2213.9976	2214.0626	-0.0650	0	DLYANTVLSGGTTMYPGIADR (No match)
277 - 297	2231.0159	2230.0086	2230.0575	-0.0489	0	DLYANTVLSGGTTMYPGIADR Oxidation (M) (No match)
345 - 357	1516.6729	1515.6656	1515.6953	-0.0297	0	QEYDESGPSIVHR (No match)

Mascot: http://www.matrixscience.com/

Spot 519

Mascot Search Results

Protein View

Match to: **gi|5031571** Score: **200** Expect: **1.9e-015**
actin-related protein 2 isoform b [Homo sapiens]

Nominal mass (M_r): **45017;** Calculated pI value: **6.30**
NCBI BLAST search of gi|5031571 against nr
Unformatted sequence string for pasting into other applications

Taxonomy: Homo sapiens
Links to retrieve other entries containing this sequence from NCBI Entrez:
gi|22122825 from Mus musculus
gi|156121073 (no taxonomy information for this entry)
gi|57093381 from Canis lupus familiaris
gi|109103168 from Macaca mulatta
gi|47117648 from Homo sapiens
gi|47117649 from Mus musculus
gi|17943200 from Bos taurus
gi|56966173 from Bos taurus
gi|56966193 from Bos taurus
gi|149243010 from Bos taurus
gi|149243032 from Bos taurus
gi|149243039 from Bos taurus
gi|149243046 from Bos taurus
gi|149243053 from Bos taurus
gi|149243060 from Bos taurus
gi|2282030 from Homo sapiens
gi|20380733 from Mus musculus
gi|26328619 from Mus musculus
gi|26353172 from Mus musculus
gi|56205119 from Mus musculus
gi|74190292 from Mus musculus
gi|119620314 from Homo sapiens
gi|119620316 from Homo sapiens
gi|119620318 from Homo sapiens
gi|119620319 from Homo sapiens
gi|148675879 from Mus musculus
gi|154425680 from Bos taurus

Fixed modifications: Carbamidomethyl (C)

Variable modifications: Oxidation (M)
Cleavage by Trypsin: cuts C-term side of KR unless next residue is P
Sequence Coverage: **25%**

Matched peptides shown in **Bold Red**

```
  1 MDSQGRKVVV CDNGTGFVKC GYAGSNFPEH IFPALVGRPI IRSTTKVGNI
 51 EIKDLMVGDE ASELRSMLEV NYPMENGIVR NWDDMKHLWD YTFGPEKLNI
101 DTRNCKILLT EPPMNPTKNR EKIVEVMFET YQFSGVYVAI QAVLTLYAQG
151 LLTGVVVDSG DGVTHICPVY EGFSLPHLTR RLDIAGRDIT RYLIKLLLLR
201 GYAFNHSADF ETVRMIKEKL CYVGYNIEQE QKLALETTVL VESYTLPDGR
251 IIKVGGERFE APEALFQPHL INVEGVGVAE LLFNTIQAAD IDTRSEFYKH
301 IVLSGGSTMY PGLPSRLERE LKQLYLERVL KGDVEKLSKF KIRIEDPPRR
351 KHMVFLGGAV LADIMKDKDN FWMTRQEYQE KGVRVLEKLG VTVR
```

Residue Number Increasing Mass Decreasing Mass

Start - End	Observed	Mr(expt)	Mr(calc)	Delta	Miss	Sequence
20 - 42	2571.3535	2570.3462	2570.3215	0.0247	0	CGYAGSNFPEHIFPALVGRPIIR (No match)
20 - 42	2571.3535	2570.3462	2570.3215	0.0247	0	CGYAGSNFPEHIFPALVGRPIIR (Ions score 27)
66 - 80	1783.8530	1782.8457	1782.8280	0.0178	0	SMLEVNYPMENGIVR 2 Oxidation (M) (No match)
66 - 80	1783.8530	1782.8457	1782.8280	0.0178	0	SMLEVNYPMENGIVR 2 Oxidation (M) (Ions score 5)
201 - 214	1613.7476	1612.7403	1612.7269	0.0134	0	GYAFNHSADFETVR (Ions score 42)
201 - 214	1613.7476	1612.7403	1612.7269	0.0134	0	GYAFNHSADFETVR (No match)
220 - 232	1643.7845	1642.7772	1642.7660	0.0112	0	LCYVGYNIEQEQK (No match)
233 - 250	1977.0723	1976.0650	1976.0466	0.0185	0	LALETTVLVESYTLPDGR (No match)
233 - 250	1977.0723	1976.0650	1976.0466	0.0185	0	LALETTVLVESYTLPDGR (Ions score 55)
300 - 316	1787.9244	1786.9171	1786.9035	0.0136	0	HIVLSGGSTMYPGLPSR Oxidation (M) (No match)

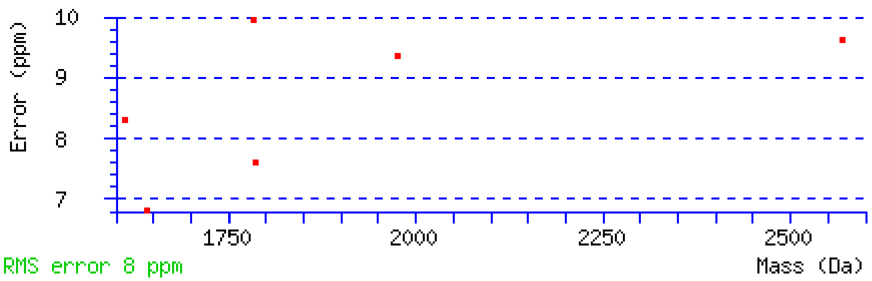

Mascot: http://www.matrixscience.com/

Spot 522

Mascot Search Results

Protein View

Match to: **gi|15277503** Score: **489** Expect: **2.4e-044**
ACTB protein [Homo sapiens]

Nominal mass (M_r): **40536**; Calculated pI value: **5.55**
NCBI BLAST search of gi|15277503 against nr
Unformatted sequence string for pasting into other applications

Taxonomy: Homo sapiens

Fixed modifications: Carbamidomethyl (C)
Variable modifications: Oxidation (M)
Cleavage by Trypsin: cuts C-term side of KR unless next residue is P
Sequence Coverage: **31%**

Matched peptides shown in **Bold Red**

1 MCK**AGFAGDD APRAVFPSIV GRPR**HQGVMV GMGQK**DSYVG DEAQSKR**GIL
51 TLKYPIEHGI VTNWDDMEKI WHHTFYNELR **VAPEEHPVLL TEAPLNPK**AN
101 LEKMTQIMFE TFNTPAMYVA IQAVLSLYAS GRTTGIVMDS GDGVTHTVPI
151 YEGYALPHAI LRLDLAGRDL TDYLMKILTE R**GYSFTTTAE R**EIVRDIKEK
201 LCYVALDFEQ EMATAASSSS LEK**SYELPDG QVITIGNER**F RCPEALFQPS
251 FLGMESCGIH ETTFNSIMKC DVDIR**KDLYA NTVLSGGTTM YPGIADR**MQK
301 EITALAPSTM KIKIIAPPER KYSVWIGGSI LASLSTFQQM WISK**QEYDES**
351 **GPSIVHRK**CF

Residue Number Increasing Mass Decreasing Mass

Start - End	Observed	Mr(expt)	Mr(calc)	Delta	Miss	Sequence	
4 - 13	976.4034	975.3961	975.4409	-0.0448	0	AGFAGDDAPR	(No match)
14 - 24	1198.6472	1197.6399	1197.6982	-0.0582	0	AVFPSIVGRPR	(No match)
14 - 24	1198.6472	1197.6399	1197.6982	-0.0582	0	AVFPSIVGRPR	(Ions score 55)
36 - 47	1354.5656	1353.5583	1353.6160	-0.0577	1	DSYVGDEAQSKR	(No match)
81 - 98	1953.9741	1952.9668	1953.0571	-0.0902	0	VAPEEHPVLLTEAPLNPK	(No match)
81 - 98	1953.9741	1952.9668	1953.0571	-0.0902	0	VAPEEHPVLLTEAPLNPK	(Ions score 128)
182 - 191	1132.4720	1131.4647	1131.5196	-0.0549	0	GYSFTTTAER	(No match)

224 - 239	1790.8082	1789.8009	1789.8846	-0.0836	0	**SYELPDGQVITIGNER**	(Ions score 133)
224 - 239	1790.8082	1789.8009	1789.8846	-0.0836	0	**SYELPDGQVITIGNER**	(No match)
276 - 297	2359.0469	2358.0396	2358.1525	-0.1129	1	**KDLYANTVLSGGTTMYPGIADR**	Oxidation (M) (No match)
277 - 297	2230.9607	2229.9534	2230.0575	-0.1041	0	**DLYANTVLSGGTTMYPGIADR**	Oxidation (M) (No match)
277 - 297	2230.9607	2229.9534	2230.0575	-0.1041	0	**DLYANTVLSGGTTMYPGIADR**	Oxidation (M) (Ions score 28)
345 - 357	1516.6348	1515.6275	1515.6953	-0.0678	0	**QEYDESGPSIVHR**	(No match)
345 - 357	1516.6348	1515.6275	1515.6953	-0.0678	0	**QEYDESGPSIVHR**	(Ions score 53)
345 - 358	1644.7235	1643.7162	1643.7903	-0.0741	1	**QEYDESGPSIVHRK**	(No match)

Mascot: http://www.matrixscience.com/

Spot 523

Mascot Search Results

Protein View

Match to: **gi|126030593** Score: **514** Expect: **7.7e-047**
Chain A, Crystal Structure Of Human Paics, A Bifunctional Carboxylase And Synthetase In Purine Bios

Nominal mass (M_r): **47650;** Calculated pI value: **6.67**
NCBI BLAST search of gi|126030593 against nr
Unformatted sequence string for pasting into other applications

Taxonomy: Homo sapiens

Fixed modifications: Carbamidomethyl (C)
Variable modifications: Oxidation (M)
Cleavage by Trypsin: cuts C-term side of KR unless next residue is P
Sequence Coverage: **35%**

Matched peptides shown in **Bold Red**

```
  1 XATAEVLNIG KKLYEGKTKE VYELLDSPGK VLLQSKDQIT AGNAARKNHL
 51 EGKAAISNKI TSCIFQLLQE AGIKTAFTRK CGETAFIAPQ CEXIPIEWVC
101 RRIATGSFLK RNPGVKEGYK FYPPKVELFF KDDANNDPQW SEEQLIAAKF
151 CFAGLLIGQT EVDIXSHATQ AIFEILEKSW LPQNCTLVDX KIEFGVDVTT
201 KEIVLADVID NDSWRLWPSG DRSQQKDKQS YRDLKEVTPE GLQXVKKNFE
251 WVAERVELLL KSESQCRVVV LXGSTSDLGH CEKIKKACGN FGIPCELRVT
301 SAHKGPDETL RIKAEYEGDG IPTVFVAVAG RSNGLGPVXS GNTAYPVISC
351 PPLTPDWGVQ DVWSSLRLPS GLGCSTVLSP EGSAQFAAQI FGLSNHLVWS
401 KLRASILNTW ISLKQADKKI RECNL
```

Residue Number Increasing Mass Decreasing Mass

Start - End	Observed	Mr(expt)	Mr(calc)	Delta	Miss	Sequence
37 - 46	1016.5259	1015.5186	1015.5046	0.0140	0	DQITAGNAAR (No match)
54 - 74	2305.2727	2304.2654	2304.2511	0.0144	1	AAISNKITSCIFQLLQEAGIK (No match)
60 - 74	1720.9462	1719.9389	1719.9229	0.0160	0	ITSCIFQLLQEAGIK (Ions score 72)
60 - 74	1720.9462	1719.9389	1719.9229	0.0160	0	ITSCIFQLLQEAGIK (No match)
103 - 111	992.5930	991.5857	991.5814	0.0043	1	IATGSFLKR (No match)
112 - 120	991.5163	990.5090	990.5134	-0.0043	1	NPGVKEGYK (No match)

117 - 125	1128.5851	1127.5778	1127.5651	0.0127	1	EGYKFYPPK (No match)
202 - 215	1644.8406	1643.8333	1643.8154	0.0179	0	EIVLADVIDNDSWR (No match)
216 - 222	830.4225	829.4152	829.4082	0.0070	0	LWPSGDR (No match)
247 - 255	1178.6068	1177.5995	1177.5879	0.0116	1	KNFEWVAER (No match)
248 - 255	1050.5112	1049.5039	1049.4930	0.0109	0	NFEWVAER (No match)
248 - 255	1050.5112	1049.5039	1049.4930	0.0109	0	NFEWVAER (Ions score 20)
248 - 261	1745.9761	1744.9688	1744.9511	0.0177	1	NFEWVAERVELLLK (No match)
286 - 298	1521.7473	1520.7400	1520.7227	0.0173	1	KACGNFGIPCELR (No match)
287 - 298	1393.6489	1392.6416	1392.6278	0.0138	0	ACGNFGIPCELR (Ions score 63)
287 - 298	1393.6489	1392.6416	1392.6278	0.0138	0	ACGNFGIPCELR (No match)
299 - 311	1410.7434	1409.7361	1409.7262	0.0099	1	VTSAHKGPDETLR (No match)
299 - 311	1410.7434	1409.7361	1409.7262	0.0099	1	VTSAHKGPDETLR (Ions score 18)
312 - 331	2092.1235	2091.1162	2091.0999	0.0163	1	IKAEYEGDGIPTVFVAVAGR (No match)
312 - 331	2092.1235	2091.1162	2091.0999	0.0163	1	IKAEYEGDGIPTVFVAVAGR (Ions score 133)
314 - 331	1850.9465	1849.9392	1849.9209	0.0183	0	AEYEGDGIPTVFVAVAGR (No match)
402 - 414	1514.9229	1513.9156	1513.8980	0.0176	1	LRASILNTWISLK (No match)
404 - 414	1245.7316	1244.7243	1244.7128	0.0115	0	ASILNTWISLK (No match)

Mascot: http://www.matrixscience.com/

Spot 524

Mascot Search Results

Protein View

Match to: **gi|15277503** Score: **526** Expect: **4.8e-048**
ACTB protein [Homo sapiens]

Nominal mass (M_r): **40536**; Calculated pI value: **5.55**
NCBI BLAST search of gi|15277503 against nr
Unformatted sequence string for pasting into other applications

Taxonomy: Homo sapiens

Fixed modifications: Carbamidomethyl (C)
Variable modifications: Oxidation (M)
Cleavage by Trypsin: cuts C-term side of KR unless next residue is P
Sequence Coverage: **49%**

Matched peptides shown in **Bold Red**

```
  1 MCKAGFAGDD APRAVFPSIV GRPRHQGVMV GMGQKDSYVG DEAQSKRGIL
 51 TLKYPIEHGI VTNWDDMEKI WHHTFYNELR VAPEEHPVLL TEAPLNPKAN
101 LEKMTQIMFE TFNTPAMYVA IQAVLSLYAS GRTTGIVMDS GDGVTHTVPI
151 YEGYALPHAI LRLDLAGRDL TDYLMKILTE RGYSFTTTAE REIVRDIKEK
201 LCYVALDFEQ EMATAASSSS LEKSYELPDG QVITIGNERF RCPEALFQPS
251 FLGMESCGIH ETTFNSIMKC DVDIRKDLYA NTVLSGGTTM YPGIADRMQK
301 EITALAPSTM KIKIIAPPER KYSVWIGGSI LASLSTFQQM WISKQEYDES
351 GPSIVHRKCF
```

Residue Number Increasing Mass Decreasing Mass

Start - End	Observed	Mr(expt)	Mr(calc)	Delta	Miss	Sequence
4 - 13	976.4631	975.4558	975.4409	0.0149	0	AGFAGDDAPR (No match)
14 - 24	1198.7153	1197.7080	1197.6982	0.0099	0	AVFPSIVGRPR (No match)
14 - 24	1198.7153	1197.7080	1197.6982	0.0099	0	AVFPSIVGRPR (Ions score 45)
36 - 47	1354.6365	1353.6292	1353.6160	0.0132	1	DSYVGDEAQSKR (No match)
70 - 80	1515.7570	1514.7497	1514.7418	0.0079	0	IWHHTFYNELR (No match)
70 - 80	1515.7570	1514.7497	1514.7418	0.0079	0	IWHHTFYNELR (Ions score 77)
81 - 98	1954.0725	1953.0652	1953.0571	0.0082	0	VAPEEHPVLLTEAPLNPK (Ions score 118)

81 - 98	1954.0725	1953.0652	1953.0571	0.0082	0	**VAPEEHPVLLTEAPLNPK** (No match)
133 - 162	3183.6201	3182.6128	3182.6069	0.0059	0	**TTGIVMDSGDGVTHTVPIYEGYALPHAILR** (No match)
133 - 162	3199.6438	3198.6365	3198.6019	0.0347	0	**TTGIVMDSGDGVTHTVPIYEGYALPHAILR** Oxidation (M) (No match)
182 - 191	1132.5386	1131.5313	1131.5196	0.0117	0	**GYSFTTTAER** (No match)
182 - 195	1629.8241	1628.8168	1628.8158	0.0011	1	**GYSFTTTAEREIVR** (No match)
201 - 223	2566.1880	2565.1807	2565.1614	0.0193	0	**LCYVALDFEQEMATAASSSSLEK** Oxidation (M) (No match)
224 - 239	1790.8983	1789.8910	1789.8846	0.0065	0	**SYELPDGQVITIGNER** (Ions score 115)
224 - 239	1790.8983	1789.8910	1789.8846	0.0065	0	**SYELPDGQVITIGNER** (No match)
276 - 297	2359.1714	2358.1641	2358.1525	0.0116	1	**KDLYANTVLSGGTTMYPGIADR** Oxidation (M) (No match)
277 - 297	2215.0752	2214.0679	2214.0626	0.0053	0	**DLYANTVLSGGTTMYPGIADR** (No match)
277 - 297	2231.0769	2230.0696	2230.0575	0.0121	0	**DLYANTVLSGGTTMYPGIADR** Oxidation (M) (No match)
312 - 320	1036.6652	1035.6579	1035.6440	0.0139	1	**IKIIAPPER** (No match)
314 - 321	923.5791	922.5718	922.5600	0.0119	1	**IIAPPERK** (No match)

Mascot: http://www.matrixscience.com/

Spot 525

Mascot Search Results

Protein View

Match to: **gi|15277503** Score: **680** Expect: **1.9e-063**
ACTB protein [Homo sapiens]

Nominal mass (M_r): **40536**; Calculated pI value: **5.55**
NCBI BLAST search of gi|15277503 against nr
Unformatted sequence string for pasting into other applications

Taxonomy: Homo sapiens

Fixed modifications: Carbamidomethyl (C)
Variable modifications: Oxidation (M)
Cleavage by Trypsin: cuts C-term side of KR unless next residue is P
Sequence Coverage: **65%**

Matched peptides shown in **Bold Red**

```
  1 MCKAGFAGDD APRAVFPSIV GRPRHQGVMV GMGQKDSYVG DEAQSKRGIL
 51 TLKYPIEHGI VTNWDDMEKI WHHTFYNELR VAPEEHPVLL TEAPLNPKAN
101 LEKMTQIMFE TFNTPAMYVA IQAVLSLYAS GRTTGIVMDS GDGVTHTVPI
151 YEGYALPHAI LRLDLAGRDL TDYLMKILTE RGYSFTTTAE REIVRDIKEK
201 LCYVALDFEQ EMATAASSSS LEKSYELPDG QVITIGNERF RCPEALFQPS
251 FLGMESCGIH ETTFNSIMKC DVDIRKDLYA NTVLSGGTTM YPGIADRMQK
301 EITALAPSTM KIKIIAPPER KYSVWIGGSI LASLSTFQQM WISKQEYDES
351 GPSIVHRKCF
```

Residue Number Increasing Mass Decreasing Mass

Start - End	Observed	Mr(expt)	Mr(calc)	Delta	Miss	Sequence
4 - 13	976.4385	975.4312	975.4409	-0.0097	0	AGFAGDDAPR (No match)
14 - 24	1198.6920	1197.6847	1197.6982	-0.0134	0	AVFPSIVGRPR (No match)
14 - 24	1198.6920	1197.6847	1197.6982	-0.0134	0	AVFPSIVGRPR (Ions score 71)
25 - 35	1187.5575	1186.5502	1186.5586	-0.0084	0	HQGVMVGMGQK Oxidation (M) (No match)
36 - 47	1354.6063	1353.5990	1353.6160	-0.0170	1	DSYVGDEAQSKR (No match)
70 - 80	1515.7280	1514.7207	1514.7418	-0.0211	0	IWHHTFYNELR (No match)
70 - 80	1515.7280	1514.7207	1514.7418	-0.0211	0	IWHHTFYNELR (Ions score 69)

81 - 98	1954.0477	1953.0404	1953.0571	-0.0166	0	VAPEEHPVLLTEAPLNPK (No match)
81 - 98	1954.0477	1953.0404	1953.0571	-0.0166	0	VAPEEHPVLLTEAPLNPK (Ions score 90)
133 - 162	3183.5798	3182.5725	3182.6069	-0.0344	0	TTGIVMDSGDGVTHTVPIYEGYALPHAILR (No match)
133 - 162	3199.5852	3198.5779	3198.6019	-0.0239	0	TTGIVMDSGDGVTHTVPIYEGYALPHAILR Oxidation (M) (No match)
169 - 176	998.4283	997.4210	997.4790	-0.0580	0	DLTDYLMK (No match)
182 - 191	1132.5132	1131.5059	1131.5196	-0.0137	0	GYSFTTTAER (Ions score 71)
182 - 191	1132.5132	1131.5059	1131.5196	-0.0137	0	GYSFTTTAER (No match)
201 - 223	2550.1887	2549.1814	2549.1665	0.0149	0	LCYVALDFEQEMATAASSSSLEK (No match)
224 - 239	1790.8694	1789.8621	1789.8846	-0.0224	0	SYELPDGQVITIGNER (Ions score 124)
224 - 239	1790.8694	1789.8621	1789.8846	-0.0224	0	SYELPDGQVITIGNER (No match)
270 - 275	777.3489	776.3416	776.3487	-0.0070	0	CDVDIR (No match)
270 - 276	905.4405	904.4332	904.4436	-0.0104	1	CDVDIRK (No match)
276 - 297	2343.1379	2342.1306	2342.1576	-0.0270	1	KDLYANTVLSGGTTMYPGIADR (No match)
276 - 297	2359.1523	2358.1450	2358.1525	-0.0075	1	KDLYANTVLSGGTTMYPGIADR Oxidation (M) (No match)
277 - 297	2215.0403	2214.0330	2214.0626	-0.0296	0	DLYANTVLSGGTTMYPGIADR (No match)
277 - 297	2231.0542	2230.0469	2230.0575	-0.0106	0	DLYANTVLSGGTTMYPGIADR Oxidation (M) (No match)
312 - 320	1036.6412	1035.6339	1035.6440	-0.0101	1	IKIIAPPER (No match)
314 - 320	795.4681	794.4608	794.4650	-0.0042	0	IIAPPER (No match)
314 - 321	923.5593	922.5520	922.5600	-0.0079	1	IIAPPERK (No match)
321 - 344	2730.3103	2729.3030	2729.4250	-0.1220	1	KYSVWIGGSILASLSTFQQMWISK (No match)
345 - 357	1516.7080	1515.7007	1515.6953	0.0054	0	QEYDESGPSIVHR (No match)
345 - 358	1644.7738	1643.7665	1643.7903	-0.0238	1	QEYDESGPSIVHRK (No match)

Mascot: http://www.matrixscience.com/

Spot 526

Mascot Search Results

Protein View

Match to: **gi|15277503** Score: **533** Expect: **9.6e-049**
ACTB protein [Homo sapiens]

Nominal mass (M_r): **40536**; Calculated pI value: **5.55**
NCBI BLAST search of gi|15277503 against nr
Unformatted sequence string for pasting into other applications

Taxonomy: Homo sapiens

Fixed modifications: Carbamidomethyl (C)
Variable modifications: Oxidation (M)
Cleavage by Trypsin: cuts C-term side of KR unless next residue is P
Sequence Coverage: **45%**

Matched peptides shown in **Bold Red**

```
  1 MCKAGFAGDD APRAVFPSIV GRPRHQGVMV GMGQKDSYVG DEAQSKRGIL
 51 TLKYPIEHGI VTNWDDMEKI WHHTFYNELR VAPEEHPVLL TEAPLNPKAN
101 LEKMTQIMFE TFNTPAMYVA IQAVLSLYAS GRTTGIVMDS GDGVTHTVPI
151 YEGYALPHAI LRLDLAGRDL TDYLMKILTE RGYSFTTTAE REIVRDIKEK
201 LCYVALDFEQ EMATAASSSS LEKSYELPDG QVITIGNERF RCPEALFQPS
251 FLGMESCGIH ETTFNSIMKC DVDIRKDLYA NTVLSGGTTM YPGIADRMQK
301 EITALAPSTM KIKIIAPPER KYSVWIGGSI LASLSTFQQM WISKQEYDES
351 GPSIVHRKCF
```

Residue Number Increasing Mass Decreasing Mass

Start - End	Observed	Mr(expt)	Mr(calc)	Delta	Miss	Sequence
4 - 13	976.4210	975.4137	975.4409	-0.0272	0	AGFAGDDAPR (No match)
14 - 24	1198.6788	1197.6715	1197.6982	-0.0266	0	AVFPSIVGRPR (Ions score 68)
14 - 24	1198.6788	1197.6715	1197.6982	-0.0266	0	AVFPSIVGRPR (No match)
36 - 47	1354.5873	1353.5800	1353.6160	-0.0360	1	DSYVGDEAQSKR (No match)
47 - 53	800.5176	799.5103	799.5279	-0.0176	1	RGILTLK (No match)
81 - 98	1954.0072	1952.9999	1953.0571	-0.0571	0	VAPEEHPVLLTEAPLNPK (No match)
81 - 98	1954.0072	1952.9999	1953.0571	-0.0571	0	VAPEEHPVLLTEAPLNPK (Ions score 102)

133 - 162	3199.4995	3198.4922	3198.6019	-0.1096	0	TTGIVMDSGDGVTHTVPIYEGYALPHAILR Oxidation (M) (No match)
182 - 191	1132.4944	1131.4871	1131.5196	-0.0325	0	GYSFTTTAER (No match)
182 - 191	1132.4944	1131.4871	1131.5196	-0.0325	0	GYSFTTTAER (Ions score 47)
182 - 195	1629.7560	1628.7487	1628.8158	-0.0670	1	GYSFTTTAEREIVR (No match)
224 - 239	1790.8362	1789.8289	1789.8846	-0.0556	0	SYELPDGQVITIGNER (No match)
224 - 239	1790.8362	1789.8289	1789.8846	-0.0556	0	SYELPDGQVITIGNER (Ions score 110)
276 - 297	2359.0830	2358.0757	2358.1525	-0.0768	1	KDLYANTVLSGGTTMYPGIADR Oxidation (M) (No match)
277 - 297	2230.9946	2229.9873	2230.0575	-0.0702	0	DLYANTVLSGGTTMYPGIADR Oxidation (M) (No match)
312 - 320	1036.6254	1035.6181	1035.6440	-0.0259	1	IKIIAPPER (No match)
314 - 321	923.5425	922.5352	922.5600	-0.0247	1	IIAPPERK (No match)
345 - 357	1516.6558	1515.6485	1515.6953	-0.0468	0	QEYDESGPSIVHR (No match)
345 - 357	1516.6558	1515.6485	1515.6953	-0.0468	0	QEYDESGPSIVHR (Ions score 62)
345 - 358	1644.7450	1643.7377	1643.7903	-0.0526	1	QEYDESGPSIVHRK (No match)

Mascot: http://www.matrixscience.com/

Spot 527

Mascot Search Results

Protein View

Match to: **gi|15277503** Score: **615** Expect: **6.1e-057**
ACTB protein [Homo sapiens]

Nominal mass (M_r): **40536**; Calculated pI value: **5.55**
NCBI BLAST search of gi|15277503 against nr
Unformatted sequence string for pasting into other applications

Taxonomy: Homo sapiens

Fixed modifications: Carbamidomethyl (C)
Variable modifications: Oxidation (M)
Cleavage by Trypsin: cuts C-term side of KR unless next residue is P
Sequence Coverage: **51%**

Matched peptides shown in **Bold Red**

```
  1 MCKAGFAGDD APRAVFPSIV GRPRHQGVMV GMGQKDSYVG DEAQSKRGIL
 51 TLKYPIEHGI VTNWDDMEKI WHHTFYNELR VAPEEHPVLL TEAPLNPKAN
101 LEKMTQIMFE TFNTPAMYVA IQAVLSLYAS GRTTGIVMDS GDGVTHTVPI
151 YEGYALPHAI LRLDLAGRDL TDYLMKILTE RGYSFTTTAE REIVRDIKEK
201 LCYVALDFEQ EMATAASSSS LEKSYELPDG QVITIGNERF RCPEALFQPS
251 FLGMESCGIH ETTFNSIMKC DVDIRKDLYA NTVLSGGTTM YPGIADRMQK
301 EITALAPSTM KIKIIAPPER KYSVWIGGSI LASLSTFQQM WISKQEYDES
351 GPSIVHRKCF
```

Residue Number Increasing Mass Decreasing Mass

Start - End	Observed	Mr(expt)	Mr(calc)	Delta	Miss	Sequence
4 - 13	976.4236	975.4163	975.4409	-0.0246	0	AGFAGDDAPR (No match)
14 - 24	1198.6704	1197.6631	1197.6982	-0.0350	0	AVFPSIVGRPR (Ions score 60)
14 - 24	1198.6704	1197.6631	1197.6982	-0.0350	0	AVFPSIVGRPR (No match)
25 - 35	1187.5331	1186.5258	1186.5586	-0.0328	0	HQGVMVGMGQK Oxidation (M) (No match)
25 - 35	1203.5217	1202.5144	1202.5535	-0.0391	0	HQGVMVGMGQK 2 Oxidation (M) (No match)
36 - 47	1354.5865	1353.5792	1353.6160	-0.0368	1	DSYVGDEAQSKR (No match)
70 - 80	1515.7006	1514.6933	1514.7418	-0.0485	0	IWHHTFYNELR (No match)

81 - 98	1954.0088	1953.0015	1953.0571	-0.0555	0	VAPEEHPVLLTEAPLNPK (Ions score 107)
81 - 98	1954.0088	1953.0015	1953.0571	-0.0555	0	VAPEEHPVLLTEAPLNPK (No match)
133 - 162	3183.5156	3182.5083	3182.6069	-0.0986	0	TTGIVMDSGDGVTHTVPIYEGYALPHAILR (No match)
182 - 191	1132.4954	1131.4881	1131.5196	-0.0315	0	GYSFTTTAER (No match)
182 - 191	1132.4954	1131.4881	1131.5196	-0.0315	0	GYSFTTTAER (Ions score 58)
182 - 195	1629.7660	1628.7587	1628.8158	-0.0570	1	GYSFTTTAEREIVR (No match)
224 - 239	1790.8352	1789.8279	1789.8846	-0.0566	0	SYELPDGQVITIGNER (Ions score 100)
224 - 239	1790.8352	1789.8279	1789.8846	-0.0566	0	SYELPDGQVITIGNER (No match)
270 - 275	777.3401	776.3328	776.3487	-0.0158	0	CDVDIR (No match)
270 - 276	905.4276	904.4203	904.4436	-0.0233	1	CDVDIRK (No match)
276 - 297	2359.1113	2358.1040	2358.1525	-0.0485	1	KDLYANTVLSGGTTMYPGIADR Oxidation (M) (No match)
277 - 297	2215.0083	2214.0010	2214.0626	-0.0616	0	DLYANTVLSGGTTMYPGIADR (No match)
277 - 297	2231.0049	2229.9976	2230.0575	-0.0599	0	DLYANTVLSGGTTMYPGIADR Oxidation (M) (No match)
312 - 320	1036.6250	1035.6177	1035.6440	-0.0263	1	IKIIAPPER (No match)
314 - 320	795.4562	794.4489	794.4650	-0.0161	0	IIAPPER (No match)
314 - 321	923.5430	922.5357	922.5600	-0.0242	1	IIAPPERK (No match)
345 - 357	1516.6764	1515.6691	1515.6953	-0.0262	0	QEYDESGPSIVHR (No match)
345 - 357	1516.6764	1515.6691	1515.6953	-0.0262	0	QEYDESGPSIVHR (Ions score 75)
345 - 358	1644.7466	1643.7393	1643.7903	-0.0510	1	QEYDESGPSIVHRK (No match)

Mascot: http://www.matrixscience.com/

Spot 530

MATRIX SCIENCE **Mascot Search Results**

Protein View

Match to: **gi|15277503** Score: **357** Expect: **3.8e-031**
ACTB protein [Homo sapiens]

Nominal mass (M_r): **40536;** Calculated pI value: **5.55**
NCBI BLAST search of gi|15277503 against nr
Unformatted sequence string for pasting into other applications

Taxonomy: Homo sapiens

Fixed modifications: Carbamidomethyl (C)
Variable modifications: Oxidation (M)
Cleavage by Trypsin: cuts C-term side of KR unless next residue is P
Sequence Coverage: **34%**

Matched peptides shown in **Bold Red**

```
  1 MCKAGFAGDD APRAVFPSIV GRPRHQGVMV GMGQKDSYVG DEAQSKRGIL
 51 TLKYPIEHGI VTNWDDMEKI WHHTFYNELR VAPEEHPVLL TEAPLNPKAN
101 LEKMTQIMFE TFNTPAMYVA IQAVLSLYAS GRTTGIVMDS GDGVTHTVPI
151 YEGYALPHAI LRLDLAGRDL TDYLMKILTE RGYSFTTTAE REIVRDIKEK
201 LCYVALDFEQ EMATAASSSS LEKSYELPDG QVITIGNERF RCPEALFQPS
251 FLGMESCGIH ETTFNSIMKC DVDIRKDLYA NTVLSGGTTM YPGIADRMQK
301 EITALAPSTM KIKIIAPPER KYSVWIGGSI LASLSTFQQM WISKQEYDES
351 GPSIVHRKCF
```

Residue Number Increasing Mass Decreasing Mass

Start - End	Observed	Mr(expt)	Mr(calc)	Delta	Miss	Sequence
4 - 13	976.4559	975.4486	975.4409	0.0077	0	AGFAGDDAPR (No match)
14 - 24	1198.7111	1197.7038	1197.6982	0.0057	0	AVFPSIVGRPR (Ions score 16)
14 - 24	1198.7111	1197.7038	1197.6982	0.0057	0	AVFPSIVGRPR (No match)
36 - 47	1354.6371	1353.6298	1353.6160	0.0138	1	DSYVGDEAQSKR (No match)
81 - 98	1954.0736	1953.0663	1953.0571	0.0093	0	VAPEEHPVLLTEAPLNPK (Ions score 93)
81 - 98	1954.0736	1953.0663	1953.0571	0.0093	0	VAPEEHPVLLTEAPLNPK (No match)
182 - 191	1132.5375	1131.5302	1131.5196	0.0106	0	GYSFTTTAER (No match)

182 - 195	1629.8258	1628.8185	1628.8158	0.0028	1	GYSFTTTAEREIVR (No match)
224 - 239	1790.8949	1789.8876	1789.8846	0.0031	0	SYELPDGQVITIGNER (No match)
224 - 239	1790.8949	1789.8876	1789.8846	0.0031	0	SYELPDGQVITIGNER (Ions score 86)
277 - 297	2231.0730	2230.0657	2230.0575	0.0082	0	DLYANTVLSGGTTMYPGIADR Oxidation (M) (No match)
312 - 320	1036.6617	1035.6544	1035.6440	0.0104	1	IKIIAPPER (No match)
314 - 320	795.4816	794.4743	794.4650	0.0093	0	IIAPPER (No match)
345 - 357	1516.7109	1515.7036	1515.6953	0.0083	0	QEYDESGPSIVHR (No match)
345 - 357	1516.7109	1515.7036	1515.6953	0.0083	0	QEYDESGPSIVHR (Ions score 56)
345 - 358	1644.8347	1643.8274	1643.7903	0.0371	1	QEYDESGPSIVHRK (No match)

Mascot: http://www.matrixscience.com/

Spot 531

Mascot Search Results

Protein View

Match to: **gi|117938762** Score: **439** Expect: **2.4e-039**
GNAS complex locus isoform f [Homo sapiens]

Nominal mass (M_r): **46179**; Calculated pI value: **5.59**
NCBI BLAST search of gi|117938762 against nr
Unformatted sequence string for pasting into other applications

Taxonomy: Homo sapiens
Links to retrieve other entries containing this sequence from NCBI Entrez:
gi|386744 from Homo sapiens
gi|14250772 from Homo sapiens
gi|57208560 from Homo sapiens
gi|57208703 from Homo sapiens
gi|147223315 from Sus scrofa
gi|147223425 from Sus scrofa

Fixed modifications: Carbamidomethyl (C)
Variable modifications: Oxidation (M)
Cleavage by Trypsin: cuts C-term side of KR unless next residue is P
Sequence Coverage: **33%**

Matched peptides shown in **Bold Red**

```
  1 MGCLGNSKTE DQRNEEKAQR EANKKIEKQL QKDKQVYRAT HRLLLLGAGE
 51 SGKSTIVKQM RILHVNGFNG EGGEEDPQAA RSNSDGSEKA TKVQDIKNNL
101 KEAIETIVAA MSNLVPPVEL ANPENQFRVD YILSVMNVPD FDFPPEFYEH
151 AKALWEDEGV RACYERSNEY QLIDCAQYFL DKIDVIKQAD YVPSDQDLLR
201 CRVLTSGIFE TKFQVDKVNF HMFDVGGQRD ERRKWIQCFN DVTAIIFVVA
251 SSSYNMVIRE DNQTNRLQEA LNLFKSIWNN RWLRTISVIL FLNKQDLLAE
301 KVLAGKSKIE DYFPEFARYT TPEDATPEPG EDPRVTRAKY FIRDEFLRIS
351 TASGDGRHYC YPHFTCAVDT ENIRRVFNDC RDIIQRMHLR QYELL
```

Residue Number Increasing Mass Decreasing Mass

Start - End Observed Mr(expt) Mr(calc) Delta Miss Sequence

153 - 161	1074.5160	1073.5087	1073.5141	-0.0054	0	ALWEDEGVR (No match)
167 - 187	2575.2354	2574.2281	2574.2675	-0.0394	1	SNEYQLIDCAQYFLDKIDVIK (No match)
188 - 200	1519.7175	1518.7102	1518.7314	-0.0212	0	QADYVPSDQDLLR (No match)
213 - 229	2039.9395	2038.9322	2038.9682	-0.0360	1	FQVDKVNFHMFDVGGQR Oxidation (M) (No match)
218 - 229	1406.6461	1405.6388	1405.6560	-0.0172	0	VNFHMFDVGGQR (No match)
218 - 229	1422.6354	1421.6281	1421.6509	-0.0228	0	VNFHMFDVGGQR Oxidation (M) (Ions score 26)
218 - 229	1422.6354	1421.6281	1421.6509	-0.0228	0	VNFHMFDVGGQR Oxidation (M) (No match)
218 - 232	1822.8003	1821.7930	1821.8216	-0.0286	1	VNFHMFDVGGQRDER Oxidation (M) (No match)
285 - 301	1945.1047	1944.0974	1944.1295	-0.0321	1	TISVILFLNKQDLLAEK (No match)
307 - 318	1501.7115	1500.7042	1500.7248	-0.0206	1	SKIEDYFPEFAR (Ions score 41)
307 - 318	1501.7115	1500.7042	1500.7248	-0.0206	1	SKIEDYFPEFAR (No match)
309 - 318	1286.5907	1285.5834	1285.5978	-0.0144	0	IEDYFPEFAR (No match)
309 - 318	1286.5907	1285.5834	1285.5978	-0.0144	0	IEDYFPEFAR (Ions score 50)
319 - 334	1774.7482	1773.7409	1773.7693	-0.0284	0	YTTPEDATPEPGEDPR (No match)
340 - 348	1258.6432	1257.6359	1257.6505	-0.0146	1	YFIRDEFLR (Ions score 49)
340 - 348	1258.6432	1257.6359	1257.6505	-0.0146	1	YFIRDEFLR (No match)
358 - 374	2182.9043	2181.8970	2181.9360	-0.0390	0	HYCYPHFTCAVDTENIR (No match)
358 - 374	2182.9043	2181.8970	2181.9360	-0.0390	0	HYCYPHFTCAVDTENIR (Ions score 115)

Mascot: http://www.matrixscience.com/

Spot 533

Mascot Search Results

Protein View

Match to: **gi|64976582** Score: **414** Expect: **7.7e-037**
MHC class I antigen [Homo sapiens]

Nominal mass (M_r): **31862**; Calculated pI value: **5.88**
NCBI BLAST search of gi|64976582 against nr
Unformatted sequence string for pasting into other applications

Taxonomy: Homo sapiens

Fixed modifications: Carbamidomethyl (C)
Variable modifications: Oxidation (M)
Cleavage by Trypsin: cuts C-term side of KR unless next residue is P
Sequence Coverage: **42%**

Matched peptides shown in **Bold Red**

```
  1 SHSMRYFYTA VSRPGRGEPR FIAVGYVDDT QFVRFDSDAA SPRMAPRAPW
 51 IEQEGPEYWD RETQKYKRQA QTDRVSLRNL RGYYNQSEAG SHTLQRMYGC
101 DVGPDGRLLR GHDQSAYDGK DYIALNEDLS SWTAADTAAQ ITQRKWEAAR
151 EAEQWRAYLE GLCVEWLRRY LENGKETLQR ADPPKTHVTH HPISDHEATL
201 RCWALGFYPA EITLTWQRDG EDQTQDTELV ETRPAGDRTF QKWAAVVVPS
251 GEEQRYTCHV QHEGLPKPLT LRW
```

Residue Number Increasing Mass Decreasing Mass

Start - End	Observed	Mr(expt)	Mr(calc)	Delta	Miss	Sequence
6 - 16	1316.6805	1315.6732	1315.6673	0.0060	0	YFYTAVSRPGR (No match)
21 - 34	1629.8300	1628.8227	1628.8198	0.0029	0	FIAVGYVDDTQFVR (No match)
21 - 34	1629.8300	1628.8227	1628.8198	0.0029	0	FIAVGYVDDTQFVR (Ions score 92)
48 - 61	1775.8070	1774.7997	1774.7950	0.0047	0	APWIEQEGPEYWDR (No match)
170 - 180	1350.7098	1349.7025	1349.6939	0.0087	1	YLENGKETLQR (No match)
186 - 201	1850.9292	1849.9219	1849.9183	0.0036	0	THVTHHPISDHEATLR (Ions score 78)
186 - 201	1850.9292	1849.9219	1849.9183	0.0036	0	THVTHHPISDHEATLR (No match)
219 - 238	2232.0090	2231.0017	2230.9938	0.0080	0	DGEDQTQDTELVETRPAGDR (No match)
243 - 255	1427.7356	1426.7283	1426.7204	0.0079	0	WAAVVVPSGEEQR (No match)

243 - 255	1427.7356	1426.7283	1426.7204	0.0079	0	WAAVVVPSGEEQR	(Ions score 66)
256 - 272	2049.0664	2048.0591	2048.0625	-0.0034	0	YTCHVQHEGLPKPLTLR	(Ions score 58)
256 - 272	2049.0664	2048.0591	2048.0625	-0.0034	0	YTCHVQHEGLPKPLTLR	(No match)

Mascot: http://www.matrixscience.com/

Spot 534

Mascot Search Results

Protein View

Match to: **gi|106879085** Score: **167** Expect: **3.8e-012**
MHC class I antigen [Homo sapiens]

Nominal mass (M_r): **31527**; Calculated pI value: **5.74**
NCBI BLAST search of gi|106879085 against nr
Unformatted sequence string for pasting into other applications

Taxonomy: Homo sapiens

Fixed modifications: Carbamidomethyl (C)
Variable modifications: Oxidation (M)
Cleavage by Trypsin: cuts C-term side of KR unless next residue is P
Sequence Coverage: **31%**

Matched peptides shown in **Bold Red**

```
  1 SHSMRYFSTS VSRPGRGEPR FIAVGYVDDT QFVRFDSDAA SQRMEPRAPW
 51 IEQEGPEYWD EETGKVKAHS QTHRVDLGTL RGYYNQSEAG SHTLQMMFGC
101 DVGSDGRFLR GYHQYAYDGK DYIALKEDLR SWTAADMAAQ ITKRKWEAAH
151 VAEQQRAYLE GTCVDGLRRY LENGKETLQR TDPPKTHMTH HPISDHEATL
201 RCWALGFYPA EITLTWQRDG EDQTQDTELV ETRPAGDGTF QKWAAVVVPS
251 GEEQRYTCHV QHEGLPKPLT LRW
```

Residue Number Increasing Mass Decreasing Mass

Start - End	Observed	Mr(expt)	Mr(calc)	Delta	Miss	Sequence
6 - 16	1256.5806	1255.5733	1255.6309	-0.0576	0	YFSTSVSRPGR (No match)
6 - 20	1695.7870	1694.7797	1694.8488	-0.0691	1	YFSTSVSRPGRGEPR (No match)
6 - 20	1695.7870	1694.7797	1694.8488	-0.0691	1	YFSTSVSRPGRGEPR (Ions score 3)
21 - 34	1629.7543	1628.7470	1628.8198	-0.0728	0	FIAVGYVDDTQFVR (Ions score 47)
21 - 34	1629.7543	1628.7470	1628.8198	-0.0728	0	FIAVGYVDDTQFVR (No match)
35 - 47	1509.6814	1508.6741	1508.6678	0.0064	1	FDSDAASQRMEPR (No match)
170 - 180	1350.6436	1349.6363	1349.6939	-0.0575	1	YLENGKETLQR (No match)
186 - 201	1898.8065	1897.7992	1897.8853	-0.0861	0	THMTHHPISDHEATLR Oxidation (M) (No match)
256 - 272	2048.9692	2047.9619	2048.0625	-0.1006	0	YTCHVQHEGLPKPLTLR (No match)

256 - 272   2048.9692   2047.9619   2048.0625   -0.1006   0   YTCHVQHEGLPKPLTLR   (Ions score 45)

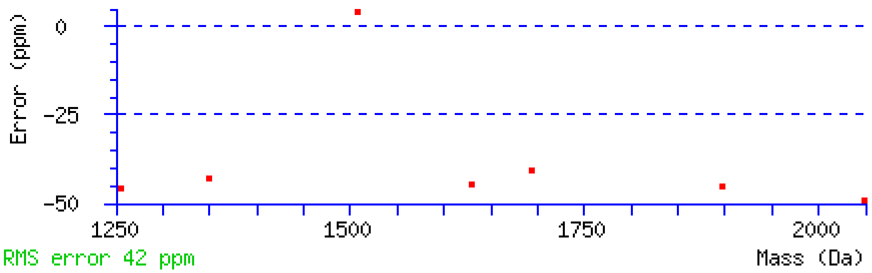

Mascot: http://www.matrixscience.com/

Spot 535

Mascot Search Results

Protein View

Match to: **gi|124517293** Score: **126** Expect: **4.8e-008**
MHC class I antigen [Homo sapiens]

Nominal mass (M_r): **31959**; Calculated pI value: **6.06**
NCBI BLAST search of gi|124517293 against nr
Unformatted sequence string for pasting into other applications

Taxonomy: Homo sapiens

Fixed modifications: Carbamidomethyl (C)
Variable modifications: Oxidation (M)
Cleavage by Trypsin: cuts C-term side of KR unless next residue is P
Sequence Coverage: **29%**

Matched peptides shown in **Bold Red**

```
  1 SHSMRYFFTS VSRPGRGEPR FIAVGYVDDT QFVRFDSDAA SQRMEPRAPW
 51 IEQEGPEYWD GETRKVKAHS QTDRENLRIA LRYYNQSEAG SHTVQRMCGC
101 DVGSDWRFLR GYHQYAYDGK DYIALKEDLR SWTAADMAAQ TTKHKWEAAH
151 VAEQLRAYLE GTCVEWLRRY LENGKETLQR TDAPKTHMTH HAVSDHEATL
201 RCWALSFYPA EITLTWQRDG EDQTQDTELV ETRPAGDGTF QKWAAVVVPS
251 GQEQRYTCHV QHEGLPKPLT LRW
```

Residue Number Increasing Mass Decreasing Mass

Start - End	Observed	Mr(expt)	Mr(calc)	Delta	Miss	Sequence
6 - 16	1316.6671	1315.6598	1315.6673	-0.0074	0	YFFTSVSRPGR (No match)
6 - 16	1316.6671	1315.6598	1315.6673	-0.0074	0	YFFTSVSRPGR (Ions score 4)
21 - 34	1629.8204	1628.8131	1628.8198	-0.0067	0	FIAVGYVDDTQFVR (No match)
21 - 34	1629.8204	1628.8131	1628.8198	-0.0067	0	FIAVGYVDDTQFVR (Ions score 30)
97 - 110	1774.7773	1773.7700	1773.7385	0.0315	1	MCGCDVGSDWRFLR Oxidation (M) (No match)
219 - 242	2637.1843	2636.1770	2636.1837	-0.0067	0	DGEDQTQDTELVETRPAGDGTFQK (No match)
256 - 272	2049.0608	2048.0535	2048.0625	-0.0090	0	YTCHVQHEGLPKPLTLR (No match)
256 - 272	2049.0608	2048.0535	2048.0625	-0.0090	0	YTCHVQHEGLPKPLTLR (Ions score 26)

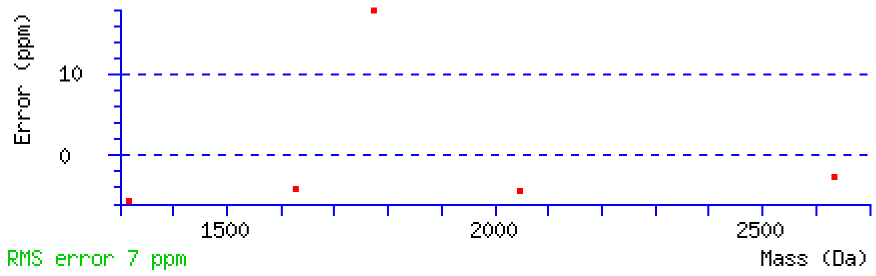

Mascot: http://www.matrixscience.com/

Spot 536

Mascot Search Results

Protein View

Match to: **gi|114793621** Score: **224** Expect: **7.7e-018**
Chain A, Structural Basis For Inhibition Of Protein Tyrosine Phosphatase 1b By Isothiazolidinone He

Nominal mass (M_r): **38453**; Calculated pI value: **6.28**
NCBI BLAST search of gi|114793621 against nr
Unformatted sequence string for pasting into other applications

Taxonomy: Homo sapiens

Fixed modifications: Carbamidomethyl (C)
Variable modifications: Oxidation (M)
Cleavage by Trypsin: cuts C-term side of KR unless next residue is P
Sequence Coverage: **38%**

Matched peptides shown in **Bold Red**

```
  1 MHHHHHHEME KEFEQIDKSG SWAAIYQDIR HEASDFPCRV AKLPKNKNRN
 51 RYRDVSPFDH SRIKLHQEDN DYINASLIKM EEAQRSYILT QGPLPNTCGH
101 FWEMVWEQKS RGVVMLNRVM EKGSLKCAQY WPQKEEKEMI FEDTNLKLTL
151 ISEDIKSYYT VRQLELENLT TQETREILHF HYTTWPDFGV PESPASFLNF
201 LFKVRESGSL SPEHGPVVVH CSAGIGRSGT FCLADTCLLL MDKRKDPSSV
251 DIKKVLLEMR KFRMGLIQTA DQLRFSYLAV IEGAKFIMGD SSVQDQWKEL
301 SHEDLEPPPE HIPPPPRPPK RILEPHN
```

Residue Number Increasing Mass Decreasing Mass

Start - End	Observed	Mr(expt)	Mr(calc)	Delta	Miss	Sequence
19 - 30	1366.6393	1365.6320	1365.6677	-0.0356	0	SGSWAAIYQDIR (No match)
31 - 39	1118.4594	1117.4521	1117.4611	-0.0089	0	HEASDFPCR (No match)
52 - 62	1378.6163	1377.6090	1377.6425	-0.0335	1	YRDVSPFDHSR (No match)
52 - 62	1378.6163	1377.6090	1377.6425	-0.0335	1	YRDVSPFDHSR (Ions score 38)
54 - 62	1059.4706	1058.4633	1058.4781	-0.0148	0	DVSPFDHSR (No match)
65 - 79	1772.8427	1771.8354	1771.8740	-0.0386	0	LHQEDNDYINASLIK (No match)
163 - 175	1574.7631	1573.7558	1573.7947	-0.0389	0	QLELENLTTQETR (Ions score 29)
163 - 175	1574.7631	1573.7558	1573.7947	-0.0389	0	QLELENLTTQETR (No match)

206 - 227	2232.0134	2231.0061	2231.0752	-0.0691	0	**ESGSLSPEHGPVVVHCSAGIGR** (No match)
206 - 227	2232.0134	2231.0061	2231.0752	-0.0691	0	**ESGSLSPEHGPVVVHCSAGIGR** (Ions score 61)
264 - 274	1261.6232	1260.6159	1260.6496	-0.0337	0	**MGLIQTADQLR** Oxidation (M) (No match)
275 - 285	1197.6206	1196.6133	1196.6440	-0.0307	0	**FSYLAVIEGAK** (No match)
299 - 320	2508.2148	2507.2075	2507.2808	-0.0733	0	**ELSHEDLEPPPEHIPPPPRPPK** (No match)
299 - 321	2664.2769	2663.2696	2663.3819	-0.1123	1	**ELSHEDLEPPPEHIPPPPRPPKR** (No match)

Mascot: http://www.matrixscience.com/

Spot 537

MATRIX SCIENCE Mascot Search Results

Protein View

Match to: **gi|48145549** Score: **357** Expect: **3.8e-031**
PGK1 [Homo sapiens]

Nominal mass (M_r): **44973**; Calculated pI value: **8.30**
NCBI BLAST search of gi|48145549 against nr
Unformatted sequence string for pasting into other applications

Taxonomy: Homo sapiens

Fixed modifications: Carbamidomethyl (C)
Variable modifications: Oxidation (M)
Cleavage by Trypsin: cuts C-term side of KR unless next residue is P
Sequence Coverage: **23%**

Matched peptides shown in **Bold Red**

```
  1 MSLSNKLTLD KLDVKGKRVV MRVDFNVPMK NNQITNNQRI KAAVPSIKFC
 51 LDNGAKSVVL MSHLGRPDGV PMPDKYSLEP VAVELKSLLG KDVLFLKDCV
101 GPEVEKACAN PAAGSVILLE NLRFHVEEEG KGKDASGNKV KAEPAKIEAF
151 RASLSKLGDV YVNDAFGTAH RAHSSMVGVN LPQKAGGFLM KKELNYFAKA
201 LESPERPFLA ILGGAKVADK IQLINNMLDK VNEMIIGGGM AFTFLKVLNN
251 MEIGTSLFDE EGAKIVKDLM SKAEKNGVKI TLPVDFVTAD KFDENAKTGQ
301 ATVASGIPAG WMGLDCGPES SKKYAEAVTR AKQIVWNGPV GVFEWEAFAR
351 GTKALMDEVV KATSRGCITT IGGGDTATCC AKWNTEDKVS HVSTGGGASL
401 ELLEGKVLPG VDALSNI
```

Residue Number Increasing Mass Decreasing Mass

Start - End	Observed	Mr(expt)	Mr(calc)	Delta	Miss	Sequence
23 - 39	2047.8960	2046.8887	2046.9904	-0.1017	1	**VDFNVPMKNNQITNNQR** Oxidation (M) (No match)
23 - 39	2047.8960	2046.8887	2046.9904	-0.1017	1	**VDFNVPMKNNQITNNQR** Oxidation (M) (Ions score 81)
107 - 123	1768.8873	1767.8800	1767.9301	-0.0500	0	**ACANPAAGSVILLENLR** (No match)
107 - 123	1768.8873	1767.8800	1767.9301	-0.0500	0	**ACANPAAGSVILLENLR** (Ions score 31)
124 - 133	1159.5178	1158.5105	1158.5668	-0.0563	1	**FHVEEEGKGK** (No match)
142 - 151	1131.5613	1130.5540	1130.6083	-0.0543	1	**AEPAKIEAFR** (No match)

247 - 264	1982.8411	1981.8338	1981.9302	-0.0964	0	**VLNNMEIGTSLFDEEGAK** Oxidation (M) (Ions score 109)
247 - 264	1982.8411	1981.8338	1981.9302	-0.0964	0	**VLNNMEIGTSLFDEEGAK** Oxidation (M) (No match)
280 - 297	2022.9382	2021.9309	2022.0309	-0.1000	1	**ITLPVDFVTADKFDENAK** (Ions score 87)
280 - 297	2022.9382	2021.9309	2022.0309	-0.1000	1	**ITLPVDFVTADKFDENAK** (No match)
324 - 330	809.3760	808.3687	808.4079	-0.0391	0	**YAEAVTR** (No match)

Mascot: http://www.matrixscience.com/

Spot 539

Mascot Search Results

Protein View

Match to: **gi|48145549** Score: **398** Expect: **3e-035**
PGK1 [Homo sapiens]

Nominal mass (M_r): **44973;** Calculated pI value: **8.30**
NCBI BLAST search of gi|48145549 against nr
Unformatted sequence string for pasting into other applications

Taxonomy: Homo sapiens

Fixed modifications: Carbamidomethyl (C)
Variable modifications: Oxidation (M)
Cleavage by Trypsin: cuts C-term side of KR unless next residue is P
Sequence Coverage: **24%**

Matched peptides shown in **Bold Red**

```
  1 MSLSNKLTLD KLDVKGKRVV MRVDFNVPMK NNQITNNQRI KAAVPSIKFC
 51 LDNGAKSVVL MSHLGRPDGV PMPDKYSLEP VAVELKSLLG KDVLFLKDCV
101 GPEVEKACAN PAAGSVILLE NLRFHVEEEG KGKDASGNKV KAEPAKIEAF
151 RASLSKLGDV YVNDAFGTAH RAHSSMVGVN LPQKAGGFLM KKELNYFAKA
201 LESPERPFLA ILGGAKVADK IQLINNMLDK VNEMIIGGGM AFTFLKVLNN
251 MEIGTSLFDE EGAKIVKDLM SKAEKNGVKI TLPVDFVTAD KFDENAKTGQ
301 ATVASGIPAG WMGLDCGPES SKKYAEAVTR AKQIVWNGPV GVFEWEAFAR
351 GTKALMDEVV KATSRGCITT IGGGDTATCC AKWNTEDKVS HVSTGGGASL
401 ELLEGKVLPG VDALSNI
```

Residue Number Increasing Mass Decreasing Mass

Start - End	Observed	Mr(expt)	Mr(calc)	Delta	Miss	Sequence
23 - 39	2047.9395	2046.9322	2046.9904	-0.0582	1	VDFNVPMKNNQITNNQR Oxidation (M) (No match)
31 - 39	1101.5048	1100.4975	1100.5322	-0.0347	0	NNQITNNQR (No match)
107 - 123	1768.9171	1767.9098	1767.9301	-0.0202	0	ACANPAAGSVILLENLR (No match)
157 - 171	1634.7383	1633.7310	1633.7848	-0.0538	0	LGDVYVNDAFGTAHR (No match)
157 - 171	1634.7383	1633.7310	1633.7848	-0.0538	0	LGDVYVNDAFGTAHR (Ions score 112)
200 - 216	1768.9171	1767.9098	1767.9882	-0.0784	0	ALESPERPFLAILGGAK (Ions score 39)

247 - 264	1982.8807	1981.8734	1981.9302	-0.0568	0	VLNNMEIGTSLFDEEGAK	Oxidation (M) (No match)
247 - 264	1982.8807	1981.8734	1981.9302	-0.0568	0	VLNNMEIGTSLFDEEGAK	Oxidation (M) (Ions score 60)
280 - 297	2022.9757	2021.9684	2022.0309	-0.0625	1	ITLPVDFVTADKFDENAK	(No match)
280 - 297	2022.9757	2021.9684	2022.0309	-0.0625	1	ITLPVDFVTADKFDENAK	(Ions score 95)

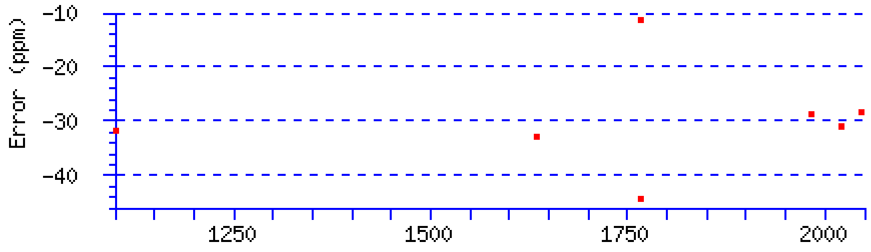

RMS error 31 ppm

Mascot: http://www.matrixscience.com/

Spot 540

Mascot Search Results

Protein View

Match to: **gi|119590846** Score: **77** Expect: **0.0042**
isocitrate dehydrogenase 1 (NADP+), soluble, isoform CRA_b [Homo sapiens]

Nominal mass (M_r): **32765**; Calculated pI value: **8.42**
NCBI BLAST search of gi|119590846 against nr
Unformatted sequence string for pasting into other applications

Taxonomy: Homo sapiens

Fixed modifications: Carbamidomethyl (C)
Variable modifications: Oxidation (M)
Cleavage by Trypsin: cuts C-term side of KR unless next residue is P
Sequence Coverage: **10%**

Matched peptides shown in **Bold Red**

```
  1 MSKKISGGSV VEMQGDEMTR IIWELIKEKL IFPYVELDLH SYDLGIENRD
 51 ATNDQVTKDA AEAIKKHNVG VKCATITPDE KRVEEFKLKQ MWKSPNGTIR
101 NILGGTVFRE AIICKNIPRL VSGWVKPIII GRHAYGDQYR ATDFVVPGPG
151 KVEITYTPSD GTQKVTYLVH NFEEGGGVAM GMYNQDKSIE DFAHSSFQMA
201 LSKGWPLYLS TKNTILKKYD GRFKDIFQEI YDQRAGLSRW QDSRSRGCPR
251 DCNPSLPHVP ERTGDVHQSH CFHFCLDQRV SPQSKA
```

Residue Number Increasing Mass Decreasing Mass

Start - End	Observed	Mr(expt)	Mr(calc)	Delta	Miss	Sequence	
30 - 49	2406.2144	2405.2071	2405.2266	-0.0195	0	LIFPYVELDLHSYDLGIENR	(No match)
30 - 49	2406.2144	2405.2071	2405.2266	-0.0195	0	LIFPYVELDLHSYDLGIENR	(Ions score 59)
101 - 109	976.5636	975.5563	975.5501	0.0062	0	NILGGTVFR	(No match)
101 - 109	976.5636	975.5563	975.5501	0.0062	0	NILGGTVFR	(No match)

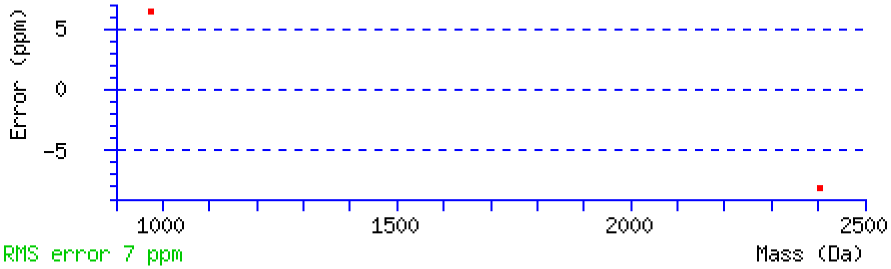

Mascot: http://www.matrixscience.com/

Spot 541

Protein View

Match to: **gi|31881779** Score: **132** Expect: **1.2e-008**
GDP-mannose pyrophosphorylase A [Homo sapiens]

Nominal mass (M_r): **46604;** Calculated pI value: **6.73**
NCBI BLAST search of gi|31881779 against nr
Unformatted sequence string for pasting into other applications

Taxonomy: Homo sapiens
Links to retrieve other entries containing this sequence from NCBI Entrez:
gi|45447090 from Homo sapiens
gi|13938607 from Homo sapiens
gi|62822505 from Homo sapiens
gi|119591161 from Homo sapiens
gi|119591163 from Homo sapiens
gi|119591164 from Homo sapiens
gi|123987258 from synthetic construct
gi|123999072 from synthetic construct

Fixed modifications: Carbamidomethyl (C)
Variable modifications: Oxidation (M)
Cleavage by Trypsin: cuts C-term side of KR unless next residue is P
Sequence Coverage: **26%**

Matched peptides shown in **Bold Red**

1 MLKAVILIGG PQKGTRFRPL SFEVPKPLFP VAGVPMIQHH IEACAQVPGM
51 QEILLIGFYQ PDEPLTQFLE AAQQEFNLPV R**YLQEFAPLG** **TGGGLYHFR**D
101 QILAGSPEAF FVLNADVCSD FPLSAMLEAH RR**QRHPFLLL** **GTTANR**TQSL
151 NYGCIVENPQ THEVLHYVEK PSTFISDIIN CGIYLFSPEA LKPLRDVFQR
201 **NQQDGQLEDS** **PGLWPGAGTI** **R**LEQDVFSAL AGQGQIYVHL TDGIWSQIKS
251 AGSALYASRL YLSR**YQDTHP** **ER**LAK**HTPGG** **PWIR**GNVYIH PTAKVAPSAV
301 LGPNVSIGKG VTVGEGVRLR ESIVLHGATL QEHTCVLHSI VGWGSTVGRW
351 AR**VEGTPSDP** **NPNDPR**ARMD SESLFKDGK**L** **LPAITILGCR** **VRIPAEVLIL**
401 **NSIVLPHK**EL SRSFTNQIIL

Residue Number Increasing Mass Decreasing Mass

Start - End	Observed	Mr(expt)	Mr(calc)	Delta	Miss	Sequence
82 - 99	2025.9779	2024.9706	2025.0107	-0.0401	0	YLQEFAPLGTGGGLYHFR (Ions score 10)
82 - 99	2025.9779	2024.9706	2025.0107	-0.0401	0	YLQEFAPLGTGGGLYHFR (No match)
133 - 146	1623.8733	1622.8660	1622.9005	-0.0344	1	QRHPFLLLGTTANR (No match)
135 - 146	1339.7300	1338.7227	1338.7408	-0.0180	0	HPFLLLGTTANR (Ions score 1)
135 - 146	1339.7300	1338.7227	1338.7408	-0.0180	0	HPFLLLGTTANR (No match)
201 - 221	2239.0376	2238.0303	2238.0665	-0.0361	0	NQQDGQLEDSPGLWPGAGTIR (No match)
265 - 272	1045.4821	1044.4748	1044.4624	0.0124	0	YQDTHPER (No match)
276 - 284	1020.5309	1019.5236	1019.5301	-0.0064	0	HTPGGPWIR (No match)
353 - 366	1494.7013	1493.6940	1493.6746	0.0194	0	VEGTPSDPNPNDPR (No match)
380 - 390	1226.7142	1225.7069	1225.7216	-0.0147	0	LLPAITILGCR (No match)
380 - 390	1226.7142	1225.7069	1225.7216	-0.0147	0	LLPAITILGCR (Ions score 21)
391 - 408	2011.2085	2010.2012	2010.2353	-0.0341	1	VRIPAEVLILNSIVLPHK (No match)

Spot 542

Protein View

Match to: **gi|180687** Score: **244** Expect: **7.7e-020**
2',3'-cyclic-nucleotide 3'-phosphodiesterase (EC 3.1.4.37)

Nominal mass (M_r): **45469;** Calculated pI value: **8.73**
NCBI BLAST search of gi|180687 against nr
Unformatted sequence string for pasting into other applications

Taxonomy: Homo sapiens
Links to retrieve other entries containing this sequence from NCBI Entrez:
gi|219401 from Homo sapiens
gi|15029671 from Homo sapiens
gi|119581189 from Homo sapiens
gi|119581191 from Homo sapiens

Fixed modifications: Carbamidomethyl (C)
Variable modifications: Oxidation (M)
Cleavage by Trypsin: cuts C-term side of KR unless next residue is P
Sequence Coverage: **22%**

Matched peptides shown in **Bold Red**

```
  1 MSSSGAKDKP ELQFPFLQDE DTVATLLECK TLFILRGLPG SGKSTLARVI
 51 VDKYRDGTKM VSADAYKITP GARGAFSEEY KRLDEDLAAY CRRRDIRILV
101 LDDTNHERER LEQLFEMADQ YQYQVVLVEP KTAWRLDCAQ LKEKNQWQLS
151 ADDLKKLKPG LEKDFLPLYF GWFLTKKSSE TLRKAGQVFL EELGNHKAFK
201 KELRQFVPGD EPREKMDLVT YFGKRPPGVL HCTTKFCDYG KAPGAEEYAQ
251 QDVLKKSYSK AFTLTISALF VTPKTTGARV ELSEQQLQLW PSDVDKLSPT
301 DNLPRGSRAH ITLGCAADVE AVQTGLDLLE ILRQEKGGSR GEEVGELSRG
351 KLYSLGNGRW MLTLAKNMEV RAIFTGYYGK GKPVPTQGSR KGGALQSCTI
401 I
```

Residue Number Increasing Mass Decreasing Mass

Start - End	Observed	Mr(expt)	Mr(calc)	Delta	Miss	Sequence	
74 - 82	1086.4805	1085.4732	1085.5141	-0.0409	1	GAFSEEYKR	(No match)
74 - 82	1086.4805	1085.4732	1085.5141	-0.0409	1	GAFSEEYKR	(Ions score 30)
83 - 92	1225.5079	1224.5006	1224.5444	-0.0438	0	LDEDLAAYCR	(No match)

83 - 93	1381.6060	1380.5987	1380.6456	-0.0468	1	LDEDLAAYCRR	(No match)
98 - 108	1324.6343	1323.6270	1323.6782	-0.0512	0	ILVLDDTNHER	(No match)
98 - 110	1609.7726	1608.7653	1608.8219	-0.0566	1	ILVLDDTNHERER	(No match)
184 - 197	1569.7830	1568.7757	1568.8310	-0.0553	1	KAGQVFLEELGNHK	(No match)
184 - 197	1569.7830	1568.7757	1568.8310	-0.0553	1	KAGQVFLEELGNHK	(Ions score 53)
185 - 197	1441.6862	1440.6789	1440.7360	-0.0571	0	AGQVFLEELGNHK	(No match)
185 - 197	1441.6862	1440.6789	1440.7360	-0.0571	0	AGQVFLEELGNHK	(Ions score 51)
205 - 213	1044.4749	1043.4676	1043.5036	-0.0359	0	QFVPGDEPR	(No match)
225 - 235	1265.6575	1264.6502	1264.6710	-0.0208	0	RPPGVLHCTTK	(No match)
242 - 255	1518.6865	1517.6792	1517.7361	-0.0569	0	APGAEEYAQQDVLK	(No match)
242 - 256	1646.7791	1645.7718	1645.8311	-0.0592	1	APGAEEYAQQDVLKK	(No match)
392 - 401	1019.4771	1018.4698	1018.5117	-0.0419	0	GGALQSCTII	(No match)

Spot 543

Protein View

Match to: **gi|34234** Score: **256** Expect: **4.8e-021**
laminin-binding protein [Homo sapiens]

Nominal mass (M_r): **31888;** Calculated pI value: **4.84**
NCBI BLAST search of gi|34234 against nr
Unformatted sequence string for pasting into other applications

Taxonomy: Homo sapiens

Fixed modifications: Carbamidomethyl (C)
Variable modifications: Oxidation (M)
Cleavage by Trypsin: cuts C-term side of KR unless next residue is P
Sequence Coverage: **16%**

Matched peptides shown in **Bold Red**

```
  1 KEEDVLKFLA AGTHLGGTNL DFQMEQYIYK RKSDGIYIIN LKRTWEKLLV
 51 AARAIVAIEN PADVSVISSR NTGQRAVLKF AAATGATPIA GRFTPGTFTN
101 QIQAAFREPR LLVVTDPRAD HQPLTEASYV NLPTIALCNT DSPLRYVDIA
151 IPCNNKGAHS VGLMWWMLAR EVLRMRGTIS REHPWEVMPD LYFYRDPEEI
201 EKEEQAAAEK AVTKEEFQGE WTAPAPEFTA TQPEVADWSE GVQVPSVPIQ
251 QFPTEDWSAQ PATEDWSAAP TAQATEWVGA TTDWS
```

Residue Number Increasing Mass Decreasing Mass

Start - End	Observed	Mr(expt)	Mr(calc)	Delta	Miss	Sequence
54 - 70	1740.9683	1739.9610	1739.9417	0.0193	0	AIVAIENPADVSVISSR (No match)
54 - 70	1740.9683	1739.9610	1739.9417	0.0193	0	AIVAIENPADVSVISSR (Ions score 83)
80 - 92	1203.6698	1202.6625	1202.6407	0.0218	0	FAAATGATPIAGR (No match)
80 - 92	1203.6698	1202.6625	1202.6407	0.0218	0	FAAATGATPIAGR (Ions score 24)
93 - 107	1698.8785	1697.8712	1697.8525	0.0187	0	FTPGTFTNQIQAAFR (Ions score 56)
93 - 107	1698.8785	1697.8712	1697.8525	0.0187	0	FTPGTFTNQIQAAFR (No match)
93 - 110	2081.0808	2080.0735	2080.0489	0.0246	1	FTPGTFTNQIQAAFREPR (Ions score 24)
93 - 110	2081.0808	2080.0735	2080.0489	0.0246	1	FTPGTFTNQIQAAFREPR (No match)

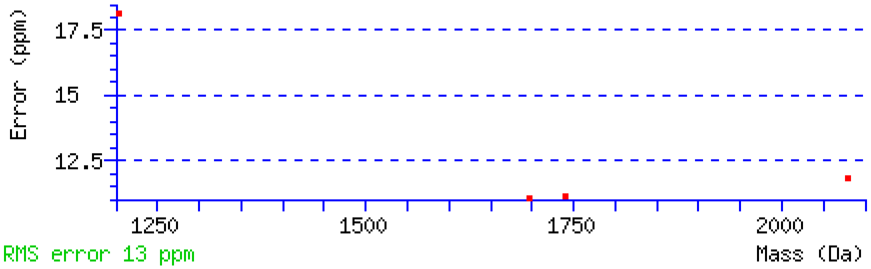

Error (ppm)
17.5
15
12.5
1250
1500
1750
2000
RMS error 13 ppm
Mass (Da)

Spot 544

Protein View

Match to: **gi|73354293** Score: **384** Expect: **7.7e-034**
MHC class I antigen [Homo sapiens]

Nominal mass (M_r): **31662**; Calculated pI value: **5.71**
NCBI BLAST search of gi|73354293 against nr
Unformatted sequence string for pasting into other applications

Taxonomy: Homo sapiens
Links to retrieve other entries containing this sequence from NCBI Entrez:
gi|150251583 from Homo sapiens

Fixed modifications: Carbamidomethyl (C)
Variable modifications: Oxidation (M)
Cleavage by Trypsin: cuts C-term side of KR unless next residue is P
Sequence Coverage: **54%**

Matched peptides shown in **Bold Red**

```
  1 SHSMRYFSTS VSRPGRGEPR FIAVGYVDDT QFVRFDSDAA SQRMEPRAPW
 51 IEQEGPEYWD EETGKVKAHS QTDRENLRIA LRYYNQSEAG SHTLQMMFGC
101 DVGSDGRFLR GYHQYAYDGK DYIALKEDLR SWTAADMAAQ ITQRKWEAAR
151 VAEQLRAYLE GTCVDGLRRY LENGKETLQR TNPPKTHMTH HPISDHEATL
201 RCWALGFYPA EITLTWQRDG EDQTQDTELV ETRPAGDGTF QKWAAVVVPS
251 GEEQRYTCHV QHEGLPKPLT LRW
```

Residue Number Increasing Mass Decreasing Mass

Start - End	Observed	Mr(expt)	Mr(calc)	Delta	Miss	Sequence
6 - 16	1256.6442	1255.6369	1255.6309	0.0060	0	YFSTSVSRPGR (No match)
6 - 20	1695.8676	1694.8603	1694.8488	0.0115	1	YFSTSVSRPGRGEPR (No match)
6 - 20	1695.8676	1694.8603	1694.8488	0.0115	1	YFSTSVSRPGRGEPR (Ions score 22)
21 - 34	1629.8364	1628.8291	1628.8198	0.0093	0	FIAVGYVDDTQFVR (Ions score 36)
21 - 34	1629.8364	1628.8291	1628.8198	0.0093	0	FIAVGYVDDTQFVR (No match)
111 - 126	1904.9299	1903.9226	1903.9104	0.0122	1	GYHQYAYDGKDYIALK (No match)
146 - 156	1328.6467	1327.6394	1327.6996	-0.0602	1	WEAARVAEQLR (No match)
157 - 169	1509.7540	1508.7467	1508.7405	0.0062	1	AYLEGTCVDGLRR (No match)
170 - 180	1350.7112	1349.7039	1349.6939	0.0101	1	YLENGKETLQR (No match)

186 - 201	1898.9076	1897.9003	1897.8853	0.0150	0	**THMTHHPISDHEATLR**	Oxidation (M) (No match)
219 - 242	2637.2153	2636.2080	2636.1837	0.0243	0	**DGEDQTQDTELVETRPAGDGTFQK**	(No match)
219 - 242	2637.2153	2636.2080	2636.1837	0.0243	0	**DGEDQTQDTELVETRPAGDGTFQK**	(Ions score 77)
243 - 255	1427.7336	1426.7263	1426.7204	0.0059	0	**WAAVVVPSGEEQR**	(No match)
256 - 272	2049.0798	2048.0725	2048.0625	0.0100	0	**YTCHVQHEGLPKPLTLR**	(No match)
256 - 272	2049.0798	2048.0725	2048.0625	0.0100	0	**YTCHVQHEGLPKPLTLR**	(Ions score 91)

Spot 545

Protein View

Match to: **gi|12598440** Score: **348** Expect: **3e-030**
DK1 lymphocyte antigen [Homo sapiens]

Nominal mass (M_r): **31963**; Calculated pI value: **6.01**
NCBI BLAST search of gi|12598440 against nr
Unformatted sequence string for pasting into other applications

Taxonomy: Homo sapiens
Links to retrieve other entries containing this sequence from NCBI Entrez:
gi|47496790 from Homo sapiens

Fixed modifications: Carbamidomethyl (C)
Variable modifications: Oxidation (M)
Cleavage by Trypsin: cuts C-term side of KR unless next residue is P
Sequence Coverage: **55%**

Matched peptides shown in **Bold Red**

```
  1 SHSMRYFFTS VSRPGRGEPR FIAVGYVDDT QFVRFDSDAA SQRMEPRAPW
 51 IEQEGPEYWD GETRKVKAHS QTHRVDLGTL RGYYNQSEAG SHTVQRMYGC
101 DVGSDWRFLR GYHQYAYDGK DYIALKEDLR SWTAADMAAQ TTKHKWETAH
151 EAEQWRAYLE GTCVEWLRRY LENGKETLQR TDAPKTHMTH HAVSDHEATL
201 RCWALSFYPA EITLTWQRDG EDQTQDTELV ETRPAGDGTF QKWAAVVVPS
251 GQEQRYTCHV QHEGLPKPLT LRW
```

Residue Number Increasing Mass Decreasing Mass

Start - End	Observed	Mr(expt)	Mr(calc)	Delta	Miss	Sequence
6 - 16	1316.6270	1315.6197	1315.6673	-0.0475	0	YFFTSVSRPGR (Ions score 18)
6 - 16	1316.6270	1315.6197	1315.6673	-0.0475	0	YFFTSVSRPGR (No match)
6 - 20	1755.8326	1754.8253	1754.8852	-0.0598	1	YFFTSVSRPGRGEPR (No match)
21 - 34	1629.7655	1628.7582	1628.8198	-0.0616	0	FIAVGYVDDTQFVR (Ions score 61)
21 - 34	1629.7655	1628.7582	1628.8198	-0.0616	0	FIAVGYVDDTQFVR (No match)
68 - 81	1590.7688	1589.7615	1589.8386	-0.0771	1	AHSQTHRVDLGTLR (No match)
68 - 81	1590.7688	1589.7615	1589.8386	-0.0771	1	AHSQTHRVDLGTLR (Ions score 8)
111 - 120	1201.4811	1200.4738	1200.5199	-0.0461	0	GYHQYAYDGK (No match)
111 - 126	1904.8474	1903.8401	1903.9104	-0.0703	1	GYHQYAYDGKDYIALK (No match)

146 - 156	1442.6846	1441.6773	1441.6374	0.0399	0	**WETAHEAEQWR**	(No match)
170 - 180	1350.6530	1349.6457	1349.6939	-0.0481	1	**YLENGKETLQR**	(No match)
186 - 201	1858.7889	1857.7816	1857.8540	-0.0724	0	**THMTHHAVSDHEATLR**	Oxidation (M) (No match)
219 - 242	2637.0781	2636.0708	2636.1837	-0.1129	0	**DGEDQTQDTELVETRPAGDGTFQK**	(No match)
219 - 242	2637.0781	2636.0708	2636.1837	-0.1129	0	**DGEDQTQDTELVETRPAGDGTFQK**	(Ions score 52)
243 - 255	1426.6873	1425.6800	1425.7364	-0.0564	0	**WAAVVVPSGQEQR**	(No match)
256 - 272	2048.9888	2047.9815	2048.0625	-0.0810	0	**YTCHVQHEGLPKPLTLR**	(No match)
256 - 272	2048.9888	2047.9815	2048.0625	-0.0810	0	**YTCHVQHEGLPKPLTLR**	(Ions score 54)

Spot 546

Protein View

Match to: **gi|5902060** Score: **217** Expect: **3.8e-017**
XRP2 protein [Homo sapiens]

Nominal mass (M_r): **40472**; Calculated pI value: **4.94**
NCBI BLAST search of gi|5902060 against nr
Unformatted sequence string for pasting into other applications

Taxonomy: Homo sapiens
Links to retrieve other entries containing this sequence from NCBI Entrez:
gi|3550283 from Homo sapiens

Fixed modifications: Carbamidomethyl (C)
Variable modifications: Oxidation (M)
Cleavage by Trypsin: cuts C-term side of KR unless next residue is P
Sequence Coverage: **27%**

Matched peptides shown in **Bold Red**

```
  1 MGCFFSKRRK ADKESRPENE EERPKQYSWD QREKVDPKDY MFSGLKDETV
 51 GRLPGTVAGQ QFLIQDCENC NIYIFDHSAT VTIDDCTNCI IFLGPVKGSV
101 FFRNCRDCKC TLACQQFRVR DCRKLEVFLC CATQPIIESS SNIKFGCFQW
151 YYPELAFQFK DAGLSIFDNT WSNIHDFTPV SGELNWSLLP EDAVVQDYVP
201 IPTTEELKAV RVSTEANRSI VPISRGQRQK SSDESCLVVL FAGDYTIANA
251 RKLIDEMVGK GFFLVQTKEV SMKAEDAQRV FREKAPDFLP LLNKGPVIAL
301 EFNGDGAVEV CQLIVNEIFN GTKMFVSESK ETASGDVDSF YNFADIQMGI
351
```

Residue Number Increasing Mass Decreasing Mass

Start - End	Observed	Mr(expt)	Mr(calc)	Delta	Miss	Sequence
11 - 25	1813.8518	1812.8445	1812.8601	-0.0156	1	ADKESRPENEEERPK (No match)
14 - 25	1499.6921	1498.6848	1498.7011	-0.0163	0	ESRPENEEERPK (No match)
26 - 32	982.4333	981.4260	981.4304	-0.0044	0	QYSWDQR (No match)
39 - 52	1633.7382	1632.7309	1632.7453	-0.0144	1	DYMFSGLKDETVGR Oxidation (M) (No match)
39 - 52	1633.7382	1632.7309	1632.7453	-0.0144	1	DYMFSGLKDETVGR Oxidation (M) (Ions score 21)
110 - 118	1183.5195	1182.5122	1182.5274	-0.0152	0	CTLACQQFR (No match)
110 - 118	1183.5195	1182.5122	1182.5274	-0.0152	0	CTLACQQFR (Ions score 25)

124 - 144	2437.2297	2436.2224	2436.2392	-0.0168	1	KLEVFLCCATQPIIESSSNIK	(No match)
231 - 251	2288.0520	2287.0447	2287.0790	-0.0343	0	SSDESCLVVLFAGDYTIANAR	(No match)
231 - 251	2288.0520	2287.0447	2287.0790	-0.0343	0	SSDESCLVVLFAGDYTIANAR	(Ions score 85)
261 - 268	939.5148	938.5075	938.5225	-0.0150	0	GFFLVQTK	(No match)

Spot 547

Protein View

Match to: **gi|113196585** Score: **210** Expect: **1.9e-016**
MHC class I antigen [Homo sapiens]

Nominal mass (M_r): **31863**; Calculated pI value: **5.42**
NCBI BLAST search of gi|113196585 against nr
Unformatted sequence string for pasting into other applications

Taxonomy: Homo sapiens

Fixed modifications: Carbamidomethyl (C)
Variable modifications: Oxidation (M)
Cleavage by Trypsin: cuts C-term side of KR unless next residue is P
Sequence Coverage: **25%**

Matched peptides shown in **Bold Red**

```
  1 SHSMRYFSTS VSRPGRGEPR FIAVGYVDDT QFVRFDSDAA SQRMEPRAPW
 51 IEQERPEYWD QETRNVKAQS QTDRVDLGTL RGYYNQSEAG SHTIQIMYGC
101 DVGSDGRFLR GYEQHAYDGK DYIALNEDLR SWTAADMAAQ ITQRKWEAAR
151 WAEQLRAYLE GTCVEWLRRY LENGKETLQR TDPPKTHMTH HPISDHEATL
201 RCWALGFYPA EITLTWQRDG EDQTQDTELV ETRPAGDGTF QKWAAVVVPS
251 GEEQRYTCHV QHEGLPKPLT LRW
```

Residue Number Increasing Mass Decreasing Mass

Start - End	Observed	Mr(expt)	Mr(calc)	Delta	Miss	Sequence
6 - 16	1256.5997	1255.5924	1255.6309	-0.0385	0	YFSTSVSRPGR (No match)
6 - 20	1695.8071	1694.7998	1694.8488	-0.0490	1	YFSTSVSRPGRGEPR (No match)
6 - 20	1695.8071	1694.7998	1694.8488	-0.0490	1	YFSTSVSRPGRGEPR (Ions score 9)
21 - 34	1629.7749	1628.7676	1628.8198	-0.0522	0	FIAVGYVDDTQFVR (Ions score 31)
21 - 34	1629.7749	1628.7676	1628.8198	-0.0522	0	FIAVGYVDDTQFVR (No match)
219 - 242	2637.1155	2636.1082	2636.1837	-0.0755	0	DGEDQTQDTELVETRPAGDGTFQK (No match)
219 - 242	2637.1155	2636.1082	2636.1837	-0.0755	0	DGEDQTQDTELVETRPAGDGTFQK (Ions score 40)
256 - 272	2049.0063	2047.9990	2048.0625	-0.0635	0	YTCHVQHEGLPKPLTLR (Ions score 59)
256 - 272	2049.0063	2047.9990	2048.0625	-0.0635	0	YTCHVQHEGLPKPLTLR (No match)

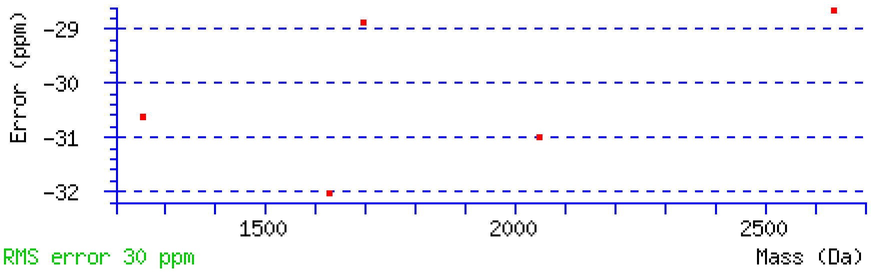

Error (ppm)
-29
-30
-31
-32
1500
2000
2500
RMS error 30 ppm
Mass (Da)

Spot 548

Protein View

Match to: **gi|62898301** Score: **485** Expect: **6.1e-044**
serine (or cysteine) proteinase inhibitor, clade B (ovalbumin), member 1 variant [Homo sapiens]

Nominal mass (M_r): **42857;** Calculated pI value: **5.90**
NCBI BLAST search of gi|62898301 against nr
Unformatted sequence string for pasting into other applications

Taxonomy: Homo sapiens

Fixed modifications: Carbamidomethyl (C)
Variable modifications: Oxidation (M)
Cleavage by Trypsin: cuts C-term side of KR unless next residue is P
Sequence Coverage: **25%**

Matched peptides shown in **Bold Red**

```
  1 MEQLSSANTR FALDLFLALS ENNPAGNIFI SPFSISSAMA MVFLGTRGNT
 51 AAQLSKTFHF NTVEEVHSRF QSLNADINKR GASYILKLAN RLYGEKTYNF
101 LPEFLVSTQK TYGADLASVD FQHASEDARK TINQWVRGQT EGKIPELLAS
151 GMVDNMTKLV LVNAIYFKGN WKDKFMKEAT TNAPFRLNKK DRKTVKMMYQ
201 KKKFAYGYIE DLKCRVLELP YQGEELSMVI LLPDDIEDES TGLKKIEEQL
251 TLEKLHEWTK PENLDFIEVN VSLPRFKLEE SYTLNSDLAR LGVQDLFNSS
301 KADLSGMSGA RDIFISKIVH KSFVEVNEEG TEAAAATAGI ATFCMLMPEE
351 NFTADHPFLF FIRHNSSGSI LFLGRFSSP
```

Residue Number Increasing Mass Decreasing Mass

Start - End	Observed	Mr(expt)	Mr(calc)	Delta	Miss	Sequence
57 - 69	1602.7355	1601.7282	1601.7586	-0.0304	0	TFHFNTVEEVHSR (Ions score 51)
57 - 69	1602.7355	1601.7282	1601.7586	-0.0304	0	TFHFNTVEEVHSR (No match)
70 - 80	1305.6648	1304.6575	1304.6836	-0.0261	1	FQSLNADINKR (No match)
97 - 110	1686.8387	1685.8314	1685.8664	-0.0350	0	TYNFLPEFLVSTQK (No match)
97 - 110	1686.8387	1685.8314	1685.8664	-0.0350	0	TYNFLPEFLVSTQK (Ions score 69)
111 - 129	2052.8877	2051.8804	2051.9184	-0.0380	0	TYGADLASVDFQHASEDAR (Ions score 135)
111 - 129	2052.8877	2051.8804	2051.9184	-0.0380	0	TYGADLASVDFQHASEDAR (No match)
111 - 130	2180.9773	2179.9700	2180.0134	-0.0433	1	TYGADLASVDFQHASEDARK (No match)
204 - 213	1218.5730	1217.5657	1217.5968	-0.0310	0	FAYGYIEDLK (No match)

276 - 290	1785.8667	1784.8594	1784.8944	-0.0350	1	FKLEESYTLNSDLAR	(Ions score 123)
276 - 290	1785.8667	1784.8594	1784.8944	-0.0350	1	FKLEESYTLNSDLAR	(No match)
364 - 375	1287.6567	1286.6494	1286.6731	-0.0236	0	HNSSGSILFLGR	(No match)

Spot 550

Protein View

Match to: **gi|1197210** Score: **493** Expect: **9.6e-045**
adenosine deaminase [Homo sapiens]

Nominal mass (M_r): **35335;** Calculated pI value: **5.60**
NCBI BLAST search of gi|1197210 against nr
Unformatted sequence string for pasting into other applications

Taxonomy: Homo sapiens

Fixed modifications: Carbamidomethyl (C)
Variable modifications: Oxidation (M)
Cleavage by Trypsin: cuts C-term side of KR unless next residue is P
Sequence Coverage: **46%**

Matched peptides shown in **Bold Red**

```
  1 MAQTPAFDKP KVELHVHLDG SIKPETILYY GRDKPLTLPD FLAKFDYYMP
 51 AIAGCREAIK RIAYEFVEMK AKEGVVYVEV RYSPHLLANS KVEPIPWNQA
101 EGDLTPDEVV ALVGQGLQEG ERDFGVKARS ILCCMRHQPI LPGHVQAYQE
151 AVKSGIHRTV HAGEVGSAEV VKEAVDILKT ERLGHGYHTL EDQALYNRLR
201 QENMHFEICP WSSYLTGAWK PDTEHAVIRL KNDQANYSLN TDDPLIFKST
251 LDTDYQMTKR DMGFTEEEFK RLNINAAKSS FLPEDEKREL LDLLYKAYGM
301 PPSASAGQNL
```

Residue Number Increasing Mass Decreasing Mass

Start - End	Observed	Mr(expt)	Mr(calc)	Delta	Miss	Sequence
12 - 32	2439.2883	2438.2810	2438.2957	-0.0147	0	VELHVHLDGSIKPETILYYGR (Ions score 131)
12 - 32	2439.2883	2438.2810	2438.2957	-0.0147	0	VELHVHLDGSIKPETILYYGR (No match)
45 - 56	1463.6455	1462.6382	1462.6373	0.0009	0	FDYYMPAIAGCR (Ions score 33)
45 - 56	1463.6455	1462.6382	1462.6373	0.0009	0	FDYYMPAIAGCR (No match)
45 - 56	1479.6372	1478.6299	1478.6322	-0.0023	0	FDYYMPAIAGCR Oxidation (M) (No match)
45 - 56	1479.6372	1478.6299	1478.6322	-0.0023	0	FDYYMPAIAGCR Oxidation (M) (Ions score 32)
62 - 70	1145.5718	1144.5645	1144.5474	0.0171	0	IAYEFVEMK Oxidation (M) (No match)
71 - 81	1248.7007	1247.6934	1247.6873	0.0061	1	AKEGVVYVEVR (No match)
73 - 81	1049.5703	1048.5630	1048.5552	0.0078	0	EGVVYVEVR (No match)
82 - 91	1129.5854	1128.5781	1128.5927	-0.0146	0	YSPHLLANSK (No match)

159 - 179	2151.1641	2150.1568	2150.1582	-0.0013	1	**TVHAGEVGSAEVVKEAVDILK**	(No match)
173 - 182	1173.6658	1172.6585	1172.6400	0.0185	1	**EAVDILKTER**	(No match)
183 - 198	1886.9070	1885.8997	1885.9070	-0.0073	0	**LGHGYHTLEDQALYNR**	(Ions score 112)
183 - 198	1886.9070	1885.8997	1885.9070	-0.0073	0	**LGHGYHTLEDQALYNR**	(No match)
230 - 248	2209.1138	2208.1065	2208.1062	0.0003	1	**LKNDQANYSLNTDDPLIFK**	(No match)
261 - 271	1388.6198	1387.6125	1387.6078	0.0048	1	**DMGFTEEEFKR**	(No match)
261 - 271	1404.6072	1403.5999	1403.6027	-0.0027	1	**DMGFTEEEFKR**	Oxidation (M) (No match)
261 - 271	1404.6072	1403.5999	1403.6027	-0.0027	1	**DMGFTEEEFKR**	Oxidation (M) (Ions score 51)
279 - 288	1207.5985	1206.5912	1206.5880	0.0032	1	**SSFLPEDEKR**	(No match)

Spot 551

Protein View

Match to: **gi|7706495** Score: **311** Expect: **1.5e-026**
DnaJ (Hsp40) homolog, subfamily B, member 11 precursor [Homo sapiens]

Nominal mass (M_r): **40774;** Calculated pI value: **5.81**
NCBI BLAST search of gi|7706495 against nr
Unformatted sequence string for pasting into other applications

Taxonomy: Homo sapiens
Links to retrieve other entries containing this sequence from NCBI Entrez:
gi|18203497 from Homo sapiens
gi|75041890 from Pongo pygmaeus
gi|7385135 from Homo sapiens
gi|6567166 from Homo sapiens
gi|6688203 from Homo sapiens
gi|12654615 from Homo sapiens
gi|22761515 from Homo sapiens
gi|30582965 from Homo sapiens
gi|37183204 from Homo sapiens
gi|55728952 from Pongo pygmaeus
gi|60655505 from synthetic construct
gi|60655507 from synthetic construct
gi|119598596 from Homo sapiens
gi|123994387 from synthetic construct
gi|124126891 from synthetic construct

Fixed modifications: Carbamidomethyl (C)
Variable modifications: Oxidation (M)
Cleavage by Trypsin: cuts C-term side of KR unless next residue is P
Sequence Coverage: **31%**

Matched peptides shown in **Bold Red**

1 MAPQNLSTFC LLLLYLIGAV IAGRDFYKIL GVPRSASIKD IKKAYRK**LAL**
51 **QLHPDRNPDD PQAQEKFQDL GAAYEVLSDS EKRKQYDTYG EEGLK**DGHQS
101 SHGDIFSHFF GDFGFMFGGT PRQQDRNIPR GSDIIVDLEV TLEEVYAGNF
151 VEVVRNKPVA RQAPGKRKCN CRQEMRTTQL GPGR**FQMTQE VVCDECPNVK**
201 LVNEER**TLEV EIEPGVRDGM EYPFIGEGEP HVDGEPGDLR** FRIKVVKHPI
251 FERRGDDLYT NVTISLVESL VGFEMDITHL DGHKVHISRD KITRPGAKLW
301 K**KGEGLPNFD NNNIK**GSLII TFDVDFPKEQ LTEEAREGIK QLLKQGSVQK
351 VYNGLQGY

Residue Number Increasing Mass Decreasing Mass

Start - End	Observed	Mr(expt)	Mr(calc)	Delta	Miss	Sequence
48 - 66	2185.1196	2184.1123	2184.0923	0.0200	1	LALQLHPDRNPDDPQAQEK (No match)
48 - 66	2185.1196	2184.1123	2184.0923	0.0200	1	LALQLHPDRNPDDPQAQEK (Ions score 22)
67 - 82	1771.8545	1770.8472	1770.8311	0.0161	0	FQDLGAAYEVLSDSEK (No match)
67 - 83	1927.9525	1926.9452	1926.9322	0.0130	1	FQDLGAAYEVLSDSEKR (No match)
67 - 83	1927.9525	1926.9452	1926.9322	0.0130	1	FQDLGAAYEVLSDSEKR (Ions score 94)
84 - 95	1430.6873	1429.6800	1429.6725	0.0076	1	KQYDTYGEEGLK (No match)
84 - 95	1430.6873	1429.6800	1429.6725	0.0076	1	KQYDTYGEEGLK (No match)
185 - 200	1999.8790	1998.8717	1998.8485	0.0232	0	FQMTQEVVCDECPNVK Oxidation (M) (No match)
207 - 217	1241.6780	1240.6707	1240.6662	0.0045	0	TLEVEIEPGVR (Ions score 33)
207 - 217	1241.6780	1240.6707	1240.6662	0.0045	0	TLEVEIEPGVR (No match)
218 - 240	2532.1284	2531.1211	2531.0910	0.0302	0	DGMEYPFIGEGEPHVDGEPGDLR Oxidation (M) (No match)
218 - 240	2532.1284	2531.1211	2531.0910	0.0302	0	DGMEYPFIGEGEPHVDGEPGDLR Oxidation (M) (Ions score 42)
302 - 315	1559.7908	1558.7835	1558.7739	0.0096	1	KGEGLPNFDNNNIK (No match)
303 - 315	1431.6934	1430.6861	1430.6789	0.0072	0	GEGLPNFDNNNIK (No match)

Spot 552

Protein View

Match to: **gi|15277503** Score: **209** Expect: **2.4e-016**
ACTB protein [Homo sapiens]

Nominal mass (M_r): **40536;** Calculated pI value: **5.55**
NCBI BLAST search of gi|15277503 against nr
Unformatted sequence string for pasting into other applications

Taxonomy: Homo sapiens

Fixed modifications: Carbamidomethyl (C)
Variable modifications: Oxidation (M)
Cleavage by Trypsin: cuts C-term side of KR unless next residue is P
Sequence Coverage: **12%**

Matched peptides shown in **Bold Red**

```
  1 MCKAGFAGDD APRAVFPSIV GRPRHQGVMV GMGQKDSYVG DEAQSKRGIL
 51 TLKYPIEHGI VTNWDDMEKI WHHTFYNELR VAPEEHPVLL TEAPLNPKAN
101 LEKMTQIMFE TFNTPAMYVA IQAVLSLYAS GRTTGIVMDS GDGVTHTVPI
151 YEGYALPHAI LRLDLAGRDL TDYLMKILTE RGYSFTTTAE REIVRDIKEK
201 LCYVALDFEQ EMATAASSSS LEKSYELPDG QVITIGNERF RCPEALFQPS
251 FLGMESCGIH ETTFNSIMKC DVDIRKDLYA NTVLSGGTTM YPGIADRMQK
301 EITALAPSTM KIKIIAPPER KYSVWIGGSI LASLSTFQQM WISKQEYDES
351 GPSIVHRKCF
```

Residue Number Increasing Mass Decreasing Mass

Start - End	Observed	Mr(expt)	Mr(calc)	Delta	Miss	Sequence
14 - 24	1198.7277	1197.7204	1197.6982	0.0223	0	AVFPSIVGRPR (Ions score 25)
14 - 24	1198.7277	1197.7204	1197.6982	0.0223	0	AVFPSIVGRPR (No match)
81 - 98	1954.1052	1953.0979	1953.0571	0.0409	0	VAPEEHPVLLTEAPLNPK (No match)
81 - 98	1954.1052	1953.0979	1953.0571	0.0409	0	VAPEEHPVLLTEAPLNPK (Ions score 71)
224 - 239	1790.9252	1789.9179	1789.8846	0.0334	0	SYELPDGQVITIGNER (Ions score 84)
224 - 239	1790.9252	1789.9179	1789.8846	0.0334	0	SYELPDGQVITIGNER (No match)

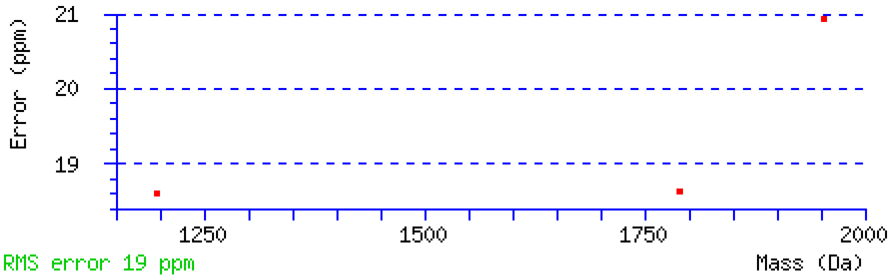
21
20
19
Error (ppm)
1250
1500
1750
2000
Mass (Da)
RMS error 19 ppm

Spot 553

Protein View

Match to: **gi|1197210** Score: **211** Expect: **1.5e-016**
adenosine deaminase [Homo sapiens]

Nominal mass (M_r): **35335;** Calculated pI value: **5.60**
NCBI BLAST search of gi|1197210 against nr
Unformatted sequence string for pasting into other applications

Taxonomy: Homo sapiens

Fixed modifications: Carbamidomethyl (C)
Variable modifications: Oxidation (M)
Cleavage by Trypsin: cuts C-term side of KR unless next residue is P
Sequence Coverage: **38%**

Matched peptides shown in **Bold Red**

```
  1 MAQTPAFDKP KVELHVHLDG SIKPETILYY GRDKPLTLPD FLAKFDYYMP
 51 AIAGCREAIK RIAYEFVEMK AKEGVVYVEV RYSPHLLANS KVEPIPWNQA
101 EGDLTPDEVV ALVGQGLQEG ERDFGVKARS ILCCMRHQPI LPGHVQAYQE
151 AVKSGIHRTV HAGEVGSAEV VKEAVDILKT ERLGHGYHTL EDQALYNRLR
201 QENMHFEICP WSSYLTGAWK PDTEHAVIRL KNDQANYSLN TDDPLIFKST
251 LDTDYQMTKR DMGFTEEEFK RLNINAAKSS FLPEDEKREL LDLLYKAYGM
301 PPSASAGQNL
```

Residue Number Increasing Mass Decreasing Mass

Start - End	Observed	Mr(expt)	Mr(calc)	Delta	Miss	Sequence
12 - 32	2439.1531	2438.1458	2438.2957	-0.1499	0	VELHVHLDGSIKPETILYYGR (No match)
45 - 56	1479.5577	1478.5504	1478.6322	-0.0818	0	FDYYMPAIAGCR Oxidation (M) (No match)
62 - 70	1129.5343	1128.5270	1128.5525	-0.0255	0	IAYEFVEMK (No match)
62 - 70	1145.5061	1144.4988	1144.5474	-0.0486	0	IAYEFVEMK Oxidation (M) (No match)
71 - 81	1248.6305	1247.6232	1247.6873	-0.0641	1	AKEGVVYVEVR (No match)
73 - 81	1049.5048	1048.4975	1048.5552	-0.0577	0	EGVVYVEVR (Ions score 10)
73 - 81	1049.5048	1048.4975	1048.5552	-0.0577	0	EGVVYVEVR (No match)
173 - 182	1173.5997	1172.5924	1172.6400	-0.0476	1	EAVDILKTER (No match)
183 - 198	1886.8025	1885.7952	1885.9070	-0.1118	0	LGHGYHTLEDQALYNR (Ions score 38)
183 - 198	1886.8025	1885.7952	1885.9070	-0.1118	0	LGHGYHTLEDQALYNR (No match)

230 - 248	2208.9912	2207.9839	2208.1062	-0.1223	1	**LKNDQANYSLNTDDPLIFK**	(No match)
261 - 271	1404.5287	1403.5214	1403.6027	-0.0812	1	**DMGFTEEEFKR**	Oxidation (M) (No match)
261 - 271	1404.5287	1403.5214	1403.6027	-0.0812	1	**DMGFTEEEFKR**	Oxidation (M) (Ions score 19)
279 - 288	1207.5326	1206.5253	1206.5880	-0.0627	1	**SSFLPEDEKR**	(Ions score 15)
279 - 288	1207.5326	1206.5253	1206.5880	-0.0627	1	**SSFLPEDEKR**	(No match)

Spot 554

Protein View

Match to: **gi|119591666** Score: **210** Expect: **1.9e-016**
septin 2, isoform CRA_c [Homo sapiens]

Nominal mass (M_r): **18259**; Calculated pI value: **6.20**
NCBI BLAST search of gi|119591666 against nr
Unformatted sequence string for pasting into other applications

Taxonomy: Homo sapiens

Fixed modifications: Carbamidomethyl (C)
Variable modifications: Oxidation (M)
Cleavage by Trypsin: cuts C-term side of KR unless next residue is P
Sequence Coverage: **59%**

Matched peptides shown in **Bold Red**

```
  1 MSKQQPTQFI NPETPGYVGF ANLPNQVHRK SVKKGFEFTL MVVGESGLGK
 51 STLINSLFLT DLYPERVIPG AAEKIERTVQ IEASTVEIEE RGVKLRLTVV
101 DTPGYGDAIN CRDCFKTIIS YIDEQFERYL HDESGLNRRH IIDNRVHCCF
151 YFISPFGHG
```

Residue Number Increasing Mass Decreasing Mass

Start - End	Observed	Mr(expt)	Mr(calc)	Delta	Miss	Sequence
4 - 29	2952.2407	2951.2334	2951.4677	-0.2343	0	QQPTQFINPETPGYVGFANLPNQVHR (Ions score 42)
4 - 29	2952.2407	2951.2334	2951.4677	-0.2343	0	QQPTQFINPETPGYVGFANLPNQVHR (No match)
51 - 66	1881.8687	1880.8614	1880.9883	-0.1269	0	STLINSLFLTDLYPER (Ions score 32)
51 - 66	1881.8687	1880.8614	1880.9883	-0.1269	0	STLINSLFLTDLYPER (No match)
78 - 91	1603.7152	1602.7079	1602.8100	-0.1021	0	TVQIEASTVEIEER (No match)
78 - 91	1603.7152	1602.7079	1602.8100	-0.1021	0	TVQIEASTVEIEER (Ions score 11)
97 - 112	1750.7285	1749.7212	1749.8355	-0.1143	0	LTVVDTPGYGDAINCR (No match)
117 - 128	1513.6565	1512.6492	1512.7460	-0.0967	0	TIISYIDEQFER (Ions score 40)
117 - 128	1513.6565	1512.6492	1512.7460	-0.0967	0	TIISYIDEQFER (No match)
129 - 139	1359.5885	1358.5812	1358.6691	-0.0878	1	YLHDESGLNRR (No match)
129 - 139	1359.5885	1358.5812	1358.6691	-0.0878	1	YLHDESGLNRR (Ions score 11)

Spot 555

Protein View

Match to: **gi|4557305** Score: **291** Expect: **1.5e-024**
aldolase A [Homo sapiens]

Nominal mass (M_r): **39851**; Calculated pI value: **8.30**
NCBI BLAST search of gi|4557305 against nr
Unformatted sequence string for pasting into other applications

Taxonomy: Homo sapiens
Links to retrieve other entries containing this sequence from NCBI Entrez:
gi|34577110 from Homo sapiens
gi|34577112 from Homo sapiens
gi|113606 from Homo sapiens
gi|28597 from Homo sapiens
gi|178351 from Homo sapiens
gi|13279257 from Homo sapiens
gi|14715001 from Homo sapiens
gi|15277571 from Homo sapiens
gi|15488981 from Homo sapiens
gi|16198435 from Homo sapiens
gi|16877049 from Homo sapiens
gi|49168540 from Homo sapiens
gi|119600339 from Homo sapiens
gi|119600340 from Homo sapiens
gi|119600341 from Homo sapiens
gi|123980324 from synthetic construct
gi|123995141 from synthetic construct

Fixed modifications: Carbamidomethyl (C)
Variable modifications: Oxidation (M)
Cleavage by Trypsin: cuts C-term side of KR unless next residue is P
Sequence Coverage: **35%**

Matched peptides shown in **Bold Red**

1 M**PYQYPALTP EQKKELSDIA HR**IVAPGK**GI LAADESTGSI AKRLQSIGTE**
51 **NTEENRR**FYR QLLLTADDRV NPCIGGVILF HETLYQK**ADD GRPFPQVIK**S
101 KGGVVGIKVD KGVVPLAGTN GETTTQGLDG LSERCAQYKK DGADFAKWRC
151 VLK**IGEHTPS ALAIMENANV LAR**YASICQQ NGIVPIVEPE ILPDGDHDLK
201 RCQYVTEKVL AAVYKALSDH HIYLEGTLLK PNMVTPGHAC TQK**FSHEEIA**
251 **MATVTALRR**T VPPAVTGITF LSGGQSEEEA SINLNAINKC PLLKPWALTF
301 SYGRALQASA LKAWGGKKEN LK**AAQEEYVK R**ALANSLACQ GK**YTPSGQAG**

351 AAASESLFVS NHAY

Residue Number Increasing Mass Decreasing Mass

Start - End	Observed	Mr(expt)	Mr(calc)	Delta	Miss	Sequence
2 - 13	1434.6515	1433.6442	1433.7190	-0.0748	0	PYQYPALTPEQK (No match)
2 - 14	1562.7400	1561.7327	1561.8140	-0.0813	1	PYQYPALTPEQKK (No match)
15 - 22	940.4355	939.4282	939.4774	-0.0491	0	ELSDIAHR (No match)
29 - 43	1488.7301	1487.7228	1487.7943	-0.0715	1	GILAADESTGSIAKR (No match)
44 - 57	1646.7314	1645.7241	1645.8019	-0.0778	1	LQSIGTENTEENRR (No match)
44 - 57	1646.7314	1645.7241	1645.8019	-0.0778	1	LQSIGTENTEENRR (Ions score 37)
88 - 99	1342.6449	1341.6376	1341.7040	-0.0664	0	ADDGRPFPQVIK (Ions score 51)
88 - 99	1342.6449	1341.6376	1341.7040	-0.0664	0	ADDGRPFPQVIK (No match)
154 - 173	2122.9934	2121.9861	2122.0840	-0.0979	0	IGEHTPSALAIMENANVLAR Oxidation (M) (No match)
154 - 173	2122.9934	2121.9861	2122.0840	-0.0979	0	IGEHTPSALAIMENANVLAR Oxidation (M) (Ions score 40)
244 - 258	1691.7543	1690.7470	1690.8348	-0.0878	0	FSHEEIAMATVTALR Oxidation (M) (No match)
244 - 259	1847.8541	1846.8468	1846.9359	-0.0891	1	FSHEEIAMATVTALRR Oxidation (M) (No match)
323 - 331	1093.5074	1092.5001	1092.5563	-0.0562	1	AAQEEYVKR (No match)
323 - 331	1093.5074	1092.5001	1092.5563	-0.0562	1	AAQEEYVKR (Ions score 35)
343 - 364	2227.9253	2226.9180	2227.0181	-0.1001	0	YTPSGQAGAAASESLFVSNHAY (No match)

Spot 557

Protein View

Match to: **gi|4557305** Score: **386** Expect: **4.8e-034**
aldolase A [Homo sapiens]

Nominal mass (M_r): **39851**; Calculated pI value: **8.30**
NCBI BLAST search of gi|4557305 against nr
Unformatted sequence string for pasting into other applications

Taxonomy: Homo sapiens
Links to retrieve other entries containing this sequence from NCBI Entrez:
gi|34577110 from Homo sapiens
gi|34577112 from Homo sapiens
gi|113606 from Homo sapiens
gi|28597 from Homo sapiens
gi|178351 from Homo sapiens
gi|13279257 from Homo sapiens
gi|14715001 from Homo sapiens
gi|15277571 from Homo sapiens
gi|15488981 from Homo sapiens
gi|16198435 from Homo sapiens
gi|16877049 from Homo sapiens
gi|49168540 from Homo sapiens
gi|119600339 from Homo sapiens
gi|119600340 from Homo sapiens
gi|119600341 from Homo sapiens
gi|123980324 from synthetic construct
gi|123995141 from synthetic construct

Fixed modifications: Carbamidomethyl (C)
Variable modifications: Oxidation (M)
Cleavage by Trypsin: cuts C-term side of KR unless next residue is P
Sequence Coverage: **53%**

Matched peptides shown in **Bold Red**

```
  1 MPYQYPALTP EQKKELSDIA HRIVAPGKGI LAADESTGSI AKRLQSIGTE
 51 NTEENRRFYR QLLLTADDRV NPCIGGVILF HETLYQKADD GRPFPQVIKS
101 KGGVVGIKVD KGVVPLAGTN GETTTQGLDG LSERCAQYKK DGADFAKWRC
151 VLKIGEHTPS ALAIMENANV LARYASICQQ NGIVPIVEPE ILPDGDHDLK
201 RCQYVTEKVL AAVYKALSDH HIYLEGTLLK PNMVTPGHAC TQKFSHEEIA
251 MATVTALRRT VPPAVTGITF LSGGQSEEEA SINLNAINKC PLLKPWALTF
301 SYGRALQASA LKAWGGKKEN LKAAQEEYVK RALANSLACQ GKYTPSGQAG
```

351 **AAASESLFVS NHAY**

Residue Number Increasing Mass Decreasing Mass

Start - End	Observed	Mr(expt)	Mr(calc)	Delta	Miss	Sequence
2 - 13	1434.6697	1433.6624	1433.7190	-0.0566	0	PYQYPALTPEQK (No match)
2 - 14	1562.7700	1561.7627	1561.8140	-0.0513	1	PYQYPALTPEQKK (No match)
15 - 22	940.4482	939.4409	939.4774	-0.0364	0	ELSDIAHR (No match)
29 - 43	1488.7504	1487.7431	1487.7943	-0.0512	1	GILAADESTGSIAKR (No match)
44 - 57	1646.7537	1645.7464	1645.8019	-0.0555	1	LQSIGTENTEENRR (No match)
61 - 87	3113.5088	3112.5015	3112.6378	-0.1363	1	QLLLTADDRVNPCIGGVILFHETLYQK (No match)
61 - 87	3113.5088	3112.5015	3112.6378	-0.1363	1	QLLLTADDRVNPCIGGVILFHETLYQK (Ions score 13)
88 - 99	1342.6659	1341.6586	1341.7040	-0.0454	0	ADDGRPFPQVIK (No match)
88 - 99	1342.6659	1341.6586	1341.7040	-0.0454	0	ADDGRPFPQVIK (Ions score 80)
112 - 134	2272.0566	2271.0493	2271.1342	-0.0848	0	GVVPLAGTNGETTTQGLDGLSER (No match)
154 - 173	2123.0151	2122.0078	2122.0840	-0.0762	0	IGEHTPSALAIMENANVLAR Oxidation (M) (No match)
154 - 173	2123.0151	2122.0078	2122.0840	-0.0762	0	IGEHTPSALAIMENANVLAR Oxidation (M) (Ions score 46)
244 - 258	1691.7808	1690.7735	1690.8348	-0.0613	0	FSHEEIAMATVTALR Oxidation (M) (No match)
244 - 259	1847.8788	1846.8715	1846.9359	-0.0644	1	FSHEEIAMATVTALRR Oxidation (M) (Ions score 25)
244 - 259	1847.8788	1846.8715	1846.9359	-0.0644	1	FSHEEIAMATVTALRR Oxidation (M) (No match)
290 - 304	1808.8860	1807.8787	1807.9443	-0.0656	0	CPLLKPWALTFSYGR (No match)
323 - 331	1093.5255	1092.5182	1092.5563	-0.0381	1	AAQEEYVKR (Ions score 44)
323 - 331	1093.5255	1092.5182	1092.5563	-0.0381	1	AAQEEYVKR (No match)
343 - 364	2227.9502	2226.9429	2227.0181	-0.0752	0	YTPSGQAGAAASESLFVSNHAY (No match)

Spot 558

Protein View

Match to: **gi|14530107** Score: **204** Expect: **7.7e-016**
OVARIAN/Breast septin delta [Homo sapiens]

Nominal mass (M_r): **38690**; Calculated pI value: **7.13**
NCBI BLAST search of gi|14530107 against nr
Unformatted sequence string for pasting into other applications

Taxonomy: Homo sapiens
Links to retrieve other entries containing this sequence from NCBI Entrez:
gi|119609869 from Homo sapiens

Fixed modifications: Carbamidomethyl (C)
Variable modifications: Oxidation (M)
Cleavage by Trypsin: cuts C-term side of KR unless next residue is P
Sequence Coverage: **24%**

Matched peptides shown in **Bold Red**

```
  1 MADTPRDAGL KQAPASRNEK APVDFGYVGI DSILEQMRRK AMKQGFEFNI
 51 MVVGQSGLGK STLINTLFKS KISRKSVQPT SEERIPKTIE IKSITHDIEE
101 KGVRMKLTVI DTPGFGDHIN NENCWQPIMK FINDQYEKYL QEEVNINRKK
151 RIPDTRVHCC LYFIPATGHS LRPLDIEFMK RLSKVVNIVP VIAKADTLTL
201 EERVHFKQRI TADLLSNGID VYPQKEFDED SEDRLVNEKF REMIPFAVVG
251 SDHEYQVNGK RILGRKTKWG TIEVENTTHC EFAYLRDLLI RTHMQNIKDI
301 TSSIHFEAYR VKRLNEGSSA MANGVEEKEP EAPEM
```

Residue Number Increasing Mass Decreasing Mass

Start - End	Observed	Mr(expt)	Mr(calc)	Delta	Miss	Sequence
21 - 38	2009.9934	2008.9861	2008.9927	-0.0066	0	APVDFGYVGIDSILEQMR (No match)
21 - 38	2009.9934	2008.9861	2008.9927	-0.0066	0	APVDFGYVGIDSILEQMR (Ions score 40)
44 - 60	1810.9303	1809.9230	1809.9082	0.0148	0	QGFEFNIMVVGQSGLGK (No match)
131 - 138	1056.4916	1055.4843	1055.4923	-0.0080	0	FINDQYEK (No match)
139 - 148	1277.6544	1276.6471	1276.6411	0.0060	0	YLQEEVNINR (Ions score 33)
139 - 148	1277.6544	1276.6471	1276.6411	0.0060	0	YLQEEVNINR (No match)
269 - 286	2226.0178	2225.0105	2225.0211	-0.0106	0	WGTIEVENTTHCEFAYLR (No match)
269 - 286	2226.0178	2225.0105	2225.0211	-0.0106	0	WGTIEVENTTHCEFAYLR (Ions score 40)

299 - 310	1438.6996	1437.6923	1437.6888	0.0035	0	DITSSIHFEAYR	(No match)
299 - 310	1438.6996	1437.6923	1437.6888	0.0035	0	DITSSIHFEAYR	(Ions score 25)

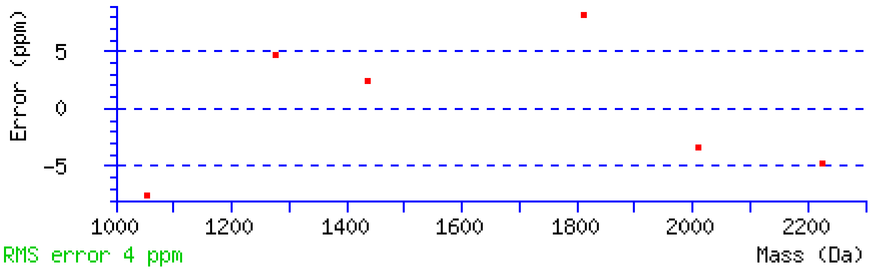

Spot 559

Protein View

Match to: **gi|4557305** Score: **150** Expect: **1.9e-010**
aldolase A [Homo sapiens]

Nominal mass (M_r): **39851**; Calculated pI value: **8.30**
NCBI BLAST search of gi|4557305 against nr
Unformatted sequence string for pasting into other applications

Taxonomy: Homo sapiens
Links to retrieve other entries containing this sequence from NCBI Entrez:
gi|34577110 from Homo sapiens
gi|34577112 from Homo sapiens
gi|113606 from Homo sapiens
gi|28597 from Homo sapiens
gi|178351 from Homo sapiens
gi|13279257 from Homo sapiens
gi|14715001 from Homo sapiens
gi|15277571 from Homo sapiens
gi|15488981 from Homo sapiens
gi|16198435 from Homo sapiens
gi|16877049 from Homo sapiens
gi|49168540 from Homo sapiens
gi|119600339 from Homo sapiens
gi|119600340 from Homo sapiens
gi|119600341 from Homo sapiens
gi|123980324 from synthetic construct
gi|123995141 from synthetic construct

Fixed modifications: Carbamidomethyl (C)
Variable modifications: Oxidation (M)
Cleavage by Trypsin: cuts C-term side of KR unless next residue is P
Sequence Coverage: **29%**

Matched peptides shown in **Bold Red**

```
  1 MPYQYPALTP EQKKELSDIA HRIVAPGKGI LAADESTGSI AKRLQSIGTE
 51 NTEENRRFYR QLLLTADDRV NPCIGGVILF HETLYQKADD GRPFPQVIKS
101 KGGVVGIKVD KGVVPLAGTN GETTTQGLDG LSERCAQYKK DGADFAKWRC
151 VLKIGEHTPS ALAIMENANV LARYASICQQ NGIVPIVEPE ILPDGDHDLK
201 RCQYVTEKVL AAVYKALSDH HIYLEGTLLK PNMVTPGHAC TQKFSHEEIA
251 MATVTALRRT VPPAVTGITF LSGGQSEEEA SINLNAINKC PLLKPWALTF
301 SYGRALQASA LKAWGGKKEN LKAAQEEYVK RALANSLACQ GKYTPSGQAG
```

351 AAASESLFVS NHAY

Residue Number Increasing Mass Decreasing Mass

Start - End	Observed	Mr(expt)	Mr(calc)	Delta	Miss	Sequence
2 - 13	1434.6360	1433.6287	1433.7190	-0.0903	0	PYQYPALTPEQK (No match)
2 - 14	1562.7229	1561.7156	1561.8140	-0.0984	1	PYQYPALTPEQKK (No match)
44 - 57	1646.7085	1645.7012	1645.8019	-0.1007	1	LQSIGTENTEENRR (No match)
44 - 57	1646.7085	1645.7012	1645.8019	-0.1007	1	LQSIGTENTEENRR (Ions score 14)
88 - 99	1342.6327	1341.6254	1341.7040	-0.0786	0	ADDGRPFPQVIK (No match)
88 - 99	1342.6327	1341.6254	1341.7040	-0.0786	0	ADDGRPFPQVIK (Ions score 27)
154 - 173	2122.9546	2121.9473	2122.0840	-0.1367	0	IGEHTPSALAIMENANVLAR Oxidation (M) (No match)
154 - 173	2122.9546	2121.9473	2122.0840	-0.1367	0	IGEHTPSALAIMENANVLAR Oxidation (M) (No match)
244 - 258	1691.7393	1690.7320	1690.8348	-0.1028	0	FSHEEIAMATVTALR Oxidation (M) (No match)
244 - 259	1847.8282	1846.8209	1846.9359	-0.1150	1	FSHEEIAMATVTALRR Oxidation (M) (No match)
323 - 331	1093.5017	1092.4944	1092.5563	-0.0619	1	AAQEEYVKR (No match)
323 - 331	1093.5017	1092.4944	1092.5563	-0.0619	1	AAQEEYVKR (Ions score 11)
343 - 364	2227.8923	2226.8850	2227.0181	-0.1331	0	YTPSGQAGAAASESLFVSNHAY (No match)

Spot 560

Protein View

Match to: **gi|4557305** Score: **401** Expect: **1.5e-035**
aldolase A [Homo sapiens]

Nominal mass (M_r): **39851;** Calculated pI value: **8.30**
NCBI BLAST search of gi|4557305 against nr
Unformatted sequence string for pasting into other applications

Taxonomy: Homo sapiens
Links to retrieve other entries containing this sequence from NCBI Entrez:
gi|34577110 from Homo sapiens
gi|34577112 from Homo sapiens
gi|113606 from Homo sapiens
gi|28597 from Homo sapiens
gi|178351 from Homo sapiens
gi|13279257 from Homo sapiens
gi|14715001 from Homo sapiens
gi|15277571 from Homo sapiens
gi|15488981 from Homo sapiens
gi|16198435 from Homo sapiens
gi|16877049 from Homo sapiens
gi|49168540 from Homo sapiens
gi|119600339 from Homo sapiens
gi|119600340 from Homo sapiens
gi|119600341 from Homo sapiens
gi|123980324 from synthetic construct
gi|123995141 from synthetic construct

Fixed modifications: Carbamidomethyl (C)
Variable modifications: Oxidation (M)
Cleavage by Trypsin: cuts C-term side of KR unless next residue is P
Sequence Coverage: **54%**

Matched peptides shown in **Bold Red**

1 M**PYQYPALTP EQKKELSDIA HR**IVAPGK**GI LAADESTGSI AKRLQSIGTE**
51 **NTEENRR**FYR **QLLLTADDRV NPCIGGVILF HETLYQKADD GRPFPQVIK**S
101 KGGVVGIK**VD KGVVPLAGTN GETTTQGLDG LSER**CAQYKK DGADFAKWRC
151 VLK**IGEHTPS ALAIMENANV LAR**YASICQQ NGIVPIVEPE ILPDGDHDLK
201 RCQYVTEKVL AAVYKALSDH HIYLEGTLLK PNMVTPGHAC TQK**FSHEEIA**
251 **MATVTALRR**T VPPAVTGITF LSGGQSEEEA SINLNAINK**C PLLKPWALTF**
301 **SYGR**ALQASA LKAWGGKKEN LK**AAQEEYVK R**ALANSLACQ GK**YTPSGQAG** 825

351 AAASESLFVS NHAY

Residue Number Increasing Mass Decreasing Mass

Start - End	Observed	Mr(expt)	Mr(calc)	Delta	Miss	Sequence
2 - 13	1434.6482	1433.6409	1433.7190	-0.0781	0	PYQYPALTPEQK (No match)
2 - 14	1562.7395	1561.7322	1561.8140	-0.0818	1	PYQYPALTPEQKK (No match)
15 - 22	940.4352	939.4279	939.4774	-0.0494	0	ELSDIAHR (No match)
29 - 43	1488.7252	1487.7179	1487.7943	-0.0764	1	GILAADESTGSIAKR (No match)
44 - 56	1490.6749	1489.6676	1489.7008	-0.0332	0	LQSIGTENTEENR (No match)
44 - 57	1646.7252	1645.7179	1645.8019	-0.0840	1	LQSIGTENTEENRR (Ions score 47)
44 - 57	1646.7252	1645.7179	1645.8019	-0.0840	1	LQSIGTENTEENRR (No match)
61 - 87	3113.4360	3112.4287	3112.6378	-0.2091	1	QLLLTADDRVNPCIGGVILFHETLYQK (No match)
88 - 99	1342.6442	1341.6369	1341.7040	-0.0671	0	ADDGRPFPQVIK (Ions score 70)
88 - 99	1342.6442	1341.6369	1341.7040	-0.0671	0	ADDGRPFPQVIK (No match)
109 - 134	2614.1863	2613.1790	2613.3245	-0.1455	1	VDKGVVPLAGTNGETTTQGLDGLSER (No match)
112 - 134	2272.0115	2271.0042	2271.1342	-0.1299	0	GVVPLAGTNGETTTQGLDGLSER (No match)
154 - 173	2122.9780	2121.9707	2122.0840	-0.1133	0	IGEHTPSALAIMENANVLAR Oxidation (M) (No match)
154 - 173	2122.9780	2121.9707	2122.0840	-0.1133	0	IGEHTPSALAIMENANVLAR Oxidation (M) (Ions score 36)
244 - 258	1691.7535	1690.7462	1690.8348	-0.0886	0	FSHEEIAMATVTALR Oxidation (M) (No match)
244 - 259	1831.8521	1830.8448	1830.9410	-0.0962	1	FSHEEIAMATVTALRR (No match)
244 - 259	1847.8434	1846.8361	1846.9359	-0.0998	1	FSHEEIAMATVTALRR Oxidation (M) (No match)
290 - 304	1808.8555	1807.8482	1807.9443	-0.0961	0	CPLLKPWALTFSYGR (No match)
323 - 331	1093.5093	1092.5020	1092.5563	-0.0543	1	AAQEEYVKR (Ions score 48)
323 - 331	1093.5093	1092.5020	1092.5563	-0.0543	1	AAQEEYVKR (No match)
343 - 364	2227.9082	2226.9009	2227.0181	-0.1172	0	YTPSGQAGAAASESLFVSNHAY (No match)

Spot 561

Protein View

Match to: **gi|6934244** Score: **188** Expect: **3e-014**
tropomodulin 3 [Homo sapiens]

Nominal mass (M_r): **39727;** Calculated pI value: **5.08**
NCBI BLAST search of gi|6934244 against nr
Unformatted sequence string for pasting into other applications

Taxonomy: Homo sapiens

Fixed modifications: Carbamidomethyl (C)
Variable modifications: Oxidation (M)
Cleavage by Trypsin: cuts C-term side of KR unless next residue is P
Sequence Coverage: **21%**

Matched peptides shown in **Bold Red**

```
  1 MALPFRKDLE KYKDLDEDEL LGNLSESELK QLETVLDDLD PENALLPAGF
 51 RQKNQTSKST TGPFDREHLL SYLEKEALEH KDREDYVPYT GEKKGKIFIP
101 KQKPVQTFTE EKVSLDPELE EALTSASDTE LCDLAAILGM HNLITNTKFC
151 NIMGSSNGVD QEHFSNVVKG EKILPVFDEP PNPTNVEESL KRTKENDAHL
201 VEVNLNNIKN IPIPTLKDFA KALETNTHVK CFSLAATRSN DPVATAFAEM
251 LKVNKTLKSL NVESNFITGV GILALIDALR DNETLAELKI DNQRQQLGTA
301 VELEMAKMLE ENTNILKFGY QFTQQGPRTR AANAITKNND LVRKRRVEGD
351 HQ
```

Residue Number Increasing Mass Decreasing Mass

Start - End	Observed	Mr(expt)	Mr(calc)	Delta	Miss	Sequence
59 - 75	1993.0232	1992.0159	1991.9952	0.0207	1	STTGPFDREHLLSYLEK (No match)
59 - 75	1993.0232	1992.0159	1991.9952	0.0207	1	STTGPFDREHLLSYLEK (No match)
82 - 93	1471.6832	1470.6759	1470.6626	0.0133	1	DREDYVPYTGEK (No match)
82 - 93	1471.6832	1470.6759	1470.6626	0.0133	1	DREDYVPYTGEK (Ions score 25)
173 - 192	2294.2268	2293.2195	2293.1953	0.0242	1	ILPVFDEPPNPTNVEESLKR (No match)
173 - 192	2294.2268	2293.2195	2293.1953	0.0242	1	ILPVFDEPPNPTNVEESLKR (Ions score 70)
239 - 252	1509.7384	1508.7311	1508.7180	0.0131	0	SNDPVATAFAEMLK Oxidation (M) (No match)
318 - 328	1328.6517	1327.6444	1327.6309	0.0135	0	FGYQFTQQGPR (Ions score 33)
318 - 328	1328.6517	1327.6444	1327.6309	0.0135	0	FGYQFTQQGPR (No match)

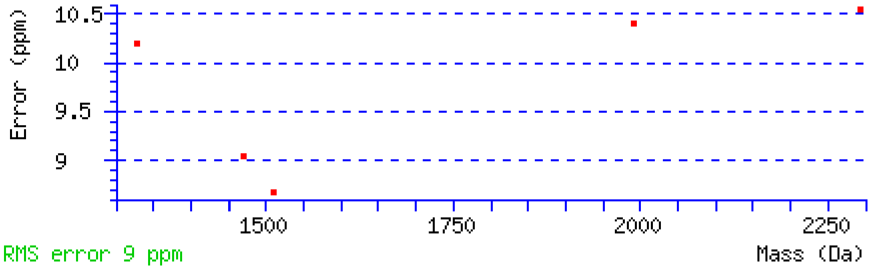

RMS error 9 ppm

Spot 562

Protein View

Match to: **gi|4557305** Score: **363** Expect: **9.6e-032**
aldolase A [Homo sapiens]

Nominal mass (M_r): **39851;** Calculated pI value: **8.30**
NCBI BLAST search of gi|4557305 against nr
Unformatted sequence string for pasting into other applications

Taxonomy: Homo sapiens
Links to retrieve other entries containing this sequence from NCBI Entrez:
gi|34577110 from Homo sapiens
gi|34577112 from Homo sapiens
gi|113606 from Homo sapiens
gi|28597 from Homo sapiens
gi|178351 from Homo sapiens
gi|13279257 from Homo sapiens
gi|14715001 from Homo sapiens
gi|15277571 from Homo sapiens
gi|15488981 from Homo sapiens
gi|16198435 from Homo sapiens
gi|16877049 from Homo sapiens
gi|49168540 from Homo sapiens
gi|119600339 from Homo sapiens
gi|119600340 from Homo sapiens
gi|119600341 from Homo sapiens
gi|123980324 from synthetic construct
gi|123995141 from synthetic construct

Fixed modifications: Carbamidomethyl (C)
Variable modifications: Oxidation (M)
Cleavage by Trypsin: cuts C-term side of KR unless next residue is P
Sequence Coverage: **36%**

Matched peptides shown in **Bold Red**

1 M**PYQYPALTP EQKKELSDIA HR**IVAPGKGI LAADESTGSI AKR**LQSIGTE**
51 **NTEENRR**FYR **QLLLTADDRV NPCIGGVILF HETLYQKADD GRPFPQVIK**S
101 KGGVVGIKVD KGVVPLAGTN GETTTQGLDG LSERCAQYKK DGADFAKWRC
151 VLK**IGEHTPS ALAIMENANV LAR**YASICQQ NGIVPIVEPE ILPDGDHDLK
201 RCQYVTEKVL AAVYKALSDH HIYLEGTLLK PNMVTPGHAC TQK**FSHEEIA**
251 **MATVTALRR**T VPPAVTGITF LSGGQSEEEA SINLNAINK**C PLLKPWALTF**
301 **SYGR**ALQASA LKAWGGKKEN LK**AAQEEYVK R**ALANSLACQ GKYTPSGQAG

351 AAASESLFVS NHAY

Residue Number Increasing Mass Decreasing Mass

Start - End	Observed	Mr(expt)	Mr(calc)	Delta	Miss	Sequence
2 - 13	1434.7460	1433.7387	1433.7190	0.0197	0	PYQYPALTPEQK (No match)
2 - 14	1562.8461	1561.8388	1561.8140	0.0248	1	PYQYPALTPEQKK (No match)
15 - 22	940.5029	939.4956	939.4774	0.0183	0	ELSDIAHR (No match)
44 - 57	1646.8326	1645.8253	1645.8019	0.0234	1	LQSIGTENTEENRR (No match)
61 - 87	3113.6621	3112.6548	3112.6378	0.0170	1	QLLLTADDRVNPCIGGVILFHETLYQK (No match)
88 - 99	1342.7332	1341.7259	1341.7040	0.0219	0	ADDGRPFPQVIK (No match)
88 - 99	1342.7332	1341.7259	1341.7040	0.0219	0	ADDGRPFPQVIK (Ions score 50)
154 - 173	2107.1187	2106.1114	2106.0891	0.0223	0	IGEHTPSALAIMENANVLAR (No match)
154 - 173	2107.1187	2106.1114	2106.0891	0.0223	0	IGEHTPSALAIMENANVLAR (Ions score 83)
154 - 173	2123.1152	2122.1079	2122.0840	0.0239	0	IGEHTPSALAIMENANVLAR Oxidation (M) (No match)
244 - 258	1675.8800	1674.8727	1674.8399	0.0328	0	FSHEEIAMATVTALR (No match)
244 - 259	1831.9719	1830.9646	1830.9410	0.0236	1	FSHEEIAMATVTALRR (No match)
244 - 259	1847.9633	1846.9560	1846.9359	0.0201	1	FSHEEIAMATVTALRR Oxidation (M) (No match)
290 - 304	1808.9751	1807.9678	1807.9443	0.0235	0	CPLLKPWALTFSYGR (Ions score 64)
290 - 304	1808.9751	1807.9678	1807.9443	0.0235	0	CPLLKPWALTFSYGR (No match)
323 - 331	1093.5797	1092.5724	1092.5563	0.0161	1	AAQEEYVKR (No match)
323 - 331	1093.5797	1092.5724	1092.5563	0.0161	1	AAQEEYVKR (Ions score 23)

Spot 563

Protein View

Match to: **gi|4885063** Score: **156** Expect: **4.8e-011**
fructose-bisphosphate aldolase C [Homo sapiens]

Nominal mass (M_r): **39830**; Calculated pI value: **6.41**
NCBI BLAST search of gi|4885063 against nr
Unformatted sequence string for pasting into other applications

Taxonomy: Homo sapiens
Links to retrieve other entries containing this sequence from NCBI Entrez:
gi|113613 from Homo sapiens
gi|28599 from Homo sapiens
gi|3005698 from Homo sapiens
gi|30582851 from Homo sapiens
gi|49456679 from Homo sapiens
gi|49456717 from Homo sapiens
gi|60655023 from synthetic construct
gi|60823250 from synthetic construct
gi|78070476 from Homo sapiens
gi|78070601 from Homo sapiens
gi|119571489 from Homo sapiens
gi|119571490 from Homo sapiens
gi|127795529 from Homo sapiens
gi|127799186 from Homo sapiens

Fixed modifications: Carbamidomethyl (C)
Variable modifications: Oxidation (M)
Cleavage by Trypsin: cuts C-term side of KR unless next residue is P
Sequence Coverage: **22%**

Matched peptides shown in **Bold Red**

1 MPHSYPALSA EQKKELSDIA LRIVAPGKGI LAADESVGSM AKR**LSQIGVE**
51 **NTEENRR**LYR QVLFSADDRV KKCIGGVIFF HETLYQKDDN GVPFVRTIQD
101 KGIVVGIKVD K**GVVPLAGTD GETTTQGLDG LSER**CAQYKK DGADFAKWRC
151 VLKISER**TPS ALAILENANV LAR**YASICQQ NGIVPIVEPE ILPDGDHDLK
201 RCQYVTEKVL AAVYKALSDH HVYLEGTLLK PNMVTPGHAC PIK**YTPEEIA**
251 **MATVTALRR**T VPPAVPGVTF LSGGQSEEEA SFNLNAINRC PLPRPWALTF
301 SYGRALQASA LNAWRGQR**DN AGAATEEFIK R**AEVNGLAAQ GKYEGSGEDG
351 GAAAQSLYIA NHAY

Residue Number Increasing Mass Decreasing Mass

Start - End	Observed	Mr(expt)	Mr(calc)	Delta	Miss	Sequence
44 - 57	1644.8271	1643.8198	1643.8226	-0.0028	1	LSQIGVENTEENRR (No match)
112 - 134	2273.1240	2272.1167	2272.1182	-0.0015	0	GVVPLAGTDGETTTQGLDGLSER (No match)
112 - 134	2273.1240	2272.1167	2272.1182	-0.0015	0	GVVPLAGTDGETTTQGLDGLSER (Ions score 19)
158 - 173	1652.9352	1651.9279	1651.9256	0.0023	0	TPSALAILENANVLAR (No match)
158 - 173	1652.9352	1651.9279	1651.9256	0.0023	0	TPSALAILENANVLAR (Ions score 59)
244 - 259	1837.9451	1836.9378	1836.9403	-0.0025	1	YTPEEIAMATVTALRR Oxidation (M) (No match)
244 - 259	1837.9451	1836.9378	1836.9403	-0.0025	1	YTPEEIAMATVTALRR Oxidation (M) (No match)
319 - 331	1421.7101	1420.7028	1420.6946	0.0082	1	DNAGAATEEFIKR (Ions score 12)
319 - 331	1421.7101	1420.7028	1420.6946	0.0082	1	DNAGAATEEFIKR (No match)

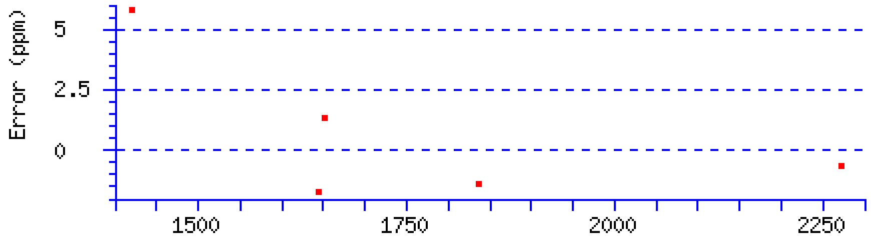

RMS error 2 ppm

Spot 564

Protein View

Match to: **gi|5031571** Score: **611** Expect: **1.5e-056**
actin-related protein 2 isoform b [Homo sapiens]

Nominal mass (M_r): **45017;** Calculated pI value: **6.30**
NCBI BLAST search of gi|5031571 against nr
Unformatted sequence string for pasting into other applications

Taxonomy: Homo sapiens
Links to retrieve other entries containing this sequence from NCBI Entrez:
gi|22122825 from Mus musculus
gi|156121073 (no taxonomy information for this entry)
gi|57093381 from Canis lupus familiaris
gi|109103168 from Macaca mulatta
gi|47117648 from Homo sapiens
gi|47117649 from Mus musculus
gi|17943200 from Bos taurus
gi|56966173 from Bos taurus
gi|56966193 from Bos taurus
gi|149243010 from Bos taurus
gi|149243032 from Bos taurus
gi|149243039 from Bos taurus
gi|149243046 from Bos taurus
gi|149243053 from Bos taurus
gi|149243060 from Bos taurus
gi|2282030 from Homo sapiens
gi|20380733 from Mus musculus
gi|26328619 from Mus musculus
gi|26353172 from Mus musculus
gi|56205119 from Mus musculus
gi|74190292 from Mus musculus
gi|119620314 from Homo sapiens
gi|119620316 from Homo sapiens
gi|119620318 from Homo sapiens
gi|119620319 from Homo sapiens
gi|148675879 from Mus musculus
gi|154425680 from Bos taurus

Fixed modifications: Carbamidomethyl (C)
Variable modifications: Oxidation (M)
Cleavage by Trypsin: cuts C-term side of KR unless next residue is P
Sequence Coverage: **52%**

Matched peptides shown in **Bold Red**

```
  1 MDSQGRKVVV CDNGTGFVKC GYAGSNFPEH IFPALVGRPI IRSTTKVGNI
 51 EIKDLMVGDE ASELRSMLEV NYPMENGIVR NWDDMKHLWD YTFGPEKLNI
101 DTRNCKILLT EPPMNPTKNR EKIVEVMFET YQFSGVYVAI QAVLTLYAQG
151 LLTGVVVDSG DGVTHICPVY EGFSLPHLTR RLDIAGRDIT RYLIKLLLLR
201 GYAFNHSADF ETVRMIKEKL CYVGYNIEQE QKLALETTVL VESYTLPDGR
251 IIKVGGERFE APEALFQPHL INVEGVGVAE LLFNTIQAAD IDTRSEFYKH
301 IVLSGGSTMY PGLPSRLERE LKQLYLERVL KGDVEKLSKF KIRIEDPPRR
351 KHMVFLGGAV LADIMKDKDN FWMTRQEYQE KGVRVLEKLG VTVR
```

Residue Number Increasing Mass Decreasing Mass

Start - End	Observed	Mr(expt)	Mr(calc)	Delta	Miss	Sequence
7 - 19	1422.7435	1421.7362	1421.7336	0.0026	1	KVVVCDNGTGFVK (No match)
20 - 42	2571.3152	2570.3079	2570.3215	-0.0136	0	CGYAGSNFPEHIFPALVGRPIIR (No match)
20 - 42	2571.3152	2570.3079	2570.3215	-0.0136	0	CGYAGSNFPEHIFPALVGRPIIR (Ions score 63)
47 - 65	2088.0579	2087.0506	2087.0568	-0.0062	1	VGNIEIKDLMVGDEASELR (No match)
54 - 65	1334.6228	1333.6155	1333.6183	-0.0028	0	DLMVGDEASELR (No match)
66 - 80	1751.8425	1750.8352	1750.8382	-0.0029	0	SMLEVNYPMENGIVR (Ions score 56)
66 - 80	1751.8425	1750.8352	1750.8382	-0.0029	0	SMLEVNYPMENGIVR (No match)
66 - 80	1767.8390	1766.8317	1766.8331	-0.0013	0	SMLEVNYPMENGIVR Oxidation (M) (No match)
66 - 80	1783.8372	1782.8299	1782.8280	0.0020	0	SMLEVNYPMENGIVR 2 Oxidation (M) (No match)
87 - 97	1392.6542	1391.6469	1391.6510	-0.0040	0	HLWDYTFGPEK (No match)
87 - 103	2105.0432	2104.0359	2104.0377	-0.0018	1	HLWDYTFGPEKLNIDTR (No match)
107 - 118	1353.7434	1352.7361	1352.7374	-0.0012	0	ILLTEPPMNPTK (No match)
181 - 187	800.4703	799.4630	799.4664	-0.0034	1	RLDIAGR (No match)
201 - 214	1613.7312	1612.7239	1612.7269	-0.0030	0	GYAFNHSADFETVR (Ions score 106)
201 - 214	1613.7312	1612.7239	1612.7269	-0.0030	0	GYAFNHSADFETVR (No match)
220 - 232	1643.7726	1642.7653	1642.7660	-0.0007	0	LCYVGYNIEQEQK (No match)
233 - 250	1977.0499	1976.0426	1976.0466	-0.0039	0	LALETTVLVESYTLPDGR (No match)
233 - 250	1977.0499	1976.0426	1976.0466	-0.0039	0	LALETTVLVESYTLPDGR (Ions score 86)
300 - 316	1771.9126	1770.9053	1770.9086	-0.0033	0	HIVLSGGSTMYPGLPSR (No match)
300 - 316	1771.9126	1770.9053	1770.9086	-0.0033	0	HIVLSGGSTMYPGLPSR (Ions score 44)
300 - 316	1787.9042	1786.8969	1786.9035	-0.0066	0	HIVLSGGSTMYPGLPSR Oxidation (M) (No match)
323 - 328	821.4495	820.4422	820.4443	-0.0020	0	QLYLER (No match)
342 - 349	995.5629	994.5556	994.5559	-0.0003	1	IRIEDPPR (No match)
352 - 366	1601.8544	1600.8471	1600.8469	0.0002	0	HMVFLGGAVLADIMK (No match)
367 - 375	1212.5466	1211.5393	1211.5393	0.0000	1	DKDNFWMTR (No match)
367 - 375	1228.5425	1227.5352	1227.5342	0.0010	1	DKDNFWMTR Oxidation (M) (No match)
369 - 375	969.4253	968.4180	968.4174	0.0006	0	DNFWMTR (No match)

Spot 565

Protein View

Match to: **gi|4885063** Score: **458** Expect: **3e-041**
fructose-bisphosphate aldolase C [Homo sapiens]

Nominal mass (M_r): **39830;** Calculated pI value: **6.41**
NCBI BLAST search of gi|4885063 against nr
Unformatted sequence string for pasting into other applications

Taxonomy: Homo sapiens
Links to retrieve other entries containing this sequence from NCBI Entrez:
gi|113613 from Homo sapiens
gi|28599 from Homo sapiens
gi|3005698 from Homo sapiens
gi|30582851 from Homo sapiens
gi|49456679 from Homo sapiens
gi|49456717 from Homo sapiens
gi|60655023 from synthetic construct
gi|60823250 from synthetic construct
gi|78070476 from Homo sapiens
gi|78070601 from Homo sapiens
gi|119571489 from Homo sapiens
gi|119571490 from Homo sapiens
gi|127795529 from Homo sapiens
gi|127799186 from Homo sapiens

Fixed modifications: Carbamidomethyl (C)
Variable modifications: Oxidation (M)
Cleavage by Trypsin: cuts C-term side of KR unless next residue is P
Sequence Coverage: **46%**

Matched peptides shown in **Bold Red**

1 MPHSYPALSA EQKKELSDIA LRIVAPGKGI LAADESVGSM AKR**LSQIGVE**
51 **NTEENRR**LYR **QVLFSADDR**V KK**CIGGVIFF HETLYQKDDN GVPFVR**TIQD
101 KGIVVGIK**VD KGVVPLAGTD GETTTQGLDG LSER**CAQYKK DGADFAKWRC
151 VLKISER**TPS ALAILENANV LAR**YASICQQ NGIVPIVEPE ILPDGDHDLK
201 RCQYVTEKVL AAVYKALSDH HVYLEGTLLK PNMVTPGHAC PIK**YTPEEIA**
251 **MATVTALRRT VPPAVPGVTF LSGGQSEEEA SFNLNAINR**C PLPRPWALTF
301 SYGRALQASA LNAWRGQR**DN AGAATEEFIK R**AEVNGLAAQ GK**YEGSGEDG**
351 **GAAAQSLYIA NHAY**

Residue Number Increasing Mass Decreasing Mass

Start - End	Observed	Mr(expt)	Mr(calc)	Delta	Miss	Sequence
44 - 57	1644.8494	1643.8421	1643.8226	0.0195	1	LSQIGVENTEENRR (No match)
61 - 69	1050.5344	1049.5271	1049.5141	0.0130	0	QVLFSADDR (No match)
73 - 96	2811.4229	2810.4156	2810.3849	0.0307	1	CIGGVIFFHETLYQKDDNGVPFVR (No match)
88 - 96	1018.5118	1017.5045	1017.4879	0.0166	0	DDNGVPFVR (No match)
109 - 134	2615.3684	2614.3611	2614.3085	0.0526	1	VDKGVVPLAGTDGETTTQGLDGLSER (No match)
112 - 134	2273.1614	2272.1541	2272.1182	0.0359	0	GVVPLAGTDGETTTQGLDGLSER (Ions score 113)
112 - 134	2273.1614	2272.1541	2272.1182	0.0359	0	GVVPLAGTDGETTTQGLDGLSER (No match)
158 - 173	1652.9529	1651.9456	1651.9256	0.0200	0	TPSALAILENANVLAR (Ions score 78)
158 - 173	1652.9529	1651.9456	1651.9256	0.0200	0	TPSALAILENANVLAR (No match)
244 - 258	1681.8672	1680.8599	1680.8392	0.0207	0	YTPEEIAMATVTALR Oxidation (M) (No match)
244 - 259	1837.9629	1836.9556	1836.9403	0.0153	1	YTPEEIAMATVTALRR Oxidation (M) (No match)
260 - 289	3101.5901	3100.5828	3100.5464	0.0364	0	TVPPAVPGVTFLSGGQSEEEASFNLNAINR (Ions score 107)
260 - 289	3101.5901	3100.5828	3100.5464	0.0364	0	TVPPAVPGVTFLSGGQSEEEASFNLNAINR (No match)
319 - 331	1421.7218	1420.7145	1420.6946	0.0199	1	DNAGAATEEFIKR (No match)
343 - 364	2244.0278	2243.0205	2242.9766	0.0439	0	YEGSGEDGGAAAQSLYIANHAY (No match)

Spot 566

Protein View

Match to: **gi|14530107** Score: **96** Expect: **5.3e-005**
OVARIAN/Breast septin delta [Homo sapiens]

Nominal mass (M_r): **38690**; Calculated pI value: **7.13**
NCBI BLAST search of gi|14530107 against nr
Unformatted sequence string for pasting into other applications

Taxonomy: Homo sapiens
Links to retrieve other entries containing this sequence from NCBI Entrez:
gi|119609869 from Homo sapiens

Fixed modifications: Carbamidomethyl (C)
Variable modifications: Oxidation (M)
Cleavage by Trypsin: cuts C-term side of KR unless next residue is P
Sequence Coverage: **12%**

Matched peptides shown in **Bold Red**

```
  1 MADTPRDAGL KQAPASRNEK APVDFGYVGI DSILEQMRRK AMKQGFEFNI
 51 MVVGQSGLGK STLINTLFKS KISRKSVQPT SEERIPKTIE IKSITHDIEE
101 KGVRMKLTVI DTPGFGDHIN NENCWQPIMK FINDQYEKYL QEEVNINRKK
151 RIPDTRVHCC LYFIPATGHS LRPLDIEFMK RLSKVVNIVP VIAKADTLTL
201 EERVHFKQRI TADLLSNGID VYPQKEFDED SEDRLVNEKF REMIPFAVVG
251 SDHEYQVNGK RILGRKTKWG TIEVENTTHC EFAYLRDLLI RTHMQNIKDI
301 TSSIHFEAYR VKRLNEGSSA MANGVEEKEP EAPEM
```

Residue Number Increasing Mass Decreasing Mass

Start - End	Observed	Mr(expt)	Mr(calc)	Delta	Miss	Sequence
21 - 38	2025.9779	2024.9706	2024.9876	-0.0170	0	APVDFGYVGIDSILEQMR Oxidation (M) (No match)
139 - 148	1277.6379	1276.6306	1276.6411	-0.0105	0	YLQEEVNINR (No match)
139 - 148	1277.6379	1276.6306	1276.6411	-0.0105	0	YLQEEVNINR (Ions score 24)
139 - 149	1405.7347	1404.7274	1404.7361	-0.0086	1	YLQEEVNINRK (No match)
299 - 310	1438.6781	1437.6708	1437.6888	-0.0180	0	DITSSIHFEAYR (No match)
299 - 310	1438.6781	1437.6708	1437.6888	-0.0180	0	DITSSIHFEAYR (Ions score 28)

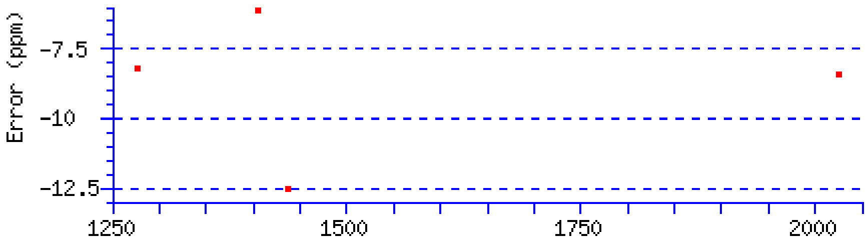

RMS error 9 ppm

Spot 567

Protein View

Match to: **gi|5031601** Score: **186** Expect: **4.8e-014**
actin related protein 2/3 complex subunit 1B [Homo sapiens]

Nominal mass (M_r): **41722**; Calculated pI value: **8.69**
NCBI BLAST search of gi|5031601 against nr
Unformatted sequence string for pasting into other applications

Taxonomy: Homo sapiens
Links to retrieve other entries containing this sequence from NCBI Entrez:
gi|3121763 from Homo sapiens
gi|2282034 from Homo sapiens
gi|12803475 from Homo sapiens
gi|14043135 from Homo sapiens
gi|31416821 from Homo sapiens
gi|51094630 from Homo sapiens
gi|119597083 from Homo sapiens
gi|119597084 from Homo sapiens
gi|119597085 from Homo sapiens
gi|119597086 from Homo sapiens
gi|123982708 from synthetic construct
gi|123997375 from synthetic construct

Fixed modifications: Carbamidomethyl (C)
Variable modifications: Oxidation (M)
Cleavage by Trypsin: cuts C-term side of KR unless next residue is P
Sequence Coverage: **15%**

Matched peptides shown in **Bold Red**

1 MAYHSFLVEP ISCHAWNKDR **TQIAICPNNH** **EVHIYEK**SGA KWTKVHELK**E**
51 **HNGQVTGIDW** **APESNR**IVTC GTDRNAYVWT LKGR**TWKPTL** **VILR**INRAAR
101 CVRWAPNENK FAVGSGSRVI SICYFEQEND WWVCKHIKKP IRSTVLSLDW
151 HPNNVLLAAG SCDFKCRIFS AYIK**EVEERP** **APTPWGSK**MP FGELMFESSS
201 SCGWVHGVCF SASGSRVAWV SHDSTVCLAD ADKKMAVATL ASETLPLLAL
251 TFITDNSLVA AGHDCFPVLF TYDAAAGMLS FGGRLDVPKQ SSQRGLTARE
301 RFQNLDKKAS SEGGTAAGAG LDSLHKNSVS QISVLSGGKA KCSQFCTTGM
351 DGGMSIWDVK SLESALKDLK IK

Residue Number Increasing Mass Decreasing Mass

Start - End	Observed	Mr(expt)	Mr(calc)	Delta	Miss	Sequence
21 - 37	2066.0020	2064.9947	2065.0051	-0.0103	0	TQIAICPNNHEVHIYEK (No match)
21 - 37	2066.0020	2064.9947	2065.0051	-0.0103	0	TQIAICPNNHEVHIYEK (Ions score 58)
50 - 66	1909.8689	1908.8616	1908.8714	-0.0098	0	EHNGQVTGIDWAPESNR (No match)
50 - 66	1909.8689	1908.8616	1908.8714	-0.0098	0	EHNGQVTGIDWAPESNR (Ions score 49)
85 - 94	1226.7677	1225.7604	1225.7546	0.0058	0	TWKPTLVILR (No match)
175 - 188	1582.7841	1581.7768	1581.7787	-0.0018	0	EVEERPAPTPWGSK (No match)
175 - 188	1582.7841	1581.7768	1581.7787	-0.0018	0	EVEERPAPTPWGSK (Ions score 40)

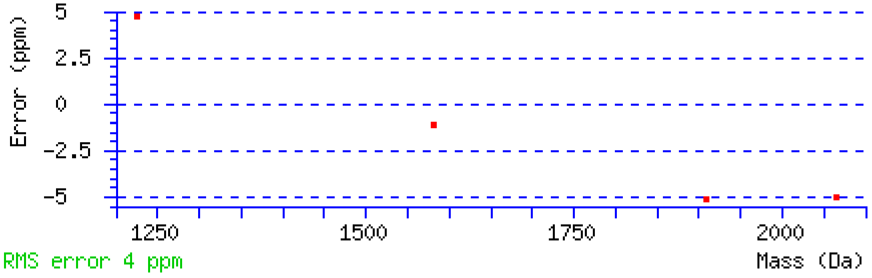

RMS error 4 ppm

Spot 568

Protein View

Match to: **gi|55597035** Score: **229** Expect: **2.4e-018**
PREDICTED: gelsolin-like capping protein isoform 9 [Pan troglodytes]

Nominal mass (M_r): **38779**; Calculated pI value: **5.88**
NCBI BLAST search of gi|55597035 against nr
Unformatted sequence string for pasting into other applications

Taxonomy: Pan troglodytes
Links to retrieve other entries containing this sequence from NCBI Entrez:
gi|114578492 from Pan troglodytes
gi|114578494 from Pan troglodytes
gi|114578496 from Pan troglodytes
gi|114578498 from Pan troglodytes
gi|114578500 from Pan troglodytes
gi|729022 from Homo sapiens
gi|187456 from Homo sapiens
gi|12653873 from Homo sapiens
gi|15778939 from Homo sapiens
gi|60655417 from synthetic construct
gi|119619923 from Homo sapiens
gi|119619924 from Homo sapiens
gi|119619925 from Homo sapiens
gi|119619926 from Homo sapiens
gi|123982696 from synthetic construct
gi|123997363 from synthetic construct

Fixed modifications: Carbamidomethyl (C)
Variable modifications: Oxidation (M)
Cleavage by Trypsin: cuts C-term side of KR unless next residue is P
Sequence Coverage: **23%**

Matched peptides shown in **Bold Red**

1 MYTAIPQSGS PFPGSVQDPG LHVWRVEKLK PVPVAQENQG VFFSGDSYLV
51 LHNGPEEVSH LHLWIGQQSS R**DEQGACAVL AVHLNTLLGE RPVQHREVQG**
101 **NESDLFMSYF PR**GLK**YQEGG VESAFHK**TST GAPAAIKKLY QVKGKKNIRA
151 TERALNWDSF NTGDCFILDL GQNIFAWCGG KSNILERNKA RDLALAIRDS
201 ERQGKAQVEI VTDGEEPAEM IQVLGPKPAL KEGNPEEDLT ADKANAQAAA
251 LYKVSDATGQ MNLTKVADSS PFALELLISD DCFVLDNGLC GKIYIWKGRK
301 ANEKER**QAAL QVAEGFISRM QYAPNTQVEI LPQGR**ESPIF KQFFKDWK

Residue Number Increasing Mass Decreasing Mass

Start - End	Observed	Mr(expt)	Mr(calc)	Delta	Miss	Sequence
72 - 96	2783.3628	2782.3555	2782.4296	-0.0741	0	DEQGACAVLAVHLNTLLGERPVQHR (No match)
72 - 96	2783.3628	2782.3555	2782.4296	-0.0741	0	DEQGACAVLAVHLNTLLGERPVQHR (Ions score 9)
97 - 112	1934.8093	1933.8020	1933.8515	-0.0495	0	EVQGNESDLFMSYFPR Oxidation (M) (Ions score 28)
97 - 112	1934.8093	1933.8020	1933.8515	-0.0495	0	EVQGNESDLFMSYFPR Oxidation (M) (No match)
116 - 127	1351.5975	1350.5902	1350.6203	-0.0301	0	YQEGGVESAFHK (No match)
307 - 319	1389.7167	1388.7094	1388.7411	-0.0317	0	QAALQVAEGFISR (No match)
307 - 319	1389.7167	1388.7094	1388.7411	-0.0317	0	QAALQVAEGFISR (Ions score 76)
320 - 335	1844.8871	1843.8798	1843.9250	-0.0452	0	MQYAPNTQVEILPQGR (No match)
320 - 335	1860.8774	1859.8701	1859.9199	-0.0498	0	MQYAPNTQVEILPQGR Oxidation (M) (Ions score 54)
320 - 335	1860.8774	1859.8701	1859.9199	-0.0498	0	MQYAPNTQVEILPQGR Oxidation (M) (No match)

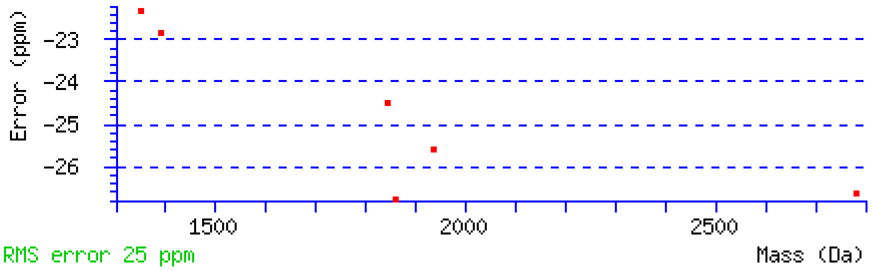

Spot 569

Protein View

Match to: **gi|20986531** Score: **351** Expect: **1.5e-030**
mitogen-activated protein kinase 1 [Homo sapiens]

Nominal mass (M_r): **41762**; Calculated pI value: **6.50**
NCBI BLAST search of gi|20986531 against nr
Unformatted sequence string for pasting into other applications

Taxonomy: Homo sapiens
Links to retrieve other entries containing this sequence from NCBI Entrez:
gi|66932916 from Homo sapiens
gi|160837810 (no taxonomy information for this entry)
gi|109093418 from Macaca mulatta
gi|114685304 from Pan troglodytes
gi|119554 from Homo sapiens
gi|182191 from Homo sapiens
gi|17389606 from Homo sapiens
gi|94717602 from Homo sapiens
gi|119579883 from Homo sapiens
gi|119579884 from Homo sapiens
gi|119579885 from Homo sapiens
gi|119579886 from Homo sapiens
gi|157928978 (no taxonomy information for this entry)

Fixed modifications: Carbamidomethyl (C)
Variable modifications: Oxidation (M)
Cleavage by Trypsin: cuts C-term side of KR unless next residue is P
Sequence Coverage: **28%**

Matched peptides shown in **Bold Red**

1 MAAAAAAGAG PEMVR**GQVFD VGPR**YTNLSY IGEGAYGMVC SAYDNVNKVR
51 VAIKK**ISPFE HQTYCQR**TLR EIKILLR**FRH ENIIGINDII R**APTIEQMKD
101 VYIVQDLMET DLYKLLK**TQH LSNDHICYFL YQILR**GLK**YI HSANVLHR**DL
151 KPSNLLLNTT CDLK**ICDFGL ARVADPDHDH TGFLTEYVAT R**WYRAPEIML
201 NSKGYTKSID IWSVGCILAE MLSNRPIFPG KHYLDQLNHI LGILGSPSQE
251 DLNCIINLKA RNYLLSLPHK NKVPWNRLFP NADSKALDLL DKMLTFNPHK
301 RIEVEQALAH PYLEQYYDPS DEPIAEAPFK FDMELDDLPK EK**LKELIFEE**
351 **TAR**FQPGYRS

Residue Number Increasing Mass Decreasing Mass

Start - End	Observed	Mr(expt)	Mr(calc)	Delta	Miss	Sequence
16 - 24	974.5137	973.5064	973.4981	0.0084	0	GQVFDVGPR (No match)
16 - 24	974.5137	973.5064	973.4981	0.0084	0	GQVFDVGPR (Ions score 26)
56 - 67	1565.7261	1564.7188	1564.7092	0.0096	0	ISPFEHQTYCQR (No match)
56 - 67	1565.7261	1564.7188	1564.7092	0.0096	0	ISPFEHQTYCQR (Ions score 46)
78 - 91	1709.9436	1708.9363	1708.9372	-0.0009	1	FRHENIIGINDIIR (No match)
118 - 135	2321.1372	2320.1299	2320.1422	-0.0123	0	TQHLSNDHICYFLYQILR (No match)
139 - 148	1209.6591	1208.6518	1208.6414	0.0104	0	YIHSANVLHR (Ions score 76)
139 - 148	1209.6591	1208.6518	1208.6414	0.0104	0	YIHSANVLHR (No match)
165 - 172	951.4816	950.4743	950.4643	0.0100	0	ICDFGLAR (No match)
173 - 191	2144.0039	2142.9966	2142.9970	-0.0004	0	VADPDHDHTGFLTEYVATR (No match)
173 - 191	2144.0039	2142.9966	2142.9970	-0.0004	0	VADPDHDHTGFLTEYVATR (Ions score 58)
343 - 353	1348.7566	1347.7493	1347.7397	0.0096	1	LKELIFEETAR (Ions score 27)
343 - 353	1348.7566	1347.7493	1347.7397	0.0096	1	LKELIFEETAR (No match)

Spot 570

Protein View

Match to: **gi|119612724** Score: **121** Expect: **1.5e-007**
actin, alpha, cardiac muscle, isoform CRA_c [Homo sapiens]

Nominal mass (M_r): **30498**; Calculated pI value: **4.88**
NCBI BLAST search of gi|119612724 against nr
Unformatted sequence string for pasting into other applications

Taxonomy: Homo sapiens

Fixed modifications: Carbamidomethyl (C)
Variable modifications: Oxidation (M)
Cleavage by Trypsin: cuts C-term side of KR unless next residue is P
Sequence Coverage: **9%**

Matched peptides shown in **Bold Red**

```
  1 MCDDEETTAL VCDNGSGLVK AGFAGDDAPR AVFPSIVGRP RHQGVMVGMG
 51 QKDSYVGDEA QSKRGILTLK YPIEHGIITN WDDMEKIWHH TFYNELRVAP
101 EEHPTLLTEA PLNPKANREK MTQIMFETFN VPAMYVAIQA VLSLYASGRT
151 TGIVLDSGDG VTHNVPIYEG YALPHAIMRL DLAGRDLTDY LMKILTERGY
201 SFVTTAEREI VRDIKEKLCY VALDFENEMA TAASSSSLEK SYELPDGQVI
251 TIGNERFRCP ETLFQPSFIG EL
```

Residue Number Increasing Mass Decreasing Mass

Start - End	Observed	Mr(expt)	Mr(calc)	Delta	Miss	Sequence	
31 - 41	1198.7177	1197.7104	1197.6982	0.0123	0	AVFPSIVGRPR	(Ions score 35)
31 - 41	1198.7177	1197.7104	1197.6982	0.0123	0	AVFPSIVGRPR	(No match)
241 - 256	1790.8997	1789.8924	1789.8846	0.0079	0	SYELPDGQVITIGNER	(No match)
241 - 256	1790.8997	1789.8924	1789.8846	0.0079	0	SYELPDGQVITIGNER	(Ions score 67)

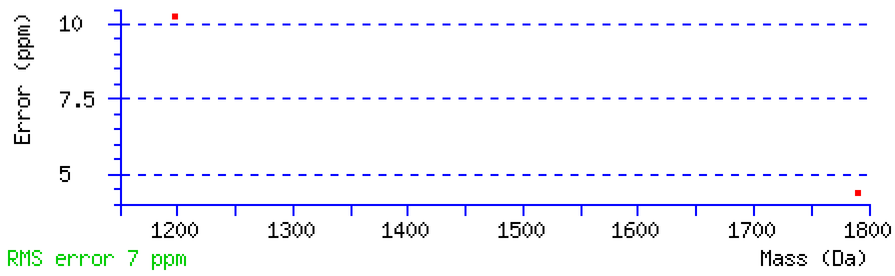

Error (ppm)
10
7.5
5
1200
1300
1400
1500
1600
1700
1800
RMS error 7 ppm
Mass (Da)

Spot 571

Protein View

Match to: **gi|5031571** Score: **609** Expect: **2.4e-056**
actin-related protein 2 isoform b [Homo sapiens]

Nominal mass (M_r): **45017**; Calculated pI value: **6.30**
NCBI BLAST search of gi|5031571 against nr
Unformatted sequence string for pasting into other applications

Taxonomy: Homo sapiens
Links to retrieve other entries containing this sequence from NCBI Entrez:
gi|22122825 from Mus musculus
gi|156121073 (no taxonomy information for this entry)
gi|57093381 from Canis lupus familiaris
gi|109103168 from Macaca mulatta
gi|47117648 from Homo sapiens
gi|47117649 from Mus musculus
gi|17943200 from Bos taurus
gi|56966173 from Bos taurus
gi|56966193 from Bos taurus
gi|149243010 from Bos taurus
gi|149243032 from Bos taurus
gi|149243039 from Bos taurus
gi|149243046 from Bos taurus
gi|149243053 from Bos taurus
gi|149243060 from Bos taurus
gi|2282030 from Homo sapiens
gi|20380733 from Mus musculus
gi|26328619 from Mus musculus
gi|26353172 from Mus musculus
gi|56205119 from Mus musculus
gi|74190292 from Mus musculus
gi|119620314 from Homo sapiens
gi|119620316 from Homo sapiens
gi|119620318 from Homo sapiens
gi|119620319 from Homo sapiens
gi|148675879 from Mus musculus
gi|154425680 from Bos taurus

Fixed modifications: Carbamidomethyl (C)
Variable modifications: Oxidation (M)
Cleavage by Trypsin: cuts C-term side of KR unless next residue is P
Sequence Coverage: **55%**

Matched peptides shown in **Bold Red**

```
  1 MDSQGRKVVV CDNGTGFVKC GYAGSNFPEH IFPALVGRPI IRSTTKVGNI
 51 EIKDLMVGDE ASELRSMLEV NYPMENGIVR NWDDMKHLWD YTFGPEKLNI
101 DTRNCKILLT EPPMNPTKNR EKIVEVMFET YQFSGVYVAI QAVLTLYAQG
151 LLTGVVVDSG DGVTHICPVY EGFSLPHLTR RLDIAGRDIT RYLIKLLLLR
201 GYAFNHSADF ETVRMIKEKL CYVGYNIEQE QKLALETTVL VESYTLPDGR
251 IIKVGGERFE APEALFQPHL INVEGVGVAE LLFNTIQAAD IDTRSEFYKH
301 IVLSGGSTMY PGLPSRLERE LKQLYLERVL KGDVEKLSKF KIRIEDPPRR
351 KHMVFLGGAV LADIMKDKDN FWMTRQEYQE KGVRVLEKLG VTVR
```

Residue Number Increasing Mass Decreasing Mass

Start - End	Observed	Mr(expt)	Mr(calc)	Delta	Miss	Sequence
7 - 19	1422.7109	1421.7036	1421.7336	-0.0300	1	**KVVVCDNGTGFVK** (No match)
20 - 42	2571.2693	2570.2620	2570.3215	-0.0595	0	**CGYAGSNFPEHIFPALVGRPIIR** (Ions score 71)
20 - 42	2571.2693	2570.2620	2570.3215	-0.0595	0	**CGYAGSNFPEHIFPALVGRPIIR** (No match)
47 - 65	2088.0320	2087.0247	2087.0568	-0.0321	1	**VGNIEIKDLMVGDEASELR** (No match)
54 - 65	1334.6105	1333.6032	1333.6183	-0.0151	0	**DLMVGDEASELR** (No match)
66 - 80	1751.8228	1750.8155	1750.8382	-0.0226	0	**SMLEVNYPMENGIVR** (Ions score 44)
66 - 80	1751.8228	1750.8155	1750.8382	-0.0226	0	**SMLEVNYPMENGIVR** (No match)
66 - 80	1767.8163	1766.8090	1766.8331	-0.0240	0	**SMLEVNYPMENGIVR** Oxidation (M) (No match)
66 - 80	1783.8196	1782.8123	1782.8280	-0.0156	0	**SMLEVNYPMENGIVR** 2 Oxidation (M) (No match)
87 - 97	1392.6443	1391.6370	1391.6510	-0.0139	0	**HLWDYTFGPEK** (No match)
87 - 103	2105.0122	2104.0049	2104.0377	-0.0328	1	**HLWDYTFGPEKLNIDTR** (No match)
104 - 118	1771.8879	1770.8806	1770.9008	-0.0202	1	**NCKILLTEPPMNPTK** Oxidation (M) (No match)
107 - 118	1353.7250	1352.7177	1352.7374	-0.0196	0	**ILLTEPPMNPTK** (No match)
107 - 118	1369.7135	1368.7062	1368.7323	-0.0260	0	**ILLTEPPMNPTK** Oxidation (M) (No match)
181 - 187	800.4585	799.4512	799.4664	-0.0152	1	**RLDIAGR** (No match)
201 - 214	1613.7120	1612.7047	1612.7269	-0.0222	0	**GYAFNHSADFETVR** (No match)
201 - 214	1613.7120	1612.7047	1612.7269	-0.0222	0	**GYAFNHSADFETVR** (Ions score 88)
220 - 232	1643.7505	1642.7432	1642.7660	-0.0228	0	**LCYVGYNIEQEQK** (No match)
233 - 250	1977.0204	1976.0131	1976.0466	-0.0334	0	**LALETTVLVESYTLPDGR** (Ions score 114)
233 - 250	1977.0204	1976.0131	1976.0466	-0.0334	0	**LALETTVLVESYTLPDGR** (No match)
295 - 316	2426.1560	2425.1487	2425.2099	-0.0612	1	**SEFYKHIVLSGGSTMYPGLPSR** (No match)
300 - 316	1771.8879	1770.8806	1770.9086	-0.0280	0	**HIVLSGGSTMYPGLPSR** (Ions score 36)
300 - 316	1787.8855	1786.8782	1786.9035	-0.0253	0	**HIVLSGGSTMYPGLPSR** Oxidation (M) (No match)
320 - 328	1191.6627	1190.6554	1190.6659	-0.0104	1	**ELKQLYLER** (No match)
323 - 328	821.4385	820.4312	820.4443	-0.0130	0	**QLYLER** (No match)
342 - 349	995.5496	994.5423	994.5559	-0.0136	1	**IRIEDPPR** (No match)
352 - 366	1601.8348	1600.8275	1600.8469	-0.0194	0	**HMVFLGGAVLADIMK** (No match)
367 - 375	1212.5305	1211.5232	1211.5393	-0.0161	1	**DKDNFWMTR** (No match)
367 - 375	1228.5272	1227.5199	1227.5342	-0.0143	1	**DKDNFWMTR** Oxidation (M) (No match)
369 - 375	969.4136	968.4063	968.4174	-0.0111	0	**DNFWMTR** (No match)

Spot 572

Protein View

Match to: **gi|55597035** Score: **368** Expect: **3e-032**
PREDICTED: gelsolin-like capping protein isoform 9 [Pan troglodytes]

Nominal mass (M_r): **38779**; Calculated pI value: **5.88**
NCBI BLAST search of gi|55597035 against nr
Unformatted sequence string for pasting into other applications

Taxonomy: Pan troglodytes
Links to retrieve other entries containing this sequence from NCBI Entrez:
gi|114578492 from Pan troglodytes
gi|114578494 from Pan troglodytes
gi|114578496 from Pan troglodytes
gi|114578498 from Pan troglodytes
gi|114578500 from Pan troglodytes
gi|729022 from Homo sapiens
gi|187456 from Homo sapiens
gi|12653873 from Homo sapiens
gi|15778939 from Homo sapiens
gi|60655417 from synthetic construct
gi|119619923 from Homo sapiens
gi|119619924 from Homo sapiens
gi|119619925 from Homo sapiens
gi|119619926 from Homo sapiens
gi|123982696 from synthetic construct
gi|123997363 from synthetic construct

Fixed modifications: Carbamidomethyl (C)
Variable modifications: Oxidation (M)
Cleavage by Trypsin: cuts C-term side of KR unless next residue is P
Sequence Coverage: **40%**

Matched peptides shown in **Bold Red**

1 MYTAIPQSGS PFPGSVQDPG LHVWRVEKLK PVPVAQENQG VFFSGDSYLV
51 LHNGPEEVSH LHLWIGQQSS R**DEQGACAVL AVHLNTLLGE RPVQHREVQG**
101 **NESDLFMSYF PRGLKYQEGG VESAFHK**TST GAPAAIKKLY QVKGKKNIRA
151 TERALNWDSF NTGDCFILDL GQNIFAWCGG KSNILERNKA RDLALAIRDS
201 ERQGK**AQVEI VTDGEEPAEM IQVLGPKPAL KEGNPEEDLT ADKANAQAAA**
251 **LYK**VSDATGQ MNLTKVADSS PFALELLISD DCFVLDNGLC GKIYIWKGRK
301 ANEKER**QAAL QVAEGFISRM QYAPNTQVEI LPQGR**ESPIF K**QFFKDWK**

Residue Number Increasing Mass Decreasing Mass

Start - End	Observed	Mr(expt)	Mr(calc)	Delta	Miss	Sequence
72 - 96	2783.2573	2782.2500	2782.4296	-0.1796	0	DEQGACAVLAVHLNTLLGERPVQHR (No match)
97 - 112	1934.7535	1933.7462	1933.8515	-0.1053	0	EVQGNESDLFMSYFPR Oxidation (M) (No match)
97 - 112	1934.7535	1933.7462	1933.8515	-0.1053	0	EVQGNESDLFMSYFPR Oxidation (M) (Ions score 30)
113 - 127	1649.7402	1648.7329	1648.8208	-0.0879	1	GLKYQEGGVESAFHK (No match)
116 - 127	1351.5581	1350.5508	1350.6203	-0.0695	0	YQEGGVESAFHK (Ions score 62)
116 - 127	1351.5581	1350.5508	1350.6203	-0.0695	0	YQEGGVESAFHK (No match)
206 - 231	2778.2910	2777.2837	2777.4520	-0.1683	0	AQVEIVTDGEEPAEMIQVLGPKPALK Oxidation (M) (No match)
232 - 253	2318.9727	2317.9654	2318.1025	-0.1371	1	EGNPEEDLTADKANAQAAALYK (No match)
307 - 319	1389.6794	1388.6721	1388.7411	-0.0690	0	QAALQVAEGFISR (Ions score 86)
307 - 319	1389.6794	1388.6721	1388.7411	-0.0690	0	QAALQVAEGFISR (No match)
320 - 335	1860.8262	1859.8189	1859.9199	-0.1010	0	MQYAPNTQVEILPQGR Oxidation (M) (Ions score 88)
320 - 335	1860.8262	1859.8189	1859.9199	-0.1010	0	MQYAPNTQVEILPQGR Oxidation (M) (No match)
342 - 348	998.5316	997.5243	997.5021	0.0222	1	QFFKDWK (No match)

Spot 573

Protein View

Match to: **gi|15277503** Score: **403** Expect: **9.6e-036**
ACTB protein [Homo sapiens]

Nominal mass (M_r): **40536;** Calculated pI value: **5.55**
NCBI BLAST search of gi|15277503 against nr
Unformatted sequence string for pasting into other applications

Taxonomy: Homo sapiens

Fixed modifications: Carbamidomethyl (C)
Variable modifications: Oxidation (M)
Cleavage by Trypsin: cuts C-term side of KR unless next residue is P
Sequence Coverage: **27%**

Matched peptides shown in **Bold Red**

```
  1 MCKAGFAGDD APRAVFPSIV GRPRHQGVMV GMGQKDSYVG DEAQSKRGIL
 51 TLKYPIEHGI VTNWDDMEKI WHHTFYNELR VAPEEHPVLL TEAPLNPKAN
101 LEKMTQIMFE TFNTPAMYVA IQAVLSLYAS GRTTGIVMDS GDGVTHTVPI
151 YEGYALPHAI LRLDLAGRDL TDYLMKILTE RGYSFTTTAE REIVRDIKEK
201 LCYVALDFEQ EMATAASSSS LEKSYELPDG QVITIGNERF RCPEALFQPS
251 FLGMESCGIH ETTFNSIMKC DVDIRKDLYA NTVLSGGTTM YPGIADRMQK
301 EITALAPSTM KIKIIAPPER KYSVWIGGSI LASLSTFQQM WISKQEYDES
351 GPSIVHRKCF
```

Residue Number Increasing Mass Decreasing Mass

Start - End	Observed	Mr(expt)	Mr(calc)	Delta	Miss	Sequence
14 - 24	1198.6904	1197.6831	1197.6982	-0.0150	0	AVFPSIVGRPR (No match)
14 - 24	1198.6904	1197.6831	1197.6982	-0.0150	0	AVFPSIVGRPR (Ions score 37)
70 - 80	1515.7329	1514.7256	1514.7418	-0.0162	0	IWHHTFYNELR (No match)
81 - 98	1954.0431	1953.0358	1953.0571	-0.0212	0	VAPEEHPVLLTEAPLNPK (Ions score 110)
81 - 98	1954.0431	1953.0358	1953.0571	-0.0212	0	VAPEEHPVLLTEAPLNPK (No match)
182 - 191	1132.5166	1131.5093	1131.5196	-0.0103	0	GYSFTTTAER (No match)
182 - 191	1132.5166	1131.5093	1131.5196	-0.0103	0	GYSFTTTAER (No match)
224 - 239	1790.8635	1789.8562	1789.8846	-0.0283	0	SYELPDGQVITIGNER (No match)
224 - 239	1790.8635	1789.8562	1789.8846	-0.0283	0	SYELPDGQVITIGNER (Ions score 93)

277 - 297	2231.0479	2230.0406	2230.0575	-0.0169	0	DLYANTVLSGGTTMYPGIADR	Oxidation (M) (No match)
345 - 357	1516.6953	1515.6880	1515.6953	-0.0073	0	QEYDESGPSIVHR	(Ions score 79)
345 - 357	1516.6953	1515.6880	1515.6953	-0.0073	0	QEYDESGPSIVHR	(No match)

Spot 574

Protein View

Match to: **gi|6005846** Score: **614** Expect: **7.7e-057**
twinfilin-like protein [Homo sapiens]

Nominal mass (M_r): **39751**; Calculated pI value: **6.37**
NCBI BLAST search of gi|6005846 against nr
Unformatted sequence string for pasting into other applications

Taxonomy: Homo sapiens
Links to retrieve other entries containing this sequence from NCBI Entrez:
gi|94730596 from Homo sapiens
gi|33337753 from Homo sapiens
gi|4468253 from Homo sapiens
gi|6807661 from Homo sapiens
gi|12653121 from Homo sapiens
gi|13111977 from Homo sapiens
gi|16741225 from Homo sapiens
gi|49065466 from Homo sapiens
gi|117646730 from synthetic construct
gi|119585598 from Homo sapiens
gi|123981702 from synthetic construct
gi|123996521 from synthetic construct

Fixed modifications: Carbamidomethyl (C)
Variable modifications: Oxidation (M)
Cleavage by Trypsin: cuts C-term side of KR unless next residue is P
Sequence Coverage: **64%**

Matched peptides shown in **Bold Red**

1 MAHQTGIHAT EELKEFFAKA RAGSVRLIK**V VIEDEQLVLG ASQEPVGRWD**
51 QDYDRAVLPL LDAQQPCYLL YRLDSQNAQG FEWLFLAWSP DNSPVRLK**ML**
101 YAATRATVKK **EFGGGHIKDE LFGTVK**DDLS FAGYQK**HLSS CAAPAPLTSA**
151 ERELQQIRIN EVKTEISVES K**HQTLQGLAF PLQPEAQR**AL QQLKQKMVNY
201 IQMKLDLER**E TIELVHTEPT DVAQLPSR**VP RDAAR**YHFFL YKHTHEGDPL**
251 ESVVFIYSMP GYKCSIKERM LYSSCKSR**LL DSVEQDFHLE IAKKIEIGDG**
301 AELTAEFLYD EVHPKQHAFK QAFAKPKGPG GKRGHKRLIR GPGENGDDS

Residue Number Increasing Mass Decreasing Mass

Start - End	Observed	Mr(expt)	Mr(calc)	Delta	Miss	Sequence
30 - 48	2038.0492	2037.0419	2037.0741	-0.0322	0	VVIEDEQLVLGASQEPVGR (No match)
49 - 55	997.3927	996.3854	996.3937	-0.0083	0	WDQDYDR (No match)
56 - 72	2033.0569	2032.0496	2032.0815	-0.0319	0	AVLPLLDAQQPCYLLYR (No match)
56 - 72	2033.0569	2032.0496	2032.0815	-0.0319	0	AVLPLLDAQQPCYLLYR (Ions score 64)
73 - 96	2777.2822	2776.2749	2776.3244	-0.0495	0	LDSQNAQGFEWLFLAWSPDNSPVR (No match)
99 - 105	825.4455	824.4382	824.4214	0.0168	0	MLYAATR (No match)
99 - 105	841.4141	840.4068	840.4163	-0.0095	0	MLYAATR Oxidation (M) (No match)
111 - 126	1733.8623	1732.8550	1732.8783	-0.0233	1	EFGGGHIKDELFGTVK (No match)
137 - 152	1667.7921	1666.7848	1666.8097	-0.0248	0	HLSSCAAPAPLTSAER (No match)
137 - 152	1667.7921	1666.7848	1666.8097	-0.0248	0	HLSSCAAPAPLTSAER (Ions score 112)
153 - 158	786.4426	785.4353	785.4395	-0.0042	0	ELQQIR (No match)
172 - 188	1933.9944	1932.9871	1933.0169	-0.0298	0	HQTLQGLAFPLQPEAQR (Ions score 113)
172 - 188	1933.9944	1932.9871	1933.0169	-0.0298	0	HQTLQGLAFPLQPEAQR (No match)
210 - 228	2135.0669	2134.0596	2134.0906	-0.0309	0	ETIELVHTEPTDVAQLPSR (No match)
210 - 228	2135.0669	2134.0596	2134.0906	-0.0309	0	ETIELVHTEPTDVAQLPSR (Ions score 113)
236 - 242	1017.5085	1016.5012	1016.5119	-0.0107	0	YHFFLYK (No match)
236 - 242	1017.5085	1016.5012	1016.5119	-0.0107	0	YHFFLYK (Ions score 39)
243 - 263	2422.1440	2421.1367	2421.1310	0.0057	0	HTHEGDPLESVVFIYSMPGYK Oxidation (M) (No match)
279 - 293	1756.8871	1755.8798	1755.9043	-0.0244	0	LLDSVEQDFHLEIAK (No match)
294 - 315	2474.2285	2473.2212	2473.2376	-0.0163	1	KIEIGDGAELTAEFLYDEVHPK (No match)
316 - 327	1400.6893	1399.6820	1399.7724	-0.0904	1	QHAFKQAFAKPK (No match)

Spot 575

Protein View

Match to: **gi|46981967** Score: **129** Expect: **2.4e-008**
growth-inhibiting protein 18 [Homo sapiens]

Nominal mass (M_r): **46519**; Calculated pI value: **6.34**
NCBI BLAST search of gi|46981967 against nr
Unformatted sequence string for pasting into other applications

Taxonomy: Homo sapiens

Fixed modifications: Carbamidomethyl (C)
Variable modifications: Oxidation (M)
Cleavage by Trypsin: cuts C-term side of KR unless next residue is P
Sequence Coverage: **20%**

Matched peptides shown in **Bold Red**

```
  1 MAPPSVFAEV PQAQPVLVFK LTADFREDPD PRKVNLGVGA YRTDDCHPWV
 51 LPVVKKVEQK IANDNSLNHE YLPILGLAEF RSCASRLALE DDSPALKEKR
101 VGGVQSLGGT GALRIGADFL ARWYNGTNNK NTPVYVSSPT WENHNAVFSA
151 AGFKDIRSYR YWDAEKRGLD LQGFLNDLEN APEFSIVVLH ACAHNPTGID
201 PTPEQWKQIA SVMKHRFLFP FFDSAYQGFA SGNLERDAWA IRYFVSEGFE
251 FFCAQSFSKN FGLYNERVGN LTVVGKEPES ILQVLSQMEK IVRITWSNPP
301 AQGARIVAST LSNPELFEEW TGNVKTMADR ILTMRSELRA RLEALKTPGT
351 WNHITDQIGM FSFTGLNPKQ VEYLVNEKHI YLLPSGRINV SGLTTKNLDY
401 VATSIHEAVT KIQ
```

Residue Number Increasing Mass Decreasing Mass

Start - End	Observed	Mr(expt)	Mr(calc)	Delta	Miss	Sequence
21 - 32	1431.6121	1430.6048	1430.6790	-0.0741	1	LTADFREDPDPR (No match)
33 - 42	1076.5680	1075.5607	1075.6137	-0.0530	1	KVNLGVGAYR (No match)
34 - 42	948.4865	947.4792	947.5188	-0.0396	0	VNLGVGAYR (No match)
61 - 81	2399.1140	2398.1067	2398.2280	-0.1213	0	IANDNSLNHEYLPILGLAEFR (No match)
100 - 114	1427.7452	1426.7379	1426.8004	-0.0624	1	RVGGVQSLGGTGALR (No match)
101 - 114	1271.6506	1270.6433	1270.6992	-0.0559	0	VGGVQSLGGTGALR (Ions score 7)
101 - 114	1271.6506	1270.6433	1270.6992	-0.0559	0	VGGVQSLGGTGALR (No match)
115 - 122	862.4333	861.4260	861.4708	-0.0448	0	IGADFLAR (No match)

260 - 267	1012.4365	1011.4292	1011.4773	-0.0481	0	NFGLYNER	(Ions score 16)
260 - 267	1012.4365	1011.4292	1011.4773	-0.0481	0	NFGLYNER	(No match)
379 - 387	1055.5513	1054.5440	1054.5923	-0.0483	0	HIYLLPSGR	(Ions score 6)
379 - 387	1055.5513	1054.5440	1054.5923	-0.0483	0	HIYLLPSGR	(No match)

Spot 576

Protein View

Match to: **gi|6005846** Score: **324** Expect: **7.7e-028**
twinfilin-like protein [Homo sapiens]

Nominal mass (M_r): **39751**; Calculated pI value: **6.37**
NCBI BLAST search of gi|6005846 against nr
Unformatted sequence string for pasting into other applications

Taxonomy: Homo sapiens
Links to retrieve other entries containing this sequence from NCBI Entrez:
gi|94730596 from Homo sapiens
gi|33337753 from Homo sapiens
gi|4468253 from Homo sapiens
gi|6807661 from Homo sapiens
gi|12653121 from Homo sapiens
gi|13111977 from Homo sapiens
gi|16741225 from Homo sapiens
gi|49065466 from Homo sapiens
gi|117646730 from synthetic construct
gi|119585598 from Homo sapiens
gi|123981702 from synthetic construct
gi|123996521 from synthetic construct

Fixed modifications: Carbamidomethyl (C)
Variable modifications: Oxidation (M)
Cleavage by Trypsin: cuts C-term side of KR unless next residue is P
Sequence Coverage: **23%**

Matched peptides shown in **Bold Red**

```
  1 MAHQTGIHAT EELKEFFAKA RAGSVRLIKV VIEDEQLVLG ASQEPVGRWD
 51 QDYDRAVLPL LDAQQPCYLL YRLDSQNAQG FEWLFLAWSP DNSPVRLKML
101 YAATRATVKK EFGGGHIKDE LFGTVKDDLS FAGYQKHLSS CAAPAPLTSA
151 ERELQQIRIN EVKTEISVES KHQTLQGLAF PLQPEAQRAL QQLKQKMVNY
201 IQMKLDLERE TIELVHTEPT DVAQLPSRVP RDAARYHFFL YKHTHEGDPL
251 ESVVFIYSMP GYKCSIKERM LYSSCKSRLL DSVEQDFHLE IAKKIEIGDG
301 AELTAEFLYD EVHPKQHAFK QAFAKPKGPG GKRGHKRLIR GPGENGDDS
```

Residue Number Increasing Mass Decreasing Mass

Start - End	Observed	Mr(expt)	Mr(calc)	Delta	Miss	Sequence
30 - 48	2038.1094	2037.1021	2037.0741	0.0280	0	VVIEDEQLVLGASQEPVGR (No match)
49 - 55	997.4272	996.4199	996.3937	0.0262	0	WDQDYDR (No match)
56 - 72	2033.1300	2032.1227	2032.0815	0.0412	0	AVLPLLDAQQPCYLLYR (No match)
56 - 72	2033.1300	2032.1227	2032.0815	0.0412	0	AVLPLLDAQQPCYLLYR (Ions score 49)
172 - 188	1934.0621	1933.0548	1933.0169	0.0379	0	HQTLQGLAFPLQPEAQR (No match)
172 - 188	1934.0621	1933.0548	1933.0169	0.0379	0	HQTLQGLAFPLQPEAQR (Ions score 75)
236 - 242	1017.5339	1016.5266	1016.5119	0.0147	0	YHFFLYK (No match)
279 - 293	1756.9346	1755.9273	1755.9043	0.0231	0	LLDSVEQDFHLEIAK (Ions score 105)
279 - 293	1756.9346	1755.9273	1755.9043	0.0231	0	LLDSVEQDFHLEIAK (No match)

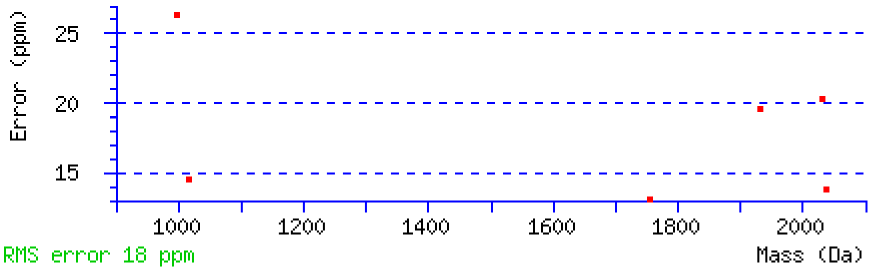

RMS error 18 ppm

Spot 577

Protein View

Match to: **gi|157502193** Score: **172** Expect: **1.2e-012**
proteasome 26S non-ATPase subunit 13 isoform 1 [Homo sapiens]

Nominal mass (M_r): **43203**; Calculated pI value: **5.53**
NCBI BLAST search of gi|157502193 against nr
Unformatted sequence string for pasting into other applications
Links to retrieve other entries containing this sequence from NCBI Entrez:
gi|3618343 from Homo sapiens

Fixed modifications: Carbamidomethyl (C)
Variable modifications: Oxidation (M)
Cleavage by Trypsin: cuts C-term side of KR unless next residue is P
Sequence Coverage: **18%**

Matched peptides shown in **Bold Red**

```
  1 MKDVPGFLQQ SQNSGPGQPA VWHRLEELYT KKLWHQLTLQ VLDFVQDPCF
 51 AQGDGLIKLY ENFISEFEHR VNPLSLVEII LHVVRQMTDP NVALTFLEKT
101 REKVKSSDEA VILCKTAIGA LKLNIGDLQV TKETIEDVEE MLNNLPGVTS
151 VHSRFYDLSS KYYQTIGNHA SYYKDALRFL GCVDIKDLPV SEQQERAFTL
201 GLAGLLGEGV FNFGELLMHP VLESLRNTDR QWLIDTLYAF NSGNVERFQT
251 LKTAWGQQPD LAANEAQLLR KIQLLCLMEM TFTRPANHRQ LTFEEIAKSA
301 KITVNEVELL VMKALSVGLV KGSIDEVDKR VHMTWVQPRV LDLQQIKGMK
351 DRLEFWCTDV KSMEMLVEHQ AHDILT
```

Residue Number Increasing Mass Decreasing Mass

Start - End	Observed	Mr(expt)	Mr(calc)	Delta	Miss	Sequence
59 - 70	1583.7214	1582.7141	1582.7415	-0.0274	0	LYENFISEFEHR (Ions score 61)
59 - 70	1583.7214	1582.7141	1582.7415	-0.0274	0	LYENFISEFEHR (No match)
71 - 85	1701.0138	1700.0065	1700.0348	-0.0283	0	VNPLSLVEIILHVVR (Ions score 13)
71 - 85	1701.0138	1700.0065	1700.0348	-0.0283	0	VNPLSLVEIILHVVR (No match)
162 - 174	1607.7246	1606.7173	1606.7415	-0.0242	0	YYQTIGNHASYYK (Ions score 23)
162 - 174	1607.7246	1606.7173	1606.7415	-0.0242	0	YYQTIGNHASYYK (No match)
187 - 196	1200.5759	1199.5686	1199.5782	-0.0096	0	DLPVSEQQER (No match)
187 - 196	1200.5759	1199.5686	1199.5782	-0.0096	0	DLPVSEQQER (Ions score 20)
253 - 270	1981.9741	1980.9668	1981.0017	-0.0349	0	TAWGQQPDLAANEAQLLR (No match)

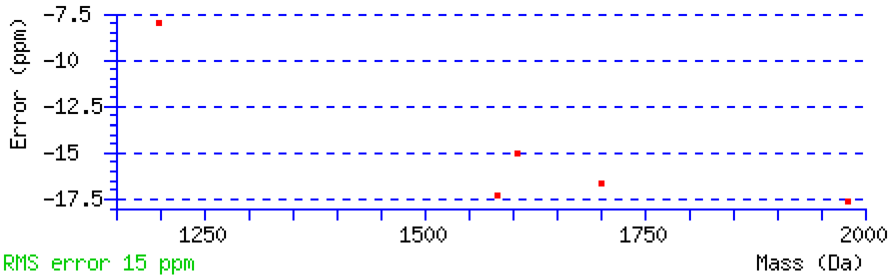

Error (ppm)
-7.5
-10
-12.5
-15
-17.5
1250
1500
1750
2000
RMS error 15 ppm
Mass (Da)

Spot 578

Protein View

Match to: **gi|6005846** Score: **628** Expect: **3e-058**
twinfilin-like protein [Homo sapiens]

Nominal mass (M_r): **39751**; Calculated pI value: **6.37**
NCBI BLAST search of gi|6005846 against nr
Unformatted sequence string for pasting into other applications

Taxonomy: Homo sapiens
Links to retrieve other entries containing this sequence from NCBI Entrez:
gi|94730596 from Homo sapiens
gi|33337753 from Homo sapiens
gi|4468253 from Homo sapiens
gi|6807661 from Homo sapiens
gi|12653121 from Homo sapiens
gi|13111977 from Homo sapiens
gi|16741225 from Homo sapiens
gi|49065466 from Homo sapiens
gi|117646730 from synthetic construct
gi|119585598 from Homo sapiens
gi|123981702 from synthetic construct
gi|123996521 from synthetic construct

Fixed modifications: Carbamidomethyl (C)
Variable modifications: Oxidation (M)
Cleavage by Trypsin: cuts C-term side of KR unless next residue is P
Sequence Coverage: **50%**

Matched peptides shown in **Bold Red**

```
  1 MAHQTGIHAT EELKEFFAKA RAGSVRLIKV VIEDEQLVLG ASQEPVGRWD
 51 QDYDRAVLPL LDAQQPCYLL YRLDSQNAQG FEWLFLAWSP DNSPVRLKML
101 YAATRATVKK EFGGGHIKDE LFGTVKDDLS FAGYQKHLSS CAAPAPLTSA
151 ERELQQIRIN EVKTEISVES KHQTLQGLAF PLQPEAQRAL QQLKQKMVNY
201 IQMKLDLERE TIELVHTEPT DVAQLPSRVP RDAARYHFFL YKHTHEGDPL
251 ESVVFIYSMP GYKCSIKERM LYSSCKSRLL DSVEQDFHLE IAKKIEIGDG
301 AELTAEFLYD EVHPKQHAFK QAFAKPKGPG GKRGHKRLIR GPGENGDDS
```

Residue Number Increasing Mass Decreasing Mass

Start - End	Observed	Mr(expt)	Mr(calc)	Delta	Miss	Sequence
30 - 48	2038.0752	2037.0679	2037.0741	-0.0062	0	VVIEDEQLVLGASQEPVGR (No match)
49 - 55	997.4077	996.4004	996.3937	0.0067	0	WDQDYDR (No match)
56 - 72	2033.0792	2032.0719	2032.0815	-0.0096	0	AVLPLLDAQQPCYLLYR (No match)
56 - 72	2033.0792	2032.0719	2032.0815	-0.0096	0	AVLPLLDAQQPCYLLYR (Ions score 77)
111 - 126	1733.8809	1732.8736	1732.8783	-0.0047	1	EFGGGHIKDELFGTVK (No match)
137 - 152	1667.8115	1666.8042	1666.8097	-0.0054	0	HLSSCAAPAPLTSAER (Ions score 96)
137 - 152	1667.8115	1666.8042	1666.8097	-0.0054	0	HLSSCAAPAPLTSAER (No match)
172 - 188	1934.0162	1933.0089	1933.0169	-0.0080	0	HQTLQGLAFPLQPEAQR (No match)
172 - 188	1934.0162	1933.0089	1933.0169	-0.0080	0	HQTLQGLAFPLQPEAQR (Ions score 130)
210 - 228	2135.0891	2134.0818	2134.0906	-0.0087	0	ETIELVHTEPTDVAQLPSR (No match)
210 - 228	2135.0891	2134.0818	2134.0906	-0.0087	0	ETIELVHTEPTDVAQLPSR (Ions score 130)
236 - 242	1017.5170	1016.5097	1016.5119	-0.0022	0	YHFFLYK (Ions score 36)
236 - 242	1017.5170	1016.5097	1016.5119	-0.0022	0	YHFFLYK (No match)
243 - 263	2422.1431	2421.1358	2421.1310	0.0048	0	HTHEGDPLESVVFIYSMPGYK Oxidation (M) (No match)
279 - 293	1756.9033	1755.8960	1755.9043	-0.0082	0	LLDSVEQDFHLEIAK (No match)
294 - 315	2474.2466	2473.2393	2473.2376	0.0018	1	KIEIGDGAELTAEFLYDEVHPK (No match)

Spot 579

Protein View

Match to: **gi|182405** Score: **168** Expect: **3e-012**
farnesyl pyrophosphate synthetase

Nominal mass (M_r): **40102**; Calculated pI value: **5.12**
NCBI BLAST search of gi|182405 against nr
Unformatted sequence string for pasting into other applications

Taxonomy: Homo sapiens

Fixed modifications: Carbamidomethyl (C)
Variable modifications: Oxidation (M)
Cleavage by Trypsin: cuts C-term side of KR unless next residue is P
Sequence Coverage: **14%**

Matched peptides shown in **Bold Red**

```
  1 DVYAQEKQDF VQHFSQIVRV LTEDEMGHPE IGDAIARLKE VLEYNAIGGK
 51 YNRGLTVVVA FRELVEPRKQ DADSLQRAWT VGWCVELLQA FFLVADDIMD
101 SSLTRRGQIC WYQKPGVGLD AINDANLLEA CIYRLLKLYC REQPYYLNLI
151 ELFLQSSYQT EIGQTLDLLT APQGNVDLVR FTEKRYKSIV KYKTAFYSFY
201 LPIAAAMYMA GIDGEKEHAN AKKILLEMGE FFQIQDDYLD LFGDPSVTGK
251 IGTDIQDNKC SWLVVQCLQR ATPEQYQILK ENYGQKEAEK VARVKALYEE
301 LDLPAVFLQY EEDSYSHIMA LIEQYAAPLP PAVFLGLARK IYKRRK
```

Residue Number Increasing Mass Decreasing Mass

Start - End	Observed	Mr(expt)	Mr(calc)	Delta	Miss	Sequence
8 - 19	1503.7405	1502.7332	1502.7630	-0.0297	0	QDFVQHFSQIVR (No match)
8 - 19	1503.7405	1502.7332	1502.7630	-0.0297	0	QDFVQHFSQIVR (Ions score 86)
20 - 37	1968.8987	1967.8914	1967.9258	-0.0344	0	VLTEDEMGHPEIGDAIAR Oxidation (M) (Ions score 46)
20 - 37	1968.8987	1967.8914	1967.9258	-0.0344	0	VLTEDEMGHPEIGDAIAR Oxidation (M) (No match)
54 - 62	961.5690	960.5617	960.5756	-0.0139	0	GLTVVVAFR (No match)
271 - 280	1190.6141	1189.6068	1189.6342	-0.0274	0	ATPEQYQILK (No match)

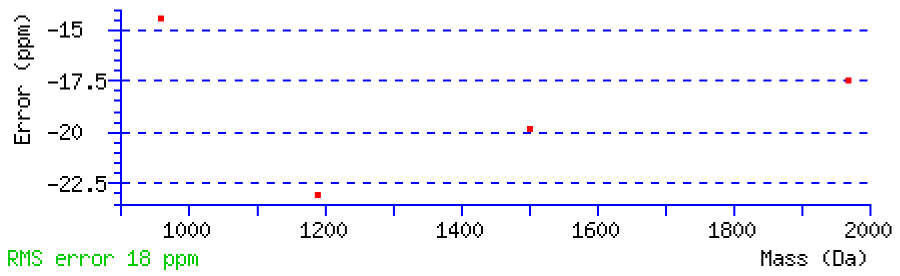
Error (ppm)
-15
-17.5
-20
-22.5
1000
1200
1400
1600
1800
2000
RMS error 18 ppm
Mass (Da)

Spot 581

Protein View

Match to: **gi|119390147** Score: **311** Expect: **1.5e-026**
Chain A, Crystal Structure Of The Heterodimeric Complex Of Human Rgs10 And Activated Gi Alpha 3

Nominal mass (M_r): **37644**; Calculated pI value: **5.58**
NCBI BLAST search of gi|119390147 against nr
Unformatted sequence string for pasting into other applications

Taxonomy: Homo sapiens

Fixed modifications: Carbamidomethyl (C)
Variable modifications: Oxidation (M)
Cleavage by Trypsin: cuts C-term side of KR unless next residue is P
Sequence Coverage: **30%**

Matched peptides shown in **Bold Red**

```
  1 KEVKLLLLGA GESGKSTIVK QMKIIHEDGY SEDECKQYKV VVYSNTIQSI
 51 IAIIRAMGRL KIDFGEAARA DDARQLFVLA GSAEEGVMTP ELAGVIKRLW
101 RDGGVQACFS RSREYQLNDS ASYYLNDLDR ISQSNYIPTQ QDVLRTRVKT
151 TGIVETHFTF KDLYFKMFDV GGQRSERKKW IHCFEGVTAI IFCVALSDYD
201 LVLAEDEEMN RMHESMKLFD SICNNKWFTE TSIILFLNKK DLFEEKIKRS
251 PLTICYPEYT GSNTYEEAAA YIQCQFEDLN RRKDTKEIYT HFTCATDTKN
301 VQFVFDAVTD VIIKNNLKEC GLY
```

Residue Number Increasing Mass Decreasing Mass

Start - End	Observed	Mr(expt)	Mr(calc)	Delta	Miss	Sequence
40 - 55	1789.0947	1788.0874	1788.0508	0.0366	0	VVVYSNTIQSIIAIIR (No match)
40 - 55	1789.0947	1788.0874	1788.0508	0.0366	0	VVVYSNTIQSIIAIIR (Ions score 59)
60 - 69	1119.6395	1118.6322	1118.6083	0.0239	1	LKIDFGEAAR (Ions score 21)
60 - 69	1119.6395	1118.6322	1118.6083	0.0239	1	LKIDFGEAAR (No match)
62 - 69	878.4584	877.4511	877.4293	0.0218	0	IDFGEAAR (No match)
102 - 111	1096.5104	1095.5031	1095.4767	0.0264	0	DGGVQACFSR (Ions score 17)
102 - 111	1096.5104	1095.5031	1095.4767	0.0264	0	DGGVQACFSR (No match)
131 - 145	1761.9498	1760.9425	1760.9057	0.0369	0	ISQSNYIPTQQDVLR (No match)
131 - 145	1761.9498	1760.9425	1760.9057	0.0369	0	ISQSNYIPTQQDVLR (Ions score 81)
150 - 161	1380.7446	1379.7373	1379.7085	0.0289	0	TTGIVETHFTFK (No match)

150 - 166	2047.0939	2046.0866	2046.0462	0.0405	1	**TTGIVETHFTFKDLYFK** (No match)
167 - 174	909.4437	908.4364	908.4174	0.0190	0	**MFDVGGQR** (No match)
167 - 174	925.4376	924.4303	924.4123	0.0180	0	**MFDVGGQR** Oxidation (M) (No match)
218 - 226	1110.5442	1109.5369	1109.5175	0.0194	0	**LFDSICNNK** (No match)
287 - 299	1586.7544	1585.7471	1585.7082	0.0389	0	**EIYTHFTCATDTK** (No match)

Spot 582

Protein View

Match to: **gi|119585457** Score: **673** Expect: **9.6e-063**
guanine nucleotide binding protein (G protein), alpha inhibiting activity polypeptide 2, isoform CR

Nominal mass (M_r): **35425**; Calculated pI value: **5.18**
NCBI BLAST search of gi|119585457 against nr
Unformatted sequence string for pasting into other applications

Taxonomy: Homo sapiens

Fixed modifications: Carbamidomethyl (C)
Variable modifications: Oxidation (M)
Cleavage by Trypsin: cuts C-term side of KR unless next residue is P
Sequence Coverage: **52%**

Matched peptides shown in **Bold Red**

```
  1 MKIIHEDGYS EEECRQYRAV VYSNTIQSIM AIVKAMGNLQ IDFADPSRAD
 51 DARQLFALSC TAEEQGVLPD DLSGVIRRLW ADHGVQACFG RSREYQLNDS
101 AAYYLNDLER IAQSDYIPTQ QDVLRTRVKT TGIVETHFTF KDLHFKMFDV
151 GGQRSERKKW IHCFEGVTAI IFCVALSAYD LVLAEDEEMN RMHESMKLFD
201 SICNNKWFTD TSIILFLNKK DLFEEKITHS PLTICFPEYT GANKYDEAAS
251 YIQSKFEDLN KRKDTKEIYT HFTCATDTKN VQFVFDAVTD VIIKNNLKDC
301 GLF
```

Residue Number Increasing Mass Decreasing Mass

Start - End	Observed	Mr(expt)	Mr(calc)	Delta	Miss	Sequence
3 - 15	1636.7281	1635.7208	1635.6834	0.0374	0	IIHEDGYSEEECR (No match)
3 - 15	1636.7281	1635.7208	1635.6834	0.0374	0	IIHEDGYSEEECR (Ions score 105)
19 - 34	1752.9990	1751.9917	1751.9491	0.0426	0	AVVYSNTIQSIMAIVK Oxidation (M) (No match)
35 - 48	1550.7562	1549.7489	1549.7194	0.0295	0	AMGNLQIDFADPSR Oxidation (M) (No match)
35 - 48	1550.7562	1549.7489	1549.7194	0.0295	0	AMGNLQIDFADPSR Oxidation (M) (Ions score 24)
54 - 77	2618.3818	2617.3745	2617.3057	0.0688	0	QLFALSCTAEEQGVLPDDLSGVIR (No match)
92 - 110	2320.1467	2319.1394	2319.0767	0.0627	1	SREYQLNDSAAYYLNDLER (No match)
92 - 110	2320.1467	2319.1394	2319.0767	0.0627	1	SREYQLNDSAAYYLNDLER (Ions score 103)
94 - 110	2076.9990	2075.9917	2075.9435	0.0482	0	EYQLNDSAAYYLNDLER (No match)
94 - 110	2076.9990	2075.9917	2075.9435	0.0482	0	EYQLNDSAAYYLNDLER (Ions score 149)

111 - 125	1746.9429	1745.9356	1745.8948	0.0408	0	IAQSDYIPTQQDVLR (No match)
111 - 125	1746.9429	1745.9356	1745.8948	0.0408	0	IAQSDYIPTQQDVLR (Ions score 98)
130 - 141	1380.7423	1379.7350	1379.7085	0.0266	0	TTGIVETHFTFK (No match)
147 - 154	925.4351	924.4278	924.4123	0.0155	0	MFDVGGQR Oxidation (M) (No match)
198 - 206	1110.5443	1109.5370	1109.5175	0.0195	0	LFDSICNNK (No match)
227 - 244	2049.0620	2048.0547	2048.0037	0.0511	0	ITHSPLTICFPEYTGANK (No match)
227 - 255	3304.7153	3303.7080	3303.5757	0.1323	1	ITHSPLTICFPEYTGANKYDEAASYIQSK (No match)

Spot 584

Protein View

Match to: **gi|119585458** Score: **314** Expect: **7.7e-027**
guanine nucleotide binding protein (G protein), alpha inhibiting activity polypeptide 2, isoform CR

Nominal mass (M_r): **20295**; Calculated pI value: **5.52**
NCBI BLAST search of gi|119585458 against nr
Unformatted sequence string for pasting into other applications

Taxonomy: Homo sapiens

Fixed modifications: Carbamidomethyl (C)
Variable modifications: Oxidation (M)
Cleavage by Trypsin: cuts C-term side of KR unless next residue is P
Sequence Coverage: **45%**

Matched peptides shown in **Bold Red**

```
  1 MKIIHEDGYS EEECRQYRAV VYSNTIQSIM AIVKAMGNLQ IDFADPSRAD
 51 DARQLFALSC TAEEQGVLPD DLSGVIRRLW ADHGVQACFG RSREYQLNDS
101 AAYYLNDLER IAQSDYIPTQ QDVLRTRVKT TGIVETHFTF KDLHFKMFDV
151 GGQRSERKKW IHCFEGVTAI IFCVA
```

Residue Number Increasing Mass Decreasing Mass

Start - End	Observed	Mr(expt)	Mr(calc)	Delta	Miss	Sequence
3 - 15	1636.6830	1635.6757	1635.6834	-0.0077	0	IIHEDGYSEEECR (No match)
3 - 15	1636.6830	1635.6757	1635.6834	-0.0077	0	IIHEDGYSEEECR (Ions score 36)
35 - 48	1550.7150	1549.7077	1549.7194	-0.0117	0	AMGNLQIDFADPSR Oxidation (M) (No match)
35 - 48	1550.7150	1549.7077	1549.7194	-0.0117	0	AMGNLQIDFADPSR Oxidation (M) (No match)
94 - 110	2076.9292	2075.9219	2075.9435	-0.0216	0	EYQLNDSAAYYLNDLER (No match)
94 - 110	2076.9292	2075.9219	2075.9435	-0.0216	0	EYQLNDSAAYYLNDLER (Ions score 100)
111 - 125	1746.8883	1745.8810	1745.8948	-0.0138	0	IAQSDYIPTQQDVLR (No match)
111 - 125	1746.8883	1745.8810	1745.8948	-0.0138	0	IAQSDYIPTQQDVLR (Ions score 82)
130 - 141	1380.7061	1379.6988	1379.7085	-0.0096	0	TTGIVETHFTFK (No match)
147 - 154	925.4210	924.4137	924.4123	0.0014	0	MFDVGGQR Oxidation (M) (No match)

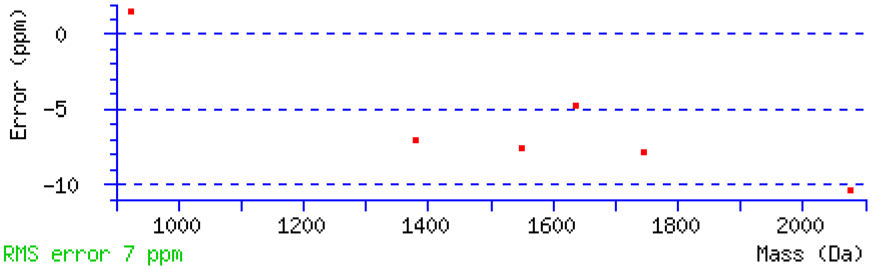

Error (ppm)
0
-5
-10
1000
1200
1400
1600
1800
2000
RMS error 7 ppm
Mass (Da)

Spot 585

Protein View

Match to: **gi|119390147** Score: **445** Expect: **6.1e-040**
Chain A, Crystal Structure Of The Heterodimeric Complex Of Human Rgs10 And Activated Gi Alpha 3

Nominal mass (M_r): **37644**; Calculated pI value: **5.58**
NCBI BLAST search of gi|119390147 against nr
Unformatted sequence string for pasting into other applications

Taxonomy: Homo sapiens

Fixed modifications: Carbamidomethyl (C)
Variable modifications: Oxidation (M)
Cleavage by Trypsin: cuts C-term side of KR unless next residue is P
Sequence Coverage: **39%**

Matched peptides shown in **Bold Red**

```
  1 KEVKLLLLGA GESGKSTIVK QMKIIHEDGY SEDECKQYKV VVYSNTIQSI
 51 IAIIRAMGRL KIDFGEAARA DDARQLFVLA GSAEEGVMTP ELAGVIKRLW
101 RDGGVQACFS RSREYQLNDS ASYYLNDLDR ISQSNYIPTQ QDVLRTRVKT
151 TGIVETHFTF KDLYFKMFDV GGQRSERKKW IHCFEGVTAI IFCVALSDYD
201 LVLAEDEEMN RMHESMKLFD SICNNKWFTE TSIILFLNKK DLFEEKIKRS
251 PLTICYPEYT GSNTYEEAAA YIQCQFEDLN RRKDTKEIYT HFTCATDTKN
301 VQFVFDAVTD VIIKNNLKEC GLY
```

Residue Number Increasing Mass Decreasing Mass

Start - End	Observed	Mr(expt)	Mr(calc)	Delta	Miss	Sequence
40 - 55	1788.9829	1787.9756	1788.0508	-0.0752	0	VVVYSNTIQSIIAIIR (No match)
40 - 55	1788.9829	1787.9756	1788.0508	-0.0752	0	VVVYSNTIQSIIAIIR (Ions score 65)
60 - 69	1119.5660	1118.5587	1118.6083	-0.0496	1	LKIDFGEAAR (No match)
60 - 69	1119.5660	1118.5587	1118.6083	-0.0496	1	LKIDFGEAAR (Ions score 42)
62 - 69	878.3931	877.3858	877.4293	-0.0435	0	IDFGEAAR (No match)
75 - 98	2531.2295	2530.2222	2530.3464	-0.1242	1	QLFVLAGSAEEGVMTPELAGVIKR Oxidation (M) (No match)
102 - 111	1096.4294	1095.4221	1095.4767	-0.0546	0	DGGVQACFSR (Ions score 33)
102 - 111	1096.4294	1095.4221	1095.4767	-0.0546	0	DGGVQACFSR (No match)
112 - 130	2321.9639	2320.9566	2321.0560	-0.0993	1	SREYQLNDSASYYLNDLDR (No match)
114 - 130	2078.8455	2077.8382	2077.9228	-0.0846	0	EYQLNDSASYYLNDLDR (No match)

114 - 130	2078.8455	2077.8382	2077.9228	-0.0846	0	**EYQLNDSASYYLNDLDR** (Ions score 92)
131 - 145	1761.8376	1760.8303	1760.9057	-0.0753	0	**ISQSNYIPTQQDVLR** (Ions score 79)
131 - 145	1761.8376	1760.8303	1760.9057	-0.0753	0	**ISQSNYIPTQQDVLR** (No match)
150 - 161	1380.6615	1379.6542	1379.7085	-0.0542	0	**TTGIVETHFTFK** (No match)
167 - 174	925.3698	924.3625	924.4123	-0.0498	0	**MFDVGGQR** Oxidation (M) (No match)
287 - 299	1586.6472	1585.6399	1585.7082	-0.0683	0	**EIYTHFTCATDTK** (No match)

Spot 586

Protein View

Match to: **gi|63252913** Score: **334** Expect: **7.7e-029**
gelsolin-like capping protein [Homo sapiens]

Nominal mass (M_r): **38760;** Calculated pI value: **5.82**
NCBI BLAST search of gi|63252913 against nr
Unformatted sequence string for pasting into other applications

Taxonomy: Homo sapiens
Links to retrieve other entries containing this sequence from NCBI Entrez:
gi|62988741 from Homo sapiens

Fixed modifications: Carbamidomethyl (C)
Variable modifications: Oxidation (M)
Cleavage by Trypsin: cuts C-term side of KR unless next residue is P
Sequence Coverage: **41%**

Matched peptides shown in **Bold Red**

```
  1 MYTAIPQSGS PFPGSVQDPG LHVWRVEKLK PVPVAQENQG VFFSGDSYLV
 51 LHNGPEEVSH LHLWIGQQSS RDEQGACAVL AVHLNTLLGE RPVQHREVQG
101 NESDLFMSYF PRGLKYQEGG VESAFHKTST GAPAAIKKLY QVKGKKNIRA
151 TERALNWDSF NTGDCFILDL GQNIFAWCGG KSNILERNKA RDLALAIRDS
201 ERQGKAQVEI VTDGEEPAEM IQVLGPKPAL KEGNPEEDLT ADKANAQAAA
251 LYKVSDATGQ MNLTKVADSS PFALELLISD DCFVLDNGLC GKIYIWKGRK
301 ANEKERQAAL QVAEGFISRM QYAPNTQVEI LPQGHESPIF KQFFKDWK
```

Residue Number Increasing Mass Decreasing Mass

Start - End	Observed	Mr(expt)	Mr(calc)	Delta	Miss	Sequence
72 - 96	2783.2722	2782.2649	2782.4296	-0.1647	0	DEQGACAVLAVHLNTLLGERPVQHR (No match)
97 - 112	1934.7444	1933.7371	1933.8515	-0.1144	0	EVQGNESDLFMSYFPR Oxidation (M) (Ions score 30)
97 - 112	1934.7444	1933.7371	1933.8515	-0.1144	0	EVQGNESDLFMSYFPR Oxidation (M) (No match)
113 - 127	1649.7289	1648.7216	1648.8208	-0.0992	1	GLKYQEGGVESAFHK (No match)
116 - 127	1351.5457	1350.5384	1350.6203	-0.0819	0	YQEGGVESAFHK (No match)
116 - 127	1351.5457	1350.5384	1350.6203	-0.0819	0	YQEGGVESAFHK (Ions score 69)
206 - 231	2778.3054	2777.2981	2777.4520	-0.1539	0	AQVEIVTDGEEPAEMIQVLGPKPALK Oxidation (M) (No match)
232 - 253	2318.9783	2317.9710	2318.1025	-0.1315	1	EGNPEEDLTADKANAQAAALYK (No match)

307 - 319	1389.6677	1388.6604	1388.7411	-0.0807	0	QAALQVAEGFISR (No match)
307 - 319	1389.6677	1388.6604	1388.7411	-0.0807	0	QAALQVAEGFISR (Ions score 77)
320 - 341	2543.1194	2542.1121	2542.2525	-0.1404	0	MQYAPNTQVEILPQGHESPIFK Oxidation (M) (No match)
320 - 341	2543.1194	2542.1121	2542.2525	-0.1404	0	MQYAPNTQVEILPQGHESPIFK Oxidation (M) (Ions score 64)
342 - 348	998.5380	997.5307	997.5021	0.0286	1	QFFKDWK (No match)

Spot 587

Protein View

Match to: **gi|110591508** Score: **73** Expect: **0.0092**
Chain A, Structure Of The Binary Complex Of The E67l Mutant Of Human Glutathione-Dependent Formalde

Nominal mass (M_r): **40407**; Calculated pI value: **7.90**
NCBI BLAST search of gi|110591508 against nr
Unformatted sequence string for pasting into other applications

Taxonomy: Homo sapiens
Links to retrieve other entries containing this sequence from NCBI Entrez:
gi|110591509 from Homo sapiens

Fixed modifications: Carbamidomethyl (C)
Variable modifications: Oxidation (M)
Cleavage by Trypsin: cuts C-term side of KR unless next residue is P
Sequence Coverage: **8%**

Matched peptides shown in **Bold Red**

```
  1 ANEVIKCKAA VAWEAGKPLS IEEIEVAPPK AHEVRIKIIA TAVCHTDAYT
 51 LSGADPEGCF PVILGHLGAG IVESVGEGVT KLKAGDTVIP LYIPQCGECK
101 FCLNPKTNLC QKIRVTQGKG LMPDGTSRFT CKGKTILHYM GTSTFSEYTV
151 VADISVAKID PLAPLDKVCL LGCGISTGYG AAVNTAKLEP GSVCAVFGLG
201 GVGLAVIMGC KVAGASRIIG VDINKDKFAR AKEFGATECI NPQDFSKPIQ
251 EVLIEMTDGG VDYSFECIGN VKVMRAALEA CHKGWGVSVV VGVAASGEEI
301 ATRPFQLVTG RTWKGTAFGG WKSVESVPKL VSEYMSKKIK VDEFVTHNLS
351 FDEINKAFEL MHSGKSIRTV VKI
```

Residue Number Increasing Mass Decreasing Mass

Start - End	Observed	Mr(expt)	Mr(calc)	Delta	Miss	Sequence	
84 - 100	1920.9369	1919.9296	1919.9121	0.0176	0	AGDTVIPLYIPQCGECK	(Ions score 46)
84 - 100	1920.9369	1919.9296	1919.9121	0.0176	0	AGDTVIPLYIPQCGECK	(No match)
341 - 356	1906.9283	1905.9210	1905.9108	0.0102	0	VDEFVTHNLSFDEINK	(No match)
341 - 356	1906.9283	1905.9210	1905.9108	0.0102	0	VDEFVTHNLSFDEINK	(Ions score 7)

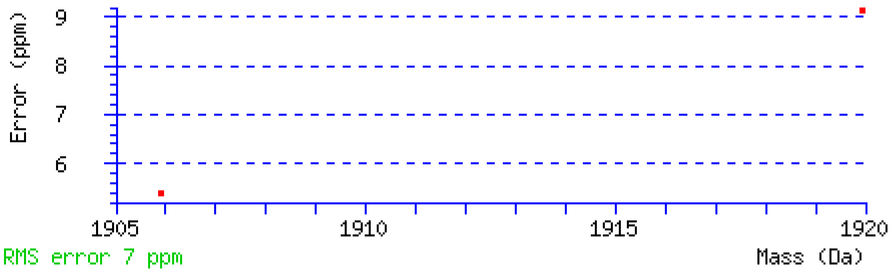

Error (ppm)
9
8
7
6
1905
1910
1915
1920
Mass (Da)
RMS error 7 ppm

Spot 588

Protein View

Match to: **gi|119390147** Score: **388** Expect: **3e-034**
Chain A, Crystal Structure Of The Heterodimeric Complex Of Human Rgs10 And Activated Gi Alpha 3

Nominal mass (M_r): **37644**; Calculated pI value: **5.58**
NCBI BLAST search of gi|119390147 against nr
Unformatted sequence string for pasting into other applications

Taxonomy: Homo sapiens

Fixed modifications: Carbamidomethyl (C)
Variable modifications: Oxidation (M)
Cleavage by Trypsin: cuts C-term side of KR unless next residue is P
Sequence Coverage: **31%**

Matched peptides shown in **Bold Red**

```
  1 KEVKLLLLGA GESGKSTIVK QMKIIHEDGY SEDECKQYKV VVYSNTIQSI
 51 IAIIRAMGRL KIDFGEAARA DDARQLFVLA GSAEEGVMTP ELAGVIKRLW
101 RDGGVQACFS RSREYQLNDS ASYYLNDLDR ISQSNYIPTQ QDVLRTRVKT
151 TGIVETHFTF KDLYFKMFDV GGQRSERKKW IHCFEGVTAI IFCVALSDYD
201 LVLAEDEEMN RMHESMKLFD SICNNKWFTE TSIILFLNKK DLFEEKIKRS
251 PLTICYPEYT GSNTYEEAAA YIQCQFEDLN RRKDTKEIYT HFTCATDTKN
301 VQFVFDAVTD VIIKNNLKEC GLY
```

Residue Number Increasing Mass Decreasing Mass

Start - End	Observed	Mr(expt)	Mr(calc)	Delta	Miss	Sequence
40 - 55	1788.9828	1787.9755	1788.0508	-0.0753	0	VVVYSNTIQSIIAIIR (No match)
40 - 55	1788.9828	1787.9755	1788.0508	-0.0753	0	VVVYSNTIQSIIAIIR (Ions score 62)
60 - 69	1119.5724	1118.5651	1118.6083	-0.0432	1	LKIDFGEAAR (No match)
60 - 69	1119.5724	1118.5651	1118.6083	-0.0432	1	LKIDFGEAAR (Ions score 35)
62 - 69	878.3986	877.3913	877.4293	-0.0380	0	IDFGEAAR (No match)
102 - 111	1096.4332	1095.4259	1095.4767	-0.0508	0	DGGVQACFSR (Ions score 16)
102 - 111	1096.4332	1095.4259	1095.4767	-0.0508	0	DGGVQACFSR (No match)
114 - 130	2078.8430	2077.8357	2077.9228	-0.0871	0	EYQLNDSASYYLNDLDR (No match)
131 - 145	1761.8402	1760.8329	1760.9057	-0.0727	0	ISQSNYIPTQQDVLR (Ions score 95)
131 - 145	1761.8402	1760.8329	1760.9057	-0.0727	0	ISQSNYIPTQQDVLR (No match)

150 - 161	1380.6589	1379.6516	1379.7085	-0.0568	0	**TTGIVETHFTFK** (No match)
150 - 161	1380.6589	1379.6516	1379.7085	-0.0568	0	**TTGIVETHFTFK** (Ions score 52)
167 - 174	925.3746	924.3673	924.4123	-0.0450	0	**MFDVGGQR** Oxidation (M) (No match)
287 - 299	1586.6526	1585.6453	1585.7082	-0.0629	0	**EIYTHFTCATDTK** (No match)

Spot 589

Protein View

Match to: **gi|95113651** Score: **177** Expect: **3.8e-013**
glutaredoxin 3 [Homo sapiens]

Nominal mass (M_r): **37693**; Calculated pI value: **5.31**
NCBI BLAST search of gi|95113651 against nr
Unformatted sequence string for pasting into other applications

Taxonomy: Homo sapiens
Links to retrieve other entries containing this sequence from NCBI Entrez:
gi|37087933 from Homo sapiens
gi|6840953 from Homo sapiens
gi|13528999 from Homo sapiens
gi|14329514 from Homo sapiens
gi|119569537 from Homo sapiens
gi|119569539 from Homo sapiens
gi|119569540 from Homo sapiens

Fixed modifications: Carbamidomethyl (C)
Variable modifications: Oxidation (M)
Cleavage by Trypsin: cuts C-term side of KR unless next residue is P
Sequence Coverage: **32%**

Matched peptides shown in **Bold Red**

```
  1 MAAGAAEAAV AAVEEVGSAG QFEELLRLKA KSLLVVHFWA PWAPQCAQMN
 51 EVMAELAKEL PQVSFVKLEA EGVPEVSEKY EISSVPTFLF FKNSQKIDRL
101 DGAHAPELTK KVQRHASSGS FLPSANEHLK EDLNLRLKKL THAAPCMLFM
151 KGTPQEPRCG FSKQMVEILH KHNIQFSSFD IFSDEEVRQG LKAYSSWPTY
201 PQLYVSGELI GGLDIIKELE ASEELDTICP KAPKLEERLK VLTNKASVML
251 FMKGNKQEAK CGFSKQILEI LNSTGVEYET FDILEDEEVR QGLKAYSNWP
301 TYPQLYVKGE LVGGLDIVKE LKENGELLPI LRGEN
```

Residue Number Increasing Mass Decreasing Mass

Start - End	Observed	Mr(expt)	Mr(calc)	Delta	Miss	Sequence
59 - 67	1046.5173	1045.5100	1045.5807	-0.0707	0	ELPQVSFVK (No match)
68 - 79	1286.6958	1285.6885	1285.6401	0.0484	0	LEAEGVPEVSEK (No match)

68 - 79	1286.6958	1285.6885	1285.6401	0.0484	0	LEAEGVPEVSEK (Ions score 3)
100 - 110	1151.6486	1150.6413	1150.5982	0.0431	0	LDGAHAPELTK (No match)
115 - 136	2422.0540	2421.0467	2421.2036	-0.1569	1	HASSGSFLPSANEHLKEDLNLR (No match)
172 - 188	2069.8330	2068.8257	2068.9490	-0.1232	0	HNIQFSSFDIFSDEEVR (No match)
172 - 188	2069.8330	2068.8257	2068.9490	-0.1232	0	HNIQFSSFDIFSDEEVR (Ions score 54)
266 - 290	2954.2576	2953.2503	2953.4443	-0.1940	0	QILEILNSTGVEYETFDILEDEEVR (No match)
320 - 332	1523.7891	1522.7818	1522.8718	-0.0900	1	ELKENGELLPILR (Ions score 41)
320 - 332	1523.7891	1522.7818	1522.8718	-0.0900	1	ELKENGELLPILR (No match)
323 - 332	1153.5972	1152.5899	1152.6502	-0.0603	0	ENGELLPILR (No match)

Spot 590

Protein View

Match to: **gi|5031601** Score: **513** Expect: **9.6e-047**
actin related protein 2/3 complex subunit 1B [Homo sapiens]

Nominal mass (M_r): **41722**; Calculated pI value: **8.69**
NCBI BLAST search of gi|5031601 against nr
Unformatted sequence string for pasting into other applications

Taxonomy: Homo sapiens
Links to retrieve other entries containing this sequence from NCBI Entrez:
gi|3121763 from Homo sapiens
gi|2282034 from Homo sapiens
gi|12803475 from Homo sapiens
gi|14043135 from Homo sapiens
gi|31416821 from Homo sapiens
gi|51094630 from Homo sapiens
gi|119597083 from Homo sapiens
gi|119597084 from Homo sapiens
gi|119597085 from Homo sapiens
gi|119597086 from Homo sapiens
gi|123982708 from synthetic construct
gi|123997375 from synthetic construct

Fixed modifications: Carbamidomethyl (C)
Variable modifications: Oxidation (M)
Cleavage by Trypsin: cuts C-term side of KR unless next residue is P
Sequence Coverage: **39%**

Matched peptides shown in **Bold Red**

1 MAYHSFLVEP ISCHAWNKDR **TQIAICPNNH** **EVHIYEK**SGA KWTK**VHELKE**
51 **HNGQVTGIDW** **APESNR**IVTC GTDRNAYVWT LKGR**TWKPTL** **VILR**INRAAR
101 CVR**WAPNENK** **FAVGSGSR**VI SICYFEQEND WWVCKHIKKP IR**STVLSLDW**
151 **HPNNVLLAAG** **SCDFK**CRIFS AYIK**EVEERP** **APTPWGSKMP** **FGELMFESSS**
201 **SCGWVHGVCF** **SASGSRVAWV** **SHDSTVCLAD** **ADKK**MAVATL ASETLPLLAL
251 TFITDNSLVA AGHDCFPVLF TYDAAAGMLS FGGRLDVPKQ SSQRGLTARE
301 RFQNLDKKAS SEGGTAAGAG LDSLHKNSVS QISVLSGGKA KCSQFCTTGM
351 DGGMSIWDVK SLESALKDLK IK

Residue Number Increasing Mass Decreasing Mass

Start - End	Observed	Mr(expt)	Mr(calc)	Delta	Miss	Sequence
21 - 37	2066.0085	2065.0012	2065.0051	-0.0038	0	TQIAICPNNHEVHIYEK (Ions score 85)
21 - 37	2066.0085	2065.0012	2065.0051	-0.0038	0	TQIAICPNNHEVHIYEK (No match)
45 - 66	2516.1941	2515.1868	2515.2203	-0.0335	1	VHELKEHNGQVTGIDWAPESNR (No match)
50 - 66	1909.8674	1908.8601	1908.8714	-0.0113	0	EHNGQVTGIDWAPESNR (No match)
85 - 94	1226.7589	1225.7516	1225.7546	-0.0030	0	TWKPTLVILR (Ions score 63)
85 - 94	1226.7589	1225.7516	1225.7546	-0.0030	0	TWKPTLVILR (No match)
104 - 118	1619.7836	1618.7763	1618.7851	-0.0088	1	WAPNENKFAVGSGSR (No match)
143 - 165	2544.2314	2543.2241	2543.2478	-0.0237	0	STVLSLDWHPNNVLLAAGSCDFK (No match)
175 - 188	1582.7819	1581.7746	1581.7787	-0.0040	0	EVEERPAPTPWGSK (No match)
175 - 188	1582.7819	1581.7746	1581.7787	-0.0040	0	EVEERPAPTPWGSK (Ions score 66)
189 - 216	3096.2549	3095.2476	3095.3033	-0.0557	0	MPFGELMFESSSSCGWVHGVCFSASGSR (No match)
189 - 216	3096.2549	3095.2476	3095.3033	-0.0557	0	MPFGELMFESSSSCGWVHGVCFSASGSR (Ions score 172)
217 - 234	2001.9514	2000.9441	2000.9625	-0.0184	1	VAWVSHDSTVCLADADKK (No match)

Spot 591

Protein View

Match to: **gi|119585457** Score: **525** Expect: **6.1e-048**
guanine nucleotide binding protein (G protein), alpha inhibiting activity polypeptide 2, isoform CR

Nominal mass (M_r): **35425**; Calculated pI value: **5.18**
NCBI BLAST search of gi|119585457 against nr
Unformatted sequence string for pasting into other applications

Taxonomy: Homo sapiens

Fixed modifications: Carbamidomethyl (C)
Variable modifications: Oxidation (M)
Cleavage by Trypsin: cuts C-term side of KR unless next residue is P
Sequence Coverage: **36%**

Matched peptides shown in **Bold Red**

```
  1 MKIIHEDGYS EEECRQYRAV VYSNTIQSIM AIVKAMGNLQ IDFADPSRAD
 51 DARQLFALSC TAEEQGVLPD DLSGVIRRLW ADHGVQACFG RSREYQLNDS
101 AAYYLNDLER IAQSDYIPTQ QDVLRTRVKT TGIVETHFTF KDLHFKMFDV
151 GGQRSERKKW IHCFEGVTAI IFCVALSAYD LVLAEDEEMN RMHESMKLFD
201 SICNNKWFTD TSIILFLNKK DLFEEKITHS PLTICFPEYT GANKYDEAAS
251 YIQSKFEDLN KRKDTKEIYT HFTCATDTKN VQFVFDAVTD VIIKNNLKDC
301 GLF
```

Residue Number Increasing Mass Decreasing Mass

Start - End	Observed	Mr(expt)	Mr(calc)	Delta	Miss	Sequence
3 - 15	1636.6329	1635.6256	1635.6834	-0.0578	0	IIHEDGYSEEECR (Ions score 57)
3 - 15	1636.6329	1635.6256	1635.6834	-0.0578	0	IIHEDGYSEEECR (No match)
35 - 48	1550.6648	1549.6575	1549.7194	-0.0619	0	AMGNLQIDFADPSR Oxidation (M) (Ions score 16)
35 - 48	1550.6648	1549.6575	1549.7194	-0.0619	0	AMGNLQIDFADPSR Oxidation (M) (No match)
54 - 77	2618.2256	2617.2183	2617.3057	-0.0874	0	QLFALSCTAEEQGVLPDDLSGVIR (No match)
54 - 77	2618.2256	2617.2183	2617.3057	-0.0874	0	QLFALSCTAEEQGVLPDDLSGVIR (Ions score 122)
94 - 110	2076.8787	2075.8714	2075.9435	-0.0721	0	EYQLNDSAAYYLNDLER (No match)
94 - 110	2076.8787	2075.8714	2075.9435	-0.0721	0	EYQLNDSAAYYLNDLER (Ions score 121)
111 - 125	1746.8391	1745.8318	1745.8948	-0.0630	0	IAQSDYIPTQQDVLR (Ions score 96)
111 - 125	1746.8391	1745.8318	1745.8948	-0.0630	0	IAQSDYIPTQQDVLR (No match)

130 - 141	1380.6606	1379.6533	1379.7085	-0.0551	0	**TTGIVETHFTFK**	(No match)
147 - 154	925.3759	924.3686	924.4123	-0.0437	0	**MFDVGGQR**	Oxidation (M) (No match)
198 - 206	1110.4702	1109.4629	1109.5175	-0.0546	0	**LFDSICNNK**	(No match)

Spot 592

Protein View

Match to: **gi|93279884** Score: **165** Expect: **6.1e-012**
Chain A, Crystal Structure Of Human Ptpa

Nominal mass (M_r): **37539**; Calculated pI value: **6.57**
NCBI BLAST search of gi|93279884 against nr
Unformatted sequence string for pasting into other applications

Taxonomy: Homo sapiens

Fixed modifications: Carbamidomethyl (C)
Variable modifications: Oxidation (M)
Cleavage by Trypsin: cuts C-term side of KR unless next residue is P
Sequence Coverage: **19%**

Matched peptides shown in **Bold Red**

```
  1 MHHHHHHSSG VDLGTENLYF QSRNFIIPKK EIHTVPDMGK WKRSQAYADY
 51 IGFILTLNEG VKGKKLTFEY RVSEAIEKLV ALLNTLDRWI DETPPVDQPS
101 RFGNKAYRTW YAKLDEEAEN LVATVVPTHL AAAVPEVAVY LKESVGNSTR
151 IDYGTGHEAA FAAFLCCLCK IGVLRVDDQI AIVFKVFNRY LEVMRKLQKT
201 YRMEPAGSQG VWGLDDFQFL PFIWGSSQLI DHPYLEPRHF VDEKAVNENH
251 KDYMFLECIL FITEMKTGPF AEHSNQLWNI SAVPSWSKVN QGLIRMYKAE
301 CLEKFPVIQH FKFGSLLPIH PVTSG
```

Residue Number Increasing Mass Decreasing Mass

Start - End	Observed	Mr(expt)	Mr(calc)	Delta	Miss	Sequence
65 - 71	956.5154	955.5081	955.5127	-0.0045	1	KLTFEYR (No match)
66 - 71	828.4261	827.4188	827.4177	0.0011	0	LTFEYR (No match)
79 - 88	1127.6608	1126.6535	1126.6710	-0.0174	0	LVALLNTLDR (No match)
79 - 88	1127.6608	1126.6535	1126.6710	-0.0174	0	LVALLNTLDR (Ions score 4)
89 - 101	1539.7224	1538.7151	1538.7365	-0.0214	0	WIDETPPVDQPSR (Ions score 26)
89 - 101	1539.7224	1538.7151	1538.7365	-0.0214	0	WIDETPPVDQPSR (No match)
289 - 295	799.4737	798.4664	798.4711	-0.0047	0	VNQGLIR (No match)
299 - 312	1745.8845	1744.8772	1744.8970	-0.0198	1	AECLEKFPVIQHFK (No match)
305 - 312	1015.5578	1014.5505	1014.5650	-0.0145	0	FPVIQHFK (No match)
305 - 312	1015.5578	1014.5505	1014.5650	-0.0145	0	FPVIQHFK (Ions score 22)

313 - 325	1324.7048	1323.6975	1323.7186	-0.0211	0	FGSLLPIHPVTSG	(No match)
313 - 325	1324.7048	1323.6975	1323.7186	-0.0211	0	FGSLLPIHPVTSG	(Ions score 13)

Spot 594

Protein View

Match to: **gi|61680217** Score: **449** Expect: **2.4e-040**
Chain A, Human Cytosolic Acetoacetyl-Coa Thiolase Complexed With Coa

Nominal mass (M_r): **41789**; Calculated pI value: **6.47**
NCBI BLAST search of gi|61680217 against nr
Unformatted sequence string for pasting into other applications

Taxonomy: Homo sapiens
Links to retrieve other entries containing this sequence from NCBI Entrez:
gi|61680218 from Homo sapiens

Fixed modifications: Carbamidomethyl (C)
Variable modifications: Oxidation (M)
Cleavage by Trypsin: cuts C-term side of KR unless next residue is P
Sequence Coverage: **23%**

Matched peptides shown in **Bold Red**

```
  1 MNAGSDPVVI VSAARTIIGS FNGALAAVPV QDLGSTVIKE VLKRATVAPE
 51 DVSEVIFGHV LAAGCGQNPV RQASVGAGIP YSVPAWSCQM IXGSGLKAVC
101 LAVQSIGIGD SSIVVAGGME NMSKAPHLAY LRTGVKIGEM PLTDSILCDG
151 LTDAFHNCHM GITAENVAKK WQVSREDQDK VAVLSQNRTE NAQKAGHFDK
201 EIVPVLVSTR KGLIEVKTDE FPRHGSNIEA MSKLKPYFLT DGTGTVTPAN
251 ASGINDGAAA VVLMKKSEAD KRGLTPLARI VSWSQVGVEP SIMGIGPIPA
301 IKQAVTKAGW SLEDVDIFEI NEAFAAVSAA IVKELGLNPE KVNIEGGAIA
351 LGHPLGASGC RILVTLLHTL ERMGRSRGVA ALCIGGGMGI AMCVQRE
```

Residue Number Increasing Mass Decreasing Mass

Start - End	Observed	Mr(expt)	Mr(calc)	Delta	Miss	Sequence
45 - 71	2793.3396	2792.3323	2792.3914	-0.0591	0	ATVAPEDVSEVIFGHVLAAGCGQNPVR (No match)
45 - 71	2793.3396	2792.3323	2792.3914	-0.0591	0	ATVAPEDVSEVIFGHVLAAGCGQNPVR (Ions score 140)
125 - 132	940.5209	939.5136	939.5290	-0.0154	0	APHLAYLR (No match)
176 - 188	1501.7349	1500.7276	1500.7532	-0.0256	1	EDQDKVAVLSQNR (No match)
195 - 210	1767.9398	1766.9325	1766.9678	-0.0353	1	AGHFDKEIVPVLVSTR (No match)
195 - 210	1767.9398	1766.9325	1766.9678	-0.0353	1	AGHFDKEIVPVLVSTR (Ions score 109)
342 - 361	1948.9608	1947.9535	1947.9948	-0.0412	0	VNIEGGAIALGHPLGASGCR (Ions score 96)

342 - 361	1948.9608	1947.9535	1947.9948	-0.0412	0	VNIEGGAIALGHPLGASGCR	(No match)
362 - 372	1307.7770	1306.7697	1306.7972	-0.0275	0	ILVTLLHTLER	(Ions score 30)
362 - 372	1307.7770	1306.7697	1306.7972	-0.0275	0	ILVTLLHTLER	(No match)

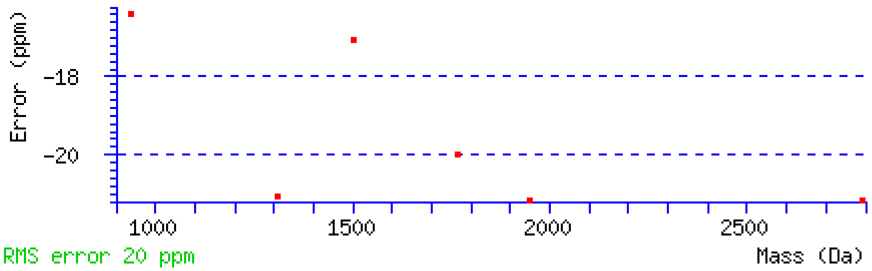

Spot 596

Protein View

Match to: **gi|20530221** Score: **245** Expect: **6.1e-020**
BLOCK 25 [Homo sapiens]

Nominal mass (M_r): **24185**; Calculated pI value: **5.61**
NCBI BLAST search of gi|20530221 against nr
Unformatted sequence string for pasting into other applications

Taxonomy: Homo sapiens

Fixed modifications: Carbamidomethyl (C)
Variable modifications: Oxidation (M)
Cleavage by Trypsin: cuts C-term side of KR unless next residue is P
Sequence Coverage: **42%**

Matched peptides shown in **Bold Red**

```
  1 MASVTRAVFG ELPSGGGTVE KFQLQSDLLR VDIISWGCTI TALEVKDRQG
 51 RASDVVLGFA ELEGYLQKQP YFGAVIGRVA NRIAKGTFKV DGKEYHLAIN
101 KEPNSLHGGV RGFDKVLWTP RVLSNGVQFS RISPDGEEGY PGELKVWVTY
151 TLDGGELIVN YRAQASQATP VNLTNHSYFN LAGQASPNIN DHEVTIEADT
201 YLPVDETLIP TGGTNRRFLQ G
```

Residue Number Increasing Mass Decreasing Mass

Start - End	Observed	Mr(expt)	Mr(calc)	Delta	Miss	Sequence
7 - 21	1447.6730	1446.6657	1446.7354	-0.0696	0	AVFGELPSGGGTVEK (No match)
22 - 30	1119.5575	1118.5502	1118.6084	-0.0581	0	FQLQSDLLR (Ions score 30)
22 - 30	1119.5575	1118.5502	1118.6084	-0.0581	0	FQLQSDLLR (No match)
52 - 68	1838.8518	1837.8445	1837.9461	-0.1016	0	ASDVVLGFAELEGYLQK (No match)
69 - 78	1107.5360	1106.5287	1106.5872	-0.0585	0	QPYFGAVIGR (No match)
69 - 78	1107.5360	1106.5287	1106.5872	-0.0585	0	QPYFGAVIGR (Ions score 37)
94 - 111	2033.9530	2032.9457	2033.0442	-0.0984	1	EYHLAINKEPNSLHGGVR (No match)
94 - 111	2033.9530	2032.9457	2033.0442	-0.0984	1	EYHLAINKEPNSLHGGVR (Ions score 90)
122 - 131	1106.5364	1105.5291	1105.5879	-0.0588	0	VLSNGVQFSR (No match)
132 - 145	1490.6245	1489.6172	1489.6936	-0.0763	0	ISPDGEEGYPGELK (No match)

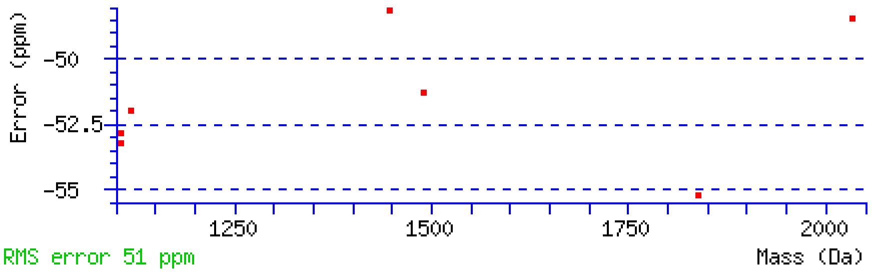
Error (ppm)
-50
-52.5
-55
1250
1500
1750
2000
RMS error 51 ppm
Mass (Da)

Spot 598

Protein View

Match to: **gi|33872740** Score: **99** Expect: **2.4e-005**
GIPC1 protein [Homo sapiens]

Nominal mass (M_r): **32792**; Calculated pI value: **5.89**
NCBI BLAST search of gi|33872740 against nr
Unformatted sequence string for pasting into other applications

Taxonomy: Homo sapiens

Fixed modifications: Carbamidomethyl (C)
Variable modifications: Oxidation (M)
Cleavage by Trypsin: cuts C-term side of KR unless next residue is P
Sequence Coverage: **26%**

Matched peptides shown in **Bold Red**

```
  1 PLGGGGSGGP QMGLPPPPPA LRPRLVFHTQ LAHGSPTGRI EGFTNVKELY
 51 GKIAEAFRLP TAEVMFCTLN THKVDMDKLL GGQIGLEDFI FAHVKGQRKE
101 VEVFKSEDAL GLTITDNGAG YAFIKRIKEG SVIDHIHLIS VGDMIEAING
151 QSLLGCRHYE VARLLKELPR GRTFTLKLTE PRKAFDMISQ RSAGGRPGSG
201 PQLGTGRGTL RLRSRGPATV EDLPSAFEEK AIEKVDDLLE SYMGIRDTEL
251 AATMVELGKD KRNPDELAEA LDERLGDFAF PDEFVFDVWG AIGDAKVGRY
301
```

Residue Number Increasing Mass Decreasing Mass

Start - End	Observed	Mr(expt)	Mr(calc)	Delta	Miss	Sequence
25 - 39	1620.8890	1619.8817	1619.8532	0.0286	0	LVFHTQLAHGSPTGR (No match)
25 - 39	1620.8890	1619.8817	1619.8532	0.0286	0	LVFHTQLAHGSPTGR (Ions score 27)
106 - 126	2212.1545	2211.1472	2211.1171	0.0302	1	SEDALGLTITDNGAGYAFIKR (No match)
106 - 126	2212.1545	2211.1472	2211.1171	0.0302	1	SEDALGLTITDNGAGYAFIKR (No match)
183 - 191	1111.5696	1110.5623	1110.5491	0.0132	1	KAFDMISQR Oxidation (M) (No match)
183 - 191	1111.5696	1110.5623	1110.5491	0.0132	1	KAFDMISQR Oxidation (M) (No match)
192 - 207	1454.7698	1453.7625	1453.7385	0.0240	0	SAGGRPGSGPQLGTGR (Ions score 6)
192 - 207	1454.7698	1453.7625	1453.7385	0.0240	0	SAGGRPGSGPQLGTGR (No match)
214 - 230	1832.9343	1831.9270	1831.8951	0.0319	1	SRGPATVEDLPSAFEEK (No match)
214 - 230	1832.9343	1831.9270	1831.8951	0.0319	1	SRGPATVEDLPSAFEEK (No match)

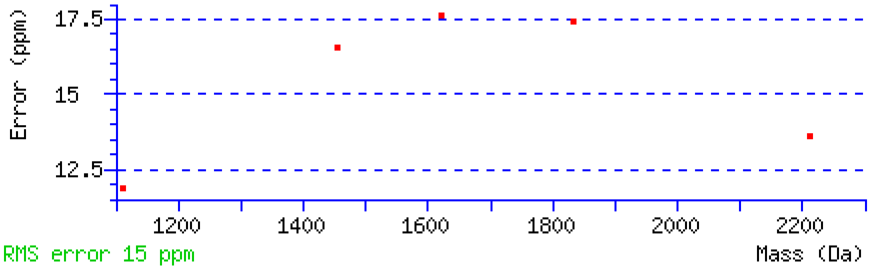
Error (ppm)
17.5
15
12.5
1200
1400
1600
1800
2000
2200
Mass (Da)
RMS error 15 ppm

Spot 599

Protein View

Match to: **gi|542837** Score: **138** Expect: **3e-009**
H+-exporting ATPase (EC 3.6.3.6) chain D, vacuolar - human

Nominal mass (M_r): **32083**; Calculated pI value: **5.16**
NCBI BLAST search of gi|542837 against nr
Unformatted sequence string for pasting into other applications

Taxonomy: Homo sapiens
Links to retrieve other entries containing this sequence from NCBI Entrez:
gi|313012 from Homo sapiens

Fixed modifications: Carbamidomethyl (C)
Variable modifications: Oxidation (M)
Cleavage by Trypsin: cuts C-term side of KR unless next residue is P
Sequence Coverage: **17%**

Matched peptides shown in **Bold Red**

```
  1 MVVEFRHMRN HAYEPLASFL DFITYSYMID NVILLITGTL HQRSIAELVP
 51 KCHPLGSFEQ MEAVNIAQTP AELYNAILVD TPLAAFFQDC ISEQDLDEMN
101 IEIIRNTLYK AYLESFYKFC TLLGGTTADA MCPILEFEAD RRAFIITINS
151 FGTELSKEDR AKLFPHCGRL YPEGLAQLAR ADDYEQVKKL ADYYPEYKLL
201 FEGAGSNPGD KTLEDRFFEH EVKLNKLAFL NQFHFGVFYA FVKLKEQECR
251 NIVWIAECIA QRHRAKIDNY IPIF
```

Residue Number Increasing Mass Decreasing Mass

Start - End	Observed	Mr(expt)	Mr(calc)	Delta	Miss	Sequence
143 - 160	2041.0215	2040.0142	2040.0527	-0.0385	1	AFIITINSFGTELSKEDR (No match)
143 - 160	2041.0215	2040.0142	2040.0527	-0.0385	1	AFIITINSFGTELSKEDR (Ions score 32)
163 - 169	886.4077	885.4004	885.4279	-0.0275	0	LFPHCGR (No match)
170 - 180	1230.6569	1229.6496	1229.6768	-0.0271	0	LYPEGLAQLAR (Ions score 66)
170 - 180	1230.6569	1229.6496	1229.6768	-0.0271	0	LYPEGLAQLAR (No match)
212 - 223	1549.7267	1548.7194	1548.7572	-0.0378	1	TLEDRFFEHEVK (No match)
212 - 223	1549.7267	1548.7194	1548.7572	-0.0378	1	TLEDRFFEHEVK (No match)

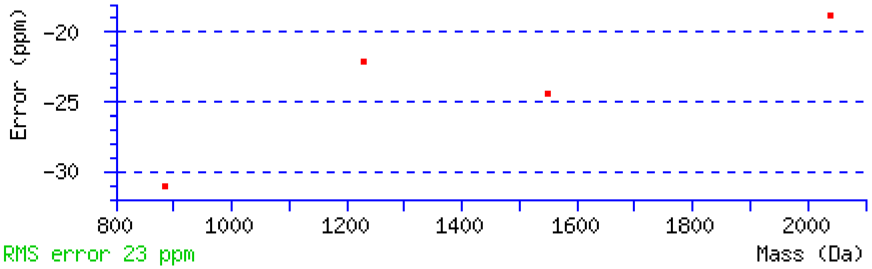
Error (ppm)
-20
-25
-30
800
1000
1200
1400
1600
1800
2000
RMS error 23 ppm
Mass (Da)

Spot 600

Protein View

Match to: **gi|54696354** Score: **120** Expect: **1.9e-007**
protein phosphatase 1, catalytic subunit, beta isoform [Homo sapiens]

Nominal mass (M_r): **37945**; Calculated pI value: **5.84**
NCBI BLAST search of gi|54696354 against nr
Unformatted sequence string for pasting into other applications

Taxonomy: Homo sapiens
Links to retrieve other entries containing this sequence from NCBI Entrez:
gi|61355907 from synthetic construct

Fixed modifications: Carbamidomethyl (C)
Variable modifications: Oxidation (M)
Cleavage by Trypsin: cuts C-term side of KR unless next residue is P
Sequence Coverage: **27%**

Matched peptides shown in **Bold Red**

```
  1 MADGELNVDS LITRLLEVRG CRPGKIVQMT EAEVRGLCIK SREIFLSQPI
 51 PLELEAPLKI CGDIHGQYTD LLRLFEYGGF PPEANYLFLG DYVDRGKQSL
101 ETICLLLAYK IKYPENFFLL RGNHECASIN RIYGFYDECK RRFNIKLWKT
151 FTDCFNCLPI AAIVDEKIFC CHGGLSPDLQ SMEQIRRIMR PTDVPDTGLL
201 CDLLWSDPDK DVQGWGENDR GVSFTFGADV VSKFLNRHDL DLICRAHQVV
251 EDGYEFFAKR QLVTLFSAPN YCGEFDNAGG MMSVDETLMC SFQILKPSEK
301 KAKYQYGGLN SGRPVTPPRT ANPPKKR
```

Residue Number Increasing Mass Decreasing Mass

Start - End	Observed	Mr(expt)	Mr(calc)	Delta	Miss	Sequence
60 - 73	1660.7495	1659.7422	1659.8038	-0.0616	0	ICGDIHGQYTDLLR (No match)
74 - 95	2582.1111	2581.1038	2581.2164	-0.1126	0	LFEYGGFPPEANYLFLGDYVDR (No match)
111 - 121	1439.7457	1438.7384	1438.7972	-0.0588	1	IKYPENFFLLR (No match)
111 - 121	1439.7457	1438.7384	1438.7972	-0.0588	1	IKYPENFFLLR (No match)
234 - 245	1571.7441	1570.7368	1570.8038	-0.0670	1	FLNRHDLDLICR (No match)
246 - 260	1795.8064	1794.7991	1794.8688	-0.0697	1	AHQVVEDGYEFFAKR (No match)
246 - 260	1795.8064	1794.7991	1794.8688	-0.0697	1	AHQVVEDGYEFFAKR (Ions score 23)
304 - 319	1761.8303	1760.8230	1760.8957	-0.0727	0	YQYGGLNSGRPVTPPR (No match)

304 - 319 1761.8303 1760.8230 1760.8957 -0.0727 0 YQYGGLNSGRPVTPPR (Ions score 39)

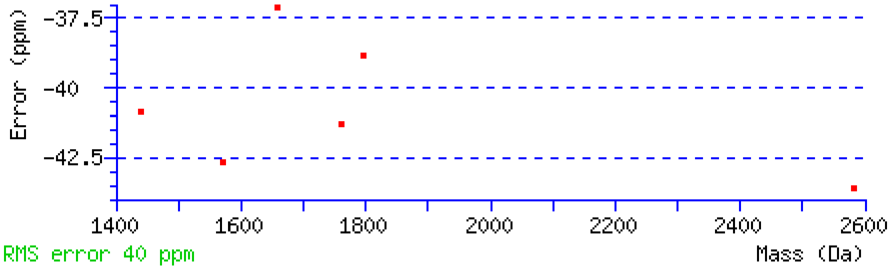

Spot 601

Protein View

Match to: **gi|4506003** Score: **343** Expect: **9.6e-030**
protein phosphatase 1, catalytic subunit, alpha isoform 1 [Homo sapiens]

Nominal mass (M_r): **38229**; Calculated pI value: **5.94**
NCBI BLAST search of gi|4506003 against nr
Unformatted sequence string for pasting into other applications

Taxonomy: Homo sapiens
Links to retrieve other entries containing this sequence from NCBI Entrez:
gi|13928710 from Rattus norvegicus
gi|78369446 from Bos taurus
gi|113205690 from Sus scrofa
gi|49065778 from Rattus norvegicus
gi|49065811 from Homo sapiens
gi|49065813 from Oryctolagus cuniculus
gi|108860895 from Bos taurus
gi|1633403 from Oryctolagus cuniculus
gi|1633404 from Oryctolagus cuniculus
gi|1679 from Oryctolagus cuniculus
gi|35451 from Homo sapiens
gi|190516 from Homo sapiens
gi|220865 from Rattus norvegicus
gi|220867 from Rattus norvegicus
gi|999339 from Rattus sp
gi|12804879 from Homo sapiens
gi|13325343 from Homo sapiens
gi|14124968 from Homo sapiens
gi|30582097 from Homo sapiens
gi|47683037 from Rattus norvegicus
gi|60656413 from synthetic construct
gi|74267665 from Bos taurus
gi|87621715 from Sus scrofa
gi|149061962 from Rattus norvegicus
gi|227433 from Rattus norvegicus
gi|1582003 from Rattus norvegicus

Fixed modifications: Carbamidomethyl (C)
Variable modifications: Oxidation (M)
Cleavage by Trypsin: cuts C-term side of KR unless next residue is P
Sequence Coverage: **43%**

Matched peptides shown in **Bold Red**

```
  1 MSDSEKLNLD SIIGRLLEVQ GSRPGKNVQL TENEIRGLCL KSREIFLSQP
 51 ILLELEAPLK ICGDIHGQYY DLLRLFEYGG FPPESNYLFL GDYVDRGKQS
101 LETICLLLAY KIKYPENFFL LRGNHECASI NRIYGFYDEC KRRYNIKLWK
151 TFTDCFNCLP IAAIVDEKIF CCHGGLSPDL QSMEQIRRIM RPTDVPDQGL
201 LCDLLWSDPD KDVQGWGEND RGVSFTFGAE VVAKFLHKHD LDLICRAHQV
251 VEDGYEFFAK RQLVTLFSAP NYCGEFDNAG AMMSVDETLM CSFQILKPAD
301 KNKGKYGQFS GLNPGGRPIT PPRNSAKAKK
```

Residue Number Increasing Mass Decreasing Mass

Start - End	Observed	Mr(expt)	Mr(calc)	Delta	Miss	Sequence
16 - 26	1183.6344	1182.6271	1182.6720	-0.0449	0	LLEVQGSRPGK (No match)
27 - 36	1215.5881	1214.5808	1214.6255	-0.0446	0	NVQLTENEIR (No match)
42 - 60	2196.1772	2195.1699	2195.2565	-0.0865	1	SREIFLSQPILLELEAPLK (No match)
44 - 60	1953.0571	1952.0498	1952.1233	-0.0735	0	EIFLSQPILLELEAPLK (No match)
61 - 74	1722.7677	1721.7604	1721.8195	-0.0591	0	ICGDIHGQYYDLLR (No match)
75 - 96	2598.1072	2597.0999	2597.2113	-0.1114	0	LFEYGGFPPESNYLFLGDYVDR (No match)
75 - 96	2598.1072	2597.0999	2597.2113	-0.1114	0	LFEYGGFPPESNYLFLGDYVDR (Ions score 72)
112 - 122	1439.7505	1438.7432	1438.7972	-0.0540	1	IKYPENFFLLR (No match)
112 - 122	1439.7505	1438.7432	1438.7972	-0.0540	1	IKYPENFFLLR (Ions score 25)
114 - 122	1198.5808	1197.5735	1197.6182	-0.0447	0	YPENFFLLR (No match)
133 - 142	1350.5691	1349.5618	1349.6074	-0.0455	1	IYGFYDECKR (No match)
235 - 246	1566.7610	1565.7537	1565.8136	-0.0599	1	FLHKHDLDLICR (No match)
247 - 260	1639.7075	1638.7002	1638.7677	-0.0675	0	AHQVVEDGYEFFAK (No match)
247 - 261	1795.8062	1794.7989	1794.8688	-0.0699	1	AHQVVEDGYEFFAKR (Ions score 68)
247 - 261	1795.8062	1794.7989	1794.8688	-0.0699	1	AHQVVEDGYEFFAKR (No match)
304 - 323	2099.0300	2098.0227	2098.1071	-0.0844	1	GKYGQFSGLNPGGRPITPPR (No match)
306 - 323	1913.9269	1912.9196	1912.9907	-0.0711	0	YGQFSGLNPGGRPITPPR (No match)
306 - 323	1913.9269	1912.9196	1912.9907	-0.0711	0	YGQFSGLNPGGRPITPPR (Ions score 13)

Spot 602

Protein View

Match to: **gi|4506003** Score: **127** Expect: **3.8e-008**
protein phosphatase 1, catalytic subunit, alpha isoform 1 [Homo sapiens]

Nominal mass (M_r): **38229**; Calculated pI value: **5.94**
NCBI BLAST search of gi|4506003 against nr
Unformatted sequence string for pasting into other applications

Taxonomy: Homo sapiens
Links to retrieve other entries containing this sequence from NCBI Entrez:
gi|13928710 from Rattus norvegicus
gi|78369446 from Bos taurus
gi|113205690 from Sus scrofa
gi|49065778 from Rattus norvegicus
gi|49065811 from Homo sapiens
gi|49065813 from Oryctolagus cuniculus
gi|108860895 from Bos taurus
gi|1633403 from Oryctolagus cuniculus
gi|1633404 from Oryctolagus cuniculus
gi|1679 from Oryctolagus cuniculus
gi|35451 from Homo sapiens
gi|190516 from Homo sapiens
gi|220865 from Rattus norvegicus
gi|220867 from Rattus norvegicus
gi|999339 from Rattus sp
gi|12804879 from Homo sapiens
gi|13325343 from Homo sapiens
gi|14124968 from Homo sapiens
gi|30582097 from Homo sapiens
gi|47683037 from Rattus norvegicus
gi|60656413 from synthetic construct
gi|74267665 from Bos taurus
gi|87621715 from Sus scrofa
gi|149061962 from Rattus norvegicus
gi|227433 from Rattus norvegicus
gi|1582003 from Rattus norvegicus

Fixed modifications: Carbamidomethyl (C)
Variable modifications: Oxidation (M)
Cleavage by Trypsin: cuts C-term side of KR unless next residue is P
Sequence Coverage: **27%**

Matched peptides shown in **Bold Red**

```
  1 MSDSEKLNLD SIIGRLLEVQ GSRPGKNVQL TENEIRGLCL KSREIFLSQP
 51 ILLELEAPLK ICGDIHGQYY DLLRLFEYGG FPPESNYLFL GDYVDRGKQS
101 LETICLLLAY KIKYPENFFL LRGNHECASI NRIYGFYDEC KRRYNIKLWK
151 TFTDCFNCLP IAAIVDEKIF CCHGGLSPDL QSMEQIRRIM RPTDVPDQGL
201 LCDLLWSDPD KDVQGWGEND RGVSFTFGAE VVAKFLHKHD LDLICRAHQV
251 VEDGYEFFAK RQLVTLFSAP NYCGEFDNAG AMMSVDETLM CSFQILKPAD
301 KNKGKYGQFS GLNPGGRPIT PPRNSAKAKK
```

Residue Number Increasing Mass Decreasing Mass

Start - End	Observed	Mr(expt)	Mr(calc)	Delta	Miss	Sequence
16 - 26	1183.6382	1182.6309	1182.6720	-0.0411	0	LLEVQGSRPGK (No match)
27 - 36	1215.6022	1214.5949	1214.6255	-0.0305	0	NVQLTENEIR (Ions score 16)
27 - 36	1215.6022	1214.5949	1214.6255	-0.0305	0	NVQLTENEIR (No match)
61 - 74	1722.7740	1721.7667	1721.8195	-0.0528	0	ICGDIHGQYYDLLR (Ions score 13)
61 - 74	1722.7740	1721.7667	1721.8195	-0.0528	0	ICGDIHGQYYDLLR (No match)
112 - 122	1439.7589	1438.7516	1438.7972	-0.0456	1	IKYPENFFLLR (Ions score 7)
112 - 122	1439.7589	1438.7516	1438.7972	-0.0456	1	IKYPENFFLLR (No match)
235 - 246	1566.7780	1565.7707	1565.8136	-0.0429	1	FLHKHDLDLICR (No match)
247 - 260	1639.7327	1638.7254	1638.7677	-0.0423	0	AHQVVEDGYEFFAK (No match)
306 - 323	1913.9390	1912.9317	1912.9907	-0.0590	0	YGQFSGLNPGGRPITPPR (No match)
306 - 323	1913.9390	1912.9317	1912.9907	-0.0590	0	YGQFSGLNPGGRPITPPR (Ions score 14)

Spot 603

Protein View

Match to: **gi|4506003** Score: **543** Expect: **9.6e-050**
protein phosphatase 1, catalytic subunit, alpha isoform 1 [Homo sapiens]

Nominal mass (M_r): **38229**; Calculated pI value: **5.94**
NCBI BLAST search of gi|4506003 against nr
Unformatted sequence string for pasting into other applications

Taxonomy: Homo sapiens
Links to retrieve other entries containing this sequence from NCBI Entrez:
gi|13928710 from Rattus norvegicus
gi|78369446 from Bos taurus
gi|113205690 from Sus scrofa
gi|49065778 from Rattus norvegicus
gi|49065811 from Homo sapiens
gi|49065813 from Oryctolagus cuniculus
gi|108860895 from Bos taurus
gi|1633403 from Oryctolagus cuniculus
gi|1633404 from Oryctolagus cuniculus
gi|1679 from Oryctolagus cuniculus
gi|35451 from Homo sapiens
gi|190516 from Homo sapiens
gi|220865 from Rattus norvegicus
gi|220867 from Rattus norvegicus
gi|999339 from Rattus sp
gi|12804879 from Homo sapiens
gi|13325343 from Homo sapiens
gi|14124968 from Homo sapiens
gi|30582097 from Homo sapiens
gi|47683037 from Rattus norvegicus
gi|60656413 from synthetic construct
gi|74267665 from Bos taurus
gi|87621715 from Sus scrofa
gi|149061962 from Rattus norvegicus
gi|227433 from Rattus norvegicus
gi|1582003 from Rattus norvegicus

Fixed modifications: Carbamidomethyl (C)
Variable modifications: Oxidation (M)
Cleavage by Trypsin: cuts C-term side of KR unless next residue is P
Sequence Coverage: **61%**

Matched peptides shown in **Bold Red**

1 MSDSEKLNLD SIIGR**LLEVQ GSRPGKNVQL TENEIR**GLCL K**SREIFLSQP**
51 **ILLELEAPLK ICGDIHGQYY DLLRLFEYGG FPPESNYLFL GDYVDR**GK**QS**
101 **LETICLLLAY KIKYPENFFL LRGNHECASI NRIYGFYDEC KR**RYNIKLWK
151 **TFTDCFNCLP IAAIVDEKIF CCHGGLSPDL QSMEQIR**RIM RPTDVPDQGL
201 LCDLLWSDPD KDVQGWGEND RGVSFTFGAE VVAK**FLHKHD LDLICRAHQV**
251 **VEDGYEFFAK R**QLVTLFSAP NYCGEFDNAG AMMSVDETLM CSFQILKPAD
301 KNKGK**YGQFS GLNPGGRPIT PPR**NSAKAKK

Residue Number Increasing Mass Decreasing Mass

Start - End	Observed	Mr(expt)	Mr(calc)	Delta	Miss	Sequence
16 - 26	1183.6791	1182.6718	1182.6720	-0.0002	0	LLEVQGSRPGK (No match)
27 - 36	1215.6362	1214.6289	1214.6255	0.0035	0	NVQLTENEIR (No match)
42 - 60	2196.2520	2195.2447	2195.2565	-0.0117	1	SREIFLSQPILLELEAPLK (No match)
44 - 60	1953.1224	1952.1151	1952.1233	-0.0082	0	EIFLSQPILLELEAPLK (No match)
61 - 74	1722.8240	1721.8167	1721.8195	-0.0028	0	ICGDIHGQYYDLLR (No match)
75 - 96	2598.2002	2597.1929	2597.2113	-0.0184	0	LFEYGGFPPESNYLFLGDYVDR (Ions score 99)
75 - 96	2598.2002	2597.1929	2597.2113	-0.0184	0	LFEYGGFPPESNYLFLGDYVDR (No match)
99 - 111	1551.8368	1550.8295	1550.8378	-0.0082	0	QSLETICLLLAYK (No match)
112 - 122	1439.8021	1438.7948	1438.7972	-0.0024	1	IKYPENFFLLR (Ions score 72)
112 - 122	1439.8021	1438.7948	1438.7972	-0.0024	1	IKYPENFFLLR (No match)
114 - 122	1198.6256	1197.6183	1197.6182	0.0001	0	YPENFFLLR (No match)
123 - 132	1157.5142	1156.5069	1156.5043	0.0026	0	GNHECASINR (No match)
133 - 141	1194.5098	1193.5025	1193.5063	-0.0037	0	IYGFYDECK (No match)
133 - 142	1350.6139	1349.6066	1349.6074	-0.0007	1	IYGFYDECKR (No match)
151 - 168	2113.9805	2112.9732	2112.9860	-0.0127	0	TFTDCFNCLPIAAIVDEK (No match)
169 - 187	2248.0215	2247.0142	2247.0234	-0.0092	0	IFCCHGGLSPDLQSMEQIR (No match)
235 - 246	1566.8152	1565.8079	1565.8136	-0.0057	1	FLHKHDLDLICR (No match)
235 - 246	1566.8152	1565.8079	1565.8136	-0.0057	1	FLHKHDLDLICR (Ions score 65)
247 - 260	1639.7769	1638.7696	1638.7677	0.0019	0	AHQVVEDGYEFFAK (No match)
247 - 261	1795.8708	1794.8635	1794.8688	-0.0053	1	AHQVVEDGYEFFAKR (No match)
306 - 323	1913.9919	1912.9846	1912.9907	-0.0061	0	YGQFSGLNPGGRPITPPR (Ions score 53)
306 - 323	1913.9919	1912.9846	1912.9907	-0.0061	0	YGQFSGLNPGGRPITPPR (No match)

Spot 604

Protein View

Match to: **gi|4502101** Score: **453** Expect: **9.6e-041**
annexin I [Homo sapiens]

Nominal mass (M_r): **38918**; Calculated pI value: **6.57**
NCBI BLAST search of gi|4502101 against nr
Unformatted sequence string for pasting into other applications

Taxonomy: Homo sapiens
Links to retrieve other entries containing this sequence from NCBI Entrez:
gi|113944 from Homo sapiens
gi|34388 from Homo sapiens
gi|12654863 from Homo sapiens
gi|23958904 from Homo sapiens
gi|47115305 from Homo sapiens
gi|54696654 from Homo sapiens
gi|54696694 from Homo sapiens
gi|54696696 from Homo sapiens
gi|55959292 from Homo sapiens
gi|60654833 from synthetic construct
gi|60819450 from synthetic construct
gi|61356742 from synthetic construct
gi|61356750 from synthetic construct
gi|61356758 from synthetic construct
gi|119582949 from Homo sapiens
gi|119582951 from Homo sapiens
gi|119582953 from Homo sapiens
gi|119582954 from Homo sapiens
gi|123980540 from synthetic construct
gi|123995325 from synthetic construct
gi|224956 from Homo sapiens

Fixed modifications: Carbamidomethyl (C)
Variable modifications: Oxidation (M)
Cleavage by Trypsin: cuts C-term side of KR unless next residue is P
Sequence Coverage: **34%**

Matched peptides shown in **Bold Red**

1 MAMVSEFLKQ AWFIENEEQE YVQTVKSSK**G GPGSAVSPYP TFNPSSDVAA**
51 **LHK**AIMVK**GV DEATIIDILT KR**NNAQRQQI K**AAYLQETGK PLDETLKK**AL
101 TGHLEEVVLA LLK**TPAQFDA DELR**AAMK**GL GTDEDTLIEI LASR**TNKEIR

151 DINRVYREEL KRDLAKDITS DTSGDFRNAL LSLAKGDR**SE DFGVNEDLAD**
201 **SDARALYEAG ER**R**KGTDVNV FNTILTTR**SY PQLRRVFQKY TKYSKHDMNK
251 VLDLELKGDI EKCLTAIVKC ATSKPAFFAE KLHQAMKGVG TRHKALIRIM
301 VSRSEIDMND IKAFYQKMYG ISLCQAILDE TKGDYEKILV ALCGGN

Residue Number Increasing Mass Decreasing Mass

Start - End	Observed	Mr(expt)	Mr(calc)	Delta	Miss	Sequence
30 - 53	2356.1523	2355.1450	2355.1494	-0.0044	0	GGPGSAVSPYPTFNPSSDVAALHK (No match)
30 - 53	2356.1523	2355.1450	2355.1494	-0.0044	0	GGPGSAVSPYPTFNPSSDVAALHK (Ions score 110)
59 - 72	1543.8601	1542.8528	1542.8617	-0.0088	1	GVDEATIIDILTKR (No match)
82 - 98	1905.0208	1904.0135	1904.0254	-0.0119	1	AAYLQETGKPLDETLKK (No match)
114 - 124	1262.5908	1261.5835	1261.5938	-0.0103	0	TPAQFDADELR (No match)
114 - 124	1262.5908	1261.5835	1261.5938	-0.0103	0	TPAQFDADELR (Ions score 30)
129 - 144	1702.8761	1701.8688	1701.8784	-0.0096	0	GLGTDEDTLIEILASR (Ions score 81)
129 - 144	1702.8761	1701.8688	1701.8784	-0.0096	0	GLGTDEDTLIEILASR (No match)
189 - 204	1739.7239	1738.7166	1738.7281	-0.0115	0	SEDFGVNEDLADSDAR (No match)
189 - 204	1739.7239	1738.7166	1738.7281	-0.0115	0	SEDFGVNEDLADSDAR (Ions score 82)
205 - 212	908.4403	907.4330	907.4399	-0.0069	0	ALYEAGER (No match)
214 - 228	1678.9055	1677.8982	1677.9049	-0.0067	1	KGTDVNVFNTILTTR (No match)
215 - 228	1550.8066	1549.7993	1549.8100	-0.0106	0	GTDVNVFNTILTTR (Ions score 23)
215 - 228	1550.8066	1549.7993	1549.8100	-0.0106	0	GTDVNVFNTILTTR (No match)

Spot 605

Protein View

Match to: **gi|42543739** Score: **117** Expect: **3.8e-007**
Chain A, Crystal Structure Of Mo25 In Complex With A C-Terminal Peptide Of Strad

Nominal mass (M_r): **39834**; Calculated pI value: **6.25**
NCBI BLAST search of gi|42543739 against nr
Unformatted sequence string for pasting into other applications

Taxonomy: Homo sapiens
Links to retrieve other entries containing this sequence from NCBI Entrez:
gi|42543741 from Homo sapiens
gi|42543742 from Homo sapiens

Fixed modifications: Carbamidomethyl (C)
Variable modifications: Oxidation (M)
Cleavage by Trypsin: cuts C-term side of KR unless next residue is P
Sequence Coverage: **14%**

Matched peptides shown in **Bold Red**

```
  1 XPFPFGKSHK SPADIVKNLK ESXAVLEKQD ISDKKAEKAT EEVSKNLVAX
 51 KEILYGTNEK EPQTEAVAQL AQELYNSGLL STLVADLQLI DFEGKKDVAQ
101 IFNNILRRQI GTRTPTVEYI CTQQNILFXL LKGYESPEIA LNCGIXLREC
151 IRHEPLAKII LWSEQFYDFF RYVEXSTFDI ASDAFATFKD LLTRHKLLSA
201 EFLEQHYDRF FSEYEKLLHS ENYVTKRQSL KLLGELLLDR HNFTIXTKYI
251 SKPENLKLXX NLLRDKSRNI QFEAFHVFKV FVANPNKTQP ILDILLKNQA
301 KLIEFLSKFQ NDRTEDEQFN DEKTYLVKQI RDLKRPAQQE A
```

Residue Number Increasing Mass Decreasing Mass

Start - End	Observed	Mr(expt)	Mr(calc)	Delta	Miss	Sequence
97 - 107	1302.7039	1301.6966	1301.7091	-0.0125	0	DVAQIFNNILR (No match)
97 - 107	1302.7039	1301.6966	1301.7091	-0.0125	0	DVAQIFNNILR (No match)
197 - 209	1620.7710	1619.7637	1619.7943	-0.0306	0	LLSAEFLEQHYDR (No match)
197 - 209	1620.7710	1619.7637	1619.7943	-0.0306	0	LLSAEFLEQHYDR (Ions score 20)
269 - 279	1379.6836	1378.6763	1378.7033	-0.0270	0	NIQFEAFHVFK (Ions score 36)
269 - 279	1379.6836	1378.6763	1378.7033	-0.0270	0	NIQFEAFHVFK (No match)
309 - 323	1914.7755	1913.7682	1913.8027	-0.0345	1	FQNDRTEDEQFNDEK (Ions score 15)

309 - 323   1914.7755   1913.7682   1913.8027   -0.0345   1   FQNDRTEDEQFNDEK   (No match)

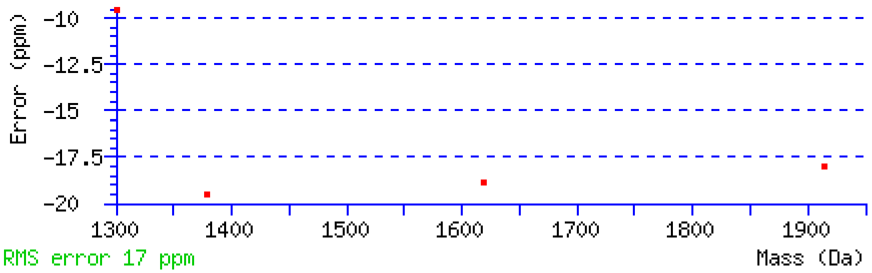

Spot 606

Protein View

Match to: **gi|1584035** Score: **303** Expect: **9.6e-026**
Lasp-1 protein

Nominal mass (M_r): **30185;** Calculated pI value: **6.11**
NCBI BLAST search of gi|1584035 against nr
Unformatted sequence string for pasting into other applications

Taxonomy: Homo sapiens

Fixed modifications: Carbamidomethyl (C)
Variable modifications: Oxidation (M)
Cleavage by Trypsin: cuts C-term side of KR unless next residue is P
Sequence Coverage: **37%**

Matched peptides shown in **Bold Red**

```
  1 MNPNCARCGK IVYPTEKVNC LDKFWHKACF HCETCKMTLN MKNYKGYEKK
 51 PYCNAHYPKQ SFTMVADTPE NLRLKQQSEL QSQVRYKEEF EKNKGKGFSV
101 VADTPELQRI KKTQDQISNI KYHEEFEKSR MGPSGGEGME PERRDSQDGS
151 SYRRPLEQQQ PHHIPTSAPV YQQPQQQPVA QSYGGYKEPA APVSIQRSAP
201 GGGGKRYRAV YDYSAADEDE VSFQDGDTIV NVQQIDDGWM YGTVERTGDT
251 GMLPDNYVED I
```

Residue Number Increasing Mass Decreasing Mass

Start - End	Observed	Mr(expt)	Mr(calc)	Delta	Miss	Sequence
50 - 59	1277.5333	1276.5260	1276.6022	-0.0762	0	KPYCNAHYPK (No match)
60 - 73	1624.6689	1623.6616	1623.7562	-0.0946	0	QSFTMVADTPENLR Oxidation (M) (No match)
60 - 73	1624.6689	1623.6616	1623.7562	-0.0946	0	QSFTMVADTPENLR Oxidation (M) (Ions score 49)
74 - 85	1443.7139	1442.7066	1442.7841	-0.0775	1	LKQQSELQSQVR (No match)
74 - 85	1443.7139	1442.7066	1442.7841	-0.0775	1	LKQQSELQSQVR (Ions score 58)
76 - 85	1202.5466	1201.5393	1201.6051	-0.0657	0	QQSELQSQVR (No match)
86 - 92	972.4075	971.4002	971.4599	-0.0597	1	YKEEFEK (No match)
95 - 109	1603.7601	1602.7528	1602.8365	-0.0837	1	GKGFSVVADTPELQR (Ions score 15)
95 - 109	1603.7601	1602.7528	1602.8365	-0.0837	1	GKGFSVVADTPELQR (No match)
97 - 109	1418.6489	1417.6416	1417.7201	-0.0785	0	GFSVVADTPELQR (No match)
97 - 109	1418.6489	1417.6416	1417.7201	-0.0785	0	GFSVVADTPELQR (Ions score 43)

122 - 128	981.3732	980.3659	980.4239	-0.0580	0	YHEEFEK (No match)
131 - 144	1521.5555	1520.5482	1520.6347	-0.0865	1	MGPSGGEGMEPERR 2 Oxidation (M) (No match)
131 - 144	1521.5555	1520.5482	1520.6347	-0.0865	1	MGPSGGEGMEPERR 2 Oxidation (M) (Ions score 21)
145 - 153	1014.3590	1013.3517	1013.4050	-0.0533	0	DSQDGSSYR (No match)
188 - 197	1067.5231	1066.5158	1066.5771	-0.0612	0	EPAAPVSIQR (No match)

Spot 607

Protein View

Match to: **gi|31645** Score: **552** Expect: **1.2e-050**
glyceraldehyde-3-phosphate dehydrogenase [Homo sapiens]

Nominal mass (M_r): **36202;** Calculated pI value: **8.26**
NCBI BLAST search of gi|31645 against nr
Unformatted sequence string for pasting into other applications

Taxonomy: Homo sapiens

Fixed modifications: Carbamidomethyl (C)
Variable modifications: Oxidation (M)
Cleavage by Trypsin: cuts C-term side of KR unless next residue is P
Sequence Coverage: **48%**

Matched peptides shown in **Bold Red**

```
  1 MGKVKVGVNG FGRIGRLVTR AAFNSGKVDI VAINDPFIDL NYMVYMFQYD
 51 STHGKFHGTV KAENGKLVIN GNPITIFQER DPSKIKWGDA GAEYVVESTG
101 VFTTMEKAGA HLQGGAKRVI ISAPSADAPM FVMGVNHEKY DNSLKIISNA
151 SCTTNCLAPL AKVIHDNFGI VEGLMTTVHA ITATQKTVDG PSGKLWRDGR
201 GALQNIIPAS TGAAKAVGKV IPELDGKLTG MAFRVPTANV SVVDLTCRLE
251 KPAKYDDIKK VVKQASEGPL KGILGYTEHQ VVSSDFNSDT HSSTFDAGAG
301 IALNDHFVKL ISWYDNEFGY SNRVVDLMAH MASKE
```

Residue Number Increasing Mass Decreasing Mass

Start - End	Observed	Mr(expt)	Mr(calc)	Delta	Miss	Sequence
4 - 13	1032.5575	1031.5502	1031.5875	-0.0373	1	VKVGVNGFGR (No match)
6 - 13	805.3982	804.3909	804.4241	-0.0332	0	VGVNGFGR (No match)
62 - 80	2113.0566	2112.0493	2112.1326	-0.0833	1	AENGKLVINGNPITIFQER (No match)
67 - 80	1613.8416	1612.8343	1612.8936	-0.0593	0	LVINGNPITIFQER (Ions score 81)
67 - 80	1613.8416	1612.8343	1612.8936	-0.0593	0	LVINGNPITIFQER (No match)
67 - 84	2041.0291	2040.0218	2040.1003	-0.0785	1	LVINGNPITIFQERDPSK (Ions score 60)
67 - 84	2041.0291	2040.0218	2040.1003	-0.0785	1	LVINGNPITIFQERDPSK (No match)
108 - 117	909.4528	908.4455	908.4827	-0.0372	0	AGAHLQGGAK (No match)
108 - 118	1065.5549	1064.5476	1064.5838	-0.0362	1	AGAHLQGGAKR (No match)
118 - 139	2401.1111	2400.1038	2400.1929	-0.0891	1	RVIISAPSADAPMFVMGVNHEK 2 Oxidation (M) (No match)

119 - 139	2245.0210	2244.0137	2244.0918	-0.0781	0	VIISAPSADAPMFVMGVNHEK	2 Oxidation (M) (No match)
146 - 162	1833.8538	1832.8465	1832.9124	-0.0659	0	IISNASCTTNCLAPLAK	(No match)
163 - 186	2611.2556	2610.2483	2610.3475	-0.0992	0	VIHDNFGIVEGLMTTVHAITATQK	Oxidation (M) (No match)
201 - 215	1411.7325	1410.7252	1410.7830	-0.0578	0	GALQNIIPASTGAAK	(No match)
220 - 234	1662.8247	1661.8174	1661.8810	-0.0636	1	VIPELDGKLTGMAFR	Oxidation (M) (No match)
228 - 234	811.3754	810.3681	810.4058	-0.0376	0	LTGMAFR	Oxidation (M) (No match)
235 - 248	1530.7382	1529.7309	1529.7871	-0.0562	0	VPTANVSVVDLTCR	(Ions score 117)
235 - 248	1530.7382	1529.7309	1529.7871	-0.0562	0	VPTANVSVVDLTCR	(No match)
310 - 323	1763.7372	1762.7299	1762.7950	-0.0651	0	LISWYDNEFGYSNR	(Ions score 96)
310 - 323	1763.7372	1762.7299	1762.7950	-0.0651	0	LISWYDNEFGYSNR	(No match)

Spot 608

Protein View

Match to: **gi|67464043** Score: **508** Expect: **3e-046**
Chain O, Crsytal Structure Of Human Liver Gapdh

Nominal mass (M_r): **36483**; Calculated pI value: **8.58**
NCBI BLAST search of gi|67464043 against nr
Unformatted sequence string for pasting into other applications

Taxonomy: Homo sapiens
Links to retrieve other entries containing this sequence from NCBI Entrez:
gi|67464044 from Homo sapiens
gi|67464045 from Homo sapiens
gi|67464046 from Homo sapiens

Fixed modifications: Carbamidomethyl (C)
Variable modifications: Oxidation (M)
Cleavage by Trypsin: cuts C-term side of KR unless next residue is P
Sequence Coverage: **46%**

Matched peptides shown in **Bold Red**

```
  1 GSHMGKVKVG VNGFGRIGRL VTRAAFNSGK VDIVAINDPF IDLNYMVYMF
 51 QYDSTHGKFH GTVKAENGKL VINGNPITIF QERDPSKIKW GDAGAEYVVE
101 STGVFTTMEK AGAHLQGGAK RVIISAPSAD APMFVMGVNH EKYDNSLKII
151 SNASCTTNCL APLAKVIHDN FGIVEGLMTT VHAITATQKT VDGPSGKLWR
201 DGRGALQNII PASTGAAKAV GKVIPELNGK LTGMAFRVPT ANVSVVDLTC
251 RLEKPAKYDD IKKVVKQASE GPLKGILGYT EHQVVSSDFN SDTHSSTFDA
301 GAGIALNDHF VKLISWYDNE FGYSNRVVDL MAHMASKE
```

Residue Number Increasing Mass Decreasing Mass

Start - End	Observed	Mr(expt)	Mr(calc)	Delta	Miss	Sequence
9 - 16	805.4125	804.4052	804.4241	-0.0189	0	VGVNGFGR (No match)
70 - 83	1613.8540	1612.8467	1612.8936	-0.0469	0	LVINGNPITIFQER (No match)
70 - 83	1613.8540	1612.8467	1612.8936	-0.0469	0	LVINGNPITIFQER (Ions score 53)
70 - 87	2041.0441	2040.0368	2040.1003	-0.0635	1	LVINGNPITIFQERDPSK (No match)
70 - 87	2041.0441	2040.0368	2040.1003	-0.0635	1	LVINGNPITIFQERDPSK (Ions score 73)
111 - 121	1065.5669	1064.5596	1064.5838	-0.0242	1	AGAHLQGGAKR (No match)

122 - 142	2229.0566	2228.0493	2228.0969	-0.0476	0	**VIISAPSADAPMFVMGVNHEK** Oxidation (M) (No match)
122 - 142	2245.0505	2244.0432	2244.0918	-0.0486	0	**VIISAPSADAPMFVMGVNHEK** 2 Oxidation (M) (No match)
149 - 165	1833.8676	1832.8603	1832.9124	-0.0521	0	**IISNASCTTNCLAPLAK** (No match)
166 - 189	2595.3003	2594.2930	2594.3526	-0.0596	0	**VIHDNFGIVEGLMTTVHAITATQK** (No match)
166 - 189	2611.3040	2610.2967	2610.3475	-0.0508	0	**VIHDNFGIVEGLMTTVHAITATQK** Oxidation (M) (No match)
204 - 218	1411.7461	1410.7388	1410.7830	-0.0442	0	**GALQNIIPASTGAAK** (No match)
223 - 230	869.5135	868.5062	868.5018	0.0045	0	**VIPELNGK** (No match)
231 - 237	795.4030	794.3957	794.4109	-0.0151	0	**LTGMAFR** (No match)
231 - 237	811.3901	810.3828	810.4058	-0.0229	0	**LTGMAFR** Oxidation (M) (No match)
238 - 251	1530.7495	1529.7422	1529.7871	-0.0449	0	**VPTANVSVVDLTCR** (No match)
238 - 251	1530.7495	1529.7422	1529.7871	-0.0449	0	**VPTANVSVVDLTCR** (Ions score 113)
313 - 326	1763.7490	1762.7417	1762.7950	-0.0533	0	**LISWYDNEFGYSNR** (Ions score 118)
313 - 326	1763.7490	1762.7417	1762.7950	-0.0533	0	**LISWYDNEFGYSNR** (No match)

Spot 609

Protein View

Match to: **gi|67464043** Score: **567** Expect: **3.8e-052**
Chain O, Crsytal Structure Of Human Liver Gapdh

Nominal mass (M_r): **36483**; Calculated pI value: **8.58**
NCBI BLAST search of gi|67464043 against nr
Unformatted sequence string for pasting into other applications

Taxonomy: Homo sapiens
Links to retrieve other entries containing this sequence from NCBI Entrez:
gi|67464044 from Homo sapiens
gi|67464045 from Homo sapiens
gi|67464046 from Homo sapiens

Fixed modifications: Carbamidomethyl (C)
Variable modifications: Oxidation (M)
Cleavage by Trypsin: cuts C-term side of KR unless next residue is P
Sequence Coverage: **47%**

Matched peptides shown in **Bold Red**

```
  1 GSHMGKVKVG VNGFGRIGRL VTRAAFNSGK VDIVAINDPF IDLNYMVYMF
 51 QYDSTHGKFH GTVKAENGKL VINGNPITIF QERDPSKIKW GDAGAEYVVE
101 STGVFTTMEK AGAHLQGGAK RVIISAPSAD APMFVMGVNH EKYDNSLKII
151 SNASCTTNCL APLAKVIHDN FGIVEGLMTT VHAITATQKT VDGPSGKLWR
201 DGRGALQNII PASTGAAKAV GKVIPELNGK LTGMAFRVPT ANVSVVDLTC
251 RLEKPAKYDD IKKVVKQASE GPLKGILGYT EHQVVSSDFN SDTHSSTFDA
301 GAGIALNDHF VKLISWYDNE FGYSNRVVDL MAHMASKE
```

Residue Number Increasing Mass Decreasing Mass

Start - End	Observed	Mr(expt)	Mr(calc)	Delta	Miss	Sequence
7 - 16	1032.5883	1031.5810	1031.5875	-0.0065	1	VKVGVNGFGR (No match)
9 - 16	805.4246	804.4173	804.4241	-0.0068	0	VGVNGFGR (No match)
70 - 83	1613.8763	1612.8690	1612.8936	-0.0246	0	LVINGNPITIFQER (Ions score 78)
70 - 83	1613.8763	1612.8690	1612.8936	-0.0246	0	LVINGNPITIFQER (No match)
70 - 87	2041.0759	2040.0686	2040.1003	-0.0317	1	LVINGNPITIFQERDPSK (No match)
70 - 87	2041.0759	2040.0686	2040.1003	-0.0317	1	LVINGNPITIFQERDPSK (Ions score 88)

111 - 120	909.4757	908.4684	908.4827	-0.0143	0	**AGAHLQGGAK** (No match)
111 - 121	1065.5781	1064.5708	1064.5838	-0.0130	1	**AGAHLQGGAKR** (No match)
121 - 142	2369.1790	2368.1717	2368.2031	-0.0314	1	**RVIISAPSADAPMFVMGVNHEK** (No match)
121 - 142	2385.1934	2384.1861	2384.1980	-0.0119	1	**RVIISAPSADAPMFVMGVNHEK** Oxidation (M) (No match)
122 - 142	2213.0933	2212.0860	2212.1020	-0.0159	0	**VIISAPSADAPMFVMGVNHEK** (No match)
122 - 142	2229.0901	2228.0828	2228.0969	-0.0141	0	**VIISAPSADAPMFVMGVNHEK** Oxidation (M) (No match)
122 - 142	2245.0989	2244.0916	2244.0918	-0.0002	0	**VIISAPSADAPMFVMGVNHEK** 2 Oxidation (M) (No match)
149 - 165	1833.8986	1832.8913	1832.9124	-0.0211	0	**IISNASCTTNCLAPLAK** (No match)
166 - 189	2595.3462	2594.3389	2594.3526	-0.0137	0	**VIHDNFGIVEGLMTTVHAITATQK** (No match)
166 - 189	2611.3616	2610.3543	2610.3475	0.0068	0	**VIHDNFGIVEGLMTTVHAITATQK** Oxidation (M) (No match)
204 - 218	1411.7684	1410.7611	1410.7830	-0.0219	0	**GALQNIIPASTGAAK** (No match)
223 - 230	869.5118	868.5045	868.5018	0.0028	0	**VIPELNGK** (No match)
231 - 237	795.4120	794.4047	794.4109	-0.0061	0	**LTGMAFR** (No match)
231 - 237	811.4027	810.3954	810.4058	-0.0103	0	**LTGMAFR** Oxidation (M) (No match)
238 - 251	1530.7708	1529.7635	1529.7871	-0.0236	0	**VPTANVSVVDLTCR** (Ions score 122)
238 - 251	1530.7708	1529.7635	1529.7871	-0.0236	0	**VPTANVSVVDLTCR** (No match)
313 - 326	1763.7743	1762.7670	1762.7950	-0.0280	0	**LISWYDNEFGYSNR** (No match)
313 - 326	1763.7743	1762.7670	1762.7950	-0.0280	0	**LISWYDNEFGYSNR** (Ions score 103)

Spot 610

Protein View

Match to: **gi|31645** Score: **667** Expect: **3.8e-062**
glyceraldehyde-3-phosphate dehydrogenase [Homo sapiens]

Nominal mass (M_r): **36202**; Calculated pI value: **8.26**
NCBI BLAST search of gi|31645 against nr
Unformatted sequence string for pasting into other applications

Taxonomy: Homo sapiens

Fixed modifications: Carbamidomethyl (C)
Variable modifications: Oxidation (M)
Cleavage by Trypsin: cuts C-term side of KR unless next residue is P
Sequence Coverage: **50%**

Matched peptides shown in **Bold Red**

```
  1 MGKVKVGVNG FGRIGRLVTR AAFNSGKVDI VAINDPFIDL NYMVYMFQYD
 51 STHGKFHGTV KAENGKLVIN GNPITIFQER DPSKIKWGDA GAEYVVESTG
101 VFTTMEKAGA HLQGGAKRVI ISAPSADAPM FVMGVNHEKY DNSLKIISNA
151 SCTTNCLAPL AKVIHDNFGI VEGLMTTVHA ITATQKTVDG PSGKLWRDGR
201 GALQNIIPAS TGAAKAVGKV IPELDGKLTG MAFRVPTANV SVVDLTCRLE
251 KPAKYDDIKK VVKQASEGPL KGILGYTEHQ VVSSDFNSDT HSSTFDAGAG
301 IALNDHFVKL ISWYDNEFGY SNRVVDLMAH MASKE
```

Residue Number Increasing Mass Decreasing Mass

Start - End	Observed	Mr(expt)	Mr(calc)	Delta	Miss	Sequence
4 - 13	1032.6122	1031.6049	1031.5875	0.0174	1	VKVGVNGFGR (No match)
6 - 13	805.4421	804.4348	804.4241	0.0107	0	VGVNGFGR (No match)
62 - 80	2113.1616	2112.1543	2112.1326	0.0217	1	AENGKLVINGNPITIFQER (No match)
67 - 80	1613.9227	1612.9154	1612.8936	0.0218	0	LVINGNPITIFQER (Ions score 70)
67 - 80	1613.9227	1612.9154	1612.8936	0.0218	0	LVINGNPITIFQER (No match)
67 - 84	2041.1313	2040.1240	2040.1003	0.0237	1	LVINGNPITIFQERDPSK (Ions score 88)
67 - 84	2041.1313	2040.1240	2040.1003	0.0237	1	LVINGNPITIFQERDPSK (No match)
108 - 117	909.4988	908.4915	908.4827	0.0088	0	AGAHLQGGAK (No match)
118 - 139	2369.2437	2368.2364	2368.2031	0.0333	1	RVIISAPSADAPMFVMGVNHEK (No match)
119 - 139	2213.1433	2212.1360	2212.1020	0.0340	0	VIISAPSADAPMFVMGVNHEK (No match)

119 - 139	2229.1406	2228.1333	2228.0969	0.0364	0	VIISAPSADAPMFVMGVNHEK	Oxidation (M) (No match)
146 - 162	1833.9469	1832.9396	1832.9124	0.0272	0	IISNASCTTNCLAPLAK	(No match)
163 - 186	2595.3994	2594.3921	2594.3526	0.0395	0	VIHDNFGIVEGLMTTVHAITATQK	(Ions score 97)
163 - 186	2595.3994	2594.3921	2594.3526	0.0395	0	VIHDNFGIVEGLMTTVHAITATQK	(No match)
163 - 186	2611.3999	2610.3926	2610.3475	0.0451	0	VIHDNFGIVEGLMTTVHAITATQK	Oxidation (M) (No match)
201 - 215	1411.8109	1410.8036	1410.7830	0.0206	0	GALQNIIPASTGAAK	(No match)
220 - 234	1646.9211	1645.9138	1645.8861	0.0277	1	VIPELDGKLTGMAFR	(No match)
220 - 234	1662.9095	1661.9022	1661.8810	0.0212	1	VIPELDGKLTGMAFR	Oxidation (M) (No match)
228 - 234	811.4207	810.4134	810.4058	0.0077	0	LTGMAFR	Oxidation (M) (No match)
235 - 248	1530.8160	1529.8087	1529.7871	0.0216	0	VPTANVSVVDLTCR	(Ions score 103)
235 - 248	1530.8160	1529.8087	1529.7871	0.0216	0	VPTANVSVVDLTCR	(No match)
235 - 254	2197.2217	2196.2144	2196.1936	0.0209	1	VPTANVSVVDLTCRLEKPAK	(No match)
310 - 323	1763.8250	1762.8177	1762.7950	0.0227	0	LISWYDNEFGYSNR	(No match)
310 - 323	1763.8250	1762.8177	1762.7950	0.0227	0	LISWYDNEFGYSNR	(Ions score 96)

Spot 611

Protein View

Match to: **gi|5453597** Score: **709** Expect: **2.4e-066**
F-actin capping protein alpha-1 subunit [Homo sapiens]

Nominal mass (M_r): **33073;** Calculated pI value: **5.45**
NCBI BLAST search of gi|5453597 against nr
Unformatted sequence string for pasting into other applications

Taxonomy: Homo sapiens
Links to retrieve other entries containing this sequence from NCBI Entrez:
gi|1705650 from Homo sapiens
gi|1336099 from Homo sapiens
gi|12652785 from Homo sapiens
gi|47115251 from Homo sapiens
gi|49456595 from Homo sapiens
gi|54695598 from Homo sapiens
gi|55962160 from Homo sapiens
gi|55962895 from Homo sapiens
gi|60655007 from synthetic construct
gi|60821852 from synthetic construct
gi|61355548 from synthetic construct
gi|119576929 from Homo sapiens
gi|119576930 from Homo sapiens
gi|123979702 from synthetic construct
gi|123994487 from synthetic construct

Fixed modifications: Carbamidomethyl (C)
Variable modifications: Oxidation (M)
Cleavage by Trypsin: cuts C-term side of KR unless next residue is P
Sequence Coverage: **54%**

Matched peptides shown in **Bold Red**

```
  1 MADFDDRVSD EEKVRIAAKF ITHAPPGEFN EVFNDVRLLL NNDNLLREGA
 51 AHAFAQYNMD QFTPVKIEGY EDQVLITEHG DLGNSRFLDP RNKISFKFDH
101 LRKEASDPQP EEADGGLKSW RESCDSALRA YVKDHYSNGF CTVYAKTIDG
151 QQTIIACIES HQFQPKNFWN GRWRSEWKFT ITPPTAQVVG VLKIQVHYYE
201 DGNVQLVSHK DVQDSLTVSN EAQTAKEFIK IIENAENEYQ TAISENYQTM
251 SDTTFKALRR QLPVTRTKID WNKILSYKIG KEMQNA
```

Residue Number Increasing Mass Decreasing Mass

Start - End	Observed	Mr(expt)	Mr(calc)	Delta	Miss	Sequence
20 - 37	2089.0120	2088.0047	2088.0064	-0.0017	0	FITHAPPGEFNEVFNDVR (Ions score 122)
20 - 37	2089.0120	2088.0047	2088.0064	-0.0017	0	FITHAPPGEFNEVFNDVR (No match)
38 - 47	1197.6942	1196.6869	1196.6877	-0.0007	0	LLLNNDNLLR (No match)
38 - 47	1197.6942	1196.6869	1196.6877	-0.0007	0	LLLNNDNLLR (Ions score 62)
48 - 66	2140.9873	2139.9800	2139.9683	0.0117	0	EGAAHAFAQYNMDQFTPVK Oxidation (M) (Ions score 91)
48 - 66	2140.9873	2139.9800	2139.9683	0.0117	0	EGAAHAFAQYNMDQFTPVK Oxidation (M) (No match)
67 - 86	2245.0767	2244.0694	2244.0658	0.0037	0	IEGYEDQVLITEHGDLGNSR (Ions score 154)
67 - 86	2245.0767	2244.0694	2244.0658	0.0037	0	IEGYEDQVLITEHGDLGNSR (No match)
104 - 118	1542.6860	1541.6787	1541.6845	-0.0058	0	EASDPQPEEADGGLK (No match)
122 - 129	937.4093	936.4020	936.3971	0.0050	0	ESCDSALR (No match)
134 - 146	1561.6716	1560.6643	1560.6667	-0.0023	0	DHYSNGFCTVYAK (No match)
147 - 166	2314.1624	2313.1551	2313.1423	0.0128	0	TIDGQQTIIACIESHQFQPK (No match)
147 - 166	2314.1624	2313.1551	2313.1423	0.0128	0	TIDGQQTIIACIESHQFQPK (Ions score 117)
179 - 193	1570.9144	1569.9071	1569.9130	-0.0058	0	FTITPPTAQVVGVLK (No match)
194 - 210	2029.0150	2028.0077	2028.0064	0.0013	0	IQVHYYEDGNVQLVSHK (No match)

Spot 612

Protein View

Match to: **gi|1905874** Score: **185** Expect: **6.1e-014**
carboxyl terminal LIM domain protein [Homo sapiens]

Nominal mass (M_r): **36604**; Calculated pI value: **6.80**
NCBI BLAST search of gi|1905874 against nr
Unformatted sequence string for pasting into other applications

Taxonomy: Homo sapiens

Fixed modifications: Carbamidomethyl (C)
Variable modifications: Oxidation (M)
Cleavage by Trypsin: cuts C-term side of KR unless next residue is P
Sequence Coverage: **23%**

Matched peptides shown in **Bold Red**

```
  1 MTTQQIDLQG PGPWGFRLVG RKDFEQPLAI SRVTPGSKAA LANLCIGDVI
 51 TAIDGENTSN MTHLEAQNRI KGCTDNLTLT VARSEHKVWS PLVTEEGKRH
101 PYKMNLASEP QEVLHIGSAH NRSAMPFTAS PASSTTARVI TNQYNNPAGL
151 YSSENISNFN NALESKTAAS GVEANSRPLD HAQPPSSLVI DKESEVYKML
201 QEKQELNEPP KQSTSFLVLQ EILESEEKGD PNKPSGFRSV KAPVTKVAAS
251 IGNAQKLPMC DKCGTGIVGV FVKLRDRHRH PECYVCTDCG TNLKQKGHFF
301 VEDQIYCEKH ARERVTPPEG YEVVTVFPK
```

Residue Number Increasing Mass Decreasing Mass

Start - End	Observed	Mr(expt)	Mr(calc)	Delta	Miss	Sequence
23 - 32	1175.6106	1174.6033	1174.5982	0.0051	0	DFEQPLAISR (No match)
23 - 32	1175.6106	1174.6033	1174.5982	0.0051	0	DFEQPLAISR (Ions score 17)
104 - 122	2119.0273	2118.0200	2118.0276	-0.0075	0	MNLASEPQEVLHIGSAHNR Oxidation (M) (Ions score 19)
104 - 122	2119.0273	2118.0200	2118.0276	-0.0075	0	MNLASEPQEVLHIGSAHNR Oxidation (M) (No match)
278 - 294	2146.9075	2145.9002	2145.9142	-0.0140	1	HRHPECYVCTDCGTNLK (Ions score 7)
278 - 294	2146.9075	2145.9002	2145.9142	-0.0140	1	HRHPECYVCTDCGTNLK (No match)
297 - 309	1671.7410	1670.7337	1670.7398	-0.0061	0	GHFFVEDQIYCEK (Ions score 65)
297 - 309	1671.7410	1670.7337	1670.7398	-0.0061	0	GHFFVEDQIYCEK (No match)
313 - 329	1947.0122	1946.0049	1946.0148	-0.0099	1	ERVTPPEGYEVVTVFPK (No match)
315 - 329	1661.8689	1660.8616	1660.8711	-0.0095	0	VTPPEGYEVVTVFPK (No match)

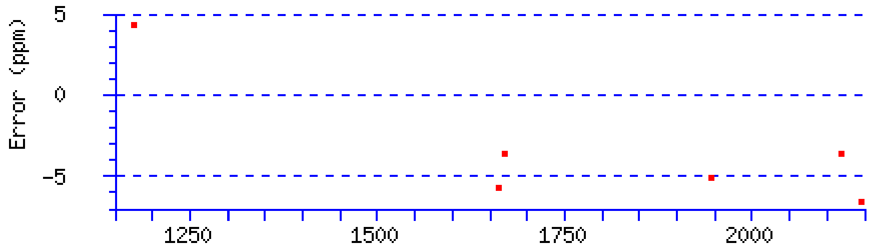

RMS error 4 ppm

Spot 615

Protein View

Match to: **gi|4502101** Score: **668** Expect: **3e-062**
annexin I [Homo sapiens]

Nominal mass (M_r): **38918**; Calculated pI value: **6.57**
NCBI BLAST search of gi|4502101 against nr
Unformatted sequence string for pasting into other applications

Taxonomy: Homo sapiens
Links to retrieve other entries containing this sequence from NCBI Entrez:
gi|113944 from Homo sapiens
gi|34388 from Homo sapiens
gi|12654863 from Homo sapiens
gi|23958904 from Homo sapiens
gi|47115305 from Homo sapiens
gi|54696654 from Homo sapiens
gi|54696694 from Homo sapiens
gi|54696696 from Homo sapiens
gi|55959292 from Homo sapiens
gi|60654833 from synthetic construct
gi|60819450 from synthetic construct
gi|61356742 from synthetic construct
gi|61356750 from synthetic construct
gi|61356758 from synthetic construct
gi|119582949 from Homo sapiens
gi|119582951 from Homo sapiens
gi|119582953 from Homo sapiens
gi|119582954 from Homo sapiens
gi|123980540 from synthetic construct
gi|123995325 from synthetic construct
gi|224956 from Homo sapiens

Fixed modifications: Carbamidomethyl (C)
Variable modifications: Oxidation (M)
Cleavage by Trypsin: cuts C-term side of KR unless next residue is P
Sequence Coverage: **51%**

Matched peptides shown in **Bold Red**

1 MAMVSEFLKQ AWFIENEEQE YVQTVKSSK**G GPGSAVSPYP TFNPSSDVAA**
51 **LHK**AIMVK**GV DEATIIDILT KR**NNAQRQQI K**AAYLQETGK PLDETLKKAL**
101 **TGHLEEVVLA LLKTPAQFDA DELR**AAMK**GL GTDEDTLIEI LASR**TNKEIR

```
151 DINRVYREEL KRDLAKDITS DTSGDFRNAL LSLAKGDRSE DFGVNEDLAD
201 SDARALYEAG ERRKGTDVNV FNTILTTRSY PQLRRVFQKY TKYSKHDMNK
251 VLDLELKGDI EKCLTAIVKC ATSKPAFFAE KLHQAMKGVG TRHKALIRIM
301 VSRSEIDMND IKAFYQKMYG ISLCQAILDE TKGDYEKILV ALCGGN
```

Residue Number Increasing Mass Decreasing Mass

Start - End	Observed	Mr(expt)	Mr(calc)	Delta	Miss	Sequence
30 - 53	2356.0520	2355.0447	2355.1494	-0.1047	0	GGPGSAVSPYPTFNPSSDVAALHK (No match)
59 - 71	1387.7078	1386.7005	1386.7606	-0.0600	0	GVDEATIIDILTK (No match)
59 - 72	1543.8066	1542.7993	1542.8617	-0.0623	1	GVDEATIIDILTKR (No match)
59 - 72	1543.8066	1542.7993	1542.8617	-0.0623	1	GVDEATIIDILTKR (Ions score 90)
82 - 98	1904.9552	1903.9479	1904.0254	-0.0775	1	AAYLQETGKPLDETLKK (No match)
82 - 98	1904.9552	1903.9479	1904.0254	-0.0775	1	AAYLQETGKPLDETLKK (Ions score 75)
99 - 113	1605.8868	1604.8795	1604.9501	-0.0705	0	ALTGHLEEVVLALLK (No match)
114 - 124	1262.5463	1261.5390	1261.5938	-0.0548	0	TPAQFDADELR (Ions score 96)
114 - 124	1262.5463	1261.5390	1261.5938	-0.0548	0	TPAQFDADELR (No match)
129 - 144	1702.8154	1701.8081	1701.8784	-0.0703	0	GLGTDEDTLIEILASR (No match)
129 - 144	1702.8154	1701.8081	1701.8784	-0.0703	0	GLGTDEDTLIEILASR (Ions score 109)
167 - 177	1213.4835	1212.4762	1212.5258	-0.0496	0	DITSDTSGDFR (No match)
186 - 204	2067.7971	2066.7898	2066.8776	-0.0878	1	GDRSEDFGVNEDLADSDAR (No match)
189 - 204	1739.6628	1738.6555	1738.7281	-0.0726	0	SEDFGVNEDLADSDAR (Ions score 101)
189 - 204	1739.6628	1738.6555	1738.7281	-0.0726	0	SEDFGVNEDLADSDAR (No match)
205 - 212	908.4076	907.4003	907.4399	-0.0396	0	ALYEAGER (No match)
214 - 228	1678.8453	1677.8380	1677.9049	-0.0669	1	KGTDVNVFNTILTTR (No match)
215 - 228	1550.7538	1549.7465	1549.8100	-0.0634	0	GTDVNVFNTILTTR (No match)
229 - 235	919.4670	918.4597	918.5035	-0.0438	1	SYPQLRR (No match)
270 - 281	1356.5912	1355.5839	1355.6543	-0.0704	0	CATSKPAFFAEK (No match)
304 - 312	1064.5027	1063.4954	1063.4856	0.0099	0	SEIDMNDIK (No match)

Spot 616

Protein View

Match to: **gi|119603918** Score: **398** Expect: **3e-035**
capping protein (actin filament) muscle Z-line, alpha 2, isoform CRA_a [Homo sapiens]

Nominal mass (M_r): **31898**; Calculated pI value: **6.46**
NCBI BLAST search of gi|119603918 against nr
Unformatted sequence string for pasting into other applications

Taxonomy: Homo sapiens

Fixed modifications: Carbamidomethyl (C)
Variable modifications: Oxidation (M)
Cleavage by Trypsin: cuts C-term side of KR unless next residue is P
Sequence Coverage: **41%**

Matched peptides shown in **Bold Red**

```
  1 MKVRIAAKFI IHAPPGEFNE VFNDVRLLLN NDNLLREGAA HAFAQYNLDQ
 51 FTPVKIEGYE DQVLITEHGD LGNGKFLDPK NRICFKFDHL RKEATDPRPC
101 EVENAVESWR TSVETALRAY VKEHYPNGVC TVYGKKIDGQ QTIIACIESH
151 QFQAKNFWNG RWRSEWKFTI TPSTTQVVGI LKIQVHYYED GNVQLVSHKD
201 IQDSLTVSNE VQTAKEFIKI VEAAENEYQT AISENYQTMS DTTFKALRRQ
251 LPVTRTKIDW NKILSYKIGK EMQNA
```

Residue Number Increasing Mass Decreasing Mass

Start - End	Observed	Mr(expt)	Mr(calc)	Delta	Miss	Sequence
9 - 26	2100.9954	2099.9881	2100.0428	-0.0546	0	FIIHAPPGEFNEVFNDVR (Ions score 100)
9 - 26	2100.9954	2099.9881	2100.0428	-0.0546	0	FIIHAPPGEFNEVFNDVR (No match)
27 - 36	1197.6771	1196.6698	1196.6877	-0.0178	0	LLLNNDNLLR (No match)
27 - 36	1197.6771	1196.6698	1196.6877	-0.0178	0	LLLNNDNLLR (Ions score 42)
56 - 75	2187.0212	2186.0139	2186.0490	-0.0351	0	IEGYEDQVLITEHGDLGNGK (No match)
123 - 135	1523.6580	1522.6507	1522.6874	-0.0367	0	EHYPNGVCTVYGK (Ions score 48)
123 - 135	1523.6580	1522.6507	1522.6874	-0.0367	0	EHYPNGVCTVYGK (No match)
136 - 155	2315.1233	2314.1160	2314.1739	-0.0579	1	KIDGQQTIIACIESHQFQAK (Ions score 105)
136 - 155	2315.1233	2314.1160	2314.1739	-0.0579	1	KIDGQQTIIACIESHQFQAK (No match)
168 - 182	1604.8877	1603.8804	1603.9185	-0.0380	0	FTITPSTTQVVGILK (No match)
183 - 199	2028.9630	2027.9557	2028.0064	-0.0507	0	IQVHYYEDGNVQLVSHK (No match)

Spot 617

Protein View

Match to: **gi|54696354** Score: **460** Expect: **1.9e-041**
protein phosphatase 1, catalytic subunit, beta isoform [Homo sapiens]

Nominal mass (M_r): **37945;** Calculated pI value: **5.84**
NCBI BLAST search of gi|54696354 against nr
Unformatted sequence string for pasting into other applications

Taxonomy: Homo sapiens
Links to retrieve other entries containing this sequence from NCBI Entrez:
gi|61355907 from synthetic construct

Fixed modifications: Carbamidomethyl (C)
Variable modifications: Oxidation (M)
Cleavage by Trypsin: cuts C-term side of KR unless next residue is P
Sequence Coverage: **30%**

Matched peptides shown in **Bold Red**

1 MADGELNVDS LITRLLEVRG CRPGKIVQMT EAEVRGLCIK SREIFLSQPI
51 PLELEAPLK**I CGDIHGQYTD LLRLFEYGGF PPEANYLFLG DYVDR**GKQSL
101 ETICLLLAYK **IKYPENFFLL R**GNHECASIN R**IYGFYDECK R**RFNIKLWKT
151 FTDCFNCLPI AAIVDEKIFC CHGGLSPDLQ SMEQIRRIMR PTDVPDTGLL
201 CDLLWSDPDK DVQGWGENDR GVSFTFGADV VSK**FLNRHDL DLICRAHQVV**
251 **EDGYEFFAK**R QLVTLFSAPN YCGEFDNAGG MMSVDETLMC SFQILKPSEK
301 KAK**YQYGGLN SGRPVTPPR**T ANPPKKR

Residue Number Increasing Mass Decreasing Mass

Start - End	Observed	Mr(expt)	Mr(calc)	Delta	Miss	Sequence
60 - 73	1660.7699	1659.7626	1659.8038	-0.0412	0	ICGDIHGQYTDLLR (No match)
60 - 73	1660.7699	1659.7626	1659.8038	-0.0412	0	ICGDIHGQYTDLLR (Ions score 71)
74 - 95	2582.1565	2581.1492	2581.2164	-0.0672	0	LFEYGGFPPEANYLFLGDYVDR (Ions score 93)
74 - 95	2582.1565	2581.1492	2581.2164	-0.0672	0	LFEYGGFPPEANYLFLGDYVDR (No match)
111 - 121	1439.7679	1438.7606	1438.7972	-0.0366	1	IKYPENFFLLR (Ions score 32)
111 - 121	1439.7679	1438.7606	1438.7972	-0.0366	1	IKYPENFFLLR (No match)
113 - 121	1198.5962	1197.5889	1197.6182	-0.0293	0	YPENFFLLR (No match)
132 - 141	1350.5790	1349.5717	1349.6074	-0.0356	1	IYGFYDECKR (No match)

234 - 245	1571.7715	1570.7642	1570.8038	-0.0396	1	FLNRHDLDLICR	(No match)
246 - 259	1639.7339	1638.7266	1638.7677	-0.0411	0	AHQVVEDGYEFFAK	(Ions score 93)
246 - 259	1639.7339	1638.7266	1638.7677	-0.0411	0	AHQVVEDGYEFFAK	(No match)
304 - 319	1761.8600	1760.8527	1760.8957	-0.0430	0	YQYGGLNSGRPVTPPR	(No match)
304 - 319	1761.8600	1760.8527	1760.8957	-0.0430	0	YQYGGLNSGRPVTPPR	(Ions score 50)

Spot 618

Protein View

Match to: **gi|119597993** Score: **445** Expect: **6.1e-040**
annexin A2, isoform CRA_c [Homo sapiens]

Nominal mass (M_r): **32600**; Calculated pI value: **5.93**
NCBI BLAST search of gi|119597993 against nr
Unformatted sequence string for pasting into other applications

Taxonomy: Homo sapiens

Fixed modifications: Carbamidomethyl (C)
Variable modifications: Oxidation (M)
Cleavage by Trypsin: cuts C-term side of KR unless next residue is P
Sequence Coverage: **47%**

Matched peptides shown in **Bold Red**

```
  1 MSTVHEILCK LSLEGDHSTP PSAYGSVKAY TNFDAERDAL NIETAIKTKG
 51 VDEVTIVNIL TNRSNAQRQD IAFAYQRRTK KELASALKSA LSGHLETVIL
101 GLLKTPAQYD ASELKASMKG LGTDEDSLIE IICSRTNQEL QEINRVYKEM
151 YKTDLEKDII SDTSGDFRKL MVALAKGRRA EDGSVIDYEL IDQDARDLYD
201 AGVKRKGTDV PKWISIMTER SVPHLQKVFD RYKSYSPYDM LESIRKEVKG
251 DLENAFLNLV QCIQNKPLYF ADRLYDSMKV RAGEIRS
```

Residue Number Increasing Mass Decreasing Mass

Start - End	Observed	Mr(expt)	Mr(calc)	Delta	Miss	Sequence
11 - 28	1844.9412	1843.9339	1843.8951	0.0388	0	LSLEGDHSTPPSAYGSVK (No match)
29 - 37	1086.5045	1085.4972	1085.4777	0.0195	0	AYTNFDAER (No match)
50 - 63	1542.8828	1541.8755	1541.8412	0.0343	0	GVDEVTIVNILTNR (No match)
50 - 63	1542.8828	1541.8755	1541.8412	0.0343	0	GVDEVTIVNILTNR (Ions score 99)
69 - 77	1111.5742	1110.5669	1110.5458	0.0212	0	QDIAFAYQR (Ions score 53)
69 - 77	1111.5742	1110.5669	1110.5458	0.0212	0	QDIAFAYQR (No match)
120 - 135	1777.8995	1776.8922	1776.8563	0.0359	0	GLGTDEDSLIEIICSR (No match)
120 - 135	1777.8995	1776.8922	1776.8563	0.0359	0	GLGTDEDSLIEIICSR (Ions score 117)
136 - 145	1244.6525	1243.6452	1243.6156	0.0296	0	TNQELQEINR (No match)
146 - 152	960.5057	959.4984	959.4786	0.0198	1	VYKEMYK (No match)
180 - 196	1908.9309	1907.9236	1907.8748	0.0488	0	AEDGSVIDYELIDQDAR (No match)

197 - 205	1036.5594	1035.5521	1035.5349	0.0173	1	**DLYDAGVKR** (No match)
213 - 220	1035.5469	1034.5396	1034.5219	0.0177	0	**WISIMTER** (No match)
213 - 220	1035.5469	1034.5396	1034.5219	0.0177	0	**WISIMTER** (Ions score 8)
213 - 220	1051.5414	1050.5341	1050.5168	0.0173	0	**WISIMTER** Oxidation (M) (No match)
228 - 233	827.4501	826.4428	826.4337	0.0091	1	**VFDRYK** (No match)
234 - 246	1588.8052	1587.7979	1587.7603	0.0377	1	**SYSPYDMLESIRK** (No match)

Spot 620

Protein View

Match to: **gi|5031601** Score: **343** Expect: **9.6e-030**
actin related protein 2/3 complex subunit 1B [Homo sapiens]

Nominal mass (M_r): **41722**; Calculated pI value: **8.69**
NCBI BLAST search of gi|5031601 against nr
Unformatted sequence string for pasting into other applications

Taxonomy: Homo sapiens
Links to retrieve other entries containing this sequence from NCBI Entrez:
gi|3121763 from Homo sapiens
gi|2282034 from Homo sapiens
gi|12803475 from Homo sapiens
gi|14043135 from Homo sapiens
gi|31416821 from Homo sapiens
gi|51094630 from Homo sapiens
gi|119597083 from Homo sapiens
gi|119597084 from Homo sapiens
gi|119597085 from Homo sapiens
gi|119597086 from Homo sapiens
gi|123982708 from synthetic construct
gi|123997375 from synthetic construct

Fixed modifications: Carbamidomethyl (C)
Variable modifications: Oxidation (M)
Cleavage by Trypsin: cuts C-term side of KR unless next residue is P
Sequence Coverage: **34%**

Matched peptides shown in **Bold Red**

1 MAYHSFLVEP ISCHAWNKDR **TQIAICPNNH** **EVHIYEK**SGA KWTK**VHELKE**
51 **HNGQVTGIDW** **APESNRIVTC** **GTDRNAYVWT** **LK**GR**TWKPTL** **VILR**INRAAR
101 CVRWAPNENK FAVGSGSRVI SICYFEQEND WWVCKHIKKP IR**STVLSLDW**
151 **HPNNVLLAAG** **SCDFK**CR**IFS** **AYIKEVEERP** **APTPWGSK**MP FGELMFESSS
201 SCGWVHGVCF SASGSR**VAWV** **SHDSTVCLAD** **ADKK**MAVATL ASETLPLLAL
251 TFITDNSLVA AGHDCFPVLF TYDAAAGMLS FGGRLDVPKQ SSQRGLTARE
301 RFQNLDKKAS SEGGTAAGAG LDSLHKNSVS QISVLSGGKA KCSQFCTTGM
351 DGGMSIWDVK SLESALKDLK IK

Residue Number Increasing Mass Decreasing Mass

Start - End	Observed	Mr(expt)	Mr(calc)	Delta	Miss	Sequence
21 - 37	2066.0786	2065.0713	2065.0051	0.0663	0	TQIAICPNNHEVHIYEK (No match)
21 - 37	2066.0786	2065.0713	2065.0051	0.0663	0	TQIAICPNNHEVHIYEK (Ions score 73)
45 - 66	2516.2974	2515.2901	2515.2203	0.0698	1	VHELKEHNGQVTGIDWAPESNR (No match)
50 - 66	1909.9371	1908.9298	1908.8714	0.0584	0	EHNGQVTGIDWAPESNR (No match)
67 - 74	921.4681	920.4608	920.4385	0.0223	0	IVTCGTDR (No match)
75 - 82	994.5577	993.5504	993.5283	0.0221	0	NAYVWTLK (No match)
85 - 94	1226.8019	1225.7946	1225.7546	0.0400	0	TWKPTLVILR (Ions score 77)
85 - 94	1226.8019	1225.7946	1225.7546	0.0400	0	TWKPTLVILR (No match)
143 - 165	2544.3484	2543.3411	2543.2478	0.0933	0	STVLSLDWHPNNVLLAAGSCDFK (No match)
168 - 174	841.4981	840.4908	840.4745	0.0163	0	IFSAYIK (No match)
175 - 188	1582.8340	1581.8267	1581.7787	0.0481	0	EVEERPAPTPWGSK (No match)
175 - 188	1582.8340	1581.8267	1581.7787	0.0481	0	EVEERPAPTPWGSK (Ions score 68)
217 - 234	2002.0381	2001.0308	2000.9625	0.0683	1	VAWVSHDSTVCLADADKK (No match)

Spot 621

Protein View

Match to: **gi|122921196** Score: **209** Expect: **2.4e-016**
Chain C, Crystal Structure Of Protein Phosphatase 2a (Pp2a) Holoenzyme With The Catalytic Subunit C

Nominal mass (M_r): **34186;** Calculated pI value: **5.13**
NCBI BLAST search of gi|122921196 against nr
Unformatted sequence string for pasting into other applications

Taxonomy: Homo sapiens
Links to retrieve other entries containing this sequence from NCBI Entrez:
gi|122921199 from Homo sapiens
gi|122921204 from Homo sapiens
gi|122921207 from Homo sapiens

Fixed modifications: Carbamidomethyl (C)
Variable modifications: Oxidation (M)
Cleavage by Trypsin: cuts C-term side of KR unless next residue is P
Sequence Coverage: **26%**

Matched peptides shown in **Bold Red**

```
  1 DEKVFTKELD QWIEQLNECK QLSESQVKSL CEKAKEILTK ESNVQEVRCP
 51 VTVCGDVHGQ FHDLMELFRI GGKSPDTNYL FMGDYVDRGY YSVETVTLLV
101 ALKVRYRERI TILRGNHESR QITQVYGFYD ECLRKYGNAN VWKYFTDLFD
151 YLPLTALVDG QIFCLHGGLS PSIDTLDHIR ALDRLQEVPH EGPMCDLLWS
201 DPDDRGGWGI SPRGAGYTFG QDISETFNHA NGLTLVSRAH QLVMEGYNWC
251 HDRNVVTIFS APNYCYRCGN QAAIMELDDT LKYSFLQFDP APR
```

Residue Number Increasing Mass Decreasing Mass

Start - End	Observed	Mr(expt)	Mr(calc)	Delta	Miss	Sequence
74 - 88	1808.7131	1807.7058	1807.7723	-0.0664	0	SPDTNYLFMGDYVDR Oxidation (M) (Ions score 16)
74 - 88	1808.7131	1807.7058	1807.7723	-0.0664	0	SPDTNYLFMGDYVDR Oxidation (M) (No match)
121 - 134	1791.7848	1790.7775	1790.8297	-0.0522	0	QITQVYGFYDECLR (No match)
121 - 134	1791.7848	1790.7775	1790.8297	-0.0522	0	QITQVYGFYDECLR (Ions score 15)
214 - 238	2655.1851	2654.1778	2654.2724	-0.0946	0	GAGYTFGQDISETFNHANGLTLVSR (Ions score 55)
214 - 238	2655.1851	2654.1778	2654.2724	-0.0946	0	GAGYTFGQDISETFNHANGLTLVSR (No match)
254 - 267	1703.7573	1702.7500	1702.8137	-0.0636	0	NVVTIFSAPNYCYR (Ions score 32)

254 - 267	1703.7573	1702.7500	1702.8137	-0.0636	0	NVVTIFSAPNYCYR	(No match)
283 - 293	1340.6118	1339.6045	1339.6560	-0.0515	0	YSFLQFDPAPR	(Ions score 7)
283 - 293	1340.6118	1339.6045	1339.6560	-0.0515	0	YSFLQFDPAPR	(No match)

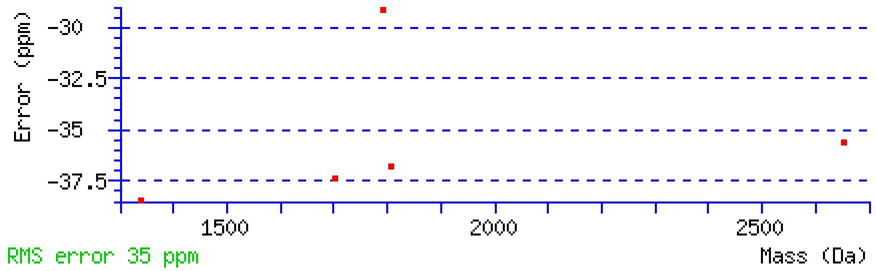

Spot 622

Mascot Search Results

Protein View

Match to: **gi|7706481** Score: **371** Expect: **1.5e-032**
calcium binding protein 39 [Homo sapiens]

Nominal mass (M_r): **40015**; Calculated pI value: **6.43**
NCBI BLAST search of gi|7706481 against nr
Unformatted sequence string for pasting into other applications

Taxonomy: Homo sapiens
Links to retrieve other entries containing this sequence from NCBI Entrez:
gi|114052110 from Bos taurus
gi|114583822 from Pan troglodytes
gi|15214082 from Homo sapiens
gi|110278901 from Bos taurus
gi|4929601 from Homo sapiens
gi|6523827 from Homo sapiens
gi|18088261 from Homo sapiens
gi|88954333 from Bos taurus
gi|119591338 from Homo sapiens
gi|119591339 from Homo sapiens
gi|158258196 (no taxonomy information for this entry)

Fixed modifications: Carbamidomethyl (C)
Variable modifications: Oxidation (M)
Cleavage by Trypsin: cuts C-term side of KR unless next residue is P
Sequence Coverage: **37%**

Matched peptides shown in **Bold Red**

```
  1 MPFPFGKSHK SPADIVKNLK ESMAVLEKQD ISDKKAEKAT EEVSKNLVAM
 51 KEILYGTNEK EPQTEAVAQL AQELYNSGLL STLVADLQLI DFEGKKDVAQ
101 IFNNILRRQI GTRTPTVEYI CTQQNILFML LKGYESPEIA LNCGIMLREC
151 IRHEPLAKII LWSEQFYDFF RYVEMSTFDI ASDAFATFKD LLTRHKLLSA
201 EFLEQHYDRF FSEYEKLLHS ENYVTKRQSL KLLGELLLDR HNFTIMTKYI
251 SKPENLKLMM NLLRDKSRNI QFEAFHVFKV FVANPNKTQP ILDILLKNQA
301 KLIEFLSKFQ NDRTEDEQFN DEKTYLVKQI RDLKRPAQQE A
```

Residue Number Increasing Mass Decreasing Mass

Start - End	Observed	Mr(expt)	Mr(calc)	Delta	Miss	Sequence
21 - 28	922.4362	921.4289	921.4477	-0.0188	0	**ESMAVLEK** Oxidation (M) (No match)
96 - 107	1430.7618	1429.7545	1429.8041	-0.0496	1	**KDVAQIFNNILR** (No match)
97 - 107	1302.6705	1301.6632	1301.7091	-0.0459	0	**DVAQIFNNILR** (No match)
97 - 107	1302.6705	1301.6632	1301.7091	-0.0459	0	**DVAQIFNNILR** (Ions score 74)
97 - 108	1458.7666	1457.7593	1457.8102	-0.0509	1	**DVAQIFNNILRR** (Ions score 24)
97 - 108	1458.7666	1457.7593	1457.8102	-0.0509	1	**DVAQIFNNILRR** (No match)
133 - 148	1838.8063	1837.7990	1837.8702	-0.0712	0	**GYESPEIALNCGIMLR** Oxidation (M) (No match)
190 - 196	882.5181	881.5108	881.5083	0.0026	1	**DLLTRHK** (No match)
195 - 209	1885.8920	1884.8847	1884.9482	-0.0635	1	**HKLLSAEFLEQHYDR** (No match)
197 - 209	1620.7468	1619.7395	1619.7943	-0.0548	0	**LLSAEFLEQHYDR** (Ions score 24)
197 - 209	1620.7468	1619.7395	1619.7943	-0.0548	0	**LLSAEFLEQHYDR** (No match)
210 - 216	949.3843	948.3770	948.4228	-0.0458	0	**FFSEYEK** (No match)
232 - 240	1041.5946	1040.5873	1040.6229	-0.0356	0	**LLGELLLDR** (No match)
232 - 248	2030.0344	2029.0271	2029.1030	-0.0758	1	**LLGELLLDRHNFTIMTK** Oxidation (M) (No match)
269 - 279	1379.6558	1378.6485	1378.7033	-0.0548	0	**NIQFEAFHVFK** (No match)
269 - 279	1379.6558	1378.6485	1378.7033	-0.0548	0	**NIQFEAFHVFK** (Ions score 80)
288 - 297	1153.6753	1152.6680	1152.7118	-0.0438	0	**TQPILDILLK** (No match)
309 - 323	1914.7389	1913.7316	1913.8027	-0.0711	1	**FQNDRTEDEQFNDEK** (No match)
309 - 323	1914.7389	1913.7316	1913.8027	-0.0711	1	**FQNDRTEDEQFNDEK** (Ions score 20)
332 - 341	1155.5734	1154.5661	1154.6043	-0.0382	1	**DLKRPAQQEA** (No match)

Mascot: http://www.matrixscience.com/

Spot 623

MATRIX SCIENCE Mascot Search Results

Protein View

Match to: **gi|62898013** Score: **128** Expect: **3e-008**
F-actin capping protein alpha-1 subunit variant [Homo sapiens]

Nominal mass (M_r): **33057**; Calculated pI value: **5.45**
NCBI BLAST search of gi|62898013 against nr
Unformatted sequence string for pasting into other applications

Taxonomy: Homo sapiens

Fixed modifications: Carbamidomethyl (C)
Variable modifications: Oxidation (M)
Cleavage by Trypsin: cuts C-term side of KR unless next residue is P
Sequence Coverage: **16%**

Matched peptides shown in **Bold Red**

```
  1 MADFDDRVSD EEKVRIAAKF ITHAPPGEFN EVFNDVRLLL NNDNLLREGA
 51 AHAFAQYNMD QFTPVKIEGY EDQVLITEHG DLGNSRFLDP RNKISFKFDH
101 LRKEASDPQP EEADGGLKSW RESCDSALRA YVKDHYSNGF CTVYAKTIDG
151 QQTIIACIES HQFQPKNFWN GRWRSEWKFT ITPPTAQVVG VPKIQVHYYE
201 DGNVQLVSHK DVQDSLTVSN EAQTAKEFIK IIENAENEYQ TAISENYQTM
251 SDTTFKALRR QLPVTRTKID WNKILSYKIG KEMQNA
```

Residue Number Increasing Mass Decreasing Mass

Start - End	Observed	Mr(expt)	Mr(calc)	Delta	Miss	Sequence
20 - 37	2088.9854	2087.9781	2088.0064	-0.0283	0	FITHAPPGEFNEVFNDVR (Ions score 57)
20 - 37	2088.9854	2087.9781	2088.0064	-0.0283	0	FITHAPPGEFNEVFNDVR (No match)
38 - 47	1197.6824	1196.6751	1196.6877	-0.0125	0	LLLNNDNLLR (No match)
67 - 86	2245.0425	2244.0352	2244.0658	-0.0305	0	IEGYEDQVLITEHGDLGNSR (No match)
67 - 86	2245.0425	2244.0352	2244.0658	-0.0305	0	IEGYEDQVLITEHGDLGNSR (Ions score 39)

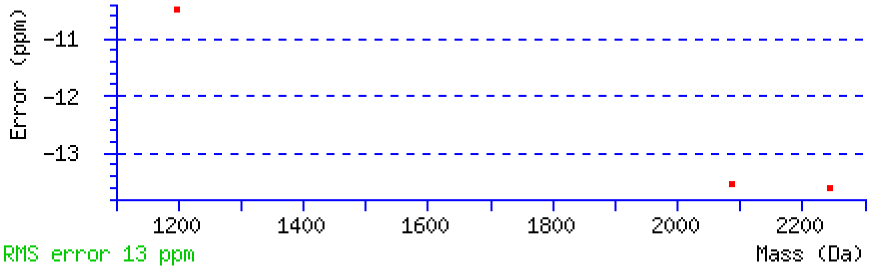

RMS error 13 ppm

Mascot: http://www.matrixscience.com/

Spot 625

{MATRIX SCIENCE} Mascot Search Results

Protein View

Match to: **gi|56966699** Score: **597** Expect: **3.8e-055**
Chain A, Annexin A2: Does It Induce Membrane Aggregation By A New Multimeric State Of The Protein

Nominal mass (M_r): **38866**; Calculated pI value: **6.92**
NCBI BLAST search of gi|56966699 against nr
Unformatted sequence string for pasting into other applications

Taxonomy: Homo sapiens

Fixed modifications: Carbamidomethyl (C)
Variable modifications: Oxidation (M)
Cleavage by Trypsin: cuts C-term side of KR unless next residue is P
Sequence Coverage: **65%**

Matched peptides shown in **Bold Red**

```
  1 MSTVHEILCK LSLEGDHSTP PSAYGSVKAY TNFDAERDAL NIETAIKTKG
 51 VDEVTIVNIL TNRSNEQRQD IAFAYQRRTK KELASALKSA LSGHLETVIL
101 GLLKTPAQYD ASELKASMKG LGTDEDSLIE IICSRTNQEL QEINRVYKEM
151 YKTDLEKDII SDTSGDFRKL MVALAKGRRA EDGSVIDYEL IDQDARDLYD
201 AGVKRKGTDV PKWISIMTER SVPHLQKVFD RYKSYSPYDM LESIRKEVKG
251 DLENAFLNLV QCIQNKPLYF ADRLYDSMKG KGTRDKVLIR IMVSRSEVDM
301 LKIRSEFKRK YGKSLYYYIQ QDTKGDYQKA LLYLCGGDD
```

Residue Number Increasing Mass Decreasing Mass

Start - End	Observed	Mr(expt)	Mr(calc)	Delta	Miss	Sequence
11 - 28	1844.8871	1843.8798	1843.8951	-0.0153	0	LSLEGDHSTPPSAYGSVK (No match)
29 - 37	1086.4805	1085.4732	1085.4777	-0.0045	0	AYTNFDAER (No match)
29 - 47	2155.0525	2154.0452	2154.0592	-0.0140	1	AYTNFDAERDALNIETAIK (No match)
48 - 63	1771.9833	1770.9760	1770.9839	-0.0079	1	TKGVDEVTIVNILTNR (No match)
50 - 63	1542.8411	1541.8338	1541.8412	-0.0074	0	GVDEVTIVNILTNR (No match)
50 - 63	1542.8411	1541.8338	1541.8412	-0.0074	0	GVDEVTIVNILTNR (Ions score 105)
69 - 77	1111.5477	1110.5404	1110.5458	-0.0053	0	QDIAFAYQR (Ions score 78)
69 - 77	1111.5477	1110.5404	1110.5458	-0.0053	0	QDIAFAYQR (No match)

89 - 104	1650.9672	1649.9599	1649.9715	-0.0116	0	SALSGHLETVILGLLK (No match)
105 - 115	1222.5854	1221.5781	1221.5877	-0.0096	0	TPAQYDASELK (No match)
120 - 135	1777.8525	1776.8452	1776.8563	-0.0111	0	GLGTDEDSLIEIICSR (Ions score 105)
120 - 135	1777.8525	1776.8452	1776.8563	-0.0111	0	GLGTDEDSLIEIICSR (No match)
136 - 145	1244.6219	1243.6146	1243.6156	-0.0010	0	TNQELQEINR (No match)
153 - 168	1811.8589	1810.8516	1810.8584	-0.0068	1	TDLEKDIISDTSGDFR (No match)
179 - 196	2064.9707	2063.9634	2063.9759	-0.0125	1	RAEDGSVIDYELIDQDAR (No match)
180 - 196	1908.8716	1907.8643	1907.8748	-0.0105	0	AEDGSVIDYELIDQDAR (No match)
197 - 204	880.4324	879.4251	879.4337	-0.0086	0	DLYDAGVK (No match)
213 - 220	1035.5211	1034.5138	1034.5219	-0.0081	0	WISIMTER (No match)
213 - 220	1035.5211	1034.5138	1034.5219	-0.0081	0	WISIMTER (Ions score 45)
213 - 220	1051.5129	1050.5056	1050.5168	-0.0112	0	WISIMTER Oxidation (M) (No match)
234 - 245	1460.6669	1459.6596	1459.6653	-0.0057	0	SYSPYDMLESIR (No match)
234 - 245	1476.6588	1475.6515	1475.6602	-0.0087	0	SYSPYDMLESIR Oxidation (M) (No match)
234 - 246	1588.7577	1587.7504	1587.7603	-0.0098	1	SYSPYDMLESIRK (No match)
234 - 246	1604.7466	1603.7393	1603.7552	-0.0158	1	SYSPYDMLESIRK Oxidation (M) (No match)
247 - 273	3194.5754	3193.5681	3193.6229	-0.0548	1	EVKGDLENAFLNLVQCIQNKPLYFADR (No match)
250 - 273	2838.3823	2837.3750	2837.4169	-0.0419	0	GDLENAFLNLVQCIQNKPLYFADR (No match)
314 - 324	1421.6826	1420.6753	1420.6874	-0.0121	0	SLYYYIQQDTK (No match)
314 - 329	2012.9475	2011.9402	2011.9527	-0.0124	1	SLYYYIQQDTKGDYQK (No match)

Mascot: http://www.matrixscience.com/

Spot 626

Mascot Search Results

Protein View

Match to: **gi|49259209** Score: **448** Expect: **3e-040**
Chain A, Human B Lactate Dehydrogenase Complexed With Nad+ And 4- Hydroxy-1,2,5-Oxadiazole-3-Carbox

Nominal mass (M_r): **36801**; Calculated pI value: **5.86**
NCBI BLAST search of gi|49259209 against nr
Unformatted sequence string for pasting into other applications

Taxonomy: Homo sapiens
Links to retrieve other entries containing this sequence from NCBI Entrez:
gi|49259210 from Homo sapiens
gi|49259211 from Homo sapiens
gi|49259212 from Homo sapiens

Fixed modifications: Carbamidomethyl (C)
Variable modifications: Oxidation (M)
Cleavage by Trypsin: cuts C-term side of KR unless next residue is P
Sequence Coverage: **43%**

Matched peptides shown in **Bold Red**

1 ATLKEK**LIAP VAEEEATVPN NKITVVGVGQ VGMACAISIL GKSLADELAL**
51 **VDVLEDKLKG EMMDLQHGSL FLQTPKIVAD KDYSVTANSK IVVVTAGVR**Q
101 QEGESRLNLV QRNVNVFK**FI IPQIVK**YSPD CIIIVVSNPV DILTYVTWKL
151 SGLPKHR**VIG SGCNLDSAR**F RYLMAEKLGI HPSSCHGWIL GEHGDSSVAV
201 WSGVNVAGVS LQELNPEMGT DNDSENWKEV HK**MVVESAYE VIK**LKGYTNW
251 AIGLSVADLI ESMLKNLSRI HPVSTMVK**GM YGIENEVFLS LPCILNAR**GL
301 TSVINQKLKD DEVAQLKKSA DTLWDIQKDL KFL

Residue Number Increasing Mass Decreasing Mass

Start - End	Observed	Mr(expt)	Mr(calc)	Delta	Miss	Sequence
7 - 22	1694.8540	1693.8467	1693.8886	-0.0419	0	LIAPVAEEEATVPNNK (Ions score 91)
7 - 22	1694.8540	1693.8467	1693.8886	-0.0419	0	LIAPVAEEEATVPNNK (No match)
23 - 42	1989.0490	1988.0417	1988.0797	-0.0380	0	ITVVGVGQVGMACAISILGK Oxidation (M) (No match)
43 - 57	1629.8173	1628.8100	1628.8508	-0.0408	0	SLADELALVDVLEDK (No match)

58 - 76	2205.0698	2204.0625	2204.0969	-0.0344	1	LKGEMMDLQHGSLFLQTPK 2 Oxidation (M) (No match)
60 - 76	1963.8920	1962.8847	1962.9179	-0.0332	0	GEMMDLQHGSLFLQTPK 2 Oxidation (M) (No match)
60 - 76	1963.8920	1962.8847	1962.9179	-0.0332	0	GEMMDLQHGSLFLQTPK 2 Oxidation (M) (Ions score 52)
77 - 90	1510.7383	1509.7310	1509.7674	-0.0364	1	IVADKDYSVTANSK (No match)
91 - 99	913.5594	912.5521	912.5756	-0.0235	0	IVVVTAGVR (No match)
119 - 126	957.5822	956.5749	956.6058	-0.0309	0	FIIPQIVK (Ions score 39)
119 - 126	957.5822	956.5749	956.6058	-0.0309	0	FIIPQIVK (No match)
158 - 169	1248.5695	1247.5622	1247.5928	-0.0305	0	VIGSGCNLDSAR (No match)
158 - 169	1248.5695	1247.5622	1247.5928	-0.0305	0	VIGSGCNLDSAR (Ions score 39)
233 - 243	1283.6161	1282.6088	1282.6478	-0.0390	0	MVVESAYEVIK Oxidation (M) (No match)
279 - 298	2312.1006	2311.0933	2311.1340	-0.0406	0	GMYGIENEVFLSLPCILNAR Oxidation (M) (No match)
279 - 298	2312.1006	2311.0933	2311.1340	-0.0406	0	GMYGIENEVFLSLPCILNAR Oxidation (M) (Ions score 62)

Mascot: http://www.matrixscience.com/

Spot 627

Mascot Search Results

Protein View

Match to: **gi|4506003** Score: **120** Expect: **1.9e-007**
protein phosphatase 1, catalytic subunit, alpha isoform 1 [Homo sapiens]

Nominal mass (M_r): **38229**; Calculated pI value: **5.94**
NCBI BLAST search of gi|4506003 against nr
Unformatted sequence string for pasting into other applications

Taxonomy: Homo sapiens
Links to retrieve other entries containing this sequence from NCBI Entrez:
gi|13928710 from Rattus norvegicus
gi|78369446 from Bos taurus
gi|113205690 from Sus scrofa
gi|49065778 from Rattus norvegicus
gi|49065811 from Homo sapiens
gi|49065813 from Oryctolagus cuniculus
gi|108860895 from Bos taurus
gi|1633403 from Oryctolagus cuniculus
gi|1633404 from Oryctolagus cuniculus
gi|1679 from Oryctolagus cuniculus
gi|35451 from Homo sapiens
gi|190516 from Homo sapiens
gi|220865 from Rattus norvegicus
gi|220867 from Rattus norvegicus
gi|999339 from Rattus sp
gi|12804879 from Homo sapiens
gi|13325343 from Homo sapiens
gi|14124968 from Homo sapiens
gi|30582097 from Homo sapiens
gi|47683037 from Rattus norvegicus
gi|60656413 from synthetic construct
gi|74267665 from Bos taurus
gi|87621715 from Sus scrofa
gi|149061962 from Rattus norvegicus
gi|227433 from Rattus norvegicus
gi|1582003 from Rattus norvegicus

Fixed modifications: Carbamidomethyl (C)
Variable modifications: Oxidation (M)

```
Cleavage by Trypsin: cuts C-term side of KR unless next residue is P
Sequence Coverage: 30%

Matched peptides shown in Bold Red

     1 MSDSEKLNLD SIIGRLLEVQ GSRPGKNVQL TENEIRGLCL KSREIFLSQP
    51 ILLELEAPLK ICGDIHGQYY DLLRLFEYGG FPPESNYLFL GDYVDRGKQS
   101 LETICLLLAY KIKYPENFFL LRGNHECASI NRIYGFYDEC KRRYNIKLWK
   151 TFTDCFNCLP IAAIVDEKIF CCHGGLSPDL QSMEQIRRIM RPTDVPDQGL
   201 LCDLLWSDPD KDVQGWGEND RGVSFTFGAE VVAKFLHKHD LDLICRAHQV
   251 VEDGYEFFAK RQLVTLFSAP NYCGEFDNAG AMMSVDETLM CSFQILKPAD
   301 KNKGKYGQFS GLNPGGRPIT PPRNSAKAKK
```

Residue Number Increasing Mass Decreasing Mass

Start - End	Observed	Mr(expt)	Mr(calc)	Delta	Miss	Sequence
16 - 26	1183.6675	1182.6602	1182.6720	-0.0118	0	LLEVQGSRPGK (No match)
27 - 36	1215.6337	1214.6264	1214.6255	0.0010	0	NVQLTENEIR (Ions score 22)
27 - 36	1215.6337	1214.6264	1214.6255	0.0010	0	NVQLTENEIR (No match)
61 - 74	1722.8110	1721.8037	1721.8195	-0.0158	0	ICGDIHGQYYDLLR (No match)
61 - 74	1722.8110	1721.8037	1721.8195	-0.0158	0	ICGDIHGQYYDLLR (No match)
75 - 96	2598.1733	2597.1660	2597.2113	-0.0453	0	LFEYGGFPPESNYLFLGDYVDR (No match)
112 - 122	1439.7910	1438.7837	1438.7972	-0.0135	1	IKYPENFFLLR (No match)
112 - 122	1439.7910	1438.7837	1438.7972	-0.0135	1	IKYPENFFLLR (No match)
247 - 260	1639.7770	1638.7697	1638.7677	0.0020	0	AHQVVEDGYEFFAK (No match)
306 - 323	1913.9813	1912.9740	1912.9907	-0.0167	0	YGQFSGLNPGGRPITPPR (No match)
306 - 323	1913.9813	1912.9740	1912.9907	-0.0167	0	YGQFSGLNPGGRPITPPR (Ions score 11)

Mascot: http://www.matrixscience.com/

Spot 628

{MATRIX SCIENCE} Mascot Search Results

Protein View

Match to: **gi|56966699** Score: **232** Expect: **1.2e-018**
Chain A, Annexin A2: Does It Induce Membrane Aggregation By A New Multimeric State Of The Protein

Nominal mass (M_r): **38866**; Calculated pI value: **6.92**
NCBI BLAST search of gi|56966699 against nr
Unformatted sequence string for pasting into other applications

Taxonomy: Homo sapiens

Fixed modifications: Carbamidomethyl (C)
Variable modifications: Oxidation (M)
Cleavage by Trypsin: cuts C-term side of KR unless next residue is P
Sequence Coverage: **34%**

Matched peptides shown in **Bold Red**

```
  1 MSTVHEILCK LSLEGDHSTP PSAYGSVKAY TNFDAERDAL NIETAIKTKG
 51 VDEVTIVNIL TNRSNEQRQD IAFAYQRRTK KELASALKSA LSGHLETVIL
101 GLLKTPAQYD ASELKASMKG LGTDEDSLIE IICSRTNQEL QEINRVYKEM
151 YKTDLEKDII SDTSGDFRKL MVALAKGRRA EDGSVIDYEL IDQDARDLYD
201 AGVKRKGTDV PKWISIMTER SVPHLQKVFD RYKSYSPYDM LESIRKEVKG
251 DLENAFLNLV QCIQNKPLYF ADRLYDSMKG KGTRDKVLIR IMVSRSEVDM
301 LKIRSEFKRK YGKSLYYYIQ QDTKGDYQKA LLYLCGGDD
```

Residue Number Increasing Mass Decreasing Mass

Start - End	Observed	Mr(expt)	Mr(calc)	Delta	Miss	Sequence
11 - 28	1844.9222	1843.9149	1843.8951	0.0198	0	LSLEGDHSTPPSAYGSVK (No match)
29 - 37	1086.4955	1085.4882	1085.4777	0.0105	0	AYTNFDAER (No match)
50 - 63	1542.8629	1541.8556	1541.8412	0.0144	0	GVDEVTIVNILTNR (No match)
50 - 63	1542.8629	1541.8556	1541.8412	0.0144	0	GVDEVTIVNILTNR (Ions score 34)
69 - 77	1111.5619	1110.5546	1110.5458	0.0089	0	QDIAFAYQR (No match)
69 - 77	1111.5619	1110.5546	1110.5458	0.0089	0	QDIAFAYQR (Ions score 15)
120 - 135	1777.8865	1776.8792	1776.8563	0.0229	0	GLGTDEDSLIEIICSR (No match)
136 - 145	1244.6370	1243.6297	1243.6156	0.0141	0	TNQELQEINR (No match)

179 - 196	2065.0125	2064.0052	2063.9759	0.0293	1	RAEDGSVIDYELIDQDAR (No match)
180 - 196	1908.9070	1907.8997	1907.8748	0.0249	0	AEDGSVIDYELIDQDAR (No match)
180 - 196	1908.9070	1907.8997	1907.8748	0.0249	0	AEDGSVIDYELIDQDAR (Ions score 57)
234 - 245	1476.6761	1475.6688	1475.6602	0.0086	0	SYSPYDMLESIR Oxidation (M) (No match)
234 - 246	1604.7874	1603.7801	1603.7552	0.0250	1	SYSPYDMLESIRK Oxidation (M) (No match)
314 - 324	1421.7112	1420.7039	1420.6874	0.0165	0	SLYYYIQQDTK (No match)

Mascot: http://www.matrixscience.com/

Spot 629

Mascot Search Results

Protein View

Match to: **gi|4503987** Score: **99** Expect: **2.7e-005**
gamma-glutamyl hydrolase precursor [Homo sapiens]

Nominal mass (M_r): **36340**; Calculated pI value: **6.67**
NCBI BLAST search of gi|4503987 against nr
Unformatted sequence string for pasting into other applications

Taxonomy: Homo sapiens
Links to retrieve other entries containing this sequence from NCBI Entrez:
gi|6016127 from Homo sapiens
gi|2951931 from Homo sapiens
gi|6090842 from Homo sapiens
gi|19263968 from Homo sapiens
gi|119607268 from Homo sapiens

Fixed modifications: Carbamidomethyl (C)
Variable modifications: Oxidation (M)
Cleavage by Trypsin: cuts C-term side of KR unless next residue is P
Sequence Coverage: **9%**

Matched peptides shown in **Bold Red**

```
  1 MASPGCLLCV LGLLLCGAAS LELSRPHGDT AKKPIIGILM QKCRNKVMKN
 51 YGRYYIAASY VKYLESAGAR VVPVRLDLTE KDYEILFKSI NGILFPGGSV
101 DLRRSDYAKV AKIFYNLSIQ SFDDGDYFPV WGTCLGFEEL SLLISGECLL
151 TATDTVDVAM PLNFTGGQLH SRMFQNFPTE LLLSLAVEPL TANFHKWSLS
201 VKNFTMNEKL KKFFNVLTTN TDGKIEFIST MEGYKYPVYG VQWHPEKAPY
251 EWKNLDGISH APNAVKTAFY LAEFFVNEAR KNNHHFKSES EEEKALIYQF
301 SPIYTGNISS FQQCYIFD
```

Residue Number Increasing Mass Decreasing Mass

Start - End	Observed	Mr(expt)	Mr(calc)	Delta	Miss	Sequence	
89 - 103	1544.8254	1543.8181	1543.8358	-0.0176	0	SINGILFPGGSVDLR	(No match)
89 - 103	1544.8254	1543.8181	1543.8358	-0.0176	0	SINGILFPGGSVDLR	(Ions score 23)

267 - 280	1677.8121	1676.8048	1676.8198	-0.0149	0	TAFYLAEFFVNEAR	(No match)
267 - 280	1677.8121	1676.8048	1676.8198	-0.0149	0	TAFYLAEFFVNEAR	(Ions score 59)

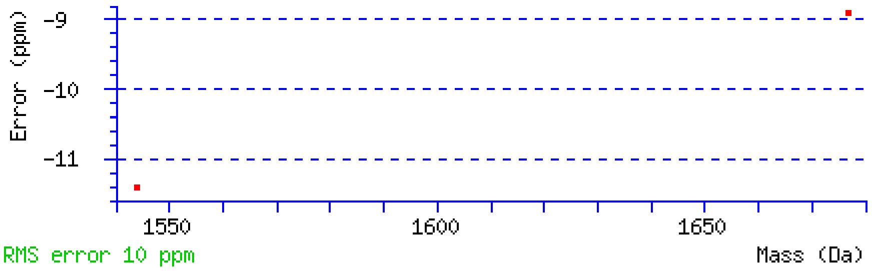

Mascot: http://www.matrixscience.com/

Spot 630

Mascot Search Results

Protein View

Match to: **gi|1584035** Score: **266** Expect: **4.8e-022**
Lasp-1 protein

Nominal mass (M_r): **30185**; Calculated pI value: **6.11**
NCBI BLAST search of gi|1584035 against nr
Unformatted sequence string for pasting into other applications

Taxonomy: Homo sapiens

Fixed modifications: Carbamidomethyl (C)
Variable modifications: Oxidation (M)
Cleavage by Trypsin: cuts C-term side of KR unless next residue is P
Sequence Coverage: **33%**

Matched peptides shown in **Bold Red**

```
  1 MNPNCARCGK IVYPTEKVNC LDKFWHKACF HCETCKMTLN MKNYKGYEKK
 51 PYCNAHYPKQ SFTMVADTPE NLRLKQQSEL QSQVRYKEEF EKNKGKGFSV
101 VADTPELQRI KKTQDQISNI KYHEEFEKSR MGPSGGEGME PERRDSQDGS
151 SYRRPLEQQQ PHHIPTSAPV YQQPQQQPVA QSYGGYKEPA APVSIQRSAP
201 GGGGKRYRAV YDYSAADEDE VSFQDGDTIV NVQQIDDGWM YGTVERTGDT
251 GMLPDNYVED I
```

Residue Number Increasing Mass Decreasing Mass

Start - End	Observed	Mr(expt)	Mr(calc)	Delta	Miss	Sequence
50 - 59	1277.5590	1276.5517	1276.6022	-0.0505	0	KPYCNAHYPK (No match)
50 - 59	1277.5590	1276.5517	1276.6022	-0.0505	0	KPYCNAHYPK (Ions score 50)
60 - 73	1624.7023	1623.6950	1623.7562	-0.0612	0	QSFTMVADTPENLR Oxidation (M) (Ions score 26)
60 - 73	1624.7023	1623.6950	1623.7562	-0.0612	0	QSFTMVADTPENLR Oxidation (M) (No match)
74 - 85	1443.7394	1442.7321	1442.7841	-0.0520	1	LKQQSELQSQVR (No match)
74 - 85	1443.7394	1442.7321	1442.7841	-0.0520	1	LKQQSELQSQVR (Ions score 15)
76 - 85	1202.5718	1201.5645	1201.6051	-0.0405	0	QQSELQSQVR (No match)
86 - 92	972.4317	971.4244	971.4599	-0.0355	1	YKEEFEK (No match)
95 - 109	1603.7898	1602.7825	1602.8365	-0.0540	1	GKGFSVVADTPELQR (No match)

97 - 109	1418.6757	1417.6684	1417.7201	-0.0517	0	GFSVVADTPELQR (Ions score 49)
97 - 109	1418.6757	1417.6684	1417.7201	-0.0517	0	GFSVVADTPELQR (No match)
122 - 128	981.3920	980.3847	980.4239	-0.0392	0	YHEEFEK (No match)
131 - 144	1521.5828	1520.5755	1520.6347	-0.0592	1	MGPSGGEGMEPERR 2 Oxidation (M) (No match)
145 - 153	1014.3791	1013.3718	1013.4050	-0.0332	0	DSQDGSSYR (No match)

Mascot: http://www.matrixscience.com/

Spot 631

MATRIX SCIENCE Mascot Search Results

Protein View

Match to: **gi|119597993** Score: **759** Expect: **2.4e-071**
annexin A2, isoform CRA_c [Homo sapiens]

Nominal mass (M_r): **32600**; Calculated pI value: **5.93**
NCBI BLAST search of gi|119597993 against nr
Unformatted sequence string for pasting into other applications

Taxonomy: Homo sapiens

Fixed modifications: Carbamidomethyl (C)
Variable modifications: Oxidation (M)
Cleavage by Trypsin: cuts C-term side of KR unless next residue is P
Sequence Coverage: **74%**

Matched peptides shown in **Bold Red**

```
  1 MSTVHEILCK LSLEGDHSTP PSAYGSVKAY TNFDAERDAL NIETAIKTKG
 51 VDEVTIVNIL TNRSNAQRQD IAFAYQRRTK KELASALKSA LSGHLETVIL
101 GLLKTPAQYD ASELKASMKG LGTDEDSLIE IICSRTNQEL QEINRVYKEM
151 YKTDLEKDII SDTSGDFRKL MVALAKGRRA EDGSVIDYEL IDQDARDLYD
201 AGVKRKGTDV PKWISIMTER SVPHLQKVFD RYKSYSPYDM LESIRKEVKG
251 DLENAFLNLV QCIQNKPLYF ADRLYDSMKV RAGEIRS
```

Residue Number Increasing Mass Decreasing Mass

Start - End	Observed	Mr(expt)	Mr(calc)	Delta	Miss	Sequence
11 - 28	1844.9426	1843.9353	1843.8951	0.0402	0	LSLEGDHSTPPSAYGSVK (No match)
29 - 37	1086.5133	1085.5060	1085.4777	0.0283	0	AYTNFDAER (No match)
29 - 47	2155.1045	2154.0972	2154.0592	0.0380	1	AYTNFDAERDALNIETAIK (No match)
48 - 63	1772.0314	1771.0241	1770.9839	0.0402	1	TKGVDEVTIVNILTNR (No match)
50 - 63	1542.8844	1541.8771	1541.8412	0.0359	0	GVDEVTIVNILTNR (Ions score 106)
50 - 63	1542.8844	1541.8771	1541.8412	0.0359	0	GVDEVTIVNILTNR (No match)
69 - 77	1111.5819	1110.5746	1110.5458	0.0289	0	QDIAFAYQR (Ions score 73)
69 - 77	1111.5819	1110.5746	1110.5458	0.0289	0	QDIAFAYQR (No match)
89 - 104	1651.0156	1650.0083	1649.9715	0.0368	0	SALSGHLETVILGLLK (No match)

105 - 115	1222.6279	1221.6206	1221.5877	0.0329	0	TPAQYDASELK (No match)
120 - 135	1777.9012	1776.8939	1776.8563	0.0376	0	GLGTDEDSLIEIICSR (No match)
120 - 135	1777.9012	1776.8939	1776.8563	0.0376	0	GLGTDEDSLIEIICSR (Ions score 135)
136 - 145	1244.6542	1243.6469	1243.6156	0.0313	0	TNQELQEINR (No match)
153 - 168	1811.9059	1810.8986	1810.8584	0.0402	1	TDLEKDIISDTSGDFR (No match)
158 - 169	1353.7037	1352.6964	1352.6572	0.0393	1	DIISDTSGDFRK (No match)
179 - 196	2065.0273	2064.0200	2063.9759	0.0441	1	RAEDGSVIDYELIDQDAR (No match)
180 - 196	1908.9216	1907.9143	1907.8748	0.0395	0	AEDGSVIDYELIDQDAR (No match)
197 - 204	880.4633	879.4560	879.4337	0.0223	0	DLYDAGVK (No match)
213 - 220	1035.5552	1034.5479	1034.5219	0.0260	0	WISIMTER (Ions score 57)
213 - 220	1035.5552	1034.5479	1034.5219	0.0260	0	WISIMTER (No match)
213 - 220	1051.5449	1050.5376	1050.5168	0.0208	0	WISIMTER Oxidation (M) (No match)
221 - 227	808.4784	807.4711	807.4603	0.0109	0	SVPHLQK (No match)
234 - 245	1460.7065	1459.6992	1459.6653	0.0339	0	SYSPYDMLESIR (No match)
234 - 245	1476.7015	1475.6942	1475.6602	0.0340	0	SYSPYDMLESIR Oxidation (M) (No match)
234 - 246	1588.8024	1587.7951	1587.7603	0.0349	1	SYSPYDMLESIRK (No match)
234 - 246	1604.7991	1603.7918	1603.7552	0.0367	1	SYSPYDMLESIRK Oxidation (M) (No match)
247 - 273	3194.6497	3193.6424	3193.6229	0.0195	1	EVKGDLENAFLNLVQCIQNKPLYFADR (No match)
250 - 273	2838.4521	2837.4448	2837.4169	0.0279	0	GDLENAFLNLVQCIQNKPLYFADR (No match)
250 - 273	2838.4521	2837.4448	2837.4169	0.0279	0	GDLENAFLNLVQCIQNKPLYFADR (Ions score 127)

Mascot: http://www.matrixscience.com/

Spot 632

Mascot Search Results

Protein View

Match to: **gi|52545806** Score: **531** Expect: **1.5e-048**
hypothetical protein [Homo sapiens]

Nominal mass (M_r): **34954;** Calculated pI value: **6.15**
NCBI BLAST search of gi|52545806 against nr
Unformatted sequence string for pasting into other applications

Taxonomy: Homo sapiens

Fixed modifications: Carbamidomethyl (C)
Variable modifications: Oxidation (M)
Cleavage by Trypsin: cuts C-term side of KR unless next residue is P
Sequence Coverage: **40%**

Matched peptides shown in **Bold Red**

```
  1 EVTRSLLQRW GASFRRGADF DSWGQLVEAI DEYQILARHL QKEAQAQHNN
 51 SEFTEEQKKT IGKIATCLEL RSAALQSTQS QEEFKLEDLK KLEPILKNIL
101 TYNKEFPFDV QPVPLRRILA PGEEENLEFE EDEEEGGAGA GSPDSFPARV
151 PGTLLPRLPS EPGMTLLTIR IEKIGLKDAG QCIDPYITVS VKDLNGIDLT
201 PVQDTPVASR KEDTYVHFNV DIELQKHVEK LTKGAAIFFE FKHYKPKKRF
251 TSTKCFAFME MDEIKPGPIV IELYKKPTDF KRKKLQLLTK KPLYLHLHQT
301 LHKE
```

Residue Number Increasing Mass Decreasing Mass

Start - End	Observed	Mr(expt)	Mr(calc)	Delta	Miss	Sequence
43 - 59	2017.8979	2016.8906	2016.9136	-0.0230	1	EAQAQHNNSEFTEEQKK (Ions score 116)
43 - 59	2017.8979	2016.8906	2016.9136	-0.0230	1	EAQAQHNNSEFTEEQKK (No match)
64 - 71	975.5272	974.5199	974.5219	-0.0019	0	IATCLELR (No match)
72 - 90	2152.0520	2151.0447	2151.0695	-0.0247	1	SAALQSTQSQEEFKLEDLK (Ions score 63)
72 - 90	2152.0520	2151.0447	2151.0695	-0.0247	1	SAALQSTQSQEEFKLEDLK (No match)
105 - 116	1443.7657	1442.7584	1442.7557	0.0027	0	EFPFDVQPVPLR (Ions score 23)
105 - 116	1443.7657	1442.7584	1442.7557	0.0027	0	EFPFDVQPVPLR (No match)
105 - 117	1599.8459	1598.8386	1598.8568	-0.0182	1	EFPFDVQPVPLRR (Ions score 45)

105 - 117	1599.8459	1598.8386	1598.8568	-0.0182	1	EFPFDVQPVPLRR (No match)
150 - 157	852.5265	851.5192	851.5228	-0.0036	0	VPGTLLPR (No match)
193 - 210	1910.9600	1909.9527	1909.9745	-0.0217	0	DLNGIDLTPVQDTPVASR (No match)
193 - 210	1910.9600	1909.9527	1909.9745	-0.0217	0	DLNGIDLTPVQDTPVASR (Ions score 113)
211 - 226	1977.9735	1976.9662	1976.9843	-0.0181	1	KEDTYVHFNVDIELQK (No match)
234 - 242	1029.5306	1028.5233	1028.5330	-0.0097	0	GAAIFFEFK (No match)
291 - 303	1627.9294	1626.9221	1626.9358	-0.0137	0	KPLYLHLHQTLHK (No match)
291 - 304	1756.9672	1755.9599	1755.9784	-0.0185	1	KPLYLHLHQTLHKE (No match)

Mascot: http://www.matrixscience.com/

Spot 633

Mascot Search Results

Protein View

Match to: **gi|5453710** Score: **121** Expect: **1.5e-007**
LIM and SH3 protein 1 [Homo sapiens]

Nominal mass (M_r): **30097**; Calculated pI value: **6.61**
NCBI BLAST search of gi|5453710 against nr
Unformatted sequence string for pasting into other applications

Taxonomy: Homo sapiens
Links to retrieve other entries containing this sequence from NCBI Entrez:
gi|3122342 from Homo sapiens
gi|2407913 from Homo sapiens
gi|119580948 from Homo sapiens
gi|119580949 from Homo sapiens
gi|123981272 from synthetic construct
gi|123996105 from synthetic construct

Fixed modifications: Carbamidomethyl (C)
Variable modifications: Oxidation (M)
Cleavage by Trypsin: cuts C-term side of KR unless next residue is P
Sequence Coverage: **24%**

Matched peptides shown in **Bold Red**

1 MNPNCARCGK IVYPTEKVNC LDKFWHKACF HCETCKMTLN MKNYKGYEKK
51 PYCNAHYPK**Q** **SFTMVADTPE** **NLRLKQQSEL** **QSQVR**YKEEF EKNKGK**GFSV**
101 **VADTPELQR**I KKTQDQISNI KYHEEFEKSR **MGPSGGEGME** **PERR**DSQDGS
151 SYRRPLEQQQ PHHIPTSAPV YQQPQQQPVA QSYGGYK**EPA** **APVSIQR**SAP
201 GGGGKRYRAV YDYSAADEDE VSFQDGDTIV NVQQIDDGWM YGTVERTGDT
251 GMLPANYVEA I

Residue Number Increasing Mass Decreasing Mass

Start - End	Observed	Mr(expt)	Mr(calc)	Delta	Miss	Sequence		
60 - 73	1624.7565	1623.7492	1623.7562	-0.0070	0	QSFTMVADTPENLR	Oxidation (M)	(No match)
60 - 73	1624.7565	1623.7492	1623.7562	-0.0070	0	QSFTMVADTPENLR	Oxidation (M)	(Ions score 16)

74 - 85	1443.7903	1442.7830	1442.7841	-0.0011	1	LKQQSELQSQVR	(No match)
74 - 85	1443.7903	1442.7830	1442.7841	-0.0011	1	LKQQSELQSQVR	(Ions score 11)
97 - 109	1418.7261	1417.7188	1417.7201	-0.0013	0	GFSVVADTPELQR	(Ions score 34)
97 - 109	1418.7261	1417.7188	1417.7201	-0.0013	0	GFSVVADTPELQR	(No match)
131 - 144	1521.6351	1520.6278	1520.6347	-0.0069	1	MGPSGGEGMEPERR 2 Oxidation (M)	(No match)
188 - 197	1067.5872	1066.5799	1066.5771	0.0029	0	EPAAPVSIQR	(No match)

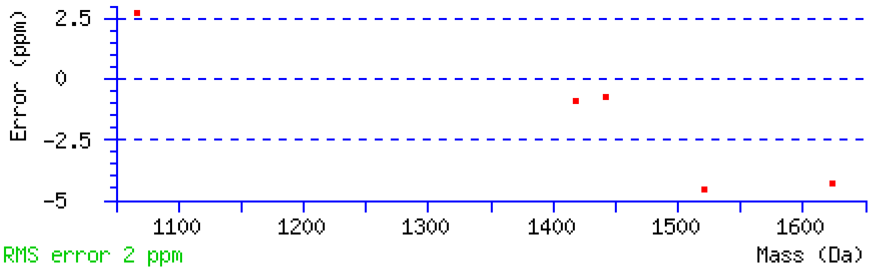

RMS error 2 ppm

Mascot: http://www.matrixscience.com/

Spot 634

Mascot Search Results

Protein View

Match to: **gi|27436969** Score: **360** Expect: **1.9e-031**
potassium voltage-gated channel, shaker-related subfamily, beta member 2 isoform 2 [Homo sapiens]

Nominal mass (M_r): **39547**; Calculated pI value: **8.81**
NCBI BLAST search of gi|27436969 against nr
Unformatted sequence string for pasting into other applications

Taxonomy: Homo sapiens
Links to retrieve other entries containing this sequence from NCBI Entrez:
gi|108996161 from Macaca mulatta
gi|2827466 from Homo sapiens
gi|56417872 from Homo sapiens
gi|119591919 from Homo sapiens

Fixed modifications: Carbamidomethyl (C)
Variable modifications: Oxidation (M)
Cleavage by Trypsin: cuts C-term side of KR unless next residue is P
Sequence Coverage: **50%**

Matched peptides shown in **Bold Red**

```
  1 MYPESTTGSP ARLSLRQTGS PGMIYRNLGK SGLRVSCLGL GTWVTFGGQI
 51 TDEMAEQLMT LAYDNGINLF DTAEVYAAGK AEVVLGNIIK KKGWRRSSLV
101 ITTKIFWGGK AETERGLSRK HIIEGLKASL ERLQLEYVDV VFANRPDPNT
151 PMEETVRAMT HVINQGMAMY WGTSRWSSME IMEAYSVARQ FNLTPPICEQ
201 AEYHMFQREK VEVQLPELFH KIGVGAMTWS PLACGIVSGK YDSGIPPYSR
251 ASLKGYQWLK DKILSEEGRR QQAKLKELQA IAERLGCTLP QLAIAWCLRN
301 EGVSSVLLGA SNADQLMENI GAIQVLPKLS SSIIHEIDSI LGNKPYSKKD
351 YRS
```

Residue Number Increasing Mass Decreasing Mass

Start - End	Observed	Mr(expt)	Mr(calc)	Delta	Miss	Sequence
1 - 12	1312.5743	1311.5670	1311.5765	-0.0095	0	MYPESTTGSPAR Oxidation (M) (No match)
17 - 26	1109.5264	1108.5191	1108.5335	-0.0144	0	QTGSPGMIYR (No match)

17 - 26	1125.5289	1124.5216	1124.5284	-0.0068	0	**QTGSPGMIYR** Oxidation (M) (No match)
133 - 157	2932.4656	2931.4583	2931.4436	0.0148	0	**LQLEYVDVVFANRPDPNTPMEETVR** (No match)
133 - 157	2948.4883	2947.4810	2947.4385	0.0425	0	**LQLEYVDVVFANRPDPNTPMEETVR** Oxidation (M) (No match)
158 - 175	2101.9363	2100.9290	2100.9179	0.0111	0	**AMTHVINQGMAMYWGTSR** 3 Oxidation (M) (No match)
176 - 189	1659.7406	1658.7333	1658.7432	-0.0099	0	**WSSMEIMEAYSVAR** (No match)
176 - 189	1675.7288	1674.7215	1674.7381	-0.0166	0	**WSSMEIMEAYSVAR** Oxidation (M) (No match)
176 - 189	1691.7318	1690.7245	1690.7330	-0.0085	0	**WSSMEIMEAYSVAR** 2 Oxidation (M) (No match)
190 - 208	2409.0774	2408.0701	2408.1041	-0.0340	0	**QFNLTPPICEQAEYHMFQR** (No match)
190 - 208	2425.0964	2424.0891	2424.0990	-0.0099	0	**QFNLTPPICEQAEYHMFQR** Oxidation (M) (No match)
209 - 221	1595.8649	1594.8576	1594.8718	-0.0142	1	**EKVEVQLPELFHK** (No match)
209 - 221	1595.8649	1594.8576	1594.8718	-0.0142	1	**EKVEVQLPELFHK** (Ions score 64)
211 - 221	1338.7244	1337.7171	1337.7343	-0.0171	0	**VEVQLPELFHK** (No match)
241 - 250	1154.5417	1153.5344	1153.5403	-0.0059	0	**YDSGIPPYSR** (Ions score 16)
241 - 250	1154.5417	1153.5344	1153.5403	-0.0059	0	**YDSGIPPYSR** (No match)
255 - 260	794.4213	793.4140	793.4122	0.0018	0	**GYQWLK** (No match)
263 - 270	959.5259	958.5186	958.5195	-0.0009	1	**ILSEEGRR** (No match)
275 - 284	1170.6791	1169.6718	1169.6768	-0.0049	1	**LKELQAIAER** (No match)
275 - 284	1170.6791	1169.6718	1169.6768	-0.0049	1	**LKELQAIAER** (Ions score 9)
277 - 284	929.5066	928.4993	928.4977	0.0016	0	**ELQAIAER** (No match)
285 - 299	1771.9121	1770.9048	1770.9273	-0.0225	0	**LGCTLPQLAIAWCLR** (No match)
329 - 348	2201.1682	2200.1609	2200.1739	-0.0130	0	**LSSSIIHEIDSILGNKPYSK** (No match)
329 - 348	2201.1682	2200.1609	2200.1739	-0.0130	0	**LSSSIIHEIDSILGNKPYSK** (Ions score 91)

Mascot: http://www.matrixscience.com/

Spot 636

Mascot Search Results

Protein View

Match to: **gi|49259209** Score: **120** Expect: **1.9e-007**
Chain A, Human B Lactate Dehydrogenase Complexed With Nad+ And 4- Hydroxy-1,2,5-Oxadiazole-3-Carbox

Nominal mass (M_r): **36801**; Calculated pI value: **5.86**
NCBI BLAST search of gi|49259209 against nr
Unformatted sequence string for pasting into other applications

Taxonomy: Homo sapiens
Links to retrieve other entries containing this sequence from NCBI Entrez:
gi|49259210 from Homo sapiens
gi|49259211 from Homo sapiens
gi|49259212 from Homo sapiens

Fixed modifications: Carbamidomethyl (C)
Variable modifications: Oxidation (M)
Cleavage by Trypsin: cuts C-term side of KR unless next residue is P
Sequence Coverage: **27%**

Matched peptides shown in **Bold Red**

```
  1 ATLKEKLIAP VAEEEATVPN NKITVVGVGQ VGMACAISIL GKSLADELAL
 51 VDVLEDKLKG EMMDLQHGSL FLQTPKIVAD KDYSVTANSK IVVVTAGVRQ
101 QEGESRLNLV QRNVNVFKFI IPQIVKYSPD CIIIVVSNPV DILTYVTWKL
151 SGLPKHRVIG SGCNLDSARF RYLMAEKLGI HPSSCHGWIL GEHGDSSVAV
201 WSGVNVAGVS LQELNPEMGT DNDSENWKEV HKMVVESAYE VIKLKGYTNW
251 AIGLSVADLI ESMLKNLSRI HPVSTMVKGM YGIENEVFLS LPCILNARGL
301 TSVINQKLKD DEVAQLKKSA DTLWDIQKDL KFL
```

Residue Number Increasing Mass Decreasing Mass

Start - End	Observed	Mr(expt)	Mr(calc)	Delta	Miss	Sequence
7 - 22	1694.8180	1693.8107	1693.8886	-0.0779	0	LIAPVAEEEATVPNNK (No match)
43 - 57	1629.7834	1628.7761	1628.8508	-0.0747	0	SLADELALVDVLEDK (Ions score 40)
43 - 57	1629.7834	1628.7761	1628.8508	-0.0747	0	SLADELALVDVLEDK (No match)
43 - 59	1870.9525	1869.9452	1870.0298	-0.0846	1	SLADELALVDVLEDKLK (No match)

58 - 76	2205.0081	2204.0008	2204.0969	-0.0961	1	LKGEMMDLQHGSLFLQTPK 2 Oxidation (M) (No match)
91 - 99	913.5386	912.5313	912.5756	-0.0443	0	IVVVTAGVR (No match)
158 - 169	1248.5417	1247.5344	1247.5928	-0.0583	0	VIGSGCNLDSAR (No match)
279 - 298	2312.0366	2311.0293	2311.1340	-0.1046	0	GMYGIENEVFLSLPCILNAR Oxidation (M) (Ions score 11)
279 - 298	2312.0366	2311.0293	2311.1340	-0.1046	0	GMYGIENEVFLSLPCILNAR Oxidation (M) (No match)

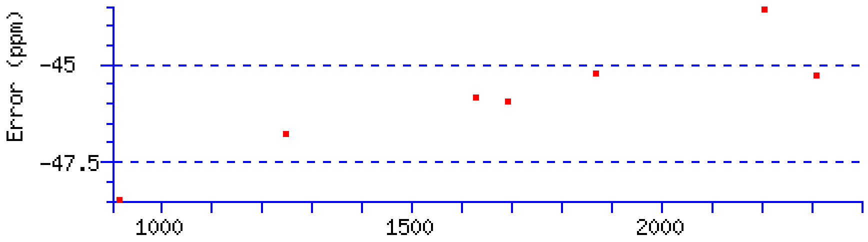

RMS error 45 ppm

Mascot: http://www.matrixscience.com/

Spot 637

{MATRIX SCIENCE} Mascot Search Results

Protein View

Match to: **gi|109148508** Score: **175** Expect: **6.1e-013**
otubain 1 [Homo sapiens]

Nominal mass (M_r): **31493**; Calculated pI value: **4.85**
NCBI BLAST search of gi|109148508 against nr
Unformatted sequence string for pasting into other applications

Taxonomy: Homo sapiens
Links to retrieve other entries containing this sequence from NCBI Entrez:
gi|44888286 from Homo sapiens
gi|13960159 from Homo sapiens
gi|28628207 from Homo sapiens
gi|78394937 from Homo sapiens
gi|119594595 from Homo sapiens

Fixed modifications: Carbamidomethyl (C)
Variable modifications: Oxidation (M)
Cleavage by Trypsin: cuts C-term side of KR unless next residue is P
Sequence Coverage: **28%**

Matched peptides shown in **Bold Red**

```
  1 MAAEEPQQQK QEPLGSDSEG VNCLAYDEAI MAQQDRIQQE IAVQNPLVSE
 51 RLELSVLYKE YAEDDNIYQQ KIKDLHKKYS YIRKTRPDGN CFYRAFGFSH
101 LEALLDDSKE LQRFKAVSAK SKEDLVSQGF TEFTIEDFHN TFMDLIEQVE
151 KQTSVADLLA SFNDQSTSDY LVVYLRLLTS GYLQRESKFF EHFIEGGRTV
201 KEFCQQEVEP MCKESDHIHI IALAQALSVS IQVEYMDRGE GGTTNPHIFP
251 EGSEPKVYLL YRPGHYDILY K
```

Residue Number Increasing Mass Decreasing Mass

Start - End	Observed	Mr(expt)	Mr(calc)	Delta	Miss	Sequence
95 - 113	2176.0176	2175.0103	2175.0959	-0.0856	1	AFGFSHLEALLDDSKELQR (No match)
152 - 176	2805.2937	2804.2864	2804.3868	-0.1004	0	QTSVADLLASFNDQSTSDYLVVYLR (No match)
152 - 176	2805.2937	2804.2864	2804.3868	-0.1004	0	QTSVADLLASFNDQSTSDYLVVYLR (Ions score 32)

177 - 185	1050.5591	1049.5518	1049.5869	-0.0351	0	LLTSGYLQR	(No match)
177 - 185	1050.5591	1049.5518	1049.5869	-0.0351	0	LLTSGYLQR	(No match)
189 - 198	1238.5422	1237.5349	1237.5879	-0.0530	0	FFEHFIEGGR	(No match)
189 - 198	1238.5422	1237.5349	1237.5879	-0.0530	0	FFEHFIEGGR	(Ions score 59)
257 - 271	1912.9558	1911.9485	1912.0246	-0.0761	0	VYLLYRPGHYDILYK	(Ions score 25)
257 - 271	1912.9558	1911.9485	1912.0246	-0.0761	0	VYLLYRPGHYDILYK	(No match)

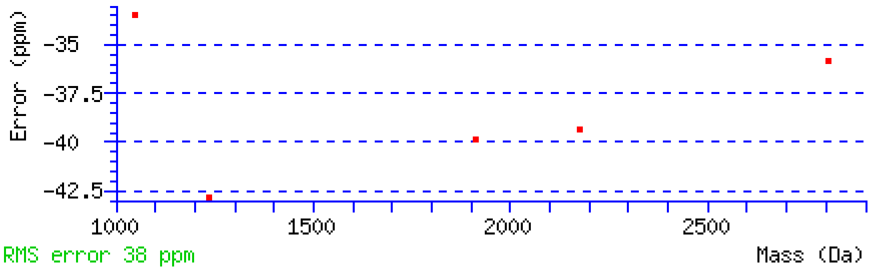

Mascot: http://www.matrixscience.com/

Spot 638

{MATRIX SCIENCE} Mascot Search Results

Protein View

Match to: **gi|306785** Score: **510** Expect: **1.9e-046**
G protein beta subunit

Nominal mass (M_r): **38061;** Calculated pI value: **5.75**
NCBI BLAST search of gi|306785 against nr
Unformatted sequence string for pasting into other applications

Taxonomy: Homo sapiens

Fixed modifications: Carbamidomethyl (C)
Variable modifications: Oxidation (M)
Cleavage by Trypsin: cuts C-term side of KR unless next residue is P
Sequence Coverage: **44%**

Matched peptides shown in **Bold Red**

```
  1 MSELEQLRQE AEQLRNQIRD ARKACGDSTL TQITAGLDPV GRIQMRTRRT
 51 LRGHLAKIYA MHWGTDSRLL VSASQDGKLI IWDSYTTNKV HAIPLRSSWV
101 MTCAYAXSGN FVACGGLDNI CSIYSLKTRE GNVRVSRELP GHTGYLSCCR
151 FLDDNQIITS SGDTTCALWD IETGQQTVGF AGHSGDVMSL SLAPNGRTFV
201 SGACDASIKL WDVRDSMCRQ TFIGHESDIN AVAFFPNGYA FTTGSDDATC
251 RLFDLRADQE LLMYSHDNII CGITSVAFSR SGRLLLAGYD DFNCNIWDAM
301 KGDRAGVLAG HDNRVSCLGV TDDGMAVATG SWDSFLKIWN
```

Residue Number Increasing Mass Decreasing Mass

Start - End	Observed	Mr(expt)	Mr(calc)	Delta	Miss	Sequence
2 - 15	1728.8866	1727.8793	1727.8802	-0.0008	1	SELEQLRQEAEQLR (Ions score 7)
2 - 15	1728.8866	1727.8793	1727.8802	-0.0008	1	SELEQLRQEAEQLR (No match)
9 - 15	873.4515	872.4442	872.4352	0.0091	0	QEAEQLR (No match)
23 - 42	2060.0571	2059.0498	2059.0367	0.0131	1	KACGDSTLTQITAGLDPVGR (Ions score 93)
23 - 42	2060.0571	2059.0498	2059.0367	0.0131	1	KACGDSTLTQITAGLDPVGR (No match)
24 - 42	1931.9595	1930.9522	1930.9418	0.0104	0	ACGDSTLTQITAGLDPVGR (No match)
24 - 42	1931.9595	1930.9522	1930.9418	0.0104	0	ACGDSTLTQITAGLDPVGR (Ions score 136)
58 - 68	1336.6176	1335.6103	1335.6030	0.0073	0	IYAMHWGTDSR (No match)

58 - 68	1352.6093	1351.6020	1351.5979	0.0041	0	**IYAMHWGTDSR** Oxidation (M) (No match)
90 - 96	805.5103	804.5030	804.4970	0.0061	0	**VHAIPLR** (No match)
130 - 137	916.4799	915.4726	915.4886	-0.0159	1	**EGNVRVSR** (No match)
138 - 150	1549.6990	1548.6917	1548.6813	0.0104	0	**ELPGHTGYLSCCR** (Ions score 114)
138 - 150	1549.6990	1548.6917	1548.6813	0.0104	0	**ELPGHTGYLSCCR** (No match)
198 - 209	1255.6022	1254.5949	1254.5914	0.0035	0	**TFVSGACDASIK** (No match)
220 - 251	3509.6125	3508.6052	3508.5629	0.0423	0	**QTFIGHESDINAVAFFPNGYAFTTGSDDATCR** (No match)
257 - 280	2756.3394	2755.3321	2755.2945	0.0376	0	**ADQELLMYSHDNIICGITSVAFSR** Oxidation (M) (No match)
305 - 314	1009.5252	1008.5179	1008.5100	0.0079	0	**AGVLAGHDNR** (No match)

Mascot: http://www.matrixscience.com/

Spot 639

Mascot Search Results

Protein View

Match to: **gi|28827795** Score: **90** Expect: **0.00021**
chromatin modifying protein 4B [Homo sapiens]

Nominal mass (M_r): **24935**; Calculated pI value: **4.76**
NCBI BLAST search of gi|28827795 against nr
Unformatted sequence string for pasting into other applications

Taxonomy: Homo sapiens
Links to retrieve other entries containing this sequence from NCBI Entrez:
gi|24636296 from Homo sapiens
gi|12314022 from Homo sapiens
gi|21708120 from Homo sapiens
gi|32879760 from Homo sapiens
gi|37362118 from Homo sapiens
gi|119596699 from Homo sapiens
gi|119596700 from Homo sapiens

Fixed modifications: Carbamidomethyl (C)
Variable modifications: Oxidation (M)
Cleavage by Trypsin: cuts C-term side of KR unless next residue is P
Sequence Coverage: **12%**

Matched peptides shown in **Bold Red**

```
  1 MSVFGKLFGA GGGKAGKGGP TPQEAIQRLR DTEEMLSKKQ EFLEKKIEQE
 51 LTAAKKHGTK NKRAALQALK RKKRYEKQLA QIDGTLSTIE FQREALENAN
101 TNTEVLKNMG YAAKAMKAAH DNMDIDKVDE LMQDIADQQE LAEEISTAIS
151 KPVGFGEEFD EDELMAELEE LEQEELDKNL LEISGPETVP LPNVPSIALP
201 SKPAKKKEEE DDDMKELENW AGSM
```

Residue Number Increasing Mass Decreasing Mass

Start - End	Observed	Mr(expt)	Mr(calc)	Delta	Miss	Sequence	
18 - 28	1153.5811	1152.5738	1152.5887	-0.0149	0	GGPTPQEAIQR	(Ions score 20)
18 - 28	1153.5811	1152.5738	1152.5887	-0.0149	0	GGPTPQEAIQR	(No match)

78 - 93	1819.9121	1818.9048	1818.9475	-0.0427	0	QLAQIDGTLSTIEFQR	(No match)
78 - 93	1819.9121	1818.9048	1818.9475	-0.0427	0	QLAQIDGTLSTIEFQR	(Ions score 53)

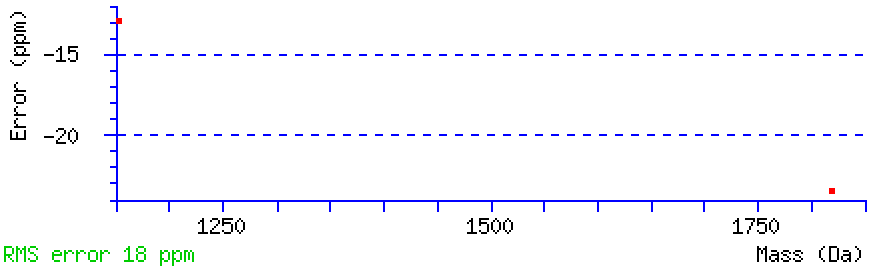

RMS error 18 ppm

Mascot: http://www.matrixscience.com/

Spot 640

Mascot Search Results

Protein View

Match to: **gi|5410300** Score: **68** Expect: **0.033**
COP9 complex subunit 4 [Homo sapiens]

Nominal mass (M_r): **46454**; Calculated pI value: **5.57**
NCBI BLAST search of gi|5410300 against nr
Unformatted sequence string for pasting into other applications

Taxonomy: Homo sapiens

Fixed modifications: Carbamidomethyl (C)
Variable modifications: Oxidation (M)
Cleavage by Trypsin: cuts C-term side of KR unless next residue is P
Sequence Coverage: **2%**

Matched peptides shown in **Bold Red**

```
  1 MAAVRQDLAQ LMNSSGSHKD LAGKYRQILE KAIQLSGAEQ LEALKAFVEA
 51 MVNENVSLVI SRQLLTDFCT HLPNLPDSTA KEIYHFTLEK IQPRVISFEE
101 QVASIRQHLA SIYEKEEDWR NAAQVLVGIP LETGQKQYNV DYKLETYLKI
151 ARLYLEDDDP VQAEAYINRA SLLQNESTNE QLQIHYKVCY ARVLDYRRKF
201 IEAAQRYNEL SYKTIVHESE RLEALKHALH CTILASAGQQ RSRMLATLFK
251 DERCQQLAAY GILEKMYLDR IIRGNQLQEF AAMLMPHQKA TTADGSSILD
301 RAVIEHNLLS ASKLYNNITF EELGALLEIP AAKAEKIASQ MITEGRMNGF
351 IDQIDGIVHF ETREALPTWD KQIQSLCFQV NNLLEKISQT APEWTAQAME
401 AQMAQ
```

Residue Number Increasing Mass Decreasing Mass

Start - End	Observed	Mr(expt)	Mr(calc)	Delta	Miss	Sequence	
95 - 106	1377.6698	1376.6625	1376.7299	-0.0674	0	VISFEEQVASIR	(Ions score 63)
95 - 106	1377.6698	1376.6625	1376.7299	-0.0674	0	VISFEEQVASIR	(No match)

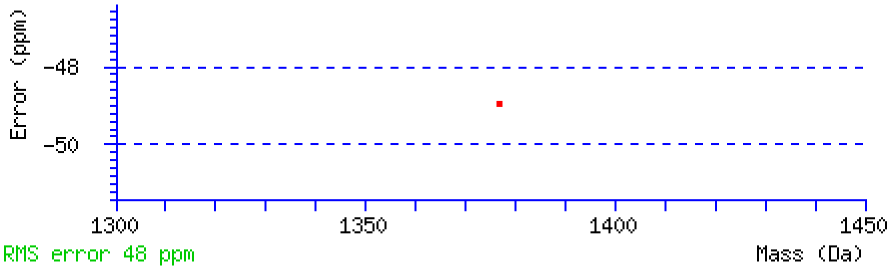

Mascot: http://www.matrixscience.com/

Spot 642

{MATRIX SCIENCE} **Mascot Search Results**

Protein View

Match to: **gi|5174539** Score: **213** Expect: **9.6e-017**
cytosolic malate dehydrogenase [Homo sapiens]

Nominal mass (M_r): **36631**; Calculated pI value: **6.91**
NCBI BLAST search of gi|5174539 against nr
Unformatted sequence string for pasting into other applications

Taxonomy: Homo sapiens
Links to retrieve other entries containing this sequence from NCBI Entrez:
gi|1708967 from Homo sapiens
gi|1255604 from Homo sapiens
gi|3133269 from Homo sapiens
gi|16306622 from Homo sapiens
gi|48146927 from Homo sapiens
gi|62822344 from Homo sapiens
gi|119620365 from Homo sapiens

Fixed modifications: Carbamidomethyl (C)
Variable modifications: Oxidation (M)
Cleavage by Trypsin: cuts C-term side of KR unless next residue is P
Sequence Coverage: **32%**

Matched peptides shown in **Bold Red**

1 MSEPIR**VLVT GAAGQIAYSL LYSIGNGSVF GK**DQPIILVL LDITPMMGVL
51 DGVLMELQDC ALPLLKDVIA TDKEDVAFK**D LDVAILVGSM PR**REGMERKD
101 LLKANVKIFK SQGAALDKYA KKSVK**VIVVG NPANTNCLTA SKSAPSIPKE**
151 **NFSCLTR**LDH NRAKAQIALK LGVTANDVKN VIIWGNHSST QYPDVNHAKV
201 KLQGKEVGVY EALKDDSWLK **GEFVTTVQQR** GAAVIKARKL SSAMSAAKAI
251 CDHVRDIWFG TPEGEFVSMG VISDGNSYGV PDDLLYSFPV VIKNKTWK**FV**
301 **EGLPINDFSR** EKMDLTAK**EL TEEKESAFEF LSSA**

Residue Number Increasing Mass Decreasing Mass

Start - End	Observed	Mr(expt)	Mr(calc)	Delta	Miss	Sequence

7 - 32	2585.2856	2584.2783	2584.3899	-0.1116	0	VLVTGAAGQIAYSLLYSIGNGSVFGK (No match)
80 - 92	1401.6896	1400.6823	1400.7333	-0.0510	0	DLDVAILVGSMPR Oxidation (M) (Ions score 8)
80 - 92	1401.6896	1400.6823	1400.7333	-0.0510	0	DLDVAILVGSMPR Oxidation (M) (No match)
126 - 142	1757.8340	1756.8267	1756.9141	-0.0874	0	VIVVGNPANTNCLTASK (No match)
143 - 157	1706.7927	1705.7854	1705.8457	-0.0603	1	SAPSIPKENFSCLTR (No match)
143 - 157	1706.7927	1705.7854	1705.8457	-0.0603	1	SAPSIPKENFSCLTR (Ions score 26)
150 - 157	1026.4274	1025.4201	1025.4600	-0.0399	0	ENFSCLTR (Ions score 5)
150 - 157	1026.4274	1025.4201	1025.4600	-0.0399	0	ENFSCLTR (No match)
221 - 230	1164.5527	1163.5454	1163.5934	-0.0480	0	GEFVTTVQQR (No match)
221 - 230	1164.5527	1163.5454	1163.5934	-0.0480	0	GEFVTTVQQR (Ions score 26)
299 - 310	1393.6615	1392.6542	1392.7037	-0.0495	0	FVEGLPINDFSR (Ions score 65)
299 - 310	1393.6615	1392.6542	1392.7037	-0.0495	0	FVEGLPINDFSR (No match)
319 - 334	1816.7803	1815.7730	1815.8414	-0.0683	1	ELTEEKESAFEFLSSA (No match)

Mascot: http://www.matrixscience.com/

Spot 643

Mascot Search Results

Protein View

Match to: **gi|62897717** Score: **253** Expect: **9.6e-021**
lactate dehydrogenase A variant [Homo sapiens]

Nominal mass (M_r): **36951**; Calculated pI value: **7.63**
NCBI BLAST search of gi|62897717 against nr
Unformatted sequence string for pasting into other applications

Taxonomy: Homo sapiens

Fixed modifications: Carbamidomethyl (C)
Variable modifications: Oxidation (M)
Cleavage by Trypsin: cuts C-term side of KR unless next residue is P
Sequence Coverage: **26%**

Matched peptides shown in **Bold Red**

```
  1 MATLKDQLIY NLLKEEQTPQ NKITVVGVGA VGMACAISIL MKDLADELAL
 51 VDVIEDKLKG EMMDLQHGSL FLRTPKIVSG KDYNVTANSK LVIITAGARQ
101 QEGESRLNLV QRNVNIFKFI IPNVVKYSPN CKLLIVSNPV DILTYVAWKI
151 SGFPKNRVIG SGCNLDSARF RYLMGERLGV HPLSCHGWVL GEHGDSSVPV
201 WSGMNVAGVS LKTLHPDLGT DEDKEQWKEV HKQVVESAYE VIKLKGYTSW
251 AIGLSVADLA ESIMKNLRRV HPVSTMIKGL YGIKDDVFLS VPCILGQNGI
301 SDLVKVTLTS EEEARLKKSA DTLWGIQKEL QF
```

Residue Number Increasing Mass Decreasing Mass

Start - End	Observed	Mr(expt)	Mr(calc)	Delta	Miss	Sequence
6 - 22	2074.0532	2073.0459	2073.0742	-0.0282	1	DQLIYNLLKEEQTPQNK (No match)
6 - 22	2074.0532	2073.0459	2073.0742	-0.0282	1	DQLIYNLLKEEQTPQNK (Ions score 103)
43 - 57	1657.8269	1656.8196	1656.8457	-0.0261	0	DLADELALVDVIEDK (No match)
58 - 73	1906.9290	1905.9217	1905.9440	-0.0223	1	LKGEMMDLQHGSLFLR 2 Oxidation (M) (Ions score 12)
58 - 73	1906.9290	1905.9217	1905.9440	-0.0223	1	LKGEMMDLQHGSLFLR 2 Oxidation (M) (No match)
60 - 73	1649.7537	1648.7464	1648.7701	-0.0237	0	GEMMDLQHGSLFLR Oxidation (M) (No match)
60 - 73	1665.7479	1664.7406	1664.7650	-0.0244	0	GEMMDLQHGSLFLR 2 Oxidation (M) (No match)
60 - 73	1665.7479	1664.7406	1664.7650	-0.0244	0	GEMMDLQHGSLFLR 2 Oxidation (M) (Ions score 25)

91 - 99	913.5780	912.5707	912.5756	-0.0049	0	**LVIITAGAR**	(No match)
119 - 126	929.5693	928.5620	928.5745	-0.0125	0	**FIIPNVVK**	(No match)
158 - 169	1248.6002	1247.5929	1247.5928	0.0002	0	**VIGSGCNLDSAR**	(Ions score 15)
158 - 169	1248.6002	1247.5929	1247.5928	0.0002	0	**VIGSGCNLDSAR**	(No match)
306 - 315	1134.5577	1133.5504	1133.5564	-0.0060	0	**VTLTSEEEAR**	(No match)

Mascot: http://www.matrixscience.com/

Spot 644

{MATRIX SCIENCE} Mascot Search Results

Protein View

Match to: **gi|13786849** Score: **133** Expect: **9.6e-009**
Chain A, Human Muscle L-Lactate Dehydrogenase M Chain, Ternary Complex With Nadh And Oxamate

Nominal mass (M_r): **36819**; Calculated pI value: **8.46**
NCBI BLAST search of gi|13786849 against nr
Unformatted sequence string for pasting into other applications

Taxonomy: Homo sapiens
Links to retrieve other entries containing this sequence from NCBI Entrez:
gi|13786850 from Homo sapiens
gi|13786851 from Homo sapiens
gi|13786852 from Homo sapiens
gi|13786853 from Homo sapiens
gi|13786854 from Homo sapiens
gi|13786855 from Homo sapiens
gi|13786856 from Homo sapiens

Fixed modifications: Carbamidomethyl (C)
Variable modifications: Oxidation (M)
Cleavage by Trypsin: cuts C-term side of KR unless next residue is P
Sequence Coverage: **21%**

Matched peptides shown in **Bold Red**

```
  1 ATLKDQLIYN LLKEEQTPQN KITVVGVGAV GMACAISILM KDLADELALV
 51 DVIEDKLKGE MMDLQHGSLF LRTPKIVSGK DYNVTANSKL VIITAGARQQ
101 EGESRLNLVQ RNVNIFKFII PNVVKYSPNC KLLIVSNPVD ILTYVAWKIS
151 GFPKNRVIGS GCNLDSARFR YLMGERLGVH PLSCHGWVLG EHGDSSVPVW
201 SGMNVAGVSL KTLHPDLGTD KDKEQWKEVH KQVVESAYEV IKLKGYTSWA
251 IGLSVADLAE SIMKNLRRVH PVSTMIKGLY GIKDDVFLSV PCILGQNGIS
301 DLVKVTLTSE EEARLKKSAD TLWGIQKELQ F
```

Residue Number Increasing Mass Decreasing Mass

Start - End	Observed	Mr(expt)	Mr(calc)	Delta	Miss	Sequence

42 - 56	1657.7889	1656.7816	1656.8457	-0.0641	0	DLADELALVDVIEDK (Ions score 75)
42 - 56	1657.7889	1656.7816	1656.8457	-0.0641	0	DLADELALVDVIEDK (No match)
57 - 72	1906.8844	1905.8771	1905.9440	-0.0669	1	LKGEMMDLQHGSLFLR 2 Oxidation (M) (No match)
57 - 72	1906.8844	1905.8771	1905.9440	-0.0669	1	LKGEMMDLQHGSLFLR 2 Oxidation (M) (Ions score 0)
59 - 72	1665.7106	1664.7033	1664.7650	-0.0617	0	GEMMDLQHGSLFLR 2 Oxidation (M) (No match)
90 - 98	913.5485	912.5412	912.5756	-0.0344	0	LVIITAGAR (No match)
118 - 125	929.5450	928.5377	928.5745	-0.0368	0	FIIPNVVK (No match)
157 - 168	1248.5629	1247.5556	1247.5928	-0.0371	0	VIGSGCNLDSAR (No match)
305 - 314	1134.5273	1133.5200	1133.5564	-0.0364	0	VTLTSEEEAR (No match)

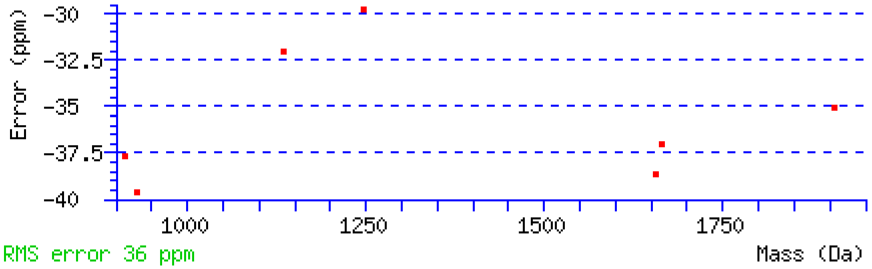

Mascot: http://www.matrixscience.com/

Spot 645

Mascot Search Results

Protein View

Match to: **gi|13786849** Score: **554** Expect: **7.7e-051**
Chain A, Human Muscle L-Lactate Dehydrogenase M Chain, Ternary Complex With Nadh And Oxamate

Nominal mass (M_r): **36819**; Calculated pI value: **8.46**
NCBI BLAST search of gi|13786849 against nr
Unformatted sequence string for pasting into other applications

Taxonomy: Homo sapiens
Links to retrieve other entries containing this sequence from NCBI Entrez:
gi|13786850 from Homo sapiens
gi|13786851 from Homo sapiens
gi|13786852 from Homo sapiens
gi|13786853 from Homo sapiens
gi|13786854 from Homo sapiens
gi|13786855 from Homo sapiens
gi|13786856 from Homo sapiens

Fixed modifications: Carbamidomethyl (C)
Variable modifications: Oxidation (M)
Cleavage by Trypsin: cuts C-term side of KR unless next residue is P
Sequence Coverage: **50%**

Matched peptides shown in **Bold Red**

```
  1 ATLKDQLIYN LLKEEQTPQN KITVVGVGAV GMACAISILM KDLADELALV
 51 DVIEDKLKGE MMDLQHGSLF LRTPKIVSGK DYNVTANSKL VIITAGARQQ
101 EGESRLNLVQ RNVNIFKFII PNVVKYSPNC KLLIVSNPVD ILTYVAWKIS
151 GFPKNRVIGS GCNLDSARFR YLMGERLGVH PLSCHGWVLG EHGDSSVPVW
201 SGMNVAGVSL KTLHPDLGTD KDKEQWKEVH KQVVESAYEV IKLKGYTSWA
251 IGLSVADLAE SIMKNLRRVH PVSTMIKGLY GIKDDVFLSV PCILGQNGIS
301 DLVKVTLTSE EEARLKKSAD TLWGIQKELQ F
```

Residue Number Increasing Mass Decreasing Mass

Start - End	Observed	Mr(expt)	Mr(calc)	Delta	Miss	Sequence

5 - 13	1119.5731	1118.5658	1118.6335	-0.0677	0	DQLIYNLLK (No match)
5 - 21	2074.0356	2073.0283	2073.0742	-0.0458	1	DQLIYNLLKEEQTPQNK (No match)
42 - 56	1657.8074	1656.8001	1656.8457	-0.0456	0	DLADELALVDVIEDK (No match)
42 - 58	1898.9834	1897.9761	1898.0248	-0.0486	1	DLADELALVDVIEDKLK (No match)
57 - 72	1874.9078	1873.9005	1873.9542	-0.0537	1	LKGEMMDLQHGSLFLR (Ions score 130)
57 - 72	1874.9078	1873.9005	1873.9542	-0.0537	1	LKGEMMDLQHGSLFLR (No match)
57 - 72	1890.9086	1889.9013	1889.9491	-0.0478	1	LKGEMMDLQHGSLFLR Oxidation (M) (No match)
57 - 72	1906.9105	1905.9032	1905.9440	-0.0408	1	LKGEMMDLQHGSLFLR 2 Oxidation (M) (No match)
59 - 72	1633.7350	1632.7277	1632.7752	-0.0475	0	GEMMDLQHGSLFLR (No match)
59 - 72	1633.7350	1632.7277	1632.7752	-0.0475	0	GEMMDLQHGSLFLR (Ions score 116)
59 - 72	1649.7301	1648.7228	1648.7701	-0.0473	0	GEMMDLQHGSLFLR Oxidation (M) (No match)
59 - 72	1649.7301	1648.7228	1648.7701	-0.0473	0	GEMMDLQHGSLFLR Oxidation (M) (Ions score 74)
59 - 72	1665.7273	1664.7200	1664.7650	-0.0450	0	GEMMDLQHGSLFLR 2 Oxidation (M) (No match)
81 - 89	1011.5106	1010.5033	1010.4668	0.0365	0	DYNVTANSK (No match)
90 - 98	913.5596	912.5523	912.5756	-0.0233	0	LVIITAGAR (No match)
99 - 105	833.3579	832.3506	832.3675	-0.0168	0	QQEGESR (No match)
118 - 125	929.5543	928.5470	928.5745	-0.0275	0	FIIPNVVK (No match)
157 - 168	1248.5698	1247.5625	1247.5928	-0.0302	0	VIGSGCNLDSAR (Ions score 68)
157 - 168	1248.5698	1247.5625	1247.5928	-0.0302	0	VIGSGCNLDSAR (No match)
171 - 176	768.3544	767.3471	767.3636	-0.0164	0	YLMGER (No match)
171 - 176	784.3449	783.3376	783.3585	-0.0209	0	YLMGER Oxidation (M) (No match)
232 - 242	1264.6390	1263.6317	1263.6710	-0.0393	0	QVVESAYEVIK (No match)
268 - 277	1167.6379	1166.6306	1166.6594	-0.0287	1	RVHPVSTMIK (No match)
268 - 277	1183.6311	1182.6238	1182.6543	-0.0304	1	RVHPVSTMIK Oxidation (M) (No match)
278 - 304	2920.5188	2919.5115	2919.5415	-0.0299	1	GLYGIKDDVFLSVPCILGQNGISDLVK (No match)
284 - 304	2289.1458	2288.1385	2288.1721	-0.0336	0	DDVFLSVPCILGQNGISDLVK (No match)
305 - 314	1134.5355	1133.5282	1133.5564	-0.0282	0	VTLTSEEEAR (No match)
317 - 327	1246.6399	1245.6326	1245.6717	-0.0391	1	KSADTLWGIQK (No match)
318 - 327	1118.5505	1117.5432	1117.5767	-0.0335	0	SADTLWGIQK (No match)

Mascot: http://www.matrixscience.com/

Spot 646

Mascot Search Results

Protein View

Match to: **gi|306785** Score: **286** Expect: **4.8e-024**
G protein beta subunit

Nominal mass (M_r): **38061**; Calculated pI value: **5.75**
NCBI BLAST search of gi|306785 against nr
Unformatted sequence string for pasting into other applications

Taxonomy: Homo sapiens

Fixed modifications: Carbamidomethyl (C)
Variable modifications: Oxidation (M)
Cleavage by Trypsin: cuts C-term side of KR unless next residue is P
Sequence Coverage: **29%**

Matched peptides shown in **Bold Red**

```
  1 MSELEQLRQE AEQLRNQIRD ARKACGDSTL TQITAGLDPV GRIQMRTRRT
 51 LRGHLAKIYA MHWGTDSRLL VSASQDGKLI IWDSYTTNKV HAIPLRSSWV
101 MTCAYAXSGN FVACGGLDNI CSIYSLKTRE GNVRVSRELP GHTGYLSCCR
151 FLDDNQIITS SGDTTCALWD IETGQQTVGF AGHSGDVMSL SLAPNGRTFV
201 SGACDASIKL WDVRDSMCRQ TFIGHESDIN AVAFFPNGYA FTTGSDDATC
251 RLFDLRADQE LLMYSHDNII CGITSVAFSR SGRLLLAGYD DFNCNIWDAM
301 KGDRAGVLAG HDNRVSCLGV TDDGMAVATG SWDSFLKIWN
```

Residue Number Increasing Mass Decreasing Mass

Start - End	Observed	Mr(expt)	Mr(calc)	Delta	Miss	Sequence
2 - 15	1728.8352	1727.8279	1727.8802	-0.0522	1	SELEQLRQEAEQLR (No match)
2 - 15	1728.8352	1727.8279	1727.8802	-0.0522	1	SELEQLRQEAEQLR (Ions score 2)
9 - 15	873.4128	872.4055	872.4352	-0.0296	0	QEAEQLR (No match)
23 - 42	2059.9924	2058.9851	2059.0367	-0.0516	1	KACGDSTLTQITAGLDPVGR (No match)
23 - 42	2059.9924	2058.9851	2059.0367	-0.0516	1	KACGDSTLTQITAGLDPVGR (Ions score 76)
24 - 42	1931.8984	1930.8911	1930.9418	-0.0507	0	ACGDSTLTQITAGLDPVGR (No match)
58 - 68	1352.5685	1351.5612	1351.5979	-0.0367	0	IYAMHWGTDSR Oxidation (M) (No match)
90 - 96	805.4775	804.4702	804.4970	-0.0267	0	VHAIPLR (No match)

138 - 150	1549.6509	1548.6436	1548.6813	-0.0377	0	**ELPGHTGYLSCCR**	(Ions score 97)
138 - 150	1549.6509	1548.6436	1548.6813	-0.0377	0	**ELPGHTGYLSCCR**	(No match)
257 - 280	2756.2327	2755.2254	2755.2945	-0.0691	0	**ADQELLMYSHDNIICGITSVAFSR**	Oxidation (M) (No match)
305 - 314	1009.4901	1008.4828	1008.5100	-0.0272	0	**AGVLAGHDNR**	(No match)

Mascot: http://www.matrixscience.com/

Spot 647

Mascot Search Results

Protein View

Match to: **gi|119589975** Score: **169** Expect: **2.4e-012**
calponin 2, isoform CRA_e [Homo sapiens]

Nominal mass (M_r): **31829**; Calculated pI value: **7.57**
NCBI BLAST search of gi|119589975 against nr
Unformatted sequence string for pasting into other applications

Taxonomy: Homo sapiens

Fixed modifications: Carbamidomethyl (C)
Variable modifications: Oxidation (M)
Cleavage by Trypsin: cuts C-term side of KR unless next residue is P
Sequence Coverage: **40%**

Matched peptides shown in **Bold Red**

```
  1 MSSTQFNKGP SYGLSAEVKN RLLSKYDPQK EAELRTWIEG LTGLSIGPDF
 51 QKGLKDGTIL CTLMNKLQPG SVPKINRSMQ NWHQLENLSN FIKAMVSYGM
101 NPVDLFEAND LFESGNMTQV QVSLLALAGK AKTKGLQSGV DIGVKYSEKQ
151 ERNFDDATMK AGQCVIGLQM GTNKCASQSG MTAYGTRRHL YDPKNHILPP
201 MDHSTISLQM GTNKCASQVG MTAPGTRRHI YDTKLQSGQV FGLGRQIYDP
251 KYCPQGTVAD GAPSGTGDCP DPGEVPEYPP YYQEEAGY
```

Residue Number Increasing Mass Decreasing Mass

Start - End	Observed	Mr(expt)	Mr(calc)	Delta	Miss	Sequence
26 - 35	1248.6198	1247.6125	1247.6146	-0.0020	1	YDPQKEAELR (No match)
26 - 35	1248.6198	1247.6125	1247.6146	-0.0020	1	YDPQKEAELR (Ions score 69)
36 - 52	1861.9700	1860.9627	1860.9621	0.0006	0	TWIEGLTGLSIGPDFQK (No match)
78 - 93	1988.9669	1987.9596	1987.9574	0.0022	0	SMQNWHQLENLSNFIK (No match)
146 - 152	939.4535	938.4462	938.4457	0.0005	1	YSEKQER (No match)
161 - 174	1476.7394	1475.7321	1475.7224	0.0097	0	AGQCVIGLQMGTNK (No match)
161 - 174	1492.7196	1491.7123	1491.7173	-0.0050	0	AGQCVIGLQMGTNK Oxidation (M) (No match)
175 - 187	1389.5857	1388.5784	1388.5813	-0.0028	0	CASQSGMTAYGTR (No match)
195 - 214	2250.1123	2249.1050	2249.0932	0.0118	0	NHILPPMDHSTISLQMGTNK Oxidation (M) (No match)

215 - 227	1335.6195	1334.6122	1334.6071	0.0052	0	CASQVGMTAPGTR (No match)
228 - 234	932.4961	931.4888	931.4875	0.0013	1	RHIYDTK (No match)

Mascot: http://www.matrixscience.com/

Spot 648

{MATRIX SCIENCE} Mascot Search Results

Protein View

Match to: **gi|55957280** Score: **131** Expect: **1.5e-008**
esterase D/formylglutathione hydrolase [Homo sapiens]

Nominal mass (M_r): **25695**; Calculated pI value: **6.41**
NCBI BLAST search of gi|55957280 against nr
Unformatted sequence string for pasting into other applications

Taxonomy: Homo sapiens

Fixed modifications: Carbamidomethyl (C)
Variable modifications: Oxidation (M)
Cleavage by Trypsin: cuts C-term side of KR unless next residue is P
Sequence Coverage: **12%**

Matched peptides shown in **Bold Red**

```
  1 LTCTEQNFIS KSGYHQSASE HGLVVIAPDT SPRGCNIKGE DESWDFGTGA
 51 GFYVDATEDP WKTNYRMYSY VTEELPQLIN ANFPVDPQRM SIFGHSMGGH
101 GALICALKNP GKYKSVSAFA PICNPVLCPW GKKAFSGYLG TDQSKWKAYD
151 ATHLVKSYPG SQLDILIDQG KDDQFLLDGQ LLPDNFIAAC TEKKIPVVFR
201 LQEHGRVKSG LHSLKRISLR FVVIESTTE
```

Residue Number Increasing Mass Decreasing Mass

Start - End	Observed	Mr(expt)	Mr(calc)	Delta	Miss	Sequence
12 - 33	2308.0730	2307.0657	2307.1243	-0.0586	0	SGYHQSASEHGLVVIAPDTSPR (No match)
12 - 33	2308.0730	2307.0657	2307.1243	-0.0586	0	SGYHQSASEHGLVVIAPDTSPR (Ions score 107)
194 - 200	858.5449	857.5376	857.5486	-0.0110	1	KIPVVFR (No match)

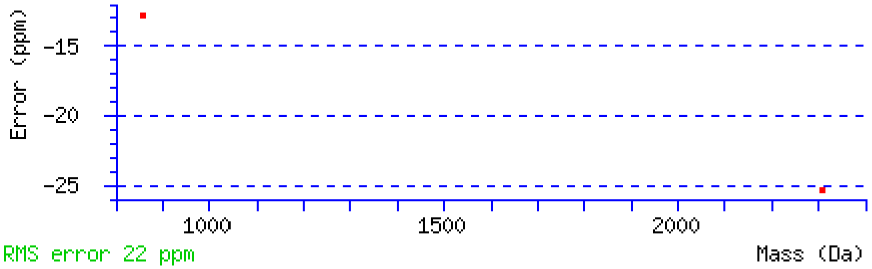

Mascot: http://www.matrixscience.com/

Spot 649

Mascot Search Results

Protein View

Match to: **gi|306785** Score: **300** Expect: **1.9e-025**
G protein beta subunit

Nominal mass (M_r): **38061**; Calculated pI value: **5.75**
NCBI BLAST search of gi|306785 against nr
Unformatted sequence string for pasting into other applications

Taxonomy: Homo sapiens

Fixed modifications: Carbamidomethyl (C)
Variable modifications: Oxidation (M)
Cleavage by Trypsin: cuts C-term side of KR unless next residue is P
Sequence Coverage: **18%**

Matched peptides shown in **Bold Red**

```
  1 MSELEQLRQE AEQLRNQIRD ARKACGDSTL TQITAGLDPV GRIQMRTRRT
 51 LRGHLAKIYA MHWGTDSRLL VSASQDGKLI IWDSYTTNKV HAIPLRSSWV
101 MTCAYAXSGN FVACGGLDNI CSIYSLKTRE GNVRVSRELP GHTGYLSCCR
151 FLDDNQIITS SGDTTCALWD IETGQQTVGF AGHSGDVMSL SLAPNGRTFV
201 SGACDASIKL WDVRDSMCRQ TFIGHESDIN AVAFFPNGYA FTTGSDDATC
251 RLFDLRADQE LLMYSHDNII CGITSVAFSR SGRLLLAGYD DFNCNIWDAM
301 KGDRAGVLAG HDNRVSCLGV TDDGMAVATG SWDSFLKIWN
```

Residue Number Increasing Mass Decreasing Mass

Start - End	Observed	Mr(expt)	Mr(calc)	Delta	Miss	Sequence
2 - 15	1728.8221	1727.8148	1727.8802	-0.0653	1	SELEQLRQEAEQLR (No match)
2 - 15	1728.8221	1727.8148	1727.8802	-0.0653	1	SELEQLRQEAEQLR (No match)
23 - 42	2059.9734	2058.9661	2059.0367	-0.0706	1	KACGDSTLTQITAGLDPVGR (No match)
23 - 42	2059.9734	2058.9661	2059.0367	-0.0706	1	KACGDSTLTQITAGLDPVGR (Ions score 55)
24 - 42	1931.8842	1930.8769	1930.9418	-0.0649	0	ACGDSTLTQITAGLDPVGR (No match)
24 - 42	1931.8842	1930.8769	1930.9418	-0.0649	0	ACGDSTLTQITAGLDPVGR (Ions score 68)
90 - 96	805.4709	804.4636	804.4970	-0.0333	0	VHAIPLR (No match)
138 - 150	1549.6345	1548.6272	1548.6813	-0.0541	0	ELPGHTGYLSCCR (No match)

138 - 150	1549.6345	1548.6272	1548.6813	-0.0541	0	ELPGHTGYLSCCR	(Ions score 100)
305 - 314	1009.4832	1008.4759	1008.5100	-0.0341	0	AGVLAGHDNR	(No match)

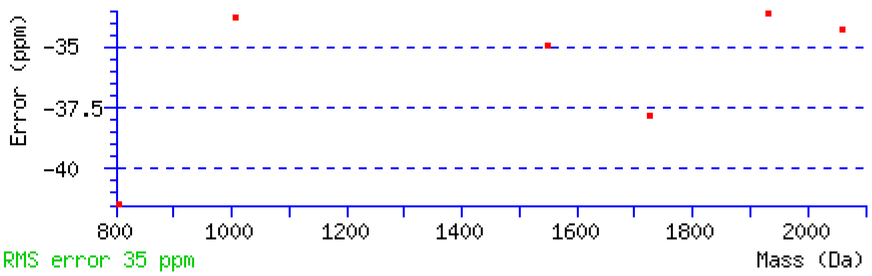

Mascot: http://www.matrixscience.com/

Spot 650

Mascot Search Results

Protein View

Match to: **gi|3212400** Score: **113** Expect: **9.6e-007**
Chain A, The Structure Of An Intermediate In Class Ii Mhc Maturation: Clip Bound To Hla-Dr3

Nominal mass (M_r): **20548**; Calculated pI value: **4.85**
NCBI BLAST search of gi|3212400 against nr
Unformatted sequence string for pasting into other applications

Taxonomy: Homo sapiens

Fixed modifications: Carbamidomethyl (C)
Variable modifications: Oxidation (M)
Cleavage by Trypsin: cuts C-term side of KR unless next residue is P
Sequence Coverage: **27%**

Matched peptides shown in **Bold Red**

```
  1 HVIIQAEFYL NPDQSGEFMF DFDGDEIFHV DMAKKETVWR LEEFGRFASF
 51 EAQGALANIA VDKANLEIMT KRSNYTPITN VPPEVTVLTN SPVELREPNV
101 LICFIDKFTP PVVNVTWLRN GKPVTTGVSE TVFLPREDHL FRKFHYLPFL
151 PSTEDVYDCR VEHWGLDEPL LKHWEF
```

Residue Number Increasing Mass Decreasing Mass

Start - End	Observed	Mr(expt)	Mr(calc)	Delta	Miss	Sequence
47 - 71	2668.2422	2667.2349	2667.3577	-0.1228	1	**FASFEAQGALANIAVDKANLEIMTK** Oxidation (M) (Ions score 43)
47 - 71	2668.2422	2667.2349	2667.3577	-0.1228	1	**FASFEAQGALANIAVDKANLEIMTK** Oxidation (M) (No match)
120 - 136	1801.9036	1800.8963	1800.9733	-0.0770	0	**NGKPVTTGVSETVFLPR** (No match)
120 - 136	1801.9036	1800.8963	1800.9733	-0.0770	0	**NGKPVTTGVSETVFLPR** (Ions score 33)
137 - 142	816.3694	815.3621	815.3926	-0.0304	0	**EDHLFR** (No match)

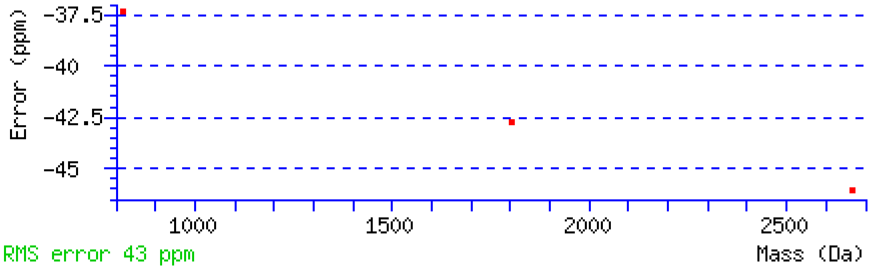

Mascot: http://www.matrixscience.com/

Spot 651

Protein View

Match to: **gi|4758018** Score: **480** Expect: **1.9e-043**
calponin 2 isoform a [Homo sapiens]

Nominal mass (M_r): **34074;** Calculated pI value: **6.95**
NCBI BLAST search of gi|4758018 against nr
Unformatted sequence string for pasting into other applications

Taxonomy: Homo sapiens
Links to retrieve other entries containing this sequence from NCBI Entrez:
gi|6226844 from Homo sapiens
gi|75055290 from Pongo pygmaeus
gi|1526432 from Homo sapiens
gi|55725108 from Pongo pygmaeus
gi|119589973 from Homo sapiens
gi|119589976 from Homo sapiens
gi|146327021 from Homo sapiens
gi|146327030 from Homo sapiens

Fixed modifications: Carbamidomethyl (C)
Variable modifications: Oxidation (M)
Cleavage by Trypsin: cuts C-term side of KR unless next residue is P
Sequence Coverage: **63%**

Matched peptides shown in **Bold Red**

```
  1 MSSTQFNKGP SYGLSAEVKN RLLSKYDPQK EAELRTWIEG LTGLSIGPDF
 51 QKGLKDGTIL CTLMNKLQPG SVPKINRSMQ NWHQLENLSN FIKAMVSYGM
101 NPVDLFEAND LFESGNMTQV QVSLLALAGK AKTKGLQSGV DIGVKYSEKQ
151 ERNFDDATMK AGQCVIGLQM GTNKCASQSG MTAYGTRRHL YDPKNHILPP
201 MDHSTISLQM GTNKCASQVG MTAPGTRRHI YDTKLGTDKC DNSSMSLQMG
251 YTQGANQSGQ VFGLGRQIYD PKYCPQGTVA DGAPSGTGDC PDPGEVPEYP
301 PYYQEEAGY
```

Residue Number Increasing Mass Decreasing Mass

Start - End	Observed	Mr(expt)	Mr(calc)	Delta	Miss	Sequence	
9 - 19	1107.5328	1106.5255	1106.5607	-0.0352	0	GPSYGLSAEVK	(No match)

26 - 35	1248.5830	1247.5757	1247.6146	-0.0388	1	**YDPQKEAELR** (No match)
26 - 35	1248.5830	1247.5757	1247.6146	-0.0388	1	**YDPQKEAELR** (Ions score 48)
36 - 52	1861.9149	1860.9076	1860.9621	-0.0545	0	**TWIEGLTGLSIGPDFQK** (No match)
36 - 52	1861.9149	1860.9076	1860.9621	-0.0545	0	**TWIEGLTGLSIGPDFQK** (Ions score 121)
53 - 66	1563.7778	1562.7705	1562.8160	-0.0455	1	**GLKDGTILCTLMNK** (No match)
53 - 66	1579.7595	1578.7522	1578.8109	-0.0587	1	**GLKDGTILCTLMNK** Oxidation (M) (No match)
78 - 93	1988.9044	1987.8971	1987.9574	-0.0603	0	**SMQNWHQLENLSNFIK** (No match)
78 - 93	2004.9031	2003.8958	2003.9523	-0.0565	0	**SMQNWHQLENLSNFIK** Oxidation (M) (Ions score 44)
78 - 93	2004.9031	2003.8958	2003.9523	-0.0565	0	**SMQNWHQLENLSNFIK** Oxidation (M) (No match)
135 - 145	1072.5648	1071.5575	1071.5923	-0.0348	0	**GLQSGVDIGVK** (No match)
146 - 152	939.4259	938.4186	938.4457	-0.0271	1	**YSEKQER** (No match)
153 - 160	957.3697	956.3624	956.3909	-0.0285	0	**NFDDATMK** Oxidation (M) (No match)
161 - 174	1476.6803	1475.6730	1475.7224	-0.0494	0	**AGQCVIGLQMGTNK** (No match)
161 - 174	1492.6697	1491.6624	1491.7173	-0.0549	0	**AGQCVIGLQMGTNK** Oxidation (M) (No match)
175 - 187	1389.5394	1388.5321	1388.5813	-0.0491	0	**CASQSGMTAYGTR** (Ions score 45)
175 - 187	1389.5394	1388.5321	1388.5813	-0.0491	0	**CASQSGMTAYGTR** (No match)
175 - 187	1405.5348	1404.5275	1404.5762	-0.0486	0	**CASQSGMTAYGTR** Oxidation (M) (No match)
175 - 187	1405.5348	1404.5275	1404.5762	-0.0486	0	**CASQSGMTAYGTR** Oxidation (M) (Ions score 33)
188 - 194	928.4724	927.4651	927.4926	-0.0275	1	**RHLYDPK** (No match)
189 - 194	772.3815	771.3742	771.3915	-0.0173	0	**HLYDPK** (No match)
195 - 214	2234.0435	2233.0362	2233.0983	-0.0621	0	**NHILPPMDHSTISLQMGTNK** (No match)
195 - 214	2250.0410	2249.0337	2249.0932	-0.0595	0	**NHILPPMDHSTISLQMGTNK** Oxidation (M) (No match)
195 - 214	2266.0359	2265.0286	2265.0882	-0.0595	0	**NHILPPMDHSTISLQMGTNK** 2 Oxidation (M) (No match)
215 - 227	1335.5671	1334.5598	1334.6071	-0.0472	0	**CASQVGMTAPGTR** (No match)
215 - 227	1351.5647	1350.5574	1350.6020	-0.0445	0	**CASQVGMTAPGTR** Oxidation (M) (No match)
215 - 228	1491.6609	1490.6536	1490.7082	-0.0546	1	**CASQVGMTAPGTRR** (No match)
215 - 228	1507.6609	1506.6536	1506.7031	-0.0495	1	**CASQVGMTAPGTRR** Oxidation (M) (No match)
228 - 234	932.4656	931.4583	931.4875	-0.0292	1	**RHIYDTK** (No match)
229 - 234	776.3697	775.3624	775.3864	-0.0240	0	**HIYDTK** (No match)
240 - 266	2893.1868	2892.1795	2892.2588	-0.0793	0	**CDNSSMSLQMGYTQGANQSGQVFGLGR** (No match)
240 - 266	2909.2180	2908.2107	2908.2537	-0.0430	0	**CDNSSMSLQMGYTQGANQSGQVFGLGR** Oxidation (M) (No match)
240 - 266	2925.2146	2924.2073	2924.2486	-0.0413	0	**CDNSSMSLQMGYTQGANQSGQVFGLGR** 2 Oxidation (M) (No match)

Spot 652

Protein View

Match to: **gi|47933379** Score: **376** Expect: **4.8e-033**
N-ethylmaleimide-sensitive factor attachment protein, alpha [Homo sapiens]

Nominal mass (M_r): **33667;** Calculated pI value: **5.23**
NCBI BLAST search of gi|47933379 against nr
Unformatted sequence string for pasting into other applications

Taxonomy: Homo sapiens
Links to retrieve other entries containing this sequence from NCBI Entrez:
gi|109125320 from Macaca mulatta
gi|114678093 from Pan troglodytes
gi|116242794 from Homo sapiens
gi|12654655 from Homo sapiens
gi|13938563 from Homo sapiens
gi|20380171 from Homo sapiens
gi|54696006 from Homo sapiens
gi|60552231 from Homo sapiens
gi|61355497 from synthetic construct
gi|90075252 from Macaca fascicularis
gi|119577895 from Homo sapiens
gi|119577899 from Homo sapiens
gi|119577900 from Homo sapiens
gi|158257920 (no taxonomy information for this entry)

Fixed modifications: Carbamidomethyl (C)
Variable modifications: Oxidation (M)
Cleavage by Trypsin: cuts C-term side of KR unless next residue is P
Sequence Coverage: **58%**

Matched peptides shown in **Bold Red**

1 MDNSGK**EAEA MALLAEAER**K VK**NSQSFFSG LFGGSSKIEE ACEIYAR**AAN
51 MFKMAKNWSA AGNAFCQAAQ LHLQLQSKHD AATCFVDAGN AFKKADPQEA
101 INCLMR**AIEI YTDMGR**FTIA AK**HHISIAEI YETELVDIEK AIAHYEQSAD**
151 **YYKGEESNSS ANK**CLLK**VAG YAALLEQYQK AIDIYEQVGT NAMDSPLLK**Y
201 SAKDYFFKAA LCHFCIDMLN AK**LAVQKYEE LFPAFSDSR**E CKLMKK**LLEA**
251 **HEEQNVDSYT ESVKEYDSIS R**LDQWLTTML LRIKK**TIQGD EEDLR**

Residue Number Increasing Mass Decreasing Mass

Start - End	Observed	Mr(expt)	Mr(calc)	Delta	Miss	Sequence
7 - 19	1419.6414	1418.6341	1418.6711	-0.0369	0	EAEAMALLAEAER Oxidation (M) (No match)
23 - 37	1549.6914	1548.6841	1548.7208	-0.0367	0	NSQSFFSGLFGGSSK (No match)
23 - 37	1549.6914	1548.6841	1548.7208	-0.0367	0	NSQSFFSGLFGGSSK (Ions score 68)
38 - 47	1253.5542	1252.5469	1252.5757	-0.0288	0	IEEACEIYAR (Ions score 28)
38 - 47	1253.5542	1252.5469	1252.5757	-0.0288	0	IEEACEIYAR (No match)
107 - 116	1184.5262	1183.5189	1183.5543	-0.0354	0	AIEIYTDMGR Oxidation (M) (Ions score 13)
107 - 116	1184.5262	1183.5189	1183.5543	-0.0354	0	AIEIYTDMGR Oxidation (M) (No match)
123 - 140	2139.0378	2138.0305	2138.0895	-0.0590	0	HHISIAEIYETELVDIEK (No match)
141 - 163	2562.0591	2561.0518	2561.1305	-0.0787	1	AIAHYEQSADYYKGEESNSSANK (No match)
168 - 180	1453.7311	1452.7238	1452.7612	-0.0374	0	VAGYAALLEQYQK (No match)
181 - 199	2093.9978	2092.9905	2093.0350	-0.0445	0	AIDIYEQVGTNAMDSPLLK Oxidation (M) (No match)
223 - 239	1999.9668	1998.9595	1999.0050	-0.0455	1	LAVQKYEELFPAFSDSR (Ions score 20)
223 - 239	1999.9668	1998.9595	1999.0050	-0.0455	1	LAVQKYEELFPAFSDSR (No match)
228 - 239	1460.6355	1459.6282	1459.6619	-0.0337	0	YEELFPAFSDSR (Ions score 81)
228 - 239	1460.6355	1459.6282	1459.6619	-0.0337	0	YEELFPAFSDSR (No match)
247 - 271	2941.2717	2940.2644	2940.3624	-0.0980	1	LLEAHEEQNVDSYTESVKEYDSISR (No match)
265 - 271	869.3724	868.3651	868.3926	-0.0275	0	EYDSISR (No match)
286 - 295	1175.5243	1174.5170	1174.5466	-0.0295	0	TIQGDEEDLR (No match)

Spot 653

Protein View

Match to: **gi|16198390** Score: **152** Expect: **1.2e-010**
Glyoxalase domain containing 4 [Homo sapiens]

Nominal mass (M_r): **33536;** Calculated pI value: **5.40**
NCBI BLAST search of gi|16198390 against nr
Unformatted sequence string for pasting into other applications

Taxonomy: Homo sapiens

Fixed modifications: Carbamidomethyl (C)
Variable modifications: Oxidation (M)
Cleavage by Trypsin: cuts C-term side of KR unless next residue is P
Sequence Coverage: **22%**

Matched peptides shown in **Bold Red**

```
  1 MAARRALHFV FKVGNRFQTA RFYRDVLGMK VLRHEEFEEG CKAACNGPYD
 51 GKWSKTMVGF GPEDDHFVAE LTYNYGVGDY KLGNDFMGIT LASSQAVSNA
101 RKLEWPLTEV AEGVFETEAP GGYKFYLQNR SLPQSDPVLK VTLAVSDLQK
151 SLNYWCNLLG MKIYEKDEEK QRALLGYADN QCKLELQGVK GGVDHAAAFG
201 RIAFSCPQKE LPDLEDLMKR ENQKILTPLV SLDTPGKATV QVVILADPDG
251 HEICFVGDEA FRELSKIDPE GSKLLDDAMA ADKSDEWFAK HNKPKASG
```

Residue Number Increasing Mass Decreasing Mass

Start - End	Observed	Mr(expt)	Mr(calc)	Delta	Miss	Sequence
5 - 12	1017.5651	1016.5578	1016.5919	-0.0341	1	RALHFVFK (No match)
31 - 42	1532.6691	1531.6618	1531.7088	-0.0470	1	VLRHEEFEEGCK (No match)
82 - 101	2067.9387	2066.9314	2067.0054	-0.0740	0	LGNDFMGITLASSQAVSNAR Oxidation (M) (No match)
82 - 101	2067.9387	2066.9314	2067.0054	-0.0740	0	LGNDFMGITLASSQAVSNAR Oxidation (M) (Ions score 67)
125 - 130	840.4068	839.3995	839.4289	-0.0294	0	FYLQNR (No match)
191 - 201	1057.4792	1056.4719	1056.5100	-0.0381	0	GGVDHAAAFGR (Ions score 9)
191 - 201	1057.4792	1056.4719	1056.5100	-0.0381	0	GGVDHAAAFGR (No match)
210 - 220	1374.6439	1373.6366	1373.6860	-0.0494	1	ELPDLEDLMKR Oxidation (M) (No match)
210 - 220	1374.6439	1373.6366	1373.6860	-0.0494	1	ELPDLEDLMKR Oxidation (M) (Ions score 17)

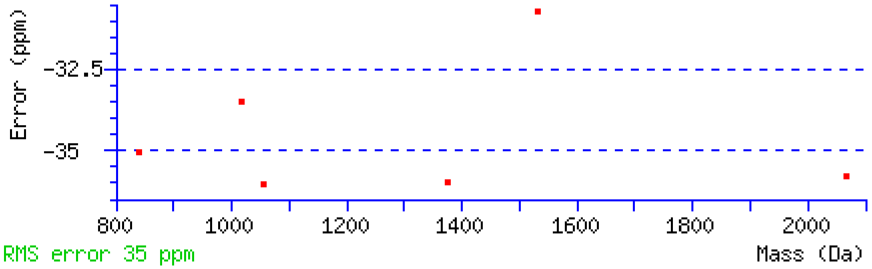

Error (ppm)
-32.5
-35
800
1000
1200
1400
1600
1800
2000
RMS error 35 ppm
Mass (Da)

Spot 654

Protein View

Match to: **gi|11056044** Score: **136** Expect: **4.8e-009**
pyrophosphatase 1 [Homo sapiens]

Nominal mass (M_r): **33095;** Calculated pI value: **5.54**
NCBI BLAST search of gi|11056044 against nr
Unformatted sequence string for pasting into other applications

Taxonomy: Homo sapiens
Links to retrieve other entries containing this sequence from NCBI Entrez:
gi|8247940 from Homo sapiens
gi|4960208 from Homo sapiens
gi|6563256 from Homo sapiens
gi|11526787 from Homo sapiens
gi|33150672 from Homo sapiens
gi|5931602 from Homo sapiens
gi|55962250 from Homo sapiens
gi|81294282 from Homo sapiens
gi|85397510 from Homo sapiens
gi|85397896 from Homo sapiens
gi|119574768 from Homo sapiens
gi|119574771 from Homo sapiens
gi|123993843 from synthetic construct
gi|127796267 from Homo sapiens
gi|127798847 from Homo sapiens

Fixed modifications: Carbamidomethyl (C)
Variable modifications: Oxidation (M)
Cleavage by Trypsin: cuts C-term side of KR unless next residue is P
Sequence Coverage: **13%**

Matched peptides shown in **Bold Red**

1 MSGFSTEER**A APFSLEYR**VF LKNEKGQYIS PFHDIPIYAD KDVFHMVVEV
51 PRWSNAKMEI ATKDPLNPIK QDVKKGKLR**Y VANLFPYK**GY IWNYGAIPQT
101 WEDPGHNDKH TGCCGDNDPI DVCEIGSKVC ARGEIIGVKV LGILAMIDEG
151 ETDWK**VIAIN VDDPDAANYN DINDVKR**LKP GYLEATVDWF RRYKVPDGKP
201 ENEFAFNAEF KDKDFAIDII KSTHDHWKAL VTKKTNGKGI SCMNTTLSES
251 PFKCDPDAAR AIVDALPPPC ESACTVPTDV DKWFHHQKN

Residue Number Increasing Mass Decreasing Mass

Start - End	Observed	Mr(expt)	Mr(calc)	Delta	Miss	Sequence	
10 - 18	1053.5338	1052.5265	1052.5290	-0.0025	0	AAPFSLEYR	(Ions score 19)
10 - 18	1053.5338	1052.5265	1052.5290	-0.0025	0	AAPFSLEYR	(No match)
80 - 88	1114.5820	1113.5747	1113.5858	-0.0111	0	YVANLFPYK	(No match)
80 - 88	1114.5820	1113.5747	1113.5858	-0.0111	0	YVANLFPYK	(Ions score 35)
156 - 177	2444.1682	2443.1609	2443.1978	-0.0369	1	VIAINVDDPDAANYNDINDVKR	(Ions score 51)
156 - 177	2444.1682	2443.1609	2443.1978	-0.0369	1	VIAINVDDPDAANYNDINDVKR	(No match)

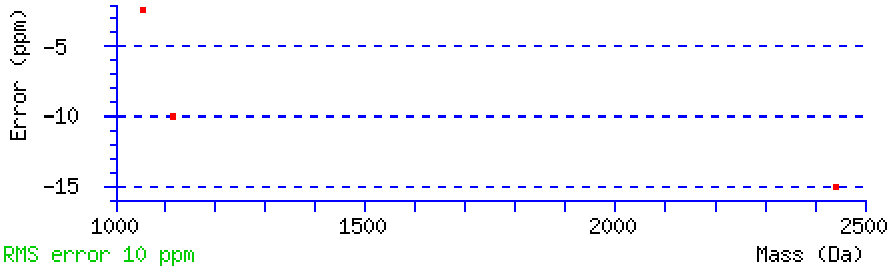

Spot 656

Protein View

Match to: **gi|55957281** Score: **237** Expect: **3.8e-019**
esterase D/formylglutathione hydrolase [Homo sapiens]

Nominal mass (M_r): **28607**; Calculated pI value: **6.29**
NCBI BLAST search of gi|55957281 against nr
Unformatted sequence string for pasting into other applications

Taxonomy: Homo sapiens

Fixed modifications: Carbamidomethyl (C)
Variable modifications: Oxidation (M)
Cleavage by Trypsin: cuts C-term side of KR unless next residue is P
Sequence Coverage: **38%**

Matched peptides shown in **Bold Red**

```
  1 MKFAVYLPPK AETGKCPALY WLSGLTCTEQ NFISKSGYHQ SASEHGLVVI
 51 APDTSPRGCN IKGEDESWDF GTGAGFYVDA TEDPWKTNYR MYSYVTEELP
101 QLINANFPVD PQRMSIFGHS MGGHGALICA LKNPGKYKSV SAFAPICNPV
151 LCPWGKKAFS GYLGTDQSKW KAYDATHLVK SYPGSQLDIL IDQGKDDQFL
201 LDGQLLPDNF IAACTEKKIP VVFRLQEGYD HSYYFIATFI TDHIRHHAKY
251 LNA
```

Residue Number Increasing Mass Decreasing Mass

Start - End	Observed	Mr(expt)	Mr(calc)	Delta	Miss	Sequence
3 - 10	934.5017	933.4944	933.5323	-0.0379	0	FAVYLPPK (No match)
3 - 15	1420.7336	1419.7263	1419.7761	-0.0498	1	FAVYLPPKAETGK (No match)
3 - 15	1420.7336	1419.7263	1419.7761	-0.0498	1	FAVYLPPKAETGK (Ions score 15)
36 - 57	2308.0591	2307.0518	2307.1243	-0.0725	0	SGYHQSASEHGLVVIAPDTSPR (No match)
36 - 57	2308.0591	2307.0518	2307.1243	-0.0725	0	SGYHQSASEHGLVVIAPDTSPR (Ions score 79)
91 - 113	2740.2380	2739.2307	2739.3213	-0.0906	0	MYSYVTEELPQLINANFPVDPQR Oxidation (M) (No match)
91 - 113	2740.2380	2739.2307	2739.3213	-0.0906	0	MYSYVTEELPQLINANFPVDPQR Oxidation (M) (Ions score 29)
158 - 169	1273.5632	1272.5559	1272.5986	-0.0426	0	AFSGYLGTDQSK (No match)
218 - 224	858.5239	857.5166	857.5486	-0.0320	1	KIPVVFR (No match)
225 - 245	2589.1570	2588.1497	2588.2335	-0.0838	0	LQEGYDHSYYFIATFITDHIR (No match)
225 - 245	2589.1570	2588.1497	2588.2335	-0.0838	0	LQEGYDHSYYFIATFITDHIR (Ions score 22)

Spot 657

Protein View

Match to: **gi|55664661** Score: **72** Expect: **0.013**
voltage-dependent anion channel 2 [Homo sapiens]

Nominal mass (M_r): **30842;** Calculated pI value: **8.00**
NCBI BLAST search of gi|55664661 against nr
Unformatted sequence string for pasting into other applications

Taxonomy: Homo sapiens
Links to retrieve other entries containing this sequence from NCBI Entrez:
gi|57209199 from Homo sapiens

Fixed modifications: Carbamidomethyl (C)
Variable modifications: Oxidation (M)
Cleavage by Trypsin: cuts C-term side of KR unless next residue is P
Sequence Coverage: **9%**

Matched peptides shown in **Bold Red**

```
  1 MATHGQTCAR PMCIPPSYAD LGKAARDIFN KGFGFGLVKL DVKTKSCSGV
 51 EFSTSGSSNT DTGKVTGTLE TKYKWCEYGL TFTEKWNTDN TLGTEIAIED
101 QICQGLKLTF DTTFSPNTGK KSGKIKSSYK RECINLGCDV DFDFAGPAIH
151 GSAVFGYEGW LAGYQMTFDS AKSKLTRNNF AVGYRTGDFQ LHTNVNDGTE
201 FGGSIYQKVC EDLDTSVNLA WTSGTNCTRF GIAAKYQLDP TASISAKVNN
251 SSLIGVGYTQ TLRPGVKLTL SALVDGKSIN AG
```

Residue Number Increasing Mass Decreasing Mass

Start - End	Observed	Mr(expt)	Mr(calc)	Delta	Miss	Sequence
178 - 185	940.4711	939.4638	939.4562	0.0076	0	NNFAVGYR (No match)
248 - 267	2103.1377	2102.1304	2102.1483	-0.0179	0	VNNSSLIGVGYTQTLRPGVK (No match)
248 - 267	2103.1377	2102.1304	2102.1483	-0.0179	0	VNNSSLIGVGYTQTLRPGVK (Ions score 43)

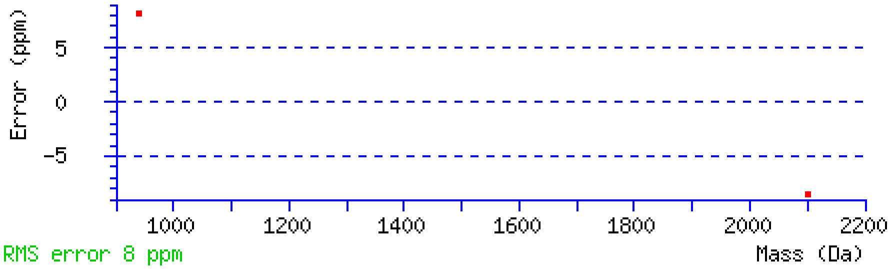

Error (ppm)
5
0
-5
1000
1200
1400
1600
1800
2000
2200
RMS error 8 ppm
Mass (Da)

Spot 658

Protein View

Match to: **gi|4507879** Score: **357** Expect: **3.8e-031**
voltage-dependent anion channel 1 [Homo sapiens]

Nominal mass (M_r): **30868;** Calculated pI value: **8.62**
NCBI BLAST search of gi|4507879 against nr
Unformatted sequence string for pasting into other applications

Taxonomy: Homo sapiens
Links to retrieve other entries containing this sequence from NCBI Entrez:
gi|130683 from Homo sapiens
gi|340199 from Homo sapiens
gi|5880859 from Homo sapiens
gi|14250132 from Homo sapiens
gi|49258076 from Homo sapiens
gi|58476644 from Homo sapiens
gi|119582685 from Homo sapiens
gi|119582686 from Homo sapiens
gi|119582687 from Homo sapiens
gi|119582689 from Homo sapiens
gi|119582690 from Homo sapiens
gi|123993015 from synthetic construct
gi|123999965 from synthetic construct

Fixed modifications: Carbamidomethyl (C)
Variable modifications: Oxidation (M)
Cleavage by Trypsin: cuts C-term side of KR unless next residue is P
Sequence Coverage: **44%**

Matched peptides shown in **Bold Red**

```
  1 MAVPPTYADL GKSARDVFTK GYGFGLIKLD LKTKSENGLE FTSSGSANTE
 51 TTKVTGSLET KYRWTEYGLT FTEKWNTDNT LGTEITVEDQ LARGLKLTFD
101 SSFSPNTGKK NAKIKTGYKR EHINLGCDMD FDIAGPSIRG ALVLGYEGWL
151 AGYQMNFETA KSRVTQSNFA VGYKTDEFQL HTNVNDGTEF GGSIYQKVNK
201 KLETAVNLAW TAGNSNTRFG IAAKYQIDPD ACFSAKVNNS SLIGLGYTQT
251 LKPGIKLTLS ALLDGKNVNA GGHKLGLGLE FQA
```

Residue Number Increasing Mass Decreasing Mass

Start - End	Observed	Mr(expt)	Mr(calc)	Delta	Miss	Sequence
21 - 28	854.4780	853.4707	853.4697	0.0010	0	GYGFGLIK (No match)
75 - 93	2175.9939	2174.9866	2175.0443	-0.0577	0	WNTDNTLGTEITVEDQLAR (Ions score 65)
97 - 110	1528.7605	1527.7532	1527.7569	-0.0036	1	LTFDSSFSPNTGKK (Ions score 42)
97 - 110	1528.7605	1527.7532	1527.7569	-0.0036	1	LTFDSSFSPNTGKK (No match)
121 - 139	2175.9939	2174.9866	2174.9724	0.0142	0	EHINLGCDMDFDIAGPSIR Oxidation (M) (No match)
164 - 174	1213.6195	1212.6122	1212.6138	-0.0016	0	VTQSNFAVGYK (No match)
175 - 197	2600.1804	2599.1731	2599.1826	-0.0095	0	TDEFQLHTNVNDGTEFGGSIYQK (No match)
225 - 236	1414.6285	1413.6212	1413.6234	-0.0022	0	YQIDPDACFSAK (Ions score 41)
225 - 236	1414.6285	1413.6212	1413.6234	-0.0022	0	YQIDPDACFSAK (No match)
237 - 256	2103.1694	2102.1621	2102.1735	-0.0113	0	VNNSSLIGLGYTQTLKPGIK (Ions score 85)
237 - 256	2103.1694	2102.1621	2102.1735	-0.0113	0	VNNSSLIGLGYTQTLKPGIK (No match)

Spot 659

Protein View

Match to: **gi|809185** Score: **145** Expect: **6.1e-010**
Chain A, The Effect Of Metal Binding On The Structure Of Annexin V And Implications For Membrane Bi

Nominal mass (M_r): **35840**; Calculated pI value: **4.94**
NCBI BLAST search of gi|809185 against nr
Unformatted sequence string for pasting into other applications

Taxonomy: Homo sapiens
Links to retrieve other entries containing this sequence from NCBI Entrez:
gi|809186 from Homo sapiens
gi|809188 from Homo sapiens
gi|809189 from Homo sapiens
gi|809190 from Homo sapiens

Fixed modifications: Carbamidomethyl (C)
Variable modifications: Oxidation (M)
Cleavage by Trypsin: cuts C-term side of KR unless next residue is P
Sequence Coverage: **8%**

Matched peptides shown in **Bold Red**

```
  1 AQVLRGTVTD FPGFDERADA ETLRKAMKGL GTDEESILTL LTSRSNAQRQ
 51 EISAAFKTLF GRDLLDDLKS ELTGKFEKLI VALMKPSRLY DAYELKHALK
101 GAGTNEKVLT EIIASRTPEE LRAIKQVYEE EYGSSLEDDV VGDTSGYYQR
151 MLVVLLQANR DPDAGIDEAQ VEQDAQALFQ AGELKWGTDE EKFITIFGTR
201 SVSHLRKVFD KYMTISGFQI EETIDRETSG NLEQLLLAVV KSIRSIPAYL
251 AETLYYAMKG AGTDDHTLIR VMVSRSEIDL FNIRKEFRKN FATSLYSMIK
301 GDTSGDYKKA LLLLCGEDD
```

Residue Number Increasing Mass Decreasing Mass

Start - End	Observed	Mr(expt)	Mr(calc)	Delta	Miss	Sequence
6 - 17	1340.5830	1339.5757	1339.6044	-0.0287	0	GTVTDFPGFDER (No match)
6 - 17	1340.5830	1339.5757	1339.6044	-0.0287	0	GTVTDFPGFDER (Ions score 17)
29 - 44	1704.8674	1703.8601	1703.8941	-0.0340	0	GLGTDEESILTLLTSR (No match)
29 - 44	1704.8674	1703.8601	1703.8941	-0.0340	0	GLGTDEESILTLLTSR (Ions score 100)

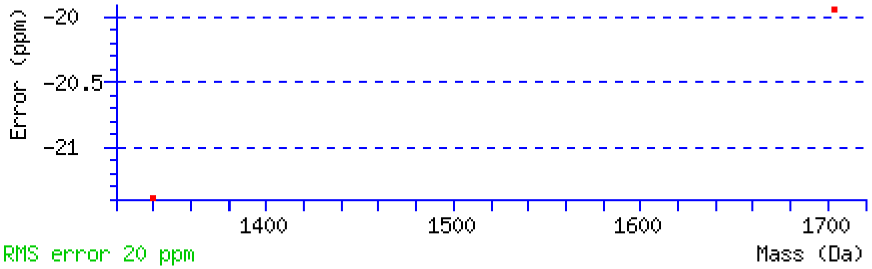
-20
-20.5
-21
Error (ppm)
1400
1500
1600
1700
RMS error 20 ppm
Mass (Da)

Spot 660

Protein View

Match to: **gi|809185** Score: **624** Expect: **7.7e-058**
Chain A, The Effect Of Metal Binding On The Structure Of Annexin V And Implications For Membrane Bi

Nominal mass (M_r): **35840**; Calculated pI value: **4.94**
NCBI BLAST search of gi|809185 against nr
Unformatted sequence string for pasting into other applications

Taxonomy: Homo sapiens
Links to retrieve other entries containing this sequence from NCBI Entrez:
gi|809186 from Homo sapiens
gi|809188 from Homo sapiens
gi|809189 from Homo sapiens
gi|809190 from Homo sapiens

Fixed modifications: Carbamidomethyl (C)
Variable modifications: Oxidation (M)
Cleavage by Trypsin: cuts C-term side of KR unless next residue is P
Sequence Coverage: **60%**

Matched peptides shown in **Bold Red**

1 AQVLR**GTVTD FPGFDER**ADA ETLRKAMK**GL GTDEESILTL LTSR**SNAQRQ
51 EISAAFKTLF GR**DLLDDLKS ELTGK**FEK**LI VALMKPSRLY DAYELK**HALK
101 GAGTNEK**VLT EIIASR**TPEE LRAIK**QVYEE EYGSSLEDDV VGDTSGYYQR**
151 **MLVVLLQANR** DPDAGIDEAQ VEQDAQALFQ AGELKWGTDE EK**FITIFGTR**
201 SVSHLRK**VFD KYMTISGFQI EETIDRETSG NLEQLLLAVV K**SIR**SIPAYL**
251 **AETLYYAMKG AGTDDHTLIR** VMVSR**SEIDL FNIRK**EFR**KN FATSLYSMIK**
301 GDTSGDYKKA LLLLCGEDD

Residue Number Increasing Mass Decreasing Mass

Start - End	Observed	Mr(expt)	Mr(calc)	Delta	Miss	Sequence
6 - 17	1340.5603	1339.5530	1339.6044	-0.0514	0	GTVTDFPGFDER (No match)
6 - 17	1340.5603	1339.5530	1339.6044	-0.0514	0	GTVTDFPGFDER (Ions score 62)
29 - 44	1704.8370	1703.8297	1703.8941	-0.0644	0	GLGTDEESILTLLTSR (Ions score 134)
29 - 44	1704.8370	1703.8297	1703.8941	-0.0644	0	GLGTDEESILTLLTSR (No match)
63 - 75	1446.7032	1445.6959	1445.7613	-0.0654	1	DLLDDLKSELTGK (No match)

79 - 88	1143.6440	1142.6367	1142.6845	-0.0478	0	**LIVALMKPSR** Oxidation (M) (No match)
89 - 96	1014.4662	1013.4589	1013.5069	-0.0480	0	**LYDAYELK** (Ions score 51)
89 - 96	1014.4662	1013.4589	1013.5069	-0.0480	0	**LYDAYELK** (No match)
108 - 116	1001.5587	1000.5514	1000.5916	-0.0402	0	**VLTEIIASR** (No match)
126 - 150	2888.1050	2887.0977	2887.2307	-0.1330	0	**QVYEEEYGSSLEDDVVGDTSGYYQR** (No match)
151 - 160	1172.6329	1171.6256	1171.6747	-0.0490	0	**MLVVLLQANR** Oxidation (M) (No match)
193 - 200	954.5005	953.4932	953.5334	-0.0402	0	**FITIFGTR** (No match)
208 - 226	2308.0310	2307.0237	2307.1092	-0.0855	1	**VFDKYMTISGFQIEETIDR** Oxidation (M) (No match)
212 - 226	1818.7985	1817.7912	1817.8505	-0.0593	0	**YMTISGFQIEETIDR** Oxidation (M) (No match)
227 - 241	1613.8383	1612.8310	1612.9035	-0.0725	0	**ETSGNLEQLLLAVVK** (No match)
245 - 259	1749.8068	1748.7995	1748.8694	-0.0699	0	**SIPAYLAETLYYAMK** Oxidation (M) (No match)
260 - 270	1155.5352	1154.5279	1154.5680	-0.0400	0	**GAGTDDHTLIR** (No match)
276 - 284	1106.5393	1105.5320	1105.5767	-0.0447	0	**SEIDLFNIR** (Ions score 71)
276 - 284	1106.5393	1105.5320	1105.5767	-0.0447	0	**SEIDLFNIR** (No match)
276 - 285	1234.6309	1233.6236	1233.6717	-0.0481	1	**SEIDLFNIRK** (No match)
276 - 285	1234.6309	1233.6236	1233.6717	-0.0481	1	**SEIDLFNIRK** (Ions score 70)
289 - 300	1418.6763	1417.6690	1417.7275	-0.0585	1	**KNFATSLYSMIK** Oxidation (M) (No match)
290 - 300	1290.5868	1289.5795	1289.6325	-0.0530	0	**NFATSLYSMIK** Oxidation (M) (No match)

Spot 662

Protein View

Match to: **gi|4826659** Score: **100** Expect: **2e-005**
F-actin capping protein beta subunit [Homo sapiens]

Nominal mass (M_r): **30952;** Calculated pI value: **5.69**
NCBI BLAST search of gi|4826659 against nr
Unformatted sequence string for pasting into other applications

Taxonomy: Homo sapiens
Links to retrieve other entries containing this sequence from NCBI Entrez:
gi|6753262 from Mus musculus
gi|54400732 from Rattus norvegicus
gi|81883689 from Rattus norvegicus
gi|158563922 (no taxonomy information for this entry)
gi|500749 from Mus musculus
gi|595257 from Homo sapiens
gi|12805189 from Mus musculus
gi|53734561 from Rattus norvegicus
gi|54695810 from Homo sapiens
gi|61355538 from synthetic construct
gi|74142535 from Mus musculus
gi|74219999 from Mus musculus
gi|78395087 from Homo sapiens
gi|80475875 from Homo sapiens
gi|80479060 from Homo sapiens
gi|119615296 from Homo sapiens
gi|123229611 from Mus musculus
gi|148681351 from Mus musculus
gi|149024409 from Rattus norvegicus

Fixed modifications: Carbamidomethyl (C)
Variable modifications: Oxidation (M)
Cleavage by Trypsin: cuts C-term side of KR unless next residue is P
Sequence Coverage: **12%**

Matched peptides shown in **Bold Red**

1 MSDQQLDCAL DLMR**RLPPQQ IEK**NLSDLID LVPSLCEDLL SSVDQPLKIA
51 RDKVVGKDYL LCDYNRDGDS YRSPWSNKYD PPLEDGAMPS ARLR**KLEVEA**
101 **NNAFDQYR**DL YFEGGVSSVY LWDLDHGFAG VILIKKAGDG SKKIKGCWDS
151 IHVVEVQEKS SGRTAHYKLT STVMLWLQTN KSGSGTMNLG GSLTRQMEKD
201 ETVSDCSPHI ANIGRLVEDM ENKIR**STLNE IYFGK**TKDIV NGLRSVQTFA

251 DKSKQEALKN DLVEALKRKQ QC

Residue Number Increasing Mass Decreasing Mass

Start - End	Observed	Mr(expt)	Mr(calc)	Delta	Miss	Sequence
15 - 23	1108.6146	1107.6073	1107.6400	-0.0327	1	RLPPQQIEK (No match)
95 - 108	1696.7876	1695.7803	1695.8216	-0.0412	1	KLEVEANNAFDQYR (Ions score 31)
95 - 108	1696.7876	1695.7803	1695.8216	-0.0412	1	KLEVEANNAFDQYR (No match)
96 - 108	1568.6956	1567.6883	1567.7266	-0.0383	0	LEVEANNAFDQYR (Ions score 35)
96 - 108	1568.6956	1567.6883	1567.7266	-0.0383	0	LEVEANNAFDQYR (No match)
226 - 235	1171.5677	1170.5604	1170.5920	-0.0316	0	STLNEIYFGK (No match)

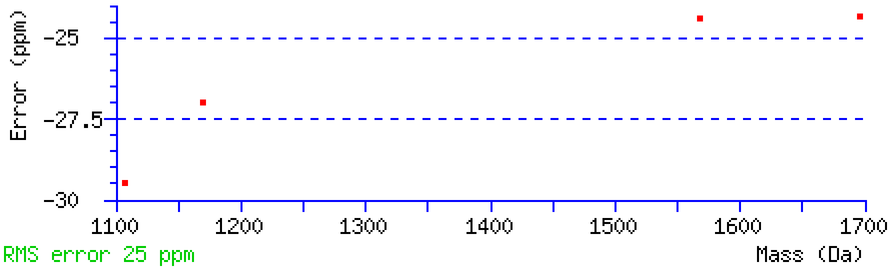

RMS error 25 ppm

Spot 663

Protein View

Match to: **gi|4826659** Score: **333** Expect: **9.6e-029**
F-actin capping protein beta subunit [Homo sapiens]

Nominal mass (M_r): **30952;** Calculated pI value: **5.69**
NCBI BLAST search of gi|4826659 against nr
Unformatted sequence string for pasting into other applications

Taxonomy: Homo sapiens
Links to retrieve other entries containing this sequence from NCBI Entrez:
gi|6753262 from Mus musculus
gi|54400732 from Rattus norvegicus
gi|81883689 from Rattus norvegicus
gi|158563922 (no taxonomy information for this entry)
gi|500749 from Mus musculus
gi|595257 from Homo sapiens
gi|12805189 from Mus musculus
gi|53734561 from Rattus norvegicus
gi|54695810 from Homo sapiens
gi|61355538 from synthetic construct
gi|74142535 from Mus musculus
gi|74219999 from Mus musculus
gi|78395087 from Homo sapiens
gi|80475875 from Homo sapiens
gi|80479060 from Homo sapiens
gi|119615296 from Homo sapiens
gi|123229611 from Mus musculus
gi|148681351 from Mus musculus
gi|149024409 from Rattus norvegicus

Fixed modifications: Carbamidomethyl (C)
Variable modifications: Oxidation (M)
Cleavage by Trypsin: cuts C-term side of KR unless next residue is P
Sequence Coverage: **26%**

Matched peptides shown in **Bold Red**

1 MSDQQLDCAL DLMR**RLPPQQ IEK**NLSDLID LVPSLCEDLL SSVDQPLKIA
51 RDKVVGK**DYL LCDYNR**DGDS YR**SPWSNKYD PPLEDGAMPS AR**LR**KLEVEA**
101 **NNAFDQYR**DL YFEGGVSSVY LWDLDHGFAG VILIKKAGDG SKKIKGCWDS
151 IHVVEVQEKS SGRTAHYKLT STVMLWLQTN KSGSGTMNLG GSLTRQMEKD
201 ETVSDCSPHI ANIGRLVEDM ENKIR**STLNE IYFGKTKDIV NGLR**SVQTFA

251 DKSKQEALKN DLVEALKRKQ QC

Residue Number Increasing Mass Decreasing Mass

Start - End	Observed	Mr(expt)	Mr(calc)	Delta	Miss	Sequence
15 - 23	1108.6384	1107.6311	1107.6400	-0.0089	1	**RLPPQQIEK** (Ions score 25)
15 - 23	1108.6384	1107.6311	1107.6400	-0.0089	1	**RLPPQQIEK** (No match)
58 - 66	1231.5338	1230.5265	1230.5339	-0.0074	0	**DYLLCDYNR** (No match)
58 - 66	1231.5338	1230.5265	1230.5339	-0.0074	0	**DYLLCDYNR** (Ions score 55)
73 - 92	2233.9895	2232.9822	2233.0109	-0.0287	1	**SPWSNKYDPPLEDGAMPSAR** Oxidation (M) (No match)
95 - 108	1696.8088	1695.8015	1695.8216	-0.0200	1	**KLEVEANNAFDQYR** (Ions score 63)
95 - 108	1696.8088	1695.8015	1695.8216	-0.0200	1	**KLEVEANNAFDQYR** (No match)
96 - 108	1568.7166	1567.7093	1567.7266	-0.0173	0	**LEVEANNAFDQYR** (No match)
96 - 108	1568.7166	1567.7093	1567.7266	-0.0173	0	**LEVEANNAFDQYR** (Ions score 97)
226 - 235	1171.5854	1170.5781	1170.5920	-0.0139	0	**STLNEIYFGK** (No match)
236 - 244	1015.5835	1014.5762	1014.5821	-0.0059	1	**TKDIVNGLR** (No match)

Spot 664

Protein View

Match to: **gi|119574080** Score: **275** Expect: **6.1e-023**
guanine nucleotide binding protein (G protein), beta polypeptide 2-like 1, isoform CRA_d [Homo sapi

Nominal mass (M_r): **30942**; Calculated pI value: **7.03**
NCBI BLAST search of gi|119574080 against nr
Unformatted sequence string for pasting into other applications

Taxonomy: Homo sapiens

Fixed modifications: Carbamidomethyl (C)
Variable modifications: Oxidation (M)
Cleavage by Trypsin: cuts C-term side of KR unless next residue is P
Sequence Coverage: **23%**

Matched peptides shown in **Bold Red**

```
  1 MWKLTRDETN YGIPQRALRG HSHFVSDVVI SSDGQFALSG SWDGTLRLWD
 51 LTTGTTTRRF VGHTKDVLSV AFSSDNRQIV SGSRDKTIKL WNTLGVCKYT
101 VQDESHSEWV SCVRFSPNSS NPIIVSCGWD KLVKVWNLAN CKLKTNHIGH
151 TGYLNTVTVS PDGSLCASGG KDGQAMLWDL NEGKHLYTLD GGDIINALCF
201 SPNRYWLCAA TGPSIKIWDL EGKIIVDELK QEVISTSSKA EPPQCTSLAW
251 SADGQTLFAG YTDNLVRVWQ VTIGTR
```

Residue Number Increasing Mass Decreasing Mass

Start - End	Observed	Mr(expt)	Mr(calc)	Delta	Miss	Sequence
1 - 6	850.4888	849.4815	849.4531	0.0285	1	MWKLTR Oxidation (M) (No match)
4 - 16	1562.7261	1561.7188	1561.7848	-0.0660	1	LTRDETNYGIPQR (No match)
4 - 16	1562.7261	1561.7188	1561.7848	-0.0660	1	LTRDETNYGIPQR (Ions score 27)
7 - 16	1192.5054	1191.4981	1191.5520	-0.0538	0	DETNYGIPQR (No match)
7 - 16	1192.5054	1191.4981	1191.5520	-0.0538	0	DETNYGIPQR (Ions score 81)
66 - 77	1309.5782	1308.5709	1308.6309	-0.0600	0	DVLSVAFSSDNR (No match)
66 - 77	1309.5782	1308.5709	1308.6309	-0.0600	0	DVLSVAFSSDNR (Ions score 49)
185 - 204	2276.0081	2275.0008	2275.1055	-0.1047	0	HLYTLDGGDIINALCFSPNR (No match)
224 - 239	1788.9119	1787.9046	1787.9880	-0.0834	1	IIVDELKQEVISTSSK (No match)
224 - 239	1788.9119	1787.9046	1787.9880	-0.0834	1	IIVDELKQEVISTSSK (Ions score 56)

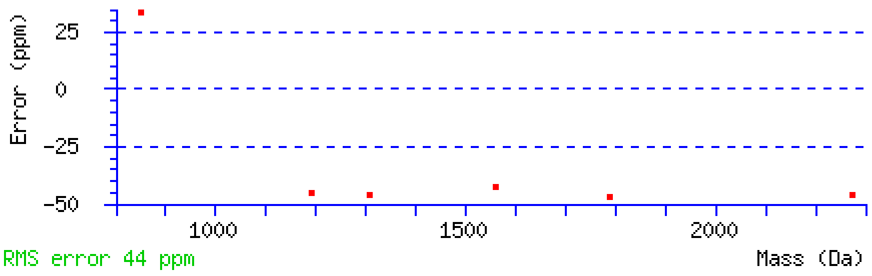
Error (ppm)
25
0
-25
-50
1000
1500
2000
RMS error 44 ppm
Mass (Da)

Spot 665

Protein View

Match to: **gi|6912494** Score: **199** Expect: **2.4e-015**
microtubule-associated protein, RP/EB family, member 1 [Homo sapiens]

Nominal mass (M_r): **30151**; Calculated pI value: **5.02**
NCBI BLAST search of gi|6912494 against nr
Unformatted sequence string for pasting into other applications

Taxonomy: Homo sapiens
Links to retrieve other entries containing this sequence from NCBI Entrez:
gi|20138589 from Homo sapiens
gi|998357 from Homo sapiens
gi|5748523 from Homo sapiens
gi|76779251 from Homo sapiens
gi|80475947 from Homo sapiens
gi|119596754 from Homo sapiens
gi|119596755 from Homo sapiens

Fixed modifications: Carbamidomethyl (C)
Variable modifications: Oxidation (M)
Cleavage by Trypsin: cuts C-term side of KR unless next residue is P
Sequence Coverage: **17%**

Matched peptides shown in **Bold Red**

```
  1 MAVNVYSTSV TSDNLSRHDM LAWINESLQL NLTKIEQLCS GAAYCQFMDM
 51 LFPGSIALKK VKFQAKLEHE YIQNFKILQA GFKRMGVDKI IPVDKLVKGK
101 FQDNFEFVQW FKKFFDANYD GKDYDPVAAR QGQETAVAPS LVAPALNKPK
151 KPLTSSSAAP QRPISTQRTA AAPKAGPGVV RKNPGVGNGD DEAAELMQQV
201 NVLKLTVEDL EKERDFYFGK LRNIELICQE NEGENDPVLQ RIVDILYATD
251 EGFVIPDEGG PQEEQEEY
```

Residue Number Increasing Mass Decreasing Mass

Start - End	Observed	Mr(expt)	Mr(calc)	Delta	Miss	Sequence
67 - 76	1320.6471	1319.6398	1319.6509	-0.0111	0	LEHEYIQNFK (Ions score 38)
67 - 76	1320.6471	1319.6398	1319.6509	-0.0111	0	LEHEYIQNFK (No match)
114 - 130	1963.8685	1962.8612	1962.8747	-0.0135	1	FFDANYDGKDYDPVAAR (Ions score 58)

114 - 130	1963.8685	1962.8612	1962.8747	-0.0135	1	FFDANYDGKDYDPVAAR	(No match)
223 - 241	2270.0471	2269.0398	2269.0644	-0.0246	0	NIELICQENEGENDPVLQR	(Ions score 62)
223 - 241	2270.0471	2269.0398	2269.0644	-0.0246	0	NIELICQENEGENDPVLQR	(No match)

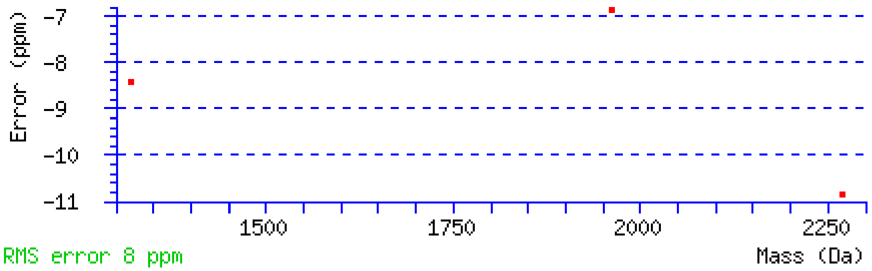

RMS error 8 ppm

Spot 666

Protein View

Match to: **gi|66360348** Score: **247** Expect: **3.8e-020**
Chain A, Crystal Structure Of Human Cbr1 In Complex With Hydroxy-Pp

Nominal mass (M_r): **30510**; Calculated pI value: **8.55**
NCBI BLAST search of gi|66360348 against nr
Unformatted sequence string for pasting into other applications

Taxonomy: Homo sapiens

Fixed modifications: Carbamidomethyl (C)
Variable modifications: Oxidation (M)
Cleavage by Trypsin: cuts C-term side of KR unless next residue is P
Sequence Coverage: **37%**

Matched peptides shown in **Bold Red**

```
  1 SSGIHVALVT GGNKGIGLAI VRDLCRLFSG DVVLTARDVT RGQAAVQQLQ
 51 AEGLSPRFHQ LDIDDLQSIR ALRDFLRKEY GGLDVLVNNA GIAFKVADPT
101 PFHIQAEVTM KTNFFGTRDV CTELLPLIKP QGRVVNVSSI MSVRALKSCS
151 PELQQKFRSE TITEEELVGL MNKFVEDTKK GVHQKEGWPS SAYGVTKIGV
201 TVLSRIHARK LSEQRKGDKI LLNACCPGWV RTDMAGPKAT KSPEEGAETP
251 VYLALLPPDA EGPHGQFVSE KRVEQW
```

Residue Number Increasing Mass Decreasing Mass

Start - End	Observed	Mr(expt)	Mr(calc)	Delta	Miss	Sequence
15 - 22	798.5049	797.4976	797.5123	-0.0146	0	GIGLAIVR (No match)
27 - 37	1177.6409	1176.6336	1176.6502	-0.0166	0	LFSGDVVLTAR (No match)
27 - 37	1177.6409	1176.6336	1176.6502	-0.0166	0	LFSGDVVLTAR (Ions score 4)
42 - 57	1652.8359	1651.8286	1651.8641	-0.0355	0	GQAAVQQLQAEGLSPR (Ions score 52)
42 - 57	1652.8359	1651.8286	1651.8641	-0.0355	0	GQAAVQQLQAEGLSPR (No match)
58 - 70	1599.7806	1598.7733	1598.8052	-0.0319	0	FHQLDIDDLQSIR (No match)
58 - 70	1599.7806	1598.7733	1598.8052	-0.0319	0	FHQLDIDDLQSIR (Ions score 57)
79 - 95	1779.8984	1778.8911	1778.9202	-0.0291	0	EYGGLDVLVNNAGIAFK (No match)
119 - 133	1738.9143	1737.9070	1737.9447	-0.0377	0	DVCTELLPLIKPQGR (Ions score 27)
119 - 133	1738.9143	1737.9070	1737.9447	-0.0377	0	DVCTELLPLIKPQGR (No match)
134 - 144	1206.6270	1205.6197	1205.6437	-0.0240	0	VVNVSSIMSVR Oxidation (M) (No match)

220 - 231	1458.7045	1457.6972	1457.7271	-0.0299	0	ILLNACCPGWVR	(No match)
220 - 231	1458.7045	1457.6972	1457.7271	-0.0299	0	ILLNACCPGWVR	(No match)

Spot 668

Protein View

Match to: **gi|1703319** Score: **398** Expect: **3e-035**
Annexin A4 (Annexin-4) (Annexin IV) (Lipocortin IV) (Endonexin I) (Chromobindin-4) (Protein II) (P3

Nominal mass (M_r): **36088**; Calculated pI value: **5.84**
NCBI BLAST search of gi|1703319 against nr
Unformatted sequence string for pasting into other applications

Taxonomy: Homo sapiens

Fixed modifications: Carbamidomethyl (C)
Variable modifications: Oxidation (M)
Cleavage by Trypsin: cuts C-term side of KR unless next residue is P
Sequence Coverage: **29%**

Matched peptides shown in **Bold Red**

```
  1 MATKGGTVKA ASGFNAMEDA QTLRKAMKGL GTDEDAIISV LAYRNTAQRQ
 51 EIRTAYKSTI GRDLIDDLKS ELSGNFEQVI VGMMTPTVLY DVQELRRAMK
101 GAGTDEGCLI EILASRTPEE IRRISQTYQQ QYGRSLEDDI RSDTSFMFQR
151 VLVSLSAGGR DEGNYLDDAL VRQDAQDLYE AGEKKWGTDE VKFLTVLCSR
201 NRNHLLHVFD EYKRISQKDI EQSIKSETSG SFEDALLAIV KCMRNKSAYF
251 AEKLYKSMKG LGTDDNTLIR VMVSRAEIDM LDIRAHFKRL YGKSLYSFIK
301 GDTSGDYRKV LLVLCGGDD
```

Residue Number Increasing Mass Decreasing Mass

Start - End	Observed	Mr(expt)	Mr(calc)	Delta	Miss	Sequence
29 - 44	1692.8682	1691.8609	1691.8729	-0.0120	0	GLGTDEDAIISVLAYR (No match)
29 - 44	1692.8682	1691.8609	1691.8729	-0.0120	0	GLGTDEDAIISVLAYR (Ions score 96)
101 - 116	1661.8060	1660.7987	1660.8090	-0.0102	0	GAGTDEGCLIEILASR (No match)
101 - 116	1661.8060	1660.7987	1660.8090	-0.0102	0	GAGTDEGCLIEILASR (Ions score 61)
124 - 134	1371.6583	1370.6510	1370.6578	-0.0068	0	ISQTYQQQYGR (Ions score 32)
124 - 134	1371.6583	1370.6510	1370.6578	-0.0068	0	ISQTYQQQYGR (No match)
142 - 150	1134.4829	1133.4756	1133.4811	-0.0055	0	SDTSFMFQR Oxidation (M) (No match)
151 - 172	2319.1731	2318.1658	2318.1865	-0.0207	1	VLVSLSAGGRDEGNYLDDALVR (No match)
161 - 172	1379.6394	1378.6321	1378.6364	-0.0043	0	DEGNYLDDALVR (No match)
193 - 200	995.5361	994.5288	994.5269	0.0019	0	FLTVLCSR (No match)

193 - 200	995.5361	994.5288	994.5269	0.0019	0	**FLTVLCSR**	(Ions score 15)
203 - 214	1570.7997	1569.7924	1569.8051	-0.0127	1	**NHLLHVFDEYKR**	(No match)
203 - 214	1570.7997	1569.7924	1569.8051	-0.0127	1	**NHLLHVFDEYKR**	(Ions score 76)

Spot 669

Protein View

Match to: **gi|77019293** Score: **270** Expect: **1.9e-022**
MHC class II antigen [Homo sapiens]

Nominal mass (M_r): **21042**; Calculated pI value: **6.09**
NCBI BLAST search of gi|77019293 against nr
Unformatted sequence string for pasting into other applications

Taxonomy: Homo sapiens

Fixed modifications: Carbamidomethyl (C)
Variable modifications: Oxidation (M)
Cleavage by Trypsin: cuts C-term side of KR unless next residue is P
Sequence Coverage: **38%**

Matched peptides shown in **Bold Red**

```
  1 RFLWQGKYKC HFFNGTERVQ FLERLFYNQE EFVRFDSDVG EYRAVTELGR
 51 PVAESWNSQK DILEDRRGQV DNYCRHNYGV GESFTVQRRV HPEVTVYPAK
101 TQPLQHHNLL VCSVSGFYPG SIEVRWFRNG QEEKAGVVST GLIQNGDWTF
151 QTLVMLETVP RSGEVYTCQV EHPSVMSPL
```

Residue Number Increasing Mass Decreasing Mass

Start - End	Observed	Mr(expt)	Mr(calc)	Delta	Miss	Sequence
25 - 34	1344.6254	1343.6181	1343.6509	-0.0328	0	LFYNQEEFVR (Ions score 33)
25 - 34	1344.6254	1343.6181	1343.6509	-0.0328	0	LFYNQEEFVR (No match)
35 - 43	1087.4454	1086.4381	1086.4617	-0.0236	0	FDSDVGEYR (No match)
35 - 43	1087.4454	1086.4381	1086.4617	-0.0236	0	FDSDVGEYR (Ions score 26)
76 - 88	1493.6759	1492.6686	1492.7058	-0.0372	0	HNYGVGESFTVQR (Ions score 87)
76 - 88	1493.6759	1492.6686	1492.7058	-0.0372	0	HNYGVGESFTVQR (No match)
89 - 100	1395.7433	1394.7360	1394.7670	-0.0309	1	RVHPEVTVYPAK (No match)
101 - 125	2838.3586	2837.3513	2837.4282	-0.0769	0	TQPLQHHNLLVCSVSGFYPGSIEVR (Ions score 50)
101 - 125	2838.3586	2837.3513	2837.4282	-0.0769	0	TQPLQHHNLLVCSVSGFYPGSIEVR (No match)

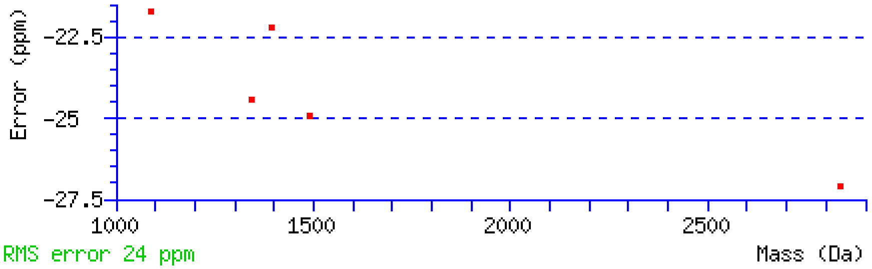

Error (ppm)
-22.5
-25
-27.5
1000
1500
2000
2500
Mass (Da)
RMS error 24 ppm

Spot 670

Protein View

Match to: **gi|157168362** Score: **481** Expect: **1.5e-043**
nucleoside phosphorylase [Homo sapiens]

Nominal mass (M_r): **32325**; Calculated pI value: **6.45**
NCBI BLAST search of gi|157168362 against nr
Unformatted sequence string for pasting into other applications
Links to retrieve other entries containing this sequence from NCBI Entrez:
gi|108935929 from Homo sapiens
gi|47115151 from Homo sapiens
gi|55925942 from Homo sapiens
gi|74353768 from Homo sapiens
gi|74355301 from Homo sapiens
gi|76779255 from Homo sapiens
gi|119586862 from Homo sapiens
gi|119586863 from Homo sapiens

Fixed modifications: Carbamidomethyl (C)
Variable modifications: Oxidation (M)
Cleavage by Trypsin: cuts C-term side of KR unless next residue is P
Sequence Coverage: **59%**

Matched peptides shown in **Bold Red**

```
  1 MENGYTYEDY KNTAEWLLSH TKHRPQVAII CGSGLGGLTD KLTQAQIFDY
 51 GEIPNFPRST VPGHAGRLVF GFLNGRACVM MQGRFHMYEG YPLWKVTFPV
101 RVFHLLGVDT LVVTNAAGGL NPKFEVGDIM LIRDHINLPG FSGQNPLRGP
151 NDERFGDRFP AMSDAYDRTM RQRALSTWKQ MGEQRELQEG TYVMVAGPSF
201 ETVAECRVLQ KLGADAVGMS TVPEVIVARH CGLRVFGFSL ITNKVIMDYE
251 SLEKANHEEV LAAGKQAAQK LEQFVSILMA SIPLPDKAS
```

Residue Number Increasing Mass Decreasing Mass

Start - End	Observed	Mr(expt)	Mr(calc)	Delta	Miss	Sequence
23 - 41	1978.9628	1977.9555	1978.0417	-0.0862	0	HRPQVAIICGSGLGGLTDK (No match)
42 - 58	2008.9235	2007.9162	2008.0054	-0.0891	0	LTQAQIFDYGEIPNFPR (Ions score 74)
42 - 58	2008.9235	2007.9162	2008.0054	-0.0891	0	LTQAQIFDYGEIPNFPR (No match)
59 - 67	881.4152	880.4079	880.4515	-0.0435	0	STVPGHAGR (No match)

68 - 76	1022.5313	1021.5240	1021.5708	-0.0468	0	LVFGFLNGR (Ions score 82)
68 - 76	1022.5313	1021.5240	1021.5708	-0.0468	0	LVFGFLNGR (No match)
102 - 123	2235.1465	2234.1392	2234.2422	-0.1030	0	VFHLLGVDTLVVTNAAGGLNPK (No match)
124 - 133	1208.5726	1207.5653	1207.6270	-0.0617	0	FEVGDIMLIR Oxidation (M) (Ions score 46)
124 - 133	1208.5726	1207.5653	1207.6270	-0.0617	0	FEVGDIMLIR Oxidation (M) (No match)
134 - 148	1664.7784	1663.7711	1663.8430	-0.0719	0	DHINLPGFSGQNPLR (No match)
134 - 148	1664.7784	1663.7711	1663.8430	-0.0719	0	DHINLPGFSGQNPLR (Ions score 56)
134 - 154	2333.0264	2332.0191	2332.1308	-0.1116	1	DHINLPGFSGQNPLRGPNDER (No match)
155 - 168	1663.6411	1662.6338	1662.7096	-0.0758	1	FGDRFPAMSDAYDR Oxidation (M) (No match)
159 - 168	1188.4374	1187.4301	1187.4917	-0.0616	0	FPAMSDAYDR Oxidation (M) (No match)
186 - 207	2489.0225	2488.0152	2488.1249	-0.1097	0	ELQEGTYVMVAGPSFETVAECR Oxidation (M) (No match)
212 - 229	1800.8723	1799.8650	1799.9450	-0.0800	0	LGADAVGMSTVPEVIVAR Oxidation (M) (No match)
212 - 229	1800.8723	1799.8650	1799.9450	-0.0800	0	LGADAVGMSTVPEVIVAR Oxidation (M) (Ions score 65)
235 - 244	1125.5737	1124.5664	1124.6229	-0.0565	0	VFGFSLITNK (No match)

Spot 671

Protein View

Match to: **gi|5031599** Score: **528** Expect: **3e-048**
actin related protein 2/3 complex subunit 2 [Homo sapiens]

Nominal mass (M_r): **34426**; Calculated pI value: **6.84**
NCBI BLAST search of gi|5031599 against nr
Unformatted sequence string for pasting into other applications

Taxonomy: Homo sapiens
Links to retrieve other entries containing this sequence from NCBI Entrez:
gi|23238211 from Homo sapiens
gi|114583271 from Pan troglodytes
gi|114583273 from Pan troglodytes
gi|114583275 from Pan troglodytes
gi|3121764 from Homo sapiens
gi|62899830 from Pongo pygmaeus
gi|2282036 from Homo sapiens
gi|12653625 from Homo sapiens
gi|30582635 from Homo sapiens
gi|55732234 from Pongo pygmaeus
gi|60655211 from synthetic construct
gi|60655213 from synthetic construct
gi|62702288 from Homo sapiens
gi|119590997 from Homo sapiens
gi|119590999 from Homo sapiens
gi|158261559 (no taxonomy information for this entry)

Fixed modifications: Carbamidomethyl (C)
Variable modifications: Oxidation (M)
Cleavage by Trypsin: cuts C-term side of KR unless next residue is P
Sequence Coverage: **39%**

Matched peptides shown in **Bold Red**

```
  1 MILLEVNNRI IEETLALKFE NAAAGNKPEA VEVTFADFDG VLYHISNPNG
 51 DKTKVMVSIS LKFYKELQAH GADELLKRVY GSFLVNPESG YNVSLLYDLE
101 NLPASKDSIV HQAGMLKRNC FASVFEKYFQ FQEEGKEGEN RAVIHYRDDE
151 TMYVESKKDR VTVVFSTVFK DDDDVVIGKV FMQEFKEGRR ASHTAPQVLF
201 SHREPPLELK DTDAAVGDNI GYITFVLFPR HTNASARDNT INLIHTFRDY
251 LHYHIKCSKA YIHTRMRAKT SDFLKVLNRA RPDAEKKEMK TITGKTFSSR
301
```

Residue Number Increasing Mass Decreasing Mass

Start - End	Observed	Mr(expt)	Mr(calc)	Delta	Miss	Sequence
1 - 9	1117.5873	1116.5800	1116.5961	-0.0160	0	MILLEVNNR Oxidation (M) (No match)
66 - 78	1479.7722	1478.7649	1478.7841	-0.0191	1	ELQAHGADELLKR (No match)
119 - 127	1101.4960	1100.4887	1100.4960	-0.0073	0	NCFASVFEK (No match)
128 - 141	1760.7595	1759.7522	1759.7801	-0.0279	1	YFQFQEEGKEGENR (Ions score 85)
128 - 141	1760.7595	1759.7522	1759.7801	-0.0279	1	YFQFQEEGKEGENR (No match)
137 - 147	1343.6895	1342.6822	1342.6741	0.0081	1	EGENRAVIHYR (No match)
180 - 186	944.4419	943.4346	943.4473	-0.0127	0	VFMQEFK Oxidation (M) (No match)
191 - 203	1450.7357	1449.7284	1449.7477	-0.0192	0	ASHTAPQVLFSHR (No match)
191 - 203	1450.7357	1449.7284	1449.7477	-0.0192	0	ASHTAPQVLFSHR (Ions score 77)
191 - 210	2257.1697	2256.1624	2256.2014	-0.0390	1	ASHTAPQVLFSHREPPLELK (No match)
211 - 230	2184.0581	2183.0508	2183.0898	-0.0390	0	DTDAAVGDNIGYITFVLFPR (Ions score 124)
211 - 230	2184.0581	2183.0508	2183.0898	-0.0390	0	DTDAAVGDNIGYITFVLFPR (No match)
238 - 248	1343.6895	1342.6822	1342.6993	-0.0171	0	DNTINLIHTFR (Ions score 40)
238 - 256	2413.1914	2412.1841	2412.2338	-0.0497	1	DNTINLIHTFRDYLHYHIK (No match)
238 - 256	2413.1914	2412.1841	2412.2338	-0.0497	1	DNTINLIHTFRDYLHYHIK (Ions score 20)

Spot 672

Protein View

Match to: **gi|4507651** Score: **428** Expect: **3e-038**
tropomyosin 4 [Homo sapiens]

Nominal mass (M_r): **28619**; Calculated pI value: **4.67**
NCBI BLAST search of gi|4507651 against nr
Unformatted sequence string for pasting into other applications

Taxonomy: Homo sapiens
Links to retrieve other entries containing this sequence from NCBI Entrez:
gi|47523730 from Sus scrofa
gi|54039746 from Sus scrofa
gi|54039751 from Homo sapiens
gi|37202 from Homo sapiens
gi|3661527 from Sus scrofa
gi|22902218 from Homo sapiens
gi|45501027 from Homo sapiens
gi|54696136 from Homo sapiens
gi|61356621 from synthetic construct
gi|90078432 from Macaca fascicularis
gi|119604927 from Homo sapiens
gi|119604929 from Homo sapiens

Fixed modifications: Carbamidomethyl (C)
Variable modifications: Oxidation (M)
Cleavage by Trypsin: cuts C-term side of KR unless next residue is P
Sequence Coverage: **53%**

Matched peptides shown in **Bold Red**

1 MAGLNSLEAV KR**KIQALQQQ ADEAEDRAQG LQRELDGERE R**REK**AEGDVA**
51 **ALNRRIQLVE EELDRAQER**L ATALQK**LEEA EKAADESER**G MKVIENRAMK
101 DEEKMEIQEM QLKEAK**HIAE EADRKYEEVA RKLVILEGEL ERAEER**AEVS
151 ELK**CGDLEEE LKNVTNNLK**S LEAASEKYSE KEDKYEEEIK LLSDKLKEAE
201 TRAEFAERTV AKLEKTIDDL EEKLAQAK**EE NVGLHQTLDQ TLNELNCI**

Residue Number Increasing Mass Decreasing Mass

Start - End	Observed	Mr(expt)	Mr(calc)	Delta	Miss	Sequence
13 - 27	1742.8461	1741.8388	1741.8594	-0.0206	1	KIQALQQQADEAEDR (No match)
14 - 27	1614.7540	1613.7467	1613.7645	-0.0178	0	IQALQQQADEAEDR (No match)
14 - 27	1614.7540	1613.7467	1613.7645	-0.0178	0	IQALQQQADEAEDR (Ions score 72)
14 - 33	2268.1104	2267.1031	2267.1254	-0.0222	1	IQALQQQADEAEDRAQGLQR (No match)
34 - 41	1003.4693	1002.4620	1002.4730	-0.0110	1	ELDGERER (No match)
45 - 55	1171.6378	1170.6305	1170.6105	0.0201	1	AEGDVAALNRR (No match)
55 - 65	1399.7418	1398.7345	1398.7466	-0.0121	1	RIQLVEEELDR (No match)
56 - 65	1243.6429	1242.6356	1242.6455	-0.0099	0	IQLVEEELDR (Ions score 49)
56 - 65	1243.6429	1242.6356	1242.6455	-0.0099	0	IQLVEEELDR (No match)
56 - 69	1727.8732	1726.8659	1726.8849	-0.0190	1	IQLVEEELDRAQER (No match)
56 - 69	1727.8732	1726.8659	1726.8849	-0.0190	1	IQLVEEELDRAQER (Ions score 55)
77 - 89	1476.6716	1475.6643	1475.6739	-0.0096	1	LEEAEKAADESER (No match)
83 - 89	777.3339	776.3266	776.3300	-0.0034	0	AADESER (No match)
117 - 124	940.4437	939.4364	939.4410	-0.0045	0	HIAEEADR (No match)
117 - 125	1068.5398	1067.5325	1067.5359	-0.0034	1	HIAEEADRK (No match)
126 - 132	894.4633	893.4560	893.4606	-0.0046	1	YEEVARK (No match)
132 - 142	1298.7556	1297.7483	1297.7605	-0.0121	1	KLVILEGELER (No match)
132 - 142	1298.7556	1297.7483	1297.7605	-0.0121	1	KLVILEGELER (Ions score 55)
133 - 142	1170.6648	1169.6575	1169.6655	-0.0080	0	LVILEGELER (No match)
133 - 146	1655.8776	1654.8703	1654.8889	-0.0186	1	LVILEGELERAEER (No match)
154 - 169	1875.8927	1874.8854	1874.9043	-0.0189	1	CGDLEEELKNVTNNLK (No match)
229 - 248	2340.1104	2339.1031	2339.1063	-0.0032	0	EENVGLHQTLDQTLNELNCI (No match)

Spot 673

Protein View

Match to: **gi|7959903** Score: **148** Expect: **3e-010**
PRO2446 [Homo sapiens]

Nominal mass (M_r): **27290**; Calculated pI value: **7.79**
NCBI BLAST search of gi|7959903 against nr
Unformatted sequence string for pasting into other applications

Taxonomy: Homo sapiens

Fixed modifications: Carbamidomethyl (C)
Variable modifications: Oxidation (M)
Cleavage by Trypsin: cuts C-term side of KR unless next residue is P
Sequence Coverage: **24%**

Matched peptides shown in **Bold Red**

```
  1 MVSISLKFYK ELQAHGADEL LKRVYGSFLV NPESGYNVSL LYDLENLPAS
 51 KDSIVHQAGM LKRNCFASVF EKYFQFQEEG KEGENRAVIH YRDDETMYVE
101 SKKDRVTVVF STVFKDDDDV VIGKVFMQEF KEGRRASHTA PQVLFSHREP
151 PLELKDTDAA VGDNIGYITF VLFPRHTNAS ARDNTINLIH TFRDYLHYHI
201 KCSKAYIHTR MRAKTSDFLK VLNRARPDAE KKEKI
```

Residue Number Increasing Mass Decreasing Mass

Start - End	Observed	Mr(expt)	Mr(calc)	Delta	Miss	Sequence
11 - 23	1479.7197	1478.7124	1478.7841	-0.0716	1	ELQAHGADELLKR (No match)
73 - 86	1760.7218	1759.7145	1759.7801	-0.0656	1	YFQFQEEGKEGENR (No match)
73 - 86	1760.7218	1759.7145	1759.7801	-0.0656	1	YFQFQEEGKEGENR (Ions score 69)
82 - 92	1343.6676	1342.6603	1342.6741	-0.0138	1	EGENRAVIHYR (No match)
136 - 148	1450.7101	1449.7028	1449.7477	-0.0448	0	ASHTAPQVLFSHR (No match)
136 - 148	1450.7101	1449.7028	1449.7477	-0.0448	0	ASHTAPQVLFSHR (No match)
183 - 193	1343.6676	1342.6603	1342.6993	-0.0390	0	DNTINLIHTFR (Ions score 11)

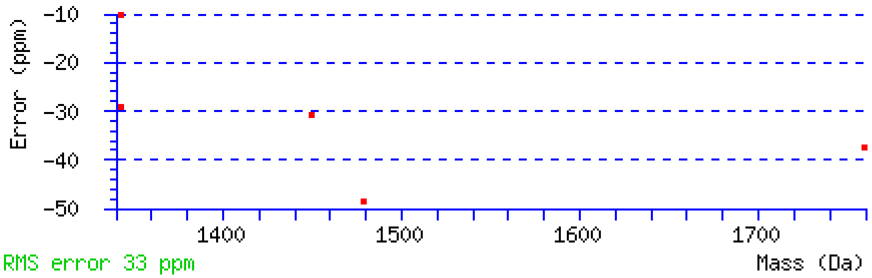

Error (ppm)
-10
-20
-30
-40
-50
1400
1500
1600
1700
RMS error 33 ppm
Mass (Da)

Spot 674

Protein View

Match to: **gi|5031599** Score: **775** Expect: **6.1e-073**
actin related protein 2/3 complex subunit 2 [Homo sapiens]

Nominal mass (M_r): **34426;** Calculated pI value: **6.84**
NCBI BLAST search of gi|5031599 against nr
Unformatted sequence string for pasting into other applications

Taxonomy: Homo sapiens
Links to retrieve other entries containing this sequence from NCBI Entrez:
gi|23238211 from Homo sapiens
gi|114583271 from Pan troglodytes
gi|114583273 from Pan troglodytes
gi|114583275 from Pan troglodytes
gi|3121764 from Homo sapiens
gi|62899830 from Pongo pygmaeus
gi|2282036 from Homo sapiens
gi|12653625 from Homo sapiens
gi|30582635 from Homo sapiens
gi|55732234 from Pongo pygmaeus
gi|60655211 from synthetic construct
gi|60655213 from synthetic construct
gi|62702288 from Homo sapiens
gi|119590997 from Homo sapiens
gi|119590999 from Homo sapiens
gi|158261559 (no taxonomy information for this entry)

Fixed modifications: Carbamidomethyl (C)
Variable modifications: Oxidation (M)
Cleavage by Trypsin: cuts C-term side of KR unless next residue is P
Sequence Coverage: **70%**

Matched peptides shown in **Bold Red**

```
  1 MILLEVNNRI IEETLALKFE NAAAGNKPEA VEVTFADFDG VLYHISNPNG
 51 DKTKVMVSIS LKFYKELQAH GADELLKRVY GSFLVNPESG YNVSLLYDLE
101 NLPASKDSIV HQAGMLKRNC FASVFEKYFQ FQEEGKEGEN RAVIHYRDDE
151 TMYVESKKDR VTVVFSTVFK DDDDVVIGKV FMQEFKEGRR ASHTAPQVLF
201 SHREPPLELK DTDAAVGDNI GYITFVLFPR HTNASARDNT INLIHTFRDY
251 LHYHIKCSKA YIHTRMRAKT SDFLKVLNRA RPDAEKKEMK TITGKTFSSR
301
```

Residue Number Increasing Mass Decreasing Mass

Start - End	Observed	Mr(expt)	Mr(calc)	Delta	Miss	Sequence
1 - 9	1117.5625	1116.5552	1116.5961	-0.0408	0	MILLEVNNR Oxidation (M) (No match)
10 - 18	1029.5759	1028.5686	1028.6117	-0.0431	0	IIEETLALK (No match)
19 - 52	3636.5735	3635.5662	3635.7167	-0.1505	0	FENAAAGNKPEAVEVTFADFDGVLYHISNPNGDK (No match)
66 - 77	1323.6484	1322.6411	1322.6830	-0.0418	0	ELQAHGADELLK (No match)
66 - 78	1479.7467	1478.7394	1478.7841	-0.0446	1	ELQAHGADELLKR (No match)
107 - 117	1214.5773	1213.5700	1213.6125	-0.0424	0	DSIVHQAGMLK Oxidation (M) (No match)
107 - 118	1370.6763	1369.6690	1369.7136	-0.0445	1	DSIVHQAGMLKR Oxidation (M) (No match)
118 - 127	1257.5714	1256.5641	1256.5971	-0.0330	1	RNCFASVFEK (No match)
119 - 127	1101.4645	1100.4572	1100.4960	-0.0388	0	NCFASVFEK (No match)
128 - 136	1175.4966	1174.4893	1174.5294	-0.0401	0	YFQFQEEGK (No match)
128 - 141	1760.7356	1759.7283	1759.7801	-0.0518	1	YFQFQEEGKEGENR (No match)
128 - 141	1760.7356	1759.7283	1759.7801	-0.0518	1	YFQFQEEGKEGENR (Ions score 104)
137 - 147	1343.6667	1342.6594	1342.6741	-0.0147	1	EGENRAVIHYR (No match)
142 - 157	1971.8549	1970.8476	1970.9043	-0.0567	1	AVIHYRDDETMYVESK Oxidation (M) (No match)
161 - 179	2083.0354	2082.0281	2082.0884	-0.0603	1	VTVVFSTVFKDDDDVVIGK (No match)
180 - 186	944.4169	943.4096	943.4473	-0.0377	0	VFMQEFK Oxidation (M) (No match)
190 - 203	1606.8108	1605.8035	1605.8488	-0.0452	1	RASHTAPQVLFSHR (No match)
191 - 203	1450.7128	1449.7055	1449.7477	-0.0421	0	ASHTAPQVLFSHR (No match)
191 - 203	1450.7128	1449.7055	1449.7477	-0.0421	0	ASHTAPQVLFSHR (Ions score 104)
191 - 210	2257.1396	2256.1323	2256.2014	-0.0691	1	ASHTAPQVLFSHREPPLELK (No match)
191 - 210	2257.1396	2256.1323	2256.2014	-0.0691	1	ASHTAPQVLFSHREPPLELK (Ions score 54)
204 - 210	825.4332	824.4259	824.4643	-0.0384	0	EPPLELK (No match)
211 - 230	2184.0303	2183.0230	2183.0898	-0.0668	0	DTDAAVGDNIGYITFVLFPR (No match)
211 - 230	2184.0303	2183.0230	2183.0898	-0.0668	0	DTDAAVGDNIGYITFVLFPR (Ions score 115)
238 - 248	1343.6667	1342.6594	1342.6993	-0.0399	0	DNTINLIHTFR (Ions score 88)
238 - 256	2413.1785	2412.1712	2412.2338	-0.0626	1	DNTINLIHTFRDYLHYHIK (No match)
249 - 256	1088.5189	1087.5116	1087.5450	-0.0334	0	DYLHYHIK (No match)
280 - 287	914.4729	913.4656	913.4981	-0.0325	1	ARPDAEKK (No match)

Spot 676

Protein View

Match to: **gi|24119203** Score: **648** Expect: **3e-060**
tropomyosin 3 isoform 2 [Homo sapiens]

Nominal mass (M_r): **29243;** Calculated pI value: **4.75**
NCBI BLAST search of gi|24119203 against nr
Unformatted sequence string for pasting into other applications

Taxonomy: Homo sapiens
Links to retrieve other entries containing this sequence from NCBI Entrez:
gi|48675927 from Sus scrofa
gi|55593362 from Pan troglodytes
gi|73961089 from Canis lupus familiaris
gi|73961115 from Canis lupus familiaris
gi|73984900 from Canis lupus familiaris
gi|109016516 from Macaca mulatta
gi|12653955 from Homo sapiens
gi|15929959 from Homo sapiens
gi|23961732 from Homo sapiens
gi|45272586 from Sus scrofa
gi|47938162 from Homo sapiens
gi|55665779 from Homo sapiens
gi|79153472 from Bos taurus
gi|119573616 from Homo sapiens
gi|119573620 from Homo sapiens

Fixed modifications: Carbamidomethyl (C)
Variable modifications: Oxidation (M)
Cleavage by Trypsin: cuts C-term side of KR unless next residue is P
Sequence Coverage: **58%**

Matched peptides shown in **Bold Red**

```
  1 MAGITTIEAV KRKIQVLQQQ ADDAEERAER LQREVEGERR AREQAEAEVA
 51 SLNRRIQLVE EELDRAQERL ATALQKLEEA EKAADESERG MKVIENRALK
101 DEEKMELQEI QLKEAKHIAE EADRKYEEVA RKLVIIEGDL ERTEERAELA
151 ESRCREMDEQ IRLMDQNLKC LSAAEEKYSQ KEDKYEEEIK ILTDKLKEAE
201 TRAEFAERSV AKLEKTIDDL EDKLKCTKEE HLCTQRMLDQ TLLDLNEM
```

Residue Number Increasing Mass Decreasing Mass

Start - End	Observed	Mr(expt)	Mr(calc)	Delta	Miss	Sequence
13 - 27	1770.8181	1769.8108	1769.8907	-0.0799	1	KIQVLQQQADDAEER (Ions score 98)
13 - 27	1770.8181	1769.8108	1769.8907	-0.0799	1	KIQVLQQQADDAEER (No match)
14 - 27	1642.7303	1641.7230	1641.7958	-0.0728	0	IQVLQQQADDAEER (Ions score 97)
14 - 27	1642.7303	1641.7230	1641.7958	-0.0728	0	IQVLQQQADDAEER (No match)
14 - 30	1998.8882	1997.8809	1997.9766	-0.0957	1	IQVLQQQADDAEERAER (No match)
34 - 40	874.3975	873.3902	873.4304	-0.0401	1	EVEGERR (No match)
41 - 54	1543.7109	1542.7036	1542.7749	-0.0713	1	AREQAEAEVASLNR (No match)
43 - 54	1316.5883	1315.5810	1315.6367	-0.0557	0	EQAEAEVASLNR (No match)
43 - 55	1472.6779	1471.6706	1471.7378	-0.0672	1	EQAEAEVASLNRR (No match)
55 - 65	1399.6849	1398.6776	1398.7466	-0.0690	1	RIQLVEEELDR (No match)
56 - 65	1243.5984	1242.5911	1242.6455	-0.0544	0	IQLVEEELDR (Ions score 63)
56 - 65	1243.5984	1242.5911	1242.6455	-0.0544	0	IQLVEEELDR (No match)
56 - 69	1727.8136	1726.8063	1726.8849	-0.0786	1	IQLVEEELDRAQER (No match)
56 - 69	1727.8136	1726.8063	1726.8849	-0.0786	1	IQLVEEELDRAQER (Ions score 64)
77 - 89	1476.6218	1475.6145	1475.6739	-0.0594	1	LEEAEKAADESER (No match)
105 - 116	1475.6477	1474.6404	1474.7701	-0.1296	1	MELQEIQLKEAK Oxidation (M) (No match)
117 - 124	940.4072	939.3999	939.4410	-0.0410	0	HIAEEADR (No match)
126 - 132	894.4233	893.4160	893.4606	-0.0446	1	YEEVARK (No match)
132 - 142	1284.6984	1283.6911	1283.7448	-0.0537	1	KLVIIEGDLER (No match)
132 - 142	1284.6984	1283.6911	1283.7448	-0.0537	1	KLVIIEGDLER (Ions score 77)
133 - 142	1156.6042	1155.5969	1155.6499	-0.0529	0	LVIIEGDLER (No match)
133 - 146	1671.8147	1670.8074	1670.8838	-0.0764	1	LVIIEGDLERTEER (No match)
154 - 162	1252.4749	1251.4676	1251.5336	-0.0659	1	CREMDEQIR Oxidation (M) (No match)
163 - 169	877.3940	876.3867	876.4375	-0.0507	0	LMDQNLK Oxidation (M) (No match)
216 - 225	1189.5696	1188.5623	1188.6237	-0.0614	1	TIDDLEDKLK (No match)
226 - 236	1461.5966	1460.5893	1460.6500	-0.0607	1	CTKEEHLCTQR (No match)

Spot 677

Protein View

Match to: **gi|13027378** Score: **257** Expect: **3.8e-021**
glucosamine-6-phosphate deaminase 1 [Homo sapiens]

Nominal mass (M_r): **32819**; Calculated pI value: **6.42**
NCBI BLAST search of gi|13027378 against nr
Unformatted sequence string for pasting into other applications

Taxonomy: Homo sapiens
Links to retrieve other entries containing this sequence from NCBI Entrez:
gi|1171639 from Homo sapiens
gi|37926898 from Homo sapiens
gi|37926899 from Homo sapiens
gi|37926900 from Homo sapiens
gi|37926901 from Homo sapiens
gi|37926902 from Homo sapiens
gi|37926903 from Homo sapiens
gi|2605949 from Homo sapiens
gi|2632113 from Homo sapiens
gi|2674175 from Homo sapiens
gi|2935438 from Homo sapiens
gi|3687211 from Homo sapiens
gi|15277501 from Homo sapiens
gi|18088723 from Homo sapiens
gi|18490843 from Homo sapiens
gi|119582294 from Homo sapiens
gi|119582295 from Homo sapiens
gi|119582296 from Homo sapiens
gi|119582297 from Homo sapiens

Fixed modifications: Carbamidomethyl (C)
Variable modifications: Oxidation (M)
Cleavage by Trypsin: cuts C-term side of KR unless next residue is P
Sequence Coverage: **43%**

Matched peptides shown in **Bold Red**

1 MKLIIILEHYS QASEWAAKYI RNR**IIQFNPG PEKYFTLGLP TGSTPLGCYK**
51 KLIEYYKNGD LSFKYVK**TFN MDEYVGLPRD HPESYHSFMW NNFFK**HIDIH
101 PENTHILDGN AVDLQAECDA FEEKIK**AAGG IELFVGGIGP DGHIAFNEPG**
151 **SSLVSR**TRVK **TLAMDTILAN ARFFDGELTK** VPTMALTVGV GTVMDAREVM
201 ILITGAHKAF ALYK**AIEEGV NHMWTVSAFQ QHPR**TVFVCD EDATLELKVK

251 TVKYFKGLML VHNKLVDPLY SIKEKETEKS QSSKKPYSD

Residue Number Increasing Mass Decreasing Mass

Start - End	Observed	Mr(expt)	Mr(calc)	Delta	Miss	Sequence
24 - 33	1142.5952	1141.5879	1141.6131	-0.0252	0	IIQFNPGPEK (No match)
24 - 33	1142.5952	1141.5879	1141.6131	-0.0252	0	IIQFNPGPEK (Ions score 30)
34 - 50	1874.9138	1873.9065	1873.9284	-0.0218	0	YFTLGLPTGSTPLGCYK (No match)
68 - 79	1441.6587	1440.6514	1440.6707	-0.0193	0	TFNMDEYVGLPR (No match)
68 - 79	1457.6471	1456.6398	1456.6656	-0.0258	0	TFNMDEYVGLPR Oxidation (M) (No match)
68 - 79	1457.6471	1456.6398	1456.6656	-0.0258	0	TFNMDEYVGLPR Oxidation (M) (Ions score 25)
80 - 95	2101.8506	2100.8433	2100.8788	-0.0355	0	DHPESYHSFMWNNFFK Oxidation (M) (No match)
127 - 156	2924.4326	2923.4253	2923.4827	-0.0573	0	AAGGIELFVGGIGPDGHIAFNEPGSSLVSR (No match)
127 - 156	2924.4326	2923.4253	2923.4827	-0.0573	0	AAGGIELFVGGIGPDGHIAFNEPGSSLVSR (Ions score 69)
161 - 172	1289.6591	1288.6518	1288.6809	-0.0291	0	TLAMDTILANAR (No match)
161 - 172	1305.6570	1304.6497	1304.6758	-0.0261	0	TLAMDTILANAR Oxidation (M) (No match)
161 - 172	1305.6570	1304.6497	1304.6758	-0.0261	0	TLAMDTILANAR Oxidation (M) (Ions score 29)
173 - 180	956.4611	955.4538	955.4650	-0.0112	0	FFDGELTK (No match)
215 - 234	2353.0718	2352.0645	2352.1069	-0.0423	0	AIEEGVNHMWTVSAFQQHPR Oxidation (M) (No match)

Spot 678

Protein View

Match to: **gi|5031599** Score: **370** Expect: **1.9e-032**
actin related protein 2/3 complex subunit 2 [Homo sapiens]

Nominal mass (M_r): **34426;** Calculated pI value: **6.84**
NCBI BLAST search of gi|5031599 against nr
Unformatted sequence string for pasting into other applications

Taxonomy: Homo sapiens
Links to retrieve other entries containing this sequence from NCBI Entrez:
gi|23238211 from Homo sapiens
gi|114583271 from Pan troglodytes
gi|114583273 from Pan troglodytes
gi|114583275 from Pan troglodytes
gi|3121764 from Homo sapiens
gi|62899830 from Pongo pygmaeus
gi|2282036 from Homo sapiens
gi|12653625 from Homo sapiens
gi|30582635 from Homo sapiens
gi|55732234 from Pongo pygmaeus
gi|60655211 from synthetic construct
gi|60655213 from synthetic construct
gi|62702288 from Homo sapiens
gi|119590997 from Homo sapiens
gi|119590999 from Homo sapiens
gi|158261559 (no taxonomy information for this entry)

Fixed modifications: Carbamidomethyl (C)
Variable modifications: Oxidation (M)
Cleavage by Trypsin: cuts C-term side of KR unless next residue is P
Sequence Coverage: **40%**

Matched peptides shown in **Bold Red**

```
  1 MILLEVNNRI IEETLALKFE NAAAGNKPEA VEVTFADFDG VLYHISNPNG
 51 DKTKVMVSIS LKFYKELQAH GADELLKRVY GSFLVNPESG YNVSLLYDLE
101 NLPASKDSIV HQAGMLKRNC FASVFEKYFQ FQEEGKEGEN RAVIHYRDDE
151 TMYVESKKDR VTVVFSTVFK DDDDVVIGKV FMQEFKEGRR ASHTAPQVLF
201 SHREPPLELK DTDAAVGDNI GYITFVLFPR HTNASARDNT INLIHTFRDY
251 LHYHIKCSKA YIHTRMRAKT SDFLKVLNRA RPDAEKKEMK TITGKTFSSR
301
```

Residue Number Increasing Mass Decreasing Mass

Start - End	Observed	Mr(expt)	Mr(calc)	Delta	Miss	Sequence
1 - 9	1117.5510	1116.5437	1116.5961	-0.0523	0	MILLEVNNR Oxidation (M) (No match)
66 - 78	1479.7383	1478.7310	1478.7841	-0.0530	1	ELQAHGADELLKR (No match)
107 - 118	1370.6747	1369.6674	1369.7136	-0.0461	1	DSIVHQAGMLKR Oxidation (M) (No match)
128 - 141	1760.7239	1759.7166	1759.7801	-0.0635	1	YFQFQEEGKEGENR (No match)
128 - 141	1760.7239	1759.7166	1759.7801	-0.0635	1	YFQFQEEGKEGENR (Ions score 90)
137 - 147	1343.6587	1342.6514	1342.6741	-0.0227	1	EGENRAVIHYR (No match)
180 - 186	944.4083	943.4010	943.4473	-0.0463	0	VFMQEFK Oxidation (M) (No match)
190 - 203	1606.7885	1605.7812	1605.8488	-0.0675	1	RASHTAPQVLFSHR (No match)
191 - 203	1450.7063	1449.6990	1449.7477	-0.0486	0	ASHTAPQVLFSHR (No match)
191 - 203	1450.7063	1449.6990	1449.7477	-0.0486	0	ASHTAPQVLFSHR (Ions score 15)
191 - 210	2257.1169	2256.1096	2256.2014	-0.0918	1	ASHTAPQVLFSHREPPLELK (No match)
211 - 230	2184.0120	2183.0047	2183.0898	-0.0851	0	DTDAAVGDNIGYITFVLFPR (No match)
211 - 230	2184.0120	2183.0047	2183.0898	-0.0851	0	DTDAAVGDNIGYITFVLFPR (Ions score 67)
238 - 248	1343.6587	1342.6514	1342.6993	-0.0479	0	DNTINLIHTFR (Ions score 35)
238 - 256	2413.1509	2412.1436	2412.2338	-0.0902	1	DNTINLIHTFRDYLHYHIK (Ions score 12)
238 - 256	2413.1509	2412.1436	2412.2338	-0.0902	1	DNTINLIHTFRDYLHYHIK (No match)

Spot 682

Protein View

Match to: **gi|4503143** Score: **212** Expect: **1.2e-016**
cathepsin D preproprotein [Homo sapiens]

Nominal mass (M_r): **45037;** Calculated pI value: **6.10**
NCBI BLAST search of gi|4503143 against nr
Unformatted sequence string for pasting into other applications

Taxonomy: Homo sapiens
Links to retrieve other entries containing this sequence from NCBI Entrez:
gi|115717 from Homo sapiens
gi|29678 from Homo sapiens
gi|179948 from Homo sapiens
gi|181180 from Homo sapiens
gi|16740920 from Homo sapiens
gi|30582659 from Homo sapiens
gi|48146011 from Homo sapiens
gi|54697170 from Homo sapiens
gi|61356567 from synthetic construct
gi|61362282 from synthetic construct
gi|119622866 from Homo sapiens
gi|119622867 from Homo sapiens
gi|119622868 from Homo sapiens
gi|123994405 from synthetic construct

Fixed modifications: Carbamidomethyl (C)
Variable modifications: Oxidation (M)
Cleavage by Trypsin: cuts C-term side of KR unless next residue is P
Sequence Coverage: **14%**

Matched peptides shown in **Bold Red**

```
  1 MQPSSLLPLA LCLLAAPASA LVRIPLHKFT SIRRTMSEVG GSVEDLIAKG
 51 PVSKYSQAVP AVTEGPIPEV LKNYMDAQYY GEIGIGTPPQ CFTVVFDTGS
101 SNLWVPSIHC KLLDIACWIH HKYNSDKSST YVKNGTSFDI HYGSGSLSGY
151 LSQDTVSVPC QSASSASALG GVKVERQVFG EATKQPGITF IAAKFDGILG
201 MAYPRISVNN VLPVFDNLMQ QKLVDQNIFS FYLSRDPDAQ PGGELMLGGT
251 DSKYYKGSLS YLNVTRKAYW QVHLDQVEVA SGLTLCKEGC EAIVDTGTSL
301 MVGPVDEVRE LQKAIGAVPL IQGEYMIPCE KVSTLPAITL KLGGKGYKLS
351 PEDYTLKVSQ AGKTLCLSGF MGMDIPPPSG PLWILGDVFI GRYYTVFDRD
401 NNRVGFAEAA RL
```

Residue Number Increasing Mass Decreasing Mass

Start - End	Observed	Mr(expt)	Mr(calc)	Delta	Miss	Sequence
195 - 205	1255.5881	1254.5808	1254.6066	-0.0258	0	FDGILGMAYPR Oxidation (M) (No match)
195 - 205	1255.5881	1254.5808	1254.6066	-0.0258	0	FDGILGMAYPR Oxidation (M) (Ions score 28)
206 - 222	1974.9885	1973.9812	1974.0244	-0.0431	0	ISVNNVLPVFDNLMQQK Oxidation (M) (Ions score 40)
206 - 222	1974.9885	1973.9812	1974.0244	-0.0431	0	ISVNNVLPVFDNLMQQK Oxidation (M) (No match)
223 - 235	1601.8005	1600.7932	1600.8249	-0.0317	0	LVDQNIFSFYLSR (Ions score 74)
223 - 235	1601.8005	1600.7932	1600.8249	-0.0317	0	LVDQNIFSFYLSR (No match)
393 - 399	963.4422	962.4349	962.4497	-0.0148	0	YYTVFDR (No match)
393 - 403	1462.6368	1461.6295	1461.6636	-0.0341	1	YYTVFDRDNNR (No match)
404 - 411	820.4261	819.4188	819.4238	-0.0050	0	VGFAEAAR (No match)

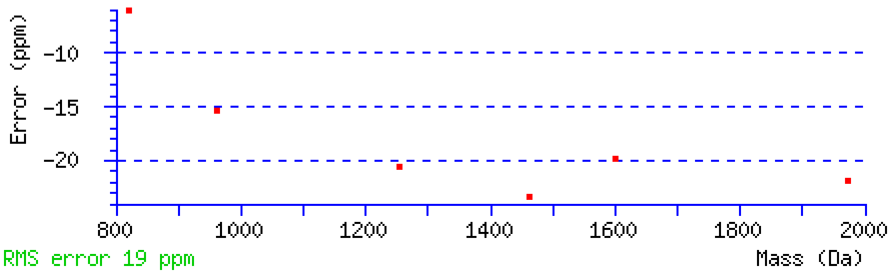

Mascot: http://www.matrixscience.com/

Spot 683

Protein View

Match to: **gi|7524354** Score: **80** Expect: **0.0021**
dimethylarginine dimethylaminohydrolase 2 [Homo sapiens]

Nominal mass (M_r): **29911**; Calculated pI value: **5.66**
NCBI BLAST search of gi|7524354 against nr
Unformatted sequence string for pasting into other applications

Taxonomy: Homo sapiens
Links to retrieve other entries containing this sequence from NCBI Entrez:
gi|6831529 from Homo sapiens
gi|33150630 from Homo sapiens
gi|4337098 from Homo sapiens
gi|4454710 from Homo sapiens
gi|5304876 from Homo sapiens
gi|12655161 from Homo sapiens
gi|15277275 from Homo sapiens
gi|55961453 from Homo sapiens
gi|55961613 from Homo sapiens
gi|119623905 from Homo sapiens
gi|119623906 from Homo sapiens
gi|123293870 from Homo sapiens
gi|126540431 from Homo sapiens

Fixed modifications: Carbamidomethyl (C)
Variable modifications: Oxidation (M)
Cleavage by Trypsin: cuts C-term side of KR unless next residue is P
Sequence Coverage: **22%**

Matched peptides shown in **Bold Red**

```
  1 MGTPGEGLGR CSHALIRGVP ESLASGEGAG AGLPALDLAK AQREHGVLGG
 51 KLRQRLGLQL LELPPEESLP LGPLLGDTAV IQGDTALITR PWSPARRPEV
101 DGVRKALQDL GLRIVEIGDE NATLDGTDVL FTGREFFVGL SKWTNHRGAE
151 IVADTFRDFA VSTVPVSGPS HLRGLCGMGG PRTVVAGSSD AAQKAVRAMA
201 VLTDHPYASL TLPDDAAADC LFLRPGLPGV PPFLLHRGGG DLPNSQEALQ
251 KLSDVTLVPV SCSELEKAGA GLSSLCLVLS TRPHS
```

Residue Number Increasing Mass Decreasing Mass

Start - End	Observed	Mr(expt)	Mr(calc)	Delta	Miss	Sequence
106 - 113	885.4993	884.4920	884.5079	-0.0159	0	ALQDLGLR (No match)
114 - 134	2235.0945	2234.0872	2234.1066	-0.0193	0	IVEIGDENATLDGTDVLFTGR (No match)
114 - 134	2235.0945	2234.0872	2234.1066	-0.0193	0	IVEIGDENATLDGTDVLFTGR (Ions score 26)
158 - 173	1668.8560	1667.8487	1667.8631	-0.0143	0	DFAVSTVPVSGPSHLR (No match)
158 - 173	1668.8560	1667.8487	1667.8631	-0.0143	0	DFAVSTVPVSGPSHLR (No match)
268 - 285	1825.9360	1824.9287	1824.9516	-0.0228	0	AGAGLSSLCLVLSTRPHS (No match)
268 - 285	1825.9360	1824.9287	1824.9516	-0.0228	0	AGAGLSSLCLVLSTRPHS (No match)

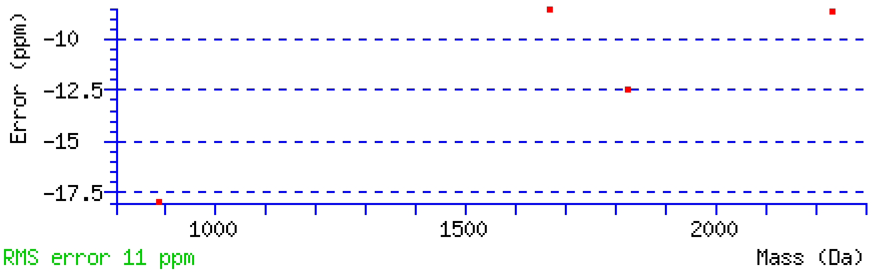

RMS error 11 ppm

Spot 684

Protein View

Match to: **gi|4588526** Score: **575** Expect: **6.1e-053**
nuclear chloride channel [Homo sapiens]

Nominal mass (M_r): **27249**; Calculated pI value: **5.02**
NCBI BLAST search of gi|4588526 against nr
Unformatted sequence string for pasting into other applications

Taxonomy: Homo sapiens
Links to retrieve other entries containing this sequence from NCBI Entrez:
gi|2073569 from Homo sapiens

Fixed modifications: Carbamidomethyl (C)
Variable modifications: Oxidation (M)
Cleavage by Trypsin: cuts C-term side of KR unless next residue is P
Sequence Coverage: **64%**

Matched peptides shown in **Bold Red**

```
  1 MAEEQPQVEL FVKAGSDGAK IGNCPFSQRL FMVLWLKGVT FNVTTVDTKR
 51 RTETVQKLCP GGELPFLLYG TEVHTDTNKI EEFLEAVLCP PRYPKLAALN
101 PESNTAGLDI FAKFSAYIKN SNPALNDNLE KGLLKALKVL DNYLTSPLPE
151 EVDETSAEDE GVSQRKFLDG NELTLADCNL LPKLHIVQVV CKKYRGFTIP
201 EAFRGVHRYL SNAYAREEFA STCPDDEEIE LAYEQVAKAL K
```

Residue Number Increasing Mass Decreasing Mass

Start - End	Observed	Mr(expt)	Mr(calc)	Delta	Miss	Sequence
21 - 29	1078.5074	1077.5001	1077.5025	-0.0024	0	IGNCPFSQR (Ions score 34)
21 - 29	1078.5074	1077.5001	1077.5025	-0.0024	0	IGNCPFSQR (No match)
30 - 37	1049.5990	1048.5917	1048.6143	-0.0226	0	LFMVLWLK (No match)
30 - 37	1065.5978	1064.5905	1064.6092	-0.0187	0	LFMVLWLK Oxidation (M) (No match)
38 - 49	1281.6602	1280.6529	1280.6612	-0.0083	0	GVTFNVTTVDTK (No match)
38 - 50	1437.7653	1436.7580	1436.7623	-0.0043	1	GVTFNVTTVDTKR (No match)
96 - 113	1844.9678	1843.9605	1843.9679	-0.0074	0	LAALNPESNTAGLDIFAK (No match)
120 - 131	1328.6354	1327.6281	1327.6367	-0.0086	0	NSNPALNDNLEK (No match)
139 - 165	2992.3721	2991.3648	2991.3832	-0.0184	0	VLDNYLTSPLPEEVDETSAEDEGVSQR (No match)
139 - 165	2992.3721	2991.3648	2991.3832	-0.0184	0	VLDNYLTSPLPEEVDETSAEDEGVSQR (Ions score 231)

166 - 183	2061.0635	2060.0562	2060.0612	-0.0049	1	**KFLDGNELTLADCNLLPK** (No match)
167 - 183	1932.9655	1931.9582	1931.9662	-0.0080	0	**FLDGNELTLADCNLLPK** (No match)
184 - 192	1095.6177	1094.6104	1094.6270	-0.0166	0	**LHIVQVVCK** (No match)
184 - 193	1223.7196	1222.7123	1222.7219	-0.0096	1	**LHIVQVVCKK** (No match)
194 - 204	1356.6981	1355.6908	1355.6985	-0.0077	1	**YRGFTIPEAFR** (Ions score 42)
194 - 204	1356.6981	1355.6908	1355.6985	-0.0077	1	**YRGFTIPEAFR** (No match)
196 - 204	1037.5400	1036.5327	1036.5341	-0.0014	0	**GFTIPEAFR** (Ions score 58)
196 - 204	1037.5400	1036.5327	1036.5341	-0.0014	0	**GFTIPEAFR** (No match)
209 - 216	957.4767	956.4694	956.4715	-0.0021	0	**YLSNAYAR** (No match)
217 - 238	2573.1375	2572.1302	2572.1162	0.0140	0	**EEFASTCPDDEEIELAYEQVAK** (No match)

Spot 685

Protein View

Match to: **gi|67464424** Score: **295** Expect: **6.1e-025**
Chain A, 14-3-3 Protein Epsilon (Human) Complexed To Peptide

Nominal mass (M_r): **26912**; Calculated pI value: **4.92**
NCBI BLAST search of gi|67464424 against nr
Unformatted sequence string for pasting into other applications

Taxonomy: Homo sapiens

Fixed modifications: Carbamidomethyl (C)
Variable modifications: Oxidation (M)
Cleavage by Trypsin: cuts C-term side of KR unless next residue is P
Sequence Coverage: **33%**

Matched peptides shown in **Bold Red**

```
  1 SMDDREDLVY QAKLAEQAER YDEMVESMKK VAGMDVELTV EERNLLSVAY
 51 KNVIGARRAS WRIISSIEQK EENKGGEDKL KMIREYRQMV ETELKLICCD
101 ILDVLDKHLI PAANTGESKV FYYKMKGDYH RYLAEFATGN DRKEAAENSL
151 VAYKAASDIA MTELPPTHPI RLGLALNFSV FYYEILNSPD RACRLAKAAF
201 DDAIAELDTL SEESYKDSTL IMQLLRDNLT LWTS
```

Residue Number Increasing Mass Decreasing Mass

Start - End	Observed	Mr(expt)	Mr(calc)	Delta	Miss	Sequence
14 - 20	816.3802	815.3729	815.4137	-0.0408	0	LAEQAER (No match)
31 - 43	1463.6418	1462.6345	1462.6973	-0.0628	0	VAGMDVELTVEER Oxidation (M) (No match)
31 - 43	1463.6418	1462.6345	1462.6973	-0.0628	0	VAGMDVELTVEER Oxidation (M) (Ions score 37)
132 - 142	1256.5397	1255.5324	1255.5833	-0.0508	0	YLAEFATGNDR (No match)
132 - 142	1256.5397	1255.5324	1255.5833	-0.0508	0	YLAEFATGNDR (Ions score 71)
132 - 143	1384.6283	1383.6210	1383.6782	-0.0572	1	YLAEFATGNDRK (Ions score 75)
132 - 143	1384.6283	1383.6210	1383.6782	-0.0572	1	YLAEFATGNDRK (No match)
155 - 171	1835.8503	1834.8430	1834.9247	-0.0817	0	AASDIAMTELPPTHPIR Oxidation (M) (Ions score 42)
155 - 171	1835.8503	1834.8430	1834.9247	-0.0817	0	AASDIAMTELPPTHPIR Oxidation (M) (No match)
172 - 191	2331.0923	2330.0850	2330.1946	-0.1096	0	LGLALNFSVFYYEILNSPDR (No match)
217 - 226	1205.5961	1204.5888	1204.6485	-0.0597	0	DSTLIMQLLR Oxidation (M) (No match)

Spot 686

Protein View

Match to: **gi|42822880** Score: **344** Expect: **7.7e-030**
similar to metallo-beta-lactamase superfamily protein [Homo sapiens]

Nominal mass (M_r): **31493**; Calculated pI value: **6.41**
NCBI BLAST search of gi|42822880 against nr
Unformatted sequence string for pasting into other applications

Taxonomy: Homo sapiens
Links to retrieve other entries containing this sequence from NCBI Entrez:
gi|23398544 from Homo sapiens
gi|119616385 from Homo sapiens
gi|158260579 (no taxonomy information for this entry)

Fixed modifications: Carbamidomethyl (C)
Variable modifications: Oxidation (M)
Cleavage by Trypsin: cuts C-term side of KR unless next residue is P
Sequence Coverage: **32%**

Matched peptides shown in **Bold Red**

```
  1 MSALEWYAHK SLGDGIFWIQ ERFYESGNRA NIWLVRGSEQ DVVIDTGLGL
 51 RSLPEYLYSS GLLQDREAKE DAARRPLLAV ATHVHFDHSG GLYQFDRVAV
101 HHAEAEALAR GDNFETVTWL SDSEVVRAPS PGWRARQFRV QAVQPTLILQ
151 DGDVINLGDR QLTVMHMPGH SRGSICLHDK DRKILFSGDV VYDGSLIDWL
201 PYSRISDYVG TCERLIELVD RGLVEKVLPG HFNTFGAERL FRLASNYISK
251 AGICHKVSTF AMRSLASLAL RVTNSRTSP
```

Residue Number Increasing Mass Decreasing Mass

Start - End	Observed	Mr(expt)	Mr(calc)	Delta	Miss	Sequence
37 - 51	1558.7921	1557.7848	1557.7998	-0.0150	0	GSEQDVVIDTGLGLR (Ions score 45)
37 - 51	1558.7921	1557.7848	1557.7998	-0.0150	0	GSEQDVVIDTGLGLR (No match)
52 - 66	1740.8590	1739.8517	1739.8730	-0.0212	0	SLPEYLYSSGLLQDR (No match)
52 - 66	1740.8590	1739.8517	1739.8730	-0.0212	0	SLPEYLYSSGLLQDR (Ions score 79)
98 - 110	1373.7163	1372.7090	1372.7211	-0.0120	0	VAVHHAEAEALAR (No match)
140 - 160	2264.1902	2263.1829	2263.2171	-0.0342	0	VQAVQPTLILQDGDVINLGDR (No match)
140 - 160	2264.1902	2263.1829	2263.2171	-0.0342	0	VQAVQPTLILQDGDVINLGDR (Ions score 96)

227 - 239	1444.7220	1443.7147	1443.7258	-0.0111	0	VLPGHFNTFGAER	(Ions score 31)
227 - 239	1444.7220	1443.7147	1443.7258	-0.0111	0	VLPGHFNTFGAER	(No match)
251 - 263	1493.7039	1492.6966	1492.7278	-0.0312	1	AGICHKVSTFAMR	Oxidation (M) (No match)

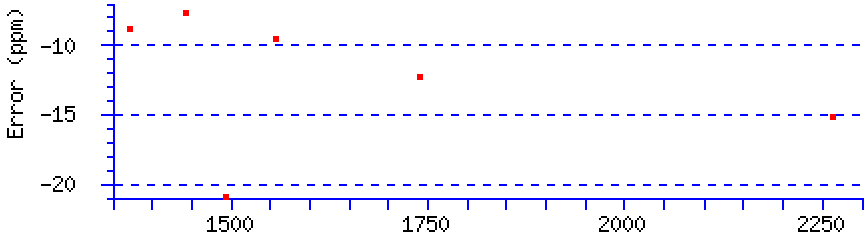

RMS error 12 ppm

Spot 687

Protein View

Match to: **gi|13543551** Score: **283** Expect: **9.6e-024**
Proteasome (prosome, macropain) subunit, alpha type, 1 [Homo sapiens]

Nominal mass (M_r): **29864**; Calculated pI value: **6.15**
NCBI BLAST search of gi|13543551 against nr
Unformatted sequence string for pasting into other applications

Taxonomy: Homo sapiens
Links to retrieve other entries containing this sequence from NCBI Entrez:
gi|30582133 from Homo sapiens
gi|61361727 from synthetic construct
gi|61361732 from synthetic construct

Fixed modifications: Carbamidomethyl (C)
Variable modifications: Oxidation (M)
Cleavage by Trypsin: cuts C-term side of KR unless next residue is P
Sequence Coverage: **39%**

Matched peptides shown in **Bold Red**

```
  1 MFRNQYDNDV TVWSPQGRIH QIEYAMEAVK QGSATVVLKS KTHAVLVALK
 51 RAQSELAAHQ KKILHVDNHI GISIAGLTAD ARLLCNFMRQ ECLDSRFVFD
101 RPLPVSRLVS LIGSKTQIPT QRYGRRPYGV GLLIAGYDDM GPHIFQTCPS
151 ANYFDCRAMS IGARSQSART YLERHMSEFM ECNLNELVKH GLRALRETLP
201 AEQDLTTKNV SIGIVGKDLE FTIYDDDDVS PFLEGLEERP QRKAQPAQPA
251 DEPAEKADEP MEH
```

Residue Number Increasing Mass Decreasing Mass

Start - End	Observed	Mr(expt)	Mr(calc)	Delta	Miss	Sequence
19 - 30	1447.6682	1446.6609	1446.7176	-0.0567	0	IHQIEYAMEAVK Oxidation (M) (Ions score 53)
19 - 30	1447.6682	1446.6609	1446.7176	-0.0567	0	IHQIEYAMEAVK Oxidation (M) (No match)
42 - 51	1107.6589	1106.6516	1106.6924	-0.0407	1	THAVLVALKR (No match)
62 - 82	2214.1462	2213.1389	2213.2280	-0.0890	1	KILHVDNHIGISIAGLTADAR (No match)
63 - 82	2086.0569	2085.0496	2085.1330	-0.0834	0	ILHVDNHIGISIAGLTADAR (Ions score 103)
63 - 82	2086.0569	2085.0496	2085.1330	-0.0834	0	ILHVDNHIGISIAGLTADAR (No match)
83 - 89	969.4183	968.4110	968.4572	-0.0461	0	LLCNFMR Oxidation (M) (No match)

97 - 107	1332.6902	1331.6829	1331.7349	-0.0520	0	FVFDRPLPVSR	(Ions score 24)
97 - 107	1332.6902	1331.6829	1331.7349	-0.0520	0	FVFDRPLPVSR	(No match)
116 - 122	843.4577	842.4504	842.4610	-0.0106	0	TQIPTQR	(No match)
194 - 208	1685.8372	1684.8299	1684.8995	-0.0696	1	ALRETLPAEQDLTTK	(No match)
244 - 263	2176.8474	2175.8401	2175.9378	-0.0977	1	AQPAQPADEPAEKADEPMEH	Oxidation (M) (No match)

Spot 688

Protein View

Match to: **gi|25188179** Score: **152** Expect: **1.2e-010**
voltage-dependent anion channel 3 [Homo sapiens]

Nominal mass (M_r): **30981**; Calculated pI value: **8.85**
NCBI BLAST search of gi|25188179 against nr
Unformatted sequence string for pasting into other applications

Taxonomy: Homo sapiens
Links to retrieve other entries containing this sequence from NCBI Entrez:
gi|12643945 from Homo sapiens
gi|2735307 from Homo sapiens
gi|3329394 from Homo sapiens
gi|34783950 from Homo sapiens
gi|117645468 from synthetic construct
gi|117645958 from synthetic construct
gi|119583622 from Homo sapiens

Fixed modifications: Carbamidomethyl (C)
Variable modifications: Oxidation (M)
Cleavage by Trypsin: cuts C-term side of KR unless next residue is P
Sequence Coverage: **26%**

Matched peptides shown in **Bold Red**

```
  1 MCNTPTYCDL GKAAKDVFNK GYGFGMVKID LKTKSCSGVE FSTSGHAYTD
 51 TGKASGNLET KYKVCNYGLT FTQKWNTDNT LGTEISWENK LAEGLKLTLD
101 TIFVPNTGKK SGKLKASYKR DCFSVGSNVD IDFSGPTIYG WAVLAFEGWL
151 AGYQMSFDTA KSKLSQNNFA LGYKAADFQL HTHVNDGTEF GGSIYQKVNE
201 KIETSINLAW TAGSNNTRFG IAAKYMLDCR TSLSAKVNNA SLIGLGYTQT
251 LRPGVKLTLS ALIDGKNFSA GGHKVGLGFE LEA
```

Residue Number Increasing Mass Decreasing Mass

Start - End	Observed	Mr(expt)	Mr(calc)	Delta	Miss	Sequence
21 - 28	874.3589	873.3516	873.4054	-0.0538	0	GYGFGMVK Oxidation (M) (No match)
97 - 110	1546.8086	1545.8013	1545.8766	-0.0753	1	LTLDTIFVPNTGKK (No match)
164 - 174	1254.5834	1253.5761	1253.6404	-0.0642	0	LSQNNFALGYK (No match)

164 - 174	1254.5834	1253.5761	1253.6404	-0.0642	0	LSQNNFALGYK	(Ions score 25)
175 - 197	2535.0784	2534.0711	2534.1825	-0.1114	0	AADFQLHTHVNDGTEFGGSIYQK	(No match)
175 - 197	2535.0784	2534.0711	2534.1825	-0.1114	0	AADFQLHTHVNDGTEFGGSIYQK	(Ions score 32)
237 - 256	2101.0879	2100.0806	2100.1690	-0.0884	0	VNNASLIGLGYTQTLRPGVK	(Ions score 39)
237 - 256	2101.0879	2100.0806	2100.1690	-0.0884	0	VNNASLIGLGYTQTLRPGVK	(No match)

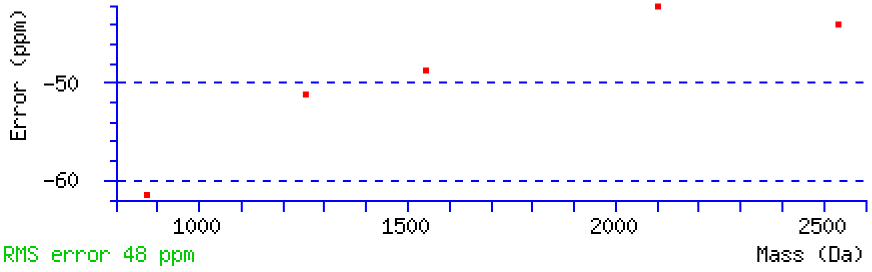

Spot 689

Protein View

Match to: **gi|1008915** Score: **571** Expect: **1.5e-052**
proteasome activator hPA28 suunit beta [Homo sapiens]

Nominal mass (M_r): **27502;** Calculated pI value: **5.44**
NCBI BLAST search of gi|1008915 against nr
Unformatted sequence string for pasting into other applications

Taxonomy: Homo sapiens
Links to retrieve other entries containing this sequence from NCBI Entrez:
gi|59896128 from Homo sapiens

Fixed modifications: Carbamidomethyl (C)
Variable modifications: Oxidation (M)
Cleavage by Trypsin: cuts C-term side of KR unless next residue is P
Sequence Coverage: **55%**

Matched peptides shown in **Bold Red**

```
  1 MAKPCGVRLS GEARKQVEVF RQNLFQEAEE FLYRFLPQKI IYLNQLLQED
 51 SLNVADLTSL RAPLDIPIPD PPPKDDEMET DKQEKKEVPK CGFLPGNEKV
101 LSLLALVKPE VWTLKEKCIL VITWIQHLIP KIEDGNDFGV AIQEKVLERV
151 NAVKTKVEAF QTTISKYFSE RGDAVAKASK ETHVMDYRAL VHERDEAAYG
201 ELRAMVLDLR AFYAELYHII SSNLEKIVTP KGEEKPSMY
```

Residue Number Increasing Mass Decreasing Mass

Start - End	Observed	Mr(expt)	Mr(calc)	Delta	Miss	Sequence
15 - 21	905.4799	904.4726	904.5130	-0.0404	1	KQVEVFR (No match)
22 - 34	1686.7371	1685.7298	1685.8049	-0.0750	0	QNLFQEAEEFLYR (Ions score 87)
22 - 34	1686.7371	1685.7298	1685.8049	-0.0750	0	QNLFQEAEEFLYR (No match)
40 - 61	2531.2380	2530.2307	2530.3642	-0.1335	0	IIYLNQLLQEDSLNVADLTSLR (No match)
40 - 61	2531.2380	2530.2307	2530.3642	-0.1335	0	IIYLNQLLQEDSLNVADLTSLR (Ions score 158)
91 - 99	1021.4126	1020.4053	1020.4698	-0.0645	0	CGFLPGNEK (No match)
132 - 145	1534.6647	1533.6574	1533.7310	-0.0736	0	IEDGNDFGVAIQEK (No match)
132 - 145	1534.6647	1533.6574	1533.7310	-0.0736	0	IEDGNDFGVAIQEK (Ions score 96)
167 - 177	1242.5591	1241.5518	1241.6040	-0.0521	1	YFSERGDAVAK (No match)
178 - 188	1352.6740	1351.6667	1351.6190	0.0477	1	ASKETHVMDYR Oxidation (M) (No match)

181 - 188	1066.4095	1065.4022	1065.4549	-0.0527	0	**ETHVMDYR** Oxidation (M) (No match)
189 - 203	1728.7908	1727.7835	1727.8590	-0.0755	1	**ALVHERDEAAYGELR** (No match)
195 - 203	1023.4268	1022.4195	1022.4668	-0.0473	0	**DEAAYGELR** (Ions score 65)
195 - 203	1023.4268	1022.4195	1022.4668	-0.0473	0	**DEAAYGELR** (No match)
204 - 210	833.4070	832.3997	832.4476	-0.0479	0	**AMVLDLR** Oxidation (M) (No match)
211 - 226	1897.8820	1896.8747	1896.9621	-0.0874	0	**AFYAELYHIISSNLEK** (No match)
232 - 239	956.3485	955.3412	955.3956	-0.0544	0	**GEEKPSMY** Oxidation (M) (No match)

Spot 690

Protein View

Match to: **gi|14149777** Score: **113** Expect: **9.6e-007**
haloacid dehalogenase-like hydrolase domain containing 2 [Homo sapiens]

Nominal mass (M_r): **28746**; Calculated pI value: **5.84**
NCBI BLAST search of gi|14149777 against nr
Unformatted sequence string for pasting into other applications

Taxonomy: Homo sapiens
Links to retrieve other entries containing this sequence from NCBI Entrez:
gi|74733528 from Homo sapiens
gi|12052886 from Homo sapiens
gi|23271196 from Homo sapiens
gi|49065400 from Homo sapiens
gi|117645780 from synthetic construct
gi|119583309 from Homo sapiens
gi|158257628 (no taxonomy information for this entry)

Fixed modifications: Carbamidomethyl (C)
Variable modifications: Oxidation (M)
Cleavage by Trypsin: cuts C-term side of KR unless next residue is P
Sequence Coverage: **28%**

Matched peptides shown in **Bold Red**

```
  1 MAACRALKAV LVDLSGTLHI EDAAVPGAQE ALKRLRGASV IIRFVTNTTK
 51 ESKQDLLERL RKLEFDISED EIFTSLTAAR SLLERKQVRP MLLVDDRALP
101 DFKGIQTSDP NAVVMGLAPE HFHYQILNQA FRLLLDGAPL IAIHKARYYK
151 RKDGLALGPG PFVTALEYAT DTKATVVGKP EKTFFLEALR GTGCEPEEAV
201 MIGDDCRDDV GGAQDVGMLG ILVKTGKYRA SDEEKINPPP YLTCESFPHA
251 VDHILQHLL
```

Residue Number Increasing Mass Decreasing Mass

Start - End	Observed	Mr(expt)	Mr(calc)	Delta	Miss	Sequence
62 - 80	2184.9907	2183.9834	2184.0950	-0.1115	1	KLEFDISEDEIFTSLTAAR (Ions score 66)
62 - 80	2184.9907	2183.9834	2184.0950	-0.1115	1	KLEFDISEDEIFTSLTAAR (No match)
86 - 97	1485.7417	1484.7344	1484.8133	-0.0789	1	KQVRPMLLVDDR Oxidation (M) (No match)

133 - 145	1373.7717	1372.7644	1372.8442	-0.0797	0	LLLDGAPLIAIHK (No match)
153 - 173	2135.9670	2134.9597	2135.0786	-0.1188	0	DGLALGPGPFVTALEYATDTK (No match)
183 - 190	996.4993	995.4920	995.5440	-0.0519	0	TFFLEALR (No match)

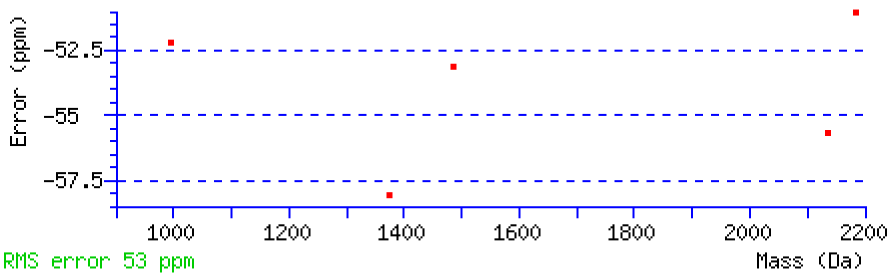

RMS error 53 ppm

Spot 691

Protein View

Match to: **gi|24158605** Score: **173** Expect: **9.6e-013**
Chain A, Cathepsin B Complexed With Dipeptidyl Nitrile Inhibitor

Nominal mass (M_r): **29571**; Calculated pI value: **5.34**
NCBI BLAST search of gi|24158605 against nr
Unformatted sequence string for pasting into other applications

Taxonomy: Homo sapiens
Links to retrieve other entries containing this sequence from NCBI Entrez:
gi|24158606 from Homo sapiens
gi|24158607 from Homo sapiens

Fixed modifications: Carbamidomethyl (C)
Variable modifications: Oxidation (M)
Cleavage by Trypsin: cuts C-term side of KR unless next residue is P
Sequence Coverage: **16%**

Matched peptides shown in **Bold Red**

```
  1 KLPASFDARE QWPQCPTIKE IRDQGSCGSC WAFGAVEAIS DRICIHTNAH
 51 VSVEVSAEDL LTCCGSMCGD GCNGGYPAEA WNFWTRKGLV SGGLYESHVG
101 CRPYSIPPCE HHVNGSRPPC TGEGDTPKCS KICEPGYSPT YKQDKHYGYN
151 SYSVSNSEKD IMAEIYKNGP VEGAFSVYSD FLLYKSGVYQ HVTGEMMGGH
201 AIRILGWGVE NGTPYWLVAN SWNTDWGDNG FFKILRGQDH CGIESEVVAG
251 IPRTDQYWEK I
```

Residue Number Increasing Mass Decreasing Mass

Start - End	Observed	Mr(expt)	Mr(calc)	Delta	Miss	Sequence
2 - 9	876.4570	875.4497	875.4501	-0.0003	0	LPASFDAR (No match)
186 - 203	1945.8837	1944.8764	1944.8934	-0.0169	0	SGVYQHVTGEMMGGHAIR Oxidation (M) (Ions score 19)
186 - 203	1945.8837	1944.8764	1944.8934	-0.0169	0	SGVYQHVTGEMMGGHAIR Oxidation (M) (No match)
186 - 203	1961.8770	1960.8697	1960.8883	-0.0186	0	SGVYQHVTGEMMGGHAIR 2 Oxidation (M) (No match)
186 - 203	1961.8770	1960.8697	1960.8883	-0.0186	0	SGVYQHVTGEMMGGHAIR 2 Oxidation (M) (No match)
237 - 253	1823.8557	1822.8484	1822.8631	-0.0147	0	GQDHCGIESEVVAGIPR (Ions score 120)
237 - 253	1823.8557	1822.8484	1822.8631	-0.0147	0	GQDHCGIESEVVAGIPR (No match)

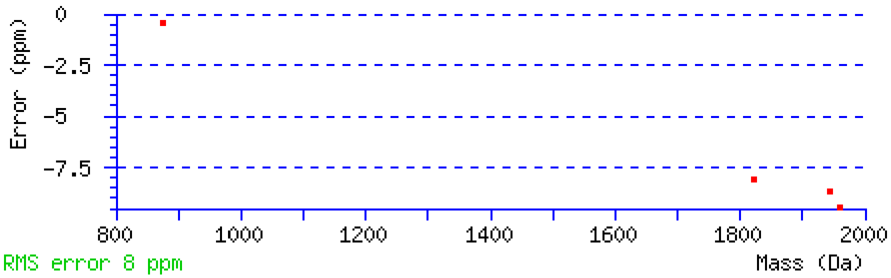
0
-2.5
-5
-7.5
Error (ppm)
800
1000
1200
1400
1600
1800
2000
Mass (Da)
RMS error 8 ppm

Spot 692

Protein View

Match to: **gi|119586505** Score: **85** Expect: **0.00065**
proteasome (prosome, macropain) activator subunit 2 (PA28 beta), isoform CRA_b [Homo sapiens]

Nominal mass (M_r): **22023**; Calculated pI value: **5.40**
NCBI BLAST search of gi|119586505 against nr
Unformatted sequence string for pasting into other applications

Taxonomy: Homo sapiens

Fixed modifications: Carbamidomethyl (C)
Variable modifications: Oxidation (M)
Cleavage by Trypsin: cuts C-term side of KR unless next residue is P
Sequence Coverage: **14%**

Matched peptides shown in **Bold Red**

```
  1 MAKPCGVRLS GEARKQVEVF RQNLFQEAEE FLYRFLPQKI IYLNQLLQED
 51 SLNVADLTSL RAPLDIPIPD PPPKDDEMET DKQEKKEVPK CGFLPGNEKV
101 LSLLALVKPE VWTLKEKCIL VITWIQHLIP KIEDGNDFGV AIQEKVLERV
151 NAVKTKVEAF QTTISKWITG PWCMSEMRQP MGSSGPWCWT
```

Residue Number Increasing Mass Decreasing Mass

Start - End	Observed	Mr(expt)	Mr(calc)	Delta	Miss	Sequence	
22 - 34	1686.7628	1685.7555	1685.8049	-0.0493	0	QNLFQEAEEFLYR	(Ions score 42)
22 - 34	1686.7628	1685.7555	1685.8049	-0.0493	0	QNLFQEAEEFLYR	(No match)
132 - 145	1534.6860	1533.6787	1533.7310	-0.0523	0	IEDGNDFGVAIQEK	(No match)
132 - 145	1534.6860	1533.6787	1533.7310	-0.0523	0	IEDGNDFGVAIQEK	(Ions score 23)

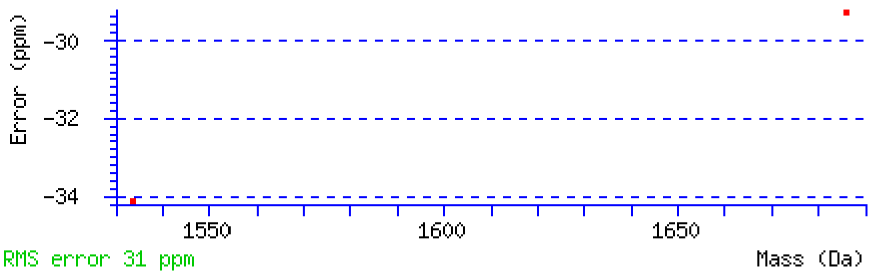

Mascot: http://www.matrixscience.com/

Spot 694

Protein View

Match to: **gi|4588526** Score: **290** Expect: **1.9e-024**
nuclear chloride channel [Homo sapiens]

Nominal mass (M_r): **27249**; Calculated pI value: **5.02**
NCBI BLAST search of gi|4588526 against nr
Unformatted sequence string for pasting into other applications

Taxonomy: Homo sapiens
Links to retrieve other entries containing this sequence from NCBI Entrez:
gi|2073569 from Homo sapiens

Fixed modifications: Carbamidomethyl (C)
Variable modifications: Oxidation (M)
Cleavage by Trypsin: cuts C-term side of KR unless next residue is P
Sequence Coverage: **30%**

Matched peptides shown in **Bold Red**

```
  1 MAEEQPQVEL FVKAGSDGAK IGNCPFSQRL FMVLWLKGVT FNVTTVDTKR
 51 RTETVQKLCP GGELPFLLYG TEVHTDTNKI EEFLEAVLCP PRYPKLAALN
101 PESNTAGLDI FAKFSAYIKN SNPALNDNLE KGLLKALKVL DNYLTSPLPE
151 EVDETSAEDE GVSQRKFLDG NELTLADCNL LPKLHIVQVV CKKYRGFTIP
201 EAFRGVHRYL SNAYAREEFA STCPDDEEIE LAYEQVAKAL K
```

Residue Number Increasing Mass Decreasing Mass

Start - End	Observed	Mr(expt)	Mr(calc)	Delta	Miss	Sequence
21 - 29	1078.4835	1077.4762	1077.5025	-0.0263	0	IGNCPFSQR (Ions score 24)
21 - 29	1078.4835	1077.4762	1077.5025	-0.0263	0	IGNCPFSQR (No match)
96 - 113	1844.9369	1843.9296	1843.9679	-0.0383	0	LAALNPESNTAGLDIFAK (No match)
139 - 165	2992.2957	2991.2884	2991.3832	-0.0948	0	VLDNYLTSPLPEEVDETSAEDEGVSQR (Ions score 148)
139 - 165	2992.2957	2991.2884	2991.3832	-0.0948	0	VLDNYLTSPLPEEVDETSAEDEGVSQR (No match)
194 - 204	1356.6705	1355.6632	1355.6985	-0.0353	1	YRGFTIPEAFR (Ions score 9)
194 - 204	1356.6705	1355.6632	1355.6985	-0.0353	1	YRGFTIPEAFR (No match)
196 - 204	1037.5178	1036.5105	1036.5341	-0.0236	0	GFTIPEAFR (Ions score 27)
196 - 204	1037.5178	1036.5105	1036.5341	-0.0236	0	GFTIPEAFR (No match)
209 - 216	957.4594	956.4521	956.4715	-0.0194	0	YLSNAYAR (No match)

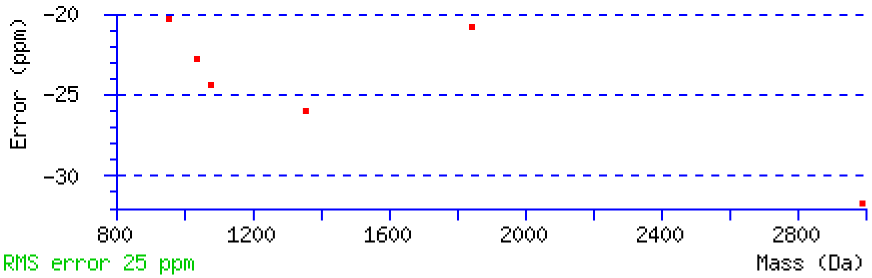

Error (ppm)
-20
-25
-30
800
1200
1600
2000
2400
2800
Mass (Da)
RMS error 25 ppm

Spot 696

Protein View

Match to: **gi|4503143** Score: **343** Expect: **9.6e-030**
cathepsin D preproprotein [Homo sapiens]

Nominal mass (M_r): **45037**; Calculated pI value: **6.10**
NCBI BLAST search of gi|4503143 against nr
Unformatted sequence string for pasting into other applications

Taxonomy: Homo sapiens
Links to retrieve other entries containing this sequence from NCBI Entrez:
gi|115717 from Homo sapiens
gi|29678 from Homo sapiens
gi|179948 from Homo sapiens
gi|181180 from Homo sapiens
gi|16740920 from Homo sapiens
gi|30582659 from Homo sapiens
gi|48146011 from Homo sapiens
gi|54697170 from Homo sapiens
gi|61356567 from synthetic construct
gi|61362282 from synthetic construct
gi|119622866 from Homo sapiens
gi|119622867 from Homo sapiens
gi|119622868 from Homo sapiens
gi|123994405 from synthetic construct

Fixed modifications: Carbamidomethyl (C)
Variable modifications: Oxidation (M)
Cleavage by Trypsin: cuts C-term side of KR unless next residue is P
Sequence Coverage: **26%**

Matched peptides shown in **Bold Red**

```
  1 MQPSSLLPLA LCLLAAPASA LVRIPLHKFT SIRRTMSEVG GSVEDLIAKG
 51 PVSKYSQAVP AVTEGPIPEV LKNYMDAQYY GEIGIGTPPQ CFTVVFDTGS
101 SNLWVPSIHC KLLDIACWIH HKYNSDKSST YVKNGTSFDI HYGSGSLSGY
151 LSQDTVSVPC QSASSASALG GVKVERQVFG EATKQPGITF IAAKFDGILG
201 MAYPRISVNN VLPVFDNLMQ QKLVDQNIFS FYLSRDPDAQ PGGELMLGGT
251 DSKYYKGSLS YLNVTRKAYW QVHLDQVEVA SGLTLCKEGC EAIVDTGTSL
301 MVGPVDEVRE LQKAIGAVPL IQGEYMIPCE KVSTLPAITL KLGGKGYKLS
351 PEDYTLKVSQ AGKTLCLSGF MGMDIPPPSG PLWILGDVFI GRYYTVFDRD
401 NNRVGFAEAA RL
```

Residue Number Increasing Mass Decreasing Mass

Start - End	Observed	Mr(expt)	Mr(calc)	Delta	Miss	Sequence
177 - 194	1905.9531	1904.9458	1905.0359	-0.0901	1	QVFGEATKQPGITFIAAK (No match)
195 - 205	1255.5598	1254.5525	1254.6066	-0.0541	0	FDGILGMAYPR Oxidation (M) (Ions score 36)
195 - 205	1255.5598	1254.5525	1254.6066	-0.0541	0	FDGILGMAYPR Oxidation (M) (No match)
206 - 222	1974.9436	1973.9363	1974.0244	-0.0880	0	ISVNNVLPVFDNLMQQK Oxidation (M) (No match)
206 - 222	1974.9436	1973.9363	1974.0244	-0.0880	0	ISVNNVLPVFDNLMQQK Oxidation (M) (Ions score 40)
223 - 235	1601.7706	1600.7633	1600.8249	-0.0616	0	LVDQNIFSFYLSR (No match)
223 - 235	1601.7706	1600.7633	1600.8249	-0.0616	0	LVDQNIFSFYLSR (Ions score 62)
288 - 309	2349.9829	2348.9756	2349.0827	-0.1071	0	EGCEAIVDTGTSLMVGPVDEVR Oxidation (M) (No match)
288 - 309	2349.9829	2348.9756	2349.0827	-0.1071	0	EGCEAIVDTGTSLMVGPVDEVR Oxidation (M) (Ions score 41)
349 - 357	1065.5004	1064.4931	1064.5390	-0.0458	0	LSPEDYTLK (No match)
393 - 399	963.4183	962.4110	962.4497	-0.0387	0	YYTVFDR (No match)
393 - 403	1462.6100	1461.6027	1461.6636	-0.0609	1	YYTVFDRDNNR (Ions score 52)
393 - 403	1462.6100	1461.6027	1461.6636	-0.0609	1	YYTVFDRDNNR (No match)
404 - 411	820.3944	819.3871	819.4238	-0.0367	0	VGFAEAAR (No match)

Mascot: http://www.matrixscience.com/

Spot 697

Protein View

Match to: **gi|847724** Score: **129** Expect: **2.4e-008**
methylthioadenosine phosphorylase

Nominal mass (M_r): **31743;** Calculated pI value: **6.75**
NCBI BLAST search of gi|847724 against nr
Unformatted sequence string for pasting into other applications

Taxonomy: Homo sapiens
Links to retrieve other entries containing this sequence from NCBI Entrez:
gi|19913487 from Homo sapiens
gi|51472043 from Homo sapiens

Fixed modifications: Carbamidomethyl (C)
Variable modifications: Oxidation (M)
Cleavage by Trypsin: cuts C-term side of KR unless next residue is P
Sequence Coverage: **28%**

Matched peptides shown in **Bold Red**

```
  1 MASGTTTTAV KIGIIGGTGL DDPEILEGRT EKYVDTPFGK PSDALILGKI
 51 KNVDCILLAR HGRQHTIMPS KVNYQANIWA LKEEGCTHVI VTTACGSLRE
101 EIQPGDIVII DQFIDRTTMR PQSFYDGSHS CARGVCHIPM AEPFCPKTRE
151 VLIETAKKLG LRCHSKGTMV TIEGPRFSSR AESFMFRTWG ADVINMTTVP
201 EVVLAKEAGI CYASIAMATD YDCWKEHEEA VSVDRVLKTL KENANKAKSL
251 LLTTIPQIGS TEWSETLHNL KNMAQFSVLL PRH
```

Residue Number Increasing Mass Decreasing Mass

Start - End	Observed	Mr(expt)	Mr(calc)	Delta	Miss	Sequence
12 - 29	1824.9396	1823.9323	1823.9628	-0.0305	0	IGIIGGTGLDDPEILEGR (Ions score 20)
12 - 29	1824.9396	1823.9323	1823.9628	-0.0305	0	IGIIGGTGLDDPEILEGR (No match)
33 - 49	1820.9509	1819.9436	1819.9719	-0.0283	0	YVDTPFGKPSDALILGK (No match)
61 - 71	1291.6630	1290.6557	1290.6615	-0.0058	1	HGRQHTIMPSK (No match)
100 - 116	1999.9980	1998.9907	1999.0262	-0.0354	0	EEIQPGDIVIIDQFIDR (No match)
100 - 116	1999.9980	1998.9907	1999.0262	-0.0354	0	EEIQPGDIVIIDQFIDR (Ions score 14)
181 - 187	903.3913	902.3840	902.3956	-0.0116	0	AESFMFR Oxidation (M) (No match)
272 - 282	1291.6630	1290.6557	1290.6754	-0.0197	0	NMAQFSVLLPR Oxidation (M) (Ions score 5)

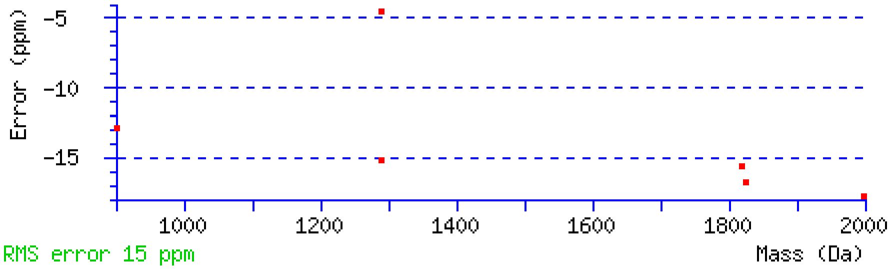

Mascot: http://www.matrixscience.com/

Spot 698

Protein View

Match to: **gi|4758484** Score: **137** Expect: **3.8e-009**
glutathione-S-transferase omega 1 [Homo sapiens]

Nominal mass (M_r): **27833;** Calculated pI value: **6.23**
NCBI BLAST search of gi|4758484 against nr
Unformatted sequence string for pasting into other applications

Taxonomy: Homo sapiens
Links to retrieve other entries containing this sequence from NCBI Entrez:
gi|6016173 from Homo sapiens
gi|10120947 from Homo sapiens
gi|8132762 from Homo sapiens
gi|2393722 from Homo sapiens
gi|12652753 from Homo sapiens
gi|55925946 from Homo sapiens
gi|55957302 from Homo sapiens
gi|119569989 from Homo sapiens

Fixed modifications: Carbamidomethyl (C)
Variable modifications: Oxidation (M)
Cleavage by Trypsin: cuts C-term side of KR unless next residue is P
Sequence Coverage: **21%**

Matched peptides shown in **Bold Red**

```
  1 MSGESARSLG KGSAPPGPVP EGSIRIYSMR FCPFAERTRL VLKAKGIRHE
 51 VININLKNKP EWFFKKNPFG LVPVLENSQG QLIYESAITC EYLDEAYPGK
101 KLLPDDPYEK ACQKMILELF SKVPSLVGSF IRSQNKEDYA GLKEEFRKEF
151 TKLEEVLTNK KTTFFGGNSI SMIDYLIWPW FERLEAMKLN ECVDHTPKLK
201 LWMAAMKEDP TVSALLTSEK DWQGFLELYL QNSPEACDYG L
```

Residue Number Increasing Mass Decreasing Mass

Start - End	Observed	Mr(expt)	Mr(calc)	Delta	Miss	Sequence
12 - 25	1320.6738	1319.6665	1319.6833	-0.0168	0	GSAPPGPVPEGSIR (Ions score 39)
12 - 25	1320.6738	1319.6665	1319.6833	-0.0168	0	GSAPPGPVPEGSIR (No match)
31 - 37	926.4101	925.4028	925.4116	-0.0088	0	FCPFAER (No match)

46 - 57	1405.8108	1404.8035	1404.8201	-0.0165	1	GIRHEVININLK	(Ions score 5)
46 - 57	1405.8108	1404.8035	1404.8201	-0.0165	1	GIRHEVININLK	(No match)
58 - 65	1095.5417	1094.5344	1094.5549	-0.0204	0	NKPEWFFK	(No match)
58 - 65	1095.5417	1094.5344	1094.5549	-0.0204	0	NKPEWFFK	(Ions score 5)
123 - 132	1074.6194	1073.6121	1073.6233	-0.0111	0	VPSLVGSFIR	(No match)
123 - 132	1074.6194	1073.6121	1073.6233	-0.0111	0	VPSLVGSFIR	(Ions score 21)

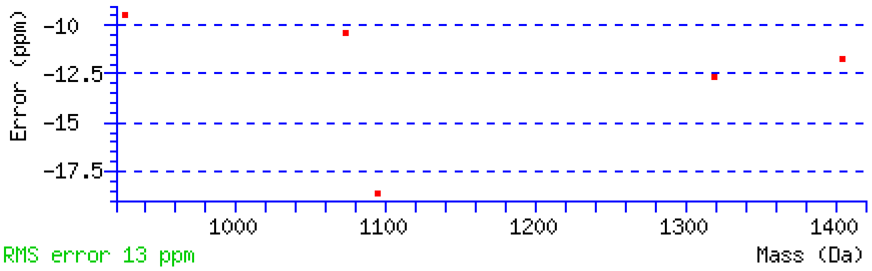

Mascot: http://www.matrixscience.com/

Spot 699

Protein View

Match to: **gi|6005842** Score: **127** Expect: **3.8e-008**
proline synthetase co-transcribed homolog [Homo sapiens]

Nominal mass (M_r): **30610**; Calculated pI value: **7.09**
NCBI BLAST search of gi|6005842 against nr
Unformatted sequence string for pasting into other applications

Taxonomy: Homo sapiens
Links to retrieve other entries containing this sequence from NCBI Entrez:
gi|12230426 from Homo sapiens
gi|4126978 from Homo sapiens
gi|12052758 from Homo sapiens
gi|15147391 from Homo sapiens
gi|49065490 from Homo sapiens

Fixed modifications: Carbamidomethyl (C)
Variable modifications: Oxidation (M)
Cleavage by Trypsin: cuts C-term side of KR unless next residue is P
Sequence Coverage: **12%**

Matched peptides shown in **Bold Red**

```
  1 MWRAGSMSAE LGVGCALRAV NERVQQAVAR RPRDLPAIQP RLVAVSKTKP
 51 ADMVIEAYGH GQRTFGENYV QELLEKASNP KILSLCPEIK WHFIGHLQKQ
101 NVNKLMAVPN LFMLETVDSV KLADKVNSSW QRKGSPERLK VMVQINTSGE
151 ESKHGLPPSE TIAIVEHINA KCPNLEFVGL MTIGSFGHDL SQGPNPDFQL
201 LLSLREELCK KLNIPADQVE LSMGMSADFQ HAVEVGSTNV RIGSTIFGER
251 DYSKKPTPDK CAADVKAPLE VAQEH
```

Residue Number Increasing Mass Decreasing Mass

Start - End	Observed	Mr(expt)	Mr(calc)	Delta	Miss	Sequence
48 - 63	1788.8486	1787.8413	1787.8624	-0.0211	0	TKPADMVIEAYGHGQR Oxidation (M) (No match)
48 - 63	1788.8486	1787.8413	1787.8624	-0.0211	0	TKPADMVIEAYGHGQR Oxidation (M) (Ions score 40)
154 - 171	1926.0247	1925.0174	1925.0370	-0.0196	0	HGLPPSETIAIVEHINAK (No match)
154 - 171	1926.0247	1925.0174	1925.0370	-0.0196	0	HGLPPSETIAIVEHINAK (Ions score 58)

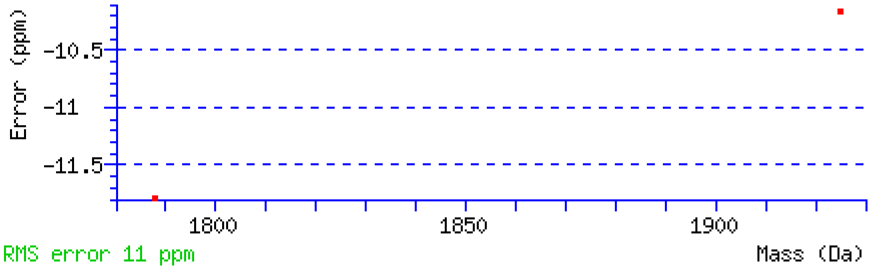

Mascot: http://www.matrixscience.com/

Spot 701

Protein View

Match to: **gi|4501887** Score: **304** Expect: **7.7e-026**
actin, gamma 1 propeptide [Homo sapiens]

Nominal mass (M_r): **42108**; Calculated pI value: **5.31**
NCBI BLAST search of gi|4501887 against nr
Unformatted sequence string for pasting into other applications

Taxonomy: Homo sapiens
Links to retrieve other entries containing this sequence from NCBI Entrez:
gi|6752954 from Mus musculus
gi|75812932 from Bos taurus
gi|147901470 from Xenopus laevis
gi|109119050 from Macaca mulatta
gi|109119052 from Macaca mulatta
gi|126308642 from Monodelphis domestica
gi|149723257 from Equus caballus
gi|54036665 from Rattus norvegicus
gi|54036675 from Trichosurus vulpecula
gi|54036676 from Bos taurus
gi|54036677 from Mus musculus
gi|54036678 from Homo sapiens
gi|82197919 from Gallus gallus
gi|134035381 from Xenopus laevis
gi|28339 from Homo sapiens
gi|57574 from Rattus rattus
gi|178043 from Homo sapiens
gi|309089 from Mus musculus
gi|3320894 from Trichosurus vulpecula
gi|12653055 from Homo sapiens
gi|12804935 from Homo sapiens
gi|13097126 from Mus musculus
gi|13938581 from Homo sapiens
gi|14602652 from Homo sapiens
gi|15012187 from Homo sapiens
gi|15082291 from Homo sapiens
gi|15929094 from Homo sapiens
gi|15990564 from Homo sapiens
gi|16041790 from Homo sapiens
gi|17511847 from Homo sapiens
gi|18256837 from Mus musculus
gi|23271069 from Mus musculus
gi|26345036 from Mus musculus

gi|26352890 from Mus musculus
gi|31565757 from Homo sapiens
gi|53126859 from Gallus gallus
gi|54696574 from Homo sapiens
gi|61357151 from synthetic construct
gi|68534877 from Mus musculus
gi|74142500 from Mus musculus
gi|74180445 from Mus musculus
gi|74204749 from Mus musculus
gi|74212584 from Mus musculus
gi|74354242 from Bos taurus
gi|122936374 from Xenopus laevis
gi|123298589 from Mus musculus
gi|123980942 from synthetic construct
gi|148702790 from Mus musculus
gi|149055008 from Rattus norvegicus
gi|151554360 from Bos taurus
gi|157928272 (no taxonomy information for this entry)
gi|158257306 (no taxonomy information for this entry)

Fixed modifications: Carbamidomethyl (C)
Variable modifications: Oxidation (M)
Cleavage by Trypsin: cuts C-term side of KR unless next residue is P
Sequence Coverage: **23%**

Matched peptides shown in **Bold Red**

```
  1 MEEEIAALVI DNGSGMCKAG FAGDDAPRAV FPSIVGRPRH QGVMVGMGQK
 51 DSYVGDEAQS KRGILTLKYP IEHGIVTNWD DMEKIWHHTF YNELRVAPEE
101 HPVLLTEAPL NPKANREKMT QIMFETFNTP AMYVAIQAVL SLYASGRTTG
151 IVMDSGDGVT HTVPIYEGYA LPHAILRLDL AGRDLTDYLM KILTERGYSF
201 TTTAEREIVR DIKEKLCYVA LDFEQEMATA ASSSSLEKSY ELPDGQVITI
251 GNERFRCPEA LFQPSFLGME SCGIHETTFN SIMKCDVDIR KDLYANTVLS
301 GGTTMYPGIA DRMQKEITAL APSTMKIKII APPERKYSVW IGGSILASLS
351 TFQQMWISKQ EYDESGPSIV HRKCF
```

Residue Number Increasing Mass Decreasing Mass

Start - End	Observed	Mr(expt)	Mr(calc)	Delta	Miss	Sequence
1 - 18	1999.0100	1998.0027	1997.8743	0.1284	0	**MEEEIAALVIDNGSGMCK** 2 Oxidation (M) (No match)
197 - 206	1132.4774	1131.4701	1131.5196	-0.0495	0	**GYSFTTTAER** (Ions score 47)
197 - 206	1132.4774	1131.4701	1131.5196	-0.0495	0	**GYSFTTTAER** (No match)
239 - 254	1790.8201	1789.8128	1789.8846	-0.0717	0	**SYELPDGQVITIGNER** (No match)
239 - 254	1790.8201	1789.8128	1789.8846	-0.0717	0	**SYELPDGQVITIGNER** (Ions score 90)
292 - 312	2230.9722	2229.9649	2230.0575	-0.0926	0	**DLYANTVLSGGTTMYPGIADR** Oxidation (M) (No match)

292 - 312	2230.9722	2229.9649	2230.0575	-0.0926	0	DLYANTVLSGGTTMYPGIADR	Oxidation (M) (Ions score 15)
327 - 335	1036.6075	1035.6002	1035.6440	-0.0438	1	IKIIAPPER	(No match)
360 - 372	1516.6477	1515.6404	1515.6953	-0.0549	0	QEYDESGPSIVHR	(No match)
360 - 372	1516.6477	1515.6404	1515.6953	-0.0549	0	QEYDESGPSIVHR	(Ions score 74)
360 - 373	1644.7399	1643.7326	1643.7903	-0.0577	1	QEYDESGPSIVHRK	(No match)

Mascot: http://www.matrixscience.com/

Spot 703

Protein View

Match to: **gi|5453990** Score: **297** Expect: **3.8e-025**
proteasome activator subunit 1 isoform 1 [Homo sapiens]

Nominal mass (M_r): **28876**; Calculated pI value: **5.78**
NCBI BLAST search of gi|5453990 against nr
Unformatted sequence string for pasting into other applications

Taxonomy: Homo sapiens
Links to retrieve other entries containing this sequence from NCBI Entrez:
gi|114652286 from Pan troglodytes
gi|1170519 from Homo sapiens
gi|6048401 from Homo sapiens
gi|186513 from Homo sapiens
gi|551491 from Homo sapiens
gi|12653169 from Homo sapiens
gi|13960124 from Homo sapiens
gi|48145677 from Homo sapiens
gi|54695544 from Homo sapiens
gi|61357852 from synthetic construct
gi|61357856 from synthetic construct
gi|119586509 from Homo sapiens
gi|123981870 from synthetic construct
gi|123996697 from synthetic construct

Fixed modifications: Carbamidomethyl (C)
Variable modifications: Oxidation (M)
Cleavage by Trypsin: cuts C-term side of KR unless next residue is P
Sequence Coverage: **51%**

Matched peptides shown in **Bold Red**

1 MAMLRVQPEA QAK**VDVFRED** **LCTKTENLLG** **SYFPK**KISEL **DAFLKEPALN**
51 **EANLSNLKAP** **LDIPVPDPVK** **EK**EKEERKKQ QEKEDKDEKK KGEDEDKGPP
101 CGPVNCNEK**I** **VVLLQR**LKPE IKDVIEQLNL VTTWLQLQIP R**IEDGNNFGV**
151 **AVQEK**VFELM TSLHTK**LEGF** **HTQISK**YFSE RGDAVTKAAK **QPHVGDYRQL**
201 **VHELDEAEYR** DIR**LMVMEIR** **NAYAVLYDII** **LK**NFEKLKKP RGETKGMIY

Residue Number Increasing Mass Decreasing Mass

Start - End	Observed	Mr(expt)	Mr(calc)	Delta	Miss	Sequence
14 - 24	1381.6783	1380.6710	1380.6707	0.0003	1	VDVFREDLCTK (No match)
25 - 35	1268.6454	1267.6381	1267.6448	-0.0067	0	TENLLGSYFPK (No match)
37 - 58	2429.3030	2428.2957	2428.2849	0.0109	1	ISELDAFLKEPALNEANLSNLK (No match)
59 - 72	1517.8551	1516.8478	1516.8500	-0.0022	1	APLDIPVPDPVKEK (No match)
110 - 116	840.5675	839.5602	839.5592	0.0010	0	IVVLLQR (No match)
142 - 155	1519.7338	1518.7265	1518.7313	-0.0048	0	IEDGNNFGVAVQEK (No match)
167 - 176	1159.6067	1158.5994	1158.6033	-0.0038	0	LEGFHTQISK (No match)
191 - 198	971.4722	970.4649	970.4620	0.0029	0	QPHVGDYR (No match)
199 - 210	1501.7236	1500.7163	1500.7208	-0.0045	0	QLVHELDEAEYR (Ions score 67)
199 - 210	1501.7236	1500.7163	1500.7208	-0.0045	0	QLVHELDEAEYR (No match)
214 - 220	923.4633	922.4560	922.4616	-0.0055	0	LMVMEIR 2 Oxidation (M) (No match)
214 - 232	2284.2593	2283.2520	2283.2370	0.0150	1	LMVMEIRNAYAVLYDIILK Oxidation (M) (No match)
221 - 232	1395.7809	1394.7736	1394.7809	-0.0073	0	NAYAVLYDIILK (No match)
221 - 232	1395.7809	1394.7736	1394.7809	-0.0073	0	NAYAVLYDIILK (Ions score 75)

Mascot: http://www.matrixscience.com/

Spot 704

Protein View

Match to: **gi|4503143** Score: **181** Expect: **1.5e-013**
cathepsin D preproprotein [Homo sapiens]

Nominal mass (M_r): **45037**; Calculated pI value: **6.10**
NCBI BLAST search of gi|4503143 against nr
Unformatted sequence string for pasting into other applications

Taxonomy: Homo sapiens
Links to retrieve other entries containing this sequence from NCBI Entrez:
gi|115717 from Homo sapiens
gi|29678 from Homo sapiens
gi|179948 from Homo sapiens
gi|181180 from Homo sapiens
gi|16740920 from Homo sapiens
gi|30582659 from Homo sapiens
gi|48146011 from Homo sapiens
gi|54697170 from Homo sapiens
gi|61356567 from synthetic construct
gi|61362282 from synthetic construct
gi|119622866 from Homo sapiens
gi|119622867 from Homo sapiens
gi|119622868 from Homo sapiens
gi|123994405 from synthetic construct

Fixed modifications: Carbamidomethyl (C)
Variable modifications: Oxidation (M)
Cleavage by Trypsin: cuts C-term side of KR unless next residue is P
Sequence Coverage: **24%**

Matched peptides shown in **Bold Red**

```
  1 MQPSSLLPLA LCLLAAPASA LVRIPLHKFT SIRRTMSEVG GSVEDLIAKG
 51 PVSKYSQAVP AVTEGPIPEV LKNYMDAQYY GEIGIGTPPQ CFTVVFDTGS
101 SNLWVPSIHC KLLDIACWIH HKYNSDKSST YVKNGTSFDI HYGSGSLSGY
151 LSQDTVSVPC QSASSASALG GVKVERQVFG EATKQPGITF IAAKFDGILG
201 MAYPRISVNN VLPVFDNLMQ QKLVDQNIFS FYLSRDPDAQ PGGELMLGGT
251 DSKYYKGSLS YLNVTRKAYW QVHLDQVEVA SGLTLCKEGC EAIVDTGTSL
301 MVGPVDEVRE LQKAIGAVPL IQGEYMIPCE KVSTLPAITL KLGGKGYKLS
351 PEDYTLKVSQ AGKTLCLSGF MGMDIPPPSG PLWILGDVFI GRYYTVFDRD
401 NNRVGFAEAA RL
```

Residue Number Increasing Mass Decreasing Mass

Start - End	Observed	Mr(expt)	Mr(calc)	Delta	Miss	Sequence
195 - 205	1239.5951	1238.5878	1238.6117	-0.0239	0	FDGILGMAYPR (No match)
195 - 205	1255.5863	1254.5790	1254.6066	-0.0276	0	FDGILGMAYPR Oxidation (M) (Ions score 20)
195 - 205	1255.5863	1254.5790	1254.6066	-0.0276	0	FDGILGMAYPR Oxidation (M) (No match)
206 - 222	1975.0043	1973.9970	1974.0244	-0.0273	0	ISVNNVLPVFDNLMQQK Oxidation (M) (No match)
223 - 235	1601.7997	1600.7924	1600.8249	-0.0325	0	LVDQNIFSFYLSR (Ions score 51)
223 - 235	1601.7997	1600.7924	1600.8249	-0.0325	0	LVDQNIFSFYLSR (No match)
288 - 309	2350.0657	2349.0584	2349.0827	-0.0243	0	EGCEAIVDTGTSLMVGPVDEVR Oxidation (M) (No match)
314 - 331	2004.9833	2003.9760	2004.0059	-0.0299	0	AIGAVPLIQGEYMIPCEK Oxidation (M) (No match)
393 - 399	963.4426	962.4353	962.4497	-0.0144	0	YYTVFDR (No match)
393 - 403	1462.6412	1461.6339	1461.6636	-0.0297	1	YYTVFDRDNNR (No match)
393 - 403	1462.6412	1461.6339	1461.6636	-0.0297	1	YYTVFDRDNNR (Ions score 21)
404 - 411	820.4233	819.4160	819.4238	-0.0078	0	VGFAEAAR (No match)

Mascot: http://www.matrixscience.com/

Spot 706

Protein View

Match to: **gi|28071074** Score: **350** Expect: **1.9e-030**
unnamed protein product [Homo sapiens]

Nominal mass (M_r): **13945**; Calculated pI value: **9.20**
NCBI BLAST search of gi|28071074 against nr
Unformatted sequence string for pasting into other applications

Taxonomy: Homo sapiens

Fixed modifications: Carbamidomethyl (C)
Variable modifications: Oxidation (M)
Cleavage by Trypsin: cuts C-term side of KR unless next residue is P
Sequence Coverage: **64%**

Matched peptides shown in **Bold Red**

```
  1 MLITILGTVK PNANRIALDF QRGNDVAFHF NPRFNENNRR VIVCNTKLDN
 51 NWGREERQSV FPFESGKPFK IQVLVEPDHF KVAVNDAHLL QYNHRVKKLN
101 EISKLGISGD IDLTSASYTM I
```

Residue Number Increasing Mass Decreasing Mass

Start - End	Observed	Mr(expt)	Mr(calc)	Delta	Miss	Sequence
1 - 15	1656.9362	1655.9289	1655.9392	-0.0103	0	MLITILGTVKPNANR Oxidation (M) (No match)
16 - 22	862.4772	861.4699	861.4708	-0.0009	0	IALDFQR (No match)
23 - 33	1273.6002	1272.5929	1272.5999	-0.0070	0	GNDVAFHFNPR (Ions score 59)
23 - 33	1273.6002	1272.5929	1272.5999	-0.0070	0	GNDVAFHFNPR (No match)
34 - 40	949.4548	948.4475	948.4525	-0.0050	1	FNENNRR (No match)
58 - 70	1497.7632	1496.7559	1496.7663	-0.0104	0	QSVFPFESGKPFK (No match)
58 - 70	1497.7632	1496.7559	1496.7663	-0.0104	0	QSVFPFESGKPFK (Ions score 80)
71 - 81	1324.7136	1323.7063	1323.7186	-0.0123	0	IQVLVEPDHFK (No match)
71 - 81	1324.7136	1323.7063	1323.7186	-0.0123	0	IQVLVEPDHFK (Ions score 24)
82 - 95	1649.8425	1648.8352	1648.8433	-0.0081	0	VAVNDAHLLQYNHR (No match)
82 - 95	1649.8425	1648.8352	1648.8433	-0.0081	0	VAVNDAHLLQYNHR (Ions score 76)

Spot 707

Protein View

Match to: **gi|33357774** Score: **324** Expect: **7.7e-028**
Chain A, The Oligomeric Structure Of Human Granzyme A Reveals The Molecular Determinants Of Substra

Nominal mass (M_r): **26328**; Calculated pI value: **9.13**
NCBI BLAST search of gi|33357774 against nr
Unformatted sequence string for pasting into other applications

Taxonomy: Homo sapiens

Fixed modifications: Carbamidomethyl (C)
Variable modifications: Oxidation (M)
Cleavage by Trypsin: cuts C-term side of KR unless next residue is P
Sequence Coverage: **30%**

Matched peptides shown in **Bold Red**

```
  1 IIGGNEVTPH SRPYMVLLSL DRKTICAGAL IAKDWVLTAA HCNLNKRSQV
 51 ILGAHSITRE EPTKQIMLVK KEFPYPCYDP ATREGDLKLL QLTEKAKINK
101 YVTILHLPKK GDDVKPGTMC QVAGWGRTHN SASWSDTLRE VEITIIDRKV
151 CNDRNHYNFN PVIGMNMVCA GSLRGGRDSC NGDSGSPLLC EGVFRGVTSF
201 GLENKCGDPR GPGVYILLSK KHLNWIIMTI KGAV
```

Residue Number Increasing Mass Decreasing Mass

Start - End	Observed	Mr(expt)	Mr(calc)	Delta	Miss	Sequence
48 - 59	1281.7094	1280.7021	1280.7200	-0.0179	0	SQVILGAHSITR (No match)
48 - 59	1281.7094	1280.7021	1280.7200	-0.0179	0	SQVILGAHSITR (Ions score 13)
71 - 83	1643.7240	1642.7167	1642.7449	-0.0282	1	KEFPYPCYDPATR (Ions score 35)
71 - 83	1643.7240	1642.7167	1642.7449	-0.0282	1	KEFPYPCYDPATR (No match)
72 - 83	1515.6300	1514.6227	1514.6500	-0.0273	0	EFPYPCYDPATR (No match)
72 - 83	1515.6300	1514.6227	1514.6500	-0.0273	0	EFPYPCYDPATR (Ions score 57)
101 - 109	1083.6312	1082.6239	1082.6488	-0.0248	0	YVTILHLPK (No match)
155 - 174	2326.0317	2325.0244	2325.0452	-0.0208	0	NHYNFNPVIGMNMVCAGSLR 2 Oxidation (M) (No match)
155 - 174	2326.0317	2325.0244	2325.0452	-0.0208	0	NHYNFNPVIGMNMVCAGSLR 2 Oxidation (M) (Ions score 14)
178 - 195	1969.7999	1968.7926	1968.8305	-0.0379	0	DSCNGDSGSPLLCEGVFR (No match)
178 - 195	1969.7999	1968.7926	1968.8305	-0.0379	0	DSCNGDSGSPLLCEGVFR (Ions score 114)

Mascot: http://www.matrixscience.com/

Spot 708

Protein View

Match to: **gi|49119653** Score: **546** Expect: **4.8e-050**
YWHAZ protein [Homo sapiens]

Nominal mass (M_r): **30100**; Calculated pI value: **4.72**
NCBI BLAST search of gi|49119653 against nr
Unformatted sequence string for pasting into other applications

Taxonomy: Homo sapiens

Fixed modifications: Carbamidomethyl (C)
Variable modifications: Oxidation (M)
Cleavage by Trypsin: cuts C-term side of KR unless next residue is P
Sequence Coverage: **62%**

Matched peptides shown in **Bold Red**

```
  1 WTAEPVRPPP PPTPDTEHPV MDKNELVQKA KLAEQAERYD DMAACMKSVT
 51 EQGAELSNEE RNLLSVAYKN VVGARRSSWR VVSSIEQKTE GAEKKQQMAR
101 EYREKIETEL RDICNDVLSL LEKFLIPNAS QAESKVFYLK MKGDYYRYLA
151 EVAAGDDKKG IVDQSQQAYQ EAFEISKKEM QPTHPIRLGL ALNFSVFYYE
201 ILNSPEKACS LAKTAFDEAI AELDTLSEES YKDSTLIMQL LRDNLTLWTS
251 DTQGDEAEAG EGGEN
```

Residue Number Increasing Mass Decreasing Mass

Start - End	Observed	Mr(expt)	Mr(calc)	Delta	Miss	Sequence
32 - 38	816.4592	815.4519	815.4137	0.0382	0	LAEQAER (No match)
48 - 61	1548.7548	1547.7475	1547.7063	0.0413	0	SVTEQGAELSNEER (Ions score 74)
48 - 61	1548.7548	1547.7475	1547.7063	0.0413	0	SVTEQGAELSNEER (No match)
62 - 69	907.5486	906.5413	906.5174	0.0239	0	NLLSVAYK (No match)
81 - 88	889.5239	888.5166	888.4916	0.0250	0	VVSSIEQK (No match)
104 - 111	1017.5992	1016.5919	1016.5502	0.0418	1	EKIETELR (No match)
106 - 111	760.4581	759.4508	759.4126	0.0382	0	IETELR (No match)
112 - 123	1418.7512	1417.7439	1417.7122	0.0317	0	DICNDVLSLLEK (No match)
124 - 135	1304.7158	1303.7085	1303.6772	0.0314	0	FLIPNASQAESK (Ions score 57)
124 - 135	1304.7158	1303.7085	1303.6772	0.0314	0	FLIPNASQAESK (No match)
141 - 147	932.4692	931.4619	931.4221	0.0398	1	MKGDYYR (No match)

141 - 147	948.4523	947.4450	947.4171	0.0280	1	MKGDYYR Oxidation (M) (No match)
148 - 159	1279.6882	1278.6809	1278.6455	0.0354	1	YLAEVAAGDDKK (No match)
160 - 177	2041.0315	2040.0242	2039.9799	0.0443	0	GIVDQSQQAYQEAFEISK (No match)
160 - 177	2041.0315	2040.0242	2039.9799	0.0443	0	GIVDQSQQAYQEAFEISK (Ions score 132)
160 - 178	2169.1301	2168.1228	2168.0749	0.0479	1	GIVDQSQQAYQEAFEISKK (No match)
178 - 187	1236.6777	1235.6704	1235.6445	0.0260	1	KEMQPTHPIR (No match)
178 - 187	1252.6681	1251.6608	1251.6394	0.0215	1	KEMQPTHPIR Oxidation (M) (No match)
179 - 187	1108.5956	1107.5883	1107.5495	0.0388	0	EMQPTHPIR (No match)
179 - 187	1124.5817	1123.5744	1123.5444	0.0300	0	EMQPTHPIR Oxidation (M) (No match)
188 - 207	2317.2642	2316.2569	2316.2041	0.0529	0	LGLALNFSVFYYEILNSPEK (No match)
214 - 232	2132.0413	2131.0340	2130.9844	0.0496	0	TAFDEAIAELDTLSEESYK (No match)
233 - 242	1189.7015	1188.6942	1188.6536	0.0406	0	DSTLIMQLLR (Ions score 55)
233 - 242	1189.7015	1188.6942	1188.6536	0.0406	0	DSTLIMQLLR (No match)
233 - 242	1205.6873	1204.6800	1204.6485	0.0315	0	DSTLIMQLLR Oxidation (M) (Ions score 37)
233 - 242	1205.6873	1204.6800	1204.6485	0.0315	0	DSTLIMQLLR Oxidation (M) (No match)

Mascot: http://www.matrixscience.com/

Spot 709

Protein View

Match to: **gi|4503143** Score: **250** Expect: **1.9e-020**
cathepsin D preproprotein [Homo sapiens]

Nominal mass (M_r): **45037;** Calculated pI value: **6.10**
NCBI BLAST search of gi|4503143 against nr
Unformatted sequence string for pasting into other applications

Taxonomy: Homo sapiens
Links to retrieve other entries containing this sequence from NCBI Entrez:
gi|115717 from Homo sapiens
gi|29678 from Homo sapiens
gi|179948 from Homo sapiens
gi|181180 from Homo sapiens
gi|16740920 from Homo sapiens
gi|30582659 from Homo sapiens
gi|48146011 from Homo sapiens
gi|54697170 from Homo sapiens
gi|61356567 from synthetic construct
gi|61362282 from synthetic construct
gi|119622866 from Homo sapiens
gi|119622867 from Homo sapiens
gi|119622868 from Homo sapiens
gi|123994405 from synthetic construct

Fixed modifications: Carbamidomethyl (C)
Variable modifications: Oxidation (M)
Cleavage by Trypsin: cuts C-term side of KR unless next residue is P
Sequence Coverage: **24%**

Matched peptides shown in **Bold Red**

```
  1 MQPSSLLPLA LCLLAAPASA LVRIPLHKFT SIRRTMSEVG GSVEDLIAKG
 51 PVSKYSQAVP AVTEGPIPEV LKNYMDAQYY GEIGIGTPPQ CFTVVFDTGS
101 SNLWVPSIHC KLLDIACWIH HKYNSDKSST YVKNGTSFDI HYGSGSLSGY
151 LSQDTVSVPC QSASSASALG GVKVERQVFG EATKQPGITF IAAKFDGILG
201 MAYPRISVNN VLPVFDNLMQ QKLVDQNIFS FYLSRDPDAQ PGGELMLGGT
251 DSKYYKGSLS YLNVTRKAYW QVHLDQVEVA SGLTLCKEGC EAIVDTGTSL
301 MVGPVDEVRE LQKAIGAVPL IQGEYMIPCE KVSTLPAITL KLGGKGYKLS
351 PEDYTLKVSQ AGKTLCLSGF MGMDIPPPSG PLWILGDVFI GRYYTVFDRD
401 NNRVGFAEAA RL
```

Residue Number Increasing Mass Decreasing Mass

Start - End	Observed	Mr(expt)	Mr(calc)	Delta	Miss	Sequence
195 - 205	1239.5865	1238.5792	1238.6117	-0.0325	0	FDGILGMAYPR (Ions score 35)
195 - 205	1239.5865	1238.5792	1238.6117	-0.0325	0	FDGILGMAYPR (No match)
195 - 205	1255.5747	1254.5674	1254.6066	-0.0392	0	FDGILGMAYPR Oxidation (M) (No match)
195 - 205	1255.5747	1254.5674	1254.6066	-0.0392	0	FDGILGMAYPR Oxidation (M) (Ions score 23)
206 - 222	1974.9825	1973.9752	1974.0244	-0.0491	0	ISVNNVLPVFDNLMQQK Oxidation (M) (No match)
223 - 235	1601.7845	1600.7772	1600.8249	-0.0477	0	LVDQNIFSFYLSR (No match)
223 - 235	1601.7845	1600.7772	1600.8249	-0.0477	0	LVDQNIFSFYLSR (Ions score 80)
288 - 309	2334.0291	2333.0218	2333.0878	-0.0660	0	EGCEAIVDTGTSLMVGPVDEVR (No match)
288 - 309	2350.0325	2349.0252	2349.0827	-0.0575	0	EGCEAIVDTGTSLMVGPVDEVR Oxidation (M) (No match)
314 - 331	2004.9648	2003.9575	2004.0059	-0.0484	0	AIGAVPLIQGEYMIPCEK Oxidation (M) (No match)
393 - 399	963.4293	962.4220	962.4497	-0.0277	0	YYTVFDR (No match)
393 - 403	1462.6260	1461.6187	1461.6636	-0.0449	1	YYTVFDRDNNR (No match)
393 - 403	1462.6260	1461.6187	1461.6636	-0.0449	1	YYTVFDRDNNR (Ions score 35)
404 - 411	820.4118	819.4045	819.4238	-0.0193	0	VGFAEAAR (No match)

Mascot: http://www.matrixscience.com/

Spot 712

Protein View

Match to: **gi|5822002** Score: **403** Expect: **9.6e-036**
Chain A, Profilin Binds Proline-Rich Ligands In Two Distinct Amide Backbone Orientations

Nominal mass (M_r): **15085**; Calculated pI value: **8.48**
NCBI BLAST search of gi|5822002 against nr
Unformatted sequence string for pasting into other applications

Taxonomy: Homo sapiens
Links to retrieve other entries containing this sequence from NCBI Entrez:
gi|5822004 from Homo sapiens
gi|157831080 (no taxonomy information for this entry)
gi|157831081 (no taxonomy information for this entry)
gi|159795064 (no taxonomy information for this entry)
gi|160285766 (no taxonomy information for this entry)

Fixed modifications: Carbamidomethyl (C)
Variable modifications: Oxidation (M)
Cleavage by Trypsin: cuts C-term side of KR unless next residue is P
Sequence Coverage: **69%**

Matched peptides shown in **Bold Red**

1 AGWNAYIDNL MADGTCQDAA IVGYKDSPSV WAAVPGK**TFV NITPAEVGVL**
51 VGKDRSSFYV NGLTLGGQKC SVIRDSLLQD GEFSMDLRTK **STGGAPTFNV**
101 TVTKTDKTLV LLMGKEGVHG GLINKKCYEM ASHLRRSQY

Residue Number Increasing Mass Decreasing Mass

Start - End	Observed	Mr(expt)	Mr(calc)	Delta	Miss	Sequence
38 - 53	1643.9528	1642.9455	1642.9293	0.0162	0	TFVNITPAEVGVLVGK (No match)
38 - 55	1915.0970	1914.0897	1914.0574	0.0324	1	TFVNITPAEVGVLVGKDR (Ions score 104)
38 - 55	1915.0970	1914.0897	1914.0574	0.0324	1	TFVNITPAEVGVLVGKDR (No match)
54 - 69	1741.9130	1740.9057	1740.8794	0.0263	1	DRSSFYVNGLTLGGQK (No match)
56 - 69	1470.7637	1469.7564	1469.7514	0.0051	0	SSFYVNGLTLGGQK (No match)
56 - 69	1470.7637	1469.7564	1469.7514	0.0051	0	SSFYVNGLTLGGQK (Ions score 118)
70 - 88	2257.1238	2256.1165	2256.0514	0.0651	1	CSVIRDSLLQDGEFSMDLR Oxidation (M) (No match)
75 - 88	1641.7604	1640.7531	1640.7352	0.0180	0	DSLLQDGEFSMDLR Oxidation (M) (No match)

75 - 88	1641.7604	1640.7531	1640.7352	0.0180	0	**DSLLQDGEFSMDLR**	Oxidation (M) (Ions score 26)
91 - 104	1379.7164	1378.7091	1378.7092	-0.0001	0	**STGGAPTFNVTVTK**	(No match)
105 - 115	1234.6924	1233.6851	1233.7002	-0.0151	1	**TDKTLVLLMGK**	Oxidation (M) (No match)
116 - 126	1151.6511	1150.6438	1150.6458	-0.0019	1	**EGVHGGLINKK**	(No match)
127 - 135	1182.4792	1181.4719	1181.4957	-0.0238	0	**CYEMASHLR**	Oxidation (M) (No match)
127 - 136	1338.5962	1337.5889	1337.5968	-0.0079	1	**CYEMASHLRR**	Oxidation (M) (No match)

Mascot: http://www.matrixscience.com/

Spot 714

Protein View

Match to: **gi|4507949** Score: **313** Expect: **9.6e-027**
tyrosine 3-monooxygenase/tryptophan 5-monooxygenase activation protein, beta polypeptide [Homo sapi

Nominal mass (M_r): **28179**; Calculated pI value: **4.76**
NCBI BLAST search of gi|4507949 against nr
Unformatted sequence string for pasting into other applications

Taxonomy: Homo sapiens
Links to retrieve other entries containing this sequence from NCBI Entrez:
gi|21328448 from Homo sapiens
gi|115432055 from Bos taurus
gi|1345590 from Homo sapiens
gi|75075938 from Macaca fascicularis
gi|148841384 from Pongo pygmaeus
gi|23114 from Homo sapiens
gi|2791552 from Homo sapiens
gi|12655023 from Homo sapiens
gi|67970822 from Macaca fascicularis
gi|90075234 from Macaca fascicularis
gi|111307057 from Bos taurus
gi|119596299 from Homo sapiens
gi|119596300 from Homo sapiens
gi|119596302 from Homo sapiens
gi|134093124 from Pongo pygmaeus
gi|158258871 (no taxonomy information for this entry)

Fixed modifications: Carbamidomethyl (C)
Variable modifications: Oxidation (M)
Cleavage by Trypsin: cuts C-term side of KR unless next residue is P
Sequence Coverage: **39%**

Matched peptides shown in **Bold Red**

1 MTMDKSELVQ KAK**LAEQAER** YDDMAAAMK**A VTEQGHELSN EER**NLLSVAY
51 KNVVGARRSS WR**VISSIEQK** TERNEKKQQM GKEYREKIEA ELQDICNDVL
101 ELLDK**YLIPN ATQPESK**VFY LK**MKGDYFRY LSEVASGDNK QTTVSNSQQA**
151 **YQEAFEISKK EMQPTHPIR**L GLALNFSVFY YEILNSPEKA CSLAKTAFDE
201 AIAELDTLNE ESYK**DSTLIM QLLR**DNLTLW TSENQGDEGD AGEGEN

Residue Number Increasing Mass Decreasing Mass

Start - End	Observed	Mr(expt)	Mr(calc)	Delta	Miss	Sequence
14 - 20	816.4529	815.4456	815.4137	0.0319	0	LAEQAER (No match)
30 - 43	1598.7681	1597.7608	1597.7332	0.0277	0	AVTEQGHELSNEER (No match)
30 - 43	1598.7681	1597.7608	1597.7332	0.0277	0	AVTEQGHELSNEER (Ions score 86)
63 - 70	903.5302	902.5229	902.5072	0.0157	0	VISSIEQK (No match)
106 - 117	1360.7400	1359.7327	1359.7034	0.0293	0	YLIPNATQPESK (No match)
123 - 129	916.4695	915.4622	915.4272	0.0350	1	MKGDYFR (No match)
123 - 129	932.4575	931.4502	931.4221	0.0281	1	MKGDYFR Oxidation (M) (No match)
130 - 140	1182.5939	1181.5866	1181.5564	0.0303	0	YLSEVASGDNK (No match)
141 - 159	2159.0447	2158.0374	2158.0178	0.0196	0	QTTVSNSQQAYQEAFEISK (No match)
141 - 159	2159.0447	2158.0374	2158.0178	0.0196	0	QTTVSNSQQAYQEAFEISK (Ions score 60)
141 - 160	2287.1602	2286.1529	2286.1127	0.0402	1	QTTVSNSQQAYQEAFEISKK (No match)
160 - 169	1252.6609	1251.6536	1251.6394	0.0143	1	KEMQPTHPIR Oxidation (M) (No match)
161 - 169	1108.5922	1107.5849	1107.5495	0.0354	0	EMQPTHPIR (No match)
161 - 169	1124.5753	1123.5680	1123.5444	0.0236	0	EMQPTHPIR Oxidation (M) (No match)
215 - 224	1189.6899	1188.6826	1188.6536	0.0290	0	DSTLIMQLLR (Ions score 29)
215 - 224	1189.6899	1188.6826	1188.6536	0.0290	0	DSTLIMQLLR (No match)
215 - 224	1205.6780	1204.6707	1204.6485	0.0222	0	DSTLIMQLLR Oxidation (M) (Ions score 36)
215 - 224	1205.6780	1204.6707	1204.6485	0.0222	0	DSTLIMQLLR Oxidation (M) (No match)

Mascot: http://www.matrixscience.com/

Spot 716

Protein View

Match to: **gi|4757768** Score: **279** Expect: **2.4e-023**
Rho GDP dissociation inhibitor (GDI) alpha [Homo sapiens]

Nominal mass (M_r): **23250**; Calculated pI value: **5.02**
NCBI BLAST search of gi|4757768 against nr
Unformatted sequence string for pasting into other applications

Taxonomy: Homo sapiens
Links to retrieve other entries containing this sequence from NCBI Entrez:
gi|109119114 from Macaca mulatta
gi|114671004 from Pan troglodytes
gi|114671006 from Pan troglodytes
gi|1707892 from Homo sapiens
gi|6980758 from Homo sapiens
gi|6980760 from Homo sapiens
gi|15826632 from Homo sapiens
gi|15826633 from Homo sapiens
gi|20379028 from Homo sapiens
gi|285979 from Homo sapiens
gi|337395 from Homo sapiens
gi|456191 from Homo sapiens
gi|13543381 from Homo sapiens
gi|13543436 from Homo sapiens
gi|14250508 from Homo sapiens
gi|14602503 from Homo sapiens
gi|16359132 from Homo sapiens
gi|16740603 from Homo sapiens
gi|18999474 from Homo sapiens
gi|20380701 from Homo sapiens
gi|30582607 from Homo sapiens
gi|48145671 from Homo sapiens
gi|49899246 from Homo sapiens
gi|61362947 from synthetic construct
gi|67971286 from Macaca fascicularis
gi|76780069 from Homo sapiens
gi|90075918 from Macaca fascicularis
gi|119610103 from Homo sapiens
gi|119610104 from Homo sapiens
gi|123980428 from synthetic construct
gi|123995241 from synthetic construct

```
Fixed modifications: Carbamidomethyl (C)
Variable modifications: Oxidation (M)
Cleavage by Trypsin: cuts C-term side of KR unless next residue is P
Sequence Coverage: 38%

Matched peptides shown in Bold Red

     1 MAEQEPTAEQ LAQIAAENEE DEHSVNYKPP AQKSIQEIQE LDKDDESLRK
    51 YKEALLGRVA VSADPNVPNV VVTGLTLVCS SAPGPLELDL TGDLESFKKQ
   101 SFVLKEGVEY RIKISFRVNR EIVSGMKYIQ HTYRKGVKID KTDYMVGSYG
   151 PRAEEYEFLT PVEEAPKGML ARGSYSIKSR FTDDDKTDHL SWEWNLTIKK
   201 DWKD
```

Residue Number Increasing Mass Decreasing Mass

Start - End	Observed	Mr(expt)	Mr(calc)	Delta	Miss	Sequence
34 - 49	1917.9840	1916.9767	1916.9327	0.0441	1	SIQEIQELDKDDESLR (No match)
34 - 49	1917.9840	1916.9767	1916.9327	0.0441	1	SIQEIQELDKDDESLR (Ions score 48)
51 - 58	949.5374	948.5301	948.5392	-0.0091	1	YKEALLGR (No match)
100 - 111	1454.7880	1453.7807	1453.7564	0.0243	1	QSFVLKEGVEYR (No match)
100 - 111	1454.7880	1453.7807	1453.7564	0.0243	1	QSFVLKEGVEYR (Ions score 11)
128 - 134	980.4889	979.4816	979.4875	-0.0059	0	YIQHTYR (No match)
128 - 135	1108.5930	1107.5857	1107.5825	0.0032	1	YIQHTYRK (No match)
139 - 152	1617.7872	1616.7799	1616.7504	0.0295	1	IDKTDYMVGSYGPR Oxidation (M) (Ions score 24)
139 - 152	1617.7872	1616.7799	1616.7504	0.0295	1	IDKTDYMVGSYGPR Oxidation (M) (No match)
142 - 152	1261.5624	1260.5551	1260.5444	0.0107	0	TDYMVGSYGPR Oxidation (M) (No match)
153 - 167	1751.8779	1750.8706	1750.8301	0.0406	0	AEEYEFLTPVEEAPK (Ions score 80)
153 - 167	1751.8779	1750.8706	1750.8301	0.0406	0	AEEYEFLTPVEEAPK (No match)
153 - 172	2296.1860	2295.1787	2295.1092	0.0695	1	AEEYEFLTPVEEAPKGMLAR Oxidation (M) (No match)

Mascot: http://www.matrixscience.com/

Spot 717

Protein View

Match to: **gi|33357774** Score: **372** Expect: **1.2e-032**
Chain A, The Oligomeric Structure Of Human Granzyme A Reveals The Molecular Determinants Of Substra

Nominal mass (M_r): **26328**; Calculated pI value: **9.13**
NCBI BLAST search of gi|33357774 against nr
Unformatted sequence string for pasting into other applications

Taxonomy: Homo sapiens

Fixed modifications: Carbamidomethyl (C)
Variable modifications: Oxidation (M)
Cleavage by Trypsin: cuts C-term side of KR unless next residue is P
Sequence Coverage: **35%**

Matched peptides shown in **Bold Red**

```
  1 IIGGNEVTPH SRPYMVLLSL DRKTICAGAL IAKDWVLTAA HCNLNKRSQV
 51 ILGAHSITRE EPTKQIMLVK KEFPYPCYDP ATREGDLKLL QLTEKAKINK
101 YVTILHLPKK GDDVKPGTMC QVAGWGRTHN SASWSDTLRE VEITIIDRKV
151 CNDRNHYNFN PVIGMNMVCA GSLRGGRDSC NGDSGSPLLC EGVFRGVTSF
201 GLENKCGDPR GPGVYILLSK KHLNWIIMTI KGAV
```

Residue Number Increasing Mass Decreasing Mass

Start - End	Observed	Mr(expt)	Mr(calc)	Delta	Miss	Sequence
48 - 59	1281.7371	1280.7298	1280.7200	0.0098	0	SQVILGAHSITR (No match)
48 - 59	1281.7371	1280.7298	1280.7200	0.0098	0	SQVILGAHSITR (Ions score 36)
71 - 83	1643.7621	1642.7548	1642.7449	0.0099	1	KEFPYPCYDPATR (No match)
71 - 83	1643.7621	1642.7548	1642.7449	0.0099	1	KEFPYPCYDPATR (Ions score 61)
72 - 83	1515.6644	1514.6571	1514.6500	0.0071	0	EFPYPCYDPATR (Ions score 70)
72 - 83	1515.6644	1514.6571	1514.6500	0.0071	0	EFPYPCYDPATR (No match)
101 - 109	1083.6577	1082.6504	1082.6488	0.0017	0	YVTILHLPK (No match)
128 - 139	1374.6466	1373.6393	1373.6324	0.0070	0	THNSASWSDTLR (No match)
155 - 174	2310.0806	2309.0733	2309.0503	0.0230	0	NHYNFNPVIGMNMVCAGSLR Oxidation (M) (No match)
155 - 174	2326.0840	2325.0767	2325.0452	0.0315	0	NHYNFNPVIGMNMVCAGSLR 2 Oxidation (M) (No match)
178 - 195	1969.8484	1968.8411	1968.8305	0.0106	0	DSCNGDSGSPLLCEGVFR (Ions score 110)
178 - 195	1969.8484	1968.8411	1968.8305	0.0106	0	DSCNGDSGSPLLCEGVFR (No match)

Spot 718

Protein View

Match to: **gi|45786143** Score: **352** Expect: **1.2e-030**
Lectin, galactoside-binding, soluble, 3 [Homo sapiens]

Nominal mass (M_r): **26238**; Calculated pI value: **8.58**
NCBI BLAST search of gi|45786143 against nr
Unformatted sequence string for pasting into other applications

Taxonomy: Homo sapiens

Fixed modifications: Carbamidomethyl (C)
Variable modifications: Oxidation (M)
Cleavage by Trypsin: cuts C-term side of KR unless next residue is P
Sequence Coverage: **31%**

Matched peptides shown in **Bold Red**

```
  1 MADNFSLHDA LSGSGNPNPQ GWPGAWGNQP AGAGGYPGAS YPGAYPGQAP
 51 PGAYPGQAPP GAYHGAPGAY PGAPAPGVYP GPPSGPGAYP SSGQPSAPGA
101 YPATGPYGAP AGPLIVPYNL PLPGGVVPRM LITILGTVKP NANRIALDFQ
151 RGNDVAFHFN PRFNENNRRV IVCNTKLDNN WGREERQSVF PFESGKPFKI
201 HVLVEPDHFK VAVNDAHLLQ YNHRVKKLNE ISKLGISGDI DLTSASYTMI
251
```

Residue Number Increasing Mass Decreasing Mass

Start - End	Observed	Mr(expt)	Mr(calc)	Delta	Miss	Sequence
130 - 144	1656.9274	1655.9201	1655.9392	-0.0191	0	MLITILGTVKPNANR Oxidation (M) (No match)
145 - 151	862.4792	861.4719	861.4708	0.0011	0	IALDFQR (No match)
152 - 162	1273.6000	1272.5927	1272.5999	-0.0072	0	GNDVAFHFNPR (Ions score 81)
152 - 162	1273.6000	1272.5927	1272.5999	-0.0072	0	GNDVAFHFNPR (No match)
163 - 169	949.4575	948.4502	948.4525	-0.0023	1	FNENNRR (No match)
187 - 199	1497.7546	1496.7473	1496.7663	-0.0190	0	QSVFPFESGKPFK (No match)
187 - 199	1497.7546	1496.7473	1496.7663	-0.0190	0	QSVFPFESGKPFK (Ions score 80)
200 - 210	1333.7134	1332.7061	1332.7190	-0.0128	0	IHVLVEPDHFK (No match)
211 - 224	1649.8337	1648.8264	1648.8433	-0.0169	0	VAVNDAHLLQYNHR (No match)
211 - 224	1649.8337	1648.8264	1648.8433	-0.0169	0	VAVNDAHLLQYNHR (Ions score 89)

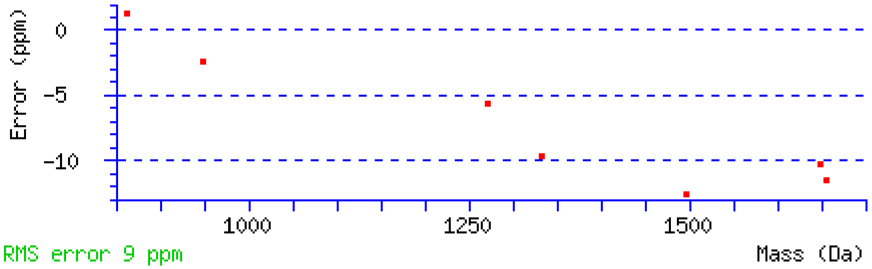

Mascot: http://www.matrixscience.com/

Spot 720

Protein View

Match to: **gi|33357774** Score: **448** Expect: **3e-040**
Chain A, The Oligomeric Structure Of Human Granzyme A Reveals The Molecular Determinants Of Substra

Nominal mass (M_r): **26328**; Calculated pI value: **9.13**
NCBI BLAST search of gi|33357774 against nr
Unformatted sequence string for pasting into other applications

Taxonomy: Homo sapiens

Fixed modifications: Carbamidomethyl (C)
Variable modifications: Oxidation (M)
Cleavage by Trypsin: cuts C-term side of KR unless next residue is P
Sequence Coverage: **51%**

Matched peptides shown in **Bold Red**

```
  1 IIGGNEVTPH SRPYMVLLSL DRKTICAGAL IAKDWVLTAA HCNLNKRSQV
 51 ILGAHSITRE EPTKQIMLVK KEFPYPCYDP ATREGDLKLL QLTEKAKINK
101 YVTILHLPKK GDDVKPGTMC QVAGWGRTHN SASWSDTLRE VEITIIDRKV
151 CNDRNHYNFN PVIGMNMVCA GSLRGGRDSC NGDSGSPLLC EGVFRGVTSF
201 GLENKCGDPR GPGVYILLSK KHLNWIIMTI KGAV
```

Residue Number Increasing Mass Decreasing Mass

Start - End	Observed	Mr(expt)	Mr(calc)	Delta	Miss	Sequence
1 - 23	2611.3950	2610.3877	2610.3951	-0.0074	1	IIGGNEVTPHSRPYMVLLSLDRK Oxidation (M) (No match)
34 - 47	1697.8330	1696.8257	1696.8467	-0.0210	1	DWVLTAAHCNLNKR (No match)
48 - 59	1281.7157	1280.7084	1280.7200	-0.0116	0	SQVILGAHSITR (No match)
48 - 59	1281.7157	1280.7084	1280.7200	-0.0116	0	SQVILGAHSITR (Ions score 59)
71 - 83	1643.7332	1642.7259	1642.7449	-0.0190	1	KEFPYPCYDPATR (No match)
71 - 83	1643.7332	1642.7259	1642.7449	-0.0190	1	KEFPYPCYDPATR (Ions score 73)
72 - 83	1515.6393	1514.6320	1514.6500	-0.0180	0	EFPYPCYDPATR (Ions score 74)
72 - 83	1515.6393	1514.6320	1514.6500	-0.0180	0	EFPYPCYDPATR (No match)
101 - 109	1083.6412	1082.6339	1082.6488	-0.0148	0	YVTILHLPK (No match)
128 - 139	1374.6245	1373.6172	1373.6324	-0.0151	0	THNSASWSDTLR (No match)
155 - 174	2310.0383	2309.0310	2309.0503	-0.0193	0	NHYNFNPVIGMNMVCAGSLR Oxidation (M) (No match)
155 - 174	2326.0413	2325.0340	2325.0452	-0.0112	0	NHYNFNPVIGMNMVCAGSLR 2 Oxidation (M) (No match)

178 - 195	1969.8120	1968.8047	1968.8305	-0.0258	0	DSCNGDSGSPLLCEGVFR	(No match)
178 - 195	1969.8120	1968.8047	1968.8305	-0.0258	0	DSCNGDSGSPLLCEGVFR	(Ions score 117)

Mascot: http://www.matrixscience.com/

Spot 721

Protein View

Match to: **gi|5803013** Score: **360** Expect: **1.9e-031**
endoplasmic reticulum protein 29 isoform 1 precursor [Homo sapiens]

Nominal mass (M_r): **29032**; Calculated pI value: **6.77**
NCBI BLAST search of gi|5803013 against nr
Unformatted sequence string for pasting into other applications

Taxonomy: Homo sapiens
Links to retrieve other entries containing this sequence from NCBI Entrez:
gi|6015110 from Homo sapiens
gi|3413293 from Homo sapiens
gi|49456295 from Homo sapiens
gi|75517097 from Homo sapiens
gi|75517652 from Homo sapiens
gi|119618396 from Homo sapiens
gi|119618397 from Homo sapiens

Fixed modifications: Carbamidomethyl (C)
Variable modifications: Oxidation (M)
Cleavage by Trypsin: cuts C-term side of KR unless next residue is P
Sequence Coverage: **42%**

Matched peptides shown in **Bold Red**

```
  1 MAAAVPRAAF LSPLLPLLLG FLLLSAPHGG SGLHTKGALP LDTVTFYKVI
 51 PKSKFVLVKF DTQYPYGEKQ DEFKRLAENS ASSDDLLVAE VGISDYGDKL
101 NMELSEKYKL DKESYPVFYL FRDGDFENPV PYTGAVKVGA IQRWLKGQGV
151 YLGMPGCLPV YDALAGEFIR ASGVEARQAL LKQGQDNLSS VKETQKKWAE
201 QYLKIMGKIL DQGEDFPASE MTRIARLIEK NKMSDGKKEE LQKSLNILTA
251 FQKKGAEKEE L
```

Residue Number Increasing Mass Decreasing Mass

Start - End	Observed	Mr(expt)	Mr(calc)	Delta	Miss	Sequence
37 - 48	1324.7212	1323.7139	1323.7074	0.0065	0	GALPLDTVTFYK (No match)
60 - 69	1247.5726	1246.5653	1246.5506	0.0148	0	FDTQYPYGEK (No match)
60 - 69	1247.5726	1246.5653	1246.5506	0.0148	0	FDTQYPYGEK (Ions score 60)

60 - 74	1894.8607	1893.8534	1893.8420	0.0114	1	FDTQYPYGEKQDEFK (No match)
70 - 75	822.4360	821.4287	821.4031	0.0256	1	QDEFKR (No match)
110 - 122	1676.8875	1675.8802	1675.8609	0.0193	1	LDKESYPVFYLFR (No match)
113 - 122	1320.6851	1319.6778	1319.6550	0.0229	0	ESYPVFYLFR (Ions score 64)
113 - 122	1320.6851	1319.6778	1319.6550	0.0229	0	ESYPVFYLFR (No match)
123 - 137	1608.7699	1607.7626	1607.7467	0.0159	0	DGDFENPVPYTGAVK (No match)
123 - 137	1608.7699	1607.7626	1607.7467	0.0159	0	DGDFENPVPYTGAVK (Ions score 79)
147 - 170	2583.2727	2582.2654	2582.2660	-0.0006	0	GQGVYLGMPGCLPVYDALAGEFIR (No match)
147 - 170	2599.3003	2598.2930	2598.2609	0.0321	0	GQGVYLGMPGCLPVYDALAGEFIR Oxidation (M) (No match)
198 - 204	937.4927	936.4854	936.4705	0.0150	0	WAEQYLK (No match)
209 - 223	1708.8059	1707.7986	1707.7774	0.0213	0	ILDQGEDFPASEMTR (No match)
209 - 223	1724.7979	1723.7906	1723.7723	0.0184	0	ILDQGEDFPASEMTR Oxidation (M) (Ions score 30)
209 - 223	1724.7979	1723.7906	1723.7723	0.0184	0	ILDQGEDFPASEMTR Oxidation (M) (No match)
244 - 253	1134.6671	1133.6598	1133.6444	0.0154	0	SLNILTAFQK (No match)

Mascot: http://www.matrixscience.com/

Spot 724

Protein View

Match to: **gi|33357774** Score: **587** Expect: **3.8e-054**
Chain A, The Oligomeric Structure Of Human Granzyme A Reveals The Molecular Determinants Of Substra

Nominal mass (M_r): **26328**; Calculated pI value: **9.13**
NCBI BLAST search of gi|33357774 against nr
Unformatted sequence string for pasting into other applications

Taxonomy: Homo sapiens

Fixed modifications: Carbamidomethyl (C)
Variable modifications: Oxidation (M)
Cleavage by Trypsin: cuts C-term side of KR unless next residue is P
Sequence Coverage: **69%**

Matched peptides shown in **Bold Red**

```
  1 IIGGNEVTPH SRPYMVLLSL DRKTICAGAL IAKDWVLTAA HCNLNKRSQV
 51 ILGAHSITRE EPTKQIMLVK KEFPYPCYDP ATREGDLKLL QLTEKAKINK
101 YVTILHLPKK GDDVKPGTMC QVAGWGRTHN SASWSDTLRE VEITIIDRKV
151 CNDRNHYNFN PVIGMNMVCA GSLRGGRDSC NGDSGSPLLC EGVFRGVTSF
201 GLENKCGDPR GPGVYILLSK KHLNWIIMTI KGAV
```

Residue Number Increasing Mass Decreasing Mass

Start - End	Observed	Mr(expt)	Mr(calc)	Delta	Miss	Sequence
1 - 22	2467.2947	2466.2874	2466.3052	-0.0178	0	IIGGNEVTPHSRPYMVLLSLDR (No match)
1 - 23	2595.3904	2594.3831	2594.4002	-0.0171	1	IIGGNEVTPHSRPYMVLLSLDRK (No match)
34 - 47	1697.8480	1696.8407	1696.8467	-0.0060	1	DWVLTAAHCNLNKR (No match)
48 - 59	1281.7230	1280.7157	1280.7200	-0.0043	0	SQVILGAHSITR (Ions score 42)
48 - 59	1281.7230	1280.7157	1280.7200	-0.0043	0	SQVILGAHSITR (No match)
71 - 83	1643.7417	1642.7344	1642.7449	-0.0105	1	KEFPYPCYDPATR (No match)
72 - 83	1515.6476	1514.6403	1514.6500	-0.0097	0	EFPYPCYDPATR (Ions score 82)
72 - 83	1515.6476	1514.6403	1514.6500	-0.0097	0	EFPYPCYDPATR (No match)
98 - 109	1438.8655	1437.8582	1437.8707	-0.0125	1	INKYVTILHLPK (No match)
101 - 109	1083.6439	1082.6366	1082.6488	-0.0121	0	YVTILHLPK (No match)
110 - 127	1961.9218	1960.9145	1960.9247	-0.0102	1	KGDDVKPGTMCQVAGWGR (No match)
110 - 127	1977.9189	1976.9116	1976.9196	-0.0080	1	KGDDVKPGTMCQVAGWGR Oxidation (M) (No match)

128 - 139	1374.6339	1373.6266	1373.6324	-0.0057	0	**THNSASWSDTLR**	(No match)
128 - 139	1374.6339	1373.6266	1373.6324	-0.0057	0	**THNSASWSDTLR**	(Ions score 76)
155 - 174	2294.0403	2293.0330	2293.0554	-0.0223	0	**NHYNFNPVIGMNMVCAGSLR**	(No match)
155 - 174	2294.0403	2293.0330	2293.0554	-0.0223	0	**NHYNFNPVIGMNMVCAGSLR**	(Ions score 88)
155 - 174	2310.0391	2309.0318	2309.0503	-0.0185	0	**NHYNFNPVIGMNMVCAGSLR**	Oxidation (M) (No match)
155 - 174	2326.0398	2325.0325	2325.0452	-0.0127	0	**NHYNFNPVIGMNMVCAGSLR**	2 Oxidation (M) (No match)
178 - 195	1969.8229	1968.8156	1968.8305	-0.0149	0	**DSCNGDSGSPLLCEGVFR**	(Ions score 101)
178 - 195	1969.8229	1968.8156	1968.8305	-0.0149	0	**DSCNGDSGSPLLCEGVFR**	(No match)
196 - 205	1051.5336	1050.5263	1050.5345	-0.0082	0	**GVTSFGLENK**	(No match)
211 - 220	1046.6121	1045.6048	1045.6171	-0.0123	0	**GPGVYILLSK**	(No match)
211 - 221	1174.7103	1173.7030	1173.7121	-0.0090	1	**GPGVYILLSKK**	(No match)

Mascot: http://www.matrixscience.com/

Spot 725

Protein View

Match to: **gi|5803013** Score: **279** Expect: **2.4e-023**
endoplasmic reticulum protein 29 isoform 1 precursor [Homo sapiens]

Nominal mass (M_r): **29032**; Calculated pI value: **6.77**
NCBI BLAST search of gi|5803013 against nr
Unformatted sequence string for pasting into other applications

Taxonomy: Homo sapiens
Links to retrieve other entries containing this sequence from NCBI Entrez:
gi|6015110 from Homo sapiens
gi|3413293 from Homo sapiens
gi|49456295 from Homo sapiens
gi|75517097 from Homo sapiens
gi|75517652 from Homo sapiens
gi|119618396 from Homo sapiens
gi|119618397 from Homo sapiens

Fixed modifications: Carbamidomethyl (C)
Variable modifications: Oxidation (M)
Cleavage by Trypsin: cuts C-term side of KR unless next residue is P
Sequence Coverage: **37%**

Matched peptides shown in **Bold Red**

```
  1 MAAAVPRAAF LSPLLPLLLG FLLLSAPHGG SGLHTKGALP LDTVTFYKVI
 51 PKSKFVLVKF DTQYPYGEKQ DEFKRLAENS ASSDDLLVAE VGISDYGDKL
101 NMELSEKYKL DKESYPVFYL FRDGDFENPV PYTGAVKVGA IQRWLKGQGV
151 YLGMPGCLPV YDALAGEFIR ASGVEARQAL LKQGQDNLSS VKETQKKWAE
201 QYLKIMGKIL DQGEDFPASE MTRIARLIEK NKMSDGKKEE LQKSLNILTA
251 FQKKGAEKEE L
```

Residue Number Increasing Mass Decreasing Mass

Start - End	Observed	Mr(expt)	Mr(calc)	Delta	Miss	Sequence
37 - 48	1324.7079	1323.7006	1323.7074	-0.0068	0	GALPLDTVTFYK (No match)
60 - 69	1247.5554	1246.5481	1246.5506	-0.0024	0	FDTQYPYGEK (No match)
110 - 122	1676.8666	1675.8593	1675.8609	-0.0016	1	LDKESYPVFYLFR (No match)

113 - 122	1320.6638	1319.6565	1319.6550	0.0016	0	ESYPVFYLFR	(Ions score 51)
113 - 122	1320.6638	1319.6565	1319.6550	0.0016	0	ESYPVFYLFR	(No match)
123 - 137	1608.7506	1607.7433	1607.7467	-0.0034	0	DGDFENPVPYTGAVK	(Ions score 35)
123 - 137	1608.7506	1607.7433	1607.7467	-0.0034	0	DGDFENPVPYTGAVK	(No match)
147 - 170	2599.2739	2598.2666	2598.2609	0.0057	0	GQGVYLGMPGCLPVYDALAGEFIR	Oxidation (M) (Ions score 64)
147 - 170	2599.2739	2598.2666	2598.2609	0.0057	0	GQGVYLGMPGCLPVYDALAGEFIR	Oxidation (M) (No match)
209 - 223	1724.7772	1723.7699	1723.7723	-0.0023	0	ILDQGEDFPASEMTR	Oxidation (M) (No match)
209 - 223	1724.7772	1723.7699	1723.7723	-0.0023	0	ILDQGEDFPASEMTR	Oxidation (M) (Ions score 13)
244 - 253	1134.6616	1133.6543	1133.6444	0.0099	0	SLNILTAFQK	(No match)

Mascot: http://www.matrixscience.com/

Spot 728

Protein View

Match to: **gi|56676393** Score: **346** Expect: **4.8e-030**
Rho GDP dissociation inhibitor (GDI) beta [Homo sapiens]

Nominal mass (M_r): **23031**; Calculated pI value: **5.10**
NCBI BLAST search of gi|56676393 against nr
Unformatted sequence string for pasting into other applications

Taxonomy: Homo sapiens
Links to retrieve other entries containing this sequence from NCBI Entrez:
gi|114643793 from Pan troglodytes
gi|1707893 from Homo sapiens
gi|20379030 from Homo sapiens
gi|404045 from Homo sapiens
gi|441455 from Homo sapiens
gi|14327952 from Homo sapiens
gi|119616742 from Homo sapiens
gi|119616743 from Homo sapiens
gi|119616744 from Homo sapiens
gi|119616746 from Homo sapiens
gi|119616747 from Homo sapiens
gi|119616748 from Homo sapiens
gi|123993255 from synthetic construct
gi|123999933 from synthetic construct

Fixed modifications: Carbamidomethyl (C)
Variable modifications: Oxidation (M)
Cleavage by Trypsin: cuts C-term side of KR unless next residue is P
Sequence Coverage: **46%**

Matched peptides shown in **Bold Red**

```
  1 MTEKAPEPHV EEDDDDELDS KLNYKPPPQK SLKELQEMDK DDESLIKYKK
 51 TLLGDGPVVT DPKAPNVVVT RLTLVCESAP GPITMDLTGD LEALKKETIV
101 LKEGSEYRVK IHFKVNRDIV SGLKYVQHTY RTGVKVDKAT FMVGSYGPRP
151 EEYEFLTPVE EAPKGMLARG TYHNKSFFTD DDKQDHLSWE WNLSIKKEWT
201 E
```

Residue Number Increasing Mass Decreasing Mass

Start - End	Observed	Mr(expt)	Mr(calc)	Delta	Miss	Sequence
5 - 21	1939.7786	1938.7713	1938.7966	-0.0253	0	APEPHVEEDDDDELDSK (No match)
5 - 21	1939.7786	1938.7713	1938.7966	-0.0253	0	APEPHVEEDDDDELDSK (Ions score 160)
22 - 30	1084.6071	1083.5998	1083.6076	-0.0078	0	LNYKPPPQK (No match)
34 - 47	1692.7775	1691.7702	1691.7923	-0.0221	1	ELQEMDKDDESLIK (No match)
34 - 47	1708.7755	1707.7682	1707.7872	-0.0190	1	ELQEMDKDDESLIK Oxidation (M) (No match)
34 - 47	1708.7755	1707.7682	1707.7872	-0.0190	1	ELQEMDKDDESLIK Oxidation (M) (Ions score 59)
51 - 63	1311.7076	1310.7003	1310.7081	-0.0078	0	TLLGDGPVVTDPK (No match)
64 - 71	855.5129	854.5056	854.4973	0.0083	0	APNVVVTR (No match)
125 - 131	966.4852	965.4779	965.4719	0.0060	0	YVQHTYR (No match)
139 - 164	2944.3379	2943.3306	2943.3999	-0.0693	0	ATFMVGSYGPRPEEYEFLTPVEEAPK (No match)
139 - 164	2944.3379	2943.3306	2943.3999	-0.0693	0	ATFMVGSYGPRPEEYEFLTPVEEAPK (Ions score 20)
139 - 164	2960.3735	2959.3662	2959.3949	-0.0286	0	ATFMVGSYGPRPEEYEFLTPVEEAPK Oxidation (M) (No match)
139 - 164	2960.3735	2959.3662	2959.3949	-0.0286	0	ATFMVGSYGPRPEEYEFLTPVEEAPK Oxidation (M) (Ions score 29)

Mascot: http://www.matrixscience.com/

Spot 729

Protein View

Match to: **gi|119595803** Score: **71** Expect: **0.015**
hCG41772, isoform CRA_a [Homo sapiens]

Nominal mass (M_r): **18896**; Calculated pI value: **8.89**
NCBI BLAST search of gi|119595803 against nr
Unformatted sequence string for pasting into other applications

Taxonomy: Homo sapiens

Fixed modifications: Carbamidomethyl (C)
Variable modifications: Oxidation (M)
Cleavage by Trypsin: cuts C-term side of KR unless next residue is P
Sequence Coverage: **19%**

Matched peptides shown in **Bold Red**

```
  1 MAFAGLTADA RIVINRARVE CQSHRLTVED PVTVEYITRY IASLKQRYTQ
 51 SNGRRPFGIS ALIVGFDFDG TPRLYQTDPS GTYHAWKANA IGRGAKSVRE
101 FLEKNYTDEA IETDDLTIKL VIKALLEVVQ SGGKNIELAV MRRDQSLKVI
151 TKFVCFLYLS VFSHDQG
```

Residue Number Increasing Mass Decreasing Mass

Start - End	Observed	Mr(expt)	Mr(calc)	Delta	Miss	Sequence
26 - 39	1634.8497	1633.8424	1633.8562	-0.0138	0	LTVEDPVTVEYITR (No match)
26 - 39	1634.8497	1633.8424	1633.8562	-0.0138	0	LTVEDPVTVEYITR (Ions score 21)
55 - 73	2065.0720	2064.0647	2064.0792	-0.0144	0	RPFGISALIVGFDFDGTPR (Ions score 25)
55 - 73	2065.0720	2064.0647	2064.0792	-0.0144	0	RPFGISALIVGFDFDGTPR (No match)

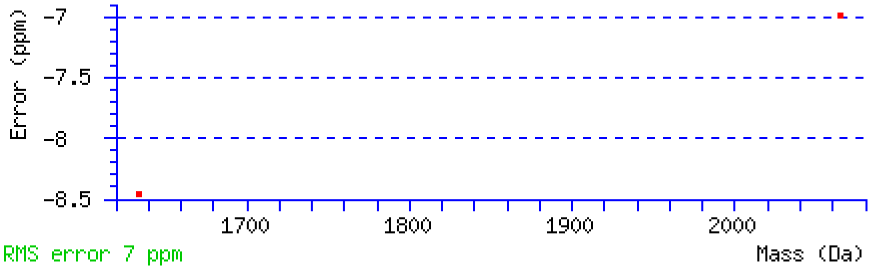

Mascot: http://www.matrixscience.com/

Spot 730

Protein View

Match to: **gi|49456447** Score: **393** Expect: **9.6e-035**
PGAM1 [Homo sapiens]

Nominal mass (M_r): **28931**; Calculated pI value: **6.67**
NCBI BLAST search of gi|49456447 against nr
Unformatted sequence string for pasting into other applications

Taxonomy: Homo sapiens

Fixed modifications: Carbamidomethyl (C)
Variable modifications: Oxidation (M)
Cleavage by Trypsin: cuts C-term side of KR unless next residue is P
Sequence Coverage: **48%**

Matched peptides shown in **Bold Red**

```
  1 MAAYKLVLIR HGESAWNLEN RFSGWYDADL SQAGHEEAKR GGQALRDAGY
 51 EFDICFTSVQ KRAIRTLWTV LDAIDQMWLP VVRTWRLNER HYGGLTGLNK
101 AETAAKHGEA QVKIWRRSYD VPPPPMEPDH PFYSNISKDR RYADLTEDQL
151 PSCESLKDTI ARALPFWNEE IVPQIKEGKR VLIAAHGNSL RGIVKHLEGL
201 SEEAIMELNL PTGIPIVYEL DKNLKPIKPM QFLGDEETVR KAMEAVAAQG
251 KAKK
```

Residue Number Increasing Mass Decreasing Mass

Start - End	Observed	Mr(expt)	Mr(calc)	Delta	Miss	Sequence
11 - 21	1312.5964	1311.5891	1311.5956	-0.0064	0	HGESAWNLENR (No match)
47 - 61	1779.7723	1778.7650	1778.7821	-0.0171	0	DAGYEFDICFTSVQK (No match)
47 - 62	1935.8712	1934.8639	1934.8832	-0.0193	1	DAGYEFDICFTSVQKR (No match)
47 - 62	1935.8712	1934.8639	1934.8832	-0.0193	1	DAGYEFDICFTSVQKR (Ions score 113)
91 - 100	1059.5525	1058.5452	1058.5508	-0.0056	0	HYGGLTGLNK (No match)
118 - 138	2433.1008	2432.0935	2432.0994	-0.0059	0	SYDVPPPPMEPDHPFYSNISK Oxidation (M) (No match)
142 - 162	2425.1321	2424.1248	2424.1478	-0.0230	1	YADLTEDQLPSCESLKDTIAR (No match)
142 - 162	2425.1321	2424.1248	2424.1478	-0.0230	1	YADLTEDQLPSCESLKDTIAR (Ions score 57)
163 - 176	1683.8933	1682.8860	1682.9031	-0.0171	0	ALPFWNEEIVPQIK (No match)
180 - 191	1306.7615	1305.7542	1305.7629	-0.0087	1	RVLIAAHGNSLR (No match)
181 - 191	1150.6639	1149.6566	1149.6618	-0.0052	0	VLIAAHGNSLR (Ions score 33)

181 - 191	1150.6639	1149.6566	1149.6618	-0.0052	0	**VLIAAHGNSLR**	(No match)
223 - 240	2131.1057	2130.0984	2130.1142	-0.0158	0	**NLKPIKPMQFLGDEETVR**	Oxidation (M) (No match)
223 - 240	2131.1057	2130.0984	2130.1142	-0.0158	0	**NLKPIKPMQFLGDEETVR**	Oxidation (M) (Ions score 32)
223 - 241	2259.2039	2258.1966	2258.2092	-0.0126	1	**NLKPIKPMQFLGDEETVRK**	Oxidation (M) (No match)

Mascot: http://www.matrixscience.com/

Spot 734

Protein View

Match to: **gi|8394076** Score: **206** Expect: **4.8e-016**
proteasome (prosome, macropain) subunit, alpha type 6 [Rattus norvegicus]

Nominal mass (M_r): **27838**; Calculated pI value: **6.34**
NCBI BLAST search of gi|8394076 against nr
Unformatted sequence string for pasting into other applications

Taxonomy: Rattus norvegicus
Links to retrieve other entries containing this sequence from NCBI Entrez:
gi|23110944 from Homo sapiens
gi|114053135 from Bos taurus
gi|55640605 from Pan troglodytes
gi|109083325 from Macaca mulatta
gi|109083327 from Macaca mulatta
gi|114652649 from Pan troglodytes
gi|149692835 from Equus caballus
gi|46397655 from Rattus norvegicus
gi|46397659 from Homo sapiens
gi|122138169 from Bos taurus
gi|21465642 from Bos taurus
gi|21465656 from Bos taurus
gi|35682 from Homo sapiens
gi|286250 from Rattus sp
gi|12804241 from Homo sapiens
gi|17389730 from Homo sapiens
gi|18490853 from Homo sapiens
gi|23273861 from Homo sapiens
gi|38328367 from Rattus norvegicus
gi|47125332 from Homo sapiens
gi|48146005 from Homo sapiens
gi|67969980 from Macaca fascicularis
gi|82571777 from Bos taurus
gi|119586281 from Homo sapiens
gi|123983062 from synthetic construct
gi|149051253 from Rattus norvegicus
gi|446773 from Homo sapiens

Fixed modifications: Carbamidomethyl (C)
Variable modifications: Oxidation (M)
Cleavage by Trypsin: cuts C-term side of KR unless next residue is P
Sequence Coverage: **24%**

Matched peptides shown in **Bold Red**

```
  1 MSRGSSAGFD RHITIFSPEG RLYQVEYAFK AINQGGLTSV AVRGKDCAVI
 51 VTQKKVPDKL LDSSTVTHLF KITENIGCVM TGMTADSRSQ VQRARYEAAN
101 WKYKYGYEIP VDMLCKRIAD ISQVYTQNAE MRPLGCCMIL IGIDEEQGPQ
151 VYKCDPAGYY CGFKATAAGV KQTESTSFLE KKVKKKFDWT FEQTVETAIT
201 CLSTVLSIDF KPSEIEVGVV TVENPKFRIL TEAEIDAHLV ALAERD
```

Residue Number Increasing Mass Decreasing Mass

Start - End	Observed	Mr(expt)	Mr(calc)	Delta	Miss	Sequence
12 - 21	1156.6066	1155.5993	1155.6036	-0.0043	0	HITIFSPEGR (Ions score 13)
12 - 21	1156.6066	1155.5993	1155.6036	-0.0043	0	HITIFSPEGR (No match)
22 - 30	1160.5883	1159.5810	1159.5913	-0.0103	0	LYQVEYAFK (No match)
31 - 43	1285.7172	1284.7099	1284.7149	-0.0050	0	AINQGGLTSVAVR (Ions score 10)
31 - 43	1285.7172	1284.7099	1284.7149	-0.0050	0	AINQGGLTSVAVR (No match)
154 - 164	1337.5142	1336.5069	1336.5216	-0.0147	0	CDPAGYYCGFK (No match)
229 - 246	1979.0198	1978.0125	1978.0370	-0.0245	1	ILTEAEIDAHLVALAERD (No match)
229 - 246	1979.0198	1978.0125	1978.0370	-0.0245	1	ILTEAEIDAHLVALAERD (Ions score 119)

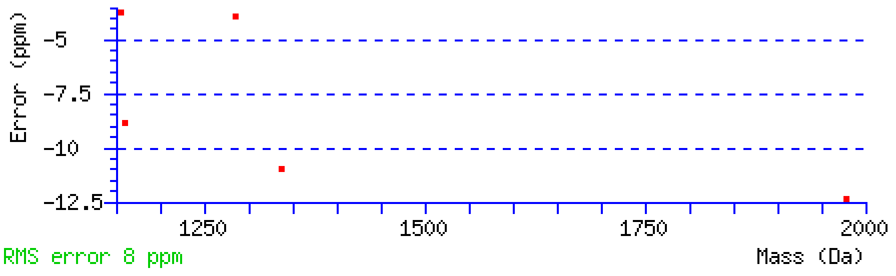

RMS error 8 ppm

Mascot: http://www.matrixscience.com/

Spot 736

Protein View

Match to: **gi|15277236** Score: **113** Expect: **9.6e-007**
TC4 [Homo sapiens]

Nominal mass (M_r): **12225;** Calculated pI value: **9.30**
NCBI BLAST search of gi|15277236 against nr
Unformatted sequence string for pasting into other applications

Taxonomy: Homo sapiens
Links to retrieve other entries containing this sequence from NCBI Entrez:
gi|27544381 from Homo sapiens

Fixed modifications: Carbamidomethyl (C)
Variable modifications: Oxidation (M)
Cleavage by Trypsin: cuts C-term side of KR unless next residue is P
Sequence Coverage: **17%**

Matched peptides shown in **Bold Red**

```
  1 MAAQGQPQVQ FKLVLVGDGG TGKTTFVKHH LTGEFEKKYV ATLGVEVHPL
 51 VFHTNRGPVK FNVWDTAGLE KFSGLRDGYY IQAQSTIIVF DVTSRVTYKN
101 VPNWHRDLV
```

Residue Number Increasing Mass Decreasing Mass

Start - End	Observed	Mr(expt)	Mr(calc)	Delta	Miss	Sequence	
38 - 56	2180.1890	2179.1817	2179.1901	-0.0084	1	KYVATLGVEVHPLVFHTNR	(No match)
38 - 56	2180.1890	2179.1817	2179.1901	-0.0084	1	KYVATLGVEVHPLVFHTNR	(Ions score 12)
39 - 56	2052.0964	2051.0891	2051.0952	-0.0060	0	YVATLGVEVHPLVFHTNR	(No match)
39 - 56	2052.0964	2051.0891	2051.0952	-0.0060	0	YVATLGVEVHPLVFHTNR	(Ions score 75)

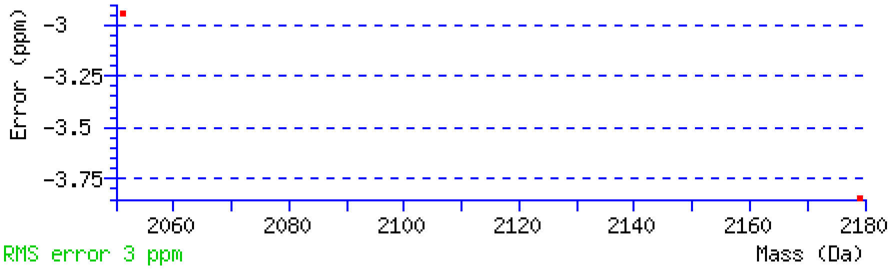

RMS error 3 ppm

Mascot: http://www.matrixscience.com/

Spot 737

Protein View

Match to: **gi|5453549** Score: **187** Expect: **3.8e-014**
thioredoxin peroxidase [Homo sapiens]

Nominal mass (M_r): **30749**; Calculated pI value: **5.86**
NCBI BLAST search of gi|5453549 against nr
Unformatted sequence string for pasting into other applications

Taxonomy: Homo sapiens
Links to retrieve other entries containing this sequence from NCBI Entrez:
gi|3024727 from Homo sapiens
gi|799381 from Homo sapiens
gi|13177646 from Homo sapiens
gi|13937985 from Homo sapiens
gi|16876997 from Homo sapiens
gi|49456371 from Homo sapiens
gi|119619402 from Homo sapiens
gi|123996703 from synthetic construct

Fixed modifications: Carbamidomethyl (C)
Variable modifications: Oxidation (M)
Cleavage by Trypsin: cuts C-term side of KR unless next residue is P
Sequence Coverage: **37%**

Matched peptides shown in **Bold Red**

```
  1 MEALPLLAAT TPDHGRHRRL LLLPLLLFLL PAGAVQGWET EERPRTREEE
 51 CHFYAGGQVY PGEASRVSVA DHSLHLSKAK ISKPAPYWEG TAVIDGEFKE
101 LKLTDYRGKY LVFFFYPLDF TFVCPTEIIA FGDRLEEFRS INTEVVACSV
151 DSQFTHLAWI NTPRRQGGLG PIRIPLLSDL THQISKDYGV YLEDSGHTLR
201 GLFIIDDKGI LRQITLNDLP VGRSVDETLR LVQAFQYTDK HGEVCPAGWK
251 PGSETIIPDP AGKLKYFDKL N
```

Residue Number Increasing Mass Decreasing Mass

Start - End	Observed	Mr(expt)	Mr(calc)	Delta	Miss	Sequence	
46 - 66	2443.0647	2442.0574	2442.0658	-0.0083	1	TREEECHFYAGGQVYPGEASR	(No match)
46 - 66	2443.0647	2442.0574	2442.0658	-0.0083	1	TREEECHFYAGGQVYPGEASR	(No match)

48 - 66	2185.9207	2184.9134	2184.9170	-0.0036	0	EEECHFYAGGQVYPGEASR (No match)
67 - 78	1292.6985	1291.6912	1291.6884	0.0028	0	VSVADHSLHLSK (No match)
165 - 173	953.5712	952.5639	952.5566	0.0073	1	RQGGLGPIR (No match)
174 - 186	1464.8364	1463.8291	1463.8347	-0.0056	0	IPLLSDLTHQISK (No match)
187 - 200	1624.7566	1623.7493	1623.7528	-0.0035	0	DYGVYLEDSGHTLR (No match)
187 - 200	1624.7566	1623.7493	1623.7528	-0.0035	0	DYGVYLEDSGHTLR (Ions score 12)
201 - 212	1359.7982	1358.7909	1358.7921	-0.0012	1	GLFIIDDKGILR (No match)
201 - 212	1359.7982	1358.7909	1358.7921	-0.0012	1	GLFIIDDKGILR (Ions score 27)
213 - 223	1225.6874	1224.6801	1224.6826	-0.0025	0	QITLNDLPVGR (No match)
213 - 223	1225.6874	1224.6801	1224.6826	-0.0025	0	QITLNDLPVGR (Ions score 21)
231 - 240	1212.6227	1211.6154	1211.6186	-0.0032	0	LVQAFQYTDK (No match)

Mascot: http://www.matrixscience.com/

Spot 738

Protein View

Match to: **gi|29708** Score: **124** Expect: **7.7e-008**
cathepsin H [Homo sapiens]

Nominal mass (M_r): **27844;** Calculated pI value: **6.99**
NCBI BLAST search of gi|29708 against nr
Unformatted sequence string for pasting into other applications

Taxonomy: Homo sapiens

Fixed modifications: Carbamidomethyl (C)
Variable modifications: Oxidation (M)
Cleavage by Trypsin: cuts C-term side of KR unless next residue is P
Sequence Coverage: **12%**

Matched peptides shown in **Bold Red**

```
  1 AEIKHKYLWS EPQNCSATKS NYLRGTGPYP PSVDWRKKGN FVSPVKNQGA
 51 CGSCWTFSTT GALESAIAIA TGKMLSLAEQ QLVDCAQDFN NYGCQGGLPS
101 QAFEYILYNK GIMGEDTYPY QGKDGYCKFQ PGKAIGFVKD VANITIYDEE
151 AMVEAVALYN PVSFAFEVTQ DFMMYRTGIY SSTSCHKTPD KVNHAVLAVG
201 YGEKNGIPYW IVKNSWGPQW GMNGYFLIER GKNMCGLAAC ASYPIPLV
```

Residue Number Increasing Mass Decreasing Mass

Start - End	Observed	Mr(expt)	Mr(calc)	Delta	Miss	Sequence
111 - 123	1474.6307	1473.6234	1473.6445	-0.0211	0	GIMGEDTYPYQGK Oxidation (M) (No match)
111 - 123	1474.6307	1473.6234	1473.6445	-0.0211	0	GIMGEDTYPYQGK Oxidation (M) (Ions score 19)
188 - 204	1797.9240	1796.9167	1796.9420	-0.0253	1	TPDKVNHAVLAVGYGEK (No match)
188 - 204	1797.9240	1796.9167	1796.9420	-0.0253	1	TPDKVNHAVLAVGYGEK (Ions score 66)

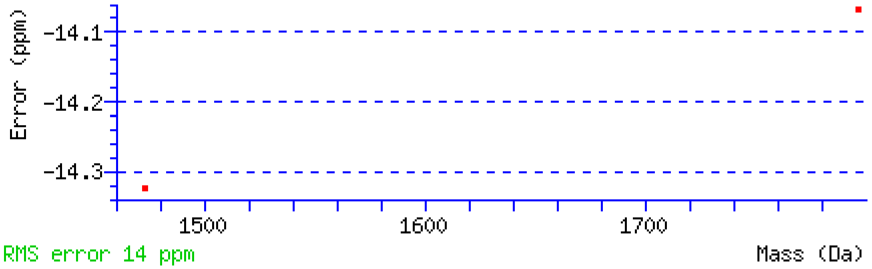

Mascot: http://www.matrixscience.com/

Spot 739

Protein View

Match to: **gi|4758638** Score: **379** Expect: **2.4e-033**
peroxiredoxin 6 [Homo sapiens]

Nominal mass (M_r): **25133;** Calculated pI value: **6.00**
NCBI BLAST search of gi|4758638 against nr
Unformatted sequence string for pasting into other applications

Taxonomy: Homo sapiens
Links to retrieve other entries containing this sequence from NCBI Entrez:
gi|114565483 from Pan troglodytes
gi|114621471 from Pan troglodytes
gi|1718024 from Homo sapiens
gi|285949 from Homo sapiens
gi|23274223 from Homo sapiens
gi|31657160 from Homo sapiens
gi|56204402 from Homo sapiens
gi|77744395 from Homo sapiens
gi|119611350 from Homo sapiens
gi|119611351 from Homo sapiens
gi|158259727 (no taxonomy information for this entry)

Fixed modifications: Carbamidomethyl (C)
Variable modifications: Oxidation (M)
Cleavage by Trypsin: cuts C-term side of KR unless next residue is P
Sequence Coverage: **51%**

Matched peptides shown in **Bold Red**

```
  1 MPGGLLLGDV APNFEANTTV GRIRFHDFLG DSWGILFSHP RDFTPVCTTE
 51 LGRAAKLAPE FAKRNVKLIA LSIDSVEDHL AWSKDINAYN CEEPTEKLPF
101 PIIDDRNREL AILLGMLDPA EKDEKGMPVT ARVVFVFGPD KKLKLSILYP
151 ATTGRNFDEI LRVVISLQLT AEKRVATPVD WKDGDSVMVL PTIPEEEAKK
201 LFPKGVFTKE LPSGKKYLRY TPQP
```

Residue Number Increasing Mass Decreasing Mass

Start - End Observed Mr(expt) Mr(calc) Delta Miss Sequence

2 - 22	2098.0688	2097.0615	2097.0854	-0.0238	0	PGGLLLGDVAPNFEANTTVGR (Ions score 85)
2 - 22	2098.0688	2097.0615	2097.0854	-0.0238	0	PGGLLLGDVAPNFEANTTVGR (No match)
42 - 53	1395.6436	1394.6363	1394.6500	-0.0137	0	DFTPVCTTELGR (Ions score 59)
42 - 53	1395.6436	1394.6363	1394.6500	-0.0137	0	DFTPVCTTELGR (No match)
85 - 97	1582.6495	1581.6422	1581.6617	-0.0194	0	DINAYNCEEPTEK (No match)
85 - 106	2649.1877	2648.1804	2648.2428	-0.0623	1	DINAYNCEEPTEKLPFPIIDDR (No match)
98 - 106	1085.5895	1084.5822	1084.5917	-0.0094	0	LPFPIIDDR (No match)
98 - 106	1085.5895	1084.5822	1084.5917	-0.0094	0	LPFPIIDDR (Ions score 41)
98 - 108	1355.7292	1354.7219	1354.7357	-0.0138	1	LPFPIIDDRNR (No match)
109 - 125	1900.9869	1899.9796	1899.9863	-0.0066	1	ELAILLGMLDPAEKDEK Oxidation (M) (No match)
133 - 141	1007.5511	1006.5438	1006.5487	-0.0049	0	VVFVFGPDK (No match)
133 - 142	1135.6404	1134.6331	1134.6436	-0.0105	1	VVFVFGPDKK (No match)
143 - 155	1432.8417	1431.8344	1431.8449	-0.0105	1	LKLSILYPATTGR (No match)
145 - 155	1191.6650	1190.6577	1190.6659	-0.0082	0	LSILYPATTGR (Ions score 28)
145 - 155	1191.6650	1190.6577	1190.6659	-0.0082	0	LSILYPATTGR (No match)
156 - 162	906.4610	905.4537	905.4606	-0.0069	0	NFDEILR (No match)
163 - 174	1356.8016	1355.7943	1355.8136	-0.0193	1	VVISLQLTAEKR (No match)

Mascot: http://www.matrixscience.com/

Spot 740

Protein View

Match to: **gi|136066** Score: **118** Expect: **3e-007**
Triosephosphate isomerase (TIM) (Triose-phosphate isomerase)

Nominal mass (M_r): **26894**; Calculated pI value: **7.10**
NCBI BLAST search of gi|136066 against nr
Unformatted sequence string for pasting into other applications

Taxonomy: Oryctolagus cuniculus
Links to retrieve other entries containing this sequence from NCBI Entrez:
gi|40889552 from Oryctolagus cuniculus
gi|40889553 from Oryctolagus cuniculus
gi|40889554 from Oryctolagus cuniculus
gi|40889555 from Oryctolagus cuniculus
gi|40889556 from Oryctolagus cuniculus
gi|40889557 from Oryctolagus cuniculus
gi|40889558 from Oryctolagus cuniculus
gi|40889559 from Oryctolagus cuniculus
gi|40889560 from Oryctolagus cuniculus
gi|40889561 from Oryctolagus cuniculus
gi|223374 from Homo sapiens

Fixed modifications: Carbamidomethyl (C)
Variable modifications: Oxidation (M)
Cleavage by Trypsin: cuts C-term side of KR unless next residue is P
Sequence Coverage: **13%**

Matched peptides shown in **Bold Red**

```
  1 APSRKFFVGG NWKMNGRKKN LGELITTLNA AKVPADTEVV CAPPTAYIDF
 51 ARQKLDPKIA VAAQNCYKVT NGAFTGEISP GMIKDCGATW VVLGHSERRH
101 VFGESDELIG QKVAHALSEG LGVIACIGEK LDEREAGITE KVVFEQTKVI
151 ADNVKDWSKV VLAYEPVWAI GTGKTATPQQ AQEVHEKLRG WLKSNVSDAV
201 AQSTRIIYGG SVTGATCKEL ASQPDVDGFL VGGASLKPEF VDIINAKQ
```

Residue Number Increasing Mass Decreasing Mass

Start - End	Observed	Mr(expt)	Mr(calc)	Delta	Miss	Sequence

33 - 52	2192.0044	2190.9971	2191.0619	-0.0648	0	VPADTEVVCAPPTAYIDFAR	(No match)
33 - 52	2192.0044	2190.9971	2191.0619	-0.0648	0	VPADTEVVCAPPTAYIDFAR	(Ions score 86)
99 - 112	1614.7825	1613.7752	1613.8161	-0.0409	1	RHVFGESDELIGQK	(No match)
99 - 112	1614.7825	1613.7752	1613.8161	-0.0409	1	RHVFGESDELIGQK	(No match)

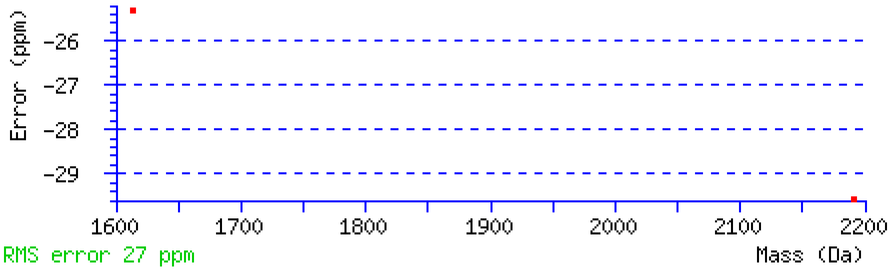

RMS error 27 ppm

Mascot: http://www.matrixscience.com/

Spot 741

Protein View

Match to: **gi|2465729** Score: **122** Expect: **1.2e-007**
TFAR15 [Homo sapiens]

Nominal mass (M_r): **24642**; Calculated pI value: **8.58**
NCBI BLAST search of gi|2465729 against nr
Unformatted sequence string for pasting into other applications

Taxonomy: Homo sapiens

Fixed modifications: Carbamidomethyl (C)
Variable modifications: Oxidation (M)
Cleavage by Trypsin: cuts C-term side of KR unless next residue is P
Sequence Coverage: **28%**

Matched peptides shown in **Bold Red**

```
  1 MRMTMEEMKN EAETTSMVSM PLYAVMYPVF NELERVNLSA AQTLRAAFIK
 51 AEKENPGLTQ DIIMKILEKK SVEVNFTESL LRMAADDVEE YMIERPEPEF
101 QALNEKARAL KQILSKIPDE INDRVRFLQT IKDIASAIKE LLDTVNNVFK
151 KYQYQNRRAL EHQKKEFVKY SKSFSDTLKT YFKDGKAINV FVSANRLIHQ
201 TNLILQTFKT VA
```

Residue Number Increasing Mass Decreasing Mass

Start - End	Observed	Mr(expt)	Mr(calc)	Delta	Miss	Sequence
36 - 45	1072.6132	1071.6059	1071.6036	0.0023	0	VNLSAAQTLR (No match)
36 - 45	1072.6132	1071.6059	1071.6036	0.0023	0	VNLSAAQTLR (No match)
70 - 82	1521.8284	1520.8211	1520.8198	0.0013	1	KSVEVNFTESLLR (No match)
71 - 82	1393.7339	1392.7266	1392.7248	0.0018	0	SVEVNFTESLLR (No match)
117 - 124	971.4828	970.4755	970.4719	0.0036	0	IPDEINDR (No match)
152 - 157	871.4053	870.3980	870.3984	-0.0004	0	YQYQNR (No match)
187 - 196	1090.6055	1089.5982	1089.5930	0.0052	0	AINVFVSANR (No match)
187 - 196	1090.6055	1089.5982	1089.5930	0.0052	0	AINVFVSANR (Ions score 3)
197 - 209	1568.9166	1567.9093	1567.9086	0.0008	0	LIHQTNLILQTFK (No match)
197 - 209	1568.9166	1567.9093	1567.9086	0.0008	0	LIHQTNLILQTFK (Ions score 47)

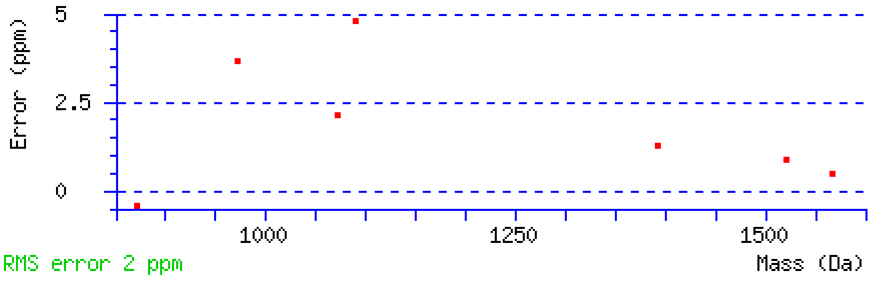

Mascot: http://www.matrixscience.com/

Spot 742

Mascot Search Results

Protein View

Match to: **gi|4507645** Score: **542** Expect: **1.2e-049**
triosephosphate isomerase 1 [Homo sapiens]

Nominal mass (M_r): **26938**; Calculated pI value: **6.45**
NCBI BLAST search of gi|4507645 against nr
Unformatted sequence string for pasting into other applications

Taxonomy: Homo sapiens
Links to retrieve other entries containing this sequence from NCBI Entrez:
gi|115392091 from Pan troglodytes
gi|39932641 from Homo sapiens
gi|39932642 from Pan troglodytes
gi|37247 from Homo sapiens
gi|176960 from Pan troglodytes
gi|339841 from Homo sapiens
gi|1200507 from Homo sapiens
gi|13937950 from Homo sapiens
gi|14043688 from Homo sapiens
gi|14424603 from Homo sapiens
gi|15079577 from Homo sapiens
gi|15929332 from Homo sapiens
gi|49456365 from Homo sapiens
gi|123993147 from synthetic construct
gi|124000137 from synthetic construct

Fixed modifications: Carbamidomethyl (C)
Variable modifications: Oxidation (M)
Cleavage by Trypsin: cuts C-term side of KR unless next residue is P
Sequence Coverage: **68%**

Matched peptides shown in **Bold Red**

```
  1 MAPSRKFFVG GNWKMNGRKQ SLGELIGTLN AAKVPADTEV VCAPPTAYID
 51 FARQKLDPKI AVAAQNCYKV TNGAFTGEIS PGMIKDCGAT WVVLGHSERR
101 HVFGESDELI GQKVAHALAE GLGVIACIGE KLDEREAGIT EKVVFEQTKV
151 IADNVKDWSK VVLAYEPVWA IGTGKTATPQ QAQEVHEKLR GWLKSNVSDA
201 VAQSTRIIYG GSVTGATCKE LASQPDVDGF LVGGASLKPE FVDIINAKQ
```

Residue Number Increasing Mass Decreasing Mass

Start - End	Observed	Mr(expt)	Mr(calc)	Delta	Miss	Sequence
7 - 14	954.4796	953.4723	953.4759	-0.0035	0	FFVGGNWK (No match)
34 - 53	2192.0559	2191.0486	2191.0619	-0.0133	0	VPADTEVVCAPPTAYIDFAR (Ions score 125)
34 - 53	2192.0559	2191.0486	2191.0619	-0.0133	0	VPADTEVVCAPPTAYIDFAR (No match)
56 - 69	1590.7286	1589.7213	1589.8235	-0.1022	1	LDPKIAVAAQNCYK (No match)
60 - 69	1137.5653	1136.5580	1136.5648	-0.0067	0	IAVAAQNCYK (No match)
70 - 85	1637.8108	1636.8035	1636.8130	-0.0094	0	VTNGAFTGEISPGMIK Oxidation (M) (No match)
86 - 99	1586.7343	1585.7270	1585.7307	-0.0036	0	DCGATWVVLGHSER (No match)
100 - 113	1614.8170	1613.8097	1613.8161	-0.0064	1	RHVFGESDELIGQK (No match)
100 - 113	1614.8170	1613.8097	1613.8161	-0.0064	1	RHVFGESDELIGQK (No match)
101 - 113	1458.7137	1457.7064	1457.7150	-0.0086	0	HVFGESDELIGQK (No match)
101 - 113	1458.7137	1457.7064	1457.7150	-0.0086	0	HVFGESDELIGQK (Ions score 64)
114 - 131	1807.9652	1806.9579	1806.9661	-0.0082	0	VAHALAEGLGVIACIGEK (No match)
114 - 131	1807.9652	1806.9579	1806.9661	-0.0082	0	VAHALAEGLGVIACIGEK (Ions score 143)
176 - 188	1466.7156	1465.7083	1465.7161	-0.0078	0	TATPQQAQEVHEK (No match)
195 - 206	1234.6036	1233.5963	1233.5949	0.0014	0	SNVSDAVAQSTR (No match)
207 - 219	1326.6638	1325.6565	1325.6649	-0.0083	0	IIYGGSVTGATCK (No match)
220 - 248	3029.6008	3028.5935	3028.5756	0.0179	0	ELASQPDVDGFLVGGASLKPEFVDIINAK (No match)

Mascot: http://www.matrixscience.com/

Spot 743

Mascot Search Results

Protein View

Match to: **gi|4507645** Score: **98** Expect: **3.3e-005**
triosephosphate isomerase 1 [Homo sapiens]

Nominal mass (M_r): **26938**; Calculated pI value: **6.45**
NCBI BLAST search of gi|4507645 against nr
Unformatted sequence string for pasting into other applications

Taxonomy: Homo sapiens
Links to retrieve other entries containing this sequence from NCBI Entrez:
gi|115392091 from Pan troglodytes
gi|39932641 from Homo sapiens
gi|39932642 from Pan troglodytes
gi|37247 from Homo sapiens
gi|176960 from Pan troglodytes
gi|339841 from Homo sapiens
gi|1200507 from Homo sapiens
gi|13937950 from Homo sapiens
gi|14043688 from Homo sapiens
gi|14424603 from Homo sapiens
gi|15079577 from Homo sapiens
gi|15929332 from Homo sapiens
gi|49456365 from Homo sapiens
gi|123993147 from synthetic construct
gi|124000137 from synthetic construct

Fixed modifications: Carbamidomethyl (C)
Variable modifications: Oxidation (M)
Cleavage by Trypsin: cuts C-term side of KR unless next residue is P
Sequence Coverage: **13%**

Matched peptides shown in **Bold Red**

1 MAPSRKFFVG GNWKMNGRKQ SLGELIGTLN AAK**VPADTEV VCAPPTAYID**
51 **FAR**QKLDPKI AVAAQNCYKV TNGAFTGEIS PGMIKDCGAT WVVLGHSERR
101 **HVFGESDELI GQK**VAHALAE GLGVIACIGE KLDEREAGIT EKVVFEQTKV
151 IADNVKDWSK VVLAYEPVWA IGTGKTATPQ QAQEVHEKLR GWLKSNVSDA
201 VAQSTRIIYG GSVTGATCKE LASQPDVDGF LVGGASLKPE FVDIINAKQ

Residue Number Increasing Mass Decreasing Mass

Start - End	Observed	Mr(expt)	Mr(calc)	Delta	Miss	Sequence
34 - 53	2192.0583	2191.0510	2191.0619	-0.0109	0	VPADTEVVCAPPTAYIDFAR (No match)
34 - 53	2192.0583	2191.0510	2191.0619	-0.0109	0	VPADTEVVCAPPTAYIDFAR (Ions score 48)
101 - 113	1458.7150	1457.7077	1457.7150	-0.0073	0	HVFGESDELIGQK (Ions score 23)
101 - 113	1458.7150	1457.7077	1457.7150	-0.0073	0	HVFGESDELIGQK (No match)

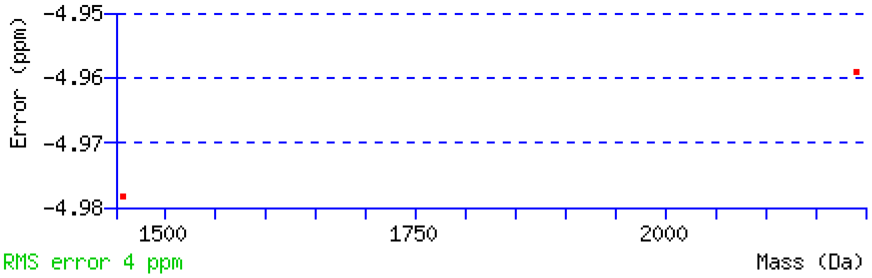

Mascot: http://www.matrixscience.com/

Spot 747

Mascot Search Results

Protein View

Match to: **gi|74760039** Score: **153** Expect: **9.6e-011**
Leucine-rich repeat-containing protein 57

Nominal mass (M_r): **27079**; Calculated pI value: **8.70**
NCBI BLAST search of gi|74760039 against nr
Unformatted sequence string for pasting into other applications

Taxonomy: Homo sapiens
Links to retrieve other entries containing this sequence from NCBI Entrez:
gi|21753132 from Homo sapiens
gi|37590646 from Homo sapiens
gi|51476178 from Homo sapiens
gi|119612968 from Homo sapiens
gi|119612969 from Homo sapiens
gi|119612970 from Homo sapiens

Fixed modifications: Carbamidomethyl (C)
Variable modifications: Oxidation (M)
Cleavage by Trypsin: cuts C-term side of KR unless next residue is P
Sequence Coverage: **19%**

Matched peptides shown in **Bold Red**

```
  1 MGNSALRAHV ETAQKTGVFQ LKDRGLTEFP ADLQKLTSNL RTIDLSNNKI
 51 ESLPPLLIGK FTLLKSLSLN NNKLTVLPDE ICNLKKLETL SLNNNHLREL
101 PSTFGQLSAL KTLSLSGNQL GALPPQLCSL RHLDVMDLSK NQIRSIPDSV
151 GELQVIELNL NQNQISQISV KISCCPRLKI LRLEENCLEL SMLPQSILSD
201 SQICLLAVEG NLFEIKKLRE LEGYDKYMER FTATKKKFA
```

Residue Number Increasing Mass Decreasing Mass

Start - End	Observed	Mr(expt)	Mr(calc)	Delta	Miss	Sequence	
86 - 98	1551.8467	1550.8394	1550.8528	-0.0134	1	KLETLSLNNNHLR	(No match)
86 - 98	1551.8467	1550.8394	1550.8528	-0.0134	1	KLETLSLNNNHLR	(No match)
87 - 98	1423.7563	1422.7490	1422.7579	-0.0089	0	LETLSLNNNHLR	(No match)

87 - 98	1423.7563	1422.7490	1422.7579	-0.0089	0	**LETLSLNNNHLR**	(No match)
99 - 111	1390.7428	1389.7355	1389.7503	-0.0148	0	**ELPSTFGQLSALK**	(Ions score 44)
99 - 111	1390.7428	1389.7355	1389.7503	-0.0148	0	**ELPSTFGQLSALK**	(No match)
112 - 131	2125.1118	2124.1045	2124.1361	-0.0315	0	**TLSLSGNQLGALPPQLCSLR**	(No match)
112 - 131	2125.1118	2124.1045	2124.1361	-0.0315	0	**TLSLSGNQLGALPPQLCSLR**	(Ions score 49)

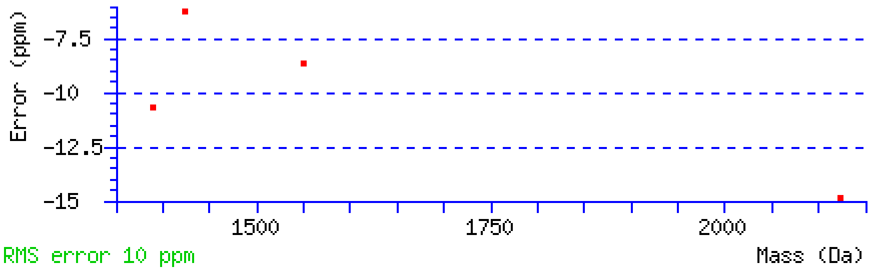

RMS error 10 ppm

Mascot: http://www.matrixscience.com/

Spot 749

Mascot Search Results

Protein View

Match to: **gi|30749675** Score: **272** Expect: **1.2e-022**
Chain A, Crystal Structures Of Cathepsin S Inhibitor Complexes

Nominal mass (M_r): **24491**; Calculated pI value: **7.64**
NCBI BLAST search of gi|30749675 against nr
Unformatted sequence string for pasting into other applications

Taxonomy: Homo sapiens
Links to retrieve other entries containing this sequence from NCBI Entrez:
gi|30749676 from Homo sapiens
gi|30749688 from Homo sapiens

Fixed modifications: Carbamidomethyl (C)
Variable modifications: Oxidation (M)
Cleavage by Trypsin: cuts C-term side of KR unless next residue is P
Sequence Coverage: **31%**

Matched peptides shown in **Bold Red**

```
  1 LPDSVDWREK GCVTEVKYQG SCGACWAFSA VGALEAQLKL KTGKLVTLSA
 51 QNLVDCSTEK YGNKGCNGGF MTTAFQYIID NKGIDSDASY PYKAMDQKCQ
101 YDSKYRAATC SKYTELPYGR EDVLKEAVAN KGPVSVGVDA RHPSFFLYRS
151 GVYYEPSCTQ NVNHGVLVVG YGDLNGKEYW LVKNSWGHNF GEEGYIRMAR
201 NKGNHCGIAS FPSYPEI
```

Residue Number Increasing Mass Decreasing Mass

Start - End	Observed	Mr(expt)	Mr(calc)	Delta	Miss	Sequence
113 - 120	998.4920	997.4847	997.4868	-0.0021	0	YTELPYGR (No match)
113 - 120	998.4920	997.4847	997.4868	-0.0021	0	YTELPYGR (Ions score 7)
113 - 125	1582.7949	1581.7876	1581.8038	-0.0162	1	YTELPYGREDVLK (Ions score 40)
113 - 125	1582.7949	1581.7876	1581.8038	-0.0162	1	YTELPYGREDVLK (No match)
126 - 141	1568.8214	1567.8141	1567.8317	-0.0176	1	EAVANKGPVSVGVDAR (No match)
142 - 149	1066.5410	1065.5337	1065.5396	-0.0058	0	HPSFFLYR (Ions score 55)
142 - 149	1066.5410	1065.5337	1065.5396	-0.0058	0	HPSFFLYR (No match)

184 - 197	1665.7231	1664.7158	1664.7331	-0.0173	0	NSWGHNFGEEGYIR	(No match)
184 - 197	1665.7231	1664.7158	1664.7331	-0.0173	0	NSWGHNFGEEGYIR	(Ions score 83)
201 - 217	1890.8582	1889.8509	1889.8730	-0.0220	1	NKGNHCGIASFPSYPEI	(No match)

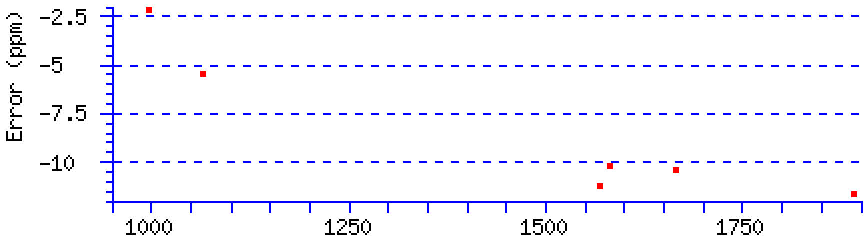

RMS error 8 ppm

Mascot: http://www.matrixscience.com/

Spot 750

Mascot Search Results

Protein View

Match to: **gi|119567805** Score: **169** Expect: **2.4e-012**
proteasome (prosome, macropain) subunit, beta type, 1, isoform CRA_a [Homo sapiens]

Nominal mass (M_r): **24634**; Calculated pI value: **8.69**
NCBI BLAST search of gi|119567805 against nr
Unformatted sequence string for pasting into other applications

Taxonomy: Homo sapiens

Fixed modifications: Carbamidomethyl (C)
Variable modifications: Oxidation (M)
Cleavage by Trypsin: cuts C-term side of KR unless next residue is P
Sequence Coverage: **34%**

Matched peptides shown in **Bold Red**

```
  1 MRCVSLFRLA LLKFTCTILA IAGEDFAIVA SDTRLSEGFS IHTRDSPKCY
 51 KLTDKTVIGC SGFHGDCLTL TKIIEARLKM YKHSNNKAMT TGAIAAMLST
101 ILYSRRFFPY YVYNIIGGLD EEGKGAVYSF DPVGSYQRDS FKAGGSASAM
151 LQPLLDNQVG FKNMQNVEHV PLSLDRAMRL VKDVFISAAE RDVYTGDALR
201 ICIVTKEGIR EETVSLRKD
```

Residue Number Increasing Mass Decreasing Mass

Start - End	Observed	Mr(expt)	Mr(calc)	Delta	Miss	Sequence
35 - 44	1146.5654	1145.5581	1145.5829	-0.0247	0	LSEGFSIHTR (No match)
35 - 44	1146.5654	1145.5581	1145.5829	-0.0247	0	LSEGFSIHTR (Ions score 16)
88 - 105	1902.9083	1901.9010	1901.9590	-0.0580	0	AMTTGAIAAMLSTILYSR 2 Oxidation (M) (No match)
88 - 105	1902.9083	1901.9010	1901.9590	-0.0580	0	AMTTGAIAAMLSTILYSR 2 Oxidation (M) (Ions score 11)
106 - 124	2280.0571	2279.0498	2279.1261	-0.0763	1	RFFPYYVYNIIGGLDEEGK (Ions score 24)
106 - 124	2280.0571	2279.0498	2279.1261	-0.0763	1	RFFPYYVYNIIGGLDEEGK (No match)
125 - 138	1545.6945	1544.6872	1544.7259	-0.0387	0	GAVYSFDPVGSYQR (Ions score 26)
125 - 138	1545.6945	1544.6872	1544.7259	-0.0387	0	GAVYSFDPVGSYQR (No match)
163 - 176	1667.7617	1666.7544	1666.8096	-0.0552	0	NMQNVEHVPLSLDR Oxidation (M) (Ions score 9)
163 - 176	1667.7617	1666.7544	1666.8096	-0.0552	0	NMQNVEHVPLSLDR Oxidation (M) (No match)

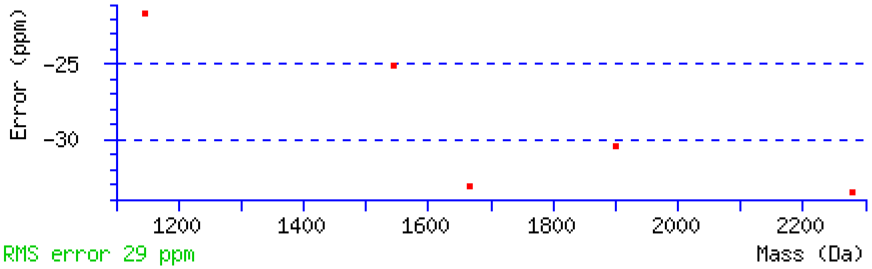

Mascot: http://www.matrixscience.com/

Spot 754

Mascot Search Results

Protein View

Match to: **gi|62897513** Score: **254** Expect: **7.7e-021**
proteasome alpha 2 subunit variant [Homo sapiens]

Nominal mass (M_r): **25938**; Calculated pI value: **7.71**
NCBI BLAST search of gi|62897513 against nr
Unformatted sequence string for pasting into other applications

Taxonomy: Homo sapiens

Fixed modifications: Carbamidomethyl (C)
Variable modifications: Oxidation (M)
Cleavage by Trypsin: cuts C-term side of KR unless next residue is P
Sequence Coverage: **23%**

Matched peptides shown in **Bold Red**

```
  1 MAERGYSFSL TTFSPSGKLV QIEYALAAVA GGAPSVGIKA ANGVVLATEK
 51 KQKSILYDER SVHKVEPITK HIGLVYSGMG PDYRVLVHRA RKLAQQYYLV
101 YQEPIPTAQL VQRVASVMQE YTQSGGVRPF GVSLLICGWN EGRPYLFQSD
151 PSGAYFAWKA TAMGKNYVNG KTFLEKRYNE DLELEDAIHT AILTLKESFE
201 GQMTEDNIEV GICNEAGFRR LTPTEVKGYL AAIA
```

Residue Number Increasing Mass Decreasing Mass

Start - End	Observed	Mr(expt)	Mr(calc)	Delta	Miss	Sequence
5 - 18	1478.6935	1477.6862	1477.7088	-0.0226	0	GYSFSLTTFSPSGK (No match)
5 - 18	1478.6935	1477.6862	1477.7088	-0.0226	0	GYSFSLTTFSPSGK (Ions score 91)
54 - 60	895.4459	894.4386	894.4447	-0.0060	0	SILYDER (No match)
71 - 84	1580.7280	1579.7207	1579.7452	-0.0245	0	HIGLVYSGMGPDYR Oxidation (M) (No match)
71 - 84	1580.7280	1579.7207	1579.7452	-0.0245	0	HIGLVYSGMGPDYR Oxidation (M) (Ions score 23)
93 - 113	2521.2942	2520.2869	2520.3376	-0.0507	0	LAQQYYLVYQEPIPTAQLVQR (Ions score 87)
93 - 113	2521.2942	2520.2869	2520.3376	-0.0507	0	LAQQYYLVYQEPIPTAQLVQR (No match)

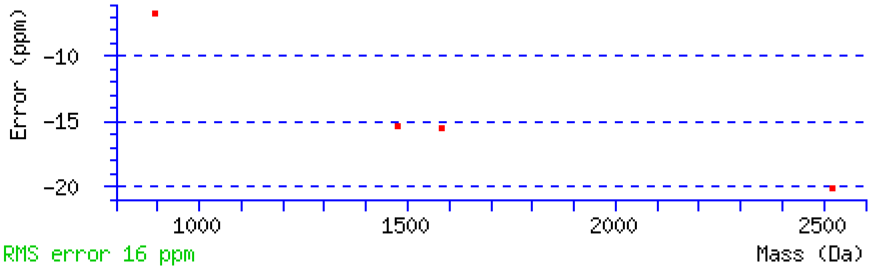

Mascot: http://www.matrixscience.com/

Spot 755

Mascot Search Results

Protein View

Match to: **gi|5107682** Score: **220** Expect: **1.9e-017**
Chain A, Structure Of The Ran-Gppnhp-Ranbd1 Complex

Nominal mass (M_r): **23307**; Calculated pI value: **9.02**
NCBI BLAST search of gi|5107682 against nr
Unformatted sequence string for pasting into other applications

Taxonomy: Homo sapiens
Links to retrieve other entries containing this sequence from NCBI Entrez:
gi|5107684 from Homo sapiens

Fixed modifications: Carbamidomethyl (C)
Variable modifications: Oxidation (M)
Cleavage by Trypsin: cuts C-term side of KR unless next residue is P
Sequence Coverage: **33%**

Matched peptides shown in **Bold Red**

```
  1 QVQFKLVLVG DGGTGKTTFV KRHLTGEFEK KYVATLGVEV HPLVFHTNRG
 51 PIKFNVWDTA GQEKFGGLRD GYYIQAQCAI IMFDVTSRVT YKNVPNWHRD
101 LVRVCENIPI VLCGNKVDIK DRKVKAKSIV FHRKKNLQYY DISAKSNYNF
151 EKPFLWLARK LIGDPNLEFV AMPALAPPEV VMDPALAAQY EHDLEVAQTT
201 ALPD
```

Residue Number Increasing Mass Decreasing Mass

Start - End	Observed	Mr(expt)	Mr(calc)	Delta	Miss	Sequence
31 - 49	2180.1599	2179.1526	2179.1901	-0.0375	1	KYVATLGVEVHPLVFHTNR (Ions score 65)
31 - 49	2180.1599	2179.1526	2179.1901	-0.0375	1	KYVATLGVEVHPLVFHTNR (No match)
32 - 49	2052.0693	2051.0620	2051.0952	-0.0331	0	YVATLGVEVHPLVFHTNR (Ions score 73)
32 - 49	2052.0693	2051.0620	2051.0952	-0.0331	0	YVATLGVEVHPLVFHTNR (No match)
70 - 88	2267.0154	2266.0081	2266.0398	-0.0317	0	DGYYIQAQCAIIMFDVTSR Oxidation (M) (No match)
93 - 99	922.4590	921.4517	921.4569	-0.0052	0	NVPNWHR (No match)
136 - 145	1214.5864	1213.5791	1213.5979	-0.0187	0	NLQYYDISAK (No match)
146 - 159	1784.8826	1783.8753	1783.9045	-0.0292	0	SNYNFEKPFLWLAR (No match)

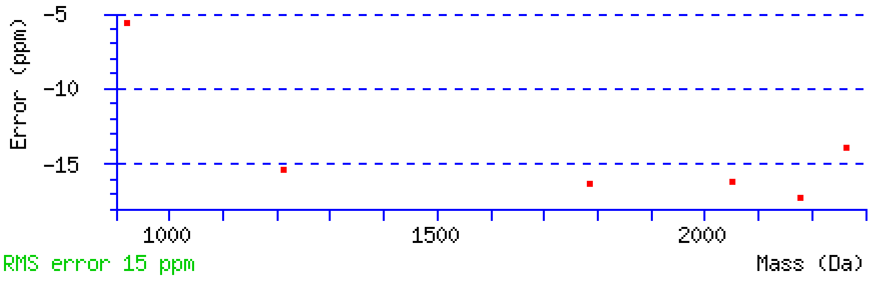

RMS error 15 ppm

Mascot: http://www.matrixscience.com/

Spot 756

Protein View

Match to: **gi|47496673** Score: **299** Expect: **2.4e-025**
GRB2 [Homo sapiens]

Nominal mass (M_r): **25246**; Calculated pI value: **6.08**
NCBI BLAST search of gi|47496673 against nr
Unformatted sequence string for pasting into other applications

Taxonomy: Homo sapiens

Fixed modifications: Carbamidomethyl (C)
Variable modifications: Oxidation (M)
Cleavage by Trypsin: cuts C-term side of KR unless next residue is P
Sequence Coverage: **45%**

Matched peptides shown in **Bold Red**

```
  1 MEAIAKYDFK ATADDELSFK RGDILKVLNE ECDQNWYKAE LNGKDGFIPK
 51 NYIEMKPHPW FFGKIPRAKA EEMLSKQRHD GAFLIRESES APGDFSLSVK
101 FGNDVQHFKV LRDGAGKYFL WVVKFNSLNE LVDYHRSTSV SRNQQIFLRD
151 IEQVPQQPTY VQALFDFDPQ EDGELGFRRG DFIHVMDNSG PNWWKGACHG
201 QTGMFPRNYV TPVNRNV
```

Residue Number Increasing Mass Decreasing Mass

Start - End	Observed	Mr(expt)	Mr(calc)	Delta	Miss	Sequence
27 - 38	1597.6671	1596.6598	1596.6878	-0.0280	0	VLNEECDQNWYK (No match)
51 - 64	1809.8451	1808.8378	1808.8708	-0.0329	0	NYIEMKPHPWFFGK Oxidation (M) (No match)
77 - 86	1212.6425	1211.6352	1211.6523	-0.0171	1	QRHDGAFLIR (No match)
77 - 86	1212.6425	1211.6352	1211.6523	-0.0171	1	QRHDGAFLIR (Ions score 33)
79 - 86	928.4930	927.4857	927.4926	-0.0069	0	HDGAFLIR (No match)
87 - 100	1452.6597	1451.6524	1451.6779	-0.0255	0	ESESAPGDFSLSVK (No match)
101 - 109	1091.5115	1090.5042	1090.5195	-0.0153	0	FGNDVQHFK (No match)
125 - 136	1506.7113	1505.7040	1505.7262	-0.0222	0	FNSLNELVDYHR (No match)
125 - 136	1506.7113	1505.7040	1505.7262	-0.0222	0	FNSLNELVDYHR (Ions score 91)
143 - 149	918.5081	917.5008	917.5083	-0.0074	0	NQQIFLR (No match)
196 - 207	1318.5648	1317.5575	1317.5706	-0.0131	0	GACHGQTGMFPR (No match)
196 - 207	1334.5508	1333.5435	1333.5655	-0.0220	0	GACHGQTGMFPR Oxidation (M) (No match)

196 - 207	1334.5508	1333.5435	1333.5655	-0.0220	0	**GACHGQTGMFPR**	Oxidation (M) (Ions score 10)
208 - 215	962.4964	961.4891	961.4981	-0.0090	0	**NYVTPVNR**	(Ions score 13)
208 - 215	962.4964	961.4891	961.4981	-0.0090	0	**NYVTPVNR**	(No match)

Mascot: http://www.matrixscience.com/

Spot 758

Protein View

Match to: **gi|7023036** Score: **161** Expect: **1.5e-011**
unnamed protein product [Homo sapiens]

Nominal mass (M_r): **25163;** Calculated pI value: **6.71**
NCBI BLAST search of gi|7023036 against nr
Unformatted sequence string for pasting into other applications

Taxonomy: Homo sapiens

Fixed modifications: Carbamidomethyl (C)
Variable modifications: Oxidation (M)
Cleavage by Trypsin: cuts C-term side of KR unless next residue is P
Sequence Coverage: **18%**

Matched peptides shown in **Bold Red**

```
  1 MSFLFSSRSS KTFKPKKNIP EGSHQYELLK HAEATLGSGN LRQAVMLPEG
 51 EDLNEWIAVN TVDFFNQINM LYGTITEFCT EASCPVMSAG PRYEYHWADG
101 TNIKKPIKCS APKYIDYLMT WVQDQLDDET LFPSKIGVPF PKNFMSVAKT
151 ILKRLFRVYA HIYHQHFDSV MQLQEGAHLN TSFKHFIFFV QEFNLIDRRE
201 LAPLQELIEK LGSKDR
```

Residue Number Increasing Mass Decreasing Mass

Start - End	Observed	Mr(expt)	Mr(calc)	Delta	Miss	Sequence	
17 - 30	1655.8535	1654.8462	1654.8678	-0.0216	1	KNIPEGSHQYELLK	(Ions score 72)
17 - 30	1655.8535	1654.8462	1654.8678	-0.0216	1	KNIPEGSHQYELLK	(No match)
31 - 42	1225.6174	1224.6101	1224.6210	-0.0109	0	HAEATLGSGNLR	(No match)
31 - 42	1225.6174	1224.6101	1224.6210	-0.0109	0	HAEATLGSGNLR	(Ions score 33)
185 - 198	1824.9188	1823.9115	1823.9358	-0.0243	0	HFIFFVQEFNLIDR	(No match)
185 - 198	1824.9188	1823.9115	1823.9358	-0.0243	0	HFIFFVQEFNLIDR	(Ions score 13)

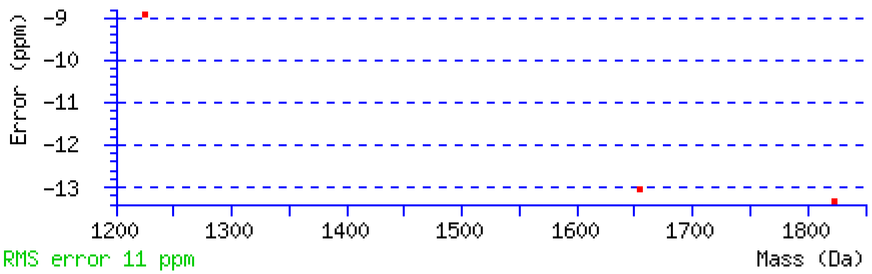

Mascot: http://www.matrixscience.com/

Spot 760

Protein View

Match to: **gi|119567805** Score: **135** Expect: **6.1e-009**
proteasome (prosome, macropain) subunit, beta type, 1, isoform CRA_a [Homo sapiens]

Nominal mass (M_r): **24634**; Calculated pI value: **8.69**
NCBI BLAST search of gi|119567805 against nr
Unformatted sequence string for pasting into other applications

Taxonomy: Homo sapiens

Fixed modifications: Carbamidomethyl (C)
Variable modifications: Oxidation (M)
Cleavage by Trypsin: cuts C-term side of KR unless next residue is P
Sequence Coverage: **60%**

Matched peptides shown in **Bold Red**

```
  1 MRCVSLFRLA LLKFTCTILA IAGEDFAIVA SDTRLSEGFS IHTRDSPKCY
 51 KLTDKTVIGC SGFHGDCLTL TKIIEARLKM YKHSNNKAMT TGAIAAMLST
101 ILYSRRFFPY YVYNIIGGLD EEGKGAVYSF DPVGSYQRDS FKAGGSASAM
151 LQPLLDNQVG FKNMQNVEHV PLSLDRAMRL VKDVFISAAE RDVYTGDALR
201 ICIVTKEGIR EETVSLRKD
```

Residue Number Increasing Mass Decreasing Mass

Start - End	Observed	Mr(expt)	Mr(calc)	Delta	Miss	Sequence
35 - 44	1146.5850	1145.5777	1145.5829	-0.0051	0	LSEGFSIHTR (No match)
35 - 44	1146.5850	1145.5777	1145.5829	-0.0051	0	LSEGFSIHTR (No match)
56 - 72	1865.8843	1864.8770	1864.8811	-0.0041	0	TVIGCSGFHGDCLTLTK (No match)
88 - 105	1902.9545	1901.9472	1901.9590	-0.0118	0	AMTTGAIAAMLSTILYSR 2 Oxidation (M) (No match)
106 - 124	2280.1057	2279.0984	2279.1261	-0.0277	1	RFFPYYVYNIIGGLDEEGK (No match)
106 - 124	2280.1057	2279.0984	2279.1261	-0.0277	1	RFFPYYVYNIIGGLDEEGK (No match)
125 - 138	1545.7189	1544.7116	1544.7259	-0.0143	0	GAVYSFDPVGSYQR (Ions score 9)
125 - 138	1545.7189	1544.7116	1544.7259	-0.0143	0	GAVYSFDPVGSYQR (No match)
143 - 162	2019.9980	2018.9907	2019.0094	-0.0187	0	AGGSASAMLQPLLDNQVGFK Oxidation (M) (No match)
163 - 176	1667.7975	1666.7902	1666.8096	-0.0194	0	NMQNVEHVPLSLDR Oxidation (M) (No match)
163 - 176	1667.7975	1666.7902	1666.8096	-0.0194	0	NMQNVEHVPLSLDR Oxidation (M) (No match)
180 - 191	1347.7614	1346.7541	1346.7557	-0.0016	1	LVKDVFISAAER (No match)

183 - 191	1007.5068	1006.4995	1006.5083	-0.0088	0	DVFISAAER	(No match)
183 - 200	1997.9912	1996.9839	1996.9853	-0.0014	1	DVFISAAERDVYTGDALR	(No match)

Mascot: http://www.matrixscience.com/

Spot 762

Protein View

Match to: **gi|55959887** Score: **216** Expect: **4.8e-017**
peroxiredoxin 1 [Homo sapiens]

Nominal mass (M_r): **19135**; Calculated pI value: **6.41**
NCBI BLAST search of gi|55959887 against nr
Unformatted sequence string for pasting into other applications

Taxonomy: Homo sapiens

Fixed modifications: Carbamidomethyl (C)
Variable modifications: Oxidation (M)
Cleavage by Trypsin: cuts C-term side of KR unless next residue is P
Sequence Coverage: **29%**

Matched peptides shown in **Bold Red**

```
  1 MSSGNAKIGH PAPNFKATAV MPDGQFKDIS LSDYKGKYVV FFFYPLDFTF
 51 VCPTEIIAFS DRAEEFKKLN CQVIGASVDS HFCHLAWVNT PKKQGGLGPM
101 NIPLVSDPKR TIAQDYGVLK ADEGISFRGL FIIDDKGILR QITVNDLPVG
151 RSVDETLRLV QAFQFTDKHG E
```

Residue Number Increasing Mass Decreasing Mass

Start - End	Observed	Mr(expt)	Mr(calc)	Delta	Miss	Sequence
8 - 16	980.5121	979.5048	979.5239	-0.0191	0	IGHPAPNFK (No match)
8 - 16	980.5121	979.5048	979.5239	-0.0191	0	IGHPAPNFK (Ions score 30)
121 - 128	894.4235	893.4162	893.4242	-0.0080	0	ADEGISFR (No match)
129 - 140	1359.7793	1358.7720	1358.7921	-0.0201	1	GLFIIDDKGILR (No match)
129 - 140	1359.7793	1358.7720	1358.7921	-0.0201	1	GLFIIDDKGILR (Ions score 51)
141 - 151	1211.6575	1210.6502	1210.6669	-0.0167	0	QITVNDLPVGR (No match)
141 - 151	1211.6575	1210.6502	1210.6669	-0.0167	0	QITVNDLPVGR (Ions score 29)
159 - 168	1196.6102	1195.6029	1195.6237	-0.0207	0	LVQAFQFTDK (No match)
159 - 168	1196.6102	1195.6029	1195.6237	-0.0207	0	LVQAFQFTDK (Ions score 27)

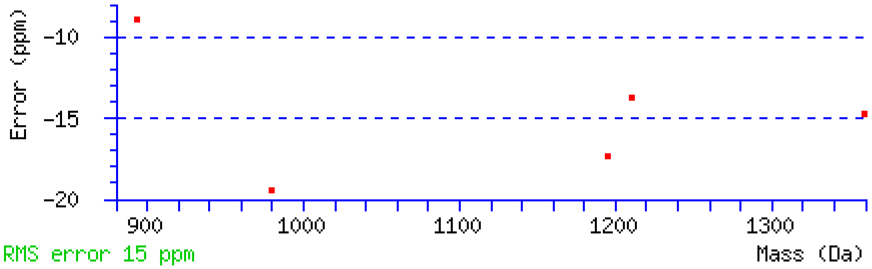

Mascot: http://www.matrixscience.com/

Spot 764

Protein View

Match to: **gi|42543006** Score: **125** Expect: **6.1e-008**
Chain A, Crystal Structure Of Human Dj-1

Nominal mass (M_r): **20063**; Calculated pI value: **6.33**
NCBI BLAST search of gi|42543006 against nr
Unformatted sequence string for pasting into other applications

Taxonomy: Homo sapiens
Links to retrieve other entries containing this sequence from NCBI Entrez:
gi|16751471 from Homo sapiens

Fixed modifications: Carbamidomethyl (C)
Variable modifications: Oxidation (M)
Cleavage by Trypsin: cuts C-term side of KR unless next residue is P
Sequence Coverage: **32%**

Matched peptides shown in **Bold Red**

```
  1 MASKRALVIL AKGAEEMETV IPVDVMRRAG IKVTVAGLAG KDPVQCSRDV
 51 VICPDASLED AKKEGPYDVV VLPGGNLGAQ NLSESAAVKE ILKEQENRKG
101 LIAAICAGPT ALLAHEIGCG SKVTTHPLAK DKMMNGGHYT YSENRVEKDG
151 LILTSRGPGT SFEFALAIVE ALNGKEVAAQ VKAPLVLKD
```

Residue Number Increasing Mass Decreasing Mass

Start - End	Observed	Mr(expt)	Mr(calc)	Delta	Miss	Sequence
13 - 27	1707.7433	1706.7360	1706.7854	-0.0494	0	GAEEMETVIPVDVMR 2 Oxidation (M) (Ions score 2)
13 - 27	1707.7433	1706.7360	1706.7854	-0.0494	0	GAEEMETVIPVDVMR 2 Oxidation (M) (No match)
13 - 28	1863.8407	1862.8334	1862.8866	-0.0531	1	GAEEMETVIPVDVMRR 2 Oxidation (M) (No match)
13 - 28	1863.8407	1862.8334	1862.8866	-0.0531	1	GAEEMETVIPVDVMRR 2 Oxidation (M) (Ions score 19)
33 - 48	1657.8236	1656.8163	1656.8617	-0.0453	1	VTVAGLAGKDPVQCSR (Ions score 26)
33 - 48	1657.8236	1656.8163	1656.8617	-0.0453	1	VTVAGLAGKDPVQCSR (No match)
49 - 63	1659.7982	1658.7909	1658.8185	-0.0276	1	DVVICPDASLEDAKK (No match)
131 - 145	1834.6963	1833.6890	1833.7410	-0.0519	1	DKMMNGGHYTYSENR 2 Oxidation (M) (No match)
131 - 145	1834.6963	1833.6890	1833.7410	-0.0519	1	DKMMNGGHYTYSENR 2 Oxidation (M) (No match)

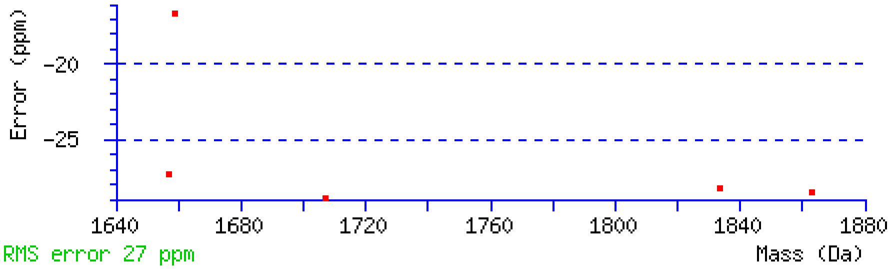

Mascot: http://www.matrixscience.com/

Spot 765

Protein View

Match to: **gi|7705704** Score: **147** Expect: **3.8e-010**
glutathione transferase kappa 1 [Homo sapiens]

Nominal mass (M_r): **25594**; Calculated pI value: **8.50**
NCBI BLAST search of gi|7705704 against nr
Unformatted sequence string for pasting into other applications

Taxonomy: Homo sapiens
Links to retrieve other entries containing this sequence from NCBI Entrez:
gi|12643338 from Homo sapiens
gi|83753782 from Homo sapiens
gi|83753783 from Homo sapiens
gi|4454690 from Homo sapiens
gi|12053371 from Homo sapiens
gi|12654777 from Homo sapiens
gi|30048184 from Homo sapiens
gi|41351794 from Homo sapiens
gi|41353193 from Homo sapiens
gi|49065316 from Homo sapiens
gi|51094528 from Homo sapiens
gi|119572265 from Homo sapiens
gi|158260183 (no taxonomy information for this entry)

Fixed modifications: Carbamidomethyl (C)
Variable modifications: Oxidation (M)
Cleavage by Trypsin: cuts C-term side of KR unless next residue is P
Sequence Coverage: **15%**

Matched peptides shown in **Bold Red**

1 MGPLPRTVEL FYDVLSPYSW LGFEILCRYQ NIWNINLQLR PSLITGIMK**D**
51 **SGNKPPGLLP R**KGLYMANDL KLLR**HHLQIP IHFPK**DFLSV MLEKGSLSAM
101 RFLTAVNLEH PEMLEKASRE LWMRVWSRNE DITEPQSILA AAEKAGMSAE
151 QAQGLLEKIA TPKVKNQLKE TTEAACRYGA FGLPITVAHV DGQTHMLFGS
201 DRMELLAHLL GEK**WMGPIPP AVNAR**L

Residue Number Increasing Mass Decreasing Mass

Start - End	Observed	Mr(expt)	Mr(calc)	Delta	Miss	Sequence
50 - 61	1250.6569	1249.6496	1249.6778	-0.0282	0	DSGNKPPGLLPR (No match)
50 - 61	1250.6569	1249.6496	1249.6778	-0.0282	0	DSGNKPPGLLPR (Ions score 36)
75 - 85	1366.7354	1365.7281	1365.7669	-0.0388	0	HHLQIPIHFPK (No match)
75 - 85	1366.7354	1365.7281	1365.7669	-0.0388	0	HHLQIPIHFPK (Ions score 62)
214 - 225	1324.6428	1323.6355	1323.6757	-0.0402	0	WMGPIPPAVNAR Oxidation (M) (No match)
214 - 225	1324.6428	1323.6355	1323.6757	-0.0402	0	WMGPIPPAVNAR Oxidation (M) (No match)

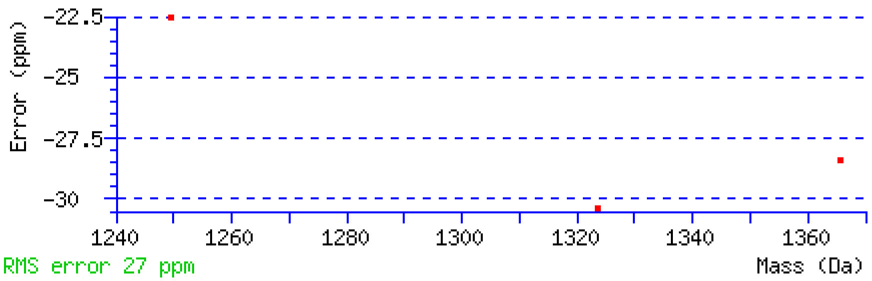

RMS error 27 ppm

Spot 766

Protein View

Match to: **gi|20664358** Score: **303** Expect: **9.6e-026**
Chain A, Crystal Structure Of A Recombinant Glutathione Transferase, Created By Replacing The Last

Nominal mass (M_r): **23430**; Calculated pI value: **5.09**
NCBI BLAST search of gi|20664358 against nr
Unformatted sequence string for pasting into other applications

Taxonomy: Homo sapiens
Links to retrieve other entries containing this sequence from NCBI Entrez:
gi|20664359 from Homo sapiens

Fixed modifications: Carbamidomethyl (C)
Variable modifications: Oxidation (M)
Cleavage by Trypsin: cuts C-term side of KR unless next residue is P
Sequence Coverage: **40%**

Matched peptides shown in **Bold Red**

```
  1 PPYTVVYFPV RGRCAALRML LADQGQSWKE EVVTVETWQE GSLKASCLYG
 51 QLPKFQDGDL TLYQSNTILR HLGRTLGLYG KDQQEAALVD MVNDGVEDLR
101 CKYISLIYTN YEAGKDDYVK ALPGQLKPFE TLLSQNQGGK TFIVGDQISF
151 ADYNLLDLLL IHEVLAPGCL DAFPLLSAYV GRLSARPKLK AFLASPEYVN
201 LPMDEKSL
```

Residue Number Increasing Mass Decreasing Mass

Start - End	Observed	Mr(expt)	Mr(calc)	Delta	Miss	Sequence
1 - 11	1337.7389	1336.7316	1336.7179	0.0137	0	PPYTVVYFPVR (Ions score 42)
1 - 11	1337.7389	1336.7316	1336.7179	0.0137	0	PPYTVVYFPVR (No match)
55 - 70	1883.9629	1882.9556	1882.9424	0.0132	0	FQDGDLTLYQSNTILR (No match)
55 - 70	1883.9629	1882.9556	1882.9424	0.0132	0	FQDGDLTLYQSNTILR (Ions score 83)
82 - 100	2133.0042	2131.9969	2131.9691	0.0278	0	DQQEAALVDMVNDGVEDLR Oxidation (M) (No match)
103 - 120	2155.0820	2154.0747	2154.0520	0.0227	1	YISLIYTNYEAGKDDYVK (Ions score 40)
103 - 120	2155.0820	2154.0747	2154.0520	0.0227	1	YISLIYTNYEAGKDDYVK (No match)
121 - 140	2126.1863	2125.1790	2125.1531	0.0260	0	ALPGQLKPFETLLSQNQGGK (No match)
121 - 140	2126.1863	2125.1790	2125.1531	0.0260	0	ALPGQLKPFETLLSQNQGGK (Ions score 62)

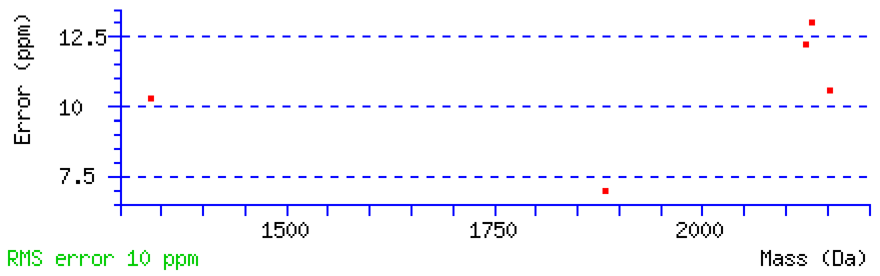

Mascot: http://www.matrixscience.com/

Spot 767

Protein View

Match to: **gi|56243533** Score: **105** Expect: **6.1e-006**
stromal cell-derived factor 2-like 1 precursor [Homo sapiens]

Nominal mass (M_r): **23812**; Calculated pI value: **6.52**
NCBI BLAST search of gi|56243533 against nr
Unformatted sequence string for pasting into other applications

Taxonomy: Homo sapiens
Links to retrieve other entries containing this sequence from NCBI Entrez:
gi|46397883 from Homo sapiens
gi|14579008 from Homo sapiens
gi|37183353 from Homo sapiens
gi|47678671 from Homo sapiens
gi|109451478 from synthetic construct
gi|109452074 from synthetic construct
gi|119579871 from Homo sapiens
gi|124375922 from Homo sapiens
gi|124376912 from Homo sapiens
gi|141797051 from synthetic construct

Fixed modifications: Carbamidomethyl (C)
Variable modifications: Oxidation (M)
Cleavage by Trypsin: cuts C-term side of KR unless next residue is P
Sequence Coverage: **22%**

Matched peptides shown in **Bold Red**

```
  1 MWSAGRGGAA WPVLLGLLLA LLVPGGGAAK TGAELVTCGS VLKLLNTHHR
 51 VRLHSHDIKY GSGSGQQSVT GVEASDDANS YWRIRGGSEG GCPRGSPVRC
101 GQAVRLTHVL TGKNLHTHHF PSPLSNNQEV SAFGEDGEGD DLDLWTVRCS
151 GQHWEREAAV RFQHVGTSVF LSVTGEQYGS PIRGQHEVHG MPSANTHNTW
201 KAMEGIFIKP SVEPSAGHDE L
```

Residue Number Increasing Mass Decreasing Mass

Start - End	Observed	Mr(expt)	Mr(calc)	Delta	Miss	Sequence	
44 - 50	890.5039	889.4966	889.4882	0.0084	0	LLNTHHR	(No match)

162 - 183	2409.1799	2408.1726	2408.2124	-0.0397	0	FQHVGTSVFLSVTGEQYGSPIR	(Ions score 48)
162 - 183	2409.1799	2408.1726	2408.2124	-0.0397	0	FQHVGTSVFLSVTGEQYGSPIR	(No match)
202 - 221	2143.0305	2142.0232	2142.0302	-0.0070	0	AMEGIFIKPSVEPSAGHDEL	Oxidation (M) (Ions score 24)
202 - 221	2143.0305	2142.0232	2142.0302	-0.0070	0	AMEGIFIKPSVEPSAGHDEL	Oxidation (M) (No match)

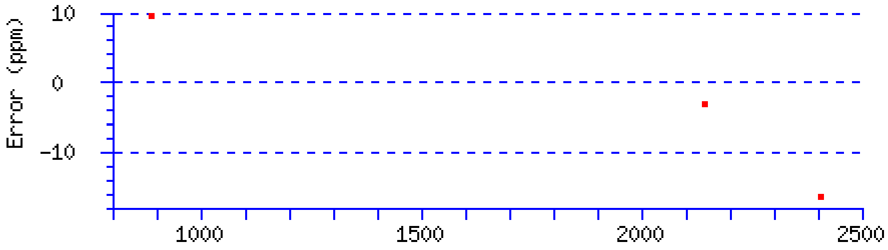

RMS error 11 ppm

Mascot: http://www.matrixscience.com/

Spot 768

Protein View

Match to: **gi|119569783** Score: **113** Expect: **9.6e-007**
peroxiredoxin 3, isoform CRA_c [Homo sapiens]

Nominal mass (M_r): **11158**; Calculated pI value: **6.06**
NCBI BLAST search of gi|119569783 against nr
Unformatted sequence string for pasting into other applications

Taxonomy: Homo sapiens

Fixed modifications: Carbamidomethyl (C)
Variable modifications: Oxidation (M)
Cleavage by Trypsin: cuts C-term side of KR unless next residue is P
Sequence Coverage: **36%**

Matched peptides shown in **Bold Red**

```
  1 MNIALLSDLT KQISRDYGVL LEGSGLALRG LFIIDPNGVI KHLSVNDLPV
 51 GRSVEETLRL VKAFQYVETH GEVCPANWTP DSPTIKPSPA ASKEYFQKVN
101 Q
```

Residue Number Increasing Mass Decreasing Mass

Start - End	Observed	Mr(expt)	Mr(calc)	Delta	Miss	Sequence
16 - 29	1462.8066	1461.7993	1461.7827	0.0167	0	DYGVLLEGSGLALR (No match)
16 - 29	1462.8066	1461.7993	1461.7827	0.0167	0	DYGVLLEGSGLALR (Ions score 28)
30 - 41	1285.7638	1284.7565	1284.7441	0.0124	0	GLFIIDPNGVIK (No match)
42 - 52	1206.6785	1205.6712	1205.6516	0.0196	0	HLSVNDLPVGR (Ions score 33)
42 - 52	1206.6785	1205.6712	1205.6516	0.0196	0	HLSVNDLPVGR (No match)

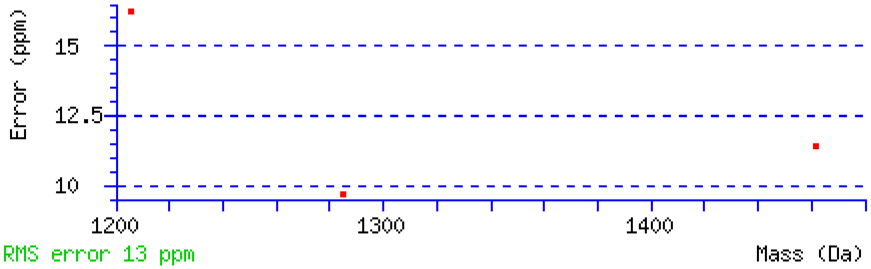

Mascot: http://www.matrixscience.com/

Spot 769

Protein View

Match to: **gi|4758086** Score: **592** Expect: **1.2e-054**
cysteine and glycine-rich protein 1 [Homo sapiens]

Nominal mass (M_r): **21409**; Calculated pI value: **8.90**
NCBI BLAST search of gi|4758086 against nr
Unformatted sequence string for pasting into other applications

Taxonomy: Homo sapiens
Links to retrieve other entries containing this sequence from NCBI Entrez:
gi|118161 from Homo sapiens
gi|181064 from Homo sapiens
gi|181071 from Homo sapiens
gi|21595352 from Homo sapiens
gi|54695910 from Homo sapiens
gi|61355815 from synthetic construct
gi|61364073 from synthetic construct
gi|119611777 from Homo sapiens
gi|158259567 (no taxonomy information for this entry)

Fixed modifications: Carbamidomethyl (C)
Variable modifications: Oxidation (M)
Cleavage by Trypsin: cuts C-term side of KR unless next residue is P
Sequence Coverage: **54%**

Matched peptides shown in **Bold Red**

```
  1 MPNWGGGKKC GVCQKTVYFA EEVQCEGNSF HKSCFLCMVC KKNLDSTTVA
 51 VHGEEIYCKS CYGKKYGPKG YGYGQGAGTL STDKGESLGI KHEEAPGHRP
101 TTNPNASKFA QKIGGSERCP RCSQAVYAAE KVIGAGKSWH KACFRCAKCG
151 KGLESTTLAD KDGEIYCKGC YAKNFGPKGF GFGQGAGALV HSE
```

Residue Number Increasing Mass Decreasing Mass

Start - End	Observed	Mr(expt)	Mr(calc)	Delta	Miss	Sequence	
16 - 32	2044.8696	2043.8623	2043.8996	-0.0372	0	TVYFAEEVQCEGNSFHK	(No match)
16 - 32	2044.8696	2043.8623	2043.8996	-0.0372	0	TVYFAEEVQCEGNSFHK	(Ions score 106)
43 - 59	1935.8730	1934.8657	1934.9043	-0.0386	0	NLDSTTVAVHGEEIYCK	(No match)

43 - 59	1935.8730	1934.8657	1934.9043	-0.0386	0	**NLDSTTVAVHGEEIYCK**	(Ions score 102)
70 - 91	2159.0229	2158.0156	2158.0541	-0.0385	1	**GYGYGQGAGTLSTDKGESLGIK**	(No match)
70 - 91	2159.0229	2158.0156	2158.0541	-0.0385	1	**GYGYGQGAGTLSTDKGESLGIK**	(Ions score 145)
92 - 108	1842.8501	1841.8428	1841.8768	-0.0340	0	**HEEAPGHRPTTNPNASK**	(Ions score 51)
92 - 108	1842.8501	1841.8428	1841.8768	-0.0340	0	**HEEAPGHRPTTNPNASK**	(No match)
152 - 168	1899.8668	1898.8595	1898.8931	-0.0336	1	**GLESTTLADKDGEIYCK**	(No match)
179 - 193	1433.6564	1432.6491	1432.6734	-0.0243	0	**GFGFGQGAGALVHSE**	(No match)
179 - 193	1433.6564	1432.6491	1432.6734	-0.0243	0	**GFGFGQGAGALVHSE**	(Ions score 78)

Mascot: http://www.matrixscience.com/

Spot 770

Protein View

Match to: **gi|2554831** Score: **128** Expect: **3e-008**
Chain A, Crystal Structure Of Human Glutathione S-Transferase P1- 1[v104] Complexed With (9r,10r)-9

Nominal mass (M_r): **23555**; Calculated pI value: **5.43**
NCBI BLAST search of gi|2554831 against nr
Unformatted sequence string for pasting into other applications

Taxonomy: Homo sapiens
Links to retrieve other entries containing this sequence from NCBI Entrez:
gi|2554832 from Homo sapiens
gi|2554839 from Homo sapiens
gi|2554840 from Homo sapiens
gi|2076717 from Homo sapiens
gi|15012036 from Homo sapiens
gi|61363361 from synthetic construct
gi|123984637 from synthetic construct
gi|123998619 from synthetic construct

Fixed modifications: Carbamidomethyl (C)
Variable modifications: Oxidation (M)
Cleavage by Trypsin: cuts C-term side of KR unless next residue is P
Sequence Coverage: **28%**

Matched peptides shown in **Bold Red**

1 M**PPYTVVYFP VR**GRCAALRM LLADQGQSWK EEVVTVETWQ EGSLKASCLY
51 GQLPK**FQDGD LTLYQSNTIL R**HLGRTLGLY GKDQQEAALV DMVNDGVEDL
101 RCK**YVSLIYT NYEAGK**DDYV K**ALPGQLKPF ETLLSQNQGG K**TFIVGDQIS
151 FADYNLLDLL LIHEVLAPGC LDAFPLLSAY VGRLSARPKL KAFLASPEYV
201 NLPINGNGKQ

Residue Number Increasing Mass Decreasing Mass

Start - End	Observed	Mr(expt)	Mr(calc)	Delta	Miss	Sequence
2 - 12	1337.7286	1336.7213	1336.7179	0.0034	0	PPYTVVYFPVR (No match)
2 - 12	1337.7286	1336.7213	1336.7179	0.0034	0	PPYTVVYFPVR (Ions score 12)
56 - 71	1883.9369	1882.9296	1882.9424	-0.0128	0	FQDGDLTLYQSNTILR (No match)

56 - 71	1883.9369	1882.9296	1882.9424	-0.0128	0	FQDGDLTLYQSNTILR	(Ions score 28)
104 - 116	1520.7582	1519.7509	1519.7558	-0.0049	0	YVSLIYTNYEAGK	(No match)
104 - 116	1520.7582	1519.7509	1519.7558	-0.0049	0	YVSLIYTNYEAGK	(No match)
122 - 141	2126.1492	2125.1419	2125.1531	-0.0111	0	ALPGQLKPFETLLSQNQGGK	(No match)
122 - 141	2126.1492	2125.1419	2125.1531	-0.0111	0	ALPGQLKPFETLLSQNQGGK	(Ions score 27)

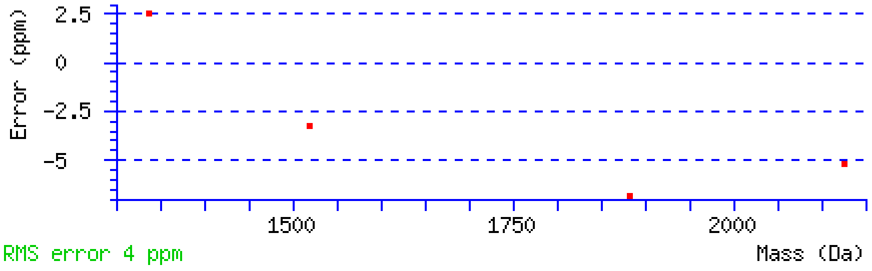

RMS error 4 ppm

Mascot: http://www.matrixscience.com/

Spot 771

Protein View

Match to: **gi|13325316** Score: **79** Expect: **0.0024**
PYD and CARD domain containing [Homo sapiens]

Nominal mass (M_r): **15078**; Calculated pI value: **6.84**
NCBI BLAST search of gi|13325316 against nr
Unformatted sequence string for pasting into other applications

Taxonomy: Homo sapiens

Fixed modifications: Carbamidomethyl (C)
Variable modifications: Oxidation (M)
Cleavage by Trypsin: cuts C-term side of KR unless next residue is P
Sequence Coverage: **17%**

Matched peptides shown in **Bold Red**

```
  1 MGRARDAILD ALENLTAEEL KKFKLQAATH QGSGAAPAGI QAPPQSAAKP
 51 GLHFIDQHRA ALIARVTNVE WLLDALYGKV LTDEQYQAVR AEPTNPSKMR
101 KLFSFTPAWN WTCKDLLLQA LRESQSYLVE DLERS
```

Residue Number Increasing Mass Decreasing Mass

Start - End	Observed	Mr(expt)	Mr(calc)	Delta	Miss	Sequence	
80 - 90	1321.6418	1320.6345	1320.6673	-0.0328	0	VLTDEQYQAVR	(No match)
80 - 90	1321.6418	1320.6345	1320.6673	-0.0328	0	VLTDEQYQAVR	(Ions score 24)
123 - 134	1467.6565	1466.6492	1466.6888	-0.0396	0	ESQSYLVEDLER	(Ions score 11)
123 - 134	1467.6565	1466.6492	1466.6888	-0.0396	0	ESQSYLVEDLER	(No match)
123 - 135	1554.6838	1553.6765	1553.7209	-0.0443	1	ESQSYLVEDLERS	(No match)
123 - 135	1554.6838	1553.6765	1553.7209	-0.0443	1	ESQSYLVEDLERS	(No match)

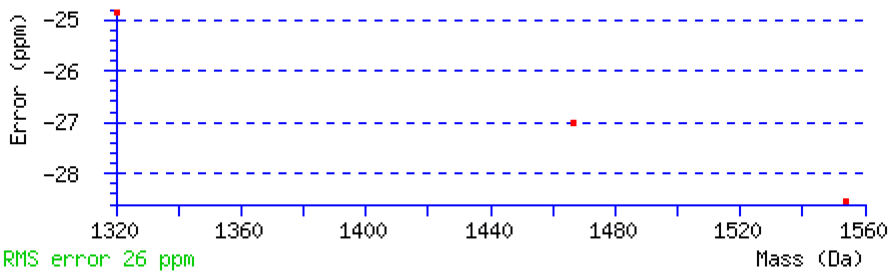

Mascot: http://www.matrixscience.com/

Spot 772

Protein View

Match to: **gi|1617118** Score: **97** Expect: **4.1e-005**
TSA [Homo sapiens]

Nominal mass (M_r): **18486;** Calculated pI value: **5.19**
NCBI BLAST search of gi|1617118 against nr
Unformatted sequence string for pasting into other applications

Taxonomy: Homo sapiens

Fixed modifications: Carbamidomethyl (C)
Variable modifications: Oxidation (M)
Cleavage by Trypsin: cuts C-term side of KR unless next residue is P
Sequence Coverage: **17%**

Matched peptides shown in **Bold Red**

```
  1 GKYVVLFFYP LDFTFVCPTE IIAFSNRAED FRKLGCEVLG VSVDSQFTHL
 51 AWINTPRKEG GLGPLNIPLL ADVTRRLSED YGVLKTDEGI AYRGLFIIDG
101 KGVLRQITVN DLPVGRSVDE ALRLVQAFQY TDEHGEVCPA GWKPGSDTIK
151 PNVDDSKEYF SKHN
```

Residue Number Increasing Mass Decreasing Mass

Start - End	Observed	Mr(expt)	Mr(calc)	Delta	Miss	Sequence
58 - 75	1863.1012	1862.0939	1862.0625	0.0315	1	KEGGLGPLNIPLLADVTR (No match)
58 - 75	1863.1012	1862.0939	1862.0625	0.0315	1	KEGGLGPLNIPLLADVTR (Ions score 19)
59 - 75	1735.0039	1733.9966	1733.9675	0.0291	0	EGGLGPLNIPLLADVTR (No match)
59 - 75	1735.0039	1733.9966	1733.9675	0.0291	0	EGGLGPLNIPLLADVTR (Ions score 18)
106 - 116	1211.7036	1210.6963	1210.6669	0.0294	0	QITVNDLPVGR (No match)
106 - 116	1211.7036	1210.6963	1210.6669	0.0294	0	QITVNDLPVGR (Ions score 19)

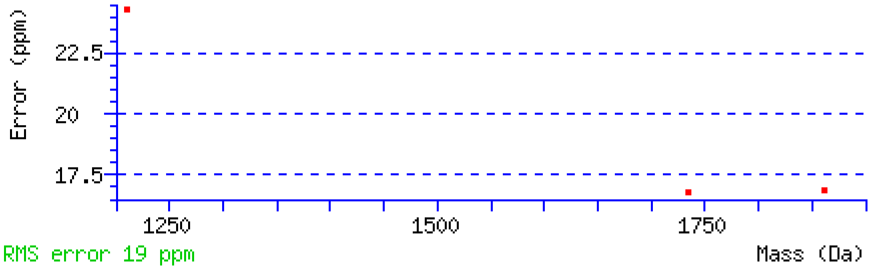

Mascot: http://www.matrixscience.com/

Spot 773

Protein View

Match to: **gi|49456283** Score: **279** Expect: **2.4e-023**
PSMB8 [Homo sapiens]

Nominal mass (M_r): **30019**; Calculated pI value: **5.53**
NCBI BLAST search of gi|49456283 against nr
Unformatted sequence string for pasting into other applications

Taxonomy: Homo sapiens

Fixed modifications: Carbamidomethyl (C)
Variable modifications: Oxidation (M)
Cleavage by Trypsin: cuts C-term side of KR unless next residue is P
Sequence Coverage: **22%**

Matched peptides shown in **Bold Red**

```
  1 MLIGTPTPRD TTPSSWLTSS LLVEAAPLDD TTLPTPVSSG CPGLEPTEFF
 51 QSLGGDGERN VQIEMAHGTT TLAFKFQHGV IAAVDSRASA GSYISALRVN
101 KVIEINPYLL GTMSGCAADC QYWERLLAKE CRLYYLRNGE RISVSAASKL
151 LSNMMCQYRG MGLSMGSMIC GWDKKGPGLY YVDEHGTRLS GNMFSTGSGN
201 TYAYGVMDSG YRPNLSPEEA YDLGRRAIAY ATHRDSYSGG VVNMYHMKED
251 GLVKVESTDV SDLLHQYREA NQ
```

Residue Number Increasing Mass Decreasing Mass

Start - End	Observed	Mr(expt)	Mr(calc)	Delta	Miss	Sequence
76 - 87	1299.6625	1298.6552	1298.6731	-0.0178	0	FQHGVIAAVDSR (Ions score 40)
76 - 87	1299.6625	1298.6552	1298.6731	-0.0178	0	FQHGVIAAVDSR (No match)
88 - 98	1095.5649	1094.5576	1094.5720	-0.0143	0	ASAGSYISALR (No match)
150 - 159	1347.5607	1346.5534	1346.5781	-0.0247	0	LLSNMMCQYR 2 Oxidation (M) (No match)
175 - 188	1591.7604	1590.7531	1590.7790	-0.0259	1	KGPGLYYVDEHGTR (Ions score 38)
175 - 188	1591.7604	1590.7531	1590.7790	-0.0259	1	KGPGLYYVDEHGTR (No match)
176 - 188	1463.6744	1462.6671	1462.6840	-0.0169	0	GPGLYYVDEHGTR (No match)
255 - 268	1661.7839	1660.7766	1660.8056	-0.0290	0	VESTDVSDLLHQYR (Ions score 120)
255 - 268	1661.7839	1660.7766	1660.8056	-0.0290	0	VESTDVSDLLHQYR (No match)

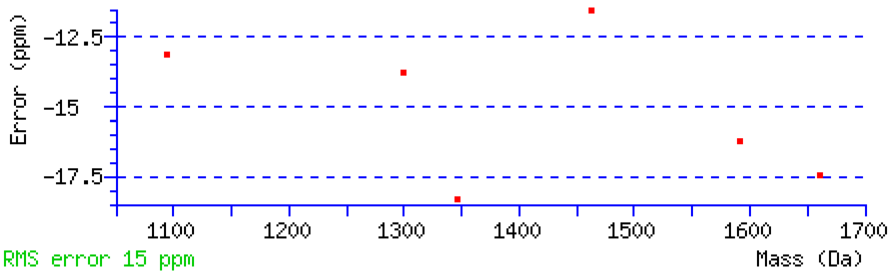

RMS error 15 ppm

Mascot: http://www.matrixscience.com/

Spot 774

Protein View

Match to: **gi|55959887** Score: **227** Expect: **3.8e-018**
peroxiredoxin 1 [Homo sapiens]

Nominal mass (M_r): **19135**; Calculated pI value: **6.41**
NCBI BLAST search of gi|55959887 against nr
Unformatted sequence string for pasting into other applications

Taxonomy: Homo sapiens

Fixed modifications: Carbamidomethyl (C)
Variable modifications: Oxidation (M)
Cleavage by Trypsin: cuts C-term side of KR unless next residue is P
Sequence Coverage: **50%**

Matched peptides shown in **Bold Red**

```
  1 MSSGNAKIGH PAPNFKATAV MPDGQFKDIS LSDYKGKYVV FFFYPLDFTF
 51 VCPTEIIAFS DRAEEFKKLN CQVIGASVDS HFCHLAWVNT PKKQGGLGPM
101 NIPLVSDPKR TIAQDYGVLK ADEGISFRGL FIIDDKGILR QITVNDLPVG
151 RSVDETLRLV QAFQFTDKHG E
```

Residue Number Increasing Mass Decreasing Mass

Start - End	Observed	Mr(expt)	Mr(calc)	Delta	Miss	Sequence
8 - 16	980.5318	979.5245	979.5239	0.0006	0	IGHPAPNFK (No match)
28 - 35	940.4567	939.4494	939.4549	-0.0055	0	DISLSDYK (No match)
93 - 109	1767.0156	1766.0083	1765.9396	0.0687	1	KQGGLGPMNIPLVSDPK Oxidation (M) (No match)
94 - 109	1638.9037	1637.8964	1637.8446	0.0518	0	QGGLGPMNIPLVSDPK Oxidation (M) (No match)
94 - 110	1795.0231	1794.0158	1793.9457	0.0701	1	QGGLGPMNIPLVSDPKR Oxidation (M) (No match)
111 - 120	1107.6167	1106.6094	1106.5971	0.0123	0	TIAQDYGVLK (No match)
111 - 128	1983.0951	1982.0878	1982.0108	0.0770	1	TIAQDYGVLKADEGISFR (Ions score 15)
111 - 128	1983.0951	1982.0878	1982.0108	0.0770	1	TIAQDYGVLKADEGISFR (No match)
129 - 140	1359.8359	1358.8286	1358.7921	0.0365	1	GLFIIDDKGILR (Ions score 52)
129 - 140	1359.8359	1358.8286	1358.7921	0.0365	1	GLFIIDDKGILR (No match)
141 - 151	1211.6990	1210.6917	1210.6669	0.0248	0	QITVNDLPVGR (No match)
141 - 151	1211.6990	1210.6917	1210.6669	0.0248	0	QITVNDLPVGR (Ions score 31)
159 - 168	1196.6575	1195.6502	1195.6237	0.0266	0	LVQAFQFTDK (No match)

Spot 775

Protein View

Match to: **gi|119627813** Score: **100** Expect: **1.9e-005**
proteasome (prosome, macropain) subunit, beta type, 2, isoform CRA_b [Homo sapiens]

Nominal mass (M_r): **20365**; Calculated pI value: **6.96**
NCBI BLAST search of gi|119627813 against nr
Unformatted sequence string for pasting into other applications

Taxonomy: Homo sapiens

Fixed modifications: Carbamidomethyl (C)
Variable modifications: Oxidation (M)
Cleavage by Trypsin: cuts C-term side of KR unless next residue is P
Sequence Coverage: **25%**

Matched peptides shown in **Bold Red**

```
  1 MEYLIDHDKM FKMSEKILLL CVGEAGDTVQ FAEYIQKNVQ LYKMRNGYEL
 51 SPTAAANFTR RNLADCLRSR TPYHVNLLLA GYDEHEGPAL YYMDYLAALA
101 KAPFAAHGYG AFLTLSILDR YYTPTISRER AVELLRKCLE ELQKRFILNL
151 PTFSVRIIDK NGIHDLDNIS FPKQGS
```

Residue Number Increasing Mass Decreasing Mass

Start - End	Observed	Mr(expt)	Mr(calc)	Delta	Miss	Sequence
46 - 60	1611.7185	1610.7112	1610.7688	-0.0576	0	NGYELSPTAAANFTR (No match)
46 - 60	1611.7185	1610.7112	1610.7688	-0.0576	0	NGYELSPTAAANFTR (Ions score 21)
102 - 120	2019.9830	2018.9757	2019.0577	-0.0820	0	APFAAHGYGAFLTLSILDR (No match)
146 - 156	1306.7131	1305.7058	1305.7444	-0.0386	0	FILNLPTFSVR (No match)
146 - 156	1306.7131	1305.7058	1305.7444	-0.0386	0	FILNLPTFSVR (Ions score 32)

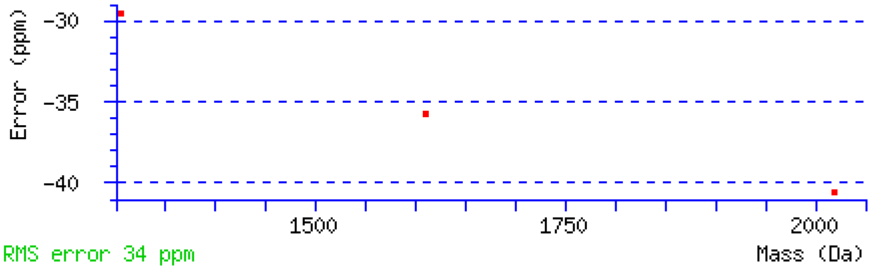

Mascot: http://www.matrixscience.com/

Spot 778

Protein View

Match to: **gi|9955007** Score: **319** Expect: **2.4e-027**
Chain A, Thioredoxin Peroxidase B From Red Blood Cells

Nominal mass (M_r): **21909;** Calculated pI value: **5.44**
NCBI BLAST search of gi|9955007 against nr
Unformatted sequence string for pasting into other applications

Taxonomy: Homo sapiens
Links to retrieve other entries containing this sequence from NCBI Entrez:
gi|9955008 from Homo sapiens
gi|9955009 from Homo sapiens
gi|9955010 from Homo sapiens
gi|9955011 from Homo sapiens
gi|9955012 from Homo sapiens
gi|9955013 from Homo sapiens
gi|9955014 from Homo sapiens
gi|9955015 from Homo sapiens
gi|9955016 from Homo sapiens

Fixed modifications: Carbamidomethyl (C)
Variable modifications: Oxidation (M)
Cleavage by Trypsin: cuts C-term side of KR unless next residue is P
Sequence Coverage: **35%**

Matched peptides shown in **Bold Red**

```
  1 XSGNARIGKP APDFKATAVV DGAFKEVKLS DYKGKYVVLF FYPLDFTFVX
 51 PTEIIAFSNR AEDFRKLGCE VLGVSVDSQF THLAWINTPR KEGGLGPLNI
101 PLLADVTRRL SEDYGVLKTD EGIAYRGLFI IDGKGVLRQI TVNDLPVGRS
151 VDEALRLVQA FQYTDEHGEV CPAGWKPGSD TIKPNVDDSK EYFSKHN
```

Residue Number Increasing Mass Decreasing Mass

Start - End	Observed	Mr(expt)	Mr(calc)	Delta	Miss	Sequence
7 - 15	972.5364	971.5291	971.5440	-0.0148	0	IGKPAPDFK (No match)
16 - 28	1334.7449	1333.7376	1333.7241	0.0135	1	ATAVVDGAFKEVK (No match)
91 - 108	1863.1202	1862.1129	1862.0625	0.0505	1	KEGGLGPLNIPLLADVTR (Ions score 50)

91 - 108	1863.1202	1862.1129	1862.0625	0.0505	1	KEGGLGPLNIPLLADVTR (No match)
92 - 108	1735.0187	1734.0114	1733.9675	0.0439	0	EGGLGPLNIPLLADVTR (Ions score 49)
92 - 108	1735.0187	1734.0114	1733.9675	0.0439	0	EGGLGPLNIPLLADVTR (No match)
92 - 109	1891.1334	1890.1261	1890.0686	0.0575	1	EGGLGPLNIPLLADVTRR (No match)
109 - 118	1179.6415	1178.6342	1178.6295	0.0048	1	RLSEDYGVLK (No match)
110 - 126	1929.0131	1928.0058	1927.9526	0.0532	1	LSEDYGVLKTDEGIAYR (No match)
110 - 126	1929.0131	1928.0058	1927.9526	0.0532	1	LSEDYGVLKTDEGIAYR (Ions score 56)
119 - 126	924.4329	923.4256	923.4348	-0.0092	0	TDEGIAYR (No match)
139 - 149	1211.6818	1210.6745	1210.6669	0.0076	0	QITVNDLPVGR (Ions score 28)
139 - 149	1211.6818	1210.6745	1210.6669	0.0076	0	QITVNDLPVGR (No match)

Mascot: http://www.matrixscience.com/

Spot 779

Protein View

Match to: **gi|4505451** Score: **198** Expect: **3e-015**
neuroblastoma RAS viral (v-ras) oncogene homolog [Homo sapiens]

Nominal mass (M_r): **21501**; Calculated pI value: **5.01**
NCBI BLAST search of gi|4505451 against nr
Unformatted sequence string for pasting into other applications

Taxonomy: Homo sapiens
Links to retrieve other entries containing this sequence from NCBI Entrez:
gi|113205598 from Sus scrofa
gi|148226664 from Bos taurus
gi|73981121 from Canis lupus familiaris
gi|109014495 from Macaca mulatta
gi|114558684 from Pan troglodytes
gi|149708783 from Equus caballus
gi|131883 from Homo sapiens
gi|118573267 from Sus scrofa
gi|20147731 from Homo sapiens
gi|35103 from Homo sapiens
gi|190927 from Homo sapiens
gi|208911 from synthetic construct
gi|13528840 from Homo sapiens
gi|37595069 from Homo sapiens
gi|54696334 from Homo sapiens
gi|54696336 from Homo sapiens
gi|56203367 from Homo sapiens
gi|60656455 from synthetic construct
gi|61357180 from synthetic construct
gi|61357186 from synthetic construct
gi|84322432 from Sus scrofa
gi|119577012 from Homo sapiens
gi|119577013 from Homo sapiens
gi|119577014 from Homo sapiens
gi|119577015 from Homo sapiens
gi|146186548 from Bos taurus

Fixed modifications: Carbamidomethyl (C)
Variable modifications: Oxidation (M)
Cleavage by Trypsin: cuts C-term side of KR unless next residue is P
Sequence Coverage: **25%**

Matched peptides shown in **Bold Red**

```
  1 MTEYKLVVVG AGGVGKSALT IQLIQNHFVD EYDPTIEDSY RKQVVIDGET
 51 CLLDILDTAG QEEYSAMRDQ YMRTGEGFLC VFAINNSKSF ADINLYREQI
101 KRVKDSDDVP MVLVGNKCDL PTRTVDTKQA HELAKSYGIP FIETSAKTRQ
151 GVEDAFYTLV REIRQYRMKK LNSSDDGTQG CMGLPCVVM
```

Residue Number Increasing Mass Decreasing Mass

Start - End	Observed	Mr(expt)	Mr(calc)	Delta	Miss	Sequence
74 - 88	1656.7627	1655.7554	1655.7977	-0.0422	0	TGEGFLCVFAINNSK (No match)
74 - 88	1656.7627	1655.7554	1655.7977	-0.0422	0	TGEGFLCVFAINNSK (Ions score 88)
89 - 97	1098.5459	1097.5386	1097.5505	-0.0119	0	SFADINLYR (No match)
89 - 97	1098.5459	1097.5386	1097.5505	-0.0119	0	SFADINLYR (Ions score 18)
136 - 147	1312.6573	1311.6500	1311.6710	-0.0210	0	SYGIPFIETSAK (No match)
150 - 161	1397.6772	1396.6699	1396.6986	-0.0287	0	QGVEDAFYTLVR (Ions score 45)
150 - 161	1397.6772	1396.6699	1396.6986	-0.0287	0	QGVEDAFYTLVR (No match)

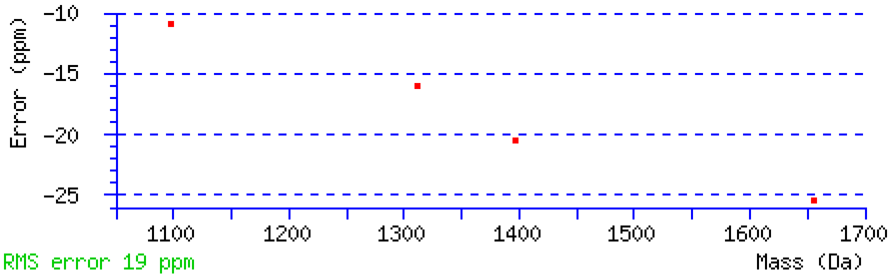

Mascot: http://www.matrixscience.com/

Spot 780

Protein View

Match to: **gi|119627813** Score: **225** Expect: **6.1e-018**
proteasome (prosome, macropain) subunit, beta type, 2, isoform CRA_b [Homo sapiens]

Nominal mass (M_r): **20365;** Calculated pI value: **6.96**
NCBI BLAST search of gi|119627813 against nr
Unformatted sequence string for pasting into other applications

Taxonomy: Homo sapiens

Fixed modifications: Carbamidomethyl (C)
Variable modifications: Oxidation (M)
Cleavage by Trypsin: cuts C-term side of KR unless next residue is P
Sequence Coverage: **39%**

Matched peptides shown in **Bold Red**

```
  1 MEYLIDHDKM FKMSEKILLL CVGEAGDTVQ FAEYIQKNVQ LYKMRNGYEL
 51 SPTAAANFTR RNLADCLRSR TPYHVNLLLA GYDEHEGPAL YYMDYLAALA
101 KAPFAAHGYG AFLTLSILDR YYTPTISRER AVELLRKCLE ELQKRFILNL
151 PTFSVRIIDK NGIHDLDNIS FPKQGS
```

Residue Number Increasing Mass Decreasing Mass

Start - End	Observed	Mr(expt)	Mr(calc)	Delta	Miss	Sequence
46 - 60	1611.7480	1610.7407	1610.7688	-0.0281	0	NGYELSPTAAANFTR (No match)
46 - 60	1611.7480	1610.7407	1610.7688	-0.0281	0	NGYELSPTAAANFTR (Ions score 26)
102 - 120	2020.0240	2019.0167	2019.0577	-0.0410	0	APFAAHGYGAFLTLSILDR (No match)
121 - 128	1000.5019	999.4946	999.5025	-0.0079	0	YYTPTISR (No match)
146 - 156	1306.7305	1305.7232	1305.7444	-0.0212	0	FILNLPTFSVR (Ions score 46)
146 - 156	1306.7305	1305.7232	1305.7444	-0.0212	0	FILNLPTFSVR (No match)
157 - 173	1938.9884	1937.9811	1938.0210	-0.0399	1	IIDKNGIHDLDNISFPK (No match)
157 - 173	1938.9884	1937.9811	1938.0210	-0.0399	1	IIDKNGIHDLDNISFPK (Ions score 81)

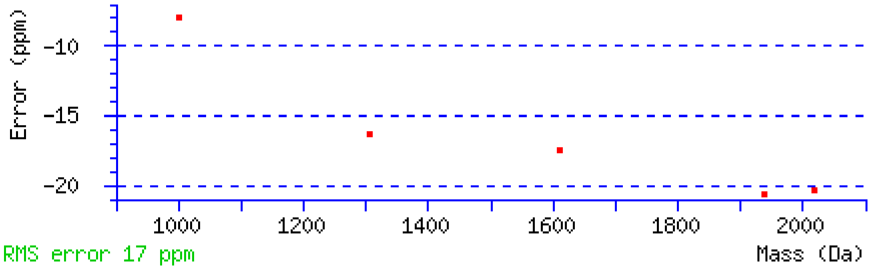

Mascot: http://www.matrixscience.com/

Spot 781

Protein View

Match to: **gi|4261934** Score: **149** Expect: **2.4e-010**
neuropolypeptide h3 [Homo sapiens]

Nominal mass (M_r): **16068**; Calculated pI value: **8.81**
NCBI BLAST search of gi|4261934 against nr
Unformatted sequence string for pasting into other applications

Taxonomy: Homo sapiens

Fixed modifications: Carbamidomethyl (C)
Variable modifications: Oxidation (M)
Cleavage by Trypsin: cuts C-term side of KR unless next residue is P
Sequence Coverage: **24%**

Matched peptides shown in **Bold Red**

```
  1 NRPTSISWDG LDSGKLYTLV LTDPDAPSRK DPKYREWHHF LVVNMKGNDI
 51 SSGTVLSDYV GSGPPKGTGL HRYVWLVYEQ DRPLKCDEPI LSNRSGDHRG
101 KFKVASFRKK YELRAPVAGT CYQAEWDDYV PKLYEQLSGK
```

Residue Number Increasing Mass Decreasing Mass

Start - End	Observed	Mr(expt)	Mr(calc)	Delta	Miss	Sequence	
16 - 29	1560.8101	1559.8028	1559.8195	-0.0167	0	LYTLVLTDPDAPSR	(No match)
16 - 29	1560.8101	1559.8028	1559.8195	-0.0167	0	LYTLVLTDPDAPSR	(Ions score 16)
47 - 66	1949.9207	1948.9134	1948.9377	-0.0243	0	GNDISSGTVLSDYVGSGPPK	(Ions score 113)
47 - 66	1949.9207	1948.9134	1948.9377	-0.0243	0	GNDISSGTVLSDYVGSGPPK	(No match)

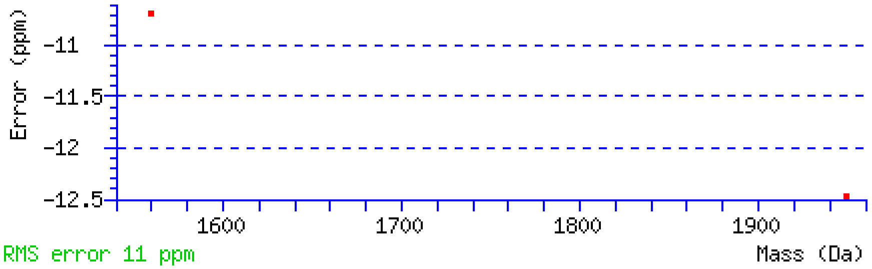

Mascot: http://www.matrixscience.com/

Spot 783

Protein View

Match to: **gi|9956026** Score: **79** Expect: **0.0027**
similar to Homo sapiens mRNA for KIAA0120 gene with GenBank Accession Number D21261.1

Nominal mass (M_r): **24609**; Calculated pI value: **8.41**
NCBI BLAST search of gi|9956026 against nr
Unformatted sequence string for pasting into other applications

Taxonomy: Homo sapiens
Links to retrieve other entries containing this sequence from NCBI Entrez:
gi|55960375 from Homo sapiens
gi|119573147 from Homo sapiens

Fixed modifications: Carbamidomethyl (C)
Variable modifications: Oxidation (M)
Cleavage by Trypsin: cuts C-term side of KR unless next residue is P
Sequence Coverage: **23%**

Matched peptides shown in **Bold Red**

```
  1 MSAFSLALAL VSSPQPPPPI GMANRGPAYG LSREVQQKIE KQYDADLEQI
 51 LIQWITTQCR KDVGRPQPGR ENFQNWLKDG TVLCELINAL YPEGQAPVKK
101 IQASTMAFKQ MEQISQFLQA AERYGINTTD IFQTVDLWEG KNMACVQRTL
151 MNLGGLAVAR DDGLFSGDPN WFPKKSKENP RNFSDNQLQE GKNVIGLQMG
201 TNRGASQAGM TGYGMPRQIL
```

Residue Number Increasing Mass Decreasing Mass

Start - End	Observed	Mr(expt)	Mr(calc)	Delta	Miss	Sequence
110 - 123	1694.7917	1693.7844	1693.8093	-0.0249	0	QMEQISQFLQAAER Oxidation (M) (No match)
110 - 123	1694.7917	1693.7844	1693.8093	-0.0249	0	QMEQISQFLQAAER Oxidation (M) (Ions score 0)
149 - 160	1231.6665	1230.6592	1230.6754	-0.0161	0	TLMNLGGLAVAR Oxidation (M) (No match)
149 - 160	1231.6665	1230.6592	1230.6754	-0.0161	0	TLMNLGGLAVAR Oxidation (M) (No match)
193 - 203	1218.6058	1217.5985	1217.6186	-0.0201	0	NVIGLQMGTNR Oxidation (M) (Ions score 28)
193 - 203	1218.6058	1217.5985	1217.6186	-0.0201	0	NVIGLQMGTNR Oxidation (M) (No match)
204 - 217	1415.5852	1414.5779	1414.5969	-0.0189	0	GASQAGMTGYGMPR 2 Oxidation (M) (No match)

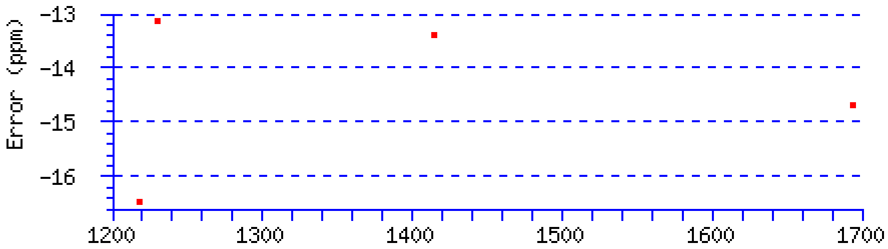

RMS error 14 ppm

Mascot: http://www.matrixscience.com/

Spot 784

Protein View

Match to: **gi|71773201** Score: **91** Expect: **0.00017**
adenine phosphoribosyltransferase isoform b [Homo sapiens]

Nominal mass (M_r): **14605**; Calculated pI value: **6.73**
NCBI BLAST search of gi|71773201 against nr
Unformatted sequence string for pasting into other applications

Taxonomy: Homo sapiens
Links to retrieve other entries containing this sequence from NCBI Entrez:
gi|114664113 from Pan troglodytes
gi|119587165 from Homo sapiens

Fixed modifications: Carbamidomethyl (C)
Variable modifications: Oxidation (M)
Cleavage by Trypsin: cuts C-term side of KR unless next residue is P
Sequence Coverage: **19%**

Matched peptides shown in **Bold Red**

```
  1 MADSELQLVE QRIRSFPDFP TPGVVFRDIS PVLKDPASFR AAIGLLARHL
 51 KATHGGRIDY IAGLDSRGFL FGPSLAQELG LGCVLIRKRG KLPGPTLWAS
101 YSLEYGKAEL EIQKDALEPG QRVVVVDDLL ATGV
```

Residue Number Increasing Mass Decreasing Mass

Start - End	Observed	Mr(expt)	Mr(calc)	Delta	Miss	Sequence	
15 - 27	1465.7629	1464.7556	1464.7401	0.0155	0	SFPDFPTPGVVFR	(Ions score 49)
15 - 27	1465.7629	1464.7556	1464.7401	0.0155	0	SFPDFPTPGVVFR	(No match)
28 - 40	1444.7970	1443.7897	1443.7721	0.0176	1	DISPVLKDPASFR	(Ions score 16)
28 - 40	1444.7970	1443.7897	1443.7721	0.0176	1	DISPVLKDPASFR	(No match)

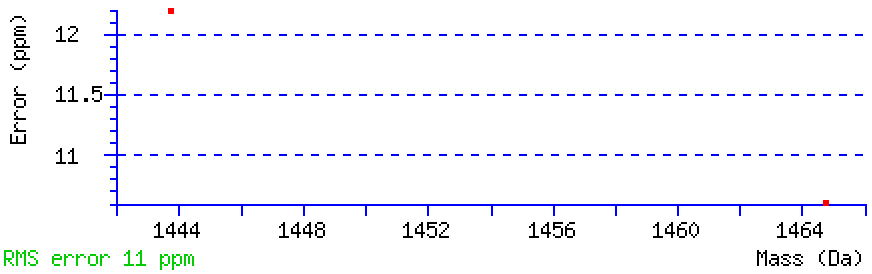

Mascot: http://www.matrixscience.com/

Spot 785

Protein View

Match to: **gi|1942609** Score: **83** Expect: **0.00088**
Chain A, Human Rap1a, Residues 1-167, Double Mutant (E30d,K31e) Complexed With Gppnhp And The Ras-B

Nominal mass (M_r): **19297;** Calculated pI value: **4.63**
NCBI BLAST search of gi|1942609 against nr
Unformatted sequence string for pasting into other applications

Taxonomy: Homo sapiens

Fixed modifications: Carbamidomethyl (C)
Variable modifications: Oxidation (M)
Cleavage by Trypsin: cuts C-term side of KR unless next residue is P
Sequence Coverage: **8%**

Matched peptides shown in **Bold Red**

```
  1 MREYKLVVLG SGGVGKSALT VQFVQGIFVD EYDPTIEDSY RKQVEVDCQQ
 51 CMLEILDTAG TEQFTAMRDL YMKNGQGFAL VYSITAQSTF NDLQDLREQI
101 LRVKDTEDVP MILVGNKCDL EDERVVGKEQ GQNLARQWCN CAFLESSAKS
151 KINVNEIFYD LVRQINR
```

Residue Number Increasing Mass Decreasing Mass

Start - End	Observed	Mr(expt)	Mr(calc)	Delta	Miss	Sequence	
150 - 163	1709.9137	1708.9064	1708.9147	-0.0083	1	SKINVNEIFYDLVR	(No match)
150 - 163	1709.9137	1708.9064	1708.9147	-0.0083	1	SKINVNEIFYDLVR	(No match)
152 - 163	1494.7904	1493.7831	1493.7878	-0.0046	0	INVNEIFYDLVR	(Ions score 57)
152 - 163	1494.7904	1493.7831	1493.7878	-0.0046	0	INVNEIFYDLVR	(No match)

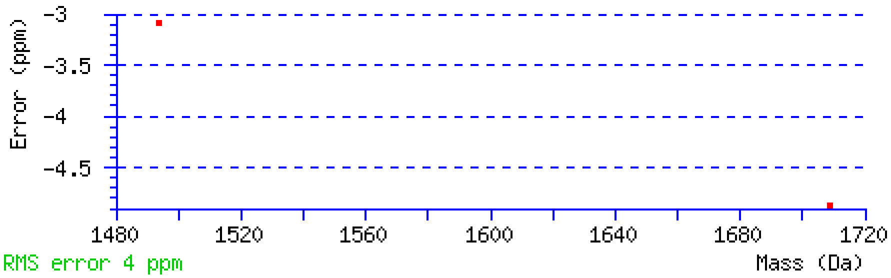

Mascot: http://www.matrixscience.com/

Spot 787

Protein View

Match to: **gi|9956026** Score: **184** Expect: **7.7e-014**
similar to Homo sapiens mRNA for KIAA0120 gene with GenBank Accession Number D21261.1

Nominal mass (M_r): **24609**; Calculated pI value: **8.41**
NCBI BLAST search of gi|9956026 against nr
Unformatted sequence string for pasting into other applications

Taxonomy: Homo sapiens
Links to retrieve other entries containing this sequence from NCBI Entrez:
gi|55960375 from Homo sapiens
gi|119573147 from Homo sapiens

Fixed modifications: Carbamidomethyl (C)
Variable modifications: Oxidation (M)
Cleavage by Trypsin: cuts C-term side of KR unless next residue is P
Sequence Coverage: **36%**

Matched peptides shown in **Bold Red**

```
  1 MSAFSLALAL VSSPQPPPPI GMANRGPAYG LSREVQQKIE KQYDADLEQI
 51 LIQWITTQCR KDVGRPQPGR ENFQNWLKDG TVLCELINAL YPEGQAPVKK
101 IQASTMAFKQ MEQISQFLQA AERYGINTTD IFQTVDLWEG KNMACVQRTL
151 MNLGGLAVAR DDGLFSGDPN WFPKKSKENP RNFSDNQLQE GKNVIGLQMG
201 TNRGASQAGM TGYGMPRQIL
```

Residue Number Increasing Mass Decreasing Mass

Start - End	Observed	Mr(expt)	Mr(calc)	Delta	Miss	Sequence
61 - 70	1109.6135	1108.6062	1108.6101	-0.0039	1	KDVGRPQPGR (No match)
62 - 70	981.5097	980.5024	980.5151	-0.0127	0	DVGRPQPGR (No match)
101 - 109	1012.5015	1011.4942	1011.5059	-0.0117	0	IQASTMAFK Oxidation (M) (No match)
110 - 123	1694.8414	1693.8341	1693.8093	0.0248	0	QMEQISQFLQAAER Oxidation (M) (No match)
110 - 123	1694.8414	1693.8341	1693.8093	0.0248	0	QMEQISQFLQAAER Oxidation (M) (Ions score 42)
149 - 160	1231.6825	1230.6752	1230.6754	-0.0001	0	TLMNLGGLAVAR Oxidation (M) (No match)
149 - 160	1231.6825	1230.6752	1230.6754	-0.0001	0	TLMNLGGLAVAR Oxidation (M) (Ions score 6)
182 - 192	1279.5868	1278.5795	1278.5840	-0.0045	0	NFSDNQLQEGK (No match)
182 - 203	2479.2676	2478.2603	2478.1920	0.0683	1	NFSDNQLQEGKNVIGLQMGTNR Oxidation (M) (No match)

193 - 203	1218.6256	1217.6183	1217.6186	-0.0003	0	**NVIGLQMGTNR**	Oxidation (M) (Ions score 23)
193 - 203	1218.6256	1217.6183	1217.6186	-0.0003	0	**NVIGLQMGTNR**	Oxidation (M) (No match)
204 - 217	1415.6163	1414.6090	1414.5969	0.0122	0	**GASQAGMTGYGMPR**	2 Oxidation (M) (No match)
204 - 217	1415.6163	1414.6090	1414.5969	0.0122	0	**GASQAGMTGYGMPR**	2 Oxidation (M) (Ions score 17)

Mascot: http://www.matrixscience.com/

Spot 790

Protein View

Match to: **gi|20151145** Score: **307** Expect: **3.8e-026**
Chain B, Crystal Structure Of The DhPH FRAGMENT OF MURINE DBS IN Complex With The Placental Isoform

Nominal mass (M_r): **21189**; Calculated pI value: **6.16**
NCBI BLAST search of gi|20151145 against nr
Unformatted sequence string for pasting into other applications

Taxonomy: Mus musculus
Links to retrieve other entries containing this sequence from NCBI Entrez:
gi|20151147 from Mus musculus
gi|20151149 from Mus musculus
gi|20151151 from Mus musculus
gi|21465836 from Homo sapiens
gi|21465838 from Homo sapiens

Fixed modifications: Carbamidomethyl (C)
Variable modifications: Oxidation (M)
Cleavage by Trypsin: cuts C-term side of KR unless next residue is P
Sequence Coverage: **26%**

Matched peptides shown in **Bold Red**

```
  1 MQTIKCVVVG DGAVGKTCLL ISYTTNKFPS EYVPTVFDNY AVTVMIGGEP
 51 YTLGLFDTAG QEDYDRLRPL SYPQTDVFLV CFSVVSPSSF ENVKEKWVPE
101 ITHHCPKTPF LLVGTQIDLR DDPSTIEKLA KNKQKPITPE TAEKLARDLK
151 AVKYVECSAL TQKGLKNVFD EAILAALEPP EPKKSRRS
```

Residue Number Increasing Mass Decreasing Mass

Start - End	Observed	Mr(expt)	Mr(calc)	Delta	Miss	Sequence
97 - 107	1403.6674	1402.6601	1402.6816	-0.0214	0	WVPEITHHCPK (No match)
97 - 107	1403.6674	1402.6601	1402.6816	-0.0214	0	WVPEITHHCPK (Ions score 73)
108 - 120	1472.8226	1471.8153	1471.8398	-0.0245	0	TPFLLVGTQIDLR (No match)
108 - 120	1472.8226	1471.8153	1471.8398	-0.0245	0	TPFLLVGTQIDLR (Ions score 52)
108 - 128	2358.2124	2357.2051	2357.2478	-0.0427	1	TPFLLVGTQIDLRDDPSTIEK (Ions score 41)
108 - 128	2358.2124	2357.2051	2357.2478	-0.0427	1	TPFLLVGTQIDLRDDPSTIEK (No match)
167 - 183	1852.9363	1851.9290	1851.9617	-0.0327	0	NVFDEAILAALEPPEPK (Ions score 68)

167 - 183   1852.9363   1851.9290   1851.9617   -0.0327   0   NVFDEAILAALEPPEPK   (No match)

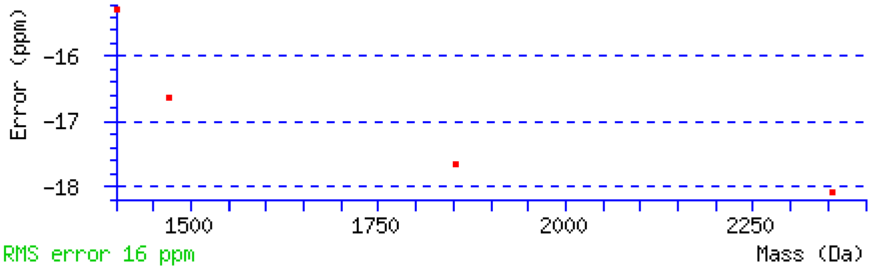

Mascot: http://www.matrixscience.com/

Spot 794

Protein View

Match to: **gi|45786143** Score: **346** Expect: **4.8e-030**
Lectin, galactoside-binding, soluble, 3 [Homo sapiens]

Nominal mass (M_r): **26238**; Calculated pI value: **8.58**
NCBI BLAST search of gi|45786143 against nr
Unformatted sequence string for pasting into other applications

Taxonomy: Homo sapiens

Fixed modifications: Carbamidomethyl (C)
Variable modifications: Oxidation (M)
Cleavage by Trypsin: cuts C-term side of KR unless next residue is P
Sequence Coverage: **31%**

Matched peptides shown in **Bold Red**

```
  1 MADNFSLHDA LSGSGNPNPQ GWPGAWGNQP AGAGGYPGAS YPGAYPGQAP
 51 PGAYPGQAPP GAYHGAPGAY PGAPAPGVYP GPPSGPGAYP SSGQPSAPGA
101 YPATGPYGAP AGPLIVPYNL PLPGGVVPRM LITILGTVKP NANRIALDFQ
151 RGNDVAFHFN PRFNENNRRV IVCNTKLDNN WGREERQSVF PFESGKPFKI
201 HVLVEPDHFK VAVNDAHLLQ YNHRVKKLNE ISKLGISGDI DLTSASYTMI
251
```

Residue Number Increasing Mass Decreasing Mass

Start - End	Observed	Mr(expt)	Mr(calc)	Delta	Miss	Sequence
130 - 144	1656.8958	1655.8885	1655.9392	-0.0507	0	MLITILGTVKPNANR Oxidation (M) (No match)
145 - 151	862.4620	861.4547	861.4708	-0.0161	0	IALDFQR (No match)
152 - 162	1273.5719	1272.5646	1272.5999	-0.0353	0	GNDVAFHFNPR (Ions score 73)
152 - 162	1273.5719	1272.5646	1272.5999	-0.0353	0	GNDVAFHFNPR (No match)
163 - 169	949.4328	948.4255	948.4525	-0.0270	1	FNENNRR (No match)
187 - 199	1497.7240	1496.7167	1496.7663	-0.0496	0	QSVFPFESGKPFK (No match)
187 - 199	1497.7240	1496.7167	1496.7663	-0.0496	0	QSVFPFESGKPFK (Ions score 57)
200 - 210	1333.6840	1332.6767	1332.7190	-0.0422	0	IHVLVEPDHFK (No match)
200 - 210	1333.6840	1332.6767	1332.7190	-0.0422	0	IHVLVEPDHFK (Ions score 17)
211 - 224	1649.8027	1648.7954	1648.8433	-0.0479	0	VAVNDAHLLQYNHR (No match)
211 - 224	1649.8027	1648.7954	1648.8433	-0.0479	0	VAVNDAHLLQYNHR (Ions score 89)

Spot 795

Protein View

Match to: **gi|32187319** Score: **83** Expect: **0.00094**
SHUJUN-1 [Homo sapiens]

Nominal mass (M_r): **17047;** Calculated pI value: **4.21**
NCBI BLAST search of gi|32187319 against nr
Unformatted sequence string for pasting into other applications

Taxonomy: Homo sapiens

Fixed modifications: Carbamidomethyl (C)
Variable modifications: Oxidation (M)
Cleavage by Trypsin: cuts C-term side of KR unless next residue is P
Sequence Coverage: **14%**

Matched peptides shown in **Bold Red**

```
  1 MFDQSQIQEF KEAFNMIDQN RDGFIDKEDL HDMLASLGKN PTDAYLDVMM
 51 NEAPGPINFT MFLTTFGEKL NGTDPEDVIG NAFACFDEEA TGTIQEDYLR
101 ELLTTMGDRF TDEEVDELYR EAPIDKKGNF NYIEFTRILK HGAKDKDD
```

Residue Number Increasing Mass Decreasing Mass

Start - End	Observed	Mr(expt)	Mr(calc)	Delta	Miss	Sequence	
110 - 120	1415.6050	1414.5977	1414.6252	-0.0275	0	FTDEEVDELYR	(No match)
110 - 120	1415.6050	1414.5977	1414.6252	-0.0275	0	FTDEEVDELYR	(Ions score 28)
128 - 137	1260.5745	1259.5672	1259.5934	-0.0262	0	GNFNYIEFTR	(Ions score 27)
128 - 137	1260.5745	1259.5672	1259.5934	-0.0262	0	GNFNYIEFTR	(No match)

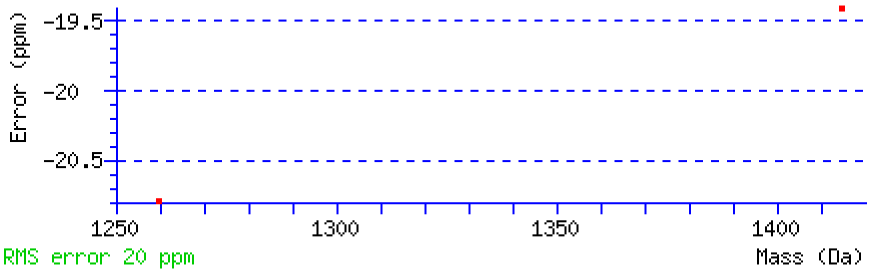

RMS error 20 ppm

Mascot: http://www.matrixscience.com/

Spot 797

Protein View

Match to: **gi|55665352** Score: **143** Expect: **9.6e-010**
ribosomal protein L11 [Homo sapiens]

Nominal mass (M_r): **15085;** Calculated pI value: **9.03**
NCBI BLAST search of gi|55665352 against nr
Unformatted sequence string for pasting into other applications

Taxonomy: Homo sapiens

Fixed modifications: Carbamidomethyl (C)
Variable modifications: Oxidation (M)
Cleavage by Trypsin: cuts C-term side of KR unless next residue is P
Sequence Coverage: **32%**

Matched peptides shown in **Bold Red**

```
  1 QDQGEKENPM RELRIRKLCL NICVGESGDR LTRAAKVLEQ LTGQTPVFSK
 51 ARYTVRSFGI RRNEKIAVHC TVRGAKAEEI LEKGLKVREY ELRKNNFSDT
101 GNFGFGIQEH IDLGIKYDPS IGIYGLDFYV V
```

Residue Number Increasing Mass Decreasing Mass

Start - End	Observed	Mr(expt)	Mr(calc)	Delta	Miss	Sequence
17 - 30	1620.8215	1619.8142	1619.7759	0.0383	1	KLCLNICVGESGDR (No match)
17 - 30	1620.8215	1619.8142	1619.7759	0.0383	1	KLCLNICVGESGDR (No match)
18 - 30	1492.7269	1491.7196	1491.6809	0.0387	0	LCLNICVGESGDR (Ions score 16)
18 - 30	1492.7269	1491.7196	1491.6809	0.0387	0	LCLNICVGESGDR (No match)
37 - 50	1546.8770	1545.8697	1545.8402	0.0295	0	VLEQLTGQTPVFSK (No match)
37 - 50	1546.8770	1545.8697	1545.8402	0.0295	0	VLEQLTGQTPVFSK (Ions score 53)
66 - 73	955.5516	954.5443	954.5069	0.0374	0	IAVHCTVR (No match)
87 - 93	964.5544	963.5471	963.5137	0.0334	1	VREYELR (No match)

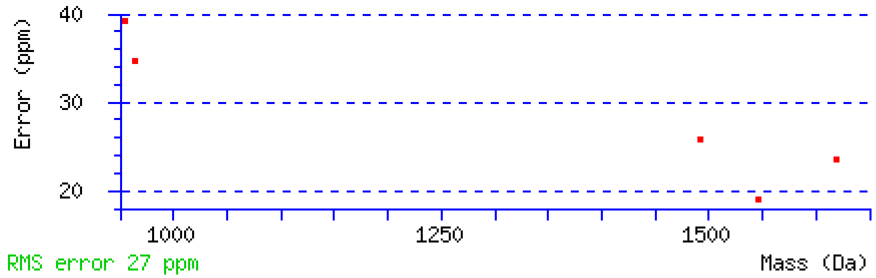

Mascot: http://www.matrixscience.com/

Spot 798

Protein View

Match to: **gi|5031595** Score: **355** Expect: **6.1e-031**
actin related protein 2/3 complex subunit 4 isoform a [Homo sapiens]

Nominal mass (M_r): **19768**; Calculated pI value: **8.53**
NCBI BLAST search of gi|5031595 against nr
Unformatted sequence string for pasting into other applications

Taxonomy: Homo sapiens
Links to retrieve other entries containing this sequence from NCBI Entrez:
gi|13386054 from Mus musculus
gi|115495705 from Bos taurus
gi|57100910 from Canis lupus familiaris
gi|38372625 from Homo sapiens
gi|38372626 from Mus musculus
gi|119361249 from Bos taurus
gi|17943204 from Bos taurus
gi|56966177 from Bos taurus
gi|56966197 from Bos taurus
gi|149243014 from Bos taurus
gi|149243029 from Bos taurus
gi|149243036 from Bos taurus
gi|149243043 from Bos taurus
gi|149243050 from Bos taurus
gi|149243057 from Bos taurus
gi|149243064 from Bos taurus
gi|2282040 from Homo sapiens
gi|2444287 from Homo sapiens
gi|12857511 from Mus musculus
gi|13397915 from Mus musculus
gi|26333123 from Mus musculus
gi|26346200 from Mus musculus
gi|74142379 from Mus musculus
gi|74151291 from Mus musculus
gi|74177948 from Mus musculus
gi|74193742 from Mus musculus
gi|74227332 from Mus musculus
gi|109939945 from Bos taurus
gi|119584405 from Homo sapiens
gi|148667042 from Mus musculus
gi|149036910 from Rattus norvegicus
gi|157169642 (no taxonomy information for this entry)

gi|160904173 (no taxonomy information for this entry)

Fixed modifications: Carbamidomethyl (C)
Variable modifications: Oxidation (M)
Cleavage by Trypsin: cuts C-term side of KR unless next residue is P
Sequence Coverage: **52%**

Matched peptides shown in **Bold Red**

```
  1 MTATLRPYLS AVRATLQAAL CLENFSSQVV ERHNKPEVEV RSSKELLLQP
 51 VTISRNEKEK VLIEGSINSV RVSIAVKQAD EIEKILCHKF MRFMMMRAEN
101 FFILRRKPVE GYDISFLITN FHTEQMYKHK LVDFVIHFME EIDKEISEMK
151 LSVNARARIV AEEFLKNF
```

Residue Number Increasing Mass Decreasing Mass

Start - End	Observed	Mr(expt)	Mr(calc)	Delta	Miss	Sequence
14 - 32	2136.0300	2135.0227	2135.0680	-0.0453	0	ATLQAALCLENFSSQVVER (No match)
14 - 32	2136.0300	2135.0227	2135.0680	-0.0453	0	ATLQAALCLENFSSQVVER (Ions score 105)
33 - 41	1107.5797	1106.5724	1106.5832	-0.0108	0	HNKPEVEVR (No match)
45 - 55	1268.7386	1267.7313	1267.7499	-0.0186	0	ELLLQPVTISR (No match)
45 - 55	1268.7386	1267.7313	1267.7499	-0.0186	0	ELLLQPVTISR (Ions score 52)
61 - 71	1186.6643	1185.6570	1185.6717	-0.0146	0	VLIEGSINSVR (No match)
61 - 71	1186.6643	1185.6570	1185.6717	-0.0146	0	VLIEGSINSVR (Ions score 37)
98 - 105	1009.5356	1008.5283	1008.5392	-0.0109	0	AENFFILR (Ions score 49)
98 - 105	1009.5356	1008.5283	1008.5392	-0.0109	0	AENFFILR (No match)
107 - 128	2676.2661	2675.2588	2675.2941	-0.0352	0	KPVEGYDISFLITNFHTEQMYK Oxidation (M) (No match)
159 - 166	948.5241	947.5168	947.5327	-0.0159	0	IVAEEFLK (No match)

Mascot: http://www.matrixscience.com/

Spot 799

Protein View

Match to: **gi|119618319** Score: **120** Expect: **1.9e-007**
actin related protein 2/3 complex, subunit 3, 21kDa, isoform CRA_b [Homo sapiens]

Nominal mass (M_r): **19108**; Calculated pI value: **8.65**
NCBI BLAST search of gi|119618319 against nr
Unformatted sequence string for pasting into other applications

Taxonomy: Homo sapiens

Fixed modifications: Carbamidomethyl (C)
Variable modifications: Oxidation (M)
Cleavage by Trypsin: cuts C-term side of KR unless next residue is P
Sequence Coverage: **24%**

Matched peptides shown in **Bold Red**

```
  1 MPAYHSSLMD PDTKLIGNMA LLPIRSQFKG PAPRETKDTD IVDEAIYYFK
 51 ANVFFKNYEI KNEADRTLIY ITLYISECLK KLQKCNSKSQ GEKEMYTLGI
101 TNFPIPGEPG FPLNAIYAKP ANKQEDEVMR AYLQQLRQET GLRLCEKVFD
151 PQNDKPSKAG RSGSRL
```

Residue Number Increasing Mass Decreasing Mass

Start - End	Observed	Mr(expt)	Mr(calc)	Delta	Miss	Sequence
15 - 25	1226.7511	1225.7438	1225.7216	0.0222	0	LIGNMALLPIR Oxidation (M) (Ions score 35)
15 - 25	1226.7511	1225.7438	1225.7216	0.0222	0	LIGNMALLPIR Oxidation (M) (No match)
26 - 34	987.5489	986.5416	986.5297	0.0119	1	SQFKGPAPR (No match)
38 - 50	1591.8024	1590.7951	1590.7453	0.0498	0	DTDIVDEAIYYFK (Ions score 48)
38 - 50	1591.8024	1590.7951	1590.7453	0.0498	0	DTDIVDEAIYYFK (No match)
124 - 130	922.3931	921.3858	921.3862	-0.0003	0	QEDEVMR Oxidation (M) (No match)

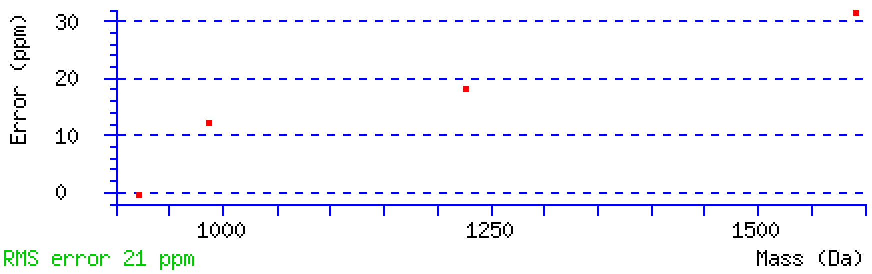

Mascot: http://www.matrixscience.com/

Spot 800

Protein View

Match to: **gi|1310882** Score: **447** Expect: **3.8e-040**
Chain A, Cyclophilin B Complexed With [d-(Cholinylester)ser8]- Cyclosporin

Nominal mass (M_r): **19705**; Calculated pI value: **9.18**
NCBI BLAST search of gi|1310882 against nr
Unformatted sequence string for pasting into other applications

Taxonomy: Homo sapiens

Fixed modifications: Carbamidomethyl (C)
Variable modifications: Oxidation (M)
Cleavage by Trypsin: cuts C-term side of KR unless next residue is P
Sequence Coverage: **69%**

Matched peptides shown in **Bold Red**

```
  1 GPKVTVKVYF DLRIGDEDVG RVIFGLFGKT VPKTVDNFVA LATGEKGFGY
 51 KNSKFHRVIK DFMIQGGDFT RGDGTGGKSI YGERFPDENF KLKHYGPGWV
101 SMANAGKDTN GSQFFITTVK TAWLDGKHVV FGKVLEGMEV VRKVESTKTD
151 SRDKPLKDVI IADCGKIEVE KPFAIAKE
```

Residue Number Increasing Mass Decreasing Mass

Start - End	Observed	Mr(expt)	Mr(calc)	Delta	Miss	Sequence
8 - 13	812.4208	811.4135	811.4228	-0.0093	0	VYFDLR (No match)
14 - 21	860.4009	859.3936	859.4035	-0.0099	0	IGDEDVGR (No match)
14 - 29	1721.8929	1720.8856	1720.9147	-0.0291	1	IGDEDVGRVIFGLFGK (No match)
22 - 29	880.5113	879.5040	879.5217	-0.0177	0	VIFGLFGK (No match)
34 - 46	1364.6780	1363.6707	1363.6983	-0.0275	0	TVDNFVALATGEK (No match)
58 - 71	1626.8030	1625.7957	1625.8235	-0.0278	1	VIKDFMIQGGDFTR (Ions score 50)
58 - 71	1626.8030	1625.7957	1625.8235	-0.0278	1	VIKDFMIQGGDFTR (No match)
58 - 71	1642.7944	1641.7871	1641.8184	-0.0313	1	VIKDFMIQGGDFTR Oxidation (M) (No match)
58 - 71	1642.7944	1641.7871	1641.8184	-0.0313	1	VIKDFMIQGGDFTR Oxidation (M) (Ions score 57)
61 - 71	1286.5640	1285.5567	1285.5761	-0.0194	0	DFMIQGGDFTR (No match)
61 - 71	1302.5541	1301.5468	1301.5710	-0.0242	0	DFMIQGGDFTR Oxidation (M) (No match)
61 - 71	1302.5541	1301.5468	1301.5710	-0.0242	0	DFMIQGGDFTR Oxidation (M) (Ions score 21)
79 - 91	1601.7330	1600.7257	1600.7521	-0.0264	1	SIYGERFPDENFK (No match)

79 - 91	1601.7330	1600.7257	1600.7521	-0.0264	1	**SIYGERFPDENFK** (Ions score 43)
85 - 91	896.4009	895.3936	895.4075	-0.0139	0	**FPDENFK** (No match)
94 - 107	1490.6550	1489.6477	1489.6772	-0.0294	0	**HYGPGWVSMANAGK** Oxidation (M) (No match)
108 - 120	1457.6963	1456.6890	1456.7198	-0.0307	0	**DTNGSQFFITTVK** (Ions score 81)
108 - 120	1457.6963	1456.6890	1456.7198	-0.0307	0	**DTNGSQFFITTVK** (No match)
134 - 142	1031.5457	1030.5384	1030.5481	-0.0096	0	**VLEGMEVVR** (No match)
134 - 142	1047.5305	1046.5232	1046.5430	-0.0197	0	**VLEGMEVVR** Oxidation (M) (No match)
153 - 166	1571.8160	1570.8087	1570.8388	-0.0301	1	**DKPLKDVIIADCGK** (No match)
167 - 177	1244.7019	1243.6946	1243.7175	-0.0229	0	**IEVEKPFAIAK** (No match)
167 - 178	1373.7400	1372.7327	1372.7601	-0.0274	1	**IEVEKPFAIAKE** (No match)

Mascot: http://www.matrixscience.com/

Spot 803

Protein View

Match to: **gi|66392203** Score: **328** Expect: **3e-028**
NME1-NME2 protein [Homo sapiens]

Nominal mass (M_r): **30346**; Calculated pI value: **9.06**
NCBI BLAST search of gi|66392203 against nr
Unformatted sequence string for pasting into other applications

Taxonomy: Homo sapiens
Links to retrieve other entries containing this sequence from NCBI Entrez:
gi|73746297 from Homo sapiens
gi|124376250 from Homo sapiens
gi|124376672 from Homo sapiens

Fixed modifications: Carbamidomethyl (C)
Variable modifications: Oxidation (M)
Cleavage by Trypsin: cuts C-term side of KR unless next residue is P
Sequence Coverage: **41%**

Matched peptides shown in **Bold Red**

```
  1 MANCERTFIA IKPDGVQRGL VGEIIKRFEQ KGFRLVGLKF MQASEDLLKE
 51 HYVDLKDRPF FAGLVKYMHS GPVVAMVWEG LNVVKTGRVM LGETNPADSK
101 PGTIRGDFCI QVGRTMANLE RTFIAIKPDG VQRGLVGEII KRFEQKGFRL
151 VAMKFLRASE EHLKQHYIDL KDRPFFPGLV KYMNSGPVVA MVWEGLNVVK
201 TGRVMLGETN PADSKPGTIR GDFCIQVGRN IIHGSDSVKS AEKEISLWFK
251 PEELVDYKSC AHDWVYE
```

Residue Number Increasing Mass Decreasing Mass

Start - End	Observed	Mr(expt)	Mr(calc)	Delta	Miss	Sequence
7 - 18	1344.7417	1343.7344	1343.7561	-0.0216	0	TFIAIKPDGVQR (Ions score 32)
19 - 27	984.6122	983.6049	983.6127	-0.0078	1	GLVGEIIKR (Ions score 19)
50 - 66	2034.0328	2033.0255	2033.0733	-0.0478	1	EHYVDLKDRPFFAGLVK (No match)
57 - 66	1149.6250	1148.6177	1148.6342	-0.0164	0	DRPFFAGLVK (Ions score 17)
57 - 66	1149.6250	1148.6177	1148.6342	-0.0164	0	DRPFFAGLVK (No match)
89 - 105	1801.8721	1800.8648	1800.9039	-0.0391	0	VMLGETNPADSKPGTIR Oxidation (M) (Ions score 73)
106 - 114	1051.4843	1050.4770	1050.4916	-0.0146	0	GDFCIQVGR (Ions score 22)

122 - 133	1344.7417	1343.7344	1343.7561	-0.0216	0	**TFIAIKPDGVQR** (No match)
134 - 142	984.6122	983.6049	983.6127	-0.0078	1	**GLVGEIIKR** (No match)
204 - 220	1785.8801	1784.8728	1784.9090	-0.0362	0	**VMLGETNPADSKPGTIR** (No match)
204 - 220	1801.8721	1800.8648	1800.9039	-0.0391	0	**VMLGETNPADSKPGTIR** Oxidation (M) (No match)
221 - 229	1051.4843	1050.4770	1050.4916	-0.0146	0	**GDFCIQVGR** (No match)

Mascot: http://www.matrixscience.com/

Spot 804

Protein View

Match to: **gi|33150554** Score: **136** Expect: **4.8e-009**
ARC16-2 [Homo sapiens]

Nominal mass (M_r): **16891;** Calculated pI value: **7.98**
NCBI BLAST search of gi|33150554 against nr
Unformatted sequence string for pasting into other applications

Taxonomy: Homo sapiens

Fixed modifications: Carbamidomethyl (C)
Variable modifications: Oxidation (M)
Cleavage by Trypsin: cuts C-term side of KR unless next residue is P
Sequence Coverage: **34%**

Matched peptides shown in **Bold Red**

```
  1 MARNTLSSRF RRVDIDEFDE NKFVDEQEEA AAAARTGPDP SEVDGLLRQG
 51 DMLRAFHAAL RNSPVNTKNQ AVKERAQGVV LKVLTNFKSS EIEQAVQSLD
101 RNGVDLLMKY IYKGFEKPTE NSSAVLLQWH EKALAVGGLG SIIRVLTARK
151 TV
```

Residue Number Increasing Mass Decreasing Mass

Start - End	Observed	Mr(expt)	Mr(calc)	Delta	Miss	Sequence
89 - 101	1461.6948	1460.6875	1460.7106	-0.0231	0	SSEIEQAVQSLDR (No match)
89 - 101	1461.6948	1460.6875	1460.7106	-0.0231	0	SSEIEQAVQSLDR (Ions score 42)
89 - 109	2348.1265	2347.1192	2347.1689	-0.0496	1	SSEIEQAVQSLDRNGVDLLMK Oxidation (M) (Ions score 3)
89 - 109	2348.1265	2347.1192	2347.1689	-0.0496	1	SSEIEQAVQSLDRNGVDLLMK Oxidation (M) (No match)
114 - 132	2200.0598	2199.0525	2199.0959	-0.0434	0	GFEKPTENSSAVLLQWHEK (No match)
133 - 144	1126.6815	1125.6742	1125.6869	-0.0127	0	ALAVGGLGSIIR (Ions score 30)
133 - 144	1126.6815	1125.6742	1125.6869	-0.0127	0	ALAVGGLGSIIR (No match)

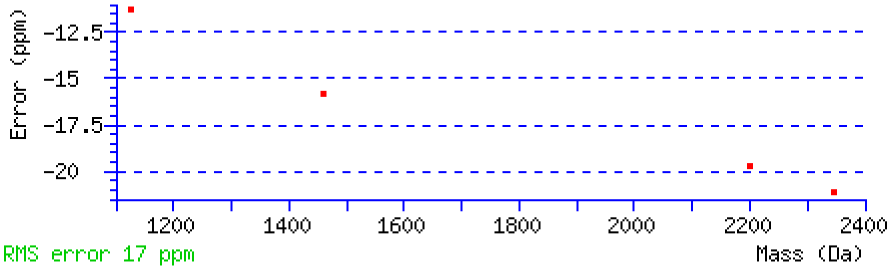

RMS error 17 ppm

Mascot: http://www.matrixscience.com/

Spot 805

Protein View

Match to: **gi|23503040** Score: **116** Expect: **4.8e-007**
Protein ARMET precursor (Mesencephalic astrocyte-derived neurotrophic factor) (MANF) (Arginine-rich

Nominal mass (M_r): **20700;** Calculated pI value: **8.70**
NCBI BLAST search of gi|23503040 against nr
Unformatted sequence string for pasting into other applications

Taxonomy: Homo sapiens

Fixed modifications: Carbamidomethyl (C)
Variable modifications: Oxidation (M)
Cleavage by Trypsin: cuts C-term side of KR unless next residue is P
Sequence Coverage: **26%**

Matched peptides shown in **Bold Red**

```
  1 MWATQGLAVA LALSVLPGSR ALRPGDCEVC ISYLGRFYQD LKDRDVTFSP
 51 ATIENELIKF CREARGKENR LCYYIGATDD AATKIINEVS KPLAHHIPVE
101 KICEKLKKKD SQICELKYDK QIDLSTVDLK KLRVKELKKI LDDWGETCKG
151 CAEKSDYIRK INELMPKYAP KAASARTDL
```

Residue Number Increasing Mass Decreasing Mass

Start - End	Observed	Mr(expt)	Mr(calc)	Delta	Miss	Sequence
43 - 59	1947.9365	1946.9292	1946.9949	-0.0656	1	DRDVTFSPATIENELIK (No match)
43 - 59	1947.9365	1946.9292	1946.9949	-0.0656	1	DRDVTFSPATIENELIK (No match)
71 - 84	1561.6621	1560.6548	1560.7130	-0.0581	0	LCYYIGATDDAATK (No match)
71 - 84	1561.6621	1560.6548	1560.7130	-0.0581	0	LCYYIGATDDAATK (Ions score 21)
85 - 101	1924.0347	1923.0274	1923.0941	-0.0667	0	IINEVSKPLAHHIPVEK (Ions score 50)
85 - 101	1924.0347	1923.0274	1923.0941	-0.0667	0	IINEVSKPLAHHIPVEK (No match)

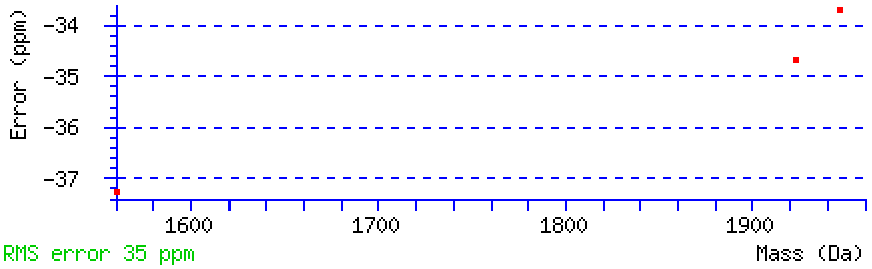

Mascot: http://www.matrixscience.com/

Spot 806

Protein View

Match to: **gi|4507149** Score: **181** Expect: **1.5e-013**
superoxide dismutase 1, soluble [Homo sapiens]

Nominal mass (M_r): **16154;** Calculated pI value: **5.70**
NCBI BLAST search of gi|4507149 against nr
Unformatted sequence string for pasting into other applications

Taxonomy: Homo sapiens
Links to retrieve other entries containing this sequence from NCBI Entrez:
gi|57113939 from Pan troglodytes
gi|134611 from Homo sapiens
gi|38503278 from Pan troglodytes
gi|36542 from Homo sapiens
gi|1237407 from Homo sapiens
gi|12654417 from Homo sapiens
gi|16356659 from Homo sapiens
gi|23503510 from Pan troglodytes
gi|30582191 from Homo sapiens
gi|38489880 from Homo sapiens
gi|47496657 from Homo sapiens
gi|49456443 from Homo sapiens
gi|56157780 from Homo sapiens
gi|60655119 from synthetic construct
gi|60655121 from synthetic construct
gi|60821897 from synthetic construct
gi|119630294 from Homo sapiens
gi|119630295 from Homo sapiens
gi|119712141 from Homo sapiens

Fixed modifications: Carbamidomethyl (C)
Variable modifications: Oxidation (M)
Cleavage by Trypsin: cuts C-term side of KR unless next residue is P
Sequence Coverage: **12%**

Matched peptides shown in **Bold Red**

1 MATK**AVCVLK GDGPVQGIIN FEQK**ESNGPV KVWGSIKGLT EGLHGFHVHE
51 FGDNTAGCTS AGPHFNPLSR KHGGPKDEER HVGDLGNVTA DKDGVADVSI
101 EDSVISLSGD HCIIGRTLVV HEKADDLGKG GNEESTKTGN AGSRLACGVI
151 GIAQ

Residue Number Increasing Mass Decreasing Mass

Start - End	Observed	Mr(expt)	Mr(calc)	Delta	Miss	Sequence
5 - 24	2172.1096	2171.1023	2171.1408	-0.0384	1	AVCVLKGDGPVQGIINFEQK (No match)
5 - 24	2172.1096	2171.1023	2171.1408	-0.0384	1	AVCVLKGDGPVQGIINFEQK (Ions score 62)
11 - 24	1501.7339	1500.7266	1500.7572	-0.0305	0	GDGPVQGIINFEQK (Ions score 88)
11 - 24	1501.7339	1500.7266	1500.7572	-0.0305	0	GDGPVQGIINFEQK (No match)

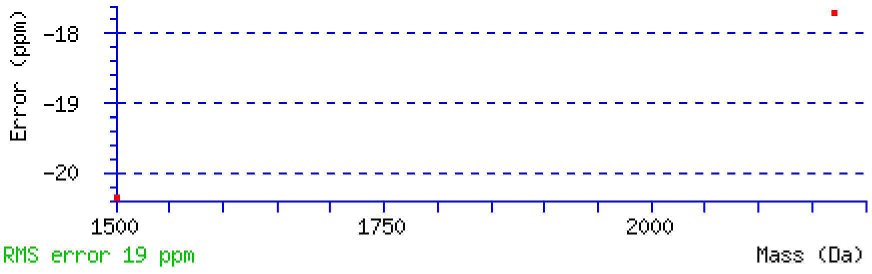

Mascot: http://www.matrixscience.com/

Spot 807

Protein View

Match to: **gi|119594857** Score: **205** Expect: **6.1e-016**
cofilin 1 (non-muscle), isoform CRA_c [Homo sapiens]

Nominal mass (M_r): **15877**; Calculated pI value: **8.54**
NCBI BLAST search of gi|119594857 against nr
Unformatted sequence string for pasting into other applications

Taxonomy: Homo sapiens

Fixed modifications: Carbamidomethyl (C)
Variable modifications: Oxidation (M)
Cleavage by Trypsin: cuts C-term side of KR unless next residue is P
Sequence Coverage: **32%**

Matched peptides shown in **Bold Red**

```
  1 MKVRKSSTPE EVKKRKKAVL FCLSEDKKNI ILEEGKEILV GDVGQTVDDP
 51 YATFVKMLPD KDCRYALYDA TYETKESKKE DLVFIFWAPE SAPLKSKMIY
101 ASSKDAIKKK LTGIKHELQA NCYEEVKDRC TLAEKLG
```

Residue Number Increasing Mass Decreasing Mass

Start - End	Observed	Mr(expt)	Mr(calc)	Delta	Miss	Sequence
18 - 28	1309.6676	1308.6603	1308.6747	-0.0144	1	AVLFCLSEDKK (Ions score 22)
18 - 28	1309.6676	1308.6603	1308.6747	-0.0144	1	AVLFCLSEDKK (No match)
57 - 64	1034.4694	1033.4621	1033.4685	-0.0064	1	MLPDKDCR (No match)
57 - 64	1050.4583	1049.4510	1049.4634	-0.0124	1	MLPDKDCR Oxidation (M) (No match)
57 - 64	1050.4583	1049.4510	1049.4634	-0.0124	1	MLPDKDCR Oxidation (M) (No match)
65 - 75	1337.6084	1336.6011	1336.6187	-0.0175	0	YALYDATYETK (Ions score 50)
65 - 75	1337.6084	1336.6011	1336.6187	-0.0175	0	YALYDATYETK (No match)
116 - 129	1790.7920	1789.7847	1789.8053	-0.0206	1	HELQANCYEEVKDR (No match)
116 - 129	1790.7920	1789.7847	1789.8053	-0.0206	1	HELQANCYEEVKDR (Ions score 77)

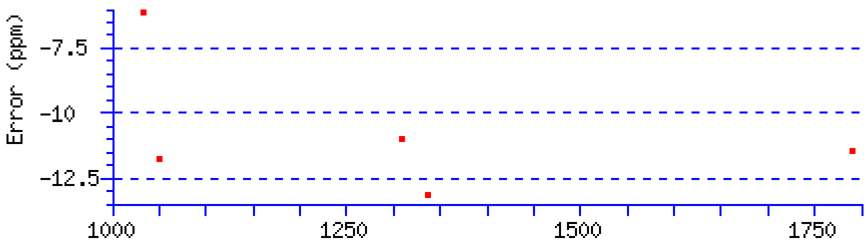

RMS error 11 ppm

Mascot: http://www.matrixscience.com/

Spot 808

Protein View

Match to: **gi|119594857** Score: **157** Expect: **3.8e-011**
cofilin 1 (non-muscle), isoform CRA_c [Homo sapiens]

Nominal mass (M_r): **15877**; Calculated pI value: **8.54**
NCBI BLAST search of gi|119594857 against nr
Unformatted sequence string for pasting into other applications

Taxonomy: Homo sapiens

Fixed modifications: Carbamidomethyl (C)
Variable modifications: Oxidation (M)
Cleavage by Trypsin: cuts C-term side of KR unless next residue is P
Sequence Coverage: **32%**

Matched peptides shown in **Bold Red**

```
  1 MKVRKSSTPE EVKKRKKAVL FCLSEDKKNI ILEEGKEILV GDVGQTVDDP
 51 YATFVKMLPD KDCRYALYDA TYETKESKKE DLVFIFWAPE SAPLKSKMIY
101 ASSKDAIKKK LTGIKHELQA NCYEEVKDRC TLAEKLG
```

Residue Number Increasing Mass Decreasing Mass

Start - End	Observed	Mr(expt)	Mr(calc)	Delta	Miss	Sequence
18 - 28	1309.6969	1308.6896	1308.6747	0.0149	1	AVLFCLSEDKK (No match)
57 - 64	1050.4894	1049.4821	1049.4634	0.0187	1	MLPDKDCR Oxidation (M) (No match)
65 - 75	1337.6393	1336.6320	1336.6187	0.0134	0	YALYDATYETK (No match)
65 - 75	1337.6393	1336.6320	1336.6187	0.0134	0	YALYDATYETK (Ions score 28)
116 - 129	1790.8236	1789.8163	1789.8053	0.0110	1	HELQANCYEEVKDR (No match)
116 - 129	1790.8236	1789.8163	1789.8053	0.0110	1	HELQANCYEEVKDR (Ions score 81)

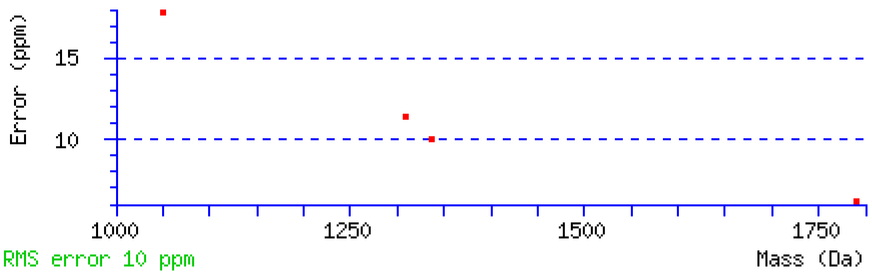

Mascot: http://www.matrixscience.com/

Spot 809

Protein View

Match to: **gi|119608074** Score: **113** Expect: **9.6e-007**
ribosomal protein L12, isoform CRA_c [Homo sapiens]

Nominal mass (M_r): **10457**; Calculated pI value: **9.89**
NCBI BLAST search of gi|119608074 against nr
Unformatted sequence string for pasting into other applications

Taxonomy: Homo sapiens

Fixed modifications: Carbamidomethyl (C)
Variable modifications: Oxidation (M)
Cleavage by Trypsin: cuts C-term side of KR unless next residue is P
Sequence Coverage: **32%**

Matched peptides shown in **Bold Red**

```
 1 MAVFTGGGVF TQESLLQIEV VPSASALIIK ALKEPPRDRK KQKNIKHSGN
51 ITFDEIVNIA RQMRHRSLAR ELSGTIKEIL GTAQSVGCNV DGRHP
```

Residue Number Increasing Mass Decreasing Mass

Start - End	Observed	Mr(expt)	Mr(calc)	Delta	Miss	Sequence	
47 - 61	1685.8658	1684.8585	1684.8532	0.0053	0	HSGNITFDEIVNIAR	(No match)
47 - 61	1685.8658	1684.8585	1684.8532	0.0053	0	HSGNITFDEIVNIAR	(Ions score 80)
78 - 93	1675.8127	1674.8054	1674.7995	0.0060	0	EILGTAQSVGCNVDGR	(Ions score 7)
78 - 93	1675.8127	1674.8054	1674.7995	0.0060	0	EILGTAQSVGCNVDGR	(No match)

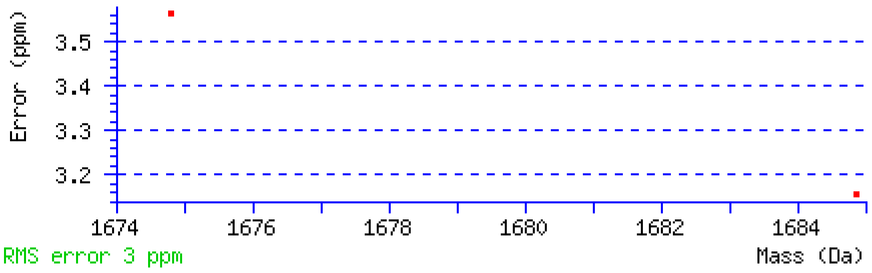

Spot 810

Protein View

Match to: **gi|5031635** Score: **293** Expect: **9.6e-025**
cofilin 1 (non-muscle) [Homo sapiens]

Nominal mass (M_r): **18719**; Calculated pI value: **8.22**
NCBI BLAST search of gi|5031635 against nr
Unformatted sequence string for pasting into other applications

Taxonomy: Homo sapiens
Links to retrieve other entries containing this sequence from NCBI Entrez:
gi|57099669 from Canis lupus familiaris
gi|114638608 from Pan troglodytes
gi|149725588 from Equus caballus
gi|116848 from Homo sapiens
gi|50513339 from Homo sapiens
gi|50513340 from Homo sapiens
gi|219545 from Homo sapiens
gi|736400 from Homo sapiens
gi|1177471 from Homo sapiens
gi|15012201 from Homo sapiens
gi|15126676 from Homo sapiens
gi|15147369 from Homo sapiens
gi|17390594 from Homo sapiens
gi|30582531 from Homo sapiens
gi|61360378 from synthetic construct
gi|119594855 from Homo sapiens
gi|119594858 from Homo sapiens
gi|123981732 from synthetic construct
gi|157928258 (no taxonomy information for this entry)

Fixed modifications: Carbamidomethyl (C)
Variable modifications: Oxidation (M)
Cleavage by Trypsin: cuts C-term side of KR unless next residue is P
Sequence Coverage: **62%**

Matched peptides shown in **Bold Red**

```
  1 MASGVAVSDG VIKVFNDMKV RKSSTPEEVK KRKKAVLFCL SEDKKNIILE
 51 EGKEILVGDV GQTVDDPYAT FVKMLPDKDC RYALYDATYE TKESKKEDLV
101 FIFWAPESAP LKSKMIYASS KDAIKKKLTG IKHELQANCY EEVKDRCTLA
151 EKLGGSAVIS LEGKPL
```

Residue Number Increasing Mass Decreasing Mass

Start - End	Observed	Mr(expt)	Mr(calc)	Delta	Miss	Sequence
14 - 21	1024.5118	1023.5045	1023.5171	-0.0126	1	**VFNDMKVR** Oxidation (M) (No match)
14 - 21	1024.5118	1023.5045	1023.5171	-0.0126	1	**VFNDMKVR** Oxidation (M) (Ions score 13)
23 - 31	1004.5094	1003.5021	1003.5185	-0.0164	1	**SSTPEEVKK** (No match)
35 - 45	1309.6913	1308.6840	1308.6747	0.0093	1	**AVLFCLSEDKK** (Ions score 53)
35 - 45	1309.6913	1308.6840	1308.6747	0.0093	1	**AVLFCLSEDKK** (No match)
46 - 53	915.5047	914.4974	914.5072	-0.0098	0	**NIILEEGK** (No match)
54 - 73	2166.1626	2165.1553	2165.0891	0.0662	0	**EILVGDVGQTVDDPYATFVK** (No match)
74 - 81	1034.4720	1033.4647	1033.4685	-0.0038	1	**MLPDKDCR** (No match)
74 - 81	1050.4613	1049.4540	1049.4634	-0.0094	1	**MLPDKDCR** Oxidation (M) (Ions score 15)
74 - 81	1050.4613	1049.4540	1049.4634	-0.0094	1	**MLPDKDCR** Oxidation (M) (No match)
82 - 92	1337.6338	1336.6265	1336.6187	0.0079	0	**YALYDATYETK** (No match)
82 - 92	1337.6338	1336.6265	1336.6187	0.0079	0	**YALYDATYETK** (Ions score 45)
133 - 146	1790.8464	1789.8391	1789.8053	0.0338	1	**HELQANCYEEVKDR** (No match)
133 - 146	1790.8464	1789.8391	1789.8053	0.0338	1	**HELQANCYEEVKDR** (Ions score 83)
153 - 166	1340.7831	1339.7758	1339.7710	0.0048	0	**LGGSAVISLEGKPL** (No match)

Mascot: http://www.matrixscience.com/

Spot 811

Protein View

Match to: **gi|119593214** Score: **91** Expect: **0.00016**
signal sequence receptor, delta (translocon-associated protein delta), isoform CRA_b [Homo sapiens]

Nominal mass (M_r): **12311**; Calculated pI value: **6.39**
NCBI BLAST search of gi|119593214 against nr
Unformatted sequence string for pasting into other applications

Taxonomy: Homo sapiens

Fixed modifications: Carbamidomethyl (C)
Variable modifications: Oxidation (M)
Cleavage by Trypsin: cuts C-term side of KR unless next residue is P
Sequence Coverage: **28%**

Matched peptides shown in **Bold Red**

```
  1 MALYADVGGK QFPVTRGQDV GRYQVSWSLD HKSAHAGTYE VRFFDEESYS
 51 LLRKAQRNNE DISIIPPLFT VSVDHRGTWN GPWVSTEVLA AAIGLVIYYL
101 AFSAKSHIQA
```

Residue Number Increasing Mass Decreasing Mass

Start - End	Observed	Mr(expt)	Mr(calc)	Delta	Miss	Sequence
43 - 53	1405.6973	1404.6900	1404.6561	0.0339	0	FFDEESYSLLR (Ions score 48)
43 - 53	1405.6973	1404.6900	1404.6561	0.0339	0	FFDEESYSLLR (No match)
43 - 54	1533.8047	1532.7974	1532.7510	0.0464	1	FFDEESYSLLRK (No match)
58 - 76	2166.1902	2165.1829	2165.1116	0.0713	0	NNEDISIIPPLFTVSVDHR (No match)
58 - 76	2166.1902	2165.1829	2165.1116	0.0713	0	NNEDISIIPPLFTVSVDHR (Ions score 6)

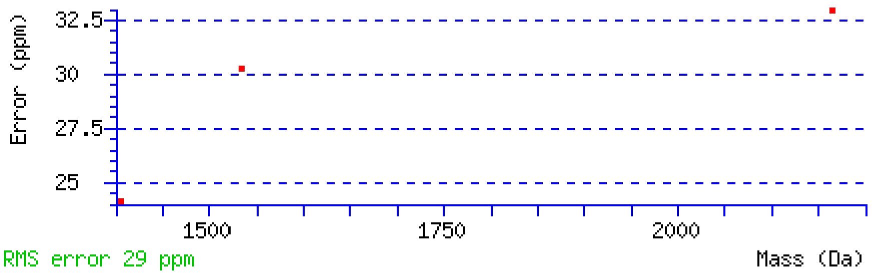

Mascot: http://www.matrixscience.com/

Spot 812

Protein View

Match to: **gi|5031635** Score: **251** Expect: **1.5e-020**
cofilin 1 (non-muscle) [Homo sapiens]

Nominal mass (M_r): **18719**; Calculated pI value: **8.22**
NCBI BLAST search of gi|5031635 against nr
Unformatted sequence string for pasting into other applications

Taxonomy: Homo sapiens
Links to retrieve other entries containing this sequence from NCBI Entrez:
gi|57099669 from Canis lupus familiaris
gi|114638608 from Pan troglodytes
gi|149725588 from Equus caballus
gi|116848 from Homo sapiens
gi|50513339 from Homo sapiens
gi|50513340 from Homo sapiens
gi|219545 from Homo sapiens
gi|736400 from Homo sapiens
gi|1177471 from Homo sapiens
gi|15012201 from Homo sapiens
gi|15126676 from Homo sapiens
gi|15147369 from Homo sapiens
gi|17390594 from Homo sapiens
gi|30582531 from Homo sapiens
gi|61360378 from synthetic construct
gi|119594855 from Homo sapiens
gi|119594858 from Homo sapiens
gi|123981732 from synthetic construct
gi|157928258 (no taxonomy information for this entry)

Fixed modifications: Carbamidomethyl (C)
Variable modifications: Oxidation (M)
Cleavage by Trypsin: cuts C-term side of KR unless next residue is P
Sequence Coverage: **52%**

Matched peptides shown in **Bold Red**

1 MASGVAVSDG VIK**VFNDMK**V RKSSTPEEVK KRKK**AVLFCL SEDKK**NIILE
51 EGK**EILVGDV GQTVDDPYAT FVKMLPDKDC RYALYDATYE TK**ESK**KEDLV**
101 **FIFWAPESAP LK**SKMIYASS KDAIKKKLTG IK**HELQANCY EEVKDR**CTLA
151 EKLGGSAVIS LEGKPL

Residue Number Increasing Mass Decreasing Mass

Start - End	Observed	Mr(expt)	Mr(calc)	Delta	Miss	Sequence
14 - 19	769.3838	768.3765	768.3476	0.0289	0	**VFNDMK** Oxidation (M) (No match)
35 - 45	1309.7135	1308.7062	1308.6747	0.0315	1	**AVLFCLSEDKK** (No match)
54 - 73	2166.1367	2165.1294	2165.0891	0.0403	0	**EILVGDVGQTVDDPYATFVK** (No match)
74 - 81	1034.5132	1033.5059	1033.4685	0.0374	1	**MLPDKDCR** (No match)
74 - 81	1034.5132	1033.5059	1033.4685	0.0374	1	**MLPDKDCR** (Ions score 17)
74 - 81	1050.5000	1049.4927	1049.4634	0.0293	1	**MLPDKDCR** Oxidation (M) (No match)
74 - 81	1050.5000	1049.4927	1049.4634	0.0293	1	**MLPDKDCR** Oxidation (M) (Ions score 14)
82 - 92	1337.6543	1336.6470	1336.6187	0.0284	0	**YALYDATYETK** (No match)
82 - 92	1337.6543	1336.6470	1336.6187	0.0284	0	**YALYDATYETK** (Ions score 48)
96 - 112	1990.1007	1989.0934	1989.0611	0.0324	1	**KEDLVFIFWAPESAPLK** (No match)
133 - 144	1519.7174	1518.7101	1518.6772	0.0329	0	**HELQANCYEEVK** (No match)
133 - 146	1790.8425	1789.8352	1789.8053	0.0299	1	**HELQANCYEEVKDR** (Ions score 86)
133 - 146	1790.8425	1789.8352	1789.8053	0.0299	1	**HELQANCYEEVKDR** (No match)

Mascot: http://www.matrixscience.com/

Spot 814

Protein View

Match to: **gi|5802966** Score: **235** Expect: **6.1e-019**
destrin isoform a [Homo sapiens]

Nominal mass (M_r): **18950;** Calculated pI value: **8.06**
NCBI BLAST search of gi|5802966 against nr
Unformatted sequence string for pasting into other applications

Taxonomy: Homo sapiens
Links to retrieve other entries containing this sequence from NCBI Entrez:
gi|51592111 from Sus scrofa
gi|62751673 from Bos taurus
gi|109092997 from Macaca mulatta
gi|46577584 from Sus scrofa
gi|46577586 from Homo sapiens
gi|75052660 from Bos taurus
gi|217682 from Sus scrofa
gi|415587 from Homo sapiens
gi|10637824 from Homo sapiens
gi|14550459 from Homo sapiens
gi|48146201 from Homo sapiens
gi|49456867 from Homo sapiens
gi|59858335 from Bos taurus
gi|119630680 from Homo sapiens

Fixed modifications: Carbamidomethyl (C)
Variable modifications: Oxidation (M)
Cleavage by Trypsin: cuts C-term side of KR unless next residue is P
Sequence Coverage: **35%**

Matched peptides shown in **Bold Red**

```
  1 MASGVQVADE VCRIFYDMKV RKCSTPEEIK KRKKAVIFCL SADKKCIIVE
 51 EGKEILVGDV GVTITDPFKH FVGMLPEKDC RYALYDASFE TKESRKEELM
101 FFLWAPELAP LKSKMIYASS KDAIKKKFQG IKHECQANGP EDLNRACIAE
151 KLGGSLIVAF EGCPV
```

Residue Number Increasing Mass Decreasing Mass

Start - End	Observed	Mr(expt)	Mr(calc)	Delta	Miss	Sequence
14 - 19	832.3892	831.3819	831.3836	-0.0017	0	IFYDMK Oxidation (M) (No match)
35 - 45	1251.6787	1250.6714	1250.6692	0.0022	1	AVIFCLSADKK (No match)
35 - 45	1251.6787	1250.6714	1250.6692	0.0022	1	AVIFCLSADKK (Ions score 24)
70 - 81	1504.6932	1503.6859	1503.6962	-0.0103	1	HFVGMLPEKDCR Oxidation (M) (No match)
70 - 81	1504.6932	1503.6859	1503.6962	-0.0103	1	HFVGMLPEKDCR Oxidation (M) (Ions score 30)
82 - 92	1307.6068	1306.5995	1306.6081	-0.0086	0	YALYDASFETK (Ions score 63)
82 - 92	1307.6068	1306.5995	1306.6081	-0.0086	0	YALYDASFETK (No match)
128 - 145	2112.9504	2111.9431	2111.9806	-0.0375	1	FQGIKHECQANGPEDLNR (No match)
133 - 145	1539.6512	1538.6439	1538.6532	-0.0092	0	HECQANGPEDLNR (No match)
133 - 145	1539.6512	1538.6439	1538.6532	-0.0092	0	HECQANGPEDLNR (Ions score 50)

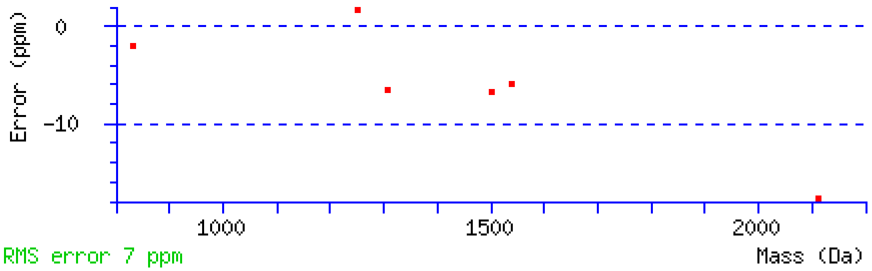

RMS error 7 ppm

Mascot: http://www.matrixscience.com/

Spot 816

Protein View

Match to: **gi|13544110** Score: **216** Expect: **4.8e-017**
CALM3 protein [Homo sapiens]

Nominal mass (M_r): **16610**; Calculated pI value: **4.33**
NCBI BLAST search of gi|13544110 against nr
Unformatted sequence string for pasting into other applications

Taxonomy: Homo sapiens

Fixed modifications: Carbamidomethyl (C)
Variable modifications: Oxidation (M)
Cleavage by Trypsin: cuts C-term side of KR unless next residue is P
Sequence Coverage: **32%**

Matched peptides shown in **Bold Red**

```
  1 MADQLTEEQI AEFKEAFSLF DKDGDGTITT KELGTVMRSL GQNPTEAELQ
 51 DMINEVDADG NGTIDFPEFL TMMARKMKDT DSEEEIREAF RVFDKDGNGY
101 ISAAELRHVM TNLGEKLTDE SLSMPLISSF CPRLFHPCLP RPDAFIS
```

Residue Number Increasing Mass Decreasing Mass

Start - End	Observed	Mr(expt)	Mr(calc)	Delta	Miss	Sequence
15 - 31	1844.9537	1843.9464	1843.8839	0.0625	1	EAFSLFDKDGDGTITTK (No match)
15 - 31	1844.9537	1843.9464	1843.8839	0.0625	1	EAFSLFDKDGDGTITTK (Ions score 46)
77 - 87	1368.6323	1367.6250	1367.5874	0.0376	1	MKDTDSEEEIR Oxidation (M) (No match)
79 - 87	1093.4818	1092.4745	1092.4571	0.0174	0	DTDSEEEIR (No match)
79 - 91	1596.7598	1595.7525	1595.7063	0.0462	1	DTDSEEEIREAFR (No match)
79 - 91	1596.7598	1595.7525	1595.7063	0.0462	1	DTDSEEEIREAFR (Ions score 6)
92 - 107	1754.9268	1753.9195	1753.8634	0.0561	1	VFDKDGNGYISAAELR (Ions score 98)
92 - 107	1754.9268	1753.9195	1753.8634	0.0561	1	VFDKDGNGYISAAELR (No match)
96 - 107	1265.6405	1264.6332	1264.6047	0.0285	0	DGNGYISAAELR (No match)

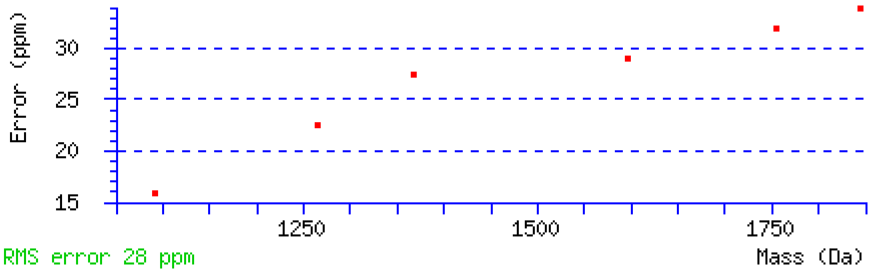

Mascot: http://www.matrixscience.com/

Spot 817

Protein View

Match to: **gi|4503545** Score: **213** Expect: **9.6e-017**
eukaryotic translation initiation factor 5A [Homo sapiens]

Nominal mass (M_r): **17049**; Calculated pI value: **5.08**
NCBI BLAST search of gi|4503545 against nr
Unformatted sequence string for pasting into other applications

Taxonomy: Homo sapiens
Links to retrieve other entries containing this sequence from NCBI Entrez:
gi|31712036 from Mus musculus
gi|51036244 from Bos taurus
gi|76096304 from Rattus norvegicus
gi|147899976 from Xenopus laevis
gi|73955431 from Canis lupus familiaris
gi|73955433 from Canis lupus familiaris
gi|126309172 from Monodelphis domestica
gi|149724243 from Equus caballus
gi|54035971 from Bos taurus
gi|54037409 from Homo sapiens
gi|54037410 from Mus musculus
gi|91207082 from Rattus norvegicus
gi|181997 from Homo sapiens
gi|453133 from Homo sapiens
gi|602245 from Homo sapiens
gi|12653915 from Homo sapiens
gi|12804783 from Homo sapiens
gi|12847343 from Mus musculus
gi|13278072 from Mus musculus
gi|14198050 from Mus musculus
gi|19353116 from Mus musculus
gi|20987352 from Homo sapiens
gi|33383427 from Homo sapiens
gi|33383429 from Homo sapiens
gi|33383432 from Homo sapiens
gi|33383436 from Mus musculus
gi|33383443 from Mus musculus
gi|33383445 from Mus musculus
gi|33383450 from Mus musculus
gi|33383453 from Mus musculus
gi|33383455 from Mus musculus
gi|33383460 from Mus musculus

gi|50399459 from Bos taurus
gi|50399461 from Bos taurus
gi|51325362 from Homo sapiens
gi|54673799 from Homo sapiens
gi|56800105 from Mus musculus
gi|60552517 from Xenopus laevis
gi|74204097 from Mus musculus
gi|74353711 from Rattus norvegicus
gi|78070768 from Homo sapiens
gi|79153705 from Bos taurus
gi|119610625 from Homo sapiens
gi|119610626 from Homo sapiens
gi|119610627 from Homo sapiens
gi|119610628 from Homo sapiens
gi|123982916 from synthetic construct
gi|123997597 from synthetic construct
gi|148680529 from Mus musculus
gi|148680530 from Mus musculus
gi|148680532 from Mus musculus
gi|148680538 from Mus musculus
gi|148680539 from Mus musculus
gi|148680543 from Mus musculus
gi|148680544 from Mus musculus
gi|149053104 from Rattus norvegicus
gi|149053105 from Rattus norvegicus
gi|149053108 from Rattus norvegicus
gi|149053109 from Rattus norvegicus
gi|149053110 from Rattus norvegicus
gi|149053111 from Rattus norvegicus
gi|149053112 from Rattus norvegicus
gi|149053113 from Rattus norvegicus
gi|149053114 from Rattus norvegicus
gi|149053115 from Rattus norvegicus
gi|149053116 from Rattus norvegicus
gi|158258667 (no taxonomy information for this entry)

Fixed modifications: Carbamidomethyl (C)
Variable modifications: Oxidation (M)
Cleavage by Trypsin: cuts C-term side of KR unless next residue is P
Sequence Coverage: **23%**

Matched peptides shown in **Bold Red**

1 MADDLDFETG DAGASATFPM QCSALRKNGF VVLKGRPCKI VEMSTSKTGK
51 HGHAK**VHLVG IDIFTGKK**YE DICPSTHNMD VPNIKR**NDFQ LIGIQDGYLS**
101 **LLQDSGEVR**E DLRLPEGDLG KEIEQKYDCG EEILITVLSA MTEEAAVAIK
151 AMAK

Residue Number Increasing Mass Decreasing Mass

Start - End	Observed	Mr(expt)	Mr(calc)	Delta	Miss	Sequence
56 - 67	1298.7083	1297.7010	1297.7393	-0.0383	0	VHLVGIDIFTGK (No match)
56 - 67	1298.7083	1297.7010	1297.7393	-0.0383	0	VHLVGIDIFTGK (Ions score 82)
56 - 68	1426.8053	1425.7980	1425.8343	-0.0363	1	VHLVGIDIFTGKK (No match)
56 - 68	1426.8053	1425.7980	1425.8343	-0.0363	1	VHLVGIDIFTGKK (Ions score 49)
87 - 109	2580.2075	2579.2002	2579.2867	-0.0864	0	NDFQLIGIQDGYLSLLQDSGEVR (No match)
87 - 109	2580.2075	2579.2002	2579.2867	-0.0864	0	NDFQLIGIQDGYLSLLQDSGEVR (Ions score 37)

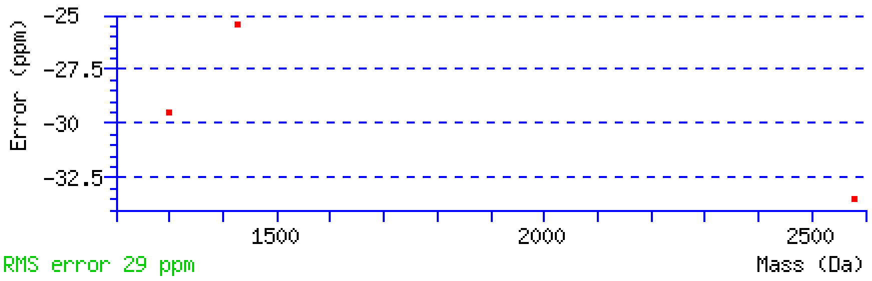

Mascot: http://www.matrixscience.com/

Spot 818

Protein View

Match to: **gi|19697925** Score: **164** Expect: **7.7e-012**
glia maturation factor gamma [Homo sapiens]

Nominal mass (M_r): **17020**; Calculated pI value: **5.95**
NCBI BLAST search of gi|19697925 against nr
Unformatted sequence string for pasting into other applications

Taxonomy: Homo sapiens

Fixed modifications: Carbamidomethyl (C)
Variable modifications: Oxidation (M)
Cleavage by Trypsin: cuts C-term side of KR unless next residue is P
Sequence Coverage: **45%**

Matched peptides shown in **Bold Red**

```
  1 MSDSLVVCEV DPELTEKLRK FRFRKETDNA AIIMKVDKDR QMVVLEEEFQ
 51 NISPEELKME LPERQPRFVV YSYKYVHDDG RVSYPLCFIF SSPVGCKRKQ
101 QMMYAGSKNR LVQTAELTKV FEIRTTDDLT EAWLQEKLSF FR
```

Residue Number Increasing Mass Decreasing Mass

Start - End	Observed	Mr(expt)	Mr(calc)	Delta	Miss	Sequence
41 - 58	2177.9863	2176.9790	2177.0561	-0.0771	0	**QMVVLEEEFQNISPEELK** Oxidation (M) (No match)
41 - 58	2177.9863	2176.9790	2177.0561	-0.0771	0	**QMVVLEEEFQNISPEELK** Oxidation (M) (Ions score 67)
59 - 67	1171.5544	1170.5471	1170.5815	-0.0344	1	**MELPERQPR** Oxidation (M) (Ions score 8)
68 - 74	905.4522	904.4449	904.4694	-0.0245	0	**FVVYSYK** (No match)
75 - 81	861.3669	860.3596	860.3776	-0.0180	0	**YVHDDGR** (No match)
99 - 108	1171.5544	1170.5471	1170.5525	-0.0054	1	**KQQMMYAGSK** (No match)
125 - 137	1549.6803	1548.6730	1548.7307	-0.0577	0	**TTDDLTEAWLQEK** (No match)

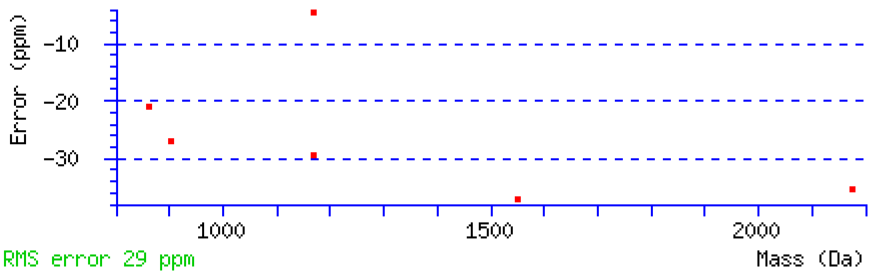

Mascot: http://www.matrixscience.com/

Spot 820

Protein View

Match to: **gi|122890670** Score: **168** Expect: **3e-012**
stathmin 1/oncoprotein 18 [Homo sapiens]

Nominal mass (M_r): **9858**; Calculated pI value: **6.75**
NCBI BLAST search of gi|122890670 against nr
Unformatted sequence string for pasting into other applications

Taxonomy: Homo sapiens

Fixed modifications: Carbamidomethyl (C)
Variable modifications: Oxidation (M)
Cleavage by Trypsin: cuts C-term side of KR unless next residue is P
Sequence Coverage: **41%**

Matched peptides shown in **Bold Red**

```
 1 MASSDIQVKE LEKRASGQAF ELILSPRSKE SVPEFPLSPP KKKDLSLEEI
51 QKKLEAAEER RKSHEAEVLK QLAEKREHEK EVLQKA
```

Residue Number Increasing Mass Decreasing Mass

Start - End	Observed	Mr(expt)	Mr(calc)	Delta	Miss	Sequence
14 - 27	1544.8945	1543.8872	1543.8470	0.0402	1	RASGQAFELILSPR (No match)
15 - 27	1388.7963	1387.7890	1387.7459	0.0431	0	ASGQAFELILSPR (No match)
15 - 27	1388.7963	1387.7890	1387.7459	0.0431	0	ASGQAFELILSPR (Ions score 75)
28 - 41	1541.8711	1540.8638	1540.8136	0.0502	1	SKESVPEFPLSPPK (No match)
28 - 41	1541.8711	1540.8638	1540.8136	0.0502	1	SKESVPEFPLSPPK (Ions score 30)
30 - 41	1326.7255	1325.7182	1325.6867	0.0316	0	ESVPEFPLSPPK (No match)
53 - 60	945.5108	944.5035	944.4926	0.0109	1	KLEAAEER (No match)

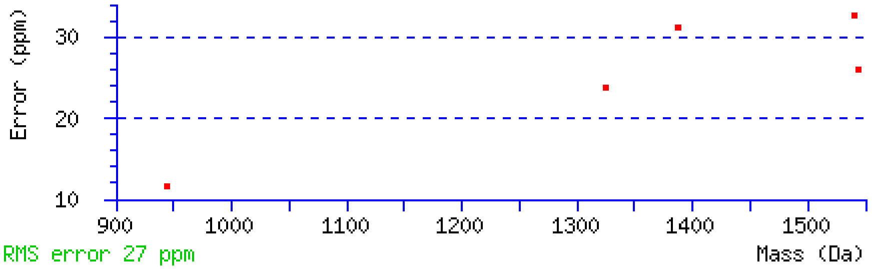

Mascot: http://www.matrixscience.com/

Spot 821

Protein View

Match to: **gi|28071074** Score: **183** Expect: **9.6e-014**
unnamed protein product [Homo sapiens]

Nominal mass (M_r): **13945**; Calculated pI value: **9.20**
NCBI BLAST search of gi|28071074 against nr
Unformatted sequence string for pasting into other applications

Taxonomy: Homo sapiens

Fixed modifications: Carbamidomethyl (C)
Variable modifications: Oxidation (M)
Cleavage by Trypsin: cuts C-term side of KR unless next residue is P
Sequence Coverage: **46%**

Matched peptides shown in **Bold Red**

```
  1 MLITILGTVK PNANRIALDF QRGNDVAFHF NPRFNENNRR VIVCNTKLDN
 51 NWGREERQSV FPFESGKPFK IQVLVEPDHF KVAVNDAHLL QYNHRVKKLN
101 EISKLGISGD IDLTSASYTM I
```

Residue Number Increasing Mass Decreasing Mass

Start - End	Observed	Mr(expt)	Mr(calc)	Delta	Miss	Sequence
16 - 22	862.5154	861.5081	861.4708	0.0373	0	IALDFQR (No match)
23 - 33	1273.6401	1272.6328	1272.5999	0.0329	0	GNDVAFHFNPR (No match)
23 - 33	1273.6401	1272.6328	1272.5999	0.0329	0	GNDVAFHFNPR (Ions score 40)
58 - 70	1497.8054	1496.7981	1496.7663	0.0318	0	QSVFPFESGKPFK (No match)
71 - 81	1324.7560	1323.7487	1323.7186	0.0301	0	IQVLVEPDHFK (Ions score 13)
71 - 81	1324.7560	1323.7487	1323.7186	0.0301	0	IQVLVEPDHFK (No match)
82 - 95	1649.8859	1648.8786	1648.8433	0.0353	0	VAVNDAHLLQYNHR (No match)
82 - 95	1649.8859	1648.8786	1648.8433	0.0353	0	VAVNDAHLLQYNHR (Ions score 56)

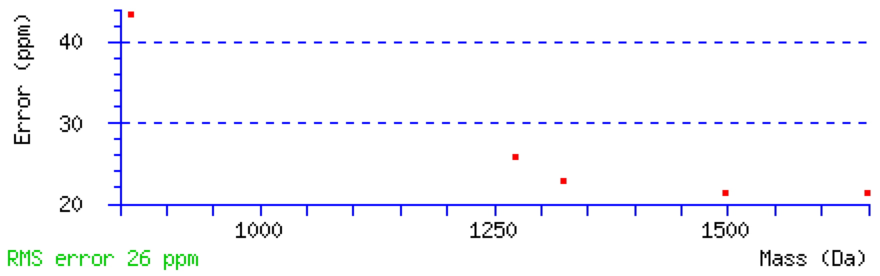

Mascot: http://www.matrixscience.com/

Spot 822

Protein View

Match to: **gi|5802966** Score: **86** Expect: **0.00054**
destrin isoform a [Homo sapiens]

Nominal mass (M_r): **18950**; Calculated pI value: **8.06**
NCBI BLAST search of gi|5802966 against nr
Unformatted sequence string for pasting into other applications

Taxonomy: Homo sapiens
Links to retrieve other entries containing this sequence from NCBI Entrez:
gi|51592111 from Sus scrofa
gi|62751673 from Bos taurus
gi|109092997 from Macaca mulatta
gi|46577584 from Sus scrofa
gi|46577586 from Homo sapiens
gi|75052660 from Bos taurus
gi|217682 from Sus scrofa
gi|415587 from Homo sapiens
gi|10637824 from Homo sapiens
gi|14550459 from Homo sapiens
gi|48146201 from Homo sapiens
gi|49456867 from Homo sapiens
gi|59858335 from Bos taurus
gi|119630680 from Homo sapiens

Fixed modifications: Carbamidomethyl (C)
Variable modifications: Oxidation (M)
Cleavage by Trypsin: cuts C-term side of KR unless next residue is P
Sequence Coverage: **18%**

Matched peptides shown in **Bold Red**

1 MASGVQVADE VCR**IFYDMK**V RKCSTPEEIK KRKKAVIFCL SADKKCIIVE
51 EGKEILVGDV GVTITDPFKH FVGMLPEKDC R**YALYDASFE TK**ESRKEELM
101 FFLWAPELAP LKSKMIYASS KDAIKKKFQG IK**HECQANGP EDLNR**ACIAE
151 KLGGSLIVAF EGCPV

Residue Number Increasing Mass Decreasing Mass

Start - End	Observed	Mr(expt)	Mr(calc)	Delta	Miss	Sequence
14 - 19	832.4180	831.4107	831.3836	0.0271	0	IFYDMK Oxidation (M) (No match)
82 - 92	1307.6545	1306.6472	1306.6081	0.0391	0	YALYDASFETK (Ions score 35)
82 - 92	1307.6545	1306.6472	1306.6081	0.0391	0	YALYDASFETK (No match)
133 - 145	1539.7000	1538.6927	1538.6532	0.0396	0	HECQANGPEDLNR (No match)
133 - 145	1539.7000	1538.6927	1538.6532	0.0396	0	HECQANGPEDLNR (Ions score 28)

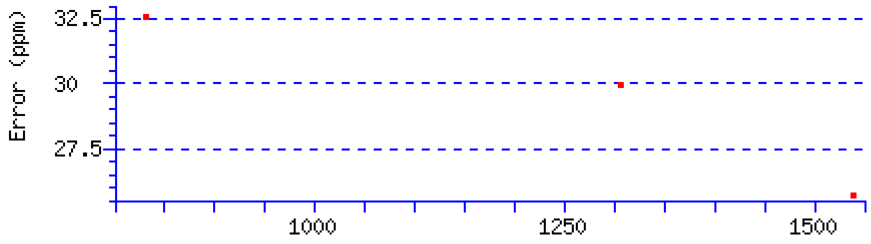

RMS error 28 ppm

Mascot: http://www.matrixscience.com/

Spot 823

Protein View

Match to: **gi|66392203** Score: **336** Expect: **4.8e-029**
NME1-NME2 protein [Homo sapiens]

Nominal mass (M_r): **30346**; Calculated pI value: **9.06**
NCBI BLAST search of gi|66392203 against nr
Unformatted sequence string for pasting into other applications

Taxonomy: Homo sapiens
Links to retrieve other entries containing this sequence from NCBI Entrez:
gi|73746297 from Homo sapiens
gi|124376250 from Homo sapiens
gi|124376672 from Homo sapiens

Fixed modifications: Carbamidomethyl (C)
Variable modifications: Oxidation (M)
Cleavage by Trypsin: cuts C-term side of KR unless next residue is P
Sequence Coverage: **42%**

Matched peptides shown in **Bold Red**

```
  1 MANCERTFIA IKPDGVQRGL VGEIIKRFEQ KGFRLVGLKF MQASEDLLKE
 51 HYVDLKDRPF FAGLVKYMHS GPVVAMVWEG LNVVKTGRVM LGETNPADSK
101 PGTIRGDFCI QVGRTMANLE RTFIAIKPDG VQRGLVGEII KRFEQKGFRL
151 VAMKFLRASE EHLKQHYIDL KDRPFFPGLV KYMNSGPVVA MVWEGLNVVK
201 TGRVMLGETN PADSKPGTIR GDFCIQVGRN IIHGSDSVKS AEKEISLWFK
251 PEELVDYKSC AHDWVYE
```

Residue Number Increasing Mass Decreasing Mass

Start - End	Observed	Mr(expt)	Mr(calc)	Delta	Miss	Sequence
7 - 18	1344.7380	1343.7307	1343.7561	-0.0253	0	TFIAIKPDGVQR (Ions score 28)
19 - 27	984.6031	983.5958	983.6127	-0.0169	1	GLVGEIIKR (Ions score 21)
40 - 49	1197.6167	1196.6094	1196.5747	0.0347	0	FMQASEDLLK Oxidation (M) (No match)
89 - 105	1801.8677	1800.8604	1800.9039	-0.0435	0	VMLGETNPADSKPGTIR Oxidation (M) (Ions score 72)
106 - 114	1051.4830	1050.4757	1050.4916	-0.0159	0	GDFCIQVGR (Ions score 22)
122 - 133	1344.7380	1343.7307	1343.7561	-0.0253	0	TFIAIKPDGVQR (No match)
134 - 142	984.6031	983.5958	983.6127	-0.0169	1	GLVGEIIKR (No match)

172 - 181	1175.6367	1174.6294	1174.6498	-0.0204	0	DRPFFPGLVK	(Ions score 49)
172 - 181	1175.6367	1174.6294	1174.6498	-0.0204	0	DRPFFPGLVK	(No match)
204 - 220	1785.8783	1784.8710	1784.9090	-0.0380	0	VMLGETNPADSKPGTIR	(No match)
204 - 220	1801.8677	1800.8604	1800.9039	-0.0435	0	VMLGETNPADSKPGTIR	Oxidation (M) (No match)
221 - 229	1051.4830	1050.4757	1050.4916	-0.0159	0	GDFCIQVGR	(No match)

Mascot: http://www.matrixscience.com/

Spot 824

Protein View

Match to: **gi|1633054** Score: **205** Expect: **6.1e-016**
Chain A, Cyclophilin A Complexed With Dipeptide Gly-Pro

Nominal mass (M_r): **18098**; Calculated pI value: **7.82**
NCBI BLAST search of gi|1633054 against nr
Unformatted sequence string for pasting into other applications

Taxonomy: Homo sapiens
Links to retrieve other entries containing this sequence from NCBI Entrez:
gi|1633056 from Homo sapiens
gi|1633058 from Homo sapiens
gi|1633063 from Homo sapiens
gi|1827592 from Homo sapiens
gi|1827593 from Homo sapiens
gi|2981729 from Homo sapiens
gi|2981731 from Homo sapiens
gi|2981732 from Homo sapiens
gi|2981733 from Homo sapiens
gi|2981734 from Homo sapiens
gi|2981735 from Homo sapiens
gi|2981736 from Homo sapiens
gi|2981757 from Homo sapiens
gi|2981759 from Homo sapiens
gi|2981760 from Homo sapiens
gi|2981761 from Homo sapiens
gi|2981762 from Homo sapiens
gi|2981763 from Homo sapiens
gi|2981764 from Homo sapiens

Fixed modifications: Carbamidomethyl (C)
Variable modifications: Oxidation (M)
Cleavage by Trypsin: cuts C-term side of KR unless next residue is P
Sequence Coverage: **43%**

Matched peptides shown in **Bold Red**

1 **VNPTVFFDIA VDGEPLGRVS FELFADKVPK** TAENFRALST GEKGFGYKGS
51 CFHR**IIPGFM CQGGDFTR**HN GTGGK**SIYGE KFEDENFILK** HTGPGILSMA
101 NAGPNTNGSQ FFICTAKTEW LDGKHVVFGK **VKEGMNIVEA MER**FGSRNGK
151 TSKKITIADC GQLE

Residue Number Increasing Mass Decreasing Mass

Start - End	Observed	Mr(expt)	Mr(calc)	Delta	Miss	Sequence
1 - 18	1946.0552	1945.0479	1944.9944	0.0535	0	VNPTVFFDIAVDGEPLGR (No match)
19 - 30	1379.7711	1378.7638	1378.7496	0.0143	1	VSFELFADKVPK (No match)
19 - 30	1379.7711	1378.7638	1378.7496	0.0143	1	VSFELFADKVPK (Ions score 51)
55 - 68	1614.7727	1613.7654	1613.7330	0.0325	0	IIPGFMCQGGDFTR Oxidation (M) (Ions score 6)
55 - 68	1614.7727	1613.7654	1613.7330	0.0325	0	IIPGFMCQGGDFTR Oxidation (M) (No match)
76 - 90	1831.9612	1830.9539	1830.9039	0.0500	1	SIYGEKFEDENFILK (Ions score 43)
76 - 90	1831.9612	1830.9539	1830.9039	0.0500	1	SIYGEKFEDENFILK (No match)
82 - 90	1154.5751	1153.5678	1153.5655	0.0023	0	FEDENFILK (No match)
131 - 143	1537.7606	1536.7533	1536.7275	0.0258	1	VKEGMNIVEAMER 2 Oxidation (M) (Ions score 6)
131 - 143	1537.7606	1536.7533	1536.7275	0.0258	1	VKEGMNIVEAMER 2 Oxidation (M) (No match)
133 - 143	1310.5844	1309.5771	1309.5642	0.0130	0	EGMNIVEAMER 2 Oxidation (M) (No match)

Mascot: http://www.matrixscience.com/

Spot 826

Protein View

Match to: **gi|1633054** Score: **218** Expect: **3e-017**
Chain A, Cyclophilin A Complexed With Dipeptide Gly-Pro

Nominal mass (M_r): **18098**; Calculated pI value: **7.82**
NCBI BLAST search of gi|1633054 against nr
Unformatted sequence string for pasting into other applications

Taxonomy: Homo sapiens
Links to retrieve other entries containing this sequence from NCBI Entrez:
gi|1633056 from Homo sapiens
gi|1633058 from Homo sapiens
gi|1633063 from Homo sapiens
gi|1827592 from Homo sapiens
gi|1827593 from Homo sapiens
gi|2981729 from Homo sapiens
gi|2981731 from Homo sapiens
gi|2981732 from Homo sapiens
gi|2981733 from Homo sapiens
gi|2981734 from Homo sapiens
gi|2981735 from Homo sapiens
gi|2981736 from Homo sapiens
gi|2981757 from Homo sapiens
gi|2981759 from Homo sapiens
gi|2981760 from Homo sapiens
gi|2981761 from Homo sapiens
gi|2981762 from Homo sapiens
gi|2981763 from Homo sapiens
gi|2981764 from Homo sapiens

Fixed modifications: Carbamidomethyl (C)
Variable modifications: Oxidation (M)
Cleavage by Trypsin: cuts C-term side of KR unless next residue is P
Sequence Coverage: **43%**

Matched peptides shown in **Bold Red**

1 **VNPTVFFDIA VDGEPLGRVS FELFADKVPK** TAENFRALST GEKGFGYKGS
51 CFHR**IIPGFM CQGGDFTR**HN GTGGK**SIYGE KFEDENFILK** HTGPGILSMA
101 NAGPNTNGSQ FFICTAKTEW LDGKHVVFGK **VKEGMNIVEA MER**FGSRNGK
151 TSKKITIADC GQLE

Residue Number Increasing Mass Decreasing Mass

Start - End	Observed	Mr(expt)	Mr(calc)	Delta	Miss	Sequence
1 - 18	1946.0632	1945.0559	1944.9944	0.0615	0	VNPTVFFDIAVDGEPLGR (Ions score 41)
1 - 18	1946.0632	1945.0559	1944.9944	0.0615	0	VNPTVFFDIAVDGEPLGR (No match)
19 - 30	1379.7819	1378.7746	1378.7496	0.0251	1	VSFELFADKVPK (No match)
19 - 30	1379.7819	1378.7746	1378.7496	0.0251	1	VSFELFADKVPK (Ions score 44)
55 - 68	1614.7830	1613.7757	1613.7330	0.0428	0	IIPGFMCQGGDFTR Oxidation (M) (No match)
55 - 68	1614.7830	1613.7757	1613.7330	0.0428	0	IIPGFMCQGGDFTR Oxidation (M) (Ions score 6)
76 - 90	1831.9666	1830.9593	1830.9039	0.0554	1	SIYGEKFEDENFILK (No match)
76 - 90	1831.9666	1830.9593	1830.9039	0.0554	1	SIYGEKFEDENFILK (Ions score 27)
82 - 90	1154.5813	1153.5740	1153.5655	0.0085	0	FEDENFILK (No match)
131 - 143	1537.7717	1536.7644	1536.7275	0.0369	1	VKEGMNIVEAMER 2 Oxidation (M) (No match)
131 - 143	1537.7717	1536.7644	1536.7275	0.0369	1	VKEGMNIVEAMER 2 Oxidation (M) (No match)
133 - 143	1310.5903	1309.5830	1309.5642	0.0189	0	EGMNIVEAMER 2 Oxidation (M) (No match)

Mascot: http://www.matrixscience.com/

Spot 828

Protein View

Match to: **gi|1633054** Score: **78** Expect: **0.0034**
Chain A, Cyclophilin A Complexed With Dipeptide Gly-Pro

Nominal mass (M_r): **18098;** Calculated pI value: **7.82**
NCBI BLAST search of gi|1633054 against nr
Unformatted sequence string for pasting into other applications

Taxonomy: Homo sapiens
Links to retrieve other entries containing this sequence from NCBI Entrez:
gi|1633056 from Homo sapiens
gi|1633058 from Homo sapiens
gi|1633063 from Homo sapiens
gi|1827592 from Homo sapiens
gi|1827593 from Homo sapiens
gi|2981729 from Homo sapiens
gi|2981731 from Homo sapiens
gi|2981732 from Homo sapiens
gi|2981733 from Homo sapiens
gi|2981734 from Homo sapiens
gi|2981735 from Homo sapiens
gi|2981736 from Homo sapiens
gi|2981757 from Homo sapiens
gi|2981759 from Homo sapiens
gi|2981760 from Homo sapiens
gi|2981761 from Homo sapiens
gi|2981762 from Homo sapiens
gi|2981763 from Homo sapiens
gi|2981764 from Homo sapiens

Fixed modifications: Carbamidomethyl (C)
Variable modifications: Oxidation (M)
Cleavage by Trypsin: cuts C-term side of KR unless next residue is P
Sequence Coverage: **19%**

Matched peptides shown in **Bold Red**

1 **VNPTVFFDIA VDGEPLGR**VS FELFADKVPK TAENFRALST GEKGFGYKGS
51 CFHR**IIPGFM CQGGDFTR**HN GTGGKSIYGE KFEDENFILK HTGPGILSMA
101 NAGPNTNGSQ FFICTAKTEW LDGKHVVFGK VKEGMNIVEA MERFGSRNGK
151 TSKKITIADC GQLE

Residue Number Increasing Mass Decreasing Mass

Start - End	Observed	Mr(expt)	Mr(calc)	Delta	Miss	Sequence
1 - 18	1946.0251	1945.0178	1944.9944	0.0234	0	VNPTVFFDIAVDGEPLGR (No match)
1 - 18	1946.0251	1945.0178	1944.9944	0.0234	0	VNPTVFFDIAVDGEPLGR (Ions score 50)
55 - 68	1614.7634	1613.7561	1613.7330	0.0232	0	IIPGFMCQGGDFTR Oxidation (M) (No match)
55 - 68	1614.7634	1613.7561	1613.7330	0.0232	0	IIPGFMCQGGDFTR Oxidation (M) (No match)

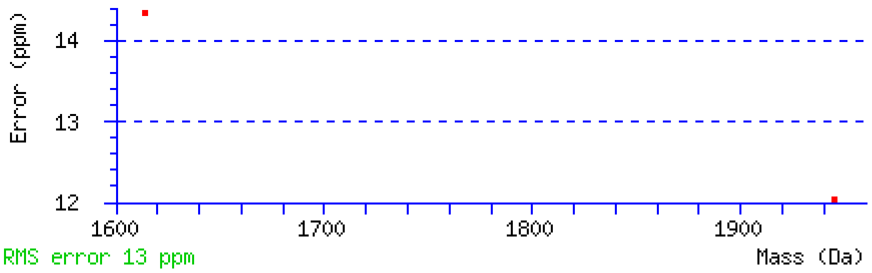

Mascot: http://www.matrixscience.com/

Spot 829

Protein View

Match to: **gi|1633054** Score: **76** Expect: **0.0053**
Chain A, Cyclophilin A Complexed With Dipeptide Gly-Pro

Nominal mass (M_r): **18098**; Calculated pI value: **7.82**
NCBI BLAST search of gi|1633054 against nr
Unformatted sequence string for pasting into other applications

Taxonomy: Homo sapiens
Links to retrieve other entries containing this sequence from NCBI Entrez:
gi|1633056 from Homo sapiens
gi|1633058 from Homo sapiens
gi|1633063 from Homo sapiens
gi|1827592 from Homo sapiens
gi|1827593 from Homo sapiens
gi|2981729 from Homo sapiens
gi|2981731 from Homo sapiens
gi|2981732 from Homo sapiens
gi|2981733 from Homo sapiens
gi|2981734 from Homo sapiens
gi|2981735 from Homo sapiens
gi|2981736 from Homo sapiens
gi|2981757 from Homo sapiens
gi|2981759 from Homo sapiens
gi|2981760 from Homo sapiens
gi|2981761 from Homo sapiens
gi|2981762 from Homo sapiens
gi|2981763 from Homo sapiens
gi|2981764 from Homo sapiens

Fixed modifications: Carbamidomethyl (C)
Variable modifications: Oxidation (M)
Cleavage by Trypsin: cuts C-term side of KR unless next residue is P
Sequence Coverage: **28%**

Matched peptides shown in **Bold Red**

1 **VNPTVFFDIA VDGEPLGR**VS FELFADKVPK TAENFRALST GEKGFGYKGS
51 CFHR**IIPGFM CQGGDFTR**HN GTGGK**SIYGE KFEDENFILK** HTGPGILSMA
101 NAGPNTNGSQ FFICTAKTEW LDGKHVVFGK VKEGMNIVEA MERFGSRNGK
151 TSKKITIADC GQLE

Residue Number Increasing Mass Decreasing Mass

Start - End	Observed	Mr(expt)	Mr(calc)	Delta	Miss	Sequence
1 - 18	1945.9941	1944.9868	1944.9944	-0.0076	0	VNPTVFFDIAVDGEPLGR (Ions score 41)
1 - 18	1945.9941	1944.9868	1944.9944	-0.0076	0	VNPTVFFDIAVDGEPLGR (No match)
55 - 68	1614.7377	1613.7304	1613.7330	-0.0025	0	IIPGFMCQGGDFTR Oxidation (M) (No match)
55 - 68	1614.7377	1613.7304	1613.7330	-0.0025	0	IIPGFMCQGGDFTR Oxidation (M) (No match)
76 - 90	1831.8960	1830.8887	1830.9039	-0.0152	1	SIYGEKFEDENFILK (No match)

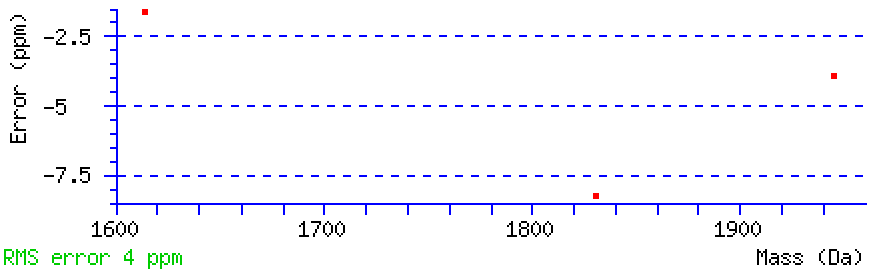

Mascot: http://www.matrixscience.com/

Spot 830

Protein View

Match to: **gi|113812151** Score: **273** Expect: **9.6e-023**
MYL6 protein [Homo sapiens]

Nominal mass (M_r): **15923**; Calculated pI value: **4.60**
NCBI BLAST search of gi|113812151 against nr
Unformatted sequence string for pasting into other applications

Taxonomy: Homo sapiens

Fixed modifications: Carbamidomethyl (C)
Variable modifications: Oxidation (M)
Cleavage by Trypsin: cuts C-term side of KR unless next residue is P
Sequence Coverage: **44%**

Matched peptides shown in **Bold Red**

```
  1 EFKEAFQLFD RTGDGKILYS QCGDVMRALG QNPTNAEVLK VLGNPKSDEM
 51 NVKVLDFEHF LPMLQTVAKN KDQGTYEDYV EGLRVFDKEG NGTVMGAEIR
101 HVLVTLGEKM TEEEVEMLVA GHEDSNGCIN YEELVRMVLN G
```

Residue Number Increasing Mass Decreasing Mass

Start - End	Observed	Mr(expt)	Mr(calc)	Delta	Miss	Sequence
4 - 11	1025.4916	1024.4843	1024.4977	-0.0134	0	EAFQLFDR (No match)
4 - 11	1025.4916	1024.4843	1024.4977	-0.0134	0	EAFQLFDR (Ions score 33)
17 - 27	1357.5953	1356.5880	1356.6166	-0.0285	0	ILYSQCGDVMR Oxidation (M) (No match)
17 - 27	1357.5953	1356.5880	1356.6166	-0.0285	0	ILYSQCGDVMR Oxidation (M) (Ions score 5)
28 - 40	1354.7045	1353.6972	1353.7252	-0.0279	0	ALGQNPTNAEVLK (No match)
70 - 84	1786.7886	1785.7813	1785.8169	-0.0356	1	NKDQGTYEDYVEGLR (No match)
70 - 84	1786.7886	1785.7813	1785.8169	-0.0356	1	NKDQGTYEDYVEGLR (Ions score 100)
72 - 84	1544.6577	1543.6504	1543.6790	-0.0286	0	DQGTYEDYVEGLR (No match)
85 - 100	1738.8065	1737.7992	1737.8355	-0.0363	1	VFDKEGNGTVMGAEIR Oxidation (M) (Ions score 32)
85 - 100	1738.8065	1737.7992	1737.8355	-0.0363	1	VFDKEGNGTVMGAEIR Oxidation (M) (No match)

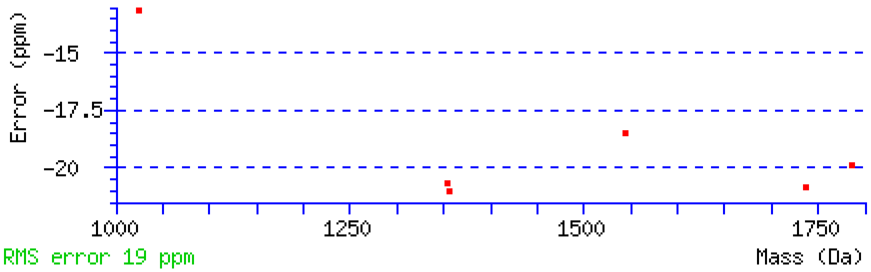

RMS error 19 ppm

Mascot: http://www.matrixscience.com/

Spot 831

Protein View

Match to: **gi|11641247** Score: **217** Expect: **3.8e-017**
chromosome 9 open reading frame 19 [Homo sapiens]

Nominal mass (M_r): **17322;** Calculated pI value: **9.44**
NCBI BLAST search of gi|11641247 against nr
Unformatted sequence string for pasting into other applications

Taxonomy: Homo sapiens
Links to retrieve other entries containing this sequence from NCBI Entrez:
gi|46397431 from Homo sapiens
gi|10798580 from Homo sapiens
gi|21654811 from Homo sapiens
gi|22760954 from Homo sapiens
gi|55662093 from Homo sapiens

Fixed modifications: Carbamidomethyl (C)
Variable modifications: Oxidation (M)
Cleavage by Trypsin: cuts C-term side of KR unless next residue is P
Sequence Coverage: **45%**

Matched peptides shown in **Bold Red**

```
  1 MGKSASKQFH NEVLKAHNEY RQKHGVPPLK LCKNLNREAQ QYSEALASTR
 51 ILKHSPESSR GQCGENLAWA SYDQTGKEVA DRWYSEIKNY NFQQPGFTSG
101 TGHFTAMVWK NTKKMGVGKA SASDGSSFVV ARYFPAGNVV NEGFFEENVL
151 PPKK
```

Residue Number Increasing Mass Decreasing Mass

Start - End	Observed	Mr(expt)	Mr(calc)	Delta	Miss	Sequence
38 - 50	1453.6700	1452.6627	1452.6844	-0.0217	0	EAQQYSEALASTR (No match)
38 - 50	1453.6700	1452.6627	1452.6844	-0.0217	0	EAQQYSEALASTR (Ions score 51)
89 - 110	2518.1885	2517.1812	2517.1535	0.0277	0	NYNFQQPGFTSGTGHFTAMVWK (No match)
120 - 132	1253.5967	1252.5894	1252.6047	-0.0153	0	ASASDGSSFVVAR (No match)
120 - 132	1253.5967	1252.5894	1252.6047	-0.0153	0	ASASDGSSFVVAR (Ions score 10)
133 - 154	2495.2136	2494.2063	2494.2531	-0.0468	1	YFPAGNVVNEGFFEENVLPPKK (No match)
133 - 154	2495.2136	2494.2063	2494.2531	-0.0468	1	YFPAGNVVNEGFFEENVLPPKK (Ions score 99)

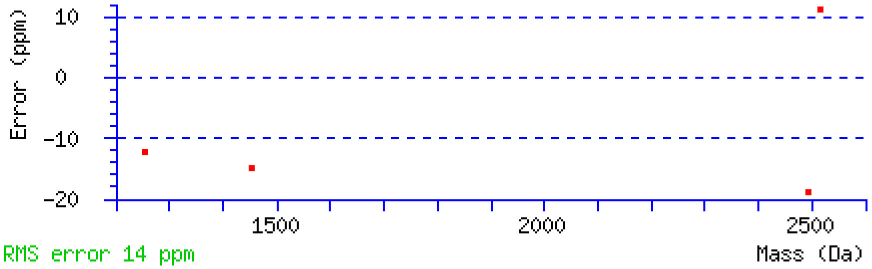

Mascot: http://www.matrixscience.com/

Spot 832

Protein View

Match to: **gi|28071074** Score: **258** Expect: **3e-021**
unnamed protein product [Homo sapiens]

Nominal mass (M_r): **13945**; Calculated pI value: **9.20**
NCBI BLAST search of gi|28071074 against nr
Unformatted sequence string for pasting into other applications

Taxonomy: Homo sapiens

Fixed modifications: Carbamidomethyl (C)
Variable modifications: Oxidation (M)
Cleavage by Trypsin: cuts C-term side of KR unless next residue is P
Sequence Coverage: **64%**

Matched peptides shown in **Bold Red**

```
  1 MLITILGTVK PNANRIALDF QRGNDVAFHF NPRFNENNRR VIVCNTKLDN
 51 NWGREERQSV FPFESGKPFK IQVLVEPDHF KVAVNDAHLL QYNHRVKKLN
101 EISKLGISGD IDLTSASYTM I
```

Residue Number Increasing Mass Decreasing Mass

Start - End	Observed	Mr(expt)	Mr(calc)	Delta	Miss	Sequence
1 - 15	1656.9441	1655.9368	1655.9392	-0.0024	0	MLITILGTVKPNANR Oxidation (M) (No match)
16 - 22	862.4972	861.4899	861.4708	0.0191	0	IALDFQR (No match)
23 - 33	1273.6139	1272.6066	1272.5999	0.0067	0	GNDVAFHFNPR (No match)
23 - 33	1273.6139	1272.6066	1272.5999	0.0067	0	GNDVAFHFNPR (Ions score 50)
34 - 40	949.4739	948.4666	948.4525	0.0141	1	FNENNRR (No match)
58 - 70	1497.7750	1496.7677	1496.7663	0.0014	0	QSVFPFESGKPFK (No match)
71 - 81	1324.7306	1323.7233	1323.7186	0.0047	0	IQVLVEPDHFK (No match)
71 - 81	1324.7306	1323.7233	1323.7186	0.0047	0	IQVLVEPDHFK (Ions score 27)
82 - 95	1649.8491	1648.8418	1648.8433	-0.0015	0	VAVNDAHLLQYNHR (Ions score 75)
82 - 95	1649.8491	1648.8418	1648.8433	-0.0015	0	VAVNDAHLLQYNHR (No match)

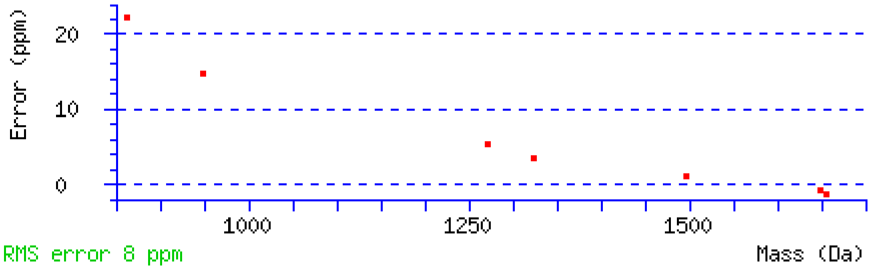

Mascot: http://www.matrixscience.com/

Spot 834

Protein View

Match to: **gi|13386490** Score: **183** Expect: **9.6e-014**
LIM domain containing 2 [Homo sapiens]

Nominal mass (M_r): **14460**; Calculated pI value: **9.23**
NCBI BLAST search of gi|13386490 against nr
Unformatted sequence string for pasting into other applications

Taxonomy: Homo sapiens
Links to retrieve other entries containing this sequence from NCBI Entrez:
gi|109116807 from Macaca mulatta
gi|109116809 from Macaca mulatta
gi|74752322 from Homo sapiens
gi|13325166 from Homo sapiens
gi|21750863 from Homo sapiens
gi|30353982 from Homo sapiens
gi|119614697 from Homo sapiens
gi|119614698 from Homo sapiens
gi|119614700 from Homo sapiens
gi|119614701 from Homo sapiens
gi|119614702 from Homo sapiens

Fixed modifications: Carbamidomethyl (C)
Variable modifications: Oxidation (M)
Cleavage by Trypsin: cuts C-term side of KR unless next residue is P
Sequence Coverage: **18%**

Matched peptides shown in **Bold Red**

```
  1 MFQAAGAAQA TPSHDAKGGG SSTVQRSKSF SLRAQVKETC AACQKTVYPM
 51 ERLVADKLIF HNSCFCCKHC HTKLSLGSYA ALHGEFYCKP HFQQLFKSKG
101 NYDEGFGRKQ HKELWAHKEV DPGTKTA
```

Residue Number Increasing Mass Decreasing Mass

Start - End	Observed	Mr(expt)	Mr(calc)	Delta	Miss	Sequence	
58 - 68	1485.5857	1484.5784	1484.6363	-0.0578	0	LIFHNSCFCCK	(No match)
58 - 68	1485.5857	1484.5784	1484.6363	-0.0578	0	LIFHNSCFCCK	(Ions score 50)

98 - 108	1229.5160	1228.5087	1228.5472	-0.0385	1	SKGNYDEGFGR	(No match)
98 - 108	1229.5160	1228.5087	1228.5472	-0.0385	1	SKGNYDEGFGR	(Ions score 40)
100 - 108	1014.3936	1013.3863	1013.4202	-0.0339	0	GNYDEGFGR	(Ions score 29)
100 - 108	1014.3936	1013.3863	1013.4202	-0.0339	0	GNYDEGFGR	(No match)
100 - 109	1142.4834	1141.4761	1141.5152	-0.0390	1	GNYDEGFGRK	(No match)
100 - 109	1142.4834	1141.4761	1141.5152	-0.0390	1	GNYDEGFGRK	(No match)

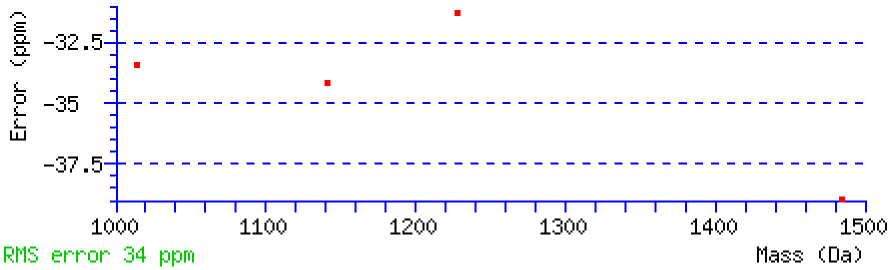

RMS error 34 ppm

Mascot: http://www.matrixscience.com/

Spot 837

Protein View

Match to: **gi|3355455** Score: **176** Expect: **4.8e-013**
R33729_1 [Homo sapiens]

Nominal mass (M_r): **11383**; Calculated pI value: **7.03**
NCBI BLAST search of gi|3355455 against nr
Unformatted sequence string for pasting into other applications

Taxonomy: Homo sapiens

Fixed modifications: Carbamidomethyl (C)
Variable modifications: Oxidation (M)
Cleavage by Trypsin: cuts C-term side of KR unless next residue is P
Sequence Coverage: **39%**

Matched peptides shown in **Bold Red**

```
 1 QQWQMSLGTS EDHQHFTCTI WRPQGKSYLY FTQFKAEVRG AEIEYAMAYS
51 KAAFERESDV PLKTEEFEVT KTAVAHRPGA FKAELSKLVI VAKASRTEL
```

Residue Number Increasing Mass Decreasing Mass

Start - End	Observed	Mr(expt)	Mr(calc)	Delta	Miss	Sequence
27 - 35	1196.5784	1195.5711	1195.5913	-0.0202	0	SYLYFTQFK (Ions score 55)
27 - 35	1196.5784	1195.5711	1195.5913	-0.0202	0	SYLYFTQFK (No match)
27 - 39	1651.8286	1650.8213	1650.8405	-0.0192	1	SYLYFTQFKAEVR (No match)
27 - 39	1651.8286	1650.8213	1650.8405	-0.0192	1	SYLYFTQFKAEVR (Ions score 42)
57 - 71	1750.8539	1749.8466	1749.8672	-0.0206	1	ESDVPLKTEEFEVTK (No match)
72 - 82	1154.6289	1153.6216	1153.6356	-0.0139	0	TAVAHRPGAFK (No match)
72 - 82	1154.6289	1153.6216	1153.6356	-0.0139	0	TAVAHRPGAFK (Ions score 9)

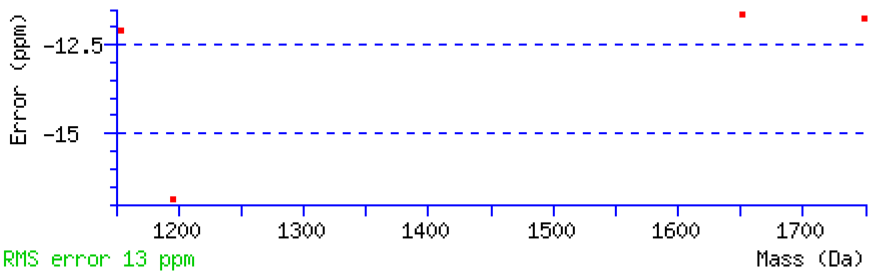

**Mascot:** http://www.matrixscience.com/

Spot 838

Protein View

Match to: **gi|119614587** Score: **115** Expect: **6.1e-007**
ubiquitin-conjugating enzyme E2D 4 (putative), isoform CRA_d [Homo sapiens]

Nominal mass (M_r): **9633**; Calculated pI value: **7.71**
NCBI BLAST search of gi|119614587 against nr
Unformatted sequence string for pasting into other applications

Taxonomy: Homo sapiens

Fixed modifications: Carbamidomethyl (C)
Variable modifications: Oxidation (M)
Cleavage by Trypsin: cuts C-term side of KR unless next residue is P
Sequence Coverage: **21%**

Matched peptides shown in **Bold Red**

1 MTVAFTTK**IY HPNINSNGSI CLDILR**SQWS PALTVSKVLL SICSLLCDPN
51 PDDPLVPEIA HTYKADREKY NRLAREWTQK YAM

Residue Number Increasing Mass Decreasing Mass

Start - End	Observed	Mr(expt)	Mr(calc)	Delta	Miss	Sequence	
9 - 26	2099.0105	2098.0032	2098.0629	-0.0597	0	IYHPNINSNGSICLDILR	(No match)
9 - 26	2099.0105	2098.0032	2098.0629	-0.0597	0	IYHPNINSNGSICLDILR	(Ions score 96)

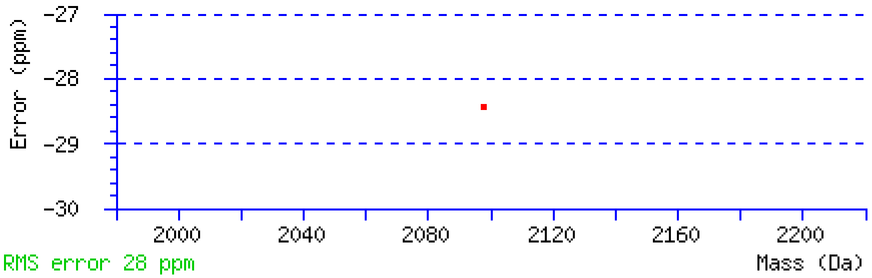

Spot 839

Protein View

Match to: **gi|83754516** Score: **111** Expect: **1.5e-006**
Chain B, Crystal Structure Of The Chip-Ubc13-Uev1a Complex

Nominal mass (M_r): **17268**; Calculated pI value: **6.18**
NCBI BLAST search of gi|83754516 against nr
Unformatted sequence string for pasting into other applications

Taxonomy: Homo sapiens
Links to retrieve other entries containing this sequence from NCBI Entrez:
gi|83754518 from Homo sapiens
gi|83754520 from Homo sapiens
gi|83754522 from Homo sapiens

Fixed modifications: Carbamidomethyl (C)
Variable modifications: Oxidation (M)
Cleavage by Trypsin: cuts C-term side of KR unless next residue is P
Sequence Coverage: **25%**

Matched peptides shown in **Bold Red**

```
  1 AGSAGLPRRI IKETQRLLAE PVPGIKAEPD ESNARYFHVV IAGPQDSPFE
 51 GGTFKLELFL PEEYPMAAPK VRFMTKIYHP NVDKLGRICL DILKDKWSPA
101 LQIRTVLLSI QALLSAPNPD DPLANDVAEQ WKTNEAQAIE TARAWTRLYA
151 MNNI
```

Residue Number Increasing Mass Decreasing Mass

Start - End	Observed	Mr(expt)	Mr(calc)	Delta	Miss	Sequence
17 - 35	2006.0118	2005.0045	2005.0479	-0.0434	1	LLAEPVPGIKAEPDESNAR (Ions score 38)
17 - 35	2006.0118	2005.0045	2005.0479	-0.0434	1	LLAEPVPGIKAEPDESNAR (No match)
95 - 104	1213.6509	1212.6436	1212.6615	-0.0178	1	DKWSPALQIR (No match)
133 - 143	1203.5801	1202.5728	1202.5891	-0.0163	0	TNEAQAIETAR (No match)
133 - 143	1203.5801	1202.5728	1202.5891	-0.0163	0	TNEAQAIETAR (Ions score 28)

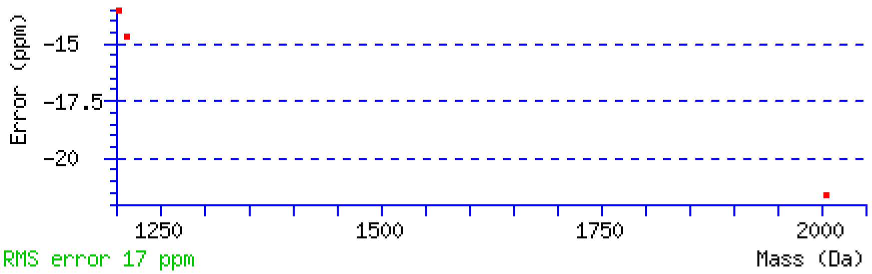

Mascot: http://www.matrixscience.com/

Spot 840

Protein View

Match to: **gi|4506923** Score: **196** Expect: **4.8e-015**
SH2 domain protein 1A [Homo sapiens]

Nominal mass (M_r): **14349;** Calculated pI value: **8.74**
NCBI BLAST search of gi|4506923 against nr
Unformatted sequence string for pasting into other applications

Taxonomy: Homo sapiens
Links to retrieve other entries containing this sequence from NCBI Entrez:
gi|74136235 from Macaca mulatta
gi|6094278 from Homo sapiens
gi|16974878 from Homo sapiens
gi|16974880 from Homo sapiens
gi|13021912 from Macaca mulatta
gi|3153108 from Homo sapiens
gi|3695069 from Homo sapiens
gi|3695071 from Homo sapiens
gi|3763971 from Homo sapiens
gi|18088434 from Homo sapiens
gi|49457015 from Homo sapiens
gi|49457039 from Homo sapiens
gi|119632253 from Homo sapiens
gi|123996977 from synthetic construct
gi|157928976 (no taxonomy information for this entry)

Fixed modifications: Carbamidomethyl (C)
Variable modifications: Oxidation (M)
Cleavage by Trypsin: cuts C-term side of KR unless next residue is P
Sequence Coverage: **60%**

Matched peptides shown in **Bold Red**

1 MDAVAVYHGK ISRETGEK**LL LATGLDGSYL LRDSESVPGV YCLCVLYHGY**
51 **IYTYR**VSQTE TGSWSAETAP GVHKRYFRKI K**NLISAFQKP DQGIVIPLQY**
101 **PVEK**KSSAR**S TQGTTGIRED PDVCLK**AP

Residue Number Increasing Mass Decreasing Mass

Start - End	Observed	Mr(expt)	Mr(calc)	Delta	Miss	Sequence
19 - 32	1504.8573	1503.8500	1503.8660	-0.0160	0	LLLATGLDGSYLLR (Ions score 77)
19 - 32	1504.8573	1503.8500	1503.8660	-0.0160	0	LLLATGLDGSYLLR (No match)
33 - 55	2814.2346	2813.2273	2813.2828	-0.0555	0	DSESVPGVYCLCVLYHGYIYTYR (No match)
33 - 55	2814.2346	2813.2273	2813.2828	-0.0555	0	DSESVPGVYCLCVLYHGYIYTYR (No match)
82 - 104	2597.4080	2596.4007	2596.4264	-0.0257	0	NLISAFQKPDQGIVIPLQYPVEK (Ions score 54)
82 - 104	2597.4080	2596.4007	2596.4264	-0.0257	0	NLISAFQKPDQGIVIPLQYPVEK (No match)
110 - 126	1876.8721	1875.8648	1875.8996	-0.0348	1	STQGTTGIREDPDVCLK (No match)
110 - 126	1876.8721	1875.8648	1875.8996	-0.0348	1	STQGTTGIREDPDVCLK (No match)

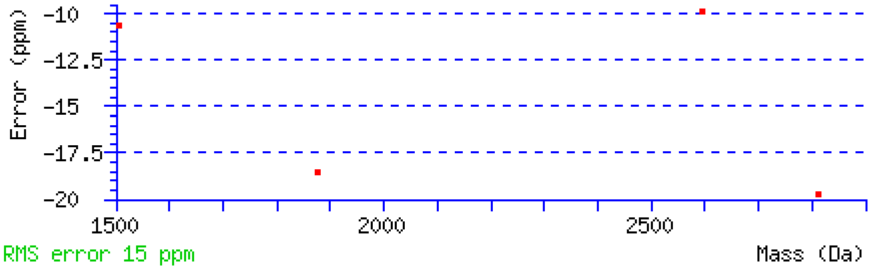

Mascot: http://www.matrixscience.com/

Spot 841

Protein View

Match to: **gi|56966036** Score: **255** Expect: **6.1e-021**
Chain A, Three Crystal Structures Of Human Coactosin-Like Protein

Nominal mass (M_r): **15898**; Calculated pI value: **5.55**
NCBI BLAST search of gi|56966036 against nr
Unformatted sequence string for pasting into other applications

Taxonomy: Homo sapiens
Links to retrieve other entries containing this sequence from NCBI Entrez:
gi|56966037 from Homo sapiens

Fixed modifications: Carbamidomethyl (C)
Variable modifications: Oxidation (M)
Cleavage by Trypsin: cuts C-term side of KR unless next residue is P
Sequence Coverage: **51%**

Matched peptides shown in **Bold Red**

```
  1 ATKIDKEACR AAYNLVRDDG SAVIWVTFKY DGSTIVPGEQ GAEYQHFIQQ
 51 CTDDVRLFAF VRFTTGDAXS KRSKFALITW IGENVSGLQR AKTGTDKTLV
101 KEVVQNFAKE FVISDRKELE EDFIKSELKK AGGANYDAQT E
```

Residue Number Increasing Mass Decreasing Mass

Start - End	Observed	Mr(expt)	Mr(calc)	Delta	Miss	Sequence
4 - 10	891.4254	890.4181	890.4280	-0.0098	1	IDKEACR (No match)
11 - 17	806.4432	805.4359	805.4446	-0.0087	0	AAYNLVR (No match)
30 - 56	3113.3281	3112.3208	3112.3832	-0.0623	0	YDGSTIVPGEQGAEYQHFIQQCTDDVR (No match)
30 - 56	3113.3281	3112.3208	3112.3832	-0.0623	0	YDGSTIVPGEQGAEYQHFIQQCTDDVR (Ions score 130)
75 - 90	1803.9448	1802.9375	1802.9678	-0.0303	0	FALITWIGENVSGLQR (No match)
102 - 109	934.4824	933.4751	933.4919	-0.0168	0	EVVQNFAK (No match)
110 - 117	993.5244	992.5171	992.5290	-0.0119	1	EFVISDRK (No match)
110 - 117	993.5244	992.5171	992.5290	-0.0119	1	EFVISDRK (Ions score 38)

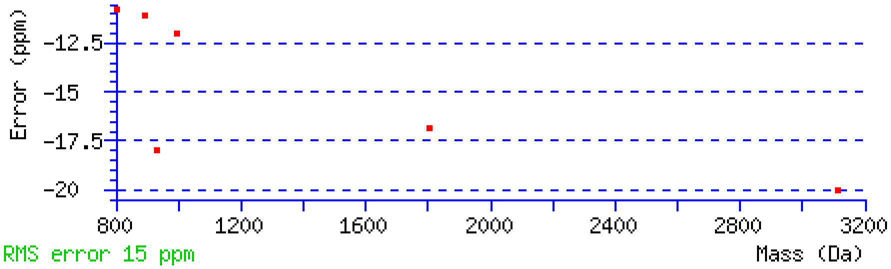

Mascot: http://www.matrixscience.com/

Spot 847

Protein View

Match to: **gi|5822002** Score: **373** Expect: **9.6e-033**
Chain A, Profilin Binds Proline-Rich Ligands In Two Distinct Amide Backbone Orientations

Nominal mass (M_r): **15085**; Calculated pI value: **8.48**
NCBI BLAST search of gi|5822002 against nr
Unformatted sequence string for pasting into other applications

Taxonomy: Homo sapiens
Links to retrieve other entries containing this sequence from NCBI Entrez:
gi|5822004 from Homo sapiens
gi|157831080 (no taxonomy information for this entry)
gi|157831081 (no taxonomy information for this entry)
gi|159795064 (no taxonomy information for this entry)
gi|160285766 (no taxonomy information for this entry)

Fixed modifications: Carbamidomethyl (C)
Variable modifications: Oxidation (M)
Cleavage by Trypsin: cuts C-term side of KR unless next residue is P
Sequence Coverage: **50%**

Matched peptides shown in **Bold Red**

```
  1 AGWNAYIDNL MADGTCQDAA IVGYKDSPSV WAAVPGKTFV NITPAEVGVL
 51 VGKDRSSFYV NGLTLGGQKC SVIRDSLLQD GEFSMDLRTK STGGAPTFNV
101 TVTKTDKTLV LLMGKEGVHG GLINKKCYEM ASHLRRSQY
```

Residue Number Increasing Mass Decreasing Mass

Start - End	Observed	Mr(expt)	Mr(calc)	Delta	Miss	Sequence
38 - 53	1643.9830	1642.9757	1642.9293	0.0464	0	TFVNITPAEVGVLVGK (No match)
38 - 55	1915.1304	1914.1231	1914.0574	0.0658	1	TFVNITPAEVGVLVGKDR (Ions score 111)
38 - 55	1915.1304	1914.1231	1914.0574	0.0658	1	TFVNITPAEVGVLVGKDR (No match)
54 - 69	1741.9470	1740.9397	1740.8794	0.0603	1	DRSSFYVNGLTLGGQK (No match)
56 - 69	1470.7905	1469.7832	1469.7514	0.0319	0	SSFYVNGLTLGGQK (No match)
56 - 69	1470.7905	1469.7832	1469.7514	0.0319	0	SSFYVNGLTLGGQK (Ions score 102)
75 - 88	1641.7908	1640.7835	1640.7352	0.0484	0	DSLLQDGEFSMDLR Oxidation (M) (Ions score 16)
75 - 88	1641.7908	1640.7835	1640.7352	0.0484	0	DSLLQDGEFSMDLR Oxidation (M) (No match)

91 - 104	1379.7428	1378.7355	1378.7092	0.0263	0	**STGGAPTFNVTVTK**	(Ions score 37)
91 - 104	1379.7428	1378.7355	1378.7092	0.0263	0	**STGGAPTFNVTVTK**	(No match)
127 - 135	1182.5101	1181.5028	1181.4957	0.0071	0	**CYEMASHLR**	Oxidation (M) (No match)
127 - 136	1338.6278	1337.6205	1337.5968	0.0237	1	**CYEMASHLRR**	Oxidation (M) (No match)

Mascot: http://www.matrixscience.com/

Spot 848

Protein View

Match to: **gi|5822002** Score: **505** Expect: **6.1e-046**
Chain A, Profilin Binds Proline-Rich Ligands In Two Distinct Amide Backbone Orientations

Nominal mass (M_r): **15085**; Calculated pI value: **8.48**
NCBI BLAST search of gi|5822002 against nr
Unformatted sequence string for pasting into other applications

Taxonomy: Homo sapiens
Links to retrieve other entries containing this sequence from NCBI Entrez:
gi|5822004 from Homo sapiens
gi|157831080 (no taxonomy information for this entry)
gi|157831081 (no taxonomy information for this entry)
gi|159795064 (no taxonomy information for this entry)
gi|160285766 (no taxonomy information for this entry)

Fixed modifications: Carbamidomethyl (C)
Variable modifications: Oxidation (M)
Cleavage by Trypsin: cuts C-term side of KR unless next residue is P
Sequence Coverage: **59%**

Matched peptides shown in **Bold Red**

```
  1 AGWNAYIDNL MADGTCQDAA IVGYKDSPSV WAAVPGKTFV NITPAEVGVL
 51 VGKDRSSFYV NGLTLGGQKC SVIRDSLLQD GEFSMDLRTK STGGAPTFNV
101 TVTKTDKTLV LLMGKEGVHG GLINKKCYEM ASHLRRSQY
```

Residue Number Increasing Mass Decreasing Mass

Start - End	Observed	Mr(expt)	Mr(calc)	Delta	Miss	Sequence
26 - 37	1213.6389	1212.6316	1212.6138	0.0178	0	DSPSVWAAVPGK (No match)
38 - 53	1643.9662	1642.9589	1642.9293	0.0296	0	TFVNITPAEVGVLVGK (No match)
38 - 55	1915.0969	1914.0896	1914.0574	0.0323	1	TFVNITPAEVGVLVGKDR (No match)
38 - 55	1915.0969	1914.0896	1914.0574	0.0323	1	TFVNITPAEVGVLVGKDR (Ions score 126)
54 - 69	1741.9216	1740.9143	1740.8794	0.0349	1	DRSSFYVNGLTLGGQK (No match)
56 - 69	1470.7832	1469.7759	1469.7514	0.0246	0	SSFYVNGLTLGGQK (Ions score 123)
56 - 69	1470.7832	1469.7759	1469.7514	0.0246	0	SSFYVNGLTLGGQK (No match)
75 - 88	1625.7821	1624.7748	1624.7403	0.0346	0	DSLLQDGEFSMDLR (No match)

75 - 88	1625.7821	1624.7748	1624.7403	0.0346	0	DSLLQDGEFSMDLR (Ions score 76)
75 - 88	1641.7738	1640.7665	1640.7352	0.0314	0	DSLLQDGEFSMDLR Oxidation (M) (No match)
91 - 104	1379.7389	1378.7316	1378.7092	0.0224	0	STGGAPTFNVTVTK (No match)
126 - 135	1294.6339	1293.6266	1293.5958	0.0308	1	KCYEMASHLR (No match)
127 - 135	1166.5332	1165.5259	1165.5008	0.0251	0	CYEMASHLR (No match)
127 - 135	1166.5332	1165.5259	1165.5008	0.0251	0	CYEMASHLR (Ions score 40)
127 - 135	1182.5225	1181.5152	1181.4957	0.0195	0	CYEMASHLR Oxidation (M) (No match)
127 - 136	1322.6321	1321.6248	1321.6019	0.0229	1	CYEMASHLRR (No match)
127 - 136	1338.6245	1337.6172	1337.5968	0.0204	1	CYEMASHLRR Oxidation (M) (No match)

Mascot: http://www.matrixscience.com/

Spot 849

Protein View

Match to: **gi|5822002** Score: **287** Expect: **3.8e-024**
Chain A, Profilin Binds Proline-Rich Ligands In Two Distinct Amide Backbone Orientations

Nominal mass (M_r): **15085;** Calculated pI value: **8.48**
NCBI BLAST search of gi|5822002 against nr
Unformatted sequence string for pasting into other applications

Taxonomy: Homo sapiens
Links to retrieve other entries containing this sequence from NCBI Entrez:
gi|5822004 from Homo sapiens
gi|157831080 (no taxonomy information for this entry)
gi|157831081 (no taxonomy information for this entry)
gi|159795064 (no taxonomy information for this entry)
gi|160285766 (no taxonomy information for this entry)

Fixed modifications: Carbamidomethyl (C)
Variable modifications: Oxidation (M)
Cleavage by Trypsin: cuts C-term side of KR unless next residue is P
Sequence Coverage: **58%**

Matched peptides shown in **Bold Red**

```
  1 AGWNAYIDNL MADGTCQDAA IVGYKDSPSV WAAVPGKTFV NITPAEVGVL
 51 VGKDRSSFYV NGLTLGGQKC SVIRDSLLQD GEFSMDLRTK STGGAPTFNV
101 TVTKTDKTLV LLMGKEGVHG GLINKKCYEM ASHLRRSQY
```

Residue Number Increasing Mass Decreasing Mass

Start - End	Observed	Mr(expt)	Mr(calc)	Delta	Miss	Sequence
38 - 55	1915.0240	1914.0167	1914.0574	-0.0406	1	TFVNITPAEVGVLVGKDR (No match)
38 - 55	1915.0240	1914.0167	1914.0574	-0.0406	1	TFVNITPAEVGVLVGKDR (Ions score 121)
56 - 69	1470.7238	1469.7165	1469.7514	-0.0348	0	SSFYVNGLTLGGQK (No match)
75 - 88	1625.7150	1624.7077	1624.7403	-0.0325	0	DSLLQDGEFSMDLR (No match)
75 - 88	1641.7067	1640.6994	1640.7352	-0.0357	0	DSLLQDGEFSMDLR Oxidation (M) (Ions score 23)
75 - 88	1641.7067	1640.6994	1640.7352	-0.0357	0	DSLLQDGEFSMDLR Oxidation (M) (No match)
91 - 104	1379.6876	1378.6803	1378.7092	-0.0289	0	STGGAPTFNVTVTK (No match)
116 - 126	1151.6868	1150.6795	1150.6458	0.0338	1	EGVHGGLINKK (No match)

127 - 135	1182.4752	1181.4679	1181.4957	-0.0278	0	CYEMASHLR	Oxidation (M) (Ions score 28)
127 - 135	1182.4752	1181.4679	1181.4957	-0.0278	0	CYEMASHLR	Oxidation (M) (No match)
127 - 136	1338.5743	1337.5670	1337.5968	-0.0298	1	CYEMASHLRR	Oxidation (M) (No match)

Mascot: http://www.matrixscience.com/

Spot 851

Protein View

Match to: **gi|42542977** Score: **440** Expect: **1.9e-039**
Chain A, X-Ray Crystal Structure Of Human Galectin-1

Nominal mass (M_r): **14868**; Calculated pI value: **5.34**
NCBI BLAST search of gi|42542977 against nr
Unformatted sequence string for pasting into other applications

Taxonomy: Homo sapiens

Fixed modifications: Carbamidomethyl (C)
Variable modifications: Oxidation (M)
Cleavage by Trypsin: cuts C-term side of KR unless next residue is P
Sequence Coverage: **61%**

Matched peptides shown in **Bold Red**

```
  1 ACGLVASNLN LKPGEXLRVR GEVAPDAKSF VLNLGKDSNN LCLHFNPRFN
 51 AHGDANTIVC NSKDGGAWGT EQREAVFPFQ PGSVAEVCIT FDQANLTVKL
101 PDGYEFKFPN RLNLEAINYM AADGDFKIKC VAFD
```

Residue Number Increasing Mass Decreasing Mass

Start - End	Observed	Mr(expt)	Mr(calc)	Delta	Miss	Sequence
19 - 28	1041.5531	1040.5458	1040.5614	-0.0156	1	VRGEVAPDAK (Ions score 24)
19 - 28	1041.5531	1040.5458	1040.5614	-0.0156	1	VRGEVAPDAK (No match)
29 - 48	2345.1301	2344.1228	2344.1745	-0.0517	1	SFVLNLGKDSNNLCLHFNPR (No match)
37 - 48	1486.6549	1485.6476	1485.6783	-0.0306	0	DSNNLCLHFNPR (Ions score 101)
37 - 48	1486.6549	1485.6476	1485.6783	-0.0306	0	DSNNLCLHFNPR (No match)
49 - 63	1647.7169	1646.7096	1646.7470	-0.0374	0	FNAHGDANTIVCNSK (Ions score 138)
49 - 63	1647.7169	1646.7096	1646.7470	-0.0374	0	FNAHGDANTIVCNSK (No match)
64 - 73	1076.4584	1075.4511	1075.4682	-0.0171	0	DGGAWGTEQR (Ions score 51)
64 - 73	1076.4584	1075.4511	1075.4682	-0.0171	0	DGGAWGTEQR (No match)
100 - 107	968.4539	967.4466	967.4650	-0.0184	0	LPDGYEFK (No match)
100 - 111	1482.7075	1481.7002	1481.7302	-0.0300	1	LPDGYEFKFPNR (No match)
112 - 127	1800.8046	1799.7973	1799.8399	-0.0426	0	LNLEAINYMAADGDFK Oxidation (M) (No match)

Spot 852

Protein View

Match to: **gi|4885413** Score: **193** Expect: **9.6e-015**
histidine triad nucleotide binding protein 1 [Homo sapiens]

Nominal mass (M_r): **13907**; Calculated pI value: **6.43**
NCBI BLAST search of gi|4885413 against nr
Unformatted sequence string for pasting into other applications

Taxonomy: Homo sapiens
Links to retrieve other entries containing this sequence from NCBI Entrez:
gi|1708543 from Homo sapiens
gi|862933 from Homo sapiens
gi|1256265 from Homo sapiens
gi|12654887 from Homo sapiens
gi|13937954 from Homo sapiens
gi|48146213 from Homo sapiens
gi|119582780 from Homo sapiens

Fixed modifications: Carbamidomethyl (C)
Variable modifications: Oxidation (M)
Cleavage by Trypsin: cuts C-term side of KR unless next residue is P
Sequence Coverage: **46%**

Matched peptides shown in **Bold Red**

1 MADEIAK**AQV ARPGGDTIFG K**IIRKEIPAK IIFEDDR**CLA FHDISPQAPT**
51 **HFLVIPK**KHI SQISVAEDDD ESLLGHLMIV GKKCAADLGL NKGYR**MVVNE**
101 **GSDGGQSVYH VHLHVLGGR**Q MHWPPG

Residue Number Increasing Mass Decreasing Mass

Start - End	Observed	Mr(expt)	Mr(calc)	Delta	Miss	Sequence
8 - 21	1416.7358	1415.7285	1415.7520	-0.0235	0	AQVARPGGDTIFGK (No match)
8 - 21	1416.7358	1415.7285	1415.7520	-0.0235	0	AQVARPGGDTIFGK (Ions score 26)
38 - 57	2291.1438	2290.1365	2290.1932	-0.0567	0	CLAFHDISPQAPTHFLVIPK (No match)
38 - 57	2291.1438	2290.1365	2290.1932	-0.0567	0	CLAFHDISPQAPTHFLVIPK (Ions score 54)
96 - 119	2563.1953	2562.1880	2562.2396	-0.0516	0	MVVNEGSDGGQSVYHVHLHVLGGR Oxidation (M) (No match)
96 - 119	2563.1953	2562.1880	2562.2396	-0.0516	0	MVVNEGSDGGQSVYHVHLHVLGGR Oxidation (M) (Ions score 55)

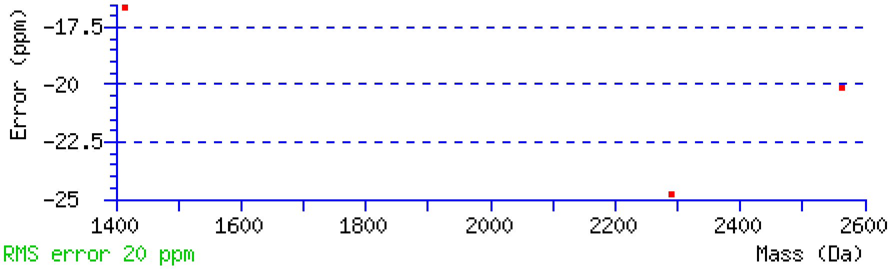

Mascot: http://www.matrixscience.com/

Spot 856

Protein View

Match to: **gi|5107718** Score: **113** Expect: **9.6e-007**
Chain A, The Structure Of The Immunophilin-Immunosuppressant Fkbp12- (C16)-Ethoxy Rapamycin Complex

Nominal mass (M_r): **11869**; Calculated pI value: **8.07**
NCBI BLAST search of gi|5107718 against nr
Unformatted sequence string for pasting into other applications

Taxonomy: Homo sapiens
Links to retrieve other entries containing this sequence from NCBI Entrez:
gi|5542074 from Homo sapiens
gi|5542076 from Homo sapiens
gi|5542078 from Homo sapiens
gi|5542080 from Homo sapiens
gi|5822314 from Homo sapiens
gi|5822315 from Homo sapiens
gi|5822316 from Homo sapiens
gi|5822317 from Homo sapiens
gi|6435613 from Homo sapiens
gi|6435614 from Homo sapiens
gi|6435615 from Homo sapiens
gi|6435616 from Homo sapiens
gi|6435617 from Homo sapiens
gi|6435618 from Homo sapiens
gi|6435619 from Homo sapiens
gi|6435620 from Homo sapiens
gi|10835851 from Homo sapiens
gi|10835853 from Homo sapiens
gi|18158938 from Homo sapiens
gi|18158939 from Homo sapiens
gi|18158940 from Homo sapiens
gi|31615448 from Homo sapiens
gi|31615449 from Homo sapiens
gi|99032085 from Homo sapiens
gi|157831086 (no taxonomy information for this entry)
gi|157831087 (no taxonomy information for this entry)
gi|157831090 (no taxonomy information for this entry)
gi|157834963 (no taxonomy information for this entry)
gi|159162287 (no taxonomy information for this entry)
gi|159162315 (no taxonomy information for this entry)
gi|159162316 (no taxonomy information for this entry)
gi|159162317 (no taxonomy information for this entry)

Fixed modifications: Carbamidomethyl (C)
Variable modifications: Oxidation (M)
Cleavage by Trypsin: cuts C-term side of KR unless next residue is P
Sequence Coverage: **41%**

Matched peptides shown in **Bold Red**

1 **GVQVETISPG DGR**TFPK**RGQ TCVVHYTGML EDGK**KFDSSR DRNKPFKFML
51 GKQEVIR**GWE EGVAQMSVGQ R**AKLTISPDY AYGATGHPGI IPPHATLVFD
101 VELLKLE

Residue Number Increasing Mass Decreasing Mass

Start - End	Observed	Mr(expt)	Mr(calc)	Delta	Miss	Sequence
1 - 13	1314.6469	1313.6396	1313.6575	-0.0178	0	GVQVETISPGDGR (Ions score 31)
1 - 13	1314.6469	1313.6396	1313.6575	-0.0178	0	GVQVETISPGDGR (No match)
18 - 34	1966.8721	1965.8648	1965.9036	-0.0388	1	RGQTCVVHYTGMLEDGK Oxidation (M) (No match)
18 - 34	1966.8721	1965.8648	1965.9036	-0.0388	1	RGQTCVVHYTGMLEDGK Oxidation (M) (Ions score 25)
58 - 71	1549.6755	1548.6682	1548.6990	-0.0308	0	GWEEGVAQMSVGQR Oxidation (M) (No match)
58 - 71	1549.6755	1548.6682	1548.6990	-0.0308	0	GWEEGVAQMSVGQR Oxidation (M) (Ions score 12)

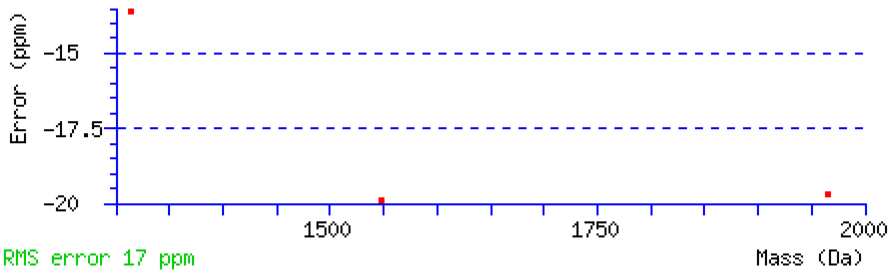

RMS error 17 ppm

Spot 857

Protein View

Match to: **gi|4503117** Score: **122** Expect: **1.2e-007**
cystatin B [Homo sapiens]

Nominal mass (M_r): **11190**; Calculated pI value: **6.96**
NCBI BLAST search of gi|4503117 against nr
Unformatted sequence string for pasting into other applications

Taxonomy: Homo sapiens
Links to retrieve other entries containing this sequence from NCBI Entrez:
gi|1706278 from Homo sapiens
gi|45476793 from Pan paniscus
gi|45476794 from Pongo pygmaeus
gi|52000705 from Gorilla gorilla gorilla
gi|7263012 from Homo sapiens
gi|291927 from Homo sapiens
gi|1235678 from Homo sapiens
gi|7768760 from Homo sapiens
gi|13097210 from Homo sapiens
gi|14714769 from Homo sapiens
gi|23496466 from Homo sapiens
gi|23496470 from Pan paniscus
gi|23496472 from Gorilla gorilla
gi|23496474 from Pongo pygmaeus
gi|30582919 from Homo sapiens
gi|49457292 from Homo sapiens
gi|61360774 from synthetic construct
gi|119629878 from Homo sapiens
gi|119629879 from Homo sapiens
gi|123980040 from synthetic construct
gi|123994795 from synthetic construct

Fixed modifications: Carbamidomethyl (C)
Variable modifications: Oxidation (M)
Cleavage by Trypsin: cuts C-term side of KR unless next residue is P
Sequence Coverage: **33%**

Matched peptides shown in **Bold Red**

1 MMCGAPSATQ PATAETQHIA DQVRSQLEEK ENKKFPVFKA VSFKSQVVAG
51 TNYFIK**VHVG DEDFVHLRVF QSLPHENKPL TLSNYQTNK**A KHDELTYF

Residue Number Increasing Mass Decreasing Mass

Start - End	Observed	Mr(expt)	Mr(calc)	Delta	Miss	Sequence	
57 - 68	1422.7156	1421.7083	1421.7051	0.0032	0	VHVGDEDFVHLR	(Ions score 30)
57 - 68	1422.7156	1421.7083	1421.7051	0.0032	0	VHVGDEDFVHLR	(No match)
69 - 89	2458.2605	2457.2532	2457.2652	-0.0119	0	VFQSLPHENKPLTLSNYQTNK	(No match)
69 - 89	2458.2605	2457.2532	2457.2652	-0.0119	0	VFQSLPHENKPLTLSNYQTNK	(Ions score 59)

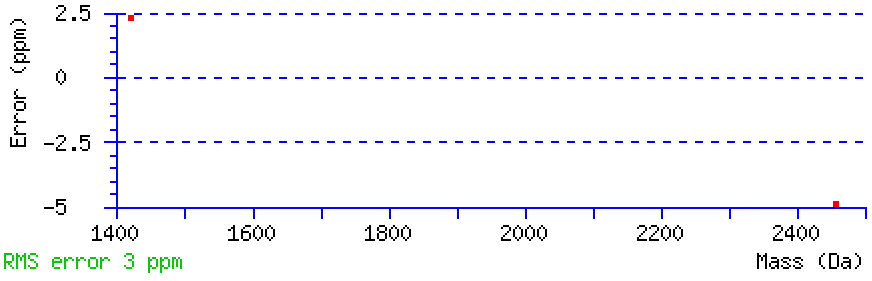

RMS error 3 ppm

Mascot: http://www.matrixscience.com/

Spot 862

Protein View

Match to: **gi|229995** Score: **232** Expect: **1.2e-018**
Chain M, Structure Of The Human Class I Histocompatibility Antigen, Hla-A2

Nominal mass (M_r): **11592**; Calculated pI value: **6.46**
NCBI BLAST search of gi|229995 against nr
Unformatted sequence string for pasting into other applications

Taxonomy: Homo sapiens

Fixed modifications: Carbamidomethyl (C)
Variable modifications: Oxidation (M)
Cleavage by Trypsin: cuts C-term side of KR unless next residue is P
Sequence Coverage: **72%**

Matched peptides shown in **Bold Red**

```
 1 IQRTPKIQVY SRHPAENGKS NFLNCYVSGF HPSDIEVDLL KNGERIEKVE
51 HSDLSFSKDW SFYLLYYTEF TPTEKDEYAC RVNHVTLSQP KIVKWDR
```

Residue Number Increasing Mass Decreasing Mass

Start - End	Observed	Mr(expt)	Mr(calc)	Delta	Miss	Sequence
7 - 19	1498.7931	1497.7858	1497.7688	0.0171	1	IQVYSRHPAENGK (No match)
20 - 41	2554.2617	2553.2544	2553.2209	0.0335	0	SNFLNCYVSGFHPSDIEVDLLK (No match)
20 - 41	2554.2617	2553.2544	2553.2209	0.0335	0	SNFLNCYVSGFHPSDIEVDLLK (Ions score 77)
46 - 58	1518.7927	1517.7854	1517.7725	0.0129	1	IEKVEHSDLSFSK (No match)
49 - 58	1148.5693	1147.5620	1147.5509	0.0111	0	VEHSDLSFSK (No match)
76 - 81	813.3341	812.3268	812.3123	0.0146	0	DEYACR (No match)
82 - 91	1122.6345	1121.6272	1121.6193	0.0080	0	VNHVTLSQPK (Ions score 47)
82 - 91	1122.6345	1121.6272	1121.6193	0.0080	0	VNHVTLSQPK (No match)
92 - 97	816.4868	815.4795	815.4653	0.0142	1	IVKWDR (No match)

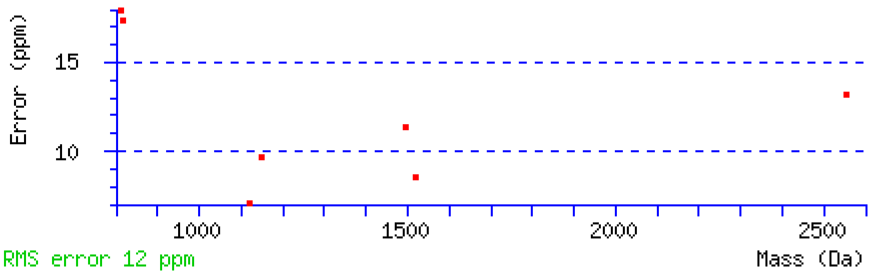

Mascot: http://www.matrixscience.com/

Spot 867

Protein View

Match to: **gi|46577124** Score: **102** Expect: **1.2e-005**
BolA-like protein 2

Nominal mass (M_r): **10281;** Calculated pI value: **6.07**
NCBI BLAST search of gi|46577124 against nr
Unformatted sequence string for pasting into other applications

Taxonomy: Homo sapiens
Links to retrieve other entries containing this sequence from NCBI Entrez:
gi|12001972 from Homo sapiens

Fixed modifications: Carbamidomethyl (C)
Variable modifications: Oxidation (M)
Cleavage by Trypsin: cuts C-term side of KR unless next residue is P
Sequence Coverage: **29%**

Matched peptides shown in **Bold Red**

```
 1 MELSAEYLRE KLQRDLEAEH VEVEDTTLNR CSCSFRVLVV SAKFEGKPLL
51 QRHRLVNACL AEELPHIHAF EQKTLTPDQW ARERQK
```

Residue Number Increasing Mass Decreasing Mass

Start - End	Observed	Mr(expt)	Mr(calc)	Delta	Miss	Sequence
37 - 52	1784.0112	1783.0039	1783.0719	-0.0680	1	VLVVSAKFEGKPLLQR (No match)
37 - 52	1784.0112	1783.0039	1783.0719	-0.0680	1	VLVVSAKFEGKPLLQR (Ions score 45)
44 - 52	1087.5824	1086.5751	1086.6185	-0.0434	0	FEGKPLLQR (Ions score 13)
74 - 82	1087.5824	1086.5751	1086.5458	0.0293	0	TLTPDQWAR (No match)

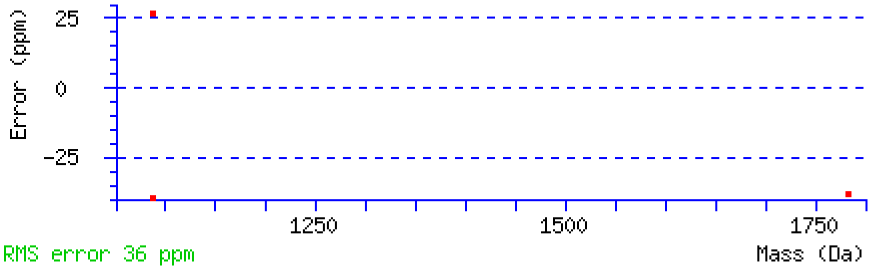

Mascot: http://www.matrixscience.com/

Spot 868

Protein View

Match to: **gi|840771** Score: **68** Expect: **0.028**
leucocyte antigen CD97 [Homo sapiens]

Nominal mass (M_r): **83872;** Calculated pI value: **6.72**
NCBI BLAST search of gi|840771 against nr
Unformatted sequence string for pasting into other applications

Taxonomy: Homo sapiens

Fixed modifications: Carbamidomethyl (C)
Variable modifications: Oxidation (M)
Cleavage by Trypsin: cuts C-term side of KR unless next residue is P
Sequence Coverage: **4%**

Matched peptides shown in **Bold Red**

```
  1 MGGRVFLAFC VWLTLPGAET QDSRGCARWC PQNSSCVNAT ACRCNPGFSS
 51 FSEIITTPTE TCDDINECAT PSKVSCGKFS DCWNTEGSYD CVCSPGYEPV
101 SGAKTFKNES ENTCQDVDEC SSGQHQCDSS TVCFNTVGSY SCRCRPGWKP
151 RHGIPNNQKD TVCEDMTFST WTPPPGVHSQ TLSRFFDKVQ DLGRDSKTSS
201 AEVTIQNVIK LVDELMEAPG DVEALAPPVR HLIATQLLSN LEDIMRILAK
251 SLPKGPFTYI SPSNTELTLM IQERGDKNVT MGQSSARMKL NWAVAAGAED
301 PGPAVAGILS IQNMTTLLAN ASLNLHSKKQ AELEEIYESS IRGVQLRRLS
351 AVNSIFLSHN NTKELNSPIL FAFSHLESSD GEAGRDPPAK DVMPGPRQEL
401 LCAFWKSDSD RGGHWATEVC QVLGSKNGST TCQCSHLSSF TILMAHYDVE
451 DWKLTLITRV GLALSLFCLL LCILTFLLVR PIQGSRTTIH LHLCICLFVG
501 STIFLAGIEN EGGQVGLRCR LVAGLLHYCF LAAFCWMSLE GLELYFLVVR
551 VFQGQGLSTR WLCLIGYGVP LLIVGVSAAI YSKGYGRPRY CWLDFEQGFL
601 WSFLGPVTFI ILCNAVIFVT TVWKLTQKFS EINPDMKKLK KARALTITAI
651 AQLFLLGCTW VFGLFIFDDR SLVLTYVFTI LNCLQGAFLY LLHCLLNKKV
701 REEYRKWACL VAGGSKYSEF TSTTSGTGHN QTRALRASES GI
```

Residue Number Increasing Mass Decreasing Mass

Start - End	Observed	Mr(expt)	Mr(calc)	Delta	Miss	Sequence
185 - 194	1224.6145	1223.6072	1223.6298	-0.0226	1	FFDKVQDLGR (Ions score 16)
329 - 342	1694.8289	1693.8216	1693.8522	-0.0306	1	KQAELEEIYESSIR (No match)

329 - 342	1694.8289	1693.8216	1693.8522	-0.0306	1	**KQAELEEIYESSIR**	(Ions score 22)
629 - 638	1224.6145	1223.6072	1223.5856	0.0217	1	**FSEINPDMKK**	Oxidation (M) (No match)

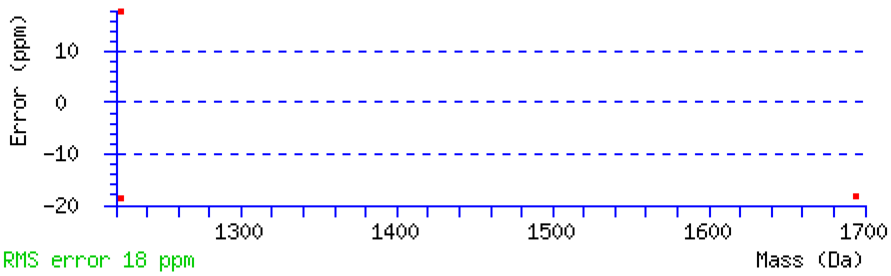

Mascot: http://www.matrixscience.com/

Spot 871

Protein View

Match to: **gi|840771** Score: **89** Expect: **0.00022**
leucocyte antigen CD97 [Homo sapiens]

Nominal mass (M_r): **83872;** Calculated pI value: **6.72**
NCBI BLAST search of gi|840771 against nr
Unformatted sequence string for pasting into other applications

Taxonomy: Homo sapiens

Fixed modifications: Carbamidomethyl (C)
Variable modifications: Oxidation (M)
Cleavage by Trypsin: cuts C-term side of KR unless next residue is P
Sequence Coverage: **4%**

Matched peptides shown in **Bold Red**

```
  1 MGGRVFLAFC VWLTLPGAET QDSRGCARWC PQNSSCVNAT ACRCNPGFSS
 51 FSEIITTPTE TCDDINECAT PSKVSCGKFS DCWNTEGSYD CVCSPGYEPV
101 SGAKTFKNES ENTCQDVDEC SSGQHQCDSS TVCFNTVGSY SCRCRPGWKP
151 RHGIPNNQKD TVCEDMTFST WTPPPGVHSQ TLSRFFDKVQ DLGRDSKTSS
201 AEVTIQNVIK LVDELMEAPG DVEALAPPVR HLIATQLLSN LEDIMRILAK
251 SLPKGPFTYI SPSNTELTLM IQERGDKNVT MGQSSARMKL NWAVAAGAED
301 PGPAVAGILS IQNMTTLLAN ASLNLHSKKQ AELEEIYESS IRGVQLRRLS
351 AVNSIFLSHN NTKELNSPIL FAFSHLESSD GEAGRDPPAK DVMPGPRQEL
401 LCAFWKSDSD RGGHWATEVC QVLGSKNGST TCQCSHLSSF TILMAHYDVE
451 DWKLTLITRV GLALSLFCLL LCILTFLLVR PIQGSRTTIH LHLCICLFVG
501 STIFLAGIEN EGGQVGLRCR LVAGLLHYCF LAAFCWMSLE GLELYFLVVR
551 VFQGQGLSTR WLCLIGYGVP LLIVGVSAAI YSKGYGRPRY CWLDFEQGFL
601 WSFLGPVTFI ILCNAVIFVT TVWKLTQKFS EINPDMKKLK KARALTITAI
651 AQLFLLGCTW VFGLFIFDDR SLVLTYVFTI LNCLQGAFLY LLHCLLNKKV
701 REEYRKWACL VAGGSKYSEF TSTTSGTGHN QTRALRASES GI
```

Residue Number Increasing Mass Decreasing Mass

Start - End	Observed	Mr(expt)	Mr(calc)	Delta	Miss	Sequence
185 - 194	1224.6095	1223.6022	1223.6298	-0.0276	1	FFDKVQDLGR (Ions score 29)
329 - 342	1694.8215	1693.8142	1693.8522	-0.0380	1	KQAELEEIYESSIR (No match)

329 - 342	1694.8215	1693.8142	1693.8522	-0.0380	1	KQAELEEIYESSIR (Ions score 30)
629 - 638	1224.6095	1223.6022	1223.5856	0.0167	1	FSEINPDMKK Oxidation (M) (No match)

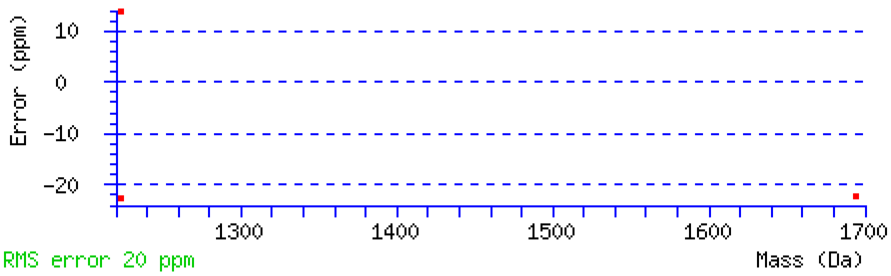

Mascot: http://www.matrixscience.com/

Spot 872

Protein View

Match to: **gi|840771** Score: **236** Expect: **4.8e-019**
leucocyte antigen CD97 [Homo sapiens]

Nominal mass (M_r): **83872**; Calculated pI value: **6.72**
NCBI BLAST search of gi|840771 against nr
Unformatted sequence string for pasting into other applications

Taxonomy: Homo sapiens

Fixed modifications: Carbamidomethyl (C)
Variable modifications: Oxidation (M)
Cleavage by Trypsin: cuts C-term side of KR unless next residue is P
Sequence Coverage: **9%**

Matched peptides shown in **Bold Red**

```
  1 MGGRVFLAFC VWLTLPGAET QDSRGCARWC PQNSSCVNAT ACRCNPGFSS
 51 FSEIITTPTE TCDDINECAT PSKVSCGKFS DCWNTEGSYD CVCSPGYEPV
101 SGAKTFKNES ENTCQDVDEC SSGQHQCDSS TVCFNTVGSY SCRCRPGWKP
151 RHGIPNNQKD TVCEDMTFST WTPPPGVHSQ TLSRFFDKVQ DLGRDSKTSS
201 AEVTIQNVIK LVDELMEAPG DVEALAPPVR HLIATQLLSN LEDIMRILAK
251 SLPKGPFTYI SPSNTELTLM IQERGDKNVT MGQSSARMKL NWAVAAGAED
301 PGPAVAGILS IQNMTTLLAN ASLNLHSKKQ AELEEIYESS IRGVQLRRLS
351 AVNSIFLSHN NTKELNSPIL FAFSHLESSD GEAGRDPPAK DVMPGPRQEL
401 LCAFWKSDSD RGGHWATEVC QVLGSKNGST TCQCSHLSSF TILMAHYDVE
451 DWKLTLITRV GLALSLFCLL LCILTFLLVR PIQGSRTTIH LHLCICLFVG
501 STIFLAGIEN EGGQVGLRCR LVAGLLHYCF LAAFCWMSLE GLELYFLVVR
551 VFQGQGLSTR WLCLIGYGVP LLIVGVSAAI YSKGYGRPRY CWLDFEQGFL
601 WSFLGPVTFI ILCNAVIFVT TVWKLTQKFS EINPDMKKLK KARALTITAI
651 AQLFLLGCTW VFGLFIFDDR SLVLTYVFTI LNCLQGAFLY LLHCLLNKKV
701 REEYRKWACL VAGGSKYSEF TSTTSGTGHN QTRALRASES GI
```

Residue Number Increasing Mass Decreasing Mass

Start - End	Observed	Mr(expt)	Mr(calc)	Delta	Miss	Sequence
185 - 194	1224.5933	1223.5860	1223.6298	-0.0438	1	**FFDKVQDLGR** (Ions score 48)
211 - 230	2137.0242	2136.0169	2136.0772	-0.0603	0	**LVDELMEAPGDVEALAPPVR** Oxidation (M) (No match)

211 - 230	2137.0242	2136.0169	2136.0772	-0.0603	0	**LVDELMEAPGDVEALAPPVR**	Oxidation (M) (Ions score 46)
231 - 246	1882.9485	1881.9412	1881.9982	-0.0570	0	**HLIATQLLSNLEDIMR**	Oxidation (M) (No match)
231 - 246	1882.9485	1881.9412	1881.9982	-0.0570	0	**HLIATQLLSNLEDIMR**	Oxidation (M) (Ions score 24)
329 - 342	1694.8071	1693.7998	1693.8522	-0.0524	1	**KQAELEEIYESSIR**	(No match)
329 - 342	1694.8071	1693.7998	1693.8522	-0.0524	1	**KQAELEEIYESSIR**	(Ions score 64)
629 - 638	1224.5933	1223.5860	1223.5856	0.0005	1	**FSEINPDMKK**	Oxidation (M) (No match)

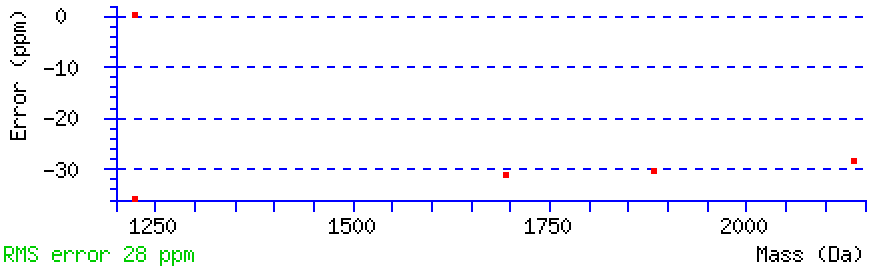

Mascot: http://www.matrixscience.com/

Spot 873

Protein View

Match to: **gi|840771** Score: **75** Expect: **0.0065**
leucocyte antigen CD97 [Homo sapiens]

Nominal mass (M_r): **83872;** Calculated pI value: **6.72**
NCBI BLAST search of gi|840771 against nr
Unformatted sequence string for pasting into other applications

Taxonomy: Homo sapiens

Fixed modifications: Carbamidomethyl (C)
Variable modifications: Oxidation (M)
Cleavage by Trypsin: cuts C-term side of KR unless next residue is P
Sequence Coverage: **7%**

Matched peptides shown in **Bold Red**

```
  1 MGGRVFLAFC VWLTLPGAET QDSRGCARWC PQNSSCVNAT ACRCNPGFSS
 51 FSEIITTPTE TCDDINECAT PSKVSCGKFS DCWNTEGSYD CVCSPGYEPV
101 SGAKTFKNES ENTCQDVDEC SSGQHQCDSS TVCFNTVGSY SCRCRPGWKP
151 RHGIPNNQKD TVCEDMTFST WTPPPGVHSQ TLSRFFDKVQ DLGRDSKTSS
201 AEVTIQNVIK LVDELMEAPG DVEALAPPVR HLIATQLLSN LEDIMRILAK
251 SLPKGPFTYI SPSNTELTLM IQERGDKNVT MGQSSARMKL NWAVAAGAED
301 PGPAVAGILS IQNMTTLLAN ASLNLHSKKQ AELEEIYESS IRGVQLRRLS
351 AVNSIFLSHN NTKELNSPIL FAFSHLESSD GEAGRDPPAK DVMPGPRQEL
401 LCAFWKSDSD RGGHWATEVC QVLGSKNGST TCQCSHLSSF TILMAHYDVE
451 DWKLTLITRV GLALSLFCLL LCILTFLLVR PIQGSRTTIH LHLCICLFVG
501 STIFLAGIEN EGGQVGLRCR LVAGLLHYCF LAAFCWMSLE GLELYFLVVR
551 VFQGQGLSTR WLCLIGYGVP LLIVGVSAAI YSKGYGRPRY CWLDFEQGFL
601 WSFLGPVTFI ILCNAVIFVT TVWKLTQKFS EINPDMKKLK KARALTITAI
651 AQLFLLGCTW VFGLFIFDDR SLVLTYVFTI LNCLQGAFLY LLHCLLNKKV
701 REEYRKWACL VAGGSKYSEF TSTTSGTGHN QTRALRASES GI
```

Residue Number Increasing Mass Decreasing Mass

Start - End	Observed	Mr(expt)	Mr(calc)	Delta	Miss	Sequence
185 - 194	1224.5841	1223.5768	1223.6298	-0.0530	1	**FFDKVQDLGR** (Ions score 12)
211 - 230	2137.0193	2136.0120	2136.0772	-0.0652	0	**LVDELMEAPGDVEALAPPVR** Oxidation (M) (No match)

329 - 342	1694.7970	1693.7897	1693.8522	-0.0625	1	**KQAELEEIYESSIR**	(Ions score 31)
329 - 342	1694.7970	1693.7897	1693.8522	-0.0625	1	**KQAELEEIYESSIR**	(No match)
629 - 638	1224.5841	1223.5768	1223.5856	-0.0087	1	**FSEINPDMKK** Oxidation (M)	(No match)

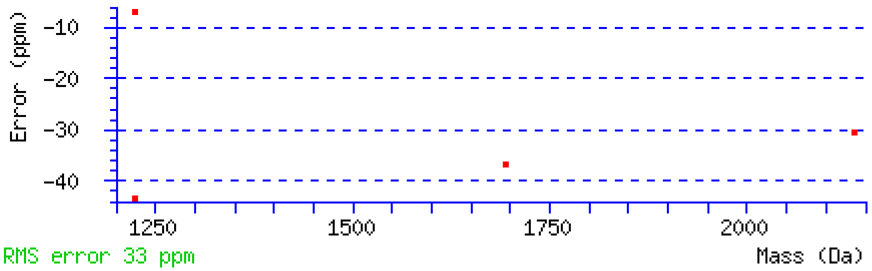

Mascot: http://www.matrixscience.com/

Spot 882

Protein View

Match to: **gi|4507789** Score: **168** Expect: **3e-012**
ubiquitin-conjugating enzyme E2L 3 isoform 1 [Homo sapiens]

Nominal mass (M_r): **18021**; Calculated pI value: **8.68**
NCBI BLAST search of gi|4507789 against nr
Unformatted sequence string for pasting into other applications

Taxonomy: Homo sapiens
Links to retrieve other entries containing this sequence from NCBI Entrez:
gi|6678481 from Mus musculus
gi|57525148 from Gallus gallus
gi|118151424 from Bos taurus
gi|73995921 from Canis lupus familiaris
gi|109093385 from Macaca mulatta
gi|149251546 from Mus musculus
gi|149720216 from Equus caballus
gi|54039805 from Homo sapiens
gi|54039806 from Mus musculus
gi|110288003 from Bos taurus
gi|6573519 from Homo sapiens
gi|10120670 from Homo sapiens
gi|1064916 from Homo sapiens
gi|1419021 from Mus musculus
gi|1488377 from Homo sapiens
gi|2739215 from Homo sapiens
gi|3928493 from Mus musculus
gi|12840704 from Mus musculus
gi|31418457 from Homo sapiens
gi|47678743 from Homo sapiens
gi|53130754 from Gallus gallus
gi|62533160 from Mus musculus
gi|74226986 from Mus musculus
gi|75947622 from Bos taurus
gi|77415470 from Mus musculus
gi|90075034 from Macaca fascicularis
gi|109451546 from synthetic construct
gi|109452140 from synthetic construct
gi|119579863 from Homo sapiens
gi|119579864 from Homo sapiens
gi|148665036 from Mus musculus
gi|148683416 from Mus musculus

Fixed modifications: Carbamidomethyl (C)
Variable modifications: Oxidation (M)
Cleavage by Trypsin: cuts C-term side of KR unless next residue is P
Sequence Coverage: **34%**

Matched peptides shown in **Bold Red**

```
  1 MAASRRLMKE LEEIRKCGMK NFRNIQVDEA NLLTWQGLIV PDNPPYDKGA
 51 FRIEINFPAE YPFKPPKITF KTKIYHPNID EKGQVCLPVI SAENWKPATK
101 TDQVIQSLIA LVNDPQPEHP LRADLAEEYS KDRKKFCKNA EEFTKKYGEK
151 RPVD
```

Residue Number Increasing Mass Decreasing Mass

Start - End	Observed	Mr(expt)	Mr(calc)	Delta	Miss	Sequence
10 - 16	916.5085	915.5012	915.5025	-0.0013	1	ELEEIRK (No match)
53 - 67	1789.9076	1788.9003	1788.9450	-0.0447	0	IEINFPAEYPFKPPK (Ions score 70)
53 - 67	1789.9076	1788.9003	1788.9450	-0.0447	0	IEINFPAEYPFKPPK (No match)
74 - 82	1128.5460	1127.5387	1127.5611	-0.0224	0	IYHPNIDEK (Ions score 8)
74 - 82	1128.5460	1127.5387	1127.5611	-0.0224	0	IYHPNIDEK (No match)
101 - 122	2483.2429	2482.2356	2482.3179	-0.0823	0	TDQVIQSLIALVNDPQPEHPLR (No match)
101 - 122	2483.2429	2482.2356	2482.3179	-0.0823	0	TDQVIQSLIALVNDPQPEHPLR (Ions score 33)

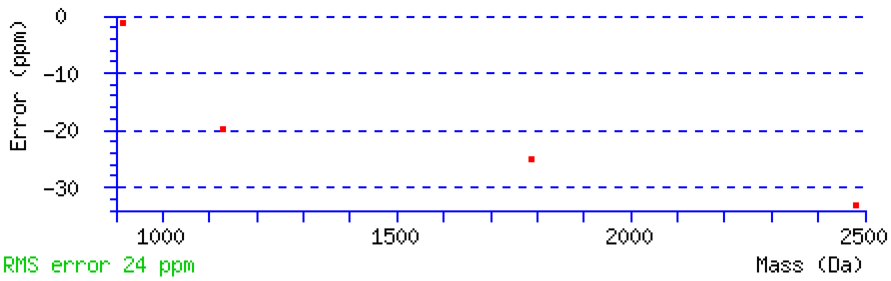

Spot 896

Protein View

Match to: **gi|21411235** Score: **112** Expect: **1.2e-006**
NADH dehydrogenase (ubiquinone) Fe-S protein 1, 75kDa (NADH-coenzyme Q reductase) [Homo sapiens]

Nominal mass (M_r): **80415**; Calculated pI value: **5.80**
NCBI BLAST search of gi|21411235 against nr
Unformatted sequence string for pasting into other applications

Taxonomy: Homo sapiens

Fixed modifications: Carbamidomethyl (C)
Variable modifications: Oxidation (M)
Cleavage by Trypsin: cuts C-term side of KR unless next residue is P
Sequence Coverage: **7%**

Matched peptides shown in **Bold Red**

```
  1 MLRIPVRKAL VGLSKSPKGC VRTTATAASN LIEVFVDGQS VMVEPGTTVL
 51 QACEKVGMQI PRFCYHERLS VAGNCRMCLV EIEKAPKVVA ACAMPVMKGW
101 NILTNSEKSK KAREGVMEFL LANHPLDCPI CDQGGECDLQ DQSMMFGNDR
151 SRFLEGKRAV EDKNIGPLVK TIMTRCIQCT RCIRFASEIA GVDDLGTTGR
201 GNDMQVGTYI EKMFMSELSG NIIDICPVGA LTSKPYAFTA QPWETRKTES
251 IDVMDAVGSN IVVSTRTGEV MRILPRMHED INEEWISDKT RFAYDGLKRQ
301 RLTEPMVRNE KGLLTYTSWE DALSRVAGML QSFQGKDVAA IAGGLVDAEA
351 LVALKDLLNR VDSDTLCTEE VFPTAGAGTD LRSNYLLNTT IAGVEEADVV
401 LLVGTNPRFE APLFNARIRK SWLHNDLKVA LIGSPVDLTY TYDHLGDSPK
451 ILQDIASGSH PFSQVLKEAK KPMVVLGSSA LQRNDGAAIL AAVSSIAQKI
501 RMTSGVTGDW KVMNILHRIA SQVAALDLGY KPGVEAIRKN PPKVLFLLGA
551 DGGCITRQDL PKDCFIIYQG HHGDVGAPIA DVILPGAAYT EKSATYVNTE
601 GRAQQTKVAV TPPGLAREDW KIIRALSEIA GMTLPYDTLD QVRNRLEEVS
651 PNLVRYDDIE GANYFQQANE LSKLVNQQLL ADPLVPPQLT IKDFYMTDSI
701 SRASQTMAKC VKAVTEGAQA VEEPSIC
```

Residue Number Increasing Mass Decreasing Mass

Start - End	Observed	Mr(expt)	Mr(calc)	Delta	Miss	Sequence	
292 - 299	969.5566	968.5493	968.5079	0.0414	1	FAYDGLKR	(No match)
409 - 417	1064.5874	1063.5801	1063.5450	0.0351	0	FEAPLFNAR	(No match)

409 - 417	1064.5874	1063.5801	1063.5450	0.0351	0	FEAPLFNAR	(No match)
519 - 538	2071.1892	2070.1819	2070.1472	0.0347	0	IASQVAALDLGYKPGVEAIR	(No match)
519 - 538	2071.1892	2070.1819	2070.1472	0.0347	0	IASQVAALDLGYKPGVEAIR	(Ions score 64)
544 - 557	1491.8387	1490.8314	1490.7915	0.0400	0	VLFLLGADGGCITR	(Ions score 13)
544 - 557	1491.8387	1490.8314	1490.7915	0.0400	0	VLFLLGADGGCITR	(No match)

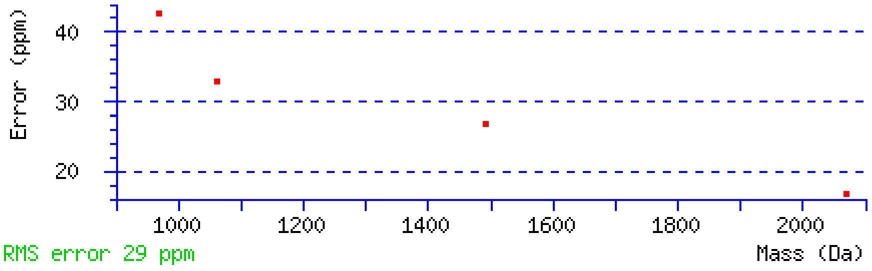

Mascot: http://www.matrixscience.com/

Spot 897

Protein View

Match to: **gi|11056036** Score: **178** Expect: **3e-013**
tubulin tyrosine ligase-like family, member 12 [Homo sapiens]

Nominal mass (M_r): **75154**; Calculated pI value: **5.33**
NCBI BLAST search of gi|11056036 against nr
Unformatted sequence string for pasting into other applications

Taxonomy: Homo sapiens
Links to retrieve other entries containing this sequence from NCBI Entrez:
gi|20455527 from Homo sapiens
gi|5911822 from Homo sapiens
gi|12654481 from Homo sapiens
gi|90403040 from Homo sapiens
gi|119593697 from Homo sapiens
gi|119593698 from Homo sapiens

Fixed modifications: Carbamidomethyl (C)
Variable modifications: Oxidation (M)
Cleavage by Trypsin: cuts C-term side of KR unless next residue is P
Sequence Coverage: **11%**

Matched peptides shown in **Bold Red**

```
  1 MEAERGPERR PAERSSPGQT PEEGAQALAE FAALHGPALR ASGVPERYWG
 51 RLLHKLEHEV FDAGEVFGIM QVEEVEEEED EAAREVRKQQ PNPGNELCYK
101 VIVTRESGLQ AAHPNSIFLI DHAWTCRVEH ARQQLQQVPG LLHRMANLMG
151 IEFHGELPST EAVALVLEEM WKFNQTYQLA HGTAEEKMPV WYIMDEFGSR
201 IQHADVPSFA TAPFFYMPQQ VAYTLLWPLR DLDTGEEVTR DFAYGETDPL
251 IRKCMLLPWA PTDMLDLSSC TPEPPAEHYQ AILEENKEKL PLDINPVVHP
301 HGHIFKVYTD VQQVASSLTH PRFTLTQSEA DADILFNFSH FKDYRKLSQE
351 RPGVLLNQFP CENLLTVKDC LASIARRAGG PEGPPWLPRT FNLRTELPQF
401 VSYFQQRERW GEDNHWICKP WNLARSLDTH VTKSLHSIIR HRESTPKVVS
451 KYIESPVLFL REDVGKVKFD IRYIVLLRSV RPLRLFVYDV FWLRFSNRAF
501 ALNDLDDYEK HFTVMNYDPD VVLKQVHCEE FIPEFEKQYP EFPWTDVQAE
551 IFRAFTELFQ VACAKPPPLG LCDYPSSRAM YAVDLMLKWD NGPDGRRVMQ
601 PQILEVNFNP DCERACRYHP TFFNDVFSTL FLDQPGGCHV TCLV
```

Residue Number Increasing Mass Decreasing Mass

Start - End	Observed	Mr(expt)	Mr(calc)	Delta	Miss	Sequence
133 - 144	1416.8051	1415.7978	1415.7997	-0.0019	0	QQLQQVPGLLHR (Ions score 12)
133 - 144	1416.8051	1415.7978	1415.7997	-0.0019	0	QQLQQVPGLLHR (No match)
241 - 252	1396.6727	1395.6654	1395.6670	-0.0016	0	DFAYGETDPLIR (No match)
241 - 252	1396.6727	1395.6654	1395.6670	-0.0016	0	DFAYGETDPLIR (Ions score 14)
307 - 322	1800.9092	1799.9019	1799.9166	-0.0146	0	VYTDVQQVASSLTHPR (No match)
307 - 322	1800.9092	1799.9019	1799.9166	-0.0146	0	VYTDVQQVASSLTHPR (Ions score 54)
378 - 389	1233.6431	1232.6358	1232.6301	0.0057	0	AGGPEGPPWLPR (No match)
395 - 407	1642.8135	1641.8062	1641.8150	-0.0088	0	TELPQFVSYFQQR (Ions score 17)
395 - 407	1642.8135	1641.8062	1641.8150	-0.0088	0	TELPQFVSYFQQR (No match)
452 - 461	1236.7019	1235.6946	1235.6913	0.0033	0	YIESPVLFLR (Ions score 18)
452 - 461	1236.7019	1235.6946	1235.6913	0.0033	0	YIESPVLFLR (No match)

Mascot: http://www.matrixscience.com/

Spot 902

Protein View

Match to: **gi|2896796** Score: **139** Expect: **2.4e-009**
D1013901 [Homo sapiens]

Nominal mass (M_r): **111236**; Calculated pI value: **5.52**
NCBI BLAST search of gi|2896796 against nr
Unformatted sequence string for pasting into other applications

Taxonomy: Homo sapiens

Fixed modifications: Carbamidomethyl (C)
Variable modifications: Oxidation (M)
Cleavage by Trypsin: cuts C-term side of KR unless next residue is P
Sequence Coverage: **5%**

Matched peptides shown in **Bold Red**

```
  1 DLLEARRPRA HECLGEALRV MHQIISKYPL LNTVETLTAA GTLIAKVKAF
 51 HYESNNDLEK QEFEKALETI AVAFSSTVSE FLMGEVDSST LLAVPPGDSS
101 QSMESLYGPG SEGTPPSLED CDAGCLPAEE VDVLLQRCEG GVDAALLYAK
151 NMAKYMKDLI SYLEKRTTLE MEFAKGLQKI AHNCRQSVMQ EPHMPLLSIY
201 SLALEQDLEF GHSMVQAVGT LQTQTFMQPL TLRRLEHEKR RKEIKEAWHR
251 AQRKLQEAES NLRKAKQGYV QRCEDHDKAR FLVAKAEEEQ AGSAPGAGST
301 ATKTLDKRRR LEEEAKNKAE EAMATYRTCV ADAKTQKQEL EDTKVTALRQ
351 IQEVIRQSDQ TIKSATISYY QMMHMQTAPL PVHFQMLCES SKLYDPGQQY
401 ASHVRQLQRD QEPDVHYDFE PHVSANAWSP VMRARKSSFN VSDVARPEAA
451 GSPPEEGGCT EGTPAKDHRA GRGHQVHKSW PLSISDSDSG LDPGPGAGDF
501 KKFERTSSSG TMSSTEELVD PDGGAGASAF EQADLNGMTP ELPVAVPSGP
551 FRHEGLSKAA RTHRLRKLRT PAKCRECNSY VYFQGAECEE CCLACHKKCL
601 ETLAIQCGHK KLQGRLQLFG QDFSHAARSA PDGVPFIVKK CVCEIERRAL
651 RTKGIYRVNG VKTRVEKLCQ AFENGKELVE LSQASPHDIS NVLKLYLRQL
701 PEPLISFRLY HELVGLAKDS LKAEAEAKAA SRGRQDGSES EAVAVALAGR
751 LRELLRDLPP ENRASLQYLL RHLRRIVEVE QDNKMTPGNL GIVFGPTLLR
801 PRPTEATVSL SSLVDYPHQA RVIETLIVHY GLVFEEEPEE TPGGQDESSN
851 QRAEVVVQVP YLEAGEAVVY PLQEAAADGC RESRVVSNDS DSDLEEASEL
901 LSSSEASALG HLSFLEQQQS EASLEVASGS HSGSEEQLEA TAREDGDGDE
951 DGPAQQLSGF NTNQSNNVLQ APLPPMRLRG GRMTLGSCRE RQPEFV
```

Residue Number Increasing Mass Decreasing Mass

Start - End	Observed	Mr(expt)	Mr(calc)	Delta	Miss	Sequence
393 - 405	1533.7443	1532.7370	1532.7371	-0.0001	0	LYDPGQQYASHVR (No match)
393 - 405	1533.7443	1532.7370	1532.7371	-0.0001	0	LYDPGQQYASHVR (Ions score 23)
616 - 628	1489.7531	1488.7458	1488.7473	-0.0015	0	LQLFGQDFSHAAR (Ions score 39)
616 - 628	1489.7531	1488.7458	1488.7473	-0.0015	0	LQLFGQDFSHAAR (No match)
699 - 708	1199.6783	1198.6710	1198.6710	0.0001	0	QLPEPLISFR (Ions score 45)
699 - 708	1199.6783	1198.6710	1198.6710	0.0001	0	QLPEPLISFR (No match)
733 - 750	1772.8905	1771.8832	1771.8812	0.0020	1	GRQDGSESEAVAVALAGR (No match)

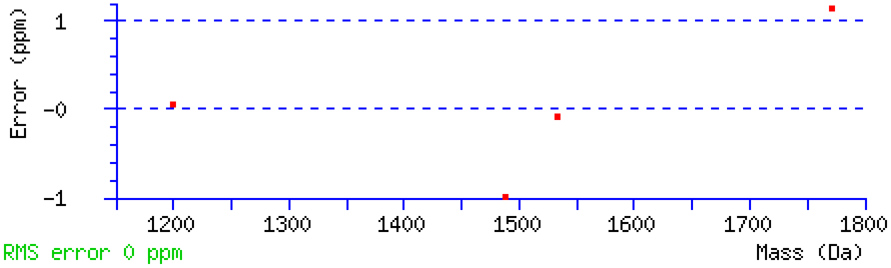

RMS error 0 ppm

Mascot: http://www.matrixscience.com/

Spot 908

Protein View

Match to: **gi|119596926** Score: **307** Expect: **3.8e-026**
leucine-rich repeats and calponin homology (CH) domain containing 4, isoform CRA_b [Homo sapiens]

Nominal mass (M_r): **70861**; Calculated pI value: **6.88**
NCBI BLAST search of gi|119596926 against nr
Unformatted sequence string for pasting into other applications

Taxonomy: Homo sapiens

Fixed modifications: Carbamidomethyl (C)
Variable modifications: Oxidation (M)
Cleavage by Trypsin: cuts C-term side of KR unless next residue is P
Sequence Coverage: **38%**

Matched peptides shown in **Bold Red**

```
  1 MAAAVAAPLA AGGEEAAATT SVPGSPGLPG RRSAERALEE AVATGTLNLS
 51 NRRLKHFPRG AARSYDLSDI TQADLSRNRF PEVPEAACQL VSLEGLSLYH
101 NCLRCLNPAL GNLTALTYLN LSRNQLSLLP PYICQLPLRV LIVSNNKLGA
151 LPPDIGTLGS LRQLDVSSNE LQSLPSELCG LSSLRDLNVR RNQLSTLPEE
201 LGDLPLVRLD FSCNRVSRIP VSFCRLRHLQ VILLDSNPLQ SPPAQVCLKG
251 KLHIFKYLST EAGQRGSALG DLAPSRPPSF SPCPAEDLFP GHRYDGGLDS
301 GFHSVDSGSK RWSGNESTDE FSELSFRISE LAREPRGPRE RKEDGSADGD
351 PVQIDFIDSH VPGEDEERGT VEEQRPPELS PGAGDRERAP SSRREEPAGE
401 ERRRPDTLQL WQERERRQQQ QSGAWGAPRK DSLLKPGLRA VVGGAAAVST
451 QAMHNGSPKS SASQAGAAAG QGAPAPAPAS QEPLPIAGPA TAPAPRPLGS
501 IQRPNSFLFR SSSQSGSGPS SPDSVLRPRR YPQVPDEKDL MTQLRQVLES
551 RLQRPLPEDL AEALASGVIL CQLANQLRPR SVPFIHVPSP AVPKLSALKA
601 RKNVESFLEA CRKMGVPEES LCQPHHILEE EGAPGRGLPY IAAVVHALLD
651 RP
```

Residue Number Increasing Mass Decreasing Mass

Start - End	Observed	Mr(expt)	Mr(calc)	Delta	Miss	Sequence
37 - 52	1658.8634	1657.8561	1657.8634	-0.0073	0	ALEEAVATGTLNLSNR (No match)
37 - 53	1814.9664	1813.9591	1813.9645	-0.0054	1	ALEEAVATGTLNLSNRR (No match)
64 - 77	1583.7426	1582.7353	1582.7475	-0.0121	0	SYDLSDITQADLSR (No match)

105 - 123	2104.0938	2103.0865	2103.1146	-0.0281	0	CLNPALGNLTALTYLNLSR	(No match)
124 - 139	1925.0554	1924.0481	1924.0604	-0.0123	0	NQLSLLPPYICQLPLR	(No match)
124 - 139	1925.0554	1924.0481	1924.0604	-0.0123	0	NQLSLLPPYICQLPLR	(Ions score 36)
148 - 162	1479.8458	1478.8385	1478.8456	-0.0071	0	LGALPPDIGTLGSLR	(Ions score 43)
148 - 162	1479.8458	1478.8385	1478.8456	-0.0071	0	LGALPPDIGTLGSLR	(No match)
163 - 185	2532.2295	2531.2222	2531.2537	-0.0314	0	QLDVSSNELQSLPSELCGLSSLR	(No match)
192 - 208	1894.0155	1893.0082	1893.0207	-0.0125	0	NQLSTLPEELGDLPLVR	(No match)
192 - 208	1894.0155	1893.0082	1893.0207	-0.0125	0	NQLSTLPEELGDLPLVR	(Ions score 30)
209 - 215	911.3976	910.3903	910.3967	-0.0063	0	LDFSCNR	(No match)
219 - 225	878.4512	877.4439	877.4480	-0.0040	0	IPVSFCR	(No match)
257 - 265	1024.5111	1023.5038	1023.4985	0.0053	0	YLSTEAGQR	(No match)
369 - 388	2180.0684	2179.0611	2179.0617	-0.0006	1	GTVEEQRPPELSPGAGDRER	(No match)
395 - 403	1072.4982	1071.4909	1071.4944	-0.0035	1	EEPAGEERR	(No match)
403 - 414	1597.8521	1596.8448	1596.8484	-0.0036	1	RRPDTLQLWQER	(No match)
418 - 429	1313.6244	1312.6171	1312.6272	-0.0101	0	QQQQSGAWGAPR	(No match)
430 - 439	1126.6940	1125.6867	1125.6869	-0.0002	1	KDSLLKPGLR	(No match)
511 - 529	1887.9028	1886.8955	1886.9082	-0.0126	0	SSSQSGSGPSSPDSVLRPR	(No match)
581 - 594	1474.8341	1473.8268	1473.8343	-0.0075	0	SVPFIHVPSPAVPK	(No match)
603 - 612	1224.5649	1223.5576	1223.5604	-0.0028	0	NVESFLEACR	(No match)
603 - 613	1352.6595	1351.6522	1351.6554	-0.0031	1	NVESFLEACRK	(No match)

Mascot: http://www.matrixscience.com/

Spot 913

Protein View

Match to: **gi|119629967** Score: **81** Expect: **0.0016**
ubiquitin associated and SH3 domain containing, A, isoform CRA_c [Homo sapiens]

Nominal mass (M_r): **51314**; Calculated pI value: **7.55**
NCBI BLAST search of gi|119629967 against nr
Unformatted sequence string for pasting into other applications

Taxonomy: Homo sapiens

Fixed modifications: Carbamidomethyl (C)
Variable modifications: Oxidation (M)
Cleavage by Trypsin: cuts C-term side of KR unless next residue is P
Sequence Coverage: **9%**

Matched peptides shown in **Bold Red**

```
  1 MAAGETQLYA KVSNKLKSRS SPSLLEPLLA MGFPVHTALK ALAATGRKTA
 51 EEALAWLHDH CNDPSLDDPI PQEYALFLCP TGPLLEKLQE FWRESKRQCA
101 KNRAHEVFPH VTLCDFFTCE DQKVECLYEA LKRAGDRLLG SFPTAVPLAL
151 HSSISYLGFF VSGSPADVIR EFAMTFATEA SLLADCSVKP CTKQLHLTLA
201 HKFYPHHQRT LEQLARAIPL GHSCQWTAAL YSRDMRFVHY QTLRALFQYK
251 PQNVDELTLS PGDYIFVDPT QQDEASEGWV IGISQRTGCR GFLPENYTDR
301 ASESDTWVKH RMYTFSLATD LNSRKDGEAS SRCSGEFLPQ TARSLSSLQA
351 LQATVARKSV LVVRHGERVD QIFGKAWLQQ CSTPDGKYYR PDLNFPCSLP
401 RRSRGIKDFE NDPPLSSCGI FQSRIAGMFE DCLVGKVTIT ATLIMASRHP
451 A
```

Residue Number Increasing Mass Decreasing Mass

Start - End	Observed	Mr(expt)	Mr(calc)	Delta	Miss	Sequence
237 - 244	1063.5767	1062.5694	1062.5610	0.0084	0	FVHYQTLR (No match)
237 - 244	1063.5767	1062.5694	1062.5610	0.0084	0	FVHYQTLR (No match)
291 - 300	1211.5786	1210.5713	1210.5618	0.0095	0	GFLPENYTDR (No match)
291 - 300	1211.5786	1210.5713	1210.5618	0.0095	0	GFLPENYTDR (Ions score 24)
359 - 368	1151.7258	1150.7185	1150.6570	0.0615	1	SVLVVRHGER (No match)
388 - 401	1797.8817	1796.8744	1796.8668	0.0076	0	YYRPDLNFPCSLPR (Ions score 13)
388 - 401	1797.8817	1796.8744	1796.8668	0.0076	0	YYRPDLNFPCSLPR (No match)

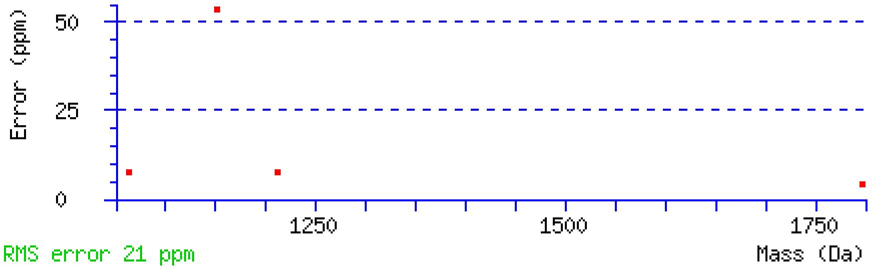

Mascot: http://www.matrixscience.com/

Spot 917

Protein View

Match to: **gi|4505257** Score: **572** Expect: **1.2e-052**
moesin [Homo sapiens]

Nominal mass (M_r): **67892**; Calculated pI value: **6.08**
NCBI BLAST search of gi|4505257 against nr
Unformatted sequence string for pasting into other applications

Taxonomy: Homo sapiens
Links to retrieve other entries containing this sequence from NCBI Entrez:
gi|127234 from Homo sapiens
gi|188626 from Homo sapiens
gi|5419633 from Homo sapiens
gi|16878176 from Homo sapiens
gi|119625803 from Homo sapiens
gi|123984467 from synthetic construct
gi|123998433 from synthetic construct

Fixed modifications: Carbamidomethyl (C)
Variable modifications: Oxidation (M)
Cleavage by Trypsin: cuts C-term side of KR unless next residue is P
Sequence Coverage: **58%**

Matched peptides shown in **Bold Red**

1 MPKTISVR**VT** **TMDAELEFAI** **QPNTTGKQLF** **DQVVK**TIGLR **EVWFFGLQYQ**
51 **DTKGFSTWLK** LNKK**VTAQDV** **RKESPLLFKF** **RAKFYPEDVS** **EELIQDITQR**
101 **LFFLQVK**EGI LNDDIYCPPE TAVLLASYAV QSK**YGDFNKE** **VHKSGYLAGD**
151 **KLLPQRVLEQ** **HKLNKDQWEE** **RIQVWHEEHR** **GMLREDAVLE** **YLKIAQDLEM**
201 **YGVNYFSIK**N KKGSELWLGV DALGLNIYEQ NDRLTPK**IGF** **PWSEIRNISF**
251 **NDKKFVIKPI** **DKKAPDFVFY** **APR**LRINKR**I** **LALCMGNHEL** **YMR**R**RKPDTI**
301 **EVQQMK**AQAR EEKHQKQMER AMLENEKKKR EMAEKEKEKI EREKEELMER
351 LKQIEEQTKK **AQQELEEQTR** **RALELEQER**K RAQSEAEKLA KER**QEAEEAK**
401 **EALLQASR**DQ K**KTQEQLALE** **MAELTARISQ** **LEMAR**QKKES EAVEWQQK**AQ**
451 **MVQEDLEKTR** AELKTAMSTP HVAEPAENEQ DEQDENGAEA SADLRADAMA
501 K**DRSEEERTT** **EAEKNER**VQK HLK**ALTSELA** **NARDESK**KTA NDMIHAENMR
551 LGRDKYKTLR QIRQGNTK**QR** **IDEFESM**

Residue Number Increasing Mass Decreasing Mass

Start - End	Observed	Mr(expt)	Mr(calc)	Delta	Miss	Sequence
9 - 27	2082.0725	2081.0652	2080.9986	0.0666	0	VTTMDAELEFAIQPNTTGK Oxidation (M) (No match)
28 - 35	976.5673	975.5600	975.5389	0.0211	0	QLFDQVVK (No match)
41 - 53	1660.8588	1659.8515	1659.7932	0.0583	0	EVWFFGLQYQDTK (No match)
54 - 60	838.4642	837.4569	837.4384	0.0185	0	GFSTWLK (No match)
65 - 72	916.5504	915.5431	915.5137	0.0294	1	VTAQDVRK (No match)
73 - 81	1136.6093	1135.6020	1135.6389	-0.0369	1	ESPLLFKFR (No match)
82 - 100	2281.2031	2280.1958	2280.1273	0.0685	1	AKFYPEDVSEELIQDITQR (No match)
84 - 100	2082.0725	2081.0652	2080.9953	0.0700	0	FYPEDVSEELIQDITQR (Ions score 49)
101 - 107	894.5632	893.5559	893.5374	0.0185	0	LFFLQVK (No match)
134 - 143	1236.5872	1235.5799	1235.5934	-0.0135	1	YGDFNKEVHK (No match)
144 - 156	1417.8273	1416.8200	1416.7724	0.0476	1	SGYLAGDKLLPQR (No match)
157 - 165	1108.6140	1107.6067	1107.6400	-0.0333	1	VLEQHKLNK (No match)
166 - 171	862.3983	861.3910	861.3617	0.0294	0	DQWEER (No match)
172 - 180	1233.6486	1232.6413	1232.6050	0.0363	0	IQVWHEEHR (No match)
181 - 193	1552.8556	1551.8483	1551.7966	0.0517	1	GMLREDAVLEYLK Oxidation (M) (No match)
185 - 193	1079.5359	1078.5286	1078.5546	-0.0260	0	EDAVLEYLK (No match)
194 - 209	1906.9980	1905.9907	1905.9182	0.0726	0	IAQDLEMYGVNYFSIK Oxidation (M) (No match)
238 - 246	1104.6187	1103.6114	1103.5763	0.0351	0	IGFPWSEIR (Ions score 43)
238 - 246	1104.6187	1103.6114	1103.5763	0.0351	0	IGFPWSEIR (No match)
247 - 254	965.5268	964.5195	964.4977	0.0218	1	NISFNDKK (No match)
255 - 262	959.5471	958.5398	958.5851	-0.0453	0	FVIKPIDK (No match)
263 - 273	1310.7328	1309.7255	1309.6818	0.0437	1	KAPDFVFYAPR (Ions score 64)
263 - 273	1310.7328	1309.7255	1309.6818	0.0437	1	KAPDFVFYAPR (No match)
264 - 273	1182.6361	1181.6288	1181.5869	0.0419	0	APDFVFYAPR (No match)
264 - 273	1182.6361	1181.6288	1181.5869	0.0419	0	APDFVFYAPR (Ions score 71)
280 - 293	1752.8840	1751.8767	1751.8157	0.0611	0	ILALCMGNHELYMR 2 Oxidation (M) (No match)
295 - 306	1488.8348	1487.8275	1487.7766	0.0510	1	RKPDTIEVQQMK Oxidation (M) (No match)
361 - 371	1387.7383	1386.7310	1386.6851	0.0459	1	AQQELEEQTRR (No match)
372 - 379	987.5416	986.5343	986.5032	0.0311	0	ALELEQER (No match)
394 - 408	1672.9063	1671.8990	1671.8427	0.0563	1	QEAEEAKEALLQASR (No match)
412 - 427	1832.0209	1831.0136	1830.9509	0.0627	1	KTQEQLALEMAELTAR (No match)
412 - 427	1848.0175	1847.0102	1846.9458	0.0644	1	KTQEQLALEMAELTAR Oxidation (M) (No match)
413 - 427	1703.9211	1702.9138	1702.8559	0.0579	0	TQEQLALEMAELTAR (No match)
413 - 427	1719.9163	1718.9090	1718.8508	0.0582	0	TQEQLALEMAELTAR Oxidation (M) (No match)
428 - 435	963.5167	962.5094	962.4855	0.0239	0	ISQLEMAR Oxidation (M) (No match)
449 - 460	1463.7656	1462.7583	1462.7085	0.0498	1	AQMVQEDLEKTR Oxidation (M) (No match)
502 - 508	920.4305	919.4232	919.3995	0.0237	1	DRSEEER (No match)
509 - 517	1077.5487	1076.5414	1076.5098	0.0317	1	TTEAEKNER (No match)
524 - 533	1045.5831	1044.5758	1044.5563	0.0195	0	ALTSELANAR (No match)
524 - 537	1504.8214	1503.8141	1503.7528	0.0613	1	ALTSELANARDESK (No match)
569 - 577	1170.5444	1169.5371	1169.5022	0.0349	1	QRIDEFESM Oxidation (M) (No match)

Mascot: http://www.matrixscience.com/

Spot 919

Protein View

Match to: **gi|81175200** Score: **618** Expect: **3e-057**
Talin-1

Nominal mass (M_r): **271766**; Calculated pI value: **5.77**
NCBI BLAST search of gi|81175200 against nr
Unformatted sequence string for pasting into other applications

Taxonomy: Homo sapiens
Links to retrieve other entries containing this sequence from NCBI Entrez:
gi|119578756 from Homo sapiens

Fixed modifications: Carbamidomethyl (C)
Variable modifications: Oxidation (M)
Cleavage by Trypsin: cuts C-term side of KR unless next residue is P
Sequence Coverage: **19%**

Matched peptides shown in **Bold Red**

```
   1 MVALSLKISI GNVVKTMQFE PSTMVYDACR IIRERIPEAP AGPPSDFGLF
  51 LSDDDPKKGI WLEAGKALDY YMLRNGDTME YRKKQRPLKI RMLDGTVKTI
 101 MVDDSKTVTD MLMTICARIG ITNHDEYSLV RELMEEKKEE ITGTLRKDKT
 151 LLRDEKKMEK LKQKLHTDDE LNWLDHGRTL REQGVEEHET LLLRRKFFYS
 201 DQNVDSRDPV QLNLLYVQAR DDILNGSHPV SFDKACEFAG FQCQIQFGPH
 251 NEQKHKAGFL DLKDFLPKEY VKQKGERKIF QAHKNCGQMS EIEAKVRYVK
 301 LARSLKTYGV SFFLVKEKMK GKNKLVPRLL GITKECVMRV DEKTKEVIQE
 351 WNLTNIKRWA ASPKSFTLDF GDYQDGYYSV QTTEGEQIAQ LIAGYIDIIL
 401 KKKKSKDHFG LEGDEESTML EDSVSPKKST VLQQQYNRVG KVEHGSVALP
 451 AIMRSGASGP ENFQVGSMPP AQQQITSGQM HRGHMPPLTS AQQALTGTIN
 501 SSMQAVQAAQ ATLDDFDTLP PLGQDAASKA WRKNKMDESK HEIHSQVDAI
 551 TAGTASVVNL TAGDPAETDY TAVGCAVTTI SSNLTEMSRG VKLLAALLED
 601 EGGSGRPLLQ AAKGLAGAVS ELLRSAQPAS AEPRQNLLQA AGNVGQASGE
 651 LLQQIGESDT DPHFQDALMQ LAKAVASAAA ALVLKAKSVA QRTEDSGLQT
 701 QVIAAATQCA LSTSQLVACT KVVAPTISSP VCQEQLVEAG RLVAKAVEGC
 751 VSASQAATED GQLLRGVGAA ATAVTQALNE LLQHVKAHAT GAGPAGRYDQ
 801 ATDTILTVTE NIFSSMGDAG EMVRQARILA QATSDLVNAI KADAEGESDL
 851 ENSRKLLSAA KILADATAKM VEAAKGAAAH PDSEEQQQRL REAAEGLRMA
 901 TNAAAQNAIK KKLVQRLEHA AKQAAASATQ TIAAAQHAAS TPKASAGPQP
 951 LLVQSCKAVA EQIPLLVQGV RGSQAQPDSP SAQLALIAAS QSFLQPGGKM
1001 VAAAKASVPT IQDQASAMQL SQCAKNLGTA LAELRTAAQK AQEACGPLEM
1051 DSALSVVQNL EKDLQEVKAA ARDGKLKPLP GETMEKCTQD LGNSTKAVSS
1101 AIAQLLGEVA QGNENYAGIA ARDVAGGLRS LAQAARGVAA LTSDPAVQAI
```

1151 VLDTASDVLD KASSLIEEAK K**AAGHPGDPE SQQR**LAQVAK AVTQALNR**CV**
1201 **SCLPGQRDVD NALR**AVGDAS KR**LLSDSLPP STGTFQEAQS RLNEAAAGLN**
1251 **QAATELVQAS R**GTPQDLARA SGRFGQDFST FLEAGVEMAG QAPSQEDRAQ
1301 VVSNLKGISM SSSKLLLAAK ALSTDPAAPN LKSQLAAAAR AVTDSINQLI
1351 TMCTQQAPGQ KECDNALREL ETVRELLENP VQPINDMSYF GCLDSVMENS
1401 KVLGEAMTGI SQNAKNGNLP EFGDAISTAS KALCGFTEAA AQAAYLVGVS
1451 DPNSQAGQQG LVEPTQFARA NQAIQMACQS LGEPGCTQAQ VLSAATIVAK
1501 HTSALCNSCR LASARTTNPT AKRQFVQSAK EVANSTANLV KTIK**ALDGAF**
1551 **TEENR**AQCRA ATAPLLEAVD NLSAFASNPE FSSIPAQISP EGRAAMEPIV
1601 ISAKTMLESA GGLIQTARAL AVNPR**DPPSW SVLAGHSR**TV SDSIKKLITS
1651 MR**DKAPGQLE CETAIAALNS CLRDLDQASL AAVSQQLAPR** EGISQEALHT
1701 QMLTAVQEIS HLIEPLANAA RAEASQLGHK VSQMAQYFEP LTLAAVGAAS
1751 KTLSHPQQMA LLDQTKTLAE SALQLLYTAK EAGGNPKQAA HTQEALEEAV
1801 QMMTEAVEDL TTTLNEAASA AGVVGGMVDS ITQAINQLDE GPMGEPEGSF
1851 VDYQTTMVRT AKAIAVTVQE MVTK**SNTSPE ELGPLANQLT SDYGRLASEA**
1901 **KPAAVAAENE EIGSHIK**HRV QELGHGCAAL VTKAGALQCS PSDAYTKKEL
1951 IECARRVSEK **VSHVLAALQA GNR**GTQACIT AASAVSGIIA DLDTTIMFAT
2001 AGTLNR**EGTE TFADHR**EGIL KTAKVLVEDT KVLVQNAAGS QEK**LAQAAQS**
2051 **SVATITR**LAD VVKLGAASLG AEDPETQVVL INAVKDVAKA LGDLISATKA
2101 AAGKVGDDPA VWQLKNSAKV MVTNVTSLLK TVKAVEDEAT KGTRALEATT
2151 EHIRQELAVF CSPEPPAK**TS TPEDFIR**MTK GITMATAKAV AAGNSCRQED
2201 VIATANLSRR AIADMLRACK **EAAYHPEVAP DVR**LRALHYG RECANGYLEL
2251 LDHVLLTLQK PSPELKQQLT GHSKRVAGSV TELIQAAEAM KGTEWVDPED
2301 PTVIAENELL GAAAAIEAAA K**KLEQLKPR**A KPKEADESLN FEEQILEAAK
2351 SIAAATSALV KAASAAQREL VAQGK**VGAIP ANALDDGQWS QGLISAAR**MV
2401 AAATNNLCEA ANAAVQGHAS QEKLISSAKQ VAASTAQLLV ACKVKADQDS
2451 EAMKRLQAAG NAVKRASDNL VKAAQKAAAF EEQENETVVV KEKMVGGIAQ
2501 IIAAQEEMLR KERELEEARK KLAQIRQQQY K**FLPSELRDE H**

Residue Number Increasing Mass Decreasing Mass

Start - End	Observed	Mr(expt)	Mr(calc)	Delta	Miss	Sequence
67 - 74	1044.5170	1043.5097	1043.5110	-0.0012	0	ALDYYMLR (No match)
67 - 74	1060.5088	1059.5015	1059.5059	-0.0044	0	ALDYYMLR Oxidation (M) (No match)
119 - 131	1516.7739	1515.7666	1515.7681	-0.0015	0	IGITNHDEYSLVR (No match)
119 - 131	1516.7739	1515.7666	1515.7681	-0.0015	0	IGITNHDEYSLVR (Ions score 45)
165 - 178	1720.7987	1719.7914	1719.7965	-0.0050	0	LHTDDELNWLDHGR (No match)
182 - 194	1552.7924	1551.7851	1551.7892	-0.0041	0	EQGVEEHETLLLR (No match)
197 - 207	1377.6063	1376.5990	1376.5996	-0.0006	0	FFYSDQNVDSR (No match)
208 - 220	1528.8446	1527.8373	1527.8409	-0.0035	0	DPVQLNLLYVQAR (No match)
346 - 358	1642.8733	1641.8660	1641.8838	-0.0178	1	EVIQEWNLTNIKR (No match)
428 - 438	1364.7264	1363.7191	1363.7208	-0.0016	1	KSTVLQQQYNR (No match)
429 - 438	1236.6321	1235.6248	1235.6258	-0.0010	0	STVLQQQYNR (No match)
593 - 613	2122.1772	2121.1699	2121.1793	-0.0093	0	LLAALLEDEGGSGRPLLQAAK (No match)
614 - 624	1085.6359	1084.6286	1084.6240	0.0046	0	GLAGAVSELLR (No match)

722 - 741	2140.0940	2139.0867	2139.0993	-0.0126	0	VVAPTISSPVCQEQLVEAGR (No match)
722 - 741	2140.0940	2139.0867	2139.0993	-0.0126	0	VVAPTISSPVCQEQLVEAGR (Ions score 67)
746 - 765	2061.9998	2060.9925	2060.9796	0.0129	0	AVEGCVSASQAATEDGQLLR (No match)
876 - 889	1523.6843	1522.6770	1522.6760	0.0010	0	GAAAHPDSEEQQQR (No match)
958 - 971	1492.8801	1491.8728	1491.8772	-0.0044	0	AVAEQIPLLVQGVR (Ions score 68)
958 - 971	1492.8801	1491.8728	1491.8772	-0.0044	0	AVAEQIPLLVQGVR (No match)
1026 - 1035	1057.6012	1056.5939	1056.5927	0.0012	0	NLGTALAELR (No match)
1097 - 1122	2573.3035	2572.2962	2572.3244	-0.0282	0	AVSSAIAQLLGEVAQGNENYAGIAAR (No match)
1172 - 1184	1349.6169	1348.6096	1348.6119	-0.0023	0	AAGHPGDPESQQR (No match)
1199 - 1207	1076.4998	1075.4925	1075.4903	0.0023	0	CVSCLPGQR (No match)
1208 - 1214	802.4158	801.4085	801.3980	0.0105	0	DVDNALR (No match)
1223 - 1241	2034.0059	2032.9986	2033.0065	-0.0079	0	LLSDSLPPSTGTFQEAQSR (No match)
1242 - 1261	2027.0419	2026.0346	2026.0442	-0.0096	0	LNEAAAGLNQAATELVQASR (No match)
1545 - 1555	1222.5685	1221.5612	1221.5625	-0.0013	0	ALDGAFTEENR (No match)
1626 - 1638	1408.6938	1407.6865	1407.6895	-0.0029	0	DPPSWSVLAGHSR (No match)
1653 - 1673	2317.1130	2316.1057	2316.1201	-0.0144	1	DKAPGQLECETAIAALNSCLR (No match)
1674 - 1690	1782.9268	1781.9195	1781.9271	-0.0076	0	DLDQASLAAVSQQLAPR (Ions score 83)
1674 - 1690	1782.9268	1781.9195	1781.9271	-0.0076	0	DLDQASLAAVSQQLAPR (No match)
1875 - 1895	2249.0667	2248.0594	2248.0607	-0.0013	0	SNTSPEELGPLANQLTSDYGR (No match)
1896 - 1917	2235.1548	2234.1475	2234.1542	-0.0066	0	LASEAKPAAVAAENEEIGSHIK (No match)
1961 - 1973	1335.7485	1334.7412	1334.7418	-0.0006	0	VSHVLAALQAGNR (No match)
2007 - 2016	1162.5239	1161.5166	1161.5050	0.0116	0	EGTETFADHR (No match)
2044 - 2057	1416.7821	1415.7748	1415.7732	0.0016	0	LAQAAQSSVATITR (No match)
2169 - 2177	1065.5223	1064.5150	1064.5138	0.0012	0	TSTPEDFIR (No match)
2221 - 2233	1453.7037	1452.6964	1452.6997	-0.0033	0	EAAYHPEVAPDVR (Ions score 34)
2221 - 2233	1453.7037	1452.6964	1452.6997	-0.0033	0	EAAYHPEVAPDVR (No match)
2322 - 2329	1011.6271	1010.6198	1010.6236	-0.0038	1	KLEQLKPR (No match)
2376 - 2398	2310.1763	2309.1690	2309.1763	-0.0073	0	VGAIPANALDDGQWSQGLISAAR (No match)
2532 - 2538	861.4872	860.4799	860.4756	0.0044	0	FLPSELR (No match)
2532 - 2541	1242.6117	1241.6044	1241.6040	0.0004	1	FLPSELRDEH (No match)

Mascot: http://www.matrixscience.com/

Spot 920

Protein View

Match to: **gi|81175200** Score: **803** Expect: **9.6e-076**
Talin-1

Nominal mass (M_r): **271766;** Calculated pI value: **5.77**
NCBI BLAST search of gi|81175200 against nr
Unformatted sequence string for pasting into other applications

Taxonomy: Homo sapiens
Links to retrieve other entries containing this sequence from NCBI Entrez:
gi|119578756 from Homo sapiens

Fixed modifications: Carbamidomethyl (C)
Variable modifications: Oxidation (M)
Cleavage by Trypsin: cuts C-term side of KR unless next residue is P
Sequence Coverage: **24%**

Matched peptides shown in **Bold Red**

```
   1 MVALSLKISI GNVVKTMQFE PSTMVYDACR IIRERIPEAP AGPPSDFGLF
  51 LSDDDPKKGI WLEAGKALDY YMLRNGDTME YRKKQRPLKI RMLDGTVKTI
 101 MVDDSKTVTD MLMTICARIG ITNHDEYSLV RELMEEKKEE ITGTLRKDKT
 151 LLRDEKKMEK LKQKLHTDDE LNWLDHGRTL REQGVEEHET LLLRRKFFYS
 201 DQNVDSRDPV QLNLLYVQAR DDILNGSHPV SFDKACEFAG FQCQIQFGPH
 251 NEQKHKAGFL DLKDFLPKEY VKQKGERKIF QAHKNCGQMS EIEAKVRYVK
 301 LARSLKTYGV SFFLVKEKMK GKNKLVPRLL GITKECVMRV DEKTKEVIQE
 351 WNLTNIKRWA ASPKSFTLDF GDYQDGYYSV QTTEGEQIAQ LIAGYIDIIL
 401 KKKKSKDHFG LEGDEESTML EDSVSPKKST VLQQQYNRVG KVEHGSVALP
 451 AIMRSGASGP ENFQVGSMPP AQQQITSGQM HRGHMPPLTS AQQALTGTIN
 501 SSMQAVQAAQ ATLDDFDTLP PLGQDAASKA WRKNKMDESK HEIHSQVDAI
 551 TAGTASVVNL TAGDPAETDY TAVGCAVTTI SSNLTEMSRG VKLLAALLED
 601 EGGSGRPLLQ AAKGLAGAVS ELLRSAQPAS AEPRQNLLQA AGNVGQASGE
 651 LLQQIGESDT DPHFQDALMQ LAKAVASAAA ALVLKAKSVA QRTEDSGLQT
 701 QVIAAATQCA LSTSQLVACT KVVAPTISSP VCQEQLVEAG RLVAKAVEGC
 751 VSASQAATED GQLLRGVGAA ATAVTQALNE LLQHVKAHAT GAGPAGRYDQ
 801 ATDTILTVTE NIFSSMGDAG EMVRQARILA QATSDLVNAI KADAEGESDL
 851 ENSRKLLSAA KILADATAKM VEAAKGAAAH PDSEEQQQRL REAAEGLRMA
 901 TNAAAQNAIK KKLVQRLEHA AKQAAASATQ TIAAAQHAAS TPKASAGPQP
 951 LLVQSCKAVA EQIPLLVQGV RGSQAQPDSP SAQLALIAAS QSFLQPGGKM
1001 VAAAKASVPT IQDQASAMQL SQCAKNLGTA LAELRTAAQK AQEACGPLEM
1051 DSALSVVQNL EKDLQEVKAA ARDGKLKPLP GETMEKCTQD LGNSTKAVSS
1101 AIAQLLGEVA QGNENYAGIA ARDVAGGLRS LAQAARGVAA LTSDPAVQAI
```

1151 VLDTASDVLD KASSLIEEAK K**AAGHPGDPE SQQR**LAQVAK AVTQALNR**CV**
1201 **SCLPGQRDVD NALR**AVGDAS KR**LLSDSLPP STGTFQEAQS RLNEAAAGLN**
1251 **QAATELVQAS R**GTPQDLARA SGR**FGQDFST FLEAGVEMAG QAPSQEDR**AQ
1301 VVSNLKGISM SSSKLLLAAK ALSTDPAAPN LKSQLAAAAR AVTDSINQLI
1351 TMCTQQAPGQ KECDNALREL ETVRELLENP VQPINDMSYF GCLDSVMENS
1401 KVLGEAMTGI SQNAKNGNLP EFGDAISTAS KALCGFTEAA AQAAYLVGVS
1451 DPNSQAGQQG LVEPTQFARA NQAIQMACQS LGEPGCTQAQ VLSAATIVAK
1501 HTSALCNSCR LASARTTNPT AKRQFVQSAK EVANSTANLV KTIK**ALDGAF**
1551 **TEENR**AQCRA ATAPLLEAVD NLSAFASNPE FSSIPAQISP EGRAAMEPIV
1601 ISAKTMLESA GGLIQTARAL AVNPR**DPPSW SVLAGHSR**TV SDSIKKLITS
1651 MR**DKAPGQLE CETAIAALNS CLRDLDQASL AAVSQQLAPR** EGISQEALHT
1701 QMLTAVQEIS HLIEPLANAA RAEASQLGHK VSQMAQYFEP LTLAAVGAAS
1751 KTLSHPQQMA LLDQTKTLAE SALQLLYTAK EAGGNPKQAA HTQEALEEAV
1801 QMMTEAVEDL TTTLNEAASA AGVVGGMVDS ITQAINQLDE GPMGEPEGSF
1851 VDYQTTMVRT AKAIAVTVQE MVTK**SNTSPE ELGPLANQLT SDYGRLASEA**
1901 **KPAAVAAENE EIGSHIK**HRV QELGHGCAAL VTKAGALQCS PSDAYTKKEL
1951 IECARRVSEK **VSHVLAALQA GNR**GTQACIT AASAVSGIIA DLDTTIMFAT
2001 AGTLNR**EGTE TFADHR**EGIL KTAKVLVEDT KVLVQNAAGS QEK**LAQAAQS**
2051 **SVATITR**LAD VVK**LGAASLG AEDPETQVVL INAVK**DVAKA LGDLISATKA
2101 AAGKVGDDPA VWQLKNSAKV MVTNVTSLLK TVKAVEDEAT KGTR**ALEATT**
2151 **EHIRQELAVF CSPEPPAKTS TPEDFIR**MTK GITMATAKAV AAGNSCRQED
2201 VIATANLSRR AIADMLRACK **EAAYHPEVAP DVR**LRALHYG RECANGYLEL
2251 LDHVLLTLQK PSPELKQQLT GHSKRVAGSV TELIQAAEAM KGTEWVDPED
2301 PTVIAENELL GAAAAIEAAA K**KLEQLKPR**A KPKEADESLN FEEQILEAAK
2351 SIAAATSALV KAASAAQREL VAQGK**VGAIP ANALDDGQWS QGLISAARMV**
2401 **AAATNNLCEA ANAAVQGHAS QEK**LISSAKQ VAASTAQLLV ACKVKADQDS
2451 EAMKRLQAAG NAVKRASDNL VK**AAQKAAAF EEQENETVVV K**EKMVGGIAQ
2501 IIAAQEEMLR KERELEEARK KLAQIRQQQY K**FLPSELRDE H**

Residue Number Increasing Mass Decreasing Mass

Start - End	Observed	Mr(expt)	Mr(calc)	Delta	Miss	Sequence
67 - 74	1044.5172	1043.5099	1043.5110	-0.0010	0	**ALDYYMLR** (No match)
67 - 74	1060.5100	1059.5027	1059.5059	-0.0032	0	**ALDYYMLR** Oxidation (M) (No match)
119 - 131	1516.7704	1515.7631	1515.7681	-0.0050	0	**IGITNHDEYSLVR** (No match)
165 - 178	1720.7976	1719.7903	1719.7965	-0.0061	0	**LHTDDELNWLDHGR** (No match)
182 - 194	1552.7906	1551.7833	1551.7892	-0.0059	0	**EQGVEEHETLLLR** (No match)
196 - 207	1505.6963	1504.6890	1504.6946	-0.0056	1	**KFFYSDQNVDSR** (No match)
197 - 207	1377.6049	1376.5976	1376.5996	-0.0020	0	**FFYSDQNVDSR** (No match)
208 - 220	1528.8418	1527.8345	1527.8409	-0.0063	0	**DPVQLNLLYVQAR** (No match)
307 - 316	1160.6300	1159.6227	1159.6277	-0.0049	0	**TYGVSFFLVK** (No match)
346 - 358	1642.8799	1641.8726	1641.8838	-0.0112	1	**EVIQEWNLTNIKR** (No match)
428 - 438	1364.7264	1363.7191	1363.7208	-0.0016	1	**KSTVLQQQYNR** (No match)
429 - 438	1236.6309	1235.6236	1235.6258	-0.0022	0	**STVLQQQYNR** (No match)
442 - 454	1395.7168	1394.7095	1394.7339	-0.0244	0	**VEHGSVALPAIMR** Oxidation (M) (No match)

593 - 613	2122.1709	2121.1636	2121.1793	-0.0156	0	LLAALLEDEGGSGRPLLQAAK (No match)
614 - 624	1085.6312	1084.6239	1084.6240	-0.0001	0	GLAGAVSELLR (No match)
722 - 741	2140.0920	2139.0847	2139.0993	-0.0146	0	VVAPTISSPVCQEQLVEAGR (Ions score 78)
722 - 741	2140.0920	2139.0847	2139.0993	-0.0146	0	VVAPTISSPVCQEQLVEAGR (No match)
876 - 889	1523.6779	1522.6706	1522.6760	-0.0054	0	GAAAHPDSEEQQQR (No match)
958 - 971	1492.8782	1491.8709	1491.8772	-0.0063	0	AVAEQIPLLVQGVR (Ions score 86)
958 - 971	1492.8782	1491.8709	1491.8772	-0.0063	0	AVAEQIPLLVQGVR (No match)
1026 - 1035	1057.6010	1056.5937	1056.5927	0.0010	0	NLGTALAELR (No match)
1097 - 1122	2573.2751	2572.2678	2572.3244	-0.0566	0	AVSSAIAQLLGEVAQGNENYAGIAAR (No match)
1172 - 1184	1349.6194	1348.6121	1348.6119	0.0002	0	AAGHPGDPESQQR (No match)
1199 - 1207	1076.5013	1075.4940	1075.4903	0.0038	0	CVSCLPGQR (No match)
1208 - 1214	802.4113	801.4040	801.3980	0.0060	0	DVDNALR (No match)
1223 - 1241	2034.0023	2032.9950	2033.0065	-0.0115	0	LLSDSLPPSTGTFQEAQSR (No match)
1242 - 1261	2027.0391	2026.0318	2026.0442	-0.0124	0	LNEAAAGLNQAATELVQASR (No match)
1242 - 1261	2027.0391	2026.0318	2026.0442	-0.0124	0	LNEAAAGLNQAATELVQASR (Ions score 82)
1274 - 1298	2717.2019	2716.1946	2716.2074	-0.0128	0	FGQDFSTFLEAGVEMAGQAPSQEDR (No match)
1545 - 1555	1222.5677	1221.5604	1221.5625	-0.0021	0	ALDGAFTEENR (No match)
1626 - 1638	1408.6919	1407.6846	1407.6895	-0.0048	0	DPPSWSVLAGHSR (No match)
1653 - 1673	2317.1187	2316.1114	2316.1201	-0.0087	1	DKAPGQLECETAIAALNSCLR (No match)
1674 - 1690	1782.9252	1781.9179	1781.9271	-0.0092	0	DLDQASLAAVSQQLAPR (Ions score 127)
1674 - 1690	1782.9252	1781.9179	1781.9271	-0.0092	0	DLDQASLAAVSQQLAPR (No match)
1875 - 1895	2249.0537	2248.0464	2248.0607	-0.0143	0	SNTSPEELGPLANQLTSDYGR (No match)
1896 - 1917	2235.1497	2234.1424	2234.1542	-0.0117	0	LASEAKPAAVAAENEEIGSHIK (No match)
1961 - 1973	1335.7477	1334.7404	1334.7418	-0.0014	0	VSHVLAALQAGNR (No match)
2007 - 2016	1162.5242	1161.5169	1161.5050	0.0119	0	EGTETFADHR (No match)
2044 - 2057	1416.7761	1415.7688	1415.7732	-0.0044	0	LAQAAQSSVATITR (No match)
2064 - 2085	2195.2012	2194.1939	2194.1844	0.0095	0	LGAASLGAEDPETQVVLINAVK (No match)
2145 - 2154	1140.5999	1139.5926	1139.5934	-0.0008	0	ALEATTEHIR (No match)
2155 - 2168	1572.7645	1571.7572	1571.7653	-0.0081	0	QELAVFCSPEPPAK (No match)
2169 - 2177	1065.5243	1064.5170	1064.5138	0.0032	0	TSTPEDFIR (No match)
2221 - 2233	1453.7020	1452.6947	1452.6997	-0.0050	0	EAAYHPEVAPDVR (No match)
2221 - 2233	1453.7020	1452.6947	1452.6997	-0.0050	0	EAAYHPEVAPDVR (Ions score 32)
2322 - 2329	1011.6304	1010.6231	1010.6236	-0.0005	1	KLEQLKPR (No match)
2376 - 2398	2310.1699	2309.1626	2309.1763	-0.0137	0	VGAIPANALDDGQWSQGLISAAR (No match)
2399 - 2423	2572.2415	2571.2342	2571.1805	0.0537	0	MVAAATNNLCEAANAAVQGHASQEK Oxidation (M) (No match)
2473 - 2491	2062.0171	2061.0098	2061.0377	-0.0279	1	AAQKAAAFEEQENETVVVK (No match)
2532 - 2538	861.4838	860.4765	860.4756	0.0010	0	FLPSELR (No match)
2532 - 2541	1242.6107	1241.6034	1241.6040	-0.0006	1	FLPSELRDEH (No match)

Spot 921

Protein View

Match to: **gi|4504505** Score: **108** Expect: **3e-006**
hydroxysteroid (17-beta) dehydrogenase 4 [Homo sapiens]

Nominal mass (M_r): **80092;** Calculated pI value: **8.96**
NCBI BLAST search of gi|4504505 against nr
Unformatted sequence string for pasting into other applications

Taxonomy: Homo sapiens
Links to retrieve other entries containing this sequence from NCBI Entrez:
gi|1706396 from Homo sapiens
gi|1050517 from Homo sapiens
gi|4165049 from Homo sapiens
gi|13111861 from Homo sapiens
gi|119569296 from Homo sapiens
gi|123981926 from synthetic construct
gi|123996759 from synthetic construct

Fixed modifications: Carbamidomethyl (C)
Variable modifications: Oxidation (M)
Cleavage by Trypsin: cuts C-term side of KR unless next residue is P
Sequence Coverage: **5%**

Matched peptides shown in **Bold Red**

1 MGSPLRFDGR VVLVTGAGAG LGR**AYALAFA ER**GALVVVND LGGDFKGVGK
51 GSLAADKVVE EIRRRGGKAV ANYDSVEEGE KVVKTALDAF GRIDVVVNNA
101 GILRDRSFAR ISDEDWDIIH RVHLRGSFQV TRAAWEHMKK QKYGRIIMTS
151 SASGIYGNFG QANYSAAKLG LLGLANSLAI EGRKSNIHCN TIAPNAGSRM
201 TQTVMPEDLV EALKPEYVAP LVLWLCHESC EENGGLFEVG AGWIGKLRWE
251 RTLGAIVRQK NHPMTPEAVK ANWKKICDFE NASKPQSIQE STGSIIEVLS
301 KIDSEGGVSA NHTSRATSTA TSGFAGAIGQ KLPPFSYAYT ELEAIMYALG
351 VGASIKDPKD LKFIYEGSSD FSCLPTFGVI IGQKSMMGGG LAEIPGLSIN
401 FAK**VLHGEQY LELYKPLPR**A GKLKCEAVVA DVLDKGSGVV IIMDVYSYSE
451 KELICHNQFS LFLVGSGGFG GKRTSDKVKV AVAIPNRPPD AVLTDTTSLN
501 QAALYRLSGD WNPLHIDPNF ASLAGFDKPI LHGLCTFGFS ARRVLQQFAD
551 NDVSRFKAIK ARFAKPVYPG QTLQTEMWKE GNRIHFQTKV QETGDIVISN
601 AYVDLAPTSG TSAKTPSEGG K**LQSTFVFEE IGRR**LKDIGP EVVKKVNAVF
651 EWHITKGGNI GAKWTIDLKS GSGKVYQGPA KGAADTTIIL SDEDFMEVVL
701 GKLDPQKAFF SGRLKARGNI MLSQKLQMIL KDYAKL

Residue Number Increasing Mass Decreasing Mass

Start - End	Observed	Mr(expt)	Mr(calc)	Delta	Miss	Sequence
24 - 32	1011.4865	1010.4792	1010.5185	-0.0392	0	AYALAFAER (No match)
24 - 32	1011.4865	1010.4792	1010.5185	-0.0392	0	AYALAFAER (Ions score 4)
404 - 419	1954.9915	1953.9842	1954.0676	-0.0833	0	VLHGEQYLELYKPLPR (No match)
404 - 419	1954.9915	1953.9842	1954.0676	-0.0833	0	VLHGEQYLELYKPLPR (Ions score 41)
622 - 634	1581.7738	1580.7665	1580.8310	-0.0645	1	LQSTFVFEEIGRR (No match)
622 - 634	1581.7738	1580.7665	1580.8310	-0.0645	1	LQSTFVFEEIGRR (Ions score 24)

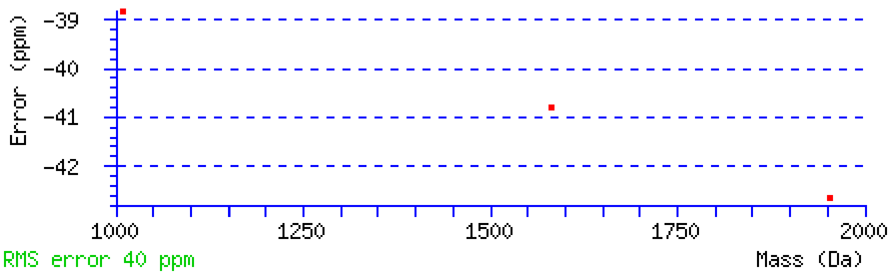

Mascot: http://www.matrixscience.com/

Spot 922

Protein View

Match to: **gi|5031855** Score: **104** Expect: **7.7e-006**
lymphocyte cytosolic protein 2 [Homo sapiens]

Nominal mass (M_r): **60265**; Calculated pI value: **5.89**
NCBI BLAST search of gi|5031855 against nr
Unformatted sequence string for pasting into other applications

Taxonomy: Homo sapiens
Links to retrieve other entries containing this sequence from NCBI Entrez:
gi|10720065 from Homo sapiens
gi|806766 from Homo sapiens
gi|16741630 from Homo sapiens
gi|30583385 from Homo sapiens
gi|61359571 from synthetic construct
gi|61359580 from synthetic construct
gi|119581883 from Homo sapiens
gi|123979556 from synthetic construct
gi|123994375 from synthetic construct

Fixed modifications: Carbamidomethyl (C)
Variable modifications: Oxidation (M)
Cleavage by Trypsin: cuts C-term side of KR unless next residue is P
Sequence Coverage: **12%**

Matched peptides shown in **Bold Red**

```
  1 MALRNVPFRS EVLGWDPDSL ADYFKKLNYK DCEKAVKKYH IDGARFLNLT
 51 ENDIQKFPKL RVPILSKLSQ EINKNEERRS IFTRKPQVPR FPEETESHEE
101 DNGGWSSFEE DDYESPNDDQ DGEDDGDYES PNEEEEAPVE DDADYEPPPS
151 NDEEALQNSI LPAKPFPNSN SMYIDRPPSG KTPQQPPVPP QRPMAALPPP
201 PAGRNHSPLP PPQTNHEEPS RSRNHKTAKL PAPSIDRSTK PPLDRSLAPF
251 DREPFTLGKK PPFSDKPSIP AGRSLGEHLP KIQKPPLPPT TERHERSSPL
301 PGKKPPVPKH GWGPDRREND EDDVHQRPLP QPALLPMSSN TFPSRSTKPS
351 PMNPLPSSHM PGAFSESNSS FPQSASLPPY FSQGPSNRPP IRAEGRNFPL
401 PLPNKPRPPS PAEEENSLNE EWYVSYITRP EAEAALRKIN QDGTFLVRDS
451 SKKTTTNPYV LMVLYKDKVY NIQIRYQKES QVYLLGTGLR GKEDFLSVSD
501 IIDYFRKMPL LLIDGKNRGS RYQCTLTHAA GYP
```

Residue Number Increasing Mass Decreasing Mass

Start - End	Observed	Mr(expt)	Mr(calc)	Delta	Miss	Sequence
205 - 221	1936.9396	1935.9323	1935.9187	0.0136	0	NHSPLPPPQTNHEEPSR (No match)
246 - 259	1577.8539	1576.8466	1576.8249	0.0217	1	SLAPFDREPFTLGK (No match)
246 - 259	1577.8539	1576.8466	1576.8249	0.0217	1	SLAPFDREPFTLGK (No match)
260 - 273	1496.8380	1495.8307	1495.8147	0.0161	0	KPPFSDKPSIPAGR (Ions score 36)
260 - 273	1496.8380	1495.8307	1495.8147	0.0161	0	KPPFSDKPSIPAGR (No match)
282 - 293	1376.8116	1375.8043	1375.7823	0.0220	0	IQKPPLPPTTER (Ions score 7)
282 - 293	1376.8116	1375.8043	1375.7823	0.0220	0	IQKPPLPPTTER (No match)
479 - 490	1335.7482	1334.7409	1334.7193	0.0216	0	ESQVYLLGTGLR (No match)
479 - 490	1335.7482	1334.7409	1334.7193	0.0216	0	ESQVYLLGTGLR (No match)

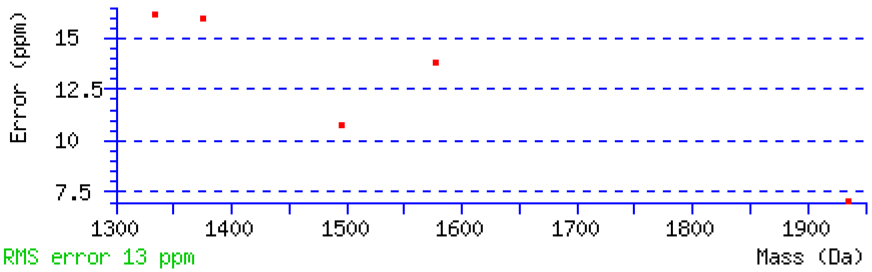

RMS error 13 ppm

Mascot: http://www.matrixscience.com/

Spot 929

Protein View

Match to: **gi|62089132** Score: **98** Expect: **3.1e-005**
echinoderm microtubule associated protein like 2 variant [Homo sapiens]

Nominal mass (M_r): **62949**; Calculated pI value: **5.74**
NCBI BLAST search of gi|62089132 against nr
Unformatted sequence string for pasting into other applications

Taxonomy: Homo sapiens

Fixed modifications: Carbamidomethyl (C)
Variable modifications: Oxidation (M)
Cleavage by Trypsin: cuts C-term side of KR unless next residue is P
Sequence Coverage: **10%**

Matched peptides shown in **Bold Red**

```
  1 PTGEIVYFVA SVAVLYSVEE QRQRHYLGHN DDIKCLAIHP DMVTIATGQV
 51 AGTTKEGKPL PPHVRIWDSV SLSTLHVLGL GVFDRAVCCV GFSKSNGGNL
101 LCAVDESNDH MLSVWDWAKE TKVVDVKCSN EAVLVATFHP TDPTVLITCG
151 KSHIYFWTLD GGSLSKRQGL FEKHEKPKYV LCVTFLEGGD VVTGDSGGNL
201 YVWGKGGNRI TQAVLGAHDG GVFGLCALRD GTLVSGGGRD RRVVLWGSDY
251 SKLQEVEVPE DFGPVRTVAE GHGDTLYVGT TRNSILQGSV HTGFSLLVQG
301 HVEELWGLAT HPSRAQFVTC GQDKLVHLWS SDSHQPLWSR IIEDPARSAG
351 FHPSGSVLAV GTVTGRWLLL DTETHDLVAI HTDGNEQISV VSFSPDGAYL
401 AVGSHDNLVY VYTVDQGGRK VSRLGKCSGH SSFITHLDWA QDSSCFVTNS
451 GDYEILYWDP ATCKQITSAD AVRNMEWATA TCVLGFGVFG IWSEGADGTD
501 INAVARSHDG KLLASADDFG KVHLFSYPCC QPRALSHKYG GHSSHVTNVA
551 FLWDDSMALT TGGKDTSVLQ WRVV
```

Residue Number Increasing Mass Decreasing Mass

Start - End	Observed	Mr(expt)	Mr(calc)	Delta	Miss	Sequence
56 - 65	1129.6654	1128.6581	1128.6403	0.0178	0	**EGKPLPPHVR** (No match)
210 - 229	2055.0684	2054.0611	2054.0730	-0.0119	0	**ITQAVLGAHDGGVFGLCALR** (No match)
253 - 266	1613.8221	1612.8148	1612.8096	0.0052	0	**LQEVEVPEDFGPVR** (No match)
253 - 266	1613.8221	1612.8148	1612.8096	0.0052	0	**LQEVEVPEDFGPVR** (Ions score 32)
348 - 366	1799.9393	1798.9320	1798.9325	-0.0005	0	**SAGFHPSGSVLAVGTVTGR** (No match)

348 - 366	1799.9393	1798.9320	1798.9325	-0.0005	0	SAGFHPSGSVLAVGTVTGR	(Ions score 31)

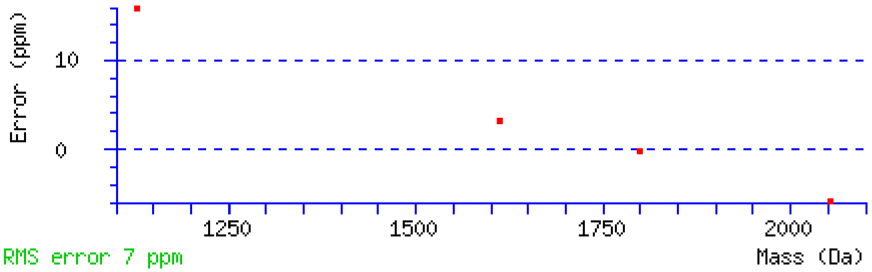

RMS error 7 ppm

Mascot: http://www.matrixscience.com/

Spot 930

Protein View

Match to: **gi|119583453** Score: **78** Expect: **0.0028**
asparaginyl-tRNA synthetase, isoform CRA_b [Homo sapiens]

Nominal mass (M_r): **63687**; Calculated pI value: **5.90**
NCBI BLAST search of gi|119583453 against nr
Unformatted sequence string for pasting into other applications

Taxonomy: Homo sapiens

Fixed modifications: Carbamidomethyl (C)
Variable modifications: Oxidation (M)
Cleavage by Trypsin: cuts C-term side of KR unless next residue is P
Sequence Coverage: **11%**

Matched peptides shown in **Bold Red**

```
  1 MVLELYVSDR EGSDATGDGT KEKPFKTGLK ALMTVGKEPF PTIYVDSQKE
 51 NERWNVISKS QLKNIKKMWH REQMKSESRE KKEAEDSLRR EKNLEEAKKI
101 TIKNDPSLPE PKCVKIGALE GYRGQRVKVF GWVHRLRRQG KNLMFLVLRD
151 GTGYLQCVLA DELCQCYNGV LLSTESSVAV YGMLNLTPKG KQAPGGHELS
201 CDFWELIGLA PAGGADNLIN EESDVDVQLN NRHMMIRGEN MSKILKARSM
251 VTRCFRDHFF DRGYYEVTPP TLVQTQVEGG ATLFKLDYFG EEAFLTQSSQ
301 LYLETCLPAL GDVFCIAQSY RAEQSRTRRH LAEYTHVEAE CPFLTFDDLL
351 NRLEDLVCDV VDRILKSPAG SIVHELNPNF QPPKRPFKRM NYSDAIVWLK
401 EHDVKKEDGT FYEFGEDIPE APERLMTDTI NEPILLCRFP VEIKSFYMQR
451 CPEDSRLTES VDVLMPNVGE IVGGSMRIFD SEEILAGYKR EGIDPTPYYW
501 YTDQRKYGTC PHGGYGLGLE RFLTWILNRY HIRDVCLYPR FVQRCTP
```

Residue Number Increasing Mass Decreasing Mass

Start - End	Observed	Mr(expt)	Mr(calc)	Delta	Miss	Sequence
142 - 149	1021.6087	1020.6014	1020.5790	0.0225	0	**NLMFLVLR** Oxidation (M) (No match)
142 - 149	1021.6087	1020.6014	1020.5790	0.0225	0	**NLMFLVLR** Oxidation (M) (No match)
257 - 262	836.3879	835.3806	835.3613	0.0194	0	**DHFFDR** (No match)
425 - 438	1704.9265	1703.9192	1703.8586	0.0606	0	**LMTDTINEPILLCR** Oxidation (M) (No match)
445 - 450	847.3899	846.3826	846.3694	0.0132	0	**SFYMQR** Oxidation (M) (No match)
478 - 490	1540.8506	1539.8433	1539.7932	0.0501	1	**IFDSEEILAGYKR** (Ions score 25)

478 - 490	1540.8506	1539.8433	1539.7932	0.0501	1	IFDSEEILAGYKR	(No match)
507 - 521	1636.8123	1635.8050	1635.7463	0.0587	0	YGTCPHGGYGLGLER	(No match)
507 - 521	1636.8123	1635.8050	1635.7463	0.0587	0	YGTCPHGGYGLGLER	(Ions score 10)

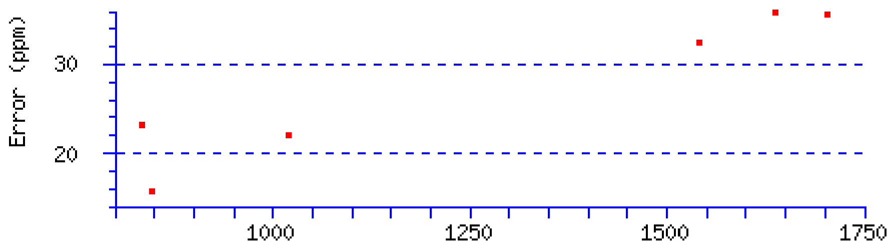

RMS error 29 ppm

Mascot: http://www.matrixscience.com/

Spot 932

Protein View

Match to: **gi|119591666** Score: **176** Expect: **4.8e-013**
septin 2, isoform CRA_c [Homo sapiens]

Nominal mass (M_r): **18259**; Calculated pI value: **6.20**
NCBI BLAST search of gi|119591666 against nr
Unformatted sequence string for pasting into other applications

Taxonomy: Homo sapiens

Fixed modifications: Carbamidomethyl (C)
Variable modifications: Oxidation (M)
Cleavage by Trypsin: cuts C-term side of KR unless next residue is P
Sequence Coverage: **34%**

Matched peptides shown in **Bold Red**

```
  1 MSKQQPTQFI NPETPGYVGF ANLPNQVHRK SVKKGFEFTL MVVGESGLGK
 51 STLINSLFLT DLYPERVIPG AAEKIERTVQ IEASTVEIEE RGVKLRLTVV
101 DTPGYGDAIN CRDCFKTIIS YIDEQFERYL HDESGLNRRH IIDNRVHCCF
151 YFISPFGHG
```

Residue Number Increasing Mass Decreasing Mass

Start - End	Observed	Mr(expt)	Mr(calc)	Delta	Miss	Sequence
51 - 66	1881.9924	1880.9851	1880.9883	-0.0032	0	STLINSLFLTDLYPER (Ions score 38)
51 - 66	1881.9924	1880.9851	1880.9883	-0.0032	0	STLINSLFLTDLYPER (No match)
97 - 112	1750.8407	1749.8334	1749.8355	-0.0021	0	LTVVDTPGYGDAINCR (Ions score 28)
97 - 112	1750.8407	1749.8334	1749.8355	-0.0021	0	LTVVDTPGYGDAINCR (No match)
117 - 128	1513.7567	1512.7494	1512.7460	0.0035	0	TIISYIDEQFER (No match)
117 - 128	1513.7567	1512.7494	1512.7460	0.0035	0	TIISYIDEQFER (Ions score 54)
129 - 139	1359.6823	1358.6750	1358.6691	0.0060	1	YLHDESGLNRR (No match)

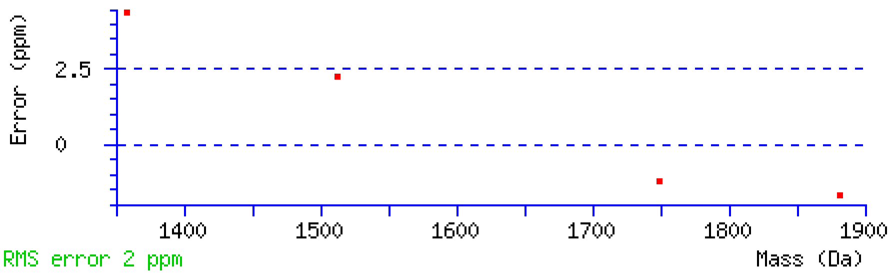

Mascot: http://www.matrixscience.com/

Spot 936

Protein View

Match to: **gi|119594451** Score: **127** Expect: **3.8e-008**
glucosidase, alpha; neutral AB, isoform CRA_a [Homo sapiens]

Nominal mass (M_r): **104930**; Calculated pI value: **5.85**
NCBI BLAST search of gi|119594451 against nr
Unformatted sequence string for pasting into other applications

Taxonomy: Homo sapiens

Fixed modifications: Carbamidomethyl (C)
Variable modifications: Oxidation (M)
Cleavage by Trypsin: cuts C-term side of KR unless next residue is P
Sequence Coverage: **16%**

Matched peptides shown in **Bold Red**

```
  1 MAAVAAVAAR RRRSWASLVL AFLGVCLGIT LAVDRSNFKT CEESSFCKRQ
 51 RSIRPGLSPY RALLDSLQLG PDSLTVHLIH EVTKVLLVLE LQGLQKNMTR
101 FRIDELEPRR PRYRVPDVLV ADPPIARLSV SGRDENSVEL TMAEGPYKII
151 LTARPFRLDL LEDRSLLLSV NARGLLEFEH QRAPRVSEEE TQGKAEKDEP
201 GAWEETFKTH SDSKPYGPMS VGLDFSLPGM EHVYGIPEHA DNLRLKVTEG
251 GEPYRLYNLD VFQYELYNPM ALYGSVPVLL AHNPHRDLGI FWLNAAETWV
301 DISSNTAGKT LFGKMMDYLQ GSGETPQTDV RWMSETGIID VFLLLGPSIS
351 DVFRQYASLT GTQALPPLFS LGYHQSRWNY RDEADVLEVD QGFDDHNLPC
401 DVIWLDIEHA DGKRYFTWDP SRFPQPRTML ERLASKRRKL VAIVDPHIKV
451 DSGYRVHEEL RNLGLYVKTR DGSDYEGWCW PGSAGYPDFT NPTMRAWWAN
501 MFSYDNYEGS APNLFVWNDM NEPSVFNGPE VTMLKDAQHY GGWEHRDVHN
551 IYGLYVHMAT ADGLRQRSGG MERPFVLARA FFAGSQRFGA VWTGDNTAEW
601 DHLKISIPMC LSLGLVGLSF CGADVGGFFK NPEPELLVRW YQMGAYQPFF
651 RAHAHLDTGR REPWLLPSQH NDIIRDALGQ RYSLLPFWYT LLYQAHREGI
701 PVMRPLWVQY PQDVTTFNID DQYLLGDALL VHPVSDSGAH GVQVYLPGQG
751 EVWYDIQSYQ KHHGPQTLYL PVTLSSIPVF QRGGTIVPRW MRVRRSSECM
801 KDDPITLFVA LSPQGTAQGE LFLDDGHTFN YQTRQEFLLR RFSFSGNTLV
851 SSSADPEGHF ETPIWIERVV IIGAGKPAAV VLQTKGSPES RLSFQHDPET
901 SVLVLRKPGI NVASDWSIHL R
```

Residue Number Increasing Mass Decreasing Mass

Start - End	Observed	Mr(expt)	Mr(calc)	Delta	Miss	Sequence
52 - 61	1145.6357	1144.6284	1144.6352	-0.0068	0	SIRPGLSPYR (No match)
52 - 61	1145.6357	1144.6284	1144.6352	-0.0068	0	SIRPGLSPYR (No match)
101 - 109	1174.6138	1173.6065	1173.6142	-0.0076	1	FRIDELEPR (No match)
101 - 109	1174.6138	1173.6065	1173.6142	-0.0076	1	FRIDELEPR (No match)
113 - 127	1680.9321	1679.9248	1679.9358	-0.0110	1	YRVPDVLVADPPIAR (No match)
174 - 182	1128.5756	1127.5683	1127.5723	-0.0040	0	GLLEFEHQR (No match)
174 - 182	1128.5756	1127.5683	1127.5723	-0.0040	0	GLLEFEHQR (Ions score 5)
245 - 255	1248.6525	1247.6452	1247.6509	-0.0057	1	LKVTEGGEPYR (No match)
247 - 255	1007.4751	1006.4678	1006.4719	-0.0041	0	VTEGGEPYR (No match)
415 - 427	1696.8967	1695.8894	1695.8157	0.0737	1	YFTWDPSRFPQPR (No match)
456 - 461	782.4550	781.4477	781.4082	0.0395	0	VHEELR (No match)
547 - 565	2161.0239	2160.0166	2160.0421	-0.0255	0	DVHNIYGLYVHMATADGLR Oxidation (M) (No match)
568 - 579	1335.6747	1334.6674	1334.6764	-0.0090	0	SGGMERPFVLAR Oxidation (M) (No match)
580 - 587	883.4374	882.4301	882.4347	-0.0046	0	AFFAGSQR (No match)
762 - 782	2390.2705	2389.2632	2389.2906	-0.0274	0	HHGPQTLYLPVTLSSIPVFQR (No match)
762 - 782	2390.2705	2389.2632	2389.2906	-0.0274	0	HHGPQTLYLPVTLSSIPVFQR (Ions score 4)
835 - 841	961.5518	960.5445	960.5504	-0.0059	1	QEFLLRR (No match)
892 - 906	1740.9141	1739.9068	1739.9206	-0.0138	0	LSFQHDPETSVLVLR (No match)
892 - 906	1740.9141	1739.9068	1739.9206	-0.0138	0	LSFQHDPETSVLVLR (Ions score 11)

Mascot: http://www.matrixscience.com/

Spot 939

Protein View

Match to: **gi|14286326** Score: **84** Expect: **0.00077**
Phosphofructokinase, liver [Homo sapiens]

Nominal mass (M_r): **85720**; Calculated pI value: **7.01**
NCBI BLAST search of gi|14286326 against nr
Unformatted sequence string for pasting into other applications

Taxonomy: Homo sapiens
Links to retrieve other entries containing this sequence from NCBI Entrez:
gi|14602832 from Homo sapiens

Fixed modifications: Carbamidomethyl (C)
Variable modifications: Oxidation (M)
Cleavage by Trypsin: cuts C-term side of KR unless next residue is P
Sequence Coverage: **11%**

Matched peptides shown in **Bold Red**

```
  1 MAAVDLEKLR ASGAGKAIGV LTSGGDAQGM NAAVRAVTRM GIYVGAKVFL
 51 IYEGYEGLVE GGENIKQANW LSVSNIIQLG GTIIGSARCK AFTTREGRRA
101 AAYNLVQHGI TNLCVIGGDG SLTGANIFRS EWGSLLEELV AEGKISETTA
151 RTYSHLNIAG LVGSIDNDFC GTDMTIGTDS ALHRIMEVID AITTTAQSHQ
201 RTFVLEVMGR HCGYLALVSA LASGADWLFI PEAPPEDGWE NFMCERLGET
251 RSRGSRLNII IIAEGAIDRN GKPISSSYVK DLVVQRLGFD TRVTVLGHVQ
301 RGGTPSAFDR ILSSKMGMEA VMALLEATPD TPACVVTLSG NQSVRLPLME
351 CVQMTKEVQK AMDDKRFDEA TQLRGGSFEN NWNIYKLLAH QKPPKEKSNF
401 SLAILNVGAP AAGMNAAVRS AVRTGISHGH TVYVVHDGFE GLAKGQVQEV
451 GWHDVAGWLG RGGSMLGTKR TLPKGQLESI VENIRIYGIH ALLVVGGFEA
501 YEGVLQLVEA RGRYEELCIV MCVIPATISN NVPGTDFSLG SDTAVNAAME
551 SCDRIKQSAS GTKRRVFIVE TMGGYCGYLA TVTGIAVGAD AAYVFEDPFN
601 IHDLKVNVEH MTEKMKTDIQ RGLVLRNEKC HDYYTTEFLY NLYSSEGNGV
651 FDCRTNVLGH LQQGGAPTPF DRNYGTKLGV KAMLWLSEKL REVYRKGRVF
701 ANAPDSACVI GLKKKAAAFS PVTELKKDTD FEHRMPREQW WLSLRLMLKM
751 LAQYRISMAA YVSGELEHVT RRTLSMDKGF
```

Residue Number Increasing Mass Decreasing Mass

Start - End	Observed	Mr(expt)	Mr(calc)	Delta	Miss	Sequence
185 - 201	1930.0480	1929.0407	1928.9625	0.0782	0	IMEVIDAITTTAQSHQR Oxidation (M) (No match)
202 - 210	1067.5900	1066.5827	1066.5481	0.0347	0	TFVLEVMGR Oxidation (M) (No match)
257 - 269	1410.8868	1409.8795	1409.8241	0.0554	0	LNIIIIAEGAIDR (Ions score 21)
257 - 269	1410.8868	1409.8795	1409.8241	0.0554	0	LNIIIIAEGAIDR (No match)
398 - 419	2160.2109	2159.2036	2159.1156	0.0880	0	SNFSLAILNVGAPAAGMNAAVR Oxidation (M) (No match)
398 - 419	2160.2109	2159.2036	2159.1156	0.0880	0	SNFSLAILNVGAPAAGMNAAVR Oxidation (M) (Ions score 10)
475 - 485	1257.7296	1256.7223	1256.6724	0.0499	0	GQLESIVENIR (No match)
475 - 485	1257.7296	1256.7223	1256.6724	0.0499	0	GQLESIVENIR (No match)
655 - 672	1908.0453	1907.0380	1906.9649	0.0731	0	TNVLGHLQQGGAPTPFDR (No match)
655 - 672	1908.0453	1907.0380	1906.9649	0.0731	0	TNVLGHLQQGGAPTPFDR (No match)

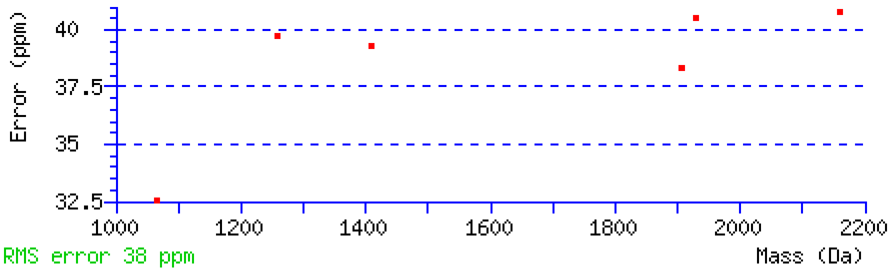

RMS error 38 ppm

Mascot: http://www.matrixscience.com/

Spot 940

Protein View

Match to: **gi|48145705** Score: **89** Expect: **0.00024**
HCLS1 [Homo sapiens]

Nominal mass (M_r): **54065**; Calculated pI value: **4.74**
NCBI BLAST search of gi|48145705 against nr
Unformatted sequence string for pasting into other applications

Taxonomy: Homo sapiens

Fixed modifications: Carbamidomethyl (C)
Variable modifications: Oxidation (M)
Cleavage by Trypsin: cuts C-term side of KR unless next residue is P
Sequence Coverage: **9%**

Matched peptides shown in **Bold Red**

```
  1 MWKSVVGHDV SVSVETQGDD WDTDPDFVND ISEKEQRWGA KTIEGSGRTE
 51 HINIHQLRNK VSEEHDVLRK KEMESGPKAS HGYGGRFGVE RDRMDKSAVG
101 HEYVAEVEKH SSQTDAAKGF GGKYGVERDR ADKSAVGFDY KGEVEKHTSQ
151 KDYSRGFGGR YGVEKDKWDK AALGYDYKGE TEKHESQRDY AKGFGGQYGI
201 QKDRVDKSAV GFNEMEAPTT AYKKTTPIEA ASSGARGLKA KFESMAEEKR
251 KREEEEKAQQ VARRQQERKA VTKRSPEAPQ PVIAMEEPAV PAPLPKKISS
301 EAWPPVGTPP SSESEPVRTS REHPVPLLPI RQTLPEDNEE PPALPPRTLE
351 GLQVEEEPVY EAEPEPEPEP EPEPENDYED VEEMDRHEQE DEPEGDYEEV
401 LEPEDSSFSS ALAGSSGCPA GAGAGAVALG ISAVALYDYQ GEGSDELSFD
451 PDDVITDIEM VDEGWWRGRC HGHFGLFPAN YVKLLD
```

Residue Number Increasing Mass Decreasing Mass

Start - End	Observed	Mr(expt)	Mr(calc)	Delta	Miss	Sequence	
49 - 58	1260.7417	1259.7344	1259.6734	0.0610	0	TEHINIHQLR	(No match)
193 - 202	1054.5702	1053.5629	1053.5243	0.0387	0	GFGGQYGIQK	(No match)
193 - 202	1054.5702	1053.5629	1053.5243	0.0387	0	GFGGQYGIQK	(No match)
322 - 331	1170.7509	1169.7436	1169.6920	0.0516	0	EHPVPLLPIR	(Ions score 18)
322 - 331	1170.7509	1169.7436	1169.6920	0.0516	0	EHPVPLLPIR	(No match)
332 - 347	1802.9761	1801.9688	1801.8846	0.0842	0	QTLPEDNEEPPALPPR	(No match)
332 - 347	1802.9761	1801.9688	1801.8846	0.0842	0	QTLPEDNEEPPALPPR	(Ions score 47)

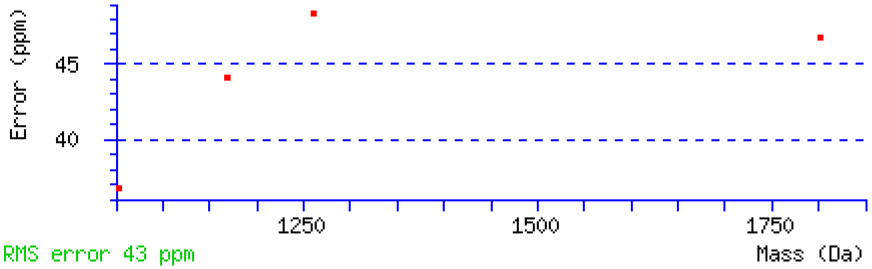

Mascot: http://www.matrixscience.com/

Spot 941

Protein View

Match to: **gi|6807647** Score: **288** Expect: **3e-024**
hypothetical protein [Homo sapiens]

Nominal mass (M_r): **85189;** Calculated pI value: **5.26**
NCBI BLAST search of gi|6807647 against nr
Unformatted sequence string for pasting into other applications

Taxonomy: Homo sapiens

Fixed modifications: Carbamidomethyl (C)
Variable modifications: Oxidation (M)
Cleavage by Trypsin: cuts C-term side of KR unless next residue is P
Sequence Coverage: **29%**

Matched peptides shown in **Bold Red**

```
  1 MPEEVHHGEE EVETFAFQAE IAQLMSLIIN TFYSNKEIFL RELISNASDA
 51 LDKIRYESLT DPSKLDSGKE LKIDIIPNPQ ERTLTLVDTG IGMTKADLIN
101 NLGTIAKSGT KAFMEALQAG ADISMIGQFG VGFYSAYLVA EKVVVITKHN
151 DDEQYAWESS AGGSFTVRAD HGEPIGRGTK VILHLKEDQT EYLEERRVKE
201 VVKKHSQFIG YPITLYLEKE REKEISDDEA EEEKGEKEEE DKDDEEKPKI
251 EDVGSDEEDD SGKDKKKKTK KIKEKYIDQE ELNKTKPIWT RNPDDITQEE
301 YGEFYKSLTN DWEDHLAVKH FSVEGQLEFR ALLFIPRRAP FDLFENKKKK
351 NNIKLYVRRV FIMDSCDELI PEYLNFIRGV VDSEDLPLNI SREMLQQSKI
401 LKAIRKNIVK KCLELFSELA EDKENYKKFY EAFSKNLKLG IHEDSTNRRR
451 LSELLRYHTS QSGDEMTSLS EYVSRMKETQ KSIYYITGES KEQVANSAFV
501 ERVRKRGFEV VYMTEPIDEY CVQQLKEFDG KSLVSVTKEG LELPEDEEEK
551 KKMEESKAKF ENLCKLMKEI LDKKVEKVTI SNRLVSSPCC IVTSTYGWTA
601 NMERIMKAQA LRDNSTMGYM MAKKHLEINP DHPIVETLRQ KAEADKNDKA
651 VKDLVVLLFE TALLSSGFSL EDPQTHSNRI YRMIKLGLGI DEDEVAAEEP
701 NAAVPDEIPL SRAMRMRLAW KKSIRLGVHS WKTCALV
```

Residue Number Increasing Mass Decreasing Mass

Start - End	Observed	Mr(expt)	Mr(calc)	Delta	Miss	Sequence
42 - 55	1544.8713	1543.8640	1543.8205	0.0435	1	ELISNASDALDKIR (No match)
70 - 82	1564.9144	1563.9071	1563.8620	0.0451	1	ELKIDIIPNPQER (No match)

73 - 82	1194.6803	1193.6730	1193.6404	0.0326	0	IDIIPNPQER	(No match)
73 - 82	1194.6803	1193.6730	1193.6404	0.0326	0	IDIIPNPQER	(Ions score 43)
169 - 177	951.4883	950.4810	950.4569	0.0241	0	ADHGEPIGR	(No match)
181 - 196	2015.0950	2014.0877	2014.0370	0.0507	1	VILHLKEDQTEYLEER	(No match)
205 - 219	1809.0054	1807.9981	1807.9508	0.0473	0	HSQFIGYPITLYLEK	(No match)
276 - 284	1151.5847	1150.5774	1150.5506	0.0269	0	YIDQEELNK	(No match)
292 - 306	1847.8541	1846.8468	1846.7897	0.0572	0	NPDDITQEEYGEFYK	(No match)
320 - 330	1348.7034	1347.6961	1347.6571	0.0391	0	HFSVEGQLEFR	(No match)
320 - 330	1348.7034	1347.6961	1347.6571	0.0391	0	HFSVEGQLEFR	(Ions score 13)
331 - 337	829.5451	828.5378	828.5221	0.0157	0	ALLFIPR	(No match)
338 - 347	1236.6686	1235.6613	1235.6298	0.0315	1	RAPFDLFENK	(No match)
338 - 347	1236.6686	1235.6613	1235.6298	0.0315	1	RAPFDLFENK	(Ions score 17)
379 - 392	1513.8287	1512.8214	1512.7783	0.0431	0	GVVDSEDLPLNISR	(No match)
379 - 392	1513.8287	1512.8214	1512.7783	0.0431	0	GVVDSEDLPLNISR	(Ions score 57)
429 - 435	891.4417	890.4344	890.4174	0.0171	0	FYEAFSK	(No match)
439 - 448	1141.5918	1140.5845	1140.5523	0.0322	0	LGIHEDSTNR	(No match)
457 - 475	2193.0081	2192.0008	2191.9327	0.0681	0	YHTSQSGDEMTSLSEYVSR Oxidation (M)	(No match)
482 - 502	2391.2329	2390.2256	2390.1753	0.0503	1	SIYYITGESKEQVANSAFVER	(No match)
492 - 502	1249.6515	1248.6442	1248.6098	0.0344	0	EQVANSAFVER	(No match)
624 - 639	1911.0924	1910.0851	1910.0373	0.0478	1	KHLEINPDHPIVETLR	(No match)
625 - 639	1782.9977	1781.9904	1781.9424	0.0480	0	HLEINPDHPIVETLR	(No match)
726 - 737	1370.6826	1369.6753	1369.7176	-0.0423	1	LGVHSWKTCALV	(No match)

Mascot: http://www.matrixscience.com/

Spot 943

Protein View

Match to: **gi|11496277** Score: **190** Expect: **1.9e-014**
mitogen-activated protein kinase kinase 1 interacting protein 1 [Homo sapiens]

Nominal mass (M_r): **13671**; Calculated pI value: **6.73**
NCBI BLAST search of gi|11496277 against nr
Unformatted sequence string for pasting into other applications

Taxonomy: Homo sapiens
Links to retrieve other entries containing this sequence from NCBI Entrez:
gi|109075110 from Macaca mulatta
gi|114595356 from Pan troglodytes
gi|114595358 from Pan troglodytes
gi|114595360 from Pan troglodytes
gi|24418562 from Homo sapiens
gi|6563292 from Homo sapiens
gi|20072808 from Homo sapiens
gi|119626517 from Homo sapiens
gi|123992864 from synthetic construct
gi|123999680 from synthetic construct

Fixed modifications: Carbamidomethyl (C)
Variable modifications: Oxidation (M)
Cleavage by Trypsin: cuts C-term side of KR unless next residue is P
Sequence Coverage: **43%**

Matched peptides shown in **Bold Red**

```
  1 MADDLKRFLY KKLPSVEGLH AIVVSDRDGV PVIKVANDNA PEHALRPGFL
 51 STFALATDQG SKLGLSKNKS IICYYNTYQV VQFNRLPLVV SFIASSSANT
101 GLIVSLEKEL APLFEELRQV VEVS
```

Residue Number Increasing Mass Decreasing Mass

Start - End	Observed	Mr(expt)	Mr(calc)	Delta	Miss	Sequence
35 - 62	2927.4780	2926.4707	2926.4572	0.0135	0	VANDNAPEHALRPGFLSTFALATDQGSK (No match)
35 - 62	2927.4780	2926.4707	2926.4572	0.0135	0	VANDNAPEHALRPGFLSTFALATDQGSK (Ions score 2)
70 - 85	2068.0061	2066.9988	2066.9883	0.0105	0	SIICYYNTYQVVQFNR (Ions score 75)

70 - 85	2068.0061	2066.9988	2066.9883	0.0105	0	**SIICYYNTYQVVQFNR**	(No match)
109 - 118	1216.6702	1215.6629	1215.6499	0.0131	0	**ELAPLFEELR**	(No match)
109 - 118	1216.6702	1215.6629	1215.6499	0.0131	0	**ELAPLFEELR**	(Ions score 69)

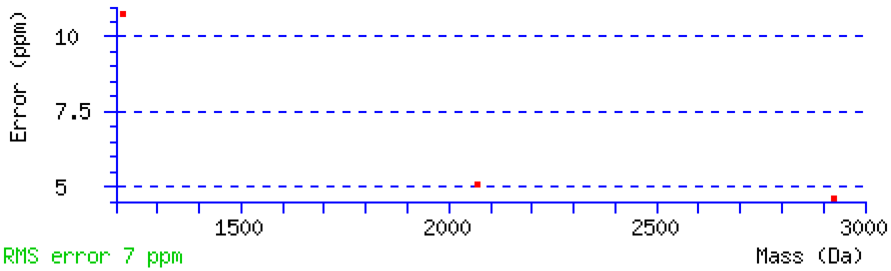

RMS error 7 ppm

Mascot: http://www.matrixscience.com/

Spot 944

Protein View

Match to: **gi|551547** Score: **72** Expect: **0.012**
prosome beta-subunit; HSBpros26 [Homo sapiens]

Nominal mass (M_r): **25950**; Calculated pI value: **5.70**
NCBI BLAST search of gi|551547 against nr
Unformatted sequence string for pasting into other applications

Taxonomy: Homo sapiens
Links to retrieve other entries containing this sequence from NCBI Entrez:
gi|743649 from Homo sapiens

Fixed modifications: Carbamidomethyl (C)
Variable modifications: Oxidation (M)
Cleavage by Trypsin: cuts C-term side of KR unless next residue is P
Sequence Coverage: **12%**

Matched peptides shown in **Bold Red**

```
  1 MDPASALYRG PITRTQNPMV TGTSVLGVKF EGGVVIAADM LGSYGSLARF
 51 RNISRIMRVN NSTMLGASGD YADFQYLKQV LGQMVIDEEL LGDGHSYSPR
101 AIHSWLTRAM YSRRSKMNPL WNTMVIGGYA DGESFLGYVD MLGVAYEAPS
151 LATGYGAYLA QPLLREVLEK QPVLSQTEAR DLVERCMRVL YYRDARSYNR
201 FQTATVTEKG VEIEGPLSTE TNWDIAHMIS GFE
```

Residue Number Increasing Mass Decreasing Mass

Start - End	Observed	Mr(expt)	Mr(calc)	Delta	Miss	Sequence
30 - 49	2028.9646	2027.9573	2027.9985	-0.0412	0	FEGGVVIAADMLGSYGSLAR Oxidation (M) (No match)
30 - 49	2028.9646	2027.9573	2027.9985	-0.0412	0	FEGGVVIAADMLGSYGSLAR Oxidation (M) (Ions score 34)
101 - 108	983.5383	982.5310	982.5348	-0.0038	0	AIHSWLTR (No match)
101 - 108	983.5383	982.5310	982.5348	-0.0038	0	AIHSWLTR (Ions score 9)

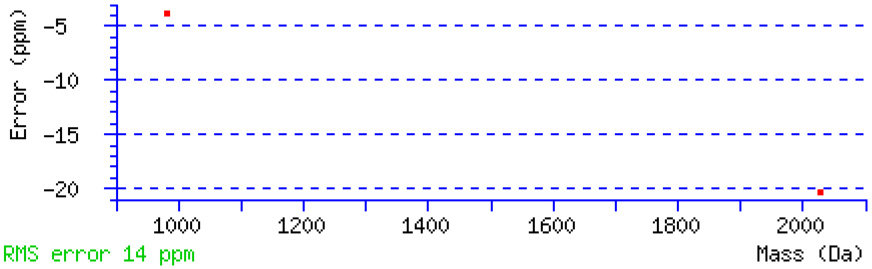

Mascot: http://www.matrixscience.com/

Spot 945

Protein View

Match to: **gi|4758638** Score: **415** Expect: **6.1e-037**
peroxiredoxin 6 [Homo sapiens]

Nominal mass (M_r): **25133;** Calculated pI value: **6.00**
NCBI BLAST search of gi|4758638 against nr
Unformatted sequence string for pasting into other applications

Taxonomy: Homo sapiens
Links to retrieve other entries containing this sequence from NCBI Entrez:
gi|114565483 from Pan troglodytes
gi|114621471 from Pan troglodytes
gi|1718024 from Homo sapiens
gi|285949 from Homo sapiens
gi|23274223 from Homo sapiens
gi|31657160 from Homo sapiens
gi|56204402 from Homo sapiens
gi|77744395 from Homo sapiens
gi|119611350 from Homo sapiens
gi|119611351 from Homo sapiens
gi|158259727 (no taxonomy information for this entry)

Fixed modifications: Carbamidomethyl (C)
Variable modifications: Oxidation (M)
Cleavage by Trypsin: cuts C-term side of KR unless next residue is P
Sequence Coverage: **42%**

Matched peptides shown in **Bold Red**

```
  1 MPGGLLLGDV APNFEANTTV GRIRFHDFLG DSWGILFSHP RDFTPVCTTE
 51 LGRAAKLAPE FAKRNVKLIA LSIDSVEDHL AWSKDINAYN CEEPTEKLPF
101 PIIDDRNREL AILLGMLDPA EKDEKGMPVT ARVVFVFGPD KKLKLSILYP
151 ATTGRNFDEI LRVVISLQLT AEKRVATPVD WKDGDSVMVL PTIPEEEAKK
201 LFPKGVFTKE LPSGKKYLRY TPQP
```

Residue Number Increasing Mass Decreasing Mass

Start - End Observed Mr(expt) Mr(calc) Delta Miss Sequence

2 - 22	2098.0654	2097.0581	2097.0854	-0.0272	0	PGGLLLGDVAPNFEANTTVGR (No match)
2 - 22	2098.0654	2097.0581	2097.0854	-0.0272	0	PGGLLLGDVAPNFEANTTVGR (Ions score 126)
42 - 53	1395.6437	1394.6364	1394.6500	-0.0136	0	DFTPVCTTELGR (No match)
42 - 53	1395.6437	1394.6364	1394.6500	-0.0136	0	DFTPVCTTELGR (Ions score 70)
85 - 97	1582.6521	1581.6448	1581.6617	-0.0168	0	DINAYNCEEPTEK (No match)
98 - 106	1085.5933	1084.5860	1084.5917	-0.0056	0	LPFPIIDDR (No match)
98 - 106	1085.5933	1084.5860	1084.5917	-0.0056	0	LPFPIIDDR (Ions score 32)
133 - 142	1135.6388	1134.6315	1134.6436	-0.0121	1	VVFVFGPDKK (Ions score 30)
133 - 142	1135.6388	1134.6315	1134.6436	-0.0121	1	VVFVFGPDKK (No match)
145 - 155	1191.6654	1190.6581	1190.6659	-0.0078	0	LSILYPATTGR (Ions score 29)
145 - 155	1191.6654	1190.6581	1190.6659	-0.0078	0	LSILYPATTGR (No match)
156 - 162	906.4651	905.4578	905.4606	-0.0028	0	NFDEILR (No match)
163 - 174	1356.8107	1355.8034	1355.8136	-0.0102	1	VVISLQLTAEKR (No match)

Mascot: http://www.matrixscience.com/

Spot 946

Protein View

Match to: **gi|119570325** Score: **69** Expect: **0.025**
phosphoglycerate mutase 1 (brain), isoform CRA_c [Homo sapiens]

Nominal mass (M_r): **14942**; Calculated pI value: **9.30**
NCBI BLAST search of gi|119570325 against nr
Unformatted sequence string for pasting into other applications

Taxonomy: Homo sapiens

Fixed modifications: Carbamidomethyl (C)
Variable modifications: Oxidation (M)
Cleavage by Trypsin: cuts C-term side of KR unless next residue is P
Sequence Coverage: **24%**

Matched peptides shown in **Bold Red**

```
  1 ARRATRRRSA AGRRYEDRRY ADLTEDQLPS CESLKDTIAR ALPFWNEEIV
 51 PQIKEGKRVL IAAHGNSLRG IVKHLEGLSE EAIMELNLPT GIPIVYELDK
101 NLKPIKPMQF LGDEETVRKA MEAVAAQGKA KK
```

Residue Number Increasing Mass Decreasing Mass

Start - End	Observed	Mr(expt)	Mr(calc)	Delta	Miss	Sequence
20 - 40	2425.1199	2424.1126	2424.1478	-0.0352	1	YADLTEDQLPSCESLKDTIAR (No match)
20 - 40	2425.1199	2424.1126	2424.1478	-0.0352	1	YADLTEDQLPSCESLKDTIAR (Ions score 44)
59 - 69	1150.6648	1149.6575	1149.6618	-0.0043	0	VLIAAHGNSLR (No match)
59 - 69	1150.6648	1149.6575	1149.6618	-0.0043	0	VLIAAHGNSLR (No match)

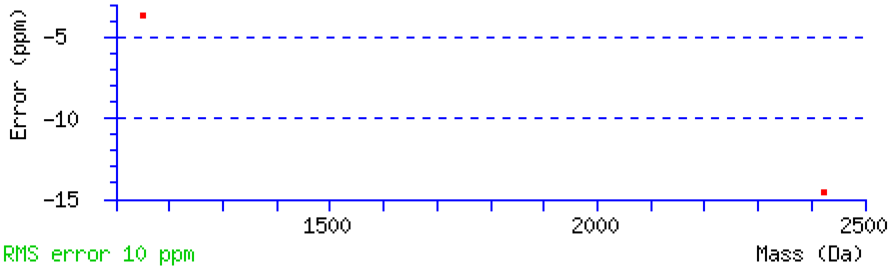

Mascot: http://www.matrixscience.com/

Spot 947

Protein View

Match to: **gi|119594432** Score: **166** Expect: **4.8e-012**
eukaryotic translation elongation factor 1 gamma, isoform CRA_d [Homo sapiens]

Nominal mass (M_r): **48749**; Calculated pI value: **6.24**
NCBI BLAST search of gi|119594432 against nr
Unformatted sequence string for pasting into other applications

Taxonomy: Homo sapiens

Fixed modifications: Carbamidomethyl (C)
Variable modifications: Oxidation (M)
Cleavage by Trypsin: cuts C-term side of KR unless next residue is P
Sequence Coverage: **19%**

Matched peptides shown in **Bold Red**

```
  1 MAAGTLYTYP ENWRAFKALI AAQYSGAQVR VLSAPPHFHF GQTNRTPEFL
 51 RKFPAGKVPA FEGDDGFCVF ESNAIAYYVS NEELRGSTPE AAAQVVQWVS
101 FADSDIVPPA STWVFPTLGI MHHNKQLDAY LKTRTFLVGE RVTLADITVV
151 CTLLWLYKQV LEPSFRQAFP NTNRWFLTCI NQPQFRAVLG EVKLCEKMAQ
201 FDAKKFAETQ PKKDTPRKEK GSREEKQKPQ AERKEEKKAA APAPEEEMDE
251 CEQALAAEPK AKDPFAHLPK STFVLDEFKR KYSNEDTLSV ALPYFWEHFD
301 KDGWSLWYSE YRFPEELTQT FMSCNLITGM FQRLDKLRKN AFASVILFGT
351 NNSSSISGVW VFRGQELAFP LSPDWQVDYE SYTWRKLDPG SEETQTLVRE
401 YFSWEGAFQH VGKAFNQGKI FK
```

Residue Number Increasing Mass Decreasing Mass

Start - End	Observed	Mr(expt)	Mr(calc)	Delta	Miss	Sequence
1 - 14	1688.6945	1687.6872	1687.7664	-0.0792	0	MAAGTLYTYPENWR Oxidation (M) (No match)
18 - 30	1347.6847	1346.6774	1346.7306	-0.0532	0	ALIAAQYSGAQVR (No match)
18 - 30	1347.6847	1346.6774	1346.7306	-0.0532	0	ALIAAQYSGAQVR (Ions score 27)
31 - 45	1707.7997	1706.7924	1706.8641	-0.0716	0	VLSAPPHFHFGQTNR (No match)
31 - 45	1707.7997	1706.7924	1706.8641	-0.0716	0	VLSAPPHFHFGQTNR (Ions score 40)
126 - 132	850.5007	849.4934	849.4596	0.0338	0	QLDAYLK (No match)
135 - 141	821.4172	820.4099	820.4442	-0.0343	0	TFLVGER (No match)
159 - 166	975.4913	974.4840	974.5185	-0.0345	0	QVLEPSFR (No match)

261 - 270	1123.5753	1122.5680	1122.6185	-0.0505	1	AKDPFAHLPK	(No match)
271 - 280	1241.6038	1240.5965	1240.6451	-0.0486	1	STFVLDEFKR	(No match)
271 - 280	1241.6038	1240.5965	1240.6451	-0.0486	1	STFVLDEFKR	(Ions score 30)

Mascot: http://www.matrixscience.com/

Spot 948

Protein View

Match to: **gi|49456373** Score: **110** Expect: **1.9e-006**
PHB [Homo sapiens]

Nominal mass (M_r): **29871**; Calculated pI value: **5.57**
NCBI BLAST search of gi|49456373 against nr
Unformatted sequence string for pasting into other applications

Taxonomy: Homo sapiens

Fixed modifications: Carbamidomethyl (C)
Variable modifications: Oxidation (M)
Cleavage by Trypsin: cuts C-term side of KR unless next residue is P
Sequence Coverage: **33%**

Matched peptides shown in **Bold Red**

```
  1 MAAKVFESIG KFGLALAVAG GVVNSALYNV DAGHRAVIFD RFRGVQDIVV
 51 GEGTHFLIPW VQKPIIFDCR SRPRNVPVIT GSKDLQNVNI TLRILFRPVA
101 SQLPRIFTSI GEDYDERVLP SITTEILKSV VARFDAGELI TQRELVSRQV
151 SDDLTERVAT FGLILDDVSL THLTFGKEFT EAVEAKQVAQ QEAERARFVV
201 EKAEQQKKAA IISAEGDSKA AELIANSLAT AGDGLIELRK LEAAEDIAYQ
251 LSRSRNITYL PAGQSVLLQL PQ
```

Residue Number Increasing Mass Decreasing Mass

Start - End	Observed	Mr(expt)	Mr(calc)	Delta	Miss	Sequence
12 - 35	2371.2185	2370.2112	2370.2443	-0.0331	0	FGLALAVAGGVVNSALYNVDAGHR (No match)
94 - 105	1396.8281	1395.8208	1395.8350	-0.0142	0	ILFRPVASQLPR (Ions score 21)
94 - 105	1396.8281	1395.8208	1395.8350	-0.0142	0	ILFRPVASQLPR (No match)
106 - 117	1444.6500	1443.6427	1443.6517	-0.0090	0	IFTSIGEDYDER (No match)
106 - 117	1444.6500	1443.6427	1443.6517	-0.0090	0	IFTSIGEDYDER (No match)
134 - 143	1149.5803	1148.5730	1148.5825	-0.0095	0	FDAGELITQR (No match)
134 - 143	1149.5803	1148.5730	1148.5825	-0.0095	0	FDAGELITQR (No match)
220 - 239	1998.0585	1997.0512	1997.0792	-0.0280	0	AAELIANSLATAGDGLIELR (No match)
220 - 239	1998.0585	1997.0512	1997.0792	-0.0280	0	AAELIANSLATAGDGLIELR (Ions score 12)
240 - 253	1606.8271	1605.8198	1605.8362	-0.0163	1	KLEAAEDIAYQLSR (No match)

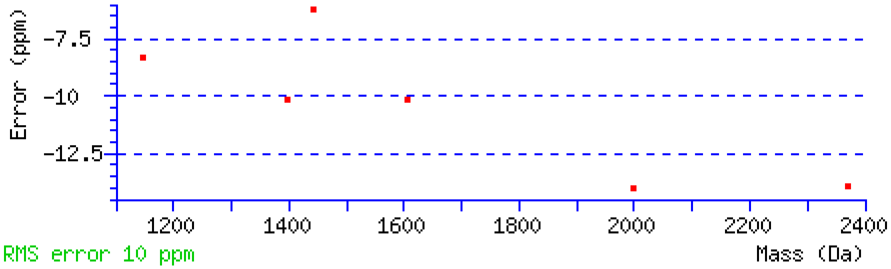

RMS error 10 ppm

Mascot: http://www.matrixscience.com/

Spot 951

Protein View

Match to: **gi|20149675** Score: **307** Expect: **3.8e-026**
EF-hand domain family, member D2 [Homo sapiens]

Nominal mass (M_r): **26795**; Calculated pI value: **5.15**
NCBI BLAST search of gi|20149675 against nr
Unformatted sequence string for pasting into other applications

Taxonomy: Homo sapiens
Links to retrieve other entries containing this sequence from NCBI Entrez:
gi|20140139 from Homo sapiens
gi|16507927 from Homo sapiens
gi|40226174 from Homo sapiens
gi|46250414 from Homo sapiens
gi|46255701 from Homo sapiens
gi|56203249 from Homo sapiens
gi|119572105 from Homo sapiens
gi|119572108 from Homo sapiens

Fixed modifications: Carbamidomethyl (C)
Variable modifications: Oxidation (M)
Cleavage by Trypsin: cuts C-term side of KR unless next residue is P
Sequence Coverage: **36%**

Matched peptides shown in **Bold Red**

```
  1 MATDELATKL SRRLQMEGEG GGETPEQPGL NGAAAAAAGA PDEAAEALGS
 51 ADCELSAKLL RRADLNQGIG EPQSPSRRVF NPYTEFKEFS RKQIKDMEKM
101 FKQYDAGRDG FIDLMELKLM MEKLGAPQTH LGLKNMIKEV DEDFDSKLSF
151 REFLLIFRKA AAGELQEDSG LCVLARLSEI DVSSEGVKGA KSFFEAKVQA
201 INVSSRFEEE IKAEQEERKK QAEEMKQRKA AFKELQSTFK
```

Residue Number Increasing Mass Decreasing Mass

Start - End	Observed	Mr(expt)	Mr(calc)	Delta	Miss	Sequence	
62 - 77	1724.7881	1723.7808	1723.8601	-0.0793	1	RADLNQGIGEPQSPSR	(Ions score 20)
63 - 77	1568.6975	1567.6902	1567.7590	-0.0688	0	ADLNQGIGEPQSPSR	(No match)
63 - 77	1568.6975	1567.6902	1567.7590	-0.0688	0	ADLNQGIGEPQSPSR	(Ions score 35)

63 - 78	1724.7881	1723.7808	1723.8601	-0.0793	1	ADLNQGIGEPQSPSRR (No match)
78 - 87	1300.6100	1299.6027	1299.6611	-0.0584	1	RVFNPYTEFK (No match)
79 - 87	1144.5087	1143.5014	1143.5600	-0.0586	0	VFNPYTEFK (No match)
79 - 91	1663.7410	1662.7337	1662.8041	-0.0704	1	VFNPYTEFKEFSR (No match)
79 - 91	1663.7410	1662.7337	1662.8041	-0.0704	1	VFNPYTEFKEFSR (Ions score 63)
109 - 118	1196.5187	1195.5114	1195.5794	-0.0680	0	DGFIDLMELK Oxidation (M) (No match)
124 - 134	1134.6277	1133.6204	1133.6556	-0.0352	0	LGAPQTHLGLK (No match)
152 - 158	937.5032	936.4959	936.5432	-0.0473	0	EFLLIFR (No match)
152 - 159	1065.5970	1064.5897	1064.6382	-0.0485	1	EFLLIFRK (No match)
152 - 159	1065.5970	1064.5897	1064.6382	-0.0485	1	EFLLIFRK (Ions score 13)
160 - 176	1759.7828	1758.7755	1758.8570	-0.0814	0	AAAGELQEDSGLCVLAR (No match)
207 - 218	1536.6471	1535.6398	1535.7103	-0.0705	1	FEEEIKAEQEER (Ions score 51)
207 - 218	1536.6471	1535.6398	1535.7103	-0.0705	1	FEEEIKAEQEER (No match)

Mascot: http://www.matrixscience.com/

Spot 952

Protein View

Match to: **gi|736704** Score: **90** Expect: **0.00021**
cytoskeleton associated protein [Homo sapiens]

Nominal mass (M_r): **21959;** Calculated pI value: **4.83**
NCBI BLAST search of gi|736704 against nr
Unformatted sequence string for pasting into other applications

Taxonomy: Homo sapiens
Links to retrieve other entries containing this sequence from NCBI Entrez:
gi|49456897 from Homo sapiens
gi|60821165 from synthetic construct

Fixed modifications: Carbamidomethyl (C)
Variable modifications: Oxidation (M)
Cleavage by Trypsin: cuts C-term side of KR unless next residue is P
Sequence Coverage: **10%**

Matched peptides shown in **Bold Red**

```
  1 MELELYGVDD KFYSKLDQED ALLGSYPVDD GCRIHVIDHS GARLGEYEDV
 51 SRVEKYTISQ EAYDQRQDTV RSFLKRSKLG RYNEEERAQQ EAEAAQRLAE
101 EKAQASSIPV GSRCEVRAAG QSPRRGTVMY VGLTDFKPGY WIGVRYDEPL
151 GKNDGSVNGK RYFECQAKYG AFVKPAVVTV GDFPEEDYGL DEI
```

Residue Number Increasing Mass Decreasing Mass

Start - End	Observed	Mr(expt)	Mr(calc)	Delta	Miss	Sequence	
34 - 43	1104.5892	1103.5819	1103.5835	-0.0016	0	IHVIDHSGAR	(No match)
34 - 43	1104.5892	1103.5819	1103.5835	-0.0016	0	IHVIDHSGAR	(Ions score 14)
56 - 66	1373.6224	1372.6151	1372.6259	-0.0107	0	YTISQEAYDQR	(No match)
56 - 66	1373.6224	1372.6151	1372.6259	-0.0107	0	YTISQEAYDQR	(Ions score 42)

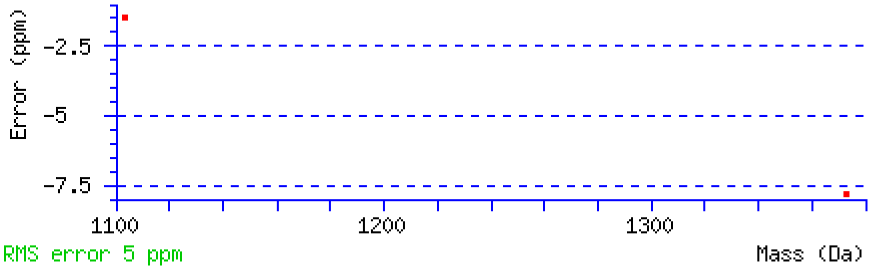

RMS error 5 ppm

Mascot: http://www.matrixscience.com/

Spot 953

Protein View

Match to: **gi|77019293** Score: **287** Expect: **3.8e-024**
MHC class II antigen [Homo sapiens]

Nominal mass (M_r): **21042**; Calculated pI value: **6.09**
NCBI BLAST search of gi|77019293 against nr
Unformatted sequence string for pasting into other applications

Taxonomy: Homo sapiens

Fixed modifications: Carbamidomethyl (C)
Variable modifications: Oxidation (M)
Cleavage by Trypsin: cuts C-term side of KR unless next residue is P
Sequence Coverage: **41%**

Matched peptides shown in **Bold Red**

1 RFLWQGKYKC HFFNGTERVQ FLER**LFYNQE EFVRFDSDVG EYRAVTELGR**
51 **PVAESWNSQK** DILEDRRGQV DNYCR**HNYGV GESFTVQR**RV HPEVTVYPAK
101 **TQPLQHHNLL VCSVSGFYPG SIEVR**WFRNG QEEKAGVVST GLIQNGDWTF
151 QTLVMLETVP RSGEVYTCQV EHPSVMSPL

Residue Number Increasing Mass Decreasing Mass

Start - End	Observed	Mr(expt)	Mr(calc)	Delta	Miss	Sequence
25 - 34	1344.6279	1343.6206	1343.6509	-0.0303	0	LFYNQEEFVR (Ions score 33)
25 - 34	1344.6279	1343.6206	1343.6509	-0.0303	0	LFYNQEEFVR (No match)
35 - 43	1087.4474	1086.4401	1086.4617	-0.0216	0	FDSDVGEYR (No match)
35 - 43	1087.4474	1086.4401	1086.4617	-0.0216	0	FDSDVGEYR (Ions score 25)
44 - 60	1871.9177	1870.9104	1870.9536	-0.0432	0	AVTELGRPVAESWNSQK (No match)
76 - 88	1493.6798	1492.6725	1492.7058	-0.0333	0	HNYGVGESFTVQR (Ions score 84)
76 - 88	1493.6798	1492.6725	1492.7058	-0.0333	0	HNYGVGESFTVQR (No match)
101 - 125	2838.3689	2837.3616	2837.4282	-0.0666	0	TQPLQHHNLLVCSVSGFYPGSIEVR (No match)
101 - 125	2838.3689	2837.3616	2837.4282	-0.0666	0	TQPLQHHNLLVCSVSGFYPGSIEVR (Ions score 62)

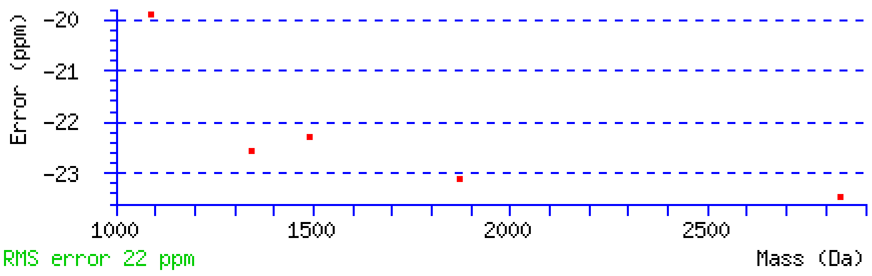

Mascot: http://www.matrixscience.com/

Spot 954

Protein View

Match to: **gi|15277503** Score: **94** Expect: **8e-005**
ACTB protein [Homo sapiens]

Nominal mass (M_r): **40536;** Calculated pI value: **5.55**
NCBI BLAST search of gi|15277503 against nr
Unformatted sequence string for pasting into other applications

Taxonomy: Homo sapiens

Fixed modifications: Carbamidomethyl (C)
Variable modifications: Oxidation (M)
Cleavage by Trypsin: cuts C-term side of KR unless next residue is P
Sequence Coverage: **9%**

Matched peptides shown in **Bold Red**

```
  1 MCKAGFAGDD APRAVFPSIV GRPRHQGVMV GMGQKDSYVG DEAQSKRGIL
 51 TLKYPIEHGI VTNWDDMEKI WHHTFYNELR VAPEEHPVLL TEAPLNPKAN
101 LEKMTQIMFE TFNTPAMYVA IQAVLSLYAS GRTTGIVMDS GDGVTHTVPI
151 YEGYALPHAI LRLDLAGRDL TDYLMKILTE RGYSFTTTAE REIVRDIKEK
201 LCYVALDFEQ EMATAASSSS LEKSYELPDG QVITIGNERF RCPEALFQPS
251 FLGMESCGIH ETTFNSIMKC DVDIRKDLYA NTVLSGGTTM YPGIADRMQK
301 EITALAPSTM KIKIIAPPER KYSVWIGGSI LASLSTFQQM WISKQEYDES
351 GPSIVHRKCF
```

Residue Number Increasing Mass Decreasing Mass

Start - End	Observed	Mr(expt)	Mr(calc)	Delta	Miss	Sequence	
81 - 98	1953.9972	1952.9899	1953.0571	-0.0671	0	VAPEEHPVLLTEAPLNPK	(No match)
81 - 98	1953.9972	1952.9899	1953.0571	-0.0671	0	VAPEEHPVLLTEAPLNPK	(Ions score 27)
224 - 239	1790.8314	1789.8241	1789.8846	-0.0604	0	SYELPDGQVITIGNER	(No match)
224 - 239	1790.8314	1789.8241	1789.8846	-0.0604	0	SYELPDGQVITIGNER	(Ions score 41)

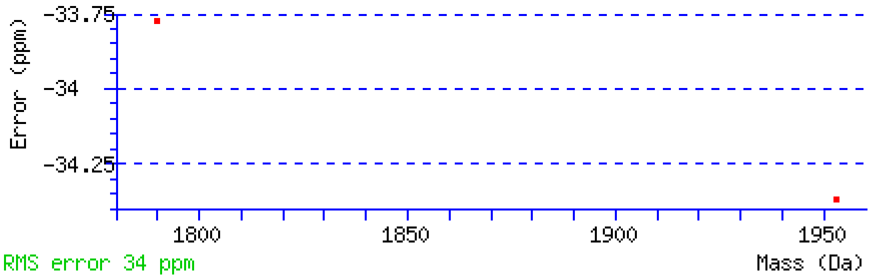

Mascot: http://www.matrixscience.com/

Spot 955

Protein View

Match to: **gi|118084558** Score: **309** Expect: **2.4e-026**
aryl hydrocarbon receptor interacting protein [Homo sapiens]

Nominal mass (M_r): **38049**; Calculated pI value: **5.83**
NCBI BLAST search of gi|118084558 against nr
Unformatted sequence string for pasting into other applications

Taxonomy: Homo sapiens

Fixed modifications: Carbamidomethyl (C)
Variable modifications: Oxidation (M)
Cleavage by Trypsin: cuts C-term side of KR unless next residue is P
Sequence Coverage: **22%**

Matched peptides shown in **Bold Red**

```
  1 MADIIARLRE DGIQKHVIQE GRGELPDFQD GTKATFHYRT LHSDDEGTVL
 51 DDSRARGKPM ELIIGKKFKL PVWETIVCTM REGEIAQFLC DIKHVVLYPL
101 VAKSLRNIAV GKDPLEGQRH CCGVAQMREH SSLGHADLDA LQQNPQPLIF
151 HMEMLKVESP GTYQQDPWAM TDEEKAKAVP LIHQEGNRLY REGHVKEAAA
201 KYYDAIACLK NLQMKEQPGS PEWIQLDQQI TPLLLNYCQC KLVVEEYYEV
251 LDHCSSILNK YDDNVKAYFK RGKAHAAVWN AQEAQADFAK VLELDPALAP
301 VVSRELQALE ARIRQKDEED KARFRGIFSH
```

Residue Number Increasing Mass Decreasing Mass

Start - End	Observed	Mr(expt)	Mr(calc)	Delta	Miss	Sequence
23 - 33	1206.5123	1205.5050	1205.5564	-0.0514	0	GELPDFQDGTK (Ions score 55)
23 - 33	1206.5123	1205.5050	1205.5564	-0.0514	0	GELPDFQDGTK (No match)
40 - 54	1659.6880	1658.6807	1658.7383	-0.0576	0	TLHSDDEGTVLDDSR (No match)
107 - 119	1396.7051	1395.6978	1395.7469	-0.0491	1	NIAVGKDPLEGQR (No match)
107 - 119	1396.7051	1395.6978	1395.7469	-0.0491	1	NIAVGKDPLEGQR (Ions score 46)
178 - 188	1233.6239	1232.6166	1232.6625	-0.0459	0	AVPLIHQEGNR (No match)
178 - 188	1233.6239	1232.6166	1232.6625	-0.0459	0	AVPLIHQEGNR (Ions score 68)
202 - 210	1116.4906	1115.4833	1115.5321	-0.0488	0	YYDAIACLK (No match)
291 - 304	1478.8029	1477.7956	1477.8504	-0.0547	0	VLELDPALAPVVSR (No match)
291 - 304	1478.8029	1477.7956	1477.8504	-0.0547	0	VLELDPALAPVVSR (Ions score 73)

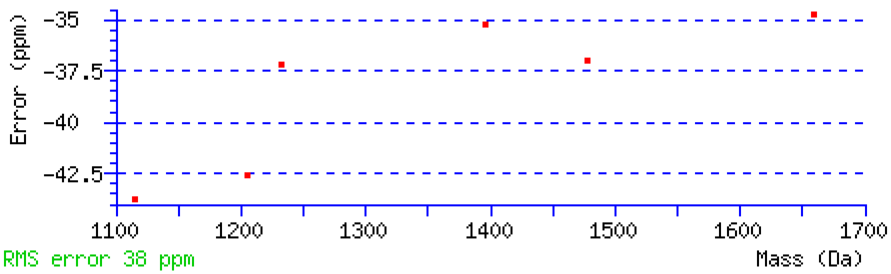

Mascot: http://www.matrixscience.com/

Spot 957

Protein View

Match to: **gi|49456653** Score: **133** Expect: **9.6e-009**
GRAP2 [Homo sapiens]

Nominal mass (M_r): **37971**; Calculated pI value: **6.55**
NCBI BLAST search of gi|49456653 against nr
Unformatted sequence string for pasting into other applications

Taxonomy: Homo sapiens

Fixed modifications: Carbamidomethyl (C)
Variable modifications: Oxidation (M)
Cleavage by Trypsin: cuts C-term side of KR unless next residue is P
Sequence Coverage: **23%**

Matched peptides shown in **Bold Red**

```
  1 MEAVAKFDFT ASGEDELSFH TGDVLKILSN QEEWFKAELG SQEGYVPKNF
 51 IDIQFPKWFH EGLSRHQAEN LLMGKEVGFF IIRASQSSPG DFSISVRHED
101 DVQHFKVMRD NKGNYFLWTE KFPSLNKLVD YYRTNSISRQ KQIFLRDRTR
151 EDQGHRGNSL DRRSQGGPHL SGAVGEEIRP SMNRKLSDHP PTLPLRQHQH
201 QPQPPQYAPA PQQLQQPPQQ RYLQHHHFHQ ERRGGSLDIN DGHCGTGLGS
251 EMNAALMHRR HTDPVQLQAA GRVRWARALY DFEALEDDEL GFHSGEVVEV
301 LDSSNPSWWT GRLHNKLGLF PANYVAPMTR
```

Residue Number Increasing Mass Decreasing Mass

Start - End	Observed	Mr(expt)	Mr(calc)	Delta	Miss	Sequence
58 - 65	1031.5095	1030.5022	1030.4984	0.0038	0	WFHEGLSR (No match)
76 - 83	980.5598	979.5525	979.5490	0.0035	0	EVGFFIIR (Ions score 28)
76 - 83	980.5598	979.5525	979.5490	0.0035	0	EVGFFIIR (No match)
84 - 97	1437.6953	1436.6880	1436.6895	-0.0015	0	ASQSSPGDFSISVR (No match)
164 - 184	2195.0513	2194.0440	2194.0548	-0.0108	0	SQGGPHLSGAVGEEIRPSMNR Oxidation (M) (No match)
164 - 184	2195.0513	2194.0440	2194.0548	-0.0108	0	SQGGPHLSGAVGEEIRPSMNR Oxidation (M) (No match)
222 - 232	1531.7240	1530.7167	1530.7228	-0.0061	0	YLQHHHFHQER (Ions score 35)
222 - 232	1531.7240	1530.7167	1530.7228	-0.0061	0	YLQHHHFHQER (No match)
317 - 330	1565.8052	1564.7979	1564.8071	-0.0092	0	LGLFPANYVAPMTR Oxidation (M) (No match)
317 - 330	1565.8052	1564.7979	1564.8071	-0.0092	0	LGLFPANYVAPMTR Oxidation (M) (Ions score 2)

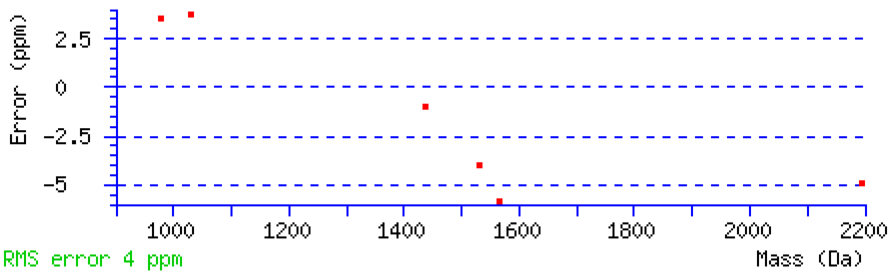

Mascot: http://www.matrixscience.com/

Spot 958

Protein View

Match to: **gi|56410847** Score: **213** Expect: **9.6e-017**
GDP dissociation inhibitor 2 [Homo sapiens]

Nominal mass (M_r): **46046**; Calculated pI value: **5.91**
NCBI BLAST search of gi|56410847 against nr
Unformatted sequence string for pasting into other applications

Taxonomy: Homo sapiens

Fixed modifications: Carbamidomethyl (C)
Variable modifications: Oxidation (M)
Cleavage by Trypsin: cuts C-term side of KR unless next residue is P
Sequence Coverage: **14%**

Matched peptides shown in **Bold Red**

```
  1 MNEEYDVIVL GTGLTECILS GIMSVNGKKV LHMDRNPYYG GESASITPLE
 51 DLYKRFKIPG SPPESMGRGR DWNVDLIPKF LMANGLMGLF EKRRFRKFLV
101 YVANFDEKDP RTFEGIDPKK TTMRDVYKKF DLGQDVIDFT GHALALYRTD
151 DYLDQPCYET INRIKLYSES LARYGKSPYL YPLYGLGELP QGFARLSAIY
201 GGTYMLNKPI EEIIVQNGKV IGVKSEGEIA RCKQLICDPS YVKDRVEKVG
251 QVIRVICILS HPIKNTNDAN SCQIIIPQNQ VNRKSDIYVC MISFAHNVAA
301 QGKYIAIVST TVETKEPEKE IRPALELLEP IEQKFVSISD LLVPKDLGTE
351 SQIFISRTYD ATTHFETTCD DIKNIYKRMT GSEFDFEEMK RKKNDIYGED
401
```

Residue Number Increasing Mass Decreasing Mass

Start - End	Observed	Mr(expt)	Mr(calc)	Delta	Miss	Sequence
56 - 68	1402.7175	1401.7102	1401.7074	0.0028	1	FKIPGSPPESMGR (No match)
56 - 68	1402.7175	1401.7102	1401.7074	0.0028	1	FKIPGSPPESMGR (Ions score 35)
130 - 148	2151.0796	2150.0723	2150.0796	-0.0073	0	FDLGQDVIDFTGHALALYR (No match)
130 - 148	2151.0796	2150.0723	2150.0796	-0.0073	0	FDLGQDVIDFTGHALALYR (Ions score 19)
149 - 163	1902.8149	1901.8076	1901.8101	-0.0025	0	TDDYLDQPCYETINR (No match)
149 - 163	1902.8149	1901.8076	1901.8101	-0.0025	0	TDDYLDQPCYETINR (Ions score 50)
346 - 357	1365.7024	1364.6951	1364.6935	0.0016	0	DLGTESQIFISR (Ions score 61)
346 - 357	1365.7024	1364.6951	1364.6935	0.0016	0	DLGTESQIFISR (No match)

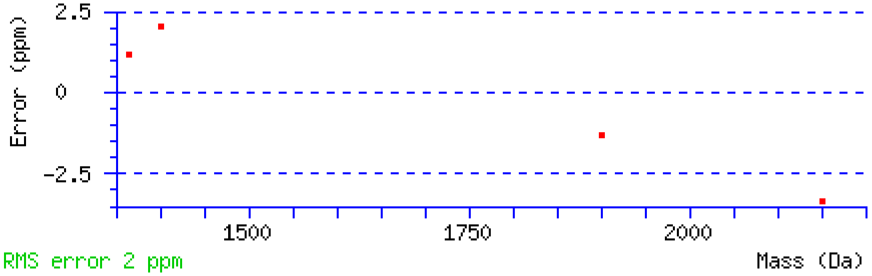

Mascot: http://www.matrixscience.com/

Spot 959

Protein View

Match to: **gi|10880979** Score: **608** Expect: **3e-056**
lymphocyte-specific protein 1 isoform 1 [Homo sapiens]

Nominal mass (M_r): **37397;** Calculated pI value: **4.69**
NCBI BLAST search of gi|10880979 against nr
Unformatted sequence string for pasting into other applications

Taxonomy: Homo sapiens
Links to retrieve other entries containing this sequence from NCBI Entrez:
gi|462553 from Homo sapiens
gi|307139 from Homo sapiens
gi|49456417 from Homo sapiens
gi|49456467 from Homo sapiens

Fixed modifications: Carbamidomethyl (C)
Variable modifications: Oxidation (M)
Cleavage by Trypsin: cuts C-term side of KR unless next residue is P
Sequence Coverage: **54%**

Matched peptides shown in **Bold Red**

```
  1 MAEASSDPGA EEREELLGPT AQWSVEDEEE AVHEQCQHER DRQLQAQDEE
 51 GGGHVPERPK QEMLLSLKPS EAPELDEDEG FGDWSQRPEQ RQQHEGAQGA
101 LDSGEPPQCR SPEGEQEDRP GLHAYEKEDS DEVHLEELSL SKEGPGPEDT
151 VQDNLGAAGA EEEQEEHQKC QQPRTPSPLV LEGTIEQSSP PLSPTTKLID
201 RTESLNRSIE KSNSVKKSQP DLPISKIDQW LEQYTQAIET AGRTPKLARQ
251 ASIELPSMAV ASTKSRWETG EVQAQSAAKT PSCKDIVAGD MSKKSLWEQK
301 GGSKTSSTIK STPSGKRYKF VATGHGKYEK VLVEGGPAP
```

Residue Number Increasing Mass Decreasing Mass

Start - End	Observed	Mr(expt)	Mr(calc)	Delta	Miss	Sequence
14 - 40	3235.3523	3234.3450	3234.4159	-0.0709	0	EELLGPTAQWSVEDEEEAVHEQCQHER (No match)
41 - 60	2246.0439	2245.0366	2245.0835	-0.0469	1	DRQLQAQDEEGGGHVPERPK (No match)
43 - 60	1974.9117	1973.9044	1973.9554	-0.0510	0	QLQAQDEEGGGHVPERPK (No match)
43 - 60	1974.9117	1973.9044	1973.9554	-0.0510	0	QLQAQDEEGGGHVPERPK (Ions score 126)
92 - 110	2064.8650	2063.8577	2063.9079	-0.0501	0	QQHEGAQGALDSGEPPQCR (Ions score 60)

92 - 110	2064.8650	2063.8577	2063.9079	-0.0501	0	QQHEGAQGALDSGEPPQCR (No match)
111 - 127	1941.8446	1940.8373	1940.8864	-0.0490	0	SPEGEQEDRPGLHAYEK (No match)
111 - 127	1941.8446	1940.8373	1940.8864	-0.0490	0	SPEGEQEDRPGLHAYEK (Ions score 55)
128 - 142	1729.7725	1728.7652	1728.8053	-0.0401	0	EDSDEVHLEELSLSK (No match)
143 - 169	2864.2280	2863.2207	2863.2379	-0.0172	0	EGPGPEDTVQDNLGAAGAEEEQEEHQK (No match)
198 - 207	1216.6356	1215.6283	1215.6571	-0.0288	1	LIDRTESLNR (No match)
227 - 243	2021.9392	2020.9319	2020.9854	-0.0534	0	IDQWLEQYTQAIETAGR (Ions score 126)
227 - 243	2021.9392	2020.9319	2020.9854	-0.0534	0	IDQWLEQYTQAIETAGR (No match)
265 - 279	1647.7694	1646.7621	1646.8012	-0.0391	1	SRWETGEVQAQSAAK (No match)
265 - 279	1647.7694	1646.7621	1646.8012	-0.0391	1	SRWETGEVQAQSAAK (Ions score 85)
267 - 279	1404.6426	1403.6353	1403.6680	-0.0327	0	WETGEVQAQSAAK (No match)
294 - 300	918.4846	917.4773	917.4970	-0.0197	1	KSLWEQK (No match)
320 - 327	816.4225	815.4152	815.4289	-0.0137	0	FVATGHGK (No match)
320 - 330	1236.6075	1235.6002	1235.6298	-0.0296	1	FVATGHGKYEK (No match)

Mascot: http://www.matrixscience.com/

Spot 960

Protein View

Match to: **gi|10880979** Score: **602** Expect: **1.2e-055**
lymphocyte-specific protein 1 isoform 1 [Homo sapiens]

Nominal mass (M_r): **37397**; Calculated pI value: **4.69**
NCBI BLAST search of gi|10880979 against nr
Unformatted sequence string for pasting into other applications

Taxonomy: Homo sapiens
Links to retrieve other entries containing this sequence from NCBI Entrez:
gi|462553 from Homo sapiens
gi|307139 from Homo sapiens
gi|49456417 from Homo sapiens
gi|49456467 from Homo sapiens

Fixed modifications: Carbamidomethyl (C)
Variable modifications: Oxidation (M)
Cleavage by Trypsin: cuts C-term side of KR unless next residue is P
Sequence Coverage: **51%**

Matched peptides shown in **Bold Red**

```
  1 MAEASSDPGA EEREELLGPT AQWSVEDEEE AVHEQCQHER DRQLQAQDEE
 51 GGGHVPERPK QEMLLSLKPS EAPELDEDEG FGDWSQRPEQ RQQHEGAQGA
101 LDSGEPPQCR SPEGEQEDRP GLHAYEKEDS DEVHLEELSL SKEGPGPEDT
151 VQDNLGAAGA EEEQEEHQKC QQPRTPSPLV LEGTIEQSSP PLSPTTKLID
201 RTESLNRSIE KSNSVKKSQP DLPISKIDQW LEQYTQAIET AGRTPKLARQ
251 ASIELPSMAV ASTKSRWETG EVQAQSAAKT PSCKDIVAGD MSKKSLWEQK
301 GGSKTSSTIK STPSGKRYKF VATGHGKYEK VLVEGGPAP
```

Residue Number Increasing Mass Decreasing Mass

Start - End	Observed	Mr(expt)	Mr(calc)	Delta	Miss	Sequence
14 - 40	3235.3123	3234.3050	3234.4159	-0.1109	0	EELLGPTAQWSVEDEEEAVHEQCQHER (No match)
41 - 60	2246.0193	2245.0120	2245.0835	-0.0715	1	DRQLQAQDEEGGGHVPERPK (No match)
43 - 60	1974.8845	1973.8772	1973.9554	-0.0782	0	QLQAQDEEGGGHVPERPK (No match)
43 - 60	1974.8845	1973.8772	1973.9554	-0.0782	0	QLQAQDEEGGGHVPERPK (Ions score 118)
92 - 110	2064.8389	2063.8316	2063.9079	-0.0762	0	QQHEGAQGALDSGEPPQCR (No match)

92 - 110	2064.8389	2063.8316	2063.9079	-0.0762	0	QQHEGAQGALDSGEPPQCR (Ions score 68)
111 - 127	1941.8167	1940.8094	1940.8864	-0.0769	0	SPEGEQEDRPGLHAYEK (Ions score 60)
111 - 127	1941.8167	1940.8094	1940.8864	-0.0769	0	SPEGEQEDRPGLHAYEK (No match)
128 - 142	1729.7476	1728.7403	1728.8053	-0.0650	0	EDSDEVHLEELSLSK (No match)
143 - 169	2864.1824	2863.1751	2863.2379	-0.0628	0	EGPGPEDTVQDNLGAAGAEEEQEEHQK (No match)
198 - 207	1216.6196	1215.6123	1215.6571	-0.0448	1	LIDRTESLNR (No match)
227 - 243	2021.9143	2020.9070	2020.9854	-0.0783	0	IDQWLEQYTQAIETAGR (Ions score 134)
227 - 243	2021.9143	2020.9070	2020.9854	-0.0783	0	IDQWLEQYTQAIETAGR (No match)
265 - 279	1647.7448	1646.7375	1646.8012	-0.0637	1	SRWETGEVQAQSAAK (Ions score 81)
265 - 279	1647.7448	1646.7375	1646.8012	-0.0637	1	SRWETGEVQAQSAAK (No match)
267 - 279	1404.6176	1403.6103	1403.6680	-0.0577	0	WETGEVQAQSAAK (No match)
294 - 300	918.4670	917.4597	917.4970	-0.0373	1	KSLWEQK (No match)

Mascot: http://www.matrixscience.com/

Spot 961

Protein View

Match to: **gi|12804709** Score: **442** Expect: **1.2e-039**
Lymphocyte-specific protein 1 [Homo sapiens]

Nominal mass (M_r): **37427**; Calculated pI value: **4.69**
NCBI BLAST search of gi|12804709 against nr
Unformatted sequence string for pasting into other applications

Taxonomy: Homo sapiens
Links to retrieve other entries containing this sequence from NCBI Entrez:
gi|62897025 from Homo sapiens

Fixed modifications: Carbamidomethyl (C)
Variable modifications: Oxidation (M)
Cleavage by Trypsin: cuts C-term side of KR unless next residue is P
Sequence Coverage: **38%**

Matched peptides shown in **Bold Red**

```
  1 MAEASSDPGA EEREELLGPT AQWSVEDEEE AVHEQCQHER DRQLQAQDEE
 51 GGGHVPERPK QEMLLSLKPS EAPELDEDEG FGDWSQRPEQ RQQHEGAQGT
101 LDSGEPPQCR SPEGEQEDRP GLHAYEKEDS DEVHLEELSL SKEGPGPEDT
151 VQDNLGAAGA EEEQEEHQKC QQPRTPSPLV LEGTIEQSSP PLSPTTKLID
201 RTESLNRSIE KSNSVKKSQP DLPISKIDQW LEQYTQAIET AGRTPKLARQ
251 ASIELPSMAV ASTKSRWETG EVQAQSAAKT PSCKDIVAGD MSKKSLWEQK
301 GGSKTSSTIK STPSGKRYKF VATGHGKYEK VLVEGGPAP
```

Residue Number Increasing Mass Decreasing Mass

Start - End	Observed	Mr(expt)	Mr(calc)	Delta	Miss	Sequence
43 - 60	1974.9186	1973.9113	1973.9554	-0.0441	0	QLQAQDEEGGGHVPERPK (Ions score 133)
43 - 60	1974.9186	1973.9113	1973.9554	-0.0441	0	QLQAQDEEGGGHVPERPK (No match)
92 - 110	2094.8816	2093.8743	2093.9184	-0.0441	0	QQHEGAQGTLDSGEPPQCR (No match)
111 - 127	1941.8514	1940.8441	1940.8864	-0.0422	0	SPEGEQEDRPGLHAYEK (No match)
111 - 127	1941.8514	1940.8441	1940.8864	-0.0422	0	SPEGEQEDRPGLHAYEK (Ions score 56)
143 - 169	2864.1926	2863.1853	2863.2379	-0.0526	0	EGPGPEDTVQDNLGAAGAEEEQEEHQK (No match)
198 - 207	1216.6311	1215.6238	1215.6571	-0.0333	1	LIDRTESLNR (No match)
227 - 243	2021.9452	2020.9379	2020.9854	-0.0474	0	IDQWLEQYTQAIETAGR (No match)

227 - 243	2021.9452	2020.9379	2020.9854	-0.0474	0	IDQWLEQYTQAIETAGR	(Ions score 97)
265 - 279	1647.7742	1646.7669	1646.8012	-0.0343	1	SRWETGEVQAQSAAK	(No match)
265 - 279	1647.7742	1646.7669	1646.8012	-0.0343	1	SRWETGEVQAQSAAK	(Ions score 60)
267 - 279	1404.6409	1403.6336	1403.6680	-0.0344	0	WETGEVQAQSAAK	(No match)
294 - 300	918.4792	917.4719	917.4970	-0.0251	1	KSLWEQK	(No match)

Mascot: http://www.matrixscience.com/

Spot 964

Protein View

Match to: **gi|6857820** Score: **361** Expect: **1.5e-031**
phosphatidylinositol-5-phosphate 4-kinase, type II, alpha [Homo sapiens]

Nominal mass (M_r): **46424**; Calculated pI value: **6.50**
NCBI BLAST search of gi|6857820 against nr
Unformatted sequence string for pasting into other applications

Taxonomy: Homo sapiens
Links to retrieve other entries containing this sequence from NCBI Entrez:
gi|73948884 from Canis lupus familiaris
gi|18266879 from Homo sapiens
gi|6760472 from Homo sapiens
gi|17390058 from Homo sapiens
gi|55664519 from Homo sapiens
gi|55665197 from Homo sapiens
gi|57160667 from Homo sapiens
gi|119606546 from Homo sapiens
gi|119606547 from Homo sapiens

Fixed modifications: Carbamidomethyl (C)
Variable modifications: Oxidation (M)
Cleavage by Trypsin: cuts C-term side of KR unless next residue is P
Sequence Coverage: **17%**

Matched peptides shown in **Bold Red**

```
  1 MATPGNLGSS VLASKTKTKK KHFVAQKVKL FRASDPLLSV LMWGVNHSIN
 51 ELSHVQIPVM LMPDDFKAYS KIKVDNHLFN KENMPSHFKF KEYCPMVFRN
101 LRERFGIDDQ DFQNSLTRSA PLPNDSQARS GARFHTSYDK RYIIKTITSE
151 DVAEMHNILK KYHQYIVECH GITLLPQFLG MYRLNVDGVE IYVIVTRNVF
201 SHRLSVYRKY DLKGSTVARE ASDKEKAKEL PTLKDNDFIN EGQKIYIDDN
251 NKKVFLEKLK KDVEFLAQLK LMDYSLLVGI HDVERAEQEE VECEENDGEE
301 EGESDGTHPV GTPPDSPGNT LNSSPPLAPG EFDPNIDVYG IKCHENSPRK
351 EVYFMAIIDI LTHYDAKKKA AHAAKTVKHG AGAEISTVNP EQYSKRFLDF
401 IGHILT
```

Residue Number Increasing Mass Decreasing Mass

Start - End	Observed	Mr(expt)	Mr(calc)	Delta	Miss	Sequence

90 - 99	1376.6477	1375.6404	1375.6416	-0.0012	1	FKEYCPMVFR (No match)
90 - 99	1376.6477	1375.6404	1375.6416	-0.0012	1	FKEYCPMVFR (Ions score 31)
92 - 99	1101.4742	1100.4669	1100.4783	-0.0114	0	EYCPMVFR (No match)
105 - 118	1655.7573	1654.7500	1654.7587	-0.0086	0	FGIDDQDFQNSLTR (No match)
105 - 118	1655.7573	1654.7500	1654.7587	-0.0086	0	FGIDDQDFQNSLTR (Ions score 91)
134 - 141	1053.5093	1052.5020	1052.5039	-0.0019	1	FHTSYDKR (No match)
184 - 197	1589.8806	1588.8733	1588.8824	-0.0090	0	LNVDGVEIYVIVTR (Ions score 82)
184 - 197	1589.8806	1588.8733	1588.8824	-0.0090	0	LNVDGVEIYVIVTR (No match)
271 - 285	1759.8997	1758.8924	1758.8974	-0.0050	0	LMDYSLLVGIHDVER (No match)
271 - 285	1759.8997	1758.8924	1758.8974	-0.0050	0	LMDYSLLVGIHDVER (Ions score 73)
271 - 285	1775.9023	1774.8950	1774.8923	0.0027	0	LMDYSLLVGIHDVER Oxidation (M) (No match)
397 - 406	1175.6345	1174.6272	1174.6386	-0.0114	0	FLDFIGHILT (No match)

Mascot: http://www.matrixscience.com/

Spot 966

Protein View

Match to: **gi|5453595** Score: **116** Expect: **4.8e-007**
adenylyl cyclase-associated protein [Homo sapiens]

Nominal mass (M_r): **51926**; Calculated pI value: **8.07**
NCBI BLAST search of gi|5453595 against nr
Unformatted sequence string for pasting into other applications

Taxonomy: Homo sapiens
Links to retrieve other entries containing this sequence from NCBI Entrez:
gi|157649073 (no taxonomy information for this entry)
gi|114555781 from Pan troglodytes
gi|114555783 from Pan troglodytes
gi|114555785 from Pan troglodytes
gi|178084 from Homo sapiens
gi|179920 from Homo sapiens
gi|15530330 from Homo sapiens
gi|30583143 from Homo sapiens
gi|48146935 from Homo sapiens
gi|61362546 from synthetic construct
gi|63102451 from Homo sapiens
gi|119627644 from Homo sapiens
gi|119627646 from Homo sapiens
gi|119627648 from Homo sapiens
gi|119627649 from Homo sapiens
gi|119627650 from Homo sapiens
gi|123994329 from synthetic construct

Fixed modifications: Carbamidomethyl (C)
Variable modifications: Oxidation (M)
Cleavage by Trypsin: cuts C-term side of KR unless next residue is P
Sequence Coverage: **13%**

Matched peptides shown in **Bold Red**

1 MADMQNLVER LERAVGRLEA VSHTSDMHRG YADSPSKAGA APYVQAFDSL
51 LAGPVAEYLK ISKEIGGDVQ KHAEMVHTGL KLERALLVTA SQCQQPAENK
101 LSDLLAPISE QIKEVITFRE KNRGSKLFNH LSAVSESIQA LGWVAMAPKP
151 GPYVK**EMNDA AMFYTNR**VLK EYKDVDKKHV DWVKAYLSIW TELQAYIKEF
201 HTTGLAWSKT GPVAKELSGL PSGPSAGSGP PPPPPGPPPP PVSTSSGSDE
251 SASR**SALFAQ INQGESITHA LK**HVSDDMKT HKNPALKAQS GPVR**SGPKPF**
301 **SAPKPQTSPS PK**RATKKEPA VLELEGKKWR **VENQENVSNL VIEDTELK**QV

351 AYIYKCVNTT LQIKGKINSI TVDNCKKLGL VFDDVVGIVE IINSKDVKVQ
401 VMGKVPTISI NKTDGCHAYL SKNSLDCEIV SAKSSEMNVL IPTEGGDFNE
451 FPVPEQFKTL WNGQKLVTTV TEIAG

Residue Number Increasing Mass Decreasing Mass

Start - End	Observed	Mr(expt)	Mr(calc)	Delta	Miss	Sequence
156 - 167	1462.6066	1461.5993	1461.6017	-0.0023	0	EMNDAAMFYTNR (Ions score 6)
156 - 167	1462.6066	1461.5993	1461.6017	-0.0023	0	EMNDAAMFYTNR (No match)
156 - 167	1478.6017	1477.5944	1477.5966	-0.0021	0	EMNDAAMFYTNR Oxidation (M) (Ions score 4)
156 - 167	1478.6017	1477.5944	1477.5966	-0.0021	0	EMNDAAMFYTNR Oxidation (M) (No match)
156 - 167	1494.5909	1493.5836	1493.5915	-0.0078	0	EMNDAAMFYTNR 2 Oxidation (M) (No match)
255 - 272	1928.0138	1927.0065	1927.0163	-0.0097	0	SALFAQINQGESITHALK (Ions score 41)
255 - 272	1928.0138	1927.0065	1927.0163	-0.0097	0	SALFAQINQGESITHALK (No match)
295 - 312	1837.9708	1836.9635	1836.9733	-0.0098	0	SGPKPFSAPKPQTSPSPK (No match)
295 - 312	1837.9708	1836.9635	1836.9733	-0.0098	0	SGPKPFSAPKPQTSPSPK (Ions score 36)
331 - 348	2073.0276	2072.0203	2072.0273	-0.0069	0	VENQENVSNLVIEDTELK (No match)

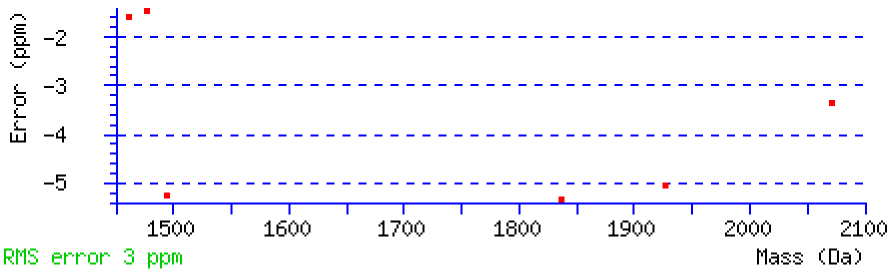

Mascot: http://www.matrixscience.com/

Spot 969

Protein View

Match to: **gi|154146191** Score: **401** Expect: **1.5e-035**
heat shock protein 90kDa alpha (cytosolic), class A member 1 isoform 2 [Homo sapiens]

Nominal mass (M_r): **85006**; Calculated pI value: **4.94**
NCBI BLAST search of gi|154146191 against nr
Unformatted sequence string for pasting into other applications

Taxonomy: Homo sapiens
Links to retrieve other entries containing this sequence from NCBI Entrez:
gi|92090606 from Homo sapiens
gi|703087 from Homo sapiens
gi|61656605 from Homo sapiens
gi|111306539 from Homo sapiens
gi|119602171 from Homo sapiens
gi|119602172 from Homo sapiens
gi|158255666 (no taxonomy information for this entry)
gi|158256646 (no taxonomy information for this entry)

Fixed modifications: Carbamidomethyl (C)
Variable modifications: Oxidation (M)
Cleavage by Trypsin: cuts C-term side of KR unless next residue is P
Sequence Coverage: **37%**

Matched peptides shown in **Bold Red**

```
  1 MPEETQTQDQ PMEEEEVETF AFQAEIAQLM SLIINTFYSN KEIFLRELIS
 51 NSSDALDKIR YESLTDPSKL DSGKELHINL IPNKQDRTLT IVDTGIGMTK
101 ADLINNLGTI AKSGTKAFME ALQAGADISM IGQFGVGFYS AYLVAEKVTV
151 ITKHNDDEQY AWESSAGGSF TVRTDTGEPM GRGTKVILHL KEDQTEYLEE
201 RRIKEIVKKH SQFIGYPITL FVEKERDKEV SDDEAEEKED KEEEKEKEEK
251 ESEDKPEIED VGSDEEEEKK DGDKKKKKKI KEKYIDQEEL NKTKPIWTRN
301 PDDITNEEYG EFYKSLTNDW EDHLAVKHFS VEGQLEFRAL LFVPRRAPFD
351 LFENRKKKNN IKLYVRRVFI MDNCEELIPE YLNFIRGVVD SEDLPLNISR
401 EMLQQSKILK VIRKNLVKKC LELFTELAED KENYKKFYEQ FSKNIKLGIH
451 EDSQNRKKLS ELLRYYTSAS GDEMVSLKDY CTRMKENQKH IYYITGETKD
501 QVANSAFVER LRKHGLEVIY MIEPIDEYCV QQLKEFEGKT LVSVTKEGLE
551 LPEDEEEKKK QEEKKTKFEN LCKIMKDILE KKVEKVVVSN RLVTSPCCIV
601 TSTYGWTANM ERIMKAQALR DNSTMGYMAA KKHLEINPDH SIIETLRQKA
651 EADKNDKSVK DLVILLYETA LLSSGFSLED PQTHANRIYR MIKLGLGIDE
701 DDPTADDTSA AVTEEMPPLE GDDDTSRMEE VD
```

Residue Number Increasing Mass Decreasing Mass

Start - End	Observed	Mr(expt)	Mr(calc)	Delta	Miss	Sequence
47 - 60	1560.8411	1559.8338	1559.8154	0.0184	1	ELISNSSDALDKIR (No match)
75 - 87	1589.8939	1588.8866	1588.8685	0.0181	1	ELHINLIPNKQDR (No match)
154 - 173	2255.9707	2254.9634	2254.9515	0.0119	0	HNDDEQYAWESSAGGSFTVR (No match)
174 - 185	1249.6345	1248.6272	1248.5768	0.0504	1	TDTGEPMGRGTK (No match)
186 - 201	2015.0625	2014.0552	2014.0370	0.0182	1	VILHLKEDQTEYLEER (No match)
192 - 201	1311.5863	1310.5790	1310.5626	0.0164	0	EDQTEYLEER (No match)
210 - 224	1778.9684	1777.9611	1777.9402	0.0209	0	HSQFIGYPITLFVEK (No match)
284 - 292	1151.5719	1150.5646	1150.5506	0.0141	0	YIDQEELNK (No match)
293 - 299	901.5389	900.5316	900.5181	0.0135	0	TKPIWTR (No match)
300 - 314	1833.8044	1832.7971	1832.7740	0.0231	0	NPDDITNEEYGEFYK (No match)
315 - 327	1527.7644	1526.7571	1526.7365	0.0206	0	SLTNDWEDHLAVK (No match)
328 - 338	1348.6812	1347.6739	1347.6571	0.0169	0	HFSVEGQLEFR (Ions score 46)
328 - 338	1348.6812	1347.6739	1347.6571	0.0169	0	HFSVEGQLEFR (No match)
339 - 345	815.5242	814.5169	814.5065	0.0105	0	ALLFVPR (No match)
346 - 355	1264.6589	1263.6516	1263.6360	0.0157	1	RAPFDLFENR (No match)
346 - 355	1264.6589	1263.6516	1263.6360	0.0157	1	RAPFDLFENR (Ions score 33)
347 - 355	1108.5585	1107.5512	1107.5349	0.0164	0	APFDLFENR (No match)
347 - 356	1236.6394	1235.6321	1235.6298	0.0023	1	APFDLFENRK (No match)
387 - 400	1513.8058	1512.7985	1512.7783	0.0202	0	GVVDSEDLPLNISR (No match)
387 - 400	1513.8058	1512.7985	1512.7783	0.0202	0	GVVDSEDLPLNISR (Ions score 71)
437 - 443	948.4564	947.4491	947.4388	0.0103	0	FYEQFSK (No match)
447 - 456	1168.5896	1167.5823	1167.5632	0.0191	0	LGIHEDSQNR (No match)
465 - 483	2245.9956	2244.9883	2244.9667	0.0216	1	YYTSASGDEMVSLKDYCTR (No match)
490 - 499	1224.6393	1223.6320	1223.6186	0.0134	0	HIYYITGETK (No match)
500 - 510	1235.6194	1234.6121	1234.5941	0.0180	0	DQVANSAFVER (Ions score 25)
500 - 510	1235.6194	1234.6121	1234.5941	0.0180	0	DQVANSAFVER (No match)
592 - 612	2462.1362	2461.1289	2461.1076	0.0214	0	LVTSPCCIVTSTYGWTANMER Oxidation (M) (No match)
632 - 647	1915.0581	1914.0508	1914.0323	0.0186	1	KHLEINPDHSIIETLR (No match)
633 - 647	1786.9636	1785.9563	1785.9373	0.0190	0	HLEINPDHSIIETLR (Ions score 51)
633 - 647	1786.9636	1785.9563	1785.9373	0.0190	0	HLEINPDHSIIETLR (No match)

Spot 971

Protein View

Match to: **gi|41281905** Score: **206** Expect: **4.8e-016**
UNC-112 related protein 2 long form [Homo sapiens]

Nominal mass (M_r): **76475;** Calculated pI value: **6.52**
NCBI BLAST search of gi|41281905 against nr
Unformatted sequence string for pasting into other applications

Taxonomy: Homo sapiens
Links to retrieve other entries containing this sequence from NCBI Entrez:
gi|41018464 from Homo sapiens
gi|30384985 from Homo sapiens

Fixed modifications: Carbamidomethyl (C)
Variable modifications: Oxidation (M)
Cleavage by Trypsin: cuts C-term side of KR unless next residue is P
Sequence Coverage: **28%**

Matched peptides shown in **Bold Red**

```
  1 MAGMKTASGD YIDSSWELRV FVGEEDPEAE SVTLRVTGES HIGGVLLKIV
 51 EQINRKQDWS DHAIWWEQKR QWLLQTHWTL DKYGILADAR LFFGPQHRPV
101 ILRLPNRRAL RLRASFSQPL FQAVAAICRL LSIRHPEELS LLRAPEKKEK
151 KKKEKEPEEE LYDLSKVVLA GGVAPALFRG MPAHFSDSAQ TEACYHMLSR
201 PQPPPDPLLL QRLPRPSSLS DKTQLHSRWL DSSRCLMQQG IKAGDALWLR
251 FKYYSFFDLD PKTDPVRLTQ LYEQARWDLL LEEIDCTEEE MMVFAALQYH
301 INKLSQSGEV GEPAGTDPGL DDLDVALSNL EVKLEGSAPT DVLDSLTTIP
351 ELKDHLRIFR IPRRPRKLTL KGYRQHWVVF KETTLSYYKS QDEAPGDPIQ
401 QLNLKGCEVV PDVNVSGQKF CIKLLVPSPE GMSEIYLRCQ DEQQYARWMA
451 GCRLASKGRT MADSSYTSEV QAILAFLSLQ RTGSGGPGNH PHGPDASAEG
501 LNPYGLVAPR FQRKFKAKQL TPRILEAHQN VAQLSLAEAQ LRFIQAWQSL
551 PDFGISYVMV RFKGSRKDEI LGIANNRLIR IDLAVGDVVK TWRFSNMRQW
601 NVNWDIRQVA IEFDEHINVA FSCVSASCRI VHEYIGGYIF LSTRERARGE
651 ELDEDLFLQL TGGHEAF
```

Residue Number Increasing Mass Decreasing Mass

Start - End	Observed	Mr(expt)	Mr(calc)	Delta	Miss	Sequence

20 - 35	1776.8667	1775.8594	1775.8577	0.0018	0	VFVGEEDPEAESVTLR (No match)
91 - 103	1579.9231	1578.9158	1578.9146	0.0012	0	LFFGPQHRPVILR (Ions score 14)
91 - 103	1579.9231	1578.9158	1578.9146	0.0012	0	LFFGPQHRPVILR (No match)
114 - 129	1765.9065	1764.8992	1764.8981	0.0012	0	ASFSQPLFQAVAAICR (No match)
135 - 143	1093.6085	1092.6012	1092.5927	0.0085	0	HPEELSLLR (No match)
167 - 179	1269.7717	1268.7644	1268.7604	0.0040	0	VVLAGGVAPALFR (No match)
167 - 179	1269.7717	1268.7644	1268.7604	0.0040	0	VVLAGGVAPALFR (Ions score 18)
268 - 276	1121.5997	1120.5924	1120.5876	0.0048	0	LTQLYEQAR (No match)
334 - 357	2620.3726	2619.3653	2619.3755	-0.0102	1	LEGSAPTDVLDSLTTIPELKDHLR (No match)
424 - 438	1703.9032	1702.8959	1702.8963	-0.0004	0	LLVPSPEGMSEIYLR (No match)
424 - 438	1719.9000	1718.8927	1718.8912	0.0015	0	LLVPSPEGMSEIYLR Oxidation (M) (No match)
439 - 447	1197.4971	1196.4898	1196.4880	0.0018	0	CQDEQQYAR (No match)
482 - 510	2782.3074	2781.3001	2781.3218	-0.0217	0	TGSGGPGNHPHGPDASAEGLNPYGLVAPR (No match)
524 - 542	2104.1392	2103.1319	2103.1436	-0.0116	0	ILEAHQNVAQLSLAEAQLR (No match)
524 - 542	2104.1392	2103.1319	2103.1436	-0.0116	0	ILEAHQNVAQLSLAEAQLR (Ions score 40)
630 - 644	1767.9323	1766.9250	1766.9355	-0.0104	0	IVHEYIGGYIFLSTR (Ions score 14)
630 - 644	1767.9323	1766.9250	1766.9355	-0.0104	0	IVHEYIGGYIFLSTR (No match)

Mascot: http://www.matrixscience.com/

Spot 973

Protein View

Match to: **gi|14530105** Score: **350** Expect: **1.9e-030**
OVARIAN/Breast septin gamma [Homo sapiens]

Nominal mass (M_r): **64894;** Calculated pI value: **7.21**
NCBI BLAST search of gi|14530105 against nr
Unformatted sequence string for pasting into other applications

Taxonomy: Homo sapiens
Links to retrieve other entries containing this sequence from NCBI Entrez:
gi|119609873 from Homo sapiens

Fixed modifications: Carbamidomethyl (C)
Variable modifications: Oxidation (M)
Cleavage by Trypsin: cuts C-term side of KR unless next residue is P
Sequence Coverage: **21%**

Matched peptides shown in **Bold Red**

```
  1 MSDPAVNAQL DGIISDFEAL KRSFEVEEVE TPNSTPPRRV QTPLLRATVA
 51 SSTQKFQDLG VKNSEPSARH VDSLSQRSPK ASLRRVELSG PKAAEPVSRR
101 TELSIDISSK QVENAGAIGP SRFGLKRAEV LGHKTPEPAP RRTEITIVKP
151 QESAHRRMEP PASKVPEVPT APATDAAPKR VEIQMPKPAE APTAPSPAQT
201 LENSEPAPVS QLQSRLEPKP QPPVAEATPR SQEATEAAPS CVGDMADTPR
251 DAGLKQAPAS RNEKAPVDFG YVGIDSILEQ MRRKAMKQGF EFNIMVVGQS
301 GLGKSTLINT LFKSKISRKS VQPTSEERIP KTIEIKSITH DIEEKGVRMK
351 LTVIDTPGFG DHINNENCWQ PIMKFINDQY EKYLQEEVNI NRKKRIPDTR
401 VHCCLYFIPA TGHSLRPLDI EFMKRLSKVV NIVPVIAKAD TLTLEERVHF
451 KQRITADLLS NGIDVYPQKE FDEDSEDRLV NEKFREMIPF AVVGSDHEYQ
501 VNGKRILGRK TKWGTIEVEN TTHCEFAYLR DLLIRTHMQN IKDITSSIHF
551 EAYRVKRLNE GSSAMANGVE EKEPEAPEM
```

Residue Number Increasing Mass Decreasing Mass

Start - End	Observed	Mr(expt)	Mr(calc)	Delta	Miss	Sequence	
23 - 38	1817.8854	1816.8781	1816.8479	0.0303	0	SFEVEEVETPNSTPPR	(Ions score 73)
23 - 38	1817.8854	1816.8781	1816.8479	0.0303	0	SFEVEEVETPNSTPPR	(No match)
23 - 39	1973.9944	1972.9871	1972.9490	0.0382	1	SFEVEEVETPNSTPPRR	(No match)

39 - 46	982.6191	981.6118	981.6083	0.0035	1	RVQTPLLR (No match)
111 - 122	1198.6404	1197.6331	1197.6101	0.0230	0	QVENAGAIGPSR (No match)
142 - 156	1764.9960	1763.9887	1763.9642	0.0246	1	RTEITIVKPQESAHR (Ions score 40)
143 - 157	1764.9960	1763.9887	1763.9642	0.0246	1	TEITIVKPQESAHRR (No match)
216 - 230	1629.9236	1628.9163	1628.8885	0.0278	0	LEPKPQPPVAEATPR (Ions score 60)
216 - 230	1629.9236	1628.9163	1628.8885	0.0278	0	LEPKPQPPVAEATPR (No match)
231 - 250	2092.9280	2091.9207	2091.8837	0.0370	0	SQEATEAAPSCVGDMADTPR (No match)
375 - 382	1056.5133	1055.5060	1055.4923	0.0137	0	FINDQYEK (No match)
383 - 392	1277.6752	1276.6679	1276.6411	0.0268	0	YLQEEVNINR (No match)
383 - 392	1277.6752	1276.6679	1276.6411	0.0268	0	YLQEEVNINR (No match)
383 - 393	1405.7676	1404.7603	1404.7361	0.0243	1	YLQEEVNINRK (No match)
439 - 447	1047.5492	1046.5419	1046.5244	0.0176	0	ADTLTLEER (No match)
543 - 554	1438.7241	1437.7168	1437.6888	0.0280	0	DITSSIHFEAYR (No match)
543 - 554	1438.7241	1437.7168	1437.6888	0.0280	0	DITSSIHFEAYR (Ions score 35)

Mascot: http://www.matrixscience.com/

Spot 974

Protein View

Match to: **gi|4505257** Score: **662** Expect: **1.2e-061**
moesin [Homo sapiens]

Nominal mass (M_r): **67892**; Calculated pI value: **6.08**
NCBI BLAST search of gi|4505257 against nr
Unformatted sequence string for pasting into other applications

Taxonomy: Homo sapiens
Links to retrieve other entries containing this sequence from NCBI Entrez:
gi|127234 from Homo sapiens
gi|188626 from Homo sapiens
gi|5419633 from Homo sapiens
gi|16878176 from Homo sapiens
gi|119625803 from Homo sapiens
gi|123984467 from synthetic construct
gi|123998433 from synthetic construct

Fixed modifications: Carbamidomethyl (C)
Variable modifications: Oxidation (M)
Cleavage by Trypsin: cuts C-term side of KR unless next residue is P
Sequence Coverage: **57%**

Matched peptides shown in **Bold Red**

```
  1 MPKTISVRVT TMDAELEFAI QPNTTGKQLF DQVVKTIGLR EVWFFGLQYQ
 51 DTKGFSTWLK LNKKVTAQDV RKESPLLFKF RAKFYPEDVS EELIQDITQR
101 LFFLQVKEGI LNDDIYCPPE TAVLLASYAV QSKYGDFNKE VHKSGYLAGD
151 KLLPQRVLEQ HKLNKDQWEE RIQVWHEEHR GMLREDAVLE YLKIAQDLEM
201 YGVNYFSIKN KKGSELWLGV DALGLNIYEQ NDRLTPKIGF PWSEIRNISF
251 NDKKFVIKPI DKKAPDFVFY APRLRINKRI LALCMGNHEL YMRRRKPDTI
301 EVQQMKAQAR EEKHQKQMER AMLENEKKKR EMAEKEKEKI EREKEELMER
351 LKQIEEQTKK AQQELEEQTR RALELEQERK RAQSEAEKLA KERQEAEEAK
401 EALLQASRDQ KKTQEQLALE MAELTARISQ LEMARQKKES EAVEWQQKAQ
451 MVQEDLEKTR AELKTAMSTP HVAEPAENEQ DEQDENGAEA SADLRADAMA
501 KDRSEEERTT EAEKNERVQK HLKALTSELA NARDESKKTA NDMIHAENMR
551 LGRDKYKTLR QIRQGNTKQR IDEFESM
```

Residue Number Increasing Mass Decreasing Mass

Start - End	Observed	Mr(expt)	Mr(calc)	Delta	Miss	Sequence
1 - 8	947.5171	946.5098	946.5269	-0.0171	1	MPKTISVR Oxidation (M) (No match)
9 - 27	2066.0388	2065.0315	2065.0037	0.0278	0	VTTMDAELEFAIQPNTTGK (No match)
9 - 27	2082.0320	2081.0247	2080.9986	0.0261	0	VTTMDAELEFAIQPNTTGK Oxidation (M) (No match)
28 - 35	976.5580	975.5507	975.5389	0.0118	0	QLFDQVVK (No match)
54 - 60	838.4611	837.4538	837.4384	0.0154	0	GFSTWLK (No match)
65 - 72	916.5455	915.5382	915.5137	0.0245	1	VTAQDVRK (No match)
82 - 100	2281.1699	2280.1626	2280.1273	0.0353	1	AKFYPEDVSEELIQDITQR (No match)
101 - 107	894.5582	893.5509	893.5374	0.0135	0	LFFLQVK (No match)
134 - 143	1236.5623	1235.5550	1235.5934	-0.0384	1	YGDFNKEVHK (No match)
144 - 156	1417.8037	1416.7964	1416.7724	0.0240	1	SGYLAGDKLLPQR (No match)
157 - 165	1108.5995	1107.5922	1107.6400	-0.0478	1	VLEQHKLNK (No match)
163 - 171	1217.6179	1216.6106	1216.5836	0.0270	1	LNKDQWEER (No match)
166 - 171	862.3893	861.3820	861.3617	0.0204	0	DQWEER (No match)
172 - 180	1233.6327	1232.6254	1232.6050	0.0204	0	IQVWHEEHR (No match)
172 - 180	1233.6327	1232.6254	1232.6050	0.0204	0	IQVWHEEHR (Ions score 37)
181 - 193	1536.8368	1535.8295	1535.8017	0.0278	1	GMLREDAVLEYLK (No match)
181 - 193	1552.8318	1551.8245	1551.7966	0.0279	1	GMLREDAVLEYLK Oxidation (M) (No match)
194 - 209	1890.9675	1889.9602	1889.9233	0.0370	0	IAQDLEMYGVNYFSIK (No match)
238 - 246	1104.6046	1103.5973	1103.5763	0.0210	0	IGFPWSEIR (Ions score 47)
238 - 246	1104.6046	1103.5973	1103.5763	0.0210	0	IGFPWSEIR (No match)
247 - 254	965.5287	964.5214	964.4977	0.0237	1	NISFNDKK (No match)
263 - 273	1310.7136	1309.7063	1309.6818	0.0245	1	KAPDFVFYAPR (No match)
263 - 273	1310.7136	1309.7063	1309.6818	0.0245	1	KAPDFVFYAPR (Ions score 57)
264 - 273	1182.6171	1181.6098	1181.5869	0.0229	0	APDFVFYAPR (No match)
264 - 273	1182.6171	1181.6098	1181.5869	0.0229	0	APDFVFYAPR (Ions score 82)
280 - 293	1720.8669	1719.8596	1719.8259	0.0338	0	ILALCMGNHELYMR (No match)
280 - 293	1736.8601	1735.8528	1735.8208	0.0321	0	ILALCMGNHELYMR Oxidation (M) (No match)
295 - 306	1472.8163	1471.8090	1471.7817	0.0274	1	RKPDTIEVQQMK (No match)
295 - 306	1488.8129	1487.8056	1487.7766	0.0291	1	RKPDTIEVQQMK Oxidation (M) (No match)
343 - 350	1079.5325	1078.5252	1078.4964	0.0288	1	EKEELMER Oxidation (M) (No match)
345 - 352	1063.5310	1062.5237	1062.5379	-0.0142	1	EELMERLK Oxidation (M) (No match)
361 - 371	1387.7152	1386.7079	1386.6851	0.0228	1	AQQELEEQTRR (No match)
372 - 379	987.5316	986.5243	986.5032	0.0211	0	ALELEQER (No match)
394 - 408	1672.8801	1671.8728	1671.8427	0.0301	1	QEAEEAKEALLQASR (No match)
412 - 427	1831.9858	1830.9785	1830.9509	0.0276	1	KTQEQLALEMAELTAR (No match)
412 - 427	1831.9858	1830.9785	1830.9509	0.0276	1	KTQEQLALEMAELTAR (Ions score 94)
412 - 427	1847.9905	1846.9832	1846.9458	0.0374	1	KTQEQLALEMAELTAR Oxidation (M) (No match)
413 - 427	1703.8906	1702.8833	1702.8559	0.0274	0	TQEQLALEMAELTAR (No match)
413 - 427	1719.8828	1718.8755	1718.8508	0.0247	0	TQEQLALEMAELTAR Oxidation (M) (No match)
428 - 435	963.5084	962.5011	962.4855	0.0157	0	ISQLEMAR Oxidation (M) (No match)
449 - 460	1447.7533	1446.7460	1446.7136	0.0324	1	AQMVQEDLEKTR (No match)
502 - 508	920.4249	919.4176	919.3995	0.0181	1	DRSEEER (No match)
509 - 517	1077.5392	1076.5319	1076.5098	0.0222	1	TTEAEKNER (No match)
524 - 533	1045.5891	1044.5818	1044.5563	0.0255	0	ALTSELANAR (No match)
538 - 550	1530.7429	1529.7356	1529.7079	0.0278	1	KTANDMIHAENMR (No match)
539 - 550	1402.6484	1401.6411	1401.6129	0.0282	0	TANDMIHAENMR (No match)
569 - 577	1154.5325	1153.5252	1153.5073	0.0179	1	QRIDEFESM (No match)

Spot 980

Protein View

Match to: **gi|119612724** Score: **109** Expect: **2.4e-006**
actin, alpha, cardiac muscle, isoform CRA_c [Homo sapiens]

Nominal mass (M_r): **30498**; Calculated pI value: **4.88**
NCBI BLAST search of gi|119612724 against nr
Unformatted sequence string for pasting into other applications

Taxonomy: Homo sapiens

Fixed modifications: Carbamidomethyl (C)
Variable modifications: Oxidation (M)
Cleavage by Trypsin: cuts C-term side of KR unless next residue is P
Sequence Coverage: **9%**

Matched peptides shown in **Bold Red**

```
  1 MCDDEETTAL VCDNGSGLVK AGFAGDDAPR AVFPSIVGRP RHQGVMVGMG
 51 QKDSYVGDEA QSKRGILTLK YPIEHGIITN WDDMEKIWHH TFYNELRVAP
101 EEHPTLLTEA PLNPKANREK MTQIMFETFN VPAMYVAIQA VLSLYASGRT
151 TGIVLDSGDG VTHNVPIYEG YALPHAIMRL DLAGRDLTDY LMKILTERGY
201 SFVTTAEREI VRDIKEKLCY VALDFENEMA TAASSSSLEK SYELPDGQVI
251 TIGNERFRCP ETLFQPSFIG EL
```

Residue Number Increasing Mass Decreasing Mass

Start - End	Observed	Mr(expt)	Mr(calc)	Delta	Miss	Sequence	
31 - 41	1198.7177	1197.7104	1197.6982	0.0123	0	AVFPSIVGRPR	(No match)
31 - 41	1198.7177	1197.7104	1197.6982	0.0123	0	AVFPSIVGRPR	(Ions score 20)
241 - 256	1790.9205	1789.9132	1789.8846	0.0287	0	SYELPDGQVITIGNER	(No match)
241 - 256	1790.9205	1789.9132	1789.8846	0.0287	0	SYELPDGQVITIGNER	(Ions score 63)

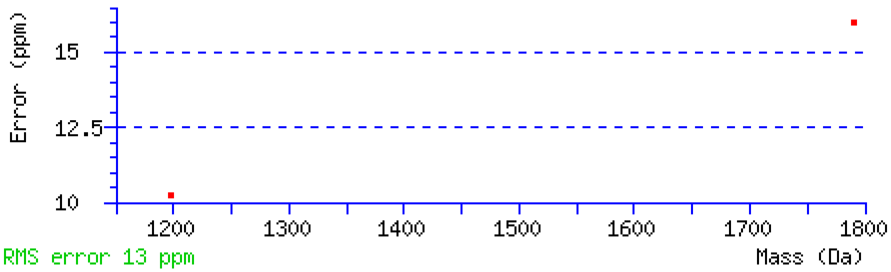

Mascot: http://www.matrixscience.com/

Spot 981

Protein View

Match to: **gi|13096112** Score: **99** Expect: **2.6e-005**
Chain A, Fidarestat Bound To Human Aldose Reductase

Nominal mass (M_r): **36099;** Calculated pI value: **6.56**
NCBI BLAST search of gi|13096112 against nr
Unformatted sequence string for pasting into other applications

Taxonomy: Homo sapiens
Links to retrieve other entries containing this sequence from NCBI Entrez:
gi|13096113 from Homo sapiens
gi|119390222 from Homo sapiens
gi|119390223 from Homo sapiens
gi|119390262 from Homo sapiens
gi|119390265 from Homo sapiens
gi|157829816 (no taxonomy information for this entry)
gi|157831933 (no taxonomy information for this entry)
gi|157834532 (no taxonomy information for this entry)
gi|157834533 (no taxonomy information for this entry)
gi|157834534 (no taxonomy information for this entry)

Fixed modifications: Carbamidomethyl (C)
Variable modifications: Oxidation (M)
Cleavage by Trypsin: cuts C-term side of KR unless next residue is P
Sequence Coverage: **12%**

Matched peptides shown in **Bold Red**

```
  1 ASRLLLNNGA KMPILGLGTW KSPPGQVTEA VKVAIDVGYR HIDCAHVYQN
 51 ENEVGVAIQE KLREQVVKRE ELFIVSKLWC TYHEKGLVKG ACQKTLSDLK
101 LDYLDLYLIH WPTGFKPGKE FFPLDESGNV VPSDTNILDT WAAMEELVDE
151 GLVKAIGISN FNHLQVEMIL NKPGLKYKPA VNQIECHPYL TQEKLIQYCQ
201 SKGIVVTAYS PLGSPDRPWA KPEDPSLLED PRIKAIAAKH NKTTAQVLIR
251 FPMQRNLVVI PKSVTPERIA ENFKVFDFEL SSQDMTTLLS YNRNWRVCAL
301 LSCTSHKDYP FHEEF
```

Residue Number Increasing Mass Decreasing Mass

Start - End	Observed	Mr(expt)	Mr(calc)	Delta	Miss	Sequence
33 - 40	892.4928	891.4855	891.4814	0.0042	0	VAIDVGYR (No match)
177 - 194	2218.0781	2217.0708	2217.0888	-0.0180	0	YKPAVNQIECHPYLTQEK (Ions score 55)
177 - 194	2218.0781	2217.0708	2217.0888	-0.0180	0	YKPAVNQIECHPYLTQEK (No match)
243 - 255	1576.8568	1575.8495	1575.8555	-0.0060	1	TTAQVLIRFPMQR Oxidation (M) (Ions score 10)
243 - 255	1576.8568	1575.8495	1575.8555	-0.0060	1	TTAQVLIRFPMQR Oxidation (M) (No match)

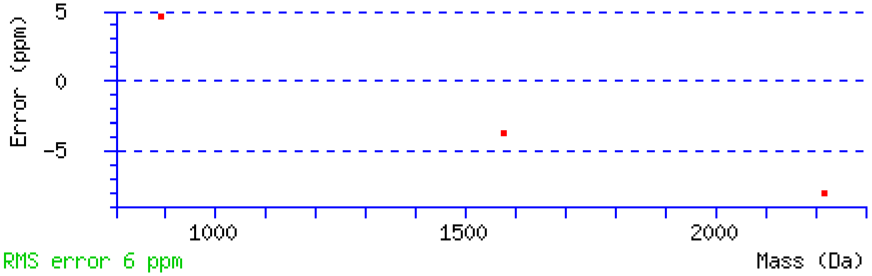

RMS error 6 ppm

Mascot: http://www.matrixscience.com/

Spot 984

Protein View

Match to: **gi|67464043** Score: **621** Expect: **1.5e-057**
Chain O, Crsytal Structure Of Human Liver Gapdh

Nominal mass (M_r): **36483;** Calculated pI value: **8.58**
NCBI BLAST search of gi|67464043 against nr
Unformatted sequence string for pasting into other applications

Taxonomy: Homo sapiens
Links to retrieve other entries containing this sequence from NCBI Entrez:
gi|67464044 from Homo sapiens
gi|67464045 from Homo sapiens
gi|67464046 from Homo sapiens

Fixed modifications: Carbamidomethyl (C)
Variable modifications: Oxidation (M)
Cleavage by Trypsin: cuts C-term side of KR unless next residue is P
Sequence Coverage: **53%**

Matched peptides shown in **Bold Red**

```
  1 GSHMGKVKVG VNGFGRIGRL VTRAAFNSGK VDIVAINDPF IDLNYMVYMF
 51 QYDSTHGKFH GTVKAENGKL VINGNPITIF QERDPSKIKW GDAGAEYVVE
101 STGVFTTMEK AGAHLQGGAK RVIISAPSAD APMFVMGVNH EKYDNSLKII
151 SNASCTTNCL APLAKVIHDN FGIVEGLMTT VHAITATQKT VDGPSGKLWR
201 DGRGALQNII PASTGAAKAV GKVIPELNGK LTGMAFRVPT ANVSVVDLTC
251 RLEKPAKYDD IKKVVKQASE GPLKGILGYT EHQVVSSDFN SDTHSSTFDA
301 GAGIALNDHF VKLISWYDNE FGYSNRVVDL MAHMASKE
```

Residue Number Increasing Mass Decreasing Mass

Start - End	Observed	Mr(expt)	Mr(calc)	Delta	Miss	Sequence
7 - 16	1032.5785	1031.5712	1031.5875	-0.0163	1	VKVGVNGFGR (No match)
9 - 16	805.4205	804.4132	804.4241	-0.0109	0	VGVNGFGR (No match)
70 - 83	1613.8604	1612.8531	1612.8936	-0.0405	0	LVINGNPITIFQER (No match)
70 - 83	1613.8604	1612.8531	1612.8936	-0.0405	0	LVINGNPITIFQER (Ions score 89)
70 - 87	2041.0531	2040.0458	2040.1003	-0.0545	1	LVINGNPITIFQERDPSK (No match)
70 - 87	2041.0531	2040.0458	2040.1003	-0.0545	1	LVINGNPITIFQERDPSK (Ions score 99)

90 - 110	2276.9973	2275.9900	2276.0306	-0.0406	0	**WGDAGAEYVVESTGVFTTMEK** (No match)
90 - 110	2293.0020	2291.9947	2292.0255	-0.0308	0	**WGDAGAEYVVESTGVFTTMEK** Oxidation (M) (No match)
111 - 120	909.4672	908.4599	908.4827	-0.0228	0	**AGAHLQGGAK** (No match)
111 - 121	1065.5703	1064.5630	1064.5838	-0.0208	1	**AGAHLQGGAKR** (No match)
121 - 142	2369.1477	2368.1404	2368.2031	-0.0627	1	**RVIISAPSADAPMFVMGVNHEK** (No match)
121 - 142	2385.1580	2384.1507	2384.1980	-0.0473	1	**RVIISAPSADAPMFVMGVNHEK** Oxidation (M) (No match)
122 - 142	2213.0703	2212.0630	2212.1020	-0.0389	0	**VIISAPSADAPMFVMGVNHEK** (No match)
122 - 142	2229.0667	2228.0594	2228.0969	-0.0375	0	**VIISAPSADAPMFVMGVNHEK** Oxidation (M) (No match)
122 - 142	2245.0676	2244.0603	2244.0918	-0.0315	0	**VIISAPSADAPMFVMGVNHEK** 2 Oxidation (M) (No match)
149 - 165	1833.8783	1832.8710	1832.9124	-0.0414	0	**IISNASCTTNCLAPLAK** (No match)
166 - 189	2595.3181	2594.3108	2594.3526	-0.0418	0	**VIHDNFGIVEGLMTTVHAITATQK** (No match)
166 - 189	2611.3313	2610.3240	2610.3475	-0.0235	0	**VIHDNFGIVEGLMTTVHAITATQK** Oxidation (M) (No match)
204 - 218	1411.7546	1410.7473	1410.7830	-0.0357	0	**GALQNIIPASTGAAK** (No match)
223 - 230	869.4982	868.4909	868.5018	-0.0108	0	**VIPELNGK** (No match)
231 - 237	795.4071	794.3998	794.4109	-0.0110	0	**LTGMAFR** (No match)
231 - 237	811.3964	810.3891	810.4058	-0.0166	0	**LTGMAFR** Oxidation (M) (No match)
238 - 251	1530.7539	1529.7466	1529.7871	-0.0405	0	**VPTANVSVVDLTCR** (Ions score 122)
238 - 251	1530.7539	1529.7466	1529.7871	-0.0405	0	**VPTANVSVVDLTCR** (No match)
313 - 326	1763.7585	1762.7512	1762.7950	-0.0438	0	**LISWYDNEFGYSNR** (No match)
313 - 326	1763.7585	1762.7512	1762.7950	-0.0438	0	**LISWYDNEFGYSNR** (Ions score 120)

Mascot: http://www.matrixscience.com/

Spot 985

Protein View

Match to: **gi|5668560** Score: **303** Expect: **9.6e-026**
serine/threonine phosphatase 1 gamma [Homo sapiens]

Nominal mass (M_r): **34493**; Calculated pI value: **5.09**
NCBI BLAST search of gi|5668560 against nr
Unformatted sequence string for pasting into other applications

Taxonomy: Homo sapiens

Fixed modifications: Carbamidomethyl (C)
Variable modifications: Oxidation (M)
Cleavage by Trypsin: cuts C-term side of KR unless next residue is P
Sequence Coverage: **28%**

Matched peptides shown in **Bold Red**

```
  1 MADLDKLNID SIIQRLLEVR GSKPGKNVQL QENEIRGLCL KSREIFLSQP
 51 ILLELEAPLK ICGDIHGQYY DLLRLFEYGG FPPESNYLFL GDYVDRGKQS
101 LETICLLLAY KIKYPENFFL LRGNHECASI NRIYGFYDEC KRRYNIKLWK
151 TFTDCFNCLP IAAIVDEKIF CCHGGLSPDL QSMEQIRRIM RPTDVPDQGL
201 LCDLLWSDPD KDVLGWGEND RGVSFTFGAE VVAKFLHKHD LDLICRAHQV
251 VEDGYEFFAK RQLVTLFSAP NYCGEFDNAG AMMSVDETLM CSFQ
```

Residue Number Increasing Mass Decreasing Mass

Start - End	Observed	Mr(expt)	Mr(calc)	Delta	Miss	Sequence
27 - 36	1242.6394	1241.6321	1241.6364	-0.0042	0	NVQLQENEIR (No match)
61 - 74	1722.8148	1721.8075	1721.8195	-0.0120	0	ICGDIHGQYYDLLR (No match)
61 - 74	1722.8148	1721.8075	1721.8195	-0.0120	0	ICGDIHGQYYDLLR (Ions score 32)
75 - 96	2598.1831	2597.1758	2597.2113	-0.0355	0	LFEYGGFPPESNYLFLGDYVDR (Ions score 69)
75 - 96	2598.1831	2597.1758	2597.2113	-0.0355	0	LFEYGGFPPESNYLFLGDYVDR (No match)
112 - 122	1439.7974	1438.7901	1438.7972	-0.0071	1	IKYPENFFLLR (Ions score 33)
112 - 122	1439.7974	1438.7901	1438.7972	-0.0071	1	IKYPENFFLLR (No match)
114 - 122	1198.6234	1197.6161	1197.6182	-0.0021	0	YPENFFLLR (No match)
235 - 246	1566.8145	1565.8072	1565.8136	-0.0064	1	FLHKHDLDLICR (No match)
247 - 260	1639.7649	1638.7576	1638.7677	-0.0101	0	AHQVVEDGYEFFAK (No match)
247 - 261	1795.8657	1794.8584	1794.8688	-0.0104	1	AHQVVEDGYEFFAKR (No match)

247 - 261	1795.8657	1794.8584	1794.8688	-0.0104	1	AHQVVEDGYEFFAKR	(Ions score 51)

Mascot: http://www.matrixscience.com/

Spot 986

Protein View

Match to: **gi|56790945** Score: **258** Expect: **3e-021**
protein phosphatase 1, catalytic subunit, alpha isoform 3 [Homo sapiens]

Nominal mass (M_r): **39405**; Calculated pI value: **6.20**
NCBI BLAST search of gi|56790945 against nr
Unformatted sequence string for pasting into other applications

Taxonomy: Homo sapiens
Links to retrieve other entries containing this sequence from NCBI Entrez:
gi|298964 from Homo sapiens

Fixed modifications: Carbamidomethyl (C)
Variable modifications: Oxidation (M)
Cleavage by Trypsin: cuts C-term side of KR unless next residue is P
Sequence Coverage: **25%**

Matched peptides shown in **Bold Red**

```
  1 MSDSEKLNLD SIIGRLLEGS RVLTPHCAPV QGSRPGKNVQ LTENEIRGLC
 51 LKSREIFLSQ PILLELEAPL KICGDIHGQY YDLLRLFEYG GFPPESNYLF
101 LGDYVDRGKQ SLETICLLLA YKIKYPENFF LLRGNHECAS INRIYGFYDE
151 CKRRYNIKLW KTFTDCFNCL PIAAIVDEKI FCCHGGLSPD LQSMEQIRRI
201 MRPTDVPDQG LLCDLLWSDP DKDVQGWGEN DRGVSFTFGA EVVAKFLHKH
251 DLDLICRAHQ VVEDGYEFFA KRQLVTLFSA PNYCGEFDNA GAMMSVDETL
301 MCSFQILKPA DKNKGKYGQF SGLNPGGRPI TPPRNSAKAK K
```

Residue Number Increasing Mass Decreasing Mass

Start - End	Observed	Mr(expt)	Mr(calc)	Delta	Miss	Sequence
7 - 21	1655.9038	1654.8965	1654.9365	-0.0400	1	LNLDSIIGRLLEGSR (No match)
7 - 21	1655.9038	1654.8965	1654.9365	-0.0400	1	LNLDSIIGRLLEGSR (No match)
72 - 85	1722.8363	1721.8290	1721.8195	0.0095	0	ICGDIHGQYYDLLR (No match)
72 - 85	1722.8363	1721.8290	1721.8195	0.0095	0	ICGDIHGQYYDLLR (Ions score 12)
86 - 107	2598.2307	2597.2234	2597.2113	0.0121	0	LFEYGGFPPESNYLFLGDYVDR (No match)
86 - 107	2598.2307	2597.2234	2597.2113	0.0121	0	LFEYGGFPPESNYLFLGDYVDR (Ions score 77)
123 - 133	1439.8127	1438.8054	1438.7972	0.0082	1	IKYPENFFLLR (No match)
123 - 133	1439.8127	1438.8054	1438.7972	0.0082	1	IKYPENFFLLR (No match)

125 - 133	1198.6351	1197.6278	1197.6182	0.0096	0	YPENFFLLR	(No match)
246 - 257	1566.8257	1565.8184	1565.8136	0.0048	1	FLHKHDLDLICR	(No match)
258 - 271	1639.7827	1638.7754	1638.7677	0.0077	0	AHQVVEDGYEFFAK	(No match)
258 - 271	1639.7827	1638.7754	1638.7677	0.0077	0	AHQVVEDGYEFFAK	(Ions score 69)

Mascot: http://www.matrixscience.com/

Spot 987

Protein View

Match to: **gi|15277503** Score: **259** Expect: **2.4e-021**
ACTB protein [Homo sapiens]

Nominal mass (M_r): **40536**; Calculated pI value: **5.55**
NCBI BLAST search of gi|15277503 against nr
Unformatted sequence string for pasting into other applications

Taxonomy: Homo sapiens

Fixed modifications: Carbamidomethyl (C)
Variable modifications: Oxidation (M)
Cleavage by Trypsin: cuts C-term side of KR unless next residue is P
Sequence Coverage: **28%**

Matched peptides shown in **Bold Red**

```
  1 MCKAGFAGDD APRAVFPSIV GRPRHQGVMV GMGQKDSYVG DEAQSKRGIL
 51 TLKYPIEHGI VTNWDDMEKI WHHTFYNELR VAPEEHPVLL TEAPLNPKAN
101 LEKMTQIMFE TFNTPAMYVA IQAVLSLYAS GRTTGIVMDS GDGVTHTVPI
151 YEGYALPHAI LRLDLAGRDL TDYLMKILTE RGYSFTTTAE REIVRDIKEK
201 LCYVALDFEQ EMATAASSSS LEKSYELPDG QVITIGNERF RCPEALFQPS
251 FLGMESCGIH ETTFNSIMKC DVDIRKDLYA NTVLSGGTTM YPGIADRMQK
301 EITALAPSTM KIKIIAPPER KYSVWIGGSI LASLSTFQQM WISKQEYDES
351 GPSIVHRKCF
```

Residue Number Increasing Mass Decreasing Mass

Start - End	Observed	Mr(expt)	Mr(calc)	Delta	Miss	Sequence
36 - 47	1354.6145	1353.6072	1353.6160	-0.0088	1	DSYVGDEAQSKR (No match)
81 - 98	1954.0472	1953.0399	1953.0571	-0.0171	0	VAPEEHPVLLTEAPLNPK (Ions score 53)
81 - 98	1954.0472	1953.0399	1953.0571	-0.0171	0	VAPEEHPVLLTEAPLNPK (No match)
182 - 191	1132.5195	1131.5122	1131.5196	-0.0074	0	GYSFTTTAER (Ions score 6)
182 - 191	1132.5195	1131.5122	1131.5196	-0.0074	0	GYSFTTTAER (No match)
224 - 239	1790.8716	1789.8643	1789.8846	-0.0202	0	SYELPDGQVITIGNER (No match)
224 - 239	1790.8716	1789.8643	1789.8846	-0.0202	0	SYELPDGQVITIGNER (Ions score 47)
277 - 297	2231.0557	2230.0484	2230.0575	-0.0091	0	DLYANTVLSGGTTMYPGIADR Oxidation (M) (No match)
312 - 320	1036.6439	1035.6366	1035.6440	-0.0074	1	IKIIAPPER (No match)

314 - 320	795.4733	794.4660	794.4650	0.0010	0	IIAPPER (No match)
314 - 321	923.5614	922.5541	922.5600	-0.0058	1	IIAPPERK (No match)
345 - 357	1516.6953	1515.6880	1515.6953	-0.0073	0	QEYDESGPSIVHR (Ions score 36)
345 - 357	1516.6953	1515.6880	1515.6953	-0.0073	0	QEYDESGPSIVHR (No match)
345 - 358	1644.7838	1643.7765	1643.7903	-0.0138	1	QEYDESGPSIVHRK (No match)

Mascot: http://www.matrixscience.com/

Spot 988

Protein View

Match to: **gi|16741698** Score: **101** Expect: **1.5e-005**
Phosphoserine aminotransferase 1 [Homo sapiens]

Nominal mass (M_r): **40782;** Calculated pI value: **7.56**
NCBI BLAST search of gi|16741698 against nr
Unformatted sequence string for pasting into other applications

Taxonomy: Homo sapiens

Fixed modifications: Carbamidomethyl (C)
Variable modifications: Oxidation (M)
Cleavage by Trypsin: cuts C-term side of KR unless next residue is P
Sequence Coverage: **9%**

Matched peptides shown in **Bold Red**

```
  1 MDAPRQVVNF GPGPAKLPHS VLLEIQKELL DYKGVGISVV EMSHRSSDFA
 51 KIINNTENLV RELLAVPDNY KVIFLQGGGC GQFSAVPLNL IGLKAGRCAD
101 YVVTGAWSAK AAEEAKKFGT INIVHPKLGS YTKIPDPSTW NLNPDASYVY
151 YCANETVHGV EFDFIPDVKG AVLVCDMSSN FLSKPVDVSK FGVIFAGAQK
201 NVGSAGVTVV IVRDDLLGFA LRECPSVLEY KVQAGNSSLY NTPPCFSIYV
251 MGLVLEWIKN NGGAAAMEKL SSIKSQTIYE IIDNSQGFYV CPVEPQNRSK
301 MNIPFRIGNA KGDDALEKRF LDKALELNML SLKGHRSVGG IRASLYNAVT
351 IEDVQKLAAF MKKFLEMHQL
```

Residue Number Increasing Mass Decreasing Mass

Start - End	Observed	Mr(expt)	Mr(calc)	Delta	Miss	Sequence
191 - 200	1037.5754	1036.5681	1036.5705	-0.0024	0	FGVIFAGAQK (No match)
191 - 200	1037.5754	1036.5681	1036.5705	-0.0024	0	FGVIFAGAQK (Ions score 17)
275 - 298	2857.3250	2856.3177	2856.3388	-0.0211	0	SQTIYEIIDNSQGFYVCPVEPQNR (No match)
275 - 298	2857.3250	2856.3177	2856.3388	-0.0211	0	SQTIYEIIDNSQGFYVCPVEPQNR (Ions score 58)

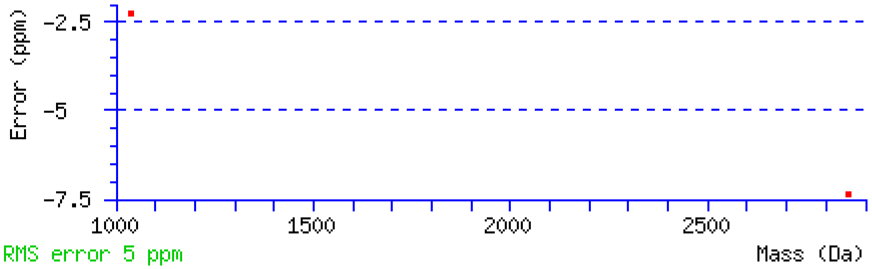

RMS error 5 ppm

Mascot: http://www.matrixscience.com/

Spot 989

Protein View

Match to: **gi|24111250** Score: **320** Expect: **1.9e-027**
guanine nucleotide binding protein (G protein), alpha 13 [Homo sapiens]

Nominal mass (M_r): **44364**; Calculated pI value: **8.11**
NCBI BLAST search of gi|24111250 against nr
Unformatted sequence string for pasting into other applications

Taxonomy: Homo sapiens
Links to retrieve other entries containing this sequence from NCBI Entrez:
gi|38258936 from Homo sapiens
gi|20147697 from Homo sapiens
gi|22478060 from Homo sapiens
gi|119609399 from Homo sapiens
gi|119609401 from Homo sapiens
gi|123993793 from synthetic construct
gi|123995545 from synthetic construct

Fixed modifications: Carbamidomethyl (C)
Variable modifications: Oxidation (M)
Cleavage by Trypsin: cuts C-term side of KR unless next residue is P
Sequence Coverage: **46%**

Matched peptides shown in **Bold Red**

```
  1 MADFLPSRSV LSVCFPGCLL TSGEAEQQRK SKEIDKCLSR EKTYVKRLVK
 51 ILLLGAGESG KSTFLKQMRI IHGQDFDQRA REEFRPTIYS NVIKGMRVLV
101 DAREKLHIPW GDNSNQQHGD KMMSFDTRAP MAAQGMVETR VFLQYLPAIR
151 ALWADSGIQN AYDRRREFQL GESVKYFLDN LDKLGEPDYI PSQQDILLAR
201 RPTKGIHEYD FEIKNVPFKM VDVGGQRSER KRWFECFDSV TSILFLVSSS
251 EFDQVLMEDR LTNRLTESLN IFETIVNNRV FSNVSIILFL NKTDLLEEKV
301 QIVSIKDYFL EFEGDPHCLR DVQKFLVECF RNKRRDQQQK PLYHHFTTAI
351 NTENIRLVFR DVKDTILHDN LKQLMLQ
```

Residue Number Increasing Mass Decreasing Mass

Start - End	Observed	Mr(expt)	Mr(calc)	Delta	Miss	Sequence
9 - 29	2337.9651	2336.9578	2337.1092	-0.1514	0	SVLSVCFPGCLLTSGEAEQQR (No match)

70 - 79	1228.5349	1227.5276	1227.5996	-0.0720	0	**IIHGQDFDQR** (No match)
70 - 79	1228.5349	1227.5276	1227.5996	-0.0720	0	**IIHGQDFDQR** (Ions score 24)
80 - 94	1822.8701	1821.8628	1821.9736	-0.1108	1	**AREEFRPTIYSNVIK** (No match)
82 - 94	1595.7426	1594.7353	1594.8354	-0.1001	0	**EEFRPTIYSNVIK** (Ions score 29)
82 - 94	1595.7426	1594.7353	1594.8354	-0.1001	0	**EEFRPTIYSNVIK** (No match)
129 - 140	1293.5065	1292.4992	1292.5853	-0.0860	0	**APMAAQGMVETR** 2 Oxidation (M) (No match)
141 - 150	1219.6488	1218.6415	1218.7124	-0.0709	0	**VFLQYLPAIR** (No match)
141 - 150	1219.6488	1218.6415	1218.7124	-0.0709	0	**VFLQYLPAIR** (Ions score 33)
176 - 200	2936.2876	2935.2803	2935.4967	-0.2163	1	**YFLDNLDKLGEPDYIPSQQDILLAR** (No match)
184 - 200	1927.9069	1926.8996	1927.0050	-0.1054	0	**LGEPDYIPSQQDILLAR** (No match)
205 - 214	1250.5282	1249.5209	1249.5978	-0.0769	0	**GIHEYDFEIK** (No match)
220 - 227	877.3517	876.3444	876.4123	-0.0679	0	**MVDVGGQR** Oxidation (M) (No match)
265 - 279	1762.8282	1761.8209	1761.9260	-0.1051	0	**LTESLNIFETIVNNR** (No match)
265 - 279	1762.8282	1761.8209	1761.9260	-0.1051	0	**LTESLNIFETIVNNR** (Ions score 80)
280 - 299	2322.1404	2321.1331	2321.2881	-0.1550	1	**VFSNVSIILFLNKTDLLEEK** (No match)
293 - 299	847.3755	846.3682	846.4334	-0.0652	0	**TDLLEEK** (No match)
325 - 331	970.4205	969.4132	969.4742	-0.0609	0	**FLVECFR** (No match)
336 - 356	2554.1086	2553.1013	2553.2724	-0.1711	0	**DQQQKPLYHHFTTAINTENIR** (No match)

Mascot: http://www.matrixscience.com/

Spot 992

Mascot Search Results

Protein View

Match to: **gi|34452679** Score: **226** Expect: **4.8e-018**
growth-inhibiting protein 1 [Homo sapiens]

Nominal mass (M_r): **34670**; Calculated pI value: **5.69**
NCBI BLAST search of gi|34452679 against nr
Unformatted sequence string for pasting into other applications

Taxonomy: Homo sapiens

Fixed modifications: Carbamidomethyl (C)
Variable modifications: Oxidation (M)
Cleavage by Trypsin: cuts C-term side of KR unless next residue is P
Sequence Coverage: **13%**

Matched peptides shown in **Bold Red**

```
  1 MRLFGGNFAH QASVARVVGQ QGRGRAGIEA SLDVQYLMSA GANISTWVYS
 51 SPGRHEGQEP FLQWLMLLSN ESALPHVHTV SYGDDEDSLS SAYIQRVNTE
101 LMKAAARGLT LLFASGDSGA GCWSVSGRHQ FRPTFPASSP YVTTVGGTSF
151 QEPFLITNEI VDYISGGGFS NVFPRPSYQE EAVTKFLSSS PHLPPSSYFN
```
[truncated: 195,375 more chars]
